# Supplementary material for: Complete telomere-to-telomere genomes uncover virulence evolution conferred by chromosome fusion in oomycete plant pathogens
Source: Nat Commun. 2024 May 30;15:4624. doi: 10.1038/s41467-024-49061-y (PMC11139960; doi:10.1038/s41467-024-49061-y)
Supplement: Supplementary file 1 — Supplementary Information [file 41467_2024_49061_MOESM1_ESM.pdf]

Supplementary information

Complete telomere-to-telomere genomes uncover virulence evolution conferred by chromosome fusion in oomycete plant pathogens

Zhichao Zhang[1,2], Xiaoyi Zhang[1,2], Yuan Tian[1,2], Liyuan Wang[1,2], Jingting Cao[1,2], Hui Feng[3], Kainan Li[1,2], Yan Wang[1,2], Suomeng Dong[1,2], Wenwu Ye[1,2*], Yuanchao Wang[1,2*]

[1]Department of Plant Pathology, Nanjing Agricultural University, Nanjing, Jiangsu 210095, China; [2]Key Laboratory of Soybean Disease and Pest Control (Ministry of Agriculture and Rural Affairs), Nanjing Agricultural University, Nanjing, Jiangsu 210095, China; [3]Tobacco Research Institute, Chinese Academy of Agricultural Sciences, Qingdao 266101, China.

[*]Corresponding authors:

Wenwu Ye (E-mail: yeww@njau.edu.cn);

Yuanchao Wang (E-mail: wangyc@njau.edu.cn).

Supplementary Figures

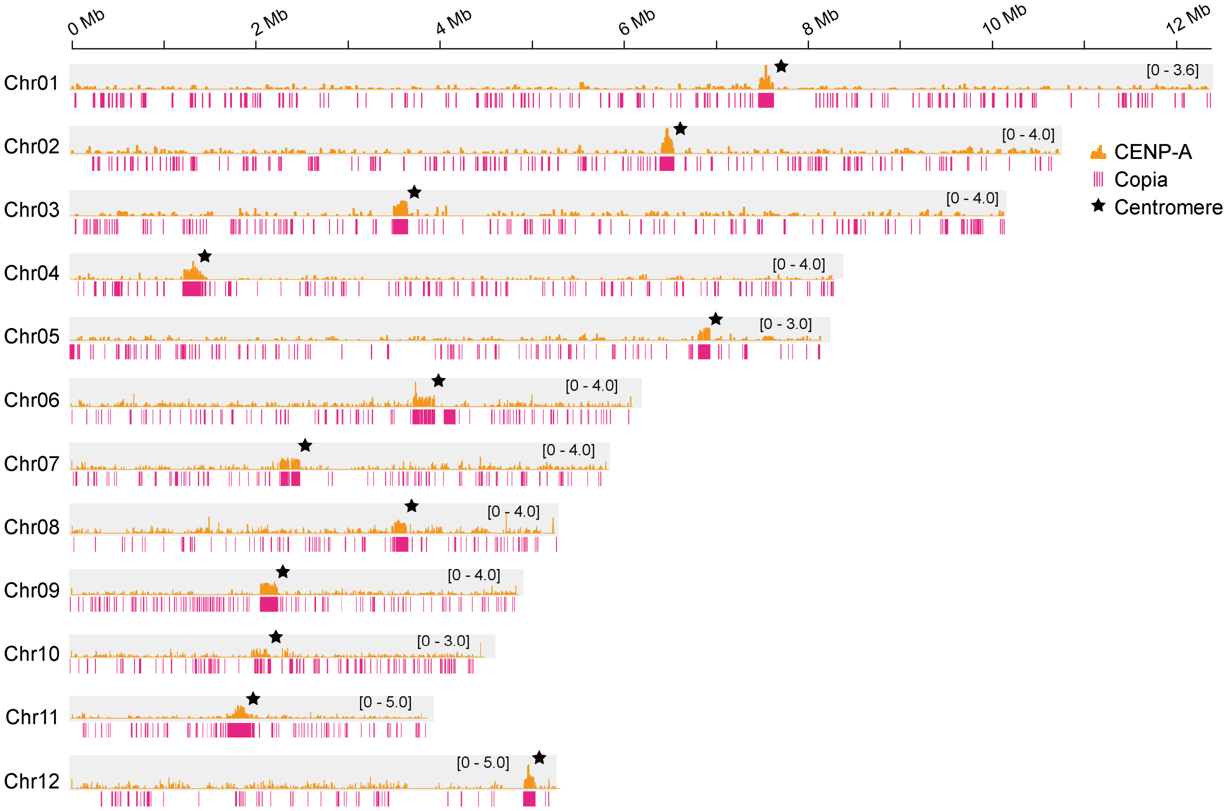

Supplementary Fig. 1 *Copia*-like transposons were enriched in centromeric regions based on CENP-A ChIP-seq data. The pentagram represents the centromeric region. Source data are provided as a Source Data file.

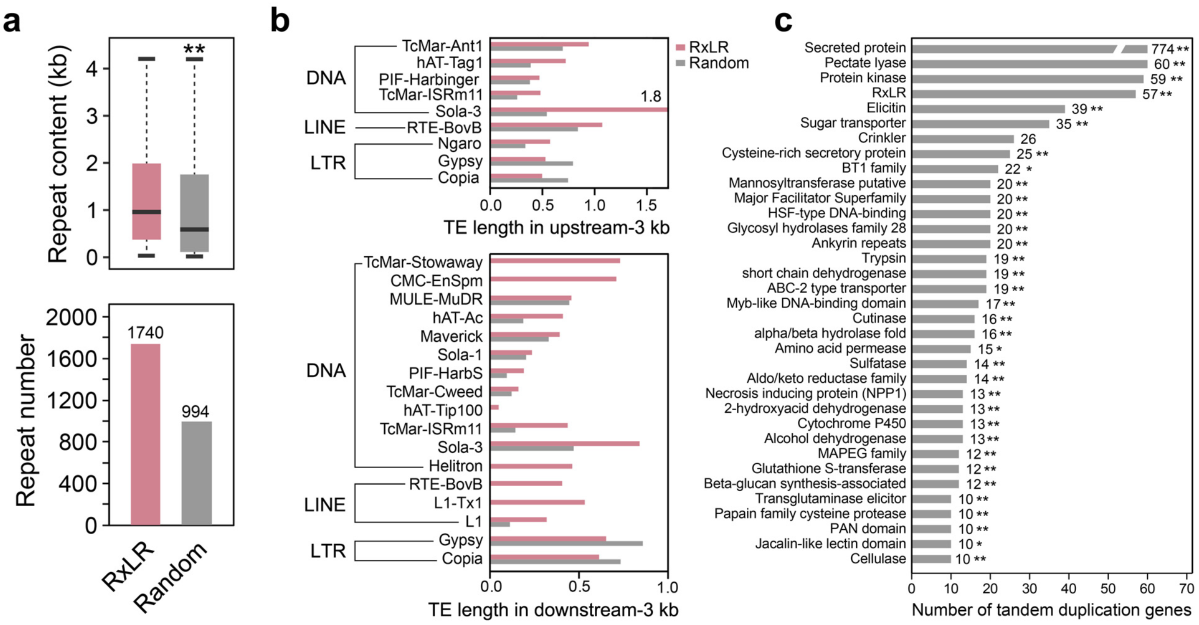

Supplementary Fig. 2 Distributions of repeats associated with RxLR and tandem duplicated genes. a, b Repeat content (p=1.6e-05) and repeat number of the upstream and downstream 3 kb of RxLR and randomly selected genes. Sample size n=724. *P*-values were calculated by two-sided Wilcoxon tests. **c** Class and number of tandem duplicated genes. Specific families enriched in tandem duplicated genes. *P*-values were calculated by one-sided hypergeometric test; **, $p < 0.01$; *, $p < 0.05$. Exact p-values were provided in Source data. Source data are provided as a Source Data file.

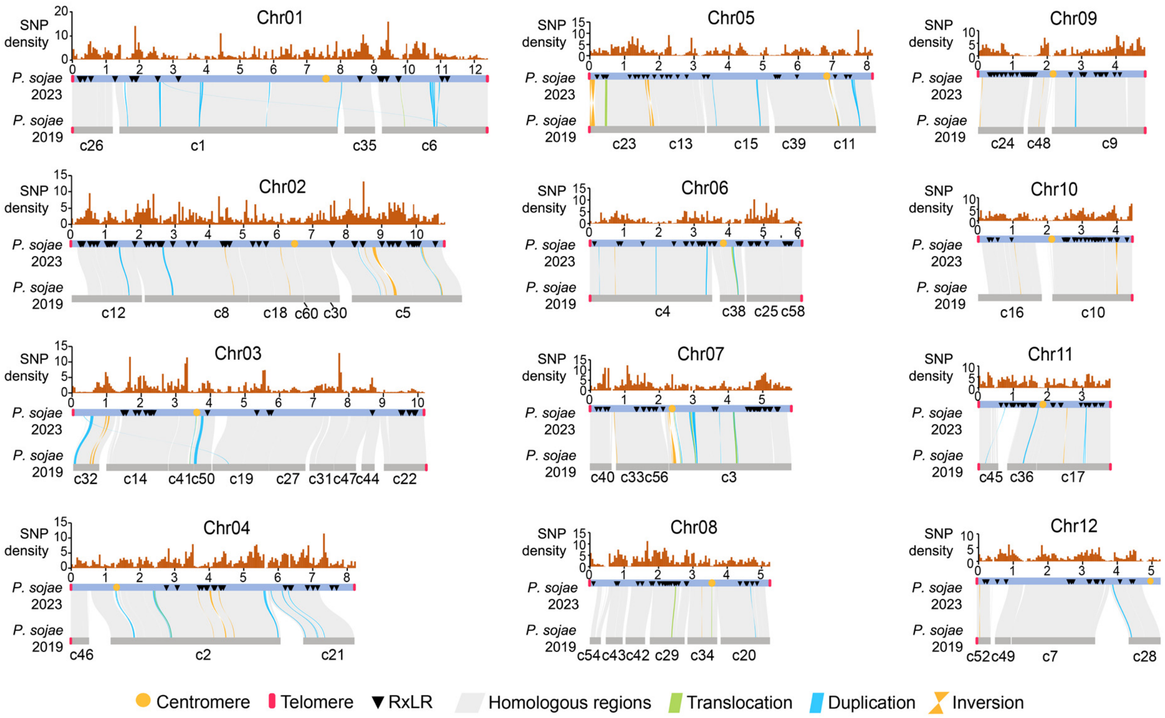

Supplementary Fig. 3 Distributions of SVs and SNPs across Chr01–Chr12. The genome collinearity between *P. sojae* 2019 and *P. sojae* 2023 shows RxLR visualized on the chromosomes of *P. sojae* 2023. Source data are provided as a Source Data file.

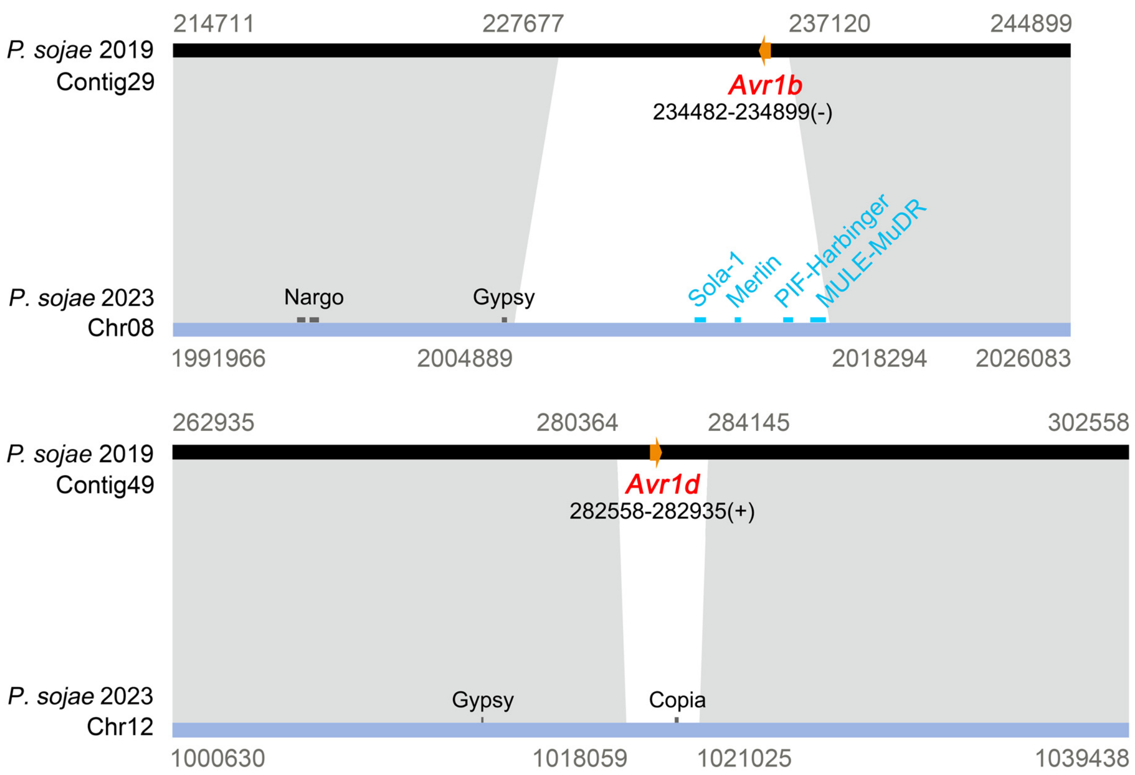

Supplementary Fig. 4 Structural variations in *Avr1b* and *Avr1d*. Orange arrows represents genes. Grey numbers represent corresponding genome coordinates. Sky-cyan boxes represent DNA transposons. Dark grey boxes represent LTR transposons. Source data are provided as a Source Data file.

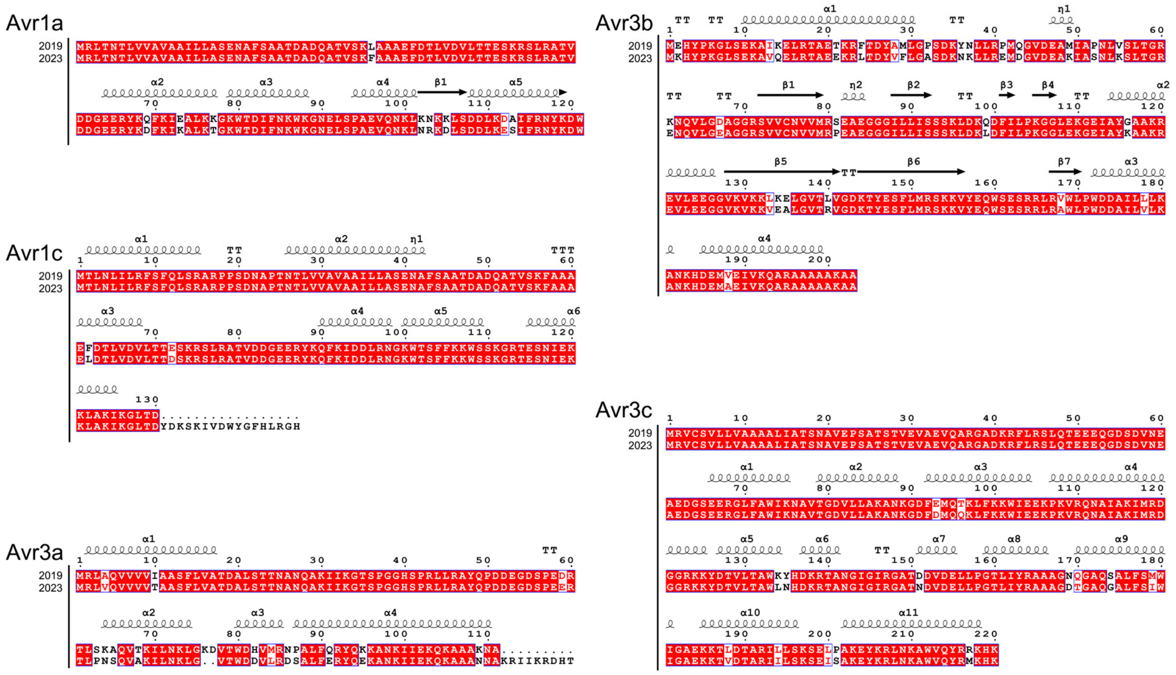

Supplementary Fig. 5 Sequence variants of *Avr1a*, *Avr1c*, *Avr3a*, *Avr3b*, and *Avr3c*. α-helices (medium squiggles), 3_{10} helices (small squiggles), π-helices (large squiggles), β-strands (arrows), strict α-turns (TTT letters), and β-turns (TT letters) mapped from the 3D structure predicted by AlphaFold2. Source data are provided as a Source Data file.

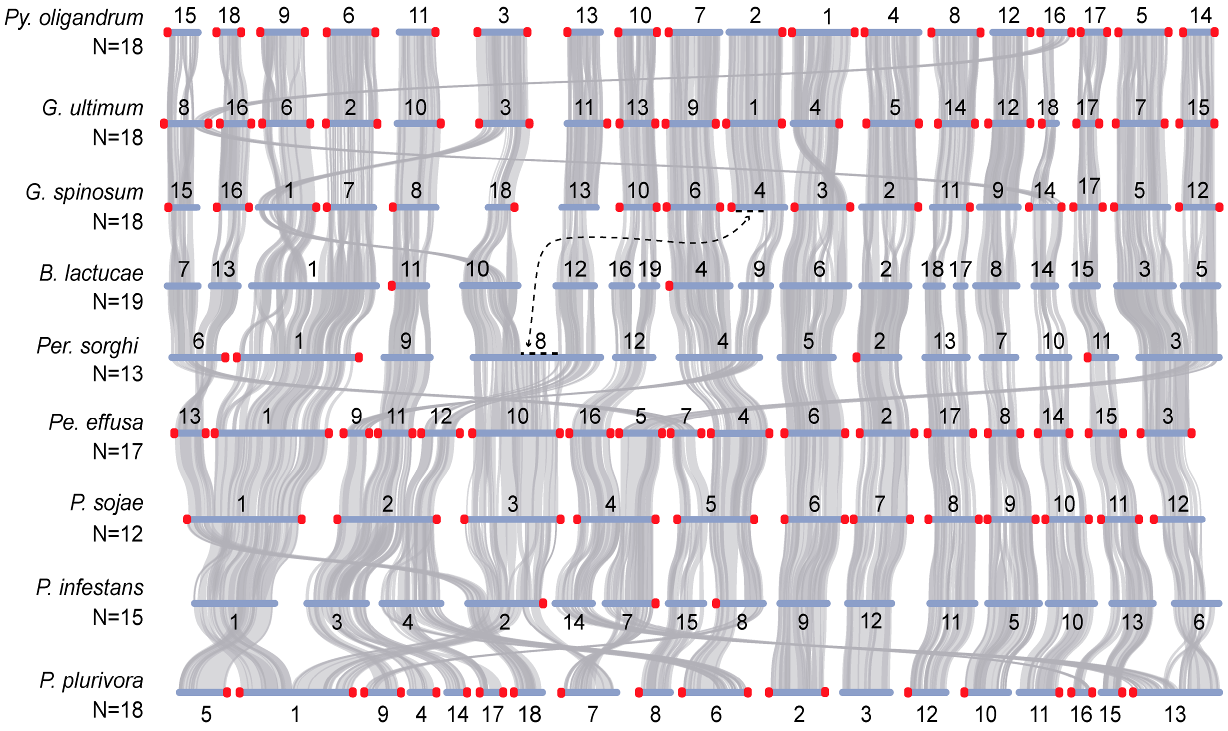

Supplementary Fig. 6 Chromosome-level synteny of 9 oomycete species. The arrow and line represents the missing collinearity in *B. lactucae*. Source data are provided as a Source Data file.

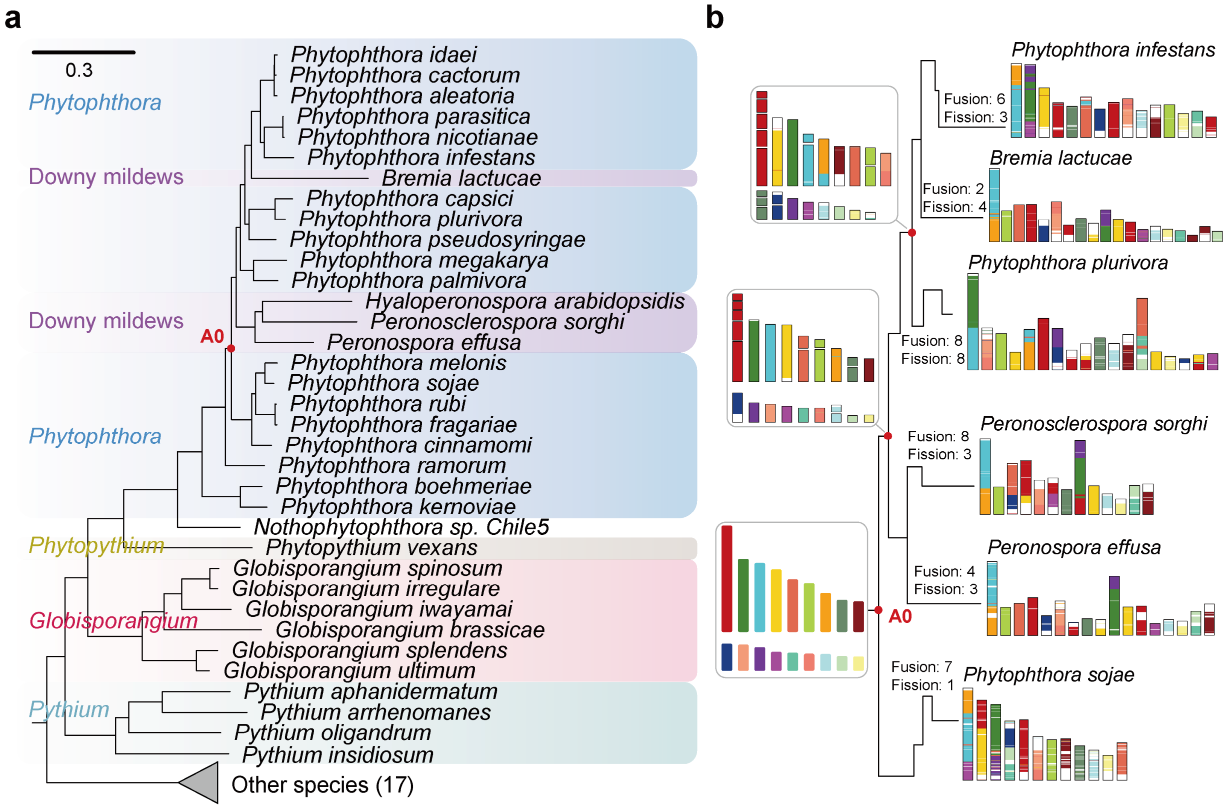

Supplementary Fig. 7 Reconstruction of Peronosporales ancestral chromosomes based on a phylogenetic tree of 52 oomycetes species. a A total of 52 species were used to construct the phylogenetic tree. **b** The 20 species belonging to node A0 were used for the reconstruction of ancestral chromosome karyotypes. Source data are provided as a Source Data file.

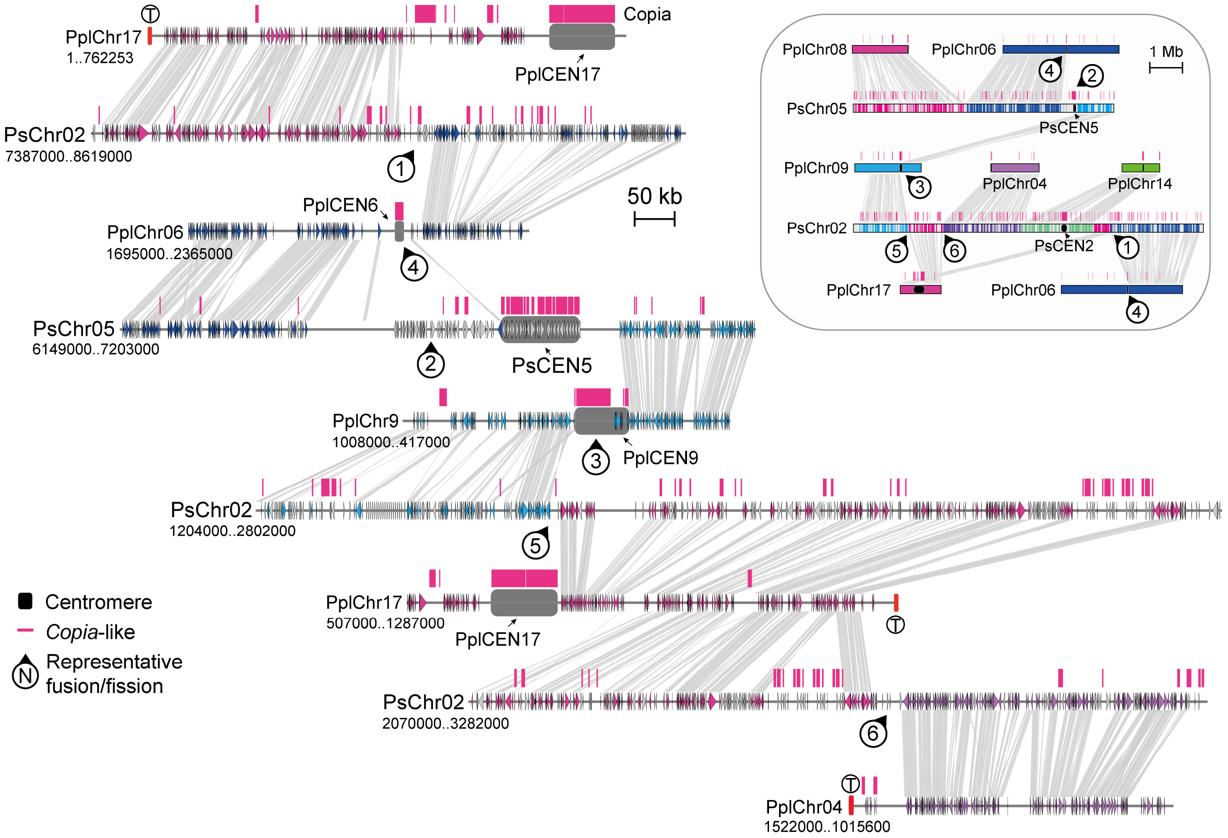

Supplementary Fig. 8 Micro-synteny of *P. plurivora* and *P. sojae* on PsChr02 and PsChr05. The shaded area in the diagram indicates the centromeric region. Source data are provided as a Source Data file.

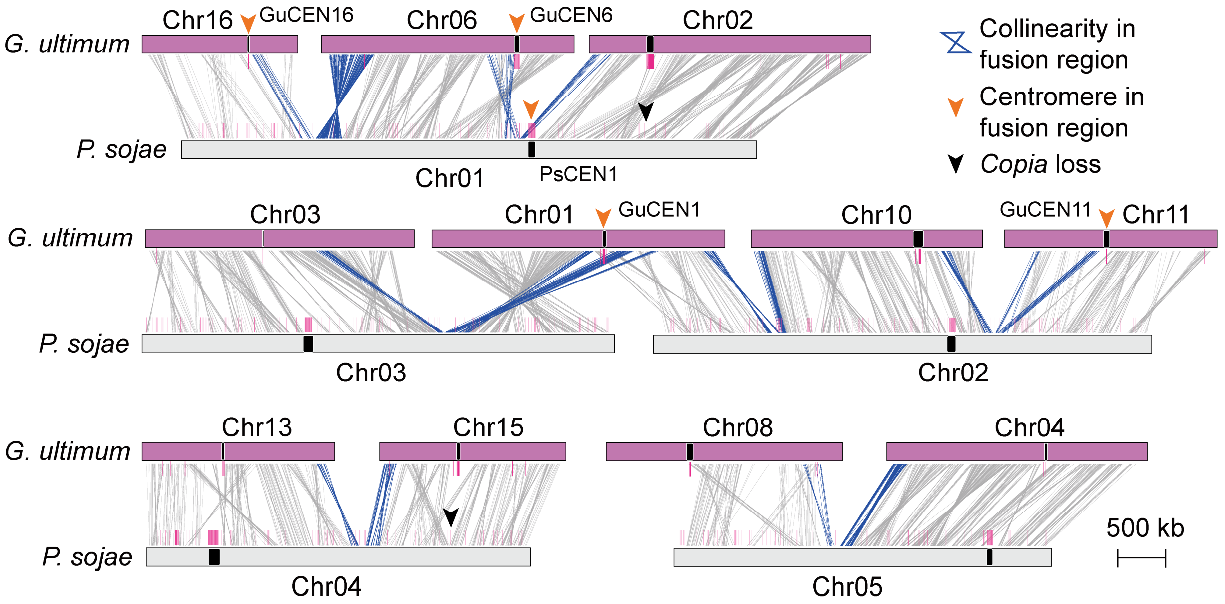

Supplementary Fig. 9 Chromosome-level synteny of *G. ultimum* and *P. sojae* in fused chromosomes. Source data are provided as a Source Data file.

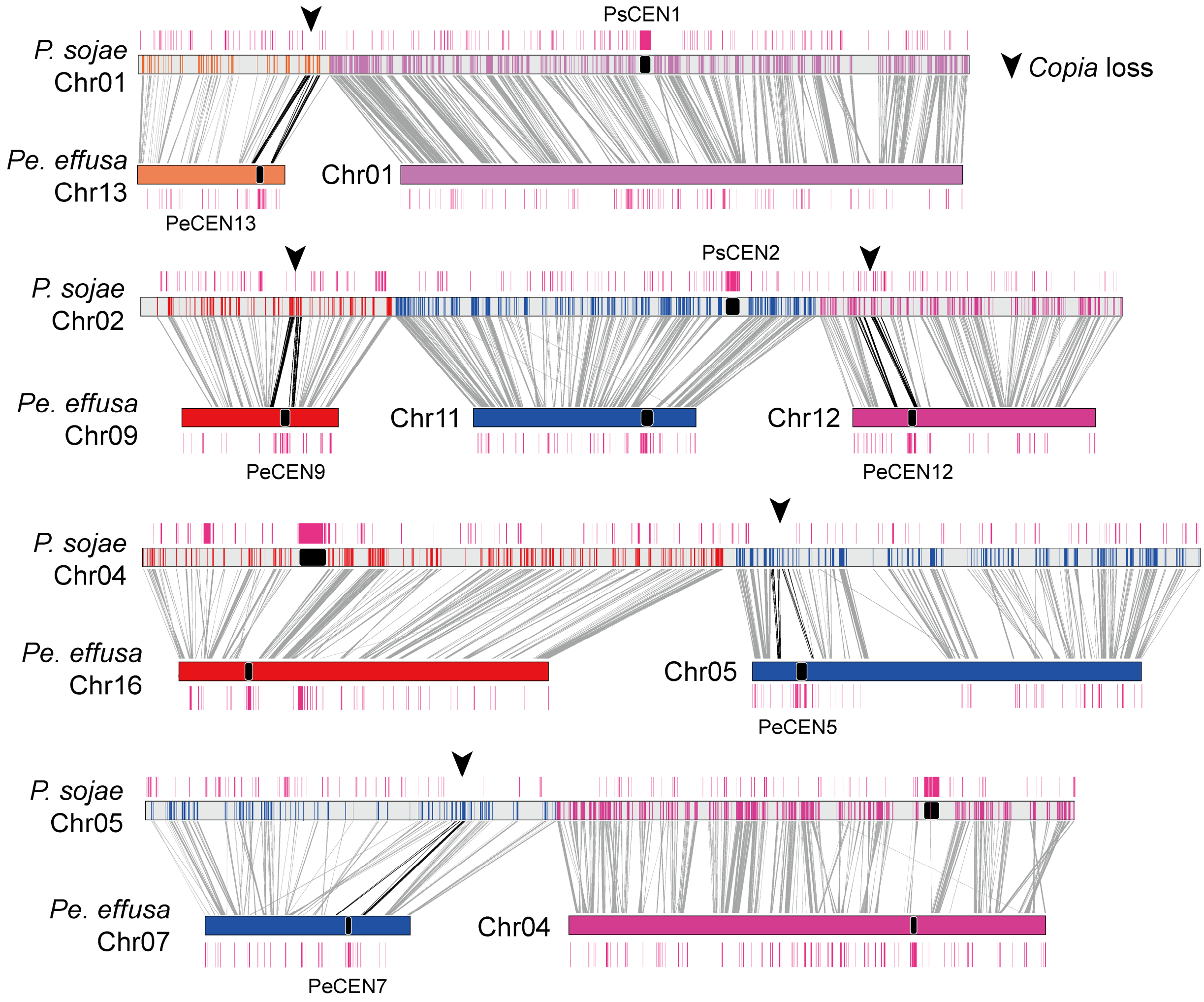

Supplementary Fig. 10 Chromosome-level synteny of *Pe. effusa* and *P. sojae* in fused chromosomes. Source data are provided as a Source Data file.

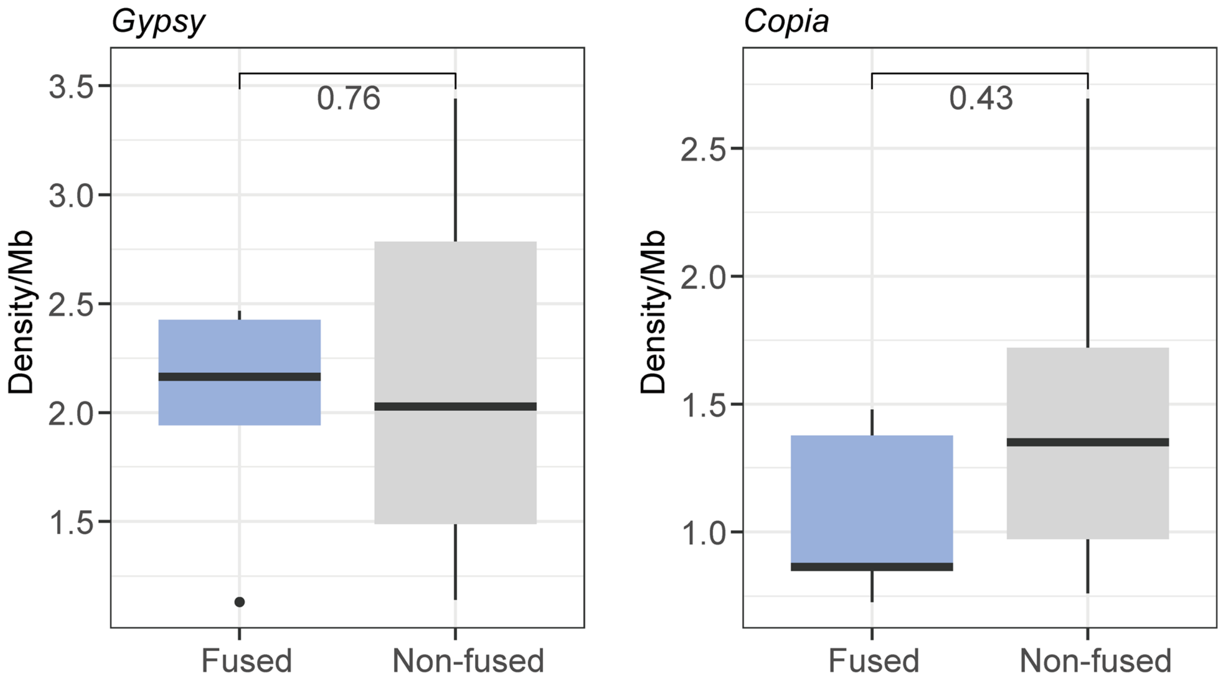

Supplementary Fig. 11 Comparison of intact *Copia* and *Gypsy* transposons density between fused and non-fused chromosomes. *P*-values were calculated by two-sided Wilcoxon tests. The center line in box plots indicates the median, the box outlines the 25th and 75th percentiles, and the whiskers extend to 1.5 times the interquartile range beyond the box edges. Sample number n=24. Source data are provided as a Source Data file.

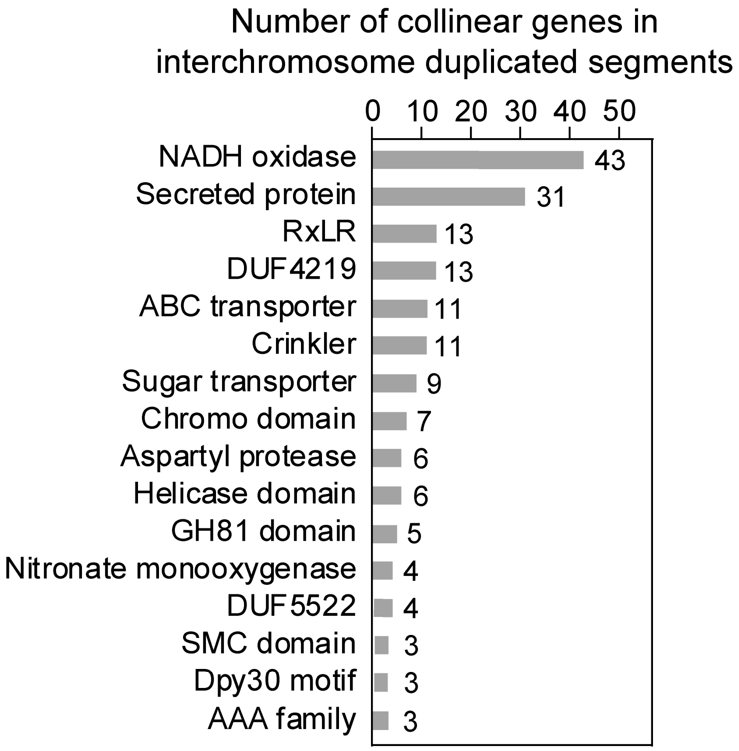

Supplementary Fig. 12 Functional annotation and statistics of collinear genes in inter-chromosome fragment duplications. Sort the bar chart in descending order by gene count.

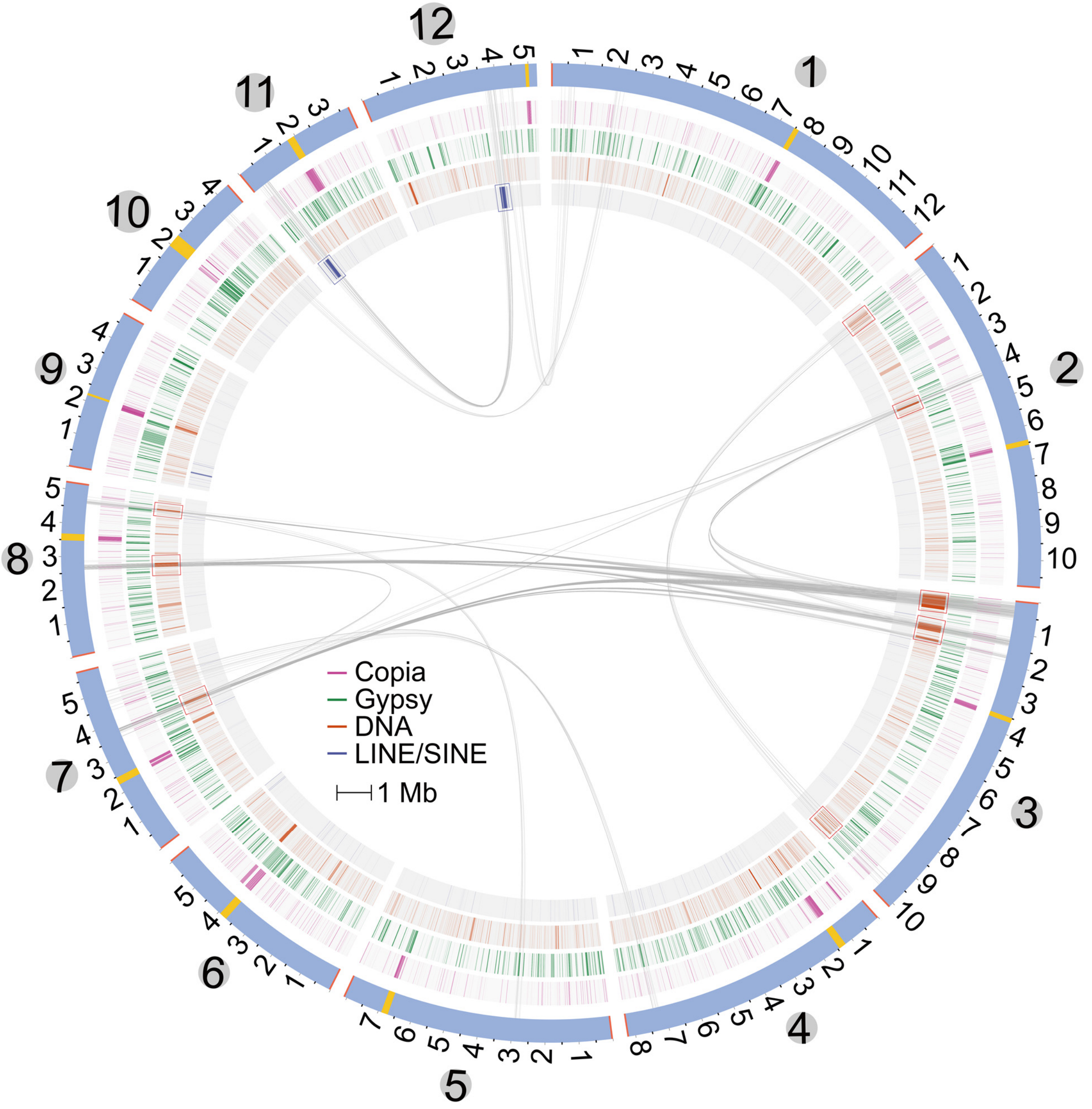

Supplementary Fig. 13 DNA transposons or LINE/SINE retrotransposons may be involved in inter-chromosome segmental duplication. The numbers on the outer ring represent the chromosome number.

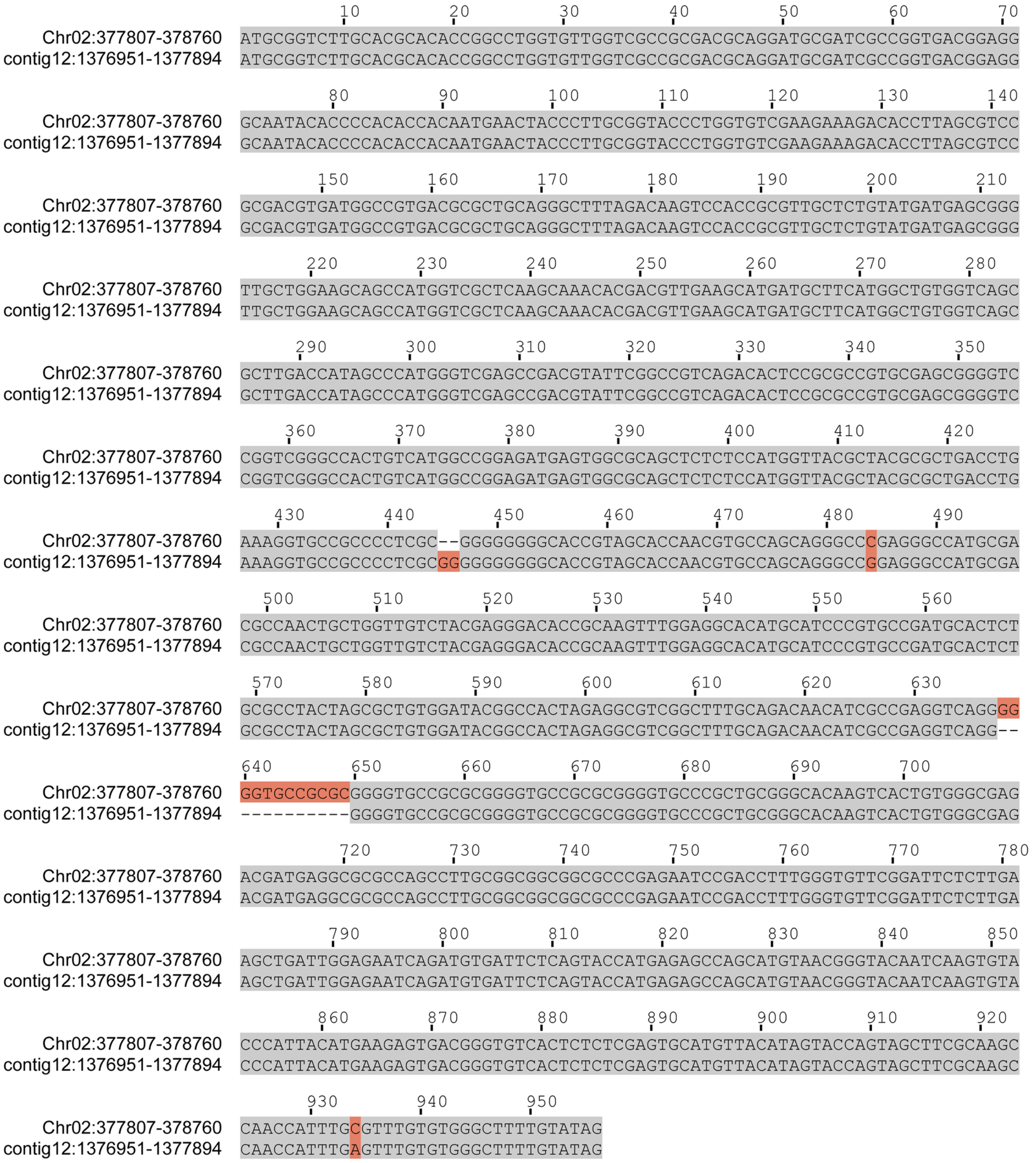

Supplementary Fig. 14 Insertion/deletion variants of *Avh054* gene between *P. sojae* 2023 and *P. sojae* 2019. The sequence above is from *P. sojae* 2023, while the sequence below is from 2019.

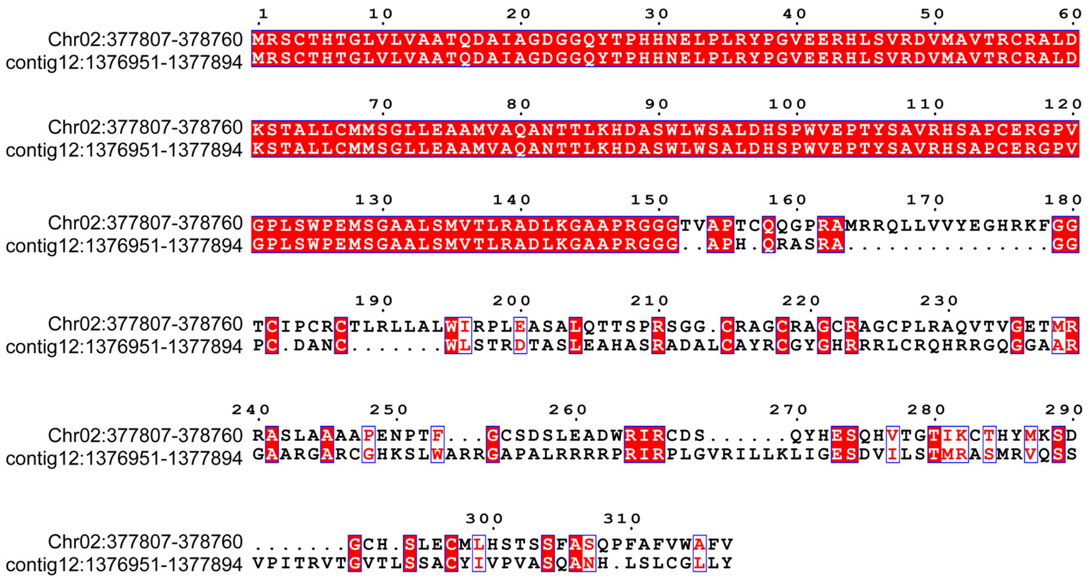

Supplementary Fig. 15 Truncated protein sequences of Avh054 in *P. sojae* 2023 and *P. sojae* 2019. The sequence above is from *P. sojae* 2023, while the sequence below is from 2019.

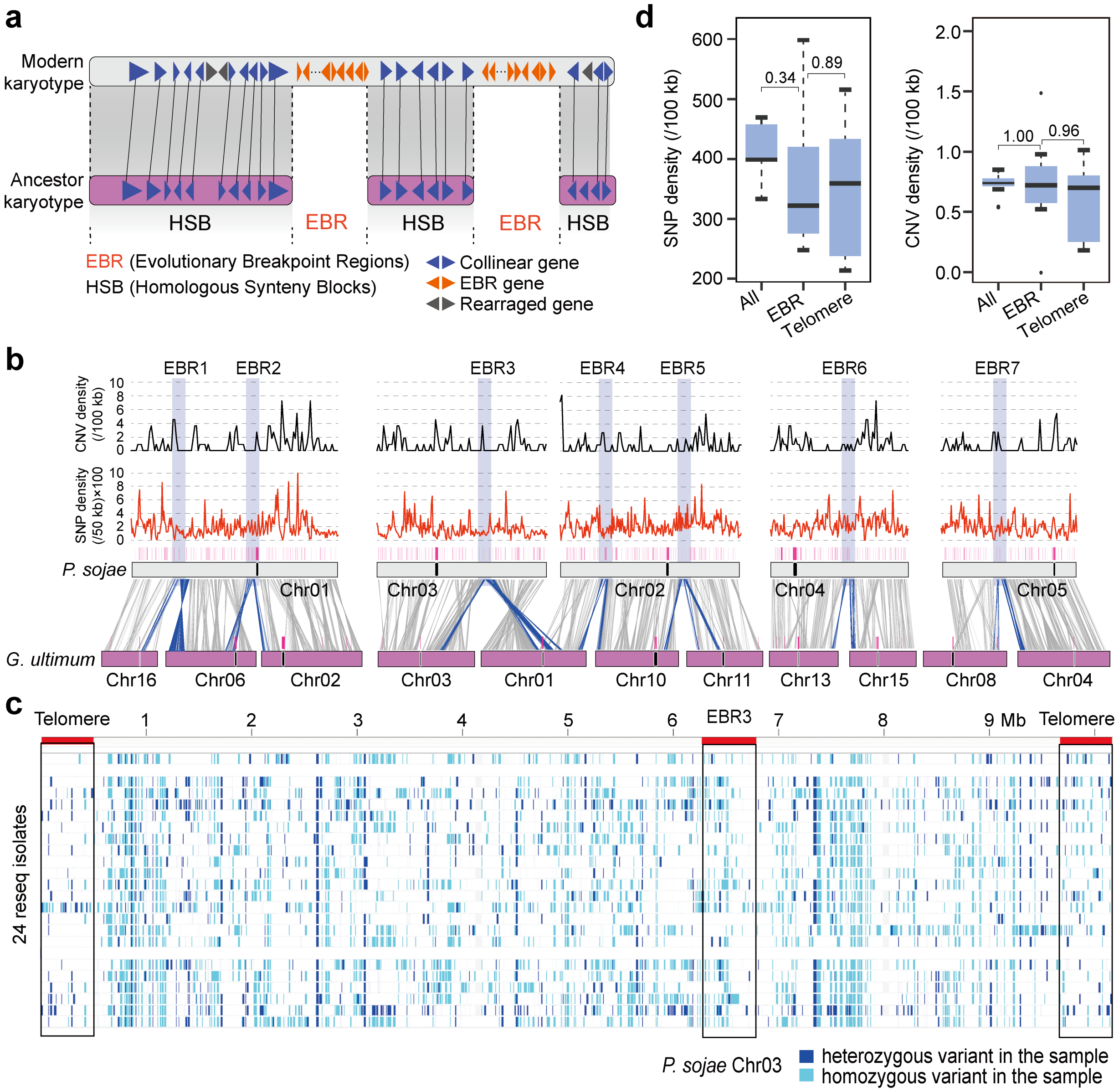

Supplementary Fig. 16 The distribution of SNPs and SVs of 24 isolates in evolutionary breakpoint regions. a Definition of HSB and EBR. **b** The distribution of SNP and CNV density statistics on *P. sojae* fused chromosomes. **c** An example of 24 resequencing isolates aligned to the *P. sojae* Chr03. The red bar represents the defined 500 kb region. **d** Statistics of Telomere 500 kb, 500 kb around EBR and genome-wide average SNPs and CNVs. The center line in box plots indicates the median, the box outlines the 25th and 75th percentiles, and the whiskers extend to 1.5 times the interquartile range beyond the box edges. *P*-values were calculated

by two-sided Wilcoxon tests. Sample size n=22. Source data are provided as a Source Data file.

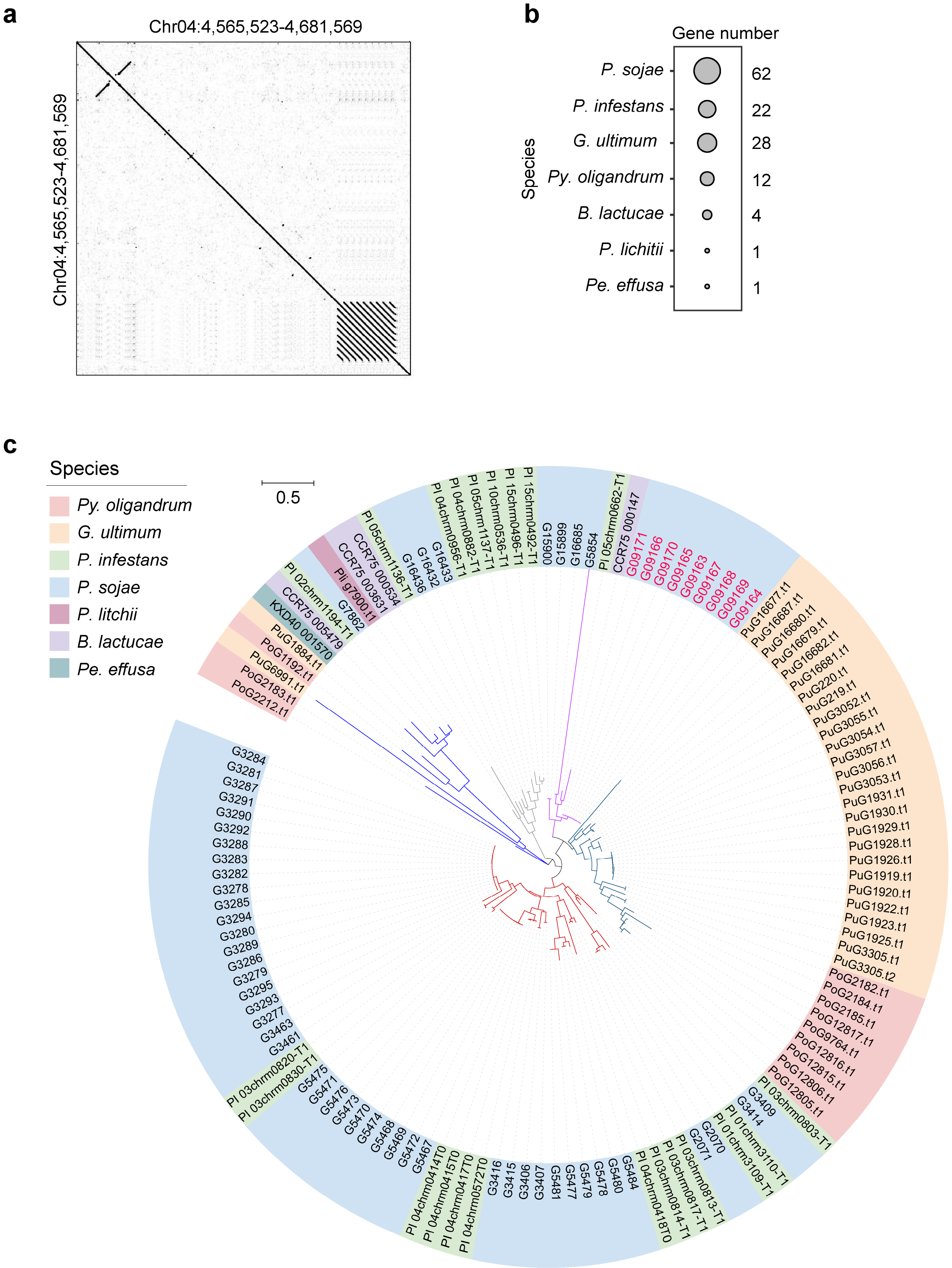
a
Chr04:4,565,523-4,681,569
Chr04:4,565,523-4,681,569
b
Gene number
Species
P. sojae
P. infestans
G. ultimum
Py. oligandrum
B. lactucae
P. lichitii
Pe. effusa
62
22
28
12
4
1
1
c
Species
Py. oligandrum
G. ultimum
P. infestans
P. sojae
P. litchii
B. lactucae
Pe. effusa
0.5
G09171
G09166
G09170
G09165
G09163
G09167
G09168
G09169
G09164

Supplementary Fig. 17 Tandem duplication of pectate lyase and phylogenetic tree. **a** Fused region of Chr04 was visualized using dot plot. Tandem duplicated genes are located in the lower right corner. **b** The gene number of PL family (PF03211) in seven species of oomycetes. The size of circle represents the number of genes. **c** A phytogenic tree was constructed based on identified PL genes in seven species of oomycetes. The nine tandem duplicated genes of the PL family in fused regions are highlighted in red font. Source data are provided as a Source Data file.

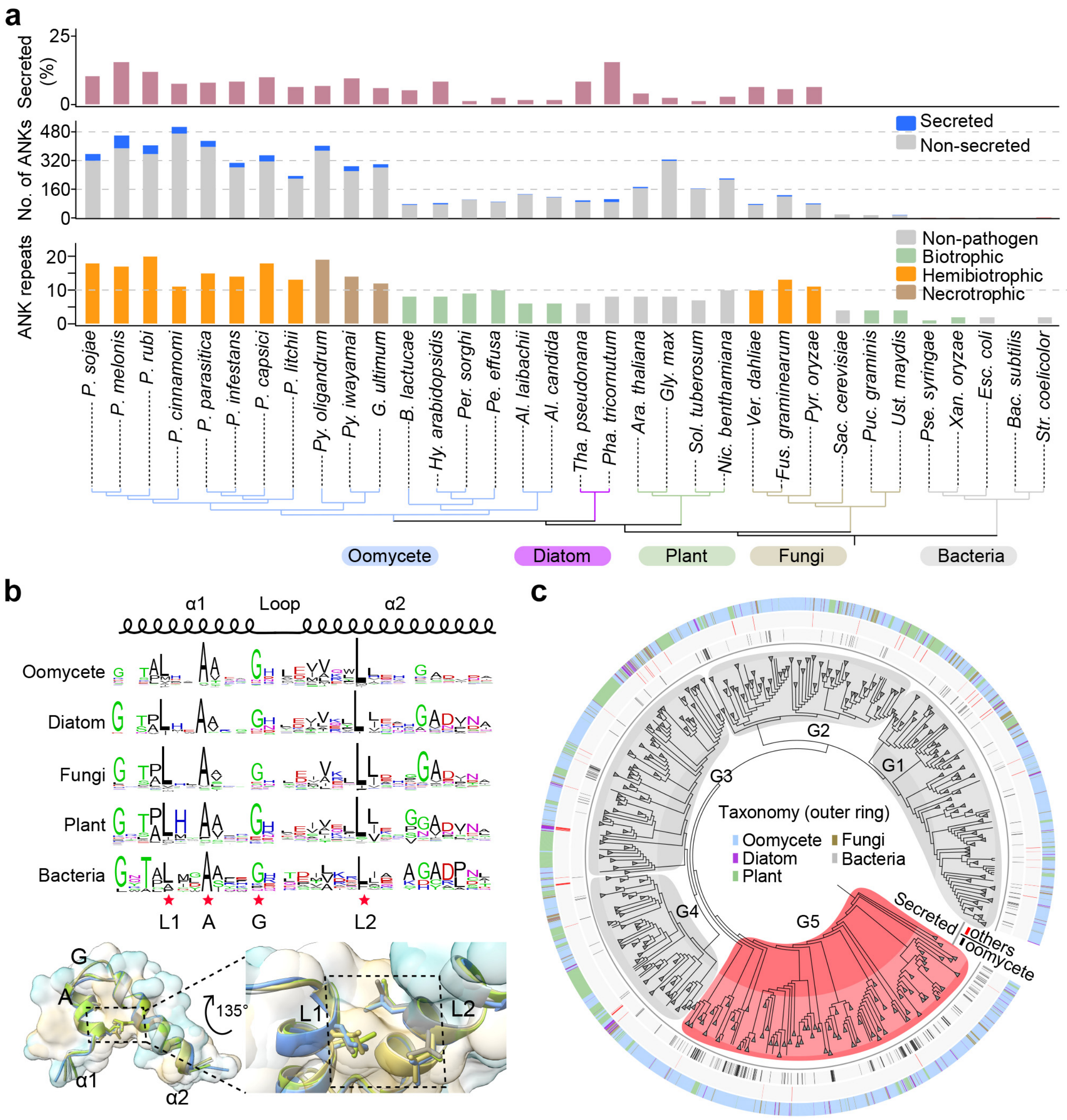

Supplementary Fig. 18 The evolution and expansion of ANK family. a The number of ANK repeat occurrence, the number of ANK genes, and the proportion of secreted-type ANK genes. The branching colors on the phylogenetic tree represent different taxonomic groups. *Pythium* species were adjusted manually based on the distribution of ANK number. **b** The conservation of ANK repeats unit was showed by Weblogo3. The four conserved amino acid residues are marked with red pentagrams and visualized within the protein structure. Hydrophilic amino

acids are represented by a sky-blue color on the protein surface, while hydrophobic amino acids are shown in golden color. **c** The phylogenetic tree was categorized into five groups based on branch topology. The inner ring represents secreted ANK proteins in oomycetes, while the middle ring represents secreted ANK proteins in other species. Source data are provided as a Source Data file.

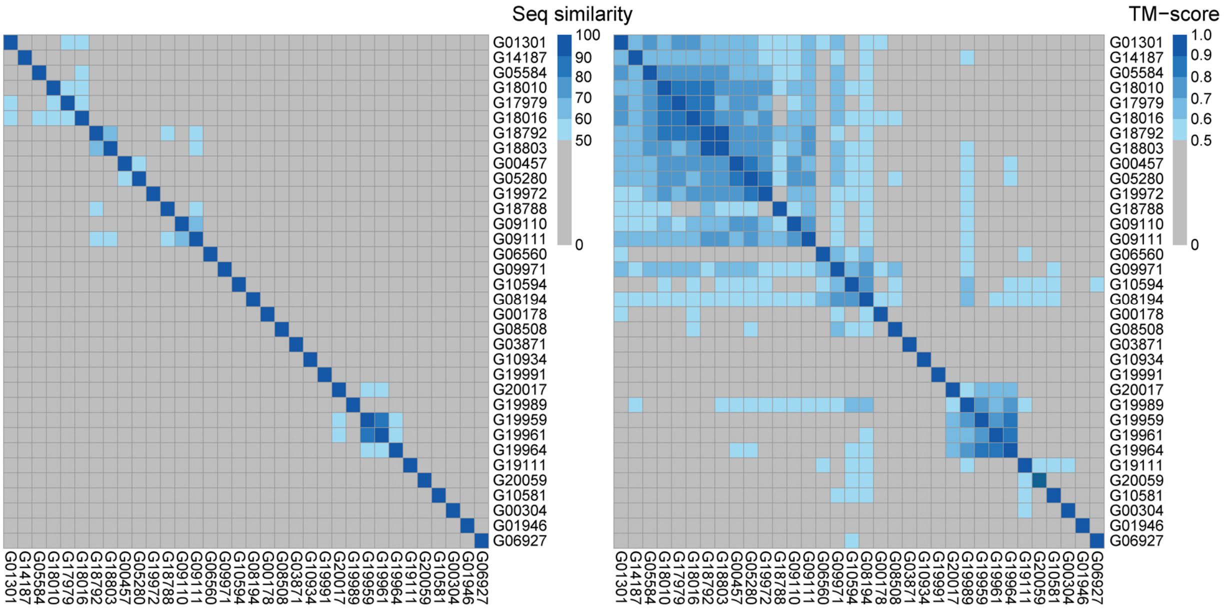

Supplementary Fig. 19 Protein sequence similarity and structural alignment TM-score in putative secreted ANKs. Source data are provided as a Source Data file.

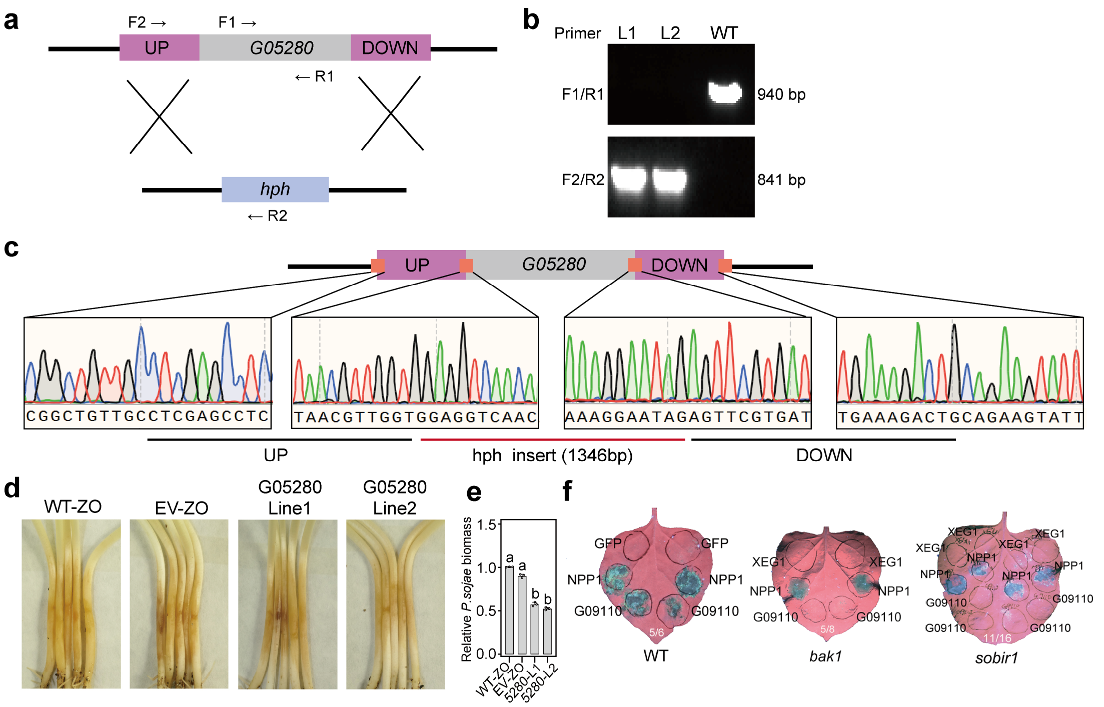

Supplementary Fig. 20 Preliminary verification of virulence and elicitor function of secreted ANKs. a The strategy of *G05280* gene replacement using the CRISPR/Cas9 mediated HDR (Homology Directed Repair). The *G05280* was replaced with *hph* gene (hygromycin B phosphotransferase). **b** PCR analysis demonstrated that *G05280* was completely replaced by the *hph* gene in mutants Line1 (L1) and Line2 (L2). All experiments were repeated three times with similar results. **c** Traces of the Sanger sequencing junction regions confirmed that *G05280* was accurately replaced in the knockout mutant. **d** Phenotypes of lesions on etiolated soybean hypocotyls. A susceptible soybean cultivar (Hefeng 47) was inoculated with approximately 100 zoospores of wild-type (WT) *P. sojae* strain (P6497), the gene knockout failed control strain (EV), and two independent knockout mutants (L1, L2) of *G05280*. The results were checked at 48 hours post infection. **e** *P. sojae* biomass was quantified by fluorescent real-time quantitative PCR (qPCR). The error bars represent SD. Data points were marked in bar plot. *P*-values were calculated by one-way ANOVA ($P<0.05$). Repeat times n=3. **f** Cell death induced in wild-type and *BAK1*- or *SOBIR1*-knockout lines *N. benthamiana*

leaves. Cell death was photographed after three days of agroinfiltration. The experiments at least replicated 3 times, yielding similar results. Source data are provided as a Source Data file.
