# Supplementary material for: Complete telomere-to-telomere genomes uncover virulence evolution conferred by chromosome fusion in oomycete plant pathogens
Source: Nat Commun. 2024 May 30;15:4624. doi: 10.1038/s41467-024-49061-y (PMC11139960; doi:10.1038/s41467-024-49061-y)
Supplement: Supplementary file 3 — Description of Additional Supplementary Files [file 41467_2024_49061_MOESM3_ESM.pdf]

Description of Additional Supplementary Files

File Name: Supplementary Data 1
Description: Comparisons of *P. sojae* 2023, 2019, and 3.0 genome assemblies

File Name: Supplementary Data 2
Description: Whole genome gene annotation of *P. sojae* 2023

File Name: Supplementary Data 3
Description: Genes in fused regions

File Name: Supplementary Data 4
Description: Genes in flexible regions between *P. sojae* 2023 and ancestral genome (A0)

File Name: Supplementary Data 5
Description: Candidate effectors were amplified from *P. sojae*

File Name: Supplementary Data 6
Description: Primer sequence of ANK effectors
