# Supplementary material for: Complete telomere-to-telomere genomes uncover virulence evolution conferred by chromosome fusion in oomycete plant pathogens
Source: Nat Commun. 2024 May 30;15:4624. doi: 10.1038/s41467-024-49061-y (PMC11139960; doi:10.1038/s41467-024-49061-y)
Supplement: Supplementary file 10 — Reporting Summary [file 41467_2024_49061_MOESM10_ESM.pdf]

nature portfolio

Corresponding author(s): Wenwu Ye
Yuanchao Wang

Last updated by author(s): May 6, 2024

Reporting Summary

Nature Portfolio wishes to improve the reproducibility of the work that we publish. This form provides structure for consistency and transparency in reporting. For further information on Nature Portfolio policies, see our Editorial Policies and the Editorial Policy Checklist.

Statistics

For all statistical analyses, confirm that the following items are present in the figure legend, table legend, main text, or Methods section.

n/a	Confirmed	
☐	☒	The exact sample size (*n*) for each experimental group/condition, given as a discrete number and unit of measurement
☐	☒	A statement on whether measurements were taken from distinct samples or whether the same sample was measured repeatedly
☐	☒	The statistical test(s) used AND whether they are one- or two-sided<br>*Only common tests should be described solely by name; describe more complex techniques in the Methods section.*
☒	☐	A description of all covariates tested
☐	☒	A description of any assumptions or corrections, such as tests of normality and adjustment for multiple comparisons
☐	☒	A full description of the statistical parameters including central tendency (e.g. means) or other basic estimates (e.g. regression coefficient) AND variation (e.g. standard deviation) or associated estimates of uncertainty (e.g. confidence intervals)
☐	☒	For null hypothesis testing, the test statistic (e.g. *F*, *t*, *r*) with confidence intervals, effect sizes, degrees of freedom and *P* value noted<br>*Give P values as exact values whenever suitable.*
☒	☐	For Bayesian analysis, information on the choice of priors and Markov chain Monte Carlo settings
☒	☐	For hierarchical and complex designs, identification of the appropriate level for tests and full reporting of outcomes
☐	☒	Estimates of effect sizes (e.g. Cohen's *d*, Pearson's *r*), indicating how they were calculated

Our web collection on statistics for biologists contains articles on many of the points above.

Software and code

Policy information about availability of computer code

Data collection	Qualified libraries were sequenced on the PacBio Sequel II platform. Library construction and PacBio HiFi reads were generated by the Beijing Genomics Institute (https://www.bgi.com/). Whole genome sequencing, de novo assembly and annotation of Phytophthora sojae JS2, Pythium ultimum 18-6, Pythium oligandrum Po34 and Pythium spinosum 2-2 strains using HiFi reads. The genome raw sequencing data generated in this study have been submitted to the NCBI BioProject database under accession number PRJNA910369. 25 re-sequencing isolates reads were downloaded from the BioProject: PRJNA578597. RNA-seq and ATAC-seq data of Phytophthora sojae were downloaded from the NCBI database (BioProject ID: PRJNA426510; PRJNA761250). Genome assembly and GFF annotation files of P. sojae 3.0, Pe. effusa, B. lactucae, P. infestans and P. plurivora were downloaded from NCBI GenBank (GCA_000149755.2; GCA_021491655.1; GCA_004359215.2; GCA_026225685.1; GCA_030027945.1). Protein structures were collected from AlphaFoldDB (https://alphafold.ebi.ac.uk/).
Data analysis	The codes used in this study are openly available at http://gitee.com/biozzc/genome_analysis.<br><br>Hifiasm v0.1433 and HiCanu v2.034 (using default parameters); TBtools v1.098 (MCScanX) and SynVisio website (http://synvisio.github.io/#/); JCVI v1.3.7; pygenomeviz v0.4.4; MUMmer v4; SyRI v1.5.4; AGORA v3.1; OrthoFinder v2.2.7; BWA v0.7.17; SAMtools v1.2; BCFtools v1.14; CNVnator v0.4.1; Circos v0.69; Augustus v3.4.0; BRAKER v2.1.5; RepeatMasker v4.1.0; RepeatModeler v2.0.2; LTR_retriever v2.9.044; LTRharvest (GenomeTools) v1.6.2; R v4.3.1; clusterProfiler v4.0; SignalP v3.0; AlphaFold2; ColabFold v1.5.5; ChimeraX v1.6.1; TM-align (http://zhanggroup.org/TM-align/); Gephi v0.10.1.

For manuscripts utilizing custom algorithms or software that are central to the research but not yet described in published literature, software must be made available to editors and reviewers. We strongly encourage code deposition in a community repository (e.g. GitHub). See the Nature Portfolio guidelines for submitting code & software for further information.

Data

Policy information about availability of data

All manuscripts must include a data availability statement. This statement should provide the following information, where applicable:

- Accession codes, unique identifiers, or web links for publicly available datasets
- A description of any restrictions on data availability
- For clinical datasets or third party data, please ensure that the statement adheres to our policy

Supporting genome and annotation data for this study are available on Zenodo [http://doi.org/10.5281/zenodo.11098592]. The raw sequencing data generated in this study have been deposited in the NCBI BioProject database under accession code PRJNA910369. The newly assembled P. sojae genome in this study has been deposited in the NCBI under accession code PRJNA1106983. RNA-seq and ATAC-seq data used in this study are under BioProject accession codes PRJNA426510 and PRJNA761250, respectively. Genome assembly and GFF annotation files were downloaded from NCBI. GenBank assembly accessions are listed as follows: GCA_009848525.1, GCA_000149755.2, GCA_021491655.1, GCA_004359215.2, GCA_026184515.1, GCA_026225685.1, and GCA_030027945.1. CENPA ChIP-seq data used in this study are under BioProject accession code PRJNA563922.

Research involving human participants, their data, or biological material

Policy information about studies with human participants or human data. See also policy information about sex, gender (identity/presentation), and sexual orientation and race, ethnicity and racism.

Reporting on sex and gender	N/A
Reporting on race, ethnicity, or other socially relevant groupings	N/A
Population characteristics	N/A
Recruitment	N/A
Ethics oversight	N/A

Note that full information on the approval of the study protocol must also be provided in the manuscript.

Field-specific reporting

Please select the one below that is the best fit for your research. If you are not sure, read the appropriate sections before making your selection.

☒ Life sciences ☐ Behavioural & social sciences ☐ Ecological, evolutionary & environmental sciences

For a reference copy of the document with all sections, see nature.com/documents/nr-reporting-summary-flat.pdf

Life sciences study design

All studies must disclose on these points even when the disclosure is negative.

Sample size	No sample size calculations were performed. Sample sizes were determined based on previous experience (Qiu M et al. G protein α subunit suppresses sporangium formation through a serine/threonine protein kinase in Phytophthora sojae. PLoS Pathog. 2020;16(1):e1008138). At least three biological replicates were performed in all experiments. Three replicates are reasonable as they allow for estimation of variability and statistical analysis. The exact sample size and number of replicates are shown in the figure legend and/or the methods section.
Data exclusions	No data were excluded from the analyses.
Replication	Experiments were repeated three times with similar results.
Randomization	In all the analyses, selected data points were randomized.
Blinding	Blinding was not relevant to our study. We conducted research related to genome evolution. Data collection and analysis were not influenced by subjective factors, therefore, no single-blind or double-blind experiments were conducted.

Reporting for specific materials, systems and methods

We require information from authors about some types of materials, experimental systems and methods used in many studies. Here, indicate whether each material, system or method listed is relevant to your study. If you are not sure if a list item applies to your research, read the appropriate section before selecting a response.

Materials & experimental systems

n/a	Involved in the study	
☐	☒	Antibodies
☒	☐	Eukaryotic cell lines
☒	☐	Palaeontology and archaeology
☒	☐	Animals and other organisms
☒	☐	Clinical data
☒	☐	Dual use research of concern
☐	☒	Plants

Methods

n/a	Involved in the study	
☒	☐	ChIP-seq
☒	☐	Flow cytometry
☒	☐	MRI-based neuroimaging

Antibodies

Antibodies used	anti-GFP (Mouse antibody; 1:5000; Abmart #M20004L))
Validation	Anti-GFP was used to validate the expression of tagged proteins in N. benthamiana.

Plants

Seed stocks	All plant seeds including the wild type and mutants were stocked in our lab.
Novel plant genotypes	No novel plant genotypes generated.
Authentication	The plant mutants generated by CRISPR-Cas9 were identified by genome-based PCR and Sanger sequencing.
