# Supplementary material for: Complete telomere-to-telomere genomes uncover virulence evolution conferred by chromosome fusion in oomycete plant pathogens
Source: Nat Commun. 2024 May 30;15:4624. doi: 10.1038/s41467-024-49061-y (PMC11139960; doi:10.1038/s41467-024-49061-y)
Supplement: Supplementary file 11 — Source Data [file 41467_2024_49061_MOESM11_ESM.zip › Zhang_et_al_SourceData/Fig. 2.zip!/Fig. 2/Fig.2a_configure_files.docx]

Files for the phylogenetic analyses of 24 single-copy gene sets.

**Contents**

#1. Files related to the phylogenetic analysis of the seven marker genes

Pages 2-49: This section contains the alignments of seven marker genes—*COX2*, nrLSU, *HSP90*, *EF1A*, *60S*, *ENOLASE*, and *β-TUB*—utilized in the phylogeny calculations (PHYLIP format).

Page 50: Presented here is the initial partitioning scheme configuration file, employed to initiate the analysis in PartitionFinder.

Page 51: Featured in this part is the refined partitioning scheme recommended by PartitionFinder. This optimized scheme was subsequently applied in constructing the phylogeny with IQ-TREE.

#2. Files related to the phylogenetic analysis of 24 single-copy genes

Pages 52-250: This segment provides the alignments of 24 single-copy genes that were analyzed to compute the phylogeny (PHYLIP format).

Pages 251-252: Here, the original partitioning scheme’s configuration file is documented, which was utilized to start the analysis in PartitionFinder.

Page 253: This page outlines the optimized partitioning scheme as advised by PartitionFinder, which was implemented in the construction of the phylogeny using IQ-TREE.

38 5228

Peronospora_belbahrii TTTTTTAATTATTGTTACTGTATTTGTATGTTGGATGTTATTTAGAGTTATTACTCTTTTTAATGAAAAAAAAAATAAAATACCAGCAACTGTAGTACATGGAGCTACTATTGAAATTATTTGGACTTCAATACCAGCTTTAATTTTATTAATGGTTGCAATACCGTCTTTTGCTTTATTATATTCAATGGATGAAGTTATTGACCCTATTATTACTTTAAAAGTAATTGGAAATCAATGGTATTGGAGTTATGAATATTCCGATAATTTAGAATTTTCTGATGAACCTTTAATTTTTGATAGTTATATGGTACAAGAAGAGGATTTAGCTATAGGTCAATTGAGACTTTTAGAAGTAGATAATCGTGTAATAGTGCCAACTAATAGTCATATTAGAGTATTAATTACTGCATCGGATGTTTTACATTCATGGGCTATTCCTTCATTAGGTATAAAATTAGATGCATGTCCGGGACGTTTAAATCAAACCGGCGAGTGAAGCGGGATGAGCTCAAGCTTAAAATCTCTGTACAAGTTTTGTATGGCGAATTGTAGTCTATAGAGGC-GTGATCAGTGTAGGCACTTGGGGTAAGTTCCTTGGAAGAGGACAGCATGGAGGGTGATACTCCCGTTTATC-CCTAAGTAGCTTATGCGTACGATCCGTTTTCTTTGAGTCGCGTTGTTTGGGAATACAGCGCAAAGTAGGTGGTAAATTCCATCTAAAGCTAAATATTGGTGCGAGACCGATAGCAAACAAGTACCGTGAGGGAAAGATGAAAAGAACTTTGAAAAGAGAGTTAAAGAGTACCTGAAACTGCTGAAAGGGAACCGAATCGTTTCCAGTGTCTATAATCCGTGGTATATTTCATTGGTTGATGTGCAGATGCATATGTTATGGCAGTGGCTTTTT---GGCTGCACCTTGCATG---TGTTAGTATGTGCGTTTGCTGGTGCCCTGTACTGCGGTGGGACGTCAATGTAAGTTCGTATACTGCGGGAAATGGCTGCTGAGGAGGTAGGGCTTACGCT-TGCGTTTGTCTGTTATATCTTGGTGGACT-AGTAGTCGTGGTTGGGACTGAGGTG-CCTACAACGTGCTTTTGAGTGGGATTGTGTCTCCGTGTGCGCCGTGTATAGATAGCTTGCTATGTATATGT---GGTTGTGTGTGGATTGATGCGAGCTTTAACTTGTTGCCGTTCATTCCGGTATTGGTATGACGAAGGCGGATTTGATTAATAATCTCGGTACGATTGCAAAGTCAGGCACTAAGGCGTTCATGGAAGCATTGGCTGCTGGTGCAGATATTAGTATGATTGGTCAGTTTGGTGTGGGGTTTTACTCTGCCTACTTGGTAGCTGATCAAGTGATTGTGCACTCGAAGCATAATGATGACGAGCAATATGTGTGGGAATCTGCTGCGGGTGGTTCGTTTACGGTGACCCCAGAC---ACATCGGAGCCTATCCCACGTGGTACACGCATTGTGTTGAAACTAAAGGAAGATATGCTTGAGTATTTGGAAGAACGCAAGCTTAAAGACCTTGTGAAGAAACACTCTGAGTTTATTGGTTTCCCTATCAAGCTTTACGTCGAAAAGACGGAGGAGAAGGAAGTGACGGATGATGAGGAGGAAGAAGAAG---AGAAGGAAGGTGAGGATGACAAGCCAAAGGTCGAAGAGGTGGAGGAAGAGGAAGGTGATGAAAAGAAGAAGAAGACGAAAAAGGTCAAGGAAGTGACGCACGAGTGGGACCACTTGAATAGCCAGAAACCTATTTGGATGCGCAAACCCGAGGATGTAACTCACGAGGAGTATGCTTCGTTCTACAAGTCGCTTACTAATGACTGGGAGGAACATGCTGGTGTCAAGCACTTTTCGGTGGAGGGACAGCTAGAGTTTAAGGCTTGTTTGTTCACGCCGAAGCGCGCTCCCTTTGACATGTTTGAGGGTGGTGCCAAGAAGAAACTCAACAACATTAAGTTGTATGTGCGTCGTGTGTTCATCATGGACAACTGCGAAGAGCTCATGCCTGAGTATCTGTCATTTGTAAAGGGCGTTGTCGACTCGGAAGATTTGCCGCTAAACATTTCGCGTGAGACGCTGCAGCAGAACAAGATCTTGCGTGTGATTAAGAAGAACCTGGTCAAGAAATGCCTGGAGATGTTTGCCGAGCTTGCTGAGGACAACGAGAAGTACAACAAGTTCTATGAGGCATTCAGCAAGAACTTA-AAGTTGGGCATCCACGAGGACTCGACCAACCGCACGAAGATTGCGAAGCTATTGCGCTACCATTCGACCAAGTCTGGTGAGGAGATGACGTCTTTGGACGACTACATCTCGCGCATGCCTGAGAACCAACCCGGTATTTATTATGTAACGGGTGAGAGCAAGAAGTCAGTGGAGAACTCGCCATTCATTGAGAAGCTTAAGAAGAAGGGATACGAGGTATTGTTCATGGTGGAGGCCATTGACGAGTACGCCGTGCAGCAGCTGAAGGAGTACGAGGGCAAGAAGCTGATTTGTGCGACCAAGGAGGGTCTCAAGATGGAAGAGACGGAGGATGAGAAGAAGTCGTTCGAGGAGGCCAAGGCTGCCACTGAAGGTCTTCACGGTTATTGACGCCCCTGGCCACCGTGATTTTATCAAGAACATGATTACGGGCACATCTCAGGCCGATTGCGCTATCCTTGTGGTAGCTTCGGGGGTGGGTGAATTTGAGGCTGGTATCTC---GAAGGAGGGCCAGACGCGCGAGCACGCTCTGTTGGCCTTTACACTTGGTGTAAAGCAGATGGTTGTGGCTATTAACAAGATGGACGACTCGTCTGTCATGTACGGCCAGGCTCGTTACGAGGAAATTAAGGCGGAGGTGACGACTTACCTGAAGAAGGTTGGCTACAAACCCGCTAAGATTCCGTTTGTGCCTATTTCGGGCTGGGAGGGTGACAACATGATTGACCGCTCGCCTAACATGGCGTGGTACAAGGGACCTTACCTTTTGGAGGCTCTTGACAACCTAAACGCGCCGAAGCGTCCATCGGACAAGCCTCTACGTCTTCCTCTCCAGGACGTTTACAAGATTGGCGGTATTGGTACGGTCCCAGTTGGCCGTGTAGAGACCGGTGTTATCAAGCCTGGTATGGTCGCTACATTTGGCCCCGTGGGTCTGTCGACGGAAGTCAAGTCTGTCGAGATGCACCACGAGTCTTTACCTGAGGCTCTTCCTGGTGACAACGTTGGCTTCAATGTCAAGAACGTGTCGGTGAAGGAGCTTCGTCGTGGTTTTGTTGCTTCGGACTCGAAGAACGA---CCCTGCGAAGGGCACGCAGGACTTTACGGCTCAGGTCATTGTGCTGAACCACCCTGGCCAAATTGGCAACGGTTACTCACCAGTGCTTGACTGCCACACGGCCCATGTTGCGTGCAAGTTCAAAGAGATTACGGAGAAGATGGACCGTCGTTCGGGTAAAATTCTTGTCCAGAACAAGAAACCCTTCATTAAATCACGTTATTGTCGTGGTGTACCTGATCCAAAAATTCGTATCTATGATGTTGGTAATAAAAAAGCATCCGTTGATACATTTCCATTTGTTGCTCATTTAGTATCAGATGAGAAGGAACAATTATCATCGGAAGCTCTTGAAGCTGCTCGTATTGCTGCTAATCGTTACTTGACGAAATATTGTGGCAAAGATAATTTTCATATGCGCATTCGTTGCCATCCGTTCCAAGTTTTACGTATCAACAAGATGCTTTCATGTGCTGGTGCTGATAGACTGCAAACGGGTATGCGTCACGCTTATGGTAAACCTGCTGGTGTTGCTGCGCGTGTAGCTATTGGTCAGCCAATTATCTCTGTGCGTTCGAAGGATTCATTTGGTCCATCGGTTGTTGAAGCTCTACGTCGTGCACGCTCCCGAGTCGGAAAAGATTTCGGCTGAAAAGCTGCTCGAGGTGTACGAGGGATTTATTGCAAAGTGCGCTGGATCGAGCAAAATTGTATCGATTGAGGATCCGTTCGATCAGGATGACTGGGAATCATGGATGAAGATAACGGAAAAGGTTGGCAAGGACGTGCAGATCGTGGGAGACGACCTGACGGTGACGAACCCGACCCGTGTGAAGAAAGCCATCGAGCAAAAGGCGTGCAATGCTCTCTTGCTGAAAGTGAACCAGATTGGCAGTATCACCGAGTCAATTGAGGCCGTGACGATGGCCAAGAAGGCTGGTTGGGCCATCATGACCAGCCATCGCAGTGGTGAGACGGAGGACACATTTATCGCTGATCTGGCTGTGGGTCTAAGCGCTGGCCAGATGGTAACAACTGGGCCAAGGGGCACTACACTGAGGGCGCCGAGCTCATTGACTCGGTGTTAGATGTTGTCCGCAAGGAGGCGGAAAGTTGTGACTGTTTGCAGGGTTTTCAAATCACGCACTCTCTTGGTGGCGGTACTGGCTCCGGTATGGGAACGCTTCTGATTTCGAAGATTCGAGAAGAGTATCCGGACCGTATCATGTGTACATACTCGGTGTGCCCGTCCCCGAAGGTGTCAGACACAGTTGTGGAGCCCTATAACGCCACGTTGTCTGTGCATCAGCTCGTCGAGAACGCCGATGAGGTCATGTGCCTTGACAACGAGGCCTTGTATGACATTTGCTTCCGTACGTTGAAGCTGACTACTCCCACTTATGGCGATTTGAACCACTTGGTGTGTGCTGCTATGTCTGGCATCACTACTTCTTTACGATTCCCTGGACAGCTGAATTCGGACCTGCGGAAACTGGCTGTGAACTTGATCCCGTTTCCGCGTCTCCACTTTTTTATGATTGGTTTTGCCCCGCTGACATCGCGTGGTTCTCAGCAGTACCGTGCTTTGACGGTGCCCGAGCTGACGCAGCAGCAATTTGATGCTAAGAACATGATGTGTGCCGCCGATCCTCGGCACGGCCGTTATTTAACTGCTGCCTGTATGTTCCGAGGACGTATGAGCACGAAGGAGGTGGATGAACAAATGTTGAACGTGCAAAACAAGAATTCATCGTACTTTGTCGAATGGATTCCCAACAACATCAAGGCTAGCGTGTGTGACATTCCACCCAAGGGCCTAAAGATGAGCACTACCTTTATCGGTAACTCAACAGCCATTCAAGAGATGTTCAAGCGTGTTTCTGAACAGTTTACGGCCATGTTCCGACGCAAGGCTTTCTTGCACTGG

Peronospora_effusa TTTTTTAATTATCGTTACTGTATTTGTTTGTTGGATGTTATTTAGAGTAATTACTCTTTTTGATGAAAAAAAAAATAAAATTCCAGCAACAGTAGTACACGGAGCTACTATTGAAATTATTTGGACTTCTATTCCAGCTTTAATTTTATTAATGGTTGCAGTACCTTCTTTTGCGTTATTATATTCAATGGATGAAGTAATTGATCCTATTATCACATTAAAAGTAATTGGTAATCAATGGTATTGGAGTTACGAGTATTCAGATAATCTAGAGTTTTCAGATGAACCTTTAATTTTTGATAGTTATATGGTACAAGAAGATGATTTAGCTATAGGCCAATTCCGACTTTTAGAAGTAGATAATCGTGTAGTAGTTCCCACTAATAGTCATATTAGAATATTAATTACTGCTTCTGATGTTTTACATTCATGGGCTATCCCATCATTAGGTATTAAATTGGATGCCTGTCCTGGACGTTTAAATCAAACCGGCGAGTGAAGCGGGATGAGCTCAAGCTTAAAATCTCCGTACAAGTTTTGTATGGCGAATTGTAGTCTATAGAGGC-GTGGTCAGCGTGGGCGCTTGGGGCAAGTTCCTTGGAAGAGGACAGCATCGAGGGTGATACTCCCGTTCATC-CCTGAGTGGCTCGCGCGTACGACCCGTTTTCTTTGAGTCGCGTTGTTTGAGAATGCAGCGCAAAGTAGGTGGTAAATTCCATCTAAAGCTAAATATTGGTGCGAGACCGATAGCATACAAGTACCGTGAGGGAAAGATGAAAAGAACTTTGAAAAGAGAGTTAAAGAGTACCTGAAACTGCTGAAAGGGAACCGAATCGTTTCCAGTGTCTATAATCCGTGACATATTTCATTGGCGAGTGTGTGCGTGCGTGTGCTATGGCAGCGGCCTTTTT--GGCTGCGCTTGGCGTG---TG-TGCTGTGTGTGCTTGCTGGTGCCCTGTGTTGCGGTGGGACGTCAATGTCAGTTCGTATGCTGCGGGAAATGGCTGCCGAGGAGGTAGGGCTTACGCT-TGCGTTTGTCTGTTATATCTTGGTGGACT-AGTCGTCGCGGTTGGGACTGAGGTG-CCTACAACGTGCTTTTGTGTGGGTTTGTGTCTCCGTGTGTACCGTGTGCGGATAGCTTGCTATGTGTGTGT---GGTTATGTATGGATTGATGTAGACTCTAACTTGTTGCCGTTCACTCTGGTATTGGTATGACTAAGGCTGATCTTATTAATAACTTGGGTACGATCGCCAAGTCGGGTACCAAGGCCTTCATGGAGGCATTGGCTGCCGGTGCTGACATCAGTATGATTGGTCAGTTTGGTGTCGGGTTTTACTCTGCCTATCTCGTGGCTGACCAAGTGGTTGTCCACTCGAAGCACAATGATGACGAGCAGTACGTGTGGGAATCTGCTGCCGGTGGATCATTCACTGTGAAGCCTGAC---ACATCGGAGCCTATTCTACGTGGTACTCGCATTGTATTGAAGTTGAAGGAGGATATGCTGGAGTACTTGGAAGAGCGCAAGTTGAAAGACTTGGTGAAGAAACACTCGGAGTTTATTGGCTTCCCTATCAAGCTTTACGTTGAGAAGACGGAGGAGAAGGAAGTGACGGATGATGAAGAGGAGGAAGAAG---AGAAGGAGGGTGAAGATGACAAGCCCAAGGTAGAGGAAGTTGATGAGGAGGAGGAAGGTGAGAAGAAGAAGAAGACGAAGAAGATTAAGGAAGTGACCCACGAGTGGGACCACTTGAACAGCCAGAAACCTATCTGGATGCGTAAACCTGAGGACGTGACCCACGAAGAATACGCTTCGTTCTACAAGTCGCTTACCAACGACTGGGAGGAACACGCTGATGTGAAGCACTTTTCGGTGGAAGGTCAGCTTGAATTCAAGGCTTGTCTGTTTACCCCCAAGCGCGCGCCATTCGACATGTTTGAGGGCGGTGCCAAGAAGAAAGTGAACAACATTAAATTGTATGTGCGTCGCGTGTTCATCATGGACAACTGCGAAGACCTCATGCCTGAGTACCTGTCATTCGTCAAGGGTGTCGTTGACTCGGAGGACTTGCCGCTGAACATTTCGCGTGAAACGTTGCAGCAGAACAAGATTTTGCGTGTGATTAAGAAGAACTTGGTCAAGAAGTGCCTCGAGATGTTTGCGGAGCTGGCTGAGGACAATGAGAAGTACAACAAGTTCTATGAGGCCTTTAGCAAGAACCTG-AAGCTGGGCATCCACGAAGACTCGACGAACCGCACGAAGATTGCCAAGCTGCTGCGTTACTACTCGACCAAGTCTGGCGAAGAGACGACGTCTTTGGACGACTACATTTCGCGCATGCCGGACAACCAGCCTGGTATTTACTACGTGACGGGCGAGAGCAAAAAGTCGGTCGAAAACTCGCCGTTCATTGAGAAGCTCAAGAAGAAGGGATACGAGGTGCTGTTCATGGTAGAGGCCATTGACGAGTACGCCGTGCAGCAGTTGAAGGAGTACGAGGGTAAGAAGCTTATCTGCGCCACCAAGGAAGGCCTCAAGATGGAGGAGACGGAGGACGAGAAGAAGTCGTTCGAGGAGGCGAAGGCTGCCACTGAGGGTCTTTACAGTCATTGACGCTCCTGGTCACCGTGACTTTATCAAGAATATGATTACGGGCACGTCGCAGGCTGATTGCGCCATTCTAGTGGTTGCTTCAGGTGTTGGTGAGTTCGAGGCTGGTATTTC---CAAGGAAGGTCAGACACGTGAGCACGCTCTATTGGCCTTTACGCTTGGTGTAAAGCAGATGGTTGTGGCTATTAACAAGATGGACGACTCGTCTGTCATGTACGGCCAGGCTCGTTACGAGGAGATTAAGGCCGAGGTTACTACTTACCTGAAGAAGGTTGGCTACAAACCTGCCAAGATCCCATTCGTGCCTATCTCTGGATGGGAAGGAGACAACATGATTGACCGCTCTGCAAACATGCCGTGGTACAAGGGACCTTACCTCCTTGAGGCTCTTGACAGTCTGAACGCGCCTAAGCGCCCGTCGGACAAACCTCTTCGTCTGCCTCTTCAGGACGTGTACAAGATTGGCGGTATTGGCACGGTTCCGGTTGGCCGTGTCGAGACTGGTGTCATTAAGCCTGGCATGGTCGCTACGTTCGGTCCCGTTGGTCTGTCGACGGAAGTCAAGTCCGTTGAGATGCACCACGAGTCTTTGCCGGAGGCTCTTCCTGGTGACAACGTTGGCTTCAACGTCAAAAACGTGTCGGTAAAGGAACTTCGTCGTGGTTTTGTCGCTTCGGACTCGAAGAACGA---CCCTGCTAAGGGCACCCAGGACTTCACGGCCCAGGTGATTGTGCTGAACCACCCTGGCCAAATCGGTAACGGCTACTCGCCAGTGCTTGACTGCCACACTGCCCACGTTGCCTGCAAGTTCAAAGAGATCATTGAGAAGATGGACCGTCGTTCGGGCAAGGTGCTTGTCCAGAACAAGAAGCCGTTTATTAAATCGCGTTACTGTCGTGGTGTGCCTGACCCAAAGATTCGTATCTATGATGTTGGTAATAAGAAAGCTTCAGTGGATACTTTTCCATTCGTAGCTCACTTGGTATCGGACGAGAAGGAACAGCTCTCCTCGGAAGCTCTTGAAGCTGCACGTATTGCTGCGAATCGTTACTTGACGAAATATTGTGGCAAGGACAATTTCCATATGCGCATTCGATGCCATCCATTCCAGGTCCTACGTATCAACAAGATGCTTTCATGTGCTGGTGCTGATAGACTTCAGACGGGTATGCGTCACGCATATGGTAAACCCGCTGGTGTAGCCGCCCGTGTGGCTATTGGTCAGCCGATTATTTCGGTACGTTCAAAGGATTCGTTTGGTCCTTCGGTCGTTGAAGCTCTTCGTCGTGCACGCTTCCGAGTCAGAGAAGATCTCGGCTGAGAAGCTTCTGGAGGTGTACGAGGGCTTTATCGCGAAGTGCGCCGGGTCGAGCAGGATCGTGTCGATTGAGGACCCGTTCGACCAGGACGACTGGGAGTCGTGGATGAAGATTACGGAAAAGGTTGGCAAGGACGTGCAGATCGTGGGAGACGACCTGACGGTGACGAACCCGACCCGTGTGAAGAAGGCCATTGAGCAGAAGGCTTGCAACGCTCTTCTGCTAAAAGTGAACCAGATCGGCAGCATCACGGAGTCGATTGAGGCCGTGACGATGGCCAAGAAGGCCGGTTGGGCTATCATGGCTAGCCACCGCAGTGGCGAGACGGAGGACACGTTCATCGCTGATCTGGCTGTTGGTCTGAGTGCCGGCCAGATGGTAACAATTGGGCTAAGGGACACTACACTGAGGGCGCCGAGCTCATTGACTCCGTTCTGGACGTCGTCCGTAAGGAGGCGGAAAGCTGTGACTGTTTGCAGGGTTTCCAGATCACGCACTCGCTTGGTGGCGGTACCGGCTCCGGTATGGGAACGCTTCTTATCTCAAAGATCCGAGAAGAGTACCCGGACCGTATCATGTGCACGTACTCGGTATGTCCATCTCCCAAGGTGTCGGACACTGTTGTAGAGCCCTACAATGCTACGCTGTCTGTACATCAGCTTGTCGAAAACGCCGATGAGGTCATGTGCCTGGATAATGAGGCCTTGTACGACATTTGCTTCCGTACGCTGAAGCTCACCACCCCCACCTATGGTGACCTGAACCACTTGGTGTGTGCCGCTATGTCGGGCATCACCACTTCCCTACGATTTCCGGGACAGCTGAACTCGGACCTGCGGAAGCTGGCCGTGAACCTGATCCCGTTCCCTCGTCTTCACTTCTTCATGATTGGTTTCGCGCCGCTGACGTCACGTGGTTCGCAGCAGTACCGTGCCTTGACGGTGCCTGAGCTGACACAGCAGCAGTTTGATGCTAAAAACATGATGTGCGCCGCCGACCCTCGCCACGGTCGCTATTTAACTGCCGCTTGTATGTTCCGCGGACGCATGAGCACGAAGGAGGTTGATGAGCAAATGCTGAACGTGCAGAACAAAAACTCGTCGTACTTTGTCGAGTGGATTCCTAACAACATCAAGGCTAGTGTGTGTGACATCCCACCCAAGGGCCTGAAGATGAGCACCACCTTTATTGGTAATTCGACAGCCATCCAAGAGATGTTCAAGCGTGTGTCTGAACAGTTCACGGCTATGTTCCGTCGTAAGGCTTTCTTGCATTGG

Peronospora_farinosa TTTTTTAATTATTGTTACTGTATTTGTTTGTTGGATGTTATTTAGAGTAATTACTCTTTTTGATGAAAAAAAAAATAAAATTCCAGCAACAATTGTGCATGGAGCTACTATTGAAATTATTTGGACTTCTATTCCAGCTTTAATTTTATTAATGGTTGCAGTACCTTCTTTTGCTTTATTATATTCAATGGATGAGGTCATTGATCCTATCATTACATTAAAAGTAATTGGTAATCAATGGTATTGGAGTTACGAATATTCAGATAATTTAGAATTTTCAGAAGAACCTTTAATTTTTGATAGTTATATGGTACAAGAGGATGATTTAGCTATAGGTCAATTTCGACTTTTAGAAGTAGATAATCGTGTAGTGGTTCCAACTAATAGTCATATTAGAATATTAATTACTGCTTCAGATGTTTTACATTCATGGGCTATCCCTTCATTAGGTATTAAATTAGATGCCTGTCCTGGACGTTTAAATCAAACCGGCGAGTGAAGCGGGATGAGCTCAAGCTTAAAATCTCCATACAAGTTTTGTATGGCGAATTGTAGTCTATAGAGGC-GTGGTCAGCGTGGGCGCTTGGGGCAAGTTCCTTGGAAGAGGACAGCATCGAGGGTGATACTCCCGTTCATC-CCTGAGTGGCTCGTGCGTACGACCCGTTTTCTTTGAGTCGCGTTGTTTGAGAATGCAGCGCAAAGTAGGTGGTAAATTCCATCTAAAGCTAAATATTGGTGCGAGACCGATAGCATACAAGTACCGTGAGGGAAAGATGAAAAGAACTTTGAAAAGAGAGTTAAAGAGTACCTGAAACTGCTGAAAGGGAACCGAATCGTTTCCAGTGTCTATAATCCGTGACATATTTCATTGGCGAGTGTGTGCGTGCGTGTGCTATGGCAGCGGCCTTTTT--GGCTGCGCTTGGCGTG---TG-TGCTGTGTGTGCTTGCTGGTGCCCTGTGTTGCGGTGGGACGTCAATGTCAGTTCGTATGCTGCGGGAAATGGCTGCCGAGGAGGTAGGGCTTACGCT-TGCGTTTGTCTGTTATATCTTGGTGGACT-AGTCGTCGCGGTTGGGACTGAGGTG-CCTACAACGTGCTTTTGTGTGGGTTTGTGTCTCCGTGTGTACCGTGTGCGGATAGCTTGCTATGTGTGTGT---GGTTATGTATGGATTGATGTAGACTCTAACTTGTTGCCGTTCACTCTGGTATTGGTATGACCAAGGCTGATCTTATTAATAACTTGGGCACGATCGCCAAGTCGGGTACCAAGGCCTTCATGGAGGCATTGGCTGCCGGTGCTGACATCAGTATGATTGGTCAGTTTGGTGTCGGGTTTTACTCTGCCTATCTCGTGGCTGACCAAGTGGTTGTCCACTCGAAGCACAATGATGACGAGCAGTACGTGTGGGAATCTGCTGCCGGTGGATCATTCACTGTGAAGCCTGAC---ACATCGGAGCCCATCCTACGTGGTACTCGCATTGTATTGAAGTTGAAGGAGGATATGCTGGAGTATTTGGAAGAGCGCAAGTTGAAAGACTTGGTGAAGAAACACTCGGAGTTTATTGGCTTCCCTATCAAGCTTTACGTTGAGAAGACGGAGGAGAAGGAAGTGACGGACGATGAAGAGGAGGAAGAAG---AGAAGGAGGGTGAAGATGACAAGCCCAAGGTAGAGGAAGTTGACGAGGAGGAGGAAGGTGAGAAGAAGAAGAAGACGAAGAAGATTAAGGAAGTGACCCACGAGTGGGACCACTTGAACAGCCAGAAACCTATCTGGATGCGTAAACCTGAGGACGTGACCCACGAAGAATACGCTTCGTTCTACAAGTCGCTTACCAACGACTGGGAGGAACACGCTGCTGTGAAGCACTTTTCGGTGGAAGGTCAGCTTGAATTTAAGGCTTGTCTGTTTACCCCCAAGCGCGCGCCATTCGACATGTTTGAGGGCGGTGCCAAGAAGAAAGTGAACAACATTAAATTGTATGTGCGTCGCGTGTTCATCATGGACAACTGCGAAGACCTCATGCCTGAGTACCTGTCGTTCGTCAAGGGTGTCGTTGACTCGGAGGACTTGCCGCTGAACATTTCGCGTGAGACGTTGCAGCAGAACAAGATATTGCGTGTGATTAAGAAGAACTTGGTCAAGAAGTGCCTCGAGATGTTTGCGGAGCTTGCTGAGGACAATGAGAAGTACAACAAGTTCTATGAGGCCTTTAGCAAGAACCTG-AAGCTGGGCATTCACGAAGACTCGACGAACCGCACGAAGATTGCCAAGCTGCTGCGTTACTACTCGACCAAGTCTGGCGAAGAGACGACGTCTTTGGACGACTACATTTCGCGCATGCCGGACAATCAGCCTGGTATTTACTACGTGACGGGCGAGAGCAAAAAGTCGGTCGAAAACTCGCCGTTTATTGAGAAGCTCAAGAAAAAGGGATACGAGGTGCTGTTCATGGTAGAGGCCATCGACGAGTACGCCGTGCAGCAGTTGAAGGAGTACGAGGGTAAGAAGCTTATCTGCGCCACCAAGGAAGGCCTCAAGATGGAGGAGACGGAGGACGAGAAGAAGTCGTTCGAGGAGGCGAAGGCTGCCACTGAGGGTCTTTACGGTCATTGACGCTCCTGGTCACCGTGACTTTATCAAGAATATGATTACGGGCACGTCGCAGGCTGATTGCGCCATTCTAGTGGTTGCTTCAGGTGTTGGTGAGTTCGAGGCTGGTATTTC---CAAGGAAGGCCAGACACGTGAGCACGCTCTATTGGCCTTTACGCTTGGTGTGAAGCAGATGGTTGTGGCTATTAACAAGATGGACGACTCGTCTGTCATGTACGGCCAGGCTCGTTACGAGGAGATTAAGGCCGAGGTTACTACTTACCTGAAGAAGGTTGGCTACAAACCTGCCAAGATCCCATTCGTGCCTATCTCTGGATGGGAAGGAGACAACATGATTGACCGCTCCGCAAACATGCCGTGGTACAAGGGACCTTACCTCCTTGAGGCCCTTGACAGTCTAAACGCGCCTAAGCGCCCGTCGGACAAGCCTCTTCGTCTACCTCTTCAGGACGTGTACAAGATTGGCGGTATTGGCACGGTACCGGTTGGCCGTGTTGAGACTGGTGTCATTAAGCCTGGTATGGTCGCTACGTTCGGTCCCGTTGGTCTGTCGACGGAAGTCAAGTCCGTTGAGATGCACCACGAGTCTTTGCCGGAGGCTCTTCCTGGTGACAACGTTGGTTTCAACGTCAAGAACGTGTCGGTAAAGGAACTGCGTCGTGGTTTCGTCGCTTCGGACTCGAAGAACGA---CCCTGCTAAGGGCACCCAGGACTTCACGGCCCAGGTGATTGTGCTGAACCACCCTGGCCAAATCGGTAACGGCTACTCGCCAGTGCTTGACTGCCACACTGCCCACGTTGCCTGCAAGTTCAAAGAGATCATTGAGAAGATGGACCGTCGTTCGGGCAAGGTGCTTGTCCAGAACAAGAAGCCGTTTATTAAATCGCGCTACTGTCGTGGTGTGCCTGACCCAAAGATTCGTATCTATGATGTTGGTAACAAGAAAGCTTCAGTGGATACTTTTCCATTCGTAGCTCACTTGGTATCGGACGAGAAGGAACAGCTCTCCTCGGAAGCTCTTGAAGCTGCACGTATTGCTGCGAATCGTTACTTGACTAAATATTGTGGCAAGGACAATTTCCATATGCGCATTCGATGCCATCCATTCCAGGTCCTACGTATCAACAAGATGCTTTCATGTGCTGGTGCTGATAGACTTCAGACGGGTATGCGTCACGCATATGGTAAACCCGCTGGTGTAGCCGCCCGTGTGGCTATTGGTCAGCCGATTATTTCGGTACGTTCAAAGGATTCGTTTGGTCCTTCGGTTGTTGAAGCTCTTCGTCGTGCACGCTCCCGAGTCAGAGAAGATCTCGGCTGAGAAGCTTCTGGAGGTGTACGAGGGCTTTATCGCGAAGTGCGCCGGGTCGAGCAGGATCGTGTCGATTGAGGACCCGTTCGACCAGGACGACTGGGAGTCGTGGATGAAGATTACGGAAAAGGTTGGCAAGGACGTGCAGATCGTGGGAGACGACCTGACGGTGACGAACCCGACCCGTGTGAAGAAGGCCATTGAGCAGAAGGCTTGCAACGCTCTTCTGCTAAAAGTGAACCAGATCGGCAGCATCACGGAGTCGATTGAGGCCGTGACGATGGCCAAGAAGGCCGGTTGGGCTATCATGGCTAGCCACCGCAGTGGCGAGACGGAGGACACGTTCATCGCTGATCTGGCTGTTGGTCTGAGTGCCGGCCAGATGGTAACAATTGGGCCAAGGGGCACTACACTGAGGGCGCCGAGCTCATTGACTCCGTTCTGGATGTCGTCCGTAAGGAGGCGGAAAGCTGTGACTGTTTGCAGGGTTTCCAGATCACGCACTCGCTTGGTGGCGGTACCGGCTCCGGTATGGGAACGCTTCTTATCTCAAAGATCCGAGAAGAGTACCCGGACCGTATCATGTGCACGTACTCGGTATGTCCATCTCCCAAGGTGTCGGACACTGTTGTAGAGCCCTACAATGCTACGCTGTCTGTACATCAGCTTGTCGAAAACGCCGATGAGGTCATGTGCCTGGATAATGAGGCCTTGTACGACATTTGCTTCCGTACGCTGAAGCTCACCACCCCCACCTATGGTGACCTGAATCACTTGGTGTGTGCCGCTATGTCGGGCATCACCACTTCCCTACGATTTCCGGGACAGCTGAACTCGGACCTGCGGAAGCTGGCCGTGAACCTGATCCCGTTCCCTCGTCTTCACTTCTTCATGATTGGTTTCGCGCCGCTGACGTCACGTGGTTCGCAGCAGTACCGTGCCTTGACGGTGCCTGAGCTGACACAGCAGCAGTTTGATGCTAAAAACATGATGTGCGCCGCCGACCCTCGCCACGGCCGCTATTTAACTGCCGCTTGTATGTTCCGCGGACGCATGAGCACGAAGGAGGTTGATGAGCAAATGCTGAACGTGCAGAACAAAAACTCGTCGTATTTTGTCGAGTGGATTCCTAACAACATCAAGGCTAGTGTGTGTGACATCCCACCCAAGGGCCTGAAGATGAGCACCACCTTTATTGGTAATTCGACAGCCATCCAAGAGATGTTCAAGCGTGTGTCTGAACAGTTCACGGCTATGTTCCGTCGTAAGGCTTTCTTGCATTGG

Peronospora_destructor TTTTTTAATTATAGTTACTGTATTTGTTTGTTGGATGTTATTTAGAGTAATTACTCTTTTTAATG--AAAAAAAATAAAATTTCAGCAACAGTAGTGCATGGAGCTACTATTGAAATTATTTGGACCACTATTCCCGCTTTAATTTTATTAATAGTTGCCGTACCTTCTTTTGCTTTATTATATTCAATGGATGAAGTAATTGATCCAATTATTACATTAAAAGTAATTGGGAATCAATGGTATTGGAGTTATGAATATTCAGATAATTTAGAATTTTCAGATGAACCTTTAATTTTTGATAGTTATATGGTACAAGAAGATGATTTAACTATAGGTCAATTACGACTTTTAGAAGTAGATAATCGTGTAGTAGTTCCTACTAATAGTCATATTAGAGTATTAATTACTGCTTCGGATGTTTTACATTCATGGGCTATTCCTTCATTAGGTATTAAATTAGATGCTTGTCCAGGACGTTTAAATCAAACCGGCGAGTGAAGCGGGATGAGCTCAAGCTTAAAATCTCCGTACAAGTTTTGTAGGGCGAATTGTAGTCTATAGAGGC-GTGGTCAGCGTGGGCGCTTAGGGCAAGTTCCTTGGAAGAGGACAGCATGGAGGGTGATACTCCCGTTCATC-CCTGAGTGGCTCGTGCGTACGACCCGTTTTCTTTGAGTCGCGTTGTTTGAGAATGCAGCGCAAAGTAGGTGGTAAATTCCATCTAAAGCTAAATATTGGTGCGAGACCGATAGCATACAAGTACCGTGAGGGAAAGATGAAAAGAACTTTGAAAAGAGAGTTAAAGAGTACCTGAAACTGCTGAAAGGGAACCGGATCGTTTCCAGTGTCTATAATCCGTGGCATATTTCATTGGCGAGTGTGTGCGTGCGTGTGCTTTGGCAGCGGCCTTTT---GGCTGCGCTTGGCGCG---TG-TGCTGTGTGTGCTTGCTGGTGCCCTGTGCTGCGGTGGGACGTCAATGTCAGTTCGTATGCTGCGGGAAATGGCTACCGAGGAGATAGGGCA-------CTTTTGAGTCTGTTATATCTCGGTGGACG-AGTCGTCGCGGTTGGGACTGAGGTG-CCTACAACGTGCTTTTGAGTGGGTTTGTGTCTCCGTGTGTGCCGTGTGCGGATAGCTTGCTATGCGTGTGT---GGTTATGCGTGGATTGATGCGGGCTTTAACTTGTTGCCGTTCATTCTGGTATTGGTATGACTAAGGCTGATCTTATTAACAACTTGGGGACGATCGCCAAATCGGGTACCAAGGCCTTCATGGAGGCCCTGGCTGCCGGTGCTGACATCAGTATGATTGGTCAGTTTGGTGTGGGGTTCTATTCTGCCTATCTCGTGGCTGACCAGGTCGTTGTCCACTCGAAGCACAATGATGACGAGCAGTACGTCTGGGAATCTGCTGCCGGTGGTTCATTCACCGTGAAGCCTGAC---ACTTCGGAGCCTATTCTACGTGGTACACGCATTGTATTGAAGCTGAAGGAGGACATGCTTGAGTATTTGGAAGAGCGCAAGTTGAAAGACTTGGTGAAGAAACACTCGGAGTTCATTGGCTTCCCTATCAAGCTTTACGTTGAGAAGACGGAGGAGAAGGAAGTGACGGATGATGAAGAGGAGGAGGAAG---AGAAGGAGGGTGAAGAGGACAAGCCCAAGGTGGAGGAAGTTGACGAGGAGGAAGAAGGTGAGAAGAAGAAGAAGACGAAGAAGATCAAGGAAGTGACCCACGAGTGGGACCACTTGAACAGTCAGAAACCTATTTGGATGCGTAAGCCTGAGGACGTGACCCACGAGGAATACGCTTCGTTCTACAAGTCGCTGACCAACGACTGGGAGGAACACGCTGCTGTGAAGCACTTTTCGGTGGAGGGTCAGCTCGAGTTTAAGGCTTGTTTATTCACTCCTAAGCGCGCGCCGTTCGACATGTTTGAGGGTGGTGCCAAGAAGAAAGTCAACAACATCAAATTGTACGTGCGTCGCGTGTTCATCATGGACAACTGCGAAGATCTCATGCCTGAGTACCTGTCGTTTGTCAAGGGTGTCGTCGACTCGGAGGACTTACCGCTGAACATTTCGCGCGAGACTTTGCAGCAGAACAAGATTTTGCGTGTGATCAAGAAGAACTTGGTCAAGAAGTGCCTGGAAATGTTTGCGGAAATTGCTGAGGACAATGAGAAGTACAACAAGTTCTATGAGGCTTTCAGCAAGAACTTGAAAGCTGGGCATCCACGAAGACTCGACGAACCGCACGAAGATTGCCAAGCTGCTACGTTACCACTCGACCAAGTCTGGCGAGGAGACGACGTCATTGGACGACTACATCTCGCGCATGCCGGACAACCAGCCTGGTATTTATTACGTGACGGGAGAGAGCAAGAAGTCGGTAGAGAACTCACCGTTCATTGAGAAGCTCAAGAAGAAGGGGTATGAGGTGCTATTTATGGTGGAGGCCATTGACGAGTACGCCGTGCAACAGTTGAAGGAGTACGAGGGTAAGAAGCTTATCTGCGCCACGAAGGAGGGCCTCAAGATGGAGGAGACGGAGGACGAGAAGAAGGCCTTTGAGGAGGCGAAGGCTGCTACTGAGGGCCTTCACGGTCATTGACGCTCCTGGCCACCGTGATTTTATCAAGAACATGATTACGGGCACTTCGCAGGCTGATTGCGCCATTCTCGTTGTGGCTTCAGGTGTGGGTGAGTTTGAGGCTGGTATCTC---CAAGGAAGGCCAGACACGTGAGCACGCTCTGCTGGCCTTTACGCTTGGTGTGAAGCAGATGGTTGTGGCTATTAACAAGATGGACGACTCGTCTGTCATGTACGGTCAGGCTCGTTACGAAGAGATCAAGGCCGAGGTCACTACTTACCTGAAGAAGGTTGGCTACAAACCTGCCAAGATTCCTTTCGTGCCTATCTCGGGATGGGAGGGAGACAACATGATTGATCGCTCGTCGAACATGCCGTGGTACAAGGGACCTTTTCTCCTTGAGGCTCTTGACAGTCTGAACCCACCAAAGCGCCCGTCGGACAAGCCTCTTCGTTTGCCTCTCCAGGACGTGTACAAGATTGGCGGAATTGGCACGGTACCGGTCGGCCGTGTGGAGACTGGTGTCATTAAACCCGGCATGGTCGCTACTTTTGGTCCCGTCGGTCTGTCGACGGAGGTCAAGTCCGTTGAGATGCACCACGAGTCTTTGCCGGAGGCTCTTCCTGGTGACAACGTTGGCTTCAACGTCAAGAACGTGTCGGTAAAGGAACTGCGTCGTGGTTTTGTCGCTTCGGACTCGAAGAACGA---CCCTGCCATGGGCACCCAGAATTTCACTGCCCAGGTGATTGTGTTGAACCACCCTGGCCAGATCGGCAATGGCTACTCGCCGGTGCTTGACTGCCACACGGCCCACGTTGCCTGCAAGTTCAAAGAGATCATTGAGAAGATGGACCGTCGTTCGGGCAAGGTACTTGTTCAGAACAAGAAGCCGTTCATTAAGTCGCGCTACTGCCGTGGTGTACCTGATTCAAAGATCCGTATCTATGATGTTGGTAACAAAAAGGCTTCAGTGGACGCCTTTCCATTCGTAGCTCACTTGGTGTCTGACGAGAAGGAACAGCTCTCATCGGAAGCTCTTGAAGCTGCACGTATTGCTGCTAACCGTTATCTGACCAAGTATTGCGGCAAGGACAATTTCCACATGCGCATTCGATGCCATCCATTCCAGGTTCTACGTATCAACAAGATGCTTTCGTGTGCTGGTGCTGATAGACTTCAGACGGGTATGCGTCACGCTTACGGGAAACCCGCGGGTGTGGCCGCCCGTGTGGCTATTGGTCAGCCGATTATCTCGGTACGTTCGAAGGACTCGTTCGGCCCTTCAGTCGTTGAAGCTCTTCGTCGTGCATGCTCCCGAGTCCGAAAAGATCTCGGCTGATAAGCTTCTGGAAGTGTACGAGGGCTTTATCGCGAAGTGCGCCGGGTCAAGCAGGATCGTGTCGATTGAGGACCCGTTCGACCAGGACGACTGGGAATCGTGGATGAAGATTACGAAAAAGGTCGGCAAGGACGTGCAGATCGTGGGAGACGATCTGACGGTGACGAACCCGACCCGTGTGAAGAAGGCCATTGAGCAAAAGGCTTGCAACGCTCTCCTGCTAAAGGTGAACCAGATCGGCAGCATCACGGAGTCGATTGAGGCCGTGACGATGGCCAAGAAGGCCGGTTGGGCTATCATGGCCAGCCACCGCAGTGGCGAGACGGAGGACACGTTCATTGCTGATCTGGCTGTTGGTCTGAGTGCCGGCCAGATGGTAACAACTGGGCCAAGGGACACTACACTGAGGGCGCCGAGCTTATTGACTCAGTTCTGGACGTTGTCCGTAAGGAGGCAGAAAGCTGTGACTGTTTGCAGGGTTTCCAGATCACGCACTCGCTTGGTGGCGGTACCGGCTCTGGTATGGGAACGCTTCTTATCTCAAAGATCCGAGAAGAGTACCCGGATCGTATCATGTGCACGTACTCGGTGTGTCCATCTCCCAAGGTGTCGGACACAGTTGTAGAGCCCTACAATGCTACGCTGTCTGTACATCAGCTTGTCGAGAACGCCGATGAGGTCATGTGCCTGGATAATGAGGCCTTATACGATATTTGCTTCCGTACGCTAAAGCTCACCACCCCCACCTATGGCGATCTGAACCACTTGGTGTGTGCCGCTATGTCGGGTATCACTACTTCCCTACGATTCCCGGGACAGCTGAACTCGGATCTGCGGAAGCTGGCCGTGAACCTGATCCCGTTCCCCCGTCTCCACTTCTTCATGATTGGTTTTGCCCCGCTGACGTCACGTGGCTCGCAGCAGTACCGTGCCCTGACGGTACCCGAGCTGACACAGCAGCAGTTTGATGCCAAAAACATGATGTGCGCCGCCGACCCTCGCCACGGCCGGTATTTAACTGCCGCCTGTATGTTTCGTGGACGCATGAGCACAAAGGAGGTTGATGAGCAAATGCTGAACGTGCAGAATAAGAACTCATCGTACTTTGTCGAGTGGATCCCTAACAACATCAAGGCTAGTGTGTGTGACATCCCACCCAAGGGCCTGAAGATGAGCACCACCTTTATTGGTAATTCGACAGCCATCCAAGAGATGTTCAAGCGTGTGTCTGAACAGTTCACGGCTATGTTTCGTCGTAAGGCTTTCTTGCATTGG

Phytophthora_boehmeriae TTACTTAATTCTTATAACTGTTTTTGTTTGTTGGTTGTTATTTAGAGTTATTACTCTTTTTAATGAAAAAAAAAATAAAATTCCATCAACTGTTGTACATGGTGCAACTATTGAAATTATTTGGACTTCAATTCCTGCTTTAATATTATTAACTGTTGCAATTCCATCTTTTGCTTTATTATATTCTATGGATGAAGTAATTGATCCAATTATTACTTTAAAAGTAATTGGAAGTCAATGGTATTGGAGTTATGAATATTCTGATAATTTAGAATTTTCAGATGAACCTTTAATTTTTGATAGTTATATGGTTCAAGAAGATGATTTAGCTATAGGTCAATTTCGATTATTAGAAGTAGATAATCGTGTTGTAGTACCTACAAATAGTCATATTCGTGTATTAATTACAGCTTCTGATGTTTTACATTCTTGGGCTATTCCTTCATTAGGTATAAAATTAGATGCATGTCCAGGTCGTTTAAATCAAACCGGCGAGTGAAGCGGGAAGAGCTCAAGCTTAAAATCTCTGCGCAAGTTTTGCGTGGCGAATTGTAGTCTATAGAAGC-GTGGTCAGCGCGAGCGCTTGGGGTAAGTTCCTTGGAAGAGGACAGCATGGAGGGTGATACTCCCGTTCATA-CCTGAGTGGCTTGTGCGTACGACCCGTTTTCTTTGAGTCGCGTTGTTTGGGAATGCAGCGCAAAGTAGGTGGTAAATTCCATCTAAAGCTAAATATTGGTGCGAGACCGATAGCGAACAAGTACCGTGAGGGAAAGATGAAAAGAACTTTGAAAAGAGAGTTAAAGAGTACCTGAAACTGCTGAAAGGGAACCGAATCGTTTCCAGTGTCTATAATCTGCGGCATATTTCATTGGCGAGTGTGTGCGTGCGTGTGCTTTTGCAGTGGCTTTTTT--GGCTGCGCTGGGTGCG---TG-CTGTGCGTGTGTTTGCTGGTGCCCTGTGCTGCGGTGGGACGTCAAGGTCAGTTCGTATGCTGCGGGAAATGGCTGCCGGGGAGGTAGGGCTTACGCT-TGCGTTTGTCTGTTATATCTTGGTGGACG-AGTCGTCGCGGTTGGGACTGAGGTG-CCTACAACGTGCTTTTGAGTGTGTGGAACTCTCTGTGTGCGCGCTGTGCGGATAGCTTGCTATGTGTGCGG---TGCTGTGCGTGGATTGAGGTCTGCTGTAACTTGTTGCCGTTCACTCTGGTATCGGTATGACGAAGGCCGACCTCATTAACAACCTGGGAACGATCGCCAAGTCGGGCACCAAGGCCTTCATGGAGGCTCTGGCTGCCGGCGCTGACATCAGCATGATCGGTCAGTTCGGTGTGGGTTTCTACTCGGCCTACCTGGTGGCCGACAAGGTGGTGGTCCACTCGAAGCACAACGACGACGAGCAGTACGTGTGGGAGTCGGCTGCCGGCGGTTCGTTCACCGTCACGCCTGAC---ACGTCGGAGCCCATCCAGCGTGGTACCCGCATCGTGCTGAAGCTCAAGGAGGACATGCTCGAGTACCTGGAGGAGCGCAAGCTGAAAGACCTGGTGAAGAAGCACTCTGAGTTCATTGGCTTCCCCATCAAGCTGTACGTCGAGAAGACGGAGGAGAAGGAAGTGACGGACGACGAGGAGGACGAGGATG---ACAAGGAGGAGGACGACGACAAGCCCAAGGTGGAGGAAGTCGAC---GAGGAGGAAGGCGAGAAGAAGAAGAAGACCAAGAAGATCAAGGAGGTGACCCACGAGTGGGACCACCTCAACAGCCAGAAGCCCATCTGGATGCGCAAGCCGGAGGACGTGACCCACGAGGAGTACGCCTCGTTCTACAAGTCGCTGACCAACGACTGGGAGGAGCACGCTGCCGTCAAGCACTTCTCTGTCGAGGGCCAGCTGGAGTTCAAGGCCTGCCTGTTCACGCCCAAGCGCGCCCCGTTCGACATGTTCGAGGGTGGTGCCAAGAAGAAACTGAACAACATTAAGCTGTACGTGCGTCGCGTGTTCATCATGGACAACTGCGAGGAGCTCATGCCCGAGTACCTGTCGTTCGTCAAGGGTGTTGTCGACTCGGAGGACCTGCCGCTGAACATTTCGCGTGAGACCCTGCAGCAGAACAAGATCCTGCGTGTGATCAAGAAGAACCTGGTGAAGAAGTGCCTGGAGATGTTCGCCGAGCTCGCCGAGGACTCGGAGAAGTACAACAAGTTCTACGAGGCCTTCAGCAAGAACCTG-AAGTTGGGCATCCACGAGGACTCGACCAACCGCACGAAGATCGCCAAGCTGCTGCGTTACCACTCGACCAAGTCGGGCGAGGAGCTGACGTCGCTCGACGACTACATTTCGCGCATGCCGGAGAACCAGCCTGGCATCTACTACGTGACGGGCGAGAGCAAGAAGTCGGTGGAGAACTCGCCGTTCATTGAGAAGCTGAAGAAGAAGGGCTACGAGGTGCTGTTCATGGTCGAGGCCATTGACGAGTACGCTGTGCAGCAGCTTAAGGACTACGAGGGCAAGAAGCTGATCTGCGCCACCAAGGAGGGTCTGGCCATGGAGGAGACCGAGGACGAGAAGAAGTCGTTCGAGGAGGCCAAGGCCGCCACTGAGGGCCTTCACGGTCATTGACGCCCCTGGCCACCGTGACTTCATCAAGAACATGATTACGGGCACCTCGCAGGCCGACTGCGCCATCCTGGTGGTGGCTTCGGGTGTGGGTGAGTTCGAGGCTGGTATCTC---GAAGGAGGGCCAGACCCGTGAGCACGCTCTGCTCGCCTTCACCCTGGGTGTGAAGCAGATGATCGTGGCCATCAACAAGATGGACGACTCCTCGGTCATGTACGGCCAGGCCCGTTACGAGGAGATCAAGAACGAGGTGACGACGTACCTGAAGAAGGTCGGCTACAAGCCCGCCAAGATCCCGTTCGTGCCCATCTCCGGCTGGGAGGGAGACAACATGATTGAGAAGTCCCCCAACATGCCGTGGTACAAGGGACCGTACCTCCTCGAGGCTCTGGACAACCTGAACCCCCCCAAGCGTCCGTCCGACAAGCCCCTCCGTCTGCCCCTCCAGGATGTGTACAAGATCGGCGGTATTGGCACGGTACCGGTCGGCCGTGTCGAGACTGGTGTGATCAAGCCGGGCATGATCGCCACCTTCGGCCCCGTGGGTCTGACCACGGAAGTCAAGTCCGTCGAGATGCACCACGAGTCTCTGCCGGAGGCTGTCCCTGGTGACAACGTTGGCTTCAACGTCAAGAACGTGTCGGTGAAGGAGCTGCGTCGTGGTTTCGTGGCTTCGGACTCGAAGAACGA---CCCGGCCAAGGGCACGCAGGACTTCACTGCTCAGGTCATTGTGCTGAACCACCCTGGCCAGATCGGCAACGGCTACTCGCCGGTGCTTGACTGCCACACGGCCCACGTTGCCTGCAAGTTCAAAGAAATCACGGAGAAGATGGACCGTCGTTCGGGCAAGGTGCTCGTCCAGAACAAGAAGCCGTTCATTAAGTCGCGCTTCTGCCGTGGTGTGCCCGACCCGAAGATCCGCATCTACGATGTCGGCAACAAGAAGGCTTCGGTCGACACCTTCCCCTTCGTTGCCCACTTGGTCTCCGACGAGAAGGAGCAGCTGTCCTCGGAGGCTCTGGAAGCCGCCCGTGTTGCTGCTAACCGTTACCTGACCAAGTACTGCGGCAAGGACAACTTCCACATGCGCATCCGCTGCCACCCCTTCCAGGTGCTGCGTATCAACAAGATGCTTTCGTGCGCTGGTGCCGATAGACTGCAGACCGGTATGCGTCACGCCTACGGTAAGCCCGCCGGTGTGGCCGCCCGCGTGGCCATCGGCCAGCCCATTATCTCGGTGCGCTCCAAGGACAACTTCGGCCCCTCGGTCGTGGAGGCTCTGCGTCGTGCACGCGCCGGAGTCGGAGAAGATCTCTGCCGCCAAGCTGACGGAGGTGTACGAGGGCTTCATCGCCAAGTGCAAGAACACGAGCGCCATCGTGTCCATCGAGGACCCGTTCGACCAGGACGACTGGGAGTCGTGGATCAACTTCACGTCGAAGGTGGGCAAGGACGTGCAGATCGTCGGTGACGACCTGACGGTGACGAACCCCACGCGTGTGCAGAAGGCCATCGACACGAAGGCTTGCAACGCTCTCCTGCTGAAGGTGAACCAGATCGGTAGCATCACGGAGTCGATCGAGGCCGTGACGATGGCCAAGAAGGCCGGCTGGGGTATCATGACCAGCCACCGCAGCGGTGAGACGGAGGACACGTTCATCGCCGACCTGGCTGTGGGTCTGAGCGCTGGCCAGATGGTAACAACTGGGCCAAGGGACACTACACGGAAGGCGCAGAGCTCATTGACTCGGTGCTGGATGTCGTCCGGAAGGAGGCCGAGTCCTGCGATTGCCTGCAGGGTTTCCAGATTACCCACTCGCTGGGTGGCGGTACCGGTTCCGGTATGGGAACGCTGCTGATCTCCAAGATCCGTGAAGAGTACCCGGACCGTATCATGTGCACGTACTCGGTGTGCCCGTCCCCCAAGGTGTCGGACACTGTGGTGGAGCCTTACAACGCGACGCTATCTGTGCACCAGCTTGTGGAGAACGCCGATGAAGTTATGTGCCTGGATAACGAGGCCCTGTACGACATTTGCTTCCGTACGCTCAAGCTCACCACCCCCACCTACGGTGATCTGAACCACCTGGTGTGTGCCGCCATGTCCGGTATCACGACGTGCCTTCGATTCCCAGGCCAGCTGAACTCCGACCTGCGTAAGCTGGCTGTGAACTTGATCCCATTCCCGCGTCTTCACTTCTTTATGATCGGTTTCGCGCCGCTGACATCACGTGGCTCGCAGCAGTACCGCGCCCTGACGGTGCCGGAGCTGACCCAGCAGCAGTTCGACGCGAAGAACATGATGTGCGCCGCTGACCCTCGCCACGGCCGTTATTTAACTTGCTCGTGCATGTTCCGTGGACGGATGAGCACGAAGGAGGTGGACGAGCAGATGCTGAACGTGCAGAACAAGAACTCTTCATACTTTGTGGAATGGATCCCGAACAACATCAAGGCTAGTGTGTGTGATATCCCGCCTAAGGGCCTGAAGATGAGCACCACGTTCATCGGTAACTCTACCGCTATCCAGGAGATGTTCAAGCGCGTGTCTGAGCAGTTCACGGCTATGTTCCGTCGTAAGGCTTTCTTGCACTGG

Phytophthora_kernoviae TTACTTAATTCTTATAACTGTTTTTGTTTGTTGGTTGTTATTTAGAGTTGTTACTCTTTTTAATGAAAAAAATAATAAAATCCCCTCAACTGTAGTACATGGTGCAACTATTGAGATTATTTGGACTTCAATTCCAGCTTTAATATTATTAACTGTAGCAATCCCATCATTTGCTTTATTATATTCAATGGATGAGGTAATTGATCCTATTATTACTTTAAAAGTAATTGGAAGTCAATGGTATTGGAGTTATGAATATTCAGATAATTTAGAATTTTCAGATGAACCTTTAATTTTTGATAGTTATATGGTTCAAGAAGATGATTTAGCTATAGGTCAATTCAGATTGTTAGAAGTAGATAATCGTGTAGTAGTTCCTACAAATAGTCATATTCGCGTATTAATTACAGCGTCAGATGTTTTACATTCTTGGGCAATTCCTTCATTAGGTATAAAATTAGATGCATGTCCTGGACGTTTAAATCAAACCGGCGAGTGAAGCGGGAAGAGCTCAAGCTTAAAATCTCTGCGCAAGTTTTGCGTGGCGAATTGTAGTCTATAGAAGC-GTGGTCAGCGCGAGCGCTTGGGGTAAGTTCCTTGGAAGAGGACAGCATGGAGGGTGATACTCCCGTTCATA-CCTGAGTGGCTTGTGCGTACGACCCGTTTTCTTTGAGTCGCGTTGTTTGGGAATGCAGCGCAAAGTAGGTGGTAAATTCCATCTAAAGCTAAATATTGGTGCGAGACCGATAGCGAACAAGTACCGTGAGGGAAAGATGAAAAGAACTTTGAAAAGAGAGTTAAAGAGTACCTGAAACTGCTGAAAGGGAACCGAATCGTTTCCAGTGTCTATAATCTGTGGCATATTTCATTGGCGAGTGTATGCGTGCGTGTGCTTTGGCAGTGGCTTTTTT--GGCTGCGCTGGGTGCG---TG-TTGTGCGTGTGTTTGCTGGTGCCCTGTGCTATGGTGGGACGTCAAGGTCAGTTCGTGTGCTGCGGGAAATGGCTGCTGAGGAGGTAGGGCTTACGCTTTGCGTTTGTCTATTATATCTTGGTGGACG-AGTCGTCGCGGTTGGGACTGAGGTG-CCTACAACGTGCTTTTGAGTCTGTGGAACTCTCTGTGTGCGTGCTGTGTGGATAGCTTGCTATGTGCGTGG---TGCTGTGCGTGGATTGAGGTCTGCTGTAACTTGTTGCCGTTCACTCTGGTATTGGTATGACGAAGGCGGACCTCATTAACAACCTAGGAACGATCGCCAAGTCGGGCACCAAGGCCTTCATGGAGGCTCTGGCTGCCGGCGCTGACATCAGTATGATTGGTCAGTTCGGTGTGGGTTTCTACTCTGCCTACCTGGTTGCGGACAAGGTGGTGGTCCACTCGAAGCACAACGACGATGAGCAGTACGTGTGGGAGTCCGCTGCTGGCGGCTCGTTCACGGTCACGCCCGAC---ACGTCGGAGCCCATCCTGCGTGGTACGCGCATTGTGATGAAACTCAAGGAGGACATGCTGGAGTACCTGGAGGAGCGCAAGCTGAAAGACCTGGTGAAGAAGCACTCTGAGTTCATTGGCTTCCCCATCAAGCTCTACGTGGAGAAGACGGAGGAGAAGGAAGTGACGGATGATGAAGAGGAGGAGGAAGACAAGGAGGAGGAGGAAGACGACAAGCCTAAGGTGGAGGAAGTCGAA---GAGGAGGAAGGCGAGAAGAAGAAGAAGACCAAGAAGATCAAGGAGGTGACCCACGAGTGGGACCACTTGAACAGCCAGAAACCTATCTGGATGCGCAAGCCTGAGGACGTGACCCACGAGGAGTACGCGTCGTTCTACAAGTCGTTGACCAACGACTGGGAGGAGCACGCCGGCGTTAAGCACTTCTCGGTGGAGGGCCAGCTGGAGTTCAAGGCGTGTCTGTTCACGCCCAAGCGTGCCCCGTTCGATATGTTCGAGGGCGGCGCCAAGAAGAAAATCAACAACATTAAGCTGTATGTGCGTCGCGTGTTCATCATGGACAACTGCGAGGATCTCATGCCTGAGTACCTGTCGTTCGTCAAGGGTGTTGTCGACTCGGAGGACCTGCCGTTGAACATCTCGCGTGAGACTCTGCAGCAGAACAAGATCCTGCGTGTGATCAAGAAGAACCTGGTGAAGAAGTGCCTGGAGATGTTCGCCGAGCTCGCCGAGGACGCGGAGAAGTACAACAAGTTCTACGAGTCGTTCAGCAAGAACCTG-AAGCTGGGCATCCACGAGGACTCGACCAACCGCACCAAGATCGCCAAGCTGCTGCGTTACCACTCAACCAAGTCGGGCGAGGAGGTGACGTCGCTGGACGACTACATCTCGCGCATGCCGGAGAGCCAGCCGGGCATTTACTACGTGACGGGCGAGAGCAAGAAGTCGGTGGAGAACTCCCCGTTCATTGAGAAGCTTAAGAAGAAGGGCTATGAGGTTCTGTTTATGGTTGAGGCCATTGACGAGTACGCCGTGCAGCAGCTGAAGGACTATGAGGGCAAGAAGCTGATCTGCGCCACCAAGGAGGGTCTGGCCATGGAGGAGACCGAGGACGAGAAGAAGTCGTTCGAGGAGGCCAAGGCCGCCACCGAGGGCCTTCACCGTCATTGACGCCCCTGGCCACCGTGACTTCATCAAGAACATGATCACGGGCACGTCGCAGGCTGACTGCGCCATCCTGGTGGTCGCTTCGGGTGTGGGCGAGTTCGAGGCTGGTATCTC---CAAGGAGGGCCAGACTCGTGAGCACGCTCTGCTTGCTTTCACTCTTGGTGTGAAGCAGATGATCGTTGCCATCAACAAGATGGACGACTCTTCGGTGATGTACGGCCAGGCCCGTTACGAGGAGATCAAGGCTGAGGTCACCACGTACCTGAAGAAGGTTGGTTACAAACCCGCTAAGATCCCGTTCGTGCCCATCTCTGGATGGGAGGGAGACAACATGATCGAGAAGTCGGGCAACATGCCGTGGTACAAGGGCCCGTACCTCCTCGAGGCTCTGGACACCCTGAACGCCCCCAAGCGTCCGTCCGACAAGCCTCTCCGTCTTCCCCTCCAGGACGTTTACAAGATCGGCGGTATTGGCACGGTACCGGTCGGCCGTGTCGAGACTGGTGTGATCAAGCCGGGCATGATCGCTACCTTCGGCCCCGTGGGTCTGTCCACGGAAGTCAAGTCCGTCGAGATGCACCACGAGTCTCTGCCTGAGGCTCTCCCTGGTGACAACGTTGGCTTCAACGTCAAGAACGTGTCGGTGAAGGAGCTGCGTCGTGGTTTCGTTGCTTCGGACTCGAAGAACGA---CCCCGCCAAGGGCACGCAGGACTTCACCGCTCAGGTGATTGTGCTGAACCACCCTGGCCAGATCGGCAACGGTTACTCTCCGGTGCTTGACTGCCACACGGCCCACGTTGCGTGCAAGTTCAAAGAGATCACGGAGAAGATGGACCGTCGYTCGGGCAAGGTGCTCGTCCAGAACAAGAAGCCCTTCATTAAGTCGCGCTTCTGCCGTGGTGTGCCCGACTCGAAGATCCGCATCTACGATGTCGGTAACAAGAAGGCTTCGGTCGACACGTTCCCGTTCGTTTGTCACTTGGTGTCTGACGAGAAGGAGCAGCTCTCCTCGGAAGCTCTTGAAGCCGCCCGTGTTGCTGCTAACCGTTACCTGACCAAGTACTGCGGCAAGGACAACTTCCACATGCGTATCCGTTGCCACCCGTTCCAGGTGCTGCGTATCAACAAAATGCTTTCGTGCGCTGGTGCCGATAGACTGCAGACGGGTATGCGTCACGCTTACGGTAAGCCCGCCGGTGTGGCTGCCCGTGTGGCCATTGGCCAGCCCATTATCTCGGTGCGTTCCAAGGACAACTTCGGCCCTTCGGTCGTGGAGGCTCTGCGTCGTGCACGCCCCAGAGTCGGAGAAGATCTCCGCTGACAAGCTGACGGAGGTGTACGAAGGCTTCATCGCCAAGTGCAAGAACTCCAGCGACATCGTGTCGATCGAGGACCCGTTCGACCAGGACGACTGGGAGTCGTGGATCAACTTCACGTCGAAGGTGGGCAATGATGTGCAGATCGTTGGTGACGACCTGACGGTGACGAACCCCACGCGTGTGCAGAAGGCCATCGACACGAAGGCTTGCAACGCTYTTCTGCTGAAGGTGAACCAGATCGGCAGCATCACGGAGTCGATTGAGGCTGTGACGATGGCCAAGAAGGCCGGTTGGGCCATCATGACTAGTCACCGTAGTGGTGAGACAGAGGACACGTTCATCGCCGATCTGGCTGTGGGTCTGAGTGCTGGTCAGATGGTAACAACTGGGCCAAGGGACACTACACGGAAGGCGCGGAGCTCATTGACTCGGTGCTGGACGTCGTCCGTAAGGAGGCCGAGTCCTGCGATTGCCTGCAGGGTTTCCAGATTACCCACTCCCTTGGTGGTGGTACCGGTTCCGGTATGGGAACGCTTCTAATCTCCAAGATTCGTGAAGAGTACCCAGACCGTATCATGTGCACGTACTCGGTGTGCCCGTCCCCCAAGGTGTCGGACACTGTGGTGGAGCCTTACAACGCGACACTTTCCGTGCACCAGCTTGTCGAGAACGCCGACGAGGTCATGTGTCTGGATAACGAGGCCCTGTACGACATTTGCTTCCGTACACTCAAGCTCACGACCCCCACCTATGGTGACCTGAACCACCTGGTCTGTGCCGCCATGTCTGGTATCACAACGTGCCTGCGTTTCCCCGGTCAGCTCAACTCTGACCTGCGTAAGCTGGCTGTCAACTTGATCCCGTTCCCGCGTCTCCACTTCTTCATGATCGGTTTCGCGCCGCTGACGTCGCGTGGATCGCAGCAGTATCGCGCCCTGACGGTGCCGGAGCTGACCCAGCAGCAGTTCGACGCTAAGAACATGATGTGCGCCGCTGACCCTCGTCATGGCCGTTATTTAACTTGCTCGTGCATGTTCCGCGGACGGATGAGCACGAAGGAGGTGGACGAGCAGATGTTGAACGTGCAGAACAAGAACTCTTCATACTTCGTGGAGTGGATCCCGAACAACATCAAGGCTAGTGTGTGTGATATCCCGCCTAAGGGCCTGAAGATGAGCACCACGTTCATCGGTAACTCTACCGCTATCCAGGAGATGTTCAAGCGTGTGTCCGAGCAGTTCACGGCTATGTTCCGTCGTAAGGCTTTCTTGCACTGG

Phytophthora_cactorum TTTTTTAATTATGATTACTGTATTTGTTTGTTGGATGTTATTTAGAGTTATAACTCTTTTTGATGAAAAAAATAATAAAATTCCTTCAACTGTTGTACATGGTGCTACTATTGAAATTATTTGGACTTCTATTCCAGCTTTAATTTTATTAACTGTTGCAGTTCCATCTTTTGCTTTATTATATTCAATGGATGAAGTAATTGATCCAATTATTACTTTAAAAGTAATTGGTAGTCAATGGTACTGGAGTTATGAATATTCAGATAATTTAGAATTCTCAGATGAACCTTTAATTTTTGATAGTTATATGGTACAAGAAGATGATTTAGCTATAGGTCAATTTAGACTTTTAGAAGTAGATAATCGTGTAGTAGTTCCAACTAATAGTCATATTAGGGTATTAATTACAGCATCAGATGTATTACATTCATGGGCAATCCCATCGTTAGGTATTAAATTAGATGCTTGTCCTGGCCGTTTAAATCAAACCGGCGAGTGAAGCGGGAAGAGCTCAAGCTTAAAATCTCCGTGCAAGTTTTGTGCGGCGAATTGTAGTCTATAGAGGC-GTGGTCAGCGTGGGCGCTTGGGGCAAGTTCCTTGGAAGAGGACAGCATGGAGGGTGATACTCCCGTTCATC-CCTGAGTTGCTCGTGCGTACGGCCCGTTTTCTTTGAGTCGCGTTGTTTGGGAATGCAGCGCAAAGTAGGTGGTAAATTCCATCTAAAGCTAAATATTGGTGCGAGACCGATAGCGAACAAGTACCGTGAGGGAAAGATGAAAAGAACTTTGAAAAGAGAGTTAAAGAGTACCTGAAACTGCTGAAAGGGAACCGAATCGTTTCCAGTGTCTATAATCCGTGGTATATTTCATTGGCGAGTGTGTGCGTGCGTGTGCTGTGGCAGCGGCTTTTTA--GGCTGCGCTCGGCGTG---TG-TGCTGTGTGTGCTTGCTGGTGCCCTGTACTGCGGTGGGACGTCAAGGTCAGTTCGTATGCTGCGGGAAATGGCTGCCGAGGAGGTAGGGCTTACGCT-TGCGTTTGTCTGTTATATCTTGGTGGACG-AGTAGTCGCGGTTGGGACTGAGGTG-CCTACAACGTGCTTTTGAGTGGGTTTGTGTCTCCGTGTGCGCCGTGTGCGGATAGCTTGCTATGCGTGTGT---GGTTGTGTGTGGATTGATGCGGGCTTTAACTTGTTGCCGTTCACTCTGGTATTGGTATGACGAAGGCTGACCTCATCAACAACCTGGGTACGATCGCCAAGTCGGGCACCAAGGCCTTCATGGAGGCTCTGGCCGCCGGCGCTGACATCAGCATGATCGGTCAGTTCGGTGTGGGTTTCTACTCGGCCTACCTGGTCGCCGACAAGGTCGTGGTGCACTCGAAGCACAACGACGACGAGCAGTATGTGTGGGAGTCCGCCGCCGGTGGCTCGTTCACTGTGACGCCCGAC---ACGTCGGAGCCCATCCAGCGTGGTACGCGCATTGTGCTGAAGCTCAAGGAGGACATGCTTGAGTACCTCGAGGAGCGCAAGCTTAAGGACCTGGTGAAGAAGCACTCGGAGTTCATTGGCTTCCCCATCAAGCTTTACGTCGAGAAGACGGAGGAGAAGGAGGTGACTGATGACGAGGAAGAGGAGGACG---AGAAGGAGGGCGAGGACGACAAGCCCAAGGTTGAGGAGGTCGAA---GAGGAGGAGGGCGAGAAGAAGAAGAAGACCAAGAAGATCAAGGAAGTGACCCACGAGTGGGACCACCTCAACAGTCAGAAGCCCATCTGGATGCGCAAGCCCGAGGACGTGACCCACGAGGAGTACGCTTCGTTCTACAAGTCGCTGACCAACGACTGGGAGGAGCACGCTGGTGTCAAGCACTTCTCGGTGGAGGGACAGCTGGAGTTCAAGGCGTGTCTGTTCACCCCCAAGCGCGCCCCGTTCGACATGTTCGAGGGCGGTGCCAAGAAGAAACTGAACAACATCAAGCTGTACGTGCGTCGCGTGTTCATCATGGACAACTGCGAGGAGCTCATGCCCGAGTACCTGTCGTTCGTCAAGGGTGTTGTCGACTCGGAGGACCTGCCGCTCAACATCTCGCGTGAGACTCTGCAGCAGAACAAGATCCTGCGTGTGATCAAGAAGAACCTGGTCAAGAAGTGCCTGGAGATGTTCGCTGAGCTGGCCGAGGACAACGAGAAGTACAACAAGTTCTACGAGTCGTTCAGCAAGAACCTG-AAGCTCGGCATCCACGAGGACTCGACCAACCGCACCAAGATCGCCAAGCTTCTGCGTTACCACTCGACCAAGTCTGGCGAGGAAGTGACGTCCCTGGATGACTACATCTCGCGTATGCCCGAGAACCAGCCTGGCATCTACTACGTGACGGGCGAGAGCAAGAAGTCGGTGGAGAACTCGCCGTTCATTGAGAAGCTCAAGAAGAAGGGCTACGAGGTGCTGTTCATGGTTGAGGCCATTGACGAGTACGCTGTGCAGCAGCTGAAGGAGTACGAGGGCAAGAAGCTCATCTGCGCCACCAAGGAGGGCCTCAAGATGGAGGAGACTGAGGACGAGAAGAAGTCGTTCGAGGAGGCCAAGGCTGCCACCGAGGGTCTTCACGGTYATTGACGCCCCTGGTCACCGTGACTTCATCAAGAACATGATTACGGGTACCTCGCAGGCCGACTGCGCCATCCTGGTGGTTGCTTCGGGYGTGGGTGAGTTYGAGGCTGGTATCTC---CAAGGAGGGCCAGACYCGTGAGCACGCTCTGCTTGCCTTCACTCTGGGTGTGAAGCAGATGATYGTYGCCATCAACAAGATGGACGACTCGTCTGTCATGTACGGCCAGGCCCGTTACGAGGAGATCAAGTCTGAGGTCACYACGTACCTGAAGAAGGTTGGCTACAAGCCCGCCAAGATCCCGTTCGTGCCCATCTCCGGCTGGGAGGGTGACAACATGATCGAYCGCTCCACMAACATGCCGTGGTACAAGGGACCTTTCCTCCTTGAGGCTCTTGACAACCTGAACGCCCCCAAGCGCCCGAGCGACAAGCCTCTGCGTCTGCCCCTCCAGGATGTGTACAAGATCGGCGGTATCGGCACGGTACCGGTCGGCCGTGTGGAGACTGGTGTCATCAAGCCTGGCATGGTCGCCACYTTCGGYCCYGTTGGTCTGTCGACGGAAGTYAAGTCCGTTGAGATGCACCACGAGTCTCTGCCTGAGGCTGTCCCTGGTGACAACGTYGGCTTCAACGTCAAGAACGTGTCGGTCAAGGAGCTGCGTCGTGGTTTCGTCGCYTCGGACTCCAAGAACGA---CCCCGCTAAGGGCACCCAGGACTTCACCGCCCAGGTGATTGTGCTGAACCACCCTGGCCAGATCGGCAACGGCTACTCGCCGGTGCTTGACTGCCACACGGCCCACGTTGCCTGCAAGTTCAAAGAGATCACGGAGAAGATGGACCGTCGTTCGGGYAAGGTGCTYGTCCAGAACAAGAAGCCGTTCATTAAGTCGCGCTTCTGCCGTGGTGTGCCCGACCCGAAGATCCGCATCTACGATGTCGGTAACAAGAAGGCTTCGGTCGACGCCTTCCCGTTCGTGGCCCACCTGGTGTCCGACGAGAAGGAGCAGCTGTCCTCGGAGGCTCTTGAGGCTGCCCGTATTGCTGCTAACCGTTACCTGACCAAGTACTGCGGCAAGGACAACTTCCACATGCGCATCCGTTGCCACCCGTTCCAGGTTCTACGTATCAACAAGATGCTTTCGTGCGCTGGTGCCGATAGACTGCAGACCGGTATGCGTCACGCTTACGGTAAGCCCTCGGGTGTTGCCGCCCGTGTGGCCATTGGCCAGCCCATTATCTCGGTGCGTTCCAAGGACTCGTTTGGCCCTTCGGTCGTGGAGGCTCTGCGTCGTGCACGCTGCGGACTCGGAGAAGATCTCTGCCGACAAGCTGCAGGAGGTGTACGAGGGCTTCATTGCCAAGTGCGCTGGCTCGAGCAAGATCGTGTCCATTGAGGACCCGTTCGACCAGGATGACTGGGAGTCGTGGGTGAAGATCACGGGCAAGGTGGGCAAGGACGTGCAGATCGTGGGCGACGACCTTACGGTGACGAACCCGACCCGTGTGAAGAAGGCCATCGAGCAGAAGGCTTGCAACGCTCTTCTGCTGAAGGTGAACCAGATTGGCAGCATCACGGAGTCGATTGAGGCCGTGACGATGGCGAAGAAGGCTGGCTGGGGAATCATGGCCAGCCACCGTAGCGGAGAGACGGAGGACACGTTCATCGCCGACCTGGCTGTGGGTCTGAGCGCGGGTCAGATGGTAACAACTGGGCCAAGGGACACTACACTGAGGGTGCCGAGCTGATTGACTCAGTGCTCGACGTTGTCCGCAAGGAGGCCGAGAGCTGTGATTGCCTTCAGGGTTTCCAGATCACGCACTCGCTTGGTGGCGGTACCGGTTCCGGTATGGGTACGCTTCTTATCTCGAAGATTCGTGAGGAGTACCCCGACCGTATCATGTGCACATACTCGGTGTGCCCGTCGCCCAAGGTGTCGGACACGGTCGTGGAGCCCTACAACGCTACGCTGTCGGTGCACCAGCTTGTCGAGAACGCCGATGAAGTCATGTGCCTGGATAACGAGGCCCTGTACGACATTTGCTTCCGTACGCTGAAGCTCACTACTCCCACCTACGGGGACTTGAACCACCTGGTGTGCGCCGCCATGTCCGGTATCACCACGTGCCTGCGTTTCCCCGGTCAGCTGAACTCGGACCTGCGTAAGCTGGCCGTGAACCTGATCCCGTTCCCTCGTCTCCACTTCTTTATGATTGGTTTCGCCCCGCTGACGTCGCGTGGCTCGCAGCAGTATCGCGCCCTGACGGTGCCAGAGCTGACCCAGCAGCAGTTCGATGCCAAGAACATGATGTGCGCCGCCGACCCTCGCCACGGCCGCTATTTAACTGCCGCGTGTATGTTCCGCGGACGTATGAGCACGAAGGAGGTTGACGAGCAGATGTTGAACGTGCAGAACAAGAACTCGTCGTACTTCGTCGAGTGGATCCCCAACAACATCAAGGCTAGCGTGTGTGACATCCCTCCTAAGGGTCTGAAGATGAGCACTACGTTCATCGGTAACTCTACTGCCATCCAGGAGATGTTCAAGCGTGTGTCCGAACAGTTTACGGCTATGTTCCGTCGTAAGGCTTTCTTGCACTGG

Phytophthora_idaei TTTTTTAATCATGATTACTGTATTTGTTTGTTGGATGTTATTTAGAGTTATAACTCTTTTTGATGAAAAAAAAAATAAAAATCCTTCAAATATAGTACATGGTGCTATTATTGAAATTATTTGGACTTCTATTCCAGCTTTAATTTTATTAATGGTTGCAATTCCATCTTTTGCTTTATTATATTCAATGGATGAAGTAATTGATCCAATTATTACTTTAAAAGTAATTGGTAGTCAATGGTATTGGAGTTATGAATATTCAGATAATTTAGAATTCTCAGATGAACCTTTAATTTTTGATAGTTATATGGTACAAGAAGATGATTTAGCTATAGGTCAATTTAGACTTTTAGAAGTAGATAATCGTGTAGTAGTTCCAACTAATAGTCATATTAGGGTATTAATTACAGCATCAGATGTATTACATTCATGGGCAATCCCATCGTTAGGTATTAAATTAGATGCTTGTCCAGGTCGTTTAAATCAAACCGGCGAGTGAAGCGGGAAGAGCTCAAGCTTAAAATCTCCGTGCAAGTTTTGTGCGGCGAATTGTAGTCTATAGAGGC-GTGGTCAGCGTGGGCGCTTGGGGCAAGTTCCTTGGAAGAGGACAGCATGGAGGGTGATACTCCCGTTCATC-CCTGAGTTGCTCGTGCGTACGGCCCGTTTTCTTTGAGTCGCGTTGTTTGGGAATGCAGCGCAAAGTAGGTGGTAAATTCCATCTAAAGCTAAATATTGGTGCGAGACCGATAGCGAACAAGTACCGTGAGGGAAAGATGAAAAGAACTTTGAAAAGAGAGTTAAAGAGTACCTGAAACTGCTGAAAGGGAACCGAATCGTTTCCAGTGTCTATAATCCGTGGTATATTTCATTGGCGAGTGTGTGCGTGCGTGTGCTGTGGCAGCGGCTTTTTT-AGGCTGCGCTCGGCGTG---TG-TGCTGTGTGTGCTTGCTGGTGCCCTGTACTGCGGTGGGACGTCAAGGTCAGTTCGTATGCTGCGGGAAATGGCTGCCGAGGAGGTAGGGCTTACGCT-TGCGTTTGTCTGTTATATCTTGGTGGACG-AGTAGTCGCGGTTGGGACTGAGGTG-CCTACAACGTGCTTTTGAGTGAGTTTGTGTCTCCGTGTGCGCCGTGTGCGGATAGCTTGCTATGCGTGTGT---GGTTGTGTGTGGATTGATGCGGGCTTTAACTTGTTGCCGTTCACTCTGGTATTGGTATGACGAAGGCTGACCTCATCAACAACCTGGGTACGATCGCCAAGTCGGGCACCAAGGCCTTCATGGAGGCTCTGGCCGCCGGCGCTGACATCAGCATGATCGGTCAGTTCGGTGTGGGTTTCTACTCGGCCTACCTGGTCGCCGACAAGGTCGTGGTGCACTCGAAGCACAACGACGACGAGCAGTACGTGTGGGAGTCCGCCGCGGGTGGATCGTTCACTGTGACGCCCGAC---ACGTCGGAGCCCATCCAGCGTGGTACGCGCATTGTGCTGAAGCTCAAGGAGGACATGCTTGAGTACCTCGAGGAGCGCAAGCTTAAGGACCTGGTGAAGAAGCACTCGGAGTTCATTGGCTTCCCCATCAAGCTTTACGTCGAGAAGACGGAGGAGAAGGAGGTGACTGACGACGAGGAAGAGGAGGACG---AGAAGGAGGGCGAGGACGACAAGCCCAAGGTTGAGGAGGTCGAA---GAGGAGGAGGGCGAGAAGAAGAAGAAGACCAAGAAGATCAAGGAAGTGACCCACGAGTGGGACCACCTCAACAGCCAGAAGCCCATCTGGATGCGCAAGCCCGAGGACGTGACCCACGAGGAGTACGCTTCGTTCTACAAGTCGCTGACCAACGACTGGGAGGAGCACGCCGGCGTCAAGCACTTCTCGGTGGAGGGCCAGCTGGAGTTCAAGGCGTGTCTGTTCACTCCCAAGCGCGCCCCGTTCGACATGTTCGAGGGCGGTGCCAAGAAGAAACTGAACAACATCAAGCTGTACGTGCGTCGCGTGTTCATCATGGACAACTGCGAGGAGCTCATGCCCGAGTACCTGTCGTTCGTCAAGGGTGTTGTCGACTCGGAGGACCTGCCGCTCAACATCTCGCGTGAGACTCTGCAGCAGAACAAGATCCTGCGTGTAATCAAAAAGAACCTGGTCAAGAAGTGCCTGGAGATGTTCGCTGAGCTGGCTGAGGACAACGAGAAGTACAACAAGTTCTACGAGTCGTTCAGCAAGAACCTG-AAGCTGGGCATCCACGAGGACTCGACCAACCGCACCAAGATCGCCAAGCTTCTGCGTTACCACTCGACTAAGTCCGGCGAGGAAGTGACATCCCTGGATGACTACATCTCGCGTATGCCCGAGAATCAGCCTGGCATCTACTACGTGACGGGCGAGAGCAAGAAGTCGGTGGAGAACTCGCCGTTCATTGAGAAGCTCAAGAAGAAGGGCTACGAGGTGCTGTTCATGGTTGAGGCCATTGACGAGTACGCTGTGCAGCAGCTGAAGGAGTACGAGGGCAAGAAGCTCATCTGCGCCACCAAGGAGGGCCTCAAGATGGAGGAGACTGAGGACGAGAAGAAGTCGTTCGAGGAGGCCAAGGCTGCCACCGAGGGTCTTCACGGTCATTGACGCCCCTGGTCACCGTGACTTCATCAAGAACATGATTACGGGYACYTCGCAAGCCGACTGCGCCATCCTGGTGGTTGCTTCGGGTGTGGGTGAGTTCGAGGCTGGTATCTC---CAAGGAGGGMCAGACKCGTGAGCACGCTCTGCTTGCCTTCACTCTGGGTGTGAAGCAGATGATCGTCGCCATCAACAAGATGGACGACTCGTCTGTCATGTACGGCCAGGCCCGTTACGAGGAGATCAAGTCTGAGGTCACCACGTACCTGAAGAAGGTTGGCTACAAGCCCGCCAAGATCCCGTTCGTGCCCATCTCCGGCTGGGAGGGTGACAACATGATCGACCGCTCCACCAACATGCCGTGGTACAAGGGACCTTTCCTCCTTGAGGCTCTTGACAACCTGAACGCCCCCAAGCGCCCGAGCGACAAGCCTCTGCGTCTGCCCCTCCAGGATGTGTACAAGATCGGCGGTATCGGCACGGTACCGGTCGGCCGTGTGGAGACTGGTGTCATCAAGCCTGGCATGGTCGCCACTTTCGGCCCCGTTGGTCTGTCGACGGAAGTCAAGTCCGTTGAGATGCACCACGAGTCTCTGCCTGAGGCTGTCCCTGGTGACAACGTCGGCTTCAACGTCAAGAACGTGTCGGTCAAGGAGCTGCGTCGTGGTTTCGTCGCTTCGGACTCCAAGAACGA---CCCCGCTAAGGGCACCCAGGACTTCACCGCCCAGGTGATTGTGCTGAACCACCCYGGCCAGATCGGCAACGGYTACTCGCCGGTGCTTGACTGCCACACGGCCCACGTTGCCTGCAAGTTCAAAGAGATCACGGAGAAGATGGACCGTCGTTCGGGCAAGGTGCTTGTCCAGAACAAGAAGCCGTTCATTAAGTCGCGCTTCTGCCGTGGTGTGCCCGACCCGAAGATCCGCATCTACGATGTCGGTAACAAGAAGGCTTCGGTCGACGCCTTCCCGTTCGTGGCCCACCTGGTGTCCGACGAGAAGGAGCAGCTGTCCTCGGAGGCTCTTGAGGCTGCCCGTATTGCTGCTAACCGTTACCTGACCAAGTACTGCGGCAAGGACAACTTCCACATGCGCATCCGTTGCCACCCGTTTCAGGTTCTGCGTATCAACAAGATGCTTTCGTGCGCTGGTGCCGATAGACTGCAGACCGGTATGCGTCACGCTTACGGTAAGCCCTCGGGTGTTGCCGCCCGTGTGGCCATTGGCCAGCCCATTATCTCGGTGCGTTCCAAGGACTCGTTCGGCCCTTCGGTCGTGGAGGCTCTGCGTCGTGCACGCTGCGGACTCGGAGAAGATCTCTGCCGACAAGCTGCAGGAGGTGTACGAGGGCTTCATTGCCAAGTGCGCTGACTCGAGCAAGATCGTGTCCATTGAGGACCCGTTCGACCAGGATGACTGGGAGTCGTGGGTGAAGATCACGGGCAAGGTGGGCAAGGACGTGCAGATCGTGGGCGACGACCTTACGGTGACGAACCCGACCCGTGTGAAGAAGGCCATCGAGCAGAAGGCTTGCAACGCTCTTCTGCTGAAGGTGAACCAGATTGGCAGCATCACGGAGTCGATTGAGGCCGTGACGATGGCGAAGAAAGCTGGCTGGGGAATCATGGCCAGCCACCGTAGTGGAGAGACGGAGGACACGTTCATCGCCGACCTGGCTGTGGGTCTGAGCGCGGGCCAGATGGTAACAACTGGGCCAAGGGACACTACACTGAGGGTGCCGAGCTGATTGACTCGGTGCTCGACGTTGTCCGCAAGGAGGCCGAGAGCTGTGATTGCCTTCAGGGTTTCCAGATCACGCACTCGCTTGGTGGCGGTACCGGTTCCGGTATGGGTACGCTTCTTATCTCGAAGATTCGTGAGGAGTACCCCGACCGTATCATGTGCACATACTCGGTGTGCCCGTCGCCCAAGGTGTCGGACACGGTCGTGGAGCCCTACAACGCTACGCTGTCGGTGCACCAGCTTGTCGAGAACGCCGATGAAGTCATGTGCCTGGATAACGAGGCCCTGTACGACATTTGCTTCCGTACGCTGAAGCTCACTACTCCCACCTACGGTGACTTGAACCACCTGGTGTGCGCTGCCATGTCCGGTATCACCACGTGCCTGCGTTTCCCCGGTCAGCTGAACTCGGACCTGCGTAAGCTGGCCGTGAACCTGATCCCGTTCCCTCGTCTCCACTTCTTTATGATTGGTTTCGCCCCGCTGACGTCGCGTGGCTCGCAGCAGTATCGCGCCCTGACGGTGCCCGAGCTGACCCAGCAGCAGTTCGATGCCAAGAACATGATGTGCGCCGCCGACCCTCGCCACGGCCGCTATTTAACTGCCGCGTGTATGTTCCGCGGACGTATGAGCACGAAGGAGGTTGACGAGCAGATGTTGAACGTGCAGAACAAGAACTCGTCGTACTTCGTCGAGTGGATCCCCAACAACATCAAGGCTAGCGTGTGTGACATCCCTCCTAAGGGTCTGAAGATGAGCACTACGTTCATCGGTAACTCTACTGCCATCCAGGAGATGTTCAAGCGTGTGTCCGAACAGTTTACGGCTATGTTCCGTCGTAAGGCTTTCTTGCACTGG

Phytophthora_nicotianae TTTTTTAATTATGATTACAGTATTTGTTTGTTGGATGTTATTTAGAGTTATTACTCTTTTTGATGAAAAAAAAAATAAAATCCCATCAACTGTTGTACATGGTGCTACTATTGAAATTATTTGGACATCTATTCCAGCTTTAATTTTATTAACAGTTGCAGTTCCATCTTTTGCATTATTATATTCAATGGATGAAGTAATTGATCCTATTATTACTTTAAAAGTAATAGGTAGTCAATGGTATTGGAGTTACGAATATTCAGATAATTTAGAATTTTCAGATGAACCTTTAATTTTTGATAGTTATATGGTACAAGAAGATGATTTAGCAATAGGTCAATTTAGACTTTTAGAAGTAGATAATCGTGTAGTAGTTCCAACTAATAGTCATATTAGAGTATTAATTACTGCATCAGATGTATTACATTCATGGGCTATTCCTTCATTAGGCATTAAATTAGATGCTTGTCCAGGTCGTTTAAATCAAACCGGCGAGTGAAGCGGGAAGAGCTCAAGCTTAAAATCTCCGTGCAAGTTTTGTGCGGCGAATTGTAGTCTATAGAAGC-GTGGTCAGCATGGGCACTTGGGGCAAGTTCCTTGGAAGAGGACAGCATGGAGGGTGATACTCCCGTTCATT-TCTGAGTTGCTCGTGCGTACGACCCGTTTTCTTTGAGTCGCGTTGTTTGGGAATGCAGCGCAAAGTAGGTGGTAAATTCCATCTAAAGCTAAATATTGGTGCGAGACCGATAGCGAACAAGTACCGTGAGGGAAAGATGAAAAGAACTTTGAAAAGAGAGTTAAAGAGTACCTGAAACTGCTGAAAGGGAACCGAATCGTTTCCAGTGTCTATAATCCGTGGTATATTTCATTGGCGAGTGTGTGCGTGCGTGTGCTGTGGCAGCGGCTTTTTT--GGCTGCGCTCGGCGTG---TG-TGCTGTGTGTGCTTGCTGGTGCTCTGTGCTGCGGTGGGACGTCAAGGTCAGTTCGTATGCTGCGGGAAATGGCTGCCGAGGAGGTAGGGCTTACGCT-TGCGTTTGTCTGTTATATCTTGGTGGACG-AGTAGTCGCGGTTGGGACTGAGGTG-CCTACAACGTGCTTTTGAGTGGGTTTGTGTCTCCGTGTRCGCCGTGTGCGGATAGCTTGCTATGCGTGTGT---GGTTGTGTGTGGATTGATGCGAGCTTTAACTTGTTGCCGTTCACTCTGGTATTGGTATGACGAAGGCTGACCTCATTAACAACCTGGGTACGATCGCCAAGTCGGGCACAAAGGCCTTCATGGAGGCTCTGGCTGCCGGCGCCGACATCAGCATGATTGGTCAGTTCGGTGTGGGTTTCTACTCTGCCTACCTGGTCGCCGACAAGGTCGTGGTGCACTCGAAGCACAACGATGACGAGCAGTACGTGTGGGAGTCCGCCGCCGGTGGCTCGTTCACCGTCACGCCCGAC---ACGTCGGAGCCCATTGCTCGTGGTACGCGCATTGTGCTGAAGCTCAAGGAGGACATGCTCGAGTACCTTGAGGAGCGCAAGCTGAAAGACCTGGTGAAGAAGCACTCGGAGTTCATTGGCTTCCCCATCAAGCTGTACGTCGAGAAGACGGAGGAGAAGGAAGTGACGGACGACGAGGAAGAGGAGGACG---AGAAGGAGGGCGAGGACGACAAGCCCAAGGTTGAGGAGGTCGAA---GAGGAGGAGGGAGAGAAGAAGAAGAAGACCAAGAAGATCAAGGAAGTGACCCACGAGTGGGACCACCTCAACAGCCAGAAGCCCATCTGGATGCGCAAGCCCGAGGACGTGACCCACGAGGAGTACGCTTCGTTCTACAAGTCGCTGACTAAYGACTGGGAGGAGCACGCCGCCGTCAAGCACTTCTCGGTGGAGGGCCAGCTGGAATTCAAGGCCTGTCTGTTCACCCCCAAGCGCGCGCCGTTCGACATGTTCGAGGGCGGTGCCAAGAAGAAACTGAACAACATCAAGCTGTACGTGCGTCGCGTCTTCATCATGGACAACTGCGAGGAGCTCATGCCCGAGTACCTGTCGTTCGTCAAGGGTGTTGTGGACTCGGAGGACCTGCCGCTTAACATCTCGCGTGAGACGCTGCAGCAGAACAAGATCCTGCGTGTGATCAAGAAGAACCTGGTCAAGAAGTGCCTCGAGATGTTCGCGGAGCTCGCCGAGGACAACGAGAAGTACAACAAGTTCTACGAGGCCTTCAGCAAGAACCTC-AAGCTCGGCATCCACGAGGACTCGACGAACCGCACCAAGATCGCCAAGCTGCTGCGTTACCACTCGACTAAGTCTGGCGAGGAGATGACTTCGCTGGACGACTACATCTCGCGTATGCCGGAGAACCAGCCCGGTATCTACTACGTGACCGGTGAGAGCAAGAAGTCGGTGGAGAACTCGCCGTTCATTGAGAAGCTCAAGAAGAAGGGCTACGAGGTGCTGTTCATGGTGGAGGCCATTGACGAGTACGCTGTGCAGCAGCTGAAGGAGTACGAGGGCAAGAAGCTCATCTGCGCCACCAAGGAGGGCCTCAAGATGGAGGAGACTGAGGATGAGAAGAAGTCGTTCGAGGAGGCTAAGGCTGCTACGGAGGGCCTTCACGGTCATTGACGCCCCTGGTCACCGTGACTTCATCAAGAACATGATTACGGGTACCTCGCAGGCCGACTGCGCCATCCTTGTGGTGGCCTCGGGTGTGGGTGAGTTCGAGGCTGGTATCTC---CAAGGAGGGCCAGACCCGTGAGCACGCTCTGCTTGCCTTCACTCTGGGTGTGAAGCAGATGATCGTCGCCATCAACAAGATGGACGACTCGTCTGTCATGTACGGYCAGGCCCGTTACGAGGAGATCAAGAACGAGGTCACCACGTACCTGAAGAAGGTCGGCTACAAGCCCGCYAAGATCCCGTTCGTGCCCATCTCTGGCTGGGAGGGTGACAACATGATCGACCGCTCCACCAACATGCCGTGGTACAAGGGCCCCTACCTCCTTGAGGCTCTTGACAACCTGAACCCCCCTAAGCGCCCGGTTGACAAGCCGCTGCGTCTTCCYCTTCAGGATGTGTACAAGATCGGCGGTATTGGCACGGTACCTGTCGGCCGTGTGGAGACCGGTGTCATCAAGCCTGGCATGGTCGCCACTTTCGGCCCCGTTGGTCTGTCGACTGAAGTCAAGTCCGTCGAGATGCACCACGAGTCTCTGCCTGAGGCCGTCCCTGGTGACAACGTCGGCTTCAACGTCAAGAACGTGTCGGTGAAGGAGCTGCGTCGTGGTTACGTCGCCTCGGACTCCAAGAACGA---CCCGGCCAAGGGCACCCAGGACTTCACCGCCCAGGTGATTGTGCTGAACCACCCCGGCCAGATCGGCAACGGCTACTCGCCCGTGCTTGACTGCCACACGGCCCACGTTGCCTGCAAGTTCAAAGAGATCACGGAGAAGATGGACCGTCGTTCGGGCAAGGTGCTCGTCCAGAACAAGAAGCCGTTCATTAAGTCGCGCTTCTGCCGTGGTGTGCCCGACCCGAAGATCCGCATCTACGATGTCGGTAACAAGAAGGCTTCGGTCGACGCCTTCCCGTTCGTTGCTCACCTGGTGTCCGACGAGAAGGAGCAGCTGTCCTCAGAGGCTCTTGAGGCTGCCCGTGTTGCTGCTAACCGTTACCTGACCAAGTACTGCGGTAAGGACAACTTCCACATGCGCATCCGTTGCCACCCGTTCCAGGTCCTGCGTATCAACAAGATGCTTTCGTGCGCCGGTGCCGATAGACTGCAGACCGGTATGCGTCACGCTTACGGTAAGCCCTCGGGTGTTGCTGCCCGTGTGGCCATCGGCCAGCCCATTATCTCGGTGCGTTCCAAGGACTCGTTCGGCCCCTCGGTCGTGGAGGCTCTGCGTCGTGCACGCTGCGGAGTCGGAGAAGATTTCGGCTGACAAGCTGCAGGAGGTGTACGAGGGCTTCATCGCCAAGTGCGCTAACTCGAGCAAGATCGTGTCCATTGAGGATCCGTTCGACCAGGACGACTGGGCTTCGTGGGTGAAGTTCACGGGCAAGGTTGGCAAGGACGTTCAGATCGTGGGCGATGACCTTACGGTGACGAACCCGACCCGTGTGAAGAAGGCTATTGAGCAGAAGGCTTGCAACGCTCTTCTGCTGAAGGTGAACCAGATTGGCAGCATCACGGAGTCGATTGAGGCCGTGACGATGGCTAAGAAGGCCGGCTGGGGCATTATGGCCAGCCACCGCAGCGGTGAGACCGAGGACACGTTCATCGCCGACCTGGCTGTGGGTCTGAGCGCCGGCCAGATGGTAACAACTGGGCCAAGGGACACTACACTGAGGGTGCCGAGCTTATTGACTCGGTGCTCGACGTCGTCCGTAAGGAGGCCGAGAGCTGTGATTGCCTTCAGGGTTTCCAGATCACGCACTCGCTTGGTGGTGGTACCGGTTCCGGTATGGGTACGCTTCTTATCTCGAAGATTCGTGAGGAGTATCCCGACCGTATCATGTGCACATACTCGGTGTGCCCGTCGCCCAAGGTGTCGGACACGGTTGTGGAGCCCTACAACGCTACGCTGTCGGTGCACCAGCTTGTTGAGAACGCCGATGAGGTCATGTGTCTGGATAACGAGGCCCTGTACGACATTTGCTTCCGTACGCTGAAGCTTACCACTCCCACCTACGGTGACCTGAACCACCTCGTGTGTGCCGCCATGTCTGGTATCACCACGTGCCTGCGATTCCCCGGTCAGCTGAACTCGGACCTGCGTAAGCTGGCCGTGAACCTGATCCCGTTCCCGCGTCTTCACTTCTTTATGATTGGTTTCGCCCCGCTGACGTCGCGTGGCTCGCAGCAGTACCGTGCCCTGACGGTGCCCGAGCTGACCCAGCAGCAGTTCGACGCCAAGAACATGATGTGCGCCGCTGACCCTCGCCACGGCCGCTATTTAACTGCTGCTTGTATGTTCCGTGGACGTATGAGCACAAAGGAGGTTGATGAGCAGATGCTGAACGTGCAGAACAAGAACTCGTCGTACTTCGTCGAGTGGATCCCCAACAACATCAAGGCTAGCGTGTGTGATATCCCGCCCAAGGGTCTGAAGATGAGCACTACGTTCATCGGTAACTCTACTGCCATCCAGGAGATGTTCAAGCGTGTGTCCGAACAGTTTACGGCTATGTTCCGTCGTAAGGCTTTCTTGCACTGG

Phytophthora_parasitica TTTTTTAATTATGATTACAGTATTTGTTTGTTGGATGTTATTTAGAGTTATTACTCTTTTTGATGAAAAAAAAAATAAAATCCCATCAACTGTTGTACATGGTGCTACTATTGAAATTATTTGGACATCTATTCCAGCTTTAATTTTATTAACAGTTGCAGTTCCATCTTTTGCATTATTATATTCAATGGATGAAGTAATTGATCCTATTATTACTTTAAAAGTAATAGGTAGTCAATGGTATTGGAGTTACGAATATTCAGATAATTTAGAATTTTCAGATGAACCTTTAATTTTTGATAGTTATATGGTACAAGAAGATGATTTAGCAATAGGTCAATTTAGACTTTTAGAAGTAGATAATCGTGTAGTAGTTCCAACTAATAGTCATATTAGAGTATTAATTACTGCATCAGATGTATTACATTCATGGGCTATTCCTTCATTAGGCATTAAATTAGATGCTTGTCCAGGTCGTTTAAATCAAACCGGCGAGTGAAGCGGGAAGAGCTCAAGCTTAAAATCTCCGTGCAAGTTTTGTGCGGCGAATTGTAGTCTATAGAAGC-GTGGTCAGCATGGGCACTTGGGGCAAGTTCCTTGGAAGAGGACAGCATGGAGGGTGATACTCCCGTTCATT-TCTGAGTTGCTCGTGCGTACGACCCGTTTTCTTTGAGTCGCGTTGTTTGGGAATGCAGCGCAAAGTAGGTGGTAAATTCCATCTAAAGCTAAATATTGGTGCGAGACCGATAGCGAACAAGTACCGTGAGGGAAAGATGAAAAGAACTTTGAAAAGAGAGTTAAAGAGTACCTGAAACTGCTGAAAGGGAACCGAATCGTTTCCAGTGTCTATAATCCGTGGTATATTTCATTGGCGAGTGTGTGCGTGCGTGTGCTGTGGCAGCGGCTTTTTT--GGCTGCGCTCGGCGTG---TG-TGCTGTGTGTGCTTGCTGGTGCTCTGTGCTGCGGTGGGACGTCAAGGTCAGTTCGTATGCTGCGGGAAATGGCTGCCGAGGAGGTAGGGCTTACGCT-TGCGTTTGTCTGTTATATCTTGGTGGACG-AGTAGTCGCGGTTGGGACTGAGGTG-CCTACAACGTGCTTTTGAGTGGGTTTGTGTCTCCGTGTGCGCCGTGTGCGGATAGCTTGCTATGCGTGTGT---GGTTGTGTGTGGATTGATGCGAGCTTTAACTTGTTGCCGTTCACTCTGGTATCGGTATGACGAAGGCTGACCTCATTAACAACCTGGGTACGATCGCCAAGTCGGGCACCAAAGCCTTCATGGAGGCTCTGGCTGCCGGCGCCGACATCAGCATGATTGGTCAGTTCGGTGTGGGTTTCTACTCTGCCTACCTGGTCGCTGACAAGGTCGTGGTGCACTCGAAGCACAACGATGACGAGCAGTACGTGTGGGAGTCCGCCGCCGGTGGCTCGTTCACCGTCACGCCCGAC---ACGTCGGAGCCCATTGCTCGTGGTACGCGCATTGTGCTGAAGCTCAAGGAGGACATGCTCGAGTACCTTGAGGAGCGCAAGCTGAAAGACCTGGTGAAGAAGCACTCGGAGTTCATTGGCTTCCCCATCAAGCTGTACGTCGAGAAGACGGAGGAGAAGGAAGTGACGGACGACGAGGAAGAGGAGGACG---AGAAGGAGGGCGAGGACGACAAGCCCAAGGTTGAGGAGGTTGAA---GAGGAGGAGGGAGAGAAGAAGAAGAAGACCAAGAAGATCAAGGAAGTGACCCACGAGTGGGACCACCTCAACAGCCAGAAGCCCATCTGGATGCGCAAGCCCGAGGACGTGACCCACGAGGAGTACGCTTCGTTCTACAAGTCGCTGACTAACGACTGGGAGGAGCACGCCGCCGTCAAGCACTTCTCGGTGGAGGGCCAGCTGGAATTCAAGGCCTGTCTGTTCACCCCTAAGCGCGCGCCGTTCGACATGTTCGAGGGCGGTGCCAAGAAGAAACTGAACAACATCAAGCTGTACGTGCGTCGCGTCTTCATCATGGACAACTGCGAGGAGCTCATGCCCGAGTACCTGTCGTTCGTCAAGGGTGTTGTGGACTCGGAGGACCTGCCGCTTAACATCTCGCGTGAGACGCTGCAGCAGAACAAGATCCTGCGTGTGATCAAGAAGAACCTGGTCAAGAAGTGCCTCGAGATGTTCGCGGAGCTCGCCGAGGACAACGAGAAGTACAACAAGTTCTACGAGGCCTTCAGCAAGAACCTC-AAGCTCGGCATCCACGAGGACTCGACGAACCGCACCAAGATCGCCAAGCTGCTGCGTTACCACTCGACTAAGTCTGGCGAGGAGATGACGTCGCTGGACGACTACATCTCGCGTATGCCGGAGAACCAGCCCGGTATCTACTACGTGACCGGTGAGAGCAAGAAGTCGGTGGAGAACTCGCCGTTCATTGAGAAGCTCAAGAAGAAGGGCTACGAGGTGCTGTTCATGGTGGAGGCCATTGACGAGTACGCTGTGCAGCAGCTGAAGGAGTACGAGGGCAAGAAGCTCATCTGCGCCACCAAGGAGGGCCTCAAGATGGAGGAGACTGAGGACGAGAAGAAGTCGTTCGAGGAGGCTAAGGCTGCTACGGAGGGCCTTCACGGTCATTGACGCCCCTGGTCACCGTGACTTCATCAAGAACATGATTACGGGTACCTCGCAGGCCGACTGCGCCATCCTTGTGGTGGCCTCGGGTGTGGGTGAGTTCGAGGCTGGTATCTC---CAAGGAGGGCCAGACCCGTGAGCACGCTCTGCTTGCCTTCACTCTGGGTGTGAAGCAGATGATCGTCGCCATCAACAAGATGGACGACTCGTCTGTCATGTACGGCCAGGCCCGTTACGAGGAGATCAAGAACGAGGTCACCACGTACCTGAAGAAGGTCGGCTACAAGCCCGCCAAGATCCCGTTCGTGCCCATCTCTGGCTGGGAGGGTGACAACATGATCGACCGCTCCACCAACATGCCGTGGTACAAGGGCCCCTACCTCCTTGAGGCTCTTGACAACCTGAACCCCCCTAAGCGCCCGGTTGACAAGCCGCTGCGTCTTCCTCTTCAGGATGTGTACAAGATCGGCGGTATTGGCACGGTACCTGTCGGCCGTGTGGAGACCGGTGTCATCAAGCCTGGCATGGTCGCCACTTTCGGCCCCGTTGGTCTGTCGACTGAAGTCAAGTCCGTCGAGATGCACCACGAGTCTCTGCCTGAGGCCGTCCCTGGTGACAACGTCGGCTTCAACGTCAAGAACGTGTCGGTGAAGGAGCTGCGTCGTGGTTACGTCGCCTCGGACTCCAAGAACGA---CCCGGCCAAGGGCACCCAGGACTTCACCGCCCAGGTGATTGTGCTGAACCACCCTGGCCAGATCGGCAACGGCTACTCGCCCGTGCTTGACTGCCACACGGCCCACGTTGCCTGCAAGTTCAAAGAGATCACGGAGAAGATGGACCGTCGTTCGGGCAAGGTGCTCGTCCAGAACAAGAAGCCGTTCATTAAGTCGCGCTTCTGCCGTGGTGTGCCCGACCCGAAGATCCGCATCTACGATGTCGGTAACAAGAAGGCTTCGGTCGACGCCTTCCCGTTCGTTGCTCACCTGGTGTCCGACGAGAAGGAGCAGCTGTCCTCAGAGGCTCTTGAGGCTGCCCGTGTTGCTGCTAACCGTTACCTGACCAAGTACTGCGGTAAGGACAACTTCCACATGCGCATCCGTTGCCACCCGTTCCAGGTCCTGCGTATCAACAAGATGCTTTCGTGCGCCGGTGCCGATAGACTGCAGACCGGTATGCGTCACGCTTACGGTAAGCCCTCGGGTGTTGCTGCCCGTGTGGCCATCGGCCAGCCCATTATCTCGGTGCGTTCGAAGGACTCGTTCGGCCCCTCGGTCGTGGAGGCTCTGCGTCGTGCACGCTGCGGAGTCGGAGAAGATTTCGGCTGACAAGCTGCAGGAGGTGTACGAGGGCTTCATTGCCAAGTGCGCTAACTCGAGCAAGATCGTGTCCATTGAGGATCCGTTCGACCAGGACGACTGGGCTTCGTGGGTGAAGTTCACGGGCAAGGTTGGCAAGGACGTTCAGATCGTGGGCGATGACCTTACGGTGACGAACCCGACCCGTGTGAAGAAGGCTATTGAGCAGAAGGCTTGCAACGCTCTTCTGCTGAAGGTGAACCAGATTGGCAGCATCACGGAGTCGATTGAGGCCGTGACGATGGCTAAGAAGGCCGGCTGGGGCATTATGGCCAGCCACCGCAGCGGTGAGACCGAGGACACGTTCATCGCCGACCTGGCTGTGGGTCTGAGCGCCGGCCAGATGGTAACAACTGGGCCAAGGGACACTACACTGAGGGTGCCGAGCTTATTGACTCGGTGCTCGACGTCGTCCGTAAGGAGGCCGAGAGCTGTGATTGCCTTCAGGGTTTCCAGATCACGCACTCGCTTGGTGGTGGTACCGGTTCCGGTATGGGTACGCTTCTTATCTCGAAGATTCGTGAGGAGTATCCCGACCGTATCATGTGCACATACTCGGTGTGCCCGTCGCCCAAGGTGTCGGACACGGTTGTGGAGCCCTACAACGCTACGCTGTCGGTGCACCAGCTTGTTGAGAACGCCGATGAGGTCATGTGTCTGGATAACGAGGCCCTGTACGACATTTGCTTCCGTACGCTGAAGCTTACCACTCCCACCTACGGTGACCTGAACCACCTCGTGTGTGCCGCCATGTCTGGTATCACCACGTGCCTGCGATTCCCCGGTCAGCTGAACTCGGACCTGCGTAAGCTGGCCGTGAACCTGATCCCGTTCCCGCGTCTTCACTTCTTTATGATTGGTTTCGCCCCGCTGACGTCGCGTGGCTCGCAGCAGTACCGTGCCCTGACGGTGCCCGAGCTGACCCAGCAGCAGTTCGACGCCAAGAACATGATGTGCGCCGCTGACCCTCGCCACGGCCGCTATTTAACTGCTGCTTGTATGTTCCGTGGACGTATGAGCACAAAGGAGGTTGATGAGCAGATGCTGAACGTGCAGAACAAGAACTCGTCGTACTTCGTCGAGTGGATCCCCAACAACATCAAGGCTAGCGTGTGTGATATCCCGCCCAAGGGTCTGAAGATGAGCACTACGTTCATCGGTAACTCTACTGCCATCCAGGAGATGTTCAAGCGTGTGTCCGAACAGTTTACGGCTATGTTCCGTCGTAAGGCTTTCTTGCACTGG

Bremia_lactucae TTTTTTAATTATGATTACAATATTTGTTTGTTGGATGTTATTTAGGGTTGTTACTCTTTTTGATGAAAAAATAAATAAAGTTCCGGCAACTATTGTTCACGGGGCTACAATTGAAATTATATGGACCTCAATTCCCGCTTTAATTCTTTTAATTGTAGCAGTACCTTCTTTTGCTTTATTATATTCAATGGATGAAATAATTGATCCTATTATTACATTAAAAGTAATCGGTAGCCAGTGGTATTGGAGCTATGAATATTCAGATAATTTAGAATTTTCAGATGAACCTTTAATTTTCGATAGTTATATGGTACAAGAGGATGATTTAGCTATAGGCCAATTTAGACTTTTAGAGGTAGATAATAGGGTAATAGTTCCAACTAATAGTCATATTCGAGTTCTGATTACAGCATCCGATGTTTTACATTCTTGGGCAATACCCTCATTAGGTATAAAACTTGATGCTTGTCCGGGGCGATTAAACCAAACCGGCGAGTGTAGCGGGATGAGCTCAAGCTTAAAATCTCCGTGCAAAAATTGCATGGCGAATTGTAGTCTATAGAGGC-GTGATTAGCGTAGGCACTTGGGGCAAGTTCCTTGGAAGAGGACAGCATTGAGGGTGATACTCCCGTTCCCT-CCCGAGTTGCTTATGCGTATAATCCGTTTTCTTTGAGTCGCGTTGTTTGAGAATGCAGCGCGAAGTAGGTGGTAAATTCCATCTAAAGCTAAATATTGGTGCGAGACCGATAGCAAACAAGTACCGTGAGGGAAAGATGAAACGAACTTTGAAAAGAGAGTTAAAGAGTACCTGAAACTGCTGAAAGGGAACCAAATCGTTTCCAGTATCTATAATCCGCGGTATATTTCATTGGCGAATGTGTGCGTGCATGTACTATGGCAGCGGCTTTTTG----CTGCGCCTTGTGTG---TG-TTTCGCATTTTCTCGCTGGTGCCCTGTACTGTGGTGGGACGTCAATGTCGGTTCGTATGCTGCGGGAAATGACTGCTGAGGAGGTAGGGCGAACGCT-TGCGTTTGTCTGTTACATCTGGGCGGACTTAGTAGTCGTGGTTGGGACTGAGGTG-CCTTTAATGTGCTTATGAGTGGGATCGAATCTCCATGTGCGCCGTGTGCGGATAGCTTGCTATGCGTGTGT---AGCTGTGTGTGGATTGAGTCGAGACGTAACTTATTGCCGTTCATTCTGGTATTGGTATGACCAAGGCTGACCTGATCAACAACTTGGGTACGATCGCCAAGTCGGGCACAAAGGCCTTCATGGAGGCGCTTGCTGCCGGTGCTGACATCAGTATGATTGGCCAGTTTGGTGTGGGTTTCTACTCGGCCTACCTCGTTGCCGATCAGGTTGTGGTCCACTCGAAGCACAACGACGATGAGCAATATGTGTGGGAGTCTGCCGCTGGAGGCTCCTTCACAGTGACTCCAGAC---ACGTCAGAGCCGATTTTGCGCGGCACGCGAATTGTCCTTAAGCTTAAGGAAGACATGCTTGAATACCTCGAGGAGCGCAAGCTTAAAGATCTAGTAAAGAAGCACTCGGAGTTCATTGGCTTCCCGATTAAGCTATACGTCGAAAAGACGGAAGAGAAGGAAGTGACTGACGACGAGGAAGACGAAGATG---AAAAGGAGGGCGACGATGACAAACCTAAGGTTGAGGAGGTTGAA---GAGGAGGAGGGCGAAAAGAAGAAGAAGACGAAGAAGGTGAAAGAAGTGACGCACGAGTGGGACCACCTTAACAGTCAGAAGCCCATCTGGATGCGCAAGCCGGAGGATGTAACCCACGAAGAGTATGCTTCCTTTTACAAGTCGCTAACCAACGACTGGGAAGAACACGCTGCTGTGAAGCACTTTTCGGTTGAGGGCCAGCTCGAATTTAAGGCCTGCCTCTTTACCCCCAAGCGTGCACCTTTCGACATGTTTGAGGGCGGTGCCAAGAAGAAATTGAACAACATCAAATTATACGTGCGTCGAGTATTCATTATGGACAACTGTGAAGACCTCATGCCTGAGTACCTCTCATTCGTCAAGGGCGTTGTCGACTCGGAGGACTTGCCGCTCAACATTTCGCGTGAAACTCTGCAGCAGAACAAGATTCTTCGTGTGATTAAGAAGAATTTGGTGAAGAAGTGTCTTGATATGTTTGCTGAGCTTGCCGAAGACAATGAAAAGTACAACAAATTCTATGAGTCGTTCAGCAAGAACCTG-AAGCTTGGCATCCACGAGGATTCCACGAACCGTACGAAAATTGCCAAGTTGCTTCGCTATCACTCGACCAAGTCTGGTGAAGAGGTCACTTCGCTTGATGACTACATCTCGCGCATGCCTGAAAACCAGCCTGGAATCTACTACGTGACGGGTGAGAGCAAGAAAGCAGTCGAGAACTCGCCATTCATCGAGAAGCTTAAAAAGAAGGGTTACGAGGTGCTTTTCATGGTTGAAGCCATCGACGAGTATGCCGTACAGCAGCTCAAGGAGTACGAGGGAAAGAAGCTCATCTGTGCCACCAAGGAGGGTTTGAAAATGGAGGAGTCGGAAGACGAGAAGAAGGCTTACGAGGAAGCGAAAGCTGCGACGGAAGGCCTTCACAGTCATTGACGCCCCTGGTCACCGCGACTTCATTAAAAACATGATTACGGGTACCTCGCAGGCTGATTGCGCCGTTTTGGTTGTTGCCTCGGGTGTGGGTGAATTCGAAGCTGGTATTTC---TAAGGAAGGCCAGACACGTGAGCACGCGTTGCTTGCTTTTACGCTCGGTGTAAAGCAAATGGTCGTTGCTATTAACAAGATGGACGACTCATCCGTGATGTACGGCCAAGCACGTTACGAAGAAATTAAGGCCGAGGTCTCGACCTACCTCAAGAAGGTTGGGTACAAGCCCGCCAAGATTCCTTGCGTGCCCATTTCCGGATGGGAGGGAGACAACATGATTGAACGCTCGCCCAACATGCCGTGGTACAAGGGACCATACCTCCTTGAAGCTCTTGACCTCTTGAACGCCCCCAAGCGTCCAAGTGACAAGCCTCTTCGTCTTCCTCTTCAGGACGTTTACAAGATTGGCGGTATTGGCACGGTTCCAGTTGGCCGTGTTGAAACTGGTGTGATTAAACCCGGTATGGTAGCTACCTTTGGCCCTGTTGGACTGTCGACTGAAGTCAAGTCTGTCGAAATGCACCACGAATCGTTGCCTGAGGCTCTACCTGGTGACAATGTTGGCTTTAACGTTAAGAATGTGTCGGTGAAGGAACTTCGCCGTGGCTTTGTCGCTTCTGATTCGAAGAATGA---CCCGGCCAAGGGCACACAAGACTTTACTGCTCAAGTCATTGTGTTGAACCACCCTGGCCAAATTGGCAATGGCTACTCGCCAGTGCTTGACTGTCACACGGCCCACGTTGCGTGCAAGTTTAAAGAAATTACGGAGAAGATGGACCGTCGTTCAGGCAAGGTGCTTGTCCAGAACAAGAAGCCTTTCATCAAATCGCGCTTCTGCCGTGGAGTACCTGACCCCAAAATTCGCATTTACGACGTGGGAAATAAGAAGGCGTCAGTGGACGCGTTTCCATACGTAGCTCATTTAGTGTCGGACGAGAAGGAGCAACTGTCGTCGGAGGCTCTCGAGGCTGCTCGAATTGCTGCTAATCGATACCTAACCAAGTTTTGCGGCAAGGACAACTTTCACATGCGTATCCGCTGCCACCCATTCCAAGTGCTTCGTATCAATAAAATGCTTTCGTGCGCTGGTGCCGACCGACTGCAGACTGGTATGCGTCACGCTTACGGCAAGCCTGCCGGTGTAGCCGCCCGCGTGGCCATTGGCCAGCCCATTATTTCAGTGCGCTCCAAAGACTCGTTTGGGCCTTCTGTCGTTGAAGCATTACGTCGCGCATGGTGCGGACTCGGAAAAGATCTCTGCTGACAAGCTGCAAGAGATTTACGAGGGCTTCATCGCCAAATGTGCGAATTCCAGCAAGCTTGTTTCCATTGAAGACCCATTTGACCAGGACGACTGGGACTCCTGGGTGAAGTTCACAAGCAAGGTTGGAAAAGACGTGCAAATTGTGGGCGATGACCTGACAGTGACAAACCCTACTCGCGTGCAAAAAGCCATCGAGCTCAAGGCGTGTAATGCCCTTTTGCTCAAGGTGAACCAAATTGGTAGCATCACGGAGTCGATTGAGGCTGTGACGATGGCTAAAAAGGCTGGCTGGGCCATAATGGCGAGCCACCGCAGCGGAGAAACTGAGGACACGTTCATTGCTGACCTAGCTGTGGGACTGAGCGCCGGTCAAATGGTAACAATTGGGCGAAAGGACATTATACCGAGGGTGCCGAGCTTATTGATTCGGTGCTTGACGTCGTTCGCAAAGAGGCAGAGAGCTGTGATTGCCTTCAGGGTTTCCAAATCACGCACTCGCTTGGTGGCGGTACTGGTTCTGGGATGGGAACGCTTCTTATTTCAAAGATTCGTGAGGAATACCCCGACCGTATCATGTGCACATACTCCGTGTGCCCTTCGCCAAAGGTATCGGATACGGTCGTGGAGCCTTACAATGCTACGCTTTCCGTGCACCAGCTCGTTGAAAACGCTGATGAAGTCATGTGCTTGGACAATGAGGCCCTGTACGATATTTGCTTTCGAACGTTGAAGCTTACCACCCCAACTTATGGTGATTTGAACCACCTGGTGTGCGCCGCCATGTCTGGTATAACTACATGCCTGCGTTTTCCAGGCCAACTGAATTCTGATCTTCGTAAATTGGCTGTAAACCTTATTCCATTCCCGCGTCTTCACTTCTTTATGATTGGCTTTGCTCCGTTGACGTCGCGTGGCTCGCAGCAATACCGTGCCCTGACGGTTCCTGAATTAACTCAACAGCAGTTTGACGCGAAGAATATGATGTGTGCCGCTGACCCTCGTCATGGTCGCTATTTAACTGCCGCATGTATGTTTCGTGGACGCATGAGCACCAAGGAGGTTGACGAGCAGATGCTTAACGTGCAGAATAAGAATTCCTCCTACTTTGTCGAGTGGATTCCCAACAACATCAAAGCTAGTGTATGTGACATTCCGCCGAAGGGTTTGAAAATGAGCACCACATTTATTGGTAATTCAACAGCTATCCAAGAGATGTTCAAGCGAGTGTCAGAACAGTTCACGGCCATGTTCCGTCGTAAGGCTTTCTTGCACTGG

Phytophthora_infestans TTTTTTAATTATGATTACTGTGTTTGTTTGTTGGATGTTATTTAGAGTTATTACTCTTTTTGATGAAAAAAAAAATAAAATACCATCAACTGTTGTACATGGAGCTACTATAGAAATTATTTGGACTTCAATTCCAGCTTTAATTTTATTAATAGTTGCAGTTCCTTCTTTCGCGTTATTATATTCAATGGATGAAGTAATTGACCCAATTATTACTTTAAAAGTAATTGGTAGTCAATGGTATTGGAGTTATGAATATTCAGATAATTTAGAATTTTCAGATGAACCTTTAATTTTTGATAGTTATATGGTACAAGAAGATGATTTAGCTATTGGTCAATTTAGACTTTTAGAAGTGGATAATCGTGTAGTAGTTCCAACTAATAGTCATATTAGAGTATTAATTACTGCATCAGATGTATTACATTCATGGGCTATACCGTCATTAGGTATAAAATTAGATGCTTGTCCAGGTCGTTTAAATCAAACCGGCGAGTGAAGCGGGAAGAGCTCAAGCTTAAAATCTCCGTGCAAGTTTTGCGCGGCGAATTGTAGTCTATAGAGGC-GTGATCAGTGTGGGCGCTTGGGGCAAGTTCCTTGGAAGAGGACAGCATGGAGGGTGATACTCCCGTTCATC-CCTGAGTTGCTCGTGCGTACGATCCGTTTTCTTTGAGTCGCGTTGTTTGGGAATGCAGCGCAAAGTAGGTGGTAAATTCCATCTAAAGCTAAATATTGGTGCGAGACCGATAGCGAACAAGTACCGTGAGGGAAAGATGAAAAGAACTTTGAAAAGAGAGTTAAAGAGTACCTGAAACTGCTGAAAGGGAACCGAATCGTTTCCAGTGTCTATAATCCGTGGTATATTTCATTGGCGAGTGTGTGCGTGCGTGTGCTGTGGCAGCGGCTTTTTT--GGCTGCGCTCGGCGTG---TG-TGCTGTGTGTGCTTGCTGGTGCCCTGTGCTGCGGTGGGACGTCAAGGTCAGTTCGTATGCTGCGGGAAATGGCCGCCGAGGAGGTAGGGCTTACGCT-TGCGTTTGTCTGTTATATCTTGGTGGACG-AGTAGTCGCGGTTGGGACTGAGGTG-CCTACAACGTGTTTTTGAGTGGGCCTGTGTCTCTGTGTGCGCCGTGTGCGGATAGCTTGCTATGCGTGTGT---GGTTGTGTGTGGATTGATGTGGGTTTTAACTTGTTGCCGTTCACTCTGGTATTGGTATGACGAAGGCTGACCTGATTAACAACCTGGGTACGATCGCCAAGTCGGGCACCAAGGCCTTCATGGAGGCTCTGGCTGCTGGCGCCGACATCAGTATGATTGGTCAGTTCGGTGTGGGTTTCTACTCGGCCTACCTGGTCGCTGACAAGGTCGTGGTGCACTCGAAGCACAACGATGACGAGCAGTACGTGTGGGAGTCTGCTGCCGGTGGCTCGTTCACTGTCACGCCYGAC---ACGTCGGAGCCCATCCAGCGCGGTACGCGCATTGTGCTGAAGCTCAAGGAGGACATGCTCGAGTACCTTGAGGAGCGCAAGCTTAAGGACCTGGTGAAGAAGCACTCGGAGTTCATTGGCTTCCCCATCAAGCTGTACGTCGAGAAGACGGAGGAGAAGGAAGTGACGGACGACGAGGAAGAGGAGGACG---AGAAGGAGGGCGAGGACGACAAGCCCAAGGTCGAGGAGGTCGAA---GAGGAGGAAGGCGAGAAGAAGAAGAAGACTAAGAAGATTAAGGAAGTGACCCACGAGTGGGACCACCTCAACAGCCAGAAGCCCATCTGGATGCGCAAGCCTGAAGACGTGACCCACGAGGAGTACGCGTCGTTCTACAAGTCGCTGACGAACGACTGGGAGGAGCACGCCGGCGTCAAGCACTTTTCGGTGGAGGGCCAGCTGGAGTTCAAGGCYTGTCTGTTCACCCCCAAGCGCGCGCCGTTCGACATGTTTGAGGGCGGCGCCAAGAAGAAAGTGAACAACATCAAGCTGTACGTGCGTCGCGTCTTCATCATGGACAACTGCGAGGAGCTCATGCCCGAGTACCTGTCGTTCGTCAAGGGTGTCGTCGACTCGGAGGACTTGCCGCTTAACATCTCGCGTGAGACGCTGCAGCAGAACAAGATCCTRCGTGTGATCAAGAAGAACCTGGTCAAGAAGTGCCTCGAGATGTTCGCCGAACTGGCTGAGGACAACGAGAAGTACAACAAGTTCTACGAGTCGTTCAGCAAGAACCTC-AAGCTGGGCATCCACGAGGACTCGACCAACCGCACCAAGATCGCCAAGCTGCTGCGTTACCACTCCACCAAGTCTGGCGAGGAAGTGACGTCGCTGGATGACTACATCTCGCGTATGCCTGAGAGCCAGCCCGGTATCTACTACGTGACTGGCGAGAGCAAGAAGTCGGTGGAGAACTCGCCATTCATTGAGAAGCTCAAGAAGAAGGGCTACGAGGTGCTGTTCATGGTGGAGGCCATTGACGAGTACGCTGTGCAGCAGCTCAAGGAGTACGAGGGCAAGAAGCTCATCTGCGCCACCAAGGAGGGCCTCAAGATGGAGGAGACGGAGGACGAGAAGAAGTCGTTCGAGGAGGCCAAGGCTGCCACTGAAGGACTTCACGGTCATTGACGCCCCTGGTCACCGTGACTTCATCAARAACATGATTACGGGTACCTCGCAGGCCGATTGCGCCATTCTGGTGGTCGCTTCGGGTGTGGGTGAGTTCGAGGCTGGTATCTC---CAAGGAGGGCCAGACTCGTGAGCACGCTCTGCTTGCCTTCACTCTGGGTGTGAAGCAGATGRTCGTCGCCATYAACAAGATGGACGACTCGTCTGTCATGTACGGCCAGGCCCGTTACGAGGAGATCAAGTCTGAGGTCACCACGTACCTGAAGAAGGTTGGCTACAAGCCCGCCAAGATCCCGTTCGTGCCYATCTCCGGCTGGGAGGGTGACAACATGATCGACCGCTCCACCAACATGCCGTGGTACAAGGGACCTTTCCTCCTTGAGGCTCTTGACAACCTGAACGCCCCCAAGCGCCCGTCKGACAAGCCGCTGCGTCTGCCCCTTCAGGAYGTGTACAAGATCGGCGGTATTGGCACGGTACCTGTCGGCCGTGTGGAGACCGGTGTCATCAAGCCTGGCATGGTCGCCACTTTCGGCCCCGTTGGTCTGTCGACTGAAGTCAAGTCYGTCGAGATGCACCACGAGTCTCTGCCTGAGGCTGTCCCTGGTGACAACGTCGGCTTCAACGTCAAGAACGTGTCGGTCAAGGAGCTGCGTCGTGGTTTCGTCGCTTCGGACTCSAAGAACGA---CCCTGCTAAGGCAACCCAGGACTTCACCGCCCAGGTGATTGTGCTGAACCACCCTGGYCAGATCGGCAACGGTTACTCGCCTGTGCTTGACTGCCACACGGCCCACGTTGCCTGCAAGTTYAAAGAGATTACGGAGAAGATGGACCGTCGTTCGGGCAAGGTGCTCGTCCAGAACAAGAAGCCGTTCATTAAGTCGCGCTTCTGCCGTGGTGTGCCCGACCCGAAGATCCGCATCTACGATGTCGGTAACAAGAAGGCTTCGGTCGACGCCTTCCCCTTTGTGTGCCACTTGGTGTCTGACGAGAAGGAGCAGCTTTCCTCGGAGGCTCTTGAGGCTGCCCGTATTGCTGCTAACCGTTACCTGACCAAGTACTGCGGCAAGGACAACTTCCACATGCGCATCCGTTGCCACCCGTTCCAGGTCCTGCGTATTAACAAAATGCTTTCGTGCGCCGGTGCCGATAGACTGCAGACCGGTATGCGTCACGCTTACGGTAAGCCCTCGGGTGTCGCTGCCCGTGTGGCYATTGGTCAGCCTATTATCTCGGTGCGYTCYAAGGACTCGTTCGGCCCTTCGGTCGTGGAGGCTCTGCGTCGTGCACGCTGCGGAGTCGGAGAAGATCCCGGCCGACAAGCTGCTGGAGGTGTACGAGGGCTTCATCGCCAAGWGCGCTGACTCGAGCAAGATCGTGTCCATTGAGGACCCGTTCGACCAGGATGACTGGGAGTCGTGGGTGAAGATCACTAGCAAGGTGGGCAAGGACGTGCAGATCGTGGGTGACGACCTCACGGTGACGAACCCGACCCGTGTAAAGAAGGCCATCGAGCAGAAGGCTTGCAATGCTCTTCTGTTGAAGGTGAACCAGATTGGCAGCATTACGGAGTCGATCGAGGCCGTGACGATGGCCAAGAAGGCCGGCTGGGGCATTATGGCCAGCCACCGTAGTGGTGAGACAGAGGACACGTTCATCGCTGACCTGGCTGTGGGTCTGAGTGCCGGCCAGATGGTAACAACTGGGCCAAGGGACACTACACTGAGGGCGCTGAGCTGATTGACTCGGTGCTTGACGTCGTTCGCAAGGAGGCAGAGAGCTGTGATTGCCTTCAGGGTTTCCAGATCACGCACTCGCTTGGTGGCGGTACCGGTTCCGGTATGGGTACGCTTCTTATCTCGAAGATTCGTGAGGAGTACCCCGATCGCATCATGTGCACATACTCGGTCTGCCCGTCGCCCAAGGTATCGGACACGGTCGTGGAGCCCTATAACGCTACGCTATCGGTACACCAGCTTGTCGAGAACGCCGATGAGGTCATGTGCCTGGACAATGAGGCCCTGTACGACATTTGCTTCCGCACATTGAAGCTCACCACCCCCACTTATGGTGACCTGAACCACTTGGTGTGTGCCGCCATGTCCGGTATTACCACGTGCCTTCGTTTCCCCGGTCAGCTGAACTCGGACCTGCGTAAGCTGGCCGTGAACCTGATCCCGTTCCCGCGTCTCCACTTCTTTATGATTGGTTTCGCTCCTCTGACATCGCGCGGCTCGCAGCAGTACCGCGCCCTGACGGTGCCCGAGCTGACCCAGCAGCAGTTCGATGCTAAGAACATGATGTGTGCCGCCGACCCTCGCCATGGCCGCTATTTAACTGCAGCTTGTATGTTCCGCGGACGCATGAGCACGAAGGAGGTTGATGAGCAGATGCTGAACGTGCAGAACAAGAACTCGTCATACTTCGTCGAGTGGATCCCCAACAACATCAAGGCTAGCGTGTGTGACATCCCGCCCAAGGGTCTGAAGATGAGCACTACGTTCATTGGTAACTCTACTGCTATCCAAGAGATGTTCAAGCGTGTGTCCGAACAGTTTACGGCTATGTTCCGTCGTAAGGCTTTCTTGCACTGG

Phytophthora_capsici TTTTTTAATTATGATTGTTGTATTTGTATGTTGGATGTTATTTAGAGTTATTACTCTTTTTGATGAAAAAAAAAATAAAATTCCAGCAACTGTTGTACATGGTGCAACTATTGAAATTATTTGGACTTCTATTCCAGCTTTAATTTTATTAACAGTAGCTGTTCCATCTTTTGCATTATTATATTCAATGGACGAAGTAATAGATCCTATTATTACTTTAAAAGTAATAGGTAGTCAATGGTATTGGAGTTATGAATATTCAGATAATTTAGAATTTTCAGATGAACCTTTAATTTTTGATAGTTATATGGTACAAGAAGATGATTTAGCAATAGGTCAATTTAGACTTTTAGAAGTAGATAATCGTGTAGTAGTTCCAACTAATAGTCATATTAGAGTATTAATTACAGCATCAGATGTTTTACATTCTTGGGCTATACCTTCATTAGGTTTAAAATTAGATGCATGTCCTGGCCGTTTAAATCAAACCGGCGAGTGAAGCGGGAAGAGCTCAAGCTTAAAATCTCCGTGCAAGTTTTGCGCGGCGAATTGTAGTCTATAGAGGC-GTGGTCAGCGTGGGCGCTTGGGGCAAGTTCCTTGGAAGAGGACAGCATGGAGGGTGATACTCCCGTTCATC-CCTGAGTGGCTCGTGCGTACGACCCGTTTTCTTTGAGTCGCGTTGTTTGGGAATGCAGCGCAAAGTAGGTGGTAAATTCCATCTAAAGCTAAATATTGGTGCGAGACCGATAGCGAACAAGTACCGTGAGGGAAAGATGAAAAGAACTTTGAAAAGAGAGTTAAAGAGTACCTGAAACTGCTGAAAGGGAACCGAATCGTTTCCAGTGTCTATAATCCGTGGCATATTTCATTGGCGAGCGTTTGCGTGCGTGTGCTGTGGCAGTGGCTTTTTT--GGCTGCGCCTGGCGTG---TG-TGCTGTGAGTGTTTGCTGGTGCCCTGTGCTGCGGTGGGACGTCAAGGTCAGTTCGTATGCTGCGGGAAATGGCTGCCGAGGAGGTAGGGCTTACGCTCCGCGTTTGTCTGTTATATCTTGGTGGACG-AGTCGTCGTGGTTGGGACTGAGGTG-CCTACAACGTGCTTTTGAGTGGGTTTGTGTCTCCGTGTGCGCCGTGTGCGGATAGCTTGCTATGCGTGTGT---GGTTGTGTGTGGATTGATGCGGGCCTTAACTTGTCGCCGTTCACTCGGGTATTGGTATGACCAAGGCCGACCTCATCAACAACCTGGGTACGATCGCCAAGTCGGGCACCAAGGCCTTCATGGAGGCTCTTGCTGCTGGCGCTGACATCAGTATGATCGGTCAGTTCGGTGTGGGTTTCTACTCTGCCTACCTGGTGGCCGACAAGGTCGTGGTGCACTCGAAGCACAACGATGACGAGCAGTACGTGTGGGAGTCGGCTGCTGGTGGCTCGTTCACTGTGACGCTCGAC---ACTTCGGAGCCCATTCTGCGTGGTACGCGCATCGTCCTGACCCTGAAGGAGGACATGCTGGAGTACCTGGAGGAGCGCAAGCTTAAAGACCTGGTGAAGAAGCACTCTGAATTCATTGGCTTCCCCATCAAGCTCTACGTCGAGAAGACGGAGGAGAAGGAGGTGACTGACGACGAGGAGGAGGAAGACG---AGAAGGAGGACGAGGACGACAAGCCCAAGGTCGAGGAGGTCGAA---GATGAGGAGGGTGAGAAGAAGAAGAAGACCAAGAAGATCAAGGAGGTGACCCACGAGTGGGACCACCTGAACTCGCAGAAGCCCATCTGGATGCGCAAGCCCGAGGATGTGACCCACGAGGAGTACGCTTCTTTCTACAAGTCGCTGACCAACGACTGGGAGGAGCACGCTGCCGTCAAGCACTTCTCGGTGGAGGGCCAGCTCGAGTTCAAGGCTTGTCTGTTCGCCCCCAAGCGTGCCCCGTTCGACATGTTCGAGGGTGGTGCCAAGAAGAAACTGAACAACATTAAGCTGTACGTGCGTCGCGTGTTCATCATGGACAACTGCGAGGAGCTCATGCCCGAGTACCTGTCGTTCGTCAAGGGTGTTGTCGACTCGGAGGACCTGCCGCTTAACATCTCGCGTGAGACCCTGCAGCAGAACAAGATCCTGCGCGTGATCAAGAAGAACCTGGTGAAGAAGTGCCTGGAGATGTTCGCGGAGCTCGCCGAGGACAACGAGAAGTACAACAAGTTCTACGAGGCCTTCAGCAAGAACCTG-AAGCTCGGCATCCACGAGGACTCGACCAACCGCACCAAGATCGCCAAGCTTCTGCGTTACCACTCGACCAAGTCTGGTGAGGAGATGACGTCCCTGGACGACTACATCTCGCGCATGCCCGAGAACCAGCCCGGCATCTACTACGTGACGGGCGAGAGCAAGAAGTCGGTGGAGAACTCGCCGTTCATTGAGAAGCTCAAGAAGAAGGGCTACGAGGTGCTTTTCATGGTTGAGGCCATCGACGAGTACGCTGTGCAGCAGCTGAAGGACTACGAGGGCAAGAAGCTCATCTGTGCCACCAAGGAGGGCCTTAAGATGGAGGAGACCGAGGACGAGAAGAAGTCGTTCGAGGAGGCCAAGGCCGCTACTGAGGGTCTTCACGGTCATTGACGCCCCTGGTCACCGTGACTTCATCAAGAACATGATTACGGGTACYTCGCAGGCYGATTGCGCCATTCTGGTGGTYGCTTCGGGTGTGGGTGAGTTCGAGGCTGGTATCTC---CAAGGAGGGCCAGACCCGTGAGCACGCYCTGCTTGCCTTCACTCTGGGTGTGAAGCAGATGATCGTTGCCATCAACAAGATGGACGACTCGTCTGTCATGTACGGCCAGGCCCGTTACGAGGAGATCAAGAACGAGGTCACCACGTACCTGAAGAAGGTTGGCTACAAGCCGGCCAAGATCCCGTTCGTGCCTATCTCCGGCTGGGAGGGTGACAACATGATTGACCGTTCCACCAACATGCCGTGGTACAAGGGACCTTACCTCCTTGAGGCTCTTGACAACCTGAACGCCCCYAAGCGYCCGGTTGACAAGCCGCTTCGTCTGCCCCTCCAGGATGTGTACAAGATCGGCGGTATTGGCACGGTACCTGTCGGCCGTGTGGAGACTGGTGTCATCAAGCCTGGCATGGTCGCCACTTTCGGCCCCGTTGGTCTGTCGACTGAAGTTAAGTCCGTGGAGATGCACCACGAGTCCCTGCCGGAGGCTGTCCCTGGTGACAACGTCGGCTTCAACGTCAAGAACGTGTCGGTGAAGGAGCTGCGTCGTGGTTTCGTCGCTTCGGACTCCAAGAACGA---CCCCGCTAAGGGCACCCAGGACTTCACCGCTCAGGTGATTGTGCTGAACCACCCTGGCCAGATCGGCAACGGTTACTCGCCGGTGCTTGATTGCCACACGGCCCACGTTGCCTGCAAGTTCAAAGAGATCACGGAGAAGATGGACCGTCGTTCGGGCAAGGTGCTCGTCCAGAACAAGAAGCCGTTCATTAAGTCGCGCTTCTGCCGTGGTGTGCCCGACCCGAAGATCCGCATCTACGATGTCGGTAACAAGAAGGCTTCAGTGGACGCCTTCCCCTTCGTTGCTCACTTGGTGTCCGACGAGAAGGAGCAGCTGTCCTCGGAGGCTCTTGAGGCTGCCCGTATTGCTGCTAACCGTTACCTGACCAAGTTCTGCGGCAAGGACAACTTCCACATGCGCATCCGTTGCCACCCGTTCCAGGTCCTGCGTATTAACAAGATGCTTTCGTGCGCCGGTGCCGATAGACTGCAGACCGGTATGCGTCACGCTTACGGTAAGCCCGCCGGTGTGGCTGCCCGTGTGGCCATTGGCCAGCCCATTATCTCGGTGCGTTCCAAGGACTCGTTCGGCCCTTCGGTCGTGGAGGCTCTGCGTCGTGCACGCTCCGGAGTCAGAGAAGATCCCGGCCGACAAGCTGCAGGAGGTGTACGAGGGCTTTATCGCCAAGTGCGCTGACTCGAGCAAGATCGTGTCGATTGAGGACCCGTTCGACCAGGACGACTGGGCGTCGTGGGTGAAGTTCACGGCCAAGGTGGGCAAGGACGTGCAGATCGTGGGCGATGATCTTACGGTGACGAACCCGACGCGTGTGAAGAAGGCCATCGAGCAGAAGGCTTGCAACGCGCTTCTGCTGAAGGTGAACCAGATTGGCAGCATCACGGAGTCGATTGAGGCCGTGACGATGGCGAAGAAGGCCGGCTGGGCCATCATGGCTAGCCACCGCAGCGGTGAGACGGAGGACACGTTCATCGCCAGCCTGGCTGTGGGTCTGAGCGCTGGCCAGATGGTAACAACTGGGCCAAGGGACACTACACAGAGGGTGCTGAGCTTATTGACTCGGTGCTTGATGTCGTCCGTAAGGAGGCTGAGAGCTGTGACTGCCTGCAGGGTTTCCAGATCACCCACTCTCTTGGTGGTGGTACCGGTTCCGGTATGGGTACGCTTCTTATCTCGAAGATTCGTGAGGAGTACCCCGACCGTATCATGTGCACATACTCGGTGTGCCCATCGCCCAAGGTGTCGGACACCGTTGTGGAGCCTTACAACGCTACGTTGTCGGTGCACCAGCTGGTCGAGAACGCCGATGAGGTCATGTGTCTGGATAACGAGGCCCTGTACGATATTTGCTTCCGTACGCTGAAGCTTACTACTCCCACTTACGGTGACCTGAACCACTTGGTGTGTGCCGCCATGTCCGGTATCACCACGTGTCTGCGTTTCCCCGGTCAGTTGAACTCGGACCTGCGTAAGCTGGCCGTGAACCTGATTCCGTTCCCGCGTCTTCACTTCTTTATGATTGGTTTCGCCCCGTTGACCTCGCGTGGATCTCAGCAGTACCGTGCCCTCACGGTGCCCGAGCTGACCCAACAGCAGTTCGATGCTAAGAACATGATGTGCGCCGCTGACCCTCGCCACGGCCGCTATTTAACTGCCGCGTGTATGTTCCGCGGACGTATGAGTACGAAGGAGGTTGACGAGCAGATGCTGAACGTGCAGAACAAGAACTCTTCGTACTTCGTCGAGTGGATCCCCAACAACATCAAGGCTAGCGTTTGTGATATTCCTCCCAAGGGTCTGAAGATGAGCACTACGTTCATCGGTAACTCGACCGCCATTCAGGAGATGTTCAAGCGTGTGTCCGAACAGTTTACGGCTATGTTCCGTCGTAAGGCTTTCTTGCACTGG

Phytophthora_plurivora_genome TTTTTTAATTATGATTGTTGTATTTGTATGTTGGATGTTATTTAGAGTTGTTACTCTTTTTGATGAAAAAAAAAATAAAATTCCAGCAACTGTTGTACATGGTGCAACTATTGAGATTATTTGGACTTCTATTCCAGCTTTAATTTTATTAACTGTAGCAATACCCTCTTTTGCATTATTATATTCAATGGATGAAGTAATTGATCCCATTATTACTTTAAAAGTAATAGGTAGTCAATGGTACTGGAGTTATGAATATTCAGATAATTTAGAATTTTCTGATGAACCTTTAATTTTTGATAGTTATATGATACAAGAAGATGATTTAGCTATAGGTCAATTTAGACTTTTAGAAGTAGATAATCGTGTAGTAGTTCCAACTAATAGTCATATTAGAGTATTAATTACAGCATCAGATGTTTTACATTCTTGGGCTATACCATCATTAGGTATAAAATTAGATGCATGTCCTGGACGTTTAAATCAAACCGGCGAGTGAAGCGGGAAGAGCTCAAGCTTAAAATCTCCGTGCAAGTTTTGCGCGGCGAATTGTAGTCTATAGAGGC-GTGGTCAGCGTGGGCGCTTGGGGCAAGTTCCTTGGAAGAGGACAGCATGGAGGGTGATACTCCCGTTCATC-CCTGAGTGGCTCGTGCGTACGACCCGTTTTCTTTGAGTCGCGTTGTTTGGGAATGCAGCGCAAAGTAGGTGGTAAATTCCATCTAAAGCTAAATATTGGTGCGAGACCGATAGCGAACAAGTACCGTGAGGGAAAGATGAAAAGAACTTTGAAAAGAGAGTTAAAGAGTACCTGAAACTGCTGAAAGGGAACCGAATCGTTTCCAGTGTCTATAATCTGTGGCATATTTCATTGGCGAGCGTTTGCGTGCGTGTGCTGTGGCAGTGGCTTTTTT--GGCTGCGCCCGGTGTG---TG-TGCTGTGAGTGTTTGCTGGTGCCCTGTGCTGCGGTGGGACGTCAAGGTCAGTTCGTATGCTGCGGGAAATGGCTGCCGAGGAGGTAGGGCTTACGCTCCGCGTTTGTCTGTTATATCTTGGTGGACG-AGTCGTCGCGGTTGGGACTGAGGTG-CCTACAACGTGCTTTTGAGTGGGTTTGTGTCTCCGTGTGCGCCGTGTGCGGATAGCTTGCTATGCGTGTGT---GGTTGTGTGTGGATGGATGCGGGCCTTAACTTGTCGCCGTTCACTCGGGTATTGGTATGACCAAGGCCGACCTCATCAACAACCTGGGAACGATCGCCAAGTCTGGCACCAAGGCCTTCATGGAGGCTCTGGCTGCCGGCGCTGACATCAGCATGATCGGTCAGTTCGGTGTGGGTTTCTACTCCGCCTACCTGGTCGCTGACAAGGTCGTGGTGCACTCGAAGCACAACGATGACGAGCAGTACGTGTGGGAGTCGGCTGCTGGTGGCTCGTTCACTGTGACGCCCGAC---ACGACGGAGCCCATTCTGCGTGGTACGCGCATCGTCCTGACGCTGAAGGAGGACATGCTGGAGTACCTGGAGGAGCGCAAGCTCAAGGACCTCGTGAAGAAGCACTCCGAGTTCATTGGTTTCCCCATCAAGCTCTACGTCGAGAAGACGGAGGAGAAGGAGGTGACTGACGATGAGGAGGAGGAAGACG---AGAAGGAGGGCGAGGACGACAAGCCCAAGGTCGAGGAGGTCGAA---GACGAGGAGGGCGAGAAGAAGAAGAAGACCAAGAAGATCAAGGAGGTGACCCACGAGTGGGACCACCTGAACTCGCAGAAGCCCATCTGGATGCGCAAGCCTGAGGACGTGACCCACGAGGAGTACGCCTCGTTCTACAAGTCGCTCACTAACGACTGGGAGGAGCACGCTGGCGTCAAGCACTTCTCGGTGGAGGGCCAGCTGGAGTTCAAGGCGTGCCTGTTCGCCCCCAAGCGCGCTCCGTTCGACATGTTCGAGGGTGGTGCCAAGAAGAAACTGAACAACATCAAGTTGTACGTGCGTCGCGTGTTCATCATGGACAACTGCGAGGAACTCATGCCTGAGTACCTGTCGTTCGTCAAGGGTGTTGTCGACTCGGAGGACCTGCCGCTTAACATCTCGCGTGAGACCCTGCAGCAGAACAAGATCCTGCGTGTCATCAAGAAGAACCTGGTGAAGAAGTGCCTGGAGATGTTCGCGGAGCTCGCCGAGGATAACGAGAAGTACAACAAGTTCTACGAGGCCTTCAGTAAGAACCTC-AAGCTCGGCATCCACGAGGACTCGACCAACCGCACCAAGATCGCCAAGCTTCTGCGTTACCACTCGACCAAGTCTGGCGAGGAGATGACGTCCCTGGACGACTACATCTCGCGCATGCCCGAGAACCAGCCCGGCATCTACTACGTGACGGGTGAGAGCAAGAAGTCGGTGGAGAACTCGCCGTTCATTGAGAAGCTCAAGAAGAAGGGATACGAGGTGCTCTTCATGGTCGAGGCTATTGACGAGTACGCTGTGCAGCAGCTGAAGGAGTACGAAGGCAAGAAGCTCATCTGCGCCACCAAGGAGGGCCTCAAGATGGAGGAGACTGAGGACGAGAAGAAGTCGTTCGAGGAGGCCAAGGCCGCCACTGAGGGCCTTCACGGTCATTGACGCCCCTGGCCACCGTGACTTCATCAAGAACATGATTACGGGCACCTCGCAGGCCGATTGCGCCATTCTGGTGGTTGCTTCGGGTGTGGGTGAGTTCGAGGCTGGTATCTC---CAAGGAGGGCCAGACTCGTGAGCACGCTCTGCTTGCCTTCACCCTGGGTGTGAAGCAGATGATCGTCGCCATCAACAAGATGGACGACTCGTCTGTCATGTACGGCCAGGCCCGTTACGAAGAGATCAAGTCGGAGGTGACGACCTACCTGAAGAAGGTGGGCTACAAGCCGGCCAAGATCCCGTTCGTGCCCATCTCCGGCTGGGAGGGTGACAACATGATTGACCGCTCCACCAACATGCCGTGGTACAAGGGACCCTTCCTTCTTGAGGCTCTTGACAACCTGAACGCCCCCAAGCGCCCGAGCGACAAGCCCCTGCGTCTGCCCCTTCAGGATGTGTACAAGATCGGCGGTATCGGCACGGTACCGGTCGGCCGTGTGGAGACTGGTGTCATCAAGCCTGGCATGGTCGCCACTTTCGGCCCCGTTGGTCTGTCGACTGAAGTCAAGTCCGTGGAGATGCACCACGAGTCTCTGCCGGAGGCTGTCCCTGGTGACAACGTCGGCTTCAACGTCAAGAACGTGTCGGTCAAGGAGCTGCGTCGTGGTTTCGTCGCTTCGGACTCCAAGAACGA---CCCCGCCAAGGCCACCCAGGACTTCACCGCCCAGGTGATTGTGCTGAACCACCCTGGCCAGATCGGCAACGGCTACTCGCCGGTGCTTGACTGCCACACGGCCCACGTTGCCTGCAAGTTCAAAGAGATCACGGAGAAGATGGACCGTCGTTCGGGCAAGGTGCTCGTCCAGAACAAGAAGCCGTTCATTAAGTCGCGCTTCTGCCGTGGTGTGCCCGACCCGAAGATCCGCATCTACGATGTCGGTAACAAGAAGGCTTCGGTGGACGCCTTCCCCTTCGTTGCTCACTTGGTGTCCGACGAGAAGGAGCAGCTGTCCTCGGAGGCTCTTGAGGCTGCCCGTATTGCTGCTAACCGTTACCTGACCAAGTACTGCGGCAAGGACAACTTCCACATGCGCATCCGTTGCCACCCGTTCCAGGTCCTGCGTATTAACAAGATGCTTTCGTGCGCCGGTGCCGATAGACTGCAGACCGGTATGCGTCACGCTTACGGTAAGCCCGCCGGTGTGGCCGCCCGTGTGGCCATTGGCCAGCCCATTATCTCGGTGCGTTCCAAGGACTCGTTCGGCCCCTCGGTCGTGGAGGCTCTGCGTCGTGCACGCTCCGGAGTCGGAGAAGATCCCGGCCGACAAGCTGCAGGAGGTGTACGAGGGCTTCATCGCGAAGTGCGCTGGCTCGAGCAAGATCGTGTCGATTGAGGACCCGTTCGACCAGGACGACTGGGAGTCGTGGGTGAAGTTCACGGCCAAGGTGGGCAAGGACGTGCAGATCGTGGGCGACGACCTTACGGTGACGAACCCGACCCGTGTGCAGAAGGCCATCGACCAGAAGGCTTGCAACGCGCTTCTGCTGAAGGTGAACCAGATTGGCAGCATCACGGAGTCGATCGAGGCCGTGACGATGGCCAAGAAGGCCGGCTGGGCCATCATGGCCAGCCACCGTAGTGGTGAGACGGAGGACACGTTCATCGCGGACCTGGCTGTGGGTCTGAGCGCGGGCCAGATGGTAACAACTGGGCCAAGGGACACTACACAGAGGGTGCCGAGCTTATCGACTCGGTGCTTGATGTCGTCCGCAAGGAGGCTGAGAGCTGTGACTGCCTGCAGGGTTTCCAGATCACCCACTCGCTTGGTGGCGGTACCGGTTCCGGTATGGGTACGCTTCTTATCTCGAAGATTCGTGAGGAGTACCCAGACCGTATTATGTGCACGTACTCGGTCTGCCCGTCGCCCAAGGTGTCGGACACCGTCGTGGAGCCCTACAACGCCACGTTGTCGGTGCATCAGCTGGTCGAGAACGCCGATGAGGTCATGTGCCTGGATAACGAGGCCCTGTACGATATTTGCTTCCGTACGCTGAAGCTCACCACGCCCACCTACGGTGACCTGAACCACCTGGTGTGCGCCGCCATGTCCGGTATCACCACGTGCCTGCGTTTCCCCGGTCAGTTGAACTCGGACCTGCGTAAGCTGGCCGTGAACCTGATCCCGTTCCCGCGTCTCCACTTCTTTATGATTGGTTTCGCTCCGCTGACCTCGCGTGGATCTCAGCAGTACCGTGCTCTTACGGTGCCGGAGCTGACCCAGCAGCAGTTCGATGCCAAGAACATGATGTGCGCCGCTGACCCTCGCCACGGCCGCTATTTAACTGCCGCGTGTATGTTCCGCGGACGTATGAGCACGAAGGAGGTTGACGAGCAGATGCTGAACGTGCAGAACAAGAACTCATCGTACTTCGTCGAGTGGATCCCCAACAACATCAAGGCTAGCGTGTGTGATATTCCTCCCAAGGGTCTGAAGATGAGCACTACGTTCATCGGTAACTCGACTGCCATCCAGGAGATGTTCAAGCGTGTGTCTGAACAGTTTACGGCTATGTTCCGTCGTAAGGCTTTCTTGCACTGG

Phytophthora_cinnamomi TTTTTTAATTACTATTACTGTTTTTGTGTGTTGGATGTTATTTAGAGTTATTACTCTTTTTGATGAAGAAAAAAATAAAATTCCAGCAACTGTTGTGCATGGAGCAACTATTGAAATTATTTGGACTTCTATACCTGCTTTAATTTTATTAACTGTTGCTATACCATCATTTGCTTTATTATATTCAATGGATGAAGTTATTGATCCAATTATTACATTAAAAGTAATAGGTAGTCAATGGTATTGGAGTTATGAATATTCAGATAATTTAGAATTTTCAGATGAACCTTTAATTTTTGATAGTTATATGGTACAAGAAGATGATTTAGCTATTGGTCAATTTAGACTTTTAGAAGTAGATAATCGTGTAGTTGTTCCAACTAATAGTCATATTAGAGTTTTAATTACAGCATCAGATGTTTTACATTCATGGGCTATACCATCATTAGGTATTAAATTAGATGCTTGTCCTGGTCGTTTAAATCAAACCGGCGAGTGAAGCGGGAAGAGCTCAAGCTTAAAATCTCCGTGCAAGTTTTGCGCGGCGAATTGTAGTCTATAGAGGC-GTGGTCAGCGTGGGCGCTTGGGGCAAGTTCCTTGGAAGAGGACAGCATGGAGGGTGATACTCCCGTTCATC-CCTGAGTGGCTCGTGCGTACGACCCGTGTTCTTTGAGTCGCGTTGTTTGGGAATGCAGCGCAAAGTAGGTGGTAAATTCCATCTAAAGCTAAATATTGGTGCGAGACCGATAGCGAACAAGTACCGTGAGGGAAAGATGAAAAGAACTTTGAAAAGAGAGTTAAAGAGTACCTGAAACTGCTGAAAGGGAACCGAATCGTTTCCAGTGTCTATAATCCGTGGCATATTTCATTGGCGAGTGTGCGCGTGCGTGTGCTGTGGCAGTGGCTTTTTT--GGCTGCGCTCGGTGCG---TG-TGCTGTGTGTGCTTGCTGGTGCCCTGTGCTGCGGTGGGACGTCAAGGTCAGTTCGTATGCTGCGGGAAATGGCTGCCGAGGAGGTAGGGCTTACGCTCCGCGTTTGTCTGTTATATCTTGGTGGACG-AGTCGTCGTGGTTGGGACTGAGGTG-CCTACAACGTGCTTTTGAGTGGGTTCGTGTCTCCGTGTGCGCCGTGTGCGGATAGCTTGCTATGCGTGTGT---GGTTGTGCGTGGATTGGTGCGGGCTTTAACTTGTCGCCGTTCACTCGGGCATCGGCATGACCAAGGCCGACCTCATCAACAACCTGGGCACGATCGCCAAGTCGGGCACCAAGGCCTTCATGGAGGCCCTGGCCGCCGGCGCCGACATCAGCATGATCGGCCAGTTCGGCGTGGGCTTCTACTCGGCCTACCTGGTGGCCGACAAGGTCGTGGTGCACTCGAAGCACAACGACGACGAGCAGTACGTGTGGGAGTCCGCCGCCGGCGGCTCGTTCACGGTCACGCCCGAC---ACGTCGGAGCCCATCCTGCGCGGCACSCGCATCGTGCTGAAGCTCAAGGAGGACATGCTCGAGTACCTCGAGGAGCGCAAGCTGAAAGACCTGGTGAAGAAGCACTCGGAGTTCATCGGCTTCCCCATCAAGCTCTACGTGGAGAAGACGGAGGAGAAGGAGGTGACTGACGACGAGGAGGAGGAGGACG---AGAAGGAGGGCGAGGACGACAAGCCCAAGGTCGAGGAGGTCGAC---GAGGAGGAGGGCGAGAAGAAGAAGAAGACGAAGAAGATCAAGGAGGTGACCCACGACTGGGACCACCTCAACTCGCAGAAGCCCATCTGGATGCGCAAGCCCGAGGACGTGACCCACGAGGAGTACGCCTCGTTCTACAAGTCGCTGACCAACGACTGGGAGGAGCACGCCGCCGTCAAGCACTTCTCGGTGGAGGGCCAGCTGGAGTTCAAGGCCTGCCTGTTCACCCCCAAGCGCGCCCCGTTCGACATGTTCGAGGGCGGCGCCAAGAAGAAACTGAACAACATCAAGCTGTACGTGCGTCGCGTGTTCATCATGGACAACTGCGAGGAGCTCATGCCCGAGTACCTCTCGTTCGTCAAGGGTGTTGTCGACTCGGAGGACCTGCCGCTGAACATCTCGCGTGAGACCCTGCAGCAGAACAAGATCCTGCGTGTGATCAAGAAGAACCTGGTCAAGAAGTGCCTGGAGATGTTCGCCGAGCTGGCCGAGGACAACGAGAAGTACAACAAGTTCTACGAGGCCTTCAGCAAGAACCTG-AAGCTGGGCATCCACGAGGACTCGACCAACCGCACCAAGATCGCCAAGCTGCTGCGTTACCACTCGACCAAGTCTGGCGAGGAGATGACGTCGCTGGACGACTACATCTCGCGCATGCCCGAGAACCAGCCCGGCATCTACTACGTGACGGGCGAGAGCAAGAAGGCCGTCGAGAACTCGCCGTTCATTGAGAAGCTCAAGAAGAAGGGCTACGAGGTGATGTTCATGGTTGAGGCCATCGACGAGTACGCCGTGCAGCAGCTCAAGGAGTACGAGGGCAAGAAGCTCATCTGCGCCACCAAGGAGGGCCTCAAGATGGAGGAGACTGAGGACGAGAAGAAGTCGTTCGAGGAGGCCAAGGCCGCCACGGAGGGCCTTCACGGTCATCGACGCCCCTGGCCACCGTGACTTCATCAAGAACATGATCACGGGCACCTCGCAGGCCGACTGCGCCATCCTGGTGGTGGCCTCCGGCGTGGGCGAGTTCGAGGCTGGTATCTC---CAAGGAGGGCCAGACCCGCGAGCACGCTCTGCTGGCCTTCACCCTGGGCGTGAAGCAGATGATCGTCGCCATCAACAAGATGGACGACTCGTCCGTCATGTACGGCCAGGCCCGTTACGAGGAGATCAAGAACGAGGTGTCCACGTACCTGAAGAAGGTGGGCTACAAGCCGGCCAAGATYCCGTTCGTGCCCATCTCCGGSTGGGAGGGCGACAACATGATCGAGAAGTCGGGCAACATGCCGTGGTACAAGGGCCCGTACCTCCTTGAGGCTCTCGACAACCTGAACCCCCCCAAGCGCCCGGTTGACAAGCCGCTGCGTCTGCCCCTCCAGGACGTGTACAAGATYGGCGGTATCGGCACGGTACCGGTCGGCCGTGTGGAGACCGGTGTCATCAAGCCTGGCATGGTCGCCACGTTCGGCCCCGTGGGTCTGTCGACGGAAGTCAAGTCCGTKGAGATGCACCACGAGTCCCTGCCGGARGCTGTCCCCGGTGACAACGTCGGCTTCAACGTCAAGAACGTGTCGGTCAAGGAGCTGCGTCGTGGCTACGTGGCCTCGGACTCCAAGAACGA---CCCGGCCAAGGGCACCCAGGACTTCACCGCCCAGGTGATCGTGCTGAACCACCCCGGCCAGATCGGCAACGGCTACTCGCCCGTGCTGGACTGCCACACGGCCCACGTTGCCTGCAAGTTCAAAGAGATCACGGAGAAGATGGACCGTCGTTCGGGYAAGGTGCTCGTCCAGAACAAGAAGCCGTTCATTAAGTCGCGCTTCTGCCGTGGTGTGCCCGACCCGAAGATCCGCATCTACGATGTCGGCAACAAGAAGGCCTCGGTGGACGCCTTCCCGTTCGTGGCCCACCTGGTGTCCGACGAGAAGGAGCAGCTGTCGTCGGAGGCCCTCGAGGCCGCCCGTATCGCCGCTAACCGTTACCTGACCAAGTTCTGCGGCAAGGACAACTTCCACATGCGCATCCGCTGCCACCCCTTCCAGGTGCTGCGTATCAACAAGATGCTTTCGTGCGCCGGTGCCGATAGACTGCAGACGGGTATGCGTCACGCCTACGGTAAGCCCTCGGGTGTGGCCGCCCGCGTGGCCATCGGCCAGCCCATTATCTCGGTGCGTTCCAAGGACTCGTTCGGCCCCTCGGTCGTGGAGGCTCTGCGTCGTGCATGCCCCCGAGTCGGAGAAGATCTCTGCTGACAAGCTGCAGGAGGTGTACGAGGGCTTCATTGCCAAGTGTGCTGGCTCGAGCAAGATCGTGTCGATCGAGGACCCGTTCGACCAGGACGACTGGGCTTCGTGGGTGAAGTTCACCGGCAAGGTCGGCGAGGACGTGCAGATCGTGGGCGATGACCTGACGGTGACGAACCCGACCCGTGTGAAGAAGGCCATCGAGCAGAAGGCTTGCAACGCCCTTCTGCTGAAGGTGAACCAGATCGGTAGCATCACGGAGTCTATTGAGGCCGTGACGATGGCGAAGAAGGCTGGCTGGGCCATCATGGCCAGCCACCGCAGCGGTGAGACGGAGGACACGTTCATCGCGGACCTGGCTGTGGGTCTGAGCGCCGGCCAGATGGTAACAACTGGGCCAAGGGACACTACACGGAGGGCGCCGAGCTCATCGACTCGGTGCTCGATGTCGTCCGCAAGGAGGCGGAGAGCTGCGACTGCCTGCAGGGWTTCCAGATCACGCACTCGCTTGGTGGCGGTACCGGTTCCGGTATGGGCACGCTTCTTATCTCCAAGATCCGTGAGGAGTACCCGGACCGTATCATGTGCACGTACTCGGTGTGYCCGTCGCCYAAGGTGTCGGACACGGTCGTGGAGCCCTACAACGCGACGCTGTCCGTGCACCAGCTTGTCGAGAACGCCGATGAGGTCATGTGCCTGGATAACGAGGCCCTGTACGACATTTGCTTCCGCACGCTGAAGCTCACGACCCCCACCTACGGTGACCTGAACCACCTGGTGTGCGCCGCCATGTCCGGCATCACCACGTGCCTGCGTTTCCCCGGCCAGCTGAACTCGGACCTGCGTAAGCTTGCCGTGAACCTGATCCCGTTCCCGCGTCTGCACTTCTTCATGATCGGCTTCGCCCCGCTGACGTCGCGTGGCTCGCAGCAGTACCGTGCCCTGACGGTRCCCGAGCTGACCCAGCAGCAGTTCGATGCTAAGAACATGATGTGCGCCGCTGACCCTCGCCACGGCCGCTATTTAACTGCCGCGTGTATGTTCCGCGGACGTATGAGCACGAAGGAGGTCGATGAGCAGATGCTYAACGTGCAGAACAAGAACTCGTCGTACTTCGTCGAGTGGATCCCCAACAACATCAAGGCTAGCGTGTGTGACATCCCGCCTAAGGGTCTCAAGATGAGCACCACGTTCATCGGYAACTCCACTGCCATCCAGGAGATGTTCAAGCGTGTGTCCGAACAGTTCACGGCTATGTTCCGTCGTAAGGCTTTCTTGCACTGG

Phytophthora_melonis TTTTTTAATTACTATTACCGTTTTTGTTTGTTGGATGTTATTTAGAGTTATTACTCTTTTTGATGAAAAAAAAAATAAAATTCCTGCAACTGTTGTACATGGTGCTACTATTGAAATTATTTGGACTTCTATTCCAGCTTTAATTTTATTAATTGTAGCTATTCCATCTTTTGCTTTATTATATTCAATGGATGAAGTAATTGATCCTATTATTACTTTAAAAGTAATTGGTAGTCAATGGTACTGGAGTTATGAATATTCTGATAATTTAGAATTTTCAGATGAACCTTTAATTTTTGATAGTTATATGGTACAAGAAGATGATTTAGCAATAGGTCAATTTAGACTTCTAGAAGTAGATAATCGTGTTGTAGTTCCAACTAATAGTCATATTAGAGTATTAATTACAGCATCAGATGTTTTACATTCATGGGCTATTCCATCATTAGGTATTAAATTAGATGCTTGTCCAGGTCGTTTAAATCAAACCGGCGAGTGAAGCGGGAAGAGCTCAAGCTTAAAATCTCCGTGCAAGTTTTGCGCGGCGAATTGTAGTCTATAGAGGC-GTGGTCAGCGTGGGCGCTTGGGGCAAGTTCCTTGGAAGAGGACAGCATGGAGGGTGATACTCCCGTTCATC-CCTGAGTGGCTCGTGCGTACGACCCGTGTTCTTTGAGTCGCGTTGTTTGGGAATGCAGCGCAAAGTAGGTGGTAAATTCCATCTAAAGCTAAATATTGGTGCGAGACCGATAGCGAACAAGTACCGTGAGGGAAAGATGAAAAGAACTTTGAAAAGAGAGTTAAAGAGTACCTGAAACTGCTGAAAGGGAACCGAATCGTTTCCAGTGTCTATAATCCGTGGCATATTTCATTGGCGAGTGTGTGCGTGCGTGTGCTGTGGCAGTGGCTTTTTT--GGCTGCGCTCGGTGTG---TG-TGCTGTGTGTGCTTGCTGGTGCCCTGTGCTGCGGTGGGACGTCAAGGTCAGTTCGTATGCTGCGGGAAATGGCTGCCGAGGAGGTAGGGCTTACGCTCCGCGTTTGTCTGTTATATCTTGGTGGACG-AGTCGTCGCGGTTGGGACTGAGGTG-CCTACAACGTGCTTTTGAGTGGGTTTGTGTCTCCGTGTGCGCCGTGTGCGGATAGCTTGCTATGCGTGTGT---GGTTGTGTGTGGATTGATGCGGGCTTTAACTTGTCGCCGTTCACTCGGGTATCGGTATGACCAAGGCCGACCTCATCAACAACCTGGGTACGATTGCCAAGTCGGGCACCAAGGCCTTCATGGAGGCCCTGGCCGCCGGCGCTGACATCAGCATGATCGGCCAGTTCGGTGTGGGTTTCTACTCCGCCTACCTGGTGGCCGACAAGGTCGTGGTGCACTCGAAGCACAACGACGACGAGCAGTACGTGTGGGAGTCCGCCGCCGGCGGCTCGTTCACGGTCACACCCGAC---ACGTCGGAGCCCATCCTGCGCGGTACCCGCATCGTGCTCAAGCTCAAGGAGGACATGCTCGAGTACCTCGAGGAGCGCAAGCTCAAAGACCTGGTGAAGAAGCACTCGGAGTTCATTGGCTTCCCCATCAAGCTCTACGTCGAGAAGACGGAGGAGAAGGAGGTGACTGACGACGAGGAGGAGGAGGACG---AGAAGGAGGGCGAGGACGACAAGCCCAAGGTCGAGGAGGTGGAC---GAGGAGGAGGGCGAGAAGAAGAAGAAGACCAAGAAGATCAAGGAGGTGACCCACGACTGGGACCACCTTAACTCGCAGAAGCCCATCTGGATGCGCAAGCCCGAGGATGTGACCCACGAGGAGTACGCCTCGTTCTACAAGTCGCTGACCAACGACTGGGAGGAGCACGCCGCCGTCAAGCACTTCTCGGTGGAGGGCCAGCTGGAGTTCAAGGCCTGCCTGTTCACCCCCAAGCGCGCCCCGTTCGACATGTTCGAGGGCGGTGCCAAGAAGAAACTCAACAACATCAAGCTCTACGTGCGTCGCGTGTTCATCATGGACAACTGCGAGGAGCTCATGCCCGAGTACCTGTCGTTCGTCAAGGGTGTCGTCGACTCGGAGGACCTGCCGCTGAACATTTCGCGTGAGACCCTGCAGCAGAACAAGATCCTGCGCGTGATCAAGAAGAACCTGATCAAGAAGTGCCTGGAGATGTTCGCGGAGCTCGCCGAGGACAACGAGAAGTACCAGAAGTTCTACGAGGCCTTCAGCAAGAACCTC-AAGCTGGGCATCCACGAGGACTCGACCAACCGCACCAAGATCGCCAAGCTGCTGCGTTACCACTCGACCAAGTCTGGCGAGGAGATGACGTCGCTGGACGACTACATCTCGCGCATGCCCGAGAACCAGCCCGGCATCTACTACGTGACGGGCGAGAGCAAGAAGGCTGTCGAGAACTCGCCGTTCATTGAGAAGCTCAAGAAGAAGGGCTACGAGGTGCTCTTCATGGTGGAGGCCATCGACGAGTACGCCGTGCAGCAGCTCAAGGAGTACGAGGGCAAGAAGCTCATCTGCGCCACCAAGGAGGGCCTCAAGATGGAGGAGACCGAGGACGAGAAGAAGTCGTTCGAGGAGGCCAAGGCCGCCACGGAGGGCCTTCACGGTCATTGACGCCCCTGGCCACCGTGACTTCATCAAGAACATGATCACGGGCACCTCGCAGGCTGACTGCGCCATTCTGGTGGTCGCCTCGGGTGTGGGCGAGTTCGAGGCTGGTATCTC---CAAGGAGGGCCAGACGCGCGAGCACGCTCTGCTTGCCTTCACCCTGGGTGTGAAGCAGATGATCGTCGCCATCAACAAGATGGACGACTCGTCTGTCATGTACGGCCAGGCCCGTTACGAGGAGATCAAGAACGAGGTGTCCACGTACCTGAAGAAGGTCGGCTACAAGCCCGCCAAGATCCCGTTCGTGCCCATCTCCGGCTGGGAGGGTGACAACATGATCGAGAAGTCGGGCAACATGCCGTGGTACAAGGGCCCCTACCTCCTTGAGGCTCTTGACAACCTGAACCCGCCCAAGCGCCCGRTTGACAAGCCYCTCCGTCTGCCCCTCCAGGACGTGTACAAGATCGGCGGTATCGGCACGGTACCGGTCGGCCGTGTGGAGACCGGTGTCATCAAGCCTGGCATGGTCGCCACKTTCGGCCCCGTTGGCCTGTCGACGGAAGTCAAGTCCGTTGAGATGCACCACGAGTCCCTGCCGGAGGCCGTCCCCGGTGACAACGTTGGCTTCAACGTCAAGAACGTGTCGGTGAAGGAGCTGCGTCGTGGCTACGTCGCYTCGGACTCCAAGAACGA---CCCGGCCAAGGGCACCCAGGACTTCACCGCCCAGGTGATCGTGCTGAACCACCCCGGCCAGATCGGCAACGGCTACTCKCCCGTGCTGGACTGCCACACGGCCCACGTTGCCTGCAAGTTCAAAGAGATCACGGAGAAGATGGACCGTCGTTCGGGCAAGGTGCTCGTCCAGAACAAGAAGCCGTTCATTAAGTCGCGCTTCTGCCGTGGTGTGCCCGACCCGAAGATCCGCATCTACGATGTCGGCAACAAGAAGGCCTCGGTCGACGCCTTCCCGTTCGTGGCCCACCTGGTGTCCGACGAGAAGGAGCAGCTGTCCTCGGAGGCCCTCGAGGCTGCCCGTATTGCCGCTAACCGTTACCTGACCAAGTTCTGCGGCAAGGACAACTTCCACATGCGCATCCGCTGCCACCCGTTCCAGGTCCTGCGTATCAACAAGATGCTTTCGTGCGCCGGTGCCGATAGACTGCAGACGGGTATGCGTCACGCCTACGGTAAGCCTTCGGGTGTCGCCGCTCGTGTGGCCATCGGCCAGCCCATTATCTCGGTGCGCTCCAAGGACTCGTTCGGCCCCTCGGTCGTCGAGGCTCTGCGTCGTGCACGCCCCCGAGTCGGAGAAGATCTCTGCCGACAAGCTGCAGGAGGTGTACGAGGGCTTCATCGCCAAGTGCGCTGGCTCGAGCAAGATCGTGTCGATCGAGGACCCGTTCGACCAGGACGACTGGGCTTCGTGGGTGAAGTTCACGGCCAAGGTCGGCGAGGACGTGCAGATCGTGGGCGACGACCTGACGGTGACGAACCCTACGCGTGTGAAGAAGGCCATTGAGCAGAAGGCTTGCAACGCTCTTCTGCTGAAGGTGAACCAGATCGGCAGCATCACGGAGTCGATTGAGGCCGTGACGATGGCGAAGAAGGCTGGCTGGGCCATCATGGCGAGCCACCGCAGCGGTGAGACGGAGGACACGTTCATCGCCGACCTGGCTGTGGGTCTGAGCGCTGGCCAGATGGTAACAACTGGGCCAAGGGACACTACACGGAGGGTGCTGAGCTCATCGACTCGGTGCTTGACGTTGTCCGTAAGGAGGCGGAGAGCTGTGACTGCCTTCAGGGTTTCCAGATCACGCACTCGCTCGGTGGCGGTACTGGTTCCGGTATGGGTACGCTTCTTATCTCCAAGATTCGTGAGGAGTACCCGGACCGTATCATGTGCACGTACTCGGTCTGCCCATCGCCTAAGGTGTCGGACACGGTCGTCGAGCCCTACAACGCTACGCTGTCCGTGCACCAGCTTGTCGAGAACGCCGATGAGGTCATGTGCCTGGATAACGAGGCCCTGTACGACATTTGCTTCCGTACGCTGAAGCTCACGACCCCCACCTACGGTGACCTGAACCACCTGGTGTGTGCCGCCATGTCCGGCATCACCACGTGCCTGCGTTTCCCCGGTCAGCTGAACTCGGACCTGCGTAAGCTTGCCGTGAACCTGATCCCGTTCCCGCGTCTCCACTTCTTCATGATCGGTTTCGCCCCGCTGACGTCGCGTGGCTCGCAGCAGTACCGTGCCCTGACGGTGCCCGAGCTGACCCAGCAGCAGTTCGACGCCAAGAACATGATGTGCGCCGCCGACCCTCGCCACGGCCGCTATTTAACTGCCGCGTGTATGTTCCGCGGACGTATGAGCACGAAGGAGGTTGATGAGCAGATGCTCAACGTGCAGAACAAGAACTCGTCATACTTCGTCGAGTGGATCCCCAACAACATCAAGGCAAGCGTGTGTGACATCCCGCCCAAGGGTCTCAAGATGAGCACTACGTTCATCGGTAACTCGACCGCTATCCAGGAGATGTTCAAGCGCGTGTCCGAACAGTTCACGGCTATGTTCCGTCGTAAGGCTTTCTTGCACTGG

Phytophthora_sojae TTTTTTAATTACCATTACTGTTTTTGTTTGTTGGATGTTATTTAGAGTTATTACTCTTTTTGATGAAAAAAAAAATAAAATTCCTGCAACTGTTATACACGGTGCTACTATTGAAATTATTTGGACTTCTATTCCAGCGTTAATTTTATTAATTGTAGCTATTCCATCTTTTGCTTTATTATACTCAATGGATGAAGTAATTGATCCTATTATTACTTTAAAAGTAATTGGTAGTCAATGGTATTGGAGTTATGAATATTCTGATAATTTAGAATTTTCAGATGAACCTTTAATTTTTGATAGTTATATGGTACAAGAAGATGATTTAGCAATAGGTCAATTTAGACTTTTAGAAGTAGATAATCGTGTTGTAGTTCCAACTAATAGTCATATTAGAGTATTAATTACCGCATCAGATGTTTTACATTCATGGGCTATTCCGTCATTAGGTATTAAATTAGATGCTTGTCCAGGTCGTTTAAATCAAACCGGCGAGTGAAGCGGGAAGAGCTCAAGCTTAAAATCTCCGTGCAAGTTTTGCGCGGCGAATTGTAGTCTATAGAGGC-GTGGTCAGCGTGGGCGCTTGGGGCAAGTTCCTTGGAAGAGGACAGCATGGAGGGTGATACTCCCGTTCATC-CCTGAGTGGCTCGTGCGTACGACCCGTGTTCTTTGAGTCGCGTTGTTTGGGAATGCAGCGCAAAGTAGGTGGTAAATTCCATCTAAAGCTAAATATTGGTGCGAGACCGATAGCGAACAAGTACCGTGAGGGAAAGATGAAAAGAACTTTGAAAAGAGAGTTAAAGAGTACCTGAAACTGCTGAAAGGGAACCGAATCGTTTCCAGTGTCTATAATCCGTGGCATATTTCATTGGCGAGTGTGCGCGTGCGTGTGCTGTGGCAGTGGCTTTTTT--GGCTGCGCTCGGTGTG---TG-TGCTGTGTGTGCTTGCTGGTGCCCTGTGCTGCGGTGGGACGTCAAGGTCAGTTCGTATGCTGCGGGAAATGGCTGCCGAGGAGGTAGGGCTTACGCTCCGCGTTTGTCTGTTATATCTTGGTGGACG-AGTCGTCGCGGTTGGGACTGAGGTG-CCTACAACGTGCTTTTGAGTGGGTTTGTGTCTCCGTGTGCGCCGTGTGCGGATAGCTTGCTATGCGTGTGT---GGTTGTGTGTGGATTGATGCGGGCTTTAACTTGTCGCCGTTCACTCGGGCATCGGTATGACCAAGGCCGACCTCATCAACAACCTGGGTACGATCGCCAAGTCGGGCACCAAGGCCTTCATGGAGGCCCTGGCCGCCGGCGCTGACATCAGCATGATCGGCCAGTTTGGTGTGGGTTTCTACTCCGCCTACCTGGTGGCCGACAAGGTCGTGGTGCACTCCAAGCACAACGACGACGAGCAGTACGTGTGGGAGTCCGCCGCCGGCGGCTCGTTCACGGTCACGCCCGAC---ACGTCGGAGCCCATCCTGCGCGGTACCCGCATCGTGCTCAAGCTCAAGGAGGACATGCTCGAGTACCTCGAGGAGCGCAAGCTCAAAGACCTGGTGAAGAAGCACTCGGAGTTCATTGGCTTCCCCATCAAGCTCTACGTCGAGAAGACGGAGGAGAAGGAGGTGACTGACGACGAGGAGGAGGAGGACG---AGAAGGAGGGCGACGACGACAAGCCCAAGGTCGAGGAGGTCGAC---GAGGAGGAGGGCGAGAAGAAGAAGAAGACCAAGAAGATCAAGGAGGTGACCCATGACTGGGACCACCTGAACTCGCAGAAGCCCATCTGGATGCGCAAGCCCGAGGACGTGACCCACGAGGAGTACGCCTCGTTCTACAAGTCGCTGACCAACGACTGGGAGGAGCACGCCGCCGTCAAGCACTTCTCGGTGGAGGGCCAGCTGGAGTTCAAGGCCTGCCTCTTCACCCCCAAGCGCGCCCCGTTCGACATGTTCGAGGGCGGTGCCAAGAAGAAACTCAACAACATCAAGCTCTACGTGCGTCGCGTGTTCATCATGGACAACTGCGAGGAGCTCATGCCCGAGTACCTGTCGTTCGTCAAGGGTGTTGTCGACTCGGAGGACCTGCCGCTGAACATCTCGCGTGAGACCCTGCAGCAGAACAAGATCCTGCGCGTGATCAAGAAGAACCTGATCAAGAAGTGCCTGGAGATGTTCGCCGAGCTCGCCGAGGACAACGAGAAGTACCAGAAGTTCTACGAGGCTTTCAGCAAGAACCTC-AAGCTGGGCATCCACGAGGACTCGACCAACCGCACTAAGATCGCCAAGCTGCTGCGTTACCACTCGACCAAGTCTGGCGAGGAGATGACGTCGCTGGACGACTACATCTCGCGCATGCCCGAGAACCAGCCCGGCATCTACTACGTCACGGGCGAGAGCAAGAAGGCCGTGGAGAACTCGCCGTTCATTGAGAAGCTCAAGAAGAAGGGCTACGAGGTGCTCTTCATGGTGGAGGCCATCGACGAGTACGCCGTGCAGCAGCTCAAGGAGTACGAGGGCAAGAAGCTCATCTGCGCCACCAAGGAGGGCCTCAAGATGGAGGAGACCGAGGACGAGAAGAAGTCGTTCGAGGAGGCCAAGGCCGCCACGGAGGGCCTTCACGGTCATTGACGCCCCTGGCCACCGTGACTTCATCAAGAACATGATCACGGGCACCTCGCAGGCCGACTGCGCCATCCTGGTGGTCGCCTCGGGTGTGGGCGAGTTCGAGGCTGGTATCTC---CAAGGAGGGCCAGACGCGCGAGCACGCTCTGCTTGCCTTCACCCTGGGTGTGAAGCAGATGATCGTCGCCATCAACAAGATGGACGACTCGTCGGTCATGTACGGCCAGGCCCGTTACGAGGAGATCAAGAACGAGGTGTCCACGTACCTGAAGAAGGTCGGCTACAAGCCCGCCAAGATCCCGTTTGTGCCCATCTCCGGCTGGGAGGGTGACAACATGATCGAGAAGTCGGGCAACATGCCGTGGTACAAGGGMCCCTACCTCCTTGAGGCTCTCGACAACCTGAACCCGCCCAAGCGCCCGCTTGACAAGCCCCTCCGTCTGCCCCTCCAGGACGTGTACAAGATCGGCGGTATYGGCACGGTACCGGTCGGCCGTGTKGAGACCGGTGTCATCAAGCCTGGCATGGTCGCCACGTTCGGCCCCGTTGGCCTGTCGACGGAAGTCAAGTCCGTTGAGATGCACCACGAGTCCCTGCCGGAGGCCGTCCCCGGTGACAACGTTGGCTTYAACGTCAAGAACGTGTCGGTGAAGGAGCTGCGTCGTGGCTACGTCGCCTCGGACTCTAAGAACGA---CCCGGCCAAGGGCACCCAGGACTTCACCGCCCAGGTKATCGTGCTGAACCACCCCGGCCAGATCGGCAACGGCTACTCGCCCGTGCTSGACTGCCACACGGCCCACGTTGCCTGCAAGTTCAAAGAGATCACGGAGAAGATGGACCGTCGYTCGGGCAAGGTGCTCGTCCAGAACAAGAAGCCGTTCATTAAGTCGCGCTTCTGCCGTGGTGTGCCCGACCCGAAGATCCGCATCTACGATGTCGGCAACAAGAAGGCCTCGGTCGACGCCTTCCCGTTCGTGGCCCACCTGGTGTCCGACGAGAAGGAGCAGCTGTCCTCGGAGGCCCTCGAGGCTGCCCGTATTGCCGCTAACCGTTACCTGACCAAGTACTGCGGCAAGGACAACTTCCACATGCGCATCCGTTGCCACCCGTTCCAGGTGCTGCGTATCAACAAGATGCTTTCGTGCGCCGGTGCCGATAGACTGCAGACGGGTATGCGTCACGCCTACGGTAAGCCCTCGGGTGTCGCCGCCCGCGTGGCCATCGGCCAGCCCATTATCTCGGTGCGCTCCAAGGACTCGTTCGGCCCCTCGGTCGTGGAGGCCCTGCGTCGTGCACGCCCCCGAGTCGGAGAAGATCTCTGCCGACAAGCTGCAGGAGGTGTACGAGGGCTTCATCGCCAAGTGCGCGGGCTCGAGCAAGATCGTGTCGATCGAGGACCCGTTCGACCAGGACGACTGGGCTTCGTGGGTGAAGTTCACGGCCAAGGTTGGCGAGGACGTGCAGATCGTGGGCGACGACCTGACGGTGACGAACCCTACGCGTGTGAAGAAGGCCATCGAGCAGAAGGCTTGCAACGCTCTTCTGCTGAAGGTGAACCAGATCGGCAGCATCACGGAGTCGATTGAGGCCGTGACGATGGCGAAGAAGGCTGGCTGGGCCATCATGGCGAGCCACCGCAGCGGTGAGACGGAGGACACGTTCATCGCTGACCTGGCTGTGGGTCTGAGCGCTGGCCAGATGGTAACAACTGGGCCAAGGGACACTACACGGAGGGTGCCGAGCTTATCGACTCGGTTCTCGACGTCGTCCGCAAGGAGGCTGAGAGCTGTGACTGCCTTCAGGGTTTCCAGATCACGCACTCGCTGGGTGGCGGTACCGGTTCCGGTATGGGTACGCTTCTTATCTCCAAGATTCGTGAGGAGTACCCGGACCGTATCATGTGCACGTACTCGGTCTGCCCGTCGCCTAAGGTGTCGGACACGGTCGTCGAGCCCTACAACGCTACGCTGTCCGTCCACCAGCTCGTTGAGAACGCCGATGAGGTCATGTGCCTGGATAACGAGGCCCTGTACGACATTTGCTTCCGTACCCTGAAGCTCACGACCCCCACCTACGGTGACCTGAACCACCTGGTGTGCGCCGCCATGTCCGGCATTACCACGTGCCTGCGTTTCCCCGGTCAGCTGAACTCGGACCTGCGTAAGCTTGCCGTGAACCTGATCCCGTTCCCGCGTCTCCACTTCTTCATGATCGGTTTCGCCCCGCTGACGTCGCGCGGCTCGCAGCAGTACCGTGCCCTGACGGTGCCCGAGCTGACCCAGCAGCAGTTCGATGCTAAGAACATGATGTGTGCCGCCGACCCTCGCCACGGCCGCTATTTAACTGCCGCGTGTATGTTCCGCGGACGTATGAGCACGAAGGAGGTTGACGAGCAGATGCTCAACGTGCAGAACAAGAACTCGTCGTACTTCGTCGAGTGGATCCCCAACAACATCAAGGCTAGCGTGTGTGACATCCCGCCCAAGGGTCTCAAGATGAGCACCACGTTCATCGGTAACTCGACCGCTATCCAGGAGATGTTCAAGCGCGTGTCCGAACAGTTCACGGCTATGTTCCGTCGTAAGGCTTTCTTGCACTGG

Phytophthora_fragariae CTTTTTAATTACTATTACTATTTTCGTTTGTTGGATGTTATTTAGAGTTATTACTCTTTTTGATGAAAAAAAAAATAAAATACCTGCAACCGTTGTACATGGTGCTACTATTGAAATTATTTGGACTTCTATTCCAGCTTTAATTTTATTAATCGTTGCTATTCCTTCTTTTGCATTATTATATTCAATGGATGAAGTTATTGATCCTATTATTACCTTAAAAGTGATTGGTAGTCAATGGTATTGGAGCTATGAGTATTCTGATAATTTAGAATTTTTAGATGAACCTTTAATTTTTGATAGTTATATGGTACAAGAAAATGATTTAGCAATAGGTCAATTTAGACTTTTAGAAGTAGATAATCGTGTAGTAGTTCCTACTAATAGTCATATTAGAGTATTAATTACAGCATCAGATGTTTTACATTCATGGGCTATTCCATCATTAGGTATTAAATTAGATGCCTGTCCTGGACGTTTAAATCAAACCGGCGAGTGAAGCGGGAAGAGCTCAAGCTTAAAATCTCCGTGCAAGTTTTGCGCGGCGAATTGTAGTCTATAGAGGC-GTGGTCAGCGTGGGCGCTTGGGGCAAGTTCCTTGGAAGAGGACAGCATGGAGGGTGATACTCCCGTTCATC-CCTGAGTGGCTCGTGCGTACGACCCGTGTTCTTTGAGTCGCGTTGTTTGGGAATGCAGCGCAAAGTAGGTGGTAAATTCCATCTAAAGCTAAATATTGGTGCGAGACCGATAGCGAACAAGTACCGTGAGGGAAAGATGAAAAGAACTTTGAAAAGAGAGTTAAAGAGTACCTGAAACTGCTGAAAGGGAACCGAATCGTTTCCAGTGTCTATAATCCGTGGCATATTTCATTGGCGAGTGTGCGCGTGCGTGTGCTGTGGCAGCGGCCTTTTT--GGCTGCGCTCGGTGCG---TG-TGCTGTGTGTGCTTGCTGGTGCCCTGTGCTGCGGTGGGACGTCAAGGTCAGTTCGTATGCTGCGGGAAATGGCTGCCGAGGAGGTAGGGCTTACGCTCCGCGTTTGTCTGTTATATCTTGGTGGACG-AGTCGTCGCGGTTGGGACTGAGGTG-CCTACAACGTGCTTTTGAGTGGGTCTGTGTCTCCGTGTGCGCCGTGTGCGGATAGCTTGCTATGCGTGTGT---GGTTGTGTGTGGATTGATGCGGGCCTTAACTTGTCGCCGTTCACTCGGGTATCGGCATGACCAAGGCCGACCTCATCAACAACCTGGGCACGATCGCCAAGTCGGGCACCAAGGCCTTCATGGAGGCCCTGGCCGCCGGCGCTGACATCAGCATGATCGGCCAGTTCGGTGTGGGCTTCTACTCGGCCTACCTGGTGGCTGACAAGGTGGTGGTGCACTCGAAGCACAACGACGACGAGCAGTACGTGTGGGAGTCCGCCGCCGGCGGCTCGTTCACCGTCACGCCCGAC---ACGTCGGAGCCCATCCTGCGCGGCACGCGCATCGTGCTGACGCTCAAGGAGGACATGCTCGAGTACCTGGAGGAGCGCAAGCTGAAAGACCTGGTGAAGAAGCACTCGGAGTTCATCGGCTTCCCCATCAAGCTCTACGTGGAGAAGACGGAGGAGAAGGAGGTGACGGACGACGAGGAGGAGGAGGACG---AGAAGGAGGGCGAGGACGACAAGCCCAAGGTCGAGGAGGTCGAC---GAGGAGGAGGGAGAGAAGAAGAAGAAGACCAAGAAGATCAAGGAGGTGACCCACGACTGGGACCACCTCAACTCGCAGAAGCCCATCTGGATGCGCAAGCCGGAGGACGTGACCCACGAGGAGTACGCGTCGTTCTACAAGTCGCTGACCAACGACTGGGAGGAGCACGCCGGCGTCAAGCACTTCTCGGTGGAGGGCCAGCTGGAGTTCAAGGCCTGTCTGTTCGCCCCCAAGCGCGCGCCGTTCGACATGTTCGAGGGCGGTGCCAAGAAGAAACTGAACAACATCAAGCTGTACGTGCGTCGCGTGTTCATCATGGACAACTGCGAGGAGCTCATGCCCGAGTACCTGTCGTTCGTCAAGGGTGTCGTCGACTCGGAGGACCTGCCGCTGAACATCTCGCGTGAGACCCTGCAGCAGAACAAGATCCTGCGCGTGATCAAGAAGAACCTGGTGAAGAAGTGCCTTGAGATGTTCGCCGAGCTGGCCGAGGACAACGAGAAGTACAGCAAGTTCTACGAGGCCTTCAGCAAGAACCTG-AAGCTGGGCATCCACGAGGACTCGACCAACCGCACCAAGATCGCCAAGCTGCTGCGTTACCACTCGACCAAGTCTGGCGAGGAGCAGACGTCGCTGGACGACTACATCTCGCGCATGCCCGAGAACCAGCCCGGCATCTACTACGTGACGGGCGAGAGCAAGAAGGCCGTGGAGAACTCGCCCTTCATTGAGAAGCTCAAGAAGAAGGGCTACGAGGTCATCTTCATGGTGGAGGCCATCGACGAGTACGCCGTGCAGCAGCTGAAGGAGTACGAGGGCAAGAAGCTCATCTGCGCCACCAAGGAGGGCCTCAAGATGGAGGAGACCGAGGACGAGAAGAAGTCGTTCGAGGAGGCCAAGGCCGCCACGGAGGGCCTTCACGGTCATCGACGCCCCTGGCCACCGTGACTTCATCAAGAACATGATCACGGGCACCTCGCAGGCCGACTGCGCCATCCTGGTGGTGGCCTCGGGTGTGGGCGAGTTCGAGGCTGGTATCTC---CAAGGAGGGCCAGACGCGCGAGCACGCTCTGCTTGCCTTCACCCTGGGTGTGAAGCAGATGGTCGTCGCCATCAACAAGATGGACGACTCGTCTGTCATGTACGGCCAGGCCCGTTACGAGGAGATCAAGTCCGAGGTGTCGACCTACCTGAAGAAGGTCGGCTACAAGCCCGCCAAGATCCCGTTCGTGCCCATCTCCGGCTGGGAGGGTGACAACATGATCGAGAAGTCCGGCAACATGCCGTGGTACAAGGGACTGTACCTCCTCGAGGCTCTCGACAACCTGAACCCGCCCAAGCGCCCGWCGGACAAGCCTCTGCGTCTGCCCCTCCAGGATGTTTACAAGATCGGCGGTATCGGCACGGTACCGGTCGGCCGTGTGGAGACCGGTGTCATCAAGCCTGGCATGGTCGCCACGTTCGGCCCCGTTGGTCTGTCGACGGAAGTCAAGTCCGTTGAGATGCACCACGAGTCTCTGCCSGAGGCTGTCCCTGGTGACAACGTCGGCTTCAACGTCAAGAACGTSTCGGTCAAGGAGCTGCGTCGTGGCTACGTCGCCTCGGACTCCAAGAACGA---CCCGGCCAAGGCCACCCAGGACTTCCTGGCCCAGGTCATTGTGCTGAACCACCCCGGCCAGATCGGCAACGGCTACTCGCCGGTCCTCGACTGCCACACGGCCCACGTTGCCTGCAAGTTCAAAGAGATCACGGAGAAGATGGACCGTCGTTCGGGCAAGGTGCTCGTCCAGAACAAGAAGCCGTTCATTAAGTCGCGCTTCTGCCGTGGTGTGCCCGACCCGAAGATCCGCATCTACGATGTCGGCAACAAGAAGGCGTCGGTCGACGCCTTCCCGTTCGTGGCCCACCTGGTGTCCGACGAGAAGGAGCAGCTGTCCTCGGAGGCCCTCGAGGCCGCCCGTATTGCTGCCAACCGTTACCTGACTAAGTACTGCGGCAAGGACAACTTCCACATGCGCATCCGTTGCCACCCGTTCCAGGTGCTGCGTATCAACAAGATGCTTTCGTGCGCCGGTGCCGATAGACTACAGACGGGTATGCGTCACGCCTACGGTAAGCCCGCCGGTGTGGCCGCCCGTGTGGCCATCGGCCAGCCCATTATCTCGGTGCGCTCCAAGGACTCGTTCGGCCCCTCGGTCGTGGAGGCTCTGCGTCGTGCACGCTGCCGAGTCGGAGAAGATCTCGGCCGACAAGCTGCAGGAGGTGTACGAGGGCTTCATCGCCAAGTGCGCGGACTCGAGCAAGATCGTGTCGATCGAGGACCCGTTCGACCAGGACGACTGGGCGTCGTGGGTGAAGATCACGAGCAAGGTGGGCAAGGACGTGCAGATCGTGGGCGACGACCTGACGGTGACGAACCCGACCCGTGTGAAGAAGGCCATTGAGCTGAAGGCGTGCAACGCCCTTCTGCTGAAGGTGAACCAGATCGGTAGCATCACGGAGTCGATTGAGGCCGTGACGATGGCGAAGAAGGCGGGCTGGGCCATCATGGCCAGCCACCGCAGCGGTGAGACGGAGGACACGTTCATCGCGGGCCTGGCTGTGGGTCTGAGCGCCGGCCAGATGGTAACAACTGGGCCAAGGGACACTACACGGAGGGTGCCGAGCTCATCGACTCGGTGCTGGACGTCGTCCGTAAGGAGGCGGAGAGCTGTGACTGCCTGCAGGGTTTCCAGATCACGCACTCGCTTGGTGGCGGTACCGGTTCCGGTATGGGTACGCTTCTTATCTCCAAGATTCGTGAGGAGTACCCGGACCGTATCATGTGCACGTACTCGGTCTGCCCGTCGCCCAAGGTGTCGGACACGGTCGTCGAGCCCTACAACGCCACGCTGTCCGTGCACCAGCTTGTCGAGAACGCCGATGAGGTCATGTGCCTGGATAACGAGGCCCTGTACGACATTTGCTTCCGTACGCTCAAGCTCACGACCCCCACGTACGGTGACCTGAACCACCTGGTGTGCGCTGCCATGTCCGGTATCACCACGTGCCTCCGTTTCCCCGGCCAGCTGAACTCGGACCTGCGTAAGCTTGCCGTGAACCTGATCCCGTTCCCGCGTCTCCACTTCTTCATGATCGGTTTCGCCCCGCTGACGTCGCGTGGCTCGCAGCAGTACCGCGCCCTGACGGTGCCCGAGCTGACGCAGCAGCAGTTCGATGCCAAGAACATGATGTGTGCCGCCGACCCTCGCCACGGCCGCTATTTAACTGCCGCGTGTATGTTCCGCGGACGTATGAGCACGAAGGAGGTTGATGAGCAGATGCTCAACGTGCAGAACAAGAACTCGTCGTACTTCGTCGAGTGGATCCCCAACAACATCAAGGCTAGCGTGTGTGACATCCCGCCCAAGGGACTGAAGATGAGCACCACGTTCATCGGTAACTCGACCGCTATCCAGGAGATGTTCAAGCGCGTGTCCGAACAGTTCACGGCTATGTTCCGTCGTAAGGCTTTCTTGCACTGG

Phytophthora_rubi CTTTTTAATTACTATTACTCTTTTCGTTTGTTGGATGTTATTTAGAGTTATTACTCTTTTTGATGAAAAAAAAAATAAAATACCTGCAACCATTGTACATGGTGCTACTATTGAAATTATTTGGACTTCTATTCCAGCTTTAATTTTATTAATTGTTGCTATTCCTTCTTTTGCATTATTATATTCAATGGATGAAATTATTGATCCTATTATTACATTAAAAGTGATTGGTAGTCAATGGTATTGGAGCTATGAGTATTCTGATAATTTAAAATTTTCAGATGAACCTTTAATTTTTGATAGTTATATGGTACAAGAAGATGATTTAGCAATAGGTCAATTTAGACTTTTAGAAGTAGATAATCGTGTAGTAGTTCCTACTAATAGTCATATTAGAGTATTAATTACAGCATCAGATGTTTTACATTCATGGGCGATTCCATCATTAGGTATTAAATTAGATGCCTGTCCTGGACGTTTAAATCAAACCGGCGAGTGAAGCGGGAAGAGCTCAAGCTTAAAATCTCCGTGCAAGTTTTGCGCGGCGAATTGTAGTCTATAGAGGC-GTGGTCAGCGTGGGCGCTTGGGGCAAGTTCCTTGGAAGAGGACAGCATGGAGGGTGATACTCCCGTTCATC-CCTGAGTGGCTCGTGCGTACGACCCGTGTTCTTTGAGTCGCGTTGTTTGGGAATGCAGCGCAAAGTAGGTGGTAAATTCCATCTAAAGCTAAATATTGGTGCGAGACCGATAGCGAACAAGTACCGTGAGGGAAAGATGAAAAGAACTTTGAAAAGAGAGTTAAAGAGTACCTGAAACTGCTGAAAGGGAACCGAATCGTTTCCAGTGTCTATAATCCGTGGCATATTTCATTGGCGAGTGTGCGCGTGCGTGTGCTGTGGCAGCGGCCTTTTT--GGCTGCGCTCGGTGCG---TG-TGCTGTGTGTGCTTGCTGGTGCCCTGTGCTGCGGTGGGACGTCAAGGTCAGTTCGTATGCTGCGGGAAATGGCTGCCGAGGAGGTAGGGCTTACGCTCCGCGTTTGTCTGTTATATCTTGGTGGACG-AGTCGTCGCGGTTGGGACTGAGGTG-CCTACAACGTGCTTTTGAGTGGGTCTGTGTCTCCGTGTGCGCCGTGTGCGGATAGCTTGCTATGCGTGTGT---GGTTGTGTGTGGATTGATGCGGGCCTTAACTTGTCGCCGTTCACTCGGGTATCGGTATGACCAAGGCCGACCTCATCAACAACCTGGGCACGATCGCCAAGTCGGGCACCAAGGCCTTCATGGAGGCCCTGGCCGCCGGCGCTGACATCAGCATGATCGGCCAGTTCGGTGTGGGCTTCTACTCGGCCTACCTGGTGGCCGACAAGGTGGTGGTGCACTCGAAGCACAACGACGACGAGCAGTACGTGTGGGAGTCCGCCGCCGGCGGCTCGTTCACCGTCACGCCCGAC---ACGTCGGAGCCCATCCTGCGCGGCACGCGCATCGTGCTGACGCTCAAGGAGGACATGCTGGAGTACCTGGAGGAGCGCAAGCTGAAAGACCTGGTGAAGAAGCACTCGGAGTTCATCGGCTTCCCCATCAAGCTCTACGTGGAGAAGACGGAGGAGAAGGAGGTGACGGACGACGAGGAGGACGAGGACG---AGAAGGAGGGCGAGGACGACAAGCCCAAGGTCGAGGAGGTCGAC---GAGGAGGAGGGAG---AGAAGAAGAAGACCAAGAAGATCAAGGAGGTGACCCACGACTGGGACCACCTCAACTCGCAGAAGCCCATCTGGATGCGCAAGCCCGAGGACGTGACCCACGAGGAGTACGCGTCGTTCTACAAGTCGCTGACCAACGACTGGGAGGAGCACGCCGGTGTCAAGCACTTCTCGGTGGAGGGCCAGCTGGAGTTCAAGGCCTGTCTGTTCGCCCCCAAGCGCGCCCCGTTCGACATGTTCGAGGGCGGCGCCAAGAAGAAACTGAACAACATCAAGCTGTACGTGCGTCGCGTGTTCATCATGGACAACTGCGAGGAGCTCATGCCCGAGTACCTGTCGTTCGTCAAGGGTGTTGTCGACTCGGAGGACCTGCCGCTGAACATCTCGCGTGAGACCCTGCAGCAGAACAAGATCCTGCGCGTGATCAAGAAGAACCTGGTGAAGAAGTGCCTCGAGATGTTCGCCGAGCTGGCCGAGGACAACGAGAAGTACAGCAAGTTCTACGAGGCCTTCAGCAAGAACCTG-AAGCTCGGGATCCACGAGGACTCGACCAACCGCACCAAGATCGCCAAGCTGCTGCGTTACCACTCGACCAAGTCTGGCGAGGAGCAGACGTCGCTTGACGACTACATCTCGCGCATGCCCGAGAACCAGCCCGGCATCTACTACGTGACGGGCGAGAGCAAGAAGGCCGTGGAGAACTCGCCCTTCATTGAGAAGCTCAAGAAGAAGGGCTATGAGGTCATCTTCATGGTGGAGGCCATCGACGAGTACGCCGTGCAGCAGCTGAAGGAGTACGAGGGCAAGAAGCTCATCTCCGCCACCAAGGAGGGCCTCAAGATGGTGGAGACCGAGGACGAGAAGAAGTCGTTCGAGGAGGCCAAGGCCGCCACGGAGGGCCTTCACGGTCATCGACGCCCCTGGCCACCGTGACTTCATCAAGAACATGATCACGGGCACCTCGCAGGCCGACTGCGCCATCCTGGTGGTAGCCTCGGGTGTGGGCGAGTTCGAGGCTGGTATCTC---CAAGGAAGGCCAGACGCGCGAGCACGCTCTGCTTGCCTTCACCCTGGGTGTGAAGCAGATGATCGTCGCCATCAACAAGATGGACGACTCGTCTGTCATGTACGGCCAGGCCCGTTACGAGGAGATCAAGTCCGAGGTGTCGACCTACCTGAAGAAGGTCGGCTACAAGCCCGCCAAGATCCCGTTCGTGCCCATCTCCGGATGGGAGGGTGACAACATGATCGAGAAGTCCGGCAACATGCCGTGGTACAAGGGACCGTACCTCCTAGAGGCTCTCGACAACCTGAACCCGCCCAAGCGCCCGTCGGACAAGCCTCTGCGTCTGCCCCTCCAGGATGTTTACAAGATCGGCGGTATCGGCACGGTACCGGTCGGCCGTGTGGAGACCGGTGTCATCAAGCCTGGCATGGTCGCCACGTTCGGCCCCGTAGGTCTGTCGACGGAAGTCAAGTCCGTTGAGATGCACCACGAGTCTCTGCAAGAGGCTGTCCCTGGTGACAACGTCGGCTTCAACGTCAAGAACGTGTCGGTCAAGGAGCTGCGTCGTGGCTACGTCACCTCGGACTCCAAGAACGA---CCCGGCCAAGGCCACCCAGGACTTCCTGGCCCAGGTCATAGTGCTGAACCACCCCGGCCAGATCGGCAACGGCTACTCGCCGGTGCTCGACTGCCACACGGCCCACGTTGCCTGCAAGTTCAAAGAGATCACGGAGAAGATGGACCGTCGTTCGGGCAAGGTGCTCGTCCAGAACAAGAAGCCGTTCATTAAGTCGCGCTTCTGCCGTGGTGTGCCCGACCCGAAGATCCGCATCTACGATGTCGGCAACAAGAAGGCGTCGGTCGACGCCTTCCCGTTCGTGGCCCACCTGGTGTCCGACGAGAAGGAGCAGCTGTCCTCGGAGGCCCTCGAGGCCGCCCGTATTGCTGCTAACCGTTACCTGACCAAGTACTGCGGCAAGGACAACTTCCACATGCGCATCCGTTGCCACCCGTTCCAGGTGCTGCGTATCAACAAGATGCTTTCGTGCGCCGGTGCCGATAGACTGCAAACGGGTATGCGTCACGCCTACGGTAAGCCCGCCGGTGTGGCCGCCCGTGTGGCCATCGGCCAGCCCATTATCTCGGTGCGCTCCAAGGACTCGTTCGGCCCCTCGGTCGTGGAGGCTCTGCGTCGTGCACGCTGCCGAGTCGGAGAAGATCTCGGCCGACAAGCTGCAGGAGGTGTACGAGGGCTTCATCGCCAAGTGCGCGGACTCGAGCAAGATCGTGTCGATCGAGGACCCGTTCGACCAGGACGACTGGGCGTCGTGGGTGAAGATCACGAGCAAGGTCGGCAAGGACGTGCAGATCGTGGGCGACGACCTGACGGTGACGAACCCGACCCGTGTGAAGAAGGCCATTGAGCTGAAGGCGTGCAACGCCCTTCTGCTGAAGGTGAACCAGATCGGCAGCATCACGGAGTCGATTGAGGCCGTGACGATGGCGAAGAAGGCGGGCTGGGCCATCATGGCCAGCCACCGCAGCGGTGAGACGGAGGACACGTTCATCGCGGACCTGGCTGTGGGTCTGAGCGCCGGCCAGATGGTAACAACTGGGCCAAGGGACACTACACGGAGGGTGCCGAGCTCATCGACTCGGTGCTGGACGTCGTCCGTAAGGAGGCGGAGAGCTGTGACTGCCTGCAGGGTTTCCAGATCACGCACTCGCTTGGTGGCGGTACCGGTTCCGGTATGGGTACGCTTCTTATCTCCAAGATTCGTGAGGAGTACCCGGACCGTATCATGTGCACGTACTCGGTCTGCCCGTCGCCCAAGGTGTCGGACACGGTCGTCGAGCCCTACAACGCCACGCTGTCCGTGCACCAGCTTGTCGAGAACGCCGATGAGGTCATGTGCCTGGATAACGAGGCCCTGTACGACATTTGCTTCCGTACGCTCAAGCTCACGACCCCCACGTACGGTGACCTGAACCACCTGGTGTGCGCTGCCATGTCCGGTATCACCACGTGCCTCCGTTTCCCCGGCCAGCTGAACTCGGACCTGCGTAAGCTTGCCGTGAACCTGATCCCGTTCCCGCGTCTCCACTTCTTCATGATCGGTTTCGCCCCGCTGACGTCGCGTGGCTCGCAGCAGTACCGCGCCCTGACGGTGCCCGAGCTGACGCAGCAGCAGTTCGATGCCAAGAACATGATGTGCGCCGCCGACCCTCGCCACGGCCGCTATTTAACTGCCGCGTGTATGTTCCGCGGACGTATGAGCACGAAGGAGGTTGATGAGCAGATGCTCAACGTGCAGAACAAGAACTCGTCGTACTTCGTCGAGTGGATCCCCAACAACATCAAGGCTAGCGTGTGTGACATCCCGCCCAAGGGACTGAAGATGAGCACCACGTTCATCGGTAACTCGACCGCTATCCAGGAGATGTTCAAGCGCGTGTCCGAACAGTTCACGGCTATGTTCCGTCGTAAGGCTTTCTTGCACTGG

Phytophthora_megakarya TTTTTTAATTATGATTACTGTATTTGTTTGTTGGATGTTATTTAGAGTTATTACTCTTTTTGATGAAAAAAAAAATAAAATTCCTTCAACTGTTGTACATGGAGCTACTATTGAAATTATTTGGACTTCAATTCCTGCATTAATTCTATTAACTGTTGCAATTCCATCTTTTGCATTATTATATTCAATGGATGAAGTAATTGATCCAATTATAACTTTAAAAGTAATAGGTAGTCAATGGTATTGGAGTTATGAATATTCAGATAATTTAGAATTTTCAGATGAGCCTTTAATTTTTGATAGTTATATGGTACAGGAGGATGATTTAGCAATTGGTCAATTTAGACTTTTAGAAGTAGATAATCGTGTAGTAGTTCCTACTAATAGTCATATTAGAGTATTAATTACAGCTTCAGATGTTTTACATTCTTGGGCTATTCCTTCATTAGGTATTAAATTAGATGCATGTCCAGGCCGTTTAAATCAAACCGGCGAGTGAAGCGGGAAGAGCTCAAGCTTAAAATCTCCGTGTAAGTTTTGCACGGCGAATTGTAGTCTATAGAGGC-GTGGTCAGCGTGGGCGCTTGGGGCAAGTTCCTTGGAAGAGGACAGCATGGAGGGTGATACTCCCGTTCATC-CCTGAGTTGCTCGTGCGTACGGCCCGTTTTCTTTGAGTCGCGTTGTTTGGGAATGCAGCGCAAAGTAGGTGGTAAATTCCATCTAAAGCTAAATATTGGTGCGAGACCGATAGCGAACAAGTACCGTGAGGGAAAGATGAAAAGAACTTTGAAAAGAGAGTTAAAGAGTACCTGAAACTGCTGAAAGGGAACCGAATCGTTTCCAGTGTCTATAATCTGTGGCATATTTCATTGGCGAGTGTGTGCGTGCGTGTACTGTGGCAGTGGCTTTTTT--GGCTGCGCTCGGTGTG---TG-TGCTGTGTGTGCTTGCTGGTGCCCTGTGCTGCGGTGGGACGTCAAGGTCAGTTCGTATGCTGCGGGAAATGGCTGCCGAGGAGGTAGGGCTTACGCT-TGCGTTTGTCTGTTATATCTTGGTGGACG-AGTAGTCGCGGTTGGGACTGAGGTG-CCTACAACGTGCTTTTGAGTGTGTTTGTGTCTCCGTGTGCGCCGTGTGCGGATAGCTTGCTATGCGTGTGT---GGTTGTGTGTGGATTGATGCGGACTTTAACTTGTTGCCGTTCACTCTGGTATCGGCATGACCAAGGCCGACCTCATCAACAACCTGGGAACGATCGCCAAGTCGGGCACCAAGGCCTTTATGGAGGCGCTGGCGGCCGGCGCCGACATCAGCATGATCGGCCAGTTTGGTGTGGGCTTCTACTCGGCCTACCTGGTTGCCGACAAGGTCGTGGTGCACTCTAAGCACAACGATGACGAGCAGTACGTGTGGGAGTCGGCTGCTGGCGGCTCGTTCACCGTCACGCCTGAC---ACGTCGGAGCCAATTCTGCGTGGTACGCGCATCGTGCTGAAGCTCAAGGAGGACATGCTCGAGTACCTCGAGGAACGCAAGCTCAAGGAYCTCGTGAAGAAGCACTCGGAGTTCATTGGCTTCCCCATCAAGCTCTACGTCGAGAAGACGGAGGAGAAGGAAGTCACCGACGACGAGGAGGAGGAAGACG---AGAAGGAGGAC---GACGACAAGCCCAAGGTCGAGGAGGTCGAA---GAAGAGGAGGGCGAGAAGAAGAAGAAGACGAAGAAAATCAAGGAGGTGACCCACGAGTGGGACCACCTCAACAGCCAGAAGCCTATCTGGATGCGCAAACCCGAGGACGTGACCCACGAGGAGTACGCSTCYTTCTACAAGTCGCTCACCAACGACTGGGAGGAGCACGCTGCTGTCAAGCATTTCTCAGTGGAGGGCCAGCTTGAGTTCAAGGCCTGCCTCTTCACCCCTAAGCGCGCTCCGTTCGACATGTTCGAAGGCGGTGCGAAGAAGAAACTTAATAACATCAAGCTGTATGTGCGYCGCGTGTTCATTATGGACAACTGCGAGGAGCTCATGCCCGAGTACCTGTCGTTCGTCAAGGGTGTCGTTGACTCGGAGGACCTGCCGCTGAACATCTCACGTGAGACCCTGCAGCAGAACAAGATCCTGCGTGTGATCAAGAAGAACCTCGTCAAAAAGTGTCTCGAGATGTTCGCGGAACTCGCCGAGGACAACGAGAAGTACAACAAGTTCTACGAGGCCTTCAGCAAGAACCTM-AAGCTCGGCATCCACGAGGACTCGACCAACCGCACGAAAATTGCCAAGCTTCTGCGTTACCACTCGACCAAGTCTGGCGAAGAGATGACGTCGCTGGACGACTACATCTCGCGCATGCCCGAGAATCAACCCGGCATCTACTACGTGACGGGCGAGAGCAAGAAGTCGGTGGAGAACTCGCCATTCATTGAGAAGCTCAAGAAGAAGGGCTACGARGTGATGTTCATGGTTGAGGCTATTGACGAGTAYGCTGTGCAGCAGCTGAAGGAGTACGAGGGCAAGAAGCTCATCTGTGCCACAAAGGAGGGCCTCAAGATGGAAGAGACAGAGGACGAGAAGAAGTCGTTCGAGGAGGCTAAGGCAGCTACGGAGGGCCTTCACGGTCATTGACGCCCCTGGCCACCGTGACTTCATCAAGAACATGATCACGGGCACGTCGCAGGCCGATTGCGCCATCCTTGTGGTGGCTTCGGGTGTGGGTGAGTTCGAGGCTGGTATCTC---CAAGGAGGGCCAGACCCGCGAGCACGCTCTGCTTGCCTTCACTCTGGGTGTGAAGCAGATGATCGTCGCCATCAACAAGATGGACGACTCGTCTGTCATGTACGGCCAGGCCCGTTACGAGGAGATCAAGTCGGAGGTCACCACGTACCTGAAGAAGGTCGGCTACAAGCCCGCCAAGATCCCGTTCGTGCCTATCTCGGGCTGGGAGGGAGACAACATGATCGACCGCTCCASCAACATGCCGTGGTACAAGGGACCTTACCTCCTYGAGGCCCTCGACAACCTGAACGCCCCCAAGCGCCCGTCGGACAAGCCGCTCCGTCTGCCCCTTCAGGATGTTTACAAGATCGGCGGTATCGGCACGGTACCGGTCGGCCGTGTGGAGACCGGTGTCATCAAGCCTGGCATGGTCGCCACTTTCGGCCCCGTGGGTCTGTCGACGGAAGTCAAGTCCGTTGAGATGCACCACGAGTCTCTGCCGGAGGCTGTCCCTGGTGACAACGTCGGCTTCAACGTCAAGAACGTGTCGGTGAAGGAGCTGCGTCGTGGTTTCGTCGCTTCGGACTCGAAGAACGA---CCCCGCCAAGGGCACCCAGGACTTCACCGCCCAGGTGATTGTGCTGAACCACCCTGGCCAGATCGGCAACGGCTACTCGCCGGTGCTTGACTGCCACACGGCCCACGTTGCCTGCAAGTTCAAAGAGATCACGGAGAAGATGGACCGTCGTTCGGGCAAGGTGCTCGTCCAGAACAAGAAGCCCTTCATTAAGTCGCGCTTCTGCCGTGGTGTGCCCGACCCGAAGATCCGCATCTACGACGTGGGTAACAAGAAGGCTTCGGTCGACGCCTTCCCCTTCGTGGCCCACTTGGTGTCTGACGAGAAGGAGCAGCTGTCGTCGGAGGCTCTGGAAGCCGCCCGTGTGGCTGCTAACCGTTACTTGACCAAGTACTGCGGCAAGGACAACTTCCACATGCGCATCCGTTGCCACCCGTTCCAGGTGCTGCGTATCAACAAGATGCTTTCGTGYGCCGGTGCCGATAGACTGCAGACGGGTATGCGTCACGCCTACGGTAAGCCYGCYGGTGTGGCCGCCCGTGTGGCCATCGGCCAGCCCATTATCTCGGTGCGCTCCAAGGACTCGTTCGGCCCTTCGGTCGTGGAGGCTCTGCGCCGTGCACGCTCCGGAGTCGGARAAGATCTCCGCCGACAAGCTGCAGGAGGTGTACGAGAGCTTCATCGCCAAGTGCGCGAACACRAGCAAGATCGTGTCGATTGAGGACCCGTTCGACCAGGACGACTGGGCGTCGTGGATCAAGTTCACTGGCAAGGTGGGCAAGGACGTGCAGATCGTGGGCGATGACCTGACGGTGACGAACCCCACCCGTGTGAAGAAGGCCATCGATGAGAAGGCCTGCAACGCTCTGTTGCTGAAGGTGAACCAGATTGGCAGCATCACGGAGTCTATCGAGGCCGTGACGATGGCGAAGAAGGCTGGTTGGGCCATCATGGCCAGCCACCGCAGCGGTGAGACGGAGGACACGTTCATCGCGGACCTGGCTGTGGGTCTGAGCGCTGGTCAGATGGTAACAACTGGGCCAAGGGACACTACACGGAGGGTGCGGAGCTCATCGACTCGGTGCTGGACGTCGTCCGTAAGGAGGCAGAGAGCTGTGACTGTCTGCAGGGTTTCCAGATCACACACTCGCTCGGTGGTGGCACTGGTTCTGGTATGGGAACGCTCCTTATCTCGAAGATTCGTGAGGAGTACCCCGATCGTATCATGTGCACGTACTCGGTCTGCCCGTCGCCCAAGGTGTCGGATACCGTCGTGGAGCCGTACAACGCGACGCTGTCAGTGCACCAGCTGGTCGAAAACGCCGATGAGGTCATGTGCCTGGATAACGAGGCCCTATACGACATTTGCTTCCGTACACTGAAGCTCACGACTCCCACGTACGGTGACTTGAACCACTTGGTGTGCGCTGCCATGTCTGGTATCACCACGTGCCTCCGTTTCCCGGGTCAGTTGAACTCGGACCTGCGTAAGCTTGCCGTGAACCTGATCCCGTTCCCGCGTCTCCACTTCTTTATGATTGGTTTCGCCCCGCTGACTTCGCGTGGCTCGCAGCAGTACCGTGCCTTGACGGTCCCTGAGCTCACCCAGCAGCAGTTCGACGCTAAGAACATGATGTGCGCTGCCGACCCTCGTCACGGCCGCTATTTAACTGCCGCGTGTATGTTCCGCGGACGTATGAGTACGAAGGAGGTTGACGAGCAGATGCTCAACGTGCAGAACAAGAACTCGTCGTACTTCGTCGAGTGGATCCCCAACAACATCAAGGCTAGCGTGTGTGATATCCCGCCCAAGGGTCTGAAGATGAGCACCACGTTCATCGGTAACTCGACTGCCATTCAGGAGATGTTCAAGCGTGTGTCCGAACAGTTCACTGCTATGTTCCGTCGTAAGGCTTTCTTGCACTGG

Phytophthora_palmivora TTTTTTAATTATGATTACTGTATTTGTTTGTTGGATGTTATTTAGAGTTATTACTCTTTTTGATGAAAAAAACAATAAAATCCCATCAACTATTGTACATGGTGCTACTATTGAAATTATTTGGACTTCTATTCCAGCTTTAATTCTATTAACTGTTGCAATTCCATCTTTTGCTTTATTATATTCTATGGATGAAGTAATTGATCCAATTATAACTTTAAAAGTTATAGGTAGTCAATGGTATTGGAGTTATGAATATTCAGATAATTTAGAATTTTCAGATGAACCTTTAATTTTTGATAGTTACATGGTACAAGAGGATGATTTAGCAATTGGTCAATTTAGAATTTTAGAAGTAGATAATCGTGTAGTAGTTCCTACTAATAGTCATATTAGAGTATTAATTACCGCATCAGATGTTTTACATTCATGGGCTATACCTTCATTAGGTATTAAATTAGATGCATGTCCGGGACGTTTAAATCAAACCGGCGAGTGAAGCGGGAAGAGCTCAAGCTTAAAATCTCCGTGCAAGTTTTGCGCGGCGAATTGTAGTCTATAGAGGC-GTGGTCAGCGTGAGCACTTGGGGCAAGTTCCTTGGAAGAGGACAGCATGGAGGGTGATACTCCCGTTCATC-CCTGAGTTGCTCGTGCGTACGACCCGTTTTCTTTGAGTCGCGTTGTTTGGGAATGCAGCGCAAAGTAGGTGGTAAATTCCATCTAAAGCTAAATATTGGTGCGAGACCGATAGCGAACAAGTACCGTGAGGGAAAGATGAAAAGAACTTTGAAAAGAGAGTTAAAGAGTACCTGAAACTGCTGAAAGGGAACCGAATCGTTTCCAGTGTCTATAATCTGTGGCATATTTCATTGGCGAGTGTGTGCGTGCGTGTGCTGTGGCAGTGGCTTTTTT--GGCTGCGCTCGGTGTG---TG-TGCTGTGTGTGCTTGCTGGTGCCCTGTGCTGCGGTGGGACGTCAAGGTCAGTTCGTATGCTGCGGGAAATGGCTGCCGAGGAGGTAGGGCTTACGCT-TGCGTTTGTCTGTTATATCTTGGTGGACG-AGTAGTCGCGGTTGGGACTGAGGTG-CCTACAACGTGCTTTTGAGTGTGTCTGTGTCTCCGTGTGCGCCGTGTGCGGATAGCTTGCTATGCGTGTGT---GGTTGTGTGTGGATTGATGCGGGCTTTAACTTGTTGCCGTTCACTCGGGTATCGGTATGACGAAGGCGGACCTTATCAACAACCTGGGTACGATCGCCAAGTCGGGTACCAAGGCTTTCATGGAAGCTCTGGCTGCCGGCGCCGACATCAGTATGATTGGTCAGTTCGGTGTGGGTTTCTACTCGGCCTACCTCGTGGCTGACAAGGTCGTGGTGCACTCGAAGCACAACGATGACGAGCAGTACGTGTGGGAGTCGGCTGCTGGCGGCTCGTTCACCGTCACGCCTGAC---ACGTCGGAGCCTATCCTGCGTGGTACGCGCATTGTCCTGAAGCTCAAGGAGGACATGCTGGAGTACCTTGAGGAGCGCAAGCTTAAAGACTTGGTGAAGAAGCACTCGGAGTTCATTGGCTTCCCTATCAAGCTCTACGTTGAGAAGACGGAGGAGAAGGAAGTGACGGATGACGAAGAGGAGGAAGACG---AGAAGGAGGGMGAGGACGACAAGCCCAAGGTTGAGGAGGTTGAA---GAGGAGGAGGGTGAAAAGAAGAAGAAGACCAAGAAGATCAAGGAGGTGACCCACGAGTGGGACCACCTCAACAGCCAGAAGCCCATCTGGATGCGCAAGCCGGAGGATGTGACACACGAGGAGTACGCCTCGTTCTACAAGTCGCTTACCAACGACTGGGAGGAGCACGCTGCTGTTAAGCACTTCTCGGTGGAAGGTCAGCTGGAGTTCAAGGCCTGCCTGTTCACCCCCAAGCGCGCTCCCTTYGACATGTTCGAGGGTGGTGCCAAGAAGAAACTGAACAACATCAAGCTGTACGTGCGTCGCGTGTTCATCATGGACAACTGCGAGGAGCTCATGCCYGAGTACCTGTCGTTTGTCAAGGGTGTTGTCGACTCGGAGGACYTGCCGCTGAACATTTCGCGTGAGACCCTGCAGCAGAACAAGATCCTGCGTGTGATYAAGAAGAACTTGGTGAAGAAGTGCCTGGAGATGTTCGCTGAACTTGCTGAGGACAACGAGAAGTACAACAAGTTCTACGAGGCCTTCAGCAAGAACCTG-AAGCTCGGYATCCACGAGGACTCGACCAACCGCACCAAGATTGCCAAGCTCCTGCGTTACCACTCGACCAAGTCGGGAGAGGAGATGACTTCGCTGGATGACTACATTTCTCGCATGCCGGAGAACCAGCCAGGCATCTACTACGTGACGGGCGAGAGCAAGAAGTCGGTGGAGAACTCGCCATTCATTGAGAAGCTCAAGAAGAAGGGYTAYGAGGTGCTGTTCATGGTTGAGGCTATTGACGAGTACGCTGTGCAGCAGCTGAAGGAGTACGAGGGCAAGAAGCTTATCTGYGCTACTAAGGAGGGCCTCAAGATGGAGGAGACTGAGGACGAGAAGAAGTCGTTCGAGGAAGCTAAGGCCGCCACGGAGGGCCTTCACGGTCATTGACGCCCCTGGCCACCGTGATTTCATCAAGAACATGATCACGGGTACCTCGCAGGCCGATTGCGCCATTCTGGTGGTTGCTTCGGGTGTGGGTGAGTTCGAGGCTGGTATCTC---GAAGGAGGGCCAGACCCGCGAGCACGCTCTGCTTGCCTTCACCCTGGGTGTGAAGCAGATGATCGTCGCCATCAACAAGATGGACGACTCGTCTGTCATGTACGGCCAGGCCCGTTACGAGGAGATCAAGTCTGAGGTCACCACGTACCTGAAGAAGGTCGGCTACAAGCCCGCCAAGATCCCGTTCGTGCCTATCTCGGGCTGGGAGGGTGACAACATGATTGACCGCTCCAGCAACATGCCGTGGTACAAGGGACCTTACCTCCTTGAGGCTCTTGACAACCTGAACGCCCCTAAGCGTCCGGTTGACAAGCCGCTCCGTCTGCCTCTTCAGGATGTGTACAAGATCGGCGGTATTGGCACGGTACCGGTCGGCCGTGTGGAGACCGGTGTCATCAAGCCTGGCATGGTCGCCACTTTCGGCCCCGTKGGTCTGTCGACGGAAGTYAAGTCCGTTGAGATGCACCACGAGTCTCTGCCGGAGGCTGTCCCTGGTGACAACGTTGGCTTCAACGTCAAGAACGTGTCGGTGAAGGAGCTGCGTCGTGGTTTCGTCGCTTCGGACTCGAAGAACGA---CCCCGCTAAGGGCACCCAGGACTTCACCGCCCAGGTGATTGTGCTGAACCACCCTGGCCAGATCGGCAACGGCTACTCGCCGGTGCTTGACTGCCACACGGCCCACGTTGCCTGCAAGTTCAAAGAGATCACGGAGAAGATGGACCGTCGTTCGGGCAAGGTGCTCGTCCAGAACAAGAAGCCGTTCATTAAGTCGCGCTTCTGCCGTGGTGTGCCCGACCCGAAGATCCGTATCTACGATGTGGGTAACAAGAAGGCTTCGGTCGACGCCTTCCCGTTCGTGGCCCACTTGGTGTCCGACGAGAAGGAGCAGCTGTCTTCGGAGGCTCTTGAAGCTGCCCGTGTTGCTGCTAACCGTTACYTGACCAAGTACTGCGGTAAGGACAACTTCCACATGCGCATCCGTTGCCACCCGTTCCAGGTCCTGCGTATCAACAAGATGCTTTCGTGCGCTGGTGCCGATAGACTGCAGACGGGTATGCGTCACGCTTACGGTAAGCCCGCTGGTGTGGCTGCCCGTGTGGCCATTGGCCAGCCCATTATCTCGGTGCGTTCCAAGGACTCGTTCGGCCCTTCGGTCGTTGAGGCTCTGCGTCGTGCACGCTCCGGATTCGGAGAAGATCCCCGCCGACAAGCTGCAGGAGGTGTACGAGGGTTTCATTGCCAAGTGCGCTGGCTCGAGCAAGATCGTGTCGATCGAGGACCCGTTCGACCAGGACGACTGGGAGTCGTGGATTAAGTTCACGGGCAAGGTTGGCAAGGACGTGCAGATCGTGGGTGACGATTTGACCGTGACGAACCCTACCCGTGTGAAGAAGGCCATCGAGCAGAAGGCTTGCAACGCCCTTCTGCTGAAGGTGAACCAGATTGGCAGCATCACGGAGTCTATTGAGGCCGTGACGATGGCCAAGAAGGCCGGCTGGGCCATCATGGCCAGCCACCGCAGTGGTGAGACGGAGGACACGTTCATCGCTGACCTGGCTGTGGGTCTGAGCGCTGGTCAGATGGTAATAACTGGGCCAAGGGACATTACACGGAGGGTGCTGAGCTGATTGACTCAGTCCTGGATGTCGTCCGTAAGGAGGCAGAGAGCTGTGACTGTCTGCAGGGTTTCCAGATCACCCACTCGCTTGGTGGTGGTACCGGTTCCGGTATGGGTACGCTTCTTATTTCTAAGATTCGTGAGGAGTATCCCGATCGTATCATGTGCACGTACTCGGTGTGCCCATCGCCTAAGGTGTCGGATACCGTCGTGGAGCCTTACAACGCTACGCTGTCGGTCCACCAGCTTGTCGAGAATGCTGATGAAGTCATGTGCCTGGATAACGAGGCCCTGTACGACATTTGCTTCCGTACGCTGAAGCTCACGACCCCCACCTACGGTGACTTGAACCACTTGGTGTGTGCTGCCATGTCCGGCATCACCACGTGCCTTCGTTTCCCGGGTCAATTGAACTCGGACCTGCGTAAGCTGGCCGTGAACCTGATCCCGTTCCCGCGTCTCCACTTCTTTATGATTGGTTTCGCCCCGCTGACTTCTCGTGGCTCACAGCAGTACCGTGCTCTGACGGTGCCTGAGCTGACCCAACAGCAGTTCGACGCTAAGAACATGATGTGCGCCGCTGACCCTCGTCACGGCCGCTATTTAACTGCCGCGTGTATGTTCCGCGGACGCATGAGCACGAAGGAGGTCGATGAGCAGATGCTCAACGTGCAGAACAAGAACTCATCGTACTTCGTTGAGTGGATCCCCAACAACATCAAGGCTAGCGTGTGTGATATCCCGCCCAAGGGTCTGAAGATGAGCACCACGTTCATTGGTAACTCGACTGCCATCCAGGAGATGTTCAAGCGTGTATCCGAACAGTTCACAGCTATGTTCCGTCGTAAGGCTTTCTTGCACTGG

Phytophthora_pseudosyringae TTTTTTAATTATGATTACTGTATTTGTTTGTTGGATGTTGTTTAGAGTTATTACTCTTTTTGATGAAAAAACAAATAAAATTCCAGCAACTATTGTACATGGTGCTACAATTGAAATTATTTGGACTACTCTTCCAGCTTTAATTTTATTAACAGTTGCCGTACCTTCTTTTGCATTATTATATTCAATGGATGAAGTAATTGATCCTATTATTACTTTAAAAGTTATAGGTAGTCAATGGTATTGGAGTTATGAATATTCAGATAATTTAGAATTTTCAGATGAACCTTTAATTTTTGATAGTTATATGGTTCAAGAAGACGATTTAGCTTTAGGGCAATTTAGAATTTTAGAAGTAGATAATCGTGTAGTTGTTCCAACTAATAGTCATATTAGGGTATTAATTACAGCATCAGATGTTTTACATTCATGGGCTATACCTTCATTAGGTATTAAATTAGATGCTTGTCCAGGTCGTTTAAATCAAACCGGCGAGTGAAGCGGGAAGAGCTCAAGCTTAAAATCTCCGTGCAAGTTTTGCACGGCGAATTGTAGTCTATAGAGGC-GTGGTCAGCGTGGGCGCTCGGGGCAAGTTCCTTGGAAGAGGACAGCATGGAGGGTGATACTCCCGTTCATC-CCTGAGTTGCTCGTGCGTACGACCCGTTTTCTTTGAGTCGCGTTGTTTGGGAATGCAGCGCAAAGTAGGTGGTAAATTCCATCTAAAGCTAAATATTGGTGCGAGACCGATAGCGAACAAGTACCGTGAGGGAAAGATGAAAAGAACTTTGAAAAGAGAGTTAAAGAGTACCTGAAACTGCTGAAAGGGAACCGAATCGTTTCCAGTGTCTATAATCCGTGGCATATTTCATTGGCGAGTGTGTGCGTGCGTGTGCTGTGGCAGCGGTCTTTAT--GGCTGCGCTCGGCGTG---TG-TGCTGTGTGTGCTTGCTGGTGCCCTGTGCTGCGGTGGGACGTCAAGGTCAGTTCGTATGCTGCGGGAAATGGCTGCCGAGGAGGTAGGGCTTACGCTCCGCGTTTGTCTGTTATATCTTGGTGGACG-AGTCGTCGCGGTTGGGACTGAGGTG-CCTACAACGTGCTTTTGAGTGGGTTTGTGTCTCCGTGTGCGCCGTGTGCGGATAGCTTGCTATGCGTGTGT---GGTTGTGTGTGGATTGATGCGGGCCTTAACTTGTTGCCGTTCACTCGGGTATCGGCATGACCAAGGCCGACCTCATCAACAACCTGGGTACGATCGCCAAGTCGGGCACCAAGGCCTTCATGGAGGCGCTGGCCGCCGGCGCGGACATCAGCATGATCGGCCAGTTCGGCGTGGGCTTCTACTCGGCCTACCTGGTCGCCGACAAGGTCGTGGTGCACTCGAAGCACAACGACGACGAGCAGTACGTGTGGGAGTCCGCCGCGGGCGGCTCGTTCACCGTGACGCCCGAC---ACGACGGAGCCCATCCTGCGCGGCACGCGCATCGTCCTGAAGCTCAAGGAGGACATGTTGGAGTACCTCGAGGAGCGTAAGCTCAAGGACCTGGTGAAGAAGCACTCGGAGTTCATTGGCTTCCCCATCAAGCTCTACGTCGAGAAGACGGAGGAGAAGGAGGTGACTGACGACGAGGAGGAAGAAGACG---AGAAGGAGGGCGAGGACGACAAGCCCAAGGTCGAGGAGGTCGAAGAGGAGGAGGAGGGTGACAAGAAGAAGAAGACCAAGAAGATTAAGGAGGTGACCCACGAATGGGACCACCTCAACAGCCAGAAGCCCATCTGGATGCGCAAGCCCGAGGACGTGACCCACGAGGAGTACGCGTCGTTCTACAAGTCGCTCACCAACGACTGGGAGGAGCACGCCGGCGTCAAGCACTTCTCGGTGGAGGGCCAACTGGAGTTTAAGGCCTGCCTGTTCACCCCCAAGCGCGCCCCGTTCGACATGTTCGAGGGCGGTGCCAAGAAGAAAGTGAACAACATCAAGTTGTACGTGCGTCGCGTGTTCATCATGGACAACTGCGAGGAGCTCATGCCCGAGTACCTGTCGTTCGTCAAGGGTGTTGTCGACTCGGAGGACCTGCCGCTGAACATCTCGCGTGAGACCCTGCAGCAGAACAAGATCCTGCGCGTGATCAAGAAGAACCTGATCAAGAAGTGCCTGGAGATGTTCGCTGAGCTCGCCGAGGACAACGAGAAGTACATTAAGTTCTACGAGGCCTTCAGCAAGAACCTG-AAGCTCGGCATCCACGAGGACTCGACCAACCGCACCAAGATCGCCAAGCTCCTGCGTTATCACTCGACCAAGTCTGGCGAGGAGATGACGTCGCTGGACGACTACATCTCGCGCATGCCCGAGAGCCAGCCCGGCATCTACTACGTGACGGGTGAGAGCAAGAAGTCCGTGGAGAGCTCGCCCTTCATTGAGAAGCTCAAGAAGAAGGGCTACGAGGTGCTGTTCATGGTGGAGGCCATTGACGAGTACGCTGTGCAGCAGCTGAAGGAGTACGAGGGCAAGAAGCTCATCTGCGCCACCAAGGAGGGCCTCAAGATGGAGGAGACCGAGGACGAGAAAAAGTCGTTCGAGGAGGCCAAGGCCGCCACGGAGGGCCTTCACGGTCATCGACGCCCCTGGCCACCGTGACTTCATCAAGAACATGATCACGGGCACGTCGCAGGCCGACTGCGCCATCCTGGTGGTGGCCTCGGGCGTGGGCGAGTTCGAGGCTGGTATCTC---CAAGGAGGGCCAGACGCGCGAGCACGCTCTGCTCGCCTTCACCCTGGGCGTGAAGCAGATGGTCGTCGCTATCAACAAGATGGACGACTCCTCTGTCATGTACGGCCAGGCCCGTTACGAGGAGATCAAGGCCGAGGTGACGACCTACCTGAAGAAGGTGGGCTACAAGCCGGTCAAGATCCCCTTCGTGCCCATCTCCGGATGGGAGGGAGACAACATGATCGACCGCTCCGCCAACATGCCGTGGTACAAGGGACCGTACCTCCTCGAGGCTCTTGACAACCTGAACGCCCCCAAGCGTCCGCTTGACAAGCCCCTCCGTCTGCCCCTCCAGGACGTGTACAAGATCGGCGGTATCGGCACGGTACCGGTCGGCCGCGTGGAGACCGGTGTCATCAAGCCTGGCATGATCGCCACGTTCGGCCCCGTGGGTCTGTCGACGGAAGTCAAGTCCGTTGAGATGCACCACGAGTCTCTGCCGGAGGCTCTCCCCGGTGACAACGTTGGCTTCAACGTCAAGAACGTGTCGGTCAAGGAGCTGCGTCGTGGCTTCGTCGCCTCGGACTCCAAGAACGA---CCCGGCCAAGGGCACGCAGGACTTCACCGCCCAGGTGATCGTGCTGAACCACCCCGGCCAGATCGGCAACGGCTACTCGCCGGTGCTTGACTGCCACACGGCCCACGTTGCCTGTAAGTTCAAAGAGATCACGGAGAAGATGGACCGTCGTTCGGGCAAGGTGCTCGTCCAGAACAAGAAGCCGTTCATTAAGTCGCGCTTCTGCCGTGGTGTGCCTGACCCGAAGATCCGCATCTACGATGTGGGTAACCGCAAGGCATCGGTGGACGCCTTCCCGTTCGTGGCCCACCTGGTGTCCGACGAGAAGGAGCAGCTGTCGTCGGAGGCCCTCGAGGCTGCCCGCATTGCCGCTAACCGTTACCTGACCAAGTACTGCGGCAAGGACAACTTCCACATGCGCATCCGTTGCCACCCGTTCCAGGTCCTGCGTATCAACAAGATGCTTTCGTGCGCCGGCGCCGATAGACTGCAGACCGGTATGCGTCACGCCTACGGTAAGCCCGCCGGTGTGGCCGCCCGCGTGGCCATCGGCCAGCCCATTATCTCGGTGCGCTCCAAGGACTCGTTCGGCCCCTCGGTCGTGGAGTCGCTGCGTCGTGCACGCTCCGGAGTCGGAGAAGATCCCGGCCGAGAAGCTGCAGGAGGTGTACGAGGGCTTCATCGCCAAGTGCGCTGACTCGAGCAAGATCGTGTCCATTGAGGACCCGTTCGACCAAGACGACTGGGAGTCGTGGGTGAAGTTCACGGGCAAGGTCGGCAAGGACGTGCAGATCGTGGGAGACGACCTTACGGTGACRAACCCCACCCGTGTGAAGAAGGCCATCGAGCTGAAGGCTTGCAACGCCCTTCTGCTGAAGGTGAACCAGATTGGCAGCATCACGGAGTCGATCGAGGCCGTGACGATGGCCAAGAAGGCCGGCTGGGCCATCATGGCCAGCCACCGCAGCGGCGAGACCGAGGACACGTTCATCGCCGACCTGGCCGTGGGCCTGAGCGCCGGCCAGATGGTAACAACTGGGCCAAGGGACACTACACCGAGGGTGCCGAGCTGATTGACTCGGTGCTCGACGTCGTCCGCAAGGAGGCTGAGAGCTGCGACTGCCTGCAGGGTTTCCAGATCACGCACTCGCTGGGTGGCGGTACCGGCTCCGGTATGGGTACGCTTCTTATCTCGAAGATTCGTGAGGAGTACCCCGACCGTATCATGTGCACGTACTCGGTGTGCCCATCGCCCAAGGTGTCGGATACCGTCGTGGAGCCCTACAACGCTACGCTCTCGGTGCACCAGCTGGTTGAGAACGCCGACGAGGTCATGTGCCTTGATAACGAGGCCCTGTACGACATTTGCTTCCGTACGCTGAAGCTCACGACCCCCACCTACGGCGACCTGAACCACCTGGTGTGCGCCGCTATGTCCGGTATCACCACGTGCCTGCGTTTCCCCGGTCAGCTGAACTCGGACCTTCGTAAGCTTGCGGTGAACCTGATCCCGTTCCCGCGTCTTCACTTCTTCATGATTGGTTTCGCCCCGCTGACCTCGCGTGGCTCGCAGCAGTACCGTGCCCTGACGGTGCCCGAGCTGACCCAGCAGCAGTTCGATGCCAAGAACATGATGTGCGCCGCTGACCCTCGCCACGGCCGCTATTTAACTGCCGCGTGTATGTTCCGCGGACGTATGAGCACGAAGGAGGTTGATGAGCAGATGCTGAACGTGCAGAACAAGAACTCGTCGTACTTCGTCGAGTGGATCCCCAACAACATCAAGGCTAGCGTCTGTGACATCCCGCCCAAGGGTCTCAAGATGAGCACCACGTTCATCGGTAACTCGACCGCCATCCAGGAGATGTTCAAGCGCGTGTCCGAACAGTTCACGGCCATGTTCCGTCGTAAGGCTTTCTTGCACTGG

Phytophthora_ramorum TTTTTTAATTATGATTACTGTATTTGTTTGTTGGATGTTATTTAGAGTTATTACTCTTTTTGATGAAAAAAAAAATAAAATTCCTTCAACGGTAGTACATGGAGCTACTATTGAAATTATTTGGACATCTATTCCAGCTTTAATTTTATTAGTTGTTGCAGTACCATCTTTTGCTTTATTATATTCAATGGATGAGGTAATTGATCCAATTATTACATTAAAAGTAATTGGTAGTCAATGGTATTGGAGTTATGAATATTCTGATAATTTAGAATTTTCTGATGAACCTTTAATTTTTGATAGTTACATGATACAAGAAGATGATTTAGCAATAGGTCAATTTAGAGTTTTAGAAGTAGATAATCGTGTAGTTGTACCAACAAATAGTCATATTAGAGTATTAATTACCGCATCAGATGTTTTACATTCATGGGCTATTCCTTCATTAGGTATTAAATTAGATGCATGTCCTGGACGTTTAAATCAAACCGGCGAGTGAAGCGGGATGAGCTCAAGCTTAAAATCTCCGTGCAAGTTTTGCACGGCGAATTGTAGTCTATAGAGGC-GTGGTCAGTGAGGGCGCTCGGGGCAAGTTCCTTGGAAGAGGACAGCATGGAGGGTGATACTCCCGTTCATC-CCTGAGTGGCTTTTGCGTACGACCCGTGTTCTTTGAGTCGCGTTGTTTGGGAATGCAGCGCAAAGTAGGTGGTAAATTCCATCTAAAGCTAAATATTGGTGCGAGACCGATAGCGAACAAGTACCGTGAGGGAAAGATGAAAAGAACTTTGAAAAGAGAGTTAAAGAGTACCTGAAACTGCTGAAAGGGAACCGAATCGTTTCCAGTGTCTATAATCCGTGGCATATTTCATTGGCGAGTGTGTGCGTGCGTGTGCTGTGGCAGTGGCTTTTTT--GGCTGCGCTCGGCGTG---TG-TGCTGTGTRTGCTTGTTGGTGCCCTGTGCTGCGGTGGGACGTCAAGGTCAGTTCGTATGCTGCGGGAAATGGCTGCCGAGGAGGTAGGGCTTACGCTCCGCGTTTGTCTGTTATATCTTGGTGGACG-AGTAGTCGCGGTTGGGACTGAGGTG-CCTACAACGTGCTTTTGAGTGTGTATGGGTCTCCGTGTGCGCCGTGTGCGGATAGCTTGCTATGCGTGTGT---GGTTGTGTGTGGATTGATGCGTACTGTAACTTGTCGCCGTTCACTCGGGTATCGGTATGACCAAGGCCGACCTCATCAACAACCTGGGCACGATCGCCAAGTCGGGCACCAAGGCCTTCATGGAGGCCCTGGCCGCCGGCGCTGACATCAGCATGATTGGCCAGTTCGGTGTGGGTTTCTACTCGGCCTACCTGGTCGCCGACAAGGTCGTGGTGCACTCGAAGCACAACGACGACGAGCAGTACGTGTGGGAGTCCGCCGCCGGTGGCTCGTTCACGGTCACGCCCGAC---ACGACGGAGCCCATCCTGCGCGGCACGCGCATCGTCCTGACGCTCAAGGAGGACATGCTCGAGTACCTCGAGGAGCGCAAGCTCAAAGACCTGGTGAAGAAGCACTCGGAGTTCATTGGCTTCCCCATCAAGCTCTACGTCGAGAAGACGGAGGAGAAGGAGGTTACCGACGACGAGGAGGAGGATGACG---AGAAGGAGGGCGAGGACGACAAGCCCAAGGTCGAGGAGGTCGAC---GAGGAGGAAGGCGAGAAGAAGAAGAAGACAAAGAAGATCAAGGAGGTCACCCACGACTGGGACCACCTGAACAGCCAGAAGCCCATCTGGATGCGCAAGCCCGAGGACGTGACCCACGAGGAGTACGCCTCGTTCTACAAGTCGCTCACCAACGACTGGGAGGAGCACGCCGGCGTGAAGCACTTCTCTGTGGAGGGACAGCTCGAGTTCAAGGCCTGTCTGTTCGCCCCCAAGCGCGCTCCGTTCGACATGTTCGAGGGTGGTGCCAAGAAGAAAATCAACAACATCAAGCTGTACGTGCGTCGCGTGTTCATCATGGACAACTGCGAGGAGCTCATGCCCGAGTACTTGTCGTTCGTCAAGGGTGTTGTCGACTCGGAGGACCTGCCGCTTAACATCTCGCGTGAGACCCTGCAGCAGAACAAGATCCTGCGTGTGATCAAGAAGAACCTCGTCAAGAAGTGCCTGGAGATGTTCGCCGACCTTGCTGAGGACAACGAGAAGTACAACAAGTTYTACGAGGCCTTCAGCAAGAACCTG-AAGCTGGGCATCCACGAGGACTCGACCAACCGCACCAAGATCGCCAAGCTCCTGCGCTACCACTCGACCAAGTCTGGCGAGGAGATGACGTCCCTGGACGACTACATCTCGCGTATGCCCGAGAGCCAGCCCGGCATCTACTACGTGACGGGCGAGAGCAAGAAGTCGGTGGAGAACTCGCCGTTCATTGAGAAGCTCAAGAAGAAGGGCTACGAGGTGCTCTTCATGGTTGAGGCCATTGACGAGTACGCCGTGCAGCAGCTGAAGGAGTACGAGGGCAAGAAGCTCATCTGCGYGACCAAGGAGGGCCTCAAGATGGAGGAGACTGAGGACGAGAAGAAGTCGTTCGAGGAGGCCAAGGCCGCCACCGAGGGCCTTCACGGTCATTGACGCCCCTGGCCACCGTGACTTCATCAAGAACATGATTACGGGCACCTCGCAGGCTGACTGCGCCATTCTGGTCGTCGCCTCGGGTGTGGGCGAGTTCGAGGCTGGTATCTC---CAAGGAGGGCCAGACGCGCGAGCACGCTCTGCTTGCCTTCACGCTCGGCGTGAAGCAGATGATCGTGGCCATCAACAAGATGGACGACTCGTCCGTCATGTACGGCCAGGCYCGTTACGAGGAGATCAAGAACGAGGTCACCACGTACCTGAAGAAGGTGGGCTACAAACCCGCCAAGATCCCGTTCGTGCCCATTTCGGGCTGGGAGGGTGACAACATGATCGAGAAGTCGGGCAACATGCCGTGGTACAAGGGACCGTACCTCCTTGAGGCTCTCGACACGCTGAACGCCCCCAAGCGTCCGTCGGACAAGCCTCTGCGYCTGCCCCTCCAGGACGTGTACAAGATCGGCGGTATCGGCACGGTACCGGTCGGCCGTGTGGAGACYGGTGTCATCAAGCCTGGCATGGTCGCCACGTTCGGCCCCGTGGGTCTGTCGACGGAAGTCAAGTCCGTTGAGATGCACCACGAGTCTCTGCCGGAGGCTGTCCCTGGTGACAACGTCGGCTTCAACGTCAAGAACGTGTCGGTGAAGGAGCTGCGTCGCGGCTTCGTCGCTTCGGACTCGAAGAACGA---CCCGGCCAAGGGCACGCAGGACTTCACCGCCCAGGTGATCGTGCTGAACCACCCCGGCCAGATCGGCAACGGCTACTCGCCGGTGCTGGACTGCCACACGGCCCACGTTGCCTGCAAGTTCAAAGAGATCACGGAGAAGATGGACCGTCGTTCGGGCAAGGTGCTCGTCCAGAACAAGAAGCCGTTCATTAAGTCGCGCTTCTGCCGTGGTGTGCCCGACCCGAAGATCCGCATTTACGATGTGGGTAACAAGAAGGCTTCGGTGGACTCCTTCCCCTTCGTGGCTCACCTGGTGTCCGACGAGAAGGAGCAGCTGTCGTCGGAGGCTCTGGAGGCCGCCCGTATTGCCGCTAACCGTTACCTGACCAAGTACTGCGGCAAGGACAACTTCCACATGCGCATTCGTTGCCACCCTTTCCAGGTGCTGCGTATCAACAAGATGCTTTCGTGCGCCGGTGCCGATAGACTGCAGACCGGTATGCGTCACGCTTACGGTAAGCCCGCCGGTGTTGCCGCCCGTGTGGCCATCGGCCAGCCCATTATCTCGGTGCGCTCCAAGGACAACTTCGGCCCCTCGGTCGTGGAGGCTCTGCGTCGTGCACGCTCCCGAGTCGGAGAAGATCTCTGCCGACAAGCTGCAGGAGATGTACGAGGGCTTCATCTCGAAGTGCGCTTCCTCGAGCAAGATTGTGTCGATCGAGGACCCGTTCGACCAGGACGACTGGGAGTCGTGGGTGAAGATCACGGGCAAGGTGGGCAAGGACGTGCAGATCGTGGGCGATGACCTGACGGTGACGAACCCCACCCGTGTGAAGAAGGCTATTGAGCTGAAGGCTTGCAACGCCCTTCTGCTGAAGGTGAACCAGATCGGCAGCATCACGGAGTCGATTGAGGCCGTGACGATGGCGAAGAAGGCCGGCTGGGCCATCATGGCCAGCCACCGCAGCGGTGAGACGGAGGACACGTTCATTGCCGACCTGGCTGTGGGTCTGAGCGCCGGCCAGATGGTAACAACTGGGCTAAGGGACACTACACGGAGGGTGCCGAGCTTATCGACTCGGTGCTCGACGTCGTCCGCAAGGAGGCCGAGAGCTGTGACTGCCTGCAGGGGTTCCAGATCACGCACTCGCTTGGTGGCGGTACCGGTTCTGGTATGGGCACGCTTCTGATCTCCAAGATCCGTGAGGAGTACCCGGACCGTATCATGTGCACGTACTCGGTGTGCCCGTCGCCCAAGGTGTCGGACACGGTCGTGGAGCCCTACAACGCCACGCTGTCGGTGCACCAGCTTGTCGAGAACGCCGACGAGGTCATGTGCCTGGATAACGAGGCGCTGTACGACATTTGCTTCCGCACGCTCAAGCTCACCACCCCCACCTACGGTGACCTGAACCACCTGGTGTGCGCCGCTATGTCCGGCATCACCACGTGCCTGCGTTTCCCGGGTCAGCTGAACTCGGACCTGCGGAAGCTGGCGGTGAACTTGATTCCGTTCCCGCGTCTTCACTTCTTCATGATTGGTTTCGCCCCGCTGACCTCGCGTGGCTCGCAGCAGTACCGTGCCCTGACGGTGCCCGAGCTGACCCAGCAGCAGTTCGACGCAAAGAACATGATGTGCGCCGCCGACCCTCGTCACGGCCGCTATTTAACTGCCGCGTGTATGTTCCGCGGACGTATGAGCACGAAGGAGGTTGAYGAGCAGATGCTGAACGTGCAGAACAAGAACTCGTCGTACTTCGTCGAGTGGATCCCTAACAACATCAAGGCTAGCGTGTGTGACATCCCGCCCAAGGGCCTGAAGATGAGCACTACGTTCATCGGTAACTCGACCGCTATCCAGGAGATGTTCAAGCGTGTGTCTGAGCAGTTTACGGCTATGTTCCGTCGTAAGGCTTTCTTGCACTGG

Hyaloperonospora_arabidopsidis TTTTTTAATTATTATTACAGTATTTGTTTGTTGGATGTTATTTAGAGTTATTACTCTTTTTGATGAAAAAAAAAATAGAATTCCTTCTACAGTAGTACATGGTGCTACGATTGAAATTATTTGGACTTCTATACCAGCTTTAATTTTATTAATTGTTGCTATTCCTTCATTTGCCTTATTATATTCAATGGATGAAGTAATTGATCCAATTATTACTTTAAAAGTAATTGGAAGTCAATGGTATTGGAGTTATGAATATTCAGATAATTTAGAATTTTCAGATGAACCTTTAATTTTTGATAGTTATATGATACAAGAAGATGATTTAACAATAGGTCAATTTAGAATTTTAGAAGTAGATAATCGCGTAGTAGTTCCAACGAATAGTCATATTCGTGTACTAATTACGGCTTCAGATGTTTTACATTCTTGGGCTATTCCTTCATTAGGGATTAAATTAGACGCATGTCCTGGTCGTTTAAACCAAACCGGCGAGTGAAGCGGGATGAGCTCAAGCTTAAAATCTCTGTCACATTTATGTGCGGCGAATTGTAGTCTATAGAAGC-GTGGTCAGTGTAGGCACTTGAGATAAGTTCCTTGAAAAAGGACAGCATGGAGGGTGAAACTCCCGTTCATA-CTTGAGTAGCTTATGCGTACGACCCGTTTTCTTTGAGTCGCGTTGTTTGAGAATGCAGCGCAAAGTAGGTGGTAAATTCCATCTAAAGCTAAATATTGGTGCGAGACCGATAGCGAACAAGTACCGTGAGGGAAAGATGAAAAGAACTTTGAAAAGAGAGTTAAAGAGTACCTGAAACTGCTGAAAGGGAACCGGATCGTTTCTAGTGTCTATAATCTGTGACATATTTCATTGGGGAGTGTGTGCGTGTGTATACTATGGCAGTGGCTTTTTT--GGCTGCGCTTGGTGTG---CG-TGCTGTATGTGCTTACTGGTGCCCTGTGTTGCGGTGGGACGTCAAGGTCGGTTTGTATACTGCGGGAAATGGCTATTGAGGAGGTAGGGCTTACGCT-TGCGTTTGTCTATTATATCTTGGTAGTTG----TGTCGTGGTTGAGACTGAGGTG-TCTACAACGTGCTTATGAGTGGGACTGTGTCTCCGTGTGCGCCGTGTGCGGATAGCTTGCTATGTGTGTGT---GGTGGTGTGTGGATTGATGCGGGCTTTAACTTGTTGCCGTTCACTCGGGCATTGGCATGACGAAGGCCGACCTCATTAACAACTTGGGCACGATCGCCAAGTCAGGCACCAAGGCGTTCATGGAAGCCCTGGCTGCTGGCGCTGACATCTCTATGATTGGTCAGTTTGGCGTGGGCTTTTACTCGGCCTACCTGGTGGCCGACAAGGTCGTGGTGCACTCGAAGCACAATGACGACGAGCAGTACGTGTGGGAATCGGCCGCTGGTGGCTCGTTTACGGTCACGCCCGAC---ACGTCGGAGCCCATTCTGCGTGGTACGCGCATTGTGCTGAAGCTCAAGGAGGACATGCTGGAGTACTTGGAGGAGCGCAAGCTCAAGGACTTGGTGAAGAAGCACTCTGAGTTCATTGGCTTTCCGATTAAGCTGTACGTGGAGAAGACGGAGGAGAAGGAGGTGACGGACGACGAGGAGGAGGAAGACG---AGAAGGAGGGCGACGAGGATAAGCCCAAGGTGGAGGAAGTTGAGGACGAGGAGGAGGGCGAGAAGAAGAAGAAGACGAAGAAGATCAAGGAGGTGACGCACGATTGGAATCACTTGAACAGCCAGAAGCCTATCTGGATGCGCAAACCGGAAGACGTGACCCACGAAGAGTATGCGTCGTTCTACAAGTCGTTGACCAACGACTGGGAGGAGCATGCGGCGGTCAAGCACTTTTCGGTGGAAGGCCAGTTGGAGTTTAAGGCGTGTTTGTTCACGCCCAAGCGCGCGCCGTTTGACATGTTTGAAGGTGGCGCCAAGAAGAAACTGAACAACATTAAGCTGTACGTGCGTCGCGTGTTCATTATGGACAACTGCGAGGACTTGATGCCCGAGTACCTTTCGTTCGTCAAGGGTGTGGTCGACTCGGAGGATTTGCCGCTGAACATTTCGCGTGAGACGCTGCAGCAGAACAAGATTTTGCGTGTGATTAAAAAGAACCTGATCAAGAAGTGTCTTGAGATGTTTGCTGAGCTTGCCGAGGACAACGAGAAGTACACCAAGTTCTACGAGGCGTTCAGCAAGAACCTG-AAGCTGGGCATCCACGAGGACTCGACCAACCGTACGAAGATTGCCAAGCTTCTGCGCTACCACTCGACCAAGTCTGGCGAGGAGTTGACTTCGCTTGATGACTACATTACGCGCATGCCGGAGAGCCAGCCTGGTATTTACTACGTGACGGGCGAGAGCAAGAAGGCGGTGGAGAACTCGCCATTCATCGAGAAGCTCAAGAAGAAGGGGTATGAAGTGCTGTTTATGGTGGATGCCATTGACGAGTACGCTGTGCAGCAGCTGAAGGAGTACGAGGGCAAGAAGCTGATCTGTGCCACCAAGGAGGGTCTCAAGATGGAGGAGTCGGAGGACGAGAAGAAGTCGTTCGAGGAAGCTAAGGCTGCCACGGAAGGACTTCACGGTCATTGACGCCCCTGGTCACCGTGACTTTATTAAGAACATGATTACGGGCACGTCCCAGGCCGATTGCGCCATCTTGGTGGTGGCTTCGGGTGTGGGTGAGTTTGAAGCTGGTATCTC---GAAGGAGGGACAGACGCGCGAGCACGCGCTGCTGGCCTTTACGCTTGGCGTGAAGCAGATGGTGGTGGCTATCAACAAGATGGACGACTCGTCTGTCATGTACGGCCAGGCCCGTTACGAGGAGATCAAGTCGGAGGTGACGACGTACTTGAAGAAGGTGGGCTACAAACCCGCCAAGATTCCGTTCGTGCCTATTTCTGGATGGGAGGGCGACAATATGATTGAGCGCTCGCCCAACATGGCGTGGTACAAGGGACCGTACCTCCTTGAGGCGCTTGACAACCTGAACCCGCCCAAGCGTCCGTCGGACAAGCCGCTTCGTTTGCCTCTCCAGGACGTGTACAAGATCGGCGGTATTGGCACGGTACCGGTGGGACGTGTGGAGACTGGTGTCATTAAGCCTGGCATGGTCGCTACGTTTGGCCCTGTGGGTCTGTCGACGGAAGTTAAGTCTGTCGAGATGCACCACGAATCTCTGCCGGAAGCTCTTCCTGGTGACAACGTTGGCTTCAACGTGAAGAACGTGTCGGTGAAGGAACTGCGTCGTGGTTTCGTTGCTTCGGACTCGAAGAACGA---CCCTGCTAAGGGAACCCAGGACTTTACCGCCCAGGTGATCGTGCTGAACCACCCTGGTCAGATTGGCAACGGTTACTCGCCGGTGCTTGACTGCCATACGGCCCACGTTGCATGCAAGTTCAAAGAGATCACGGAGAAGATGGACCGTCGTTCGGGTAAGGTGCTCGTCCAGAACAAGAAACCGTTCATCAAGTCCCGCTACTGCCGTGGTGTGCCGGACCCGAAGATCCGCATCTACGACGTGGGGAACAAGAAAGCTTCGGTCGACACCTTCCCGTTCGTTGCCCACTTGGTCTCCGATGAGAAGGAGCAATTGTCTTCGGAAGCTTTGGAAGCTGCGCGTATTGCTGCCAATCGGTACTTGACCAAATACTGCGGCAAGGACAATTTCCACATGCGCATCCGTTGCCATCCGTTTCAAGTCCTGCGTATCAACAAGATGCTTTCGTGTGCTGGTGCCGATCGACTGCAGACGGGTATGCGTCACGCTTACGGTAAGCCTGCGGGTGTAGCTGCTCGTGTGTCCATTGGTCAGCCCATTATCTCGGTACGATCGAAGGACTCGTTCGGCCCATCGGTCGTTGAGGCTTTGCGCCGTGCATGCTGTGGACTCGGAGAAGATCTCGGCCGACAAGCTGCTGGAGGTGTACGAGGGCTTTATTGCCCAGTGTGCTAGGTCGAGCAGCATTGTGTCGATTGAGGACCCGTTCGACCAAGACGACTGGGAGTCGTGGAAGAAGATCACGGTCAAGGTCGGCAAGGACGTGCAGATCGTGGGCGACGACTTGACGGTGACGAACCCGACCCGTGTGAAGAAGGCTATTGAACAGAAGGCGTGCAACGCTCTTCTGCTGAAGGTGAACCAGATCGGTACCATCACCGAGTCGATTGAGGCCGTGACGATGGCAAAGAAGGCCGGATGGGGCATCATGGCTAGCCACCGCAGCGGGGAGACGGAGGACACGTTCATTGCGGACCTGGCGGTGGGTCTAAGCGCTGGCCAGATGGTAACAATTGGGCCAAGGGACACTACACGGAGGGTGCAGAGCTTATTGACTCGGTGCTGGACGTTGTCCGCAAAGAGGCTGAGAGCTGTGACTGCCTACAAGGTTTCCAGTTCACGCACTCGCTCGGCGGTGGTACCGGCTCTGGTATGGGCACGCTTCTTATCTCAAAGATCCGAGAAGAGTATCCGGACCGTATTATGTGCACATACTCCGTGTGTCCGTCCCCCAAGGTGTCGGACACTGTCGTCGAGCCCTACAACGCTACGTTGTCGGTGCACCAGCTCGTGGAGAACGCTGATGAAGTCATGTGCCTTGACAATGAGGCCCTGTATGACATTTGCTTCCGCACGCTGAAGCTCACGACTCCTACTTACGGTGATCTGAATCACTTGGTGTGTGCTGCTATGTCTGGTATCACGACGTGCCTACGTTTTCCCGGTCAGCTGAACTCAGACCTCCGTAAGCTGGCTGTGAACTTGATTCCGTTTCCACGTCTTCACTTCTTCATGATTGGTTTCGCTCCGCTGACGTCGCGAAGCTCGCAGCAGTACCGTGCGTTGACGGTGCCCGAGCTGACGCAGCAGCAGTTCGATGCTAAAAACATGATGTGTGCCGCCGACCCCCGCCATGGCCGCTATTTAACTGCCGCGTGTATGTTCCGCGGACGTATGAGTACGAAGGAGGTGGATGAGCAAATGCTGAACGTGCAAAACAAGAACTCGTCGTACTTCGTCGAGTGGATTCCCAACAACATCAAGGCTAGCGTGTGTGACATCCCGCCCAAGGGGTTGAAGATGAGTACCACCTTCATCGGTAACTCGACCGCAATCCAGGAGATGTTCAAGCGCGTGTCTGAGCAATTTACGGCCATGTTTCGTCGTAAGGCTTTCTTGCACTGG

Peronospora_matthiolae TTTTTTAATTATTATTACAGTATTTGTTTGTTGGATGTTATTTAGAGTTATTACTCTTTTTGATG-AAAAAAAAATAAAATACCTTCAACAATAGTACATGGTGCTACTATTGAAATTATTTGGACTTCTATACCAGCTTTAATTTTATTAATTGTTGCAATACCTTCATTTGCTTTATTATATTCAATGGATGAAGTAATTGATCCAATTATTACTTTAAAAGTAATAGGAAGTCAATGGTATTGGAGTTATGAATATTCAGATAATTTAGAATTTTCAGATGAACCTTTAATTTTTGATAGTTATATGATACAAGAAGATGATCTAACAATAGGTCAATTTAGGATTTTAGAAGTAGATAATCGTGTAGTAGTTCCAACTAATAGTCACATTCGTGTACTAATTACGGCCTCAGACGTTTTACATTCTTGGGCTATTCCTTCCTTAGGTATTAAATTAGATGCATGTCCAGGTCGTTTAAATCAAACCGGCGAGTGAAGCGGGATGAGCTCAAGCTTAAAATCTCCGTCACATTTATGTGCGGCGAATTGTAGTCTATAGAAGC-GTGGTCAGTGTGGACACTTGAGATAAGTTCCTTGAAAAAGGACAGCATGGAGGGTGAAACTCCCGTTCATA-CTTGAGTAGTTTATGCGTACGACCCGTTTTCTTTGAGTCGCGTTGTTTGAGAATGCAGCGCAAAGTAGGTGGTAAATTCCATCTAAAGCTAAATATTGGTGCGAGACCGATAGCGAACAAGTACCGTGAGGGAAAGATGAAAAGAACTTTGAAAAGAGAGTTAAAGAGTACCTGAAACTGCTGAAAGGGAACCGGATCGTTTCTAGTGTCTATAATCTGTGACATATTTCATTGGGGAGTGTGTGCGTGTGTGTACTATGGCAGTGGCTTTTTT--GGCTGCGCTTGGTGTG---CG-TGCTGTATGTGCTTACTGGTGCCCTGTGTTGCGGTGGGACGTCAAGGTCGGTTTGTATACTGCGGGAAATGGCTATTGAGGAGGTAGGGCTTACGCT-TGCGTTTGTCTATTATATCTTGGTAGTTG----TGTCGTGGTTGAAACTGAGGTG-TCTACAACGTGCTTATGAGTGGGACTGCGTCTCCGTGTGCGCCGTGTGCGGATAGCTTGCTATGTGTGTGT---GGTGGTGTGTGGATTGATGCGGGCTTTAACTTGTTGCCGTTCACTCGGGCATTGGCATGACGAAGGCCGACCTCATTAACAACTTGGGCACGATCGCCAAGTCAGGCACCAAGGCGTTCATGGAAGCCCTGGCTGCCGGCGCTGACATTTCGATGATTGGTCAGTTTGGCGTGGGCTTTTACTCGGCCTACCTGGTGGCTGACAAGGTCGTCGTGCACTCGAAGCACAATGACGACGAGCAGTACGTGTGGGAATCGGCTGCTGGTGGCTCGTTTACGGTCACGCCCGAC---ACGTCGGAGCCCATTCTGCGTGGCACGCGCATTGTGCTGAAGCTTAAGGAGGACATGCTGGAGTACTTGGAGGAGCGCAAGCTCAAGGACTTGGTGAAGAAGCACTCTGAGTTTATTGGCTTTCCGATCAAGCTGTATGTGGAGAAGACGGAGGAGAAGGAGGTGACGGACGACGAGGAGGAGGAGGACG---AGAAGGAGGGCGAGGAGGACAAGCCCAAGGTGGAGGAAGTTGAGGACGAGGAGGAGGGTGAGAAGAAGAAGAAGACGAAGAAGATTAAGGAGGTGACGCACGAGTGGAACCACTTGAACAGCCAGAAGCCGATCTGGATGCGCAAACCGGAAGACGTGACCCACGAAGAGTATGCGTCGTTCTACAAGTCGTTGACTAACGACTGGGAGGAGCACGCGGCGGTCAAGCACTTTTCGGTGGAAGGCCAGCTGGAGTTCAAAGCGTGTTTGTTCACGCCCAAGCGCGCGCCGTTTGACATGTTTGAAGGAGGCGCCAAGAAGAAACTGAACAACATCAAGCTGTACGTGCGTCGCGTGTTCATTATGGACAACTGCGAGGACTTGATGCCCGAGTACCTTTCGTTCGTCAAGGGTGTGGTCGACTCGGAGGACTTGCCGCTGAACATTTCGCGTGAGACGCTGCAGCAGAACAAGATTCTGCGTGTGATTAAGAAGAACTTGATCAAGAAGTGTCTGGAGATGTTCGCTGAGCTTGCCGAGGACAACGAGAAGTACACCAAGTTCTACGAGGCGTTCAGCAAGAACCTG-AAGCTGGGCATCCACGAGGACTCAACGAACCGTACGAAGATTGCCAAGCTTCTGCGCTACCACTCGACCAAGTCTGGCGAAGAGTTGACGTCGCTTGATGACTACATTACGCGCATGCCGGAGAGTCAGCCTGGTATTTACTACGTGACGGGCGAGAGCAAGAAGGCGGTGGAGAACTCGCCGTTCATCGAGAAGCTCAAGAAGAAGGGGTATGAAGTGCTGTTTATGGTGGATGCCATTGATGAGTACGCTGTGCAGCAGCTGAAGGAGTACGAGGGCAAGAAGCTGATCTGTGCCACCAAGGAGGGTCTCAAGATGGAGGAGTCGGAAGATGAGAAGAAGTCATTCGAGGAGGCCAAAGCTGCCACGGAGGGACTTCACGGTCATTGACGCCCCTGGTCACCGTGACTTTATTAAGAACATGATTACGGGCACGTCCCAGGCTGATTGCGCCATCTTGGTGGTGGCTTCGGGTGTGGGTGAGTTTGAAGCTGGTATCTC---GAAGGAGGGTCAGACGCGCGAGCACGCGCTGCTGGCCTTTACGCTTGGCGTGAAGCAGATGGTGGTGGCTATCAACAAGATGGACGACTCGTCTGTCATGTACGGCCAGGCCCGTTACGAGGAGATCAAGTCGGAAGTAACGACGTACTTGAAGAAGGTTGGCTACAAACCTGCCAAGATTCCGTTCGTGCCTATTTCTGGATGGGAGGGCGACAACATGATTGAGCGCTCGCCCAACATGGCGTGGTACAAGGGCCCATACCTTCTTGAGGCGCTTGACAACCTGAACCCGCCCAAGCGTCCATCGGACAAGCCGCTTCGTCTGCCTCTCCAGGACGTGTACAAGATTGGCGGTATTGGCACGGTACCAGTGGGACGTGTGGAGACTGGTGTCATTAAGCCTGGCATGGTCGCTACGTTTGGCCCTGTGGGTCTGTCGACGGAAGTCAAGTCTGTCGAGATGCACCACGAATCTCTGCCGGAAGCTCTTCCTGGTGACAACGTTGGTTTCAACGTGAAGAACGTGTCGGTGAAGGAACTGCGTCGTGGTTTCGTTGCTTCGGACTCGAAGAACGA---CCCTGCTAAGGGAACCCAGGACTTTACCGCCCAGGTGATCGTGCTGAACCACCCTGGTCAGATTGGCAACGGTTACTCGCCGGTGCTTGACTGCCACACGGCCCACGTTGCATGCAAGTTCAAAGAGATCACGGAGAAGATGGACCGTCGTTCGGGCAAGGTGCTCGTTCAGAACAAGAAACCGTTCATCAAGTCCCGCTACTGCCGTGGTGTGCCGGACCCGAAGATCCGCATCTACGATGTAGGGAACAAGAAAGCTTCGGTCGACACGTTCCCGTTCGTTGCCCACTTGGTCTCCGATGAGAAGGAGCAATTGTCTTCGGAAGCTTTGGAAGCTGCGCGTATTGCTGCCAATCGGTACTTGACCAAGTACTGCGGCAAGGACAATTTCCACATGCGCATCCGTTGCCATCCGTTCCAAGTTCTGCGTATTAACAAGATGCTTTCGTGTGCTGGTGCCGATCGACTGCAGACGGGTATGCGTCACGCTTACGGTAAGCCTGCGGGCGTAGCAGCTCGCGTGTCCATTGGCCAGCCCATTATCTCGGTACGATCGAAGGACTCGTTTGGCCCATCGGTCGTTGAAGCTTTGCGTCGTGCATGCTGCGGACTCGGAGAAGATCTCGGCCGACAAGCTGCTGGAGGTGTACGAGGGCTTTATTGCCCAGTGTGCCAGGTCGAGCAGCATCGTCTCGATTGAGGACCCGTTCGACCAAGACGACTGGGAGTCGTGGAAGAAGATTACGGCCAAGGTCGGCAAGGACGTACAGATCGTGGGCGACGACTTGACAGTGACGAACCCGACCCGTGTGAAGAAGGCTATTGAACAGAAGGCGTGTAACGCTCTTCTGCTGAAGGTGAACCAGATCGGTACCATCACCGAGTCGATTGAGGCCGTGACGATGGCAAAGAAGGCCGGATGGGGCATCATGGCTAGCCACCGCAGCGGGGAGACGGAGGACACGTTCATTGCGGACCTGGCGGTGGGTCTAAGTGCTGGCCAGATGGTAACAATTGGGCCAAGGGACACTACACGGAGGGTGCGGAGCTTATTGACTCGGTGCTGGACGTTGTCCGCAAAGAGGCTGAGAGCTGTGACTGCCTACAAGGTTTCCAGTTCACGCACTCGCTCGGCGGTGGTACCGGCTCTGGTATGGGCACGCTGCTTATCTCAAAGATCCGAGAAGAGTATCCGGATCGTATTATGTGCACGTACTCCGTGTGTCCGTCCCCCAAGGTGTCGGACACTGTCGTCGAGCCCTACAACGCTACGTTGTCGGTGCATCAGCTCGTGGAGAACGCTGATGAAGTCATGTGCCTTGACAATGAGGCCCTGTATGACATTTGCTTCCGCACGCTAAAGCTCACGACTCCTACTTACGGTGATCTGAATCACTTGGTGTGTGCTGCTATGTCTGGTATCACGACGTGTCTACGTTTTCCGGGTCAGCTGAACTCAGACCTCCGCAAGCTGGCCGTGAACTTGATTCCGTTTCCACGTCTTCACTTCTTCATGATTGGGTTCGCTCCGCTGACGTCGCGAAGCTCGCAGCAGTACCGTGCGTTGACGGTGCCCGAGCTGACGCAGCAGCAGTTCGATGCTAAAAACATGATGTGTGCCGCCGACCCTCGCCATGGCCGCTATTTAACTGCCGCGTGTATGTTCCGCGGACGTATGAGTACGAAGGAGGTGGATGAGCAAATGCTGAACGTGCAGAACAAGAACTCGTCGTACTTCGTCGAGTGGATTCCCAACAACATCAAGGCTAGCGTGTGTGACATCCCGCCCAAGGGGTTGAAGATGAGTACCACCTTTATCGGTAACTCGACCGCAATCCAGGAGATGTTCAAGCGCGTGTCTGAGCAATTTACGGCCATGTTTCGTCGTAAGGCTTTCTTGCACTGG

Hyaloperonospora_brassicae TTTTTTGATTGTTATTACAGTATTTGTTTGTTGGATGTTATTTAGAGTTATTACTCTTTTTAATG-AAAAAAAAATAAAATTCCTGCAACTGTTGTTCATGGTGCTACTATTGAAATTATTTGGACGTCTATTCCGGCTTTAATTTTATTAATTGTTGCAATACCTTCATTTGCTTTATTATATTCAATGGATGAAGTAATTGATCCTATTATTACTTTAAAAGTAATTGGAAGTCAATGGTATTGGAGTTATGAATATTCAGATAATTTAGAATTTTCGGATGAACCTCTAATTTTTGATAGTTATATGATACAAGAAGATGATTTAACAATAGGTCAATTTAGACTTTTAGAAGTAGATAATCGCGTAATAGTACCTACTAATAGTCATATTCGAGTATTAATTACGGCTTCAGATGTATTACATTCGTGGGCTATTCCTTCACTAGGTATTAAATTAGATGCATGTCCTGGACGTTTAAATCAAACCGGCGAGTGAAGCGGGATGAGCTCAAGCTTAAAATCTCTGTCACAATTTTGTGCGGCGAATTGTAGTCTATAGAGGC-GTGATCAGTGTAGGTACTTGAGATAAGTTTCTTGAAAAAGAACAGCATGGAGGGTGAAACTCCCGTTCATA-CTTAAGTGGCTTGCACGTACGATCCGTTTTCTTTGAGTCGCGTTGTTTGAGAATGCAGCGCAAAGTAGGTGGTAAATTCCATCTAAAGCTAAATATTGGTGCGAGACCGATAGCGAACAAGTACCGTGAGGGAAAGATGAAAAGAACTCTGAAAAGAGAGTTAAAGAGTACCTGAAACTGCTGAAAGGGAACCGAATCGTTTCTAGTGTCTATAATCTATGACATATTTCATTGGGAAGTATGTGCGTGTGTATACTATGGCAGCGGCTTTTTTTGAGCTGCGCTTGGTGTG---CG-TGCTGTATGTGCTAACTGGTGCCCTGTGTTATAGTGGGACGTCAAGGTCAGTTTGTATGCTGCGGGAAATGGCTACTAGGGAGGTAGGGCTTACGCT-TGCGTTTGTCTATTATATCTTGGTAGTTG----TGTCGTGGTTGAGACTGAGGTGTTCTACAACGTGCTTTTGAGTGGGTCTGTGTCTCCGTGTGCGCCGTGTGCAGATAGCTTGCTATGTGTGTGT---GGTGGTGTGTGGATTGATGTGGGCTTTAACTTGTCGCCGTTCACTCGGGCATTGGCATGACGAAGGCCGACCTCATTAACAACTTGGGCACGATCGCCAAGTCGGGCACGAAGGCGTTCATGGAGGCGCTGGCGGCCGGCGCCGACATTAGCATGATTGGCCAGTTTGGCGTGGGCTTTTACTCGGCCTACTTGGTGGCCGACAAGGTCGTGGTGCACTCGAAGCACAATGACGACGAGCAGTACGTGTGGGAGTCGGCGGCCGGCGGCTCGTTTACTGTCACGCCCGAC---ACGTCGGAGCCGATTCTGCGCGGCACGCGCATTGTGTTGAAGCTCAAGGAGGACATGCTCGAGTACTTGGAGGAGCGCAAGCTCAAGGACCTGGTGAAGAAGCACTCGGAGTTTATCGGCTTTCCCATCAAGTTGTACGTGGAGAAGACGGAGGAGAAGGAGGTGACGGACGACGAGGAGGAGGAGGACG---AGAAGGAGGGCGAGGAGGACAAGCCCAAGGTTGAGGAGGTGGAGGACGAGGAGGAGGGCGAGAAGAAGAAGAAGACGAAGAAGATTAAGGAGGTGACGCACGAGTGGAACCACTTGAACAGCCAGAAGCCCATCTGGATGCGCAAGCCGGAAGACGTGACGCACGAGGAGTATGCGTCGTTCTACAAGTCGCTGACGAACGACTGGGAGGAGCACGCGGGCGTCAAGCACTTTTCGGTGGAGGGTCAGTTGGAGTTCAAGGCGTGTCTGTTTACGCCCAAGCGCGCGCCGTTCGACATGTTTGAAGGCGGTGCTAAGAAGAAACTGAACAACATTAAACTGTACGTGCGTCGCGTGTTCATTATGGACAACTGCGAGGACCTCATGCCCGAGTACCTGTCGTTCGTCAAGGGTGTGGTCGACTCGGAGGACTTGCCGCTGAACATCTCGCGTGAGACGCTGCAGCAGAACAAGATCCTGCGTGTGATCAAGAAGAACCTGGTGAAGAAGTGTCTCGAGATGTTTGCCGAGCTCGCCGAGGACAATGAGAAGTACAACAAGTTCTACGAGGCGTTCAGCAAGAACCTG-AAGCTGGGCATCCACGAGGACTCGACGAACCGCACCAAGATCGCCAAGCTGCTGCGCTACCACTCGACCAAGTCGGGCGAAGAGGTGACGTCGCTTGACGACTACATCACGCGCATGCCCGAGAGCCAGCCGGGTATCTACTACGTGACGGGCGAGAGCAAGAAGGCGGTGGAGAACTCGCCGTTCATCGAGAAGCTCAAGAAGAAGGGTTACGAAGTGCTGTTTATGGTGGATGCCATTGACGAGTACGCTGTGCAGCAGCTCAAGGAGTACGAGGGCAAGAAGCTGATCTGCGCCACGAAGGAGGGCCTCAAGATGGAGGAGTCGGAGGACGAGAAGAAGTCGTTCGAGGAAGCCAAAGCCGCTACGGAGGGACTTCACGGTCATTGACGCGCCGGGACACCGTGACTTTATCAAGAACATGATTACCGGCACGTCGCAGGCCGACTGCGCGATTCTGGTCGTGGCCTCGGGCGTGGGCGAGTTCGAAGCGGGTATCTC---GAAGGAGGGCCAGACGCGCGAGCACGCGCTGCTGGCGTTCACGCTGGGCGTGAAGCAGATGGTCGTGGCCATTAACAAGATGGACGACTCGTCGGTCATGTACGGCCAGGCCCGGTACGAGGAGATCAAGTCGGAGGTGACGACGTACTTGAAGAAGGTGGGCTACAAACCCGCCAAGATTCCGTTCGTGCCGATCTCGGGCTGGGAGGGCGACAACATGATTGAGCGCTCGCCCAACATGGCGTGGTACAAGGGCCCGTACCTGCTCGAGGCGCTGGACAACCTGAACCCGCCCAAGCGTCCGTCGGACAAGCCGCTGCGGCTGCCTCTCCAGGACGTGTACAAGATTGGCGGTATCGGCACGGTACCGGTGGGGCGTGTCGAGACGGGTGTGATCAAGCCGGGTATGGTCGCGACGTTTGGCCCCGTGGGTCTGTCGACGGAAGTCAAGTCGGTCGAGATGCACCACGAGTCGCTGCCCGAGGCGCTGCCGGGTGACAATGTCGGCTTTAACGTGAAGAACGTGTCGGTGAAGGAGCTGCGTCGTGGGTTTGTGGCGTCGGACTCGAAGAACGA---CCCGGCCAAGGGCACGCAGGACTTCACGGCGCAGGTGATTGTGCTGAACCACCCTGGCCAGATTTGCAACGGCTACTCGCCCGTGCTGGACTGCCACACGGCGCACGTTGCGTGCAAGTTCAAAGAGATTATCGAGAAGATGGACCGTCGTTCGGGCAAGGTGCTCGTCCAGAACAAGAAGCCGTTCATCAAGTCCCGCTACTGCCGCGGCGTGCCGGACCCCAAGATCCGCATCTACGACGTGGGCAACAAGAAGGCGTCGGTCGACCTCTTCCCCTTTGTGGCGCACTTGGTCTCGGACGAGAAGGAGCAGTTGTCGTCCGAGGCGCTCGAAGCGGCGCGCATTGCCGCGAACCGGTACCTGACCAAGTACTGCGGCAAAGACAACTTCCACATGCGCATCCGTTGCCACCCGTTCCAAGTGCTGCGTATCAACAAAATGCTTTCGTGCGCTGGTGCCGATCGACTGCAGACGGGTATGCGTCACGCCTACGGCAAACCGGCGGGCGTGGCTGCGCGCGTGTCCATTGGTCAGCCCATTATCTCAGTGCGCTCGAAGGACTCGTTCGGTCCGTCGGTCGTTGAAGCGCTGCGTCGTGCAGGCAGCTGACGCCGACAAGCTCTCGGCCGACAAGCTACTGGAGGTCTACGAGGGCTTTATTGCCACGTGTGCGGCCTCGAGCAGCATCGTGTCGATCGAGGACCCGTTTGACCAGGACGACTGGGCCTCGTGGGTCAAGATCACGAGCAAAGTAGGCAAAGACGTGCAGATTGTGGGCGACGATTTGACCGTCACGAACCCCACGCGTGTGAAGAAGGCGATCGAACAGAAGGCGTGCAATGCGCTTCTGCTCAAGGTGAACCAGATTGGCAGCATCACCGAGTCGATTGAGGCCGTGACGATGGCGAAGAAGGCCGGATGGGGCATCATGGCCAGCCACCGCAGCGGCGAGACTGAAGACACGTTCATTGCAGACCTGGCAGTGGGTCTGAGCGCGGGCCAGATGGTAACAACTGGGCCAAGGGACACTATACGGAGGGTGCGGAGCTCATTGATTCGGTGCTGGACGTTGTCCGTAAAGAGGCCGAGAGCTGTGACTGCCTACAGGGTTTCCAGTTCACGCACTCGCTTGGCGGTGGTACTGGCTCGGGTATGGGCACGCTTCTTATCTCGAAGATCCGCGAGGAATACCCGGATCGCATCATGTGCACGTACTCCGTGTGCCCGTCCCCGAAGGTGTCGGACACGGTGGTGGAGCCCTACAATGCGACGCTGTCGGTGCACCAGCTCGTTGAAAACGCTGACGAAGTCATGTGCCTGGACAACGAGGCCCTGTACGACATTTGCTTCCGTACGCTGAAACTCACCACCCCTACGTATGGTGACCTGAACCACTTGGTGTGCGCTGCTATGTCTGGCATCACGACGTGTCTACGTTTTCCCGGCCAGCTGAACTCCGACCTGCGTAAGCTAGCCGTGAACCTGATTCCGTTTCCACGTCTCCACTTCTTCATGATCGGATTCGCTCCGCTGACGTCGCGAAGCTCGCAGCAGTACCGTGCGCTGACGGTGCCCGAGCTCACGCAGCAGCAGTTTGATGCGAAGAACATGATGTGCGCTGCAGACCCTCGCCATGGCCGTTATTTGACCGCCGCGTGTATGTTCCGCGGTCGCATGAGTACGAAGGAGGTGGACGAGCAAATGCTGAACGTGCAGAACAAGAATTCGTCGTACTTCGTCGAGTGGATTCCGAATAACATTAAGGCTAGCGTGTGCGACATCCCGCCCAAGGGCCTGAAGATGAGTACGACGTTTATCGGCAACTCGACCGCGATCCAGGAGATGTTCAAGCGTGTGTCTGAACAGTTTACGGCTATGTTTCGTCGTAAGGCTTTCTTGCACTGG

Plasmopara_halstedii TTTTTTAATTATGATCACTGTATTTGTTTGTTGGATGTTATTTAGAGTTGTTACTCTTTTTGATGAAAAAAAAAATAAGGTTCCATCAACTGTTGTACATGGTGCAACTATTGAAATTATATGGACCACGATCCCCGCTTTAATTTTATTAATTATCGCAATACCTTCATTCGCATTATTATATTCTATGGATGAAATAATTGACCCGATTATTACATTAAAAGTAATTGGTAGCCAATGGTATTGGAGTTATGAATACTCAGATAATTTAGAATTTTCAGATGAACCTTTAATTTTTGATAGTTATATGGTACAAGAAAATGATTTAGAAATAGGACAATTTAGACTTTTAGAAGTTGATAAYCGTGTGGTAGTTCCAGTTAACAGTCATATTCGTGTATTAATTACCGCATCCGATGTTTTACATTCTTGGGCAATACCTTCATTAGGTATAAAATTAGATGCGTGTCCAGGCCGTTTAAATCAAACCGGCGAGTGAAGCGGGATGAGCTCAAGCTTAAAATCTCCATACAATTTTTGTATGGCGAATTGTAGTCTATAGAAGC-GTGGTCAGTGTAGGCACTTGGGGTAAGTTCCTTGGAAAAGGACAGCATGGAGGGTGATACTCTCGTTTATC-TCTAAGTTGCTTATACGTACGACCCGTTTTCTTTGAGTCGCGTTGTTTGAGAATGCAGCGCAAAGTAGGTGGTAAATTCCATCTAAAGCTAAATATTGGTGCGAGACCGATAGCAAACAAGTACCGTGAGGGAAAGATGAAAAGAACTTTGAAAATAGAGTTAAAGAGTACCTGAAACTGCTGAAAGGGAACCAAATCGTTTCCAGTGTCTATAATCCGTGGTATATCACATCGGCGAGCGTGTGTGTGTGTGTGCTGTTGTAGTGGCCTTTT---GGTTACGCTCGGCGTA---TG-TGCTGTATATGCTTGCTGGTGCCCTGTACTGCGGTGGGACGTCAAGGTCAGTTCGTATGCTGCGGGAAATGACTACCAAGGAGGTAGGGCTTACGCT-TGCGTTTGTCTGTTATATCTTGGTAGACG-AGTAGTCGCGGTTGGGACTGAGGTG-CCTACAACGTGCTTTTGAGTGGGCTTGGGTCTTCGTGTGCGCCGTGTGCGGATAGCTTGCTATGCGTGTGT---GGTTGTGTATGGATTGATGCGAGCCTTAACTTGTAGTCGTTCACTCAGGTATCGGCATGACAAAGGCTGACTTGATTAACAACCTTGGTACGATTGCCAAGTCCGGTACTAAGGCGTTTATGGAGGCATTGGCCGCCGGCGCTGACATCAGTATGATCGGTCAGTTCGGTGTGGGTTTCTACTCAGCTTATCTCGTCGCGGACCAGGTGGTTGTCCACTCGAAACACAACGACGATGAGCAGTATGTATGGGAGTCTGCTGCCGGTGGCTCGTTCACAGTGACACCTGAC---ACGTCCGAGCCGATTCCACGTGGTACGCGTATTGTGTTGAAACTCAAGGAGGACATGCTTGAATACCTTGAGGAACGCAAGCTGAAAGACTTGGTTAAGAAGCACTCAGAGTTCATTGGCTTCCCAATCAAGCTTTACGTCGAGAAGACGGAAGAAAAAGAAGTGACGGACGACGAAGAGGAGGAGGATG---AGAAGGAAGGTGATGACGACAAGCCTAAGGTCGAAGAGGTCGAA---GAAGAAGAAGGCGAGAAGAAGAAAAAGACCAAAAAGATTAAAGAGGTGACGCATGAGTGGGACCATCTAAACAGCCAGAAGCCAATCTGGATGCGCAAGCCCGAAGACGTGACTCACGAGGAGTATGCTTCGTTTTACAAGTCGCTGACCAACGACTGGGAAGAACACGCAGCTGTTAAGCACTTTTCAGTTGAAGGACAGCTCGAGTTCAAGGCTTGTCTTTTCACTCCCAAGCGTGCGCCGTTTGACATGTTTGAAGGTGGCGCAAAGAAGAAACTTAACAACATCAAGTTGTACGTGCGTCGCGTGTTCATCATGGACAACTGCGAGGAGCTTATGCCGGAGTATCTGTCCTTCGTTAAGGGTGTCGTTGATTCGGAAGATTTGCCACTAAACATTTCGCGTGAAACTCTGCAACAAAACAAGATCTTGCGCGTGATCAAGAAGAATTTGGTAAAGAAGTGCCTGGAAATGTTTGCCGAGTTAGCTGAAGACAATGAGAAGTACAACAAATTCTACGAGGCGTTCAGCAAGAATCTC-AAGCTAGGCATCCACGAGGATTCAACAAACCGTTCCAAGATTGCTAAATTGCTTCGCTACCACTCGACCAAATCTGGGGAGGAGTTGACGTCACTGGACGACTACATTTCCCGCATGCCAGAGAACCAGCCTGGTATCTACTATGTCACGGGCGAGAGCAAGAAAGCGGTTGAGAACTCTCCGTTCATTGAGAAGCTCAAAAAGAAAGGCTACGAGGTCTTGTTCATGGTTGAAGCCATTGATGAGTACGCTGTGCAGCAGCTAAAGGAGTACGAGGGCAAAAAGCTTATCTGCGCCACTAAGGAAGGTCTCAAGATGGAGGAGACTGAAGATGAGAAGAAGTCCTTCGAGGAAGCTAAAGCCGCCACGGAAGGCTTTCACGGTCATTGACGCCCCTGGACACCGTGACTTTATCAAGAACATGATCACGGGCACATCCCAGGCCGATTGTGCTATTCTTGTGGTTGCTTCGGGTGTTGGCGAATTTGAGGCTGGTATCTC---GAAGGACGGTCAGACACGTGAGCACGCTCTGCTTGCTTTCACCCTTGGTGTGAAGCAGATGGTTGTTGCTATTAACAAGATGGACGACTCCTCTGTTATGTACGGCCAATCTCGTTACGAGGAAATTAAGGCCGAGGTTACCACCTACCTGAAGAAGGTTGGCTACAAGCCCGCCAAGATCCCTTTCGTGCCTATTTCTGGATGGGAGGGTGACAACATGATCGACCGTTCGGCTAACATGCCATGGTACAAGGGACCATACCTTCTCGAGGCCCTTGACAACTTGAACCCACCCAAGCGTCCAAGTGACAAGCCTCTTCGTCTGCCTCTTCAGGACGTTTACAAGATCGGCGGTATTGGCACGGTTCCGGTCGGTCGTGTCGAGACCGGAGTTATTAAACCCGGTATGGTTGCTGTATTTGGCCCCACTGGTCTATCTACTGAAGTTAAGTCCGTTGAAATGCACCACGAGTCTTTGCCAGAGGCTGTGCCTGGTGACAACGTTGGTTTCAACGTAAAGAACGTATCAGTAAAGGAACTTCGTCGTGGTTTCGTGGCTTCGGACTCGAAGAACGA---CCCTGCCAAGGGCACCCAGGACTTCACTGCTCAGGTCATTGTGTTGAACCACCCTGGTCAGATTGGCAACGGTTACTCGCCTGTGCTTGATTGCCACACGGCCCACGTTGCTTGCAAGTTCAAAGAGATTACGGAGAAGATGGACCGTCGTTCGGGCAAGGTTCTTGTCCAAAACAAGAAGCCGTTTATTAAGTCGCGCTTCTGCCGTGGTGTTCCTGACCCGAAGATTCGCATCTACGATGTGGGTAACAAGAAGGCCTCGGTGGACGCCTTTCCATACGTAGCTCACCTTGTGTCCGACGAAAAGGAGCAGTTGTCATCAGAAGCCCTCGAGGCTGCTCGTATTGCTGCCAATCGATACTTAACCAAGTTTTGCGGCAAGGACAACTTTCATATGCGTATTCGCTGTCACCCATTCCAGGTCTTGCGTATCAACAAAATGCTTTCGTGTGCTGGTGCTGATAGGCTGCAGACTGGTATGCGTCACGCCTACGGTAAGCCTGCTGGTGTGGCTGCTCGCGTGGCAATTGGTCAGCCAATTATCTCGGTTCGATCTAAAGATTCATTTGGCCCCTCTGTTGTGGAGGCGTTGCGTCGTGCATGCTGCGGAATCTGAGAAATTTACGGCCGACAAGTTGCAGGAGGTATACGAAGGCTTTATTGCTAAGTGTGCTAACTCTAGTAAGATCGTATCAATTGAGGACCCATTTGACCAGGACGACTGGGAGTCATGGGTTCAGTTTACGGCTAAAGTCGGCGAAAGCGTGCAGATTGTTGGCGATGACCTTACGGTGACGAATCCGACCCGTGTGAAAAAGGCCATTGAAAAAAAGGCTTGCAACGCTCTTTTGCTCAAGGTGAACCAAATTGGCAGTATAACGGAATCGATCGAAGCCGTGACGATGGCCAAGAAAGCAGGCTGGGCAATTATGGCCAGTCACCGTAGTGGTGAGACTGAGGACACGTTCATTGCAGATTTGGCAGTAGGCTTGAGCGCCGGCCAGATGGTAACAATTGGGCGAAAGGACATTATACCGAGGGTGCCGAGCTGATTGATTCCGTGCTAGACGTTGTCCGCAAGGAAGCAGAAAGTTGCGATTGTCTTCAAGGTTTTCAAATTACGCACTCACTTGGTGGTGGTACCGGCTCCGGAATGGGCACACTTCTTATTTCGAAAATTCGGGAAGAGTATCCCGACCGAATCATGTGCACCTACTCGGTGTGCCCATCGCCTAAGGTATCAGATACGGTAGTAGAGCCTTACAATGCCACATTGTCGGTGCACCAGCTAGTCGAGAACGCTGATGAAGTTATGTGCTTAGACAACGAAGCTTTGTACGACATTTGCTTTCGTACGTTAAAACTCACCACGCCTACTTACGGTGACTTGAATCACTTGGTGTGTGCTGCCATGTCTGGTATTACCACATGCTTGCGATTTCCTGGTCAGCTAAATTCAGACTTGCGGAAGTTGGCCGTCAATCTGATTCCATTTCCCCGTCTTCATTTTTTTATGATTGGTTTTGCACCTTTGACATCACGTGGCTCGCAGCAGTATCGTGCGTTGACAGTACCTGAGTTGACCCAGCAGCAGTTTGATGCTAAAAACATGATGTGCGCCGCTGACCCTCGCCACGGTCGCTATTTAACTGCCGCGTGTATGTTTCGAGGGCGCATGAGCACGAAGGAAGTTGATGAGCAGATGCTGAATGTGCAAAACAAAAATTCTTCTTATTTCGTCGAGTGGATTCCCAACAACATCAAGGCGAGTGTGTGTGACATCCCGCCTAAGGGTTTAAAAATGAGCACCACGTTTATTGGTAATTCGACTGCTATTCAGGAGATGTTTAAGCGAGTGTCAGAACAGTTCACGGCAATGTTCCGTCGTAAGGCTTTCTTGCATTGG

Plasmopara_muralis TTTTTTAATTATGATAACTGTATTCGTTTGTTGGATGTTATTTAGAGTTGTTACTCTTTTTGACGAAAAAAAAAACAAAACACCCGCGACCGTTGTACATGGTGCAACTATTGAAATTATTTGGACTTCTATTCCTGCTTTAATTTTATTAATTATTGCTATACCATCGTTTGCATTATTGTATTCTATGGATGAAATAATTGATCCTATTATTACTTTAAAAGTAATTGGAAGTCAATGGTATTGGAGTTATGAATATTCTGATAATTTAGAATTTTCAGATGAGCCTTTAATTTTTGATAGCTATATGGTTCCGGAAAGTGATTTAGCAATAGGTCAATTTAGATTATTAGAAGTTGATAATCGTGTAATAGTTCCAACTAATAGTCATATTCGTGTATTGATTACTGCGTCAGATGTTTTACACTCATGGGCGATACCTTCATTGGGAATAAAATTAGATGCATGTCCGGGCCGCTTAAATCAAACCGGCGAGTGAAGCGGGATGAGCTCAAGCTTAAAATCTCCGTGCAATTTTTGCGCGGCGAATTGTAGTCTATAGAAGC-GTGGTCAGTATGGGCACTTGGGTTAAGTTCCTTGGAAGAGGACAGCATGGAGGGTGATACTCCCGTTCATC-CCTAAGTTGCTTGTGCGTACGACCCGTTTTCTTTGAGTCGCGTTGTTTGAGAATGCAGCGCAAAGTAGGTGGTAAATTCCATCTAAAGCTAAATATTGGTGCGAGACCGATAGCATACAAGTACCGTGAGGGAAAGATGAAAAGAACTTTGAAAATAGAGTTAAAGAGTACCTGAAACTGCTGAAAGGTAACCAAATCGTTTCCAGTGTCTATAATCCGTGGTATATTTCATTGGCGAGCGTATGCGTGCGTGTGCTGTTGCAGTGGCCTTTTT--GGCTGCGCTCGGCGTG---TG-TGCTGTGTGTGCTTGCTGGTGCCCTGTGCTATGGTGGGACGTCAAGGTCAGTTCGTATGCTGCGGGAAATGGCCACTGAGGAGGTAGGGCTTACGCT-TGCGTTTGTCTGTTATATCTTGGTGGACG-AGTAGTCGCGGTTGGGACTGAGGTG-CCTACAACGTGCTTTTGAGTGGATTTGGGTCTCCGTGTGCGCCGTGTGCGGATAGCTTGCTATGCGTGTGT---GGTTGTGTATGGATTGATGCGAGTCGTAACTTGTAGCCGTTCACTCGGGTATTGGTATGACTAAAGCCGACCTGATTAACAACCTTGGTACGATCGCCAAGTCGGGTACTAAGGCCTTTATGGAGGCCTTGGCCGCTGGAGCTGACATCAGTATGATTGGTCAGTTTGGTGTGGGTTTCTACTCTGCGTACCTTGTCGCAGACCAGGTGGTGGTCCACTCTAAGCACAACGACGACGAGCAGTATGTATGGGAATCTGCTGCTGGTGGATCGTTCACAGTGACCCCCGAC---ACGTCGGAGCCGATTCTTCGCGGCACGCGCATTGTGCTTAAGCTGAAGGAGGACATGCTTGAGTACCTGGAGGAACGTAAGCTCAAAGACTTGGTGAAGAAACACTCCGAGTTCATTGGCTTCCCTATCAAACTCTACGTTGAGAAGACAGAGGAAAAGGAAGTGACGGATGACGAGGAAGAAGAAGACG---AGAAGGAAGGCGACGATGACAAGCCTAAGGTCGAGGAAGTTGAG---GAAGAGGAAGGCGAAAAGAAGAAAAAGACGAAGAAGATCAAGGAAGTGACCCATGAGTGGGACCACATTAATAGTCAGAAGCCCATCTGGATGCGCAAGCCTGAAGACGTAACTCACGAGGAGTACGCTTCGTTCTACAAGTCGCTGACCAACGATTGGGAGGAACATGCCGCTGTAAAACACTTCTCAGTTGAAGGACAGCTCGAGTTCAAGGCGTGTCTGTTTACGCCGAAGCGCGCGCCGTTCGACATGTTTGAAGGTGGCGCCAAAAAGAAACTCAACAACATCAAGTTGTACGTGCGTCGCGTGTTCATTATGGACAATTGCGAGGAGTTGATGCCTGAGTACCTGTCGTTCGTCAAGGGTGTTGTTGATTCGGAAGACTTGCCGCTTAACATTTCACGTGAAACGCTGCAACAAAACAAGATCTTGCGCGTGATTAAGAAAAACTTGGTCAAGAAGTGCCTCGAAATGTTTGCCGAGCTTGCTGAAGACAATGAGAAGTACAACAAGTTCTACGAGTCTTTCAGCAAGAACCTC-AAACTTGGTATTCATGAGGACTCGACCAACCGCACGAAGATTGCCAAGCTGCTACGCTACCACTCCACCAAGGCGGGCGAAGAACTCACGTCACTGGACGACTACATTTCTCGAATGCCGGAGAACCAGCCTGGTATTTACTACGTGACGGGCGAAAGCAAGAAGGCGGTTGAAAACTCACCGTTTATTGAAAAGCTTAAGAAGAAGGGCTACGAGGTCTTGTTCATGGTTGAGGCTATTGACGAGTACGCTGTGCAGCAGTTGAAGGAGTATGAGGGTAAGAAGCTCATCTGCGCCACGAAGGAAGGCCTTAAAATGGAGGAGACGGAAGACGAGAAGAAGTCGTTCGAGGAAGCCAAAGCTGCCACGGAGGGACTTCACGGTCATTGACGCACCTGGTCACCGTGACTTCATTAAAAACATGATCACGGGCACGTCACAGGCCGATTGCGCCATTCTCGTGGTTGCTTCGGGTGTGGGTGAATTTGAGGCGGGTATCTC---TAAGGAAGGCCAGACTCGAGAGCACGCATTGCTCGCCTTTACGCTTGGAGTGAAGCAGATGGTCGTTGCTATCAACAAGATGGACGACTCCTCCGTCATGTACGGCCAGGCCCGCTACGAAGAGATTAAGGCGGAAGTCACTACGTACCTGAAGAAGGTGGGGTACAAGCCTGCGAAGATTCCGTTCGTGCCCATTTCTGGATGGGAAGGAGACAATATGATTGACCGTTCGCCCAACATGGCGTGGTACAAGGGTCCCTACCTTCTTGAAGCCCTTGACAATTTGAACGCTCCCAAGCGTCCAAGTGACAAGCCTCTGCGTTTGCCGCTTCAGGATGTTTACAAGATTGGCGGTATTGGCACGGTCCCGGTCGGCCGTGTCGAGACGGGTATTATCAAGCCTGGTATGATTACCACGTTCGGTCCCACCGGACTGTCAACAGAAGTCAAGTCTGTTGAAATGCACCACGAGTCTCTGCCAGAGGCTGTCCCTGGTGACAACGTTGGCTTCAACGTCAAGAATGTGTCAGTCAAGGAGCTGCGTCGTGGTTTCGTGGCTTCGGATTCGAAGAATGA---CCCTGCTAAGGGCACACAGGACTTCACGGCCCAAGTCATTGTTTTGAATCACCCTGGCCAGATTTGCAATGGTTACACTCCTGTGCTGGACTGTCACACATGCCACGTTGCCAGCAAGTTTAAAGAGATTACGGAGAAGATGGACCGTCGTTCCGGCAAGGTGCTTGTCCAGAACAAGAAGCCGTTTATCAAGTCGCGCTTCTGCCGTGGCGTGCCTGACCCGAAGATTCGCATCTACGATGTGGGTAACAAAAAGGCTTCGGTGGACGCCTTCCCATACGTGGCTCACCTCGTGTCCGACGAAAAGGAGCAGTTGTCATCGGAGGCTCTTGAGGCTGCTCGTATTGCTGCCAACCGATATTTGACTAAATTTTGCGGCAAAGACAACTTTCACATGCGTATTCGTTGCCACCCTTTTCAGGTTCTCCGTATCAACAAAATGCTTTCGTGCGCTGGTGCCGATAGGCTACAGACCGGTATGCGTCACGCTTACGGTAAGCCTGCCGGTGTGGCCGCTCGCGTGGCCATCGGTCAGCCTATTATTTCGGTACGCTCCAAAGACTCGTTTGGTCCCTCAGTTGTGGAGGCTTTGCGCCGTGCGTGGTGCGGACTCGGAGAAGATCTCGGCCGACAAGTTGCAGGAGGTGTATGAGGGCTTTATTGCCAAGTGTGCTGACTCTAGCAAGATCGTGTCCATTGAAGACCCATTTGATCAGGATGACTGGGAGTCGTGGGTTAAGTTCACGGCGAAGGTTGGCGAAGACGTCCAGATTGTCGGCGACGACCTGACGGTGACGAACCCTACTCGTGTGAAAAAGGCCATTGAACAGAAGGCGTGCAATGCTCTTTTGCTTAAAGTGAACCAGATTGGCAGCATTACCGAGTCGATTGAGGCCGTGACGATGGCCAAGAAGGCTGGCTGGGCTATCATGGCCAGCCATCGTAGTGGTGAGACTGAAGACACCTTCATTGCAGACCTGGCGGTGGGCTTGAGTGCCGGTCAGGTGGTAACAATTGGGCCAAAGGACACTATACCGAGGGTGCTGAGCTGATTGATTCTGTGCTGGACGTCGTCCGGAAAGAGGCAGAAAGCTGTGACTGTCTCCAAGGTTTTCAAATTACGCACTCGCTTGGGGGCGGTACCGGCTCTGGAATGGGCACACTTTTGATCTCGAAAATTCGTGAAGAGTATCCTGACCGAATCATGTGCACCTACTCAGTTTGCCCGTCGCCCAAGGTATCTGACACGGTCGTTGAGCCCTACAATGCAACGCTGTCCGTGCATCAGCTAGTTGAAAATGCAGACGAGGTCATGTGCCTAGATAATGAAGCATTGTACGACATTTGCTTTCGTACCCTGAAACTTACCACTCCCACGTACGGCGATCTGAATCACTTGGTTTGTGCCGCCATGTCTGGCATAACCACATGCCTTCGATTTCCCGGACAATTGAATTCGGACTTACGAAAGTTGGCTGTTAATCTGATTCCGTTCCCCCGTCTTCATTTCTTTATGATTGGATTTGCACCACTGACATCGCGTGGCTCGCAGCAGTATCGTGCGTTGACAGTACCTGAGTTGACCCAGCAGCAGTTCGATGCGAAGAATATGATGTGCGCCGCTGACCCTCGCCACGGCCGCTATTTAACAGCCGCGTGTATGTTCCGCGGACGTATGAGCACGAAGGAGGTTGATGAGCAGATGCTGAACGTGCAGAACAAAAACTCCTCGTACTTTGTTGAATGGATTCCCAACAATATTAAGGCTAGTGTGTGTGACATTCCACCCAAGGGTTTGAAAATGAGCACTACGTTTATTGGCAACTCGACGGCTATTCAAGAAATGTTCAAGCGAGTGTCCGAACAGTTTACAGCTATGTTCCGTCGCAAGGCTTTCTTGCACTGG

Plasmopara_viticola TTTTTTAATTATGATAACTGTATTCGTTTGTTGGATGTTATTTAGAGTTGTTACTCTTTTTGACGAAAAAAAAAACAAAACACCCGCGACCGTTGTACATGGTGCAACTATTGAAATTATTTGGACTTCTATTCCTGCTTTAATTTTATTAATTATTGCTATACCATCGTTTGCATTATTGTATTCTATGGATGAAATAATTGATCCTATTATTACTTTAAAAGTAATTGGAAGTCAATGGTATTGGAGTTATGAATATTCTGATAATTTAGAATTTTCAGATGAGCCTTTAATTTTTGATAGCTATATGGTTCCGGAAAGTGATTTAGCAATAGGTCAATTTAGATTATTAGAAGTTGATAATCGTGTAATAGTTCCAACTAATAGTCATATTCGTGTATTGATTACTGCGTCAGATGTTTTACACTCATGGGCGATACCTTCATTGGGAATAAAATTAGATGCATGTCCGGGCCGCTTAAATCAAACCGGCGAGTGAAGCGGGATGAGCTCAAGCTTAAAATCTCCGTGCAATTTTTGCGCGGCGAATTGTAGTCTATAGAAGC-GTGGTCAGTATGGGCACTTGGGTTAAGTTCCTTGGAAGAGGACAGCATGGAGGGTGATACTCCCGTTCGTC-CCCAAGTTGCTTGTGCGTACGACCCGTTTTCTTTGAGTCGCGTTGTTTGAGAATGCAGCGCAAAGTAGGTGGTAAATTCCATCTAAAGCTAAATATTGGTGCGAGACCGATAGCATACAAGTACCGTGAGGGAAAGATGAAAAGAACTTTGAAAATAGAGTTAAAGAGTACCTGAAACTGCTGAAAGGTAACCAAATCGTTTCCAGTGTCTATAATCCGTGGTATATTTCATTGGCGAGTGTATGCGTGCGTGTGCTGTTGCAGTGGCCTTTTT--GGCTGCGCTCGGCGTG---TG-TGTTGTGTGTGCTTGCTGGTGCCCTGTGCTGTGGTGGGACGTCAAGGTCAGTTCGTATGCTGCGGGAAATGGCCACTGAGGAGGTAGGGCTTACGCT-TGCGTTTGTCTGTTATATCTTGGTGGACG-AGTAGTCGCGGTTGGGACTGAGGTG-CCTACAACGTGCTTTTGAGTGGATTTGGGTCTCCGTGTGCGCCGTGTGCGGATAGCTTGCTATGCGTGTGT---GGCTGTGTATGGATTGATGCGAGTCGTAACTTGTAGCCGTTCACTCGGGTATTGGTATGACGAAAGCCGACCTAATTAACAACCTTGGTACGATCGCCAAGTCGGGTACTAAGGCCTTCATGGAGGCTTTGGCCGCCGGTGCCGACATCAGTATGATTGGTCAGTTTGGTGTGGGTTTCTACTCTGCGTACCTTGTCGCAGACCAGGTGGTGGTCCACTCGAAGCACAACGACGACGAGCAGTATGTATGGGAATCTGCTGCCGGTGGATCGTTCACAGTGACCCCCGAC---ACGTCGGAGCCGATTCTTCGCGGTACGCGCATTGTGCTAAAGCTAAAGGAGGACATGCTTGAGTACCTGGAGGAACGTAAGCTCAAAGACTTGGTGAAGAAACACTCCGAGTTCATTGGCTTCCCTATCAAGCTCTACGTCGAGAAGACGGAGGAGAAGGAAGTGACGGATGACGAGGAAGAGGAAGACG---AAAAGGAAGGTGACGACGACAAGCCTAAAGTCGAGGAAGTCGAG---GAAGAGGAAGGCGAAAAGAAGAAGAAGACGAAGAAGATCAAGGAAGTGACCCATGAGTGGGACCACATCAACAGTCAGAAGCCCATCTGGATGCGCAAGCCTGAAGACGTAACTCACGAGGAGTACGCTTCGTTCTACAAGTCGCTGACCAACGATTGGGAGGAACATGCCGCTGTGAAACACTTTTCAGTCGAAGGACAGCTCGAGTTTAAGGCGTGTCTGTTTACGCCGAAGCGCGCGCCGTTTGACATGTTTGAGGGTGGCGCCAAAAAGAAACTCAACAACATTAAGTTGTACGTGCGCCGCGTGTTCATCATGGACAATTGCGAGGAGTTGATGCCTGAGTACCTATCGTTCGTCAAGGGTGTTGTTGATTCGGAAGACTTGCCGCTGAACATTTCACGTGAAACGCTGCAACAAAACAAGATCTTGCGCGTGATTAAGAAAAACTTGGTCAAGAAGTGCCTCGAAATGTTTGCCGAGCTTGCCGAAGACAATGAGAAGTACAACAAGTTCTACGAATCGTTCAGCAAAAACCTC-AAGCTTGGTATTCATGAGGACTCTACCAATCGCACCAAGATTGCCAAGCTGCTACGCTACCACTCCACCAAGGCGGGCGAAGAACTCACGTCACTGGACGACTACATTTCTCGCATGCCGGAGAACCAGCCTGGCATCTACTACGTGACGGGTGAAAGCAAGAAGGCGGTTGAGAACTCGCCCTTTATTGAAAAGCTCAAAAAGAAGGGTTACGAGGTGTTGTTCATGGTTGAGGCTATCGACGAGTACGCTGTGCAGCAGTTGAAGGAGTATGAGGGTAAGAAGCTCATTTGCGCCACGAAGGAAGGCCTCAAAATGGAGGAGACGGAAGACGAGAAGAAGTCGTTCGAGGAAGCGAAAGCTGCCACGGAGGGACTTCACAGTCATTGACGCACCTGGTCACCGTGACTTTATTAAAAACATGATCACGGGCACGTCTCAGGCCGATTGCGCCATTCTCGTGGTTGCTTCGGGCGTGGGTGAGTTTGAGGCGGGTATCTC---TAAGGAAGGCCAGACTCGTGAGCACGCATTGCTTGCCTTTACGCTTGGAGTGAAGCAGATGGTCGTTGCTATCAACAAGATGGACGACTCCTCCGTCATGTACGGCCAGGCTCGCTACGAAGAGATTAAGGCGGAAGTCACGACGTACCTAAAGAAGGTGGGCTACAAGCCTGCGAAGATCCCGTTCGTGCCCATTTCTGGATGGGAAGGAGACAATATGATTGACCGTTCGCCCAACATGGCGTGGTACAAGGGTCCTTACCTTCTTGAAGCCCTTGACAACTTGAACGCTCCCAAGCGTCCAAGTGACAAGCCTCTGCGTTTGCCGCTTCAGGATGTTTACAAGATTGGCGGCATTGGCACGGTCCCGGTCGGCCGTGTCGAGACGGGTATTATCAAGCCCGGTATGGTTACCACGTTCGGTCCCACCGGACTGTCAACAGAAGTCAAGTCTGTTGAAATGCATCACGAGTCTCTGCCGGAGGCTGTCCCTGGTGACAACGTTGGCTTCAACGTTAAGAACGTGTCAGTCAAGGAGCTGCGTCGTGGTTTCGTGGCTTCGGACTCGAAGAATGA---CCCTGCTAAGGGTACACAGGACTTCACGGCTCAAGTCATTGTGTTGAACCACCCTGGCCAGATTTGCAATGGTTACTCTCCTGTGTTGGACTGTCACACAGCCCACGTTGCCTGCAAGTTCAAAGAGATCACGGAGAAGATGGACCGTCGTTCCGGCAAGGTGCTTGTCCAGAACAAGAAGCCGTTTATCAAGTCGCGCTTCTGCCGTGGCGTGCCTGACCCAAAGATTCGCATCTACGATGTGGGTAACAAAAAGGCTTCGGTAGACGCCTTCCCATACGTGGCTCACCTCGTGTCCGACGAAAAGGAGCAGTTGTCATCGGAGGCCCTCGAGGCTGCTCGTATTGCTGCCAATCGATACTTGACTAAATTTTGCGGCAAAGACAATTTCCACATGCGTATTCGTTGCCACCCTTTTCAAGTTCTTCGTATCAACAAAATGCTTTCGTGCGCTGGTGCCGATAGGCTACAGACTGGCATGCGTCACGCTTACGGTAAGCCCGCCGGTGTGGCCGCTCGCGTGGCTATCGGTCAGCCTATTATTTCGGTACGCTCCAAAGACTCGTTTGGCCCCTCAGTTGTAGAGGCTTTGCGTCGTGCGTGGTGCGGACTCGGAGAAGATCTCGGCCGACAAGTTGCAGGAGGTGTATGAGGGTTTCATTGCCAAGTGCGCTGACTCTAGCAAGATCGTGTCCATTGAAGACCCATTTGACCAGGACGACTGGGAGTCGTGGATTAAGTTCACGGCGAAGGTTGGCGAAGACGTCCAGATTGTCGGCGACGACCTGACGGTGACGAACCCGACCCGTGTGAAAAAGGCCATTGAACAGAAGGCGTGCAATGCCCTTTTGCTTAAGGTGAACCAGATTGGCAGCATTACCGAGTCGATTGAGGCCGTGACGATGGCCAAGAAGGCTGGCTGGGCTATCATGGCCAGCCACCGTAGTGGTGAGACTGAAGACACATTCATTGCCGACCTGGCGGTGGGCTTGAGTGCCGGTCAGATGGTAACAATTGGGCCAAAGGACACTACACCGAGGGTGCTGAGTTGATCGATTCTGTGCTGGACGTCGTTCGAAAAGAGGCAGAAAGCTGTGACTGCCTTCAAGGTTTTCAAATTACGCACTCGCTTGGAGGCGGTACCGGCTCTGGAATGGGCACACTTTTAATCTCGAAAATTCGTGAAGAGTACCCTGATCGAATTATGTGCACCTACTCTGTTTGCCCGTCGCCCAAGGTTTCTGACACTGTCGTCGAGCCCTACAATGCCACGTTGTCGGTGCACCAGCTAGTTGAAAATGCTGACGAGGTCATGTGCCTGGACAATGAAGCCTTGTACGACATTTGCTTTCGTACTCTGAAACTCACCACTCCCACGTACGGCGACTTGAATCACTTGGTTTGTGCCGCCATGTCTGGCATCACCACATGCCTTCGATTTCCCGGACAGCTGAATTCGGACTTACGAAAATTGGCTGTTAACCTGATTCCGTTCCCCCGTCTTCATTTCTTTATGATTGGATTTGCACCACTGACATCGCGCGGCTCGCAGCAGTATCGTGCGTTAACTGTACCTGAGTTAACCCAGCAGCAGTTTGATGCAAAAAATATGATGTGTGCCGCTGATCCTCGCCACGGCCGCTATTTAACAGCTGCGTGTATGTTCCGCGGACGTATGAGCACGAAGGAGGTTGACGAGCAGATGCTAAATGTTCAGAACAAAAACTCTTCGTACTTTGTTGAATGGATTCCCAACAATATCAAGGCTAGTGTGTGTGACATTCCGCCCAAGGGCCTGAAAATGAGCACCACGTTTATTGGTAACTCTACGGCTATTCAGGAAATGTTTAAGCGAGTATCCGAACAGTTTACAGCTATGTTCCGTCGTAAGGCTTTCTTGCACTGG

Peronosclerospora_sorghi TTTTTTAATCATTATTACTGTATTTGTTTGTTGGATGTTATTTAAAGTAATAATTCTTTTTAATGAAAGAAAAAATAAAATTCCAAGTACCATTGTACATGGTGCAACTATAGAGATTATTTGGACTTCTATTCCTGCTTTAATATTATTAGTTATAGCTATTCCTTCTTTTGCTCTATTATATTCAATGGATGAAGTGATTGATCCAATTATTACTTTAAAGGTGATAGGTAGTCAATGGTATTGGAGTTATGAATATTCTGATAATTTAGAATTTTCACATGAACCTTTAATTTTTGATAGTTATATGATACAAGAAGATGATTTATCAATTGGTCAGTTTAGACTTTTAGAAGTTGATAATCGAGTTTTAGTTCCAACTAATAGTCATATTAGAATATTAATTACAGCTTCTGATGTTTTACATTCATGGGCCATACCTTCATTAGGAATTAAATTAGATGCTTGTCCAGGTAGATTAAACCAAACCGGCGAGTGAAGCGGGATGAGCTCAAACTTAAAATCTCCATGCAAGTTTTGTATGGCGAATTGTAGTCTATGGAGGC-GTGGTCAGTGTGGCGACTTGGGACAAGTTCCTTGGAAAAGGACAGCATGGAGGGTGATACTCCCGTTCATATCCTGAGTTGCTTACGCGTACGACCCGTTTTCTTTGAGTCGCGTTGTTTGGGAATGCAGCGCAAAGTAGGTGGTAAATTCCATCTAAAGCTAAATATTGGTGCGAGACCGATAGCAAACAAGTACCGTGAGGGAAAGATGAAAAGAACTTTGAAAAGAGAGTTAAAGAGTACCTGAAACTGCTGAAAGGGAACCGAATCGTTTCCAGTGTCTATAATCCGTGGTATATTTCATTGGCGAGTGCGTGCGTGCGTGTATTGTGGCAGTGGC--TTTT--AGCTGCGCTCGGTATG---TG-TGCTGCGTGTGTTTGCTAGTGCCCTGTGCTACGGTGGGACGTCAAGGTCAGTTCGTATACTGCGGGAAATGGCTGCCGAGGAGGTAGGGCTTACGCT-TGCGTTTGTCTATTATATCTTGGCGGACG-AGTAGTCGCGGTTGGGACTGAGGTG-TCTATAACGTGCTTTTGAGTGGGTCTGCGTCTCCGTGTACGCCGTGTGCGGATAGCTTGCTATGTGCGTGT---GGCTGTGTATGGATTGATGCGGACTTTAACTTGTTGCCGTTCATTCCGGAATCGGTATGACGAAGGCTGACTTGATCAACAACTTGGGTACGATCGCGAAGTCAGGTACCAAGGCATTCATGGAGGCCTTGGCTGCTGGTGCCGACATCAGTATGATTGGTCAGTTTGGTGTGGGTTTCTACTCTGCCTACCTTGTCGCTGACAAGGTTGTGGTGCACTCTAAGCACAATGACGACGAGCAGTACGTATGGGAGTCTGCAGCAGGCGGTTCTTTCACTGTAACGCCAGAT---ACCTCGGAGCCTATTCCACGTGGTACACGTATCGTGCTGAAGCTCAAGGAAGACATGCTCGAATACCTTGAGGAGCGCAAGTTGAAAGACCTAGTGAAGAAGCACTCGGAGTTTATTGGCTTCCCTATCAAGTTGTACGTCGAAAAGACGGAGGAAAAAGAAGTCACGGATGACGAGGAGGAGGAGGATG---AAAAGGAGGGTGACGAAGACAAGCCCAAGGTTGAGGAGGTTGAA---GAGGAGGAGGGTGAAAAGAAGAAGAAAACGAAGAAGATCAAGGAAGTGACCCACGAATGGGACCACTTGAACAGCCAGAAACCGATCTGGATGCGCAAGCCAGAAGATGTGACTCACGAGGAGTACGCTTCGTTCTACAAGTCACTCACCAACGACTGGGAGGAACATGCTGCGGTAAAGCACTTTTCTGTGGAAGGTCAGTTGGAGTTCAAGGCCTGTCTCTTCACTCCCAAGCGTGCTCCGTTTGACATGTTCGAGGGTGGTGCCAAGAAGAAACTAAACAACATTAAGTTGTACGTGCGTCGCGTGTTTATCATGGACAACTGTGAAGAGCTCATGCCAGAGTACCTGTCATTTGTCAAGGGTGTTGTTGACTCCGAGGACTTGCCGTTGAACATCTCGCGTGAGACGCTCCAGCAGAACAAGATTTTACGCGTCATTAAGAAGAACTTGGTCAAGAAATGCTTGGAAATGTTTGCCGAACTTGCTGAGGACAACGAAAAGTACAACAAGTTCTATGATGCGTTCAGCAAGAACTTG-AAGCTCGGTATCCATGAGGACTCCACGAACCGCTCCAAGATTGCAAAGTTGCTGCGCTACCACTCGACCAAGTCTGGGGAGGAGATGACGTCTCTCGACGACTACATTTCGCGCATGCCTGAAAACCAGCCTGGCATTTACTACGTGACGGGTGAGAGCAAGAAGTCTGTTGAGAACTCGCCATTCATTGAGAAACTGAAGAAGAAGGGCTACGAAGTGCTGTTCATGGTTGAAGCCATTGACGAGTACGCTGTGCAGCAGCTGAAGGAGTACGAGGGCAAGAAGCTAATCTGTGCCACCAAAGAGGGTCTTAAGATGGAGGAGACTGAAGACGAAAAAAAGGCTTTCGAAGAAGCTAAGGCTGCCACTGAGGGTCTTCACGGTCATTGACGCCCCCGGCCACCGTGACTTTATTAAGAACATGATAACCGGCACGTCGCAGGCTGATTGCGCCATTCTGGTTGTCGCTTCAGGTGTGGGTGAATTCGAGGCTGGTATTTC---CAAGGAAGGACAAACACGTGAGCACGCTCTTTTGGCTTTCACTCTTGGTGTGAAGCAGATGATTGTCGCTATTAACAAGATGGACGACTCGTCTGTCATGTACGGCCAGGCTCGTTACGAGGAAATCAAGTCGGAAGTAACGACTTACCTAAAAAAGGTTGGCTACAAGCCCGCCAAAATTCCATTTGTGCCTATCTCTGGATGGGAGGGTGACAACATGATTGACCGTTCCCCCAACATGCCTTGGTACAAGGGACCGTTCCTTCTTGAGGCTCTTGACAACCTCAACCCACCCAAGCGCCCATCAGACAAGCCTCTTCGTCTTCCCCTCCAGGACGTTTACAAGATCGGCGGTATTGGCACGGTACCGGTTGGCCGTGTCGAAACTGGTATCATTAAGCCTGGTATGGTTGCCACTTTCGGTCCTGTTGGTCTTTCAACGGAAGTCAAGTCGGTCGAAATGCACCACGAGTCTTTGCCCGAGGCTGTCCCAGGCGACAACGTTGGCTTCAATGTCAAGAATGTTTCAGTGAAGGAACTCCGTCGTGGTTTCGTTGCCTCCGACTCTAAGAATGA---CCCCGCTAAGGGTACTTCGGACTTCACGGCTCAAGTCATTGTGCTAAATCACCCTGGCCAGATTGGTAATGGTTACTCGCCTGTGCTTGATTGCCACACGGCCCACGTTGCCTGCAAGTTCAAAGAAATTACGGAAAAGATGGACCGTCGTTCCGGTAAGGTGCTTGTCCAGAATAAGAAGCCTTTTATTAAGTCGCGCTTCTGCCGCGGTGTGCCTGACCCTAAGATCCGCATCTACGATGTTGGTAACAAGAAGGCTTCTGTGGATGCCTTCCCCTTCGTGGCTCATTTGGTGTCGGACGAAAAAGAGCAACTTTCTTCGGAGGCTCTTGAAGCCGCCCGTATTGCTGCTAACCGCTACCTGACAAAGTTTTGCGGAAAAGACAACTTCCACATGCGCGTCCGATGCCATCCTTTTCAAGTTCTGCGTATTAATAAAATGCTTTCCTGTGCTGGAGCAGACAGACTGCAGACGGGAATGCGTCACGCTTACGGTAAGCCTGCTGGTGTAGCCGCCCGAGTTTCTATTGGACAGCCTATTATATCGGTGCGTTCCAAGGACTCGTTTGGTCCCTCCGTCGTCGAGGCTCTGCGTCGTGCACACCCCTGAGTCGGAAAAGATCTCGGCTGAGAAGCTGCTGGAGGTGTACGAAGGGTTTATTGAGAAGTGTGCGGGGTCGAGCCAGATCGTGTCGATTGAGGATCCGTTCGACCAAGATGACTGGGAGTCATGGGTGAAAATCACGAGCAAGATCGGCAAGGACGTGCAGATTGTAGGTGATGACCTTACGGTGACGAATCCGACCCGTGTAAAGAAGGCTATTGAGCAGCACGCTTGCAACGCACTCTTGCTCAAGGTGAACCAGATTGGCAGCATCACGGAGTCGATTGAAGCGGTGACCATGGCGAAGAAGGCTGGCTGGGCTATCATGGCTAGTCACCGTAGTGGGGAGACGGAGGACACGTTTATCGCCGATTTAGCTGTCGGTCTGAGCGCCGGTCAGATGGTAACAACTGGGCCAAGGGACACTACACTGAGGGTGCTGAGCTCATTGACTCGGTTCTAGACGTTGTTCGTAAAGAGGCCGAGAGTTGTGACTGTTTACAAGGATTTCAGATCACGCATTCACTTGGTGGCGGTACCGGCTCCGGCATGGGCACACTTCTTATCTCAAAGATCCGGGAAGAATACCCAGACCGAATCATGTGCACGTACTCAGTATGTCCATCCCCTAAGGTGTCAGACACAGTCGTGGAGCCTTACAACGCTACGCTCTCGGTTCATCAGCTTGTCGAGAATGCTGATGAGGTCATGTGTCTGGATAACGAAGCTTTGTACGACATTTGCTTCCGCACTCTGAAGCTTACTACTCCCACCTACGGTGATCTGAACCACTTAGTATGTGCCGCAATGTCTGGTATCACAACATGCTTGCGATTCCCTGGGCAGCTGAACTCGGACCTGCGCAAGCTGGCCGTAAACTTGATCCCGTTCCCGCGTCTCCACTTCTTTATGATTGGATTTGCCCCGTTGACGTCACGTGGCTCACAGCAATACCGTGCCCTAACAGTGCCCGAACTGACGCAGCAGCAATTCGATGCAAAAAATATGATGTGTGCCGCTGACCCTCGTCACGGCCGCTACTTAACTGCCGCGTGTATGTTCCGCGGACGCATGAGTACAAAGGAGGTTGACGAGCAAATGCTGAATGTGCAAAACAAGAACTCATCGTATTTCGTCGAATGGATTCCTAACAATATCAAAGCTAGCGTGTGTGACATTCCACCCAAGGGCTTGAAGATGAGCACTACGTTCATCGGGAACTCGACAGCGATCCAGGAAATGTTCAAGCGTGTCTCTGAACAGTTCACGGCCATGTTCCGTCGTAAAGCCTTCTTGCATTGG

Pythium_aphanidermatum TTTCTTAATTATTGTAACAGTTTTTGTTTGTTGGATGTTATTTAGAGTAATTACTTTATTTGATGAAAAAAAAAATCCAATACCTGCTACATTTGTACATGGTGCTACTATTGAAATTATTTGGACAACAATTCCAGCATTAATTTTATTAACAGTAGCTGTTCCTTCTTTTGCATTATTATATTCTATGGATGAAATTATTGATCCTATAATTACTTTAAAAGTAATAGGTAGTCAATGGTATTGGAGCTATGAATATTCTGATAATTTAGAATTTGCTGATGAACCTTTAATTTTTGATAGTTATATGGTACAAGAAAATGATTTAGAAATAGGTCAATTTAGATTATTAGAAGTAGATAACCGTGTTGTGGTGCCAACAAATAGTCATATTAGAGTATTAATTACAGCATCTGATGTATTACACTCTTGGGCTATTCCTTCTTTAGGTGTTAAATTAGATGCTTGTCCAGGTCGTTTAAATCAAACCGGCGAGTGAAGCGGGATGAGCTCAAGCTTAAAATCTCTGTGTCAGTTTGGCATGGCGAATTGTAGTCTATGGAGGC-GCTGTCAGTGCGACTATTCGGGGTAAGTTCCTTGGAAGAGGACAGCATCGAGGGTGATACTCCCGTATGTA-CCTGTGTAG-TTGCGCGTACGACACGTTTTCTTTGAGTCGCGTTGTTTGGGAATGCAGCGCAAAGTAGGTGGTAAATTCCATCTAAAGCTAAATATTGGTGCGAGACCGATAGCGAACAAGTACCGTGAGGGAAAGATGAAAAGAACTTTGAAAAGAGAGTTAAAGAGTACCTGAAACTGCTGAAAGGGAACCGAATCGTTTCCAGTGTCTATAATCTATGGCATATTTCATTGGCGCGTGGATGTGTGT-AGCGTTTTGGAAGTAGGTTTCTG-------CTCCTGGCGT----TG-CTTTGCGTTCGCTTGCTGGTGCCCTGTGCTGTAGTGGGACGTCAGAGTCAGTTCGTATGTTGCGGGAAATGGTTATTGGGGARGTAGGTCG-------CTTCGGTGGCTGTTATATCCTGGTAACT--AGTCGTCGTGGCTGGGACTGAGGTG-CCTACAATGTGCTTTCGAGTCTGCGGGCCTCTCGTTTGGCTGTTTGCTTTGATAGCTTGCTATGTTAGTGA---ACGTGTCAGGCGATTGAGACCTGTAGTAACTTGTTGCCGTTCACTCGGGTATTGGTATGACCAAGGCCGACCTCATCAACAACCTCGGTACCATTGCCAAGTCGGGCACCAAGGCCTTCATGGAGGCGCTTGCTGCCGGCGCTGACATTTCGATGATTGGTCAGTTCGGTGTCGGTTTCTACTCGGCCTACCTCGTTGCTGACAAGGTTGTCGTGCACTCGAAGCACAACGACGATGCCCAGTACGTTTGGGAGTCGGCCGCCGGTGGCTCGTTCACGGTCACCCCAGAC---ACTTCGGAGCCAATCCTTCGTGGTACCCGCATTGTCCTCAAGTTGAAGGAGGATATGCTCGAGTACCTTGAGGAGCGCAAGCTGAAGGACCTCGTCAAGAAGCACTCAGAGTTTATTGGCTTCCCAATCAAGCTCTACGTTGAGAAGACGGAGGAGAAGGAGGTCACGGATGATGAGGAAGACGAGG---------AGGAGGGCGACGACGACAAGCCAAAGGTCGAGGACGTCACC---GAAGAAGAGGGTGAGAAGAAGAAGAAGACGAAGAAGATCAAGGAGGTCTCGCACGACTGGAACCACCTCAACAGCCAGAAGCCAATCTGGATGCGCAAGCCTGAGGATGTGACCCATGAGGAGTACGCTTCGTTCTACAAGTCGCTCACTAACGACTGGGAGGAACACCTCGCCGTCAAGCACTTCTCGGTTGAAGGTCAGCTTGAGTTCAAGGCCTGCCTGTTTGCTCCTAAGCGTGCTCCATTTGACATGTTCGAGGGCGGTGCTCGCAAGAAGCTGAACAACATCAAGCTTTACGTCCGTCGTGTGTTCATTATGGACAACTGCGAGGAGCTCATGCCAGAGTATCTGTCGTTCGTGAAAGGTGTTGTCGACTCGGAGGACCTTCCGCTCAACATCTCCCGTGAGACTCTTCAGCAGAACAAGATTCTTCGTGTCATCAAGAAGAACCTTGTCAAGAAGTGCCTTGAGATGTTCGCTGAGATCGCCGAGGACTCGGAGAAGTACAACAAGTTCTACGAGGCCTTCAGCAAGAACCTT-AAACTCGGTATCCACGAGGACTCGACCAACCGCAACAAGATCGCCAAGCTCCTCCGTTACCACTCGACCAAATCGGGCGAGGACGTCACCTCCCTCGATGACTACATTAGCCGCATGCCTGAGAACCAGCCGGGTATCTACTATGTCACCGGCGAGAGCAAGAAGGCTGTGGAGAACTCGCCATTCATTGAGAAGCTCAAGAAGAAGGGCTACGAGGTTCTCTTCATGGTCGACGCCATTGATGAGTACGCCGTCCAGCAGCTGAAGGAGTACGAGGGCAAGAAGCTTATCTGTGCGACTAAGGAGGGCCTCAAGATGGAGGAGACCGAGGATGAGAAGAAGGCGTTTGAGGAGGCCAAGGCCGCCACTGCTGGTTCTCACGATCATCGATGCAGGCGGCCACCGCGACTTTTTTCGGCACACAATCGCTGGTGCGGCACAAGCTGACTGCGCGGTCCTCACAGTTGCTGCAAATGACGGTGAATTTGAATGCGGTATGGCGGGTGGCACTGGGTTGACGAAGGAACACGCGTTGGTTGCGTTTGCCATCGGGATCAAGCAACTCATCGTCCTGGTGACCAAGATGGACGCAATGTCGACACCCTACAGCCAGACACGATTCGACTCAATCAAGGGTGAGATGGGAGATTACTTGGCGAAGGTTGGATTTAAGGATGAGGACGTACAGTTTGTCCCAGTATCAGGATGGGAGGGTGAGAACCTTATGAGCAAGTCGACCCATATGCCATGGTACGAAGGGCCGACCCTACTTGAAGCGATAGATACTCTGATCGAGCCGAAGAGAGCGATCGACAAACCCCTTCGTCTCCCTGTGCAGAACGTTTATAAGATTGCCGGTGTGGGCACTGTCGTCACTGCCCGCGTCGAGACAGGTTTGCTTCGAGTTGGAACGCCTTTGGTTGTAGGGCCAACCAATGTGCGGGGGTCAGTTTTCTCGATCGAATCGCACCATCGCCAGCTCACGGAAGCATCCGCAGGAACCTGTGTGGGTATCAACATCAATAACGTCGCAAAGAACGACATCAAACGAGGATTTGTCCTCTCAGATGAGCGACATGA---TGCGGCAAGGGCAACTGTCGAGTTCACAGCTCAAATTGTCATTCTGAAGCACCCGGGATTGATCTCCAAAGGTTATACTCCGACAGTACACTGCCACACACTCCACACTGGGTGCAAGATCACGCAGATCGTCTCCAAACTGAATCGGTTAACGGGCGAGGAGGTGGTCCAGAACAAGAAGCCGTTCATTAAGTCGCGCTTCTGCCGTGGTGTGCCGGACCCGAAGATCCGCATCTACGAGGTCGGCAACAAGAAGGCGTCGGTCGACACCTTCCCATTCGTTGCCCACTTGGTCTCGGATGAGAAGGAGCAGCTCTCGTCGGAGGCTCTTGAGGCCGCCCGTATTGCCGCTAACCGTTACCTCGTGAAGTACTGCGGCAAGGACAACTTCCACATGCGTATCCGTTGCCACCCATTCCACGTTCTCCGTATCAACAAGATGCTTTCGTGCGCTGGTGCCGATCGTCTCCAGACCGGTATGCGTCACGCTTACGGTAAGCCACAGGGTACCGCTGCCCGTGTTGAGATTGGCCAGCCAATTATCTCGGTCCGCTCGAAGGACAACTTCGGCCCAGCCGTTGTTGAGGCTCTTCGTCGTGCACGCCGCTGACAAGGACAAGCTCCCAGTCGACAAGCTCATTGAGCTGTACGAGAGCTTCATCACCAAGTGCAAGGGCACCAGCCCAATTGTGTCGATCGAGGACCCATTTGACCAGGAGGACTGGGCGTCGTGGATCAAGTTCACCGCCAAGGTTGGCAAGGACGTGCAGATTGTGGGTGACGACCTTACGGTCACGAACCCAACCCGTGTTGCCCGCGCCATTGAGACCAAGGCCTGTAACGCCCTTCTCCTCAAGGTCAACCAGATTGGTAGCGTGACCGAGTCGATCGAGGCTGTGACCATGGCCAAGAAGGCTGGTTGGGCTATCATGACCTCGCACCGCTCGGGTGAGACTGAGGATGCGTTCATCGCTGACCTTGCCGTCGGTTTGAGCGCCGGTCAGATGGTAACAACTGGGCCAAGGGTCACTACACGGAGGGTGCTGAGCTCATCGACTCGGTGCTTGACGTCGTCCGCAAGGAGGCTGAGAGCTGTGACTGCCTTCAGGGTTTCCAGATCACCCACTCGCTTGGTGGTGGTACCGGTTCTGGTATGGGTACCCTCCTCATCTCGAAGATTCGTGAAGAGTACCCAGACCGTATCATGTGCACGTACTCGGTGTGCCCATCGCCAAAGGTCTCGGATACCGTCGTTGAGCCATACAACGCCACGCTTTCGGTCCACCAGCTTGTCGAGAACGCCGATGAGGTGATGTGTCTTGACAACGAAGCCCTTTACGATATCTGTTTCCGTACCCTGAAGCTCACGACGCCAACCTACGGTGATCTGAACCACCTTGTGTGTGCTGCCATGTCGGGTATCACGACCAGTCTGCGTTTCCCAGGTCAGCTGAACTCGGATCTTCGTAAGCTTGCCGTCAACCTTATCCCGTTCCCACGTCTCCACTTCTTCATGGTCGGTTTCGCTCCGCTCACCTCGCGCGGCTCGCAGCAGTACCGTGCCCTTACGGTGCCAGAGCTGACCCAGCAGCAGTTCGACGCCAAGAACATGATGTGTGCCGCTGATCCTCGTCACGGTCGTTACCTGACCGCCGCTTGTATGTTCCGTGGTCGTATGAGCACCAAGGAGGTCGACGAGCAGATGCTCAACGTTCAGAACAAGAACTCGTCGTACTTCGTTGAGTGGATCCCGAACAACATCAAGGCCAGCGTTTGTGACATCCCACCAAAGGGTCTCAAGATGAGCGCCACGTTCATCGGTAACTCGACTGCCATCCAGGAGATGTTCAAGCGTGTCAGCGAGCAGTTCACGGCCATGTTCCGTCGTAAGGCCTTCTTGCACTGG

Pythium_iwagamai TTTTTTAATTGTGGTAACTATTTTTGTTTGTTGGATGTTATTTAGAGTTATTACTCTTTTTGATGAAAAAAAAAATAAAATTCCTTCTACTGTTGTACATGGTGCAACAATTGAAATTATTTGGACTTCAGTACCTGCTTTAATTTTATTAACAGTAGCAGTACCTTCATTTGCTTTATTATATTCAATGGATGAAGTTATTGACCCAATTATAACTTTAAAAGTAATAGGTAGTCAATGGTATTGGAGCTATGAATATTCAGATAATTTAGAATTTTCAGATGAACCTTTAATTTTTGATAGTTATATGGTACAAGAAAATGATTTAGAAATTGGTCAATTTAGACTTTTAGAAGTAGATAACCGTATTGTGGTACCAACTAATAGTCATATTAGAGTATTAATAACAGCATCTGATGTTTTACATTCTTGGGCAATACCTTCTTTAGGTATTAAATTAGATGCTTGTCCTGGTCGTTTAAATCAAACCGGCGAGTGAAGCGGGATGAGCTCAAGCTTAAAATCTCTGTGCAAGTTTTGCATGGCGAATTGTAGTCTATAGAGGC-GCTGTCAGTGCGGCCGCTTGGGGTAAGTTCCTTGGAAGAGGACAGCATCGAGGGTGATACTCCCGTTTGTA-CCTGAGTGGTTTGCGCGTACGACACGTTTTCTTCGAGTCGCGTTGTTTGGGACTGCAGCGCAAAGTAGGTGGTAAATTCCATCTAAAGCTAAATATTGGTGCGAGACCGATAGCGAACAAGTACCGTGAGGGAAAGATGAAAAGAACTTTGAAAAGAGAGTTAAAGAGTACCTGAAACTGCTGAAAGGGAAGCGAATCGTATCCAGTGTCTATAATCCGTGGCATATTTCATTGGCGATTGTGTGCGTGC-GGCGCTTCGGAAGTGGCTTTTGT-------CGCTCCGTGTGTTGTT-TTCTGCGTGCGGTTGCTGGTGCCCTGTGTCGCGGTGGGACGTCAGAGTCAGTTCGTATGCTGCGGGAAATGGCTACTGAGGAGGTAGGACGGGGGCT-TGCTCCTGTCTGTTATATCTTGGTAGTT--AGTGGTCGTGGTTGGGACTGAGGTG-CCTACAACGTGCTTTGGAGTGTTCCGGGTTCTCACGTGGTGTGCTGTTTGTATAGCTTGCTATGCTTGTGG--TGTTCACCGTTTGATGGAGTCTGGTTTGTGCTTCTTGCCGTTCACTCGGGTATTGGTATGACCAAGGCCGACCTCATCAACAACCTCGGTACGATCGCCAAGTCGGGCACGAAGGCCTTTATGGAGGCGCTCGCCGCCGGCGCCGACATTTCGATGATCGGTCAGTTCGGTGTTGGTTTCTACTCGGCCTACTTGGTCGCTGACAAGGTTGTCGTGCACTCCAAGCACAACGACGACGAGCAGTACGTGTGGGAGTCCGCCGCTGGCGGCTCGTTCACGGTCACCGCTGAC---ACTTCGGAGCCGATTGCCCGCGGTACGCGCATCGTGCTTAAGCTCAAGGAGGACATGCTCGAGTACCTCGAGGAGCGCAAGATCAAGGACCTTGTGAAGAAGCACTCTGAATTCATCGGTTTCCCGATCAAGCTCTACGTTGAGAAGACGGAGGAGAAGGAAGTCACCGACGACGAGGAGGACGAAGACG---AGAAGGAAGACGAGGATGACAAGCCGACCGTCGAGGAGGTCACC---GAGGAGGAGGGTGACAAGAAGAAGAAGACGAAGAAGATCAAGGAGGTGACTCACGAATGGGACCACCTCAACAGCCAGAAGCCGATCTGGATGCGCAAGCCAGAAGACGTGACCCACGAGGAGTACGCTTCGTTCTACAAGTCGCTGACGAACGACTGGGAGGAGCACAGCGCCGTGAAGCACTTCTCGGTGGAGGGCCAGCTTGAGTTCAAGGCGTGCCTGTTCACGCCAAAGCGCGCGCCATTCGACATGTTCGAGGGCGGTGCGAAGAAGAAGGTGAACAACATCAAGCTGTACGTGCGTCGCGTGTTCATCATGGACAACTGCGAGGACCTCATGCCAGAGTACCTGTCGTTCGTCAAGGGTGTTGTTGACTCGGAGGACCTGCCGCTGAACATTTCGCGTGAGACGCTCCAGCAGAACAAGATCCTGCGCGTGATCAAGAAGAACCTGATCAAGAAGTGCCTTGAGATGTTCGCGGAGCTCGCCGAGGACAACGAGAAGTACAAGACGTTCTACGAGGCGTTCAGCAAGAACCTG-AAGCTCGGCATCCACGAGGACTCGACGAACCGCACCAAGATCGCCAAGCTCCTCCGTTACCACTCGACCAAGTCGGGTGAGGACCTGACGTCGCTTGACGACTACATCTCGCGCATGCCAGAGAGCCAGCCAGGCATCTACTACGTGACGGGCGAGAGCAAGAAGGCCGTCGAGAACTCGCCGTTCATCGAGAAGCTCAAGAAGAAGGGCTACGAGGTGATCTTCATGGTGGAGGCCATTGACGAGTACGCCGTGCAGCAGCTGAAGGAGTACGAGGGCAAGAAGCTCATCAGCGCGACGAAGGAGGGCCTCAAGATGGAGGAGTCCGAGGACGAGAAGAAGTCGTTCGAGGAGGCAAAGGCCGCCACCGCGGGCCTTCACTATGATTGACGCGCCGGGCCACCGTGACTTTGCCAGGAACATGATCGCAGGCACAGCGCAAGCCGACGCCACGCTCCTGGTCGTCGCTGCAGGCGAAGAAGAGTTCGAGGAACAGTTCAC---ACCGACGGGCCAGCTCCGTGAGCACGCACTGCTGGCGTTCACACTCGGCATCAAGCAGCTAGTCGTCGCGGTCAACAAGATGGACTGCGAGGCGGTTGCGTACAGCGAAGAGCGCTTCAACTCCGTCAAGAGCCACGTGAAGAGCTACTTGAAGAAAGTGGGCTTTAGCACGAGCGCGATGCTGTTCGTACCCATCTCAGGCTGGCTCGGTGACAACCTGACCGACAAGTCAGAGTCCATGCCCTGGTACGACGGTCCATGTCTGCTAGACGCTTTGGATACGCTCACTGTACCACGACGAGCGGCCGACAAGCCGCTGCGAATCGCGGTTCAGAGTGTGTGCGACCTCCGGAACGTTGGTACAGTCGTCGTCGGCCGCGTGATGACTGGCGTGCTCACCCCCGGGTTGGACGTGACCTTTGGACCCATGAACATCACTGCGGAAGTGGGTACCATCGAGCGCCATGGTCAAACTCTAACTGAGGCACGACCGGGTGACCAAATTGGCTTCACGCTCAAGGACATCACGTGGAGGGACATCTACCGGGGCTATGTGGTCTCGGATGCGAGACTCGA---CCCGGCGAAGCGAGCGGTCGAGTTCACAGCCCAAGTCGTAATTCTCAATGCACCCGGCTTGATCAGCGCCGGGTACACGCCCGTGGTACACTGTCACACAGCACGCACGACGTGCCGCTTCAGTAAGATCACGGAGAAGATTGACCGGCAGACGGGCAAAGTCGTGGTCCAGAACAAGAAGCCGTTCATCAAGTCGCGTTTCTGCCGTGGTGTGCCAGACCCGAAGATCCGCATCTACGATGTCGGTAACAAGAAGGCGTCGGTCGACACCTTCCCGTTCGTTGCCCACTTGGTGTCCGACGAGAAGGAGCAGCTCTCGTCGGAGGCCCTCGAGGCCGCCCGTATTGCCGCGAACCGTTACCTGACCAAGCACGCTGGCAAGGACAACTTCCACATGCGCATCCGTTGCCACCCGTTCCAGGTGCTGCGTATCAACAAGATGCTTTCGTGCGCCGGTGCTGATAGACTTCAGACCGGTATGCGTCACGCCTACGGTAAGCCAGCCGGTGTTGCCGCGCGTGTGGCCATTGGCCAGCCGATTATCTCGGTGCGCTCGAAGGACCAGTTCGGCCCTGCGGTCGTTGAGGCCCTGCGTCGTGCACGCTGCCGACTCGGACAAGCTCTCGGCCGACAAGCTCATCGAGCTCTACGAGAGCTTCATCGCCAAGTGCAAGAACACGAGCCCGATCGTGTCGATCGAGGACCCATTTGACCAGGACGACTGGGCCTCGTGGATCAAGTTCACGGCCAAGGTCGGTGCGGAAGTGCAGATCGTCGGTGACGACCTCACAGTCACGAACCCAACGCGCGTGCAGAAGGCGATTGACTCGAAGGCGTGCAACGCGCTCCTGCTCAAGGTGAACCAGATCGGTTCGGTGACGGAGAGCATTGAGGCCGTGACGATGGCGAAGAAGGCTGGCTGGGCCATCATGACGAGCCACCGCTCGGGAGAGACCGAGGACTCGTTCATCGCCGACCTGGCCGTGGGTCTGAGCGCTGGCCAGATGGTAACAACTGGGCCAAGGGTCACTACACGGAAGGCGCCGAGCTGATTGACTCGGTCTTGGATGTCGTCCGCAAGGAGGCGGAGAGCTGTGACTGCCTCCAGGGGTTCCAGATCACTCACTCACTGGGTGGTGGTACCGGTTCGGGTATGGGAACGCTCCTCATCTCCAAGATCCGTGAAGAGTACCCAGACCGCATCATGTGCACGTACTCGGTGTGTCCATCGCCAAAGGTGTCTGACACCGTTGTGGAACCATACAACGCCACTCTGTCGGTTCACCAGCTTGTTGAGAACGCCGATGAAGTTATGTGCCTCGACAACGAAGCCCTGTACGACATTTGCTTCCGCACGCTGAAGCTCACGACCCCGACCTATGGTGACCTGAACCACTTGGTGTGCGCCGCCATGTCGGGAATCACTACGTGCCTGCGCTTCCCAGGTCAGCTGAACTCGGACCTCCGTAAGCTGGCCGTGAACTTGATTCCGTTCCCGCGTCTCCACTTCTTCATGATTGGTTTCGCGCCGCTGACCTCACGTGGCTCCCAGCAGTACCGTGCGCTAACTGTGCCAGAGCTGACCCAGCAGCAGTTCGACGCGAAGAACATGATGTGCGCCGCTGACCCTCGCCACGGTCGCTATCTGACTGCTGCGTGCATGTTCCGTGGCCGTATGAGCACCAAGGAAGTCGACGAGCAGATGCTGAACGTCCAGAACAAGAACTCATCGTACTTTGTGGAGTGGATCCCAAACAACATCAAGGCGAGCGTGTGTGACATCCCTCCAAAGGGTCTGAAGATGAGTACGACGTTTGTCGGTAACTCGACTGCCATCCAGGAGATGTTCAAGCGTGTGTCTGAGCAGTTCACGGCGATGTTCCGTCGTAAGGCTTTCTTGCACTGG

Pythium_irregulare TTTTTTAATCGTTGTAACTATTTTTGTTTGTTGGATGTTATTTAGAGTTATTACTCTTTTTGATGAAAAAAAAAATAAAATTCCTTCTACTGTTGTACATGGTGCAACTATTGAAATTATTTGGACTTCAGTACCAGCTTTAATTTTATTAACTGTAGCAGTACCTTCATTTGCTTTATTATATTCAATGGATGAAGTTATTGATCCTATTATAACTTTAAAAGTAATAGGAAGTCAATGGTATTGGAGTTATGAATATTCAGATAATTTAGAATTTTCTGATGAACCTTTAATTTTTGATAGTTATATGGTACAAGAAAATGATTTAGAAATAGGTCAATTTAGACTTTTAGAAGTAGATAATCGTGTAGTAGTTCCAACTAATAGTCATATTAGAGTATTAATTACAGCTTCAGATGTTTTACATTCATGGGCAATACCTTCTTTAGGTATTAAATTAGATGCTTGTCCAGGTCGTTTAAATCAAACCGGCGAGTGAAGCGGGATGAGCTCAAGCTTAAAATCTCTGTGCCAGTTTGGCATGGCGAATTGTAGTCTATAGAGGC-GTTGTCAGTGCGGCTGTGCGGGGTAAGTTCCTTGGAAGAGGACAGCATCGAGGGTGATACTCCCGTTTGTA-CCTGTGCGGCTTGCGCGTACGACACGTTTTCTTCGAGTCGCGTTGTTTGGGACTGCAGCGCAAAGTAGGTGGTAAATTCCATCTAAAGCTAAATATTGGTGCGAGACCGATAGCGAACAAGTACCGTGAGGGAAAGATGAAAAGAACTTTGAAAAGAGAGTTAAAGAGTACCTGAAACTGCTGAAAGGGAAGCGAATCGTATCCAGTGTCTATAATCTGCGACATATTTCATTGGCGACAGTGTGCGTGCTTTTGCGTCGGAAGCGGCTTTTGT-------TGCTCTGTGC-TTTTG-CTGTGCGCGCTGTTGCTGTTGCCCTGTGTTGCGGTGGGACGTCAGAGTCAGTTCGTATGCTGCGGGAAATGGCCGCCGAGGAGGTAGGTCAGGATTT-CGGTCTTGGCTGTTATATCTTGGTGGTT--TGTTGTCGTGGTTGGGACTGAGGTG-CCTACAATGTGCTTTTGAGTGTGCCGGGTTCTCATTTGGTGGACTGTTTGGATAGCTTGCTATGCTTGTGGTTTTTTCATCGAGTGATTGAGTCTGGTGGTAACTTATTGCCGTTCACTCGGGTATCGGTATGACCAAGGCGGATCTTATCAACAACCTTGGTACGATCGCCAAGTCTGGCACGAAGGCCTTCATGGAAGCGCTTGCCGCCGGTGCCGACATTTCGATGATCGGTCAGTTCGGTGTGGGTTTCTACTCGGCCTACCTGGTCGCGGACAAGGTCGTGGTGCACTCGAAGCACAACGACGACGAGCAGTACGTGTGGGAGTCCGCCGCCGGCGGTTCGTTCACGGTTACTCCGGAC---ACGTCGGAGCCGATTGCCCGCGGTACGCGCATTGTGCTCAAGCTCAAGGAAGACATGCTTGAGTACCTGGAAGAGCGCAAGATCAAGGACCTCGTGAAGAAGCACTCTGAGTTCATTGGTTTCCCAATCAAGCTCTACGTCGAGAAGACGGAGGAGAAGGAAGTGACTGACGACGAGGAGGACGAAGACG---AGAAGGAAGGTGAGGACGACAAGCCAACCGTCGAGGAGGTCACT---GAGGAAGAAGGTGACAAGAAGAAGAAGACCAAGAAGATCAAGGAGGTGACTCACGAGTGGGACCACTTGAACAGCCAGAAGCCGATCTGGATGCGCAAGCCAGAAGATGTGACCCACGAGGAGTACGCTTCGTTCTACAAGTCGTTGACCAACGACTGGGAGGAGCACAGCGCCGTGAAGCACTTCTCGGTGGAAGGCCAGCTCGAATTCAAGGCGTGCCTGTTCACCCCAAAGCGTGCGCCATTCGACATGTTCGAGGGCGGTGCCAAGAAGAAGATCAACAACATCAAGCTGTATGTGCGTCGCGTGTTCATCATGGACAACTGCGAGGACCTGATGCCAGAGTACCTGTCGTTCGTGAAGGGTGTTGTCGACTCGGAGGACTTGCCATTGAACATTTCGCGTGAGACGCTTCAGCAGAACAAGATCCTGCGTGTGATCAAGAAGAACCTGATCAAGAAGTGCTTGGAGATGTTCGCGGAGCTGGCCGAGGACACTGAGAAGTACAAGACGTTCTACGAGGCGTTCAGCAAGAACTTG-AAGCTCGGTATCCACGAGGACTCGACGAACCGCACCAAGATCGCCAAGCTGCTTCGCTACCACTCGACCAAGTCTGGTGAGGACTTGACGTCGCTCGACGACTACATCTCGCGCATGCCAGAGAGCCAGCCAGGCATCTACTACGTGACCGGCGAGAGCAAGAAGGCCGTCGAGAACTCCCCATTCATTGAGAAGTTGAAAAAGAAGGGTTACGAGGTGATCTTCATGGTGGAGGCGATCGACGAGTACGCTGTGCAGCAGCTGAAGGAGTACGAGGGCAAGAAGCTGATCAGCGCCACGAAGGAGGGTCTCAAGATGGAGGAGACTGAGGACGAGAAGAAGTCGTTCGAGGAGGCGAAGGCCGCCACCAGTGGCGTTCACGGTATTGGATGCGCCGGGTCACCGTGATTTCATCAAGAACATGATCATCGGGACGTCGCAGGCCGACGCCGCACTCTTGGTGGTCGCTGCAGGCGACGCGGAGTTCGTCAGCGGACTCAC---CCCGACCGGACAGACCCGTGAGCACGCGCTGCTGGCGTTCACGCTCGGCGTGAAGCAGATGGTGGTCGCAGTGAACAAGATGGACAGCGAATCGGTAAACTACAGTCAAAACCGCTTCGAGTACGTCAAGGGCGAAGTGAGCGCATACTTGACGAAAGTGGGCTTCAACGCCGACGCGATCCCGTTTGTCCCCATCTCGGGCTGGTGCGGTGACAACATGACGGACAAGTCTGAGAACACGCCCTGGTACGATGGCCCCTGCCTGCTTGATGCGCTCGACACGCTCACTGTCCCACGACGAGCAGCTGACAAGCCGCTGCGTGTGCCGATTCAGAACGTGTACAAGATCGGCGGCATTGGCACGGTCGCCGTCGGTCGTGTCGAGACGGGCGTGCTCGCCCCAGGACAGGACGTGACGTTCGCACCTGTGAACGTGACTGCTGAAGTCGGCTCGGTTGAGAGACATCGTAAGCCGTTGGCCGAGGCGCGACCGGGCGACCATGTCGGCTTCAATGTGAAGGACGTCTCTGCAAAGGAGATCTCTCGCGGCTGCGTCGCGTCGGACCCGAAGCTTGA---CCCAGCAAAGGACACGATCGAGTTTACGGCGCAGGTTGTGATCCTGAACACGCCCGGCTTGATCTCCGCCGGATACACGCCGATCCTGCACTGCCACACGGCGCGCGTCGCGTGCGAGTTCAAGCAGATCACCGAGAAGATCGATCGGCAGTCGGGCAAAGTGGTGGTCCAGAACAAGAAGCCGTTCATCAAGTCGCGTTTCTGCCGTGGTGTGCCAGACCCGAAGATCCGCATCTACGATGTCGGCAACAAGAAGGCGTCGGTCGACACCTTCCCATTCGTTGCCCACTTGGTGTCGGACGAGAAGGAGCAGCTTTCGTCGGAAGCCCTTGAAGCCGCCCGTATTGCCGCGAACAGATACCTGACCAAGCACGCCGGCAAGGACAACTTCCACATGCGCATCCGTTGCCACCCATTCCAGGTTCTGCGTATCAACAAGATGCTTTCGTGCGCCGGTGCCGATAGACTGCAGACCGGTATGCGTCACGCTTACGGTAAGCCAGCGGGTGTTGCCGCCCGTGTGGCGATTGGCCAGCCAATTATCTCGGTGCGCTCCAAGGACCAGTTCGGCCCAGCGGTCGTCGAGGCTCTTCGTCGTGCACGCCGCGGACTCGGACAAGATCTCTGCCGACAAGCTCATTGAGCTGTACGAGAGCTTCATTGCCAAGTGCAAGAACACGAGCCCAATCGTGTCCATTGAAGACCCATTTGACCAGGACGACTGGGCTTCGTGGATCAAGTTCACCGCCAAGGTGGGCAAGGACGTGCAGATCGTCGGTGACGACCTCACGGTGACGAACCCAACGCGCGTGCAGAAGGCGATTGACTCCAAGGCGTGCAATGCGCTCCTGCTCAAGGTGAACCAGATCGGCTCGGTGACTGAGAGTATCGAAGCCGTGACGATGGCGAAGAAGGCTGGCTGGGCCATCATGACGAGCCACCGTTCGGGTGAGACCGAAGACTCGTTCATCGCCGATTTGGCGGTCGGTTTGAGCGCTGGCCAGATGGTAACAACTGGGCCAAGGGTCACTACACTGAAGGCGCTGAGTTGATCGACTCTGTCCTGGATGTCGTCCGCAAGGAAGCTGAGAGCTGTGACTGCCTGCAGGGTTTCCAGATCACCCACTCCCTCGGTGGTGGTACCGGTTCGGGTATGGGGACGCTTCTCATCTCCAAGATCCGTGAAGAATACCCAGACCGCATCATGTGCACGTACTCGGTCTGCCCATCGCCAAAGGTGTCGGACACCGTTGTGGAGCCATACAACGCCACCCTGTCGGTCCACCAGCTGGTCGAAAACGCCGATGAAGTGATGTGCTTGGACAATGAAGCCTTGTACGATATTTGCTTCCGCACGCTGAAGCTGACGACCCCAACCTACGGTGACTTGAACCACTTGGTGTGTGCCGCCATGTCCGGTATCACCACGTGCCTGCGTTTCCCTGGTCAGTTGAACTCGGACCTCCGCAAGCTTGCCGTGAACTTGATCCCGTTCCCACGTCTCCACTTTTTCATGATTGGTTTCGCGCCGTTGACCTCGCGTGGCTCCCAGCAATACCGTGCGTTGACTGTCCCAGAGCTGACCCAGCAGCAGTTCGACGCGAAGAACATGATGTGCGCCGCCGATCCTCGCCACGGTCGTTATTTAACTGCTGCTTGTATGTTCCGCGGACGCATGAGCACGAAGGAAGTCGATGAGCAGATGCTGAACGTCCAGAACAAGAACTCGTCGTACTTCGTGGAATGGATTCCAAACAACATCAAGGCGAGCGTGTGTGACATCCCTCCAAAGGGTCTCAAGATGAGTACCACGTTCATCGGTAACTCGACTGCCATCCAGGAGATGTTTAAGCGTGTGTCTGAGCAGTTCACGGCAATGTTCCGTCGCAAGGCTTTCTTGCACTGG

Pythium_oligandrum TTTTTTAATTGTTGTAACTATTTTTGTTTGTTGGATGTTATTTAGAGTTATTACTCTTTTTGATGAAAAAAAAAATAAAATTCCTTCTACTGTTGTACATGGTGCAACTATTGAAATTATTTGGACTTCAGTACCTGCTTTAATTTTATTAACTGTAGCTGTACCTTCATTTGCTTTATTATATTCAATGGATGAAGTTATTGATCCTATTATAACTTTAAAAGTAATAGGAAGTCAATGGTATTGGAGTTATGAATATTCTGATAATTTAGAATTTTCTGATGAACCTTTAATTTTTGATAGTTATATGGTACAAGAAAATGATTTAGAAATAGGTCAATTTAGACTTTTAGAAGTAGATAATCGTGTAGTAGTACCAACTAATAGTCATATTAGAGTATTAATTACAGCTTCAGATGTGTTACATTCATGGGCTATACCTTCTTTAGGTATTAAATTGGATGCTTGTCCAGGTCGTTTAAATCAAACCGGCGAGTGAAGCGGGATGAGCTCAAGCTTAAAATCTCTGTGCCAGTTTGGCATGGCGAATTGTAGTCTATGGAGGC-GCTGTCAGTGCGATTGCGTGGGTTAAGTTCCTTGGAAGAGGACAGCATCGAGGGTGATACTCCCGTGTATG-CCTGTGCAA-TTGTGCGTACGACACGTTTTCTTTGAGTCGCGTTGTTTGGGAATGCAGCGCAAAGTAGGTGGTAAATTCCATCTAAAGCTAAATATTGGTGCGAGACCGATAGCGAACAAGTACCGTGAGGGAAAGATGAAAAGAACTTTGAAAAGAGAGTTAAAGAGTACCTGAAACTGCTGAAAGGGAATCGAATCGTTTCCAGTGTCTATAATCTGTGGCATATTTCATTGGCGAGTGTGTGCGTGC-ATTGTTTTGGAAGCAGTTTTCTG-------TTCCTGGCTG----TG-TTGTGCATATGCTTGCTGGTGCCCTGTGTCGCGGTGGGACGTCAGAGTCAATTCGTAAGTTGCGGGAAATGGCTGTTGGGGAGGTAGGTCA-------CTTCGGTGGCTGTTATATCCTGATAGCT--AGTAGTCGTGGCTGGGATTGAGGTG-CCTACAACGTGCTTTCGAGTGTCTGGGTGTCTCGTTTGGTAAGTTATCTTGACAGCTTGCTGTGTTGGTGA---TATTATCGAGCGATTGATGTCTGGTCTAACTTGTTGCCGTTCACTCGGGTATTGGTTTGACCAAGGCCGACCTCATCAACAACCTCGGTACCATTGCCAAGTCGGGCACCAAGGCGTTTATGGAGGCCCTTGCTGCCGGTGCTGATATCTCGATGATCGGTCAGTTCGGTGTCGGTTTCTACTCGGCCTACCTCGTCGCCGACAAGGTGGTCGTGCACTCGAAGCACAACGACGATGCCCAGTACGTGTGGGAGTCGGCTGCCGGTGGTTCGTTCACCGTGTCGCCTGAC---ACGTCGGAGCCAATTGCCCGTGGTACGCGCATTGTGCTCAAGTTGAAGGAGGACATGCTCGAATACCTCGAGGAGCGCAAGCTCAAGGACCTCGTCAAGAAGCACTCGGAGTTCATTGGTTTCCCAATCAAGCTGTATGTCGAGAAGACGGAGGAGAAGGAAGTGACTGATGACGAAGAGGACGAGG---------AGGAAGCCGATGACGACAAGCCAAAGGTTGAGGACGTCACC---GACGAGGAGGGTGAGAAGAAGAAGAAGACGAAGAAGATCAAGGAGGTCACCCACGACTGGAACCACCTCAACAGCCAGAAGCCAATCTGGATGCGCAAGCCAGAGGATGTGACCCACGAGGAGTACGCCTCGTTCTACAAGTCGTTGACCAACGACTGGGAGGAGCACTTGGCTGTCAAGCACTTCTCGGTTGAGGGTCAGCTTGAATTCAAGGCCTGCTTGTTCGCGCCAAAGCGTGCTCCATTCGACATGTTCGAGGGTGGTGCCCGCAAGAAGCTCAACAACATCAAGCTCTACGTGCGTCGTGTGTTCATCATGGACAACTGCGAGGAGCTCATGCCTGAGTACTTGTCGTTCGTCAAGGGTGTTGTCGACTCGGAGGACTTGCCATTGAACATTTCGCGTGAGACCCTCCAGCAGAACAAGATCTTGCGTGTCATCAAGAAGAACTTGGTCAAGAAGTGCTTGGAGATGTTCGCTGAGATCGCCGAGGACTCGGAGAAGTACAACAAGTTCTACGAGGCTTTCGGCAAGAACCTC-AAGCTTGGTATCCACGAGGATTCGACCAACCGCAACAAGATTGCCAAGCTCCTCCGTTACCACTCGACCAAGTCGGGTGAGGACGTGACCTCTCTTGACGACTACATCAGCCGCATGCCAGAGAACCAGCCAGGTATCTACTACGTGACTGGTGAGAGCAAGAAGGCTGTCGAGAACTCGCCATTCATTGAGAAGCTCAAGAAGAAGGGCTACGAAGTTCTCTTCATGGTTGAGGCCATCGATGAGTACGCCGTCCAGCAATTGAAGGAGTACGAGGGCAAGAAGCTCATCTGTGCCACCAAGGAGGGCCTCAAGATGGAGGAGACCGAGGATGAGAAGAAGTCGTTCGAGGAGGCCAAGGCTGCCACTGAGGGCATTCGCGGTGGTCGATACTCCTGGGCATTTGGACTACTATAAAAACACAATCTCTGGAATCGCTCTTGGGGATTGCGGCCTTCTCGTGGTACCAGCC-----------GTTTGGGTCTCGCAGAGC-GATGAGATGGCTGTTGTGCGTGATCAGCTCGTGACTGCGTTTACATTTGGCATCCGGCAAGTGATCGTGGCTGTCAACAAGATGGATA------CAGTTGAATACGCACAGGAGAGTTTTGACCAAGTCAAAACAGAAGTCGCAGCGATCATGAAGTCGACTGGCTTCGTGCCCGAAAAGGTGTATCTCATTCCTGTTTCTGGATGGGCAGGCGATAACTTGATCCAGAAGTCGACTCGGATGACCTGGTTCGATGGGCCGACACTCATTGACGCACTTGATCATGTCGTTTCACCCCGTCGCGCAGCGGAGAAGCCGCTCCGGGTGTCTTTGCTTGAAGCATACAAGATTAGTGGAGTCGGAACTGTTGCAGTCGGACGCGTTGAGACCGGCACTATGAAACCTGGTATGACGATCGCGTTCAGTCCGACTGGTTCCATTTCGGCTGTTCAATCTATCGAAATGAGTCACCAAGCCGTGACCGAAGCTCGACCAGGTGATAACATCGGCTTTAGCCTTGCAGGTATCTCTGCTAAGGAATTGAAGCGAGGCTATATTGCATCCGAGGATCTGCGGGA---CCCAGCGCGACAGGCCATCGAATTCACAGCACAAGTCGTCTTTGCCCACCTCGACGGGTTGCTCTCAGTTGGTTCGACTATTGTCGTGCACTGCCACACAACGCGAGTGACATGCCGGGTCAAGAGTATCTTCGCCAAGCTGGATCGACAAACGGGAACCGTTGTTGTCCAGAACAAGAAGCCGTTCATCAAGTCCCGCTTCTGCCGTGGTGTGCCGGACCCAAAGATCCGCATCTACGAAGTCGGCAACAAGAAGGCTTCGGTCGACACGTTCCCTTTCGTGGCCCACTTGGTCTCGGATGAGAAGGAACAGCTCTCGTCGGAGGCGCTTGAAGCCGCTCGTATTGCCGCTAACCGTTACCTCGTGAAGTACTGCGGCAAGGACAACTTCCACATGCGTATCCGTTGCCACCCATTCCACGTCTTGCGTATCAACAAGATGCTTTCGTGCGCTGGTGCCGATCGTCTCCAGACCGGTATGCGTCACGCTTACGGTAAGCCACAGGGTACCGCCGCCCGTGTGGAGATCGGCCAGCCAATTATTTCGGTGCGCTCGAAGGACAACTTCCAGGCCGCCGTCGTTGAGGCTTTGCGTCGTGCACGCCGCCGACAAGGACAAGCTCCCAGCTGACAAGCTCATTGAGTTGTACGAGAGCTTCATCGCCAAGTGCAAGGGCTCGAGCCCAATCGTGTCGATTGAGGACCCATTTGACCAGGATGACTGGGACTCGTGGGTCAAGTTCACCGCCAGGGTTGGCCAGGAAGTGCAGATTGTCGGCGACGATCTCACGGTCACCAACCCAACGCGTGTGGCCCGTGCCATCGAAAAGAAGGCCTGTAACGCTCTCTTGCTCAAGGTGAACCAGATTGGTAGCGTCACCGAGAGTATCGAGGCCGTGACCATGGCCAAGAAGGCCGGCTGGGCCATCATGACGTCGCACCGCAGTGGTGAGACCGAGGATGCGTTCATTGCCGACCTTGCCGTCGGTCTCAGCGCTGGTCAGATGGTAACAACTGGGCCAAGGGTCACTACACCGAAGGTGCGGAGTTGATCGACTCGGTCCTCGATGTTGTCCGCAAGGAAGCCGAGAGCTGTGACTGCCTTCAGGGTTTCCAGATCACGCACTCGCTCGGTGGTGGTACCGGTTCGGGTATGGGTACTCTCTTGATCTCCAAGATCCGTGAGGAATACCCAGACCGTATCATGTGCACGTACTCGGTGTGCCCTTCGCCAAAGGTGTCGGACACCGTCGTTGAGCCTTACAACGCTACCCTCTCGGTGCACCAGCTCGTCGAAAACGCCGATGAAGTCATGTGCTTGGACAACGAAGCTCTTTACGATATCTGCTTCCGTACGCTCAAGTTGACGACTCCAACCTACGGTGACTTGAACCACTTGGTGTGCGCGGCTATGTCGGGTATCACTACGTGCTTGCGTTTCCCAGGTCAGCTCAACTCGGATCTCCGCAAGTTGGCCGTGAACTTGATTCCGTTCCCACGTCTCCACTTCTTCATGATTGGTTTCGCTCCATTGACGTCGCGTGGCTCGCAGCAGTACCGTGCCCTCACTGTCCCAGAGTTGACCCAGCAGCAGTTCGATGCCAAGAACATGATGTGTGCCGCTGATCCTCGCCACGGTCGTTACTTGACCGCCGCTTGTATGTTCCGTGGTCGTATGAGCACCAAGGAAGTCGACGAGCAGATGTTGAACGTCCAGAACAAGAACTCGTCGTACTTCGTCGAGTGGATTCCAAACAACATCAAGGCCAGCGTGTGTGATATCCCACCAAAGGGCTTGAAGATGAGCACGACCTTCGTCGGTAACTCGACCGCCATTCAGGAGATGTTCAAGCGTGTCTCGGAGCAGTTCACTGCCATGTTCCGTCGTAAGGCTTTCTTGCACTGG

Pythium_spinosum TTTTTTAATTGTTGTAACTATTTTTGTTTGTTGGATGTTATTTAGAGTTATTACTCTTTTTGATGAAAAAAAAAATAAAATTCCTTCTACTGTTGTACATGGTGCAACTATTGAAATTATTTGGACTTCAGTACCCGCTTTAATTTTATTAACAGTAGCTGTACCTTCATTTGCTTTATTATATTCTATGGATGAAGTTATTGATCCAATTATAACTTTAAAAGTAATAGGAAGTCAATGGTATTGGAGTTATGAATATTCTGATAATTTAGAATTTTCTGATGAACCTTTAATTTTTGATAGTTATATGGTTCAAGAAAATGATTTAGAAATAGGTCAATTTAGACTTTTAGAAGTAGATAATCGTGTGGTAGTACCAACTAATAGTCATATTAGAGTATTAATTACAGCTTCAGATGTTTTACATTCATGGGCTATACCTTCTTTAGGTATTAAATTAGATGCTTGTCCAGGTCGTTTAAATCAAACCGGCGAGTGAAGCGGGATGAGCTCAAGCTTAAAATCTCTGTGCCAGTTTGGCATGGCGAATTGTAGTCTATAGAGGC-GTTGTCAGTGCGGCTGTGCGGGGTAAGTTCCTTGGAAGAGGACAGCATCGAGGGTGATACTCCCGTTTGTA-CCTGTGCGGCTTGCGCGTACGACACGTTTTCTTCGAGTCGCGTTGTTTGGGACTGCAGCGCAAAGTAGGTGGTAAATTCCATCTAAAGCTAAATATTGGTGCGAGACCGATAGCGAACAAGTACCGTGAGGGAAAGATGAAAAGAACTTTGAAAAGAGAGTTAAAGAGTACCTGAAACTGCTGAAAGGGAAGCGAATCGTATCCAGTGTCTATAATCTGCGGCATATTTCATTGGCGGCGGTGTGCGTGCTTTTGCATCGGAAGCGGCTTTTGT-------TGCTCTGTGC-TTTTG-CTGTGCGTGCTGTTGCTGGTGCCCTGTGTTGTAGTGGGACGTCAGAGTCAGTTCGTATGCTGCGGGAAATGGCTGCTGAGGAGGTAGGTTAGGACTT-CGGTCTTGGCTGTTATATCTTGGTGGTT--TGTTGTCGTGGTTGGGACTGAGGTG-CCTACAATGTGCTTTTGAGTGTGCCGGGTTCTCATTTGGTGGACTGTTTGGATAGCTTGCTATGCTTGTGG-TTTTTCACCGAGTGATTGAGTTTGGTGGTAACTTATTGCCGTTCACTCGGGTATCGGGATGACCAAGGCAGATCTTATCAACAACCTCGGTACGATCGCCAAGTCCGGCACGAAGGCCTTCATGGAAGCGCTTGCCGCCGGTGCCGACATTTCGATGATCGGTCAGTTCGGTGTGGGTTTCTACTCGGCCTACCTGGTCGCGGACAAGGTCGTGGTGCACTCGAAGCACAACGACGACGAGCAGTACGTGTGGGAGTCCGCCGCCGGCGGTTCGTTCACGGTCACTGCGGAC---ACGTCGGAGCCGATTGCCCGCGGTACGCGCATTGTGCTCAAGCTCAAGGAAGACATGCTTGAGTACCTGGAAGAGCGCAAGATCAAAGACCTTGTGAAGAAGCACTCCGAGTTCATTGGTTTCCCAATCAAGCTCTATGTCGAGAAGACGGAGGAGAAGGAAGTGACTGACGACGAGGAGGACGAAGACG---AGAAGGAAGGCGAGGACGACAAGCCAACCGTCGAGGAGGTCACT---GAGGAAGAAGGCGACAAGAAGAAGAAGACCAAGAAGATCAAGGAGGTGACCCACGAGTGGGACCACTTGAACAGCCAGAAGCCGATCTGGATGCGCAAGCCAGAAGATGTGACCCACGAGGAGTACGCTTCGTTCTACAAGTCGCTGACCAACGACTGGGAGGAGCACAGCGCCGTGAAGCACTTCTCGGTGGAAGGCCAGCTCGAGTTCAAGGCGTGCCTGTTCACCCCAAAGCGTGCGCCATTCGACATGTTCGAGGGCGGTGCCAAGAAGAAGATCAACAACATCAAGCTGTACGTGCGTCGCGTGTTCATCATGGATAACTGCGAGGACCTGATGCCAGAGTACCTGTCGTTCGTGAAGGGTGTTGTCGACTCGGAGGACTTGCCATTGAACATTTCGCGTGAGACGCTTCAGCAGAACAAGATCCTGCGCGTGATCAAGAAGAACCTGATCAAGAAGTGCTTGGAGATGTTCGCGGAGCTGGCCGAGGACACTGAGAAGTACAAGACGTTCTACGAGGCGTTCAGCAAGAACTTG-AAGCTCGGTATCCACGAGGACTCGACGAACCGCACCAAGATCGCCAAGCTGCTTCGCTACCACTCGACCAAGTCTGGCGAGGACTTGACGTCGCTCGACGACTACATCTCGCGCATGCCAGAGAGCCAGCCAGGCATCTACTACGTGACCGGCGAGAGCAAGAAGGCCGTTGAGAACTCCCCATTCATTGAGAAGTTGAAGAAGAAGGGCTACGAGGTGATCTTCATGGTGGAGGCGATCGACGAGTACGCTGTGCAGCAGCTGAAGGAGTACGAGGGCAAGAAGCTGATCAGCGCCACGAAGGAGGGTCTCAAGATGGAGGAGACTGAGGACGAGAAGAAGTTGTTCGAGGAGGCGAAGGCCGCCACCAGCGGCGTTCACGGTGCTGGACGCGCCGGGTCACCGCGATTTCATCAAGAACATGATCACCGGGACGTCGCAGGCCGACACCGCGCTCTTGGTGGTCGCTGCAGGTGACACGGAGTTCGCCGACGGACTCGC---CCCGACTGGACAGACCCGCGAGCACGCGCTGCTGGTGTTCACGCTCGGCGTGAAGCAGATGGTGGTCGCGGTGAACAAGATGGACAGCGAATCGGTAAACTACAGTCAAGACCGCTTCGAGTACGTCAAGGGCGAAGTGAGCGCGTACTTGACGAAAGTGGGCTTCAACGCTGACGCGATCCCGTTTGTCCCCATCTCGGGCTGGTGCGGCGACAACATGACCGACAAGTCCGAGAATATGCCCTGGTACGACGGTCCCTGCCTGCTTGGTGCGCTTGACACGTTCATTACCCCACGACGAGCAGCTGACAAGCCGCTGCGTGTGCCGATTCAGAACGTGTACAAGATCGGCGGCATTGGCACGGTCGCCGTCGGTCGCGTCGAGACGGGCGTGCTCGCCCCAGGACAGGACGTGACGTTCGCACCTGTGAACGTGACTGCTGAAGTCGGCTCGGTCGAGAGACATCGTGAGCCGTTGGCTGAGGCGCGGCCGGGCGACCAGGTTGGCTTTAATGTGAAGGGCGTCTCTGCAAAGGAGATCTCTCGCGGCTGCGTCGCGTCGGACCCGAAGCTTGA---CCCGGCAAAGAACACGATCGAGTTCACGGCGCAGGTCGTGATCCTGAACACGCCCGGCTTGATCTCCGCCGGGTACACGCCGATTCTGCATTGCCACACGGCGCGCGTCGCATGCAAGTTCAAGCAGATCACCGAGAAGATCGATCGGCAGTCGGGCAAAGTGGTGGTCCAGAACAAGAAGCCGTTCATCAAGTCGCGTTTCTGCCGTGGTGTGCCAGACCCGAAGATCCGCATCTACGATGTCGGCAACAAGAAGGCGTCGGTCGACACCTTCCCATTCGTTGCCCACTTGGTGTCGGACGAGAAGGAGCAGCTTTCGTCGGAAGCCCTCGAAGCCGCCCGTATTGCCGCGAACAGATACCTGACCAAGCACGCTGGCAAGGACAACTTCCACATGCGCATCCGTTGCCACCCATTCCAGGTGCTGCGTATCAACAAGATGCTTTCGTGCGCCGGTGCCGATAGACTGCAGACCGGTATGCGTCACGCTTACGGTAAGCCAGCGGGTGTTGCCGCCCGTGTGGCGATTGGCCAGCCAATTATTTCGGTGCGCTCCAAGGACCAGTTCGGCCCAGCGGTCGTCGAGGCTCTTCGTCGTGCACGCCGCGGACTCGGACAAGATCTCGGCCGACAAGCTCATTGAGCTGTACGAGAGCTTCATCGCCAAGTGCAAGAACACGAGCCCAATCGTGTCCATTGAGGACCCATTTGACCAGGACGACTGGGCTTCGTGGATCAAGTTCACCGCCAAGGTGGGCAAGGACGTGCAGATCGTCGGTGACGACCTCACGGTGACGAACCCAACGCGCGTGCAGAAGGCGATTGACTCCAAGGCGTGCAATGCGCTCCTGCTCAAGGTGAACCAGATCGGCTCGGTGACTGAGAGTATCGAAGCCGTGACGATGGCGAAGAAGGCTGGCTGGGCCATCATGACGAGCCACCGTTCGGGTGAGACCGAAGACTCGTTCATCGCCGATTTGGCGGTCGGTTTGAGCGCCGGTCAGATGGTAACAACTGGGCCAAGGGTCACTACACTGAAGGCGCTGAGTTGATCGACTCCGTCCTGGATGTCGTCCGCAAGGAAGCTGAGAGCTGTGACTGCCTGCAGGGTTTCCAGATCACCCACTCCCTCGGTGGTGGTACCGGTTCGGGTATGGGCACGCTCCTCATCTCCAAGATCCGTGAAGAATACCCAGACCGCATCATGTGCACTTACTCGGTCTGCCCATCGCCAAAGGTGTCGGACACCGTCGTGGAACCGTACAACGCCACCCTGTCGGTCCACCAGCTGGTCGAAAACGCCGATGAAGTGATGTGCTTGGACAATGAAGCCTTGTACGATATCTGCTTCCGCACGCTGAAGCTGACGACCCCAACCTACGGTGACTTGAACCACTTGGTGTGTGCCGCCATGTCCGGTATCACCACGTGCCTGCGTTTCCCTGGTCAGTTGAACTCGGACCTCCGCAAGCTCGCCGTGAACTTGATCCCGTTCCCACGTCTCCACTTCTTCATGATTGGTTTCGCGCCGTTAACCTCGCGTGGCTCCCAGCAGTACCGTGCGTTGACTGTCCCAGAGCTGACCCAGCAGCAGTTCGACGCGAAGAACATGATGTGCGCCGCCGATCCTCGCCACGGTCGTTATTTAACTGCTGCTTGTATGTTCCGCGGACGCATGAGCACGAAGGAAGTCGATGAGCAGATGCTGAACGTCCAGAACAAAAACTCGTCGTACTTCGTGGAATGGATTCCAAACAACATCAAGGCGAGCGTGTGTGACATCCCTCCAAAGGGTCTCAAGATGAGTACCACGTTCATCGGTAACTCGACTGCCATCCAGGAGATGTTCAAGCGTGTGTCTGAGCAGTTCACGGCAATGTTCCGTCGCAAGGCTTTCTTGCACTGG

Pythium_ultimum TTTTTTAATCGTTGTAACTGTTTTTGTTTGTTGGATGTTATTTAGAGTTATTACTCTTTTTGATGAAAAAAAAAATAAAATACCTTCAACTGTTGTACATGGTGCTACTATTGAAATTATTTGGACTACAATACCAGCTTTAATTTTATTAACTGTAGCTGTTCCATCATTTGCTTTATTATATTCAATGGATGAAGTAATTGATCCTATTATTACTTTAAAAGTAATTGGTAGCCAATGGTATTGGAGTTATGAATATTCTGATAATTTAGAGTTTTCAGATGAACCTTTAATTTTTGATAGTTATATGGTACAAGAAAATGATTTAGAAATAGGTCAATTTAGACTTTTAGAAGTAGATAATCGTGTAGTTGTTCCAACTAATAGCCATATTAGAGTATTAATTACAGCTTCTGATGTTTTACATTCATGGGCGATACCTTCATTAGGTGTTAAATTAGATGCTTGTCCTGGTCGTTTAAATCAAACCGGCGAGTGAAGCGGGAAGAGCTCAAGCTTAAAATCTCTATGCAAGTTTTGCATGGCGAATTGTAGTCTATAGAGGC-GCTGTCAGTGCAGCTATTTGGGATAAGTTCCTTGGAAGAGGACAGCATCGAGGGTGATACTCCCGTTTGTG-CCTGAGTAGCTTGTGCGTACGACACGTTTTCTTTGAGTCGCGTTGTTTGGGACTGCAGCGCAAAGTAGGTGGTAAATTCCATCTAAAGCTAAATATTGGTGCGAGACCGATAGCGAACAAGTACCGTGAGGGAAAGATGAAAAGAACTTTGAAAAGAGAGTTAAAGAGTACCTGAAACTGCTGAAAGGGAAGCGAATCGTATCCAGTGTCTATAATCTGTAGCATATTTCATTGGCGAGTAGGTGCGTGC-AGTATGTAAGAAGTGGTTTTCTG-------CTCTTTGTGC----TG-TTCTGCGTTTGCTTGTTGGTGCCCTGTGTTGCGGTGGGACGTCAGAGTCAGTTCGTATGCTGTGAGAAATGGCTACTGAGGAGGTAGGTTAGGAGCT-TGCTCTTGGCTGTTATATCTTGGTAGTT--AGTTGTCGTGGTTGGGACTGAGGTG-CCTACAATGTGCTTTAAAGTGTTTGGGACTCTCGTTTGGTTACCTGTTTGGATAGCTTGCTATGCTGACGG---AGTGATTGGTCGATTGAGATCTAGAGTAACTT-TTGCCGTTCATTCGGGTATTGGTATGACCAAGGCCGACCTCATCAACAACTTGGGTACGATCGCCAAGTCGGGCACGAAGGCTTTTATGGAAGCGCTGGCGGCCGGTGCCGATATTAGTATGATTGGTCAGTTCGGTGTCGGTTTCTACTCGGCGTACCTTGTCGCCGACAAGGTCGTCGTCCACTCGAAGCACAACGACGATGAACAATACGTTTGGGAATCCGCCGCTGGTGGTTCGTTCACCGTGACCCCAGAC---ACTTCGGAGCCAATTGCGCGCGGTACCCGCATTGTGTTGAAGCTGAAGGAGGACATGCTTGAATACTTGGAAGAGCGCAAGATCAAGGACCTTGTGAAGAAGCACTCGGAGTTCATTGGTTTCCCGATCAAGCTCTACGTCGAGAAGACGGAAGAGAAGGAAGTCACTGATGACGAAGAAGA------AG---AGGAGGAAGGCGACGATGACAAGCCTACCGTTGAGGAAGTGACT---GAGGAAGACGGTGAGAAGAAGAAGAAGACCAAGAAGATTAAGGAAGTGACCCACGAATGGGACCACCTCAACTCGCAGAAGCCAATCTGGATGCGCAAGCCTGAGGATGTGACCCACGAGGAGTACGCCTCGTTCTACAAGTCGTTGACCAACGACTGGGAGGAGCACTCGGCCGTGAAGCACTTCTCGGTTGAAGGTCAGCTTGAGTTCAAGGCGTGTTTGTTCACACCAAAGCGTGCTCCATTCGACATGTTCGAGGGTGGTGCCAAGAAGAAGCTGAACAACATCAAGCTTTACGTGCGTCGTGTGTTCATCATGGACAACTGCGAGGAGCTCATGCCTGAGTACTTGTCGTTCGTGAAGGGTGTTGTCGATTCGGAGGACTTGCCATTGAACATTTCGCGTGAAACCCTCCAGCAGAACAAGATCCTGCGTGTGATCAAGAAGAACTTGATCAAGAAGTGCTTGGAAATGTTTGCGGAGCTCGCTGAAGACACTGAGAAGTACAAGGCTTTCTACGAGGCGTTCAGCAAGAACTTG-AAGCTCGGTATCCACGAAGACTCAACCAACCGCACCAAGATTGCTAAGCTCCTTCGCTACCACTCGACCAAGTCTGGCGAAGATTTGACCTCGCTCGATGACTACATTTCGCGCATGCCAGAGAGCCAGCCAGGCATCTACTACGTGACTGGTGAGAGCAAGAAGGCTGTTGAGAACTCGCCATTCATTGAGAAGTTGAAGAAGAAGGGTTACGAAGTCATCTTCATGGTTGAAGCCATCGATGAGTACGCCGTCCAGCAGTTGAAGGAGTACGAGGGCAAGAAGCTCATTTCGGCGACGAAGGAAGGCCTCAAGATGGAGGAGAGCGAAGATGAGAAGAAGGCTTTCGAAGAGGCCAAGGCTGCCACCAGCGGCGTTCACGGCGATCGACACCCCCGGTCACCGGGATTTCATCCGAAACATGATCACTGGTACAACGCAGGCAGATGCCGCGTTGCTCGTCACTTCTGCCGATCCAGACGCACACAATGGCGAAGCTGC---GCAGTTTGGTAACGCGCATGAGCACGCGCTGCTGGCGTTCATGCTTGGCGTGAAGCAAGTCGTGGTCGCTGTCAACAAGATGGACCACGAGTCGGTCATGTACAGCCGAGCTCGGTACGAAGAGGTCAAGAGCAAGGTCGCAGGGTACTTGATTAATATCGGGTTCAAGTCCAGCCTGGTCCAGTTCGTACCGGTCTCTGGGTGGCTTGGAGACAACTTGATGAATCGCTCAACTAGCATGCCGTGGTACGACGGACCCACGCTGCTTGAGGCCCTGCAGCAATGGCGCCCGCCACGACGCTCCGTCGACAAGCCACTTCGGATGCTGATTGATAACTTCTACAAGATTAGAGGTGTCGGGATGGTGATCACTGGACGTGTGGAGACAGGAGTGTTGACACCCGGGATGACCATTTCGATCGCACCAAAGGGACTCACGGCGCAAGTCGTGTCGATAGAAATGCACCATCGACAGCTCTCGGAAGCCGTGGCAGGCAGCATTGTGGGCGTAAACGTCAAAGGCGTGGGGGCAAAGGACCTGAAGCGCGGGTTTGTGATCTCGGATCCGCAGAAGGA---CCCAGCGAGGGGTACGAAGAGCTTCACGGCCGAGATGGTCGTGCTACAGCACCCAGGCCAGATCCGACCTGGATACGTGCCAGTTCTGCATTGCCACACCAGTCAAGTTGCGTGCAATCTTGTTGCGATCACTCAGAAGATCGACCGGGAGACGGGCAAAGTGTTTGTCCAGAACAAGAAGCCTTTCATCAAGTCTCGCTTCTGCCGTGGTGTGCCAGACCCAAAGATCCGCATCTACGATGTTGGTAACAAGAAGGCGTCGGTCGACCAGTTCCCATACGTTGCTCACTTGGTGTCGGACGAGAAGGAACAGTTGTCGTCGGAAGCCCTCGAAGCTGCGCGTATTTCGGCCAACCGATACTTGACCAAGTACTGTGGCAAGGACAACTTCCACATGCGTATCCGTTGCCACCCATTCCAGGTGCTGCGTATCAACAAGATGCTTTCGTGCGCTGGTGCCGATAGACTTCAAACCGGTATGCGTCACGCTTACGGTAAGCCAACGGGTGTCGCCGCCCGTGTCGCCATTGGTCAGCCAATTATCTCGGTGCGCTCCAAGGACAACTTTGGCCCAGCCGTCGTTGAGGCCCTTCGTCGTGCACGCCGCCGACTCGGACAAGATCCCTGCCGACAAGCTCATTGAATTGTACGAGAGCTTCATCGCCAAGTGCAAGGAGTCGAGCCCGATTGTGTCGATTGAGGACCCATTTGACCAGGACGACTGGGCGTCGTGGGTCAAGTTCACGGACAAGGTGGGCAAGGACGTGCAGATTGTCGGTGACGACCTCACGGTCACGAACCCAACGCGTGTGCAGAAGGCCATTGATTTGAAGGCGTGCAATGCGCTCTTGCTCAAGGTGAACCAGATCGGCTCGATCACGGAGAGTATCGAGGCCGTGACCATGGCCAAGAAGGCCGGTTGGGCGATCATGACCTCGCACCGTTCGGGCGAGACTGAGGACTCGTTCATCGCCGACTTGGCGGTCGGTTTGAGCGCCGGTCAGATGGTAACAACTGGGCCAAGGGTCACTATACGGAAGGCGCTGAATTGATCGACTCGGTCTTGGATGTCGTCCGCAAGGAAGCTGAGAGCTGCGACTGCCTGCAAGGTTTCCAGATCACCCACTCCCTCGGTGGTGGTACCGGTTCCGGTATGGGTACGCTTTTGATCTCTAAGATCCGTGAAGAATACCCAGATCGTATCATGTGCACGTACTCGGTGTGCCCATCGCCAAAGGTGTCGGATACCGTCGTCGAACCATACAATGCCACGCTTTCGGTCCACCAGTTGGTCGAAAACGCCGATGAAGTCATGTGTTTGGATAACGAGGCTCTCTACGATATCTGCTTCCGTACCCTGAAGTTGACGACCCCAACGTACGGTGACTTGAACCACTTGGTGTGTGCTGCCATGTCCGGTATCACGACGTGCCTGCGATTCCCAGGTCAATTGAATTCGGACTTGCGTAAGCTCGCGGTGAACTTGATCCCGTTCCCACGTCTCCACTTCTTCATGATTGGTTTCGCGCCATTGACCTCCCGTGGTTCGCAGCAGTACCGTGCTTTGACCGTCCCAGAATTGACCCAGCAACAATTCGACGCGAAGAACATGATGTGCGCCGCCGATCCTCGCCACGGTCGTTATTTAACTGCTGCCTGTATGTTCCGTGGCCGCATGAGCACCAAGGAAGTCGATGAACAAATGCTCAACGTGCAGAACAAGAACTCGTCGTACTTTGTGGAGTGGATTCCAAACAACATCAAGGCCAGCGTGTGTGATATCCCACCAAAGGGTCTGAAGATGAGTACCACCTTCGTTGGTAACTCGACTGCGATCCAGGAGATGTTCAAGCGTGTGTCGGAGCAGTTCACGGCCATGTTCCGTCGTAAGGCTTTCTTGCACTGG

Pythium_vexans TTTTTTAGTTATTATAACAGTTTTTGTTTTTTGGATGTTATTTAGAGTTATTTATCTTTTTGATGAAAAAAAAAATAAAAATCCAGCAACTGTTATACATGGTGCTACTATTGAAATTATTTGGACTACTATTCCAGCTTTAATCTTATTAGTTGTAGCTATTCCTTCTTTTGCTTTATTATATTCAATGGATGAAGTAATAGATCCTATAATAACCGTTAAAGTTATAGGTAGTCAATGGTATTGGAGCTATGAATATTCAGATAATTTAGAATTTTCTGATGAACCTTTAATTTTTGATAGTTACATGGTTCAAGAAGATGATTTAGAAATAGGTCAATTAAGAGTATTAGAAGTTGATAATCGTGTAGTTGTTCCTACTAATAGTCATATAAGAGTTTTAATAACATCTTCAGATGTTTTACATTCATGGGCTATTCCTTCTTTAGGTATTAAATTAGATGCTTGCCCAGGTCGTTTAAATCAAACCGGCGAGTGAAGCGGGAAGAGCTCAAGCTTAAAATCTCTGTGCAAGTTTTGCATGGCGAATTGTAGTCTATGGATGC-GTTATCAGTGCAGCCGCGCGGGCCAAGTTCCTTGGAAGAGGACAGCATGGAGGGTGATACTCCCGTTCCTG-CCTGTGCGTGTTGTGCGTACGATGCGTTTTCTTTGAGTCGCGTTGTTTGGGAATGCAGCGCAAAGTAGGTGGTAAATTCCATCTAAAGCTAAATATTGGTGCGAGACCGATAGCGAACAAGTACCGTGAGGGAAAGATGAAAAGAACTTTGAAAAGAGAGTTAAAGAGTACCTGAAACTGCTGAAAGGGAACCGGGTCGTTGCCAGTGTCTATAATCTGCGGCATATTTCATTGTCGGATGCGGCCGTGCGGCGCGTTTGGTAGTGGGTTCGTC----CTGCACCGCGTGT----TG-CTGTGGTTGTGTTTGGTGGTGCCCTGTGCTGTGGTGGGACGTCAGAGTCAGTTCGTATGCTGCGGGAAATGGCTGCTGGGGAGGTAGGTCGGGGGTT-CGCCCTCGGCTGTTATATCCTGGTGGACT-AGTCGTCGTGGTTGGGACTGAGGTG-CCTACAACGTGCTTTTGAGTGTGGAGGTTTCTTCGTTTGCGCGTCGGTTGGATAGCTTGCTATGCAGTTGG---CGTTGTGTGCGGATGGATGTCTTTGCTAACTTGTTGCCGTTCACTCGGGTATCGGCATGACGAAGGCTGACCTGATCAACAACCTCGGCACGATCGCCAAGTCTGGCACGAAGGCGTTCATGGAGGCGCTCCAGGCTGGCGCCGACATCAGCATGATTGGCCAGTTCGGTGTGGGTTTCTACTCGGCGTACCTCGTCGCCGACAAGGTGATCGTGCACTCGAAGCACAACGACGACGAGCAGTACGTGTGGGAGTCTGCTGCCGGCGGCTCGTTCACGGTGACGCCGGAC---ACGTCGGAGCCGATTGCCCGCGGCACGCGCATCGTGATGAAGCTGAAGGAGGACATGCTCGAGTACCTTGAGGAGCGCAAGCTGAAGGATCTGGTGAAGAAGCACTCGGAGTTCATTGGCTTCCCGATCAAGCTGTACGTCGAGAAGACGGAGGAGAAGGAGGTGACGGACGACGAGGAGGACGAGGACG---AGAAGGAGGGCGAGGACGACAAGCCGACGGTCGAGGAGGTGACCGAGGAGGAGGAGGGCGAGAAGAAGAAGAAGACGAAGAAGATCAAGGAGGTGACCCACGAGTGGAACCACCTGAACAGCCAGAAGCCCATCTGGATGCGCAAGCCGGAGGACGTGACCCACGAGGAGTACGCGTCGTTCTACAAGTCGCTGACGAACGACTGGGAGGAGCACGCTTCGGTGAAGCACTTTTCGGTGGAGGGCCAGCTGGAGTTCAAGGCGTGCCTGTTCACGCCGAAGCGCGCGCCGTTCGACATGTTCGAGGGTGGTGCCAAGAAGAAGCTGAACAACATCAAGCTGTATGTGCGCCGCGTGTTCATCATGGACAACTGCGAGGAGCTGATGCCGGAGTACCTGTCGTTCGTCAAGGGCGTTGTCGACTCGGAGGACCTGCCGCTGAACATTTCGCGTGAGACGCTCCAGCAGAACAAGATTCTGCGCGTGATCAAGAAGAACCTGGTGAAGAAGTGCCTGGAGATGTTCGCGGAGATCGCCGAGGACACGGAGAAGTACAACAAGTTCTACGAGGCGTTCAGCAAGAACCTG-AAGCTGGGCATCCACGAGGACTCGACGAACCGCACGAAGATCGCGAAGCTGCTGCGCTACCACTCGACGAAGTCGGGTGAGGAGGTGACGTCGCTGGACGACTACATCTCGCGCATGCCGGAGAACCAGCCGGGCATCTACTACGTGACGGGCGAGAGCAAGAAGGCCGTGGAGAACTCGCCGTTCATTGAGAAGCTGAAGAAGAAGGGCTACGAGGTGCTGTTCATGATCGACGCCATTGACGAGTACGCGGTGCAGCAGCTGAAGGAGTACGAGGGCAAGAAGCTGATCTGCGCGACGAAGGAGGGTCTGAAGATGGAGGAGTCGGAGGACGAGAAGAAGCAGTACGAGGAGGCGAAGGCCGCGACGGAGGGTCTTCACGGTGATCGACGCTCCGGGCCACCGTGACTTCATCAAGAACATGATCACGGGCACGTCGCAGGCCGACTGCGCCATCCTTGTGGTTGCGTCGGGTGTTGGCGAGTTCGAGGCTGGTATCTC---GAAGGAGGGCCAGACGCGCGAGCACGCTCTGCTTGCCTTCACGCTCGGCGTGAAGCAGATGATCGTCGCGATCAACAAGATGGACGACTCGTCGGTGATGTACGGCGAGGCTCGTTACACGGAGATCAAGAACGAGGTGACTGCTTACCTGAAGAAGGTTGGCTACAAGCCGGCCAAGATCCCGTTCGTGCCTATTTCGGGCTGGGAGGGCGACAACATGATTGAGCGCTCGAGCAACATGCCGTGGTACAAGGGACCTTACCTCCTTGAGGCGCTCGACAGCCTGAACGCGCCTAAGCGTCCGTCGGACAAGCCGCTCCGTCTGCCGCTCCAGGATGTGTACAAGATCGGCGGTATCGGCACGGTACCGGTCGGCCGTGTTGAGACGGGTGTGCTGAAGCCTGGCATGGTTGCGACGTTCGGCCCTGTGGGTCTGTCGACGGAAGTGAAGTCTGTGGAGATGCACCACGAGTCGCTGCCGGAGGCTGTCCCGGGTGACAACGTTGGCTTCAACGTGAAGAACGTGTCGGTGAAGGAGCTCCGCCGTGGCTTCGTTGCTTCGGACTCGAAGAACGA---CCCTGCCAAGGGCACGGCTGACTTCACGGCCCAGGTGATTGTGCTGAACCACCCTGGCCAGATCGGCAACGGCTACGCTCCGGTGCTCGACTGCCACACGGCCCACGTTGCTTGCAAGTTCAAGGAGATCACGGAGAAGATGGACCGTCGTTCGGGCAAGGTGCTCGTCCAGAACAAGAAGCCGTTCATCAAGTCGCGCTTCTGCCGCGGCGTGCCGGACCCCAAGATCCGCATCTACGACGTGGGCAACAAGAAGGCGTCGGTGGACACGTTCCCGTTCGTGGCCCATCTTGTGTCGGATGAGAAGGAGCAGCTGTCGTCGGAGGCTCTCGAGGCCGCCCGCGTTGCTGCCAACCGTTACCTGACCAAGTACTGCGGCAAGGACAACTTCCACATGCGCATCCGTTGCCACCCGTTCCAGGTGCTGCGTATCAACAAGATGCTTTCGTGCGCCGGCGCCGATCGTCTGCAGACGGGTATGCGCCACGCTTACGGCAAGCCGCAGGGCACGGCCGCCCGTGTGGCTATCGGCCAGCCGATTATCTCGGTGCGCTCGAAGGACCAGTTCGGCCCCGCGGTCGTTGAGGCCCTGCGTCGTGCACGCTCCGGACTCGGAGAAGCTGCCGGCTGACAAGCTGCAGGAGCTGTACGAGGGCTTCATCGCGAAGACGAAGGACACGAGCCCGATCGTGTCGATCGAGGACCCGTTTGACCAGGACGACTGGAACTCGTGGATCAAGTTCACGGCTGCCGTCGGCCAGGACGTGCAGATCGTCGGTGACGACCTCACGGTGACGAACCCTACCCGTGTGAAGAAGGCCATCGACACGAAGGCGTGCAACGCTCTTCTGCTGAAGGTGAACCAAATCGGCTCGATCAGCGAGTCGATCGAGGCCGTGACGATGGCTAAGAAGGCCGGCTGGGCGATTATGACGTCGCACCGTTCGGGCGAGACCGAGGACACGTTCATCGCTGACCTGGCTGTGGGTCTGAGCGCTGGTCAGATGGTAACAACTGGGCGAAGGGACACTACACGGAAGGCGCGGAGCTGATTGACTCGGTGCTGGACGTTGTCCGGAAGGAGGCGGAGAGCTGCGATTGCCTCCAGGGCTTCCAGATCACGCACTCGCTCGGCGGTGGTACCGGCTCCGGTATGGGCACGCTCCTGATCTCCAAGATCCGCGAGGAGTACCCGGACCGTATTATGTGCACGTACTCGGTGTGTCCGTCGCCGAAGGTGTCGGACACGGTCGTCGAGCCCTACAACGCCACGCTCTCGGTGCACCAGCTTGTCGAGAACGCCGATGAGGTCATGTGCCTGGACAACGAGGCTCTGTACGACATTTGCTTCCGTACGCTCAAGCTGACGACCCCGACGTACGGCGACCTCAACCACCTTGTGTGCGCTGCGATGTCGGGCATCACGACCTGTCTGCGATTCCCCGGTCAGCTGAACTCTGACCTCCGGAAGCTGGCTGTGAACCTGATCCCGTTCCCGCGTCTCCACTTCTTTATGATTGGGTTCGCGCCGCTGACGTCGCGCGGCTCGCAGCAGTACCGTGCTCTGACCGTTCCGGAGCTGACCCAGCAGCAGTTCGACGCGAAGAACATGATGTGCGCCGCGGATCCTCGCCACGGTCGTTACCTGACTGCCGCGTGTATGTTCCGTGGCCGCATGAGCACGAAGGAGGTGGACGAGCAGATGCTGAACGTGCAGAACAAGAACTCGTCGTACTTCGTGGAGTGGATCCCGAACAACATCAAGGCCAGTGTGTGTGACATTCCGCCGAAGGGTCTGAAGATGAGCACCACGTTCATCGGCAACTCGACTGCCATTCAGGAGATGTTCAAGCGCGTCTCGGAGCAGTTCACGGCGATGTTCCGCCGCAAGGCCTTCTTGCACTGG

Saprolegnia_parasitica TTTTTTAGTATTAATTGTAATATTTGTTAGTTGGATTTTAGCTAGATGTATTTTTTTTTTTAATGAAAATACTAATAAAAAAGCTGAAACTTTTGTACATGGTACTGTTTTAGAAATTGTATGGACCATTACTCCAGCTCTTATTTTAATTATTATAGCAATACCTTCATTTTCATTATTATATGCTATGGATGAAGTTATTGATCCTATTTTAACTTTAAAAGTTATAGGTAGTCAATGGTATTGGAGTTATGAATATTCTGATGCTATGG---------ATGATTCTATTTTTTTTGATAGTTATATGGTATTAGAAGAAGATTTAGACAAAGGTCAATTCCGTCTTTTAGAAGTAGATAATCGTGTAGTAGTTCCTACTAATACACATATTCGTGTAATTATTACAGCTACTGATGTTTTACATTCATGGGCTGTACCTTCTTTAGGTGTAAAATTAGATGCTTGTCCAGGTAGATTAAATCAAACCGGCGAGTGAAGCGGGAAGAGCTCAAGCTTAAAATCTCCAT-----ACTTGTATGGCGAATTGTAGTCTATTGAGGCTGTTGTCAGCATCGCGATTGGGGACAAGTCCCTTGGAAAAGGGCAGCATAGAGGGTGATACTCCCGTCTCTG-CTCCAATCTGCGGTGTGTACGATACGCTTTCTTTGAGTCGAGTTGTTTGGGAATACAGCTCAAAGTAGGTGGTAAATTCCATCTAAAGCTAAATATTGGTGCGAGACCGATAGCGAACAAGTACCGTGAGGGAAAGATGAAAAGAACTTTGAAAAGAGAGTTAAAGAGTACCTGAAACTGTTGAAAGGGAACCGTATCGTTTCCAGTGT-TGTATCCTATG-CATATTTCATTCGTGG------------------------A------------------------------------------GCAATTTGCGGGTGCCCTGTGCATGGGTGTGACGTCAGGGTCAGTTTGTATGCTGCGGGAGATGGTCAGTGAGGAGGTGCGTCA-------CTTCGGTGAAAGTTATATCTCGCTGTCT--AGTAGCCGTGGTTGAGACTGAGGTG-CCTACAACATGCCTTAAGGTTCCGTGTTGTCTCGTGTTGGTGACTGCTTTGATAGCTTGCTATGTTTGTGG---TTTATTAGTGCGATTGATGATGCGGATAACTT-TTGCTGTTCACTCTGGTCTCGGCATGACCAAGGCCGACCTCGTGAACAACCTCGGCACGATCGCCAAGTCGGGCACGAAGGCCTTCATGGAGGCCCTCCAGGCCGGCGCCGATATCTCGATGATCGGCCAGTTCGGCGTCGGTTTCTACTCGGCCTACTTGGTCGCTGACCGCGTCACGGTGCACTCGAAGCACAACGACGACGAGCAGCACGTGTGGGAGTCGGCCGCCGGCGGCTCGTTCACGGTCACGCGCGACACGACGTCGGAGCCCATCCAGCGTGGTACGCGCATCGTCCTCAAGCTCAAGGAGGACATGCTCGAGTACCTCGAGGAGCGCAAGCTCAAGGACCTCGTCAAGAAGCACTCTGAGTTCATTGGCTTCCCGATCCGCCTCTACGTCGAGAAGACGACGGAGAAGGAGGTCACGGACGACGAGGACGAGGAAGAAG---AGGAGAAGGACGGCGACGACAAGCCCAAGATTGAGGAAGTC------ACGGACGACGACGACAAGAAGAAGAAGACCAAGAAGATCAAGGAGGTCTCGCACGAGTGGGACCACCTCAACGCGCAGAAGCCGATCTGGATGCGCAAGCCCGAGGATGTGACGCACGAGGAGTACGCTGCCTTCTACAAGTCGCTCACGAACGACTGGGAGGAGCATGCTTCGGTCAAGCACTTCTCGGTCGAGGGCCAGCTCGAGTTCAAGGCGTGCCTCTTCACGCCCAAGCGCGCGCCGTTCGACATGTTCGAGGGTGGTGCCAAGAAGAAGCTCAACAACATCAAGCTCTACGTGCGCCGCGTCTTCATCATGGACAACTGCGAGGACCTCATGCCCGAGTACCTCTCGTTCGTCAAGGGTGTGGTCGACTCGGAGGACTTGCCGCTCAACATCTCGCGCGAGACGCTCCAGCAGAACAAGATCCTCCGCGTCATCAAGAAGAACCTCGTCAAGAAGTGCTTGGACATGTTCAACGAGCTCGCCGAAGACGCCGAGAAGTACAAGAAGTTCTACGAGGCCTTCTCGAAGAACCTC-AAGCTCGGCATCCACGAGGACTCGACCAACCGCACCAAGATTGCCAAGCTCTTGCGCTACCACTCGACCAAGTCGGGCGAGGACCTCACGTCGCTCGACGACTACATCTCGCGCATGCCCGAGAACCAGCCCGGCATCTACTACGTGACGGGCGAGTCGAAGAAGTCGGTCGAGAACTCGCCGTTCATTGAGAAGCTCAAGAAGAAGGGCTATGAAGTCCTCTTCATGGTCGACCCCATCGACGAGTACGCCGTGCAGCAGCTCAAGGACTACGAGGGCAAGAAGCTCATCTGCGCCACCAAGGAAGGCCTCGACATGTCCGAGTCGGAGGACGAGAAGAAGTCGTTCGAGGAGGCCAAGGCCGCCACGGAAGGCCTTCACGGTCATTGATGCCCCGGGTCACCGCGATTTCATCAAGAACATGATCACGGGTACCTCGCAGGCCGATTGCGCCATCCTTGTCGTCGCCTCGGGCGTCGGTGAGTTCGAGGCCGGTATCTC---GAAGGA-GGCCAGACGCGCGAGCACGCGCTCCTCGCCTTCACGCTCGGCGTGCGCCAGATGATCGTCGCCATCAACAAGATGGACGACTCGTCGGTCATGTACGGCCAGGCCCGTTACGAGGAAATCAAGGAGGAAGTCTCGAACTACCTCAAGAAGGTCGGCTACAAGCCCGCCAAGGTCCCGTTCGTCCCGATCTCGGGCTGGGAGGGCGACAACATGATCGAGAAGTCCGGCAACATGCCCTGGTACAAGGGCCCGTGCCTCCTCGAAGCGCTTGACAACCTCAACCC-CCCAAGCGCCCCACCGACAAGGCCCTCCGCCTCCCGCTCCAGGATGTTTACAAGATTGGCGGTATTGGCACGGTCCCGGTCGGCCGTGTCGAGACGGGTGTCCTCAAGCCCGGCATGGTCGTCCAGTTCGGCCCCGTCGGCCTCCAGACGGAAGTCAAGTCGGTCGAGATGCACCACGAGTCGCTCCCCGAGGCTGTCCCGGGTGACAACGTCGGCTTCAACGTCAAGAACGTGTCGGTCAAGGAACTCCGCCGTGGTTACGTCGCTTCGGACTCGAAGAACGA---CCCCGCCAAGGGCACGGAGAACTTCACCGCCCAGGTGATTGTCCTCAACCACCCCGGCCAGATCGGCAACGGCTACTCGCCGGTGCTTGATTGCCACACGGCCCACGTCGCCTGCAAGTTCAAGGAGATCACGCAGAAGATGGATCGCCGTTCGGGTAAGGTCCTCGTCCAGAACAAGAAGCCGTTCATCAAGTCGCGCTACTGCCGTGGTGTGCCGGACCCGAAGATCCGCATCTACGATGTCGGTAACAAGAAGGCGTCCGTCGACACCTTCCCGTTCGTGTGCCACTTGGTGTCGGACGAGAAGGAACAGCTCTCGTCGGAGGCGCTTGAAGCCGCCCGCATTTCCGCCAACCGTTACTTGACCAAGCACTGCGGCAAGGACAACTTCCACATGCGCATCCGCTGCCACCCGTTCCAGGTCCTCCGTATCAACAAGATGCTTTCGTGCGCCGGTGCCGATCGTCTCCAGACCGGTATGCGTCACGCTTACGGCAAGCCCACGGGTGTCGCCGCCCGCGTCGCCATTGGCCAGCCCATTATCTCGGTGCGCTCCAAGGACTCGTTCGGCCCCGCCGTCGTCGAGGCCCTTCGCCGTGCACGCCCCGGAAGACCAGAAGATCTCGGCCGATGCGCTCATTGAAGTGTACAAGTCGTTCATCGACCAGTGCAAGGACTCGAGCCCGATCGTCTCGATCGAGGACCCGTTTGACCAGGACGACTGGGCTTCGTGGATCAAGTTCACGGAGGCCGTCGGCGACCACGTCCAGATTGTCGGTGACGATCTTACGGTCACCAACCCCACGCGCGTCAAGAAGGCCATTGAGACCAAGGCTTGCAACGCCCTCCTCCTCAAGGTCAACCAGATCGGCTCGATCTGGGAGTCGATCGAGGCCGTGACCATGTCGAAGAAGGCCGGCTGGGGCATCATGGCCTCGCACCGCTCGGGCGAAACCGAGGACTCGTTCATTGCGGACCTCGCCGTCGGCCTCAGCGCCGGCCAGATGGTAACAACTGGGCCAAGGGCCACTACACGGAAGGCGCTGAGCTCATTGACTCGGTCCTCGACGTTGTCCGCAAGGAAGCCGAGAGCTGCGACTGCCTCCAGGGTTTCCAGATTACGCACTCGCTCGGTGGTGGTACCGGTTCCGGTATGGGTACGCTCTTGATCTCCAAGATCCGCGAAGAGTACCCCGACCGTATCATGTGCACGTACTCGGTCTGCCCGTCGCCAAAGGTCTCGGACACGGTCGTCGAGCCGTACAACGCCACGCTCTCGGTCCACCAGCTCGTCGAAAACGCCGATGAGGTCATGTGTCTCGATAACGAGGCGCTCTACGATATTTGCTTCCGCACGCTCAAGCTCACGACGCCCACGTACGGCGACTTGAACCACTTGGTCTGCGCCGCCATGTCGGGTATCACGACGTGCCTCCGCTTCCCCGGTCAGTTGAACTCGGATCTTCGCAAGCTCGCCGTCAACTTGATCCCGTTCCCGCGTCTCCACTTCTTCATGATTGGTTTCGCGCCCTTGACGTCGCGTGGCTCGCAGCAGTACCGCGCGCTCACGGTCCCCGAGCTTACACAGCAGCAGTTTGATGCCAAGAACATGATGTGCGCCGCCGATCCTCGCCACGGCCGCTACCTCACGGCTGCCTGCATGTTCCGTGGCCGCATGTCCACGAAGGAAGTCGATGAGCAGATGCTCAACGTCCAGAACAAGAACTCGTCGTACTTCGTCGAGTGGATCCCGAACAACATCAAGGCCAGCGTGTGCGACATCCCGCCGAAGGGCCTTAAGATGTCGACGACGTTCATCGGTAACTCGACGGCGATCCAGGAGATGTTCAAGCGCGTCTCGGAGCAGTTCACGGCCATGTTCCGGCGGAAGGCTTTCTTGCATTGG

\## ALIGNMENT FILE ##

alignment = All_align_result.phy;

\## BRANCHLENGTHS: linked | unlinked ##

branchlengths = unlinked;

\## MODELS OF EVOLUTION: all | allx | mrbayes | beast | gamma | gammai | <list> ##

models = allx;

\# MODEL SELECCTION: AIC | AICc | BIC #

model_selection = aicc;

\## DATA BLOCKS: see manual for how to define ##

[data_blocks]

cox1_pos1 = 1-489\3;

cox1_pos2 = 2-489\3;

cox1_pos3 = 3-489\3;

nrLSU = 490-1207;

HSP90_pos1 = 1208-2614\3;

HSP90_pos2 = 1209-2614\3;

HSP90_pos3 = 1210-2614\3;

EF1a_pos1 = 2615-3489\3;

EF1a_pos2 = 2616-3489\3;

EF1a_pos3 = 2617-3489\3;

60S_pos1 = 3490-3922\3;

60S_pos2 = 3491-3922\3;

60S_pos3 = 3492-3922\3;

Enolase_pos1 = 3923-4328\3;

Enolase_pos2 = 3924-4328\3;

Enolase_pos3 = 3925-4328\3;

TUB_pos1 = 4329-5228\3;

TUB_pos2 = 4330-5228\3;

TUB_pos3 = 4331-5228\3;

\## SCHEMES, search: all | user | greedy | rcluster | rclusterf | kmeans ##

[schemes]

search = greedy;

#nexus

begin sets;

charset Subset1 = 1-489\3;

charset Subset2 = 2-489\3 3-489\3;

charset Subset3 = 490-1207 3925-4328\3 3492-3922\3;

charset Subset4 = 3490-3922\3 4330-5228\3 1208-2614\3 3923-4328\3;

charset Subset5 = 1209-2614\3 1210-2614\3;

charset Subset6 = 2615-3489\3;

charset Subset7 = 2616-3489\3 2617-3489\3;

charset Subset8 = 3491-3922\3 3924-4328\3 4331-5228\3;

charset Subset9 = 4329-5228\3;

charpartition PartitionFinder = GTR+G+X:Subset1, TIM+I+G+X:Subset2, GTR+I+G+X:Subset3, TVM+I+G+X:Subset4, GTR+I+G+X:Subset5, GTR+I+G+X:Subset6, TVM+G+X:Subset7, TVM+I+G+X:Subset8, SYM+I:Subset9;

end;

38 22340

Bremia_lactucae ATGACTACCACCGCCAAGTGGCCAAAGAGCTGGCCGCTTTCTTCCACATCTTTTACCGCTTTGGAGGAGTCTCCAACCGCTTTGATCTTTTTCTCAAGAATATTCTCGTGGGCTCCATGTGCATTGGAGCCGTCGTCTTCCTCGTCCTTTGCGTCATTGTCGTACGCCGCACCATCCTCCACATCCGAAGGCCGCTGCTCAGCGCACATCGATGACCACCTCGTCCATGGAGCTTCTCGCTGTTGGAATGCTTACATTCCTCGCAATCAGCGCCCTCGGGGGATTGCTAGGAGAAGGTATTTACAGACGAGCAATGCCGTGCGCTTTCATGCAGACCACTTGATCACAACATTTAATGACACTGAGCTGCCAGCTGATGCCTTTAAGATGTCGGTCGAAGCAATGGACTCGTACACGCGTCCAAGGACTTGTGGCACCAGTCGGATACATTGTTACCAAAGAATTACTCGGAAATTGTTCAAGATTTGGAATTTCGCTATTTTTTGCTTAAATCGTCCACAAATGGGGCCATTTTAGCTGTCGCACTTGCGAGCTTGTTGTCCATTGCATCTATTGGGTGGGCCTTGGTCTCGCCACTTCGTGTCTCGATGTTCATTCTTCTCAGCGTTATTCCGATCTCACACACGCTTGTTGGTGCCTATTTAGCGTCCACAATTATGACAGCTGACTTTTGTGCAGCCCCAATAAATAGTACGCTTGAAATTATTGGCACGTGTCCCGCAAACGCGTCATGCCATTTGGCAACGCTGTGGTGACTAAATGCAACTCACCAAGGAGAAATTGGCCGCCGTATGAAAATCGAATTTCTTGACCCCATTGGAGCAGCTGTGGGCGTCATGCTCGGGTATTTTGCAGGCACAGTAACTAATTGGGAGACGTTTTCTCTCATTCAGACGTGGCGCTGGCCATTTCTTCTTCAGTTTCTTGCGCTTTTGCCGCTTAATGTTGCCATTTTTTTCGTACCCCCAAAACATCTGTTGATCCGCCGGGAGACCGCTGACCACGAGCCGTCAATTGCCAGTACCCCCATGAGCACACGGTCGACGCCGTATCACGACGAAGAAGACGCGTTGCTGGCAAGCGCCAGCATGCAACGTACGGGGCCTTGGACACCAGGGATACGAACAAGGCGCGTTTGCGGACGGAATGTACGAATTGCTGCATCTCCCCATCTTCTGTTTGATCGTCTTTGGTCTGACGACCGTCTACTTTGTCGTCACGGGAGTGCAGTACTGGAGCACGATCTTTATGATCAAATCGCTCCATGCGTCCAAGTATTTGGTCAATGGCCTCTTTGTCATTGTCTCGGGCACGGGCCCGATCTTGGGCGTTTTCTATGGAGGGTGGCTGATTGACCGATACGGAGGCTATCTTGGCGTCGAGCAGCGAGCGAAGGCTCTGGGGATATGCATGATTCTTGGTCTCATTGCTTTCTTCATTTCAGCAGTGACGACGTTTTTTGAGGACATTTATATCACGGCGGGATGTCTGTGGCTCCTCCTGTTTTTTGGTGGAGCCGTCCTTCCCGCGCATCGCGCTCTTGCTTCATCGTTCTCCGTCATGATTTTTAACTTGTGCGGCTACGCGCTGTCGCATGAGTGACCTTCAGATGGTGACAGGTGCCAAGTGTTTCGTTCCAGACGAGACACATGTGTGGCTCGCGGCCGAGGTAACGCGCGACGAGAATTCGGGTGATGGAAAGACGCGCAAAATATATTGCAAGGTTGAGTTGCCCAATGGAGCTTGAGGAGCGCTGCGTCGACATGTTGTCCAAAAAGACCAAGGCCCTTCTGCTGTGCATCAGCTTGAGTCGCTTCCATACCAGAATGAGAATGTGGGTTCTGAGGGAATCGAGGACATGATTACACTTAATTACCTGCACGAGGCAGCCATTTTGTACAATGTCAAAAAGCGCTTTCTCTGCGAGTTACCGTATACTTACACGGGCGATATATGTATCGCAGTTAATCCCTATAAGTGGCTTCCAGATCTTTACGCGGAAGAGCAGCACATGCGCTACCTTAACCAGCCTAAAGAGGAGTTGCCGCCACATGTGTACGCCACATCCGTCGCTGCTTATGAGGGTATGCGATGCTCGGAGATCAACCAATCTATCTTGGTTAGTGGTGAGTCTGGGGCTGGTAAAACTGAGACAACGAAGATTCTTATGAATCATTTGGCTACAATTGCTGGCGGCCTTAATAATTCCACGATTAAGAAGATTATTGAAGTGAACCCGCTTCTTGAATCCTTTGGTAATGCCAAAACCGTCCGAAATGACAATTCCAGCCGATTTGGCAAATTCACACAGCTGCAATTTGATCTAAACGGCACACTTGTTGGCGCTAAGTGCCGGACATACTTGCTGGAGAAGACGCGTGTCATTCAGCACGAAGCGCCTGAGCGCAACTACCACATATTTTATCAATCGATTCTTCTGATGTTGCATCAGAGCTGCAGCTCGATCCGTCCAAGCATTACGCTTACACAGGGAACAATACGGCACAGAAGATCGAAGGCTTGTCCGATGAAAAACACTTTAACAAGACACGCGAGGCTCTCGAACTAGTCGGTCTTTCTTACGATGACCAGCAACCATTATTTGAAGTGCTAGCAGGTGTGCTCCACCTTGGTGAGGTACAGCTGCTATCGGATCCGACGGATGACGAAAAGTCTCTTATCAGTGAGTCTGACGATGGTGCACAGTCCACCACGCAGATGCTTGGTGTCACATGGGAGGCTCTTCAGACGGCGCTTTGCTCGCGTACAATGCGTGCCGTGAATGATGTATACAGCGTGCCGCTCAAGAAAGAGCTGGCCATGGACTGCCGTGATGCTCTCGCTAAGGCTATTTACTCGAATGTGTTTGACTGGCTTGTGACTACTATTAATCAGTCACTCGCTGACGATGCCAATATGGCTAATCACGTGGGAGTGCTCGATATTTTTGGTTTTGAGCATTTTAAGCATAACTCATTTGAGCAGTTTTGTATCAATTTTGCTAACGAGAAGTTGCAGCAAAAATTTACGCAAGACGTGTTCAAAACTGTACAGATTGAATACGAGGAGGAAGGGATCGTGTGGGACCACATTGAATACGCGGACAACCAGGATGTTTTAAATGTGATTGAGTCCCGTATGGGGATTATTTCGCTGTTGAATGAAGAGGTGATGCGGCCGAAGGGTAATGAAGAGTCATTTATGTCCAAGGTGACGTCATTGCATAAGGAG---GATATGGCACACGTCATTGAATTTCCTCGGACGTCGCGGACCGAATTTCTTATCAAGCACTATGCTGCTCCCGTAATGTATGACTCAGTTGGGTTCCTTGAGAAACACAAAGATTCGTTGCTGCCTGACTTATCCGAGCTTATGCGGGGTTCTGACAAACCATTTATTGCCAAGCTTTTTGAGCCGAAGGAGGATCCTACGTCGACGCAAAC---------GGGGTGGCGCTCTGTCCATCACGACTGTCGGCACTCAATTTAAAGAAAGTCTAACGGAATTAATGGCCAACATTAACTCGACTCATGTGCACTATGTGCGTTGCATAAAGCCCAATCCGATAAA---------GAGCGCAACGGTGATGGATCAAAGCATGGTGGTGTCTCAATTGCGGTGTGCTGGTGTCATTGAAGCTATTCGCATTTCCCGTGCAGCCTACCCGAATCGGTTGCAACACACAGAAATCCTCAACAAATTCTGGCTGTTTGTGCCTAGTGGCGGCAACACTGTAGAAGAAAAGTGCCAGTTGCTCATGGACAAGCTGAAATTGGAGACCCCCAGGCAATATCAGATGGGAAAAACTCGCGTGTACTTCCAGTTGGGAGTGCTCGAAGAACTTGAGGACCGTCGCAAAAAGTTCCTTGATGCCAAGGCTACGTTTCTGCAGTATACCATGGTTGGTTTCACGCAACGTATTAAGTATCTTAGAAAATTGGAAGCAATCATTAAGTTCAGTTATTCGGTGCGTCATTGCCATGCGCAGGTACAACACCTTTTTGAAAGGCCTCATTTTGGCGCAAGCCCAATGGCGAGGCATTCAGGGACGCAAGGTAGCTGCCGACGTTAAGTGCAACCACTATGCAGTCATTATCCAGCGATATATGCGTGGCCTCGTTAAGCGTCATCGCTACGTAAAAATGCGCGCGATGGTTATTCGTGTGCAGTCTATGGTTCGTATGATGATACAGCGACCAAAGTATTTGGCTGCGCTTGATGAAAAGCGACGTGAAGCGGATATGGCTTATCAGCTACATAAGTTAAAGGCCGCTCTGCAGGACGAACAAGAGCGAAATGCTCAGCT------GCAGCGCC---GTAATAGCGTGAC--AACTGCGCATCAGCTGCCTCAAGCGTTGTCATGGCTGATGCTGGTGGGATGATCGAGACTCTAACAGACGAGAACAAAAAGCTCCGCGAGAAGAACGAGGTCATGAAAGTCTCCATGAAGGCCTTAAAAGCCGAAGTCGAGAAGTTTAAAAGTGATAAAGAGGTATCATCAGCTGGTAATCACGTAAAGCTCCGTCAGTTGCAGGACACTGTGCGTGAAAAGGACAAAAGAATAGTGCAGCTTGAGGCTGACAACAAAAAGCTGACTGAGCAGATCGCGAAACTTCATGCAGATGGTATCGT---TCCTGAGATAAAGGCTACACCTAAGAAGTCAATCTTTCGCACGCTGGGCTCCAAGAAGGAGACAAAACCGC---------------GCGAAACCGTGCTGATGGACTCGCTGACTGGCTCGGATGATTTAAATGGGCGGGAGACTCGACAGTCGGA---GCGTCATTTTCCGCGCATGC---CGAGCATGAGTGTAGGAACT---CGTTTCTGGAACACAAATAAG---AAGATTGAA------------AACGGTGACGGTCATGACGTCTCCGAGGTCGACGGTGAAGATAGCCCTACTAATCTACGCGCAATACGATTGTAAAGAGCATGGAAGTTGGGGTTACTGGTGCGATGTCGAGTATCAAGGGTCGTATG---------TCGGTCGTGAAGGGTATGTACGAAGCAAGCGAACGAATAGGATCGCCCAGTGCGTGAGTCGTTCAATTTGGACGCGTTGCCAGAGGTTTCGTTGCCTGTTGGATGGGAGGCCAAGGTGTCACGCTCCACTGGCCGTGTGTACTACGTAAACCGCAAGCATGGCAAATCACAGTTCGAACGCCCTACTCTAGCCTCGCTTAA------GGCCCAAAAACTAGCGCGCCAAAAGTCAGGTAGCGCCGATTGAGGCTATAGTCGCTGATTTTGGCTGTGGAGATGCCAAATTGGCCGAGAGCGTTCCAAACAAGGTGCATTCGTTTGACTTAGTGTCCCTTGACATTGCCATATATTGCTTGGCACTGATGGGTAAAAGTGTGCGTGAGTATGTGCGTGAAGTATACCGTGTCTTGAAGCCCCAAGGCGTGCTGAAGATTGCAGAGGTTCAAAGTCGCTTTGAGAGTGCGTCGATAGGTGGATGGTGGCGGTATTTGTATTAACTGCAAAGGGTGTGGTAGTGACAATTCGAAACTCGACAGTATTTGTGAAGAGATCAAGTACGTCATCATTGTAGGTCAAAAGACCGGCGAGGCCGTGGCCATGCCGTTTCCCGATATTAGCCGCGATTTAAAGACATTGCTGGGCCACACCACATCCATGATCACCGACATCGCTGTAAATCTCGTCGCTGCTGCTGACAGCGGATCGTGATGAAAAGCTTCGCGTGTCCAGGTTTCCCAATGCTGCGATTATCGAAAGCTATTGCTTGGGACACGCAGCGAGTGTCACTAAAATCGCATGTAGTATAGTGACACCAGCGCTGGTGGTGTCGACGTCCATGGACAACACGTTAAAACTGTGGCAAATGAGCACAGGCACGTTTGCAATTTGTCCGAAGACAATATTGTTGCAGTGCTTGTGAATAACGAATTTTTACGGCTTTTCAGTATCGTTAAAATCCCTTTTTGCAGCTATTCACGATTTCGGCAAGCGCCCACGGCGTTTGATCGCTTTGCGTGACTTCCGAAAGCTGCAGAACGATCCACCTTCGGGTGTTTCCGGTGCTCCGATGGATAATAACATCATGCTATGGCAGGCTGTCATCTTTGGACCGGATGATACGCCGTGGGAAGGCGGTACCTTCAACTTAACGCTCGAGTTTAGCGAAGATTATCCCAATAAGGCGCCGACGGTTAAGTTTATCACCAAAATGTTCCATCCAAATATCTACAACGATGGCCAGATCTGCTTGGATATTTTACAGAATCAGTGGTCGCCGATCTACGATATTTCTGCTATATTAACGTCCGTTCAGTCGCTTTTGTGTGATCCAAATCCGAACTCGCCGGCCAATTCGGAGGCTGCTCGCCTATTTCAGGAAAATCGACGGGAGTACAATCGCCGCGTGCGTGAAATTGTCGAGCAAAGCTGGCAGGCGATCGATGGTATCGGCCGCAAAGATGCGTGGTGCGGAGAATCGCATGAATGCCGGTCGCCCCTTTACCGCATGGTTAGACAATGTCTCGTCGGACCGTGGTCTCTGTGGTGGTATTAACTCGGGTATTGCCAAGACCACCCGCAAACAGATCATTCACAGATTTTTGTGATTGGAGACAAGGCCCGTGCTCAGTTGCGCCGCGACCTTGGGGACCGTATACGTGGCAACGTCACGGAGACGTTCCAATCGCCTCCCAATTTCACGGTCGCCAGTGCCATTGCCGAGGCTGTGGAATCGGAGAGGAGTCGTCTCGCATGGCGGCGATGGAGAATGCGACGACGAACGCAGAGGACCTCACGCTCACCCTGGTGTACAACAAAGCTCGCCAGGCCCGTATTACCACCGAGCTCATTGAAATTATCTCGGGAGCTGCTTCGCTCGATAGTGACTGGTAGCGTCGTGAGCGTCCAGAGCGTCGCGCAATTCGATGAAGCGACAGCACGTGAGTCAATGCTGTCGGTGAGTTTCTTTTGGGCTGATTTCCAAGCTTGTCGTCCAAATGGACAGCTCGATGTGGGGCCATAGCGTGCTAGAAAAGCTTGAAGGTGCCAACGTGGCCGAGCTGGCCAAGCGTGTCGGCAGTATCGTCTTAATCGACTTATTAATGCATCCCCTGTGATGCTCTTTATGAAAGGGACTCCCACGGAACCCAAGTGTGGGTTTAGTCGGCAAATCGTAGCGCTTTTAGATGATGAAAAGATTCAATATGGCACCTTTGATATTTTAACTGACGAAGACGTGCGTCAAGGACTTAAGGACTTGTCACAATGGGCTACATTCCCGCAACTTTACATCCACGGGTCGTTTGTAGGTGGATTAGATATTCTTCAAGAAAAACAATCAAAAGGATTGCTAATTGAGCAACTCGGGGTCGAAGAAGCCACAGCTACGGTTGAAGCGAATTTACGTGATCTCGTCGCGTCGGCACCAGTTTTACTCTTTATGAAAGTTTGATATCTTGTGTGGAGCTACGAACGCCAGGACCAAGACATTTCAAAGGTTGTGACGCTCGCGCCCTCCTCGTTCCGTCGGGAGATGTGCGGAGGCGATTGCGTGGAGTCCGAAATCGCATGGCGATGGCATTTCGTGACCCTGTGCAGGAGACGGGAGAGTTTTGTATTGTGCAGCTCCTCGCGTCACGTCCAAGCGCACGACTCTTCATTCCCGCGGTCTCTCAGCTATTGACTGGCAAGTTTAATGTGCTCCACCGGGACCATCGCAGTGAAGTCAAGTCGCTTTGTATCCATTCCAAGCGTCAATGTTTGTACACAGGCGGTATGGATGGGCTACTTATACGATATGATCTGAACAAAGGCACTAGTGATGTCGTAATCGAGCGTCGTAAACACATTTCCAAGATTAATTCGGTACTTGAGCATCCACATAACCCGAATTTGCTTCTAGTGAGCTCGGTAGAATCGTCGGTACAATTTAATACTGCATGATCTACAGCGATATGATTCAAACGTGGCAGGCAGCTCTATGAGTCAGTACGTCGTACCACGGTGGTCGCCTGCAGGCTACCACGTTTCATGCGGTAGCAAATGCGCTATTGCCACGGCAGCTTTTCAACTCCCACGTCAGCCACCATCGGGGCCTCGTTCTTGCAGAAGCGTGTGATTGTTGGGGGAAAGCTGACGCTACAAATTTGGGATACAGCTGGTCAAGAGCGCTTTCGCTCGATGGCGCCAATGTACTACCGCAATGCTAAAGCCGCCATCTTGGTCTTTGACCTACAGAGCGAAGCCAGCTTCGAAAAAATTCAGGAGTGGCTGCACTTGCAGCATCACGTTGGCGACGACATGTTGGCAACAAGAGCGACGTTTTGACTTCACAAAGGCCCAAAAGCTTGCGAATGAGATCGGTGCACTAGTGTTTCGCACTAGTGCCAAAACAGGGGAAGGAGTGCAGTCGCTTTTTGAAAGCCTGGCGGTGCAGCTGCTAAAGAAGCACGACCAGGAACAGCGGAGAGCTTTTACGTCGTGTACGTTCCGGCTGATGAGACGAAAGAACTAGAAGAATGGACGATCAAGCTTCCAAAGGACAAGGAGGCACAGCTTGGCTGTTTGACAGAGCGTCTGCGTGCTCACTACAAGAGCCGCGCCATCCAAGGAGCAACAGAGACCTTTAAGCAGCAATTACTGGCTCAAATGCCCAAAGGTGCCACACTCACAGATACTATGCTTGAGATGATGCATGGACAGTCTCGTGGACTCAATCCCGCTTGTCCTCAATACGGCTGAAGTCAAGCACGTTTGAGGTGAAAGGCGATGCATTTCTTGCTCGGGTTTTCGATAGACGACTTTGTGCGCCTGGATTTTCGGTTAATGGAAGTATCAGGTGATGCACCGTGGGTGCAACTGGCCAAATCGCAGAGGTCACGCAGTCTCTCGAAGATATTTTGGCATTAGGCCAGGGTCTGGACGATGAGGACTACAAGGCCGAGCTCGAAGTGCTGCAGAACGATCCGAACTCCAATCTTTACTCGGCTGTCACCTTTGAGGAGCTAAATCTACCAGAGCAGCTTCTAAAGGGCGTTTACAATATGAAATTTGCCAAACCGTCAAAGATTCAATCAGTAGCGCTCCCACTCATTCTCGCCAATCCGCCAGAAAATCTAATTGGCCAGGCGCAGAGCGGCACCCCACCCGTGAGTTAGTGCGTCAAATTATGGCTGTGGTTAACGCGATGGGTAAATTGTGGACACATTCCTGGCCATCCCTGGCAACGACCTCGCTCGTGGGGAGACTTTAAAAGCTCAGGTTATTGTGGGTACCCCCGGCAAGGTCGAGGGTATGATTAAGAAGAAGCAGCTGGAGACTCGTGATGTCAAAATATTTGTGCTGGATGAAGCCGACGTGATGGTTGCTGAAGACGGCATGCGCGAGCGCTCCGTGGCAATCAAGCGATTTATTAAGAATCGCGCATGTCAGTACCTGCTCTTTTCAGCCACATATGCGGACGAGGTGCGCGACTTTGCGCAAAAGATGGTGCCGGACCATAATATTATCACGGTCAAGAAGGAAAAACTCACTTTAGACGGTATCAAGCAGTTCTGGATCGATTGCAAGTCCCACGGTAACAAATTTCAGGTGCTGTCAGACATCTTTGCCATCTTGAGCATTGGCAAATGTGTCATTTTCGTACAACAGCGCGAAACGGCAAAAGAGTTAACACACAGTATGCGTACCAATGGCCATTCCGTTGGTATCTTGCATGGCGCTGACATGGCCAAGGAAGTGCGTGATCAAGTGATTGACGAGTTTCGAGCTGGTACAACCAACGTGCTGATCACCACAAACGTGCTTGCCCGTGGTATTGACGTAGCAGGTGTGTCACTGGTGGTTAATTTCGACATTCCATTGACTCGGGACCGTCGCCCCGATCCAGAGACGTACCTGCACCGTATCGGTCGAACGGGCCGTTTTGGTCGCAAGGGTTGTGCGATTAACTTTGTCCATGACGACAAGAGCAAGCAGGATCTTGCTGAGATTGAAGAATACTATGCACGCCCGATTACGCAGGCACCTTCAGATGACATTGAAGAACTTGAGAAATTGCTAGCATGGGGGGTAGCAAACGTCGGCTGGCCAAGCTTGGGAAGGAGCAACGCAAAGGCATGCGCATTTATCCACGCGAGCCCAAGAAAAAACCCTCGGGCACCGACAAGACATATTACTACTTGAAAGACGTCATGTTTCTCGCGCATGAGCCCGTCTTGCATCACTTTCGTGCATTCAAGACGTTTTTAAAGAAAGTGACGAAAGCCATGGGCAAGAAAGATTACAGCACCGCCATGAAAAAACACGAAAATCGTCCATTGTATGAACTAAATCACATTGTCAAAGAACGGTATCCTCGTTTCGTCGATGCGCTACAAGACTTGGATGATGCCTTGTGTGGCAAAACTATATTGTCGAATCCCATGCATTGACCAAAGTATTTGTAAGTATCAAGGGCATTTACTACCAAGCAATTGTTCACGGCCAGATTCTTACGTGGCTCGTACCCCATCAATTCACACCCACGGTCGATAAACGTGTCGACGTGCGTGTAATGCTCACGTTTCTCGAGTTCTATCAAGTCCTCCTTAAATTTGTAAACGCTCAGCTGTATCTGCACCTTGGCATTGCATATCCTCCCAAGATTAATTTGCAATTGGATGCCGCAGGCGCGCAATTAGCGGCATTAAAACTACCAGCAAAAAGAGTCGGAAAAACGCATTAAACTTTCCCAACTCATCCAAAGAACCAACTCCACTGCATTTGTTTACGAATTTGACGTTTTTTTTATCGCGCGAAGTGCCATCCGCGTGTCTTGAACTCGTGCTTCGATCCCACGGCGCCACATTGGCTTGGAATGGTGTCGGGTCTCCTTATCCTGAACAAAGTCCTCGAATTACGCACCACGTGATGGATCGACCGCATCAAGGTCATCGGTACTTTAATCGGGAGTACGTGCAGCCTCAGTGGATCTTTGATTCCGTGAATAATGGCATGCTTTTGCCATTGACGCGGTATCGCAAGGAATTAGAGCGACTTAAGTCGGCAACTCAAGTGTTGCGGGAAGTCGGAGTAATTGAGGCGGAAGAAGACGATGGGAGATGTATGTGACTGAGTTACAAGCGGAAAGGCATGGCAATCGCGTGTCGAACAAGAAGCAGAAGGAGGACGAAGCGAACGAGTTGAAAGAACTTGCCAAGACGATGATGTCGAAAAAAGCCAAGCGGTTGTATGACCGGATGCAGTATGGATTGGGGAAAAAAGCCGACAAGATACAGATGCTGGAGGATAAACGACGTAAGATTGCTGCAAGACAAAAAAAGTAATAGCTGGTGCATGTTCAGGCATGATTTCGGCGCTAGCATTTCAGCCATTGGGGTGCTGCGATGGCGTGGCACGTCGCCCACTCTCATGCGCGTAGCTGGCGGTGCCGGGCTTTACTTTGTCACGCTTGACCATTGTCTGAACATGTTCCCGTCATCAGCAATCAATACATTTTTGGCCGGAGCCTTTGCGCGTACTTTTGCGGGCAGCATCATGTCACCCTTAACAATCGTCAAATCATGGCTGGATGCCGCCCGGCACATTTGATAGTAAGGGGACGTGCCATTTTCTGGGCTGTATGTGCTCGTATACACAACACCCAGCTGATGTGGTCAAGACACGCATGCATGCGGCAAACGGTGAGCAAAATATATCGCTACGAGGGTCTTTGGGGCTTTGCAAAGGGCATTATTCCGCGAGTCGCCAAACGAACGCTGTCAACCGCAGTCACGTGGACAATTTATGAGCTACTCCTTCGACCCGTCAGGTTTGGGCCACGCTAGTGAAGCGTTTAAATTGGCCAAGGAGACGGAGCATACGAAGCAGTTGGAGAACCAAGTCAAAATAAAGGAGAACGAGGCGCTCTACAAGCAATATGAGATCGTACGCGTACAAAAGGAAGGCGAGGAGCGCCGCAAGACACTTGAGGAAGAAACGCGTCAGCACCAGCAGCGCGCTCAGTACCAAGACCAGCTCAAGCGCAAGCAGTATGCTGACCAGCTTGCTGCACAGAAATACATGAAGGAGCAGGAGCTAAAAAAGCAAGAAGAAATCCTAGCTCGTCAAGAAGCCTCTCGCCGGAAAACGCTAGATTACGAGGCGGAGCTGCGTCAAAAGACCGAGTTAGCTAGAGTTGCGGCTGAAACTGAAGGCCGCATTAAGCAAGAGCGTCTCAATCACGACTTGCACTTGGAAGAGGACCGCGTGCGTGCGAAGGAGCATCGTGAGACTGTACTGGAGGCTATTAAGTTGGCAGGCAATACTGTCGGCTCGGGTATAATGACTTTTGTCGGCGACAGCGAGAAACTTACCGCAACTGTGGTGTCGCTGACGGCATTGGCTGTCGGTATCTACACTGCTAAAGTTTCGACGAACATTGCGGGCAAGTACGTCGAAGCACGTATGGGAAAACCATCTCTAGTGCGTGAGACGTCACGTCGCTCGGTAGCGCAGGTGGTTGCCAATCCCATTCCATCGATCAAGCGTTCGCTGCGTCTTCAAAAAGCAACGAATGCCCTTGAGGGCGTCGTTTTGGAGCCAAAGCTTGACGAGCGACTACGCAGCGTGGCGTTGTCAACATTCAATACGAAAAAGAACCGCGCGCCATTTCGCCACTTGCTGTTACACGGTCCACCTGGCACTGGCAAGACGCTATTTGCAAAGGCACTGGCTCGTCACTCAGGTCTCGAATACGCTATCCTTACTGGTGGAGATGTTTCCCCGCTAGGCCGCGAAGGTGTCACGGAGATTCATAAGTTATTCGATTGGGCGTCGCACAGCCGTCGGGGTCTGCTTTTGTTTGTCGATGAGGCCGATGCTTTTTTGCAGAAGCGAAGTAACACCGTCATGAGTGAAGACATGCGCAACGCGCTAAATGCTTTTTTGTATCGTACGGGTGAAGCAAGCGACAAGTTTATGATTGTTTTTGCGAGTAACCAGCCAGAGCAATTTGATTGGGCTATTAATGATCGTATTGATGAGATGGTCGAATTTCGCTTGCCTGGTTTTGATGAGCGGGTACGCATGCTAAAACAGTATTTTGAAAACTACATTCGGGCACCGAAAAACTCGCGTGCTAAGAAGATATACGTGAAGGTATTATCTTCACGATGGACCCCAAAGCCAACTTCCTCGTGCCTTGACTCATATTTTTTATCTCGAGACACTTAAGAACGTGTCCGGGGAAGCTCAGACAGTTTTCGATGCTCTTAACAAAAGTCCATGCGCATCTATTGGATTGTTTTTACGGTAGTGCTTGGCAATGGGTTATACACGTCCTTCACTGGCAAGGACGAGTGGTTTTTCGTGAAATGGCTCACGAGCAACACCCCATCCGAGTATCGCATCTCTGAGAAGTGGGACAAATGTTTGGAGAATTTTACGCTTTATTTTGGTGCCGGCCTTGTCGCGGGAGGGCTCACATCCCTTGTTTTGGCGCGCACTGGTACTGGCCGTGGTCTGATTACAGGTGTTGGTGTGCAGGCGCCGGATCGAGCTGGAAAACGTGTCAGATGGCATTCGCAGGAGATGCACAGGCATCCCTGGATAAGGCCGATAAGATCGTGGAAGACTTGCCAGCATACCTGCAGCAGACGATTGTCAATTTAGTGACGGCCTCGACTACACAGCAGGCGCTGCAAAATATGCACTGTTGGATTATGTAAACAAGATTGCATCGAGGTACTTGAGACCTACTTGGCTGAGCAAGTGGAGAATAAGACCGTGGATATTGATGCCAACCTGGCGTTACTCAAGCTGTACCAAATGTACCCGTCCGTGGCCAGCGCCAAGAGCCCTTGTTCTTATAAAAGGAATTATGGCTCTTCCCTCTACCTTCTTCACTGGAGTCTACGATGGTTCCGGAAAATATCCGCGAGGATGCCAATGTATCTGCAGCGCTACGAGCGGGCTTTATGCTTCAGTCATGTCTGTTCGAGGAGTTTTGGAAGGAGCGAGGGACACTTGCCGATGAGGTGCCAGACTTTCTAGAGTCCGTGCGCGCCTATATTCTAACGGCCGTTAGCCACAGCCATAGCGTCATCTCCACACAGGTCATGAAGGCCAAATTAAGCGTGCCTGACAAAGAGGTGGCGGATATTGTTGCTATGCAATGGACGTAGACGATCTGATTACTATTGATTCCAATGAAAATAATCAAATGCAGGCCAAGAAAGTTCAGGAAAAAATCGATTTTGAGGATGTGCTCAAGGTCATTCGTGTCTTATCAAGATCTAATGTATGTGCCAGTGGTGGTGGAGTGGCGGATGCCGAGGAAGTGTGTGAGAGTCTTGCGAAGCAGCTGAGGCTTAAACGAGGATCACAAGTGCTGCCCTGTTCGACTGGCGTCATTGGCTGGCGTATTCCTGTGGACGCTATGGTCGAAAATCTCCCGAAACTTGTCAAAAATTTGCAGTCCGACTCGGTATTACCAGCTGCTGAGGGCATCATGACGACCGATTTGTACCCAAAGATTCGCTCTATGGACGTATGTGGTGGTCGAATTGTTGGGATTGCCAAGGGTGCGGGAATGATCGAACCGAATATGGCTACCATGTTGTCCTTCATTCTTACTGATCTGTCGATTTCGCGTGACGTACTGCGGCAGCTCCTGGCGGATGTGGTTGGTGACACGTACAATTCGCTAAGTGTGGACACCGATCAAAGCACATCAGACACGGTAACGCTCGTGTCGTCAGACCAAATTCCGTTTGACATGAA---GCAGTTGGACAGGTTTAAAAAGGCTTTGCACGACGTGTGTCTTGGATTAAGTGAAGACATTGTGCGTAACGCTGAGGGGGCACATCACGTAATGCGTGTGCGTGTGAGCGGTGCATTGTCCAAGGAGATGGCCAAGGGAGTTGGAAAGTCTGTGGTTAACTCCCCTTTGCTCAAGTGCGCGATAGCGGGCAATGACCCTAATGTCGGCCGTCTCGTTATGGCTGTGGGTAAGTACATGGGTCTCCATTTTAAAGACGTAAATATTCAGCGCCGTATGAAGATTCACATGGGTGGCGTTCTCATTTTCGAAAATGGTGAGATGGTACTCAACGACGAAATTGAAGCCAAACTTGTGGCTCACATGCGCGATGCGATGCTAACTGAGCCCACGCGCGGCGGACTCGATGCGAGCTCGCACAATTTCCCTCCGCATGGAAAGTTTGTCGAGATTAAAATAGACTTGGGAGTTGGCTCTGAAGAATCGCAAGTGCTGGGGATTGATTTAACTCACGAGTATGTGGCCATCAACGCCGACTACCGCTCGTAATGGCGCC---GAAGAAGGGAAGGACCGCCGCCGCCTACTTAGCAACTTTGGGCGTGGCCCCTTC---CCCCGGAGGTGAAAAGAAAGATGAC---AAGAAGAAGAAAAAGAAAGGAAAGAAAAAGCCCAGAAGAAGCCCACGTCGGCTGCAGCAAAGGCCGTGA-TGGAGCGCCAGGCAGTTATGAAAGCTGAGCAAGAGCGTCTTGAAAGGGAACGTATAGAACAGGAGCGAATCGATCAGGAGGAGGAGGCAAAGCGCTTGGCTGAGATTGAGCGTGAAGAGGAGATACGTCGCAAGAAAAAGGAAGCGCGAGCTGCCAAGATCGAGCGTCAGAAAAAGGAAGGCACGTACATGACCAAGGCTCAAAAGCAGAAAGCAGCCAAAGCTCAAGCTGCTCTTGAGACCATGCAGCAGGTTGGTTTGGTGCCTGAAGCT---------GGGGATAAGAAGAAGGTTTTGTACTCGAATAACAATAAAAAGAGCTCGAACAAGAGGAAGAAGCGCTCGAGGAGGTTGTAGCTCCCGACGCATGGGACGACGAAGACTGGGAGACTAGCAAAGTTGTGGCATCGTTGGAGGACAAACTAAGAGAAGAAGAAGCAGGACTACTTGCGGTTGCAAGGCATTGCGCTCAAAAAGCGACTAGAAGAGCAGGCAGAACAAATGCGTCAGGACGATCACGAAGAACGACAGCGTTCGAAAGCAAAGGTCGAGGCTGAGCGTAAGAAAAAGGAAGCGGCCGAGCGTCGTCGCCAGCGCGAGGCCGCAGTGCGCGCTAAGGTGTCATTAGATGATCTGCGCTCCCCTATCTGCTGCATCATGGGTCATGTCGACACTGGTAAGACAAAGCTTCTTGACAATATTCGCCGGACCAAGGTTCAGGACGCGGAAGCTGGTGGTATTACCCAACAGATTGGGGCTACCTTTTTTCCTGTTGATGCGATTAAACAAAAGACGGCTCGACTTGCCGAGACAATGAAGCTTGAGTATCGTTTACCTGGTCTGCTTGTGATCGATACACCTGGTCACGAGTCGTTTACAAATTTGCGGTCTCGCGGTTCGTCGCTGTGCGATATTGCTATACTGGTGGTGGATATTATGCACGGTCTGGAGCCACAGACTTTGGAATCACTGCGCTTGCTGCGACAGAAGAAGGCCCCTTTCGTTGTGGCACTAAATAAAATCGATCGATGTTACGGTTGGAAAACGATGCTTGATATGCCTGTTCAGGAAGCACTCAAGCACCAGAATGAGCACGTCATTCGCGAGTTTGAAGACCGCATGAAGTCAATTATCGTGGAGTTTGCCGAGCAAAAGCTAAATGCTGAGGTGTATTGGCGCAATAAGGATTTGGCTCACACGGTCTCTCTTATACCTACCAGTGCTATCTCTGGCGAAGGTGTGTCAGACCTCTTGATGATGCTGACGCGCTTAACGCAGGAGCGTATGGTCAAATCGCTTGCATTTGTAGATATTCTACAGTGTACTGTGCTTGAAGTGAAGGTAATTGAAGGCTTAGGTACCACAATTGATACGATATTAGTGAACGGCACTCTAGAAGAAGGTGCAACGATCGTAGTGTGCACATTAGATGGCCCAGTGGTGACAACGATTCGTTCATTGCTTACTCCGCATCCCATGAAGGAAATTCGCGTAAAAGGCGAGTACATTCATCACCAGAAGATAAAGGCTGCAATGGGCGTCAAAATTTGCGCACAGGGTCTTGAAAAAGCCGTAGCGGGCACTCAAATTCATGTGGTTGGCCCTGATGACGACATCGAAGAGCTTAAGGATTCAGTAATGTCGGACCTAACAAGCATTTTAGACTCAGTAAAATCTGCTCGCCGTGGCGTTATGGTTCAAGCCTCCACGTTAGGTGCTCTGGAGGCTCTGCTAGAATTTCTCCGTACGTGTGATCCGCCCATTCCGGTCTCTTGCGTGAACATTGGTCCGGTGCACAAGAAGGACGTCATGCGAGCGTCCGTGCAGCTGGAGCACCAGCCCGAATTTGCCACTATTCTTGCGTTTGATGTTAAGGTTCACAGTGACGCCACTGAGCTAGCAACAGAACTTGGTGTGCGCATTTTCACTGCCGACATCATTTACCACCTGTTCGACCAGTTCACGGCATACATGGACAACTTCCGCTCCGCCCGTCGAGAGGAATTTGCGGAGGTGGCTGTATTCCCGTGTGTGCTGAAAATACTTCCCAATTGTATCTTCAATAAAAAAGACCCCATTATCCTCGGCGTTGATGTTGAGGAGGGCATCGTTAAAGTGGGTACGCCTCTCGTGGTCCCATCTGCAGGCG---GCTTGCTTGTCGGCAAGGTAGGCTCCATCGAGCGCGAGCACAAGGAGGTGGACCGCGCCAAGAAGGGTGCTTCTGTGGCCGTGCGAATTGATAACGAAAGCAGTGTTATGTATGGCCGGCATTTCGACCATAAAAATCAACTTGTGTCGCGCCTGACCCGCGAAAGCATTGACGCGCTTAAAGAGAACTTTCGGGACGATCTGCAAAAAGAGGACTGGCAGCTTGTCATTAAGCTTAAAAAGGTGTTTGATATTATAATGGAGGGCTGGCTCACGAAGCGCCGAGACCACATGAACCTTATTTGGCTGGATCGTTACTTTCTTTTGGATGGCAACCAGCTTCGCTACTATCGCAAACGTGGTGACCCCGCGCCACGAGGGACGTACATTCTCACCGACGCGTGTGTTGTGAGTCCTGTCTTCAATTCCGAAGAGAAACACAAACACCATGGGCCCGTGTGGATGTTTCGGATTACATGAGAAGTCGTACAAGAAGGATCGATTTCTTGATCTGGGGGCAAAAAGCGAAGAAAATGCCAAGGAATGGAAGCGTGCAATTGAGAATGCCGTACGATCGTTAAGGGAATTGGTGGCCCATCAAAAGAAGCTGAAAACGGATGCCGAGAT---TTTCCCCACACCTACGCAC---------GTGCCAAGGATGTTAAAGGCCGATGAAGACACAATGTCTCTTTTTAATCTTGACGCCGACAATGTTATGGCAGAGCAAGCGTGGTGGCTTGCTCGAGTGGAAGGAGGTCTCCGAATCATGCAGGAATGTCCTCTTGGACCGAACGTCGCCGCCAATTTGTACGCACAACACGCTGCAACAACCAACGGCTTGTTTATAAGTGCCTGCGTAAGCGCCGCCTTGAGCTTTGCCTGCATGTTTTCGTATGCAAACAATATGCTCTTGAGTTTTGGTAGCGCACTTGTAGCCATGACAGTGTGTTGCGTTGCTGTGACGCCAACCCAAGCTCCTGAGATCCCGAGTTTGCGTGTCTCGCAAGTCGTTCATGGCTCGCCTACCGAAGTATTTCGTCTTGTCATGAATAGCAAGCGATTCCAGCGATGGGATGCCGCTACAGCCACGATGCGTGTGATTCAGCAACTTGATGATCATGCGGATATTGTGTACGTGACGCAGCGACCGACGCAATTGTGGCCATGGTGGCAAAAAGCCCGCGACCTCGTACTAATGCGCTACTGGCGACGGGAAGAGGACGGATCGTACTTTGTCATGTACCAGAGCATCGTGCATCCCGAGTGTCGTGTGCGCCACAATTACGTGCGTGCGAATTTTCTTGGCGGTGGTTTCGTGATTGCTCCGCAGCGTGTCCCAAGT---GGATCGATCCGTACGCTCGTGACGTACGTGGTTCGGTACGATCCTGGAGGTTG---GTCGTATATTTACCATCAACTTGGTATGGCGGTGGATCTCGTGATGCCCATGCTGCGAAGTGTCGTGGGTATTCGGGATGAAATGAGTGCCACGGACTTTATTACACCCAATGTCTCGATGGCACCTGCTGGAGACGG--AAGACTGATGTAGAAGTATTGA---CCCATTTGGCGTCGTCACGGGGGTTCAAAAGCTCGAA---ACGAGTTTGCCTGAAAAAATGTGGGCTGAGCCGGACTCGTCTCTCTTTTGTGTTCGTGGACACAATTACTTGACCGATAAGAAGAAGCTTCCGTCGGCACCTGCAATGTTCCACACGATGGGGGTCGACCTCTTGTCGTTTGAGAGTCCTGCAGAGAGGTACAACATTTCGTCGCGTTCGGACAGCATTGGAAGCACGAG---CTCGAGATTCACGTTTGTTGTCAACATGATCATTCCGAGTCCCGAGAATGTCTGCATGGTATTTTATTTCCAGCCAGTGCGTGACAATGTGTTTGAGGATGGTTCGGGTTTTTCCGAGCTTTTGAATGATTTTATTGATGGTGATGATCAATTTCGAAACTCCCGCTTTAAGCTTATTCCTACGGTCGTGGAAGGCAGCTTTATAATTAAACAGTCCGTTGGATCGAAACCCACCTTGCTTGGAAACAAGCTCAAGTGTCTGTACCATCGTGGAGAAAACTACTTTGAAGTGGACATTGATATCTCGTCCAATTCAGTTGCGAATACGGTGGTGGGTATGGTGCAAGGGGTGACCAAATCACTCGTGGTGGACCTGGCATTTCTGCTTGAGGCTCAGTCCGATGAAGAGTTACCAGAAGTTATCCTTGGCGCAGTTCGCATGCAGCACATTTCTCTTGATTCCCCACGCCGCATGGTATCGCATGGGGAATGGGCAGGAAGAATGAATCAACATTCCGAGGCATACGCCGAAGCTCTGCGCTTAAATCTGCAGCGACTCATTGTCTACGCACGATCGGCTGATACGGGACTGCAGCGAGAAATCGCCGAGAAACTCGCCAATGAAGCCGTCAAGATCGGCAGGTGCAGATTGTCGAGCTAGATGGACTGCAGCTGTTGCTTCCACTAACAAAATCAACGGACACTGAGGTGCAGCGTCTCGCAGCACACGCGCTTGCAAATCTATCCGTAAATTCTGAAAACCAGTCTAAGATGGCAACAGAGGGAGGCATCGACATGCTCATTGATCTCTTGAGCAGTACAAACGAGCACGTGCAGCGTCAAGCCGCCAAAGCACTGGCCAACCTCGGAGTGAATGTGGATAATAAGGAGCGGATTGCCAAAGCCGGAGGGATTCAACCGCTTATTGACTTGGCCAGTTCCCGTCAAATCGGCGTTGCTGTCGAAGCTGTTGCAGCACTTGCAAATCTGGCCGTCAATG---ATGCGAATGAAGTCGAGATTGCCCGGAAAGGAGGTTTAAAGCCAATTATCAATGGAGCACACGCCGAGTCTGTTGAGCTACAGTCTCAAGTCGCGCGTGCATTACGCAACTTATCCGTCAATCCGGAGAACAAGCAAGCGATCGTAGATTTTGGCGGAGTTGAAGCTCTTCAGTCTCTTGTACGTTCCTCCAACGATCGAATATGCCAACAAGCCACACGAGCGCTAGTGCCTAATGTCGTGTTAACGCGCTCTTCACCTTCAACCTGGTG------GTCGTACCCGTGGCCCGCGTGGCTGAGGTCAAGCAGGAGGC------TATGCCATTCGAAAGTGCCATGGGCAGGATCGATTATCCAATTAAAGCCAAGACAGTG---------ATGGTGTCTG---CAACCCCAA---AAACTGCAGCTAGT---GGCTTCTTGTCCATTTCCGACCGCTTTCCGCTCTCGAAGGTTGGAAAAGTTAGCTTTAAGATGGCGTCCGTTCCAATCAGTCG---ACTGCTTAATG---GCACACCCGAGATGACAC---AGGCAGAGCAA-------------ATGACTGTGCTTGTGGAAGGTGGCACA---AATGACATG---ACCATTAAGGCTGAG---CCTGAAAGTGAATGATGTGTACAATTACCGAGCCACGGA--GGTAGAAGAAATTGAAATGGAGCAACTTCGATGGAATATTCAAGAGCAGGTTTTTCAGCGCTACTTCCATCAAGCACAAAAGTACGATGAGTGGGGGCGGTTAGAGGAACAGGGCGCCGCGTCTTTTTCATTCCAGGCGGACGACGAGAAAATATTAAAGCCGAGAAGCAATTCCATGACAATGCTGTCCGTGACGCCATGGGGAAGGGTAACGCCACCTGGGTCAAGCAGCGGCGGGATCGTTTTGAAGAGGAAATTCGACTGTTTTACGACGAGATAAGGCACAAACAGGAGCTGATGCTTACACGTCTAGAAAAGGTGTCGCCGTTTTCGAAATTCCCAATTCTCGGCAACCGATTTTTGCTTCTTCGGCTGCGTGGGAAAGGAGGCAACGGAGAAGTATGGGATGTTGTTGACTACGTGAACAACAAGCAGCTTTGTGCTCTCAAGCTCTCCACTTCGATTCGACACGCCCAGCGCGAACACCACATGCATACTGAATCATCCTA--ACATTGTTCGTGTCGGCGAGAATCCTTTTCTCATTCGGTACCAGCAGCATCAGTACACTGCGTTTACTGTCACAAGCGTCCAGTCAGATCTGCAACAACTTATTGAAG---TTTATGAGCATTTGGATGACGACTCAGCATACAGGGTGCTCGCACAGCTTTTAAACGCTTTAAAATACTTGCACGATGGGCATTGCTCATTACGACCTGAAGCCGTCAAATGTTTTAATTGATCACGACGGGTCCGTGAAGCTG------ACAGACTTCGACTTGACACGCGATGTTAGGACAGCAACGTTTAACTCCACTGCCGGCACTCTGCGATACCTTCCTCCTGAGTGCTTTCGTCCAAGCCGAAGCGA---------TTGCGAAGCCACGGCCGAGAAAGCCGACATGTGGATGGTGGGTATATTGTACTGCATGATGGTGACTGGAAAACACCCAATCCTTCCAGACAAGTCGACACAAGCAGAAGTCAAGACAATAATGTCTCGATACGCTGGC---GAATTTCGATACGCGCGACCGGTTTCCGACCTGTCTCGTTGGATCTTAGAAGGATGTCTCCATCCACACCCGCGGTGTCGACCAACAGCTGCACAACTTATGGTTGCGCTCCAA

Hyaloperonospora_arabidopsidis TGGACTACCACCGCCAAGTCGCTCGTGAACTCGTAACTTTCTTCCACACTTTCTACCGCTTTGGGGCAGTCTCCAACCGCTTCGATTTGTTTCTCCAGAACCTCCTGCTCGGGTCGCTGGTCATTGGCGTCGTCGTCTTTATGTTTCTCTGCATCCTCTTCGTGCGCCGCACGATTCTGCACATTCAAAGACTCCAAAATGCCGCACGGAAATGGACACGACGTCAATGGAGCTGCTGGCAGTCGCGTTGCTGAGTGTGTTGGCGATTAGCGCCCTGGGGGGACTATTGGGAGGAGCTGTTTAAACACGAGTGACGCTTTGCGGGTCAATGCTGACAATTTGATCACGTCATTCTACGAGAGCGAATTGCCAGCTGAGGCCTTTGAGCTGTCGATTGAAGCGTTGGACTCGTGCACGCATCGAAAGATCTGTACAACCAATCGGATGCGATGCTACCGAAGAACTACTTGGATATGGCTAACGACTGGCAGCTGAGCTACTTTATGCTGGAGTCGTCGACAAATGGTGCAATTCTGGCTGTTGTGCTGGCCAGTTTCTTGTCCATCGCGTCCGTTGGGTGGGCAATGGTCTCACCCCTGCGTCTATCGATTCTAGTGATCTTGAGTGTCGTGCCGATTTCGCACACGCTCGTTGGCGCCTACTTGTCGTCGACGATCATGGTGGCGGATTTTTGTGCTGCACCGATGAACAATACGCTCGAACTCGTGGGCTCATGCCCTGCAAACGCCTCCTCCCTTTTGCTGACGCTTCGGCTACACAAAGCATCTCACCAAGGAGACGTTGGACGTCGCATGAAGAAAGACTTTCTGGATCCGATCAGATCAACTGTGGGCGTCATGCTCGGCTACTTTATCGGCACAGTGAGCAACTGGGAGAGTTCCCCGCACGTCCAGACGTGGCGCTGGCCCTTTCTGCTGCAGTTTCTAGCACTGCTGCCAGTCAACATGGCGATCTTTCTCGTGCCTTCGAAGCACTTGATCATCCGCAGTGAGACCTTTGAGCTGGAGCCACTTGACGATTTCAAGCTGGTGGTGATCAAATCCAAGTCAACGTGGCAAGAGAATACTAGCTCTCGATTTTCTACCAAAAGAGACTCTATGGGTCACTTGCTACGAGGTGTATGAGCAAGGCGCGTTTTCGGACGGCTTACACGAGCTGTTGCGCATACCTGTGTTCTGTCTCATCGTATTCGGCCTCACGACGGTGTACTTTGTCGTCACGGGCGTGCAGTACTGGAGCACCATCTTTATGATCAAGTCATTGCACGCATCAAAGTACCTCGTCAATGCGCTCTTCGTCGTCGTTTCCGGTACAGGACCCATTCTCGGCGTATTCTTCGGTGGGTGGCTCATTGACCGATACGGAGGATACAGAGGTGTGGCACAGCGCGCAAAGGCTCTGGGAATCTGCATGATTCTTGGGCTGACAGCCTTTTTTATCTCGGCGGTGACGACGTTCTTCAATGACGTCTTCATTACCTCTGGCTTCTTGTGGCTGCTGCTCTTCTTCGGCGGCTCGATTCTGCCAGCACATCGTGCGATCGCTTCGTCATTCTCCGTGATGGTGTTCAATTTATTTGGTTACTCGCTGTCGCATGAACGACCTCCAGATGGTCGTCGGTGCCAAGTGCTTCGTCCCCGACGAGACGCACGTGTGGCTGGCCGCCCAAGTCCTGCGGGACGAGAACTCGGGCGACGGCAAGACGCGCAAGATCTACTGCAGAGTGGAGCTCCCGGATGGGCACGGAAGAGCGCTGCGTGGACATGCTGGCCAAGAAGACCAAGGCGCTGCTGCCGTGCACGAGCTCGAGACGCTGCCGTATCAGAACGAGAACGTCGGGTCTGAGGGCATTGAAGACATGATTACGCTCAATTACCTGCACGAGGCGGCGATTCTGTACAACATTAAAAAGCGCTTTTTATGCGAGCTCCCGTATACGTATACGGGCGACATTTGTATCGCCATTAACCCGTACAAGTGGCTCCCGGAGCTCTACGCCGAGGAGCAGCACCGACGGTACTTGAATCAACCCAAGGAAGAGCTCCCGCCCCACGTGTATGCGACATCTGTCGCTGCTTATGACAATATGCGACGCTCCGAGTGGAACCAGTCGATCCTCGTGAGTGGCGAGTCGGGAGCGGGCAAGACCGAGACGACCAAGATCCTCATGAACCACCTCGCGACGATCGCGGGCGGCATGAACAACTCGACGATCAAGCGCATTATAGAAGTCAATCCACTGCTCGAGTCGTTTGGCAATGCCAAGACCGTCCGCAACGACAACTCGAGTCGATTCGGCAAGTTTACACAGCTCCAGTTTGACCAGAGGGGCACGCTCGTGGGCGCCAAGTGTCGCACGTATCTCTTGGAGAAGACACGTGTGATCCAGCACGAAGCCCCGGAGCGCAATTACCATATCTTTTACCAGTGGACTCGCCCGACATGGCGGCAGAGCTCCAGCTCGACTCGACGAAACATTATGTGTACACGGGCAACAACACGGCGCAAAAGATTGAAGGATTGTCGGACGAGAAGCACTTTATCAAGACACGAGAGGCACTGGCGCTCGTGGGACTCTCACGTGAAGAGCAGCAGCCACTGTTTGAAGTGCTGGCGGGCGTGCTGCACCTTGGTGAAGTGCAGTTGCTATCCGACGCATCGGACGATGAGAAATCGAATATCGCTGTGGGTGATGGTGGCGCGATCTCGGCGACTGCGATGCTGGGCGTCACATGGGAAGCACTTGAGACTGCGCTGTGTTCGCGTACCATGCGCGCGGTGAACGATGTGTACAGTGTGCCACTGAAGAAAGAGCTGGCGATGAACTGTCGTGATGCCCTGGCAAAGGCTATTTACTCGAATGTGTTCGACTGGCTCGTGACGACAATCAATCACTCTCTCGCTGATGATAAGAATATGTCGAATCACGTCGGTGTGCTCGATATTTTCGGTTTCGAGCATTTCAAGCACAACTCGTTCGAGCAGTTTTGCATTAATTACGCTAACGAAAAGTTGCAACAGAAGTTTACTCAGGATGTGTTCAAGACTGTCCAGATTGAGTATGAGGAAGAAGGTATCGTGTGGGACCACATTGAGTATGCGGACAACCAAGACGTGCTGACCGTGATTGAGTCGCGCATGGGTATTATTTCCTTATTGAACGAAGAGGTGATGCGACCGAAGGGTAGCGAGGAGTCGTTCATGTCCAAGGTGACATCTCTGCACAAGGAT---GACATGACACACGTGATTGAGTTTCCCCGCACGTCTCGTACCGAGTTTCTTATCAAGCACTATGCTGCTCCGGTCATGTATGATTCGGTGGGCTTTCTGGAAAAACACAAGGATTCGCTGCTGCCTGACCTATCCGAGCTCATGCGTGGTTCGTCAAAGCCATTCATCGCGGAGCTCTTTGAACCGAAGCCGGAGCCCAAGTCGCCGGAAGC---------GTGGCGGTGCTTTGTCCATCACGACTGTCGGTACTCAGTTCAAAGAGAGCTTAACAGAGCTGATGGCGACGATCAACTCCACCCAAGTTCACTACGTGCGTTGCATCAAGCCCAACCCGATTAA---------GAGCTCTACAGTCATGGATCAGTCCATGGTGGTCTCTCAACTGCGATGTGCAGGTGTAATTGAAGCTATTCGTATCTCACGTGCAGCGTACCCGAATCGGTTGCAGCACACAGAGATTCTGGACAAGTTTTGGCTCTTTGTACCAAGCGGTGGTAATACTCCGGTCGAAAAATGTCAACTACTCATGGACAAGCTGAAAATAGAGTCTCCAGTGCAATACCAGATGGGTAAGACCCGGGTATACTTCCAATTGGGTGTGCTTGAAGAGCTCGAAGATCGTCGTAAGAAATTTCTGGATGCGAAGGCGACGTACTTGCAAAATGTCATGCTTGGCTTCACGCAGCGTATCAAGTACCTCCGACAACTGGCTGCGATTATCAAGCTCAGTTATCCGATGTGTGATTGCTATGCGAAGGTACAACACATTTATGAACGGCCTCATTTTGATGCAGGCCCACTGGCGAGGTTATCAAGGTCGAAAGGTAGCTGCAGAAGTCAAGAGCAACCACTACGCTGTCATCATTCAGCGGTATGTGCGCGGCTTTGTCAAGCGTCATAGTTACGTTAACATGCGGGCGATGGTCATTCGTGTTCAAGCCGTGGTTCGAATGACGATTCAGAGGCCAAAATACCTCGCTGCCCTCGACGAGAAACGTCGTGAGGCTGATATGGCGTACCAACTGAATAAGTTGAAGGCCGCTCTGCAAGAAGAGCAGGAAAGGAATGTGCAGCT------ACAACGTC---GTAGTAGTGTGAC--GACGGCGCAACTGCCGCCTCGAGCGTGGTGATGGCTGACGCTGGTGGCATGATTGAAACTCTTACGAATGAGAATAAGAAGTTGCGTGAGAAGAATGTAGAAATGAAGGTTGCGATGAAGGGTTTGAAGGTCGAGATTGAAAAATTCAAGAGTGACAAAGAATTCTCGTCGGCTGGCAATCACGTCAAGCTTCGTCAGCTGCAGGACACGGTGCGCGAGAAAGACAAGAAGATCAAGTTGCTCGAGACCGACAACAAGAAGCTCGTGGAGCAACTTGCAAAGATTTACGTGGACGG---TGT---GCCTGAGAAGAAGTCAACGCAGAAGAAGTCCATCTTCCGTACGCTGGGGTCCAAGAAGGAGAAGAAGCCTC---------------GTGAGACCGTGCTGATGGACTCACTTACTGGTTCGGACGACCTTAACAGTCGGGAGACGCGTCAGTCAGA---GCGCCATTTCCCTCGCATGC---CAACGATGAGCGTAGGCACC---CGCTTCTGGAAT---AACAAG---AAGGGTGTT------------AACGGCGACGGCTTTGACGTTTCGGAGGTCGAAGGTGAGGATAGCCCGACGGACCTGCGCGCAACACGATAGTGAAAAGCGTCGAGGTGGGTGTGACGGGAGCCATGTCGAGTCTGAAGGGTCGCATG---------TCGGTCGTGAAGGGCATGTATGAAGTAAGCGGACGAACAGGATCGCCCTGTGCGCGAGTCGTTCAACCTGGATGCGCTGCCCGAGGTATCCCTGCCTGTTGGCTGGGAGGCCAAGGTGTCCCGCTCAACGGGCCGCGTGTACTATGTGAACCGCAAGCTCGGCAAGTCTCAGTTCGAGCGCCCTACACTAGCCTCGGTGAA------GGCCCAGAAGCTAGCGCGTCAGAAGTCAGCGAGCGGGCTGTAAGGCAGTAGTGGCTGATTTCGGCTGTGGGGATGCAAGGCTGGCTGAGAGCGTGTCCAACAAAGTTCACTCGTTTGATCTAGTGTCCCTCGACATTGGCGTGTACTGTTTGGCACTCATGGGGACGAGTGTCAGCGAGTACGTACGTGAAGTGTACCGTGTGTTAAGACCTGGAGGCGTGTTGAAGATTGCAGAGGTGAAAAGCCGTTTTGAGAGTGAGACACTGGGCGGATGGGTCTAGTCTTGATCGCAGCTGCGCACGAGATCGTCGTTACTGTGCGCAATACGACGGTGTTCGTCAACAGATGAAGCACGCAGTCATTTTAGGCCAAAAAACAGGCGAGGCTGTGGCTGTGCCTTTTCCGGACGTAGACCGAGACCTCCGGATGCTGCTGGGCCACACGACGTCGATGATCACCCACGTGGCTGTAAATCTCGTCACTGCTACTGACGGCTGATCGGGATGAAAAGCTGCGCGTGTCGAGGTTTCCGAATGCTGCTATTATTGAGAGCTACTGCTTGGGCCATAAGGCTAGTCTCACCAAGGTAGCCTGTAGTGCAGTCACGCCGGACCTCGTCGTGTCGACGTCCATGGACAATACGATGAAGCTCTGGAACATGGAGACAGGAAACTTAGCGATTTGTCCCAAGACGACGTTGTGGCTGTGCTTGTGAACTTTCGACACGTGCGATTCTTCGAAATTGTGAGGATCCGTTTGTGCAGTTGTTCTCCATTTCAAATGGCAACTGATGTGTTCAACCACTGTGCGTGACTTTCGCAAGTTGCAGAACGACCCACCGTCGGGCGTCTCGGGCGCTCCCATGGACAACAATATCATGCTCTGGCAGGCCGTTATTTTTGGACCGGACGATACACCGTGGGAAGGTGGGACGTTTAATCTGACGCTCGAGTTTAGTGAAGACTATCCGAACAAGGCACCAACTGTCAAATTTATCACGAAAATGTTCCATCCAAATATCTACAACGACGGTCAGATCTGTCTGGACATTTTGCAGAACCAGTGGTCGCCCATCTACGACATCTCGGCGATCCTCACGTCCATCCAGTCGCTGCTGTGTGACCCGAACCCGAACTCGCCAGCAAACTCGGAGGCCGCGCGGCTTTTCCAAGAAAATCGACGGGAGTACAATCGCCGCGTGCGCGAGATTGTCGAACAGAGTTGGCAGGCGATCGATGGTGTCAGCTGCTAAAATGCGCGGTGCTGAGAACCGCATGAACGCCGGTCGTCCATTTACAGCCTGGCTGGACAACGTGTCGTCCGATCGTGGTCTCTGTGGAGGGATCAACTCGGGCATTGCCAAGACGGCTCGCAAACAGACCAGTCGCAGATCTTTGTCATTGGAGACAAGGCCCGTGCCCAGCTGCGTCGTGACCTGGGCGAGCGCATTCGTGGGAACGTGACGGAGACGTACCAGTCGCCGCCAAACTTTACCGTGGCCAGTGCCATTGCCGAGGCTGTCGAGTCGGAGAAGAGTCGTCGCGTATGGCGGCCATGGAAAACGCCACCACGAACGCGGAGGACCTCACGCTCACGCTCGTGTACAACAAGGCTCGCCAGGCTCGTATTACCACGGAGCTCATTGAAATTATTTCGGGTGCCGCTTCGTTGGACCGTCACTGGCAGCGTTAGCAACGTCCAGAGCGTCGCACAGTTCGACGACGCCGTCGCACGTGACTCCACGCTCTCGGTCACGTATTTCTACGCCGAGTTCCACGCCTGTCGTCCAAACGGCCAGCTCGATGTGGGGCTCCGTGGTACTCGAGAAACTCGAGGGTACAAACGTAGCCGTGCTCGCTAAACGTGTCGACAGGATCGACTGGCGAAACTCCTATATGCCGCGCCGGTGATGCTGTTTCTAAAAGGCACGCCGAGTGAACCCAAGTGTGACGCTAGTCGGGATGTTGTCGTACTACTGAATGAAGAGGAGATCGCGTTCAGTACGTTTGACGTGCTCCAGGACCAAGATGTGCAAGAAGGACTAAAGCTCGTGAGCAAGTCGTCGATGTTCCCGCAACTTTATGTGAACAGCTCGTTCATTGGGGGGCTCGAAGTGCTGAAGGAGATCAAGGCCAAAGGATCAATTGCCGACCACATTGGACTCGAGCCAACCGATGCAGCGTTCCAAGCGAGTCTGCGTGCTTTGGTGAACTCGGCACCGGTGTTGCTGTTTATGAAGTTTTGATATCTTGTGTGGAGCTACCAGCGTCAGGACCAAGATATCGCCAAGGTAGTGACGCTGACGCCGTCGTCGTTTCGGCGGAGTATGTGCAGAGGCTATTTCATGGAGTCCGAAACCGCATGGCCATGGCATTTCGTGATCCTCTGGAAGAGAAGGGGGAATTTTGTATCGTGCAGCTTCTCGTATCACTTCGAAGCGCACCACGTTACATCCCCGTGGTATTTCGACAATTGGCTGGTAAGTGGAGTGTGTTGCACCGCGACCATCGCAGTGAAGTGAAATCGCTTTGTATCCATTCCAAGCGAGACTGTGTGTACACGGGTGGCTTTGATGGTCTCGTGATTCGATACGACGTGAACAAGGGACGGACGGAAACAGTGATGGAGCGCCGGAAGCCCATCTGCAAGATCAACTCGATCCTCGAGCATCCACACAACCCGAATTTGCTTCTGGTCAGCAGTGTGGAGCAGTCAGCACAACTTGTTGCTGCATGACCTTCAGCGATATGACAGTAACGTTTCGGGCAGCTCGATGAGTCAGTATGTCGTACCGCGCTGGTCACCGGCGGGTTATCACGTCTCGTGTGGCAGCAAATGCGCTATTGCCACGGGAGCTTCTCCACGCCCACGTCCGCTACAATTGGCGCGTCGTTTCTACAGAAACGGGTCATTGTCAACGGGAAATTGACGCTCCAGATTTGGGACACGGCCGGTCAAGAGCGGTTTCGGTCCATGGCGCCGCTGTACTATCGGAATGCCAAGGCGGCGATCCTCGTGTTTGATCTGCAGAGTGAAGCGAGTTTTGAGAAAATTCAAGAGTGGCTGCATTTGCAGCATTATGTGGACGATGATATGTGGGTAACAAGAGCGACGTTTTGACTTCCGTAAAGCGCAAATGTTTGCAGACAAGATTGGAGCTCGAGTATTTCGCACAAGTGCCAAAACGGGGGAAGGCGTGCAGGCACTTTTTGAGAGTTTAGCTGTGCAGCTGCTGAAGAAACACGACCAGGAGCAGCGGAGAGTTTCTACGTGGTGTATGTACCGGCAGATGAGACCAAGGAGCTGGAAGAATGGGCAGTCAAGCTCCCCAGTGACAAGGAGGCGCAGCTGGGCTGTTTAACCAAGCGACTTCGAGTGCATTTCAAGGGAAACTCCATCCAAGCAGCAGCAGAGACGTTCAAACAGCAACTACTGGCTCAAATGCCTGAGGGTGCGACGCTCACGGATGAGATGTTTCAAATGGTACATGGACAGTCTAGTGGACACCATTCCACTCGTACTTAACACGCCGGGCGTCAAGCACGTTGGAAGTAAAGGGCGATGCATTCATCGGCCGCGTATTTGATAGACGATTTTGTCAGGATGGATTTCCGACTCAGTGAGATATCAGGTGATGCCCCATGGGTGCAGACGGCAAAGTCGCAGAGGTTACACAGTCGCTTGAGGAAATCCTAGCACTTGGACAAGGGCTGGACGATCAGGACTACAAGGCCGAGCTCGAAGTGCTACAGAATGATCCGAATTCGAATCTGTACTCGGCAGTAACGTTCGAAGAGCTGAATTTACCAGAACCGCTGCTTAAGGGCGTGTACAACATGAAGTTCGCCAAGCCGTCCAAGATCCAGTCGGTGGCGTTGCCTTTGATCTTGGCTGATCCACCCGAGAACCTTATTGGTCAGGCACAAAGTGGCATCCCACTCGTGAGCTGGTTCGGCAGATTATGGCTGTGGTGAACGCCATGGGCAAATTGTCGAGACGTTTTTGGCGATCCCAGGCAACGACGTGGCTCGTGGAGAGCGGTTGACAGCCCAAGTGATTGTAGGAACTCCTGGCAAGGTCGAGGGTATGATCAAGAAGAAGCAGCTCGACACGCGCGACGTGAAGATCTTTGTGTTGGACGAGGCCGATGTGATGGTTGCTGAAGATGGTATGCGCGAGCGCTCTGTATCAATCAAGCGACTGATTAAGAACCGAGCTTGTCAGTACCTCCTGTTCTCGGCTACGTACGCTGACGACGTGCGGGAGTTTGCCCAGAAAATGGTGCCGGACCACAACATCATCACAGTCAAGAAGGAGAAGCTCACCCTTGACGGCATCAAGCAGTTCTGGATTGACTGCAAGACTCGTGACAACAAATACCAGGTGCTCTCGGACATTTTTGCCGTCCTGTCGATTGGGAAGTGCGTCATCTTTGTGCAGCAGCGCGAAACGGCCAAGGAGCTGACGCGTCGGATGCGTGAGAAGGGCCACATGGTTGGGATCCTGCATGGTGCTGACATGGCCAAGGAGGTGCGCGATCAAGTCATCGATGAGTTTCGAGCTGGCACGACCAACGTGCTGATCACTACGAATGTGCTTGCTCGTGGTATCGATGTGGCGGGGGTATCCCTTGTCGTGAACTTTGACATCCCGCTTACTCGAGACCAGCGCCCGGATCCCGAAACGTACTTGCACCGTATCGGTCGTACTGGTCGTTTCGGCCGCAAAGGCTGTGCCATCAACTTTGTCTACGACGACAAAAGCAAGCAAGACCTTGCGGATATCGAAGAGCACTATGCGCGGCCAATAATTCAGGCTCCCGCGGACGATATCGAGGAACTCGAGAAGTTGCTGGCTTGGGGAGCAGCAAGCGCCGGCTGTCGAGGTTTGGCAAGGAACAGCGCAAGGGCATGCGTATTTACCCACGAGAGCCCAAGAAGAAGGTGTCGGGCAAGGACAAGACGTACTACTACCTCAAGGACGTCATGTTCTTGGCGCATGAGCCAGTGCTGAGGAAGTTCCGCGCTTTCAAGACGTTCATGAAACAAGTGACAAAAGCCATGGGGCGGAAAGACTATGCAAATGCCAAGCGCAAGCACGAGAATCGCCCGACGTACGAGCTCGATCACATTGTCAAGGAGCGCTATCCGCGCTTTGTTGATGCCCTAGGAGATCTGGATGACGCGCTGTGTGGCAGAACTACATTGTTGCTACGCGAGCCCTTTCCAAGGTTTTTGTGAGCATTAAGGGCATCTACTATCAAGCAAATGTGCAAGGCCAGACCATTACGTGGCTGGTGCCACATCAGTTTACACCGACAGTGGACAAGCGCGTGGACGTGCGGGTCATGCTGACGTTTTTGGAGTTTTATGAAGTGCTGCTTAAGTTCGTCAACGCACAGTTGTACATGCACATCGGAGTCGTGTACCCGCCAAAGATTAACTTGCAACTTGATTCGGCTGGTGCACAACTAGCTGCGATTGAATTAGCAACAGAAGGAGTCTGAGAAGCGGATTAAGCTACAATGAGCAACGCAAGGAGTGCAGTGAGAAGAGCTTGTTCAGTGGGCTGACCTTCTTCTTGTCCCGAGAAGTACCTTCGGCTTGTTTGGAACTGGTGCTGCGATCGCACGGCGCGACTCTTGGTTGGGACGGCGCTGGTTCTCCTTTCGATGAGAAAAGCTCACGCATCACGCACCATGTCATGGATCGGCCGCACCAGGGTCATCGCTACTTCAACCGCGAGTACGTGCAGCCGCAGTGGGTATTTGACTCGGTGAACAATGGCATTCTGCTGCCTCTGGCACGTTACCGCAAGGAGCTGGACAAGCTCAAGTCGGCTGTGCAGGTGCTGCGCGAGGTCGGTGCCGTTGGTGATGATGATGAGGACGGGAAGTCTATGCCGCTGACCTGAAGGCTGAGGTGGATGGCAACAAGAAGTCAAAGCGTGTTAAGAAGGAGGAAGAGGCAGCAGAGCTGAAGGAGTTGGCCAAGACTATGATGTCGAAGAAGGCCAAGCATCTGTACGATCGTATGCAGTACGGTTTGGGTAAGAAGGCTGACAAGATCCAGAAGCTCGAGGACAAGCGCGCCATGCTCGAGGCAAGACGAAGAAGCTCCTGGCAGGTGCGCTCTCTGGTATGATCTCCGCGCTGGCTTTCCAGCCTCTGGGGTGATGCGCTGGCGTGGCACATCCCCAACACTCGTGCGCGTAGCCGGCGGCGCGGGTCTCTACTTTGCTACTCTGGACTACTGTTTGAACGTATTCCCGAGCTCTGCAGTCAATACGTTTCTCGCTGGAGCTGCTGCTCGTACGTTTGCAGGCAGCGTCATGTCACCCTTTACTATTGTGAAGGCGCGGATGGATGCCCTCGGGTGCCTTTCGTAGCAGAGCGACGTACCGTTCTCTGGACTCTATGTGCTGCTTTACACGCCATCCGGCCGATGTGATAAAGACACGTATGCATGCGCCAAACAGTAGCCAAGATGTACCACCACGAAGGCCTGCGTGGCTTTGCGAAGGGCATCGTTCCGCGCGTAATTAAGCGCACGCTTTCCACTGCAGTGACATGGACGATCTATGAGCTACTCGTTCGACCCGTCAGGTCTGGGTCACGCCAAGGAAGCGTTCAATTTGGCCAAAGAGACGGAGCGGACCAAGCAGATTGAGAGTCAGGCGAAGATCAAGGAGAACGAGGCTTTGTACAAACAGTACGAGATCGTGCGGGTGCAGAAAGAGGGCGAAGAGCGCCGCAAAACGCTCGAAGAAGAGACGCGTCAGCACCAACAGCGTGCGCAGTATCAGGACCAGCTCAAGCGCAAGCAGTATGCAGACCAATTGGCCGCGCAGAAGTACATGAAGGAGCAGGAGCTCAAGAAGCAGGAGGAGATCCTGGCTCGTCAGGAAGCTTCACGTCGTAAAACGCTCGACTATGAAGCTGAACTGCGTCAGAGGACAGAGATGGCCAAAGTGGCGGCGGAGACAGAAGGACGTATCAAGCAGGAGCGTCTTAACCATGATCTGCATCTGGAAGAGGCCCGTGTGCGTGCGAAGGAGTACCGTGAGACGGTCATGGAAGGGATCAAGCTGGCAGGCAATACTGTCGGCTCTGGTGTCATGTCCTTTGTCGATGACAAGGAGAAACTCACTGCCACGGTTGTCTCTTTGACAGCTCTTGCTGCTGGAATTTACACGGCCAAGGTCTCAACTCGTGTTGCAGGCAAGTACGTTGAGGCGCGTATGGGAAAGCCCTCGCTAGTGCGTGAAACTTCGCGCCGCTCGGCTGCACAGGTGCTGGCGAATCCTATTCCGTCCATTAAGCGTGCGCTGCGTCTACAGAAGCCGACGGATGCATTGGAAGGCGTCGTGCTTGAGCCCAAGCTGGACGAACGTCTGCGCAGCGTTGCTGTGTCCACGTTCAACACGAAGAAGAACCGTGCGCCATTCCGCCACTTGCTTTTGCACGGCCCGCCGGGTACGGGTAAGACGCTGTTTGCCAAAGCGTTGGCGCGTCACTCTGGTCTGGAGTATGCTATTCTCACGGGTGGTGATGTTGCCCCGCTGGGCCGCGAAGGCGTGACGGAGATCCACAAGCTGTTTGACTGGGCATCGCACAGCCGTCGTGGGTTGCTGCTGTTCGTTGACGAAGCTGATGCGTTTCTCCAGAAACGTAGCAATACTGTCATGAGTGAAGACATGCGTAATGCGCTGAACGCCTTCCTGTACCGCACAGGTGAGGCGAGCGACAAGTTTATGGTTGTCTTTGCAAGCAACCAGCCGGAGCAGTTTGACTGGGCCATCAATGATCGTATTGATGAGATGGTGGAGTTCCGTTTGCCTGGCTTTGATGAGCGTGTGCGCATACTGAAGCAGTACTTCGAAGACTACATCCGTGCTCCCAAGAACTCGCGTGCTAAGAAGATTTATGTGAAGGCTCTATCTTCACGCTGGACCCTGTCGCCCACTTTCTCGTGCCAAAACACGGATCTTGCAACTCGAAATACTGCAAGGCGTGTCCAGTGAAGCTCAACTGATCTTTGATGCTCTGAACAAGAGCCCATGCGTCTGTACTGGATCGCCTTTGTGCTGGTCCTGGGCAACGGTGTCTACACGTTTATTGCAGGTCGAGACGAGTCGTTTCTACTGGACAAACTCAAGAGACAGATTCCGTCCGAGTACCGCATCTCGGAGAAGTGGGACAAGTGTCTCGAGAACTTTGCGCTCTACTTCGGCACTGGCCTCATGGCTGGCGGCCTCACGTCCCTCGTCTTGGCCCGTTCAGGTGCTGGTCGTGGACTGGTGACTGGCTTGGGCGGGCAGGAGCTGGCTCGAGCTGGACCACGTGTCAGATGGCGTTTGCAGGCCACGAGAAAGCCGCTTTGAACAAGACGGACAAGGCGGTGGGGGACTTGCCGGCGTTCCCTCACCAGACGATCGTGGACTTTGTGACGGCGTCCGCCACGCAGCAGCAGCTCCTGCACGTGCACTCGCGGCTTCTGTGACCAAGCCACTGTGCAAGTGTTGGAATCGTACTTAGCCGAACAGGTGCAACAGACGACGGTCGATGTGGACGCGAACCTAGCGTTGCTCAAGTTGTACCAGTTGTACCCATCCACGCTGAAGACCGCGAGCGACGGTGCTGATCAAGGGCATGATGGCGCTCCCTTCGACGTTCTTCACGGGCGTCGACGATGGTCCCGGAGAGTATCCGCGAGGATGCTCATGTCTCGGCGGTGCTGCACACGGGCTTTATGCTCCAGTCGTGTCTATTTGAAGACTTTTGGAAGACGGATGTGACGTTTGCGGACGAGGTGCCGGGCTTCTTGGAGTCCGTGCGCGCTTACATCTTGGTGGCCATCTGCCGCAGCCACAGTGTGATTTCCACGGACGTTTTCAAGGCCAAACTCAATGTGTCGGACCAGCAAGTGGCTGACATTGTCGCCGAGCAGTGGACGCTGGTAATTTAATTCAGATTAAGCCCAATGAGCTCAACCAGATGCAAGCGAAGAAGGTTCAGGAGAATATTCAGTACGAAGACGTGCTTAAGGCCATCCACGTTCTTTCTAAATCGAACGTCTGTGCCAGTGGCGGCGGCGTTGCTGACGCTGAGGAGGTCTGTGAGAGCCTGGCCAAGCACTTGAGACTCGATGCCGGTACCCAAGTGCTGCCCTGTTCGACGGGCGTCATTGGCTGGCGTATCCCGGTGGATGCTATGGTAGCAAACCTCCCAAAGCTCGTCAAGAAGCTGCAGTCTGACTCGGTGCTGCCTGCTGCGGAGGGGATCATGACCACGGATCTGTACCCGAAGGTTCGCTCTATGGATGTGTGCGGTGGCCGAATCGTTGGCATTGCCAAAGGAGCTGGTATGATTGAACCTAATATGGCTACGATGCTGTCGTATATTCTCACTGATTTGTCGATTCCTCGTGACGTGCTGCGTCAGTTGCTAAAGGAAGTCGTCGGAGATACGTACAACTCACTTAGTATAGATACGGACCAGAGCACGTCGGATACGGTGGCGCTCGTGTCGTCGGACATGATCCCCTTCGACATGAA---GCATCTGGACGAGGTCAAGAAGGCACTGTACGAGGTGTGCCTCGGACTCAGTGAAGACGTCGTGCGAAACGGTGAGGGTGCACACCACGTTATCCGGGTGAGCGTGGGTGGCGCCTTGACGAAAGAGATGGCCAAGGGCGTCGGCAAGTCCGTGGTGAACTCGCCCCTGCTCAAGTGTGCAGTAGCTGGTAACGATCCGAACGTGGGTCGTCTTGTCATGGCTGTGGGTAAGTACATGGGTCTGCATTTCAAGGATGTGAACATTCAGCGCCGGATGAAGGTCCGCATGGGTGGCGTCCTCATTTTCGAGAACGGGGAGATGGTGCTGAACGACGATATCGAGGCTAAACTTGTGGCCCATATGCGTGACGCTATGCTGATCGAGCCTACGCGTGGCGGACTCGACGCTAGCTCGCACAATTACCCGCCGCATGGAAAGTTCGTTG---------------------------------------AGGTATTGGGTATTGACCTCACCCACGAGTACGTGGCAATTAACGCGGACTACCGCTCGTAATGGCACCCGGAAAAAAAGGGAAGACTGCTGCCGCGTACTTGGCGTCTCTAGGCGTCGCGTCTGC---AGCTGGGAGCGAGAAGAAGGAGGACCCGAAGAAGAAGAAGAAGAAGGGCAAGAAGAAGCCCACAAGAAGCCCGTGTCGGCTGCTGCCAAGCTGCTGA-TGGAGCGCCAGGTGGCGCTGAAGGCTGAGCAGGACCGGCTGGAGCAGGAACGACTGGAGCTCGAGCGCGTGGCGAAAGAGGAAGAGGCCAAGCGCGTGGCCGAGATGGAGGTCGAGGAAGAGCTTCGGCGCAAGAAGAAGGAGGCGCGAGCGGCCAAGATTGAGCGGCAGAAGAAGGAGGGCACGTACATGACCAAGGCCCAGAAACAGAAGGCGGCCAAGGCTCAGGCGGCTCTGGAGGCGATGCAGCAGGCAGGTCTTGTGCCGGAAGCTGCG------GGCGAGAAGAAGAAGGTGGTGTATGCCAATAAGAAAAAACAGAACAAGGACATGAGGAAGAAGAGGAAGAAGAAAGTGGAGCCCCCGATGCATGGGACGACGACGACTGGGAGACGAGCGATGTCGTGGCTTCACTGGAAGATAAACTAGCAGGAGAAGGAGCAAGAGCGTTTACGTCAACAGGGCATTGCGCTGAAGAAGCGTCAAGAAGAGCAGGCGGAGCAGATGCGAGCGGAGGCTGAAGAGGAGCATCAACGCTCGAAGGCCAAGATGGAGGCGGAGCGTAAGAAGAAGGAGGCTGCTGATCGACGTCGCGAACGTGAGGCCATTGCACGAGCCAATGTATCGCTGGACAATCTGCGTTCACCTATTTGCTGTATCATGGGTCATGTTGATACTGGTAAGACGAAGCTGCTTGACAACATTCGTAAGACGAAAGTGCAAGACGCCGAAGCCGGTGGTATTACTCAGCAAATCGGTGCTACTTTTTTCCCTGTGGAGGCTATCAAGCAGAAGACAGCTCGGCTTATCGAGACAATGAAGCTCGAGTTCCGTCTGCCCGGTTTGCTAGTGATCGATACGCCTGGTCACGAGTCGTTCACAAATCTGCGTAATCGTGGTTCGTCGCTATGTGATATCGCCATTTTAGTGGTGGACATTATGCACGGTTTGGAGCCTCAGACTCTGGAATCACTGCGCTTGCTGCGTCAAAAGAAAGCACCGTTTGTCGTGGCGCTGAACAAAATCGATCGTTGTTACGGTTGGAAGACGATGCCTGATATGCCCATTCAAGAGGCGCTTAAGCATCAAAACGAACACGTCATTCGCGAGTTCGAGGATCGCATGAAAGCGATTATTGTAGAGTTTGCCGAGCAGAAGTTGAATGCCGAGGTTTACTGGCGCAACAAAAACTTGGCGAACACAGTATCGCTCATTCCTACCAGTGCCATCTCGGGCGAAGGTGTGCCGGACTTGTTGATGATGCTGACACGCTTGACACAGGAGCGCATGGCGAAGTCATTGGCGTTCGTCGACATCTTACAGTGTACCGTGCTAGAAGTCAAGGTCATTGAAGGTCTTGGTACGACCATTGATACGATCCTAGTGAATGGTACACTGGTAGAAGGCTCTACTATAGTTGTGTGTACCTTGGATGGGCCTGTTGTGACAAGCATCCGTTCACTGCTGACTCCGCATCCAATGAAAGAAATTCGAGTCAAGGGTGAGTACATCCACCACCAAAAGATGAAGGCTGCCATGGGTGTAAAGATTTGCGCGCAAGGTCTCGAAAAAGCAGTGGCCGGTACACAGATTTACGTGGTTGGTCCTGACGACGATGTTGACGAGCTGAAGGATACCGTTATGTCCGACCTGACGAGTATTCTGGACTCCGTGAAGGCTACCCGCCGTGGTGTGATGGTACAAGCTTCCACTCTTGGTGCGTTGGAAGCGCTTCTAGAATTCTTGCGCACATGTGATCCGCCTATCCCGGTGTCCTGTGTGAACATTGGCCCCGTATACAAGAAGGATGTAATGCGAGCTTCAGTGCAGCTCGAGCACCAGCCAGAATTTGCTACCATATTGGCGTTCGACGTGAAGGTTCACAACGATGCGACTGAGCTCGCCTCCGAGCTTGGCGTGCGCATTTTTACCGCTGACATCATTTACCACCTGTTTGATCAGTTTACGGCGTATATGGACAACTTTCGGATAGCCCGACGTGAGGAATTCGAGGAGGTTGCAGTGTTCCCTTGCGTGCTCAAGATCGTTCCCAGCTGTGTGTTTAACAAGAAAGACCCAATCATTCTTGGTGTCGACGTTGAAGAGGGCATTCTCAAAGTAGGCACCCCTCTCGTAGTGCCATCTGCCGGTG---GGTTCTTGGTCGGGAAGGTGGGCTCTATCGAGCGCGAGCACAAAGAGGTCGACCGTGCTAAGAAGGGCGCGTCGGTTGCTGTGCGGATAGACAACGAGAGCAGTGTCATGTACGGCCGTCACTTTGACCACAAGAACAAGCTGGTGTCGCGCTTGACTCGTGAGAGTATCGACGCACTAAAAGAGAATTTCCGGGATGATCTTCAGAAAGAGGACTGGCAGCTCGTCATCAAGCTGAAGAAGGTGTTCGATATCATTATGGAGGGCTGGCTCACCAAGCGCCGCGACCATATGAACCTCATCTGGCGAGACCGGTACTTCCTCTTGGACGGCAATCAGCTGCGCTATTTCCGTAAACGAGGCGACTTGGCTCCTCGGGGGACGTACATTTTGACGGACAATTGTGTCGTGAGTCCCGTCTTTAACTCGGAGGAGAAGCACAAGCACCACGGACCTGTCTGGATGTTCCGCATCACGTGAAAAGTCGTACAAGAAGGACCGGTTCCTGGACCTTGGCGCCAAGAGTGAAGAGCACGCAAGAGAGTGGAAACGCGCTCTTGAAAACGCCGTGCGCTCGCTCCAGGAGCTCGTGGCGCATCAGCGCAAACTCAAGACGGACGCTGAGAT---CTTTCCAACGCCCACGCAC---------GTTCCAAGGATGCTCAAAACGGACGAAGATACCGTGTCGCTGTTTAACTTGGACGCGGATAATGTCAAGGCCGAGCAGGCGTGGTGGCTCGCACGGGTGGAAGGGGGACTGCGGATCATGCAGGAGTGTCCGTTGGGGCCGAACGTAGCGGCGAATTTGTACGCACAGCACGCAGCAACGACGAACGGACTAATCCTGAGCGCTCTGCTCGGTGCTGCACTGAGCTTCAGCAGCATGTACTCGTTCGCGAACAACATGCTGCTGAGTTTCCTGACTGCACTTGGAGCGATTGTCGTGTGCTGTGTCGCCGTGACGCCAACACAAGCGCCCGAGATCCCGAGCTTCCGTGTCTCTCAAGTGGTGCATGGATCGCCGACGGAAGTCTTTCGACTCATTATGAACTCAAAGCGATTCCAGCGATGGGACGCAGCAACTGCGACGTTGCGTGTGGTGCAGCAGCTGGATAACAATGCGGACATTGTCTACGTGACGCAACGTCCGACACGCTTGTGGCCGCTGTGGCAGAAAGCTCGTGATCTTGTGTTCATGCGCTACTGGCGACGCGAAGAAGATGGGTCGTACTTTGTCATGTATCAGAGCATTGACCACCCTGAATGCCGCGTGCGTCACAACTACGTGCGAGCAGATATACTGGGTGGCGGCTTTGTGATTGCGCCGCAACGCGTTCCCAGT---GGATCCATTCGCACGCTCGTGACCTACGTGCTCCGGTACGACCCTGGCGGCTG---GTCACGCATTTACCATCAACTCGGCATGGATGTGGACTTGGTCATGCCCATGTTGCGCAGCGTGATTGGCATTCGTGACGAAATGAGCGCCACGGACTTTATCACTCCTAATGTCTCAATGGCGCCTGATGGTGAGGACAGGTGCGGATCG---AGCGTGGA---CTCTATGGGTGTTGTCATGGGTGTGCAGAAGCTGCCGACCACTAGTCTACCGGAGTCAATGTGGGCCGAGCCCGACGCGTCGCTTTTCAGTGTGCGTGGACACAACTACTTGAATGACAAGAAGAAGATCCCATCGGCGCCAGCGATGTTCCACGTCGTGGGTGTGGACTTGCTGTCGTTCGAGAGTGCAGCTGAGCGATACGATATTTCGTCTCGTGTCGACAGCATTGGCCGCACCAG---CTCCAAGTTTACGTTTGTCGTGAACATGATCATCCCGGGTCCAGAGAATACGTGCATGGTGTTCTACTTCCACCCAGCACGTGACAATGTGTTTGAAGACGGCTCGCCATTTTCCGAGCTACTGAACGACTTCTTCGATGGCGACGACCAGTTTCGGAACTCGCGCTTCAAGCTCATTCCTACTGTCGTAGAGGGCAGCTTCATCATCAAGCAGTCGGTTGGATCCAAGCCGACACTGCTGGGCAATAAGCTGAAGTGCCTGTATTACCGTGGCGAAAACTACTTCGAGGTGGACGTCGACATCTCGTCCAACTCGGTTGCCAATACTGTCGTGGGAATGGTACAAGGTGTGACCAAGTCGTTGGTGGTGGACATGGCGTTCCTGCTTGAGGCTCAGTCCGATGAAGAGCTGCCTGAAATTATCCTCGGAGCTGTTCGCTTGCAGCATGTTTCTCTGGACAATGCCCGTCGCGCACCAAAGCATGGGGAATACCTACTACCGCCACTTCCACCTTCTGAAGTGTTGACGGAAGCCCTGCACGTGAACTTGCAACAACTGGTCGCCTACGCGCACTCAGCAGACACTTCGCTCCAGCGAGAAGTGGCCGAGAAATTGGCCAATGAGGCTGTGAAAATCGACAGGTGCAGATTGTGGAATTAAATGGACTGCAGCTGCTGCTGCCACTGACCAAGTCGACGGACACGGAGGTGCAACGACTTGCAGCGCACGCTCTCGCCAACCTCTCGGTCAATTCGCAGAATCAGTCGAAAATGGCGACTGAAGGAGGCATCGGGATGCTCATTGATCTCTTGGGTAGCTCGAACGAGCACGTACAGCGCCAAGCGGCCAAGGCGCTGGCTAACCTAGGCGTAAATGTGGATAACAAGGAGCGAATCGCAAAGGCAGGCGGCATCAAACCGCTCATTGACTTGGCAAGCTCGCGTCAAATAGGCGTCGCAGTTGAGGCTGTTGCGGCACTGGCAAATCTGGCTGTCAATG---ATGCCAATGAGATCGAGATCGCACGTAAAGGCGGCTTGAAGTCTATCATTGATGGAGCACACTCGGAGTCGATGGAGCTCCAATCCCAAGTGGCACGTGCACTACGTAACCTTTCAGTCAACCCGGAGAACAAGCAAGCGATTGTGGACTTGGGTGGTGTAGAGGCTCTGCAGTCACTTGTGCGCTCCACTAACGACCGAATATGCCAGCAAGCTACGCGGGCACTCGTGTCCAACGTGGTGCTCACGCGCTCGTCGCCCTCAATCTCGTGCGCGCTCTCGCGCCTGTCGC------TTGTGCGGCGACGGAAGATACG---AGGCAGGACTACGAGCCGATAGTCATGGCCGTCGAGGCCGCAGCCACCATCAAGACGATCC------GTGACGTACTTAATCCAACGCCCC---ACGACTCTGCACGA---AGCTTTTTGTCCATCGCGGATGTGGCGCCACTGTCCAATGTGTCGCTCGCCAAGGTGGGCACG---CCCTCTACAGTCCGCTA---TGTGCACGACG---ACAC------------------GGTGGATGATG----------ATGCGACAGTG------AGAGAGGAAGCAGACGGTGATGTG---ACGACGATGGACGAC---GGTGA---TGGGCGTCGTGTACCGGTACAATGCGACGGA--GGTGGAGGAAGATGAGATGGAGGAACTTCGTTGGAATATCCATCAGCAAGTGTTTCAGCGGTACTCTCAGCTCGAGCAAAAGTGCGACGAGTGGGGACAACTGGTAGAGCAAGATACCGAGTGGTTTTTGTCCACGGTGTCAGACGGGAACGGATTATGGCCGAAAAGAAGTTTCATGACAATGCGGTTCGTGATGCCATGGGGAAGGCAAACGCTGCGTGGGTGGAGGAGCGGCGCAATCGGGTTGAGGAGGAA----------------------------------------------------------------------------------------------GCAATCGATTTTTGCCACTTCGTCTGCGAGGAAAGGGTGGACACGGGGAAGTGTGGGATGTGGTGGATTATGCTAACAACAAGAAGCTCTGCGCGCTCAAGCTGTCGACTTCCATTCGCCACGCCGAACGCGAGCACCACGTGCATACTCAAGCACCCGA--ATATTGTTCAAGTTGGCGAAGTGGCGCTGCTCATTCGCTACCAACAGCAGCAATACACAGCCTTCACTGTCGCAAGTGTCCATTCCGACTTGCAGCAGCTTATTGAG--------------------------------------------------------------------------------TGGGCATTGCCCACTACGACCTGAAGCCGTCGAACATTTTGATCGATCAGGACGGGTGTGTAAAACTG------ACTGACTTTGACTTGGCCCGCGATGCCAAATCTGCTACGTCAAGCTCTACAGTTGGCACTCTACGGTACCTCCCACCCGAGTGTTTTCGTCCGAAACGAGGCGA---------TTGCGAAGCCACAGCTGAAAAAGCGGATATGTGGATGGTAGGTATCGTCTATTGCATGATGATTACTGGGAAACACCCGATCTGCCCAGACAAGCCAACCCCTGCGGAGTTCAAGTCAATGATGAGTCGGTACACTGGT---GCGATACAATACGCTCGACCAGTCTCTGATCTGTCGAGGTGGATTATACAAGGATGTCTCCAGCCCGACCCAAGGTGCCGACCAACGGCCGCGCAATTGCTGGTGGCACTACAG

Hyaloperonospora_brassicae CGGACTACCGCCGCCACGTCGCGCGTGACCTAGTGGCCTTCTTCCACTCGCTCGGCCGCTTCGGCGTCGCCTCGAACCGCTTCGACCTGTTCCTCGAGAACCTCCTGCTCGGGTCGCTCGGCTTTGGCGTCGCCGTCTTTGCTGTCCTCTGCGTCCTCGTCGTGCGGCGCACGATACTGCACGTCCACTTCGTCGGAGACGTCGCTCGGACGCAGACACGACGTCAACAGAGTTAGTGGCAGTCGCACTGCTGAGTGTGTTGGCCCTCAGTGCCCTGGGCGGACTCCTGGGAGAAGCTGCTCGCACGCGAGTGACGCACTGCGGGTCACTGCTGACAATTTGATCACGTCGTTCAATGAGACTGAGGTACCCGCTGCGGCGTTTGAGCTGTCCATTGAAGCACTGGACTTGTGCATGCGTCGAAAGACCTGTGGAACCAATCGGACGCAATGCTGCCGACGGACTTTTCGGACATGGCGAGCGACTGGGAAGCGAGCTACTTTCTGCTCGAGGCCTCGACGAACGGCGCGATCTTGGCTGTCGTGCTCGCGAGCTTCTTGTCCATCGCGTCTGTCGGGTGGGCCATGGTCTCTCCCCTGCGTCTCTCGATTCTCGTGATCTTGAGCGTCGTGCCGATCTCGCACACGCTCGTTGGCGCCTACTTGTCCTCGACAGTTATGGTGGCGGACTTTTGCGCGGCGCCGATGAACAGTACGCTCGAGCTCGTGGGCGCATGCCCTGGGAACGCTTCCTGCCGTTTGCCGATGCTGTGGCCACGCCGAGCGTCGCGCCAAGGAGACGTTGGCCGTCGCATGAAGAAGGAGTTTTTGGACCCGATTGGACCAACTGTGGGCGTCATGCTCGGCTACTTTATCGGCACCGTGAGCAACTGGGAGAGCTCGCCGCACGTGCAGACGTGGCGCTGGCCGTTCCTGCTGCAGTTCCTTGCGCTGCTGCCGGTCAACATGGCGATCTTCCTCGTGCCCACGAAGCACCTGATCATCCGCAGCGAGACCTGCGAGCTTGAGCCGCCAGCAGCGGGCACTCCGATGAGCACGAGGTCGACCACGTACCACGACGAAGAAGATGCGTTGCTTGCCAGTGCGAGTATGCATCATATGGTGCCTTGGACACGAAGTGTACGAGCAGGGCGCGTTTTCGGACGGCCTGCACGAGCTGTTGCGCATCCCCGTGTTCTGTTTGATCGTGTTCGGTCTCACGACGGTATACTTTGTCGTCACGGGCGTGCAGTACTGGAGCACCATCTTCATGATCAAGTCACTGCACGCGTCGAAGTACCTGGTGAACGCGCTCTTTGTCGTTGTGTCTGGTACAGGGCCCATTCTCGGCGTGTTCTTTGGCGGGTGGCTCATTGACCGATATGGAGGATACATAGGGGTGGAACAGCGCGCTAAGGCTCTGGGAATTTGCATGATTCTTGGACTCACAGCCTTTTTTATTTCGGCGGTGACGACGTTCTTCAACGACGTCTTCGTTACCTCCGGCTTCTTGTGGCTGCTGCTTTTCTTCGGCGGCTCGATCCTGCCAGCACACCGCGCGCTCGCTTCGTCGCTCTCGGTGATGGTGTTCAATCTGTTCGGCTACTCGCTGTCGCATGAGCGACCTGCAGATGGTCACCGGGGCCAAGTGCTTTGTCCCCGACGAGACGCACGTGTGGCTGGCCGCGCAAGTCCTGCGCGACGAGAACTCGGGCGACGGCAAGACGCGCAAGATCTACTGCACCGTGGAGCTCCCGGACGGGGACGGAAGAGCGCTGCGTGGACATGCTGGCCAAGAAGACCAAGGCGCTGCTGCCGTGCACGAGCTCGAGACGCTGCCGTACCAGAACGAGCACGTGGGCCCCGAGGGCATTGAAGACATGATTACGCTCAATTACCTGCACGAGGCTGCGATTCTGTTTAACATTAAAAAGCGCTTTTTGTGCGAGCTCCCGTATACGTACACCGGCGACATTTGCATCGCCATTAACCCGTACAAGTGGCTCCCCGAGCTCTACGCCGAGGAGCAGCACCGACGGTACTTGCACCAGCCCAAGGAAGAGCTCCCGCCGCACGTGTACGCGACGTCGGTCGCGGCCTACGACAACATGCGGCGCTCGGAGTGGAACCAGTCGATCCTCGTGAGCGGCGAGTCGGGCGCGGGCAAGACCGAGACGACCAAGATCCTCATGAACCACCTCGCGACGATCGCGGGCGGCATGAACAACTCGACGATCAAGCGCATCATCGAAGTGAACCCGCTACTCGAGTCGTTTGGCAACGCTAAGACCGTCCGGAACGACAACTCGAGTCGCTTTGGCAAGTTCACGCAGCTCCAGTTTGACCAGCAGGGCACGCTCGTGGGCGCCAAGTGCCGCACGTACCTCTTGGAGAAGACGCGCGTGATCCAGCACGAAGCGCCCGAGCGCAACTACCACATTTTTTACCAGTGGACTCGCCCGCCATGGCGGCGGAGCTCCAGCTCGACGCGACCAAACACTATGTCTACACGGGCAACAACACGGCGCAAAAGATTGAGGGGCTGTCGGACGAAAAGCACTTTGGCAAGACGCGCGAAGCGCTGGCGCTCGTAGGGCTCTCGCACGAAGAGCAGCAGCCGCTGTTTGAAGTGCTGGCGGGAGTGCTGCACCTGGGCGAAGTACAGCTGCAGTCGGACGCGTCGGACGATGAGAAGTCGAACATCGCCGAAGGCGATCGCGGCGCGAGCTCGGCGACCGAGATGCTGGGCGTCACGTGGGCAGCACTGGAGACTGCGCTGTGTTCGCGGACCATGCGCGCGGTGAACGACGTGTACAGTGTGCCGCTGAAGAAAGAGCTGGCGATGAACTGCCGTGACGCGCTGGCGAAGGCTATCTACTCGAACGTGTTTGACTGGCTCGTGACGACGATCAATCACTCGCTTGCGGACGATAAGAACATGGCGAACCACGTCGGTGTGCTGGACATTTTCGGGTTCGAGCACTTTAAGCACAACTCGTTCGAGCAGTTCTGCATCAATTATGCAAACGAGAAGCTCCAGCAGAAGTTTACGCAGGATGTGTTCAAGACTGTGCAGATTGAGTACGAGGAAGAAGGGATTGTGTGGGACCATATCGAGTACGCGGACAACCAGGACGTGTTGACCGTGATTGAGTCGCGCATGGGTATTATTTCCTTGTTGAACGAAGAGGTGATGCGACCGAAAGGTAGCGAGGAGTCGTTCATGTCCAAGGTGACATCGCTGCACAAGGAT---GACATGACGCACGTCATTGAGTTCCCTCGCACGTCTCGTACCGAGTTCCTCATCAAGCACTATGCCGCGCCAGTCATGTATGATTCGGTGGGCTTTCTGGAGAAACACAAGGACTCGCTGCTGCCTGACCTCTCTGAGCTCATGCGCGGTTCGTCAAAGCCATTCATCGCAGAGCTTTTCGAACCAAAGCCGGAGCCCACGTCGCCGAAAGC---------GCGGCGGTGCGTTGTCCATCACGACCGTCGGTACTCAGTTCAAAGAGAGTCTGACAGAGTTGATGGCGACGATCAACTCTACCCAAGTTCACTACGTGCGATGCATCAAGCCCAACCCGATCAA---------GAGCTCTTCTGTCATGGACCAGTCTATGGTGGTGTCCCAACTGCGATGCGCTGGTGTGATTGAAGCCATTCGTATCTCACGTGCAGCGTACCCGAATCGGTTGCAGCATACGGAGATTCTGGACAAGTTTTGGCTGTTTGTGCCAAGCGGCGGTAACACGCCGGTCGAAAAGTGTCAACTGCTCATGGACAAGCTGAAAATAGAGTCCCCAGTGCAGTATCAGATGGGTAAGACCCGGGTGTACTTCCAGTTGGGAGTGCTGGAAGAGCTCGAAGACCGTCGCAAGAAATTTCTGGATGCCAAGGCCACGTACCTGCAGACTGTCATGATTGGCTTCACGCAGCGCATCAAGTACCTTCGTCAACTGGCTGCGATCATCAAGCTCAGTAATCCGATGCGTGATTGCGATGCGAAGGTACAACACGTTTATGCATGGCCACATTCTGATGCAGGCTCACTGGCGAGGTTATCAAGGCCGAAAGATTGCGGCAGAAGTCAAGAGCAACCACTACGCTGTCATCATCCAGCGTTATGTGCGCGGCTTTGTGAAGCGTCACCGTTACGTCAACATGCGTATGATGGTTATTCGCGTTCAAGCCTTGGTTCGTATGATGATCCAGAGGCCGAAGTACCTTGCCGCGCTCGAGGAGAAACGTCGTGAGGCTGACATGGCGTATCAACTAAACAAGTTGAAGGCCGCTCTGCAAGAAGAGCAGGAAAGGAATGTCCAGTT------GCAACGTC---GCAGTAGTGTGAC--GACGGCGCGACTGCTGCCTCGAGCGTGGTGATGGCCGACGCCGGTGGCATGATCGAGACTCTGACGGATGAGAACAAGAAGTTGCGCGAAAAGAATGGAGAAATGAAGATTGCAATGAAGAGTCTGAAGGTCGAGATTGAGAAGTTCAAGAGCGACAAGGAGTTCTCGTCGGCTGGCAACCACGTGAAGCTTCGTCAACTGCAAGACACGGTGCGCGAGAAAGACAAGAAGATCCAAATGCTTGAGGCCGACAACAAGAAGCTGGTGGAGCAGCTCGCAAAGATCTACGTGGACGG---TGT---GCCGGAGAAGAAGTCGACGCAGAAGAAGTCGATCTTCCGCACGCTAGGGTCCAAGAAGGAGAAGAAGCCTC---------------GCGAGACCGTGCTTATGGACTCACTCACTGGGTCGGATGACCTGAACAGCCGAGAGACGCGTCAGTCAGA---GCGCCATTTCCCTCGCATGC---CGACCATGAACGTCGGCACT---CGCTTCTGGAACTCGAACAAG---AAGGGTAGC------------AACAGCGACGGCTTTGACGTTTCAGAGGTCGATGGTGAGGACAGTCCGACGGACCTGCGCGCAACACGATCGTCAAGAGCGTGGAGGTGGGCGTAACGGGGGCCATGTCGAGTCTGAAGGGTCGTATG---------TCGGTCGTGAAGGGCATGTACGAAGCAAGCGCACGAACACGATCGCCCTGTGCGCGAGTCGTTCAACCTGGACGCGCTGCCCGAGGTGTCGCTGCCCGTCGGCTGGGAGGCCAAGGTGTCCCGCTCAACGGGTCGCGTGTACTATGTGAACCGAAAGCTGGGCAAGTCGCAGTTCGAGCGCCCGACGCTCGCGTCGGTGAA------GGCCCAGAAGCTGGCGCGCCAGAAGTCGGCAAGCGGACAGTGAGGCAGTGGTGGCGGATTTCGGCTGTGGGGATGCCAGGCTGGCCGAGAGCGTGCCAAATAAGGTGCACTCGTTCGACCTCGTGTCTCTCGACATTGGCGTGTATTGCTTAGCGCTCATGGGGACGAGTGTCCGCGAGTACGTGCGCGAAGTACACCGCGTGCTAAAGCCTGGGGGCGTGCTGAAGATTGCCGAAGTGAAGAGCCGCTTTGAAAGTGAGACGCTGGGCGGATGGTGCAGGTGTTTACGGCAGCAGCGCACGACGTCGTCGTGACCGTGCGCGACAAGACGGTATTCGCAAAGAGACCAAGTACATTGTGGTTTTGGGCCAAAAGACGGGTGAGGCTGTGGGTGTGCCTTTCCCGGACTTGGACCGAGACCTCCGGACGCTGTTGGGCCACACAACGTCGATGATCACCGACGTGGCGACGAATCTCGTCACTGCTACTGACGGCCGATCGGGATGAAAAGATGCGCGTGTCGAGGTTTCCCAATGCTGCTATTATTGAGAGCTACTGCTTGGGCCACAAGGCTAGTCTCACCAAGGTGGCGTGTAGTATCGTCACGCCAGAGCTCGTCGTGTCGGCGTCAATGGACAATACGCTGAAGCTCTGGAACATGAAGACGGGCACACTGGCAATCAGCCCCAAGACGACATTGTGGCGGTTCTCGTGAATTGTCAGCACGCGCGATTCTTCGAGATTGTGAAGACCCGTTTGTGCAGCTGTTCTCCATTTCATTACGCAACTGATGTGTTCAACCGCTGTGCGCGACTTTCGCAAGTTGCAGAACGACCCGCCGTCCGGTGTCTCGGGCGCCCCCATGGACAACAACATCATGCTCTGGCAGGCCGTTATTTTTGGACCCGACGACACGCCGTGGGAAGGTGGGACGTTCAATCTGACGCTCGAGTTCAGTGAAGACTATCCGAACAAGGCGCCGACCGTGAAGTTTGTCACGAAGATGTTCCATCCAAACATCTACAATGACGGTCAGATTTGTCTGGACATTTTGCAGAACCAGTGGTCGCCGATCTACGACATCTCGGCGATCCTGACGTCCATCCAGTCGCTGCTGTGTGACCCGAATCCGAACTCGCCGGCAAACTCGGAAGCAGCGCGTCTTTTCCAAGAAAATCGACGGGAATACAATCGCCGCGTGCGCGAGATCGTCGAGCAGAGTTGGCAGGCAATTGATGGTGTCTGCCGCCAAGATGCGCGGCGCGGAGAACCGCATGACCGCTGGTCGGCCGTTCACTGCGTGGCTGGACAACGTGTCGTCGGACCGCGGGCTCTGTGGCGGCATCAACTCGGGCATTGCCAAAACCACTCGCAAACAGAGCACGCGCAGATCTTTGTCATTGGCGACAAGGCCCGTGCCCAGCTGCGCCGTGACCTCGGCGACCACATCCGTGGCAACGTGACGGAAGTGTACCAGTCGCCTCCGAACTTTACAGTGGCCAGCGCGATCGCTGAGGCAGTCGAGTCGGAGAAGAGTCGTCGCGCATGGCGGCCATGGAGAACGCCACGACGAACGCCGAGGACCTCACGCTCACGCTCGTGTACAACAAGGCTCGCCAGGCCCGTATCACCACGGAGCTCATTGAAATCATTTCGGGTGCCGCGTCGCTGGACCGTCACGGGCAGCGTCGGAAGCGTCTCGAGCGTCGCGCACTTTGACGACGCGACGGCGCGCGACTCGACGCTCTCGGTCACGTACTTCTACGCCGAGTTCCACGCCTGTCGTCCCAACGGGCAGCTCGACGTGGGGCCCAATTGTGCTCGAGAAACTCGAGGGAACGAACGTCGCAGCGCTCGCGAAGCGGGTCGACAGGTTCGACTGGCAAAGCTGTTGCGTGCAGCGCCCGTGATGCTGTTTCTCAAGGGCACACCGAGTGACCCCAAGTGTGACGCGAGTCGCGACGTGGTCGCGTTGCTGAACGAAGAGACGATCGCGTTCAGTGCGTTCGACGTGGATCAGGACCACGACGTGCAGGAAGGACTGAAGCTCGTGTCGACGTCGTCGACGTTCCCGCAGCTCTATGTACATGGCGCGTTCGTTGGTGGACTGGATCGTCTCAAGGAGATCAAGGCGACAGGGTCGATTGCGGACCACTTTGGAGTCGAGCCAACCGATGCGGCGTTCCAGGAGAGTCTGCGTGCACTGGTGAACTCGGCCCCGGTGTTGCTGTTTATGAAGTTTCGACATTTTGTGTGGAGTTACCAGCGTCAGGATCAAGACATTGCCAAGGTGGTGACGCTGACGCCGTCGTCGTTCCGGCGGAGTATGTGCAGAGGCTGTATCATGGAGTCCGAAACCGCATGGCCATGGCGTTTCGTGATCCTGTGGATGACAAGGGAGAGCTCTGCATTGTGCAGCTTCCCGCATCACTTCGAAGCGCACGATTCTGCATCCACGCGGTATTTCCACGATCGGGTGGTAAGTGGAGCGTACTGCACCGTGACCATCGCAGTGAAGTGAAGTCGCTTTGCATCCATTCCAAGCGCGATTGTTTGTACACGGGTGGATTTGACGGGCTGTTGATTCGGTACGACGTGAACAAGGGATGCACTGAGACAGTGATGGAGCGCCGCAAGCACATTTGCAAGATCAACTCGATCCTCGAGCATCCACACAACCCGAATTTGCTTCTCGTTAGCAGCGTCGAGCAGTCAGCACAACATGTTGTTGTATGACGTCCAGCGGTACGACAGCAGAGTGTCGGGTAGCTCAACGAGTCAGTACATCGTACCGCGGTGGTCACCTGCAGGCTACCACGTCTCGTGTGGCAGCAAATGCGCTATTGCCACGGCAGCTTCTCGACGCCCACGTCCGCGACCATTGGCGCGTCGTTCCTCCAGAAACGAGTCATTGTCAACGGGAAACTGACGCTCCAGATATGGGACACGGCGGGCCAAGAGCGCTTCCGGTCCATGGCGCCGCTCTACTACCGCAACGCAAAGGCCGCGATCCTCGTGTTTGACCTGCAGAGCGAAGCGAGTTTCGAGAAGATTCAAGAGTGGCTGCATTTGCAGCATTACGTGGACGACGACATGTGGGCAACAAGAGCGACGTTTTGACTTTCGGAAAGCACAGGCGTTTGCCGACAAGATTGGCGCTCGAGTGTTTCGCACGAGTGCCAAGACGGGTGAAGGCGTACAGGCGCTGTTTGAGAGTTTAGCAGTGCAGCTGCTGAAGAAACACGATCAAGAGCAGCGCGGCGTTTCTACGTAGTGTACGTGCCGGCAGATGCGGACAAAGGACTGGAAGAATGGGCCGTTGAACTGCCCAGTGATAAGGAGGCGCAACTGGGCTGTCTGACCGATAGATTGCGAGCGCACTTCAAGGGCAACGCCATCCACGCAGCAGCGGAGACGTTCAAACAGCAACTCGTGGCTCAAATGCCGGAGGGTGCTACGCTCACGGATGAGATGTTCCAGATGGTGCATGGACAGTCTTGTGGACTCGATTCCACTCGTCCTCAACACGCCGAGCGTCAAGCACGTTGGAAGTCAAGGGTGATGCATTCATTGGACGTGTGTTTGATAGATGATTTCGTCAGGATGGACTTCCGACTGAGTGAGGTTTCCGGTGATGCCCCGTGGGTGCAGACGGCAAAGTCGCAGAGGTGACGCAGTCGCTCGAGGAGATCCTGGCGCTCGGCCAGGGGCTGGACGACCAGGACTACAAGGCCGAGCTCCAAGTGCTGCAGAACGATCCGAATTCAAATCTCTACTCGGCCGTAACCTTCGAAGAGCTCAACTTACCAGAGCCGCTGCTCAAGGGCGTGTACAACATGAAGTTCGCAAAGCCGTCGAAGATTCAGTCGGTCGCGTTGCCGCTGATCTTAGCTGATCCGCCCGAGAACCTCATTGGCCAGGCACAGAGCGGCATCCCACTCGGGAGCTCGTGCGGCAGATCATGGCTGTGGTGAATGCCATGGGCAAATTGTCGAGACGTTTTTGGCGATTCCAGGCAACGACGTTGCTCGGGGCGAGCGCTTGACGGCTCAAGTGATTGTCGGCACCCCGGGCAAAGTTGAGGGCATGATCAAGAAGAAGCAGCTCGAAACGCGCGATGTGAAGATTTTTGTGCTGGACGAGGCCGACGTGATGGTTGCAGAAGATGGTATGCGCGAGCGCTCTGTGGCGATCAAGCGGATGATCAAGAACCGTGCGTGCCAGTACCTCTTGTTCTCGGCGACGTACGCGGACGACGTGCGGGAGTTTGCCCAGAAGATGGTGCCTGATCACAACATTATTACCGTCAAGAAGGAGAAGCTCACTCTAGACGGTATCAAGCAGTTCTGGATCGACTGTAAGACTCGCGACAACAAGTTCCAAGTGCTCTCGGACATTTTTGCAGTCTTGACGATCGGCAAGTGCGTCATCTTTGTGCAGCAGCGCGAAACAGCCAAGGAACTGACGCGTCGTATGCGTGAGAAGGGCCACATGGTGGGTATCTTGCATGGTGCCGACATGGCGAAGGAGGTGCGCGACCAGGTGATCGATGAGTTCCGTGCCGGTACAACCAATGTGCTGATCACTACGAATGTGCTCGCTCGTGGCATCGATGTGGCGGGTGTGTCACTTGTCGTGAACTTTGACATCCCGCTAACGCGCGACCAACGCCCGGACCCCGAAACGTACTTGCACCGTATCGGTCGTACAGGTCGTTTTGGCCGCAAAGGCTGCGCCATCAACTTTGTCTACGACAACAAGAGCAAGCAAGACCTTGCGGATATTGAAGAGCACTACGCGCGACCTATCACTCAGGCTCCCGCGGACGATATCGAGGAACTCGAGAAGCTGCTGGCCTGGGGAGCAGCAAGCGCCGGCTGTCCCGGTTCGGCAAGGAGCAGCGCAAGGGCATGCGCATTTACCCGCGCGAGCCCAAGAAGAAGGTGTCGGGCAAGGACAAGACGTACTACTACCTCAAGGACGTGATGTTCTTGGCGCATGAGCCCGTGCTGAAGAAGTTTCGCGCGTTCAAGACGTTCATGAAGCAGGTGACCAAGGCCATGGGTCGAAAAGACTATGAGAACGCCAAGCGCAAGCACGAGAACCGCCCGACTTACGAGCTCGACCACATTGTCAAGGAGCGGTATCCGCGCTTTGTCGATGCACTGGGCGATCTGGACGACGCGCTTTGTGGCAGAACTACATTGTGGCCACTCGGGCGCTTTCGAAGGTCTTTGTGAGCATTAAGGGCATCTACTACCAAGCAAATGTGCAAGGCCAGACCGTCACGTGGCTGGTGCCGCATCAGTTTACACCAACAGTCGACAAACGCGTGGACGTGCGGGTGATGCTCACGTTTTTGGAGTTTTACGAAGTGCTGCTCAAGTTTGTCAACGCGCAGTTGTACATGCAGATTGGAGTGGCGTATCCGCCAAAGATCAACTTGCAGCTGGATTCAGCTGGTGCTCAACTTGCTGCGATTGAATTAGCAGCAGAAGGAGTCGGAGAAGCGGATCAAGCTACAGTGAGCAACGCAAGGAGCGCAGTGAGAAGAGTCTGTTCAGCGGGCTGACGTTCTTTTTGTCCCGTGAGGTACCTTCGGCTTGCTTGGAACTAGTGCTGCGCTCGCACGGCGCTACTCTTGGCTGGGATGGCGCTGGTTCTCCTTTTGATGAGAAAAGTCCACGCATCACGCACCACGTCATGGATCGTCCGCACCAGGGTCATCGCTACTTCAATCGCGAGTACGTGCAGCCGCAATGGGTGTTCGATTCGGTGAACAATGGCATCTTGTTGCCTCTCGCGCGATACCGCAAGGAGCTGGACAAGCTGAAATCGGCTGTGCAGGTGCTGCGCGAGGTCGGTGCAATTGGTGACGATGAAGAGGATGGGAAGTGTATGCTGCTGACCTGAAGGCTGAGATGAATGGCGACAAGAAGTCAAAGCGCGTCAAGAAGGAGGAAGAAGCGGCCGAGCTGAAGGAATTGGCCAAGACCATGATGTCGAAGAAGGCCAAGTATTTGTACGACCGCATGCAGTACGGTCTGGGCAAGAAGGCCGACAAGATCCAGAAGCTCGAGGACAAGCGTGCCATGCTTGATGCAAGAACAGGAAGCTCGCGGCCGGTGCGCTCTCGGGTGTGATCTCCGCGCTGGCGTTTCAGCCTCTAGGGCGCTGCGCTGGCGTGGCACGTCGCCAACGCTCGTGCGCGTCGCTGGCGGCGCAGGTCTCTACTTTGCAACGCTGGACTACTGTCTGCACGTGTTCCCGAGCTCTGCAGTCAATACGTTTCTCGCTGGAGCTGCTGCCCGTACATTTGCAGGCAGCGTCATGTCGCCCTTTACGATCGTGAAGGCGCGGATGGATGCCACCAGGCGCCTGTGAAAGCAGAGGGACGTACCGTTCTCTGGTCTCTATGTGCTACTGTATACGCCATCCGGCCGATGTTGTTAAGACACGTATGCATGCGTCAAACAGTGGCCAAGATGTACCACCATGAAGGCCCGCGTGGCTTTATGAAGGGCATTGTTCCGCGTGTGATCAAACGCACCCTTTCCACTGCAGTAACATGGACGATCTACGAGCTACTCGTTTGACCCGTCGGGTTTAGGGCACGCGAAAGAGGCATTCAATTTGGCCAAAGAGACGGAGCGGACGAAGCAGATGGAGAGTCAGGCGAAGATCAAGGAGAACGAAGCGCTGTACAAACAGTATGAGATCGTGCGCGTGCAGAAAGAGGGCGAAGAGCGCCGCAAAACGCTCGAGGAAGAGACGCGCCAGCACCAGCAGCGCGCGCAGTACCAGGACCAGCTCAAGCGCAAGCAGTACGCGGACCAATTGGCCGCGCAGAAGTACATGAAGGAGCAGGAGCTCAAGAAACAGGAGGAGATTCTGGCTCGCCAAGAAGCTTCGCGTCGCAAGACGCTCGACTATGAAGCTGAACTGCGGCAGAGGACTGAGATGGCCAAAGTGGCGGCCGAGACCGAGGGACGTATTAAACAGGAGCGCCTTAATCATGATCTGCACCTGGAAGAGGCTCGTGTGCGTGCGAAGGAGTACCGCGAGACGGTCATGGAAGGGATCAAGCTCGCGGGCAACACGATCGGATCTGGCATTATGGCCTTTGTCGACGACAAGGAGAAGCTCACGGCCACAGTTGTCTCCTTGTCAGCTCTGGCTGTTGGAATTTACACGGCCAAAGTCTCAACTGGTGTTGCAGGCAAGTACATTGAGGCTCGTATGGGAAAGCCGTCGCTCGTGCGTGAGACTTCGCGGCGATCGGCTGCACAGGTGCTGGCCAATCCCATTCCGTCCATCAAGCGCGTGCTGCGTCTGCAAAAGCCAACAGACGCATTGGAGGGCGTCGTGCTGGAGCCTAAGCTGGACGAGCGTCTGCGCAGTGTTGCTGTGTCCACGTTCAACACGAAAAAGAACCGTGCGCCGTTCCGCCACTTGCTGTTGCACGGCCCGCCGGGTACGGGTAAGACGCTGTTCGCCAAAGCGCTGGCCCGCCACTCTGGTCTGGAGTATGCTATTCTCACAGGCGGTGATGTTGCCCCGTTGGGCCGAGAAGGCGTGACGGAGATCCACAAGCTGTTTGACTGGGCGTCGCACAGTCGTCGTGGTTTGCTGTTGTTTGTCGACGAAGCTGATGCATTTCTGCAGAAGCGCAGCAACACTGTCATGAGTGAAGACATGCGCAATGCATTGAATGCTTTTCTGTACCGCACGGGTGAGGCCAGCGATAAGTTCATGGTTGTCTTTGCGAGCAACCAGCCAGAGCAGTTTGACTGGGCCATCAACGATCGCATCGATGAAATGGTGGAATTCCGCTTGCCGGGCTTTGATGAACGCGTGCGCATCCTGAAGCAGTACTTTGAAGACTACGTGCGTGCTCCCAAGAACTCGCGCGCCAAGAAGATTTATGCGAAGGCTCTATCTTCACGCTGGACCCGATCGCCAACTTCCTTGTGCCAAGACACGGATCTTGCAACTGGAAGTACTGCAGGGAGTGTCCGTGGAAGCGCAAATGATCTTTGACGCGCTGAACAAGAGCCCATGCGCCTCTACTGGATCGTCTTTGCGCTCGTCCTCGGCAACGGAGTGTACACGTTTGTTGCCGGGAAAGACGAGTCGTTTCTACTCGAAAAGCTCAAGAGACAGATCCCGTCCGAATACCGCATCTCGGAGAAGTGGGACAAGTGTCTGGAGAACTTTGCGCTGTACTTCGGTACCGGTCTCGTGGCCGGTGGGCTCACGTCGCTTGTTCTGGCGCGTTCGGGTGCCGGCCGCGGACTGGTGACAGGACTGGGCGTGCAGGTGCTGGCTCGAGCTGGACGACGTGCCAAATGGCGTTTGCTGGCAATGAGCAGGCCGCTCTGAACAAGACGGACAAGGCGGTGGGGGACTTGCCGGCGTTCCCCCACCAGACGATCGTGGACTTTGCCACGGCGTCCGCCACGCAGCAGCAGCTCCTGCACGTGCACCTGTGGCTTCTGTGCAGAGTCTGCCGTGCCGGTGCTGGAAGCGTACTTGGCTGAACAAGTGCAGGCCTCGAGCGTCGATGTGGATGCGAACTTGGCTCTGCTCAAGTTGTACCAGTTGTACCCATCGACACTGAAGGCAGCGAGCCACGGTTCTGATCAAGGGCGTCATGGCGCTCCCGTCCACGTTCTTCACGGGCGTCGACTATGGTCCCTGAGAGCATTCGCGAGGACGCGGATGTATCGGCAGTGCTTGACACGGGCTTTATGCTCCAGTCGTGTCTCTTCGAAGACTTTTGGAAGAAGGACGTGGCGTTTGCATCCCACGTGCCGGGCTTTCTGGAGTCAGTGCGCGCCTACATCTTGATGGCCGTCTACCGCAGCTGCAGTGTCGTTTCCACGGACGTTTTCAAGGCCAAGCTCAATGTGTCGGACCAGCAAGTGGCTGACATTGTCGCCGAGCAGTGGACGCGGGTAATTTAATTCACATCAAGCCCAATGAGCACAATCAGATGCAAGCGAAGAAGGTTCAGGAGAAGATCCAGTACGAGGACGTGCTCAAGGTCATCCACGTTCTTTCTAAATCGAACGTCTGCGCCAGTGGCGGTGGGATAACGGACGCTGAGGAGGTTTGCGAGAGCCTGGCCAAGCACTTGAGGCTGGAAGCCGGCTCGCAGGTACTGCCCTGCTCGACGGGCGTCATTGGCTGGCGCATCCCCGTGGACGCCATGGTGGCAAATCTCCCGAAGCTCGTCAATAATCTTCAGTCGGATTCGGTGCTACCTGCTGCGGAGGGGATTATGACTACTGATCTGTACCCGAAGGTGCGCTCCATGGACGTCTGTGGCGGCCGAATCGTCGGCATTGCCAAAGGAGCCGGTATGATTGAACCCGACATGGCTACGATGCTGTCGTACATTCTCACGGATCTGTCGATTCCTCGAGACGTGTTGCGCCAGTTGCTAAAGGAAGTGGTCGGAGATACGTACAACTCCCTCAGTATCGATACGGATCAGAGCACGTCGGATACAGTAGCGCTTGTGTCGTCGGACATGATCCCCTTTGACATGAA---GCATCTGGACGAGGTCAAAAAGGCACTGTACGAGGTGTGCCTCGGACTTAGCGAAGATGTCGTGCGGAACGGTGAGGGTGCGCACCACGTTATTCGGGTAAGCGTGGGTGGCGCATTGACGAAAGAGATGGCCAAAGGCGTCGGCAAGTCCGTGGTGAACTCGCCTTTGCTCAAGTGTGCAGTAGCTGGCAACGACCCGAACGTGGGCCGTCTTGTTATGGCTGTGGGGAAGTACATGGGTCTCCACCACAAGGACGTAAACATTCAGCGCCGGATGAAGGTCTATATGGGCGGCGTGCTTATTTTCGAGAACGGGGAAATGGTGCTGAACGACGACATCGAGGCTAAACTAGTGGCCCACATGCGTGACGCCATGCTGATCGAGCCTACGCGCGGCGGACTCGACGCTAGTTCGCACAACTACCCGCCACACGGAAAGTTCGTTGAGGTTGACATTGACTTGGGCATTGGATCGGAAGCGACGCAGGTACTGGGCATCGACCTTACCCACGAGTACGTGGCCATTAACGCCGACTACCGCTCGTAATGGCACCCGCGCGAAAGGGAAAGACTGCTGCTGCGTACTTGGCGTCACTGGGCGTAGCGCCTGT---CGCTGGCCACGAGAAGAAGGACGAGCTGAAGAAGAAGAAGAAGAAGGGCAAGAAGAAGCCCACAAGAAGCCCATGTCGGCGGCTGCGAAGGTGCTGA-TGGAGCGGCAGGCGGCGCTGAGGGCCGAGCAGGAGCGCGCGGAGCAGGAACGACTGGAGCTCGAGCGCATTGCGCAGGAGGAAGAGGCCAAGCGCTTGGCCGAGATGGAGGCAGAAGAAGAGCTTCGGCGCAAGAAGAAAGAGGCGCGTGCAGCCAAGATCGAGCGGCAGAAGAAGGAAGGCACGTACATGACGAAGGCGCAGAAACAGAAAGCGGCCAAAGCGCAGGCGGCATTGGAAGCCATGCAGCAGGCCGGTCTTGTGCCGGAAGCTACT------GGCGAGAAGAAAAAGGTGGTGTATGCCGACAAGAAACGGCAAAACTATGAGCGGACGAAGAGGAAGAAGAGGAGACTGAAGCGCCCGATGCGTGGGACGACGACGACTGGGAGACTAGCAACGTCGTGGCCTCGCTGGAAGATAAGCTAGCAAAAGAAGGAGCAAGAGCGTCTACGTCAGCAGGGCATTGCGCTGAAGAAACGCCAAGAGGAGCAGGCAGAGCAGATGCGAGCTGAGGCTGAGGAGGAGCATCAACGCTCCAAGGCCAAAATCGAGGCGGACCGTAAAAAGAAAGAGGCTGCTGATCGACGTCGCGAACGCGAGGCCGTTGCACGAGCCAACGTGTCTCTGGACAACCTGCGTTCCCCCATTTGCTGTATCATGGGTCATGTTGATACCGGTAAAACGAAGTTGCTTGACAACATTCGTAAGACCAAAGTGCAAGACGCCGAAGCCGGTGGTATCACTCAGCAAATCGGTGCTACCTTTTTTCCTGTGGAAGCTATCAAGCAGAAGACGGCTCGGCTTATTGAGACAATGAAGCTCGAGTTCCGTCTGCCCGGTTTGCTAGTGATTGACACACCTGGCCACGAGTCATTTACGAATCTGCGTAATCGTGGTTCGTCGCTATGTGACATCGCCATTTTGGTGGTTGACATAATGCACGGTTTGGAGCCTCAGACTCTGGAATCGCTACGCCTACTGCGTCAAAAGAAAGCGCCGTTTGTCGTGGCACTCAACAAAATCGACCGATGTTACGGCTGGAAGACGATGCCTGATATGCCTGTCCAGGAAGCGCTGAAGCATCAGAACGAGCACGTCATTCGTGAGTTTGAGGATCGAATGAAAGCGATTATCGTGGAGTTTGCCGAGCAGAAGTTAAATGCCGAGGTCTACTGGCGCAACAAAAACCTGGCGCACACAGTGTCGCTTATCCCTACCAGTGCCATCTCCGGCGAAGGTGTGCCGGACTTGTTGATGATGCTGACACGCTTGACGCAGGAGCGCATGGCCAAGTCTTTGGCCTTCGTCGACATCTTGCAGTGTACCGTGCTAGAAGTGAAGGTTATCGAGGGTCTCGGTACGACAATTGATACGATCCTGGTAAACGGTACACTGGAAGAAGGGTCAACCATCGTAGTGTGTACCTTGGACGGGCCCGTAGTGACAACCATCCGTTCGTTACTGACTCCACACCCAATGAAAGAGATCCGAGTCAAGGGTGAGTATATCCACCACCAAACGATAAAGGCTGCCATGGGCGTAAAAATCTGCGCGCAAGGTCTTGAAAAAGCTGTAGCCGGTACACAGATTTACGTGGTTGGCCCCGACGACGATGTTGAGGAGCTGAAGGATTCTGTAATGTCCGATCTAACGAGTATTCTGGACTCGGTGAAGGCCACCCGTCGTGGTGTGATGGTTCAAGCTTCCACTCTTGGTGCTTTGGAAGCGCTTCTGGAATTCTTACGCACATGTGATCCGCCTATTCCGGTGTCCTGTGTGAACATTGGCCCCGTATACAAGAAAGACGTGATGCGAGCTTCTGTGCAACTCGAGCACCAGCCGGAATACGCGACCATACTGGCGTTTGACGTGAAGGTTCACAACGACGCGACTGAGTTGGCTTCCGAGCTTGGCGTGCGCATTTTTACAGCTGACATCATTTACCACCTGTTTGACCAGTTTACGGCGTATATGGAAAACTTTCGGATGGCCCGTCGCGAGGAATTCGAGGAAGTTGCTGTGTTTCCTTGCGTGCTCAAGATCGTTCCCAACTGTGTTTTTAACAAGAAGGACCCAATCATTCTTGGTGTCGACGTTGAAGAGGGCATTCTCAAAGTAGGCACGCCTCTCGTAGTCCCATCCGCCGGTG---GCTTCTTGGTCGGCAAGGTGGGTTCGATCGAACGCGAGCACAAAGAGGTCGACCGTGCGAAGAAGGGTGCATCGGTTGCTGTGCGGATAGACAACGAGAGCAGTGTCATGTACGGCCGTCACTTTGACCACAAGAACAAGCTAGTGTCGCGCTTGACCCGTCAGAGTATCGATGCGCTGAAAGAGAATTTTCGGGATGATCTTCAGAAAGAGGATTGGCAGCTCGTGATCAAGCTAAAGAAGGTGTTCGACATCATTATGGAGGGCTGGCTCACCAAGCGCCGCGACCACATGAACCTCATCTGGCGGGACCGGTACTTCCTCTTGGACGGCAACCAGCTGCGCTATTTCCGTAAACGGGGCGACTCGGCCCCTCGCGGGACGTACATCTTGACGGACAACTGCGTCGTGAGTCCCGTGTACAACTCGGAGGAGAAGCACAAGCACCACGAGCCCGTGTGGATGTTCCGCATCACGTGAAAAGTCGTACAAGAAGGACCGGTTCCTCGATCTCGGAGCCAAGAGCGAAGAGCACGCAAAAGAGTGGAAACGCGCAATCGAGAACGCCGTGCGGTCGCTCCAGGAGCTCGTGGCGCACCAGCGCAAGCTCAAGACAGACGCGGAGAT---CTTTCCCACGCCCACGCAC---------GTCCCGAGGATGCTCAAGGCGGACGAAGACACTGTGTCGCTGTTCAACTTGGATGCGGATAACGTCAAAGCGGAGCAGGCGTGGTGGCTCGCACGGGTGGAAGGGGGACTGCGGATCATGCAGGAGTGTCCGCTGGGACCGAACGTTGCAGCGAACCTGTACGCGCAGCACGCAGCGACCACGAACGGGCTGATTGCGAGTGCTCTGCTCGGCGCTGCACTGAGTTTCAGCAGCATGTACTCGTTCGCTAACAATATGCTGCTGAGTTTCCTGACTGCGCTCGGAGCGATTGCAGTCTGCTGTGTCGCAGTGACGCCAACGCAAGCGCCCGAGATCCCGAGCTTTCGTGTCTCGCAAGTGGTGCATGGCTCGCCCACTGAAGTCTTTCGACTCATCATGAACTCGAAGCGATTCCAGCGATGGGACGCGGCAACTGCGACGATGCGTGTCGTGCAGCAGCTGGATAACAACGCGGACATTGTCTACGTGACGCAACGTCCGACACGGTTGTGGCCGTTGTGGCAGAAGGCTCGTGATCTTGTGTTTATGCGCTACTGGCGACGCGAAGAAGACGGCTCGTACTTTGTCATGTACCAGAGCATTGATCACCCTGAGTGCCGCGTGCGTCACAATTACGTGCGAGCAGATATATTGGGTGGCGGCTTTGTGATTGCGCCGCAACGCGTTCCGAGT---GGATCGATTCGCACGCTGGTGTCGTACGTGCTCCGATACGACCCTGGCGGCTG---GTCACGCATTTACCACCAGCTTGGCATGGATGTGGACTTGGTCATGCCCATGTTGCGCAGCGTTATCGGCATTCGTGATGAAATGAGCGCCACTGACTTTATCACTCCCAACGTCTCGATGGCGCCTGACGCCGAGGACGAGCGCGGATCGCGAAGCGTAGA---CTCTATGGGTGTTGTCATGGGTGTGCAAAAGCTGCAG---ACGAGTCTACCGGAGTCGATGTGGGCCGAGCCCGACGCTTCGCTTTTCAGTGTGCGTGGACACAACTACTTGAATGACAAGAAGAAGATTCCATCGGCGCCAGCAATGTTCCACGTCGTGGGTGTGGACTTGCTGTCGTTCGAGAGTGCAGCTGAGAGATATGATATCTCGTCTCGTGTGGACAGCATTGGCCGCACCAG---CTCCAAGTTTACGTTTGTCGTGAACATGATCATTCCAGGCCCAGAGAACACGTGCATGGTGTTCTACTTCCACCCGGCGCGCGACAACGTGTTCGAGGACGGCTCACCGTTTTCCGAGCTATTGAACGACTTCTTCGATGGCGACGACCAGTTCCGGAACTCGCGCTTCAAGCTCATTCCCACTGTCGTGGAGGGCAGCTTCATCATCAAGCAATCGGTTGGGTCTAAGCCGACGCTGCTGGGCAACAAGCTCAAGTGTCTGTATTACCGCGGCGAAAATTACTTCGAGGTGGACATCGATATTTCGTCCAATTCGGTTGCCAACACCGTCGTCGGAATGGTACAAGGTGTGACCAAGTCGTTGGTGGTGGACATGGCTTTCCTGCTTGAGGCGCAATCCGACGAAGAGCTACCTGAGATCATTCTTGGCGCTGTTCGCTTGCAGCACGTTAGCCTCGACAATGCGCGCCGCAAGCCAAAGCATGGGCAACGAATTCTTCTGGCACTGCTGCGTTCCGAAATGCTAACGGAAGTGCTGCACGCGAATCTGCAGCAGCTGATCGCTTATGCGCACTCGGCTGACACGTCGCTGCAGCGAGAAGTGGCCGAGAAGTTGGCCAATGAGGCCGTGAAAATCGACAGGTACAGATCGTGGAGCTGGACGGATTGCAGCTGTTGCTGCCACTGACCAAGTCGACGGACACGGAGGTGCAACGCCTTGCGGCGCACGCGCTGGCCAACCTCTCTGTGAATTCAGAAAACCAGTCAAAAATGGCGACTGAAGGAGGCATTGGGATGCTTATTGAGCTCTTGGGCAGCCCGAACGAGCACGTACAGCGTCAAGCTGCAAAGGCGCTAGCAAATCTTGGTGTAAACGTGGACAACAAGGAGCGAATCGCCAAGGCGGGCGGCATCAAACCGCTCATTGACTTGGCAAGCTCCCGCCAAATAGGCGTCGCTGTCGAGGCCGTTGCGGCACTTGCCAATCTCGCCGTCAACG---ACGCTAATGAGGTGGAAATTGCACGCAAAGGCGGCTTGAAGTCTATCATTGATGGAGCACACTCGGAGTCGATGGAGCTTCAGTCGCAAGTGGCACGTGCACTGCGCAATCTTTCTGTCAACCCGGAGAACAAGCAAGCGATCGTGGAGTTGGGTGGAGTGGAGGCTCTGCAGTCGCTGTTGCGGTCTACCAATGACCGGATATGCCAGCAAGCTACGCGAGCGCTCGTGCTGAACGTGGTGCTCACGCGCTCGTCGCCGTCCATCTCGTGCGCGCCGTCGCGCCCGTCGCGC---TGTGCGCAGCGCCGCACGAGACG---AGACAGCGCTACGACCACATGGTCGTGGCCGTCGAGGCGTCGTCGACGAGCCAGACGGTGA------ACGGCTTGCTGGACCCCACGCGCC---ACGACTCGGCGCGA---AGCTTCTTGTCCATTGCCGACGTCGCTCCGCTGTCGAACGTGTCGCTTGCGAAGGTGGGGACG---CCGTCGACGGCCCGCTA---CGTACTGGACG---AGAC------------------GGTGAATGAC-------------GCGACCGTG------CGAGCAACGAGAGACAGCGACGAG---AGCGCGACG-----------------TGGGCGACGTGTACCGGTACCACGCGACGGA--CGTGGAAGAAGACGAGATGGAGGAACTTCGTTGGAAGATCCTCGAGCAGGTGTTCCAGCGGTACTCGCAGCAAGAGCAAAAGTGCGAAGAGTGGGGCCGATATACGGAACAAGACACCGAGTGGCCTTTGTGCACGGTGCAAGACGCGCGAGTATTATGGCCGAGAAGAAGTTCCACGACAATGCCGTTCGTGACGCTATGGGCAAAGCGAACGCTACGTGGGTGGAAGAGCGACGCAATCGGGTGCAGGAGGAAGTCCGTCAGTTTTACGACGAAATGCGGCACAAACAGGCGTTGACGCTGACCCGTCTGGAAAAAGTGTCGCCGTTCTCCAAGTTTCCGGTTCTTGGCAACCGCTTTTTACCGCTTCGTCTGCGAGGAAAAGGAGGCCACGGGGAAGTGTGGGACGTGGTCGATTATGCTAACAACAAGGAGCTTTGCGCTCTCAAGCTGTCGACGTCCATTTGCCACGCCCAGCGCGAACATCGCGTGCATACTCGGCCACCCGA--ACATTGTTCAAGTTGGCAAAGTGTCGTTCATTATTCGATACCAGCGGCGACTCTACACTGCGTTCACTGTCGCTAGTGTCCATTCCGACCTGCAGCAGCTTATTGAGA---TGTACGGGTACTTCGACGACGACTCTGCATACAAGGTGCTTTCGCAGCTATTGAGTGCCCTGAAGTACTTGCACGATGGGCGTTGCTCACTACGATTTGAAGCCGTCGAACATACTGATCGATCAAGACGGGTGTGTAAAGCTG------ACTGACTTTGACCTGGCGCGCGATGTTAAATCGCCTACGTCAAAGTCTACAGTCGGGACTCTACGGTACCTTCCGCCTGAGTGTTTTTGTCCGAAACGGGGTGA---------TTGCGCAGCCACAGCCGAGAAGGCCGATATGTGGATGGTGGGAGTCGTCTATTGCATGATGATCACTGGAAAACACCCGATCTGTCCAGATAAGCCGACCCATGCGGAAGTGAAGTCAATGATGAGTCGGTACAAGGGC---GAGTTACGCTACGCGCGGCCTGTTTCGGATTTGTCAAGGTGGATCTTACAAGGATGTCTTCAGCCAGATCCACGGTGCCGACCAACGGCCGCGCAATTGCTGCTGGCACTACAA

Peronospora_belbahrii TCGATTATCACCGTCAAGTCGCCCACGAGCTTATCACGTTTTTTCATTCTTTCTATCGCTTTGGCTCAGCTTCGAACCAATTTAATTTATTTATACAGAATGTTCTCTTTGGTTCACTCTGCATCGGTGCGATTGTCTTTCTCCTGCTCTCTCTTATCGTCATACGACGCACTATTTTGCACATTCCTAAGCTTCTGTACACCGCATTTTGACAAACTCTACGTCCGTGGAGCTCTTGGCAGTGAGTCTTTTCACGTTTCTAGCAATTAGCGCCCTTGCCGGACTACTAGGAGAAGCCGTCTACAGACAAGTAATGCCTTGCGTGCTAATGCTGACAATCTGGTCACATCGTTTAATGAGAGTGAACTGCCAGCTGCTGCGTTCAAGCTGTCGATTGAAGCACTGGGCTCGTGCACACGTCTAAAGACCTATGGAATCAAACA---GCGGTGCTACCTCAAAATTACTCAGATATGGCTACGAACTGGGAGTTGAGCTACTTTGCTTTAAAGTTATCGACGAACGGAGCGATTCTTGCCGTAGCGCTTGCCAGCTTCTTGTCCATCGCGTCAGTTGGATGGGCGATGGTCTCGCCTCTTCGTGTGTCCATTCTAGTGATCCTAAGTGTCGTGCCAATTTCGCACACGCTTCTTGGTGCATACCTGTCGTCAACAATCATGACAGCGGATTTTTGCGCTGCACCCATGAACAACACACTTAATCTTGTGGGCTCATGTCCGGCAAATGCGTCTTGCCTTTTGCTGACGGTGTGGCTACGCAACGCGACCGGTCAAGGAGATGCCGGTCGTCGCATGAAGAAGGAGTTTTTAGACCCCATTGGAGCAACTGTGGGTGTCATGCTTGGGTATTTTGCTGGAACGGTGAGCAATTGGGAGAACGCTTCACTCATTCAAACATGGCGTTGGCCATTTCTCTTGCAATTTATGGCTCTTGTGCCGATTAATATTGCCATCTTTTTCGTACCCAAGAGACACCTTGTTATTCGTACTGAGATCAGTGAGATGGACCCGCCTGTGGTTGGCACGCCCATGAGTTCAAGATCGACAACGTATCATGACGAAGAAGACGCGCTACTCGCGAGTGCAAGTATGTAACATACGGCGCCCTAGACGCGACGATTACCAACACGGCGCGTTCTCGGACGGTATGCATGAGCTTTTGCACATACCTGTGTTCTGTTTGATCGTGTTTGGCCTCACAACAGTGTACTTCGTTGTCACGGGCGTGCAGTACTGGAGCACCATCTTTATGATCAAGTCGCTGCACGCGTCCAAGTATTTGGTCAACGCGCTTTTTGTTGTCGTATCTGGGACAGGACCAATCTTGGGCGTATTTTTTGGTGGATGGCTTATTGACCGATGTGGAGGATATATTGGCGTGGAGCAGCGTGCGAGAGCACTGGGCATTTGCATGATTTTGGGGCTGACAGCCTTCTGTATATCGGCGGTGGCGACGTTCTTCAACAACGTTTACGTTACCTCCGCTTTCTTGTGGCTGTTGCTTTTCTTCGGGGGTGCTATCCTACCAGCGCACCGTGCGCTCGCTTCCTCGTTTTCTGTTATGATCTTCAATCTGTTTGGGTACTCGCTCTCGCATGAGTGATTTACAAATGGTCATCGGTGCAAAATGTTTTGTACCAGACGAGACACACGTGTGGCTTGCAGCTGAAGTATTACGGGATGAGAATTCAGGTGATGGAAAGACACGTAAGATATTTTGTAAAGTGGAATTACCTGATGGCAATGGAAGAACGATGTGTGGATATGTTGGAAAAGAAAACAAAAGCATTATTGCTGTACATCAACTTGAGACTTTACCATATCAGAATGAAACGGGTGGATCGGATGGGATTGAAGATATGATTACACTTAATTATTTACATGAGGCTGCTATTTTGTTTAACATTAAGAAACGTTTTCTATGTAAATTACCCTATACATATACGGGTGATATTTGTATTGCAATTAATCCTTATCAATGGCTACCTGATCTCTACACGGAGGAACAACATTTACGATATCTTCATCAACCAAAGGAAGAACTGCCTCCACATGTTTACGCTACATCAGTTGCTGCATACGATAATATGCGACGATCTAAATTGAATCAATCGATCTTAGTTAGTGGAGAGTCGGGTGCTGGAAAGACGGAGACGACCAAGATTCTGATGAACCACTTGGCTACTATTGCTGGTGGTATGAATAATTCAACGATTAAGAGAATTATTGAAGTCAATCCACTTCTTGAGTCGTTTGGTAATGCCAAGACAGTACGAAACGATAATTCCAGTCGATTTGGCAAGTTTACACAACTACAATTTGACAGGAATGGTACGCTCGTGGGTGCTAAATGCCGCACGTATTTGCTAGAAAAGACACGTGTCATTCAGCACGAAGCACCGGAGCGCAATTACCACATCTTTTACCAGTGGACTCTTCTGACATTGCATCAGAGCTACAGCTTGACTCGACAAAAACTTACATGTATACGGGTAACAACACGGCACAAAAGATCGAGGGACTTTCCGACGAGAAACACTTTAACAAGACACGTGAGGCACTGGAGCTTGTCGGGCTGTCTCGTGATGATCAGCAGCCACTGTTTGAGGTGCTGGCAGGTGTGCTTCACCTTGGTGAAGTGCAGTTGCAATCAGATGCATCAGACGATGAGAAATCTCTCATTGCTGTGGGTGACAGTGGTGCGAATGCTGCTACTCAGATGCTTGGTGTCACATGGGAAGCACTTGAAGCTGCACTGTGTTCGCGTACGATGCGTGCCGTAAACGATGTGTACAGCGTACCGCTGAAGAAGGAGCTGGCTATGGATTGCCGTGACGCACTTGCCAAAGCAATTTACTCCAACGTGTTTGACTGGCTTGTGGCCACGATTAACCAGTCCCTGGCTGACGATGCCAATATGTCAAACCACGTGGGTGTACTCGATATTTTTGGTTTTGAGCACTTCAAGCACAACTCGTTTGAGCAGTTTTGTATTAACTATGCCAACGAGAAGCTTCAGCAAAAGTTCACGCAGGATGTATTCAAGACTGTCCAGATTGAGTACGAGGAAGAAGGTATCGTATGGGATCACATTGAGTACGCGGATAATCAAGATGTGTTGACGGTGGTTGAGTCGCGCATGGGCATCATTTCTCTGTTGAACGAAGAGGTAATGCGACCAAAGGGTAGCGAGGAGTCCTTTATGTCAAAGGTGACATCCCTGCACAGGGAA---GACATGGCACATGTTATTGAATTCCCTCGTACATCACGTACTGAGTTCCTGATAAAGCACTATGCTGCTCCTGTCATGTATGATTCGGTGGGATTCTTGGAGAAGCACAAGGATTCGCTGCTGCCTGATCTGTCTGAGCTCATGCGTGGTTCGTCTAAACCCTTCATCGCGAAGCTCTTTGAACCGAAGCCAGAACCCAAGTCGACGAAAGC---------GTGGTGGTGCTTTATCTATCACGACTGTCGGTACTCAATTCAAAGAGAGTTTGACGGAACTGATGGCGACTATTAATTCTACTCGAGTCCACTACGTGCGTTGTATTAAGCCGAATCCAATCAA---------AAGCTCGACGGTTATGGATCAGTCCATGGTGGTGTCTCAATTGCGATGTGCTGGTGTGATTGAAGCCATTCGTATCTCTCGTGCAGCATACCCGAATAGGTTGCAACATACAGAGATTTTGAGCAAGTTTTGGCTGTTTGTACCTAGCGGTGGTAACACTGCGGTAGAAAAGTGTCAATTACTTATGGACAAACTGAAACTGGAGTCTCCAGTGCAGTACCAAATGGGTAAGACCCGTGTGTACTTTCAATTGGGTGTACTTGAAGAACTCGAAGATCGTCGTAAGAAGTTCCTGGATGCCAAGGCCACATACCTACAGAATGTGATGGTTAGTTTCACGCAGCGCATCAAGTACCTTCGACAACTGGAAGCTATCATTAAGCTCAGTGATCAGGTGCGTGATTGCTATGCGGAGATACAACACGTTCATGAACGGCCTCATCTTGGCGCAAGCTCAATGGCGAGGCATTCAGGGTCGGAAGGTGGCAGCGGAGGTCAAGAGCAACCACTATGCTGTTATCATTCAGCGGTACGTGCGCGGCTATGTCAAGCGCCACCGTTACGTTAAACTGCGGGCGATGGTTGTCCGCGTCCAGGCTGTGGTTCGTATGACGATTCAGCGACCGAAGTATCTTGCTGCGCTTGAAGAGAAACGTCATGAAGCTGACATGGCTTACCAGCTGGACAAGTTGAAGGCCGCTTTGCAAGAAGAGCAGGAAAGGAATGCCCAGCT------GCAGCGCC---GCAGTAGCGTGAC--GACAGCGCGACTGCTGCATCGAGTGTGGTGATGGCTGATGCTGGTGGTATGATTGAAACCCTTACAGATGAGAACAAGAGGCTGCGTGAGAAGAACGAGGAAATAAAGTCTGCGATGAAGGGATTAAAGGCGGAGATTGAGAAGTTCAAGAGCGATAAGGAGTTCTCATCGGCTGGCAATTACGTCAAAGTCTGTCAGCTGCAGGACACTGTGCGCGAGAAGGACAAGAAGATCAAGATGCTCGAGGCCGACAACAAAAAGCTGGTAGAGCAGCTTGCAAAGATCCACGTGAACGG---TTT---GCCCGAGAAGAAGTCTACGCAGAAGAAGTCAATCTTCCGTACGCTAGGGTCCAAAAAGGAGAAGCAGCCGC---------------GTGAGGCCGTGCTGATGGACTCGCTCACTGGCTCGGATGACCTTAATCGTCGGGAGACGCGTGGATCGGA---GCGTCAGTTCCCTCGCATGC---CGACTATGAGCGTAGGCTCT---CGCTTTTGGAACTCAAGCAAG---AAGGGCGAT------------AACGGCGACGGCTTTGACGTCTCGGAGGTTGACGGTGAAGACAGTCCCACGAACTAACGCGCAACACGATCGTGAAGAGCATGGAGGTAGGAGTGACTGGTGCCATGTCGAGTCTGAAGGGTCGAATG---------TCGGTCGTGAAGGGCATGTATGAAGCAAACGTACAAACAAGATCGCCCTGTGCGCGAGTCATTTAACTTAGATGCGTTGCCAGAAGTCTCTCTGCCTGTCGGTTGGGAGGCTAAGGTATCCCGCTCGACGGGACGCGTGTACTACGTGAACCGCAAGCTTGGCAAGTCCCAGTTTGAGCGCCCCACGCTAGCTTCACTGAA------GGCGCAAAAGCTGGCGCGTCAGAAGTCGGCTAACGTGGAGTAAGGCGGTTGTGGCAGATTTTGGCTGTGGAGATGCAAGGCTGGCAGAAAGTGTATCGAACAAGGTGCATTCGTTTGATCTCGTGTCTCTTGACATTGGCGTCTACTGTTTGGCACTTATGGGCACAAGTGTACGCGAGTATGTGCAAGAAGTATATCGTGTGCTCAAACCTAGAGGCGTGTTGATGATTGCCGAGGTAAAAAGCCGCTTTGAGAGTGAGACACTGGGTGGATGGTAATAGTGTTTGTTGCGTCGGCGCAAGGCGTCGTGGTAACAGTGCGCAATACGACAGTGTTTGTGGAGAGATGAAGCACGTAGTCATTTTGGGCCAGAAGACGGGCGAAGTTGTGGCTGTGCCCTTCCCGGATGTGGCACGCGACCTAAAGATGCTATTAGGCCACACAACGTCTATGATTACCCACGTGGCTCTCAATCTCGTCCCTGCTTTTGACAGCCGATCGTGATGAGAAGCTGCGTGTGTCTAGGTTTCCAAATGCTGCGATTATTGAGAGCTACTGCTTGGGTCATGCTGCCAGTCTGACCAAGGTGGCTTGTAGTTCTGTCACGCCGGAACTTGTTGTCTCGACGTCCATAGACAACACGCTAAAGCTTTGGGATATGACGACGGGCAAGTTGTCAATTTGCTCTAAGACGACATTGTGGCAGTCTTAGTAAACTATCAGTATGTGCGATTCTTTGAGATTGAGAGAACCCGTTTCTTCAGTTATATTCCATTTCAAAAGAACTCTAACGTGTTAAACTGCTGTGCGTGATTTTCGTAAGCTTCAGAATGATCCACCATCGGGTGTATCAGGTGCTCCTATGGACAATAACATTATGCTCTGGCAGGCGGTTATTTTTGGACCAGATGATACGCCGTGGGAAGGTGGAACGTTTAATTTGACGCTTGAGTTTAGTGAAGATTATCCAAACAAGGCACCGACTGTCAAGTTTATTACGAAAATGTTTCACCCAAACATCTATAACGATGGTCAGATCTGTCTGGATATTTTACAGAACCAATGGTCGCCGATCTATGACATCTCTGCCATCCTCACGTCCATTCAATCGCTGTTATGTGACCCGAACCCGAACTCACCGGCAAACTCGGAGGCTGCACGCCTTTTCCAGGAAAATCGACGCGAGTACAATCGACGCGTACGTGAGATTGTCGAGCAAAGCTGGCAGGCGATTGATGGTGTCAGCTGCTAAGATGCGTGGCGCTGAGAACCGTATGAATGCTGGTCGCCCCTTTACGGCATGGTTGGACAATGTCTCGTCAGACCGTGGTCTGTGCGGTGGTATCAACTCAGGTATTGCCAAAGCTGCCCGTAAGCAGAACATTCACAAATCTTTGTGATTGGTGACAAGGCTCGAGCCCAGCTTCGTCGTGACCTTGGCAACTGCATCCGCGGTAATGTAACCGAGACGTTCCAGTCGCCCCCTAATTTCATGGTTGCTAGCGCCATTGCGGAGGCTGTCGAATCGGAGAAGAGTCGTCGCGCATGGCTGCGATGGAAAACGCCACCACAAATGCTGAGGACCTCACGCTCACGCTCGTGTACAACAAGGCTCGCCAAGCCCGTATTACCACGGAGCTGATTGAAATTATCTCGGGTGCTGCTTCTCTGGACCATAACTGGTAGTGTTCTTAACATCCAAAGCGTGAGTCAATTTGATGAAATCACAGCACGTGATTCGACTCTTTCAATCACCTTTTTCTGGGCTGATTTTCAAGTTTGTCGTCCAAATGGACAATTGGATATCGGGTCGTTCGATATTGCATAAAGTAGAAGGCGCGAATGTCGCGAAATTGGCGAAACACGTTTACAAGATCGTCTGAAGAAACTAATCCATGCATCACCCGTGATGCTCTTTATGAAGGGTAATCCAATGGAACCGAAATGTGGGTTTAGTCAAGAAATGATCACATTACTGAATGAAGAGAAGATTCAGTTTGGTACTTTTGATATCTTGACTGACAATGAAGTACGTCAAGGATTGAAACAATTTTCAAACTGGCCGACATTTCCACAGCTCTATGTACATGGTTGTTTGATTGGTGGTCTTGATATTGTCAAGGAAATCAAGTCTGAAGGATCGATCCGTGAACAATTGAGTGTAGAGGAAGCGGAAGCAATACTCCAAGAGAGTCTTGGTGCATTGGTACATTCTGCACCTGTGTTGCTATTTATGAAAGTTTGATATTTTATTTGGAGCTATCAACGTCAGGATCAAGCTATCACTAAGGTAGTGTCTCTGCAGCCATCGTCATTCCGCGAGAGTATGTGCGGAAGCCATTGCATGGAGCCCGAAACCGTATGGCCATGGCATTCCGTGATGCTCTCGATGAAAAGGGAGAGTTTTGCATTGTGCAGCTTCCCGTATCACGTCAAAATGCACTACTTTACATCCAAGAGGTATTTCTACAATCGGCTGGCAAGTGGAGCGTCCTGCACCGGGACCACCGTAATGAAGTTCGATCGCTTTGCGTTCATTCCAAGCGTGATTGCTTGTACACTGGTGGTTTTGACGGGTTGTTGATCCGGTATGACTTGAACAAGGGGCTTACGGAAACCGTGATGGAACGTCGTAAGCATATATCTAAGATCAACTCGGTCCTGGAACATCCACACAACCCAAATTTGCTGTTGGTCAGTTGTGTGGAGCAGGAGGCACAGCTTGTTGCTGCATGATCTTCAGCGCGATGACGCCAATGTGACGAGTGGTTCAACGAGTCAATACGTCGTGCCGCGGTGGTCGCCTGCAGGATATCACGTCTCATGTGGTAGCAAATGCGATACTGTCATAATAGCTTTTCTACGCCTACTTCTGCAACTATTGGTGCTTCGTTTCTACAAAAGCGTGTGATTATTGGTGGAAAATTAACGCTTCAGATTTGGGATACGGCTGGTCAAGAACGTTTTCGTTCAATGGCGCCAATGTATTATCGCAATGCAAAAGCTGCAATTCTTGTTTTTGATTTACAGAGTGAAGCAAGCTTTGAAAAGATTAAAGAGTGGCTACATTTGCAACATCATGTGGATGATAACATGTGGGGAATAAGAGTGACGTTTTGACTTTTCAAAGGCTCAGCAATTTGCAAATGAGATTGGAGCGCTTGTGTTTCGCACGAGTGCCAAGACGGGCGAAGGAGTGCAGTCGCTGTTTGAAAGCCTAGCGGTACAATTGCTTAAGAAGCACGATCAGGAACAACGGAGAGTTTCTATGTGGTGTATGTACCGGAAGATGAGAATAAAGATCTCGAGGAGTGGGCAATTAAGCTACCGGCAGACAAGGAGGAGCAGCTAGGTTGTTTAACGGAACGGCTACGCGAGCATTTCAAGGGCGGAAGCATTCAAGCAACAGCAGAAACTTTCAAGCAACAACTGCTTGCCCAATTACCCAAGGGCGCCGCACTTACCGATGAGATGATGAAGATGATGCATGGATAGTCTTGTGGACTCAATTCCGCTTGTGCTGAATACGTCGGACGTCAAACATATTGGAAGTTAAGGGTGATGCTTTCATTGGACGGGTCTTTGATAGACGATTTTGTGCGTATGGACTTCCGGTTGAGCGAGGTATCAGGTAGTGCACAGTGGATGCAGGTGGCAAAACTTCAGAAGTAACGCAATCATTGGAGGATATTCTGGCACTAGGTCAAGGTCTAGACGATCAAGACTACAAAGCTGAGCTTGAGGTGCTTCAGAATGATCCAAATTCTAATTTATACTCGGCCGTTACATTTGAAGAGCTAAATTTGCCGGAATCGTTGCTAAAAGGTGTATACAATATGAAGTTTGCGAAACCATCCAAGATTCAGTCCGTCGCTTTACCGCTTATTTTAGCAGATCCACCTGAAAACCTCATTGGTCAGGCACAAAGTGGAATCCGACTCGTGAACTTGTACGACAAATTATGGCCGTGGTGAATGCAATGGGCAAATTGTCGAGACATTTCTAGCTATTCCAGGCAATGATTTGGCACGTGGAGAGAGTTTAAAGGCTCAAGTGATTGTCGGAACTCCTGGTAAAGTGGAGGGTATGATCAAGAAGAGACAGCTCGACACTCGTGATGTGAAGATTTTTGTACTGGATGAAGCCGATGTGATGGTGGCTGAGGACGGTATGCGAGAGCGTTCCGTGGCTATTAAACGTCTAATCAAGAACCGTGCTTGCCAATATCTTCTTTTCTCGGCCACGTATGCTGACGACGTGCGTGATTTTGCCCAGAAGATGGTGCCGGATCACAATATCATTACGGTCAAGAAGGAGAAACTTACTCTTGACGGGATTAAACAGTTTTGGATTGACTGCAAAACCTGTGACAACAGGTTTGAGGTGCTTTCGGACATTTTTGCCGTCTTGACGATTGGAAAATGCGTCATTTTCGTGCAGCAGCGTGAGACCGCTAAGGAACTGACGCGTCGTATGCGTGAGAAGGGCCATATGGTTGGAATCCTGCACGGTGCCGATATGGCGAAGGAGGTACGCGACCAGGTCATCGATGAGTTTCGTGCCGGCACGACCAACGTGCTGATCACGACGAATGTGCTTGCTCGTGGGATTGATGTGGCTGGTGTGTCACTTGTCGTGAACTTTGACATCCCACTTACGCGCGACCATCGCCCGGATCCTGAGACTTATTTGCACCGTATCGGTCGTACGGGTCGTTTTGGTCGCAAGGGTTGCGCTATCAATTTCGTGTACGACAACAAGAGTAAGCAGGATCTTGCAGAAATTGAAGAGTATTACGCGCGGCCCATTATTCAGGCACCTGCAGATGACATTGAGGAACTCGAGAAGCTACTAGCGTGGGGGGTAGTAAACGTCGTTTAGCTAAATTTGGTAAAGAACAACGCAAAGGAATGCGAATTTATCCACGTGAACCCAAGAAAAAGACAGGAGGCAAGGAGAAGACGTATTACTATCTGAAAGACGTCATGTTCGTAGCTCATGAACCAGTTTTGGAAAAGTTTCGAGCGTTTAAAACGTTTATGAAAAAGATCAAGAAAGCAATGGGAAGAAAGGATTATCAAGAAGCGAAACGAAAACATGAGAATCGACCACTGTATGAATTGGATCATCTTGTGAAAGAACGGTATCCACGCTTTATTGATGCACTTGGTGATTTAGATGATGCCTTGTGTGGCAGAATTATATTGTGGTGACTCGATCACTTACAAAGGTTTTTGTAAGTATTAAAGGGATTTACTATCAAGCTCATGTACAAGGTCAGACGATGACGTGGCTTGTACCGCATCAATTCACACCAACTGTGGATAAACGAGTGGATGTGAGAGTTATGCTCACGTTTTTAGAGTTTTATCAAGTACTGCTCAAGTTTGTCAATGCTCAATTGTATATGCAGATTGGAGTCGCGTATCCTCCAAAGATTAATTTACATCTTGATTCAGCAGGTGCTCTACTTGCTGCGATTGAATTAGCAGCAAAAGGAGTCGGAGAAGCGACTCAAGCTATAGTGAGCAACGCAAGGAGCGCAGTGAGAAGAGTCTCTTTAGTGGTCTCACCTTTTTCTTGTCTCGTGAGGTTCCTTCGGCTTGTTTAGAATTGGTGCTACGCTCGCACGGCGCTACCTTGGGCTGGGACGGCGCTGGTTCTCCGCTTGATGAGAAGAACACGTGCATCACGCATCACATTATGGATCGTCCTCATCAGGGTCACCGATACTTTAACCGTGAGTATGTCCAGCCGCAGTGGGTGTTCGACTCAGTAAACAATGGCATCTTGCTGCCTCTGGCGCGCTATCGCAAGGAGCTTGAAAAGCTGAAATCAGCCTCACAGGTGCTGCGCGAGGTTGGCGCTGTTGGCGTCGAAGATGAGGACGAGAAGCATACGCTGCTGGTCTTAAGGCCGAAGTGGACGGCAGCAAGAAGTCCAAGCGTGTTAAGAAGGAAGAAGATGCCGCCGAGCTCAAGGAGCTAGCAAAGACCATGATGTCGAAGAAGGCCAAGCGTCTCTACGACCGTATGCAGTACGGTCTGGGCAAGAAGGCTGACAAGATTCAAAAGCTCGAGGACAAACGTGCCAAGCTAGAGGCAACACTAGAAAACTTCTTGCAGGCGCAATGTCAGGAATGATTTCGGCTCTAGCATTCCAGCCACTGGGGCGGTACGATGGCGTGGCACGTCGCCCACGCTCGTGCGTGTAGCGGGTGGTGCTGGTCTCTACTTTGCCACGTTAGACTACTGCCTGCATATGTTTCCAAGCTCTGCAGCCAACACGTTTTTAGCTGGAGCCTTGTCACGAACATTTGCCGGCGGAATTATGTCGCCTTTGACTATTGTAAAAGCCCGGATGGATTCCTCCTGGCACTTTCCACAGCAGATGGATGTACCATTTTCTGGGCTCTATGTGCTCATTTATACGGCATCCAGCTGATGTTATAAAGACACGGATGCATTCGTCAAACGGTGGCAAAGATCTATCACCATGAAGGCATGGGTGGCTTTGCGAAGGGCATCATTCCGCGTGTCGTCAAACGAACGCTCTCTACTGCAGTCACGTGGACGATATACGAGCTACTCATTTGATCCATCAGGTCTGGGTCACGCCAAGGAAGCGTTTAGCTTGGCTAAGGAGACAGAGCACTCTAAACAGATGGAAAGCCAGGCCAAAATTAAGGAGAATGAGGCGTTATACAAACAGTACGAGATCGTGCGCGTGCAGAAGGAGGGCGAGGAACGCCGAAAGACGCTTGAAGAGGAGACGCGCCAGCACCAGCAGCGTGCACAGTACCAAGACCAACTGAAGCGGAAGCAGTATGCTGATCAGCTAGCTGCTCAGAAATATATGAAAGAGCAAGAGCTACAAAAGCAGGAGGAGATTCTTGCCCGTCAGGAAGCTTCGCGTCGTAAAACGCTTGACTATGAGGCCAAGCTACGTCAGAAGACAGAGCTCGCCAAAGTGGCGGCCGAAACCGAGGGGCGCATTAAACAGGAGCGTCTGAACCACGATTTGCACCTGGAAGAAGCCCGTGTGCGCGCCAAGGAGTACCGTGAGACGATCATGGAAGGTATTAAGCTTGCGGGGAACACATTTGGCTCCGGTATCATGGCATTTGTGGACGATAAAGAAAAACTTACGACTACTGTTGTCTCCTTGACGGCCCTGGCTGCTGGTATATATACCGCTAAGGTTTCCACTAGCGTTGCGGGCAAGTACATTGAGGCTCGAATGAGCAAGCCGTCACTTGTTCGAGAGACTTCACGCCGCTCGGCTGCACAGGTCGTGGCAAACCCTATTCCGTCCATTAAGCGTGCTTTGCGTCTACAGAAGGCGACGGATGCGTTGAAAGGTGTCGTGCTTGAGCCTAAGCTGGACGAGCGTCTGCGTAGCATCGCTGTGTCTACATATAACACGAAAAAGAACCGTGCACCGTTCCGTCACTTGCTTCTGCATGGCCCACCCGGTACGGGCAAGACGCTGTTCGCCAAGGCACTGGCACGTCACTCTGGCTTGGAGTACGCCATCCTCACGGGTGGCGATGTTGCTCCATTGGGCCGCGAGGGCGTGACGGAGATCCACAAGTTGTTCGACTGGGCTTCGCACAGCCGTCGCGGTCTGCTGTTGTTCGTTGATGAAGCTGATGCATTCTTGCAGAAGCGTAGTAACACCGTCATGAGTGAGGATATGCGCAACGCATTAAATGCCTTTCTGTACCGTACAGGTGAGGCCAGCGATAAGTTCATGATTGTATTCGCCAGTAACCAGCCGGAGCAGTTTGACTGGGCTATTAACGATCGTATTGATGAGATGGTGGAGTTTCGCCTGCCTGGATTCAATGAGCGTGTACGTATGCTAAAGCAGTACTTTAACGATTACATCCGCGCTCCAAAGAACTCGCGTGCCAAGAAGATTTATGTGAAGGCGTTATATTTACGGTAGACCCCATTGCAAACTTTCTTGTGCCATGACACGCATCTTTCGTTTGGAAGCACTGGAGGGCGTGTCTGGTGAAGCTCAGGCGATTTTTGATGCTTTGAACAAGAACCTATGCGCATGTATTGGATTGTCTTTACGGTTGTACTAGTCAATGGCTTGTACACGTACTTTGGTGGTAAGGATGAATCGTTTCTAGTGGAGAAGCTCAAGAAAAAGATCCCGTCTGAGTACCGCATCTCGGAGAAATGGGACAAGTGTCTGGAGAACTTTGCACTGTACTTTACCACTGGTCTCGTGGCTGGTGGTCTCACGTCTGTGGTTCTAGCACGTTCTGGTGCTGGTCGTGGACTGATTACGGGTCTAGGTGAATGGGCGCCGGTTCGAGCTGGACGACTTGTCAAATGGTATTTGCAGGTGATGCACAGGCAGCTCTGAAAAAGACGAAAAAGGCAGTAAACGACTTGCCGGCTTTTAAGCACCAGACAATCGTGGATTTTGTAACAGCCTCAATCACGCAGCAAAAGCTATTATCAATACATTCGTGGGTTCTGTGAGCAAATTCACGTTCAAATCTTAGAAACGTATTTGACCGAGCAGGTGCAAAATTCGTTTGTAGACATTGACGCTAATTTAGCGCTACTCAAGTTGTATCAGTTGTATCCATCGACAATTAAAGCCGAGAGTTAACGTTCTAGTTAAGGGTATCATGACGCTTCCATCTACATTCTTTACTGGTGACTACGATGATCCCTGAGAGCACAAGCGAGGATGTTAATGTAACGGCGGCGCTGCACACGGGTTTCATGCTCCAGTCGTGTCTGTTTGAGGATTTCTGGAAACAGGATGTGACGTTTGCGGACAACGTGCAGGGCTTTTTGGAATCGGTCCGCGCTTATATTTTGAAAGCTATACGCTGTAGCCACAGTGTAATCTCTACTGATGTCTTTAAAGCCAAGCTTAATGTATCGGACGAGGAAGTAGCGGAGATTGTTGCCGAGAAGTGGACTTGAATAATTTGATTCAGATTAATCCTAACAAGGACAATCAGATGCAGGCCAAGAAGGTTCAGGAGAACATCGAGTTTGAGGACGTGCTCAAGGTCATTCACACCCTTTCTAGATCCAATGTCTGTGCCAGTGGTGGTGGCGTCGCAGATGCAGAGGAAGTCTGTGAAAGTCTGGCCAAACACTTGAGACTGGATGGAGGCACTCAGATATTGCCTTGTTCCACGGGCGTCATTGGATGGCGCATTCCTGTAGACGCCATGGTGGAAGAACTTCCCAAGCTCGTCAGTAATTTACAGTCCGACTCGGTACTACCAGCTGCTGAGGGGATCATGACGACTGATCTGTACCCAAAGGTGCGCTCTATCGATGTGTGTGGAGGTAGAATCGTCGGTATTGCCAAAGGAGCCGGCATGATTGAACCCAACATGGCTACTATGCTGTCTTATATTCTTACTGATCTTTCAATTCCGCGTGATGTGTTACGTCAACTGCTGACGGAGGTAGTTGAAAACACATACAACTCACTCAGTGTAGACACGGACCAGAGCACGTCGGACACGGTGACGCTTGTGTCGTCAGACCAGATCCCTTTCGACATCAA---GCAGTTGGACGAGTTCAAAAAAGCGCTGCATGATGTCTGCCTTGGCTTAAGCGAAGACGTTGTGCGCAATGGTGAAGGTGCGCATCACGTTATGCGGGTGACGGTGAGTGGTGCTTTATCGAAAGAGATGGCCAAAGGCGTTGGCAAGTCCGTGGTGAACTCGCCTCTCCTCAAGTGTGCAGTGGCTGGCAACGACCCGAACGTAGGTCGTCTTGTCATGGCCGTGGGGAAGTATATGGGCTTGCATCACAAGGATGTGAGCATTCAGCGCCGCATGAAGATCCGAATGGGTGGCGTGGTCATTTTTGAGAACGGCGAGATGGTGCTGAACGATGATATCGAGGCCAAACTTGTTGCTCACATGCGTAGCGCAATGCTGATCGAGCCCACGCGCGGTGGACTTGACGCCAGCTCGCACAACTACCCGCCGCACGGAAAGTTTGTCGAGATCGAGATTGATTTGGGTGTTGGATCCGAGAAGACTCAGGTGCTGGGCATCGACCTTACCCACGAGTACGTGGCCATCAACGCCGACTATCGCTCGTAATGGCACC---GAAGAAAGGAAAGACTGCTGCTGCTTACTTGGCGTCTCTTGGTGTCGCCCCAGT---CGCTGATGGAGAGAAAAAAGACGAT---AAGAAGAAGAAGAAAAAAGGAAAAAAGAAACTTAAAAGAAGCCCATGTCTGCTGCGGCCAAGGCACTGA-TGGAGCGACAGGCAGCTTTAAGGGCTGAGCAGGAGCGCCTAGAGCAAGAACGTTTAGAGCAAGAGCGTATTGCTCAGGAGGAGGAAGCCAAACGGTTGGCAGAAGTTGAGGCGGAGGAGGAGATTCGCCGCAAGAAGAAGGAGGCGCGCGCCGCCAAGATCGAACGGCAAAAGAAGGAAGGCACGTACATGACTAAAGCCCAGAAGCAAAAGGCGGCAAAAGCTCAGGCAGCGTTGGAGGCCATGCATCAGGCAGGTCTCGTGCCGGAATCTGCT------GGTGAGAAAAAGACAGTCGTGTACTCGGACAACAAGAAGAAGAACAATAATAATAGGAGGAAAAGGAAGTGGCGGCTGAAGCTCCAGACGCGTGGGATGATGAGGATTGGGAGACGAGCGATGTTGTGGCCTCGCTTGAAACTAAATTAGCAGAAGAAGGTGCAAGAGCGTCTGCGTCAGCAAGGAATTGCACTGAAGAAGCGTCAGGAAGAGCAGGCCGAGCAGATGCGACTGGAGGCTGAAGAGCTGCATCAGCGCTCAAAAGCTAAACTCGAGGCTGATCGCAAAAAGAAAGAGGCTGCTGATCGTCGTCGCGAGCGTGAGGCCGCCGCACGAGCCAATGTGTCGTTGGACAACTTGCGCTCACCTATTTGCTGTATCATGGGTCACGTCGATACCGGAAAAACGAAACTGCTCGACAATATACGCAAAACCAAGGTGCAGGACGCTGAAGCCGGTGGAATAACCCAGCAGATTGGTGCAACCTTCTTCCCCGTAGAAGCTATAAAACAAAAGACAGCTCGTCTCAATGAGACAATGAAGCTGGAGTTCCGCCTGCCTGGCCTGCTTGTGATCGACACACCTGGTCACGAGTCGTTCACAAATCTTCGTAATCGTGGTTCATCTCTGTGTGATATCGCGATTTTAGTGGTTGACATTATGCACGGTTTGGAGCCGCAGACCCTGGAGTCACTCCGCTTGCTGCGCCAAAAAAAGGCACCATTTGTCGTCGCACTTAACAAAATTGATCGTTGCTATGGCTGGAAGACAACGCCCGACATGCCCGTTCAGGAGGCACTCAAGCTCCAGAATGAGCACGTCATTCGCGAGTTTGAGGACCGTATGAAAGCAATTATTGTGGAATTTGCCGAGCAGAAGTTAAACGCCGAGGTTTACTGGCGCAATAAAGATTTGGCGCACACGGTCTCGCTCATCCCAACCAGTGCTATCTCCGGCGAGGGTGTGTCTGATCTGCTGATGATGCTGACTCGATTAACACAGGAGCGTATGGCGAAGTCATTGGCGTTTGTCGATATTTTGCAATGTACTGTGCTTGAAGTAAAGGTGATAGAAGGTCTCGGCACAACGATTGATACGATTTTAGTGAATGGTACACTAGAAGAAGGGGCTACTATCGTTGTGTGTACATTGGATGGGCCTGTTGTAACAACCATCCGCTCGTTGCTGACTCCGCACCCTATGAAAGAAATTCGTGTTAAGGGTGAGTACATCCACCATCAAAAGATGCGAGCTGCGATGGGTGTGAAGATTTGCGCACAGGGTTTGGAGAAAGCTGTTGCTGGCACACAGATCTACGTGGTTGGCCCTGATGACGATGTCGAGGAGCTGAAGGATTTGGTTATGTCCGACCTTACTGGTATTTTGGACTCAGTGAAGGCTACCCGCCGCGGTGTGATGGTTCAGGCTTCTACTCTTGGTGCTTTGGAGGCACTTCTGGAGTTCTTGCGAACGTGTGACCCGCCCATTCCTGTATCTTCTGTCAACATCGGTCCCGTGCACAAGAAGGATGTTATGCGAGCTTCTGTTCAGCTCGAGCACCAGCCCGAATTTGCTACAATTCTAGCGTTTGACGTGAAGGTCCACAACGACGCAACTGAGCTGGCATCTGAGCTTGGCGTGCGCATTTTCACGGCTGACATTATATATCACCTGTTTGATCAGTTTACGGCATACATGGACAACTTCCGTAGTGCTCGTCGCGAGGAATTTGAGGAGGTTGCTGTGTTTCCTTGCGTGCTCAAGATCCTCCCTAACTGCGTCTTTAACAAGAAGGACCCGATTATTCTTGGTGTCGACATTGAAGAGGGTATTCTCAAGGTTGGTACGCCTCTTGTGGTGCCGTCCGCTGGCG---GCTTTTTGGTCGGTAAGGTGGGATCTATCGAACGTGAGCACAAGGAAGTGGACCGCGCTAAAAAGGGTGCATCGGTGGCCGTTCGAATTGACAATGAAGGTAGCGTCATGTATGGCCGTCACTTTGACCACAAAAACAAGTTGGTGTCACGCTTGACCCGTCAGAGTATTGACGCGCTGAAAGAAAATTTTCGAGAAGATCTTCAGAAGGAGGACTGGCAGCTTGTCATCAAGCTCAAAGCAGTGTTTGATATCATTATGGAAGGCTGGCTTACAAAACGTCGTGATCATATGAATCTTATCTGGCGAGACCGTTATTTTCTCTTGGATTATAATCAGCTTCGTTATTATCGTAAACGAGGTGATCCAGCACCTCGAGGGACTTATATTTTAACCGACGGATGCGTTGTAAGCTCTGTGTTTAATTCCGAGGAGAAACATAAACATCATGGTCCAGTCTGGATGTTTCGGATCACATGAAAAATCCTATAAAAAGGACCGATTTCTGGATTTAGGTGCGAAAAGTGAAGAAAATGCAAAGGAATGGAAACGAGCGCTTGAAAATGCCGTTCGTTCATTAAGAGAACTCATGGCACATCAGCGGAAACTCAAGACAGATGCTGAAAT---ATTTCCCACACCTACACAC---------GTACCACGAATGCTCAAGGCCGATGAAGATACAGTCTCACTATTTAATCTAGATGCTGATAATGTCAAGGCAGAACAAGCTTGGTGGTTGGCTCGAGTTGAAGGAGGATTACGGATTATGCAAGAATGTCCATTGGGACCAAATGTTGCAGCGAATTTGTACGCGCAACACGCAGCGGCGAAGAATGGGCTTATCTTTAGTGCTGTGATTGGTGCTGCACTGAGCTTTAGTATCATGTACTCGTTTGCTAATAATATGCTACTGAGTTTCATGACGGCATTGGCGGCTGTTATAGTGTGTTGTGTTGCTGTGACACCTACGCAAGCATTAGAGATTCCGAGCTTTCGTGTCTCGCAAGTGGTGCATGGATCGCCGACAGAGGTCTTTAGGCTGATCATGAACTCGAAGAGATTTCAACGATGGGACCCAGCGACGGCGACCATGCGTGTTGTACAGCAGCTGGATGACCACGCCGACATTGTGTACGCGACGCAGCGACCGACGCACTTGTGGCCTCTGTGGCAGAAAGCTCGTGATCTGGTGTTCATGCGGTATTGGCGACGCGAAGAAGACGGGTCTTACTTTGTTATGTACCAGAGCGTCGACCATCCGGAGTGCCGGATGCGTCACAACTACGTGCGAGCGAACATATTGGGTGGCGGTTTCATAATTGCTCCCCAGCGCGTTCCAAGT---GGATCCATTCGCACACTGGTTACATACGTGCTTCGATACGATCCTGGTGGTTG---GTCGCGTATCTACCACCAGCTTGGCATGGATGTGGACTTGGTCATGCCTCAGCTGCGCAGTGTTGTGGGCATTCGCGACGAAATGAGCGCCACTGACTTTATCACACCTAATGTCTCGATGGCGCCTGCTGGAGAAGGCAGAAGCTGGTCAGAAAGCGTGGA---CTCCATGGGTGTTGTCATGGGTGTGCAGAAACTTCAG---ACCAGTCTGCCGGAGAAGATGTGGGCCGAGCCCGACGCCTCCCTTTTCAGTGTGCGCGGCCATAACTACTTGAACGACAAGAAAAAGATTCCGTCAGCGCCAGCAATGTTCCACACCGTGGGTGTGGATCTGTTGTCGTTCGAGAGTGCAGCCGAGAGGTACAACATTTCGTCTCGTGCAGATAGTGTTGGTCGACAAAG---CTCCAAGTTCACGTTTGTTGTGAATATGATCATTCCGAGTCCGGAAAACACGTGCATGGTGTTCTACTTTCATCCTGTGCGTGACAGCGTGTTTGAGGACGGCTCACCCTTTTCCGAGCTGCTGAACGACTTTTTTGATGGCGACGACCAGTTCCGAAATTCGCGCTTCAAGCTCATTCCTACTGTCGTCGAGGGTAGCTTCATTATCAAGCAGTCGGTCGGATCCAAGCCAACACTTTTGGGCAACAAACTGAAGTGCCTTTACCATCGTGGAGACAACTACTTTGAGGTTGACATTGACATTTCGTCAAACTCGGTAGCCAACACAGTCGTGGGCATGGTGCAAGGTGTTACCAAATCGCTAGTGGTAGACATGGCATTCTTGCTCGAGGCCCAATCAGACGAAGAGCTGCCTGAAAATATCCTTGGCGCAGTGCGCCTGCAACACATTTCTCTTGAAAACCCTCGTCGTATGTCAAAGCATGGGAAACGAGCAGGAAGGATGAAATTCCATTGCGAGGCCTTTGCTCAAGCTTTGAGGGTTAATTTACAGCGTTTGGTCGCATACGCGTGCTCGGCGGAAACGTCGATACAACGAGAAATTGCTGAGAAATTGGCAAATGAAGCTGTAAAGATCGACAGGTGCAGATTGTGGAGTTGGATGGATTGAAGCTGTTACTTCCATTGACCAAGTCAAAGGACACAGAAGTGCAGCGTCTAGCAGCGCATGCTCTTGCCAACCTTTCTGTGAATTCTGAGAATCAGTCGAAAATGGCGACGGAAGGAGGCATTGACATGCTTATTGACCTCTTGGGTAGCTCGAACGAGCACGTGCAGCGTCAGGCTGCAAAGGCGCTTGCCAATCTCGGTGTCAATGTGGATAACAAGGAACGAATCGCTAAGGCGGGAGGCATCAAGCCACTTATCGATTTGGCTAGCTCCTGCCAGATTGGAGTTGCTGTCGAGGCTATTGCAGCACTTGCAAATCTAGCTGTCAATG---ACGCCAATGAGGTAGAGATTGCACGTAAAGGAGGTTTGAAGCCTATTATTGATGGAGCGCATTTGGAGTCTGTTGAGCTTCAGTCCCAAGTGGCACGTGCACTTCGCAACCTGTCTGTCAACCCTGAAAACAAACAAGCGATTGTGGATTTAGGTGGGGTAGAGGCTCTGCAGTTACTCGTTCGTTCTCCTAACGACCGGATATGCCAGCAAGCTACGCGTGCACTCGTTATTACGACATTCCTCACGCGCTCATCACCTACAATCTATTG---GTTGCACCACCTGTGACGCGTGTAGCTGATGTGAAGGAAGAAGC------TGTACCGTATGACAATGCGCTTGTTGATGTGGAGTATTCAATGACAGTCAAGACAGTT---------GTTGTACCTACTTCGATGCCAT---ACGATGCAACGGGA---AGTTTTTTTTCCATATCCGATCGCTTTCCGCTTTCCAAGGTGGGAAAAGTTAGTTGTAGTATGGCGTCTTTTCCCATGAGCCG---TTTACTGAATG---GTACACCCGAGATGACGC---AGGTAGATCAG-------------ATGACAGTAATTGCAGAAGACGTGACACTAAACGACAATAAAAACATTATAGCTGAA---CCGCAAGTTGGGGGATATATACCGATACCAGGCTACTGA--TGTGGAAGAAAATGAAATGGAGCAACTTCGATGGGATATTCAAGAACAAGTGTTTCAGCGATATTTTCAGCAAGGACAGAAGTACGAAGAGTGGGGGAAATTGGAAGAGCATGGCACCGAGTCTTTTTCGTTCCAGGTGGAAGAAGGGAATACATAATGGCCGAGAAAAGGTTTCACGACAATGCCGTTCGTGACGCCATGGTGAAGGGAAATGCCACGTGGGTCAAGGAGCGACGAGCTCGGTTTGAGGAGGAGCTTCGTCAATTTAGCGATGAAACACGGCACAAACAGGAGTTAATGCTTACACGTCTTGAAAAAGTGTCGCCGTTCTCTAAATTTCCAATTCTTGGCAACCGTTTTTTGCTGCTTCGATTACGAGGAAAAGGAGGCAACGGAGAAGTATGGGATGTTGTCGATTATGCCAATAATAAACAGGATTGCGCTCTTAAGCTGTCTACTTCGATTCGCCACGCTCAACGTGAACACAATACGCACACTCAACCACCCCA--ACATCGTCCAGGTCGGCAAAGTGCCGTTTCTAATTCGATACCAGCAGCAACAGTACACAGCGTTCACTGTTGCACGTGTTCAATCAGACTTGCAGCAAATCATTGAGA---TGTACGAGTACTTGGACGATGATTCTGCATACAAGGTGCTTTCACAGCTTCTGAGCTCACTGAAGTATTTGCACGATGGGCGTTGCTCACTACGACTTGAAGCCGTCGAACATATTAATCGACTACGACGGGTGTGTGAAGCTG------ACTGACTTCGACTTGACGCGCGATGCCAAGACAGCAACGTCGAGCTTTACGGTTGGCACTCTGCGGTACCTCCCTCCCGAGTGCTTTCGACCAAATCGAGCCGA---------TTGTGAAGCGACCGCTGAGAAGGCAGACATGTGGATGGTAGGGATTGTTTACTGCATGATGGTCACTGGAAAACACCCTATCTGCCCTGACAAGTCAACGAAAGCGGAAGTGAAGTCATTGATGAGTCAGTACACTGGT---CAGCTTCGATACGCGCGGCCAATTTCCGATCCATCCCGGCGGATCTCACAATGGTGCCTTCACCCGGATCCAAGGTGTCGACCAACGGCCGCGCAATTGCTAGTGGCACTCCAC

Peronospora_destructor TGGATTACCACCGTGAAGTTGCCCGTGAGCTTGCCGCTTTCTTTCACTCCTTCTATCGCTTTGGTTCTGCCTCGAACCGCTTTGATCTGTTCCTGCACAACGTTCTACTGGGCTCGCTCTGCATTGGCGCCGTCGTCTTTTTTGTGCTCGCCGTTATTGTCGTACGACGTACTGTGTTGCACATCCGTAGGCTTATGTACGCCGCACATCGACGGACTCTACGTCCGTGGAACTGCTAGCAGTGGGTCTCTTGACATTTCTGGCAATCAGTGCCCTTGGGGGATTGCTAGGAGAAGCTGTCTGCAGATAAGTGACGCCTTGCGTATCAATGCTGATAATCTGGTCACGTCATTCAATGAGAGTGAGCTACCAGCTGATGCGTTTAAACTATCGATTGAAGCACTGGACTGGTGCACACGTCGAAGGATCTATGGAACCAGACGGATGCACTGCTGCCCAACACCTTCTTAGATGTAGCCAAGGACTGGGAGTTCAGCTATTTTGTCTTAAAGTCATCGACAAATGGAGCAATTCTTGCAGTGACGCTTGCCAGCTTCTTGTCCATCGCGTCGGTTGGGTGGGCGATGGTCTCGCCTCTTCGTGTGTCCATTCTCGTGATCTTGAGTGTCGTGCCAATTTCGCACACACTTATTGGGGCTTATCTGTCGTCGACAATTATGACGGCGGACTTTTGCGCTGCACCCATGAGCAACACGCTCAATCTCGTGGGCCCATGCCCGGCAAACGCGTCCTGCCTTTTGCTGACGCTGTGGCAACGCAATGCGTCCGGTCAAGGAGATGCTGGTCGTCGCATGAAGAAGGAGTTCTTAGACCCCATTGGAGCAATTGTGGGTGTCATGCTTGGCTATTTTGCAGGCACAGTGAGCAATTGGGAGAGCTCGACGCTTATTCAGACGTGGCGTTGGCCTTTTCTACTGCAATTTGTAGCTCTTCTGCCTATTAATGTCGCTATCTTTTTCGTACCGACGAAACATCTCGTGATTCGAAGTGAGATCAGTGGGATACAACCGCATGTGGCCGGCACACCCATGAGTGCGAGGTCGACGACGTATCATGACGAAGAAGACGCACTGCTTGCTAGCGCGAGCATGCAACATACGGCGCCTTAGACAGGAGGTGTACAGCCAAGGCGCGTTCTCGGACGGCGTGTATGAGCTGTTGCACATGCCCGTGTTCTGTCTGATCGTGTTTGGTCTCACGACAGTGTATTTCGTTGTCACGGGCGTGCAGTACTGGAGCACCATCTATATGATTAAGTCGCTGCATGCATCCAAGTATCTCGTCAACGCGCTTTTCGTCGTCGTCTCTGGCACAGGACCTATCCTGGGCGTATTCTTTGGTGGATGGCTGATTGACCGATCTGGAGGATATATCGGCGTGGAGCAGCGTGCGAGGGCACTCGGTATTTGCATGAGTCTGGGTCTGACAGCCTTTTTTATTTCGGTGGTGGCGACGTTCTTCAACAACGTCTTCATTACCTCCGGCTTCTTATGGTTGTTGCTTTTCTTCGGCGGTGCAATCCTACCAGCACACCGTGCACTCGCTTCTTCATTTTCCGTCATGGTGTTCAACCTGTTTGGTTACTCGCTCTCACATGAGCGACTTGCAGATGGTCGTAGGTGCCAAGTGCTTTGTGCCAGACGAGACACACGTGTGGCTCGCAGCTGAAGTCGTACGTGACGAGAACTCGGGCGATGGCAAGACACGCAAGATTTACTGCAAAGTGGAGCTGCCTGATGGAAAAGGAGGAGCGCTGTGTGGACATGTTGTCGAAGAAAACCAAGACCCTCATGCTGTGCATCAACTGGAGACGCTGCCGTATCAGAATGAGAACGTTGGGCCCGAAGGCATTGAAGACATGATTACACTCAACTACTTGCATGAAGCTGCTATTCTCTTCAACATTAAAAAGAGGTTCCTCTGTGAACTACCGTATACGTATACAGGGGACATTTGTATTGCCATTAACCCTTACAAATGGCTACCTGATCTCTACGCAGAGGAGCAACATCTACGGTATCTTAACCAGCCTAAGGAGGAACTTCCTCCACACGTGTACGCCACATCGGTTTCTGCCTATGACCATATGCGACGGTCGAAACTTAACCAGTCTATTCTAGTCAGTGGGGAGTCTGGCGCGGGTAAGACAGAGACCACTAAGATCCTTATGAATCACTTGGCTACTATTGCTGGTGGTATGAACAATTCGACTATTAAAAGGATTATTGAAGTGAATCCACTTCTTGAATCCTTTGGTAATGCCAAGACAGTACGAAACGACAACTCGAGTCGATTTGGAAAATTTACACAGCTGCAGTTCGATAGGAAGGGTACACTTGTGGGTGCTAAATGCCGCACGTACCTGCTAGAGAAGACCCGTGTTATCCAACACGAAGAGCCGGAGCGCAATTACCACATCTTCTACCAGTGGACTCTCCCGACATTGCATCGGAGCTACAGCTTGACTCGACCAAACATTACGTGTACACGGGTGACAACACGGCACAAAAGATCGAGGGACTCTCTGATGAGAAACACTTTAACAAGACACGGGAGGCACTTGAGCTTGTGGGGCTGTCGCGTGGTGACCAGCAGCCGCTCTTTGAAGTGCTAGCGGGTGTGCTTCACCTTGGTGAAGTGCAGCTGCAATCCGACGCATCAGATGATGAGAAATCTCTCATCACGGTGGGTGACCGTGGTGCGAATGCTGCTACTGAGATGTTTGGTGTGACATGGGAAGCGCTCGAAACTGCGCTGTGTTCGCGTACCATGCGTGCCGTGAACGATGTGTACAGCGTGCCGCTGAAGAAGGAGCTGGCTATGGACTGCCGTGATGCACTTGCCAAGGCGGTTTACTCGAACGTGTTCGACTGGCTTGTGGCCACGATTAACCAGTCTCTCGCTGATGATTCCAATATGCGGAACCACGTGGGTGTGCTCGATATCTTCGGTTTTGAACACTTCAAGCATAACTCGTTCGAGCAGTTCTGTATCAACTACGCCAATGAGAAGCTTCAGCAGAAATTCACTCAGGATGTGTTTAAGACTGTCCAGATTGAGTACGAGGAAGAAGGTATCGTATGGGATCACATTGAGTATGCGGATAATCAGGATGTGCTGAGCATTGTTGAGTCGCGCATGGGCATCATTTCACTGTTAAACGAAGAGGTGATGCGACCCAAGGGTTGCGAAGAGTCCTTCATGTCCAAGTTGACGTCCCTGCACAAGGAT---GACATGTCACACGTTATTGAGTTCCCTCGCACATCACGTACCGAGTTCCTGATCAAGCACTATGCTGCTCCTGTTATGTATGATTCGGTGGGATTCTTGGAGAAGCACAAGGATTCGTTGTTGCCTGACCTGTCCGAGCTCATGCGCGGTTCCTCCAAGCCTTTTATCGCAAAGCTCTTCGAACCAAAGCCGGAACCTAAGCCGCCGAAAGC---------GTGGTGGTGCTTTGTCTATCACGACTGTCGGTACTCAATTTAAGGAGAGTCTGACAGAGCTGATGGCGACCATTAACTCCACCCGAGTCCACTACGTGCGGTGCATCAAGCCTAATCCGATTAA---------GAGCTCGACGGTTATGGATCAGTCTATGGTGGTGTCTCAATTGCGGTGTGCTGGTGTGATTGAAGCCATTCGTATCTCACGTGCTGCGTACCCAAATAGGTTGCAGCACATCGAAATTCTGGATAAGTTCTGGCTGTTTGTACCGAGCGGTGGTAACACTGCGGTAGAAAAGTGCCAACTACTCATGGACAAACTGAAACTAGAGTCCCCAGCGCAGTACCAGATGGGTAAGACCCGCGTATACTTTCAATTGGGTGTGCTCGAAGAGCTCGAAGATCGTCGTAAAAAATTTCTGGATGCCAAAGCCACATACCTGCAGAATATCATGGTTGGTTTCACGCAGCGTATCAAGTACCTTCGACAACTGGAAGCCATCATCAAGTTCAGTTATCAGGTGTGTGATTGCTATGCGGAGATACAACACGTTCGTGAACGGCCTCGTTTTGGCGCAAGCTCGCTGGCGAGGCATTCAGGGCCGGAAGATTGCAGTGGTTGTCAAGAACAACCACTACGCTGTCATCATCCAGCGGTATGTGCGCGGCTATGTCAAGCGTCACTCTTACGGTAAAATGCGGGCAGCGGTTGTCCGTGTACAAGCCATAGTTCGTATGACGATCCAGCGTCCAAAGTATCTGGCTGCTCTCGAAGAAAAACGTCATGAGGCTGACATGGCTTACCAGCTGAACAAGTTGAAGGCCGCTCTGCAGGAAGAGCAGGACAGGAATGCCCAGCT------GCAGCGCC---GGAGTAGCTTAAC--GACGGCACGTCTGCTGCCTCGAGCGTGGTGATGGCTGATGCTGGTGGCATGATCGAAACTCTCCAAGATGAGAACAGAAAGTTGCGTGAGAAGAACGAGGGAATAAAGATCGCGATGAAGGAGTTGAAGGCGGAGATCGAGAAGTTCAAAAGTGACAAGGAATTTTCATCGGCTGGCAACCACGTCAAGGTTCGTCAGCTGCAGGACACTGTGCGTGACAAAGAGAAGAAGATTAAGCTGCTCGAGATCGACAACAAGAAGCTGGCGGAGCAGCTTGCAAAGCTTCAAGTGGACGG---TGC---ACCCGAGAGGAAGACCACGCCGAAGAAGTCAATCTTCCGCACGCTAGGGTCCAAGAAGGAGAAGCAACCGC---------------GTGAGACCGTGCTGATGGACTCGCTTACTGGCTCGGATGACCTTAACCATCGGGATACTCGTGGGCCGGA---GCGTCAATTTCCGCGTATGC---CGAGCATGAGCGTGGGCTCC---CGCTTCTGGAACTCTAACAAG---AAGGGCGAT------------AGCGGCGATGGTTTCGATGTCTCGGAGGTTGAAGGTGAGGATAGTCCTACGAACCCACGCGCAACACGATCGTGAAGAGCATGGAGGTTGGAGTGACGGGTGCCATGTCAAGTTTGAAGGGTCGAATG---------TCGGTCGTGAAAGGCATGTACGAAACAAACGCTCGAACAGGATCGCCCTGTGCGCGAATCGTTCAACCTGGATGCGTTACCGGAAGTGTCCTTGCCTGTCGGATGGGAGGCCAAGGTGTCCCGCTCTACGGGACGCGTGTACTATGTGAATCGCAAGCTTGGCAAGTCTCAGTTCGAGCGTCCCACACTAGCCTTACTCAA------GGCACAAAAGATGGCGCGTCAGAAGTCGGCTAACACGGAATGAGGCAGTTGTGGCAGATTTCGGCTGTGGAGATGCAAGGTTGTCAGAAAGTGTGCCGAACAAGGTGCACTCGTTCGATTTAGTATCCTTCGACATTGGCGTGTACTGTTTGGCGCTTATGGGGACAAGTGTACGTGAGTATGTACGTGAAGTACATCGTGTGCTCAAGCCTGGAGGCGTGTTGATGGTTGCCGAGGTCAAGAGCCGCTTTGAAAATAAGACGCTGAGTGGATGGTGGCAGTCTTTGTCGCGACGGCGCATAACGTCGTGGTGACAGTGCGCAACATAATAGTGTTTGTGGAGAGACAAAGTACGCAGTTATTGTAGGACAAAAGACGGGAGAGGCTGTAGCTGTGCCTTTCCCGGATGTAGCCCGTGACCTCAAGACACTGTTGGGCCACACCACGTCCATGGTCACCGACGTGGCTGTCAATTTCGTCACTGCTTCTGACAGCTGATCGTGATGAGAAGCTACGTGTATCTCGATTTCCAAATGCCACGATCATTGAAAGCTACTGCTTGGGCCATGCTGCCAGTCTGACCAAACTGGCTTGCAGTTCTGTCACGCCAGAACTTGTCGTTACGACTTCCATGGACAATACGCTGAAGCTTTGGAATATGATGACGGGGAAGCTGGCGATTTGTCTCAAGACGACATTGTGGCGGTGCTCGTAAACTACCAGTTCGTGCGATTTTACGAGATCGTGAGAACCCGTTCCTGCAACTGTTTTTCATTTCAAAGGATGACTGACGTGTTCAACTGCTGTGCGTGACTTTCGCAAGCTTCAGAATGACCCGCCATCGGGTGTCTCGGGTGCCCCCATGGACAATAATATCATGCTCTGGCAGGCAGTCATTTTTGGACCAGATGACACGCCCTGGGAAGGCGGCACTTTTAATCTCACGCTCGAGTTTAGTGAAGATTATCCAAACAAGGCACCGACTGTCAAGTTTATCACCAAAATGTTCCACCCAAACATCTATAACGATGGGCAGATCTGCTTGGACATTTTGCAGAACCAATGGTCGCCGATTTACGACATTTCCGCCATCCTCACGTCCATTCAGTCGTTACTATGCGATCCGAACCCGAACTCGCCCGCCAACTCGGAGGCCGCACGCCTTTTTCAAGAAAATCGACGAGAATACAACCGGCGTGTGCGTGAGATTGTCGAGCAGAGCTGGCAAGCCATTGATGGTATCGGCTGCCAAGATGCGTGGTGCGGAGAACCGCATGAATGTCGGTCGCCCCTTTACAGCTTGGCTGGATAACATTTCTTCGGACCGCGGTCTTTGTGGCGGTATTAATTCGGGGATTGCCAAGGCCACCCGCAAGCAGACAACTCGCAGATTTTTGTCATTGGTGACAAGGCGCGTGCCCAATTACGTCGTGACCTTGGTACCCATATCCGCGGCAATGTAGCCGAGACGTTTCAGTCACCACCTAACTTTACAGTAGCTAGCGCTATCGCGGAGGCTGTCGAATCGGAGAGGAGTCGTCGCGTATGGCTGCCATGGAGAACGCCACCACAAACGCCCAGGACCTCACGCTCACGCTCGTGTACAACAAGGCTCGCCAGGCTCGTATTACCACGGAGCTGATTGAAATTATTTCGGGTGCCGCCTCCCTGGACAGTGACTGGTAGCGTCGTGAGCGTGCAAAATGTCGCCCAATTCGATGACGCGACTGCACGCGACTCCACTTTCTCCGTGACGTTCTTCTGGGCTGATTTCCAAGCTTGTCGTCCTAATGGACAATTGGATGTAGGGTCGTAAGGTGGTGGATAAATTAGAGGGTGCTAATGTAGCAGAGTTGGCGAAACGGGTCTAGAATATCGTCTCAAGAAATTAATCAATGCATCGTCAGTTATGCTCTTTATGAAGGGTAATCCAACGGAACCAAAATGTGGCTTTAGTCGGCAGATTGTCACGCTACTGAACGATGAGAAGATTCAATTTGGAACGTTTGATATTCTCAATGATAACGAAGTGCGTCAAGGGTTGAAACAATTGTCGGACTGGCCGACGTTCCCGCAGCTTTACGTGAATGGTAAACTGGTTGGTGGGCTGGATATTGTCAAGGAGATGAAGTCGGAAGGATCAATTGTTGAGCAATTAGGCCTGGAAGAAGCGGAAGCGACGTTTCAAGAAAGTCTGTGTGCATTGATAAAAGCAGCACCTGTGCTGCTTTTTATGAAGGTTTGATATTTTGTATGGAGCTATCAACGCCAGGACCAAGATATCGCTAAGGTAGTGACTTTGATGCCATCATCGTTTCGCGAGGGTATGTGCGGAAGCCATCTCATGGAACCCGAAACCGGATGGCCATGGCATTTCGTGACCCTGTGAACGAAAAGGGAGAGTTCTGCATTGTGCAGCTTCCCGTATCACCTCGAAGTGCACCACTTTACATCCAAGAGGTATTTCTACAATTGACTGGCAAGTGGAGCGTGCTGCACCGCGACCACCGTAGTGAAGTTAAATCGCTTTGCGTTCATTCCAAGCGTGATTGCTTGTACACTGGTGGTCTTGATGGCCTGTTGGTCCGGTACGATTTGAACAAGGGACGTACGGAAACAGTGTTGGAACGTCGCAAACACATATCTAAGATCAACTCGGTCTTAGAGCATCCACACAACCCGAATCTGCTTTTGGTCAGTTGTGTGGAGCAGTAGGCACAGCTTGTTGCTGCATGACCTCCAGCGATATAATGACAATGTGTCAAGTGGCGCGACGAGTCAGTACGTCGTGCCGCGGTGGTCGCCTGCAGGCTATCACGTATCATGTGGTAGCAAATGCGTTACTGCCACAACAGCTTCTCGACGCCCACTTCCGCCACTATTGGAGCTTCGTTCCTACAGAAGCGCGTTATCGTCGGAGGGAAACTGACGCTGCAGATCTGGGACACGGCCGGCCAGGAACGTTTCCGCTCTATGGCGCCCATGTATTATCGTAATGCAAAGGCAGCTATTTTGGTCTTTGATTTACAAAGCGAAGCTAGTTTCGAGAAAATCAAAGAGTGGCTGCACCTGCAGCATCATGTGGGTGACGATATGTGGGCAACAAAAGCGACGTTTTGACTTTTCAAAGGCCCAACAGCTCGCCGATGAGATCGGTGCTCTGGTGTTCCGCACGAGCGCCAAGACGGGTGAAGGAGTGCAGTCGCTGTTTGAGAGCCTAGCGGTGCAGCTGCTTCAGAAGCATGACCAGGAGCAGCGGAGGGTTTCTACGTGGTGTATGTACCGGTAGATGATAGTAAAGATCTAGAAGAGTGGGATGTGAAATTACCTGTAGACAAGGAGGCGCAACTTGGCTGTTTAACGGAACGATTACGCGAGCATTTCAAGGGCGGAAACATCCAAGCAACAACAGAGGCTTTCAAGCAGCAACTGCTTGCTCAACTGCCCAAGGGCGCTACACTTACCAATGAAACGCTGCAGACGATGCATGGATAGTCTTGTGGACTCGATCCCGCTCGTGCTCAACATGTCCGACGTTAAGCATGTTGGAAGTTAAGGGTGATGCATTTATTGGACGGGTATTTGATAGATGATTTCGAGCGTATGGACTTCCGATTGAGCGACGTATCAGGTGATGCTCCGTGGGTGCAGACGGCAAAGTTTCAGAGATCACGCAGTCACTAGAGGAGATCCTGGCACTGGGACAAGGTTTAGACGATCAGGATTACAAGGCTGAGCTCGAAGTGCTTCAGAATGATCCAAATTCGAATTTGTATTCCGCCGTCACATTCGAAGAGCTAAATTTACCAGAGAAACTGCTTAAAGGCGTATACAACATGAAGTTCGCGAAACCGTCCAAGATTCAATCGGTAGCACTGCCGCTGATCTTGGCTGATCCGCCCGAGAACCTCATTGGTCAGGCACAAAGTGGCATCCTACTCGTGAGCTCGTGAGACAGATTATGGCGGTAGTAAACGCCATGGGCAAGTTGTGGAGACTTTTCTCGCTATTCCAGGCAACGATTTGGCTCGTGGAGAAAGTTTAAAGGCTCAAGTGATTGTGGGAACCCCTGGAAAAATTGAGGGTATGATCAAGAAGAAGCAACTGGACACTCGCAACGTGAAAATTTTCGTGCTGGATGAGGCAGATGTGATGGTGTCTGAGGACGGTATGCGCGAGCGCTCGGTGGCTATCAAGCGTCTGATCAAGAATCGTGCTTGTCAGTACCTCCTATTTTCGGCAACATACGCTGACGACGTGCGTGACTTTGCCCAGAAGATGGTGCCGGACCACAATATTATTACGGTCAAGAAGGAGAAGCTGACTCTCGATGGCATTAAACAGTTTTGGATCGACTGCAAGACCCGTGACAACAAATTTCAGGTGCTCTCGGACATTTTTGCCGTCCTGACTATCGGCAAGTGCGTTATTTTCGTCCAGCAGCGCGAGACCGCCAAGGAGTTGACGCGTCGTATGCGTGACAAGGGCCACATGGTCGGAATCTTGCACGGTGCTGACATGGCCAAGGAAGTACGCGACCAAGTCATCGACGAGTTTCGTGCTGGCACGACAAACGTGCTGATTACCACTAATGTGCTGGCTCGTGGTATTGATGTCGCGGGTGTGTCGCTTGTCGTGAACTTTGATATCCCGCTTACGCGAGACCACCGCCCGGATCCCGAAACGTACCTGCACCGTATCGGCCGTACGGGTCGTTTTGGCCGCAAGGGCTGCGCTATCAATTTTGTGTACGACAACAAGAGTAAGCAGGACCTTGCAGAGATTGAAGAGTACTACGCGCGGCCTATTACACAGGCTCCCGCCGACGACATTGAGGAACTCGAGAAGTTGCTGGCTTGGGGGGTAGTAAGCGTCGATTGGCCAAGTTTGGAAAAGAGCAGCGCAAGGGCATGCGAATTTATCCACGTGAACCAAAGAAAAAGCCCTCGGGGAAAGACAAGACGTACTATTACATTAAAGACGTCATGTTCTTGGCCCATGAACCCGTCCTCGACAAGTTTCGAGCTTTTAAAACCTTTATGAAAAAGATCAAGAAAGCTATGGGTAAAAAAGACTATGAAGATGCCAAGCGTAAACATGAGAATCGACCTACATACGAGCTCGACCATATTGTCAAGGAACGGTATCCACGCTTTGTTGATGCTCTAGGAGACCTGGATGACGCCCTTTGTGGCAGAACTATCTTGTTACTACTAGAGCACTTACTAAGATTTTTGTGAGCATTAAGGGTATCTACTATCAGGCTAATGTACAGGGTCAAATTGTCACGTGGTTGGTGCCACATCAATTCACTCCGACAGTGGACAAACGAGTGGACGTGAGAGTCATGCTCACGTTCTTGGAGTTTTGTCAAGTGCTGCTGAAGTTTGTCAACGCTCAATTGTACATGCAAATTGGAGTGGCTTATCCTCCCAAAATTAATTTGCAGCTTGATTCAGCTGGCGCTCAACTTGCTGCGATTGAACTAGCAGCAAAAGGAATCAGAAAAGCGTATCAAGCTACAGCGAGCAGCGCAAGGAGCGCAGTGAAAAGAGTCTGTTTAGTGGTCTGACCTTCTTCTTGTCTCGAGAAGTTCCTTCGGCTTGTTTGGAACTGGTGCTGCGCTCGCACGACGCCACTTTGGGCTGGGATGGCGCTGGGTCTCCCTTTGATGAGAAGAATACAGGCATTACGCACCACGTTATGGATCGTCCTCACCAGGGTCACCGTTACTTCAACCGTGAGTATGTGCAGCCGCAGTGGGTGTTCGACTCCGTGAACAATGGCATTCTGCTTCCTTTGACTCGCTACCGCAAGGAGCTGGAGAAGCTCAAATCGGCCAAGCAGGTGCTGCGCGAGGTTGGTGCCGTCGGCGTCGAAGATGAGGATGAGAGGCTTACGCTGCCGACCTTAAGGCTGAGGTGGATGGCAACAAGAAGACTAAGCGTGTCAAGAAGGAGGAGGAGGCCGCAGAGCTCAAGGAGCTGGCCAAGACCATGATGTCGAAGAAGGTCAAGGGTTTGTACGACCGTATGCAGTATGGTCTGGGCAAGAAAGCTGACAAAATTCAGAAGCTCGAGGACAAGCGTGTCAAGCTGGAGGCAAGACGAAGAAGCTTCTAGCAGGCGGACTGTCAGGAATGATCTCAGCTCTAGCCTTCCAACCGCTGGGGCGTTACGCTGGCGCGGCACGTCGCCCACGCTCGTGCGCGTAGCAGGCGGTGCCGGTCTCTACTTTGCCACGCTTGACTACTGTCTGAACGTGTTCCCGAGCTCTGCAGTCAACACGTTTTTAGCTGGAGCCTTTTCCCGAGCATTTGCAGGAGGAATTGTATCACCGTTTACAATCGTGAAGGCGCGGATGGATACCTCCTGGCACTTTCGACAGTAAAGGGACGTGCCTTTCTCTGGACTATATGTGCTCATATACACGGCATCCGGCCGATGTCGTAAAAACACGCATGCATGCGTCAAACGGTGGCCAAGATCTACTGCGACGAAGGCCTGCATGGGTTCGCGAAGGGCATCGTTCCGCGTGTCATCAAGCGAACGCTCTCTACTGCAGTTACATGGACGATCTACGAGCTACTCGTTCGATCCATCGGGTCTGGGTCACGCCAAGGAGGCGTTTAATCTGGCCAAGGAGAGTGAACGCACCAAACAGATAGAAACCCAGGCCAAAATCAAGGAGAATGAGGCTCTTTACAAGCAGTACGAGATCGTGCGCGTGCAAAAGGAAGGCGAGGAGCGCCGTAAGACGCTCGAGGAGGAGACGCGCCAGCACCAACAGCGTGCGCAATACCAAGACCAGCTTAAGCGCAAACAGTACGCCGACCAGTTGGCTGCTCAGAAGTACATGAAAGAACAGGAGCTCAAAAAACAGGAGGAGATCCTCGCCCGTCAAGAAGCTTCGCGTCGCAAAACGCTCGACTATGAAGCCGAGCTGCGTCAGAAGACAGAGCTTTCTAGAGTGGCAGCTGAGACTGAAGGGCGCATCAAACAGGAGCGTCTTAATCACGACCTGCATTTAGAAGAAACCCGTGTGCGTGCCAAGGAGTATCGCGAGACGGTCATGGAAGGTATCAAGCTGGCGGGTAACACTATCGGTTCCGGTGTCATGGCTTTTGTGGACGATAAGGAAAAACTAACGGCCACTGTTGTCTCTCTTACGGCTCTGGCTGCTGGTATTTATTCCGCCAAGGTTTCTACTAGCGTTGCGGGTAAGTACATCGAAGCTCGAATGGGTAAGCCGTCGCTAGTTCGAGAGACTTCGCGTCGCTCGGCTGCGCAGGTAGTCGCCAATCCTATTCCATCCATCAAGCGTGTGCTGCGTCTGCAGAAGGCGACAGATGCGCTGGAGGGTGTCGTGCTTGAGCCTAAGCTGGACGCGCGTCTGCGTAGTGTCGCTGTGTCGACGTTTAACACGAAAAAAAACCGCGCGCCGTTCCGCCACTTGCTGCTGCACGGCCCGCCCGGTACGGGTAAGACGCTGTTCGCGAAGGCTCTGGCACGTCACTCTGGCCTGGAGTATGCCATCCTCACGGGTGGTGATGTTGCCCCACTTGGTCGCGAGGGTGTGACGGAGATCCACAAGCTGTTCGACTGGGCTTCGCACAGCCGTCGCGGTCTGCTGTTGTTCGTTGATGAAGCTGATGCATTCCTGCAGAAGCGTAGCAACACCGTCATGAGTGAGGATGTGCGCAACGCGTTGAATGCCTTCCTGTACCGCACAGGCGAAGCCAGCGACAAGTTTATGATTGTGTTCGCCAGCAACCAACCAGAGCAGTTTGATTGGGCCATTAATGATCGTATTGATGAAATGGTGGAGTTCCGCTTGCCTGGCTATGACGAGCGCGTACGCATGCTGAAGCAGTACTTTGATGACTACATCCGGGCTCCTAAGAACTCGCGTGCCAAGAAAATTTACGTGAGGGTGTGATCTTTACGGTAGACCCAGTCGCCAACTTTCTTGTGCCAAGACACGCATCTTTCAGCTTGAAGCATTGCAAGGCGTGTCTGGCGAAGCTCAGACTATCTTTGATGCTCTTAATAAAAGCCCATGCGCCTCTATTGGATCGTCTTTACCGTGGTATTGGGCAATGGATTGTACACGTATTTTACTGGAAAGGACGAGCTATTTTTAGTGGAGAAATTTACGAAAAAGATCCCGTCCGAGTACCGTATCTCGGAAAAATGGGACAAATGTCTCGAGAACTTTACGCTTTACTTTGGTGGTGGCCTTGTGGCTGGTGGCCTCACATCTCTCGTGCTAGCACGTTCTGGTGCTGGTCGTGGATTGATCACGGGTCTAGGTGCACGGGCGCTGGATCAAGCTGGACAACGTGTCAGATGGCGTTCACAGGTGATGCACAGGCAGCTCTGAAAAAGACGGAAAAGGCTGTGGATGATTTGCCGGCTTTCCAGCGACAGACAATCGTGGATTTTGTAAAGGCTTCAGCTTTGCAGCAACAGCTATTGGGAGTGCATACGGGGTTTCTGCGAGGAGACGAATGTGAAGATCTTGGAGACGTATTTAGCTGAACAAGTGCAGAACTCATTCGTGGATATTGACGCCAATTTGGCACTACTGAAGCTGTATCAAGTATACCCGTCCACGACCAATGCCGAAAGCCAATGTTCTAATCAAGGGTGTCATGACGCTTCCCTCTACATTTTTCACTGGTGTCCACGATGATCCCGGAGAGTACAAGCGAGGACGCCAATGTGAAGGTGGCACTGCAGACGGGCTTTATGCTTCAGTTGTGCCTGTTTGAGGATTTCTGGAAGGCGGATGTGACGTTTGCGAACAAAGTGCAGGGATTTTTGGAGTCGGTTCGCGCTTACATCTTGATGGCCATCTGCCACAGTCACAGTGTAATTTCGACCGACGTCTTGAAGGCCAAGCTCAATGTGTCGGACGAAGAAGTAGCTGAAATTGTCGCGGAGAAGTGGACTCGGGCAACTTGATTCAGATTAGCCCGAACGAAGACAATCAGATGCAGGCCAAAAAGGTCCAGGAGAACATCGAGTTTGACGACGTGCTCAAGGTCATTCACACCCTTTCTAGATCGAACGTCTGTGCCAGTGGTGGTGGTGTGACAGACGCAGAGGAGGTCTGTGACAATTTGGCCAAGCACTTGAGACTGGACGGAGGGACCCAGGTGCTGCCGTGCTCCACGGGTGTCATTGGCTGGTGTATCCCAGTGGACGAAATGGTGGAGAACCTCCCCAAGCTCATTAAAAACTTGCAATCTGACTCGGTACTACCTGCTGCTGAAGGGATTATGACCACTGATTTGTACCCAAAGATCCGCTCTATTGACGTGTGTGGAGGTAGAATCGTCGGTATCGCCAAGGGTGCTGGTATGATTGAACCCAACATGGCTACGATGCTGTCGTATATTCTTACTGATCTTTCGATTCCACGTAATGTGCTGCGTCAACTACTGACGGAGGTGGTTGGAGATACGTACAATTCGCTCAGTATCGATACGGACCAGAGCACGTCGGACACGGTGACACTCGTGTCATCAGACCAGATCCCCTTTGACATCAA---GCAGCTGGACGAGTTCAAGAAGGCGCTGCATAAGGTCTGCCTCGGACTGAGTGAAGATATTGTCCGCAATGGTGAGGGTGCACACCATGTCATGCGGGTGATCGTGCGTGGTGCGTTGTCGAAAGAGATGGCCAAAGGTGTCGGCAAGTCCGTGGTGAACTCGCCTCTGCTTAAGTGTGCAGTGGCTGGCAACGACCCGAATGTGGGCCGTCTTCTCATGGCTGTGGGCAAGTACATGGGCTTGCAGCACAAGGATGTGAGCATTCAGCACCGTATGAAGATTCGAATGGGTGGTGTAGTCATTTTCGAAAACGGCGAGATGGTGCTGAATGATGATATTGAAGCCAAGCTTGTGGCTCACATGCGTGGCGCGATGCTGATCGAGCCCACGCGCGGTGGACTCGATGCCAGCTCGCACAACTACCCACCTCACGGGAAGTTTGTCGAGATTGAGATTGATCTGGGCGTTGGAAACGAGAAGACTCAGGTACTGGGTATTGACCTTACCCACGAGTACGTGGCCATCAACGCCGACTATCGATCGTAATGGCGCC---GAAAAAGGGAAAGACTGCTGCTGCTTACCTGGCGTCTCTGGGCGTGACCTCCTC---CATTGGAGGTGAAAAGAAGGACGAC---AAGAAGAAGAAGAAAAAGGGCAAAAAGAAACTAAAAAGAGACCCATGTCAGCTGCAGCCAAGGCGCTGGCTGGAGCGACAGATGGCCTTAAAGGCCGAGCAGGAACA----------------------------------------------------------CTGGCGGAAATTGAGGCAGAGGAGGAGATCCGCCGCAAAAAGAAGGAGGCACGTGCCGCCAAGATCGAACGGCAGAAGAAGGAAGGCACGTACATGACTAAGGCTCAAAAGCAGAAGGCAGCAAAGGCTCAAGTGGCATTGGAGGCCATGCAACAGGCGGGTCTCATGCCGGAATCTGTT------GGAGAGAAGAAGACTGTCGTGTACTTAGACAACAAAAAGAAGAGCAAGAAGAGCAAGAGGAAAAAGAGGAGGAGGCCGAAGCCCCAGACGCGTGGGATGACGAGGATTGGGAGGCTAGCGATGTCGTGGCCTCGCTAGAAGATAAACTAGCAGAAGAAAGCGCAGGAGCGTCTGCGCCAGCAGGGTATTGCACTAAAAAAGCGTCGGGAGGAGCAGGCCGAGCAGATGCGTCTGGAGGCTGAGGAGCAGCATCAGCTCTCTAAGGCTAAACTCGAGGCTGAGCGCAAGAAGAAGGAGGCTGCCGATCGTCGCCGCGACCGTGAGGCTGCCGCACGAGCCAATGTGTCGCTGGACAATTTGCGCTCACCTATTTGCTGTATCATGGGGCACGTAGATACTGGTAAGACGAAATTGCTCGACAACATCCGTAAAACCAAGGTGCAGGATGCTGAAGCCGGCGGTATTACCCAGCAGATTGGTGCGACCTTCTTCCCTGTAGAGGCTATAAGACAGAAGACGGCTCGACTCAACGAGACAATGAAGCTGGATTTTCGGCTGCCTGGTCTGCTTGTGATCGATACACCTGGTCACGAGTCGTTTACGAACCTGCGTAATCGTGGCTCATCGCTGTGTGATATCGCTATATTGGTGGTTGATATAATGCACGGTTTGGAGCCTCAGACTCTGGAATCACTGCGTTTACTGCGCCAAAAGAAGGCACCATTCGTTGTGGCGCTTAACAAGATCGATCGTTGCTATGGCTGGAAAACAATGCCCGACATGCCCGTCCAGGAGGCACTTAAGCACCAGAATGAGCACGTCATTCGCGAGTTTGAGGATCGGATGAAAGCAATTATTGTAGAGTTTGCCGAGCTGAAGTTGAATGCCGAGGTTTACTGGCGCAATAAAGACTTGGCGCACACGGTCTCGCTGATCCCTACCAGTGCTATCTCGGGTGAGGGTGTGCCGGATCTGTTGATGATGCTGATTCGATTGACGCAGGAGCGTATGGCGAAGTCATTGGCATTCGTCGATATCTTGCAGTGTACGGTGCTAGAAGTGAAGGTGATCGAGGGTCTTGGTACGACCATTGATACGATTTTAGTGAATGGCACCCTAGAAGAAGGCACCAGTATTGTTGTGTGTACCTTAGATGGGCCTGTTGTGACAACTATTCGTTCATTACTGACTCCGCACCCTATGAAGGAAATTCGCGTCAAGGGCGAGTATATCCACCATCAAAAGATGAAGGCTGCTATGGGTGTGAAGATTTGCGCGCAGGGTCTTGAGAAAGCTGTTGCAGGTACACAGATTCACGTGGTTGGTCCTGATGATGATGTCGAGGAGCTGAAGGATTCTGTTATGTCCGACCTGACGAGCATTTTGGACTCGGTGAAGGCTACCCGCCGCGGTGTATTGGTTCAGGCTTCTACTCTTGGCGCTCTGGAAGCGCTTCTGGAGTTCTTGCGGACATGTGACCCGCCCATTCCGGTGTC---------------------------------------------------------------------------------------------------------TCACAATGACGCAACTGAGCTGGCATCCGAGCTTGGCGTGCGCATTTTTACGGCCGACATTATTTACCACTTGTTTGATCAGTTTACGGCGTACATGGACAACTTTCGTATGGCTCGCCGTGAGGAATTTGAGGAGACCGCTGTGTTTCCCTGTGTGCTCAAGATCCTTCCCGAGTGCGTTTTCAACAAAAAGGATCCCATCATTCTCGGTGTCGACATTGAAGAGGGCATCCTCAAGGTGGGTACGCCCCTCGTGGTGCCGTCCGCTGGTG---GGTTTTTCGTTGGCAAGGTGGGGTCCATCGAACGTGAGCACAAGGAGGTGGATCGCGCTAAGAAGGGCGCGTCGGTGGCCGTTCGGATAGACAACGAAGGCAGCGTCATGTATGGCCGCCACTTTGACCACAAAAACAAGCTCGTGTCGCGCTTGACCCGTCAGAGTATTGACGCACTGAAAGAGAATTTCCGAGAAGATCTTCAAAAGGAAGACTGGCAGCTCGTCATCAAGCTTAAGGCGGTGTTTGATATCATT----------------------------------------------------------------------------------------------------------------------------------------------------------------------------------------------------------------------------------------------------------------------------------------------------------------------------------------------------------------------------------------------------------------------------------------------------------------------------------------------------------------------------------------------------------------------------------------------------------------------------------------------------------ATGTATTCCTTTGCTGGTAATATGCTGCTGAGTTTCATGACGGCTATGGTAGCTATTGGTGTGTGTTGTGTGGCAGTGACTCCGACACAAACACCCGAGATTTCGAGCTTTCGGGTTTCCCAAGTCGTGCATGGATCTCCGACTGAGGTTTTTAGACTCCTTATGAATTCAAAGAGATTTCAACGATGGGATTCAGCAACAGCGACTATGCGTGTTGTCCAGCAATTGGATGACCATGCTGATATTGTCTATGTGACACAACGGCCGACGTACTTGTGGCCATTATGGCAGAAAGCTCGTGATCTCGTGTTCATGCGCTATTGGCGACGTGAAGAAGACGGGTCGTACTTTGTCATGTACCAGAGCATGGACCATCCCGAGTGTCGGGTGCGTCACAATTACGTACGAGCAAACATTCTGGGTGGCGGCTTTGTTATTGCTCCCCAGCGTGTTCCAGGT---GGCACCATTTGTACGCTCATCACTTACGTGCTCCGATACGATCCTGGTGGTTG---GTCGCGTATTTACCACCAGCTCGGTATGGATGTGGACTTGGTCATGCCTCAGCTGCGTAGTGTTGTGGGCATTCGCGACGAAATGAGCGCCACCAACTTCATCACACCCAACGTCTCGATGGCGTCAGCCGGAGAAGGCAGAAGCTGGTGAGGAAGCGTAGA---CTCCATGGGTGTTGTCATGGGTGTGCAGAAGCTTCAG---ACTAGTCTGCCGGAGAAGATGTGGGCCGAGCCTGACGCCTCTCTTTTCAGTGTGCGTGGCCACAATTATCTGAACGACAAGAAAAAGATTCCGTCAGCGCCGACAATGTTTCACACTGTAGGCGTGGATCTATTTTCATTTAGCAGTGCAGCCGAGCGATACAACGTTTCGTCTCGTGCGGACAGTGTTGGTTGCACTAG---CTCCAAGTTTACATTTCTTGTGAACATGATAATCCCGGGTACAGAAAACACGTGCATGGTGTTTTACTTCCATCCCGCGCGTGATAATGTGTTCGAAGACGGCTCGCCTTTTTCCGAGCTGCTGAACGACTTCTTTGATGGTGACGATGAATTCCGGAACTCGCGCTTCAAACTCATTCCGACCGTCGAAGAGGGTAGCTTCATTATCAAGCAGTCGGTCGGTTCGAAGCCGACACTCTTGGGTAACAAGTTAAAGTGCTCTTACTACCGTGGAGACAACTACTTCGAAGTGGACATCGACATTTCGTCGAACTCGGTTGCTAACACAGTCATGGGCATGGTGCAAGGTGTTACCAAATCGCTAGTGGTAGACATGGCATTCCTGCTTGAGGCTCAATCCGACGAAGAGCTGCCTGAAATCATCCTTGGTGCTGTGCGCCTGCAGCACGTTTCTCTTGACAACCCGCGTCGCATGCCACAGCATGGGGAATGAGTAGTAAAAAGGAGACCAAAGCTCGAAGTCTTGACGGAAGTTCTGCGTGTGAATTTGCAACGGTTGATCGTCTATGCGTGCTCAGCAGATACGGCTTTACAGCGAGAAATTGCCGAGAAATTGGCCAATGAGGCTGTCAAGATCGACAAGTACAAATTGTCGAGTTGGACGGCCTGCAGCTGCTGCTACCATTGACCAAGTCAAAGGATACAGAGGTGCAGCGTCTCGCAGCACACGCTCTCGCCAACCTCTCCGTCAACTCTGAGAATCAGTCGAAAATGGCCACGGAAGGAGGCATCAATATGCTTATTGACCTCTTGAGCAGTCTGAACGAACACGTGCAGCGCCAAGCTGCAAAGGCACTAGCTAATCTTGGCGTGAACGTGGATAACAAGGAACGAATCGCTAAGGCGGGTGGCATCCAACCGCTTATCGACTTGGCTAATTCTCGCCAGATTGGAGTCGCTGTCGAGGCCATTGCAGCACTTGCAAATCTAGCTGTTAACG---ACGCCAACGAGGTGGAGATTGCACGAAAAGGAGGCTTGAAGCCTATAATTGACGGAGCGCACTCGGAATCTGTTGAGCTCCAGTCGCAGGTGGCACGTGCCCTTCGCAACCTGTCTGTCAATCCCGAGAACAAACAAGCGATTGTGGAGTTGGGTGGAGTAGAGGCTCTGCAGTCACTTGTCCGTTCTACCAACGACCGGATATGCCAGCAGGCTACGCGCGCACTCGTTCCTACTGTGATCCTCACACGCTCATCACCTACAATCTATTG---GTCGTAACGCCCGTCGCTCGTGTTGCTCAAGTGAAGAAGGAATA------TCTGCCGCATGGTGGCAATTCATTAGACGTCGAGTACATTAAGACAGTCCAGACAGCT---------GACGTTCCTACAGCAATGACGG---ACGATACAAGCCGT---GGCTTTTCATCGATTTCCGACCGCTTTCCACTCTCTAAAGTAGGAAAAGTCCGTTGTAGCATGGCCCCTTTCTCAATAAGTCG---CTTGCTAAGTGCTACTACGCCCGAGTTAACAC---AGGTGGATCAG-------------ATGACAGTGCTT---GAAGACGGTACAGTGGACGATGAT---ACGATCGTTGCTGAA---GAAGAGAATGAGTGATGTATACCGGCATCAAGCTACGGA--CATGGAAGAAATCGAGATGGAGCAACTTCGCTGGAAGATCCAAGAACAAGTTTTCCAGCGATATTTCCAGCAAGAACAAAAGTATGAAGAATGGGGAAAATTAGACGAGCAAGACACCGAGTATGCTTCGTTCCAGGTGAAAGACGAGCAAATATATTGGCCGAGAAGAAGTTTCATGACAATGCTGTTCGAGACGCTATGGGAAAGGGAAATGCTACGTGGGTTAAGGAGCGACGAGCTCGGTTTGAGGATGAAATTCGTCAATTTTAAGAAGAAACAAGACACAAACAAGAGTTAATGCTTACACGTCTTGAAAAAGTGTCGCCATTCTCGAAGTTCCCCATTCTTGGCAACCGTTTTTTGCTGCTTCGTCTCCGAGGAAAGGGAGGCAACGGTGAAGTATGGGATGTTGTTGATTACGCCAACAATAAACTGATGTGTGCTCTCAAATTGTCGACTTCAATTCGCCACGCTCAACGTGAACACAACACGCACACTTAATCACCCCA--ACATTGTCCAGGTCGGCGAAGTGCCCTTCCTCATTCGATACGAGCAGCAGCAGTATACAGCGTTCACCGTTGCAAGTGTTCAATCGGACTTGCAGCAGCTTATTGAGA---TGTACGAATACTTGGATGATGATTCTGCATACAAGGTACTTTCGCAGCTGCTGAGCTCGCTAAAATATTTGCACGATGGGCGTTGCTCACTACGACCTGAAGCCGTCGAATATTTTAATCGATCGCGACGGGTGTGTGAAGTTG------ACTGATTTTGACTTGACGCGCCTTGCTAAGGCAGCTACGTCGAGCTCTAGGGTTGGCACTCTGCGGTACCTTCCTCCCGAGTGTTTCCGTCCAACCCGAGTCGA---------TTGCGAAGCTACGGCGGAGAAGGCGGACATGTGGATGGTAGGGATTGTATATTGCATGATGGTCACTGGAAAACACCCCATCTGCCCTAACAAGTCGACGCAAGCGGAAGTGGAGTCAGTGATGCGTAAGTACACGGGC---GAGGTCCGATATGCGCGGCCAGTTTGCGATCTAACCCGGTGGATCTTACAAGGGTGTCTTCACCCAGACCCAAGATGCCGACCAACGGCCGCGCAATTGCTAGTGGCGCTCCAG

Peronospora_effusa TGGATTACCACCGTGAAGTCGCCCGTGAGCTTGCCGCGTTCTTCCACTCCTTCTACCGCTTTGGTTCTGCCTCAAACCGCTTTGATCTGTTCCTGCACAACGTTTTATTGAGCTCGCTCTGCATTGGCACCGTCGTCTTTTTTGTGCTCTCCGTTATTGTTGTACGACGTACTGTGCTGCACATCCGTAGGCTTATGTACGTCGCACATCGACGGACTCTACATCCGTGGAAGTGCTAGCAGTGGGCCTCTTGACGTTCTTGGCAATCAGTGCCCTCGGGGGATTGCTAGGAGAAGCTGTCTGCAGACAAGTGACGCCTTGCGTGTCAATGCCGATAATCTGGTCACGTCGTTCAACGAGAGTGAACTACCAGCTGATGCGTTCAAACTATCGATTGAAGCACTGGACTGGTGCACACATCAAAGAATTTATGGAACCAGACGGACGCACTGCTGCCCAAAAACTTCTCAGAGGTGGCCAAGGACTGGGAACTCAGCTACTTTGTCTTGAAGTCATCGACGAATGGAGCTATTCTTGCGGTAACACTTGCCAGTTTCTTGTCCATTGCGTCGGTTGGGTGGGCGATGGTCTCGCCTCTTCGTGTGTCCATTCTCGTGATCTTGAGTGTCGTACCGATCTCGCACACACTAATTGGCGCTTATCTGTCATCGACAATCATGACGGCGGACTTTTGTGCTGCACCTATGAGCAACACGCTCAATCTCGTAGGCCCATGCCCGGCAAATGCGTCCTACCTTTTGCTGACGCTGTGGCTACGCAATGCATCCGGTCAAGGAGATGTCGGTCGTCGCATGAAGAAGGAGTTCTTGGACCCCATCGGAGCAATTGTGGGCGTCATGCTTGGCTATTTTGCAGGCACAGTGAGCAATTGGGAGAGCTCGACGCTTATGCAGACGTGGCGTTGGCCTTTTCTACTGCAGTTTGTAGCCTTGTTGCCTATTAATGTCGCGATCTTTTTCGTACCGACGAAACATCTCGTGATTCGAAGTGAGATCAGTGGGATACAGCCGCATGTGGCCGGCACGCCCATGAGTGCGAGGTCAACGACGTATCATGACGAGGAAGACGCACTGCTTGCTAGCGCGAGCATGCAACATACGGCGCCTTGGACAGGAGGTGTACAGCCAAGGCGCGTTCTCGGAAGGCGTGTATGAGCTGTTGCATATGCCCGTGTTCTGTCTGATTGTGTTTGGCCTCACGACAGTGTACTTCGTTGTCACGGGCGTGCAGTACTGGAGCACCATCTACATGATCAAGTCGCTGCATGCATCCAAGTATCTCGTTAACGCGCTTTTCGTGGTCGTCTCTGGCACAGGACCTATCCTGGGCGTATTTTTTGGTGGATGGCTGATTGACCGATCTGGAGGATATATCGGCGTTGAGCAGCGTGCGAGGGCGCTGGGTATCTGCATGAGTCTGGGGCTGACAGCCTTCTTTATTTCGGCGGTGGCGACGTTCTTCGACAACGTCTTTATTACCTCCGGCTTCTTGTGGCTGTTACTTTTCTTCGGCGGTGCAATCCTACCAGCACACCGTGCGCTCGCTTCTTCATTTTCCGTCATGGTCTTTAACCTGTTTGGTTACTCGCTCTCGCATGAGCGACTTGCAGATGGTCGTAGGTGCCAAGTGCTTTGTGCCAGACGAGACACACGTGTGGCTCGCAGCTGAAGTCTTACGTGACGAGAACACGGGCGATGGCAAGACACGCAAGATTTACTGCAAGGTTGAGCTGCCTGATGGAGAGTGAGGAGCGCTGTGTCGACATGTTGTGTAAGAAGACCAAGGCACTCATGCTGTGCATCAACTGGAGACGCTGCCGTATCAGAACGAGAACGTGGGGTCCGAAGGGATTGAAGACATGATTACACTCAACTACTTGCATGAAGCTGCTATTCTCTTCAACATTAAGAAGAGATTTCTCTGTGAACTACCGTATACGTATACAGGGGATATTTGTATTGCCATTAACCCTTACAAATGGCTACCTGATCTCTACGCAGAGGAGCAACATCTACGGTATCTTAACCAGCCGAAGGAGGAACTCCCTCCCCACGTGTACGCCACATCAGTTTCTGCTTATGACCATATGCGACGGTCGAAACTTAATCAGTCTATTCTAGTCAGTGGTGAGTCTGGTGCTGGTAAGACAGAGACCACTAAGATCCTTATGAATCATTTGGCTACTATTGCTGGTGGTATGAACAATTCGACTATTAAGAGGATTATTGAAGTGAATCCACTTCTTGAATCCTTTGGTAATGCCAAGACCGTACGAAACGACAACTCGAGTCGATTTGGAAAGTTTACACAGCTGCAGTTCGATAGAAAGGGTACACTTGTGGGTGCTAAATGCCGCACGTACCTGCTAGAGAAGACACGTGTCATCCAACACGAAGAGCCGGAGCGCAACTACCACATCTTCTACCAGTGGATTCTCCCGACATTGCATCGGAGCTACAGCTCGACTCGAAAAAACATTACGTGTACACGGGTGACAATACGGCACAAAAGATTGAGGGACTTTCTGATGGGAATCACTTTAACAAGACACGGGAGGCACTTGAGCTTGTGGGGCTGTCGCGTGGTGACCAGCAGCCGCTGTTTGAAGTGCTAGCGGGTGTGCTTCATCTTGGTGAAGTGCAGCTGCAATCAGACGCATCAGACGATGAGAAATCTCTCATAACGGTGGGTGACCGTGGTGCGAGTGCTGCTACTGAGATGTTTGGCGTCACATGGGAAGCGCTTGAAACTGCATTGTGTTCGCGTACCATGCGTGCCGTGAACGATGTGTACAGCGTGCCACTGAAGAAGGAGCTGGCTATGGACTGCCGTGATGCACTTGCGAAGGCGGTCTACTCCAACGTGTTTGACTGGCTTGTGGCCACGATTAACCAATCTCTCGCTGATGATTCCAACATGGGGAACCACGTGGGTGTACTCGATATCTTCGGTTTTGAGCATTTCAAGCACAACTCGTTCGAGCAGTTCTGTATTAACTATGCCAACGAGAAGCTTCAGCAGAAGTTCACTCAGGATGTGTTTAAGACTGTCCAGATTGAGTACGAGGAAGAAGGTATCGTCTGGGATCACATTGAGTATGCGGATAACCAAGATGTGCTGAGCGTTGTTGAGTCTCGCATGGGCATCATTTCACTGTTGAACGAAGAGGTGATGCGACCGAAGGGTTGCGAGGAGTCCTTCATGTCCAAGTTGACGTCCCTGCATAAGGAC---GACATGTCACACGTTATTGAGTTCCCTCGCACATCACGTACCGAGTTCCTGATCAAGCACTATGCTGCTCCTGTCATGTATGATTCGGTGGGATTCTTGGAGAAGCACAAGGATTCTCTGTTGCCTGACCTGTCCGAGCTCATGCGCGGTTCGTCCAAGCCTTTCATCGCAAAGCTCTTTGAACCAAAGCCGGAACCTAAGCCGTCGAAAGC---------GTGGTGGTGCTTTGTCTATCACGACTGTCGGTACTCAATTTAAGGAGAGCTTGACAGAGCTGATGGCGACCATTAACTCCACTCGAGTCCACTACGTGCGGTGCATCAAGCCTAATCCGATTAA---------GAGCTCAACGGTTATGGATCAGGCCATGGTGGTGTCTCAATTACGGTGTGCTGGTGTGATTGAAGCCATTCGTATCTCACGTGCTGCGTACCCGAATAGGTTGCAGCACATCGAGATTTTGGATAAGTTCTGGCTGTTTGTACCGAGCGGTGGTAACACTGCGGTAGAAAAGTGTCAACTACTCATGGACAAACTGAAACTAGAGTCCCCAGCGCAGTACCAGATGGGTAAGACTCGTGTATACTTTCAATTGGGTGTGCTCGAAGAGCTTGAAGATCGCCGTAAGAAATTTCTGGATGCCAAGGCCACATACCTGCAAAATGTCATGGTTGGTTTCACACAGCGTATCAAGTACCTTCGACAACTGGAAGCTATCATCAAGTTCAGTTATCAGGTGTGTGATTGCTATGCGGAGATACAACACGTTCATGAATGGCCTCGTTTTGGCGCAAGCTCGCTGGCGAGGCATCCAGGGCCGGAAGATTGCAGTGGTTGTCAAGAGCAACCACTATGCTGTCATCATCCAGCGGTATGTGCGCGGCTATGTCAAGCGTCACTCTTACGGCAAAATGCGGGCGGCGGTTGTCCGTGTACAAGCCATCGTTCGTATGACGATCCAGCGCCCAAAGTACTTGGCTGCTCTCGAAGAAAAACGTTACGAGGCTGACATGGCTTACCAGCTCAACAAGTTGAAGGCCGCTCTTCAGGAAGAGCAGGACAGGAATGCCCAGCT------GCAACGTC---GGAGTAGCTTGAC--GACGGCACGTCTGCTGCCTCGAGCGTGGTGATGGCTGATGCTGGTGGCATGATCGAAACTCTCCAAGATGAGAACAAGAAGCTGCGCGAGAAGAACGAGGGAATAAAGGTCGCGATGAAGGAGTTGAAGGCGGAGATCGAGAAGTTTAAAAGTGACAAGGAATTCTCATCGGCTGGCAACCATGTCAAGGTCCGTCAGCTGCAGGACACTGTGCGCGACAAGGAGAAGAAGATTAAGCTGCTCGAGATCGACAATAAGAAGCTGACGGAGCAGCTTGCAAAGCTTCAAGTGGACGG---TGT---ACCCGAGAAGAAGGCCACGCCGAAGAAGTCAATCTTCCGCACGCTAGGGTCCAAGAAGGAGAAGCAACCGC---------------GTGAGACCGTGCTGATGGACTCGCTTACTGGCTCGGAGGATCTTAACCATCGGGAGACTCGTGGGTCGGA---GCGTCAGTTTCCGCGTATGC---CGAGCATGAGCGTGGGCTCC---CGCTTCTGGAACTCTAACAAG---AAGGGCGAT------------AACGGCGATGGCTTCGACGTCTCGGAGGTTGAAGGTGAGGATAGTCCTACGAACCCACGCGCAACACGATCGTGAAGAGCATGGAGGTTGGAGTGACGGGAGCCATGTCAAGTTTGAAGGGCCGAATG---------TCGGTCGTGAAAGGCATGTATGAAGCAAACGCACGAACAGGATCGCCCTGTGCGCGAGTCGTTCAACCTGGATGCGTTACCGGAAGTGTCTCTGCCTGTCGGATGGGAGGCCAAGGTGTCTCGCTCAACGGGACGCGTGTACTATGTGAATCGCAAGCTTAGCAAGTCTCAGTTCGAGCGTCCCACACTAGCCTCCCTCAA------GGCACAAAAGCTGGCGCGTCAGAAGTCGGCTAACACGGAATAAGGCAGTTGTGGCAGATTTCGGCTGTGGAGATGCAAGGTTGTCAGAAAGTGTGCCGAACAAGGTGCACTCGTTCGATTTAGTATCTTTCGACATTGGCGTGTACTGTTTAGCGCTTATGGGGACAAGTGTACGCGAGTATATACGTGAAGTACATCGTGTGCTCAAGCCTGGAGGCATATTGATGGTTGCCGAGGTCAAGAGCCGCTTTGAGAGTGAGACGCTTGGCGGATGGTAGGAGTCTTTATCGCGACGGCGCATAACGTCGTGGTGACAGTGCGTAATACGACAGTGTTTGTGGGGAGATCAAGTATGCAGTTATTGTAGGCCAAAAGACGGGTGAGGCTGTAGCTGTGCCTTTCCCGGATGTGGCCCGTGACCTCAAGACGCTGTTGGGCCACACCACGTCCATGGTCACGGACGTGGCTGTCAATTTCGTCACTTCTTCTGACAGCTGATCGTGATGAGAAGTTACGTGTATCGCGATTTCCAAATGCTACGATCATTGAAAGCTACTGCTTGGGCCATACTGCGAGTCTGACCAAACTGGCTTGCAGTTCTGTCACGCCAGAACTCGTCGTTACGACGTCCATGGACAACACGCTGAAGCTCTGGAATATGATGACGGGGAAGCTAGCGATTTGTCACAAGACAACATTGTGGCGGTGCTCGTAAACTACCAGTACGTGCGATTTTTCGAGATCGTGAGAAGCCGTTCCTGCAATTGTTTTCCATTTCAAAGGATGACTGACGTGTTCAACTGCTGTGCGTGATTTTCGCAAGCTTCAGAATGATCCACCATCGGGTGTCTCGGGTGCTCCCATGGACAACAATATTATGCTCTGGCAGGCTGTCATCTTTGGACCCGATGATACGCCATGGGAAGGTGGTACTTTTAATCTCACGCTCGAGTTTAGTGAAGATTATCCAAACAAGGCACCGACCGTCAAATTTATCACCAAGATGTTCCACCCAAACATCTATAACGATGGTCAGATCTGCTTGGACATTTTACAGAACCAATGGTCGCCGATTTACGACATTTCCGCCATCCTTACGTCCATTCAGTCCCTGCTATGCGATCCCAATCCGAACTCACCCGCTAACTCGGAAGCCGCACGTCTTTTTCAAGAAAATCGACGAGAATACAACCGGCGTGTCCGTGAGATTGTCGAGCAGAGCTGGCAGGCCATTGATGGTATCGGCTGCTAAAATGCGTGGTGCTGAGAACCGCATGAATGTCGGTCGCCCTTTTACTGCGTGGCTGGATAATGTCTCTTCGGACCGTGGTCTTTGTGGTGGTATCAATTCAGGTATTGCCAAGGCTGCTCGCAAGCAGACAAGTCACAGATCTTTGTCATTGGTGACAAAGCTCGTGCCCAATTACGTCGTGATCTTGGTACCCATATCCGTGGCAATGTAACCGAGACGTTCCAGTCACCCCCGAACTTTACGGTAGCTAGTGCTATCGCAGAGGCTGTCGAATCGGAGAGGAGTCGTCGCGTATGGCTGCCATGGAGAACGCTACCACAAACGCCCAGGACCTCACGCTTACGCTCGTGTACAACAAGGCTCGCCAGGCCCGTATTACCACGGAGCTGATTGAAATTATTTCGGGCGCCGCCTCGCTGGACAGTGACTGGTAGCGTCGTGAACGTGCAAAATGTGACTCAATTTGACCAAGTGACTGCCCGGGATTCGACTTTTTCCGTGCTTTTTTTCTGGGCGGAGTTTCAAGCTTGTCGTCCTAATGGACAATTGGATATCGGGTCGTACGATGGTGGATAAATTAGAAGGTGCTAATGTCGCTGAATTGGCGAAACGGGTCTTGAAGATCGTCTTAAGAAATTGATGAATGCTTCATTTGTAATGCTCTTTATGAAGGGCACTCCAATGGAACCAAAATGTGGCTTTAGTCGACAGATTGTCACGTTATTGAACGATGAGAAGATTCAATTTGGAGCTTTTGATATTCTCAAGGACAATGACGTGCGTCAAGGGTTGAAACAATTGGTTGACTGGCCAACGTTTCCGCAACTTTATGTAAATGGTGAACTCATTGGTGGGCTGGATATTGTCAAGGAGATGAAGTCGGAAGGATCAATTGTCGAGCAATTAGGGAGGGAAGAAGCAGAAGCGACATTCCAAGAAAGTCTTTGTGCATTGATAAAGTCAGCACCTGTGCTGCTTTTTATGAAGGTTTGATATTTTGTATGGAGCTATCAACGCCAGGATCAAGATATCGCTAAGGTAGTGACTTTGATGCCATCATCGTTCCGCGAGGGTATGTGCGGAAGCCATCTCTTGGAGCCCGAAACCGGATGGCCATGGCATTTCGTGATCCTGTGAACGAAAAGGGAGAGTTCTGCATTGTGCAGCTTCCCGTATCACCTCGAAGTGCACTACATTACATCCAAGAGGTATTTCTACGATCGACTGGCAAGTGGAGCGTGCTGCACCGCGACCATCGTAGTGAAGTTAAATCGCTTTGCGTTCATTCCAAGCGTGATTGCTTGTACACTGGTGGTCTTGATGGCCTGTTGGTCCGGTACGATTTGAACAAGGGGCGTACGGAAACCGTGTTGGAACGTCGTAAGCACATTTCGAAGATCAACTCGGTCCTAGAGCATCCACACAATCCGAATTTGCTTCTGGTCAGTTGTGTGGAGCAGTAGGCACAGCTTGTTGTTGCATGACCTTCAGCGATACGATGGCAATGTGTCGAGTGGCGCGATGAGTCAGTACGTCGTGCCGCGGTGGTCGCCTGCAGGCTATCACGTATCATGTGGTAGCAAATGCGCTACTGCCACAACAGCTTCTCTACGCCCACATCCGCCACTATTGGAGCTTCGTTCCTACAGAAGCGCGTGATCGTCGGAGGGAAACTGACGCTGCAGATCTGGGACACAGCCGGTCAGGAACGCTTTCGCTCTATGGCGCCCATGTATTATCGAAATGCAAAGGCAGCTATTCTGGTCTTTGATTTACAGAGTGAAGCTAGTTTTGAGAAAATCAAAGAGTGGCTGCACCTGCAACATCATGTGGGTGACGATATGTGGGCAACAAGAGCGACGTTTTGACTTTTCCAAGGCCCAGCAGCTCGCCGACGAGATCGGTGCTCTGGTGTTCCGCACGAGCGCCAAGACGGGTGAAGGAGTGCAGTCATTGTTTGAGAGCCTAGCGGTGCAGCTGCTGAAGAAGCATGACCAGGAGCAGCGGAGAGTTTCTACGTGGTGTATATACCGGTAGATGCGAGTAAAGATCTCGAAGAGTGGACAGTAAAGCTACCTGTAGACAAGGAGGCACAACTTGGCTGTTTAACGGAACGACTACGTGAGCATTTCAAAAGTGGAAACATCCAAGCAACAACAGAGACTTTTAAGCAGCAACTACTCACTCAATTGCCCAAGGGCGCTACACTTACCGATGAAATGCTGCAAATGATGCATGGACAGTCTTGTGGACTCGATCCCGCTTGTGCTCAACACGCCCGATGTGAAGCATGTTGGAAGTTAAGGGTGATGCATTTATTGGACGGGTATTTGATAGACGATTTCGAGCGTATGGACTTCCGATTGAGTGACGTATCAGGTGATGCACCGTGGGTGCAGACGGCAAAGCTGCAGAGATCACGCAGTCATTAGAGGAGATTCTGGCGCTAGGTCAAGGTTTAGATGATCAGGATTACAAGGCTGAGCTTGAAGTGCTACAGAATGATCCGAATTCGAATTTATATTCGGCCGTTACATTCGAAGAGCTCAATTTGCCCGAGAAACTGCTAAAAGGCGTATACAATATGAAGTTTGCGAAACCGTCCAAGATTCAATCTGTAGCACTGCCACTGATCTTGGCTGATCCGCCCGAGAACCTCATTGGTCAGGCACAAAGTGGTATCCTACTCGTGAACTTGTGAGACAAATTATGGCTGTAGTAAACGCCATGGGCAAATTGTCGAGACTTTTCTTGCTATTCCAGGCAATGACTTGGCTCGTGGAGAAAGTTTAAAGGCTCAAGTGATTGTGGGAACGCCTGGAAAAGTTGAGGGGATGATTAAGAAGAAGCAACTGGACACTCGTGACGTGAAAATTTTTGTGCTGGATGAGGCAGATGTGATGGTGTCGGAGGACGGTATGCGCGAGCGCTCTGTGGCTATCAAGCGGCTGATCAAGAACCGTGCTTGTCAGTACCTTTTATTTTCGGCAACATATGCTGACGACGTGCGTGAATTTGCTCAGAAGATGGTGCCGGACCACAATATTATTACGGTCAAGAAGGAGAAGCTGACTCTCGATGGCATTAAACAGTTTTGGATCGACTGCAAGACCCGTGACAACAAGTTTCAGGTGCTTTCGGACATTTTTGCCGTTTTGACTATCGGCAAGTGCGTTATTTTCGTCCAGCAGCGCGAGACCGCCAAGGAGTTGACGCGTCGTATGCGTGACAAGGGCCACATGGTTGGAATCCTGCACGGTGCTGACATGGCCAAGGAGGTACGCGACCAAGTCATCGACGAGTTCCGTGCTGGCACGACCAACGTACTGATTACCACTAATGTGCTGGCTCGTGGTATTGATGTCGCGGGTGTGTCGCTCGTCGTGAACTTTGATATCCCGCTCACACGAGACCACCGCCCGGATCCCGAAACGTACCTGCACCGTATCGGCCGTACGGGTCGTTTTGGCCGCAAGGGTTGTGCTATTAATTTCGTACACGACAACAAGAGTAAGCAGGACCTTGCAGAAATTGAAGAGTACTACGCGCGGCCCATTACACAGGCTCCCGCCGATGACATTGAGGAACTCGAGAAGTTGCTGGCTTGGGGGGTAGTAAGCGTCGATTGGCCAAGTTTGGTAAAGAACAGCGCAAGGGCATGCGAATTTATCCACGTGAACCAAAGAAAAAGGTCTCTGGGAAAGATAAAACGTATTATTACATTAAAGACATCATGTTCTTGGCCCATGAACCAGTTCTCGACAAGTTTCGAGCATTTAAAACGTTCATGAAAAAAGTGACGAAAGCAATGGGTCGAAAAAACTATGAAGATGCCAAGCGGAAACATGACAATCGACCTACTTATGAGCTGGATCATATTGTGAAAGAACGGTATCCACGCTTTGTTGATGCTCTTGGAGATTTGGATGATGCCCTTTGTGGCAGAATTATATTGTCGTGACGAGAGGACTTACAAAGATCTTTGTAAGCATTAAGGGGATTTACTACCAGGCTAATGTACAAGGTCAAGTCATTACGTGGTTGGTGCCGCATCAATTTACTCCTACAGTCGACAAACGAGTGGATGTAAGAGTTATGCTTACGTTCTTGGAGTTTTATGAAGTGCTACTGAAGTTTGTCAATGCTCAATTGTACATGCAAATTGGCGTAGCTTATCCTCCTAAAATCAATTTGCAGCTTGATTCAGCCGGTGCTCAACTTGCTGCGATTGAACTAGCAGCAAAAGGAATCTGAAAAGCGGATCAAGTTACAGCGAACAGCGTAAAGAGCGCAGTGAAAAGAGTCTGTTCAGTGGTCTGACCTTCTTCTTGTCTCGAGAAGTTCCTTCGGCTTGTTTGGAACTGGTGCTGCGCTCGCACGGGGCCACGTTGGGCTGGGACGGCGCTGGATCTCCCTTTGATGAGAAAAGCACGCGCATTACGCATCACGTAATGGATCGTCCTCACCAGGGTCACCGTTACTTTAACCGCGAGTATGTGCAGCCGCAGTGGGTGTTCGACTCCGTAAACAATGGCATTTTGCTTCCTCTGGCTCGCTACCGCAAGGAGCTGGAAAAGCTCAAATCGGCAAAGCAGGTGCTGCACGAGGTTGGTGCCGCTGGCGTCGAAGAGGAGGATGAGAGGTTTACGCTGCCGACCTTAAGGCTGAGGCGGATGGCAACAAGAAGACGAAGCGTATCAAGAAGGAGGAGGAGGCTGCAGAGCTCAAGGAGCTAGCCAAGACCATGATGTCGAAGAAGGCCAAGCGTTTGTACGACCGTATGCAGTATGGTCTGGGCAAGAAGGCTGACAAAATTCAGAAGCTCGAGGACAAGCGTGTCAAACTGGAGGCAGGACGAAAAAGCTTCTAGCAGGCGCACTGTCAGGAATGATCTCAGCTCTTGCCTTCCAGCCGCTGGGGCGTTACGCTGGCGTGGTACGTCACCCACGCTCGTGCGCGTTGCAGGCGGTGCCGGTCTGTACTTTGCCACGCTGGACTATTGTTTAAACGTGTTTCCGAGCTCTGCAGTCAACACGTTTTTAGCTGGAGCCTTATCACGAACATTTGCAGGAGGAATTGTATCACCTTTTACGATCGTGAAGGCGCGGATGGATACCTCCTGGCACGTTCGACAGTAAAGGGACGTGCCTTTCTCTGGACTCTATGTGCTCATTTACACGGCATCCGGCCGATGTCGTAAAAACACGCATGCATGCGTCAAACGGTGGCCAAGATCTATTGCGACGAAGGCCTGCACGGGTTTGCGAAGGGCATCGTTCCGCGTGTCGTCAAGCGAACACTCTCTACTGCAGTCACATGGACGATCTACGAGCTACTCGTTCGACCCATCGGGTTTGGGTCACGCGAAAGAAGCGTTTAATCTAGCCAAGGAGGCTGAACGCACCAAACAGTTAGAAAATCAGTCTAAAATTAAGGAAAACGAGGCTCTCTATAAGCAGTACGAGATCGTGCGCGTGCAAAAGGAGGGCGAGGAGCGCCGTAAGACGCTCGAGGAGGAGACGCGCCAGCACCAACATCGTGCGCAATACCAAGATCAGATTAAGCGTAAACAGTATGCAGATCAGTTGGCTGCTCAGAAATACATGAAGGAACAGGAGTTAAAAAAACAGGAGGAGATCCTCGCCCGTCAAGAAGCTTCGCGTCGCAAAACGCTCGACTATGAGGCCGAGCTGCGTCAGAAAACAGAGCTTGCACGAGTGGCAGCTGAGACTGAAGGGCGCATTAAACAGGAACGTCTCAATCACGACCTGCATTTAGAAGAAGCCCGTGTGCGTGCCAAGGAATATCGCGAGACGGTCATGGAAGGTATCAAGCTGGCGGGTAACACTATTGGTTCCGGTGTCATGGCTTTTGTGGACGATAAGGAAAAACTCACGGCCACTGTGGTCTCTTTGACGGCTCTGGCTGTTGGTATTTATACCGCCAAGGTTTCTACTAGCGTTGCAGGTAAGTACATTGAAGCTCGAATGGGCAAGCCGTCGCTGGTTCGAGAGACTTCGCGTCGCTCGGCTGCTCAGGTAATCGCCAATCCCATTCCATCTATTAAGCGTGCGCTGCGTCTGCAGAAGGCGACAGATGCGCTGGAGGGTGTCGTGCTTGAGCCTAAGCTGGACGAGCGTCTGCGTAGTGTCGCTGTGTCGACGTTTAACACGAAAAAAAATCGCGCGCCGTTTCGCCACTTGCTGCTACACGGCCCACCCGGTACGGGTAAAACGCTGTTCGCCAAGGCTCTGGCACGTCACTCCGGCTTAGAGTATGCCATCCTTACGGGTGGTGATGTTGCCCCACTTGGTCGCGAGGGTGTGACAGAGATCCACAAGTTGTTCGACTGGGCTTCGCACAGCCGTCGCGGTCTGCTGTTGTTTGTTGATGAAGCTGATGCATTCCTGCAGAAGCGTAGCAACACTGTCATGAGTGAGGATATGCGCAACGCATTGAATGCCTTCCTGTACCGCACAGGTGAGGCCAGCGACAAGTTCATGATTGTGTTCGCCAGCAACCAGCCAGAACAGTTTGACTGGGCCATTAATGATCGTATCGATGAGATGGTGGAATTCCGCTTACCTGGCTATGACGAGCGTGTACGCATGCTGAAGCAGTACTTTGATGACTACATCCGGGCTCCCAAGAACTCACGTGCTAAGAAGATTTACGTGAGGGCGTGATCTTTACGGTGGACCCAGTCGCCAACTTTCTTGTGCCAAGACACGCATCTTCCAGCTTGAAGCATTGCAGGGCGTGTCTGGCGAAGCTCAGGCTATCTTTGATGCTCTTAATAAGAGCCCATGCGCATTTATTGGATCGTCTTTACGGTCGTATTAGGCAATGGATTGTATACGTATTGTACGGGCAAGGACGAGTCATTTTTCGTCGAGAAAGTCAAGAAAAAGATCCCGTCCGAGTACCGTATCTCTGAAAAATGGGACAAATGTCTTGAGAACTTTACACTTTATTTTGGTGCTGGTCTTGTGGCTGGTGGCCTCACGTCTCTTGTGCTCGCACGTTCTGGTGCTGGTCGTGGACTGATCACGGGTCTGGGTGTACGGGGGCTGGATCAAGCTGGACAACGTGTCAGATGGCGTTTGCAGGTGATGTACAGACAGCTTTGAAAAAGACGGAAAAGGCTGTGGATGATTTGCCGGCTTTCCAGCAACAGACAATCGTGGATTTTGTAACGGTTTCAGCCATACAACAGCAATTATTAGAAGTGCATACGGGGATTTTATGAAGAAATTCACGTGAAGATTCTAGAGACGTATCTCACTGAGCAAATGCAGAATTCCTTTGTCGACATTGATGCCAATTTGGCACTACTGAAGCTGTATCAACTATATCCGTCCACGTCCAATGTCGAGAGCCAATATTCTAATAAAAGGTGTCATGACGCTTCCCTCGACATTCTTTACGGGTGTCGACGATGATCCCAGAGAGCACAAGCGAGGATGTCAATGTGAAGGCAGCACTGGAGACAGGCTTTTTACTCCAGTCGTGTCTATTTGAAGATTTCTGGAAAATGGATCTGACGGTTGCAAACAAGGTGCAGGGATTTGTAGAGTCGGTCCGTGCTTACATTTTGACGGCCATCTCCCGTAGTCACAGTGTCATTTCGATCGACGTCTTGAAGGCCAAGCTCAATGTATCGGACGAGGAAGTAGCTGAAATTGTCGCGGAGAAGTGGACTCGGGCAATCTGATCCAGATTACTCCAAACGAAGACAATCAGATGCAGGCCAAGAAGGTCCAGGAGAACATTGAGTTTGAGGACGTGCTCAAGATATCGATCGAAGAAGCGGGATCGAACGTCTGTGCCAGTGGCGGTGGTGTTGCAGATGCAGAGGAAGTCTGTGATAATCTATCCAAGCACTTGAGACTGGACGGAGGGACCCAAGTGCTGCCCTGCTCTACGGGTGTCATTGGCTGGCGAATCCCAGTGGACGAGATGGTGGAAAACCTTCCCAAGCTTATCAAAAATTTGCAATCTGACTCGGTACTACCTGCTGCTGAAGGGATCATGACCACTGATTTGTACCCAAAGATCCGCTCTATTGACGTATGTGGAGGTAGAATTGTTGGTATTGCCAAAGGGGCCGGGATGATTGAACCTAATATGGCTACGATGCTGTCGTATATTCTTACTGGTGAGTACTGA-----------------------------------------------------------------------------------------------------------------------------------------------------------------------------------------------------------------------------------------------------------------------------------------------------------------------------------------------------------------------------------------------------------------------------------------------------------------------------------------------------------------------------------------------------------------------------------------------------------------------------------------------------------------------------------------------------------ATGGCGCC---GAAAAAGGGGAAGACCGCTGCTGCTTATCTAGCGTCATTAGGCGTGACTTCGTC---CATTGGAGGTGAGAAAAAGGACGAC---AAGAAGAAGAAGAAAAAGGGCAAAAAGAAGTCCAAAAGAGACCCATGTCAGCTGCAGCCAAGGCGCTCA-TGGAGCGACAGACGGCCTTGAAAGCCGAGCAGGAACGTCTAGAGAAAGAACGTCTAGAGCAAGAACGTCTTGCGCAGGAAGAGGAAGCTAAAAGACTGGCGGAAATTAAGGCGGAGGAGGAGATTCGCCGCAAAAAAAAGGAGGCACGCGCTGCCAAGATTGAACGGCAGAAGAAGGAAGGCACGTATATGACTAAAGCCCAAAAGCAAAAGGCAGCAAAGGCTCAAGCGGCATTGGAGGCCATGCAACAGGCGGGTCTCGTGCCGGAATATGCT------GGTGAGAAGAAGACGGTCGTGTATATCGATAACAAAAAGAAGAACAAGAAGAGCAGGAGGCAAAACAGGAGGAGGCCGAAGCCCCAGACGCGTGGGATGACGAAGATTGGGAGGCCAGCGATGTCGTGGCCTCGCTAGAAGATAAGCTAGCAGAAGAAGGAGCAGGAGCGTCTGCGTCAGCAGGGTATTGCACTAAAGAAGCGTCAGGAGGAGCAGGCCGAGCAGATGCGTCTGGAGGCTGAGGAGCAACATCAGCGCTCCAAGGCTAAACTCGAAGCTGAACGCAAAAAGAAGGAGGCTGCCGACCGTCGTCGCGACCGTGAGGCCGCCGCACGAGCCAATGTGTCGCTGGACAATTTGCGCTCACCTATTTGCTGTATCATGGGTCACGTCGATACTGGTAAGACGAAGTTGCTCGACAATATCCGCAAAACCAAGGTGCAGGATGCTGAAGCTGGCGGTATTACCCAGCAAATTGGTGCGACCTTCTTCCCTGTAGAGGCTATAAAACAGAAGACGGCTCGGCTTAACGAGACAATGAAGCTGGATTTCCGGCTGCCTGGTCTGCTTGTGATCGATACACCCGGTCACGAATCGTTCACGAATCTGCGTAATCGTGGCTCATCACTCTGTGATATCGCTATTTTGGTGGTTGATATAATGCACGGTCTGGAGCCTCAGACTTTGGAATCACTGCGCCTGCTGCGCCAAAAGAAGGCACCATTCGTTGTGGCGCTTAACAAGATCGATCGTTGCTATGGCTGGAAGACGATGCCCGACATGCCCGTTCAGGAGGCACTTAAGTACCAGAATGAGCACGTCATTCGCGAGTTTGAGGACCGCATGAAAGCAATTATTGTGGAGTTTGCCGAGCAGAAGTTGAACGCCGAGGTCTACTGGCGCAATAAAGACTTGGCGCACACGGTCTCGCTGATCCCTACCAGTGCTATCTCGGGTGAGGGTGTGCCGGATCTGCTGATGATGCTGATTCGTTTGACGCAGGAGCGTATGGCAAAGTCATTGGCGTTCGTCGATATCTTGCAGTGTACGGTGTTAGAAGTGAAGGTGATCGAGGGTCTCGGTACGACCATTGATACAATCTTAGTGAATGGTACACTGGAAGAAGGCACCACTATTGTTGTGTGTACATTAGATGGGCCTGTTGTGACAACTATTCGCTCATTACTGACTCCGCATCCTATGAAAGAAATTCGCGTTAAAGGCGAGTATATCCACCATCAAAAGATGAAGGCTGCTATGGGTGTGAAGATTTGCGCGCAGGGTCTTGAGAAAGCTGTTGCCGGTACACAGATTCACGTGGTTGGCCCAGATGATGATGTCGAGGAGCTAAAAGATTCTGTCATGTCCGACCTGACAAGCATTTTGGACTCGGTGAAGGCTACCCGCCGCGGTGTATTGGTTCAGGCTTCTACTCTTGGCGCTCTGGAAGCGCTTCTGGAGTTCTTGCGGACGTGTGACCCGCCCATTCCGGTGTCCTGTGTTAACATTGGTCCAGTACACAAGAAGGATGTTATGCGAGCTTCTGTGCAGCTCGAGCATCAGCCCGAATTCGCTACAATTTTGGCATTTGACGTGAAGGTCCACAATGACGCCACTGAGCTGGCATCCGAGCTTGGCGTGCGCATTTTCACGGCTGACATTATTTACCACTTGTTCGATCAGTTTACGGCGTACATGGACAACTTCCGTATGGCACGCCGTGAAGAATTTGAGGAGACCGCTGTGTTTCCCTGCGTGCTCAAGATCCTTCCCGAGTGCGTTTTCAACAAGAAGGATCCTATCATTCTCGGTGTCGACATTGAAGAGGGTATCCTTAAGGTGGGTACGCCTCTCGTGGTGCCGTCTGCTGGTG---GGTTTCTGGTTGGCAAAGTGGGATCCATCGAACGTGAGCACAAGGAGGTGGACCGCGCTAAGAAGGGCGCATCGGTGGCCGTTCGGATAGACAACGAAGGCAGCGTCATGTACGGCCGTCACTTTGACCACAAAAACAAGCTGGTGTCCCGCTTGACCCGTCAGAGTATTGACGCACTGAAAGAGAATTTTCGAGAAGATCTTCAGAAAGAAGACTGGCAGCTCGTCATTAAGCTCAAGGCGGTGTTCGATATCATTATGGAAGGATGGCTTACGAAACGTCGGGACCATATGAACTTGATTTGGCGGGATCGCTATTTTCTTTTGGATTGTAACCAGTTACGATATTATCGTAAACGTGGAGATCCAGCACCTCGGGGTACATATATCCTAACTGATGGATGTATCGTGAGTTCTGTGTTTAATTCCGAAGAAAAACATAAACATCATGGCCCCGTGTGGATGTTTCGGATCACTTGAAAAACAAGCGAAAAAAGACCGATTTTTAGATTTGGGAGCCAAAAGTGAAGAAAATGCTAAAAATTGGAAAAAAGCCTTGGAAACAGCTGTTCGATCGTTGCAGGAATTGGTGCTCCATCAACGAAAACTCAAATTTGATTCCGAGAA---TTTTCCAACGCCGACATAT---------GTTCCACGGATGCTCCAAGCTGATGAAGACACAATGTCTTTATTTAATTTAGATGCTGATAATGTCAAGGCTGAACAAGTGTGGTGGTTGGCTCGAGTGGAAGGAGGATTACGGATCATGCAGGAATGTCCATTGGGACCAAATGTTGCTGCAACGTTGTGTTTTCAACATGCAACGACGACAAATGGATTGATTTGTAGTGCTGTCCTGGGAGCAGCATTGAGTTTTAGTATCATGTATTCCTTTGCTATGAATATGTTGCTAAGTTTCATGACGGCTATGATGGCTATTGGTGTTTGTTGTGTAGCCGTGATGCCAACTCAAACACCAGAGATTTCAAGCTTTCGAGTATCACAAGTTGTACATGGATCACCGACTGAAGTGTTTCGACTCATTATGAATTCCAAACGATTTCAACGATGGGATGCTGCAACAGCAACGATGCGTGTTGTGCAGCAATTGGATGATCATGCTGATATTGTCTATGTGACACAACGACCGACGTACTTGTGGCCATTATGGCAAAAAGCTCGTGATCTTGTCTTTATGCGTTATTGGCGACGTGAAGAAGACGGATCGTACTTTGTCATGTACCAGAGCATGGACCATCCCGAGTGTCGGGTGCGTCACAATTACGTACGAGCAAACATTCTCGGTGGCGGCTTTGTTATTGCTCCCCAACGTGTTCCAGGT---GGAACCATTCGTACGCTCGTCACGTATGTGCTCCGATACGATCCTGGTGGTTG---GTCGCGCATTTACCACCAGCTCGGTATGGATGTGGACTTGGTCATGCCTCAACTGCGCAGTGTTGTGGGCATTCGCGATGAAATGAGCGCCACTGACTTCATCACACCCAACGTCTCCATGGCGTCAGCTGGCGAAGGCAGAAGCTGGTCAGGAAGCGTAGA---CTCTATGGGTGTTGTCATGGGTGTGCAGAAGCTTCAG---ACCAGTTTGCCAGAGAAGATGTGGGCCGAGCCTGACGCCTCTCTTTTCAGTGTGCGAGGCCACAATTACCTAAACGACAAGAAGAAGATTCCGTCAGGACCGGCAATGTTTCACACTGTAGGTGTGGATCTATTGTCATTCAGCAGTGCAGCCGAGAGATACAACATTTCGTCTCGTGCGGACAGTGTTGGTCGCACTAG---CACCGAGTTTACGTTTGTTGTGAACATGATCATCCCGGGTACCGAAAACACGTGCATGGTGTTTTACTTTCATCCTGCGCGTGATAATGTGTTCGAGGACGGCTCGCCTTTTTCCGAGCTGCTGAACGACTTCTTTGATGGCGACGATGAATTCCGGAACTCGCGCTTCAAACTCATTCCGACCGTGGAGGAAGGTAGCTTCATTATCAAGCAGTCGGTCGGTTCAAAGCCGACACTCCTGGGCAACAAGTTGAAGTGCCTTTACTACCGTGGAGACAACTACTTTGAAGTGGACATTGACATTTCGTCGAACTCGGTTGCTAACACAGTCGTGGGCATGGTGCAAGGTGTTACCAAATCGCTGGTGGTAGACATGGCATTCCTGCTCGAGGCTCAATCCGACGAAGAGCTGCCTGAAATCATCCTTGGTGCCGTGCGCCTACAACACGTTTCTCTTGACAACCCGCATCGCATGCCACAGCATGGGGAATGGACAGCAAAAATGAGATTAACGTCCGAAGTCTTGACGGAAGCTCTACGTGTGAATCTGCAACAATTGATCGTCTTTGCGTGCTCTGCGGATACGGCTTTGCAACGAGAAATTGCCGAGAAATTGGCCAATGAGGCTGTCAAGATCGACAAGTACAAATAGTCGAATTGGACGGACTGCAGCTGCTGCTACCATTAACCAAGTCAAAGGATGCAGAAGTGCAGCGTCTAGCAGCACATGCTCTTGCCAACCTCTCTGTCAATTCTGAGAATCAGTCCAAAATGGCCACGGAAGGAGGCATCGACATGCTTATTGACCTCTTGAGTAGTTCAAACGAACACGTGCAGCGTCAAGCTGCAAAGGCACTTGCTAATCTTGGCGTGAACGTGGACAACAAGGAACGAATCGCTAAGGCGGGTGGCATTAAACCGCTGATTGACTTGGCTAACTCGCACCAGATCGGAGTCGCTGTCGAGGCCATTGCAGCACTTGCAAATTTGGCTGTCAATGGTAATGTTTTTTTGACAAACACTGCTTTCTGA---------------------------------------------------------------------------------------------------------------------------------------------------------------------------------------------------------CCTACTGTGGCCCTCACGCGCTCATCACCTACAATCTAGTG---GTCGTCACGCCCGTCGCTCGTGTTGCTCAAGTGAAGAAGGAATA------TCTGCCGTATGATGACAACTCATTGGATGTCGAGTACATTAAGACAATTCCAACGGTT---------CACGTTCCTTCAGTAATGACGG---ACAAGACAAGTCGT---GGCTTTGTGTCGATTTCCGACCGCTTTCCACTCTCGAAAGTGGGAAAAGTTCGTTGTAGCATAGCCCCTTTCTCAATAAGGCG---TTTGCTAAGTG---CTACGCCCGAGTTAACAC---TTGTGGATCAG-------------ATTACAGTGCTT---GAAAACGATACAGTGAATGATAAT---ACGATCGTTGCTGAAAAGCAAGAGAATGAGTGATGCGTATAGGCATCAAGCTACGGA--CGTGGAAGAAATTGAGATGGAGCAACTTCGATGGAATATCCAAAAACAGGTTTGCCAGCGATATTTCCAGCAGGAACAAAAGTATGAAGAATGGGGAAAATTTGACGAGCAAGACACCGAGTATTCTTCGTTCCAGGTGGAAGACGAGCAAATATATTGGCCGAGAAGAAGTTTCATGACAATGCTGTTCGAGACGCTATGGGAAAGGGAAATGCTACGTGGGTCAAGGAGCGACGAAATCGGTTTGAGGAGGAAATTCGTCAGTTCTAAGAAGAAACGAGACACAAACAAGAGTTAATGCTTACACGTCTTGAAAAAGTGTCACCATTTTCGAAGTTTCCCATCCTTGGCAATCGTTTTTTGCTGCTTCGTCTGCGAGGAAAGGGAGGCAACGGTGAAGTATGGGATGTTGTTGATTACGCCAACAATAAACAGGAGTGCGCTCTCAAGCTGTCGACTTCAATTCGCCACGCTCAACGCGAACACAACACGCACGCTTCATCACCCCA--ACATTGTCCAGGTCGGCGACGTGCCCTTCCTCATTCGATACGAGCAGCAGCAGTACACAGCGTTCCCGGTTGCAAGTGTTCAATCGGACTTGCAGCAACTTATTGAGA---TGTACGAGTACTTAGATGATGATTCTGCATACAAGGTGCTTTCGCAGCTGCTGAGCTCGCTGAAGTATTTGCACGATGGGCCTTGCTCACTATGACCTGAAGCCGTCGAACATTCTCATCGACCACGACGGGTGTGTGAAGCTG------ACTGATTTTGGCCTGACGCGCATTGCTAAGGCAGCTACGTCGAGCTCTAGGGTTGGCACTCTGCGGTACCTTCCTCCCGAGTGTTTCCGTCCAACCCGAGTCGG---------TTGCGAAGCTACGGCGGAGAAGGCGGACATGTGGATGGTAGGGATTGTATACTGCATGATGGTCACTGGAAAACACCCCATCTATTCTAACAAATTGACGCAAGCGGAAGTGGAGGCAGTGATGGGTAAGTACACTGGC---GAGGTCCAATATGCGCGGCCAGTTTGCGATCTAACCCGGTGGATCTTACAAGGGTGCCTTCACCCGGACCCAAGATGCCGACCAACGGCCGCGCAATTGCTGGTGGCACTCCAG

Peronospora_farinosa TGGATTACCACCGTGAAGTCGCCCGTGAGCTTGCCGCGTTCTTTCACTCCTTCTACCGCTTTGGTTTTGCCTCAAACCGCTTTGATCTGTTCCTGCACAACGTTTTATTGGGCTCGCTCTGCATTGGCACCGTCGTCTTTTTTGTGCTCTCCGTTATTGTTGTACGACGTACTGTGCTGCACATTCGTAGGCTTATGTACGTCGCACATCGACGGACTCTACATCCGTGGAAGTGCTAGCAGTGGGCCTCTTGACGTTCTTGGCAATCAGTGCCCTCGGGGGATTGCTAGGAGAAGCTGTCTGCAGACAAGTGACGCCTTGCGTGTCAATGCCGACAATCTGGTCACGTCGTTCAACGAGAGTGAAGTACCAGCTGATGCGTTCAAATTATCGATTGAAGCACTGGACTGGTGCACACATCAAAGGATTTATGGAACCAGACGGACGCACTGCTGCCCAAAAACTTCTCAGAGGTGGCCAAGGACTGGGAACTCAACTACTTTGTCTTGAAGTCATCGACGAATGGAGCTATTCTTGCGGTAACACTTGCCAGTTTCTTGTCCATCGCGTCGGTTGGGTGGGCGATGGTCTCGCCTCTTCGTGTGTCCATTCTCGTGATCTTGAGTGTCGTACCGATCTCGCACACACTAATTGGCGCTTATCTGTCATCGACAATCATGACGGCGGACTTTTGTGCTGCACCTATGAGCAACACGCTCAATCTCGTAGGCCCATGCCCGGCAAATGCGTCCTACCTTTTGCTGGCGCTGTGGCTACGCAATGCATCCGGTCAAGGAGATGTCGGTCGTCGCATGAAGAAGGAGTACTTGGACCCCATCGGAGCAATTGTGGGCGTCATGCTTGGCTATTTTGCAGGCACAGTGAGCAATTGGGAGAGCTCGACGCTTATGCAGACGTGGCGTTGGCCTTTTCTACTGCAGTTTGTAGCCTTGTTGCCTATTAATGTCGCGATCTTTTTCGTACCGACGAAACATCTCGTGATTCGAAGTGAGATCAGTGGGATACAGCCGCATGTGGCCGGCACACCAATGAGTGCAAGGTCAACGACGTATCATGACGAGGAAGACGCACTGCTTGCTAGCGCGAGCATGCAACATACGGCGCCTTGGACAGGAGGTGTACAGCCAAGGCGCGTTCTCGGAAGGCGTGTATGAGCTGTTGCATATGCCCGTGTTCTGTCTGATTGTGTTTGGCCTCACGACAGTGTACTTCGTTGTCACGGGCGTGCAGTACTGGAGCACCATCTACATGATCAAGTCGCTGCATGCATCCAAGTATCTCGTTAACGCGCTTTTCGTGGTCGTCTCTGGCACAGGACCTATCCTGGGCGTATTTTTTGGTGGATGGCTGATTGACCGATCTGGAGGATATATCGGCGTGGAGCAGCGTGCGAGGGCACTGGGTATTTGCATGAGTCTGGGGCTGACAGCCTTCTTTATTTCGGCGGTGGCGACGTTCTTCGACAACGTCTTTATTACCTCCGGCTTCTTGTGGTTGTTACTTTTCTTCGGCGGCGCAATCCTACCAGCACACCGTGCGCTCGCTTCTTCATTTTCCGTCATGGTCTTTAACCTGTTTGGTTACTCGCTCTCTCATGAGCGACTTGCAGATGGTCGTAGGTGCCAAGTGCTTTGTGCCAGACGAGACACACGTGTGGCTCGCAGCTGAAGTCTTACGTGACGAGAACACGGGCGATGGCAAGACACGCAAGATTTACTGCAAGGTTGAGCTGCCTGATGGAGAGTGAGGAGCGCTGTGTCGACATGTTGTGTAAGAAGACCAAGGCACTCATGCTGTGCATCAACTGGAGACGCTGCCGTATCAGAACGAGAACGTGGGGTCCGAAGGGATTGAAGACATGATTACACTCAACTACTTGCATGAAGCTGCTATTCTCTTCAACATTAAGAAGAGATTTCTCTGTGAACTACCGTATACGTATACAGGGGACATTTGTATTGCCATTAACCCTTACAAATGGCTACCTGATCTCTACGCAGAGGAGCAACATCTACGGTATCTTAACCAGCCGAAGGAGGAACTCCCTCCACACGTGTACGCCACATCAGTTTCTGCTTATGACCATATGCGACGGTCGAAACTCAACCAGTCTATTCTAGTCAGTGGTGAGTCTGGTGCTGGTAAGACAGAGACCACTAAGATCCTTATGAATCATTTGGCTACTATTGCTGGTGGTATGAACAATTCGACTATTAAGAGGATTATTGAAGTGAATCCACTTCTTGAATCCTTTGGTAATGCCAAGACCGTACGAAACGACAACTCGAGTCGATTTGGAAAGTTTACACAGCTGCAGTTCGATAGAAAGGGTACACTTGTGGGTGCTAAATGCCGCACGTACCTGCTAGAGAAGACACGTGTCATCCAACACGAAGAGCCGGAACGCAACTACCACATCTTCTACCAGTGGATTCTCCCGACATTGCATCGGAGCTACAGCTCGACTCGAAAAAACATTACGTGTACACGGGTGACAATACGGCACAAAAGATCGAGGGACTTTCTGATGGGAATCACTTTAACAAGACACGGGAGGCTCTTGAGCTTGTGGGGCTGTCGCGTGGTGACCAGCAGCCGCTGTTTGAAGTGCTAGCGGGTGTGCTTCATCTTGGTGAAGTGCAGCTGCAATCAGACGCATCAGACGATGAGAAATCTCTCATAACGGTGGGTGACCGTGGTGCGAGTGCTGCTACTGAGATGTTTGGCGTCACATGGGAAGCGCTTGAAACTGCATTGTGTTCGCGTACCATGCGTGCCGTGAACGATGTGTACAGCGTGCCACTGAAGAAGGAGCTGGCTATGGACTGCCGTGACGCACTTGCGAAGGCGGTCTACTCCAACGTGTTTGATTGGCTTGTGGCCACGATTAACCAATCTCTCGCTGATGATTCCAATATGGGGAACCACGTGGGTGTACTCGATATCTTCGGTTTTGAGCATTTCAAGCACAACTCGTTCGAGCAGTTCTGTATTAACTATGCCAACGAGAAGCTTCAGCAGAAGTTCACTCAGGATGTGTTTAAGACTGTCCAGATTGAGTACGAGGAAGAAGGTATCGTCTGGGATCACATTGAGTATGCGGATAACCAAGATGTGCTGAGTGTTGTGGAGTCCCGCATGGGCATCATTTCACTGTTGAACGAAGAGGTGATGCGACCCAAGGGTTGCGAGGAGTCTTTCATGTCCAAGTTGACGTCCCTGCACAAGGAC---GACATGTCACACGTTATTGAGTTCCCTCGCACATCACGTACCGAGTTCCTGATCAAGCACTATGCTGCACCTGTCATGTATGATTCGGTGGGATTCTTGGAGAAGCACAAGGATTCGCTGTTGCCTGACCTGTCCGAGCTCATGCGCGGTTCGTCCAAGCCTTTCATCGCAAAGCTCTTTGAACCAAAGCCGGAACCTAAGCCGTCGAAAGC---------GTGGTGGTGCTTTGTCTATCACGACTGTCGGTACTCAATTTAAGGAGAGCTTGACAGAGCTGATGGCGACCATTAACTCAACTCGAGTCCACTACGTGCGGTGCATCAAGCCTAATCCGATTAA---------GAGCTCAACGGTTATGGATCAGGCCATGGTGGTGTCTCAATTACGGTGTGCTGGTGTGATTGAAGCCATTCGTATCTCACGTGCTGCGTACCCGAATAGGTTGCAGCACATCGAGATTTTGGATAAGTTCTGGCTGTTTGTACCGAGCGGTGGTAACACTGCGGTAGAAAAGTGTCAACTACTCATGGACAAACTGAAACTAGAGTCCCCAGCGCAGTACCAGATGGGTAAGACTCGTGTATACTTTCAATTGGGCGTGCTCGAAGAGCTTGAAGATCGCCGTAAGAAATTTCTGGATGCCAAGGCCACATACCTGCAAAATGTCATGGTTGGTTTCACACAGCGTATCAAGTACCTTCGACAACTGGAAGCTATCATCAAGTTCAGTTATCAGGTGTGTGATTGCTATGCGGAGATACAACACGTTCATGAATGGCCTCGTTTTGGCGCAAGCTCGCTGGCGAGGCATTCAGGGCCGGAAGATTGCAGTGGTTGTCAAGAGCAACCACTATGCTGTCATCATCCAGCGGTATGTGCGCGGCTATGTCAAGCGTCACTCTTACGGCAAAATGCGGGCGGCGGTTGTCCGTGTACAAGCCATCGTTCGTATGACGATCCAGCGCCCAAAGTACTTGGCTGCTCTCGAAGAAAAACGTTACGAGGCTGACATGGCTTACCAGCTCAACAAGTTGAAGGCCGCTCTTCAGGAAGAGCAGGACAGGAATGCCCAGCT------GCAACGTC---GGAGTAGCTTGAC--GACGGCACGTCTGCTGCCTCGAGCGTGGTGATGGCTGATGCTGGTGGCATGATCGAAACTCTCCAAGATGAGAACAAGAAGCTGCGCGAGAAGAACGAGGGAATAAAGGTCGCGATGAAGGAGTTGAAGGCGGAGATCGAGAAGTTTAAAAGTGACAAGGAATTCTCATCGGCTGGCAACCACGTCAAGGTTCGTCAGCTGCAGGACACTGTGCGCGACAAGGAGAAGAAGATTAAGCTGCTCGAGATCGACAATAAGAAGCTGACGGAGCAGCTTGCAAAGCTTCAAGTGGACGG---TGT---ACCCGAGAAGAAGGCCACGCCGAAGAAGTCAATCTTCCGCACGCTAGGGTCCAAGAAGGAGAAGCAACCGC---------------GTGAGACCGTGCTGATGGACTCGCTTACTGGCTCGGAGGATCTTAACCATCGGGAGACTCGTGGGTCGGA---GCGTCAGTTTCCGCGTATGC---CGAGCATGAGCGTGGGCTCC---CGCTTCTGGAACTCTAACAAG---AAGGGCGAT------------AACGGCGATGGCTTCGACGTCTCGGAGGTTGAAGGTGAGGATAGTCCTACGAACCCACGCGCAACACGATCGTGAAGAGCATGGAGGTTGGAGTGACGGGAGCCATGTCAAGTTTGAAGGGCCGAATG---------TCGGTCGTGAAAGGCATGTATGAAGCAAACGCACGAACAGGATCGCCCTGTGCGCGAGTCGTTCAACCTGGATGCGTTACCGGAAGTATCTCTGCCTGTCGGATGGGAGGCCAAGGTGTCTCGCTCAACGGGACGCGTGTACTATGTGAATCGCAAGCTTAGCAAGTCTCAGTTCGAGCGTCCCACACTAGCCTCCCTCAA------GGCACAAAAGCTGGCGCGTCAGAAGTCGGCTAACACGGAATAAGGCAGTTGTGGCAGATTTCGGCTGTGGAGATGCAAGGTTGTCAGAAAGTGTGCCGAACAAGGTGCACTCGTTCGATTTAGTATCTTTCGACATTGGCGTGTACTGTTTAGCGCTTATGGGGACAAGTGTACGCGAGTATATACGTGAAGTACATCGTGTGCTCAAGCCTGGAGGCATATTGATGGTTGCCGAGGTCAAGAGCCGCTTTGAGAGTGAGACGCTTGGCGGATGGTAGGAGTCTTTGTCGCGACGGCGCATAACGTCGTGGTGACAGTGCGTAATACGACAGTGTTTGTGGGGAGATCAAGTACGCAGTTATTGTGGGCCAAAAGACGGGTGAGGCTGTAGCTGTGCCCTTCCCGGATGTGGCCCGTGACCTCAAGACGCTGTTGGGCCACACCACGTCCATGGTCACGGACGTGGCTGTCAATTTCGTCACTGCTTCTGACAGCTGATCGTGATGAGAAGTTACGTGTATCGCGATTTCCAAATGCTACGATTATTGAAAGCTACTGCTTGGGCCATACTGCGAGTCTGACCAAACTGGCTTGCAGTTCTGTCACGCCAGAACTCGTCGTTACGACGTCCATGGACAACACGCTGAAGCTCTGGAATATGATGACGGGGAAGCTGGCGATTTGTCACAAGACGACATTGTGGCGGTGCTCGTAAACTACCAGTACGTGCGATTTTTCGAGATCGTAAGAAGCCGTTCCTGCAATTGTTTTCCATTTCAAAGGATGACTGACGTGTTCAACTGCTGTGCGTGATTTTCGCAAGCTTCAGAATGATCCACCATCGGGTGTCTCGGGTGCTCCCATGGACAACAATATTATGCTCTGGCAGGCTGTCATCTTTGGACCCGATGATACGCCATGGGAAGGCGGTACTTTTAATCTCACGCTCGAGTTTAGTGAAGATTATCCAAACAAGGCACCGACCGTCAAATTTATCACCAAGATGTTCCATCCAAACATCTATAACGACGGTCAGATCTGCTTGGACATTTTACAGAACCAATGGTCGCCGATTTACGACATTTCCGCCATCCTTACGTCCATTCAGTCCCTGCTATGCGATCCCAATCCGAACTCACCCGCTAACTCGGAAGCCGCACGTCTTTTTCAAGAAAATCGACGAGAATACAACCGGCGTGTCCGTGAGATTGTCGAGCAGAGCTGGCAGGCCATTGATGGTATCGGCTGCTAAAATGCGTGGTGCTGAGAACCGCATGAATGTCGGTCGCCCTTTTACTGCGTGGCTGGATAATGTCTCTTCGGACCGTGGCCTTTGTGGTGGTATCAATTCGGGTATTGCCAAGGCTGCTCGCAAGCAGACAAGTCACAGATCTTTGTCATTGGTGACAAAGCTCGTGCCCAATTACGTCGTGATCTTGGTACCCGTATCCGCGGCAATGTAACCGAGACGTTCCAGTCACCGCCAAACTTTACGGTAGCTAGTGCTATCGCAGAGGCTGTCGAATCGGAGAAGAGTCGTCGCGTATGGCTGCCATGGAGAACGCTACCACAAACGCTCAGGACCTCACGCTTACGCTCGTGTACAACAAGGCTCGCCAGGCCCGTATTACTACGGAGCTGATTGAAATTATTTCGGGCGCCGCCTCGCTGGACAGTGACTGGTAGCGTCGTGAACGTGCAAAATGTGACCCAATTTGACCAAGTGACTGCCCGGGATTCGACTTTTGCCGTGATTTTTTTCTGGGCGGAGTTTCAAGCTTGTCGTCCTAATGGACAATTGGATATCGGGTCGTACGATGGTGGATAAATTAGAAGGGGCTAATGTCGCTGAATTGGCGAAACGGGTCTTGAAGATCGTCTTAAGAAATTGATGAATGCTTCCTTTGTGATGCTCTTTATGAAGGGCACTCCAATGGAACCAAAATGTGGCTTTAGTCGACAGATTGTCACGTTATTGAACGATGAAAAGATTCAATTTGGAGCTTTTGATATTCTCAAGGACAATGACGTGCGTCAAGGGTTGAAACAATTGGTTGACTGGCCAACGTTTCCGCAACTTTATGTAAATGGTGAACTCATTGGTGGGCTGGATATTGTCAAGGAGATGAAGTCGGAAGGATCAATTGTCGAGCAATTAGGGAGGGAAGAAGTAGAAGCGACATTCCAAGAAAGTCTTTGTGCATTGATAAAGTCAGCACCTGTGCTGCTTTTTATGAAGGTTTGATATTTTGTATGGAGCTATCAACGCCAGGATCAAGATATCGCTAAGGTAGTGACTTTGATGCCATCATCGTTCCGCGAGGGTATGTGCGGAAGCCATCTCTTGGAGCCCGAAACCGGATGGCCATGGCATTTCGTGATCCTGTGAACGAAAAGGGAGAGTTCTGCATTGTGCAGCTTCCCGTATCACCTCGAAGTGCACTACATTACATCCAAGAGGTATTTCTACAATCGACTGGCAAGTGGACCGTGCTGCACCGCGACCATCGTAGTGAAGTTAAATCGCTTTGCGTTCATTCCAAGCGTGATTGCTTGTACACTGGTGGTCTTGATGGCCTGTTGGTCCGGTACGATTTGAACAAGGGGCGTACGGAAACCGTGTTGGAACGTCGTAAGCACATTTCGAAGATCAACTCGGTCCTAGAGCATCCACACAATCCGAATTTGCTTCTGGTCAGTTGTGTGGAGCAGTAGGCACAGCTTGTTGTTGCATGACCTTCAGCGATACGATGGCAATGTGTCGAGTGGCGCGATGAGTCAGTACGTCGTGCCGCGGTGGTCGCCTGCAGGCTATCACGTATCATGTGGTAGCAAATGCGTTACTGCCACAATAGCTTCTCTACGCCCACATCCGCCACTATTGGAGCTTCGTTCCTACAGAAGCGCGTGATCGTCGGAGGGAAACTGACGCTGCAGATCTGGGACACGGCCGGTCAGGAACGCTTTCGCTCTATGGCGCCCATGTATTATCGAAATGCAAAGGCAGCTATTTTGGTCTTTGATTTACAGAGTGAAGCTAGTTTTGAGAAAATCAAAGAGTGGCTGCACCTGCAACATCATGTGGGTGACGATATGTGGGCAACAAGAGCGACGTTTTGACTTTTCCAAGGCCCAGCAGCTCGCCGACGAGATCGGTGCTCTGGTGTTCCGCACGAGCGCCAAGACGGGTGAAGGAGTGCAGTCATTGTTTGAGAGCCTAGCGGTGCAGCTGCTGAAGAAGCATGACCAGGAGCAGCGGAGAGTTTCTACGTGGTGTATATACCGGTAGATGCGAGTAAAGATCTCGAAGAGTGGACAGTGAAGCTACCTGTAGACAAGGAGGCACAACTTGGCTGTTTAACGGAACGACTACGTGAGCATTTCAAAAGTGGAAACATCCAAGCAACAACAGAGACTTTTAAGCAGCAACTACTCACTCAATTGCCCAAGGGCGCTACACTTACCGATGAAATGTTGCAAATGATGCATGGACAGTCTTGTGGACTCGATCCCGCTTGTGCTCAACACGCCCGATGTGAAGCATGTTAGAAGTTAAGGGTGATGCATTTATTGGACGGGTATTTGATAGACGATTTCGAGCGTATGGACTTCCGATTGAGTGACGTATCAGGTGATGCACCGTGGGTGCAGACGGCAAAGCTGCAGAGATCACGCAGTCATTAGAGGAGATCCTGGCGCTAGGTCAAGGTTTAGATGATCAGGATTACAAGGCTGAGCTGGAAGTGCTACAGAATGATCCGAATTCGAATTTATATTCGGCCGTTACATTCGAAGAGCTCAATTTGCCCGAGAAACTGCTAAAAGGCGTATACAATATGAAGTTTGCGAAACCGTCCAAGATTCAATCTGTAGCACTGCCACTGATCTTGGCTGATCCGCCCGAGAACCTCATTGGTCAGGCACAAAGTGGTATCCTACTCGTGAACTTGTGAGACAAATTATGGCTGTAGTAAACGCCATGGGCAAATTGTCGAGACTTTTCTTGCTATTCCAGGCAATGACTTGGCTCGTGGAGAAAGTTTAAAGGCTCAAGTGATTGTGGGAACGCCTGGAAAAATTGAGGGGATGATTAAGAAGAAGCAACTGGACACTCGTGACGTGAAAATTTTTGTGCTGGATGAGGCAGATGTGATGGTGTCGGAGGACGGTATGCGCGAGCGCTCTGTGGCTATCAAGCGGCTGATCAAGAACCGTGCTTGTCAGTACCTTTTATTTTCGGCAACATACGCTGACGACGTGCGTGAATTTGCTCAGAAGATGGTACCGGACCACAATATTATTACGGTCAAGAAGGAGAAGCTGACTCTCGATGGCATTAAACAGTTCTGGATCGACTGCAAGACCCGTGACAACAAGTTTCAGGTGCTTTCGGACATTTTTGCTGTTTTGACTATCGGCAAGTGCGTTATTTTCGTCCAGCAGCGCGAGACCGCCAAGGAGTTGACGCGTCGTATGCGTGACAAGGGCCACATGGTTGGAATCCTGCACGGTGCTGACATGGCCAAGGAGGTACGCGACCAAGTCATCGACGAGTTCCGTGCTGGCACGACCAACGTACTGATTACCACTAATGTGCTGGCTCGTGGTATTGATGTCGCGGGTGTGTCGCTCGTCGTGAACTTTGATATCCCGCTCACACGAGACCACCGCCCGGATCCCGAAACGTACCTGCACCGTATCGGCCGTACGGGTCGTTTTGGCCGCAAGGGTTGTGCTATTAATTTCGTACACGACAACAAGAGTAAGCAGGACCTTGCAGAGATTGAAGAGTACTACGCGCGGCCCATTACACAGGCTCCCGCCGATGACATTGAGGAACTCGAGAAGTTGCTGGCTTGGGGGGTAGTAAGCGTCGATTGGCCAAGTTTGGTAAAGAACAGCGCAAGGGCATGCGAATTTATCCACGTGAACCAAAGAAAAAGGTCTCTGGGAAAGATAAAACGTATTATTACATTAAAGACATCATGTTCTTGGCCCATGAACCAGTTCTCGACAAGTTTCGAGCATTTAAAACGTTTATGAAAAAAGTGACGAAAGCAATGGGTCGAAAAAACTATGAAGATGCCAAGCGGAAACATGACAATCGACCTACTTATGAGCTGGATCATATTGTGAAAGAACGGTATCCACGCTTTATTGATGCTCTTGGAGATTTGGATGATGCCCTTTGTGGCAGAATTATATTGTCGTGACGAGAGGACTTACAAAGATCTTTGTAAGCATTAAGGGGATTTACTACCAGGCTAATGTACAAGGTCAAGTCATTACGTGGTTGGTGCCACATCAATTTACTCCTACAGTTGACAAACGAGTGGATGTAAGAGTTATGCTTACGTTCTTGGAGTTTTATGAAGTGCTACTGAAGTTTGTCAATGCTCAATTGTACATGCAAATTGGCGTAGCTTATCCTCCTAAAATCAATTTGCAGCTTGATTCAGCCGGTGCTCAACTTGCTGCGATTGAACTAGCAGCAAAAGGAATCTGAAAAGCGGATCAAGTTACAGCGAACAGCGTAAAGAGCGCAGTGAAAAGAGTCTGTTCAGTGGTCTGACCTTCTTCTTGTCTCGAGAAGTTCCTTCGGCTTGTTTGGAACTGGTGCTGCGCTCGCACGGCGCCGCTTTGGGCTGGGACGGCGCTGGATCTCCCTTTGATGAGAAAAACACGCGCATTACGCACCACGTAATGGATCGTCCTCACCAGGGTCACCGCTACTTCAATCGCGAGTATGTGCAGCCGCAGTGGGTGTTTGACTCAGTGAACAATGGCATTTTGCTTCCTCTGGCTCGCTACCGCAAGGAGCTGGAAAAGCTCAAATCGGCCAAGCAGGTGCTGCACGAGGTTGGTGCCGCTGGCGTCGAAGAGGAGGATGAGAGGTTTACGCTGCTGACCTCAAGGCTGAGGCGGATGGCAACAAGAAGACGAAGCGTGTCAAGAAGGAGGAGGAGGCCGCAGAGCTCAAGGAGCTAGCCAAGACCATGATGTCGAAGAAGGCCAAGCGTTTGTACGACCGTATGCAGTATGGTCTGGGCAAGAAGGCTGACAAAATTCAGAAGCTCGAGGACAAGCGTGTCAAGCTGGAGGCAGGACGAAAAAGCTTCTAGCAGGCGCACTGTCAGGAATGATCTCAGCTCTTGCCTTCCAGCCGCTGGGGCGTTACGCTGGCGTGGTACGTCACCCACGCTCGTGCGCGTTGCTGGCGGTGCCGGTCTTTACTTTGCCACGCTGGACTATTGTTTGAACGTGTTCCCGAGCTCTGCAGTCAACACGTTTTTAGCTGGAGCCTTATCACGAACATTTGCAGGAGGAATTGTATCACCTTTTACGATCGTGAAGGCGCGGATGGATACCTCCTGGCACGTTCGACAGTAAAGGGACGTGCCTTTCTCTGGACTCTATGTGCTCATTTACACGGCATCCGGCCGATGTCGTAAAAACACGCATGCATGCGTCAAACGGTGGTCAAGATCTACTGCGACGAAGGCCTGCACGGGTTTGCGAAGGGCATCGTTCCGCGCGTCGTCAAGCGAACACTCTCTACTGCAGTCACATGGACGATCTACGAGCTACTCGTTCGACCCATCGGGTTTGGGTCACGCGAAAGAAGCGTTTAATCTGGCCAAGGAGGCTGAACGCACCAAACAGTTTGAAAACCAGTCTAAAATTAAGGAAAACGAGGCTCTCTATAAGCAGTACGAGATCGTGCGCGTGCAAAAGGAGGGCGAGGAGCGCCGTAAGACGCTCGAGGAGGAGACGCGCCAGCACCAACAGCGTGCGCAATACCAAGATCAGCTTAAGCGTAAACAGTATGCAGATCAGTTGGCTGCTCAGAAGTACATGAAGGAACAGGAGTTAAAAAAACAGGAGGAGATCCTCGCCCGTCAAGAAGCTTCGCGTCGCAAAACGCTCGACTATGAGGCCGAGCTGCGTCAGAAAACAGAGCTTGCACGAGTGGCAGCTGAGACTGAAGGGCGCATTAAACAGGAACGTCTCAATCACGATCTGCATTTAGAAGAAGCCCGTGTGCGTGCCAAGGAATATCGCGAGACGGTCATGGAAGGTATCAAGCTGGCGGGTAACACTATTGGTTCCGGTGTCATGGCTTTTGTGGACGATAAGGAAAAACTCACGGCCACTGTGGTCTCTTTGACGGCTCTGGCTGTTGGTATTTATACCGCCAAGGTTTCTACTAGCGTTGCAGGTAAGTACATTGAAGCTCGAATGGGCAAGCCGTCGCTGGTTCGAGAGACTTCGCGTCGCTCGGCTGCTCAGGTAATCGCCAATCCCATTCCATCCATTAAGCGTGTGCTGCGTCTGCAGAAGGCGACAGATGCGCTGGAGGGTGTCGTGCTTGAGCCTAAGCTGGACGAGCGTCTGCGTAGTGTCGCTGTGTCGACGTTTAACACGAAAAAAAATCGCGCGCCGTTTCGCCACTTGCTGCTACACGGCCCACCCGGTACGGGTAAGACGCTGTTCGCCAAGGCTCTGGCACGTCACTCCGGCTTAGAGTATGCCATCCTTACGGGTGGTGATGTTGCCCCACTTGGTCGCGAGGGTGTGACAGAGATCCACAAGTTGTTCGACTGGGCTTCGCACAGCCGTCGCGGTCTGCTGTTGTTTGTTGATGAAGCTGATGCATTCCTGCAGAAGCGTAGCAACACTGTCATGAGTGAGGATATGCGCAACGCATTAAATGCCTTTCTGTACCGCACAGGTGAGGCCAGCGACAAGTTCATGATTGTGTTCGCCAGCAACCAGCCAGAACAGTTTGACTGGGCCATTAATGATCGTATCGATGAGATGGTGGAATTCCGCTTGCCTGGCTATGACGAGCGCGTACGCATGCTGAAGCAGTACTTTGATGACTACATCCGGGCTCCCAAGAACTCGCGTGCCAAGAAGATTTACGTGAGGGCGTGATCTTTACGGTGGACCCAGTCGCCAACTTTCTTGTGCCAAGACACGCATCTTCCAGCTTGACGCATTGCAGGGCGTGTCTGGCGAAGCTCAGGCTATCTTTGATGCTCTTAATAAGAGCCCATGCGCATTTATTGGATCGTCTTTACGGTCGTATTGGGCAATGGATTGTATACGTATTGTACGGGCAAGGACGAGTCATTTTTCGTTGAGAAAATCAAGAAAAAGATCCCGTCCGAGTACCGTATCTCTGAAAAATGGGACAAATGTCTTGAGAACTTTACACTTTATTTTGGTGCTGGTCTTGTGGCTGGTGGCCTCACGTCTCTTGTGCTCGCACGTTCTGGTGCTGGTCGTGGACTGATCACGGGTCTCGGTGTACGGGGGCTGGATCAAGCTGGACAACGTGTCAGATGGCGTTTGCAGGTGATGTACAGACAGCTCTGAAAAAGACGGAAAAGGCTGTGGATGATTTGCCGGCTTTCCAGCAACAGACAATCGTGGATTTTGTAACGGTTTCAGCCATACAACAGCAATTATTAGAAGTGCATACGGGGATTTTATGAAGAAATTCACGTGAAGATTCTAGAGACGTATCTCACTGAGCAAATGCAAAATTCCTTTGTCGACATTGATGCCGATTTGGCACTACTGAAGCTGTATCAACTATATCCGTCCACGTCCAATGTCGAGAGCCAATATTCTAATGAAAGGTGTCATGACGCTTCCCTCGACATTCTTTACGGGTGTCAACGATGATCCCAGAGAGTACAAGCGAGGATGTCAATGTGAAGGCAGCACTGCAGACAGGCTTTTTACTCCAGTCGTGTCTATTTGAGGATTTCTGGAAAATGGATCTGACGGTTGCGAACAAGGTGCAGGGATTTGTAGAGTCGGTCCGTGCTTACATTTTGACAGCCATCTCCCGTAGTCACAGTGTCATTTCGATCGACGTCTTGAAGGCCAAGCTCAATGTGTCGGACGAGGAAGTAGCTGAAATTGTCGCGGAGAAGTGGACTCGGGCAACCTGATCCAGATTACTCCAAACGAAGATAATCAGATGCAGGCCAAGAAGGTCCAGGAGAACATTGAGTTTGAGGACGTGCTCAAGGTCATTCACACCCTTTCTAGATCGAACGTCTGTGCCAGTGGCGGTGGTGTTGCAGACGCAGAGGAAGTCTGTGATAATCTAGCCAAGCACTTGAGACTGGACGGAGGGACCCAAGTGCTGCCCTGCTCTACGGGTGTCATTGGCTGGCGTATCCCAGTGGACGAGATGGTGGAAAACCTTCCCAAGCTTATCAAAAATTTGCAATCTGACTCGGTACTACCTGCTGCTGAAGGGATCATGACTACTGATTTGTACCCAAAGATCCGCTCTATTGACGTGTGTGGAGGTAGAATTGTCGGCATTGCCAAAGGGGCCGGGATGATTGAACCTGATATGGCTACGATGCTGTCGTATATTCTTACTGATCTTTCGATACCGCGTGATGTGTTGCGTCAACTGCTGACGGAGGTGGTTGGAGACACGTACAACTCGCTCAGTATCGATACGGACCAAAGCACGTCGGATACGGTGACACTCGTGTCGTCAGACCAGATCCCGTTTGACATGAA---GCAGCTGGACGAATTCAAGAAGGCGCTGCATAAGGTCTGCCTCGGACTGAGTGAAGATATTGTCCGCAATGGTGAGGGTGCACACCATGTCATGCGGGTGACCGTGAGTGGCGCGTTATCGAAAGACATGGCAAAAGGTGTCGGCAAGTCCGTGGTGAACTCGCCTCTGCTTAAGTGTGCAGTGGCTGGCAACGATCCGAATGTGGGCCGTCTTCTCATGGCTGTGGGCAAGTACATGGGCTTGCATCACAAGGATGTGAGCATTCAGCATCGTATGAAGATCCGAATGGGTGGTGTGGTCATTTTCGAAAATGGCGAGATGGTGCTGAATGATGATATTGAAGCCAAACTTGTGGCTCACATGCGTGGCGCGATGCTGATTGAGTCCACGCGGGGTGGACTTGATGCCAGCTCGCACAACTATCCGCCTCACGGGAAGTTTGTCGAGATTGAGATTGATCTGGGTGTTGGATCCGAGGAAACTCAAGTACTGGGTATCGACCTTACTCACGAGTACGTGGCCATCAACGCCGACTATCGATCGTAATGGCGCC---GAAAAAGGGGAAGACCGCTGCTGCTTATCTAGCGTCATTAGGCGTGACTTCGTC---CATTGGAGGTGAGAAAAAGGACGAC---AAGAAGAAGAAGAAAAAGGGCAAAAAGAAATCCAAAAGAGACCCATGTCAGCTGCAGCCAAGGCGTTAA-TGGAGCGACAGACGGCCTTGAAAGCCGAGCAGGAACGTCTAGAGAAAGAACGTCTAGAGCAAGAACGTCTTGCGCAGGAAGAGGAAGCTAAAAGACTGGCGGAAATTAAGGCGGAGGAGGAGATTCGCCGCAAGAAGAAGGAGGCACGCGCTGCCAAGATTGAACGGCAGAAGAAGGAAGGCACGTATATGACTAAAGCCCAAAAGCAAAAGGCAGCAAAGGCTCAAGCGGCATTGGAGGCCATGCAACAGGCGGGTCTCGTGCCGGAATATGCT------GGTGAGAAGAAGACGGTCGTGTATATCGATAACAAAAAGAAGAACAAGAAGAGCAGGAGGCAAAACAGGAGGAGGCCGAAGCCCCAGACGCGTGGGATGACGAGGATTGGGAGGCCAGCGATGTCGTGGCCTCGCTAGAAGATAAGCTAGCAGAAGAAGGAGCAGGAGCGTCTGCGTCAGCAGGGTATTGCACTAAAGAAGCGTCAGGAGGAGCAGGCCGAGCAGATGCGTCTGGAGGCTGAGGAGCAACATCAGCGCTCCAAGGCTAAACTCGAAGCTGAGCGCAAAAAGAAGGAGGCTGCCGACCGTCGTCGCGACCGTGAGGCCGCCGCACGAGCCAATGTGTCGCTGGACAATCTGCGCTCGCCTATTTGCTGTATCATGGGTCACGTCGATACTGGTAAGACGAAGTTGCTCGACAATATCCGCAAAACCAAGGTGCAGGATGCTGAAGCTGGCGGTATTACCCAACAAATTGGTGCGACCTTCTTCCCTGTAGAGGCTATAAAACAGAAGACGGCTCGGCTTAACGAGACAATGAAGCTGGATTTCCGGCTGCCTGGTCTGCTTGTGATCGATACACCCGGTCACGAATCGTTCACGAATCTGCGTAATCGTGGCTCATCACTCTGTGATATCGCTATTTTGGTGGTTGATATAATGCACGGTCTGGAGCCACAGACTCTGGAATCACTGCGCCTGCTGCGCCAAAAGAAGGCACCATTCGTTGTGGCGCTTAACAAGATCGATCGTTGCTATGGCTGGAAGACGATGCCCGACATGCCCGTTCAGGAGGCACTTAAGCACCAGAATGAGCACGTCATTCGCGAGTTTGAGGACCGCATGAAAGCAATTATTGTGGAGTTTGCTGAGCAGAAGTTGAACGCCGAGGTCTACTGGCGCAATAAAGACTTGGCGCACACGGTCTCGCTGATCCCTACCAGTGCTATTTCGGGTGAGGGTGTGCCGGATCTGCTGATGATGCTGATTCGTTTGACGCAGGAGCGTATGGCAAAGTCATTGGCGTTCGTCGATATCTTGCAGTGTACGGTGTTAGAAGTGAAGGTGATCGAGGGTCTCGGTACGACCATTGATACGATTTTAGTGAATGGTACACTGGAAGAAGGCACCACTATTGTTGTGTGTACATTAGATGGGCCTGTTGTGACAACTATTCGCTCATTACTGACTCCGCACCCTATGAAAGAAATTCGCGTTAAAGGCGATTATATCCACCATCAAAAGATGAAGGCTGCTATGGGTGTGAAGATTTGCGCGCAGGGTCTTGAGAAAGCTGTTGCCGGTACACAGATTCACGTGGTTGGCCCAGATGATGATGTCGAGGAGCTAAAAGATTCTGTCATGTCCGACCTGACAAGCATTTTGGACTCGGTGAAGGCTACCCGCCGCGGTGTATTGGTTCAGGCTTCTACTCTTGGCGCTCTGGAAGCGCTTCTGGAGTTCTTGCGGACGTGTGACCCGCCCATTCCGGTGTCCTGTGTTAACATTGGTCCAGTACACAAGAAGGATGTTATGCGAGCTTCTGTGCAGCTCGAGCATCAGCCCGAATTCGCTACAATTTTGGCATTTGACGTGAAGGTCCACAATGACGCCACTGAGCTGGCATCCGAGCTTGGCGTGCGCATTTTCACGGCTGACATTATTTACCACTTGTTCGATCAGTTTACGGCGTACATGGACAACTTCCGTATGGCACGCCGTGAAGAATTTGAAGAGACCGCTGTGTTTCCCTGCGTGCTCAAGATCCTTCCCGAGTGCGTTTTCAACAAGAAGGATCCCATCATTCTCGGTGTCGACATTGAAGAGGGTATCCTTAAGGTGGGTACGCCTCTCGTGGTGCCGTCTGCTGGTG---GGTTTCTGGTTGGCAAAGTGGGATCCATCGAACGTGAGCACAAGGAGGTGGACCGCGCTAAGAAGGGCGCATCGGTGGCCGTTCGGATAGACAACGAAGGCAGCGTCATGTACGGCCGTCACTTTGACCACAAAAACAAGCTGGTGTCCCGCTTGACCCGTCAGAGTATTGACGCACTGAAAGAGAATTTTCGAGAAGATCTTCAGAAAGAAGACTGGCAGCTCGTCATTAAGCTCAAGGCGGTGTTTGATATCATTATGGAAGGATGGCTTACAAAACGTCGTGATCATATGAACTTGATTTGGCGGGATCGCTATTTTCTTTTGGATTGTAACCAGTTACGATATTATCGTAAACGTGGGGATCCAGCACCTCGGGGTACATATATCCTAACCGATGGATGTATTGTGAGTCCTGTCTTCAATTCCGAAGAAAAACATAAACATCATGGCCCCGTGTGGATGTTTCGGATTACATGAAAAACAAGCCAAAAAAGACCGATTTTTAGATTTGGGAGCCAAAAGTGAAGAAAATGCTAAAAATTGGAAAAAAGCCTTGGAAACAGCTGTTCGATCGTTGCAGGAATTGGTGCTCCATCAACGAAAACTCAAATCAGATTCCGAGAA---TTTTCCAACCCCGACATAT---------GTTCCACGGATGCTCCAAGCTGATGAAGACACAATGTCTTTATTTAATTTAGATGCTGATAATGTCAAGGCTGAACAAGTGTGGTGGTTGGCTCGAGTGGAAGGAGGATTACGGATCATGCAGGAATGTCCATTGGGACCAAATGTTGCTGCAACGTTGTGTTTTCAACATGCAACGACGACAAATGGATTGATTTGTAGTGCTGTCCTGGGAGCAGCATTGAGTTTTAGTATCATGTATTCCTTTGCTATGAATATGTTGCTAAGTTTCATGACGGCTATGATGGCTATTGGTGTTTGTTGTGTAGCAGTGATGCCAACTCAAACACCAGAGATTTCAAGCTTTCGGGTATCACAAGTTGTACATGGATCACCGACTGAAGTGTTTCGACTCATTATGAATTCCAAACGATTTCAACGATGGGATGCTGCAACAGCAACGATGCGTGTTGTGCAGCAATTAGATGATCATGCTGATATTGTCTATGTGACACAACGACCGACGTACTTGTGGCCATTATGGCAAAAAGCTCGTGATCTTGTCTTTATGCGCTATTGGCGACGTGAAGAAGACGGATCGTACTTTGTCATGTACCAGAGCATGGACCATCCCGAGTGTCGGGTGCGTCACAATTACGTACGAGCAAACATTCTCGGTGGCGGCTTTGTTATTGCTCCCCAACGTGTTCCAAGT---GGAACCATTCGTACGCTCGTCACTTATGTGCTCCGATACGATCCTGGTGGTTG---GTCGCGCATTTACCACCAGCTCGGTATGGATGTGGACTTGGTCATGCCTCAGCTGCGCAGTGTTGTGGGCATTCGCGATGAAATGAGCGCCACTGACTTCATCACACCCAACGTCTCGATGGCGTCAGCTGGAGAAGGCAGAAGCTGGTGAGGAAGCGTAGA---CTCTATGGGTGTTGTCATGGGTGTGCAGAAGCTTCAG---ACTAGTTTGCCAGAGAAGATGTGGGCCGAGCCTGACGCCTCTCTTTTCAGTGTGCGTGGCCACAATTACCTAAACGACAAGAAGAAGATTCCGTCAGCACCGGCAATGTTTCACACTGTCGGTGTGGATTTATTGTCCTTCAGCAGTGCAGCCGAGAGATACAACATTTCGTCTCGTGCGGACAGTGTTGGTCGTACTAG---CACCAAGTTTACGTTTGTTGTGAACATGATCATCCCGGGTACCGAAAACACGTGCATGGTGTTTTACTTTCATCCTGCGCGTGATAATGTGTTCGAAGACGGCTCACCTTTTTCCGAGCTGCTGAACGACTTCTTTGATGGCGACGATGAATTCCGGAACTCGCGCTTCAAACTCATTCCGACCGTGGAGGAGGGTAGCTTCATTATCAAGCAGTCGGTCGGTTCAAAGCCGACACTCCTGGGCAACAAGTTGAAGTGCCTTTACTACCGTGGAGACAACTACTTTGAAGTGGACATTGACATTTCATCGAACTCGGTTGCTAACACAGTCGTGGGCATGGTGCAAGGTGTTACCAAATCGCTGGTGGTAGACATGGCATTCCTGCTCGAGGCTCAATCCGACGAAGAGCTGCCTGAAATTATCCTTGGTGCCGTGCGTCTGCAACACGTTTCTCTTGACAACCCGCATCGCATGCCACAGCATGGGGAATGGACAGCAAAAATGAGATTTACGTTCGAAGTCTTGACGGAAGCTCTACGTGTGAATCTGCAACGGTTGATCGTCTTTGCGTGCTCTGCGGATACGACTTTGCAACGAGAAATTGCCGAGAAATTGGCCAATGAGGCTGTCAAGATCGACAAGTACAAATAGTCGAATTGGACGGACTGCAGCTGCTGCTACCATTAACCAAGTCAAAGGATGCAGAGGTGCAGCGTCTAGCAGCACATGCTCTTGCCAACCTCTCCGTCAATTCTGAGAATCAGTCTAAAATGGCCACGGAAGGAGGCATTGATATGCTAATTGACCTCTTAAGTAGTTCAAACGAACACGTGCAGCGTCAAGCTGCAAAGGCACTTGCTAATCTTGGCGTGAACGTGGACAACAAGGAACGAATCGCTAAGGCGGGTGGCATTAAACCGCTTATTGACTTGGCTAACTCGCACCAGATCGGAGTCGCTGTCGAGGCCATTGCAGCACTTGCAAATTTGGCTGTCAATG---ACGCCAATGAGGTGGAAATTGCACGAAAAGGAGGCCTGAAGCCTATTATTGACGGAGCGCACTCGGAATCTATTGAACTCCAGTCGCAGGTGGCACGTGCACTTCGTAATCTGTCTGTCAATCCCGAGAACAAACAAGCGATTGTGGACTTAGGTGGGGTAGAAGCTCTGCAGTCACTTGTCCGTTCTATCAACGATCGGATATGCCAGCAGGCTACACGTGCGCTCGTTCCTACTGTGGCCCTCACGCGCTCATCACCTACAATCTAGTG---GTCGTCACGCCCGTCGCTCGTGTTGCTCAAGTGAAGAAGGAATA------TCTGCCGTATGATGACAACTCATTGGATGTCGAGTACATTAAGACAATTCCAACGGTT---------CAAGTTCCTTCAGTAATGACGG---ACAAGACAAGTCGT---GGCTTTGTGTCGATTTCCGACCGCTTTCCACTCTCGAAAGTGGGAAAAGCTCGTTGTAGCATAGCCCCTTTCTCAATAAGGCG---TTTGCTAAGTG---CTACGCCCGAGTTAACAC---TGGTGGATCAG-------------ATTACAGTGCTT---GAAAACGATACAGTGAACGATAAT---ACGATCGTTGCTGAAAAGCAAGAGAATGAGTGACGCGTACCGGCATCAAGCTACGGA--CGTGGAAGAAATTGAGATGGAGCAACTTCGATGGAATATCCAAAAACAGGTTTGCCAGCGATATTTCCAGCAGGAACAAAAGTATGAAGAATGGGGAAAATTTGACGAGCAAGACACCGAGTATTCTTCGTTCCAGGTGGAAGACGAGCAAATATATTGGCCGAGAAGAAGTTTCATGACAATGCTGTTCGAGACGCTATGGGAAAGGGAAATGCTACGTGGGTGAAGGAGCGACGAACTCGGTTTGAGGAGGAAATTCGTCAGTTCTAAGAAGAAACGAGACACAAACAAGAGTTAATGCTTACACGTCTTGAAAAAGTGTCACCATTTTCGAAGTTTCCCATTCTTGGCAATCGTTTTTTGCTGCTTCGTCTACGAGGAAAGGGAGGCAACGGTGAAGTATGGGATGTTGTTGATTACGCCAACAATAAACAGGAGTGCGCTCTCAAGCTGTCGACTTCAATTCGCCACGCTCAACGCGAACACAACACGCACACTTCATCACCCCA--ACATTGTCCAGGTCGGCGACGTGCCCTTCCTCATTCGATACGAGCAGCAGCAGTACACAGCGTTTTCTGTTGCAAGTGTTCAATCGGACTTGCAGCAACTTATCGAGA---TGTACGAGTACTTGGATGATGATTCTGCATACAAGGTGCTTTCGCAGCTGCTGAGCTCGCTGAAGTATTTGCACGATGGGCCTTGCTCACTATGACCTGAAGCCGTCGAACATTCTCATCGACCACGACGGGTGTGTGAAGCTG------ACTGATTTTGGCCTGACGCGCATTGCTAAGGCAGCTACGTCGAGCTCTAGGGTTGGCACTCTGCGGTACCTTCCTCCCGAGTGTTTCCGTCCAACCCGAGTCGA---------TTGCGAAGCTACGGCGGAGAAGGCGGACATGTGGATGGTAGGGATTGTATACTGCATGATGGTCACTGGGAAACACCCCATCTATTCTAACAAATTGACGCAAGCGGAAGTGGAGGCAGTGATGGGTAAGTACACTGGC---GAGGTCCAATATGCGCGGCCAGTTTGCGATCTAACCCGGTGGATCTTACAAGGGTGCCTTCACCCGGACCCAAGATGCCGACCAACGGCCGCGCAATTGCTGGTGGCACTCCAG

Peronospora_matthiolae TGGACTACCACCGCCAAGTCGCTCGCGAACTCGTGACTTTCTTCCACGCTTTCTACCGCTTCGGGGCAGTCTCCAACCGCTTCGATTTGTTTCTTCAGAACCTCCTGCTCGGGTCGCTGATCATTGGCGTCGTCGTGTTTACGTTTCTCTGCGTCCTCGTTGTGCGCCGCACAATTCTGCACATTCAAAGACTCAAAAATGCCGCATGGAAATGGACACGACGTCAATGGAGCTGCTGGCAGTCGCGTTGCTGAGTGTGTTGGCGATTAGCGCCCTGGGGGGACTATTGGGAGAAGCTGTCTGCACACGAGTGACGCTTTGCGGATCAATGCTGACAATTTGATCACGTCATTTGACGAGATCGAATTGCCAGCTGAGGCCTTTAAGTTATCGATTGAAGCGCTGGACTCGTGCACGCATCGAAAGATCTGTACAACCAATCGGATGCGATGCTACCGAAGAACTACTTGGATATGGCCAATGACTGGCAGCTGAGCTACTTTATGCTGGAGTCGTCGACAAATGGTGCTATTCTGGCTGTTGTGCTGGCCAGCTTCTTGTCCATCGCGTCTGTTGGGTGGGCAATGGTCTCACCCCTGCGTCTATCGATTCTAGTGATCTTAAGTGTCGTGCCGGTTTCGCACACGCTTGTTGGCGCCTACTTGTCATCGACGATCATGGTGGCGGACTTTTGTGCTGCGCCGATGAACAATACGCTCGAACTCGTGGGCTCATGCCCTGCAAATGCTTCCTTCCTTTTGCTGACGCCTTGGCTGCACAAAGCTTCTCACCAAGGAGACGTTGGACGTCGCATGAAAAAAGGCTTTCTGGATCCGATCGAGCCAAATTTGGGCGTCATGCTCGGCTACTTTATCGGTACCGTGAGCAACTGGGAGAGTTCCCCGCACGTCCAGACGTGGCGCTGGCCCTTTCTGCTGCAGTTTCTAGCACTGCTGCCGGTCAACATGGCGATCTTCCTCGTGCCTTCGAAGCACTTGATCATCCGCAGTGAGACCTTCGAGCTCGAGCCACCAGCGGCGGGCACGCCAATGAGTTCGAGGTCGACGACATACCATGACGAAGAAGACGCATTGCTTGCTGGTGCGAGTATGCATCCTATGGCGCCTTGGATACGAGGTGTATGAGCAAGGCGCGTTTTCGGACGGCTTACGCGAGCTGTTGCGCATACCTGTGTTCTGTCTCATTGTGTTCGGCCTCACGACGGTGTACTTTGTCGTCACGGGCGTGCAGTACTGGAGCACCATCTTCATGATCAAGTCACTGCACGCATCAAAGTACCTCGTCAATGCGCTGTTCGTCGTCGTTTCCGGTACAGGACCCATTCTCGGCGTATTCTTCGGTGGGTGGCTCATTGACCGATACGGAGGATACAGAGGTGTGGCACAGCGTGCAAAGGCTCTGGGAATCTGCATGATTCTCGGGCTGACAGCCTTTTTTATCTCGGCGGTGACGACGTTCTTCAACGACGTCTTCATTACCTCTGGCTTCTTGTGGCTGCTGCTCTTCTTTGGCGGCTCGATTCTGCCAGCACATCGCGCGATCGCTTCGTCATTCTCCGTGATGGTGTTCAATTTATTCGGTTACTCTCTGTCGCATGAGCGACCTCCAGATGGTCGTCGGTGCCAAGTGCTTCGTCCCTGACGAGACGCACGTGTGGCTGGCCGCCCAAGTCCTGCGGGACGAGAACTCGGGCGACGGCAAGACGCGCAAGATCTACTGCACAGTGGAGCTCCCCGACGGGCTCGGAAGAGCGCTGCGTAGACATGCTGGCCAAGAAGACCAAGGCGCTGCTGCCGTGCACGAGCTCGAGACGCTGCCGTATCAGAACGAGAACGTCGGGCCGGAGGGCATTGAAGACATGATTACGCTCAATTACCTGCACGAGGCGGCGATTCTGTACAACATTAAAAAGCGCTTTTTATGTGAGCTCCCGTATACGTATACCGGCGACATTTGCATCGCTATCAACCCGTACAAGTGGCTCCCGGAGCTCTACGCCGAAGAGCAGCACCGACGGTACTTGAACCAACCCAAGGAAGAGCTCCCGCCCCACGTGTATGCGACGTCTGTCGCTGCTTATGACAACATGCGACGCTCTGAGTGGAACCAGTCGATCCTTGTGAGTGGCGAGTCGGGAGCGGGCAAGACCGAGACGACCAAGATCCTCATGAACCACCTCGCGACGATCGCGGGCGGCATGAACAACTCGACGATCAAGCGCATTATTGAAGTCAATCCGCTGCTCGAGTCGTTTGGCAATGCCAAGACCGTCCGCAACGACAACTCGAGTCGATTCGGCAAGTTTACACAGCTCCAGTTTGACCAGAGGGGCACGCTCGTGGGCGCCAAGTGTCGCACGTATCTCTTGGAGAAGACACGGGTGATCCAGCACGAGGCCCCGGAGCGCAATTACCATATCTTTTACCAGTGGACTCGCCCGATATGGCGGCAGAGCTCCAGCTCGACTCGACGAAACATTATGTGTACACGGGCAACGACACGGCGCAAAAGATTGAAGGATTGTCGGACGAGAAGCACTTTATCAAGACACGAGAGGCACTGGCGCTCGTGGGACTCTCACACGAAGAGCAGCGGCCACTGTTTGAAGTGCTGGCGGGCGTGCTGCACCTTGGTGAAGTGCAGTTGCTATCCGACGCATCGGACGATGAGAAATCGAATATCGCTGTGGGTGATTGTGGCGCGATCTCGGCGACTGCGATGCTGGGCGTCACGTGGGGAGCACTCGAGACTGCGTTGTGTTCGCGTACCATGCGGGCGGTGAACGATGTGTACAGTGTGCCGCTGAAGAAAGAGTTGGCGATGAACTGTCGTGATGCCCTGGCTAAGGCTATTTACTCGAATGTGTTCGACTGGCTCGTGACGACAATCAATCACTCTCTCGCTGATGACAAGCATATGTCGAATCACGTCGGCGTGCTCGATATTTTCGGTTTCGAGCATTTCAAGCACAACTCGTTCGAGCAGTTTTGCATTAATTACGCTAACGAGAAGCTGCAGCAGAAGTTTACTCAGGATGTGTTCAAGACTGTCCAGATTGAGTACGAGGAAGAAGGTATCGTGTGGGACCACATTGAGTATGCGGACAACCAAGATGTGCTGACCGTGATTGAGTCGCGCATGGGTATTATTTCCTTACTGAACGAAGAGGTGATGCGACCGAAGGGTAGCGAGGAGTCGTTCATGTCCAAGGTGACGTCTCTGCACAAGGAT---GACATGACGCACGTGATTGAGTTTCCCCGCACGTCTCGTACCGAGTTCCTTATCAAGCACTATGCTGCTCCAGTCATGTATGATTCGGTGGGCTTTCTGGAAAAACATAAGGATTCGCTGCTGCCTGATCTATCCGAGCTCATGCGTGGTTCGTCAAAGCCATTCATCGCGGAGCTCTTTGAACCGAAGCCGGAGCCCAAGTCGCCGGAAGC---------GTGGCGGGGCTTTGTCTATCACGACTGTCGGTACTCAGTTCAAAGAGAGCTTAACAGAGCTGATGGCGACGATCAACTCCACCCAAGTTCACTACGTGCGTTGCATCAAGCCCAACCCGATCAA---------AAGCTCCACAGTCATGGATCAGTCCATGGTGGTCTCTCAACTGCGATGTGCAGGTGTAATTGAAGCTATTCGTATCTCACGTGCAGCGTACCCGAATCGGTTGCAGCACACAGAGATTCTGGATAAGTTTTGGCTCTTTGTACCAAGCGGTGGCAACACTCCGGTCGAAAAATGTCAACTACTCATGGACAAGCTGAAAGTAGAGTCTCCAGTGCAATACCAGATGGGTAAGACCCGGGTGTACTTCCAATTGGGTGTGCTTGAAGAGCTCGAAGATCGTCGTAAGAAATTTCTGGATGCGAAGGCAACGTACTTGCAAAATGTCATGCTTGGCTTCACGCAGCGTATCAAGTACCTCCGACAACTGGCTGCGATTATCAAGCTCAGTTATCCGATGTGTGATTGCTATGCGAAGGTACAACACATTTATGAACGGCCTCATTTTGATGCAGGCTCACTGGCGAGGTAACCAAGGTCGAAAGGTAGCTGCAGAAGTCAAGAGCAACCACTACGCTGTCATCATTCAGCGGTATGTGCGCGGCTTTGTCAAGCGTCATAGTTACGTCAACATGCGGGCGATGGTCATTCGTGTTCAAGCCGTGGTTCGAATGACGATCCAGAGGCCAAAATACCTCGCTGCACTCGACGAGAAACGTCGGGAGGCTGATATGGCGTACCAACTGAATAAGTTGAAGGCTGCTCTGCAGGAAGAGCAAGAAAGGAATGTGCAGCT------ACAACGTC---GTAGTAGTGTGAC--GACGGCGCAACTGCCGCCTCGAGCGTGGTGATGGCTGACGCTGGTGGCATGATTGAAACTCTTACGGACGAGAATAAGAAGTTGCGTGAGAAGAATGTAGAAATGAAGGTTGCGATGAAGGGTTTGAAGGTCGAGATTGAAAAATTCAAGAGTGACAAAGAATTCTCGTCGGCTGGCAATCACGTCAAGCTTCGTCAGCTGCAGGACACGGTGCGCGAGAAAGACAAGAAGATCAAGTTGCTCGAGGCCGATAACAAGAAGCTCGTGGAGCAACTTGCAAAGATTTACGTGGACGG---TGT---GCCGGAGAAGAAGTCAACGCAGAAGAAGTCCATCTTCCGTACGCTGGGGTCCAAGAAGGAGAAGAAGCCTC---------------GTGAGACCGTGCTGATGGACTCGCTCACTGGTTCGGATGACCTTAACAGTCGGGAGACGCGTCAGTCAGA---GCGCCATTTCCCTCGCATGC---CAACAATGAGCGTCGGCACC---CGCTTCTGGAAT---AACAAG---AAGGGTGGT------------AACGGCGACGGCTTTGACGTTTCGGAGGTTGAAGGTGAGGATAGCCCGACGGACCTGCGCGCAACACGATCGTGAAAAGCGTCGAGGTGGGTGTGACGGGGGCCATGTCGAGTCTGAAGGGTCGCATG---------TCGGTCGTGAAGGGCATGTATGAAGCAAGCGGACGAACAGGATCGCCCTGTGCGCGAGTCGTTTAACCTGGATGCGCTGCCCGAGGTATCCCTGCCCGTTGGCTGGGAGGCCAAGGTGTCCCGCTCAACGGGCCGCGTGTACTATGTGAACCGCAAGCTCGGCAAGTCTCAGTTCGAGCGCCCTACACTAGCTTCGGTGAA------GGCCCAGAAGCTGGCGCGTCAGAAGTCAGCGAACGGGGTGTAACGCAGTCGTGGCTGATTTCGGCTGTGGGGATGCAAGGTTGGCTGAGAGCGTGCCCAACAAAGTCCACTCGTTTGATCTAGTGTCCCTCGACATTGGCGTGTACTGTTTGGCACTCATGGGAACGAGTGTCCGCGAGTACGTACGGGAAGTGTACCGTGTGCTAAAACCTGGAGGTGTGTTGAAGATTGCAGAGGTGAAAAGCCGTTTTGAGAGTGAGACACTGGGCGGATGGGTCTAGTCTTGATCGCATCTGCGCACGAGATCGTCGTTACTGTGCGCAATACGACGGTGTTCGTCAACAGATGAAGTACGCAGTCATCTTAGGCCAAAAAACAGGCGAGGCTGTGGCTGTGCCTTTTCCGGACGTAGACCGAGACCTCCGGATGCTGCTGGGCCACACGACGTCGATGATCACCCACGTAGCTGTAAATCTCGTCACTGCTACTGACGGCTGATCGGGATGAAAAGCTGCGCGTGTCAAGGTTCCCGAATGCTGCTATTATTGAGAGCTACTGCCTGGGCCATAAGGCTAGTCTCACCAAGGTGGCCTGTAGTGCAGTCACGCCGGACCTCGTCGTGTCGACGTCCATGGACAATACGATAAAGTTATGGAACATGGGAATAGGAAAGTTAGCGATTTGTCCCAAGACGACGTTGTGGCTGTGCTTGTGAACTTTCGACACGTGCGATTCTTCGGAATCGTGAGGACCCGTTTTTGCAGTTGTTCTCCATGTCAAATGGCACCTGATGTGTTTAACCGCTGTGCGTGACTTTCGCAAGTTGCAGAACGACCCGCCGTCGGGCGTCTCGGGCGCTCCCATGGACAACAATATCATGCTCTGGCAGGCCGTTATTTTTGGACCGGACGATACACCGTGGGAAGGTGGGACGTTTAATCTGACGCTCGAGTTTAGTGAAGACTATCCGAACAAGGCGCCGACTGTCAAATTTATCACGAAAATGTTCCATCCAAACATCTACAACGACGGTCAGATTTGTCTGGACATTTTGCAGAACCAGTGGTCGCCCATCTACGACATCTCGGCGATCCTCACGTCCATCCAGTCGCTGCTGTGCGACCCGAACCCGAACTCGCCGGCAAACTCGGAGGCCGCGCGACTTTTCCAAGAAAATCGACGGGAGTACAATCGCCGCGTGCGCGAGATCGTCGAACAGAGTTGGCAGGCGATCGATGGTGTCAGCTGCTAAAATGCGCGGCGCTGAGAACCGCATGAACGCCGGTCGTCCATTTACAGCCTGGCTGGACAACGTGTCGTCCGATCGTGGTCTCTGTGGAGGCATCAACTCGGGCATTGCCAAGACGGCTCGCAAACAGACCAGTCGCAGATCTTTGTCATTGGAGACAAGGCCCGTGCCCAGCTGCGTCGTGACCTGGGCGAGCGCATCCGTGGCAACGTGACGGAGACGTACCAGTCGCCACCGAACTTTACCGTGGCCAGTGCCGTTGCCGAGGCTGTCGAGTCGGAGAAGAGTCGTCGCGTATGGCGGCTATGGAAAACGCCACCACGAATGCGGAGGACCTCACGCTCACGCTCGTGTACAACAAGGCTCGCCAGGCTCGTATTACCACGGAGCTCATTGAGATCATTTCGGGTGCCGCTTCGTTGGACCGTCACTGGCAGTGTCACCACCGTCCAGAGCATCGCGCAGTTCGACGACGCCGTCGCACGTGACTCCACGCTCTCGGTCACGTATTTCTACGCCGAATTCCACGCCTGTCGTCCAAACGGCCAGCTCGATGTGGGGCCCCGTGGTACTCGAGAAACTCGAGGGTACAAACGTCGCCGTGCTCGCTAAACGTGTCGACAGGATCGACTGACGAAACTCCTGGATGCCGCGCCGGTGATGCTGTTTCTAAAGGGCACGCCCAGCGAGCCCAAGTGTGACGCTAGTCGAGATGTTGTCGCCCTACTGAATGAAGCGGAGATTGCGTTCAGTACGTTTGACGTGCTCCAGGACCAAGACGTGCAAGAAGGACTAGCGCTCGTGAGCAAGTCGTCGACGTTCCCGCAGCTTTATGTGAACAGCTCGTTCGTTGGGGGGCTCGAGGAGCTCAAGGAGATCAAGGCCAAAGGATCGATTGCCGACCACATTGGACTGGAGCCAACCGATGCAGCGTTCCAAGCGAGTCTGCGTGCTTTGGTGAACTCGGCCCCGGTGTTGCTGTTTATGAAGTTTTGATATCTTGTGTGGAGTTACCAGCGTCAAGACCAAGATATCGCCAAGGTAGTGATGCTGACACCGTCGTCGTTTCGGCGGAGTATGTGCAGAAGCTATTTCATGGAGTCCGAAACCGCATGGCCATGGCATTTCGTGATCCTCTGGAAGAGAAGGGGGAATTTTGTATCGTGCAGCTCCCCGTATCACTTCGAAGTGCACCACGTTACATCCCCGTGGTATTTCGACGATTGGCTGGTAAGTGGAGTGTGTTGCACCGCGACCATCGCAGTGAAGTGAAATCGCTTTGTATCCATTCCAAGCGGGACTGTGTGTACACGGGTGGCTTTGATGGCCTCGTGATACGATACGACGTGAACAAGGGACGGACGGAAACAGTGATGGAGCGCCGGAAGCCCATCTGCAAGATCAACTCGATCCTCGAGCATCCACACAACCCGAATTTGCTTCTGGTCAGCAGCGTGGAGCAGTCAGCACAACTTGTTGCTGCATGACCTTCAGCGATATGACAGTAGAGTGTCGGGCAGCTCGATGAGTCAGTATGTCGTACCGCGCTGGTCACCAGCGGGTTATCACGTCTCGTGTGGCAGCAAATGCGCTATTGCCACGGCAGCTTCTCCACGCCCACGTCCGCTACAATTGGCGCGTCGTTTCTACAGAAACGGGTCATTGTCAACGGGAAATTGACGCTCCAGATTTGGGACACGGCTGGTCAAGAGCGGTTTCGGTCCATGGCGCCGCTGTACTATCGGAATGCCAAGGCGGCGATCCTCGTGTTTGATCTGCAGAGTGAAGCGAGTTTCGAGAAGATTCAAGAGTGGCTGCATTTGCAGCATTACGTGGACGATGATATGTGGGTAACAAGAGCGACGTTTTGACTTTCGTAAAGCGCAAATGTTTGCAGACAAGATTGGAGCTCGAGTGTTTCGCACAAGTGCCAAGACGGGGGAAGGCGTGCAGGCACTTTTTGAGAGTTTAGCAGTGCAGCTGCTGAAGAAACACGATCAGGAGCAGCGGAGAGTTTCTACGTGGTGTATGTGCCGGCAGATGAGACCAAGGAGCTGGAAGAATGGGCAGTCAAGCTCCCCGGTGACAAGGAGGCGCAGCTGGGCTGTTTAACCGAGCGACTTCGAGTGCATTTCAAGGGAAACTCTATCCAAGCAGCAGCAGAGACGTTCAAACAGCAACTACTGGCCCAAATGCCAGAGGGTGCGACGCTCACGGATGAGATGTTTCAAATGGTACATGGACAGTCTCGTGGACTCCATTCCACTCGTACTTAACACGCCGGGCGTCAAGCACGTTAGAAGTCAAAGGCGATGCATTCATCGGCCGCGTATTTGATAGACGATTTCATCAGGATGGATTTCCGACTCGGTGAGATATCAGGTGATGCCCCATGGGTGCAGACGGCAAAGTCGCAGAGGTTACACAGTCGCTCGAGGAAATCCTAGCACTTGGACAAGGGCTGGACGACCAGGACTACAAGGCCGAGCTCGAAGTGCTACAGAACGATCCGAATTCGAATCTGTACTCGGCAGTAACGTTCGAAGAGCTGAATTTACCGGAGCCGTTGCTCAAGGGCGTGTACAACATGAAGTTTGCCAAGCCGTCCAAGATCCAGTCGGTGGCGTTGCCTTTGATCTTGGCTGATCCACCCGAGAACCTTATTGGTCAGGCACAAAGTGGCATCCCACTCGTGAGCTGGTTCGGCAGATTATGGCTGTGGTAAACGCCATGGGCAAATTGTCGAGACGTTTTTGGCAATCCCAGGCAACGACGTGGCTCGTGGAGAGCGGTTAACAGCCCAAGTGATTGTAGGAACTCCTGGCAAGGTCGAAGGTATGATCAAGAAGAAGCAGCTCGACACTCGCGACGTGAAGATCTTTGTGTTGGACGAGGCCGATGTGATGGTTGCTGAAGATGGTATGCGCGAGCGCTCTGTATCAATCAAGCGTCTGATTAAGAACCGAGCTTGTCAGTACCTCTTGTTCTCGGCTACGTACGCTGATGACGTGCGGGAGTTTGCCCAGAAAATGGTGCCTGACCACAACATCATCACAGTCAAGAAGGAGAAGCTCACCCTTGACGGCATCAAGCAGTTCTGGATTGACTGCAAGACTCGTGACAACAAATACCAGGTGCTCTCGGACATCTTTGCCGTCCTGTCGATCGGGAAGTGCGTCATCTTTGTGCAGCAGCGCGAAACGGCCAAAGAGCTGACGCGTCGGATGCGTGAGAAGGGCCACATGGTTGGGATCCTGCATGGTGCTGACATGGCCAAGGAGGTGCGCGATCAAGTCATCGATGAGTTCCGAGCTGGCACGACCAACGTGCTGATCACTACGAATGTGCTTGCTCGTGGAATCGATGTGGCGGGGGTATCCCTTGTCGTGAACTTCGACATCCCGCTTACTCGAGACCAGCGCCCGGATCCCGAAACGTACTTGCACCGTATCGGTCGTACCGGTCGTTTCGGCCGCAAAGGCTGTGCCATCAACTTTGTCTACGACGACAAAAGCAAGCAGGACCTTGCGGATATCGAAGAGCACTATGCGCGGCCGATAATTCAGGCTCCCGCGGACGATATCGAGGAACTCGAGAAGTTGCTGGCTTGGGGAGCAGCAAGCGCCGGCTGTCGAGGTTTGGCAAGGAACAGCGCAAGGGCATGCGTATTTACCCACGAGAGCCCAAGAAGAAGGTGTCGGGCAAGGACAAGACGTACTACTACCTCAAGGACGTCATGTTCTTGGCGCATGAGCCAGTGCTGAGGAAGTTCCGCGCTTTCAAGACGTTTATGAAACAAGTGACAAAAGCCATGGGGCGGAAAGATTATGCAAATGCCAAGCGCAAGCACGAGAATCGTCCGACGTACGAGCTCGATCACATTGTCAAGGAGCGCTATCCGCGCTTTGTTGATGCACTGGGAGATCTGGATGATGCGCTGTGTGGCAGAACTACATTGTTGCCACGCGAGCGCTTTCTAAGGTTTTTGTGAGCATTAAGGGCATCTACTATCAAGCCAATGTGCAAGGCCAGACCATTACGTGGCTGGTGCCACATCAGTTTACACCGACAGTGGACAAGCGCGTGGACGTGCGGGTCATGCTGACGTTTCTGGAGTTCTATGAAGTGCTGCTTAAGTTTGTCAACGCACAGCTGTACATGCACATCGGAGTCGCGTACCCGCCAAAGATTAACTTGCAGCTTGATTCAGCTGGTGCACAACTAGCTGCGATTGAATTAGCAACAGAAGGAGTCTGAGAAGCGGATTAAGCTATAATGAACAACGCAAGGAATGCAGTGAGAAGAGCTTGTTCAGTGGGCTGACTTTCTTCTTGTCCCGCGAAGTACCTTCGGCTTGTTTGGAACTGGTGCTGCGATCGCACGGCGCGACTCTTGGTTGGGACGGCGCTGGTTCTCCTTTCAATGAAAAGAGCTCACGCATCACGCACCACGTCATGGATCGGCCGCATCAGGGTCATCGCTACTTTAACCGCGAGTACGTGCAGCCGCAGTGGGTATTTGACTCGGTAAACGATGGCATCCTGCTGCCTCTGGCGCGTTACCGCAAGGAGCTGAACAAGCTCAAGTCGGCTGTGCAGGTGCTGCGCGAGGTCGGTGCTGTTGGTGATGACGATGAGGACGGGAAGTCTATGCCGCTGACCTGAAAGCTGAGATGGATGGCAACAAGAAGTCAAAGCGTGTCAAGAAAGAGGAAGAGGCAGCAGAGCTGAAGGAGTTGGCCAAGACTATGATGTCGAAGAAGGCCAAGCATCTGTACGATCGAATGCAGTACGGTTTGGGCAAGAAGGCCGACAAGATCCAGAAGCTCGAGGACAAGCGCGCCATGCTCGAGGCAAGACGAAGAAGCTCCTGGCAGGTGCGCTCTCTGGTATGATCTCCGCGCTGGCTTTCCAGCCTCTGGGGCGATGCGCGGGCGTGGCACATCCCCAACACTCGTGCGCGTAGCCGGCGGCGCGGGTCTCTACTTTGCCACTCTGGACTACTGTTTGAACGTGTTCCCGAGCTCTGCAGTCAATACGTTTCTCGCTGGAGCTGCTGCTCGTACGTTTGCAGGAAGCGTCATGTCACCCTTTACTATTGTGAAGGCGCGGATGGATGCCCTCGGGCGCCTTTCATGGCAGAGGGATGTACCGTTCTCTGGACTCTATGTGCTGCTTTATACGCCATCCGGCCGATGTGGTAAAGACACGTATGCATGCGCCAAACAGTAGCCAAGATGTACCACCACGAAGGCCTGCGTGGCTTTACGAAGGGCATCGTTCCACGCGTAATTAAGCGCACGCTTTCCACTGCAGTGACATGGACGATCTATGAGCTACTCGTTCGACCCGTCCGGTCTGGGTCACGCCAAGGACGCGTTCAATTTGGCCAAAGAGACGGAGCGGACCAAGCAGATTGAGAGTCAGGCGAAGATCAAGGAGAACGAGGCTCTGTACAAACAGTACGAGATCGTGCGGGTGCAGAAAGAGGGCGAAGAGCGCCGCAAAACGCTCGAAGAAGAGACGCGTCAGCACCAACAGCGTGCGCAGTATCAGGACCAGCTCAAGCGCAAGCAGTATGCAGACCAATTGGCCGCGCAGAAGTACATGAAGGAGCAGGAGCTCAAGAAGCAGGAGGAGATCCTGGCTCGTCAGGAAGCTTCACGTCGCAAAACGCTCGACTATGAAGCTGAACTGCGTCAGAGGACAGAGATGGCCAAAGTGGCGGCGGAGACAGAAGGACGTATCAAGCAGGAGCGTCTTAACCATGATCTGCATCTGGAAGAGGCCCGTGTGCGTGCGAAGGAGTACCGTGAGACGGTCATGGAAGGGATCAAGCTGGCGGGCAATACTATCGGCTCTGGTGTCATGTCCTTTGTCGATGACAAGGAGAAACTCACTGCCACGGTTGTCTCTTTGACAGCTCTTGCTGCTGGAATTTACACGGCCAAGGTCTCAACTCGTGTTGCAGGCAAGTACGTTGAGGCGCGTATGGGAAAGCCGTCGCTAGTGCGTGAAACTTCGCGCCGCTCGGCTGCACAGGTGTTGGCGAATCCTATCCCGTCCATTAAGCGTGCGCTGCGTCTACAGAAGCCGACGGATGCATTGGAAGGCGTCGTGCTTGAGCCCAAGCTGGACGAGCGTCTTCGCAGCGTTGCTGTGTCCACGTTCAACACGAAGAAGAACCGTGCGCCGTTCCGTCACTTGCTGTTGCACGGCCCGCCGGGTACGGGTAAGACGCTGTTTGCCAAAGCGTTGGCGCGTCACTCTGGTCTGGAGTATGCTATTCTCACGGGTGGTGATGTTGCCCCGCTGGGCCGCGAAGGCGTGACGGAGATCCACAAGCTGTTTGACTGGGCATCGCACAGCCGTCGTGGGTTGCTGCTGTTCGTTGACGAAGCTGATGCGTTTCTCCAGAAACGTAGCAATACTGTCATGAGTGAAGACATGCGTAATGCGCTGAACGCCTTCCTGTACCGCACAGGTGAGGCAAGCGACAAGTTTATGGTTGTCTTTGCAAGCAACCAGCCAGAGCAGTTTGACTGGGCCATCAATGATCGCATTGATGAGATGGTGGAGTTCCGTTTGCCTGGCTTTGATGAGCGTGTGCGCATACTGAAGCAGTACTTCGAAGACTATATCCGTGCTCCCAAGAACTCGCGTGCTAAGAAGATTTATGTGAAGGCTCTATCTTCGCGCTGGACCCTCTCGCGCACTTCCTCGTGCCAAAACACGGATCTTGCAACTCGAGATACTGCAAGGCGTGTCTAGTGAAGCTCAACTGATCTTCGATGCTCTGAACAAGAGCCCATGCGTCTCTACTGGATCGTCTTTGCGCTGGTCCTGGGCAACGGAGTCTACACGTTTACTGCAGAGCGAGACGAGTCGTTTCTACTGGACAAGGTCAAGAGACAGATTCCGTCCGAGTACCGCATCTCGGAGAAGTGGGACAAGTGTCTCGAGAACTTTGCGCTCTACTTCGGCACTGGCCTCGTCGCTGGCGGCCTCACGTCCCTCGTCTTGGCCCGTTCAGGTGCTGGGCGTGGACTGGTGACGGGCCTGGGCGGGCAGGAGCTGGCTCGAGCTGGACCACGTGTCAGATGGCGTTTGCTGGCCACGAGAAGGCTGCTCTGAACAAGGCGGACAAGACGGTGGGGGACTTGCCGGCGTTCCCGCCCCAGACGATCGTGGACTTTGCGACGGCGTCGGCCACGCAGCAGCAGCTCCTGCACGTGCACTCGCGGCCTGTGTGACCAAGCCACCGTGCACGTGCTGGAGTCGTACTTAGCGGAACAGGTGCAACACACGACGGTCGATGTGGACGCGAACCTCGCGCTGCTCAAGTTGTACCAGTTGTACCCATCCACGCTCAAGGCCGCGAGCGACGGTGCTCATCAAGGGCATGATGGCGCTCCCTTCGACGTTCTTCACGGGCGTCGACGATGGTCCCCGAGAGTATCCGCGAGGATGCTCATGTCTCGGCGGTGCTGCGCACGGGCTTTATGCTCCAGTCGTGTCTATTCGAAGACTTTTGGAAGACGGACGTGGCGTTTGCGGACCAGGTGCCGGGCTTCTTGGAGTCTGTGCGCGCTTACATCTTGGTGGCCATCTGCCGCAGCCACAGTGTGATTTCCACGGACGTTTTCAAGGCCAAACTCAATGTGTCAGACCAGCAAGTGGCTGACATTGTCGCCGAGCAGTGGACGCTGGTAATTTAATTCAGATCAAGCCCAATGAGCTCAACCAGATGCAAGCGAAGAAGGTTCAGGAGAATATTCAGTACGAAGACGTGCTTAAGGCCATCCACGTTCTTTCTAAATCGAACGTCTGCGCCAGTGGCGGCGGCGTAGCAGACGCTGAGGAGGTCTGTGAGAGCCTGGCCAAGCACTTGAGACTCGATGCCGGTACCCAAGTGCTGCCCTGTTCGACGGGCGTCATTGGCTGGCGCATCCCGGTGGATGCTATGGTAGCAAACCTCCCCAAGCTCGTCAAGAAGCTGCAGTCGGACTCGGTGCTGCCTGCTGCCGAGGGGATCATGACCACGGATCTGTACCCGAAGGTTCGCTCTATGGATGTGTGCGGTGGCCGGATCGTTGGCATTGCCAAAGGAGCTGGCATGATTGAACCTAATATGGCTACGATGCTGTCGTATATTCTCACTGATTTGTCGATTCCTCGTGACGTGCTGCGTCAGTTGCTGAAGGAAGTCGTCGGAGATACGTACAACTCGCTTAGTATAGATACGGACCAGAGCACGTCGGATACGGTGGCGCTCGTATCGTCAGACATGATCCCCTTCGATATGAA---GCATCTGGAAGAGGTCAAGAAGGCACTGTACGAGGTGTGCCTCGGACTCAGTGAAGACGTCGTGCGAAACGGTGAGGGTGCACATCACGTTATCCGGGTGAGCGTGGGTGGCGCCTTGACGAAAGAGATGGCCAAGGGCGTCGGCAAGTCCGTGGTGAACTCGCCCCTGCTCAAGTGTGCAGTAGCTGGCAACGATCCAAACGTGGGTCGCCTTGTCATGGCTGTGGGTAAGTACATGGGTCTACATTACAAGGATGTGAACATTCAGCGCCGGATGAAGGTCCGCATGGGTGGCGTCCTCATTTTCGAGAACGGAGAGATGGTGCTGAACGACGATATCGAGGCTAAACTTGTGGCCCACATGCGTGACGCTATGCTGATCGAGCCTACGCGTGGCGGACTCGACGCTAGTTCACACAACTACCCGCCGCACGGGAAGTTCGTTGAGGTTGACATCGACTTGGGCGTCGGGTCTGCGTCGAGTGAGGTATTGGGTATTGACCTCACGCACGAGTACGTGGCCATCAACGCGGATTACCGCTCGTAATGGCACCGGGGAAAAAAGGGAAGACTGCTGCCGCGTACTTGGCGTCTCTAGGCGTCGCCTCTGC---CGCTGGAGGCGAGAAGAAGGAGGACACGAAGAAGAAGAAGAAGAAGGGCAAGAAGAAGCCCGCAAGAAGCCCATGTCGGCTGCTGCCAAGCTGCTGA-TGGAGCGCCAGGCGGCGCTGAAGGCCGAGCAGGACCGGCTCGAGCAGGAACGACTGGAGCTCGAGCGCGTGGCAAGAGAGGAAGAGGCCAAGCGTGTGGCCGAGATGGAGGCCGAGGAGGAGCTGCGGCGCAAGAAGAAGGAGGCGCGAGCGGCCAAGATTGAGCGGCAGAAGAAGGAGGGCACGTACATGACCAAGGCCCAGAAACAGAAGGCGGCCAAGACTCAGGCGGCTCTGGAGGCGATGCAGCAGGCAGGTCTTGTGCCGGAAGTTGCG------GGCGAGAAGACGAAGGTGGTGTATGCCAATAAGAAAAAGCAGAGCAAGGTCATGACGAAAAAGAGGCAGAGGAAAGTGGAGCCCCCGATGCATGGGACGACGACGATTGGGAGACGAGCGATGTCGTGGCTTCACTGGAAGATAAACTAGCAGGAGAAGGAGCAAGAGCGTTTACGTCAACAGGGCATTGCGCTGAAGAAGCGTCAAGAGGAGCAGGCGGAGCAGATGCGAGCGGAAGCTGAAGAGGAGCATCAACGCTCGAAGGCCAAGATGGAGGCGGAGCGTAAGAAGAAAGAGGCTGCCGATCGACGTCGCGAACGTGAGGCCATTGCACGAGCCAATGTCTCGCTGGACAATCTGCGTTCACCTATTTGCTGTATCATGGGTCATGTTGATACTGGTAAGACGAAGCTGCTTGACAACATCCGTAAGACAAAAGTGCAAGACGCTGAAGCCGGTGGTATCACTCAGCAAATTGGTGCTACTTTTTTCCCTGTGGAGGCTATCAAGCAGAAGACGGCTCGGCTTATCGAGACAATGAAGCTGGAGTTCCGTCTGCCCGGTTTGCTAGTGATCGATACACCTGGTCACGAGTCGTTCACAAATCTGCGCAATCGTGGTTCGTCGCTATGTGATATCGCCATTTTAGTGGTGGACATTATGCACGGATTGGAGCCTCAAACTCTGGAATCGTTGCGCTTGCTGCGTCAGAAAAAAGCACCGTTTGTCGTGGCGCTGAACAAAATCGATCGTTGTTACGGCTGGAAGACGATGCCTGATATGCCCATTCAAGAAGCGCTAAAGCATCAAAACGAACACGTCATTCGCGAGTTCGAGGATCGCATGAAAGCGATTATTGTAGAATTTGCCGAGCAGAAGTTGAATGCCGAGGTTTACTGGCGCAACAAAAACTTGGCAAACACAGTCTCGCTCATTCCTACCAGTGCCATCTCGGGCGAAGGTGTGCCGGACTTGTTGATGATGCTGACACGCTTGACACAGGAGCGCATGGCGAAGTCGTTGGCGTTCGTCGACATCTTACAGTGTACCGTGCTAGAAGTCAAGGTCATTGAAGGTCTTGGTACGACTATTGATACGATTTTGGTGAATGGTACACTGGTAGAAGGCTCTACTATAGTTGTGTGTACCTTGGATGGGCCTGTTGTGACAAGCATCCGTTCACTGCTGACTCCGCATCCGATGAAAGAAATTCGAGTCAAGGGGGAATACATCCACCACCAAAAGATGAAGGCTGCCATGGGTGTAAAGATTTGCGCGCAAGGTCTCGAAAAAGCTGTAGCCGGTACACAGATCTACGTGGTTGGTCCTGACGACGATGTTGACGAGCTGAAGGATACCGTCATGTCCGACCTGACGAGTATTCTGGACTCGGTGAAGGCTACCCGCCGTGGTGTGATGGTACAAGCTTCCACTCTTGGTGCGTTGGAAGCGCTTCTAGAATTCTTGCGCACATGTGATCCGCCTATCCCGGTGTCCTGTGTGAACATTGGCCCCGTATACAAGAAGGATGTAATGCGAGCTTCAGTGCAGCTCGAGCACCAGCCAGAATTTGCTACCATATTGGCGTTTGACGTGAAGGTTCACAACGACGCGACTGAGCTCGCCTCCGAGCTTGGCGTGCGCATTTTTACCGCTGATATCATTTACCACCTGTTTGATCAGTTTACGGCGTATATGGACAACTTTCGGATGGCCCGGCGTGAGGAATTCGAGGAGGTTGCAGTGTTCCCTTGCGTGCTCAAGATCGTTCCCAGCTGTGTGTTTAACAAGAAAGACCCAATCATTCTTGGTGTCGACGTTGAAGAGGGCATTCTCAAAGTAGGCACCCCTCTCGTAGTACCATCTGCCGGTG---GGTTCTTGGTCGGGAAGGTGGGCTCTATCGAGCGCGAGCACAAAGAGGTCGACCGTGCTAAGAAGGGCGCGTCGGTTGCTGTGCGGATAGACAACGAGAGCAGTGTCATGTACGGCCGTCACTTTGACCACAAGAACAAGCTGGTGTCGCGCTTGACTCGTGAGAGCATCGACGCACTAAAAGAGAATTTCCGGGATGATCTTCAGAAAGAGGACTGGCAGCTCGTCATCAAGCTGAAGAAGGTGTTCGATATCATTATGGAGGGCTGGCTCACCAAGCGCCGCGACCACATGAACCTCATATGGCGGGACCGGTACTTCCTCTTGGACGGCAATCAGCTGCGCTACTTCCGTAAACGGGGCGACTTGGCTCCTCGCGGGACGTACATTCTGACGGACAATTGTGTCGTGAGTCCCGTGTTTAACTCGGAGGAGAAGCACAAGCACCACGGACCTGTCTGGATGTTCCGCATCACGTGAAAGGTCGTACAAGAAGGACCGGTTCTTGGACCTTGGTGCCAAGAGTGAAGAGCACGCGAGGGAGTGGAAACGCGCTCTTGAGAACGCCGTGCGCTCGCTCCAGGAGCTCGTGGCGCATCAGCGCAAACTCAAGACGGACGCCGAGAT---CTTTCCAACGCCCACGCAC---------GTCCCGAGGATGCTCAAAACGGACGAAGATACCGTGTCGCTGTTTAACTTGGACGCGGATAATGTCAAGGCCGAGCAGGCGTGGTGGCTCGCACGGGTGGAAGGGGGACTGCGGATCATGCAGGAGTGTCCGTTAGGGCCAAACGTGGCGGCGAATTTGTACGCGCAGCACGCAGCAACGACGAACGGACTGATCCTGAGCGCTCTGCTCGGTGCTGCACTGAGCTTCAGCAGCATGTACTCGTTCGCGAACAACATGCTGCTGAGTTTCCTGACTGCTCTTGGAGCGATTGTCGTGTGCTGTGTCGCCGTGACGCCAACACAAGCGCCTGAGATCCCGAGCTTCCGTGTCTCTCAAGTAGTGCACGGATCGCCGACTGAAGTCTTTCGACTTATTATGAACTCGAAGCGATTCCAGCGATGGGATGCAGCAACTGCGACGTTGCGTGTGGTACAGCAGCTGGATAACAATGCGGACATTGTCTACGTGACACAACGTCCGACACGCTTGTGGCCGCTGTGGCAGAAAGCTCGTGATCTTGTGTTCATGCGCTACTGGCGACGCGAAGAAGATGGATCGTACTTTGTCATGTATCAGAGCATTGACCACCCTGAATGCCGCGTGCGTCACAACTACGTGCGAGCAGATATACTGGGTGGCGGTTTTGTGATTGCGCCGCAACGCGTTCCCAGT---GGATCCATTCGCACGCTCGTAACCTACGTGCTCCGGTACGACCCTGGCGGCTG---GTCACGCATTTACCATCAACTCGGCATGGATGTGGACTTGGTCATGCCCATGTTGCGCAGCGTGATTGGCATTCGTGACGAAATGAGCGCCACGGACTTTATCACTCCTAACGTCTCAATGGCGCCCGATGGTGAGGACAGGTGCGGACCG---AGCGTGGA---CTCGATGGGTGTTGTCATGGGTGTGCAGAAGCTGCCGACCACTAGTCTACCGGAGTCAATGTGGGCCGAGCCCGACGCGTCGCTATTCAGTGTGCGTGGACACAACTACTTGAATGACAAGAAGAAGATCCCATCAGCGCCAGCGATGTTCCACGTCGTGGGTGTGGACTTGCTGTCGTTCGAGAGCGCAGCTGAGCGATACGATATTTCGTCTCGTGTCGACAGCATTGGCCGCACCAG---CCCCAAGTTTACGTTTGTCGTGAACATGATCATCCCGGGTCCAGAAAACACGTGCATGGTGTTCTACTTCCACCCTGCACGTGACAATGTGTTCGAAGACGGCTCGCCATTTTCCGAGCTACTGAACGACTTCTTCGATGGCGACGACCAGTTTCGGAACTCGCGCTTCAAGCTCATTCCTACTGTCGCAGAGGGCAGCTTTATCATCAAGCAGTCTGTTGGATCCAAGCCGACACTGCTGGGCAATAAGCTGAAGTGCCTGTATTACCGTGGCGGGAACTACTTCGAGGTGGACATCGACATCTCGTCCAACTCGGTTGCCAATACTGTCGTGGGAATGGTACAAGGTGTAACCAAGTCGTTGGTGGTGGACATGGCGTTCCTGCTCGAGGCTCAGTCCGATGAAGAGCTGCCTGAAATTATCCTTGGTGCAGTTCGCTTGCAGCATGTTTCTCTAGACAATGCCCGTCGCGCGCCAAAGCATGGGGAACACCCACTACCTCCGCGTCCACCTTCTGAAGTGTTGACGGAAGCCCTGGACGCGCATTTGCACCAACTGGTCGCCTACGCGCACTCAGCCGACACTTCGCTCCAGCGAGAAGTGGCCGAGAAATTGGCCAATGAGGCGGTGAAAATCGGCAGGTGCAGATTGTGGAACTAAACGGACTGCAGCTGCTGCTGCCACTGACCAAGTCGACGGACACGGAGGTGCAACGACTTGCAGCGCACGCTCTTGCCAACCTCTCGGTCAATTCGGAGAATCAGTCGAAAATGGCGACTGAAGGAGGCATTGGGATGCTCATTGATCTCTTGAGCAGCTCGAACGAGCACGTTCAGCGCCAAGCGGCCAAGGCACTGGCTAACCTAGGCGTAAATGTGGATAACAAGGAGCGAATCGCAAAGGCAGGCGGCATCAAACCGCTCATTGACTTGGCAAGCTCGCGGCAAATAGGCGTCGCAGTTGAGGCTGTTGCGGCACTTGCAAATTTGGCTGTCAATG---ATGCCAATGAGGTCGAGATCGCACGTAAAGGCGGCTTGAAGTCTATCATTGATGGAGCACACTCAGAGTCGATGGAGCTCCAATCCCAAGTGGCACGTGCACTACGCAACCTTTCAGTCAACCCGGAGAACAAGCAAGCGATTGTGGACTTGGGTGGTGTGAAGGCTCTGCAGTCACTTGTGCGCTCCACTAACGACCGGATATGCCAGCAAGCTACGCGGGCACTCGTGTCCAACGTGGCGCTTACGCGCTCGTCGCCCTCAATCTCGTGCGCGCTCTCGCGCCTGTCGC------TTGTGCGGCGACGGAAGATACG---CGGCAGGACTACGACCCGTTGGTCATGGCCGTCGAGGCCGCAGCGACGATCAAGACGATCC------GTGACGTGCTTAATCCAACGCCCC---ACGACTCTGCACGA---AGCTTTTTGTCCATCGCGGATGTGGCGCCACTGTCCAAGGTGTCGCTCGCCAAGGTGGGCACG---CCCTCTACGGTCCGCTA---TGTGCACGACG---ACAC------------------GGTGGATGACG----------ATGCGACAGTG------AGAGAGGAAGCAGACGGCGATGTG---ACGACGATGGACGAC---GGCGA---TGGGCGTCGTGTACCGGTACAATGCGACGGA--GGTGGAGGAAGATGAGATGGAGGAACTTCGCTGGACTATCCATCAGCAAGTGTTTCAGCGATACTCTCAGCTCGAGCAAAAGTGCGACGAGTGGGGACAACTGGCAGAGCAAGATACCGAGTGGTTTTCGTTCACGGTGTCAGACGGGAACGGATTACGGCCGAAAAGAAGTTTCACGACAATGCGGTTCGTGATGCCGTGGGAAAAGCAAACGCTGCGTGGGTGGAGGAGCGGCGCAATCGCGTTGACGAGGAAGTCCGTCAGTTTTACGACGAAATGCGGCACAGACAGGCGTTGACGCTGACACGTCTGGAAAAGATGTCCCCATTCTGTAAGTTTCCGATCCTAGGCAATCGATTTTTGCCACTTCGTCTGCGAGGAAAGGGTGGACATGGGGAAGTGTGGGACGTGGTGGATTTTGCCGACAACAAGAAGCTCTGCGCGCTCAAGCTATCGACTTCCATTTGCCACGCCAAACGCGAGCACCACGTGCATACTCAAGCACCCGA--ATATTGTTCAAGTCGGCGAAGTGCCACTGCTCATTCGCTACCAACAGCAGCAATACACAGCGTTCACTGTAGCTAGTGTCCATTCCGACTTGCAGCAGCTTATTGAGA---TACATGAATTCTTCGACGACGATTCGGCATACAAGGTGCTTTCTCAGCTTTTGAGTGCCCAGAGGTACCTGCACGATGGGCATCGCTCACTACGACCTGAAGCCATCGAACATTTTGATCGATCAGGACGGGTGTGTGAAACTG------ACTGACTTCGACTTAGCCCGCGATGCCAAATCTACTACGTCAAATTCTACAGTTGGCACTCTACGGTACCTCCCGCCCGAGTGTTTTCGTCCGAAACGAGGCGA---------TTGCGAAGCCACGGCTGAAAAAGCGGATATGTGGAT------------------------------------------------------------------------------GTCAATGATGAGTCGGTACACTGGA---GCGATACAGTACGCTCGGCCAGTCTCCGATCTGTCGAGGTGGATTATACAAGGATGCCTTCAGCCCGACCCAAGGTGCCGACCCACGGCCGCGCAATTGCTGGTGGCGCTACAG

Peronosclerospora_sorghi GATGCTGTCGTCGCTA-----CGGAACAACTCGCGGCCTTCTTCCACTCTTTCTATCGTTTTGGATGGGACTCGAATGACTTTGACCTGTTCCTGAATAGCATTGTCTTGGCGTTCCTCTGCATCGGCGCTCTTGTCTTTCTTGTACTCTGTGTGATCGTCGTGCGGCGCACGGTTCTGCATATTCGTAGGCTTCTGTGCGTCGCACCTCGATCAATGATACGACGGTGGAACTCCTTGCTGTTGGACTATTAACGTTCCTGGCTATTAGCGCTCTTGGCGGACTCCTAGGAGAAGCGATTTGGACACTAGTGACGCAGTGCGTTATACTGCTGACAATCTCATCACGTCGTTTAACGAAAGTGAATTGCCAGCAGATGCATTCAAACTGTCCATTGAAGCACTGGACTCGTGCATACCTCCAAGGGCTTATGGAATCAGACGGACGCACTCTTGCCGAAGAATTACTCAGAATTAGCAAAAGACTGGGAGTTTAGCTACTTTGTGGTCAAGTCGTCCACGAACGGCGCCATTCTGGCGGTCACACTGGCTAGTTTCTTGTCCATTGCGTCCGTGGGATGGTCCATGGTCTCGCCCCTGCGTGTGTCCATTCTCGTCATCTTGAGCGTCGTGCCCATTTCCCACACCCTTATTGGCGCGTATCTATCGTCTACAATCATGACGGCGGACTTTTGTGCGGCGCCTATGAATCATACGCTCGAACTTGTGGGCACATGTCCAGCGAACGCGTCCTGCCGTTTGCCGATGCTGTCGCGACAGAACGCATCGCACGAAGGAGAGATGGGCCGTCGTATGAAGCGAGACTTCTTGGACCCCATTGGAGCAATTGTGGGCGTCATGCTCGGTTACTTCGCTGGCACTGTAAGCAATTGGGAGAGCTTCTCGCTTATTCAGACATGGCGCTGGCCCTTCCTCCTCCAGTTTCTAGCGCTTTTACCCATTAATCTTGCCATCTTCTGCGTGCCTGAGAAACATCTCGTGATTCGCAGCGAGCATTACGAGGTGGAACCGCCAGTGGCTGGTACACCCATGAGTACAAGATCGACAACGTATCACGATGAAGAAGACGCTTTGCTTGCTAGTGCCAGTATGCAACATACGGTGCCTTGGACACGAGGTGTACGAACAGGGCGCGCTTTCTGAAGGGATATACGAGCTTTTGCATCTACCCGTCTTCTGCTTGATCGTCTTCGGCCTCACGACCGTATACTTTGTTGTCACGGGCGTCCAATACTGGAGCACCATTTTTATGATCAAGTCGCTACATGCCTCCAAGTATCTGGTGAATGCGCTCTTTGTCGTTGTGTCCGGAACAGGGCCAATCCTGGGTGTATTTTTTGGTGGGTGGCTCGTTGACCGATATGGAGGATATATTGGCGTAGACCAGCGTGCTAAGGCATTGGGCATTTGCATGATTCTGGGACTCACGGCGTTTTCAATCTCAGCGGTGGCGACGTTCTTCGACAACGTCTACACTACCTCTAGTTTTTTGTGGCTCTTGCTCTTTTTTGGTGGAGCTATCTTACCAGCGCACCGTGCACTCGCTTCTTCGTTTTCCGTTATGATATTCAATCTCTTTGGTTACTCTCTCTCGCATGAGCGATCTTCAGATGGTACCGGGTGCCAAGTGCTTCGTGCCAGACGAGACGCACGTCTGGCTTGCAGCCGAAGTGCTTCGCGACGAAAACTCAGGTGACGGCAAGACACGTAAAATTTATTGTAAGGTCGAGTTGCCAGACGGAGATAGAGGAGCGATGCGTAGACTTGCTCTCAAAGAAGATAAAGACGTTGTTGCCGTACATCAGCTCGAGACGTTACCGTATCAGAACGAAAACGTGGGTCCTCAGGGCATCGAAGATATGATCACGCTCAACTATCTACACGAGGCCGCAATTTTGTTCAACATCAAGAAACGCTTCCTGTGTGAGCTGCCATATACTTACACGGGGGACATTTGCATCGCTATTAATCCGTACAAGTGGTTACCTGACCTGTACGCTGAAGAGCAACATTTACGATACCTTAATCATCCTAAGGAAGAACTACCCCCGCACGTGTACGCCACTTCGGTTGCCGCCTATGACAATATGCGGCGATCGCAACGCAACCAGTCGATTCTGGTCAGTGGCGAATCGGGTGCTGGTAAGACTGAGACAACCAAGATCCTGATGAACCATCTCGCTACCATTGCTGGTGGAATGAACAACTCTACTATTAAGAGGATTATTGAAGTGAATCCGCTACTAGAGTCCTTCGGTAATGCCAAGACGGTGCGAAATGACAACTCCAGTCGATTCGGCAAGTTTACTCAGCTACAGTTTGACGTGAATGGCACACTCGTAGGAGCGAAGTGCCGCACCTACCTGTTAGAGAAAACTCGTGTCATTCAACACGAAGCCCCGGAGCGCAATTACCATATTTTCTATCAGTCGACTCTCCCGACATCGCATCGGAACTACAGCTTGACCCTACAAAACACTACGCGTACACAGGTGACAACACGGCACAGAAAATCGAGGGCCTTTCTGATGAAAAGCATTTTAATAAGACACGCGAAGCACTTGAGCTTGTGGGTCTATCACGCGAAGACCAGCGACCGCTGTTCGAGGTACTGGCTGGTGTCCTTCATCTTGGTGAGGTACAGTTGCTGTCTGACGCATCAGACGATGAGAAGTCTCTTATCGCTCCAGGCGACGTTGGTGCTACTTCTGCCACCCAAATGCTAGGTGTCACGTGGGAGGCTCTTGAAACTGCCCTGTGTTCACGTACCATGCGTGCCGTGAACGATGTATACAGCGTGCCGCTGAAGAAGGAGTTGGCTATGGATTGTCGTGACGCCCTCTCCAAAGCCATTTACTCCAATGTATTCGACTGGCTTGTAGCCACGATTAACCAGTCCCTTGCTGATGATACCAACATGGCGAACCACGTTGGTGTGCTCGATATTTTCGGTTTCGAGCACTTTAAACACAACTCATTCGAACAATTCTGCATCAACTACGCCAACGAGAAACTTCAGCAGAAGTTTACCCAGGACGTTTTCAAGACCGTCCAGATTGAGTATGAGGAAGAAGGTATCGTGTGGGACCACATTGAGTACGCAGATAACCAAGATGTGTTAACTGTGATCGAGTCGCGCATGGGTATTATTTCTTTGCTTAACGAAGAGGTAATGCGACCGAAGGGAAGCGAAGAGTCCTTCATGTCTAAGGTAACTTCATTACACAAAGAT---GACATGACTCATGTCATTGAGTTCCCTCGAACATCTCGTACCGAGTTCCTTATCAAGCACTATGCTGCTCCTGTCATGTATGATTCGGTGGGTTTCTTGGAAAAACACAAAGACTCGCTGCTGCCTGATCTGTCTGAGCTCATGCGTGGTTCATCGAAGCCATTTATCGCGAAGCTCTTCGAGCAAAAGCCGGAGCCTGCGTCGTCGAAAAC---------GCGGTGGTGCTTTGTCAATTACAACCGTTGGAACTCAATTTAAAGAGAGTTTGACGGAGTTGATGGCAACCATCAACTCCACTCGTGTCCACTATGTGCGTTGCATTAAGCCCAACCCGATTAA---------GAGCTCGACTGTCATGGATCAGATGATGGTTGTTTCTCAGTTGCGTTGTGCTGGTGTAATTGAAGCCATTCGTATCTCTCGCGCAGCTTACCCGAACAGATTGCAGCATATGGAGATTTTGGACAAATTTTGGCTGTTTGTACCAAGCGGTGGGAACAGTGCGGAAGAAAAGTGTCAGCTACTGATGGACAAACTAAAATTAGAATCTCCGGTTCAATACCAGATGGGTAAGACCCGGGTGTACTTTCAGCTTGGCGTGCTTGAAGAACTGGAAGATCGCCGTAAGAAATTTCTTGACGCGAAAGCCACGTACGTGCAGTATGTCATGCTTGGCTTCACACAACGAATTAAGTACCTTCGTCAGCTCGAAGCGATCATCAAGTTCAGTGATCAAATGTGTGATTGCTATGCGGAGATACAATACTTTTAGGAAAGGTATCGTGCTGACGCAAGCTCGCTGGCGGGGCATTCAAGGGCGAAGGGTGGCTGCAGAAGTTAAAAGCAACCACTACGCTGTCATCATTCAACGGTATATGCGTGGTTTTGTCAAGCGTCATCATTACGTTAAGATGCGGGCCATGGTCATTCGTGTCCAGGCTGTAGTCCGTATGACGATTCAGCGACCAAAGTACCGCGCTGCCCTTGAAGAAAAGCGTCGTGAGGCTGATATGGCTTATCAACTAAACAAACTGAAGGCCGCACTCCAGGAGGAACAGGAAAGAAACGCCCAGCT------GCAACGTC---GCAGTAGCGTCAC--GACAGCGCTTCTGCTGCTTCAAGCGAAGTGATGGCTGATGCTGGTGGCATGATTGAAACTCTCACCGACGAAAACAGGAAGTTACGTGAAAAGAACGAGGAAATGCGTGTTGCGATGAAGGGATTGAAATCTCAAATTGAGAAATTCAAGAAAGATAAAGAACTTGCGTCGGCTGGAAACCACGTTAAGGTACGCCAGCTGCAGGACACTGTGCGCGATAAGGACAAGAAAATCACGCAGCTTGAGATTGACAATAAGAAATTGATGGAGCAAGTTGCAAAACTTCAGATAGAAGG---TAT---ACCTGAGAGGCAGCCGACGCAGAAGAAATCGATCTTCCGCACAATAGGATCCAAAAAGGAGAAGAAGCCGC---------------GTGAGACCGTGCTAATGGACTCCCTTACCGGTTCGGATGACTTAAACAGTCGTGAGACGCGCCATCCGGA---GCGTCATTTCCCGCGCATGC---CAAGCATGAGCGTGGGTTCT---CGTTTCTGGAACACCAACAAG---AAGGGTGAT------------ACCGGCGACGGCTTCGACGTCTCTGAGGTTGAAGGTGAGGACAGCCCAACGAACCCACGCGCAACACGATCGTGAAAAGCGTGGAGGTGGGCGTGACGGGCGCCATGTCGACTTTGAAGGGCCGTATG---------TCGGTCGTAAAGGGCATGTATGAAACAAGCGTTCAAACAAGATCGCCCAGTGCGTGAGTCGTTCAACTTGGATGCGTTGCCAGAAGTGTCTTTGCCTGTCGGTTGGGAAGCCAAGGTGTCTCGTTCGACGGGTCGCGTGTACTATGTGAACCGCAAGTTGGGCAAGTCTCAGTTTGAGCGCCCGACGCTAGCCTCGTTAAA------GGCACAGAAGTTGGCGCGTCAAAAGTCGGTAAGCGGGGTG-AAGGCTATTGTGGCGGATTTCGGCTGTGGAGACGCCCGCTTGGCGGAGAGCGTGTCGAACAAAGTGCACTCGTTCGATCTAGTATCTCTCGATATTGGAGTGTACTGCTTGGCATTGATGGGGACGAGTGTTCGGGAGTATGTGCGCGAGGTGCATCGGGTGCTCAAGCCAGGAGGTGTCTTGAAGGTCGCCGAGGTCAAGAGCCGTTTCGAGAGCGAAGCGCTGGGCGGATGGTTGGAGTGTTCATAGCGACCGCCCAGGGCGTCGTAGTCACAGTGCGCAGCAAAACGGTTTTTGTGGAGAAACTAAGTACGCAGTTATTTTGGGTCAAAAGACGGGTGATGTAGTGGCTGTGCCATTCCCGGACCTGGACCGTGACCTTAAGGCATTGCTGGGACACACTACATCGATGGTCACCAACGTAGCGGTGAACTTCATCACTTCTACTTACAGCCGACAGAGATGAAAAGCTGCGCGTGTCGAAGTTTCCAAACACTGCTGTCATTGTAAGCTACTGCTTGGGCCATGTTGCTAGTCTGACCAAGGTGGCATGCAGCACAGTCACGCCGGAACTTGTCGTGTCGACTTCGATGGATAACATGCTAATGCTTTGGGATATGATTACTGGTAAATTGACAATTTGCCCGAAGACAACACAGTCGTGGTGCTGGTAAACAACCAGTACGTGCGATTCTTCGATATCACCAGAACCCATTTTTACAGTTATTTTCTATCTCAACGGGCGATTGATGCGTTCAACTGCTGTGCGCGATTTTCGCAAGTTGCAGAATGATCCGCCGTCTGGCGTGTCAGGTGCTCCGATGGATAACAATATTATGCTCTGGCAGGCCGTCATTTTCGGACCAGACGATACGCCATGGGAAGGTGGCACGTTTAACCTGACACTCGAGTTTAGTGAAGATTACCCGAATAAAGCACCCACCGTCAAGTTTATCACGAAAATGTTCCACCCCAACATTTATAATGACGGTCAGATCTGTCTGGATATTCTCCAAAACCAGTGGTCGCCGATTTATGACATTTCCGCCATCCTCACCTCCATCCAGTCGCTGCTCTGTGATCCCAACCCCAACTCGCCCGCGAACTCGGAGGCCGCGCGACTATTCCAGGAAAATCGACGAGAGTATAACCGTCGTGTGCGTGAAATTGTGGAGCAGAGCTGGCAGGCCATTGATGGTCTCGGCGGCAAAGATGCGTGGCGCTGAGCAGCGTATGAAGGCGGGACGACCATTTACGGCGTGGTTGGACGCCGTCTCATCGGATCGTGGCCTCTGTGGTGGCATCAATTCCGGCATTGCTAAAGTCACGCGGAAGCAGACCGGGCGCATCTTTTTGTGATTGGGGACAAGGCCCGCGCCCAATTGCGTCGGGACCTGAGCCAGAACATTCGGGGAAACGTCACGGAGACGTATCAGACGCCCCCAAACTTTATGGTCGCAAGTGCCATTGCCGAGGCGGTCGACGGCGTGAGGAGTCGTCGCGCATGGCTGCGATGGAGAATGCCACGACGAATGCGCAAGATTTGACGCTCACGCTCGTGTACAACAAGGCGCGACAGGCGCGGATTACGACGGAACTCATTGAAATCATCTCGGGCGCCGCGTCCTTGGACCATCCCTGGCACCGTCGTGAGTATACAGAGCGTCGCGCAGTTGAACGAAGCCACGGCGCGGGACGCGACGCTCTCGATCGTCTACTTTTGGGCCGATTTTCACGCCTGTCGTCCCAACGGCCAGCTCGACGTGGGGCCCCAACAAGCTCGAGACGCTCGAGGGCGCGAGTGTCGCCGAGTTGGCCAAGCGCGTCTGGCGTATCGGCTCGAGAAACTCATAACCGCGGCCCCCGTCATGCTCTTTATGAAGGGGACACCCCGTGCCCCGCAATGTGGTTTCAGTCGGGACCTCGTGGCGCTCTTAAATGCCGAGCACATTCCGTTTGGCACGTTTGATATTCTCCAAGACGACGACGTGCGTCAAGGGCTCAAGACGTTCTCGAACTGGCCCACGTACCCGCAGCTCTATGTGAATGGCAAGTTGATTGGGGGCCTGGATATTGTCAAGGAGATGCAGGCGGAGGGTCCGCTCGTGGACCAATTGGGCGTCGAGGCCGCGGCGGACGCGCTGGCAACGAGTTTAAGAGCACTCGTCCGCTCGGCGCCCGTGCTCGTATTCATGAAAATTTGACATTTTGTGTGGAGCTACCAGCGTCACGACCAAGACATTGCTAAGGTGGTGACGTTAACGCCATCGTCTTTCCGGAAGGATATGTGCTGAAGCTATCTCGTGGAGTCCGAAATCGCATCGCCATGGCATTTCGTGATGCTCTGGACGAAATGGGTGAGTTCTGCGTGGTGCAGCTTCTCGTATTACCTCGAAGCGTACCACGTTACATCCACGTGGTATTTCTGCGATCGACTGGCAAGTGGCATGTGCTTCATCGTGACCACCGGAGCGAAGTCAAGTCGCTCTGCGTTCATTCCAAGCGTGATTGCTTATATACGGGTGGTCTCGACGGCCTCTTGATTCGATACGACATGAATAAGGGTTGTACGGAAACAGTGCTGGAGCGCCGGAAGCCCATATGCAAGATCAATTCGGTGCTCGAGCACCCACATAACCCAAACCTGCTTCTAGTGAGTTGTGTGGAGCAGGAGGCACAGCATGCTACTGCACGATCTACAGCGCGATGGCAACAAGGTGTCAGGTGGGTCCATGAGTCAGTATGTCGTCCCGCGGTGGTCGCCTGCGGGGTACCACGTCTCGTGTGGCAGTAAATGCGCTATTGCCACGGAAGCTTTTCCACTTCCACGTCAGCTACCATTGGCGCTTCCTTTTTGCAGAAACGTGTGATCGTTGGCGGGAAGCTGACGCTGCAGATCTGGGACACGGCCGGCCAGGAGCGCTTTCGCTCCATGGCACCCATGTACTACCGGAATGCCAAGGCGGCGATTTTAGTGTTTGATTTGCAGAGTGAAGCGAGCTTTGAAAAGATTAAAGAATGGCTGCATCTGCAGCATCACGTGGGCGACGATATGTGGGGAACAAAAGTGATGTTTTGACTTTTCCAAGGCCCAACAATTTGCCAACGAGATTGGGGCGCTGGTCTTCCGCACGAGCGCGAAGACGGGGGAAGGGGTCCAGACGCTCTTTGAGAGCTTAGCAGCACAATTGCTCAAGAAGCACGATCAGGAGAAGCGAAGAACTTTTACGTCGTGTTCATACCAGCAGATGAAGGGAAAGAGCTCGAAGAGTGGGCCGTTGCGCTGCCAGCGGACAAAGAGGCACAACTGGGCTGTTTGACGGAAAGACTACGTCAGCATTTTAAAGGAGGAAGAGTCCAAACAGCAGCAGAGACTTTCAAGCAGCAACTGCTTGCTCAATTACCCAAGGGCGCGACGCTGACCGACGAAATGGTGCAGACGATGCATGGATAGCCTTGTGGACTCGATTCCGCTCGTGCTCAACACGCCCGACGTCAAACACGTTGGAGGTCAAGGGTGATGCATTCATTGGACGCGTGTATGATAGATGATTTTGTGCGTATGGATTTTCGCTTGAGTGAAGTGTCTGGTGATGCCCCGTGGGTGCAAATAGCAAAGTCACAGAAGTGACGCAGTCTCTCGAGGACATTCTAGCGTTAGGTCAAGGGTTGGACGACGAAGATTATAAGGCGGAGCTCGAGGTACTACAAAATGATCCGAATTCGAATTTATACTCGGCCGTCACGTTCGAAGAGTTGAATTTACCCGAGCCACTTCTAAAGGGTGTCTACAGCATGAAGTTTGCCAAGCCGTCCAAGATTCAGTCCGTGGCGCTCCCATTGATTTTGGCAGATCCACCGGAAAACTTGATCGGTCAAGCCCAAAGTGGCATCCCACGCGCGAGCTCGTGCGTCAGATTATGGCCGTGGTAAACGCCATGGGCAAATTGTCGACACTTTTCTCGCGATTCCCGGCAATGACGTTGCGCGTGGCGAGACGGTCACGGCGCAAATCATTGTGGGCACGCCCGGCAAAGTCGAGGGCATGATCAAGAAGCGACAGCTCGAGACGCGGGACGTCAAGATCTTTGTGCTCGATGAAGCCGATGTCATGGTCGCGGAAGATGGCATGCGCGAGCGGTCCGTGGCCATCAAGCGTCTCATCAAGAACCGGGCGTGTCAGTATCTATTATTCTCTGCCACGTACGCGGATGACGTGCGCGAGTTTGCGCAAAAGATGGTGCCCGACCATAATATCATCACGGTCAAGAAGGAAAAGCTCACGCTCGATGGGATCAAGCAGTTTTGGATCGAGTGCAAGTCGCGCGAGCACAAGTTGCAAGTCTTGTCGGACATTTTTGCCGTCTTGGCCATTGGCAAGTGTGTGATTTTCGTGCAACAGCGCGAGACGGCCAAGGACGTGACGCGGTGGATGCGGGACAAGGGGCATATGGTCGGGATTCTCCATGGCGCCGACATGGCCAAGGAAGTGCGCGATCAAGTCATTGACGAGTTCCGCGCTGGCACGACGAACGTGCTCATTACGACGAACGTACTCGCACGTGGGATTGATGTGGCCGGTGTCTCGCTCGTCGTGAACTTTGACATTCCCTTGACGCGGGACCAACGCCCGGATCCGGAAACCTACTTGCACCGGATTGGACGCACGGGTCGATTTGGACGCAAGGGCTGTGCGATCAATTTCGTCCATGACAACAAGAGCAAGCAGGACCTCGCCGAGATTGAAGATTACTATGCGCGCGCGATTACGCAAGCCCCTGCAGACGACCTGGAACAATTGCAAAAGCTGCTCGCGTGGGGGGCAGCAAGCGTCGTCTGGCTAGGTTTGGGAAGGAGCAGCGCAAGGGAATGAGCATTTATCCACGCGAGCCCAAGAAGAAACCGTCGGGGAAAGATAAGACGTATTATTACATTAAGGATGTCCTGTTCCTGGCCCATGAACCCGTGCTCGAGAAGTTTCGCGCGTTCAAGACGTTTATGAAAAAAGTCAAGAAAGCCATGGGCAAGAAACACTATGACGATGCCAAGCGCAAGCATGAACATCGTCCGACGTATGAACTCGATCACATTGTCAAGGAACGGTACCCGCGGTTTGTGGATGCGCTGGGAGACTTGGACGACGCCTTGTGTGGCAGAACTTTATTGTCGTTTCACGAGCGTTGACCAAGGTCTTTGTGAGCATTAAGGGCATCTACTACCAGGCCAATGTCCAAGGTCAGCCTATTACGTGGGTCGTGCCGCATCAATTCACACCAACGGTCGACAAACGTGTGGATGTGCGAGTGATGCTCACGTTCCTCGAGTTCTATACTGTGCTCTTGAAGTTCGTGAACGCCCAATTGTACATGCAGATGGGAGTGGCGTACCCACCAAAGATTAATTTGCAGCTCGATTCGGCAGGTGTGCAGCTTGCTGCGATTGAATTAGCAGCAGAAAGAGTCAGAAAAGCGGATTAAACTTCAACGAACAGCGCAAAGAGCGCAGTGAGAAGAGCTTGTTTAGTGGTCTCACCTTCTTCTTGTCACGGGAAGTTCCGGCCGCTTGTTTGGAGCTGGTGCTGCTCTCCCACGGTGCAACGCTGGGTTGGGACGGCCCCGGCTCGCCATTTGATGAAAAAAGTGAGCGCATTACGCATCATATCATGGATCGTCTGCACCAAGGTCACCGTTACTTTAACCGCGAGTACTTGCAGCCGCAGTGGGTGTTTGACTCTGTGAACAACGGCATTTTGCTGTCGACAGCGCGCTACCGCAAGGAGCTAGATAAGATGAAGTCTGCCGTGCAGGTGCTACGCGAGTTGGGCGCCGTTGGCGACGAAGAGGAGGACAAGTCGCTTATGCTGCAGATCTCAAGGCTGAAATGGATGGCGACCACAAGTCAAAGCGTGTGAAGAAAGAAGAGGAAGCTGCGGAGCTTAAGGAACTGGCCAAGACCATGATGTCAAAAAAGGCCAAGCGTTTGTATGACCGGATGCAGTACGGTCTCGGTAAAAAAGCCGACAAGATTCAAAAGCTTGAAGACAAGCGTGCCCAGCTCGAGGCAAGACAAAGAAGCTGCTGGCAGGGGCGCTGTCTGGGATAACTTCAGCTCTAGCCTTCCAGCCGCTAGGGTGTTGCGTTGGCGAGGAACGTCCCCTACGCTTGTGCGTGTAGCGGGTGGTGCTGGTCTTTATTTCGCTGCGCTGGATTACTGCTTAAACGCGATCCCAACCTCTGCAGGCAATACGTTTTTAGCTGGAGCCTCTGCACGCACATTTGCTGGCAGTATCATGTCACCTTTTACTATTGTGAAGGCGCGGATGGATTCCTCCCGACACTTTCGACAGCAAAGGGACGTGCCATTTTCTGGACTTTATGTGCTCATGTACACGGCATCCTGCCGATGTCGTTAAGACGCGGATGCATGCGCCAAACAGTGGCCAAGATTTACTGCCACGAAGGTCTGCGTGGCTTCGCCAAGGGCATCGTTCCACGTGTAGTCAAGCGTACTCTATCCACTGCTGTCACGTGGACGATTTACGAGCTACTCTTTCGATCCATCGGGTTTGGGCCATGCCAAGGAAGCTTTCAATTTGGCCAAGGAAGCGGAACGCACCAAACAAATGGAACACCAGGCAAAGGTTAAGGAAAATGAAGCTCTTTATAAGCAGTACGAGATCGTGCGCGTGCAAAAGGAGGGTGAGGAGAAGCGCAAGACACTTGAGGAGGAGACACGTCAGCACCAACAACGTGCGCAATACCAGGATCAGCTCAAGCGTAAACAATACGCGGATCAACTTGCCGCTCAAAAATACATGAAAGAGCAGGAACTTAAAAAGCAGGAGGAGATTCTTGCTCGTCAGGAAGCTTCGCGTCGCAAAACGCTCGAATATGAAGCTGAGCTGCGCCAAAAAACAGAATTGGCCAAGGTTGCGGCTGAAACTGAAGGACGCATTAAGCAGGAGCGTCTCAACCACGATCTGCATCTCGAGGAGGCCCGTGTACGCGCCAAGGAGTACCGCGAGACTGTCTTGGAAGCTATTAAGCTGGCTAGTACCACTGTTGGCTCTGGTTTTATTGCATTTGTTGACGACAAAGAGAAACTTACGACCACTGTGGTCTCACTGACGGCTTTGGCGGCTGGAATCTACACGGCAAAGGTTTCAACTAGCGTTGCTGGCAAGTACATTGAAGCTCGTATGGGAAAGCCTTCGCTTGTGCGCGAGACTTCGCGTCGCTCGTTTAGGCAAGTATTGACCAATCCTATCACATCCATCAAGCGTGCTTTGAATCTGTATAAGGCATCTGATGCACTCGAAGGTGTCGTGCTTGAGCCCAAGCTAGCTGATCGTCTGCGCAGTGTTGCTATGTCTACGTTTAACACAAAGAAAAATCGCGCGCCCTTTCGCCACTTGCTGCTACACGGACCGCCTGGTACGGGTAAGACGCTGTTCGCTAAGGCTCTGGCACGTCACTCCGGTTTGGAGTACGCCATACTCACGGGTGGTGATGTCGCCCCCCTCGGCCGTGAGGGAGTGACAGAGATCCATAAGCTATTCGACTGGGCATCACATAGTCGTCGGGGTTTGTTGTTATTCGTCGATGAAGCTGATGCATTTTTGCAAAAGCGTAGCAACACTGTCATGAGCGAGGACATGCGTAACGCTTTAAACGCCTTCCTTTATCGCACAGGCGAGGCCAGCGACAAATTTATGATAGTGTTTGCTAGCAACCAGCCGGAGCAGTTTGATTGGGCTATTAATGATCGCATTGATGAAATGGTGGAGTTCCGCTTGCCCGGCTTCGATGAGCGCGTACGAATGCTAAAACAGTACTTTGACAACTACATCCGTGCAACCAAGAATTCGCGTGCTAAAAAGATTCATGTGAGGGTGTCGTTTTTACAATAGACCCTGTAGCCAACTTTCTCGTGCCAAAACGCTCATCTTCCAGCTTGAAATATTGTCGGGCGTCTCCGCTGAAGCTCAAGATATCTTTGATGCTCTCAATAAGAGCCCATGCGCCTATACTGGATTCTCTTTGCGCTTGTGATGGGCAATGGTCTGTACACCTATGTGACGGGTGAAGACGAATGGGTTTTGGTCGAGAAGCTCCGGCCACCGATCCCGTCTGAGTACCGCATCTCGGAAAAATGGGACAAGTGTATTGAAAATTTCACACTCTACTTTGGCGCTGGTCTCGTCGCTGGTGGCCTCACGTCTCTTGTGTTAGCGCGTTCTGGTACTGGCCGTGGACTGATTACAGGTATCGGTGTTCCGGCGCTGGATCGAGTTGGACGACCTGTCAGATGGCGTTCGCGGGCGATGAAAAGGCAGCGCTGGACAAGTCGGACAAGGCTTTAAGTGACTTGACGGCGTCCCACCACCAGACGATCGTGGATTTTGTAACGCCCTCATCCACGCAACAGCAGCTCTTGGTTGTACACTCGTGGTTTCTGTGACAAGGATAATGTAAAGATCCTTGAGGCGTATCTGGTGGAGCAGATGCAGAACTCCACAGTGGACATTAGTGCTAACCTGGCACTGCTCAAGCTGTATCAGTTGTACCCGGGCACAGCCAACGCAGAAAGCCAACGTTTTGGCCAAGGGTGTTATGATATTGCCGTCCACCTTCTTCACTGGTGTCCACAATGATCTCCGAGAGTTGTCGCGCGGATACCAATGTAACGGGGATATTGGGTGCGGGTTTTATGCTTCAGTCGTGTCTCTTCGAGGATTTCTGGAAGGAACACCTAACGTTTGCCAGCAGGGTACCCGGATTCCTCGAGTCAGTACGAACCTTCATACTGAAGGTCGTCAGCCAAAGCCACAGTGTGATTTCTACGGATGTATTGAAATTGAAGCTTAACCTATCTGATAACGAAGTAAGCGATATTATAGCGGAGAAATGGAAGTGGGTAATCTGATCCAAATTAATCCCAACGAAGACAACCAAATGCAGTTTAAAAAAATCCAGGAAAATATCGAGCTCGATGGCGTGCTCAAGGTCATTCACACCCTTTCAAGATCTAACGTCTGTGCTAATGGCGGAGGTGTCGCTGATGCGGAAGAAGTTTGTGATCAACTGGCTAAACATTTGAGACTTGCGAGCGGGACACAAATACTACCATGTTCTACCGGTGTCATTGGCTGGCGAATTCCTGTTGACGCGATGGTGGAAAATTTGCCAAAACTTGTCAAGAATTTGCAAGCTGATACCATATTACCTGCTGCTGAGGGGATTATGAC---------------------------------------------------------------------------------------------------------------------TACAGATCTTTCGATTCCTCGAGAAGTGCTTCGTCAAATATTGAAGGAAGCGGTGGAAGACACATACAACTCTCTCAGTGTTGACACTGACCAGAGCACGTCGGATACGGTGGCATTAGTGTCTTCGGATCAAATTCCGTTCGATCTAAA---GGACTTGGATGTTTTCAAGGAAGCGTTGCGAACCGTGTGTCTCCGATTAAGTGAAGATATTGTGCGCAATGCTGAAGGTGCACATCACGTTATCCGCGTGCGTGTGGCTGGTACCTTGTCGAAAGAAATGGCCAAAGGCGTTGGTAAGTCTGTCGTAAACTCGCCTCTGCTCAAATGTGCAGTGGCGGGCAATGACCCAAACGTGGGTCGTCTTGTCATGGCGGTGGGCAAGTACATGGGTTTGCATCATAAGGACGTAAATATTCAGCGCCGGATGAAAATCCGTATGGGTGGAGTTGTTATTTTTGAGAACGGCGAAATGGTGCTGAACGACGACATTGAGGCGAAACTTGTGGCTCACATGCGCGATGCAATGCTCATTGAGCCTACTCGAGGTGGACTTGATGCCAGCTCGCATAACTTTCCACCACATGGAAAATTTGTTGAGATTGAAGTTGATTTGGGAGTTGGATCTGAGGAGACCCAAGTTCTGGGCATTGACCTTACCCACGAATATCTCATGGCGAAAGAGGTA-----------ATGGCGCC---GAAAAAGGGAAAGACGGCTGCCGCGTACCTGGCATCTCTTGGGGTTGCCCCCGT---CACTGGCGGAGAGAAGAAAGATGAG---AAGAAGAAGAAGAAAAAGGGTAAGAAGAAAGTGGGAAGAAGCCCATGTCGGCTGCGGCCAAGGCGCTGA-TGGAACGTCAGGCCCTTTTGAAAGCCGAGCAGGAGCGAATAGAGAAGGAACGGTTAGAGCAGGAGCGCATTGCCAAAGAAGAGGAGGCCAAGCGTCTGGCGGCCATCGAGGCGGAAGAGGAGCTTCGCCGGAAAAAAAAGGAGGCGCGGGCGGCTAAGATTGAGCGGCAAAAGAAGGAGGGAACGTACATGACCAAAGCCCAGAAACAGAAGGCTGCGAAGGCTCAAGCGGCTTTGGAGGCAATGCAGCAGGCAGGTCTTGTCCCTGAGGCTACT------GGTGAGAAGAAGAAAGTGGTGTTTTCGGATAAAAAAAAGAAGAAAAAGAATACTAGGAACAAGAGAGTAAGGAAATTGAAGCTCCAGAAGCGTGGGATGACGAGGATTGGGAGACGAGCGATGTCGTGGCGTCGCTGGAAGATAAGATAACAGAAAAAAGAGCAGGAGCGATTACGGCAACAGGGTATTGCTCTCAAGAAACGTCAAGAAGAATTAGCTGAGCAGATGCGTCAGGAGGCCGAGGAGGAATGTCAGCGGTCCAAGGCAAAGCTCGAAGCCGAGCGCAAGAAGAAAGAAGCGGCCGATCGTCGCCGCGAGCGGGAAGCTGCCGCACGAGCCAATGTCTCTTTGGACAACCTGCGCTCGCCGATTTGCTGTATTATGGGTCATGTCGATACGGGTAAGACGAAATTGCTGGACAATATCCGGAAAACAAAGGTGCAGGACGCCGAAGCTGGAGGCATTACGCAGCAGATTGGTGCGACCTTTTTTCCTGTGGAAGCGATTAAACAAAAGACGGCTCGACTCATTGAGACGATGAAATTGGAATTTCGGTTGCCTGGTTTGCTCGTGATTGATACGCCTGGTCACGAATCGTTCACCAACTTGCGTACCCGTGGGTCGTCGTTGTGTGACATTGCCATTTTGGTCGTTGACATTATGCACGGTCTCGAGCCCCAAACCCTCGAGTCTTTGCGCCTCTTGCGTCAGAAAAGGGCGCCGTTCGTTGTTGCGCTCAACAAGATTGATCGATGCTATGGCTGGAAAACGATGCCTGATATGCCCGTCCAGGAGGCGCTCAAGTATCAGAACGAACACGTTCTTCGCGAGTTTGAGGACCGCGCCAAAGCGATTATCGTCGAGTTTGCCGAGCAAAAGTTGAATGCTGAAGTGTACTGGCGAAACAAAGACTTGGCGCACACGGTCTCGCTCATTCCCACGAGTGCTATTTCCGGTGAGGGTGTGCCCGATTTGCTCATGATGCTGACGCGATTGACGCAGGAGCGGATGGCAAAGTCCCTGGCATTTGTCGACATTTTGCAGTGTACTGTACTCGAAGTGAAGGTAATCGAGGGTCTTGGTACGACCATTGATACGATTCTCGTGAATGGGACGCTGGAGGAAGGCGCGACGATCGTCGTGTGTACATTAGACGGTCCTGTGGTCACAACGATTCGATCCTTGTTGACGCCGCACCCGATGAAAGAAATTCGAGTCAAGGGCGAATACCTTCACCATCAAAAGATGAAAGCGGCCATGGGTGTGAAAATTTGTGCGCAAGGGCTCGAAAAAGCCGTCGCGGGGACTCAAATTTACGTCGTGGGGCCTGACGATGATGTCGAGGAGCTCAAGGACTCGGTCATGTCGGATCTCACGAGTATTTTGGACTCGGTCAAGGCCACTCGGCGCGGTGTCATGGTGCAAGCGTCGACACTGGGTGCGCTCGAGGCGCTTTTGGAATTCCTCCGTACGTGTGACCCGCCCATTCCCGTTTCATGTGTCAACATTGGACCCGTGCACAAGAAAGACGTCATGCGCGCGTCGGTGCAGCTCGAACACCAGCCCGAGTTTGCAACGATCCTGGCGTTTGACGTCAAGGTGCATCATGACGCCACGGAGTTGGCATCCGAGCTCGGGGTGCGGATTTTCACGGCGGACATCATTTACCACCTCTTTGATCAGTTTACGGCGTACATGGAGAATTTCCGTATGGCTCGGCGCGAGGAATTCGAAGAAGTGGCCGTGTTTCCGTGCGTGCTCAAGATCCTGCCGACGTGCATCTTTAACAAGAAGGACCCGATTATTCTTGGTGTGGATGTCGAAGAAGGCATCCTCAAGGTGGGGACGCCGCTCGTCGTCCCCTCCGCCGGTG---GGTTCTTGGTGGGCAAGGTGGGCTCGATTGAACGCGAGCACAAGGAGGTGGACCGCGCGAAAAAAGGCGCGTCCGTGGCCGTGCGCATTGACAATGAGGGGAGTGTCATGTATGGCCGACATTTTGACCACAAGCACAAGTTGGTCTCGCGCCTGACGCGCCAAAGCATTGACGCATTGAAAGAGAATTTTCGGGAAGATCTGCAAAAGGAAGACTGGCAGCTCGTGATAAAGCTCAAGAAGGTGTTTGATATCATTATGGAAGGGTGGCTCACCAAACGTCGTGACCATATGAATCTCATCTGGCGAGACCGCTACTTTCTCCTGGACGGCAACCAGCTTCGCTACTACCGCAAACGTGGCGACCCAGCGCCTCGAGGGACGTACATTTTAACCGATGGGTGTGTCGTAAGCAACGTCTTCAACTCTGAGGAGAAACATAAACATCACGGCCCCGTGTGGATGTTTCGCATTACGTGAAAAATCGTACAAGAAGGACCGGTTCCTGGATCTAGGGGCCAAGAGTGAAGAGGATGGAAAGATCTGGAAACGTGCGTTGGAGAACGCGGTGCGGTCCCTACGCGAGCTGGTCGCGCATCAACGGAAACTCAAGACCGACGCCGAGAT---TTTTCCAACACCGCCCCAT---------GCGCCTCGCATGCTCAAGACGGACGAGGATACTGTGTCGCTCTTTAATCTCGACGCCGATAATGTCAAGGCCGAGCAGGCGTGGTGGATCGCACGCGTCGAAGGCGGGCTGCGCATTCTGCAGGAATGTCCCTTGGGACCCAATGTGGCCGCGAATTTGTATGCGCAACATGCCGCTACGACCAATGGCTTGATCTTTCGTGCCCTCGTGGGCGCCGCCCTGAGTTTCAGTACCATGTACTCGTTTGCCCACAACATGTTGCTCAGTTTCGTCACGGCCATGGCGACGATTGTCGTGTGTTGTGTGGCCGTGACGCCGACGCAAGCGCCCGAGATCCCGAGCTTTCGTGTGTCCCAAGTCGTGCATGGATCTCCCACCGAAGTGTTTCGACTTATTATGAACTCGAAACGATTCCAGCGATGGGATGCTGCGACCGCTACGATGCGTGTGGTGCAGCAGCTCGATGACCAGGCCGATATCGTCTACGTGACCCAACGGCCGACACTTTTGTGGCCATTGTGGCAGAAAGCACGGGATCTTGTCTTTATGCGCTACTGGCGACGAGAAGAAGACGGCTCGTACTTTGTCATGTGCCAGAGTATCGATCATCCCGAGTGTCGCGTACGTCACAACTATGTGCGCGCGAATATTTTAGGTGGTGGCTTTGTCATCGCTCCCCAACGTGTCCCCAGT---GGATCCATTCGTACCCTCGTAACTTACGTCCTTCGGTACGATCCTGGCGGCTG---GTCTCGTATTTACCACCTGCTCGGGATGGATGTGGACTTGGTCATGCCCATGCTACGCAGTGTCGTGGGGATTCGCGATGAAATGAGTGCGACCGACTTTATCACGCCCAACGTGTCCGTGGCGCCATCTGGAGACGG-------GGGTCAAGAAGCATCGA---CTCGATGGGTGTCGTCATGGGCGTCCAGAAGCTTCAG---ACGAGTTTGCCCGAGAAGATGTGGGCCGAGCCGGATGCGTCGCTCTTCAGTGTGCGCGGGCACAATTACCTGAGTGACAAGAAGAAAATCCCCTCCGCGCCTGCCATGTTCCATACGGTAGGAGTGGATCTCTTGTCGTTCGAGAATGCCAGCGAGAGGTACCACATTTCGTCGCGGCCCGACAGTATTGGCTGCACGAG---CTCGAAATTTACGTTTGTGGTCAACATGATCATTCCAGGGCCCGACAACACGTGCATGGTCTTCTACTTCCATCCTGTGCGTGACAATGTGTTTGAGGATGGCTCGCCCTTTTCGGAATTGCTCAACGATTTCTTTGATGGCGACGACCAGTTCCGGAACTCGCGCTTTAAACTCATTCCCACCGTGGTAGAAGGCAGTTTCATTATTAAACAATCCGTCGGCTCCAAGCCCACCCTCTTAGGAAACAAGCTCAAGTGTCTTTACTATCGTGGAGATAACTACTTTGAAGTCGACATTGATATTTCATCCAACTCAGTGGCCAATACAGTCGTTGGCATGGTCCAAGGCGTGACGAAATCACTCGTGGTGGATATGGCGTTCCTGCTCGAAGCCCAATCCGATGAAGAGCTGCCGGAAATTATTCTTGGCGCTGTGCGTCTGCAGCACGTGTCGCTAGACAACCCGCGTCGCATGCCCAAGCATGGGAAACGGGCAACATGGATGAGCTCAGATTTTGAAGCTTTTACACAAGTTTTGCGAGTAAATCTGCAGCGTTTAATAGTCTACGCACGCTCAGCGGATGCATCGCTGCAGCGAGAAGTCGCCGAGAAATTGGCCAACGAAGCTGTTAAGATCGACAAGTACAAATTGTGGAGTTGGACGGACTGCAGCTGTTGCTGCCATTGACCAAGTCCAAGGATACTGAGGTACAGCGTCTGGCAGCGCATGCTCTGGCCAACCTTTCGGTTAATTGTAAGGGATGTTGTTA-------------------------------------------------------------------------------------------------------------------------------------------------------------------------------------------------------------------------------------------------------------------------------------------------------------------------------------------------------------------------------------------------------------------------------------------------------------------------------CCTAACGAGGTGCTCATGCGTTCGTCCCCCACCCTCTGCTG---GCCGTGGTACCCGTAGCCCGCGTAGCCGAACTGAAGGATGAAAC------TGTGCCATATAGTGGTGCTATGGTAGATGGGGAATACTCAACTACACTCGAAGCGGTT---------GGTGTGTCATCAACAGCGCCCT---ACGACGGGGCTTCA---GTTTTTTTTTCTATCGCGGATCGTTTTCCACTCTCCAAGGTTGGAAAAGTTAGCGGCAGCATAGCACCCAGTTCAGTGAGACG---CTTGCTGAATG---ATTTCCCCGAGATGATAC---AAGATGACGAA-------------ATGACAGTGCTA---AACGAAGCCATGTGGGATGACAAT---ACAGAAAATTCTGAG---GTGAACGCTGAGGGACGTTTACCGGTACGAAGCTTCGGA--CATTGAAGAAAACGAGATGGAGCAACTTCGATGGAACATTCAAGAACAGGTTTTCCACCGACATTTCCTGCAACGACAGAAGTACGAGGAGTGGGGAAAGTTAGAGGAGAAAGACTCCGAGTATGTTTTGTCCGGGGAGGACGACGAAAATGTATGATAGCCGAAAAACGATTCCACGACAAAGCCTATCGAGATGCAATGGGCAAAGGAAATGCCAGCTGGGTTAAGGAGCGTCGAGATCTGTTTGAGGAGGAACTTCGTCAGTTTTAAAAAGAAAAGAGGCACAAACAGGAGTTAATGCTTACACATCTTGAAAGAGTATCACCATTCTCTAAATTCCCAATTCTTGGCAACCGCTTCATACCTCTTCGTCTGCGTGGAAGAGGTGGAAATGGAGAAGTATGGGATGTCATAGATTATGCCAACAACAAGCAGCTATGCGCTCTCAAGCTATCAACTTCAATTAACCACGCTCAACGCGAACATGATACCCACACTCAACCACCCAA--ATATTGTTCGGGTAGGCGAAATGCCTTTTCTGATTCGATATCAGCAACAGTTGTACACGGCGTTCACTCTTGCTAGGGTGGAATCAGACTTACTACAAGTCATTGAAA---CGCACGAGTATTTGGACGAAGACTCAGCATACAAGGTACTTTCGCAGCTTCTGAGCGCTTTAAAGTATTTGCACGATTGGAATTGCTCATTACGATCTGAAACCATCAAACATTCTGATCGACCGCGACTGGTATGTGAAGCTG------ACCGATTTTGACTTAGCGCGATACGCAAAAGCAGCTACGTCCAACTCTAAGGTTGGCACTCTCCGGTATCTTCCTCCCGAATGTTTCCGTCCAAATCGGAGTGA---------TTGCGAAGCCACAGCTGAGAAGGCGGATACGTGGATGGTAGGAATCATTTACAATATGATGCTCACTGGTAAACATCCAATTTGCCCTGATAAATCGACGCAAGCGGAAGTCAAGTCTGCGCTGGCCCAGTACACAGGC---GAGCTTCGATACGCCCGACATATTTCCGACCTATCTCGATGGGTTTTACAAGGATGCCTTCATCCAGATCCACGCTGCCGACCAACGGCTGCACAACTATTGGTGGCGCTAAAT

Phytophthora_boehmeriae CGGACTACCACCAGCAAGTGGCTCGAGAGCTGGCCGCGTTCTTCCACGACTTCTACCGCTTCGGAGCTGCCTCCAATCGCTTCGATCTGTTCCTCCACAACGTGCTCTTCGGCTCGCTCTGCATCGGAGCCGTCGTGTTTCTCATGCTGTGCGTCATCGTCGTGCGGCGATGCATCCTGCACATCCGCAGGCATTTGTACGACGCACAGAGATGGACTCCACGTCTGTGGAGCTCATGGCAGTGGTGCTGTTGACGTTCTTAGCTGTGAGTGCTCTGGGGGGACTCTTGGGAGAAGCTGTTTACAGACCAGCGACGCCCTTCGTGTCACTGCAGACGACTTGGTGACATCTTTTAATGAGTCGGAGCTACCTGCTGAGGCCTATGAACTCTCGGTGGAGTCTCTGGACTCCTTCATGCGTCTAAAGAATTGTGGAACCAGACAGACGCGCTACTGCCCCAGAACTACTCAGATATTGCCAAGGAGTGGGAGTTCAGCTACTTCGTGCTCAAGTCTTCGACCAACGGGGCAATCCTGGCAGTGGCACTTGCCAGCTTCTTGTCCATTGCGTCGGTTGGTTGGGCGCTGGTGTCGCCACTGCGTGTCTCCATCTTGGTGATCTTGAGTGTTGTTCCTATCTCACACACCCTCATTGGAGTGTATTTGGCGTCGACGATCATGACGGCGGACTTTTGTGCGGCGCCGATGAACAGCACGCTAGAGCTGCTGGACTCATGTCCGGCTAACGCGTCCTGCCTTTTCACTACTACACAGCCAGACTACGCTTCGCGCCACGGAGAGGTTGGTCTTCGGATGAAG---GAGTACCTGGATCCGATTGGCGCAGCTCTGGGGGTTATGCTTGGCTATTTTGCAGGCACAGTCTCCAATTGGGAGAGCTTTTCGCTCATACAGACGTGGCGCTGGCCTTTTCTCGTGCAATTCTTGGCACTGCTCCCTATCAATGTCGCCATTTTCTTCGTGCCCAAGAAGCATCTTGTGATTCGCAGTGAGATCGACGAATTTGAGCCGCCCGTGGCTGGAACGCCCATGAGCACGAGGTCGACTACCTATCATGACGAGGAGGATGCTCTGCTTGCCAGTGCTAGTATGCGACCTATGGTGCACTGGACACGAGGTATATGAACAGGGGGCATTTTCTGATGGTGTGTACGAACTACTCCACATTCCAGTGTTTTGCCTGATTGTGTTCGGGCTTACAACCGTGTACTTTGTGGTCACGGGTGTGCAGTACTGGAGCACAATCTTTATGATCAAGTCGCTTCATGCATCCAAGTATTTGGTAAACGGTTTATTCGTCGTGGTGTCCGGGACAGGGCCCATTTTGGGCGTGTTCTTTGGTGGTTGGATTATCGATCGATACGGTGGATATATCGGTGTGGAGCAGCGAGCCAAGGCGCTGGGCATTTGCATGATCTTGGGTCTGATCGCTTTCTCTATCTCGGCGGTGACAACGTTCTTCGATGACGTTTACATTACGTCGGGCTTTCTGTGGCTGCTACTCTTTTTTGGGGGAGCCATCCTTCCAGCACACCGCGCACTTGCCTCGTCATTCTCCGTCATGGTCTTCAATCTGTTTGGTTATGCGCTCTCGCATGAGCGACCTGCAGATGGTGGTGGGGGCCAAGTGCTTCGTGCCGGACGAGACGCACGTGTGGCTCGCAGCCGAAGTGCTGCGGGACGAGAACTCTGGCGACGGCAAGAGCCGCAAGATCTACTGCACCGTGGAGCTCCCCGACGGAGACGGAGGAGCGATGCGTCGACATGCTCTCCAAGAAGACCAAGGCGCTGCTGACGTGCACCAGCTCGAGTCTCTGCCGTATCAGAACGAGAACTTGGGACCTGAAGGCATCGAAGACATGATCACGCTCAACTACCTGCACGAGGCTGCCATCCTCTACAACGTCAAGAAGCGCTTCCTGCAGAACCTTCCTTACACGTACACGGGCGACATCTGTATCGCTGTGAACCCGTACCAGTGGCTCCCAGACCTTTATGCGGAGGAGCAGCACCTCCGGTACCTGAACCAGCCCAAGGAGGAGCTCCCCCCGCACGTGTACGCCACGTCGGTGGCTGCCTACGACAACATGAGACGCTCTGAACGCAACCAGTCCATTCTGGTGAGTGGTGAGTCCGGTGCCGGTAAGACGGAGACCACCAAGATCCTCATGAACCACTTGGCTACCATCGCTGGTGGTCTGAACAACTCGACCATCAAGAAGATTATTGAGGTGAACCCTCTCCTGGAATCCTTCGGTAATGCCAAGACCGTGCGTAACGACAACTCCAGTCGTTTCGGTAAGTTCACGCAGCTCCAGTTCGACAAGAACGGCACGCTTGTGGGCGCCAAGTGCCGCACGTATCTTCTGGAGAAGACGCGTGTCATCCAGCACGAATCCCCCGAACGCAACTACCACATCTTCTACCAGTGGAGTCCGCCGATATCGCCCCGGAGCTGCTGCTGGATCCGTCCAAGGTCTATGCGTACACGGGTGACAATACGGTGCAGAAGATTGAGGGGATGTCTGATGCCACTCACTTCAACAAAACGCGCGAGGCACTGACATTGGTTGGCCTCTCGCCCGATGACCAGAAGCCGCTGTTCGAGGTTCTTGCTGGTGTGCTTCACCTCGGTGAAGTCAGCTTGATGTCGGACCCGTCGGATGATGAGAAGTCTCTCATTACCGAAGGTGATAGTGGTGCAGAGGCCGCGACCAAAATGCTTGGTGTCACTTGGGAGGCACTGCAGACTGCGCTCTGTTCCCGTACCATGCGTGCTGTCAATGATGTATACAGTGTGCCGCTGAAAAAGGAGTTGGCGATGGACTGCCGTGACGCACTCGCCAAGGCTATCTACTCCAACGTGTTCGACTGGCTTGTTGCTACGATTAACCAGTCGCTTGCTGACGATGCCAACATGGCGAACCACGTGGGTGTACTGGATATTTTCGGTTTTGAGCACTTCAAACATAACTCGTTCGAGCAGTTCTGTATCAACTACGCCAATGAGAAGCTCCAGCAGAAGTTCACACAGGATGTGTTCAAGACTGTGCAGATTGAGTACGAAGAAGAAGGCATCGTTTGGGATCACATTGAGTACGCAGATAATCAGGACGTGTTGACCGTGATTGAGTCGCGCATGGGTATTATCTCGCTGCTTAACGAAGAGGTCATGCGGCCGAAGGGTAACGAAGAATCGTTCATGTCGAAGGTGACCTCGCTGCACAAAGAT---GACATGGCTCACGTTATTGAATTCCCCCGCACGTCTCGCACGGAATTCCTTATTAAACACTACGCTGCCCCTGTCATGTATGATTCCGTTGGATTCCTGGAAAAGCACAAGGACTCGCTGCTGCCCGATTTGTCGGAGCTCATGCGTGGTTCGTCGACACCGTTCATCTCTAAGCTCTTTGAGCCGAAGCCGGAGCCTAAGTCGCCGAAAGC---------GCGGTGGAGCCTTGTCCATCACGACTGTCGGAACACAGTTTAAGGAGAGCTTGACAGAACTGATGGCCACCATCAACTCAACGAAGGTGCACTACGTGCGTTGCATCAAACCCAACCCGATCAA---------GAGCTCGACAGTCATGGATCAGGAAATGGTGGTATCCCAGCTGCGTTGTGCTGGTGTTATTGAAGCCATTCGTATTTCCCGTGCGGCTTATCCCAACCGCCTACAGCACATTGAAATCTTGGACAAATTCTGGCTGTTTGTGCCAAGTGGAGGTGAGACGGCAGCGGACAAATGCCAGCTTCTCATGGACAAACTGAAACTCGAGACGCCTACCCAATACCAAATGGGTAAGACCCGTGTGTACTTCCAGTTGGGAGTACTTGAGGAGCTAGAGGACCGCCGTAAGAAGTTCCTGGACGCCAAGGCCACGTACCTGCAGAACATCATGGTGGGTTTCACCCAGCGTATCAGATACTTACGGCAGCTGGAGGCGATCATTAAGCTCCGTCATTCGATGCGTGATTGCTATGCGGAGATACAACACATTCCACAAGGGCATCGTTTTGGCGCAAGCTCGGTGGAGAGGTATTCAGGGACGCAAGTTGGCTGCCGACGTGAAGAGCAACCACTACGCTGTCATCATCCAGCGTTACGTGCGTGGATTCGTTAAGCGTCACCGCTACGTTAAGATGCGTGCGATGGTCATTCGTGTACAGGCTGTGGTCCGTATGACCATCCAGCGACCGAAGTACCTGGCTGCTCTTGAGGAGAAGCGTCGCGATGCTGACATGGCATACCAGCTTCAGAAATTGAAGGCAGCTTTGCAGGAGGAACAGGAGCGTAATGCGCAGCT------GCAGCGTC---GCAGCAGTGTTAC--TACGTCGCGACTGCTGTTTCAAACGAGGTAATGGCTGACGCCGGCGGCATGATTGAGACTCTCACAAACGAGAACACGAAGCTTCGTGAAAAGAATGAGGAAATGAAGGTATCGATGAAGGGATTGAAAAAGGAAATCGAGAAGTTCAAGAGCGACAAGGAATTCTCGTCGGCAGGCAACCACGTTAAGGTCCGCCAGCTTCAAGACACGGTGCGCGATAAGGACAAGAAGATTAGTCAGCTCGAGGCCGAGAACAAGAAGTTGACAGAGCAGCTGAACAAACTTCAGGTGGAGGG---TGT---TCCCGAGAAGAAGACGACACCGAAGAAGTCGATCTTCCGCACCCTGGGATCTAAGAAGGAGAAGAAGCCGC---------------GCGAGACTGTGCTCATGGATTCGCTGACTGGTTCGGACGACCTGAACCGCGGAGAGACACGTCCGTCAGA---GCGCCACTTCCCGCGTATGC---CCAGCATGAGTGTGGGCTCC---CGCTTCTGGGGCTCAAACAAG---AAGGGAGAT------------AACGGTGACGGCTTCGACGTATCGGAGGTTGATGGTGAGGACAGCCCCACAAACCAACGCGCAACACAATCGTGAAGAGCATGGAGGTGGGTGTGTCTGGTGCCATGTCCACTCTGAAGGGCCGGATG---------TCTGTCGTGAAGGGCATGTACGAAACAAGCGTAGCAGCAGGACCGTCCCGTGCGTGAGTCATTCAACCTGGACGCACTTCCGGAGGTGTCGCTTCCGGTCGGTTGGGAGGCCAAGGTGTCTCGCTCCACCGGCCGCGTGTACTACGTGAACCGTAAGCTAGGCAAGTCTCAGTTCGAGCGCCCAACGCTGGCCTCGCTGAA------AGCGCAGAAGCTGGCTCGTCAGAAATCTGGCAACGCGCTG-AAGGCGGTTGTGGCAGATTTTGGATGCGGAGATGCACGACTCGCTGAGAGTGTGTCAAACAAGGTGCACTCCTTCGATCTGGTGTCACTCGATATCGGCGTGTACTGTTTGGCGCTGATGGGGAAAAGCGTTCGTGAATATGTTCGTGAAGTGCATCGAGTGCTCAAGCCTGGAGGAGTGTTGAAGGTAGCCGAGGTCAAAAGCCGATTTGAGAGCGAGTCGCTTGGAGGATGGTGGCGGTGTTCGTAGCTGCTGCGGATGACGTCGTGGTGACTGTCCGCAACACGACGGTGTTCGTGAAGAGACTAAATACGCAGTTGTTGTGGGCCAGAAGACGGGCGAGGCAGTAGCTGTGCCGTTTCCCGTCGTGGACGGAGACCTTAAGACACTGCTGGGACACACGACGTCGATGGTCACTCACATTGCTGTTAACCTCGTCGCTGCTATTGACGGCGGACAGAGATGAGAAGATTAGAGTGTCGAGGTTCCCTACTGCTGCTATCATTGAGAGCTATTGTTTGGGCCATGCGGCGAGTCTGACCAAGGTGGCGTGCAGTGCGCTCACGCCCGAGCTGGCGGTGTCCACGTCGATGGATAACACGTTGAAGCTGTGGGAGATGCAGTCGGGCAAGTTGGCGATCTGTCCCAAGACGATGTTGTGGCTGTGCTTGTAAACTATGAGCACGCACGGTTTTTTGAGATCGTGAAAACCCGTTCTTGCAGCTGTACACTTTGACGAAGGACAGAGACTGCATTCAGCCGCTGTGCGCGACTTCCGCAAGCTCCAGAACGACCCGCCGTCGGGTGTCTCGGGCGCGCCAATGGACAACAATATCATGCTCTGGCAGGCCGTCATCTTTGGTCCCGACGACACGCCGTGGGAAGGAGGCACCTTCAACCTAACGCTGGAGTTCAGCGAGGACTACCCGAACAAGGCGCCGACAGTCAAGTTCATCACGAAAATGTTCCACCCCAACATCTACAATGACGGACAGATCTGTCTGGACATCCTGCAGAACCAGTGGTCTCCCATCTACGACATCTCCGCCATCTTGACGTCCATTCAGTCGCTGCTGTGCGACCCGAACCCGAACTCGCCAGCAAACTCGGAGGCAGCGCGACTCTTCCAGGAGAACAGACGGGAGTACAACCGTCGGGTGCGCGAGATCGTCGAGCAGAGCTGGCAGGCGATCGATGGTGTCGGCCGCCAAGATGCGTGGCGCCGAGAACCGCATGAACGCCGGCCGTCCCTTTGTGGCGTGGCTGGACAAGGTCTCCTCGGACCGCGGACTGTGCGGCGGCATCAACTCTGGCATTGCCAAGACCACCCGCAAGCAGCCAACACTCAGATCTTCGTCATTGGCGACAAGGCTCGTGCCCAGCTGCGTCGTGACCTCGGCGACAACATCCGCGGCAACGTGACGGAGACGTTCTCGTCGCCCCCGAACTTCACGGTGGCCAGCGCCATCGCGGAGGGGGTGGACAACGAGAGGAGTCCTCGCGTATGGCTGCCATGGAGAACGCCACCACGAACGCCGAGGACCTCACCCTCACGCTCGTGTACAACAAGGCCCGTCAGGCCCGTATTACCACGGAGCTGATTGAAATTATTTCGGGTGCCGCCTCCCTGGACGATCACTGGGAGCGTCGTGAGCGTCCAGAGCGCGGCGCAGTTCGACGAGGCGACTGCGCGGGAGTCGACTCTGTCCGTGACTTTCTTCTGGGCGGAATTCCAGCTCTGCCGCCCCAACGGCCAACTGGACGTGGGGCCGCGCGGTGCTGGACAAACTGGAAGGCGCCAATGTGGCGGAACTGGCCAAGCGCGTCTGCAGTACCGTCTGAAGAAACTCATCAGTGCGTCCCCAGTGATGCTCTTCATGAAGGGCAATCCAACTGAGCCAAAGTGCGGCTTTAGTCGGCAGATGGTGGCGCTGCTGAACGAGGAGAAGATTCAGTTTGGCACATTTGACATTCTCAATGATGAGGAAGTGCGTCAAGGCCTGAAGCAGTTTTCCAACTGGCCCACGTTCCCGCAGCTCTACGTGAACGGCTCTTTGGTCGGAGGGCTAGATATCATCAAGGAGATGAAGTCGGAAGGATCGATTGTGGAGCAGCTTGGCGTCGAGGAGTCAGAGGCGGCTTTCCAGGAAAGTTTGAAGGCGCTTGTCAACTCTGCGCCGGTTCTGCTCTTTATGAAGCTTTGACATTCTCTGTGGAGCTATCAGCGCCAGGACCAGGATATTGCAAAGGTGGTGAGTCTTCTTCCGTCCTCTTTCCGGCAAGAGATGTGCTGAGGGTATTTCGTGGAGCCCGAAATCGGCTGGCGATGGCATTTCGCGATCCGATTGATGGGAGAGGAGAATTCTGTACTGTACAGTTACCCGAATCACGTCTAAGCGTACTACACTGCATTCTCGAGGCATATCTACGATTGATTGGCAAGTGGAGTGTGCTTCATCGAGACCACCGTAGCGAAGTCAGATCTCTCTGTGTGCACTCTAAGCGCGACAGTTTATACACGGGTGGTCTTGATGGACTGCTCATTCGCTATGACATGAACAAGGCACGTACAGAAATTGTGATGGAGCGTCGCAAGCCCATTTCGAAGATCAATGCGGTGCTGGAGCATCCGCACAACCACAATTTGCTTCTGGTCAGCTGCGTGGAGCAGTAAGCACAACCTGCTCCTCCACGATCTTCAGCGATACCGTGGCGATATGTCAGGCAGTTCGATGAGCCAGTATGTTGTGCCGCGATGGTCGCCAGCAGGCTATCACGTATCCTGCGGCAGCAAATGCGCTACTGCCACGGCAGCTTCTCCACGCCCACGTCGGCGACCATCGGGGCCTCGTTCCTGCAGAAGCGCGTGATCGTCGCGGGGAAACTGACGCTGCAGATTTGGGACACGGCCGGCCAGGAGCGCTTCCGGTCCATGGCGCCAATGTATTACCGCAACGCGAAGGCCGCGATTCTGGTGTTCGACCTGCAGAGCGAAGCGAGCTTCGAGAAGATCAAAGAGTGGCTGCACCTGCAGAACCACGTCGGGGACGACATGTGGGCAACAAGAGCGACGTTTTGACTTTGCCAAAGTGCAGCAGTTTGCAGATGAGATCGGCGCCCTGGTGTTCCGTACCAGCGCCAAGACTGGAGAAGGAGTACAGGCGCTGTTTGAGAGCTTGGCAGTGCAGCTGCTCAAGAAACACGACCAGGAGCAGCGGAGAGCTTCTACGTGGTGTATATCCCTTCGGACGAGAGCAAGGCCTTGGAGGAATGGGCGGTGGATCTTCCTGCCGACAAGGAGGCGCAGCTCGGGTGTCTGACGGAGCGTCTGCGCGCGCACTTCAAGGCCGGCGCCATCCAAGCGCGAGCAGAGACTTTCAAGAAGCAGCTTATGAGTCAACTGCCCAAGGGCGCCACGATCACCGACGAGATGCTGCAGATGATGTATGGACAGTTTGGTGGACTCGGTCCCTCTGGTGCTTAACACGCCGGAGGTCAAGCACGTTTGAGGTCAAGGGCGATGCATTCATTGGTCGAGTCTTCGATAGACGACTTCGAGCGCAAGGACTTCCGGTTGAGTGAGGTTACAGGTGACGCGCCGTGGGTGCAAATGGGCAAGCAACAGAGGTGACGCAGTCGCTGGAGGAGATTCTGGCGCTGGGCCAAGGACTGGACGATCAGGACTACAAGGCGGAGCTGGAGGTGCTGCAGAATGACCCAAACTCGAATTTGTACTCGGCCGTCACGTTTGAGGAGCTCAATTTACCGGAGGCGCTGCTCAAGGGCGTGTACAACATGAAGTTCGCCAAGCCGTCCAAGATTCAATCGGTGGCTCTGCCGTTGATTCTCGCCGATCCGCCCGAGAACCTTATTGGCCAGGCTCAGAGTGGAATCCCACCCGCGAACTGGTGCGCCAGATTATGGCTGTAGTAAACGCTATGGGCAAATTGTTGAAACTTTTCTGGCCATCCCAGGCAATGACTTGGGTCGCGGAGAGTCACTCACAGCTCAGGTGATTGTCGGGACTCCTGGCAAGGTGGAAGGTATGATCAAGAAGAAGCAACTGGACACGCGCAACCTCAAGATCTTTGTGCTGGACGAAGCTGATGTCATGGTTGCCGAGGACGGCATGCGCGAGCGCTCAGTGGCTATCAAGCGTCTGATCAAGAACCGCTCGTGCCAGTTCCTTCTCTTCTCAGCAACGTACGCCGACGACGTGCGCGATTTTGCTCAGAAGATGGTGCCCGACCACAACATTATTACGGTCAAGAAGGAGAAGCTCACCCTGGATGGCATCAAGCAGTTCTGGATCGACTGTAAGACTCGTGATAACAAATTCCAGGTGCTGTCGGACATTTTCGCTATTTTGTCTATCGGCAAATGTGTCATCTTCGTACAACAGCGTGAGACGGCGAAGGAGTTGACACGCAGCATGCGTGAGAAGGGCCACTCGGTGGGCATTTTGCATGGTGCAGACATGGCCAAGGAAGTGCGTGACCAGGTCATCGATGAATTCCGCGCAGGCACGACCAACGTGCTGATCACGACAAACGTGCTCGCTCGTGGTATCGATGTGGCAGGCGTGTCACTCGTGGTGAACTTCGACATCCCGCTCACACGAGATCATCGCCCGGACCCGGAGACGTATTTGCACCGTATTGGTCGTACTGGACGTTTCGGCCGCAAGGGCTGCGCCATTAACTTCGTGTACGACAACAAGAGCAAGCAGGATTTGGCTGAGATCGAAGAGTACTACGCGCGCCCTATTACGCAGGCTCCAGCAGACGACATTGAGGAGCTGGAGAAGCTGCTTGCCTGGGGGGCAGCAAGCGCCGCTTGGCCAAGCTCGGCAAGGAGCAGCGCAAGGGCATGCGAATTTACCCTCGGGAGCCCAAGAAGAAGCTCTCGGGCAAGGACAAGACGTACTACTACATCAAGGACGTGATGTTCCTGGCCCACGAGCCCGTGCTCGAGAAGTTCCGCGCCTTTAAGACCTTCATGAAGAAGGTCAAGAAGGCCATGGGCAAGAAGGACTACGAAGACGCCAAGCGCAAGCACGAGAACCGCCCGACCTACGAGCTGGACCACATTGTCAAGGAGAGGTACCCGCGCTTTGTCGACGCGCTCGGAGATCTGGACGACGCGCTGTGTGGCAGAACTACGTGGTGGCTACTCGTGCGTTGACCAAGGTCTTTGTGAGCATCAAGGGCATCTACTACCAGGCCAATGTGCAGGGACAGACCATCACGTGGTTGGTGCCGCACCAATTCACGCCAACCATGGACAAGCGTGTGGATGTTCGTGTGATGCTAACGTTCCTGGAGTTTTACCAGGTTCTGCTCAGGTTCGTCAACGCGCAGCTGTACATGCAGATTGGCGTGGCGTACCCGCCCAAGATTAATCTGTCGTTGGATGCTGCTGGTGCCCAACTTGCTGCGATTGAGCTAGCAGCAGAAGGATTCGGAGAAACGCATCAAACTACAATGAGCAGCGCAAGGAACGCAGCGAGAAGAGTCTATTCAGCGGTCTTTGCTTCTTCTTGTCGCGTGAAGTGCCTGCGGCGTGCCTGGAGCTGGTGCTGCGCTCGCACGGTGCTACGCTTGGCTGGGATGGTGCCGGATCGCCTTTTGATGAGAAGAGCGAACGCATCACGCACCACATCATGGACCGCCCTCATCAAGGACACCGTTACTTTAACCGCGAGTACGTGCAGCCGCAATGGGTGTTTGACTCTGTAAACAACGGTATTCTGCTGCCTCTGGCTCGTTACCGCAAGGAGCTGGAGAAGCTCAAGTCTGCTTCACAGGTTCTGCGCGAGGTTGGCGCCGTGGGCGACGAGGACGAGGAC-AGAGGCGTACGCTGCGGATCTCAAGGCCGAGCTGGATGGCAACAAAAAGTCAAAGCGTGCGAAGAAGGAGGAGGAGGCAGCCGAACTCAAGGAGCTGGCGAAGACGATGATGTCGAAGAAGTCGAAGCGTCTGTACGACCGTATGCAGTACGGTCTAGGCAAGAAGGCTGACAAGATTCAGAAGCTCGAGGACAAGCGCGCCAAGCTGGAGGCAAGACGCGGAAGCTCCTCGCTGGCGCATTGTCCGGCGTGATCTCGGCGCTCGCCTTCCAGCCGCTTGGGCGGTCCGCTGGCGCGGCACTTCCCCCACTCTCGTGCGAGTGGCAGGCGGCGCCGGACTCTACTTTGCTACGCTGGACCACTGCATGAACCTGTTCCAGCAGACGGCAGTCAATACATTCCTAGCTGGAGCCTTTGCACGGACATTTGCAGGCGGAGTGATGTCGCCCTTCACGATCGTCAAGGCAAGGATGGATCCCTCCAGGCACTTTTACCAGCAAACGGACGTGCCGTTCTCCGGACTATATGTGCTCATTTATACCGCACCCAGCGGATGTGGTGAAGACGCGCATGCATACGACAATCAGTGGCAAAGATCTACCGCCATGAGGGGTTGAGTGGCTTCGCGAAAGGGATATTACCACGTGTGGTGAAACGCACTTTATCCACTGCTGTCACCTGGACGATTTACGAGCTACTCGTTCGACCCGTCGGGTCTGGGCCACGCCAAGGAAGCTTTCAACTTGGCCAAGGAGACGGAACGCACCAAGCAGATGGAGAACCAGGCCAAGATCAAGGAGAACGAGGCGCTCTACAAGCAGTACGAGATCGTGCGTGTGCAGAAGGAGGGCGAGGAGCGTCGCAAGACGCTGGAGGAGGAGACGCGTCAGCACCAGCAGCGTGCGCAGTACCAGGACCAGCTCAAGCGCAAGCAATACGCTGACCAGCTGGCAGCTCAGAAGTACATGAAGGAGCAGGAGCTCAAGAAGCAGGAGGAGATTCTCGCTCGTCAGGAGGCTTCGCGTCGCAAGACGCTCGACTACGAGGCTGAACTGCGCCAGAAGACGGAGCTGGCCAAGGTCGCGGCTGAGACGGAAGGTCGTATCAAGCAGGAGCGACTCAACCACGACCTGCACCTCGAGGAAGCACGTGTGCGTGCTAAGGAGTATCGTGAGACGGTCATGGAAGGTATCAAGCTGGCAGGTAACACTGTCGGATCGGGAATTATGGCCTTCGTGGACGACAAGGAGAAGCTCACTGCTACGGTGGCGTCGTTGACGGCGTTGGCTGTCGGTATTTACACGGCCAAGGTGTCCACTAGCGTTGCTGGCAAGTACATCGAGGCGCGCATGGGCAAGCCGTCGCTGGTGCGCGAGACGTCTCGTCGTTCGGCAACACAGGTTGTGGCGAACCCAATCCCGTCGATTAAGCGTGCGCTGCGTCTGCAGAAGGCGACCGATGCTCTGGAGGGTGTGGTGCTGGAGCCCAAGCTCGACGCGCGTCTTCGCAGCGTGGCCGTGTCGACTTTTAACACGAAGAAGAATCGTGCGCCGTTCCGCCACTTGCTGTTGCACGGCCCGCCTGGTACGGGTAAGACGCTGTTCGCCAAGGCTCTGGCGCGTCACTCTGGTCTGGAATACGCCATTCTTACCGGTGGTGATGTGGCCCCGCTTGGTCGTGAGGGTGTGACTGAGATCCACAAGCTGTTCGACTGGGCATCGCACAGCCGTCGCGGTCTGCTGCTGTTCGTCGATGAGGCAGATGCGTTCCTTCAGAAGCGTAGCAACACGGTGATGAGCGAGGACATGCGTAATGCGCTGAACGCCTTCCTGTACCGCACGGGTGAGGCCAGCGACAAATTCATGATCGTGTTCGCGAGTAACCAGCCGGAACAGTTCGACTGGGCCATCAACGATCGTATCGATGAGATGGTGGAGTTCCGACTGCCGGGCTTTGACGAGCGTGTGCGCATGCTCAAGCAGTACTTCCGTGACTACATTAGTGAGCCGAAGAACTCGCGGGCGAAGAAGATTTACGCGAGGGCGTTATCTTCACTCTTGATCCCGTCGCAAACTTCCTCGTGCCAAGACGCGGATCTTCCAAATAGAGACACTGCAGGGAGTTTCTGCTGAAGCTCAGACCATTTTTGACGCACTTAACAAGAGGCCATGCGCGTCTACTGGATCGTCTTTGGCGTCGTGATCCTCAACGGCGTCTACACGTACTTGGCAGGCAAAGATGAGTCGTTTTTGGTGGACAAGTTGCGGAGAAAGATTCCGTCGGAGTACCGCATCTCGGAGAAGTGGGACAAGTGTCTGGAGAACTTCACGCTCTACTTCGGCGCTGGCCTGGTGGCTGGCGGCCTCACGTCCCTCGTCTTGGCTCGCTCTGGCGCTGGTCGTGGACTGATCACTGGAGTGGGCGCGCAGGCGCTGGGTCGAGCTGGACGACGTGCCAAATGGCGTTCGCAGGCGACGCAAAGGCGGCTCTGGACAAGACGGACAAGGCCATTGGTGATTTGCCGGCCTTCAAGCAGCAGACGACCGTGGACTTCGTGGCGGCCTCGGCCACGCAGCAGCAGCTGCAGGCGGTGCACCCGCGGCTTCTGCGAACAAGCCAGCGTGAAGGTGCTGGAAACGTACTTAGCCGAGCAGGTCAAGGCTTCCAGCGTGGACATTGACGCCAACCTGGCGCTGCTCAAGCTGTACCAGATCTACCCGGCCACGGCCAACGCCGAGAGCGACGGTGCTGGTCAAGGGCGTCATGTCGCTGCCGTCCACGTTCTTCACAGGCGTCCACGATGGTGCCGGAGAGCATCCGCGAGGATGCCAATGTGGCGGCTGTGCTGAACGCTGGCTTCCTGTTGCAGTCGTGTCTGTTTGAGGACTTCTGGAAGGCGGATGTGACGTTCGCGGAGAAGGTGCCTGGATTTGTGGAGTCGGTGCGCGCGTACATTCTGGGCGCCATCAGTCGTAGCCACAACGCTATTACCACTGACGTGTTCAAGGCGAAGCTGAACGTGTCTGACAAGGAAGTTGCGGACATTGTGGCTGAGAAGTGGTCGAAAACAATGTGATCCAAATCAGCCCCAACGAGGGCAACCAGATGCAGGCCAAGAAGATTCAGGAGAACATCGAGTTCGAGGATGTGTTGAAGGTCATCCACACCCTTTCTAGATCCAATGTCTGTGCAAGCGGCGGGGGCGTTGCCGACGCTGAGGAGGTCTGCGATCAACTGGCCAAGCATCTGAGACTGGAAGCAGGCGCCCAAGTGCTGCCGTGCTCCACTGGCGTCATTGGCTGGAGGATCCCAGTGGACGAGATGGTGGAGAACCTCCCAAAGCTCGTGGCCAATTTGCAGTCGGACTCGGTGCTGCCTGCTGCTGAAGGAATTATGACGACGGATTTGTACCCGAAGGTTCGCTCGATGGACGTGTGTGGCGGTCGCATTGTCGGCATTGCTAAGGGAGCTGGCATGATTGAGCCCAACATGGCCACCATGCTATCCTACATCCTAACGGACCTGTCGGTCCCGCGTGACGTGCTGCGTCAGCTTCTGACGGAGGTGGTAGCGGACACGTACAATGCCCTCAGTGTGGACACGGACCAGAGCACGTCTGATACCGTGACGCTGGTGTCATCGGACCAGATCCCCTTCGACATGGC---GCAGCTGAACGAATTCAAGGCTGCGCTCAAGGAGGTGTGTCTGGGACTGAGCGAAGACATTGTCCGCAACGGTGAGGGCGCACACCACGTCATGCGTGTGAGTGTCCGTGGCGCTGTGTCCAAGGAAATGGCTAAGGGAGTCGGTAAGTCTGTGGTGAACTCGCCACTTCTCAAATGCGCAGTGGCTGGCAACGACCCGAACGTGGGTCGGGTTGTGATGGCTGTGGGCAAGTACATGGGCACATACCACAAGGACGTGAACATTCAGCGTCGTATGTCCATTCGTATGGGTGGCGTTTCAATCTTTGAAAACGGCGAAATGATTCTCAATGACGACAATGAAGCTAAGCTTGTGAAGCACATGAAAGATGCGATGCTGATCGAGCCCACCCGTGGTGGCCTCGATGCCAGTTCGCACAATTACCCTCCACACGGAAAGTTTGTCGAGCTGGAGATTGACCTGGGTGTTGGATCCGAGGAGACTCAGGTACTGGGCATTGACCTTACGCACGAGTACGTCGCTATCAACGCGGATTACCGCTCG--ATGGCGCC---TAAAAAGGGGAAGACCGCCGCCGCCTACTTGGCGTCTCTGGGTGTGGCCCCCAC---TGCGGGAGGGGACAAGAAGGACGAC---AAGAAGAAGAAGAAAAAGGGAAAAAAGAAACCCAAAAGAAGCCGATGTCCGCAGCTGCCAAGGCGCTTA-TGGAGCGTCAGGCTGCGCTGAAGGCGGAACAGGAGCGTCTGGAGCAGGAGCGTCTGGATCAGGAACGTATCGCTGAGGAGGAGGAGGCCAAGCGTCTTGCCGAAATTGAGCAGCAGGAGGAGATTCGCCGTAAGAAGAAAGAAGCTCGTGCTGCCAAGATTGAACGACAGAAGAAGGAGGGAACGTACATGACGAAGGCGCAGAAGCAGAAGGCGGCCAAGGCGCAAGCGGCGCTGGAAGCTATGCAGCAGGCTGGTCTCGTGCCTGAAGCGGCCGGT---GAGGAGAAAAAGAAGGTCGTGTACTCGGATAACAAGAAGAAGAACAAAAAGACCAGGAGGAAGAGGAGGAAGAAGCAGAGGCACCGGACGCGTGGGACGACGAGGACTGGGAGACAAGTGATGTGGTCGCCTCTTTGGAGGATAAGATAACAGAAGAAGGAACAGGAGCGGTTACGTCAGCAGGGTATTGCGCTCAAGAAGCGTCAAGAAGAACAGGCTGAGCAAATGCGCGTAGAAGCGGAGGAGGAACGTCAGCGTTCAAAGGCCAAGATGGAAGCCGAGCGCAGAAAGATGGAAGCAGCTGAGCGCCGTCGCGAGCGTGAAGCTGCTGCGCGTGCTAATGTATCTGTGGATAATCTGCGCTCCCCCATCTGTTGTATCATGGGCCACGTCGACACGGGTAAAACTAAACTTCTCGACAACATTCGGAAAACCAAGGTGCAGGATGCTGAGGCCGGTGGTATTACCCAGCAGATTGGTGCGACGTTCTTCCCTGTGGAAGCCATTAAGCAGAAAACGGCGCGACTCGCCGAGACCATGAAGCTGGAGTATCGTCTTCCTGGCCTATTAGTGATTGATACGCCCGGTCACGAATCTTTCACGAACCTTCGGACACGTGGTTCGTCATTGTGTGATATCGCCATTTTGGTGGTTGATATCATGCACGGTCTGGAGCCCCAGACGCTGGAATCGCTGCGCTTGCTGCGTCAGAAGAAAGCTCCTTTCGTGGTGGCGCTCAACAAGATTGATCGTTGCTATGGCTGGAAGACGATGCCCGACATGCCTGTCCAGGAGGCTCTTAAGTATCAGAACGAACACGTCATTCGCGAGTTTGAGGACCGTGCCAAAGCAATTATGGTGGAATTTGCTGAGCAGAAGTTGAACGCTGAAATTTATTGGCGGAACAAGGATCTGGCTCACACGGTGTCCCTCATCCCAACTAGTGCTATTTCCGGTGAAGGTGTACCGGATCTGTTGATGATGCTGACGCGCTTGACTCAGGAACGCATGGCTAAGTCACTTGCATTCGTGGACATTTTGCAATGTACCGTTCTGGAAGTCAAAGTCATCGAGGGATTGGGTACAACAATCGATACGATCCTCGTGAACGGTACGCTAGACGAAGGCGCAACTATTGTGGTTTGTACCTTGGACGGCCCTGTCGTGACAACTATCCGTTCATTATTGACTCCACACCCGATGAAGGAGATCCGCGTGAAGGGTGAATACCTTCATCACCCGAGGATGAAGGCTGCCATGGGTGTGAAGATTTGTGCGCAGGGCCTTGAAAAGGCTGTGGCTGGCACGCAAATTTACGTGGTTGGACCCGACGATGATGTCGAAGAACTAAAGGACTCTGTCATGTCTGACTTGACGGGCATTCTGGACTCCGTGAAAGCTACTCGCCGCGGTGTGATGGTTCAGGCGTCCACCCTTGGTGCACTTGAAGCGCTTCTAGAGTTTTTGCGCACGTGTGACCCTCCTATTCCTGTGTCGTGCGTGAACATTGGCCCCGTTCACAAGAAGGATGTGATGCGTGCATCTGTCCAGTTGGAGCATCAGCCAGAGTTCGCCACTATTCTAGCTTTCGACGTGAAAGTACACAACGATGCCACAGAGTTGGCAAATGAGCTGGGAGTGCGCATTTTCACTGCGGACATCATTTACCACTTGTTCGATCAGTTCACGGCGTACATGGATAACTTCCGTATGGCTCGCCGTGAGGAGTTCGAGGAGGTTGCCGTGTTCCCATGCGTTCTGAAGATCCTCCCCAACTGTGTCTTCAACAAGAAGGATCCGATTATTCTTGGCGTAGACGTCGAGGAAGGAATTCTTAAGATTGGCACACCTCTCGTGGTGCCATCCGCCGGAG---GTTTCCTGGTGGGTAAAGTGGCCTCCATCGAGCGCGATCACAAGGAGGTTGACCGCGCGAAGAAGGGCGCATCTGTGGCAGTGCGGATAGACAACGAGGGAAGCGTCATGTACGGCCGCCACTTCGATCACAAGAACAAGCTGGTGTCACGTTTGACTCGCCAGAGCATCGATGCGTTGAAAGAAAATTTCCGGGAAGACTTGCAGAAGGAGGACTGGCAGCTCGTCATCAAGCTCAAGAAGGTGTTCGATATCATTATGGAGGGATGGCTCACCAAGCGCCGAGACCACATGAACCTCATCTGGCTGGACAGATACTTCCTTCTGGACGGGAACCAGCTCCGCTACTACCGGAAACGAGGCGATCCTGCCCCCAGAGGCACGTACATCCTCACTGACGGCTGCGTAGTGAGCCCAGTGTTCAACTCGGAGGAGAAGCACAAACACCACGGCCCCGTGTGGATGTTCCGCATCACTTGAGAAATCCTACAAGAAGGACCGATTTCTGGACTTGGGCGCCAAGAGCGAGGAAAATGCCAAAGAGTGGAAGAGAGCGCTGGAAAACGCAGTCCGATCGCTGAGGGAGCTGGTGCTGCACCAGCGCAAACTCAAGACGGACGCAGAGAT---CTTCCCGACCCCCACGCAC---------GCCCCGAGACTGCTCAAGGCCGACGAGGACACAGTCTCGCTGTTTAATTTGGACGCTGACAACGTCAAGGCAGAGCAAGGGTGGTGGCTGGCCAGAGTGGAGGGAGGACTGCGAGTCATGCAGGAATGTCCGCTGGGACCGAGTGTGGCGGCCAATCTGTACGCACAACATGCAGCCACGACCAACGGACTCATCTTCAGTGCGTGCGTCGGGGCTGCACTAAGCTTCAGCAGCATGTACTCCTTCGCAAACAACATGCTGCTGAGCTTCATCACAGCGTTGGCGGCGATTGCTGTCTGTTGCGCTGCGGTGACACCCACCCAAGCGCCTGAGATCCCGAGTTTCCGCGTCTCACAAGTGGTCCATGGATCGCCCACGGAGGTTTTCCGTTTGATCATGAACTCAAAGAGGTTTCAGAGATGGGAATCGGCGACCGCTACCATGCGTGTTGTGCAGCAATTGGATGACCACGCGGATATCGTGTACGTCACGCAAAGACCAACACATCTGTGGCCGCTCTGGCAAAAGGCCCGTGATCTCGTGCTCATGCGCTATTGGCGGCGTGAGGAAGATGGCTCTTACTTTGTCATGTACCAGAGTATCGACCATCCTGAGTGTCGCGTGCGCCACAACTACGTGCGCGCGAACCTACTGGGTGGTGGCTTCGTTATCGCCCCACAACGCGTTCCAAGC---GGATCCATTCGCACATTGGTGACGTACGTGCTCCGATATGACCCTGCAGGCTG---GTCGCGCATCTATCATCAACTGGGCATGGACGTGGACCTGGTCATGCCCATGCTACGTAGCGTCGTTGGTATTCGCGATGAGATGAGTGCCACAGACTTTATCACACCTAACGTTACGATGCCATCAGCG---GAAGGCGGAGGCTGGTCAAGAAGTGTGGA---CTCTATGGGTGTGGTTATGGGGGTGCAGAAACTGCAG---ACCAGCCTTCCAGAGAAAATGTGGGGAGAGCCTGACGCTTCTCTCTTCAGTGTGCGTGGCCACAACTACCTGAGTGACCGCAAAAAGATCCCGTCATCGCCTGCCATGTTTCATACGGTAGGCGTGGACTTGCTGTCGTTTGATAGCCCAGGCGAGAGATACAATCTCTCGGCGCGCCCAGATAGCATTGGCCGAACCAG---CTCAAAATTCACATTTGTGGTGAATATGATCATTCCAGGCTCGGACAACACTAGTATGGTGTTCTACTTCCAGCCCGTCCGAGACAACATCTTTGAGGATGGCTCTCCCTTCTCGGAGTTACTGAATGACTTTATCGACGGCGACGACCAGTTCCGAAACTCGCGCTTCAAGCTCATTCCCACTGTCGTCGAAGGGAGCTTCATTATCAAACAGTCAGTCGGATCGAAGCCGACGTTATTGGGCAACAAGCTGAAGTGCCCGTACTACCGCGGTGACAACTACTTCGAAGTGGACATCGACATTTCGTCCAACTCTGTGGCTAACACGGTGACAGGTATGGTGCAAGGTGTCACAAAGTCGCTCGTGGTGGACATGGCGTTCTTGTTGGAAGCTCAAAGCGACGAAGAGCTGCCTGAGATCATCCTTGGTGCTGTCCGGTTGCAACACATATCGCTCGACAACCCGCGTCGCATGCCGAAGCATGGGGAACGGGCAGCAAGGACGAGTTCACCTTCTGAGGCCTTCACCGAAGCGCTTCGAGTGAATCTGCAGCGTCTCATCGTGTACGCGCGTTCGGCAGATACGGCGCTGCAGCGTGAAGTCGCTGAGAAGTTGGCCAACGAAGCTGTGAAGACCGACAAGTGCAGATTGTGGAGCTGGACGGTCTGCAGTTGCTGTTACCACTCACCAAGTCGAAGGATACGGAAGTGCAGCGTCTGGCGGCGCACGCACTTGCCAACCTCTCAGTGAACTCGGAGAATCAATCGAAAATGGCAACGGAGGGGGGTATCGACATGCTGATCGACCTCCTCGGCAGCTCCAATGAACATGTCCAACGACAAGCAGCGAAGGCCCTTGCCAATCTTGGCGTGAACGTGGATAACAAGGAGCGAATTGCGAAAGCGGGTGGCATCAAGCCGCTCATCGACCTCGCCAGCTCTCGTCAAATCGGCGTCGCAGTGGAGGCCATCGCTGCGCTCGCAAATCTCGCTGTCAACG---ACGCGAATGAGGTTGAAATTGCACGCAAGAGTGGGCTAAAGCCTATCATCGATGGCGCTCACTCAGAGTCCATAGAGCTGCAGTCCCAAGTAGCTCGCGCTCTGCGCAATCTCTCAGTCAACCCGGAGAACAAGCAAGCGATCGTGGAGTTGGGCGGCGTCGAGGCGCTACAATCACTAGTGCGCTCTACAAACGATCGGATATGCCAGCAGGCCACACGTGCTCTTGTCCAGCCCAAGGCCGTCACTCGGTCCTCGCCCACCATCTGCTG---GCAGTGGCGCCTGTGGCTCGAGTGGCCGAAGTGAAGGAGGAGAT------CGTGCCTTACGACGG---CATGGTGGACGTCGAGTACTCGGCGACAGTCAAGACGGTG---------GTGGTGCCTGCGATCGCAGTGG---GGGGCGCCGCGGCC---TGCTTCTCGTCCATCTCAGACCGTTTCCCTCTGCCCAAAGTGGGGAAAGTGAACTGCGGCATGGCTCCGATCCCCATTAGTCG---TTTGCTCAATG---CAACCCCAGAAATGACAC---AAGCAGATCAG-------------ATGACGGTGCTGGTGGAAGACCCGACGGTGGACGACAAC---ACTGTCAAGGTGGAG---CCAGAGACTGAACGACGTGTACAGATACCAAGCTACTGA--TGTGGAAGAAAACGAGATGGAGCAACTCCGATGGAACATTCAAGAGCAGGTTTTCCAACGATACTTTGAGCAGGGTCAAAAATTCGAGGAGTGGGGCAAAGTGGAGGAGAGAGACTCCATGTTTTCTTCATTCCTGGCTCACGAAGAGAAAGCATCATGGCAGAAAAGCGGTTTCATGACAACGCTGTGCGAGATGCAATGGGGAAAGGAAACGCCAATTGGGTCAAAGATCGACGATCACGTTTTGAGGAAGAGATCCGCATGTTCTACCAAGAAACTAGCCACAAGCAAGAGTTGATGCTGACGCGGCTTGAGAAGATTTCTCCGTTTTCTAAGTTTCCGATCCTTGGCAATCGTTTCTTACTTCTCCGACTTCGAGGCAAGGGAGGCAATGGAGAAGTGTGGGATGTCATTGACTATGCCAATGACAAACAAACGTGTGCTCTCAAGCTGTCGACATCAATTCGACACGCGCAGCGTGAGCACCAGACTCACACTTAACCACCCCA--ACATCGTTCAGATCGGCGACGTACCCTTTCTCATCCGATACCAACAGCAGCAGTACACGGCATTCACGGTAGCAAGCGTCCAGTCAGACTTGCAGCACCTCATCGAGG---TATTCGAGTATTTCGACGATGATTCTGCATACAAGGTGATTTCACAGCTTCTGAGTGCCCTGAAATACTTGCACGATGAGCACTGCCCACTACGACTTGAAGCCGTCCAATATCCTGATCGACCACGATGGGTGCGTTAAGCTA------ACCGACTTCGATTTAGCGCGCGACGCCAAGGCTGCTACGTCAAACTCTACTGTCGGGACACTGCGCTACCTTCCCCCGGAGTGTTTCCGGCCTAACCGAGGCGA---------CTGCGAAGCCACAGCTGAGAAGGCAGACATGTGGATGGTGGGCATTGTGTATTGCATGATGGTCACCGGAAAGCACCCGATTTGCCCGGATAAATCGACTCTCGCAGATGTGAAGGCAATGATGGTGCAGTACACTGGC---GAGCTCCGGTATTCACGTCCAATTTCGAATCTGTCTCGATGGATACTGCAGGGCTGTCTCCACCCTGACCCACGGTGTCGACCAACAGCTGCACAATTGCTTGTGGCTTTGCAA

Phytophthora_cactorum TGGACTACCACCGCCAGGTGGCCAGGGAAGTGGCCGCCTTCTTCCACTCCTTCTACCGCTTCGGCGCCGCCTCTAACCGCTTCGATCTGTTCCTGCACAACGTTCTCTTTGGCTCCTTGTGCATCGGGGCCGTCGTCTTCCTCGTGCTCTGCGTGATCGTCGTGCGCCGCACCATCCTGCACATCCGCAGGCCTTTGTTCGACGCACATCGATGGATTCTACGTCCGTGGAGCTACTGGCCGTCGGACTGCTGACATTCCTGGCTATTAGCGCACTCGGAGGACTGCTGGGAGAAGCTGTCTGCAGACCAGCAACGGCTTGCGCGTCAATGCTGATAATTTAATCACGTCATTCAACGACAGTGAACTACCAGCTGAAGCCTTCAAGATGTCAGTGGAGGCGCTGGGCTAGTGCACGCGTCCAAGGAGCTGTGGAATCAGACGGACGCGCTGCTGCCCAAGAACTACTCAGATATTGCCAAGGAGTGGGAGTTCAGCTACTTCGTGCTCAAGTCGTCGACCAACGGCGCCATTCTTGCGGTATCACTGGCCAGTTTCCTGTCTATTGCATCGGTCGGATGGGCCATGGTCTCGCCACTGCGCGTCTCGATTCTGGTGATCCTGAGCGTTGTACCCATTTCACACACGCTGATCGGTGCCTACCTGTCGTCGACGATCATGACGGCGGACATCTGTGCCGCTCCCTTGAACAACACGCTCGAACTGATCGGCACGTGTCCAGCCAACGCTTCCTGCCTTTCGCTGACGCTATGGCAACGTAGCGCTTCTCGTCAGGGACCAACTGGCCAGCGTATGAAGAGAGAATTCCTGGACCCCATCGGAGCAACTGTGGGCGTCATGCTCGGCTATTTTGCAGGAACTGTGAGCAATTGGGAGAGTTTCTCTCTCATCCAGACGTGGCGCTGGCCGTTCCTGCTCCAGTTTCTGGCTCTGCTGCCTATTAATGTAGCCATCTTCTTCGTACCAAAGAAACACTTGGTGATTCGCAGTGAGATCAACGAGTTGGAGCCACCTGTGGCCGGGACGCCCATGAGCACAAGATCCACGACGTATCACGACGAAGAAGACGCGTTGCTCGCCAGTGCCAGCATGCAACGTACGGGGCTTTGGACACGAGGTGTACGAACAGGGCGCCTTCTCGGAGGGCGTCTACGAGCTGCTGCATATCCCAGTCTTCTGCTTGATCGTCTTCGGCCTCACGACCGTCTACTTCGTCGTCACTGGCGTGCAGTATTGGAGCACCATCTTTATGATCAAGTCGCTGCATGCGTCCAAGTATCTCGTCAACGCGCTCTTCGTCGTTGTCTCCGGCACGGGACCGATCCTCGGCGTGTTCTTTGGTGGCTGGCTTATCGACAGATACGGAGGATACATCGGCGTGGAACAGCGCGCAAAAGCTCTGGGTATCTGCATGATCCTGGGACTCACAGCGTTCGCGATCTCAGCGGTGACGACGTTCTTCGACGACATTTACATCACTGCTGGGTTCCTGTGGCTCTTGCTCTTCTTCGGTGGCGCCATCCTGCCAGCGCATCGTGCGCTCGCCTCGTCCTTCTCCGTGATGGTCTTCAATCTGTTCGGCTACGCGCTTTCTCATGAGCGACTTGCAGATGGTGGTGGGCGCCAAGTGCTTTGTGCCGGATGAGACGCACGTGTGGCTCGCGGCCGAGGTATTGCGTGACGAGAACTCGGGTGATGGCAAGACGCGCAAGATCTACTGCAACGTGGAGCTGCCCGATGGAAACCGAGGAGCGCTGCGTGGACATGCTGTCCAAGAAGACAAAGGCGCTGCTGATGTGCACCAGCTCGAGTCGCTTCCCTACCAGAATGAGAACGTGGGGGCCGAGGGCATCGAGGACATGATCACACTCAACTATTTGCACGAGGCCGCCATCCTGTTTAACATCAAGAAGCGCTTCCTCTGCGAGCTGCCCTACACGTACACAGGCGATATCTGCATCGCTATTAACCCCTATAAATGGCTCCCGGACTTGTACGCTGAAGAGCAGCACCTCCGCTACCTCAACCAGCCCAAGGAGGAACTGCCCCCGCACGTGTACGCCACATCTGTCGCTGCCTACGACAGCATGCGGCGTTCGGAGCGCAACCAGTCCATTCTGGTCAGCGGTGAATCCGGTGCCGGTAAAACGGAGACTACTAAGATCCTCATGAACCACCTGGCCACCATCGCCGGTGGTCTCAATAACTCCACCATCAAGAGGATTATCGAGGTGAATCCACTGCTCGAGTCTTTCGGTAACGCCAAGACTGTCCGTAACGACAACTCCAGTCGATTCGGCAAGTTCACACAGCTTCAGTTCGACACAAACGGCACACTTGTCGGCGCTAAGTGCCGCACGTATCTTCTGGAGAAGACGCGTGTCATCCAGCACGAAGCGCCTGAACGCAACTACCATATCTTCTACCAGTTGACTCCTCCGACATTGCGTCGGAGTTGCAGCTCGATTCGACGAAGCATTATGTGTACACGGGTGACAACACGGCACAGAAGATCGAAGGGCTCTCTGACGAGAAACACTTCAACAAGACACGCGAGGCACTGGAGCTCGTTGGTCTGTCTCGGGATGACCAGCGGCCACTATTTGAAGTTCTGGCCGGTGTGCTCCACCTCGGTGAGGTGCAGCTGCAGTCAGACCCTGCAGATGACGAGAAGTCTCTCATCACTGAGGGCGACAGTGGTGCAAATTCTGCCACGGAGATGTTGGGAGTCACGTGGGAGGCACTTCAGACGGCTCTGTGTTCGCGTACGATGCGTGCTGTGAACGATGTGTACAGTGTACCGCTCAAGAAGGAGCTGGCCATGGACTGTCGTGATGCCCTCGCCAAGGCCATCTACTCCAATGTGTTCGACTGGCTTGTGGCTACGATTAACCAGTCGCTTGCTGACGATGCCAACATGGCCAACCACGTGGGTGTACTCGATATCTTCGGTTTCGAGCACTTCAAGCACAACTCGTTCGAGCAGTTCTGCATTAACTACGCCAACGAGAAGCTTCAGCAGAAGTTCACACAGGATGTGTTCAAGACGGTGCAAATTGAGTACGAGGAGGAAGGCATCGTGTGGGACCACATTGAGTACGCGGATAACCAGGACGTGCTGTCCGTGATTGAGTCGCGCATGGGTATCATTTCGCTGCTGAACGAAGAGGTGATGCGACCGAAGGGTAGTGAGGAGTCCTTCATGTCCAAGGTGACGTCCCTGCACAAGGAT---GATATGGCGCACGTTATTGAGTTCCCTCGCACGTCGCGTACCGAGTTCCTCATCAAGCACTACGCCGCTCCTGTCATGTACGACTCGGTGGGATTCCTGGAGAAGCACAAGGACTCGCTGCTGCCTGACCTGTCCGAGCTCATGCGTGGTTCGTCCAAGCCGTTCATCGCCAAGCTCTTCGAGCCCAAGCCGGAGCCTAAGCCGCCGGAAGC---------GTGGTGGTGCTCTGTCCATCACGACTGTGGGCACCCAGTTCAAGGAGAGTCTGACGGAGCTGATGGCTACCATCAACTCGACCCGTGTGCACTACGTGCGTTGTATCAAGCCCAACCCGATCAA---------GAGTGCAACTGTCATGGACCAAGCTATGGTGGTGTCCCAGTTGCGATGTGCCGGTGTGATTGAGGCCATTCGTATCTCGCGTGCAGCGTATCCGAACCGGTTGCAGCACACGGAGATCCTGGACAAGTTCTGGCTGTTTGTTCCGAGTGGCGGCGAGACGGCTGCAGACAAGTGCCAGCTGCTCATGGACAAGCTTAAGCTGGAGACCCCCACTCAGTACCAGATGGGTAAGACCCGTGTGTACTTCCAGCTAGGCGTCCTTGAAGAGCTTGAGGATCGCCGTAAGAAGTTCCTCGATGCCAAGGCCACATACCTGCAGAACATCATGGTTGGCTTCACGCAGCGTATCAAGTATCTCCGGCAACTGGAAGCAATCATCAAGTTCAGTGATTCGGTGCGTGATTGCCATGCGCAGATACAACACGTTCTTGAAGGGCCTCATCTCGGCGCAAGCTCACTGGCGAGGCATGCAGGGCCGCAAGGTGGCAGCTGAAGTCAAGAGCAACCACAACGCTGTCATTATCCAGCGGTATGTGCGTGGATTTGTCAAGCGTCACCGGTACGTTAAGATGCGCGAGATGGTCATCCGTGTGCAAGCTGTGGTTCGTATGACGATCCAGCGGCCAAAGTATCTGGCTGCTCTCGAGGAGAAGCGGCGTGAGGCGGACATGGCGTATCAGCTGCACAAGCTGAAGGCTGCTCTGCAGGAAGAGCAGGAGCGGAATGCGCAGCT------GCAGCGCC---GCAGCAGCGTGGC--GACGGCGCGTCCGCTGCCTCGAGTGTTGTGATGGCTGATGCCGGCGGAATGATCGAGACTCTCACAGACGAGAACAAGAAGCTACGTGAGAAGAACGAGGATATGAAGGTTACGATGAAGGGCTTGAAGGCGGAGATCGAGAAGTTTAAGAGCGACAAGGAGTTCTCGTCGGCCGGTAACCACGTGAAGGTCCGCCAACTGCAAGACACAGTGCGTGACAAGGACAAGAAGATCTCGCAGCTCGAGGCCGAAAACAAGAAGCTGACGGAGCAGATCGCCAAGCTTCACGCGGAAGGTGTTGT---TCCGGAGAAGAAGCCCACGCCGAAGAAGTCGATCTTCCGCACGCTGGGCTCCAAGAAGGAGAAGAAGCCTC---------------GCGAGACAGTGCTGATGGACTCGCTCACCGGCTCGGACGACCTCAACCGTCGGGAGACGCGTCCGTCGGA---GCGCCACTTCCCGCGCATGC---CGAGCATGAGTGTGGGCTCT---CGATTCTGGAACTCGAACAAG---AAGGGCGAC------------AACGGCGACGGCTTCGACGTCTCGGAGGTTGAAGGTGAGGACAGCCCCACGAACCCACGCGCAACACGATCGTGAAGAGCATGGAAGTGGGTGTGACCGGTGCCATGTCGAGTCTGAAGGGCCGCATG---------TCGGTCGTCAAGGGGATGTACGAAGCAAGCGCACGAACAGGACCGCCCCGTGCGCGAGTCGTTCAACCTGGACGCGTTGCCTGAGGTCTCACTTCCTGTCGGCTGGGAGGCCAAGGTGTCGCGCTCGACGGGTCGTGTGTACTACGTGAACCGCAAGCTCGGCAAGTCGCAGTTCGAGCGCCCCACTCTGGCCTCTCTCAA------GGCGCAGAAGTTGGCGCGCCAAAAGTCGGCTAACGGGCTG-AAGGCTGTGGTCGCTGATTTCGGCTGCGGAGATGCCAGACTGGCTGAAAGCGTGCCGAATAAAGTGCACTCGTTCGACTTGGTGTCCATCGATATTGCCGTGTACTGCTTGGCTCTTATGGGAACGAGTGTCCGTGAGTATGTGCGTGAAGTGTACCGTGTGCTGAATCCAGGAGGCGTGCTGAAAGTGGCTGAGGTCAAGAGTCGCTTCGAGAGCGAGTCACTCGGAGGATGGTGGCGGTGTTTGTGACGGCGGCGCAGGGCGTTGTGGTGACGCTGCGCAACACCACGGTGTTCGTGGAGAGACCAAATACGCAGTTATTGTGGGCCAGAAGACAGGCGAGGCCGTGGCCATGCCGTTCCCGGACGTGGACCGCGACCTAAAGACTCTACTGGGCCACACCACGTCCATGGTCACCCACGTGGCCGTAAATCTCATCGCTGCTACTAACGGCCGACCGTGATGAGAAGCTGCGTGTGTCAAGGTTCCCGAATGCTGCGATCATTGAGAGCTACTGCTTGGGACACGCTGCCAGTCTGACCAAGGTGGCCTGCAGTGCAGTGACCCCCGACCTTGTGGTGTCCACGTCCATGGACAACACTCTAAAGCTGTGGCAGATGACGACGGGCAAACTGGCCATTTGCCCCAAGACGACATTGTGGCCGTGCTTGTGAACTACCAGCACGTGCGATTCTTCGAGATCGTGAGAACCCATTCCTGCAGTTGTTTTCGATCTCAAAGGGCAAGTGCTGCATTCACCCGCTGTGCGCGACTTTCGCAAGCTTCAGAACGACCCGCCGTCGGGCGTGTCGGGTGCCCCCATGGACAACAACATCATGCTCTGGCAGGCCGTCATCTTCGGACCGGACGACACACCCTGGGAAGGCGGCACTTTCAACCTGACGCTTGAGTTCAGCGAAGACTACCCCAACAAGGCACCGACCGTCAAGTTCATCACGAAAATGTTCCACCCTAACATCTACAATGACGGCCAGATCTGCCTGGACATTCTGCAGAACCAATGGTCGCCGATCTACGACATCTCGGCCATCCTCACGTCCATCCAATCGCTGCTGTGTGATCCCAACCCGAACTCGCCAGCCAACTCGGAGGCCGCACGCCTGTTCCAGGAGAACCGACGGGAGTACAACCGCCGCGTGCGCGAGATCGTCGAGCAAAGCTGGCAGGCGATCGATGGTGTCGGCCGCCAAGATGCGTGGCGCTGAGAACCGCATGAACGACGGCCGCCCCTTCACCGCGTGGCTGGACAATGTCTCGTCGGACGCTGGTCTGTGCGGCGGTATCAACTCGGGTATTGCCAAGACCACCCGCAGGCAGACCACTCGCAGATCTTCGTGATCGGCGACAAGGCCCGCGCCCAGCTGCGTCGCGACCTCGGCGACAACATCCGTGGCAACGTCACGGAGACATACCAGTCGCCCCCCAACTTCACGGTGGCCAGCGCCATCGCGGAGGCTGTCGAGTCGGAGAGCAGTCGTCCCGTATGGCTGCCATGGAGAACGCCACCAGCAACGCTGAGGACCTCACGCTCACGATTGTGTACAACAAGGCTCGCCAGGCCCGTATTACCACGGAACTGATTGAAATTATCTCGGGTGCCGCCTCGCTGGACAGTGACTGGCAGCGTGGTGAGCGTCCAGAGCGTGGCGCAGTTTGACGAGGCGACGGCGCGCGAGTCGACGCTGTCCGTGAGTTTCTTTTGGGCCGAGTTCCAGGCCTGTCGCCCCAATGGGCAGCTGGACGTGGGGCCGCGCCGTGCTGGACAAACTCGAAGGCGCCAATGTGGCCGAGCTGGCCAAGCGCGTCTCCAATACCGCCTCAAGAAGCTCATCAGTGCGTCGCCCGTCATGCTCTTCATGAAGGGCAACCCGACGGAGCCCAAGTGCGGCTTTAGTCGACAGATAGTGGCGCTTCTGAACGAAGAAAAAGTCCAATTCGGCACGTTTGACATCCTCAATGACGACGAAGTGCGTCAAGGACTCAAGCAGTTCTCCAACTGGCCCACGTATCCACAACTCTACGTGAACGGGTCGCTTATTGGCGGTCTGGACATCGTCAAGGAGATGAAGTCGGAGGGATCCATCGTCGAGCAACTAGGCGTCGAGGAGGCTGAGGCTGCCTTCCAGGAGAGTCTGCATGCGCTCGTCAACTCAGCTCCAGTGCTGCTCTTCATGAAACTTCGACATCCTGTGTGGAGCTACCAACGCCAGGACCAAGACGTCGCCAAGGTGGTGACTCTCACGCCACCCTCATTCCGTCGTGATATGTGCCGAGGCCATCTCATGGAGCCCGAAACCGCATGGCCATGGCATTCCGTGATCCTTTGCAGGAAAAGGGAGAGTTCTGCATTGTGCAGCTCCTCGTATCACCTCGAAGCGCACTGTTCTGCATTCACGAGGTATTTCTGCGATCGACTGGCAAGTTTAATGTGCTGCACCGAGACCACCGCAGCGAAGTCAAGTCGCTCTGTGTTCACTCTCAGCGTGACTGTTTGTACACGGGCGGTCTTGACGGATTGTTGATCCGATATGACCTGAAGAAGGGGCGTAGTGAAGTTGTGATGGAGCGTCGCAAACGTATCTCCAAGATCAACGCAGTACTGGAGCATCCACACAATCCGAATCTTCTTTTGGTCAGCTCCGTGGAGCAGTAGACACAATTTGTTGCTGCACGACCTGCAACGCTATGATCGTAACGTGTCGGGAAGCTCGATGAGTCAGTACGTCGTGCCGCGGTGGTCGCCTGCTGGTTATCACGTCTCGTGCGGCAGCAAATGCGCTACTGCCACGGAAGTTTCTCGACACCCACGTCAGCGACCATCGGGGCCTCGTTCCTGCAGAAGCGCGTGATCGTCGGTGGGAAACTGACGCTTCAGATATGGGATACAGCCGGCCAGGAGCGCTTCCGCTCCATGGCGCCCATGTATTACCGCAATGCCAAGGCGGCTATCCTGGTCTTTGACCTTCAGAGCGAAGCCAGCTTCGAGAAGATCAAGGAGTGGCTTCACCTGCAGCATCACGTGGGTGACGACATGTGGGCAACAAGAGCGACGTTTCGACTTTGCCAAGGCCCAGCAGTTCGCGGATGAAATCGGTGCGCTGGTGTTCCGCACGAGCGCCAAGACGGGTGAAGGAGTGCAGTCACTGTTCGAGAGTCTGGCGGTGCAGCTACTTAAGAAGCACGATCAGGAGCAGCGAAGAGCTTCTACGTTGTGTACGTGCCAGCGGATGAGGCCAAGGAGCTGGAGGAGTGGACGGTCAAGCTGCCGGAAGACAAGGAGGCGCAGCTCGGCTGCTTGACGGAGCGTCTACGCAAGCATTTTAAGGGCGGATCCATCCAAGCAGCAGCAGAGACTTTCAAGCAGCAATTGCTGGCCCAGTTGCCCAAGGGCGCCACATTGACGGATGAAATGCTGCAGATGATGCATGGACAGCCTTGTGGACTCGATCCCGCTCGTGCTCAACACGCCCGACGTCAAGCACGTTGGAGGTCAAGGGCGACGCATTCATTGGACGAGTGTTCGATAGACGACTTTGTGCGTATGGACTTCCGGTTGAGTGAGGTTTCGGGTGACGCGCCTTGGGTGCAGGTGGCCAAGGTGCAGAGGTCACGCAGTCGCTAGAGGAGATCCTGGCACTGGGCCAGGGCCTAGACGACGAGGACTACAAGGCCGAGCTCGAAGTGTTGCAGAACGACCCCAACTCCAACCTCTACTCGGCTGTCACCTTCGAGGAACTCAACCTACCGGAGGCGCTGCTCAAGGGCGTGTACAACATGAAATTCGCCAAGCCGTCCAAGATTCAGTCCGTGGCGCTGCCACTGATTCTCGCCGATCCTCCAGAGAACCTGATCGGCCAGGCCCAGAGTGGCACCCCACCCGCGAACTGGTGCGACAGATTATGGCCGTGGTGAACGCCATGGGCAAGTTGTGGACACGTTCCTGGCCATCCCCGGCAACGATCTGGCTCGCGGTGAGACGCTGAAGGCACAGGTGATCGTCGGTACCCCCGGCAAGGTGGAGGGTATGATCAAGAAGAAGCAACTGGACACTCGTGACGTCAAGATCTTCGTGCTGGACGAGGCTGACGTGATGGTAGCCGAGGACGGCATGCGCGAGCGCTCCGTGTCGATCAAGCGCCTCATCAGGAACCGCGCGTGCCAGTACCTGCTCTTCTCGGCCACGTACGCTGACGATGTGCGCGACTTTGCCCAGAAAATGGTGCCCGACCACAACATTATCACAGTCAAGAAGGAGAAGCTCACGCTGGACGGCATCAAGCAGTTCTGGATCGACTGCAAGACCCGCGACAACAAGTTCCAGGTGCTGTCGGACATTTTCGCCATCCTGAGCATCGGCAAGTGCGTCATTTTCGTGCAGCAGCGTGAGACCGCTAAGGAACTGACGCGTAGTATGCGTGAGAAGGGTCACTCGGTCGGCATCCTGCACGGCGCCGACATGGCCAAGGAGGTGCGTGACCAGGTTATCGACGAGTTCCGTGCCGGTACAACCAACGTGCTGATCACCACGAACGTTCTGGCTCGCGGTATCGATGTGGCGGGCGTCTCACTTGTCGTGAACTTCGACATCCCGCTGACTCGCGATCACCGTCCGGACCCGGAGACGTATCTGCACCGTATCGGCCGTACCGGTCGGTTCGGCCGCAAGGGCTGCGCTATTAACTTCGTCTACGACAACAAGAGCAAGCAGGACCTCGCCGAGATTGAAGAGTACTACGCACGTCCGATTACTCAGGCCCCCGCAGACGACATTGAGGAACTTGAGAAGCTACTGGCTTGGGGGGCAGCAAGCGCCGCCTGGCCAAGTTCGGCAAGGAGCAGCGCAAGGGCATGCGAATCTACCCTCGCGAGCCCAAGAAGAAACCTTCGGGCAAGGATAAGACGTACTACTACCTCAAAGACGTCATGTTCCTGGCCCACGAGCCGGTTCTGGAGAAGTTCCGGGCGTTTAAAACCTTTATGAAAAAAGTCAAGAAGGCCATGGGCAAGAAGGACTACGAAGATGCCAAACGCAAGCACGAGAACCGCCCTACCTATGAGCTGGACCACATTGTTAAGGAGCGTTATCCGCGCTTCGTGGACGCTCTAGGAGACCTGGACGACGCTCTGTGTGGCAGAACTACGTTGTAGCCACTAGAGCCTTGACCAAGGTGTTTGTGAGCATTAAGGGCATCTACTACCAGGCTAACGTCCAGGGTCAGACCATCACGTGGCTCGTGCCCCACCAGTTCACCCCAACGGTGGATAAACGTGTGGATGTGCGTGTGATGCTCACGTTCCTCGAGTTCTACGAGGTTCTACTCAAGTTCGTGAACGCGCAGCTCTACCTGCAGATCGGTGTGGCTTATCCGCCCAAGATCAACCTCCAGTTGGACGCTGCTGGTGCGCAGTTGGCTGCTCTGGAGCTAGCAGCAGAAGGAGTCGGAGAAGAGGATCAAGCTACAGCGAGCAGCGCAAGGAACGCAGCGAGAAGAGCTTGTTCAGTGGCTTGACGTTCTTCCTGTCGCGTGAAGTGCCTTCTGCGTGTCTGGAGCTGGTGCTGCGCTCGCACGGCGCTACGTTGGGCTGGGACGGCGAGGGATCTCCGTTCGACGAGAAGAGCTCGCGTATCACGCACCACATCATGGATCGTCCGCATCAAGGACATCGGTACTTCAACCGCGAGTACGTGCAGCCTCAGTGGGTGTTTGACTCGGTGAACAATGGCATGTTGTTGCCGTTGACGCGTTACAGAAAAGAACTGGAGAAACTCAAGTCTGCCTCGCAGGTTCTACGTGAGGTGGGGGCTGTGGGCGACGAAGAAGAAGACGGGAGGCCTACGCCGCGGATCTCAAGGCTGAGATGGATGGTAACAAGAAGTCGAAGCGTGTTAAGAAGGATGAGGAGGCTGCAGAGCTCAAGGAACTGGCTAAGACTATGATGTCCAAGAAGGCCAAGCGTCTGTACGACCGCATGCAGTACGGTCTGGGCAAGAAGGCTGACAAGATTCAGAAGCTTGAGGACAAGCGTGCCAAGGTCGAAGCAAGACCAAGAAGCTGCTGGCGGGCGCGCTGTCAGGTATGATCTCGGCGTTGGCCTTTCAGCCTTTGGGGCGCTGCGCTGGCGCGGCACGTCTCCCACGCTTGTGCGCGTGGCTGGCGGTGCCGGACTCTACTTCGCCACGCTGGATCACTGCCTCAACGTGTTCCCACACTCTGCAGTCAACACGTTCCTGGCTGGAGCCTTCGCGCGTACTTTTGCAGGTGGCGTGATGTCGCCCTTCACTATTGTCAAGGCGCGGATGGATGCCGCCCGGTACTTTCGACAGCAAACGGACGTGCCATTTTCTGGACTGTATGTTCTCATATACACGGCACCCAGCCGATGTGGTGAAGACACGCATGCATGCGACAGACCGTGGCCAAGATCTATCACCACGAAGGTCTGCGTGGCTTCGCGAAGGGTATCATTCCGCGTGTGGTCAAACGAACGCTGTCCACCGCAGTCACGTGGACAATCTACGAGCTACTCGTTCGACCCGTCGGGTCTGGGTCACGCCAAGGAAGCCTTTAACCTGGCCAAGGAGACGGAGCGCACCAAGCAGATGGAGAATCAGGCCAAGATTAAGGAGAACGAGGCGCTCTACAAGCAGTACGAAATCGTGCGCGTGCAGAAGGAGGGCGAGGAGCGCCGTAAGACGCTCGAGGAGGAGACGCGCCAGCACCAGCAGCGTGCGCAATACCAGGACCAGCTCAAGCGCAAGCAATACGCTGACCAGCTGGCTGCCCAGAAGTACATGAAGGAGCAGGAACTTAAGAAACAGGAGGAGATCCTCGCCCGCCAGGAGGCCTCACGCCGAAAGACGCTCGACTATGAGGCTGAGCTGCGTCAGAAGACTGAGCTGGTCAAGGTCGCGGCCGAGACTGAGGGCCGCATTAAGCAGGAGCGTCTTAACCACGACCTGCACCTCGAGGAGGCCCGTGTGCGTGCCAAGGAGTACCGCGAGACCGTCATGGAGGGTATTAAGCTGGCTAGTAACACCGTCGGATCCGGCATTATGACCTTCGTGGACGATAAGGAGAAGCTCACGGCCACTGTGGTCTCTCTGACCGCTCTGGCCGTCGGTATCTACACCGCTAAGGTGTCCACCAGCGTTGCAGGCAAGTACATTGAGGCACGCATGGGAAAGCCGTCGCTGGTGCGTGAGACGTCGCGTCGCTCAGCTGCGCAGGTGCTGGCCAACCCCATTCCGTCCATCAAGCGCGCGCTGCGTCTGCAGAAGGCGACGGATGCCCTCGAGGGCGTCGTGCTTGAGCCCAAGTTGGACGAGCGTCTGCGCAGCGTGGCTGTGTCCACGTTTAACACGAAGAAGAACCGTGCGCCGTTCCGCCACTTGCTGCTGCACGGCCCGCCCGGTACAGGTAAGACGCTGTTCGCTAAGGCTCTTGCTCGTCACTCCGGTCTCGAGTACGCCATCCTTACGGGTGGTGATGTTGCCCCATTGGGTCGCGAGGGTGTGACTGAGATCCACAAGCTGTTCGACTGGGCATCGCACAGCCGTCGCGGTCTGCTGCTGTTCGTCGATGAGGCTGATGCGTTCCTGCAGAAGCGTAGCAACACCGTCATGAGCGAGGACATGCGTAACGCACTGAATGCCTTCCTGTACCGCACGGGTGAGGCCAGCGACAAGTTCATGATCGTGTTCGCCAGTAACCAGCCGGAACAATTCGACTGGGCCATCAACGATCGTATCGATGAGATGGTGGAGTTCCGCCTGCCCGGATTCGACGAGCGTGTGCGCATGCTGAAGCAGTACTTCGACGACTACATCCGTGCGCCGACGAACTCGCGTGCCAAGAAGATTTACGCGAGGGCGTTATTTTCACGCTGGACCCTGTAGCCAACTTCCTCGTGCCAAGACGCGCATCTTCCAGCTCGAGGCGCTGCAAGGCGTGTCAGGCGAAGCTCAGGCTATTTTCGATGCACTCAACAAGAACCTATGCGCATCTACTGGGTCGTCTTCGTCGTGGTGCTGGGCAACGGACTGTATACGTACTTCGCCGGCAAGGACGAGTCGTTCTTAGTGGAAAAGCTCAAGAGAAAAATCCCGTCCGAGTACCGCATCTCTGAGAAGTGGGACAAGTGCCTGGAGAACTTCACACTCTACTTTGGCGCTGGCCTTGTTGCTGGCGGCCTCTCATCCCTCGTGCTGGCTCGTTCTGGAGCTGGTCGCGGACTGGTCACGGGTCTAGGCGTGCGGGCGCTGGGTCAAGCTGGACGACGTGCCAGTTGGCATTCGCGGGCAACACACAGGCAGCTCTGGACAAGACGGACAAGGCCGTGGGCGACTTGCCAGCCTTCCAGCAGCAGACGATCGTGGACTTTGTGACGGCCTCGGCCACGCAGCAGCAGCTGCAGACCGTGCACCCGTGGCTTCTGCGAGGAGGCCAGTGTGAAGGTCTTGGAGACGTACCTGGCCGAGCAGGTGCAGAACAAGACCGTGGACATTGACGCCAGCCTGGCGCTGCTCAAACTGTACCAGGTGTACCCGGCCACGACCAACGCCGAGAGCCAAGGTGCTGGTCAAGGGCGTCATGGCACTGCCTTCCACCTTCTTCACGGGTGTCTACCATGGTCCCGGAGAGCATCCGCGAGGATGCCAACGTGACTGCGGTGCTTCACACGGGCTTTATGCTCCAGTCGTGCCTGTTCGAGGACTTTTGGAAGGAGAAGGTGTCTTTCGCCGAGAAGGTGCCGGGCTTCCTCGAGTCGGTGCGCGCCTACATCTTGACAGCCATCAGCCGCAGCCACAGCGTCATCTCCACGGAGGTCTTTAAGGCCAAGCTTAACGTGTCTGACAAGGAGGTGGCCGAGATCGTTGCCGAGAAGTGGACGCCGACAACTTGATCCAGATCAACCCCAACGAGGACAACCAGATGCAGGCCAAGAAGGTTCAGGAGAACATTGAGTTCGAGGACGTGCTCAAGGTCATCCACACCCTTTCTAGATCCAACGTCTGCGCCAGTGGCGGCGGCGTGGCTGATGCCGAGGAGGTCTGTGAGAATCTAGCCAAGCACTTGAGGCTAGACGGGGGCTCCCAGGTGCTGCCCTGCTCCACGGGTGTCATTGGATGGCGTATCCCGGTGGATGCCATGGTCGAGAACCTCCCCAAGCTCGTCAACGGCCTGCAGTCCGACTCGGTTCTACCTGCTGCCGAGGGCATTATGACCACTGATTTGTACCCGAAGATCCGCTCCATGGACGTGTGTGGCGGCCGCGTCGTCGGCATTGCCAAGGGTGCTGGTATGATTGAACCCAACATGGCGACTATGCTGTCCTACATCCTCACCGATCTGTCGATTCCGCGTGACGTGCTGCGTCAGCTGCTGACGGAGGTGGTTGGAGACACGTACAACTCGCTCAGTGTGGACACGGACCAGAGCACGTCCGACACGGTGACGCTCGTTTCGTCGGACCAGATCCCGTTCGACATGAA---GCAGCTGGACGAGTTCAAGAAGGCTCTGCATGAGGTGTGTCTGGGACTGAGCGAGGACATTGTACGCAACGGTGAGGGTGCGCACCACGTGATGCGTGTGCGTGTGAGTGGCGCGTTGTCCAAGGAGATGGCCAAGGGCGTCGGCAAGTCGGTGGTCAACTCGCCTCTGCTCAAGTGTGCGGTCGCAGGCAACGACCCCAACGTGGGCCGCCTCGTCATGGCTGTGGGCAAGTACATGGGTCTGCACCACAAGGACGTGAACATTCAGCGTCGCATGAAGATCCGGATGGGTGGCGTTCTCATCTTCGAGAACGGCGAGATGGTGCTCAACGACGATATTGAGGCCAAGCTGGTGGCCCACATGCGCGACGCGATGCTGATTGAGCCTACCCGTGGTGGCCTCGACGCCAGCTCGCACAACTACCCGCCACACGGGAAGTTTGTCGAGATGGAGATTGATCTGGGTGTTGGCTCCGAGGAGACTCAGGTGTTGGGCATCGACCTTACGCACGAGTACGTGGCCATTAATGCCGACTACCGCTCG--ATGGCGCC---GAAGAAGGGGAAAACTGCCGCCGCGTACCTGGCGTCCCTGGGTGTGGCCCCTAC---TGCGGGAGGTGAGAAGAAGGACGAC---AAGAAGAAGAAAAAGAAAGGAAAGAAGAAACCCAAAAGAAGCCCATGTCGGCCGCGGCGAAGGCCCTGA-TGGAGCGCCAGGCGGCCATGAAGGCCGAGCAGGAGCGCCTCGAGCAGGAGCGTCTGGAGCAGGAGCGCATCGCCCAGGAGGAAGAGGCCAAGCGCCTGGCCGAGATCGAGCACGAGGAGGAGATTCGCCGCAAGAAGAAAGAGGCGCGCGCCGCCAAGATCGAGCGTCAGAAGAAGGAAGGAACGTACATGACCAAGGCCCAGAAACAGAAGGCGGCCAAGGCCCAGGCCGCACTAGAGGCCATGCAGCAGGCGGGTCTCGTGCCCGACGCTGCT------GGCGAGAAGAAGAAGGTCGTGTACACGGACAACAAGAAGAAAAACAAGAAGAACAGGTGGAAGAGGAGGAGGAGGCCGAGGCACCCGATGCGTGGGATGACGAGGACTGGGAGACCAGCGATGTTGTGGCCTCACTGGAGGATAAGTTAGCAGAAGAAGGAGCAGGAGCGTCTACGCCTGCAGGGAATCGCTCTGAAGAAACGTCAGGAGGAGCAGGCCGAACAGATGCGTCTGGACGCCGAGGAGGAGCGTCAGCGCTCCAAGGCAAAGCTCGAGGCTGAGCGCAAGAAGAAGGAGGCAGCCGAGCGTCGTCGTGAGCGTGAGGCTGCTGCGCGTGCCAACGTTTCTCTGGACAACCTCCGTTCACCTATTTGCTGTATCATGGGCCACGTCGATACTGGTAAGACGAAGCTTCTTGACAACATCCGTCGGACCAAGGTGCAGGACGCCGAAGCTGGTGGTATTACCCAGCAGATTGGTGCGACCTTCTTCCCCGTGGAGGCGATTAAGCAGAAGACGGCGCGCCTTGCCGAAACCATGAAGCTAGATTACCGCCTGCCCGGTTTGCTTGTGATCGATACGCCCGGTCACGAGTCGTTCACGAACCTGCGTAATCGTGGTTCGTCGCTCTGTGATATCGCCATTCTGGTGGTGGATATCATGCACGGACTGGAGCCGCAGACTCTGGAATCACTGCGTCTGCTACGTCAGAAGAAGGCCCCGTTTGTGGTGGCGCTGAATAAGATCGATCGTTGCTACGGATGGAAAACCATGCCCGACATGCCCGTCCAGGAGGCACTCAAGCACCAGAACGAGCACGTCATTCGCGAGTTCGAGGATCGCATGAAAGCGATCATTGTGGAGTTTGCTGAGCAGAAGCTGAACGCCGAGGTGTATTGGCGCAACAAGGACTTGGCGCACACGGTATCGCTTATCCCCACTAGTGCTATCTCGGGTGAAGGTGTGCCGGACCTGTTGATGATGCTGACGCGATTGACTCAGGAACGCATGGCCAAGTCGCTGGCGTTCGTTGACATCCTGCAGTGTACTGTGCTCGAAGTGAAGGTCATTGAAGGTCTTGGTACAACGATTGATACAATTCTGGTGAATGGTACGCTAGAGGAAGGTGCAACTATTGTAGTGTGTACATTGGATGGACCAGTTGTGACAACGATCCGTTCGTTGCTGACTCCACACCCGATGAAGGAAATCCGTGTCAAGGGCGAGTATATCCACCACCAGAAGATGAAGGCTGCTATGGGTGTCAAGATTTGCGCTCAGGGCCTTGAGAAGGCAGTTGCTGGTACGCAGATTTACGTGGTTGGCCCCGATGATGACGTTGAGGAGCTGAAGGACTCCGTCATGTCCGATCTGACGGGTATTCTGGACTCCGTGAAGGCTACCCGCCGCGGTGTAATGGTTCAGGCTTCCACCCTTGGTGCCCTGGAAGCGCTTCTGGAGTTCTTGCGCACATGTGACCCGCCCATCCCAGTGTCCTGCGTGAACATCGGCCCCGTCCACAAGAAGGACGTTATGCGTGCTTCGGTGCAGCTGGAGCACCAGCCTGAGTTTGCCACCATCCTGGCGTTCGACGTGAAAGTCCACAGTGACGCTACTGAGCTGGCCACAGAGCTTGGAGTGCGCATTTTCACTGCCGATATCATTTACCACCTGTTCGATCAGTTCACGGCGTACATGGACAACTTCCGTATGGCCCGTCGTGAGGAGTTCGAGGAGGTGGCTGTGTTCCCTTGTGTGTTGAAGATCCTGCCCAACTGCGTCTTCAACAAGAAGGACCCGATCATTCTCGGTGTGGACGTTGAGGAGGGTATCCTTAAGATCGGCACGCCACTCGTGGTGCCGTCCGCCGGCG---GTTTCCTGGTCGGCAAAGTGGGCTCCATCGAACGCGAGCACAAGGAGGTGGACCGCGCCAAGAAGGGAGCGTCTGTGGCTGTGCGGATAGACAACGAGGGCAGCGTCATGTACGGTCGCCACTTCGACCACAAGAACAAGCTGGTGTCGCGTCTGACACGCCAGAGCATCGACGCACTGAAGGAAAATTTCCGGGAAGACTTGCAGAAGGAGGACTGGCAGCTCGTCATCAAGCTCAAGAAGGTGTTCGATATCATTATGGAGGGCTGGCTCACCAAGCGCCGCGACCACATGAATCTCATCTGGCTGGATCGCTATTTCCTCCTGGACGGCAACCAGCTCCGCTACTACCGCAAACGCGGCGACCCTGCGCCTCGAGGGACCTACATTCTGACTGATGGCTGCGTAGTGAGCCCCGTGTTCAATTCCGAGGAGAAACACAAGCACCACGGGCCCGTCTGGATGTTCCGCATCACTTGAAAAGTCGTACAAGAAGGACCGATTTTTGGATCTAGGAGCCAAGAGCGAGGAGAACGCCAAGGAGTGGAAACGCGCTCTGGAGAACGCCGTGCGGTCGCTGCAAGAGCTCGTAGCGCACCAGAAGAAGCTCAAGACCGACGCCGAGAT---CTTCCCCACGCCCACACAC---------GTCCCGAGGATGTTGAAGGCCGATGAAGACACGATGTCTCTCTTTAATCTGGACGCAGACAATGTCAAGGCGGAGCAGGCCTGGTGGTTGGCCAGAGTGGAAGGGGGGCTCAGAATCATGCAGGAATGTCCGCTTGGACCCAACGTTGCGGCCAATCTGTACGCACAGCATGCAGCGACGACAAACGGCCTGATCTTCAGTGCGTGTGTGAGCGCGGCGCTGAGTTTTAGCAGCATGTATTCCTTCGCGAACAACATGTTGCTGAGCTTTATGACGGCACTGGTGGCCATTGCCGTGTGCTGCGCTGCGGTGACGCCTACACAGGCTCCCGAGATCCCGAGCTTCCGTGTGTCGCAGGTGGTGCATGGCTCCCCAACTGAAGTCTTCAGACTCATCATGAACCCCAAGAGGTTCCAGAGATGGGACTCAGCGACGGCGACCATGCGCGTGGTGCAGCAGCTGGACGACCACGCGGATATCGTGTACATTACGCAGCGCCCGACGCACTTGTGGCCGCTGTGGCAGAAAGCTCGCGACCTTGTGCTCATGCGCTACTGGAGACGTGAAGAGGACGGATCGTACTTTGTCATGTACCAGAGTATCGAGCACCCGGAGTGTCGTGTGCGTCACAATTATGTGCGTGCGAACTTGCTGGGCGGCGGCTTCGTTATCGCTCCCCAGCGTGTTCCCAGT---GGATCTATCCGTACCCTGGTAAGCTACGTGCTTCGTTACGATCCTGGGGGCTG---GTCACGGATTTACCACCAGCTCGGTATGGACGTGGATCTGGTGATGCCCATGCTGCGTAGTGTTGTGGGTATTCGCGACGAAATGAGCGCCACGGACTTCATCACGCCCAATGTGTCGATGGCTCCTGCTGCAGAGGGCGGAGGTTGGACAGGAAGCGTGGA---CTCTATGGGAGTTGTGATGGGCGTACAGAAGCTGCAG---ACCAGTCTCCCGGAGAAGATGTGGGCCGAGCCTGACGCCTCTCTCTTCAGCGTCCGTGGACACAACTATCTGAACGACAAGAAGAAAATTCCTTCGGCGCCGGCGATGTTCCACACTGTGGGCGTGGATCTGTTGTCGTTCGAGAGTGCAGCCGAGAGGTACAACATCTCGTCTCGTCCGGACAGCATCGGCCGCACCAG---CTCCAAGTTCACATTCGTTGTGAACATGATCATTCCTAGCCCAGAGAATGTCTGCATGGTCTTCTACTTCCAACCTGTGCGTGACAACGTGTTCGAGGACGGCTCTGGCTTCTCCGAGCTGCTGAACGACTTCTTCGACGGCGACGACCAGTTCCGGAACTCGCGCTTCAAGCTCATCCCCACTGTCGTTGAAGGCAGCTTCATTATCAAACAGTCGGTCGGTTCCAAGCCAACACTGCTCGGTAACAAGCTGAAATGTCCGTACCACCGAGGAGAAAACTACTTCGAAGTGGACATCGATATCTCGTCCAACTCAGTTGCCAACACGGTGGTGGGGATGGTGCAAGGAGTGACCAAGTCGCTGGTGGTGGACATGGCGTTCCTGCTCGAAGCCCAGACCGACGAAGAGCTGCCTGAAGTCATCCTGGGCGCTGTTCGCATGCAGCACATTTCGCTTGACAACCCGCGCCGCATGCCCAAGCATGGGGAACGGGCAGCAAGGACGAGTTCACGTTTTGAGGCATTCACCGAGGCTCTGCGTGTGAATCTGCAGCGACTCATCGTCTACGCGCGTTCGGCCGATACGGCGCTGCAGCGAGAAGTCGCCGAGAAACTGGCCAACGAGGCTGTCAAGACCGACAGGTGCAGATTGTAGAGCTGGACGGACTGCAGCTGCTGCTGCCGCTGACCAAGTCGAAGGACACGGAGGTGCAGCGTCTAGCAGCGCACGCACTCGCCAACCTCTCAGTCAATTCTGAGAACCAGTCCAAAATGGCAACGGAGGGAGGCATCGACATGCTGATCGATCTACTGGGCAGCACGAATGAGCACGTACAGCGGCAGGCCGCGAAGGCGCTTGCCAACCTCGGCGTGAATGTGGACAACAAGGAGCGGATCGCCAAGGCGGGCGGCATTAAACCGCTCATTGACTTGGCCAGTTCTCACCAGATCGGCGTCGCCGTCGAGGCCATCGCAGCGCTTGCCAATCTGGCTGTCAACG---ACGCCAATGAGGTCGAGATCGCACGTAAAGGAGGCTTGAAGCCAATTATTGCCGGAGCGCACTCCGAGTCCGTGGAGCTTCAGTCCCAAGTGGCTCGCGCACTACGCAACCTATCGGTCAATCCGGAGAACAAGCAAGCGATCGTGGAGCTGGGCGGGGTGGAGGCTCTACAGTCACTTGTACGTTCCACCAACGACCGGATATGCCAGCAGGCTACACGCGCCCTCGTCCCCAACGTGGTCCTCACGCGCTCCTCGCCCACCACCTGTTG---GCCGTAGCGCCTGTAGCTCGCGTGGCTGAAGTCAAGGAGGAAGC------GATGCCCTACGACGGCGCAATGGTGGACGTCGAGTACTCGACCACAGTCAAGACGGTC---------ATTGTGCCTGCAGCAAAGCCCA---ACGACACGACCGGA---GGCTTCTCGTCCATCTCCGACCGCTTCCCGCTCTCCAAAGTGGGGAAAGTCAGCTTTGGCATGGCTCCTATTCCCATGAGCCG---CTTGCTCAACG---CCACGCCGGAGATGACAC---AGGCGGACCAG-------------ATGACTGTGCTCGTGGATGATGGCACCGTGGATGATAAC---ACCATAAAGGCTGAG---CCGGAAGGTGAGCAACGTGTACAAGTACCAGGCCACTGA--CGTAGAAGAGAACGAGATGGAGCAACTTCGATGGAACATCCAAGAGCAGGTTTTCCAGCGGTACTTCAAGCAAGGACAGAAGTTCGAGGAATGGGGACAACTGGAGGAGAAAGACTCCGAGTGTTCTTCATTCCGGGTGGACGCAGAGAGAATATCCTGGCCGAGAAGCGGTTCCACGACAACGCTGTCCGAGACGCGATGGGAAAGGGAAACGCCACTTGGGTCAAGGAGCGACGGAATCGATTCGAGGCCGAAATCCGCAGATTCTACGACGAAACCAGGCACAAACAGGAGCTGATGCTCACGCGCCTTGAAAAAGTGTCTCCGTTCTCCAAGTTCCCGATTCTTGGCAACCGGTTTCTGCTGCTTCGTCTGCGAGGAAAAGGAGGCAATGGAGAAGTATGGGATGTCGTGGACTACGCCAACAACAAGCAGCTCTGCGCTCTTAAGCTCTCCACCTCAATTCGACACGCCCAACGCGAACACCACACGCACACTCAACCACCCCA--ACATTGTTCAGGTGGGCGAGGCCCCCTTCCTTATCCGATACCAGCAGCAGCAGTATACTGCGTTCACTGTGGCAAGCGTTCAGTCCGATCTTCAGCAACTCATTGAGA---TGTTCGAGCACTTTGATGATGACTATGCATACAGAGTGCTTTCCCAGCTTCTAAACGCACTGAAGTACTTGCACGATGGGCGTTGCTCACTACGACCTGAAGCCGTCGAACATTCTGATCGACCAGGGCGGGTGTGTGAAGCTA------ACCGACTTCGACTTGACTCGCGACGCCAAGGCACCTACGTCCACCTCTACCGTTGGAACTCTGCGATATCTCCCTCCTGAGTGCTTCCGTCCGAACCGCAGCGA---------CTGTGAAGCGACGGCTGAGAAGGCGGACATGTGGATGGTGGGCATCGTGTACTGCATGATGGTTACGGGAAAGCACCCAATCTGCCCGGACAAGTCGACGCAAGCGGAAGTGAAAGCGACGATGGCTCAGTACACTGGC---GAACTTCGATATGCGAGGACTATTTCTGATCTGTCTCGGTGGGTCTTACAGGGCTGCCTGCACCCCGACCCGCGGTGTCGGCCAACGGCCGCACAATTGCTGGTGGCGCTCCAG

Phytophthora_capsici TGGACTACCACCGCCAAGTGGCCCGGGAATTGGCCGCCTTCTTCCACTCGTTCTACCGCTTCGGCGCCTCGTCCAACCGCTTCGACCTGTTCCTGCACAACGTCCTCTTTGGCTCACTCTGTATCGGAGCTGTCGTCTTCCTCGTGCTCTGCGTCATTGTGGTGCGCCGCACCGTCCTGCACATCCGCAGGCGTTTGTTCGCCGCACGTCGATGGAATCCACATCCACGGAGCTGATGGCAGTCGGACTATTGACTTTCCTAGCTATTAGCGCACTAGGAGGGCTATTAGGTGAAGCCGTCTCCAGACCAGCAACGCCCTACGTGTCAATGCAGATGGGTTGATCACGTCGTTCAACGACAGTGAACTCCCTGCTGAAGCTTTCAAGATGTCGGTGGAGGCTCTGGGCTCGTGCACGCGTCCAAGGAACTGGCTAACGTGACGAACGCGCTGCTGCCCAGTAACTACTCGGAGATCGCCAAAGACTGGCAGTTCAGCTACTTCGTACTCAAGTCGTCTACAAACGGAGCGATCTTGGCTGTAGCTCTCGCCAGCTTCTTGTCCATCGCGTCTGTTGGTTGGGCGATGGTTTCCCCGCTGAGAGTCGCCATTCTCGTGATTCTGAGCGTTGTACCCATCTCGCACACTTTGATCGGTGCGTACTTATCGGCAACGATCTTGACGGCGGACTTCTGTGCTGCTCCATTGAACAACACACTTGGTCTGGTTGGAACATGTCCTGCCAATGTGTCCTGCCTTTTGCTGAGGCGATGACAACGGCATGCAGCGCAACAAGGAGATATTGGCCAACGCATGAAGAAAGAGTTCCTGGATCCAATTGGAGCAACTATGGGGGTTATGTTGGGTTATTTCGCGGGAACAGTGAGCAATTGGGAGACTTTTGCGCTCATTCAGACGTGGCGGTGGCCGTTTTTACTGCAGTTTTTCGCTCTTTTGCCCATTAATGTGGCCATTTTCTTCGTGCCAAAGAAACATTTGATTATTCGCAGTGAGACGAGCGAGATGGAGCCGCCAGTGGCTGGAACCCCCATGAGCACGAGATCCACCACGTACCACGACGAAGAGGACGCGCTACTGGCCAGTGCCAGTATGCGACCTACGGCGCTTTAGACACGAGGTGTACGAACAGGGCGCGTTCTCGGAAGGGGTGTACGAGCTGCTGCACATTCCCGTCTTCTGCTTGATCGTCTTCGGCCTCACAACCGTGTACTTTGTCGTCACGGGCGTGCAGTACTGGAGTACCATCTTCATGATCAAGTCCCTGCACGCGTCCAAGTACCTGGTGAACGCGCTCTTCGTCGTCGTCTCCGGTACTGGACCCATCCTGGGCGTCTTCTTTGGCGGCTGGCTGATCGACCGGTACGGAGGCTACATCGGCGTGGAGCAGCGCGCGAAGGCGCTGGGTATTTGTATGATCCTGGGCCTCACGGCGTTCTGCATCTCGGCGGTCACGACGTTCTTCGACGACATCTACATGACCGCCGGCTTTCTGTGGCTCCTGCTCTTCTTCGGTGGCGCAATCCTGCCAGCGCACCGCGCACTGGCGTCCTCGTTCTCTGTCATGGTCTTCAACCTCTTCGGCTACGCGCTGTCGCATGAGCGACCTGCAGATGGTGGTGGGGGCCAAGTGCTTCGTGCCGGACGAGACCCACGTGTGGCTCGCGGCCGAAGTCCTGCGCGACGAAAACTCGGGAGACGGCAAGACCCGCAACATCTACTGCAAGGTGGAGCTGCCCGACGGAAACCGAAGAGCGCTGTGTGGATATGCTGTCCAAGAAGACCAAGGCGCTGCTGCTGTGCACCAGCTGGAGTCGCTGCCGTATCAAAACGAGAACGTGGGCCCTGAAGGAATTGAAGACATGATCACACTCAACTACCTGCACGAAGCCGCCATTCTGTTCAATATCAAGAAACGTTTCCAGTGCGAGCTGCCGTACACTTACACGGGTGATATTTGTATCGCTATCAATCCTTACAAGTGGCTCCCGGACCTCTACGCCGAGGAGCAGCACCTCCGCTACCTCAACCAGCCCAAGGAGGAGCTTCCCCCGCACGTCTACGCCACGTCAGTGGCTGCTTACGACAATATGCGGCGCTCGGAACGCAACCAGTCTATCCTGGTGAGTGGAGAATCCGGTGCCGGTAAGACGGAAACGACGAAGATCCTAATGAACCACTTGGCTACCATTGCCGGTGGTCTGAACAACTCTACTATTAAGAGGATTATCGAAGTGAACCCATTGCTCGAGTCCTTCGGTAATGCCAAGACCGTCCGTAACGACAACTCCAGTCGATTCGGAAAGTTCACACAGCTGCAGTTTGACGCTAACGGCACACTCGTCGGCGCCAAATGCCGCACGTACTTGTTGGAGAAGACTCGTGTGATTCAGCATGAAGCACCTGAACGCAACTACCACATCTTCTACCAGTGGACTCCTCTGAGATTGCACCGGAGCTGCAGCTCGACCCATCTAAGCACTACGTGTACACGGGTGACAACACAGCACAGAAGATCGAAGGCCTCTCGGACGAGAAGCACTTTAACAAGACACGCGAGGCGCTCGAGCTGGTGGGTCTGTCTCGCGACGACCAGAAGCCTCTCTTTGAGGTGCTGGCTGGTGTTCTTCACCTCGGTGAGGTGCAGTTGCAGTCGGACCCAACTGATGATGAGAAATCTCTCATCACGGAAGGTGACAGTGGCGCTCTTTCTGCCACGCAGATGCTCGGTGTTACGTGGGAGGCGCTACAGACCGCTCTGTGTTCGCGTACCATGCGTGCTGTGAACGACGTGTACAGCGTGCCACTGAAGAAGGAGCTGGCTATGGACTGTCGTGATGCACTCTCTAAGGCCATCTACTCGAACGTCTTTGACTGGCTTGTGCAAACAATTAACCAGTCCCTTGCTGACGATGCTCATATGTCCAACCACGTTGGTGTCCTTGATATCTTCGGTTTCGAGCACTTCAAGCACAACTCGTTCGAGCAGTTCTGTATTAACTACGCCAACGAGAAACTGCAGCAGAAGTTCACGCAGGATGTGTTCAAGACTGTGCAGATCGAGTACGAAGAGGAAGGTATCGTGTGGGACCACATCGAGTACGCCGATAACCAGGATGTGCTGACCGTGATTGAATCGCGCATGGGTATCATTTCACTACTGAATGAAGAGGTGATGCGACCGAAGGGTAACGAGGAGTCCTTCATGTCCAAGGTGACATCCCTACACAAGGAT---GACATGGCACACGTCATTGAGTTCCCTCGTACGTCACGCACAGAGTTCCTTATCAAGCATTATGCTGCTCCTGTGATGTACGATTCGGTGGGCTTCCTGGAGAAGCACAAGGACTCGCTGCTGCCTGATTTGTCCGAGCTTATGCGTGGTTCGTCCAAGCCCTTCATCGCCAAGCTCTTCGAACCCAAGCCAGAGCCCAAGCCGCCGAAAGC---------GTGGTGGTGCTCTGTCCATCACGACTGTCGGCACCCAGTTCAAGGAGAGTCTGACCGAGTTGATGGCTACTATTAACTCGACTCGCGTGCATTACGTACGTTGCATCAAGCCCAACCCGATTAA---------GAGTTCCACGGCCATGGACCAGAACATGGTGGTTTCCCAGTTGCGATGCGCTGGTGTGATTGAAGCCATTCGCATCTCGCGTGCAGCGTACCCGAACCGGTTGCAACACACGGAGATCCTGGACAAGTTTTGGCTATTTGTGCCTAGTGGTGGTGACACTGCAGCAGAGAAGTGCCAACTGCTAATGGACAAGCTCAAACTGGAATCCCCGACACAGTACCAGATGGGTAAGACCCGCGTATACTTCCAACTTGGCGTACTTGAAGAGCTCGAGGACCGTCGAAAGAAGTTCCTGGATGCCAAGGCCACTTACCTGCAGAACATCATGGTTGGCTTCACCCAGCGTATCAAATACCTCCGACAATTGGAAGCGATCATCAAGTTCAGTGATCCGCTGCGTAATTGCTATGCGCAGATACAACACGTTCCTGAAGGGCCTCATTTCAGCGCAAGCGCACTGGCGAGGCATGCAGGGCCGTAAACTGGCTGCTGATGTCAAGAGTAACCACAATGCTGTCATCATCCAGCGGTATGTGCGTGGATTTGTCAAGCGCCACCGTTACACTAAGATGCGCGCGATGGTCATCCGCGTGCAGGCTGTGGTGCGTATGACGATCCAGCGACCAAAGTATCTTGCTGCTCTCGAGGAGAAGCGTCGTGAGGCAGACATGGCTTACCAGCTGAATAAGCTAAAGGCTGCGCTGCAGGAAGAACAAGAGCGGAATGCGCAGCT------GCAGCGCC---GCAGCAGCGTGGC--GACGGCTCTTCCGCTGCCTCGAGCGTGGTCATGGCTGACGCTGGCGGTATGATCGAGACTCTTACGGAAGAGAACAAGAAGCTGCGTGAGAAGAACGAGGACATGAAGGTGACGATGAAGGGCTTGAAGGCGGAGATCGAGAAGTTCAAGAGCGACAAAGAGTTCTCATCTGCTGGTAACCATGTCAAGGTCCGCCAGCTGCAGGACACTGTGCGCGACAAGGACAAGAAGATCTCGCATCTTGAGGCCGAAAACAAGAAGCTCACGGAGCAGATTGCCAAGCTTCACGAGGATGGCGTTGT---TCCCGAGAAGAAGCCCACACCGAAGAAGTCGATCTTCCGCACGCTGGGCTCCAAGAAGGAGAAGAAGCCTC---------------GTGAGACTGTGCTGATGGACTCGCTCACCGGCTCGGACGACCTTAACCGTCGGGAGACGCGTCCTTCGGA---GCGTCACTTCCCGCGCATGC---CGAGCATGAGCGTGGGTTCT---CGCTTCTGGAACTCTAACAAG---AAGGGCGAT------------ACCGGCGACGGGTTTGATGTCTCGGAAGTTGACGGCGAGGACAGCCCCACGAACCCACGCGCAATACGATCGTGAAGAGCATGGAAGTGGGCGTGACCGGTGCCATGTCGAGTCTGAAGGGCCGCATG---------TCGGTCGTGAAGGGCATGTACGAAGCAAGCGTACGAACAAGACCGCCCTGTGCGCGAGTCGTTCAACCTAGATGCGTTGCCGGAGGTCTCTCTCCCTGTGGGTTGGGAAGCGAAGGTGTCGCGCTCAACGGGTCGTGTGTACTACGTCAACCGCAAGCTGGGCAAGTCCCAGTTCGAGCGCCCCACTTTAGCGTCGCTGAA------GGCGCAGAAGCTGGCGCGCCAGAAATCGGGTAATGCGCTGTAAGGCTGTGGTCGCCGATTTCGGCTGTGGAGATGCTAGACTGGCCGAGAGTGTGCCAAACAAGGTGCACTCGTTCGATTTGGTGTCCCTCGATATTGCTGTGTACTGCTTGGCTCTCATGGGGACAAGTGTTCGCGAATATGTGCGAGAAGTTCACCGCGTGTTGAAGCCTGGAGGTGTGCTGAAAGTAGCTGAAGTGAAGAGCCGCTTTGAGAGCGAGTCACTCGGTGGATGGTGGCGGTGTTCGTAGCGGCGGCCCAGGACGTCGTAGTGACTGTGCGCAATACGACGGTGTTCGTTGAGAGACTAAGTACGCGGTTATTGTGGGTCAGAAGACTGGAGAAGCTGTGGCTGTTCCCTTCCCAGACGTCGACCGCGACCTGAAGACTCTACTGGGTCACACTACGTCCATGGTAACTCATGTGGCTGTGAACCTCGTCATTGCTGCTTACAGCCGACCGGGATGAGAAACTGCGAGTGTCGAGGTTCCCTAACGCTGCAATCATTGAGAGTTACTGCTTAGGCCACGCTGCAAGTCTCACGAAGGTGGCGTGCAGTGCTGTGACGCCGGATCTAGTTGTGTCCACGTCCATGGACAACACGCTGAAGCTGTGGCAGATGTCTACAGGCAAACTGGCGATCTGTCCTAAGACAACGTTGTGGCGGTGCTCGTGAACTACCAGCACGCGCGATTCTTCGAGATCGTGAGGCTCCGTTTCTGCAATTGTTTTCGATCTCCAAGGTGTACCGCTGCATTCAACTGCTTTGCGCGACTTCCGCAAGCTTCAGAACGACCCGCCGTCTGGTGTGTCGGGCGCCCCCATGGACAACAACATCATGCTCTGGCAGGCTGTCATCTTCGGGCCCGATGACACTCCCTGGGAAGGAGGTACCTTCAATCTGACGCTTGAATTCAGCGAGGACTACCCAAACAAGGCGCCGACCGTCAAGTTCATCACGAAAATGTTCCACCCCAACATCTACAACGACGGTCAGATCTGTCTGGACATCCTGCAGAACCAGTGGTCGCCGATCTACGACATCTCAGCCATCCTCACGTCCATCCAATCGCTGCTGTGCGATCCCAACCCGAACTCGCCAGCCAACTCGGAGGCCGCGCGTCTCTTCCAGGAAAACCGACGGGAGTACAACCGCCGTGTTCGTGAGATCGTCGAGCAGAGCTGGCAGGCCATCGATGGTGTCGGCCGCCAAGATGCGTGGCGCTGAGAACCGCATGAACGCCGGTCGCCCCTTCACTGCGTGGCTGGACAACGTCTCGTCGGACCGTGGTCTGTGCGGTGGTATTAACTCTGGTATTGCCAAGACCACCCGTAAGCAGACCACTCGCAGATCTTCGTGATCGGTGACAAGGCTCGTGCTCAGCTGCGTCGCGACCTGGGTGACAACATCCGTGGCAACGTCACGGAGACGTACCAGTCGCCCCCGAACTTCACGGTGGCCAGTGCCATCGCGGAGGCTGTCGAGTCGGAGAGGAGTCGTCGCGTATGGCTGCCATGGAGAACGCCACCACCAACGCTGAGGACCTCACGCTTACGCTCGTGTACAACAAGGCCCGCCAGGCTCGTATTACCACGGAGTTGATTGAAATTATCTCGGGTGCCGCCTCTCTGGACAGTGACCGGCAGCGTCGTGAGCGTCCAGAGCGCCGCACAGTTCGATGAAGCCACTGCGCGGGAGTCGACTCTGTCCGTCTCCTTCTTCTGGGCTGAATTCCAGGCCTGTCGCCCTAACGGCCAATTGGACGTCGGGTCGCGCTGTGCTGGACAAGCTGGAAGGCGCCAACGTGGCGGAACTGGCCAAGCGCGTCTACAGTACCGTCTCAAAAAGCTCATCAGTGCTTCTCCTGTGATGCTCTTCATGAAGGGCAACCCAACGGAGCCCAAATGTGGCTTCAGTCGGCAGATGGTGGCTCTGCTTAACGAAGAGAAGATCCAGTTCGGGACTTTCGACATTCTCAATGATGACGAAGTGCGTCAAGGGCTCAAGCAGTTCTCCAACTGGCCTACGTACCCGCAGCTGTACGTGAACGGCACACTCGTTGGTGGTCTGGACATTCTCAAGGAGATGAAGTCGGACGGATCTATTGTGGAACAGCTCGGCGTTGAAGAAGCAGAAGCAGCTTTCCAGGAGAGTCTACGTGCACTGGTGAATTCTGCTCCTGTGCTCTTGTTCATGAAGCTTCGACATTTTGTGTGGAGCTACGAGCGAAAGAGGCAAGAAGTTGCCAAGGTGGTGAGTCTGACACCTACATCGTTCCGGAAGGAGATGTGCTGAATCCATTTCATGGAGCCCGAAATCGAATGGCTATGGCGTTTCGAGATTCTCTGGAGGATTTTGGAGAGTTCTGCATTGTGCAGCTCCCCGTATTACCTCGAATTGCACAGTTTTGCATCCACGAGGTATTGCTGCGATTGACTGGTAAGTTTAATGTTCTACACCGAGACCACAAAAGCGAAGTCAAAGCGCTCTGTGTGCACTCATCTCGTACCTGTTTGTACACGGGTGGCCTGGACGGACTGGTGATTCGTTACGACCTGAACAAGGGCTACAGGGATGTCTGGATAGAACGCCGCAAAC--ATCTCCAAGATCAACTCGATCCTGGAGCACCCGCACAATCCGAATCTTCTCATGGTCAGCTCGATTGAACATTACGCAGAATCTGTTGCTGCACGACCTTCAGCCCTACGACCCCAATGTGTCAGGCACCGGAATGAGTCAGTACCTGGTTCCACGGTGGTCTCCTGCTGGTTACCACGTGTCTTGCGGCAGTAAATGCGCTACTGCCACGGCAGCTTCTCCACACCCACGTCGGCCACCATCGGGGCTTCATTCCTACAGAAGCGCGTAATCGTCGGGGGGAAGCTGACACTGCAGATCTGGGATACAGCAGGTCAGGAGCGCTTCCGCTCAATGGCTCCTATGTACTACCGTAACGCTAAGGCAGCCATCCTGGTGTTCGATCTACAGAATGAGGCCAGCTTTGAGAAAATTAAAGAATGGCTACATCTACAGCATCACGTGGGTGATGACATGTGGGCAACAAGAGCGACGTTTTGACTTCTCCAAGGCCCAGCAGTTCGCAGATGAGATCGGCGCCCTCGTTTTCCGGACGAGTGCTAAGTCGGGCGAGGGGGTGCAATCACTGTTTGAGAGCCTGGCGGTTCAGCTGCTTAAGAAACATGACCAAGAACAGCGGAGAGCTTCTACGTGGTCTACGTACCTGCAGATGAGAGCAAAGAGCTGGAGGAGTGGACGGTGAAGCTGCCCAAGGACAAGGAGGCTCAACTGGGATGTCTTACGGAACGTCTGCGCGATCACTTTAAGCGGGGAGCCATCGGAGAAGCAGCAGAGACCTTCAAGCAACAATTGCTGGCTCAGTTGCCTAAAGGCGCCACTATGACTGATGAAATGCTGCAAATGATGCATGGACAGTCTGGTGGACTCCATTCCTCTCGTACTTAACACACCCGACGTCAAGCACGTTGGAGGTGAAGGGTGATGCCTTTATTGGACGGGTCTTTGATAGATGACTTCGTTCGCATGGATTTCCGGTTGGGGGAGGTGTCGGGTGACGCGTCTTGGGTGCAGACAGCCAAATTACAGAGGTTACGCAGTCGCTGGAGGAGATCTTGGCGCTGGGTCAGGGACTGGACGATCAGGACTACAAGGCCGAACTGGAAGTGCTGCAGAACGACCCGAACTCGAACCTTTACTCTGCCGTCACCTTTGAGGAACTGAATCTACCGGAGCCGCTGCTCAAGGGCGTGTACAACATGAAGTTCGCTAAGCCGTCGAAGATTCAGTCGGTGGCGCTGCCTCTCATCCTAGCCGACCCTCCAGAGAACCTGATCGGACAAGCCCAGAGCGGTATCCCACTCGTGAGCTTGTGCGGCAGATTATGGCTGTAGTGAACGCCATGGGCAAATTGTGGAGACGTTCTTGGCCATCCCTGGCAACGATCTGGCACGTGGTGAGACACTGACGGCTCAAGTGATCGTCGGTACTCCAGGAAAGATTGAAGGAATGATCAAGAAGAAGCAGTTGGACACCCGCAACGTCAAGATCTTCGTACTGGATGAGGCCGATGTGATGGTTGCAGAAGACGGCATGCGTGAACGCTCGGTTGCTATCAAACGCATGATCAAGAACCGTGCGTGCCAGTACCTGCTGTTCTCAGCCACGTACGCTGATGATGTGCGCGACTTCGCTCAGAAGATGGTGCCTGACCACAACATTATCACGGTCAAGAAGGAGAAGCTCACGCTGGATGGCATTAAGCAGTTCTGGATCGACTGCAAGACCCGTGACAACAAGTTCCAGGTGCTGTCGGACATTTTCGCCATTTTGACCATCGGCAAGTGCGTCATCTTCGTGCAGCAGCGTGAGACTGCCAAGGAGCTCACGCGTCGTATGCGTGAGAAGGGCCACTCAGTTGGCATTCTGCACGGTGCTGACATGGCTAAGGAAGTGCGTGACCAGGTGATTGACGAGTTCCGTGCTGGCACGACCAACGTTTTGATCACCACCAACGTGTTGGCTCGAGGTATCGACGTGGCGGGTGTTTCTCTTGTTGTGAACTTTGATATCCCGCTGACGCGCGATCACCGCCCGGACCCGGAGACGTATCTGCACCGTATCGGGCGTACTGGTCGTTTCGGTCGCAAGGGCTGTGCTATTAACTTTGTGTACGACAACAAGAGCAAGCAAGACCTCGCGGAGATCGAAGAGTACTACGCACGCCCTATTACGCAGGCTCCTGCCGACGACATCGAAGAACTCGAGAAGATGTTGGCCTGGGGGGCAGTAAGCGCCGTCTGGCCAAGCTCGGCAAGGAGCAGCGCAAAGGGATGCGGATTTACCCTCGCGAACCCAAGAAGAAACCGTCGGGTAAAGACAAGACGTACTACTATATCAAAGATGTCATGTTCCTGGCTCACGAACCGGTATTGGAGAAGTTTCGAGCGTTTAAAACGTTCATGAAGAAAGTGAAAAAAGCTATGGGCAAAAAAGACTATGAAGATGCCAAACGCAAGCACGAGAACCGGCCGACGTATGAATTGGACCATATTGTCAAGGAAAGGTATCCTCGGTTTGTGGACGCTTTGGGAGATCTCGATGATGCCCTTTGTGGCAGAACTACATTGTAGCCACTCGTTCATTGACCAAAGTGTTCGTGAGTATCAAGGGAATTTACTACCAGGCCAATGTTCAGGGACAGACCATCACGTGGCTCGTACCTCACCAGTTCACGCCTACAGTGGACAAACGGGTGGATGTGCGTGTGATGCTGACGTTCCTCGAGTTCTACGAGGTTCTACTCAAGTTCGTCAACGCCCAGCTCTACATGCAGATTGGGGTGGCGTACCCTCCCAAGATCAACCTCCAGTTGGATGCTGCTGGAGCTCAACTGGCTGCGTTGGAGCTAGCAGCAGAAGGAGTCCGAGAAGCGGATCAAGCTACAGCGAGCAACGGAAGGAGCGCAGCGAGAAGAGTCTGTTTGGTGGATTGACCTTCTTCTTGTCGCGTGAAGTACCGTCGGCTCCTCTGGAGCTTGTGCTGCGTTCGCATGGTGCTACGCTTGGCTGGGACGGAGAGGGTTCTCCTTTCGATGAGAAGAGCTCTCGTATCACGCACCACATCATGGATCGCCCGCACCAAGGCCACCGCTACTTTAACCGCGAATACGTGCAGCCACAGTGGGTGTTCGACTCTGTGAACAACGGGATTCTACTGCCTCTTGCTCGCTACCGCAAGGAACTGGAGAAGCTCAAGTCGGCGTCGCAGGTGCTGCGTGAGGTCGGAGCTGTTGGAGACGAGGAAGAGGACGGGAGGCGTACGCTGCTGATCTTAAGGCTGAAATGGACGGGAACAAGAAGTCGAAGAAGGCCAAGAAGGAGGAGGAAGCTGCGGAACTCAAGGAACTGGCCAAGACTATGATGACGAAGAAGTCGAAGCGTCTGTACGACCGCATGCAGTACGGTCTGGGCAAGAAGGCTGACAAGATCCAGAAGCTCGAGGATAAGCGTGCTAAGATCGAGGCAAAACCAAGAAGCTACTGGCGGGTGCACTCTCGGGTATGATATCGGCGCTGGCGTTTCAACCTTTGGGGTGCTACGCTGGCGTGGAACGTCCCCCACACTCGTGCGTGTAGCTGGCGGTGCAGGTCTCTACTTCGCTACACTGGATCACTGCCTCAACATGTTCCCACACACTGCAGTCAACACGTTCCTAGCTGGAGCCTTTGCACGCACATTCGCTGGAGGCGTCATGTCGCCCTTTACTATTGTCAAAGCAAGAATGGATACCGCCTGGTACATTCGACAGCAAAGGGACGTGCCATTCTCCGGGCTGTACGTGCTCATTTACACGGCATCCTGCTGACGTGGTTAAGACGCGCATGCATGCGCCAGACGGTGGCTAAGATCTACCGTCATGAGGGTCTGCGCGGATTCGCAAAGGGAATTCTTCCGCGTGTGGTCAAGCGAACGCTATCCACCGCGGTCACCTGGACAATCTACGAGCTACTCGTTCGACCCGTCGGGTCTAGGTCATGCGAAGGAGGCTTTCAACTTGGCGAAGGAGACGGAGCGCACCAAACAGATGGAGAATCAGGCCAAGGTGAAGGAGAACGAGGCGCTGTACAAGCAGTACGAAATCGTCCGCGTGCAGAAGGAGGGCGAGGAGCGCCGCAAGACGCTCGAGGAGGAGACGCGCCAGCACCAACAACGTGCACAGTACCAGGACCAACTTAAGCGCAAGCAATATGCCGATCAGCTGGCCGCTCAAAAGTACATGAAGGAACAGGAACTCAAGAAGCAAGAGGAAATCCTCGCTCGTCAGGAGGCCTCGCGCCGCAAGACCCTCGACTACGAGGCCGAGCTGCGCCAAAAAACTGAGCTGGCCAAGGTTGCGGCTGAAACTGAGGGCCGTATCAAGCAGGAACGTCTCAACCACGACCTCCACCTCGAGGAGGCCCGTGTGCGTGCCAAGGAGTACCGCGAGACCGTCATGGAGGGTATTAAGTTGGCTGGTAACACGGTCGGCTCCGGTATTATGACCTTCGTTGATGACAAGGAGAAGCTTACGGCTACGGTGGTGTCTCTGACGGCCCTCGCTGTTGGTATTTACACCGCCAAAGTGTCTACCAACGTGGCGGGTAAGTACATCGAGGCTCGCATGGGCAAGCCGTCTCTTGTTCGCGAAACCTCGCGTCGTTCGGTTGCGCAGGTGCTAGCTAACCCCATCCCGTCCATCAAGCGTGCCCTTCGTCTGCAGAAAGCTACGGATGCTCTCGAGGGTGTCGTGCTTGAGCCCAAGCTGGATGAGCGTCTGCGTAGCGTGGCTGTGTCCACTTTCAACACGAAGAAGAACCGTGCGCCGTTCCGCCACTTGTTACTGCACGGCCCTCCTGGTACGGGTAAGACGTTATTCGCCAAGGCTCTGGCTCGTCACTCCGGTCTGGAGTACGCTATCCTGACGGGTGGTGATGTTGCCCCCCTGGGCCGTGAGGGTGTTACGGAGATTCACAAGCTGTTTGACTGGGCGTCTCACAGCCGTCGTGGTCTGCTGCTGTTCGTTGATGAGGCCGATGCGTTCCTGCAGAAGCGTAGCAACAGCGTTATGAGCGAAGATATGCGCAACGCCCTGAACGCTTTCCTGTACCGCACTGGTGAGGCTAGCGATAAGTTCATGATCGTGTTTGCCAGTAACCAGCCGGAGCAATTCGACTGGGCCATTAACGATCGTATCGATGAAATGGTTGAGTTCGGTCTTCCAGGCTTTGATGAGCGTGTGCGCATGCTGAAGCAGTACTTCAATGACTACATCCGTGCCCCGAAGAACTCGCGAGCGAAGAAAATTTACGCGAGGGTGTTATCTTCACGGTGGACCCCGTGGCTAACTTCCTTGTCCTAAGACACGGATCTTTCAGCTCGCAGCCTTGCAGGGTGTATCAGGCGAAGCTCAGAGCATTTTTGATGCTCTCAACAAGAGCCCATGCGCATCTACTGGCTCATCTTCGCTGTGGTGCTGGGCAACGGATTGTACACGTACTTTGCCGGGAAGGACGAGTCGTTTCTGGTGGAGAAGCTCAAGAGAAAAATCCCGTCCGAGTACCGTATCTCCGAGAAGTGGGACAAGTGCCTGGAGAACTTCACGCTCTACTTCGGTGCTGGCCTCGTGGCTGGCGGCCTCACGTCCCTCGTTCTAGCGCGCTCGGGCGCTGGTCGTGGACTTGTGGCTGGACTGGGCGTGCGGGTGCTGGATCGAGCTGGACGACGTGCCAGATGGCGTTTGCTGGTGACGCACAGGCAGCTCTGGACAAAACGGACAAGGCTGTGGGCGACTTGCCGGCCTTCCAACAGCAGACGATCGTGGACTTTGTGACGGCCTCGGCCACGCAGCAGAAGCTGCAGACTGTGCACACGCGGCTTCTGTGAGAAGGACGGTGTGTCCACCTTGGAAACGTACCTGGCCGAGCAGGTGCAGAACAAGTTCGTGGACATCGACGCTAACCTGGCGCTGCTCAAGCTCTACCAGGTGTACCCTGCTACGGTTAATGCCGATAGCTAAGGTTCTGGTGAAGGGCGTCATGGCGCTTCCCTCTACCTTTTTCACTGGTGTCCACGATGATCCCTGAGAGCATCCGTGAGGACGCTAATGTGACGGCGGCGCTGCACACTGGTTTCATGCTGCAGTCGTGCCTCTTTGAGGACTTCTGGAAGGAGGACGTGAGCTTCGCCGAGAAGGTGCCAGGCTTCCTGGAGTCGGTGCGCGCTTACATTTTGACGGCTATCAGCCGTAGCCACAGCGCTATCTCTACGGAAGTTTTCAAGGCCAAACTCAACGTGTCTGACAAGGAGGTGGCCGACATTGTCGCCGAGAAGTGGACGCCGACAATTTGATTCAGATCAACCCCAACGAGGATAACCAGATGCAGGCCAAGAAGGTTCAGGAGAACATCGAGTTCGAGGACGTGCTCAAGGTCATCCACACCCTTTCTAGATCCAATGTCTGTGCTAGCGGTGGTGGTGTGGCTGACGCAGAGGAGGTCTGTGATAACCTGGCCAAGCACTTGAGGCTGGAGGGAGGCACACAAGTGCTGCCCTGCTCTACAGGCGTCATCGGTTGGCGTATCCCTGTCGACGCTATGGTCGAGAACCTGCCCAAACTCGTCAACAACTTACAGTCCGATTCGGTGCTACCTGCTGCCGAGGGAATCATGACTACTGACTTGTACCCGAAGATCCGCTCCATGGACGTGTGCGGCGGTCGTATCGTCGGTATTGCCAAGGGTGCGGGTATGATCGAGCCCAACATGGCTACGATGCTGTCCTACATCCTTACGGACCTGTCGATTCCGCGTGACGTGTTGCGTCAACTGCTGACGGAAGTGGTGGCGGATACGTACAATGCGCTCAGTGTGGACACCGACCAGAGTACGTCTGACACGGTGACGCTCGTTTCGTCCGACCAGATCCCGTTCGACCTGAA---GCAGCTGGGCGAATTCAAGAAGGCACTGCACGAGGTGTGTCTTGGTCTAAGCGAGGATATTGTGCGCAACGGTGAAGGTGCGCACCACGTCATGCGTGTGCGTGTGAGTGGTGCCTTGTCCAAGGAAATGGCCAAGGGAGTGGGCAAGTCGGTGGTCAACTCGCCGCTACTCAAGTGCGCAGTGGCGGGCAACGACCCGAACGTGGGTCGTCTGGTCATGGCTGTGGGCAAGTACATGGGTCTGCACCACAAGGACGTGAACATCCAGCGCCGTATGAAGATCCGGATGGGTGGCGTTCTTATCTTCGAGAACGGGGAGATGGTGCTCAACGACGAGATTGAGGCCAAACTCGTGGCGCACATGCGCGACGCAATGTTGATCGAGCCCACGCGTGGAGGCCTTGACGCCAGCTCGCACAACTATCCACCGCACGGAAAGTTTGTCGAGATGGAGGTCGACTTGGGTGTTGGGTCCGAGGAGACGCAGGTGCTGGGCATCGATCTGACGCACGAGTACGTGGCTATCAATGCCGACTACCGCTCGTAATGGCGCC---GAAGAAAGGGAAGACCGCCGCCGCCTACCTTGCGTCTCTGGGCGTCACCCCCGC---TGCTGGAGGTGAGAAAAAAGACGAC---AAGAAAAAGAAGAAGAAAGGAAAGAAGAAGCCCAAAAAAAGCCCACGTCAGCCGCTGCCAAAGCTCTGA-TGGAGCGTCAGGCGGCCATGAAGGCTGAGCAGGAGCGCCTGGAGCAGGAGCGTTTGGAGCAGGAACGTCTCGCTGAGGAGGAGGAAGCCAAGCGCCTGGCCGAGATCGAGCGCGAGGAAGAGATCCGCCGCAAGAAAAAGGAGGCACGTGCTGCCAAGATTGAGCGCCAGAAGAAAGAAGGAACGTACATGACCAAGGCTCAGAAGCAGAAGGCTGCCAAGGCTCAGGCTGCTCTGGAGGCTATGCAACAGGCGGGCCTCGTCCCCGATGCCACA------GGCGAGAAGAAGAAGGTTGTATACTCGAACAATAAGAAGAACAAGAAAAACAACAGGAGGAAGAG---GAGGAGGACGAGGCCCCCGACGCCTGGGATGATGAAGACTGGGAGACCAGTGATGTGGTCGCCTCGCTGGAGGACAAGTTAGCAGAAGAAGGAGCAGGAACGCCTGCGTCAACAGGGCATTGCACTGAAGAAGCGCCAAGAGGAACAGGCGGAACAGATGCGCCAGGAGGCTGAGGAGGAGCGCCAGCGTTCTAAGGCGAAACTTGAAGCCGAGCGCAAGAAGAAAGAAGCAGCTGAACGTCGTCGCGAGCGTGAGGCTGCCGCTCGTGCCAACGTATCCCTAGACAACCTGCGTTCTCCTATTTGTTGTATTATGGGCCACGTCGATACTGGTAAAACGAAGCTTCTCGACAACATTCGTAAGACCAAGGTGCAGGACGCCGAAGCAGGTGGTATTACACAGCAGATTGGTGCCACATTCTTCCCTGTGGATGCTATTAAGCAGAAGACAGCACGTCTCGCCGAGACGATGAAGCTGGAGTACCGCCTGCCTGGTCTGCTTGTGATCGATACGCCTGGTCACGAGTCTTTCACGAACCTGCGTAATCGTGGTTCGTCACTGTGTGATATCGCCATTTTGGTGGTTGATATCATGCACGGTCTAGAGCCTCAGACGCTCGAATCACTGCGCCTGCTCCGCCAGAAGAAGGCCCCATTTGTGGTTGCTCTTAACAAAATCGATCGTTGCTACGGCTGGAAGACTATGCCTGACATGCCTGTTCAGGAGGCCCTCAAACACCAGAACGAGCACGTCATCCGCGAGTTCGAGGACCGCATGAAGTCGATCATTGTGGAGTTTGCTGAACAGAAGCTGAACGCTGAGGTGTACTGGCGTAACAAGGATTTGGCGCGCACGGTCTCGCTCATTCCTACCAGTGCTATCTCAGGAGAAGGTGTGCCGGATCTGCTGATGATGCTAACCCGATTGACGCAGGAACGTATGGCCAAGTCATTGGCATTTGTAGACATCCTACAGTGTACTGTGCTCGAAGTGAAGGTCATTGAGGGTCTCGGTACGACAATTGATACGATCCTGGTAAATGGTACTCTGGAGGAAGGTGCAACTATTGTAGTGTGTACATTGGATGGACCCGTCGTGACAACGATTCGTTCGCTGCTGACGCCGCACCCCATGAAGGAAATCCGTGTTAAGGGAGAATACCTCCACCACCAGAAGATGAAGGCTGCTATGGGTGTGAAGATTTGCGCACAGGGCCTTGAAAAGGCAGTCGCTGGCACCCAGATTTACGTGGTTGGACCCGATGACGATGTTGAAGAGCTGAAGGACTCCGTTATGTCTGACCTGACAGGTATCTTGGACTCCGTGAAGGCCACTCGTCGTGGCGTCATGGTTCAAGCTTCCACTTTGGGTGCCCTTGAGGCCCTTTTGGAGTTCTTGCGTACTTGTGACCCTCCCATTCCGGTGTCGTGCGTGAACATCGGCCCTGTGCACAAGAAGGATGTCATGCGCGCATCAGTACAGCTGGAACATCAGCCTGAGTTCGCCACTATTTTGGCGTTCGACGTGAAGGTTCACAGCGATGCCACTGAGCTGGCTTCTGAGCTTGGCGTGCGTATTTTCACGGCAGACATCATTTACCACCTTTTCGACCAGTTTACGGCGTACATGGACAACTTCCGAATGGCTCGTCGCGAGGAGTTCGCTGAGGTCGCTGTATTCCCGTGTGTACTGAAGATCCTGCCTAATTGCGTTTTCAACAAAAAGGACCCGATCATTCTCGGTGTTGACGTCGAGGAGGGTATTTTAAAGATCGGCACCCCGCTCGTCGTCCCGTCCGCCGGAG---GTTTTCTGGTTGGTAAGGTTGGCTCCATCGAACGCGAACACAAGGAGGTGGATCGTGCCAAGAAGGGCGCGTCTGTCGCTGTGCGGATAGACAACGAGGGCAGCGTAATGTACGGCCGCCATTTCGACCACAAGAACAAACTGGTGTCGCGTCTCACCCGCCAGAGCATTGATGCGCTAAAGGAGAATTTCCGGGAAGACCTGCAGAAGGAGGACTGGCAGCTTGTCATCAAGCTCAAGAAGGTGTTCGATATCATTATGGAGGGCTGGCTCACCAAGCGCCGTGACCACATGAACCTCATATGGCTGGAGCGCTACTTCCTCTTGGACGGCAATCAGCTTCGTTACTACCGCAAACGTGGCGATCCAGCACCCCGAGGGACCTACATTCTGACTGATGGCTGTGTCGTGAGCCCTGTGTTCAATTCCGAGGAGAAACACAAGCACCACGGCCCCGTTTGGATGTTCCGCATCACCTGAAAAGTCCTACAAGAAGGACAGATTTTTGGATCTAGGGGCCAAGAGCGAGGAGAACGCCAAGGAGTGGAAACGGGCGCTGGAGAACGCCGTCCGATCGTTGAGAGAGCTGGTGGCGCATCAGAAGAAGCTCAAAACCGACTCCGAGAT---CTTCCCCACGCCCACACAC---------GTCCCGAGGATGCTCAAGACAGACGAAGACACTGTTTCTCTCTTTAATCTGGACGCTGACAACGTCAAAGCGGAGCAGGCCTGGTGGCTGGCCAGAGTGGAAGGAGGTCTCCGTATCATGCAGGAATGTCCGCTTGGCCCTAATGTCGCTGCCAATCTGTTCGCGCAACATGCAGCCACAACCAATGGACTGATCTTTAGTGCGTGTGTGGGTGCGGCTCTGAGCTTCAGCAGCATGTACTCGTTCGCGAACAACATGCTCCTGAGCTTCATAACGGCGTTGGTGGCGATTGCCGTCTGCTGTGTCGCTGTAACGCCAACACAGGCGCCCGAAATCCCGAGCTTCCGAGTCTCGCAGGTGGTGCATGGATCTCCAACTGAAGTGTTCCGACTCATCATGAACCCCAAGAGATTCCAGCGCTGGGATTCCGGAACTGCCACTATGCGGGTGATACAGCAGCTGGATGACCACGCGGACATTGTGTACGTGACGCAGCGGCCGACACACTTATGGCCACTGTGGCAGAAGGCTCGGGATCTTGTGCTTATGCGCTACTGGCGACGTGAAGAGGACGGATCGTACTTTGTCATGTACCAGAGCATCGAGCATCCAGAGTGTCGTGTGCGTCACAACTACGTCCGTGCGAATCTATTGGGTGGTGGCTTTGTGATCGCTCCCCAACGTGTTCCAAGT---GGATCCATCCGTACATTGGTCACGTACGTGCTTCGCTACGACTCGGGCGGTTG---GTCGCGCATCTACCACCAACTCGGCATGGACGTGGACCTGGTGATGCCTATGCTGCGCAGTGTTGTGGGCATTCGGGATGAAATGAGCGCTACAGACTTCATCACGCCTAACGTTTCGGTGGCTCCTGCAGGCGAAGGCAACAGCTGCTCAAGAAGTGTGGA---CTCCATGGGCGTTGTCCTGGGCGTGCAGAAGCTTCAA---ACGAGTCTTCCGGAGAAGATGTGGGCCGAACCGGATGCTTCTCTCTTTAGTGTGCGAGGCCACAACTACCTGAACGACAAGAAGAAGATCCCGTCGGCTCCAGCGATGTTCCACACCATGGGCGTGGATTTATTGTCGTTCGAGAGCCCAGCGGAGAGATACAACATCTCAGCCCGTTCGGACAGCATCGGCCGCACCAG---CTCCAAGTTCACGTTCGTAGTCAACATGATTATCCCCGGTCCGGAGAACATTTGTATGGTCTTCTACTTTCAGCCGAGACGCGACAACGTCTTCGAGGACGGATCGCCCTTCTCGGAGCTACTGAACGACTTTTTCGATGGCGATGACCAATTCCGGAACTCGCGCTTCAAGCTTATCCCCACTGTAGTTGAGGGCAGCTTCATCATCAAGCAGTCGGTTGGGTCCAAGCCGACGCTGCTCGGCAACAAGCTCAAGTGTCCGTACCACCGCGGAGAGAACTACTTCGAAGTGGACATCGACATTTCGTCCAACTCAGTGGCCAACACCGTCGTGGGAATGGTGCAGGGTGTGACCAAGTCGCTGGTGGTTGACATGGCGTTTTTGCTCGAGGCCCAGTCCGACGAAGAGCTGCCCGAAATCATCCTGGGCGCTGTTCGCATGCAGCACATCTCGCTTGATCACCCTCGCCGTATGCCCAAGCATGGGGAACGGGGAGCAAAGACGAGTTCACCTTCTGACGCCTTCACGGAAGCTCTGCGCGTAAATTTGCAGCGTCTCATCGTCTACGCGCGTTCAGCCGACACAGCGCTACAGCGGGAAGTAGCCGAGAAGTTGGCCAACGAGGCTGTTAAGACCGACAGGTGCAGATCGTAGAGCTGGACGGACTGCAACTGCTGCTTCCGCTCACCAAGTCCAAGGATACGGAGGTGCAGCGGTTGGCTGCACATGCACTCGCCAACCTATCGGTTAACTCCGAGAACCAGTCGAAAATGGCGACGGAAGGTGGAATTGACATGCTCATTGATCTCTTGAGTAGCACAAACGAGCACGTACAGCGGCAGGCAGCAAAGGCACTCGCCAACCTCGGCGTGAACGTCGACAACAAGGAGAGGATCGCCAAGGCTGGTGGCATTAAACCGCTCATCGACTTGGCTAGTTCTCGTCAGATCGGCGTCGCTGTTGAGGCCATTGCGGCACTTGCAAATCTAGCGGTCAACG---ATGCCAATGAGGTGGAAATTGCCCGTAAAGGTGGCTTGAAACCGATTATTGAAGGCGCGCACTCCGAGTCCGTGGAGCTTCAGTCCCAAGTGGCTCGAGCACTGCGTAACTTGTCTGTCAACCCGGAGAACAAACAAGCGATCGTGGAGCTGGGAGGGGTGGAGGCTTTGCAATCCCTTGTGCGCTCCACCAACGACCGAATATGCCAGCAGGCTACACGCGCGCTCGTCCCCAACGTGGTGCTCACACGCTCCTCACCCACAATTTGCTG------GTGGCACCTGTAGCTCGCGTGGCCGAAGTCAAGGAAGAGAC------TGTAACTTACAACGGAACTACGGTGAGTGTCGAGTACACGGCTGCTGTCAAGGCTGTCGTAGTCCCCGTCGTGTCTACTACGAAGCCCA---ACGATAAGGCTGGA---GGTTTCTCGTCTATCTCCGACCGTTTCCCGTTGTCTAAAGTGGGGAAAGTCAGCTGCGGAATGGCACCAATCCCCATGAGCCG---TTTGCTTAATG---CTACACCGGAGATGACAC---AGGTAGACCAG-------------ATGACTGTGCTGGTGGAAGATGGCACAGTGGACGACAAC---ACCATTAAAGCCGAG---CCGGAAGGTGAATGACGTGTACAGATACCCGGCAACAGA--AGTAGAAGAAAACGAAATGGAGCAACTTCGATGGAACATCCAAGAGCAGGTTTTCCAAAGATACTTCCATCAAGGACAGAAAGACGAGGAGTGGGGGCGACTGGAAGAGCAAGACGCCGAGTATTCTTCATTCCTGGTGGACGACGAGAAAACATATTGGCTGAGAAGCGATTCCACGACAATGCCGTCCGCGACGCCATGGGAAAGGGTAATGCTACATGGGTCAAAGATCGACGAGAACAGTTCGAGGTGGAGATCCGTCAGTTCTACGATGAAACTCAACACAAGCAAGAGCTGATGCTTACCCGTCTTGAAAAGGTTTCTCCGTTCTCCAAATTTCCGATTCTTGGCAATAGGTTTCTGCTGCTTCGCCTGAGAGGAAAGGGGGGCAACGGAGAAGTGTGGGACGTCATTGACTACGCCAACAACAAGCAGCTCTGCGCTCTCAAACTATCCACTTCAATTCGACACGCTCAACGTGAGCATCACACGCACACTCAACCACCCCA--ACATTGTTCAGGTCGGCGATGCGCCATTCCTGATTCGATACCAGCAGCAGCAATACACTGCATTCACCGTTGCTAGCGTTCACTCCGACCTTCAGCAAATTATCGAGA---TGTACGAGTATTTGGATGATGACTCTGCATACAAGGTTCTTTCACAACTGTTGAGTGCGCTAAAGTATTTGCACGATGGGCGTTGCTCACTACGATCTGAAGCCGTCAAATATTCTGATCGACCACGACGGGTGTGTGAAGCTG------ACCGACTTCGATTTGACTCGCGACGCCAAGGCAGCTACTTCGAACTCGACTGTTGGGACTCTTCGTTACCTTCCTCCTGAGTGCTTCCGACCAAACCGCAGCGA---------CTGCGAAGCCACTGCTGAAAAGGCAGACATGTGGATGGTAGGCATCGTGTACTGCATGATGGTTACGGGAAAGCACCCGGTCTGTCCGGACAAGTCAACGCACGCGGATGTGAAGACGACGATGGCTCAGTATACGGGC---GAGCTTCGATATGCACGGCCCATTTCGGACCTATCTCGGTGGATTTTGCAGGGATGCCTCCACCCAGACCCGCGATGTCGACCAACGGCTGCACAGTTGCTTGTGGCACTTCAT

Phytophthora_cinnamomi TGGACTACCACCGCCAGGTGGCGCGCGAGCTGGCCGCCTTCTTCCACTCCTTCTACCGCTTCGGCGCCGCCTCCAACCGCTTCGACCTGTTCCTGCACAACGTGCTCCTGGGCTCGCTCTGCATCGGCGCCGTCGTCTTCCTCGTGCTCTGCGTCGTCGTGGTGCGGCGCTGCGTGCTGCACATCCGCAGGCCTATGTGCGCCGCACCTCGATGGACTCGACGTCGGTGGAACTGCTGGCTGTGGGGCTGTTGACCTTCCTGGCCATTAGTGCTCTGGGGGGCCTACTGGGAGAGGCCGTTTGGAGACCAGCAACGCCCTCCGCCTCAACGCAGACGGCCTCATCACCTCGTTCAACGACAGTGAACTCCCCGCCGAAGCCTTCAAGATGTCGGTGGAGGCGCTGGCCTCGTGCACGCGTCCAGGGAGCTCTGGAACCAGACGGACGCGCTGCTGCCCAAGGACTTCTCGGGGCTCGCCAAGGACTGGGAGTTCAGCTACTTCGTGCTCAAGTCGTCCACCAACGGCGCCATCCTCGCGGTGACGCTGGCCAGCTTCCTGTCCATCGCCTCGGTCGGTTGGGCCATGGTCTCGCCGTTGCGGGTCTCCATCATGGTCATCCTGAGCGTCGTGCCCATCTCGCACACGCTGATCGGCGCCTACCTGGCGTCGACGATCATGACGGCGGACTTCTGCGCGGCCCCGATGAACAACACGCTCCAACTCGTGGGCTCGTGTCCTGCGAACGCGTCCTGCCCTTTGCTGACGCGGTGGGCACGCAACGCGTCTCGCCAGGGAGACGTTGGCCAGCGCATGAAGAAGGAATTCCTGGACCCCATCGGAGCAACTCTGGGCGTCATGCTCGGCTACTTCGCCGGCACGGTCAGCAACTGGGAGAGCTTCTCGCTCGTGCAGACGTGGCGCTGGCCCTTCCTGCTGCAGTTCCTGGCGCTGCTGCCCATCAACGTCGCCATCTTCTTCGTGCCCAAGAAGCACCTCGTCATCCGCAGCGAGACCAGCGAGCTGGAGCCCCCGGTGGCCGGCACGCCCATGAGCACCAGATCCACCACGTACCACGACGAGGAGGACGCGCTCCTCGCCAGCGCGAGCATGCAACGTACGGCGCATTGGACACGAGGTCTACGAGCAGGGCGCGTTTTCGGAGGGCGTGTACGAGCTGATGCACATTCCCGTCTTCTGTTTGATCGTGTTTGGTCTCACGACCGTGTACTTCGTCGTCACGGGCGTACAGTACTGGAGCACGATTTTCATGATCAAGTCGCTGCATGTGTCCAAGTACCTGGTGAATGGACTGTTCGTCGTGGTATCCGGAACAGGGCCCATTCTAGGTGTGTTTTTCGGAGGCTGGATCATCGATAGATATGGAGGATACATCGGTGTGGAGCAGCGGGCGAAGGCTTTAGGGATTTGCATGATCCTGGGCTTGACAGCCTTTTCGATTTCCGCGGTGACGACGTTCTTCGACGACGTTTACATCACTGCCGGTTTCCTGTGGCTGTTGCTGTTCTTCGGCGGTGCCATCCTGCCAGCGCACCGCGCGCTTGCCTCCTCGTTCTCAGTGATGGTCTTCAACCTGTTCGGGTACGCGCTCTCGCATGAGCGACCTGCAGATGATGGTGGGCGCCAAGTGCTTCGTGCCGGACGAGACGCACGTGTGGCTCGCGGCCGAGGTGCTGCGCGACGAGAACTCGGGCGACGGCAAGACGCGCAACATCTACTGCAAGGTGGAACTGCCCGACGGAGACCGAGGAGCGCTGCGTGGACATGCTGTCCAAGAAGACCAAGGCGCTGCTGCCGTGCACCAGCTCGAGTCGCTGCCCTACCAGAACGAGAACGTCGGGCCCGAGGGCATCGAGGACATGATCACGCTCAACTACCTGCACGAGGCCGCCATCCTGTACAACGTCAAGAAGCGCTTCCTCTGCGAGCTGCCCTACACGTACACGGGCGACATCTGCATCGCCGTGAACCCGTACAAGTGGCTGCCAGACCTCTACGCGGAGGAGCAGCACCTGCGCTACCTCAACCAGCCCAAGGAGGAGCTGCCCCCGCACGTGTACGCCACCTCCGTGGCCGCCTACGATAACATGCGCCGCTCGGAGCGCAACCAGTCCATCCTCGTGAGCGGAGAGTCCGGCGCCGGCAAGACGGAGACCACCAAGATCCTCATGAACCACCTGGCCACCATCGCCGGCGGTCTCAACAACTCGACCATCAAGAGGATTATCGAGGTCAACCCGCTGCTCGAGTCCTTCGGTAACGCCAAAACGGTGCGCAACGACAACTCGAGTCGATTCGGCAAGTTCACGCAGCTGCAGTTCGACAAGAACGGCACGCTCGTGGGCGCCAAGTGCCGCACCTACCTGCTGGAGAAGACGCGTGTCATTCAGCACGAAGCGCCCGAGCGCAACTACCACATCTTCTACCAGTGGACTCCCCCGATATTGCCCCCGAGCTGCAGCTCGAGTCGGGCAAGCACTACGTGTACACGGGCGACAACACGGCCCAGAAGATCGAGGGGCTCTCGGACGCCAATCACTTCAACAAGACGCGCGATGCGCTGGAGCTGGTGGGTCTGTCGCGCGATGACCAGCGTCCGCTGTTCGAGGTGCTGGCTGGTGTGCTGCACCTTGGTGAGGTGCAGCTGCAGTCCGACCCGTCGGATGACGAGAAGTCTCTCATCACGGAGGGCGACAGCGGCGCGAATTCTGCTACGCAGATGCTGGGTGTTACGTGGGAGGCCCTTCAGACGGCCCTGTGTTCGCGCACCATGCGCGCTGTAAACGACGTCTACAGCGTGCCGCTCAAGAAGGAGCTGGCTATGGACTGCCGTGACGCACTCGCTAAGGCCATCTACTCCAATGTGTTCGACTGGCTCGTGACCACGATTAACCAGTCGCTTGCCGATGATGCAAACATGGCCAACCACGTGGGTGTGCTTGATATCTTCGGTTTCGAACACTTCAAGCACAACTCGTTCGAGCAGTTCTGCATCAACTACGCCAACGAGAAGCTTCAGCAGAAGTTCACCCAGGATGTTTTCAAGACCGTGCAGATCGAGTACGAGGAGGAAGGCATTGTTTGGGACCACATCGAGTACGCGGATAACCAGGATGTGTTGACCGTGATTGAGTCGCGCATGGGTATTATCTCGTTGCTGAACGAAGAAGTGATGCGTCCTAAGGGTAACGAGGAGTCGTTCATGTCCAAGGTTACTTCCCTGCATAAGGAT---GACATGGCGCACGTGATCGAGTTCCCCCGCACATCACGCACCGAGTTTTTGATCAAGCATTACGCCGCTCCGGTCATGTACGACTCGGTGGGCTTCCTCGAGAAGCACAAGGATTCGCTGCTGCCTGATCTGTCCGAGCTCATGCGCGGTTCGTCCAAGCCGTTCATCGCTAAGCTCTTCGAGCCGAAGCCGGAGCCAAAGACGCCGAAAGC---------GTGGCGGTGCACTGTCCATCACGACCGTCGGTACCCAGTTCAAGGAGAGTCTGACAGAGCTGATGGCTACGATCAACTCGACGCGTGTGCACTACGTGCGTTGCATCAAGCCCAACCCGGTCAA---------GAGTGCGACGGTCATGGACCAGAACATGGTGGTGTCTCAGTTGCGCTGCGCAGGTGTAATTGAGGCCATTCGTATCTCGCGCGCGGCGTACCCGAACCGATTGCAGCACACTGAGATCCTGGACAAGTTCTGGCTGTTTGTGCCGAGTGGTGGTGAGACTGCGGCGGAAAAATGCCAGCTGCTTATGGACAAGTTGAAGCTCGAGTCGCCCACGCAGTACCAGATGGGTAAGACTCGTGTATACTTCCAGCTTGGCGTCCTTGAAGAGCTTGAGGACCGTCGCAAGAAGTTCCTGGACGCCAAGGCGACGTACCTGCAGAACATCATGCTTGGTTTCACGCAGCGTATCAAGTACCTCCGACAACTGGAAGCAATCATCAAGTTCGGTGATCCGTTGCGTGATTGCCATGCGGAGATACAACACGTTCCTGAACGGCCTTGTGAAGGCGCAAGCTCACTGGCGAGGAATTCAAGGCCGTAAGGTGGCGGCCGATGTGAAGAGCAACCACAACGCTGTCATCATCCAGCGGTATGTGCGTGGTTTCGTCAAGCGTCACCGCTACGTTAAGATGCGCTCGATGGTCATCCGTGTGCAGGCTGTGGTTCGCATGACAATTCAGCGGCCCAAGTACCTGGCCGCTCTCGAGGAGAAGCGCCGCGAGGCTGACATGGCTTACCAGCTGCAAAAGCTGAAGGCGGCTCTGCAGGAGGAGCAGGAGCGGAATGCGCAGCT------GCAGCGCC---GTAGCAGCTTGGT--GGCGGAGCTTCCGTTGCCTCGAGGGAGGTCATGGCCGACGCTGGTGGCATGATCGAGACTCTCACGGATGAGAACAAGAAGCTGCGTGAGAAGAACGAGGAGATGAAGGTTACAATGAAAAGCTTGAAGGTGGAGATCGAGAAGTTCAAGAGCGACAAGGAGTTCTCGTCGGCTGGCAACCACGTTAAGGTCCGCCAGCTGCAGGACACCGTGCGCGACAAGGAAAAGAAGATCTCCCAGCTAGAGGCCGAAAACAAGAAGCTGACGGAGCAGATCGCCAAGCTTCACGAGGAGGGTGTTGT---AACTGAGAAGAAGACGACCCCGAAGAAGTCGATCTTCCGCACTCTGGGCTCCAAGAAGGAGAAGAAGCCGC---------------GCGAGACCGTGCTGATGGACTCTCTCACGGGATCCGACGACCTTAACCGCCGGGAGGCGCGTATGTCGGA---GCGCCACTTCCCGCGCATGC---CGAGTATGAGCGTGGGCTCG---CGCTTCTGGAACTCGAACAAG---AAGGGCGAC------------AACGGCGATGGCTTCGATGTCTCGGAGGTTGACGGTGAGGACAGCCCTACGAACCCACGCGCAACACGATCGTGAAGAGCATGGAGGTGGGTGTTACGGGCGCCATGTCGACTCTGAAGGGCCGTATG---------TCGGTAGTGAAGGGCATGTACGAAGCAAGCGCACGAACAGGATCGCCCCGTGCGCGAGTCGTTCAACCTGGATGCGTTGCCGGAAGTATCTCTTCCCGTGGGCTGGGAGGCCAAGGTGTCGCGCTCGACTGGCCGCGTGTACTACGTGAACCGCAAGCTGGGCAAGTCCCAGTTCGAGCGCCCGACGCTGGCCTCGCTCAA------GGCGCAGAAGCTGGCGCGCCAGAAGTCGGCTAACGCGCCTTAAGGCGGTGGTGGCGGATTTCGGCTGTGGAGACGCCAGGCTGGCGGAGAGCGTGCCGAACAAGGTGCACTCGTTCGACCTGGTGTCCCTTGATATCGGTGTGTACTGCCTGGCGCTTATGGGGACGAGTGTGCGCGAGTATGTGCGCGAGGTGTACCGTGTTTTGAAGCCTGGTGGCGTGTTGAAGGTTGCGGAGGTGAAAAGTCGATTTGAGAGCGAGTCGCTGGGCGGATGGTGGCGGTGTTCGTGGCGGCGGCGCGGGGCGTCGTGGTGACGGTGCGCAACACGACGGTGTTCGTGGAGAGACCAAGTACGCCGTCGTGGTGGGCCAGAAGACGGGCGAGGCCGTGGCCGTGCCGTTCCCCGACGTGGACCGCGACCTTAAGACGCTGCTGGGCCACACCACGTCTATGGTGACCCACGTGGCCGTGAACCTCGTCGCTGCTGCTGACGGCCGACCGCGACGAGAAGCTGCGCGTCTCCAGGTTCCCAAACGCCGCGATCATCGAGAGCTACTGCCTGGGACACGCTGCTAGTCTGACCAAGTTGGTGTGTAGCGCTGTCACGCCGGAGCTCGTGGTGTCCACGTCCATGGACAACACGCTCAAGTTGTGGGAGATGAAGACGGGCAAGCTCGCAATCTGCCCCACGACAACATTGTGGCTGTGCTCGTGAACTACCAGCACGTGCGGTTCTTTGAGATCGTGAAAACCCTTTCCTACAGTTCTTCTCAATTGCACAGGGCCGCTGCCGCGTTCAACTGCTGTGCGCGACTTCCGCAAGCTGCAGAACGACCCGCCCTCGGGCGTGTCGGGCGCCCCCATGGACAACAACATCATGCTCTGGCAGGCCGTCATCTTCGGACCGGACGACACGCCCTGGGAAGGCGGCACCTTCAACCTGACGCTTGAGTTCAGCGAGGACTACCCGAACAAGGCGCCGACCGTCAAGTTCATCACTAAAATGTTCCACCCCAACATCTACAACGACGGCCAGATCTGCCTGGACATCCTGCAGAACCAGTGGTCGCCCATCTACGACATCTCGGCCATCCTCACGTCCATCCAGTCGCTGCTGTGCGACCCGAACCCGAACTCGCCGGCCAACTCGGAGGCCGCGCGCCTCTTCCAGGAGAACCGACGGGAGTACAACCGCCGCGTGCGCGAAATCGTCGAGCAGAGCTGGCAGGCGATCGATGGTGTCGGCCGCCAAGATGCGCGGCGCCGAGAACCGCATGAACGCCGGCCGCCCCTTCACGGCGTGGCTGGACAACGTCTCGTCCGACCGCGGCCTGTGCGGCGGCATCAACTCGGGCATCGCCAAGACCACGCGCAAGCAGACCGGTCGCAGATCTTCGTGATCGGCGACAAGGCCCGCGCCCAGCTGCGCCGCGACCTCGGCGACAACATCCGCGGCAACGTGACGGAGACGTACCAGACGCCCCCCAACTTCACGGTGGCCAGCGCCATCGCGGAGGCCGTCGACTCGGAGAGGAGTCGTCGCGCATGGCCGCCATGGAGAACGCCACCACCAACGCCGAGGAGCTCACGCTCACGCTCGTCTACAACAAGGCGCGCCAGGCCCGCATTACCACGGAGCTCATTGAAATTATCTCGGGTGCCGCCTCCCTGGACGGTGACTGGCAGCGTGGCGAACGTGCAGAGCGCGGCGCAGTTCGACGAGGCCACGGCGCGCGAGTCGACGCTGTCCGTGACCTTCTTCTGGGCCGAGTTCCAGGCCTGCCGCCCCAACGGGCAGCTGGACGTGGGGCCGCGCCGTGCTGGACAAGCTCGAGGGCGCCAACGTGGCCGAGCTGGCCAAGCGCGTCTGCAGTACCGCCTCAAGAAGCTCATCAGCGCCTCGCCCGTCATGCTCTTCATGAAGGGCAACCCGACGGAGCCCAAGTGCGGCTTCAGCCGGCAGATGGTGGCGCTGCTGAACGAGGACAAGATCCAGTTCGGCACCTTCGACATCCTCAACGACGACGAGGTGCGCCAGGGGCTCAAGCAGTTCTCCAACTGGCCCACGTACCCGCAGCTCTACGTCAACGGCTCGCTCGTGGGTGGCCTCGACATCCTCAAGGAGATGAAGTCCGAGGGCTCGGTCGTCGAGCAGCTCGGCGTCGAGGAGGCCGAGGCCGCCTTCCAGGAGAGCCTGCGCACGCTCGTCAGCTCGGCGCCCGTGCTGCTCTTCATGAAGGTTCGACATCTTGTGTGGAGTTACCAGCGCCAGGACCAAGACATCGCAAAGGTGGTGACTCTGATGCCTTCGTCCTTCCGCAGGGAGATGTGCCGAGGCCATTTCGTGGAGCCCGAAACCGCATGGCCATGGCATTTCGCGACCCCGTAGACAACCAGAGTGAGTTTTGCGTTGTGCAGCTGCTCGTCTCACCTCGAAGCGCACATCTATACATCAACGAGGTATTTCTGCGATCGACTGGCAAGTTCAGCGTGCTGCACCGCGACCACCGTAGTGAGGTTCGATCGCTATGCGTGCATTCGAAGCGCGACTGCTTGTACACCGGAGGTCTGGATGGCCTGATGATTCGATACGATCTGAACGAGGGGTGCACCCAGATTGTGATCGAGCGCCGCAAGCATATCTCCAAGATCAACGCCGTGCTGGAGCACCCGCACAACCCGAACCTGTTGTTGGTCAGTTGCGTGGAACAGTAGCCACAACTTGCTGCTGCATGACCTCCAGCCATACGAACCGAATGTGTCGGGTGGTACCATGAGCCAGTACGTTGTGCCGAGGTGGTCGCCTGCAGGCTATCACGTGTCGTGCGGCAGTAAATGCGCTACTGCCACGGCAGCTTCTCCACGCCCACGTCGGCCACCATCGGGGCCTCGTTCCTGCAGAAGCGCGTGATCGTGGGGGGGAAACTGACGCTGCAGATCTGGGACACGGCCGGCCAGGAGCGCTTCCGCTCCATGGCGCCCATGTACTACCGCAACGCCAAGGCAGCCATCCTGGTGTTCGACCTGCAGAGCGAGGCTAGCTTCGAGAAGATCAAGGAGTGGCTGCACCTGCAGCACCACGTGGGGGACGACATGTGGGCAACAAGAGCGACGTTTCGACTTCTCCAAGGCCCAGCAGTTCGCGGACGAGATCGGCGCGCTGGTGTTCCGCACGAGCGCCAAGACAGGCGAAGGGGTGCAGGCGCTGTTCGAGAGTCTGGCGGTGCAGCTGCTCAAGAAGCACGACCAGGAGCAGCGGAGAGCTTCTTCGTGGTGTACGTGCCCGCGGATGAGCGCCAGGAGCTGGAGGAGTGGGCGGTGGCGCTGCCGCAGGACAAGGAGGCGCAGCTGGGCTGCCTGACGGAGCGGCTTCGCGCGCACTTCAAGGCGGGCGCCATCCGAGCGGCAGCAGAGACGTTCAAGCAGCAACTGCTGGCGCAGCTGCCCCAGGGCGCCTCCATGACGGACGAGATGCTGCAGATGATGCATGGACAGCCTCGTGGACTCGATCCCGCTCGTGCTCAACTCGCGCGACGCGCGGCACGTTGGAGGTCAAGGGCGACGCGTTCATCGGCCGCGTGTTCGACAGACGACTTTGTGCGTATGGACTTCCGTCTGAGCGAAGTGTCGGGTGATGCTCCGTGGGTGAAGCAGGGCAAGCAGCAGAGGTCACGCAGTCGCTGGAGGAGATCCTGGCGCTGGGCCAGGGCCTGGACGACGAGGACTACAAGGCCGAGCTGGAGGTGCTGCAGAACGACCCCAACTCGAACCTCTACTCGGCCGTCACCTTCGAGGAGCTCAACCTGCCGGCGCCCCTGCTCAAGGGCGTGTACAACATGAAGTTCGCCAAGCCGTCCAAGATCCAGTCGGTGGCGCTGCCGCTCATCCTCGCCGATCCGCCTGAGAACCTGATCGGCCAGGCGCAGAGCGGCACCCCACGCGTGAGCTGGTGCGCCAGATCATGGCCGTGGTGAACGCCATGGGCAAGTTGTGGACACGTTCCTGGCCATCCCCGGCAACGACCTGGCCCGCGGTGAGACGCTCAAGGCGCAGGTGGTGGTGGGCACCCCCGGCAAGGTCGAGGGCATGATCAAGAAGAAGCAGCTGGACACCCGCAGCGTCAAGATCTTCGTGCTGGACGAGGCCGACGTCATGGTGGCCGAGGACGGCATGCGCGAGCGCTCGGTGGCGATCAAGCGTCTGATCAAGAACCGCGCGTGCCAGTACCTGCTGTTCTCGGCCACGTACGCTGACGACGTGCGCGACTTCGCCCAGAAAATGGTGCCCGACCACAACATTATCACGGTGAAGAAGGAGAAACTCACCCTCGACGGCATCAAGCAGTTCTGGATCGACTGCAAGACGCGCGAGAACAAGTTCCAGGTGCTGTCGGACATTTTCGCCATCCTGACCATCGGCAAGTGCGTCATCTTCGTGCAGCAGCGTGAGACCGCCAAGGAGCTGACCCGTGCCATGCGTGAGAAGGGCCACTCGGTTGGCATCCTGCACGGTGCCGACATGGCGAAGGAGGTGCGCGACCAGGTCATCGACGAGTTCCGCGCCGGCACGACCAACGTGCTGATTACCACCAACGTGTTGGCGCGTGGTATCGATGTGGCGGGTGTGTCGCTTGTGGTGAACTTTGACATCCCTCTTACGCGAGACCACCGCCCCGACCCGGAGACGTACCTGCACCGTATCGGTCGTACGGGTCGATTCGGCCGCAAGGGTTGCGCTATCAACTTTGTGTACGACAACAAGAGCAAACAGGACCTCGCCGAGATTGAGGAATACTACGCGCGGCCGATCACCCAGGCCCCCGCTGACGACATCGAGGAGCTCGAGAAGCTGCTGGCTTGGGGGGCAGCAAGCGCCGCCTGGCCAAGCTGGGCAAGGAGCAGCGCAAGGGCATGCGCATCTACCCGCGCGAGCCCAAGAAGAAGCCCTCGGGCAAGGACAAGACGTACTACTACGTCAAGGACGTGATGTTCCTGGCGCACGAGCCCGTGCTGGACAAGTTCCGCGAGTTCAAGACCTTCATGAAGAAGGTCAAGAAGGCCATGGGCAAGAAGCACTACGAGGACGCCAAGCGCAAGCACGCCAACCGCCCCACGTACGAGCTCGACCACATCGTCAAGGAGCGCTACCCGCGCTTCGTGGACGCGCTGGGCGACCTGGACGACGCGCTGTGCGGCAGAACTACGTCGTGGCCACGCGCGCGCTCACCAAGGTCTTCGTGAGCATCAAGGGCATCTACTACCAGGCCAACGTGCAGGGCCAGACCGTCACGTGGCTCGTGCCCCACCAGTTCACCCCCACCGTGGACAAGCGCGTGGACGTGCGCGTCATGCTCACCTTCCTCGAGTTCTACGAGGTGCTGCTCAAGTTCGTCAACGCCCAGCTCTACATGCAGATCGGCGTGGCCTACCCGCCCAAGATCAACCTGCAGCTGGACGCGGCGGGCGCGCAGCTCGCGGCGCTCGAGCTAGCAGCAGAAGGACTCCGAGAAGCGTATCAAGTTACAGCGAGCAGCGTAAGGAGCGCAGCGAGAAGAGCCTTTTCAGCGGCCTGACCTTCTTCTTGTCTCGTGAGGTGCCTTCCGCCACCCTGGAGCTGGTGCTGCGCTCGCACGGCGCTACGCTTGGCTGGGACGGCGCTGGCTCGCCTTTCGACGAGAAGAGCACTCGTATCACGCACCACATCATGGATCGCCCGCACCAGGGCCACCGTTACTTCAACCGCGAGTACGTGCAGCCGCAGTGGGTGTTCGACTCGGTCAACAACGGCATTCTGCTGCCGCTGACGCGTTACCGCAAGGAGCTGGAGAAGCTCAAGTCGGCCTCGCAGGTGCTGCGCGAGGTTGGCGCCGTGGGCGACGAAGACGAGGATGGGAGGCCTATGCTGCTGACCTCAAGGCCGAGATGGACGGCAACAAGAAGTCGAAGCGCGCCAAGAAGGAGGAGGAAGCCGCGGAGCTTAAGGAGCTGGCTAAGACCATGATGTCGAAGAAGGCCAAGCGTCTGTACGACCGCATGCAATACGGTCTGGGCAAGAAGGCCGACAAGATTCAGAAGCTCGAGGACAAGCGCGCCAAGGTCGAGGCAAGACCAAGAAGCTGCTGGCAGGCGCGCTGTCCGGCATGATCTCGGCGCTGGCCTTCCAGCCGCTGGGGCGCTGCGCTGGCGCGGCACGTCGCCCACGCTCGTGCGCGTGGCGGGCGGCGCAGGGCTCTACTTCGCCACGCTGGACCACTGCCTCAACCTGTTCCCGCAGTCGGCCGTCAACACTTTCCTGGCCGGAGCCTTCGCGCGCACGTTTGCGGGCGGCGTCATGTCGCCGTTCACGATCGTGAAGGCGAGGATGGATGCCCCCGGGCACATTCGACAGCAAACGGACGTACCGTTCTCCGGTCTCTACGTGCTCATCTACACGGCACCCGGCCGACGTGGTGAAGACGCGCATGCATGCGCCAAACGGTGGCCAAGATCTTCCGCCACGAGGGCATACGCGGATTCGCTAAGGGCATCATCCCGCGTGTGGTCAAGCGCACGCTGTCCACCGCCGTCACATGGACGATATACGAACTACTCGTTCGACCCGTCGGGTCTGGGCCACGCCAAGGAGGCCTTCAACCTGGCCAAGGAGACCGAGCGCACCAAGCAGATGGAGAACCAGGCCAAGATCAAGGAGAACGAGGCGCTCTACAAGCAGTACGAGATCGTACGCGTGCAGAAGGAGGGCGAGGAGCGCCGCAAGACGCTCGAGGAGGAGACGCGCCAGCACCAGCAGCGCGCGCAGTACCAGGACCAGCTCAAGCGCAAGCAGTACGCCGACCAGCTGGCCGCGCAGAAGTACATGAAGGAGCAGGAGCTCAAGAAGCAGGAGGAGATCCTCGCTCGTCAGGAGGCTTCGCGCCGCAAGACACTCGACTACGAGGCCGAGCTGCGCCAGAAGACGGAGCTGGCCAAGGTGGCCGCCGAGACCGAGGGACGCATCAAGCAGGAGCGTCTCAACCACGACCTGCACCTCGAGGAGGCCCGCGTGCGTGCTAAGGAGTACCGCGAGACGGTCATGGAGGGCATCAAGCTGGCGGGTAACACTGTCGGTTCGGGTATCATGGCCTTCGTCGACGACAAGGAGAAGCTTACGGCCACCGTGGCCTCGCTGACGGCCCTGGCTGTCGGTATCTACACCGCCAAGGTCTCCACGAGCGTTGCGGGCAAGTACATTGAGGCGCGCATGGGCAAGCCGTCGTTGGTGCGTGAGACGTCGCGCCGTTCGGCGACGCAGGTGCTGGCCAACCCCATCCCGTCGATCAAGCGTGCGCTGCGTCTGCAGAAGGCGACGGACGCCCTGGAGGGCGTTGTGCTCGAGCCCAAGCTGGACGAGCGTCTGCGCAGCGTTGCTGTGTCCACGTTCAACACGAAGAAGAACCGCGCGCCGTTCCGCCACCTGCTGCTGCACGGCCCGCCCGGTACGGGTAAGACCCTGTTCGCCAAGGCTCTGGCGCGTCACTCCGGCCTAGAGTACGCCATCCTCACGGGTGGTGATGTCGCCCCGCTGGGCCGCGAGGGTGTGACGGAGATCCACAAGCTGTTCGACTGGGCGTCGCACAGCCGTCGTGGTCTGCTGCTGTTCGTCGATGAGGCTGATGCGTTCCTGCAGAAACGTAGCAACACCGTCATGTCCGAGGACATGCGTAACGCATTGAACGCCTTCCTGTACCGCACGGGTGAGGCCAGCGACAAGTTCATGATCGTGTTCGCCAGTAACCAGCCGGAGCAGTTCGACTGGGCCATCAACGATCGTATCGATGAGATGGTGGAGTTCCGCCTGCCGGGCTTCGACGAGCGTGTGCGCATGCTGAAGCAGTACTTCGACGACTACATTCGTGCGCCGAAGAACTCGCGTGCCAAGAAGATTTACGCGAGGGCGTCATCTTCACTCTGGACCCCGTGGCCAACTTCCTCGTGCCAAGACGCGCATCTTCCAGCTCGAGGCGCTGGAGGGCGTCACCGGTGAAGCCCAGGCCATCTTCGACGCGCTCAACAAGAGGCCATGCGCCTCTACTGGGTCGTCTTCGGGCTGGTGCTGGCCAACGGCCTGTACACGTACGTGGCCGGCAAGGACGAGTCGTTCCTGGTGGAGAAGCTCAGGAGAAAGATCCCGTCCGAGTACCGCATCTCCGAGAAGTGGGACAAGTGCCTGGAGAACTTCACGCTGTACTTCGGCGCCGGCCTCGTGGCTGGAGGCCTCACGTCCCTCGTGCTGGCGCGTTCCGGCGCGGGTCGCGGACTAATCACGGGTCTGGGCGCTCCGGCGCCGGCTCGAGCTGGACGACGTGCCAACTGGCGTTCGCCGGCAACCCACAGACCGCGCTGGACAAGACGGACAAGGCTGTGGGCGACTTGCCGGCCTTCCAGCAGCAGACGATCGTGGACTTCGTGACGGCCTCAGCCACGCAGCAGCAGCTGCAGGCCGTGCACCCGCGGCTTCTGCGAGCAGGCCAGCGTGCAGGTCCTGGAGACGTACCTGGCCGAGCAGGTCAAGAACTCGACCGTGGACATCGACGCCAACCTGGCGCTGCTCAAGCTGTACCAGGTGTATCCGGCCACGGCCAACGCTGAGAGCCACGGTGCTCGTCAAGGGCGTCATGGCGCTGCCCTCCACCTTCTTCACTGGCGTCCACCATGGTCCCTGAGAGCGTCCGCGAGGATGCCAACGTGACGGCGGTGCTGCACGCCGGCTTCCTGCTGCAGTCGTGCCTGTTCGAGGACTTCTGGAAGGAGAACGTGGCCTTCGCCGAGAAGGTGCCTGGTTACCTGGAGTCGGTGCGTGCGTACATCCTGATGGCCATCAGCCGCAGCCACAGCGCCATCTCCACGGGCGTCCTGAAGACCAAGCTCAACGTGTCTGACAAGGAGGTGGCCGACATCGTCGCCGAGAAGTGGACGCGGCTGACCTGATCCACATCAACCCCAACGAGGACAACCAGATGCAGGCCAAGAAGGTCCAGGAGAACATCGAGTTCGAGGACGTGCTCAAGGTCATCCACACCCTTTCTAGATCCAACGTCTGCGCCAGCGGCGGCGGCGTGGCCGACGCGGAGGAGGTCTGCGACGGTCTGGCCAAGCACCTGCGGCTGGACGGCGGCGCGCAGGTGCTGCCCTGCTCCACGGGCGTCATCGGCTGGCGCATCCCCGTGGACGCCATGGTCGAGAACCTGCCCAGGCTCGTGGCCAACCTGCAGTCCGACTCGGTGCTGCCGGCTGCCGAGGGTATCATGACCACGGACCTGTACCCGAAGATCCGCTCCATGGACGTGTGCGGCGGCCGCATCGTCGGCATTGCCAAGGGCGCCGGCATGATTGAGCCCAACATGGCCACCATGCTGTCCTACATCCTCACGGACCTGTCGATTCCGCGTGACGTGCTCCGTCAGCTGCTGACGGAGGTGGTGGGCGACACGTACAACTCGCTCAGCGTGGACACGGACCAGAGCACGTCCGACACGGTGACCCTCGTGTCGTCGGACCAGATCCCCTTCGACATGGA---CAAGCTGGACGAGTTTAAGAAGGCGCTGCACGAGGTGTGTCTGGGCCTGAGCGAGGACATCGTGCGCAACGGCGAGGGCGCGCACCACGTCATGCGCGTGCGCGTGCGTGGCGCCGTGACCAAGGAGATGGCCAAGGGTGTGGGTAAGTCGGTGGTGAACTCGCCGCTGCTCAAGTGCGCTGTGGCCGGTAACGACCCGAACGTGGGCCGTCTTGTCATGGCCGTTGGCAAGTACATGGGCCTGCACCACAAGGACGTGGATATCCAGCGCCGCATGACGATCCGCATGGGCGGGGTCGAAATCTTCGAGAACGGCGAGATGGTGCTCAACGACGAGATTGAGGCCAAGCTTGTGGCCCACATGCGCGACGCCATGCTGATCGAGCCCACCCGTGGTGGCCTTGACGCTAGCTCGCACAACTACCCGCCGCACGGGAAGTTTGTCGAGATGGAGATTGACTTGGGTGTTGGTTCCGAGGAGACTCAGGTGCTGGGTATCGACCTTACGCACGAGTACGTGGCCATTAACGCGGACTACCGGTCGTAATGGCGCC---GAAGAAGGGGAAGACCGCCGCCGCGTACCTGGCGTCGCTGGGCGTGGCCCCCGC---CGCCGGCGGAGAGAAGAAGGACGAC---AAGAAGAAGAAGAAGAAGGGCAAGAAGAAGCCCAAAAGAAGCCCATGTCGGCCGCGGCCAAGGCGCTGA-TGGAGCGCCAGGCCGCGCTCAAGGCGGAGCAGGAGCGCCTGGAGCAGGAGCGCCTGGAGCAGGAGCGCATCGCGCAGGAGGAGGAGGCCAAGCGCCTCGCCGAGATCGAGCGCGAGGAGGAGGCCCGCCGCAAGAAGAAGGAGGCGCGCGCCGCCAAGATCGAGCGCCAGAAGAAGGAGGGCACGTACATGACCAAGGCCCAGAAGCAGAAGGCGGCCAAGGCCCAGGCCGCCCTCGAGGCCATGCAGCAGGCGGGCATCGTGCCCACCGCCGGC------GGCGAGAAGAAGAAGGTCGTGTACTCGGACAACAAGAAGAAGAACAAGAAGAACAAGAGGAGGAGCAGGAGGAGGACGAGGCCCCCGACGCCTGGGATGACGAGGACTGGGAGACCAGCGACGTCGTGGCCTCGCTGGAGGACAAGCTAGCAGAAGAAGGAGCAGGAGCGTCTGCGCCAGCAGGGTATCGCGCTCAAGAAGCGCCAGGAGGAGCAGGCCGAGCAGATGCGCCAGGAGGCGGAGGAGGAGCGCCAGCGGTCCAAGGCCAAGATGGAGGCGGAGCGTAAGAAGAAGGAGGCTGCTGAGCGTCGTCGCGAGCGCGAGGCCGCGGCGCGCGCCAACGTGTCCCTGGACAACCTCCGTTCGCCCATTTGCTGTATCATGGGTCACGTCGATACCGGTAAGACCAAGCTGCTTGACAACATCCGTAAAACCAAGGTGCAGGACGCAGAGGCTGGTGGTATTACCCAGCAGATTGGTGCGACCTTCTTCCCCGTGGAGGCCATCAAGCAGAAGACGGCGCGTCTCGCCGAGACGATGAAGCTCGACTACCGCCTGCCCGGTTTGCTTGTGATTGATACGCCTGGTCACGAGTCTTTCACGAACTTGCGTTCGCGTGGTTCGTCGCTGTGTGATATCGCCATTCTGGTGGTTGATATCATGCACGGTCTGGAACCGCAGACGTTGGAGTCGCTGCGCCTGCTGCGTCAGAAGAAGGCCCCGTTCGTGGTGGCGCTGAACAAGATCGATCGTTGCTACGGCTGGAAGACGATGCCCGATATGCCCGTCCAGGAGGCCCTCAAGCACCAGAACGAGCACGTCATTCGCGAGTTCGAGGACCGCATGAAGTCGATTATCGTCGAGTTTGCCGAGCAGAAGCTGAACGCCGAGGTGTACTGGCGCAACAAGGACCTAGCGCACACGGTCTCGCTCATTCCTACCAGTGCTATCTCGGGCGAGGGTGTCCCCGATCTGCTTATGATGTTGACGCGCTTGACGCAGGAGCGCATGGCGAAGTCGCTGGCTTTCGTGGATATTCTGCAGTGTACCGTGCTTGAGGTGAAGGTTATCGAGGGTCTCGGTACCACAATCGATACGATTCTGGTGAATGGTACACTGGAAGAAGGATCGACTATCGTCGTGTGTACACTAGACGGACCTGTTGTGACGACGATCCGTTCGTTGCTGACCCCGCACCCCATGAAGGAAATTCGTGTGAAGGGCGAGTACCTCCACCACCAGAAGATGAAGGCCGCTATGGGTGTCAAGATTTGCGCGCAGGGGCTTGAGAAGGCCGTCGCTGGTACGCAGATTTACGTGGTTGGCCCTGACGACGACGTTGAGGAGCTCAAGGACTCGGTCATGTCCGACCTGACGGGCATCCTGGACTCGGTGAAGGCCACCCGCCGCGGTGTGATGGTGCAAGCTTCGACCCTGGGTGCGCTTGAGGCCCTGCTGGAGTTCTTGCGTACGTGTGACCCGCCCATTCCGGTCTCGTGCGTAAACATCGGACCCGTGCACAAGAAGGATGTTATGCGTGCCTCAGTGCAGCTGGAGCACCAGCCCGAGTTCGCCACCATCCTGGCGTTCGACGTGAAGGTTCACAGCGACGCCACGGAGCTGGCCTCGGAACTCGGTGTGCGCATTTTCACAGCTGACATTATCTACCACCTGTTCGACCAGTTCACGGCGTACATGGACAACTTCCGCCAGGCGCGTCGCGAGGAGTTCGCGGAGGTTGCCGTGTTCCCGTGCGTGCTGAAGATCCTGCCCAACTGCGTGTTCAACAAGAAGGACCCGATCATTCTTGGCGTGGACGTGGAAGAGGGCATCCTGAAGGTGGGCACGCCGCTCGTGGTGCCGTCCGCGGGCG---GCTTCCTGGTGGGCAAGGTGGGCTCCATCGAGCGCGAGCACAAGGAGGTGGACCGCGCCAAGAAGGGCGCGTCCGTGGCCGTGCGGATAGACAACGAGGGCAGCGTCATGTACGGCCGCCACTTCGACCACAAGAACAAGCTCGTGTCGCGCCTGACGCGCCAGAGCATCGACGCGCTGAAGGAGAATTTCAGGGAAGACCTGCAGAAGGAGGACTGGCAGCTCGTCATCAAGCTCAAGAAGGTGTTCGATATCATTATGGAGGGCTGGCTCACCAAGCGCCGCGACCACATGAACCTCATCTGGCTGGATCGCTACTTCCTGCTGGACGGCAACCAGCTCCGCTACTACCGCAAGCGCGGCGACCCGGCGCCTCGAGGCACGTACATCCTGACGGACGCGTGCGTGGTGAGCCCCGTGTTCAACTCGGAGGAGAAGCACAAGCACCACGGGCCCGTCTGGATGTTCCGCATCACGTGAGAAGTCGTACAAGAAGGACCGGTTCCTGGACCTGGGCGCCAAGAGCGAGGAGAACGCCAGGGAGTGGAAGCGCGCGCTCGAGAACGCCGTGCGCTCCATCCGCGAGCTCGTGGCGCACCAGAAGAAGCTCAAGACGGACGCCGAGAT---CTTCCCCACGCCCACGCAC---------GTGCCGCGGCTGCTGAAGACCGACGAGGACACGGTGTCGCTCTTCAATCTGGACGCGGACAACGTCAAGGCGGAGCAGGCCTGGTGGCTGGCCAGGGTGGAGGGAGGCCTCCGGATCATGCAGGAGTGTCCGCTCGGGCCGAACGTCGCGGCCAATCTGTACGCGCAGCACGCAGCCACCACGAACGGACTCATCTTCAGTGCCCTCGTGGGCGCGGCCTTGAGTTTCAGTAGCATGTACTCGTTCGCGAACAATATGCTGCTGAGCTTCCTGACGGCGCTGGCGGCCATTGCCGTGTGCTGCGCTGCGGTGACGCCGACGCAGGCGCCGGAGATCCCGAGCTTCCGGGTCTCGCAGGTCGTGCACGGATCCCCGACGGAGGTCTTCAGGCTCATCATGAACTCCAAGAGATTCCAGAGATGGGACTCGGCCACGGCCACCATGCGCGTGATTCAGCAGCTGGACGACCACGCCGATATCGTCTACGTGACGCAGCGGCCAACGCACTTGTGGCCACTGTGGCAAAAGGCGCGCGACCTCGTGCTCATGCGCTACTGGCGCCGTGAGGAGGACGGATCCTACTTTGTCATGTACCAGAGCATCGAGCACCCAGAGTGCCGTGTGCGTCACAACTACGTCCGTGCTAACCTGCTGGGCGGTGGCTTTGTCATTGCTCCTCAGCGTGTTCCTAGT---GGATCCATTCGTACGCTGGTTACCTACGTGCTCCGATACGACCCGGGTGGCTG---GTCGCGCATTTACCACCAACTCGGTATGGATGTGGATCTGGTCATGCCGATGTTGCGCAGCGTTGTTGGCATCCGCGATGAAATGAGCGCCACGGACTTCGTTACACCTAACGTCTCGGTGGCACCTGCTGGAGAAGGCGGAGGTTGGCCAGGAAGTGTGGA---CTCCATGGGCGTTGTTATGGGCGTGCAGAAGCTTCAA---ACCAGTCTACCGGAGAAGATGTGGGCCGAACCCGATGCGTCGCTCTTTAGCGTGCGTGGCCACAACTACCTGAGCGACAAGAAGAAGATCCCGTCGGCGCCGGCAATGTTCCACACAGTGGGCGTGGATTTACTGTCGTTCGAGAGTGCTGCCGAAAGGTACAACATCTCTTCTCGGCCGGACAGCATTGGACGTACGAG---CTCCAGATTCACATTCGTCGTGAACATGATCATCCCAGGACCGGAGAACACGTGCATGGTATTCTACTTCCAGCCCGTGCGTGACAACGTGTTCGAGGACGGCTCGCCCTTCTCGGAGTTGCTGAACGACTTCTTCGATGGCGACGACCAGTTCCGGAACTCGCGCTTCAAGCTCATTCCCACCGTCGTCGAGGGTAGCTTCATCATCAAGCAGTCGGTCGGCTCCAAGCCCACGCTGCTCGGCAACAAGCTGAAGTGTCCGTACCACCGTGGAGAGAACTACTTCGAGGTGGATATCGACATTTCGTCCAACTCAGTCGCCAATACTGTCGTCGGCATGGTGCAAGGGGTGACCAAGTCGCTAGTGGTGGACATGGCTTTCCTGCTGGAGGCCCAGACCGACGAAGAACTGCCTGAAATTATCCTCGGAGCTGTTCGCATGCAGCACATTTCGCTGGACAACCCCCGCCGGATGCCGAAGCATGGGGAACGGACAGCAAGGACGAGTTCACCTTCTGAAGCCTTCACCGAGGCGCTGCGCGTGAACCTGCAGCGGCTGATCGTCTACGCGCGCTCGGCGGACACGGCGCTGCAGCGAGAGGTGGCCGAGAAGCTGGCCAACGAGGCCGTCAAGACCGACAGGTGCAGATCGTGGAGCTGGATGGCCTGCAGCTGCTGCTGCCGCTCACCAAGTCCAAGGACACGGAGGTGCAGCGGCTGGCCGCCCACGCACTCGCCAACCTCTCGGTCAACTCGGAGAACCAGTCCAAGATGGCGACGGAGGGAGGCATCGACATGCTCATTGACCTCCTGAGCAGCACCAACGAGCACGTGCAGCGGCAGGCGGCGAAGGCCCTCGCCAACCTCGGCGTGAACGTGGACAACAAGGAGCGGATCGCCAAGGCGGGAGGCATTAAACCGCTCATTGACTTGGCCAGCTCGCGCCAGATCGGTGTCGCCGTGGAGGCTATTGCTGCGCTGGCCAATCTGGCTGTCAACG---ATGCCAACGAGGTGGAGATCGCACGCAAAGGCGGCTTGAAGCCAATCATTGACGGCGCGCACTCGGAATCTGTGGAGCTGCAGTCCCAAGTAGCACGTGCACTACGCAACCTATCGGTGAATCCGGAGAACAAGCAAGCGATTGTGGAGCTGGGCGGAGTGGAGGCATTGCAGTCACTAGTGCGCTCCACGAACGATCGGATATGCCAGCAGGCTACTCGTGCGCTCGTCCCCAACGTGGTGCTCACGCGCTCGTCCCCCACAACCTGCTG---GCCGTGGCGCCCGTGGCCCGCGTGGCCGACGTCAAGCAGGAGGC------CGCGCCCTACGA---CGCCATGGTGGACGTCGAGTACTCGGCCACCGTCAAGACGGTC---------GCCGTGCCCGCGGCCGCCGCCTCCGACGCCGCGGCCGGC---GGCTTCTCGTCCATCTCGGACCGCTTCCCGCTCTCCAAGGTGGGCAAGGTGAGCTGTGGCATG---CCCATCCCCATGAGTCG---CCTGCTCAACG---CCACGCCCGAGATGACGC---AGGCGGACCAG-------------ATGACGGTGCTCGTCGAGGACGGCACGGTCGACGACAAC---ACCATCAAGGCCGAG---CCCGAGGGTGAGCGACGTGTACCGTTACCAGGCCACGGA--CGTCGAGGAGAACGAGATGGAGCAACTGCGATGGAACATCCAGGAGCAGGTTTTCCAGAGATACTTCCAGCAGGGACAGAAGTACGAGGAGTGGGGCAAGCTCGAGGAGCAGGACACCGCGTCTTCTTCATCCCAGGCGGACGACGGGAAAACATCTTGGCCGAGAAGCGGTTCCACGACAACGCCGTCCGGGACGCCATGGGGAAGGGGAATGCTACGTGGGTTAAGGAGCGACGAGCACGGTTCGAGGAAGAGATCCGCCTGTTCTATGAAGAAACACGACACAAGCAGGAGCTCATGCTTACGCGGCTCGAAAAGGTGTCTCCGTTCTCCAAGTTCCCGATTCTTGGCAACCGTTTCCTCCTGCTTCGGCTGCGAGGAAAGGGAGGCAACGGGGAGGTGTGGGATGTCGTTGACTACGCCAACTGCAAGCAACTCTGCGCGCTCAAGCTGTCCACGTCGATTCGACACGCCCAACGCGAGCACCAAACACACACTCAACCACCCCA--ATATCGTCCAGGTTGGTGAGGTGCCCTTCCTCATCCGGTACCAACAGCAACAATACACCGCTTTCACCGTTGCAAGCGTCCAATCTGACTTGCAGCAGCTCATTGAGA---TGTACGAGTACCTGGACGATGACTCTGCCTACAAGGTGCTCTCGCAGCTCCTGAGCGCGCTCAAGTACTTGCACGATGGGTGTTGCTCACTACGACTTGAAGCCGTCCAACATTCTGATCGACCACGACGGATGTGTGAAGCTG------ACGGACTTTGACTTGACACGCAACGCCAAATCCGCTACGTCGAACTCGACGGTGGGCACTCTCCGCTACCTCCCTCCTGAGTGCTTCCGCCCAAACCGGGGCGA---------TTGCGAAGCCACGGCTGAGAAGGCGGATATGTGGATGGTTGGAATCGTGTACTGCATGATGGTCACTGGAAAACATCCGATCTGCCCGGACAAGTCCACGCCAGCGGAAGTCAAGTCCACGATGGCTCAGTATACGGGT---GAGCTTCGATACGCACGACCGATTTCCGACCTGTCGCGATGGATCTTACAGGGATGCCTCCACCCGGACCCACGGTGTCGGCCAACAGCCGCTCAGTTGCTGGTGGCAATGCAG

Phytophthora_fragariae TGGACTACCACCGCCAGGTGGCGCGCGAGCTGGCCGCCTTCTTCCACTCCTTCTACCGCTTCGGCGCCCCCTCCAGCCGCTTCGACCTGTTCCTGCACAACGTGCTCTTCGGCTCGCTCTGCATCGGCGCCGTCGTCTTCCTCGTGCTCTGCGTCATCGTCGTGCGGCGCTGCATCTTGCACATCCGCAGGCCTATGTGCGACGCACGTCGATGGACTCCACGTCGGTGGAGCTGCTGGCCGTGGGACTTCTGACCTTCCTGGCCATTAGTGCGCTGGGCGGACTGCTGGGAGAAGCTGTCTGGAGACCAGCAACGCTCTGCGCGTCAATGCAGACAACCTCATCACGTCGTTCAACGACAGTGAGCTACCAGCAGACGCCTTCAGGATGTCGGTGGAGGCGCTGGCCTCGTGCACGCGTCCAAGGAGCTCTGGAACCAGACAGACCTGCTGCTGCCCAAGAACTTCTCGGGGCTCGCCAAGGACTGGGAGTTCAGCTACTTCGTGCTCAAGTCGTCGACCAACGGCGCCATTCTAGCGGTGACTCTGGCCAGCTTCCTGTCCATTGCGTCGGTCGGGTGGGCCATGGTCTCGCCTCTGAGGATCTCGATCATGGTCATCTTGAGCGTCGTGCCCATCTCGCACACTCTGATCGGCGCCTACCTGTCGTCGACGATCCTGACGGCAGACTTCTGTGCTTCCCCGATGAACAACACGCTCGGGCTCGTGGGCTCGTGTCCTGCGAACGCGTCTTGCCTTTTGCTGATGCGGTGGATAAGCAACGCGTCACACCGAGGAGAGATCGGTCAGCGCATGAAGAAGGAGTTCCTGGACCCCATCGGAGCAACTGTGGGCGTCATGCTCGGCTACTTCGCCGGCATGGTCAGCAACTGGGAGAGCTTCTCGCTCGTGCAGACGTGGCGCTGGCCCTTCCTGCTGCAGTTCCTGGCGCTGCTGCCCATCAACGTCGCCATCTTCTTCGTGCCCACCAAGCACCTCGTCATCCGCAGCGAGGCCAGCGAGCTGGAGCCGCCGGTGGCCGGCACGCCCATGAGCACCCGGTCCACGACGTACCACGACGAGGAGGACGCGCTCCTCGCCAGCGCGAGCATGCCACGTACGGCGCCTTGGACACGAGGTCTACGAGCAGGGCGCGTTTTCGGAGGGGGTGTATGAGCTCATGCACATCCCCGTGTTCTGTTTGATTGTGTTTGGGCTCACGACCGTGTACTTCGTAGTCACGGGAGTGCAGTATTGGAGCACCATCTTCATGATCAAGTCGCTGCATGCGTCCAAGTACCTGGTCAACGGACTTTTTGTCGTCGTGTCCGGAACTGGACCCATTCTCGGCGTGTTTTTCGGTGGATGGATCATCGACAGATATGGAGGATACATCGGTGTGGAGCAGCGCGCCAAGGCTTTAGGGATCTGCATGATCCTGGGGCTGACAGCCTTCTCGATTTCGGCAGTGACGACGTTCTTCGATGACGTTTACATCACTGCCGGCTTCCTATGGCTGCTGCTCTTCTTCGGCGGCGCCATCCTGCCGGCGCACCGCGCGCTCGCGTCGTCGTTCTCAGTGATGGTGTTCAACCTGTTCGGGTATGCGCTCTCGCATGAGCGACCTGCAGATGGTGGTGGGCGCCAAGTGCTTCGTGCCGGACGAGACGCACGTGTGGCTCGCGGCCGAGGTGCTGCGCGACGAGAACTCGGGCGACGGCAAGACGCGCAACATCTACTGCACGGTGCAGCTGCCCGACGGAGACGGAGGAGCGATGCGTGGACATGCTGTCCAAGAAGACCAAGGCGCTGCTGCCGTGCACCAGCTCGAGTCGCTGCCCTACCAGAACGAGAACGTGGGGCACGAGGGCATCGAGGACATGATCACGCTCAACTACCTGCACGAGGCCGCCATCCTGTTCAACGTCAAGAAGCGCTTCCTCCGCGAGCTTCCCTACACGTACACGGGCGACATCTGCATCGCCGTGAACCCGTACAAGTGGCTCCCGGACCTCTACGCGGAGGAGCAGCACCTGCGCTACCTCAACCAGCCCAAGGAGGAGCTGCCCCCGCACGTGTACGCCACCTCCGTGGCCGCCTACGACAACATGCGCCGCTCGGAGATCAACCAGTCCATCCTCGTGAGCGGAGAGTCCGGCGCCGGTAAGACGGAGACCACCAAGATCCTTATGAACCACCTGGCCACCATCGCCGGCGGTCTCAACAACTCGACCATCAAGAGGATTATCGAGGTCAACCCGCTGCTCGAGTCCTTTGGTAACGCCAAGACGGTGCGCAACGACAACTCGAGTCGCTTCGGCAAGTTCACGCAGCTGCAGTTCGACCAGAACGGCACGCTCGTCGGCGCCAAGTGCCGCACGTACCTGCTCGAGAAGACGCGTGTGATCCAGCACGAAGCGCCCGAGCGCAACTACCACATCTTCTACCAGTGGACT---CAGACATCGCGCCCGAGCTGCAGCTCGAGTCGGGCAAGCACTACGTGTACACGGGCGACAACACGGCGCAGAAGATCGAGGGACTCTCGGACGCCAAGCACTTCAACAAGACGCGCGAGGCGCTGGAGCTCGTGGGTCTGTCGCCCGATGACCAGCGACCGCTGTTTGAGGTGCTGGCTGGTGTGCTGCACCTTGGTGAGGTGCAACTGCAGTCCGACCCGTCTGATGACGAGAAGTCTCTCATCACTGAGGGCGACATTGGCGCCAGCTCTGCTACCCAGATGCTTGGTGTTACCTGGGAGGCCCTTCAGACGGCTCTGTGTTCGCGCACCATGCGCGCTGTGAACGATGTCTACAGCGTGCCGCTCAAGAAGGAGCTGGCTATGGACTGTCGTGACGCGCTTTCCAAGGCTATTTACTCGAACGTGTTCGACTGGCTCGTGACCACGATTAACCAGTCGCTTGCTGACGATGCGAACATGGCCAACCACGTGGGTGTGCTTGATATCTTCGGTTTCGAGCACTTCAAGCACAACTCGTTCGAGCAGTTCTGTATTAACTACGCCAATGAGAAACTTCAGCAGAAGTTCACGCAGGACGTGTTCAAGACCGTGCAGATTGAGTACGAGGAGGAAGGCATTGTTTGGGACCACATCGAGTACGCGGATAACCAGGATGTGCTGACCGTGATTGAGTCGCGCATGGGTATCATCTCGCTGCTGAATGAAGAAGTGATGCGTCCCAAGGGTAACGAGGAGTCCTTCATGTCCAAGGTTACTTCCCTGCACAAGGAC---GATATGGCGCACGTGATTGAGTTCCCTCGCACATCGCGCACCGAGTTCCTCATCAAGCACTACGCCGCTCCGGTCATGTACGATTCGGTGGGCTTCCTGGAAAAGCACAAGGACTCGCTGCTACCTGATCTGTCCGAGCTCATGCGCGGTTCGTCCAAGCCGTTCATTGCCAAGCTCTTCGATCCGAAGCCGGCGCCTAAGACGTCGGAAGC---------GTGGTGGTGCTTTGTCCATCACGACTGTCGGTACCCAGTTCAAGGAGAGTCTGACAGAGCTGATGGCCACGATCAACTCGACACGCGTGCACTATGTGCGTTGCATTAAGCCCAACCCGATCAA---------GAGTGCGACGGTCATGGACCAGAACATGGTGGTGTCCCAGTTGCGTTGCGCAGGTGTCATTGAGGCCATTCGTATCTCGCGCGCGGCATACCCGAACCGATTGCAGCACACTGAGATCCTGGACAAGTTCTGGCTGTTCGTGCCGAGTGGCGGTGATACTGCAGCGGAAAAGTGCCAGCTGCTTATGGACAAGTTGAAGCTCGAGACGCCGGTGCAGTACCAGATGGGTAAGACTCGTGTATACTTCCAGCTTGGTGTCCTTGAAGAGCTTGAAGACCGCCGCAAGAAGTTCTTGGATGCCAAGGCGACCTACCTGCAGAACATCATGCTTGGTTTCACGCAGCGTATCAAGTACCTCCGACGACTGGAAGCGATCATCAAGTTCGGTGATCCGGTGCGTGATTGCCATGCGGAGATACAACACGTTCATGAACGGTCTTGTTAAAGCTCAAGCTCACTGGCGAGGCATTCAGGGCCGTAAGGTGGCGGCCGATGTCAAGAGCAACCACAACGCTGTCATTATCCAGCGGTATGTGCGTGGTTTTGTCAAGCGTCACCGCTACGTTAAGATGCGCTCCATGGTCATCCGCGTGCAGGCCGTGGTTCGCATGACGATTCAGCGGCCCAAGTACCTGGCCGCTCTCGAGGAGAAGCGCCGCGAGGCTGATATGGCTTACCAGCTGCAGAAGCTCAAGGCAGCTCTGCAAGAGGAACAGGATCGGAATGCACAGCT------GCAGCGCC---GTAGTAGTGTGGT--GATGACGCATCCGTTGCGTCAAGCGAGGTTATGGCTGACGCTGGCGGCATGATCGAGACTCTTACGGGTGAGAACAAGAAGCTGCGTGAGAAGAACGAGGAGATGAAGGTTACGATGAAGGGCTTGAAAGCGGAGATCGAGAAGTTCAAAAGTGACAAGGAGTTCTCGTCGGCTGGCAACCACGTTAAGGTCCGCCAGCTGCAGGACACCGTGCGCGACAAGGACAAGAAGATCTCGCATCTTGAGGCCGAAAACAAGAAGCTTACGGAGCAGATCGCCAAGCTTCACGCGGAGGGTGTTGT---ACCTGAGAAAAAGTCGATCCCGAAGAAGTCGATTTTCCGCACTCTGGGCTCGAAGAAGGAAAAGAAGCCGC---------------GCGAGACCGTGCTGATGGACTCGCTCACGGGCTCAGACGACCTCAACCGCCGGGAGGCTCGTATCTCGGA---GCGCCACTTCCCGCGCATGC---CGAGTATGAGCGTGGGCTCG---CGCTTCTGGAACTCGAACAAG---AAGGGCGAC------------AACGGCGATGGCTTCGACGTCTCGGAGGTTGACGGTGAGGACAGCCCCACGAACCCACGCGCAACACGATCGTGAAGAGCATGGAGGTGGGTGTCACGGGCGCTATGTCGACCCTGAAGGGCCGCATG---------TCGGTCGTGAAGGGCATTTACGAAGCAAGCGCACGAACAGGATCGACCCGTGCGCGAGTCATTCAACCTGGACGCGCTGCCGGAGGTCTCTCTTCCCGTCGGTTGGGAGGCCAAGGTGTCGCGCTCTACGGGCCGCGTGTACTACGTGAACCGCAAGCTGGGCAAGTCCCAGTTTGAGCGCCCCACGCTGGCCTCGCTCAA------GGCGCAGAAACTGGCGCGCCAGAAGTCGGCTAACGCGCTGTAAGGCGGTGGTGGCGGATTTCGGCTGCGGAGACGCCAGGCTGGCCGAGAGCGTGCCGAACAAGGTGCACTCGTTCGATCTGGTGTCCCTCGACATCGGCGTGTATTGCCTGGCACTTATGGGGACGAGTGTGCGCGAGTATGTGCGCGAGGTGTACCGCGTGCTGAAGCCCGGCGGCGTGTTGAAGGTGGCGGAGGTCAAGAGTCGATTTGAGAGCGAGTCGCTGGGCGGATGGTGGCGGTGTTTGTGGCTGCGGCGCGGGGCGTCGTGGTGACGGTGCGCAACACGACGGTGTTCGTGGAGAGACCAAGTACGCTGTCGTTGTGGGCCAGAAGACGGGCGAGGCCGTGGTAGTGCCGTTCCCGGACGTGGACCGCGACCTCAAGACGCTGCTGGGCCACACTACGTCGATGGTCACCCATGTGGCCGTGAACCTCGTCGCTGCTTCTGACGGCCGATCGTGATGAAAAACTGCGCGTCTCGAGGTTCCCGAACGCTGCTATCATTGAGAGCTACTGCCTGGGGCATGCTGCTAGTCTGACCAAGGTGGCGTGCAGTGCTGTCACGCCGGAGCTTGTGGTGTCCACGTCCATGGACAACACACTCAAGTTGTGGGAGATGAAGACGGGGAAACTGGCGATTTGCCCCACGACGACATTGTGGCGGTGCTCGTGAACTACCAGCACGTGCGGTTCTTCGAGATCGTGAGAACCCGTTCCTGCAGTTGTTTTCGATTTCAAAGGGCCGCTGCCGTGTTCAACTGCGGTGCGCGACTTCCGCAAGCTGCAGAACGACCCGCCGTCGGGCGTGTCGGGCGCCCCCATGGACAACAACATCATGCTCTGGCAGGCCGTCATCTTCGGACCGGATGACACGCCCTGGGAAGGCGGCACCTTCAACCTGACGCTCGAGTTCAGCGAGGACTACCCGAACAAGGCGCCGACTGTCAAGTTCATCACGAAAATGTTCCACCCCAACATCTACAACGACGGCCAGATCTGCCTGGACATCCTGCAGAACCAGTGGTCGCCGATCTACGACATCTCGGCCATCCTCACGTCCATCCAGTCGCTGCTGTGCGATCCGAACCCGAACTCGCCGGCCAACTCGGAGGCCGCGCGCCTCTTCCAGGAGAACCGGCGGGAGTACAACCGCCGCGTGCGTGAGATCGTCGAGCAGAGCTGGCAGGCGATCGATGGTGTCGGCCGCCAAGATGCGCGGCGCCGAGAACCGCATGAACGCCGGCCGCCCCTTCACGGCGTGGATGGACAACGTCTCGTCCGACCGCGGCCTCTGCGGCGGCATCAACTCGGGCATCGCCAAGACTGCGCGCAAGCAGACCACTCGCAGATCTTCGTGATCGGCGACAAGGCCCGCGCCCAGCTGCGTCGTGACCTCGGAGACAACATCCGCGGCAACGTGACGGAGACGTACCAGTCGCCCCCCAACTTCACGGTGGCCAGCGCCATCGCGGAGGCCGTCGACTCGGAGAGGAGTCGTCGCGTATGGCCGCCATGGAGAACGCCACCACGAACGCCGAGGACCTCACGCTCACGCTCGTGTACAACAAGGCTCGCCAGGCCCGCATTACCACGGAGCTCATTGAAATTATCTCGGGTGCCGCCTCGCTCGACGGTGACTGGCAGCGTGGTGACCATCCAGAGCGCGGCGCAGTTCGACGTGGCCACGGCGCGCGAGTCGACGCTGTCCGTGGCCTTCTTCTGGGCCGAGTTCCAGGCCTGCCGCCCCAACGGGCAGCTGGACGTGGGGCCGCGCCGTGCTGGACAAGCTCGAGGGCGCCAACGTGGCCGAGCTGGCCAAGCGCGTCTGCAGTACCGCCTCAAGAAGCTCATCAGCGCGTCCCCCGTCATGCTCTTCATGAAGGGCAACCCGACGGAGCCCAAGTGCGGCTTCAGTCGGCAGCTCGTGGCGCTGCTGAACGACGACAAGATCCAGTTCGGCACCTTCGACATCCTGAACGACGACGAGGTGCGGCAGGGGCTCAAGCAGTTCTCCGACTGGCCCACGTACCCGCAGCTCTACGTGAACGGCGCGCTGGTCGGAGGGCTGGACATCGTCAAGGAGATGAAGGCGGACGGCTCGATCGTCGAGCAGCTCGGCGTCGAGGAGGCCGAGGCCGCGTTCCAGGAGAGTCTGCGCGCGCTCGTGCTGTCGGCGCCTGTGCTGCTCTTCATGAAGGTTCGACATCTTGTGTGGAGTTACCAACGCCAGGACCAAGATATCGCCAAGGTGGTGACGCTGATGCCATCGTCTTTCCGCCGGGAAATGTGCCGAGGCCATTTCGTGGAGTCCGAAACCGCATGGCCATGGCATTTCGCGATCCCGTCGACGGAAAGGGTGAATTTTGCATTGTGCAGCTTCTCGACTAACCTCAAAGCGCACTACCCTGCATCAACGAGGTATTTCTGCGATCGACTGGCAAGTGGAACGTGCTGCACCGCGACCACCGCAGTGAAGTTCGATCGCTCTGTGTGCACTCGAAGCGCGACTGCTTGTACACGGGTGGTCTGGATGGCCTATTGATCCGATATGATCTCAACAAAGGGCGCACCCAGAACGTGATCGAACGCCGCAAGCACATCTCCAAGATCAACGCGGTGCTGGAGCACCCGCACAACCCGAACCTGCTTCTGGTCAGTTGCGTGGAGCAGTAATCACAACTTGTTGCTGCATGATCTCCAACGCTACGACCCGAAAGTGTCGGGTGGTGCCATGAGTCAGTACGTTGTGCCGAGGTGGTCGCCTGCAGGCTATCACGTGTCGTGCGGCAGCAAATGCGCTACTGCCACGGCAGCTTCTCCACGCCCACGTCGGCCACTATCGGGGCCTCGTTCCTGCAGAAGCGCGTGATTGTGGGCGGGAAACTGACGCTGCAGATCTGGGACACGGCCGGCCAGGAGCGTTTCCGCTCCATGGCGCCCATGTACTACCGCAACGCCAAGGCGGCCATCCTGGTCTTCGACCTGCAGAGCGAGGCCAGCTTCGAGAAGATCAAGGAGTGGCTGCACCTGCAGCACCACGTGGGTGACGATATGTAGGCAACAAGAGCGACGTTTCGACTTCTCCAAGGCCCAGCAGTTCGCAGACGAGATCGGCGCGCTCGTGTTCCGCACCAGCGCCAAGACGGGCGAAGGGGTACAGGCACTGTTCGAGAGTCTCGCGGTGCAGCTGCTCAAGAAGCATGACAAGGAGCAGCAGAGAGCTTCTTCGTGGTGTACGTGCCCGCCGATGAGCGCAAGGAGCTGGAGGAGTGGGCGGTGGCGCTGCCCAAGGACAAGGAGGCGCAGCTCGGCTGTCTGACGGAGCGACTGCGCGCGCACTTCAAGGCGGGGGCCATCCAAGCAGCAGCAGAGACGTTCAAGCAGCAACTGCTGGCTCAGCTGCCCAAGGGCGCCACTATGACGGGCGAGATGTTGCAGATGATGCATGGACAGCCTCGTGGACTCGATCCCGCTCGTGCTCAACACGCCCGAAGCCAAGCACGTTGGAGGTCAAGGGCGATGCATTCATCGGCCGGGTGTTTGATAGACGACTTTGTGCGTATGGATTTCCGGTTGAGTGAGGTGTCGGGTGGCGCTGCGTGGGTGCAACAGGGCAAGCAGCAGAGGTCACGCAGTCGCTGGAGGAGATCCTGGCGCTGGGCCAGGGCCTGGACGACCAGGACTACAAGGCCGAGCTCGAGGTGCTGCAGAACGACCCCAACTCGAACCTCTACTCGGCCGTCACCTTCGAGGAGCTCAACCTACCGGAGCTCCTGCTCAAGGGCGTGTACAACATGAAGTTCGCCAAGCCGTCCAAGATCCAGTCGGTGGCGCTGCCGCTCATCCTCGCCGACCCACCAGAGAACCTGATCGGCCAGGCGCAGAGCGGCACCCCACACGCGAGCTGGTGAGGCAGATTATGGCTGTGGTGAACGCCATGGGCAAGTTGTGGACACGTTCCTGGCCATCCCCGGTAACGACCTGGCACGTGGCGAGACTCTGAAGGCTCACGTGGTCGTAGGCACCCCCGGCAAGGTCGAGGGCATGATCAAGAAGAAGCAGCTGGACACCCGCAACGTCAAGATCTTCGTGCTGGATGAGGCCGATGTCATGGTTGCCGAGGACGGCATGCGCGAGCGCTCCGTGGCGATTAAGCGTCTGATTAAGAACCGTGCGTGTCAGTACCTGCTGTTCTCGGCCACGTACGCTGACGACGTGCGCGACTTCGCCCAGAAGATGGTGCCCGACCACAACATTATCACGGTCAAGAAGGAGAAGCTGACGCTGGACGGCATCAAGCAGTTCTGGATCGACTGCAAGTCTCGCGAGAACAAGTTCCAGGTGCTGTCGGACATTTTCGCCATCCTGACCATCGGCAAGTGCGTCATTTTCGTACAGCAGCGTGACACCGCCAAGGAGTTGACGCGTGCGATGCGTGAGAAGGGCCACTCAGTTGGCATCCTGCACGGTGCCGACATGGCGAAGGAGGTGCGCGACCAGGTCATCGATGAATTCCGCGCTGGCACGACCAATGTGCTGATTACCACAAACGTGCTGGCGCGTGGCATTGATGTGGCGGGTGTGTCTCTTGTGGTGAACTTTGACATCCCTCTGACGCGAGACCACCGTCCCGACCCGGAGACGTACCTGCACCGTATCGGTCGTACGGGTCGTTTCGGCCGCAAGGGCTGCGCTATTAACTTCGTCTACGACAACAAGAGCAAGCAGGACCTGGCGGAGATTGAGGAGTACTACGCGCGTCGTATTACCCAGGCTCCTGCTGACGACATTGAGGAACTCGAGAAGCTGCTGGCTTGGGGGGCAGCAAGCGCCGCCTGGCCAAGTTCGGCAAGGAGCAGCGCAAGGGCATGCGTATCTACCCGCGCGAGCCCAAGAAGAAGCCCTCGGGCAAGGACAAGACGTACTACTACCTCAAGGACGTCATGTTCCTGGCGCACGAGCCCGTGCTCGAGAAGTTCCGCGAGTTCAAGACGTTCATGAAGAAAGTCAAGAAGGCCATGGGCAAGAAGGACTACGAGGACGCCAAGCGCAAGCACGAGCACCGACCCACGTACGAGCTCGACCACATCGTCAAGGAGCGCTACCCGCGCTTCGTGGACGCGCTGGGCGACCTGGACGACGCACTGTGTGGCAGAACTACGTCGTGGCCACGCGCGCGCTCACCAAGGTCTTCGTGAGCATCAAGGGCATCTACTACCAGGCCAACGTGCAGGGACAGACCATCACGTGGCTCGTGCCCCACCAGTTCACCCCCACCGTGGACAAGCGCGTGGACGTGCGCGTCATGCTCACGTTCCTCGAGTTCTACGACGTGCTGCTTAAGTTCGTCAACGCCCAGCTCTACATGCAGATCGGCGTGGCCTACCCGCCCAAGATCAACCTGCAGCTGGACGCGGCCGGTGTGCAGCTCGCGGCGCTGGAGCTAGCAGCAGAAGGACTCCGAGAAGCGCATCAAGCTACAGCGAGCAGCGCAAGGAGCGCAGCGAGAAGAGTCTGTTCAGCGGTCTCACCTTCTTCCTGTCGCGCGAGGTGCCTTCGGCCTGCCTCGAGCTGGTGCTGCGCTCGCACGGCGCAACTCTCGGCTGGGACGGTGCTGGGTCGCCTTTTGACGAGAAGAGCTCGCGCATTACGCACCACATCATGGATCGTCCGCACCAGGGCCACCGCTACTTTAACCGCGAGTACGTGCAGCCGCAGTGGGTGTTCGACTCGGTCAACAACGGCGTTCTGCTGCCTCTGACTCGGTACCGCAAGGAGCTCGAGAAGCTCAAATCGGCCTCGCAGGTGCTTCGCGAGGTCGGCGCCGTGGGCGACGAAGACGAGGACGGGAGGCTTACGCCACTGACCTTAAGGCTGAGATGGACGGCAACAAGAAGTCGAAGCGGGTCAAGAAGGAGGAGGAGGCTGCGGAGCTCAAGGAGCTGGCCAAGACCATGATGTCGAAGAAGTCCAAGCGCCTGTACGACCGTATGCAATACGGCCTGGGCAAGATGGCCGACAAGATTCAGAAGCTCGAGGACAAGCGCGCTAAGCTGGAGGCAAGACCAAGAAGCTGTTAGCTGGCGCGCTGTCTGGCATGATCTCGGCGCTGGCCTTCCAGCCGCTGGGGCGGTTCGCTGGCGCGGCACGTCGCCCACGCTCGTGCGCGTGGCGGGTGGCGCCGGCCTCTACTTCGCCACGCTGGACCACTGCCTCAACCTGTTCCCGCAGTCGGCGGCGAACACTTTCCTGGCCGGAGCCTTCGCGCGCACGTTCGCAGGCGGCGTAATGTCGCCATTCACGATCGTGAAGGCGAGGATGGATCCCCCCGGGCACCTTCGACAGCAAACGGACGTGCCGTTCTCCGGCCTCTACGTGCTCATCTACACGGCACCCGGCCGACGTGGTAAAAACGCGCATGCATGCGGCAAACAGTGGCCAAGATCTACCGCCACGAAGGCCTGCGCGGATTCGCCAAGGGGATCGTTCCGCGTGTAGTCAAGCGCACGCTGTCTACTGCAGTCACGTGGACGATATACGAGCTACTCGTTCGACCCGTCGGGTCTGGGCCACGCCAAGGAGGCCTTCAACCTGGCCAAGGAGACGGAACGCACCAAGCAGACGGAGAACCAGGCCAAGATCAAGGAGAACGAGGCGCTCTACAAGCAGTACGAGATCGTGCGCGTGCAGAAGGAGGGCGAGGAGCGCCGCAAGACGCTCGAGGAGGAGACGCGCCAGCACCAGCAGCGCGCGCAGTACCAGGACCAGCTCAAGCGCAAGCAGTACGCGGACCAGCTGGCCGCCCAGAAGTACATGAAGGAGCAGGAGCTCAAGAAGCAGGAGGAGATCCTCGCTCGCCAGGAGGCCTCGCGCCGCAAGACCCTCGACTACGAGGCCGAGCTGCGCCAGAAGACGGAGCTGGCCAAGGTGTCCGCGGAGACCGAAGGCCGCATCAAGCAGGAGCGTCTCAACCACGACCTGCACCTCGAGGAGGCCCGCGTGCGTGCGAAAGAGTACCGCGAGACGGTCATGGAGGGCATCAAGCTGGCGGGAAACACTGTCGGCTCGGGGATTATGACCTTCGTCGACGACAAGGAGAAGCTTACGGCCACCGTGGCCTCGCTCACGGCCCTGGCTGTTGGCATTTACACTGCCAAGGTCTCCACTAGCGTTGCGGGCAAGTACATTGAGGCGCGCATGGGCAAACCGTCGTTGGTGCGCGAAACGTCGCGTCGCTCGGCTACCCAGGTGGTGGCCAACCCCATCCCGTCGATCAAGCGTGCGCTGCGTCTGCAGAAGGCGACGGACGCCCTGGAGGGCGTTGTGCTCGAGCCCAAGCTGGACGAGCGTCTGCGCAGCGTGGCCGTGTCCACGTTCAACACGAAGAAGAACCGCGCGCCGTTCCGCCACTTGCTGCTGCACGGCCCGCCCGGTACGGGTAAGACCCTGTTCGCCAAGGCTCTGGCGCGTCACTCCGGCCTGGAGTACGCCATTCTCACGGGTGGTGATGTCGCCCCACTGGGCCGAGAGGGTGTGACGGAGATCCACAAGCTGTTTGACTGGGCGTCGCACAGCCGTCGCGGTCTGCTGCTGTTCGTCGATGAAGCCGATGCGTTCCTCCAGAAGCGTAGCAACACCGTCATGTCCGAGGACATGCGTAACGCGCTGAATGCTTTCCTGTACCGCACAGGTGAGGCCAGCGACAAGTTCATGATCGTGTTCGCCAGTAACCAGCCGGAGCAGTTCGACTGGGCCATCAACGATCGTATCGATGAGATGGTGGAGTTCCGCTTGCCGGGCTTCGACGAGCGTGTGCGCATGCTGAAGCAGTACTTCGACGACTACATTCGTGCGCCGAAGAACTCGCGCGCCAAGAAGATCTACGCGAGGGCGTCATCTTCACGCTGGACCCTGTGGCCAACTTCCTCGTGCCAGGACGCGCATCTTCCAGCTTGAGGCTCTGGAGGGTGTAACGGGTGAAGCTCAGGCCATCTTCGATGCACTCAATAAGAGGCCATGCGCGTGTACTGGCTCGTCTTCGCGCTGGTGGTGGCCAACGGCCTGTTCACATACGCAACCGGCAAGGACGAGTCGTTCCTGGTGGAGAAGCTCAAGAGAAAGATCCCGTCCGAGTACCGCATCTCCGAGAAGTGGGACAAGTGCCTGGAGAACTTTACGCTGTACTTCGGAGCTGGCCTCGTGGCTGGCGGCCTCACGTCCCTCGTGCTGGCGCGTTCTGGCGCGGGTCGCGGGCTGATCACGGGTCTGGGTGCGCCGGTGCCGGCTCGAGCTGGACGACGTGCCAGATGGCATTCGCAGGCGACGCACAGGCCGCGCTGGACAAGACGGACAAGGCTGTGGGCGACTTGCCCGCCTTCCTGCAGCAGACGATCGTGGACTTCGTGTCGGCCTCGGCCACGCAGCAGCAGCTGCAGGCTGTCCACCCGCGGCTTCTGCGAGCAAGCCAGCGTGAAGGTCCTGGAGACGTACCTGGCCGAGCAGGTCAAGAACTCGACCGTGGACATCGACGCCAACCTGGCGCTGCTCAAGCTGTACCAGGTGTACCCGGCCACGGCCAACGCCGAGAGCCACGGTGCTGGTCAAGGGCGTCATGGCGCTGCCCTCCACGTTCTTCACGGGCGTCCACCATGGTCCCGGAGAACATCCGCGAAGATGTCAATGTGACGGCGGTGCTGCACGCGGGCTTCCTGCTGCAGTCGTGCCTGTTTGAGGACTTCTGGAAGGAGAGCGTGGCTTTCGCCGAGAAGGTGCCGGGCTACCTGGAGTCGGGGCGCGCGTACATCCTGACGGCCATCAGCCGCAGCCACAGCGCCATCTCCACGGACGTCCTTAAGGCCAAGCTCAACGTCTCCGACAAGGAGGTGGCTGACATCGTCGCCGAGAAGTGGACGCGGCCAACCTGATCCAGATCAACCCCAACGAGGACAACCAGATGCAGGCCAAGAAGGTCCAGGAGAACATCGAGTTCGAGGACGTGCTCAAGGTCATCCACACCCTTTCTAGATCCAACGTCTGCGCCAGCGGCGGCGGCGTGGCCGACGCAGAGGAGGTCTGCGAGAGCCTGGCCAAGCACCTGCGGCTGGAGGGCGGCGCGCAGGTGCTGCCCTGCTCCACGGGCGTCATCGGCTGGCGCATCCCCGTGGACGCCATGGTCGAGAACCTGCCCAAGCTCGTGAGCAACCTGCAGTCCGACTCGGTGCTGCCTGCTGCTGAAGGCATCATGACCACGGACCTGTACCCCAAGATCCGCTCCATGGACGTGTGCGGCGGCCGCATCGTGGGCATCGCCAAGGGCGCCGGCATGATCGAGCCCAACATGGCTACAATGCTGTCGTACATCCTGACAGACCTGTCGATTCCGCGTGACGTGTTGCGTCAGCTGCTGACGGAGGTGGTGGGCGACACGTACAACGCGCTCAGTGTGGACACGGACCAGAGCACGTCCGACACGGTGACGCTCGTGTCGTCCGACCAGATCCCGTTCGACATGGA---CAAGCTCGACGAGTTCAAGAAGGCGCTGCACGAGGTCTGTCTCGGACTGAGCGAGGACATTGTGCGCAACGGAGAGGGCGCGCACCACGTCATGCGCGTGCGCGTGCGTGGCGCCGTGACCAAGGAGATGGCCAAGGGGGTGGGCAAGTCGGTGGTCAACTCGCCGCTGCTCAAGTGCGCCGTGGCCGGCAACGACCCGAACGTGGGTCGCCTTGTCATGGCCGTCGGCAAGTACATGGGTCTGCACCACAAGGACGTGGACATCCAGCGCCGCATGAAGATCCGCATGGGTGGCGTCGTCATCTTCGAGAACGGGGAGATGGTGCTCAACGACGAGATCGAGGCCAAGCTGGTGGCGCACATGCGCGACGCGATGCTGATCGAGCCCACGCGCGGTGGCCTCGACGCCAGCTCGCACAACTTCCCGCCCCACGGGAAGTTCGTCGAGATGGAGATTGACTTGGGCGTTGGTTCCGAGGAGACTCAGGTGCTGGGCATCGACCTCACGCACGAGTATGTGGCCATTAACGCGGACTACCGCTCGTAATGGCGCC---GAAAAAGGGGAAGACCGCCGCCGCGTACCTGGCGTCTCTGGGCGTGGCCCCCGC---CGCTGGAGGCGAGAAGAAGGACGAC---AAGAAGAAGAAGAAAAAGGGCAAGAAGAAACCCAAAAGAAGCCCATGTCGGCGGCGGCCAAGGCGCTGA-TGGAGCGCCAGGCGGCCCTCAAGGCGGAGCAGGAGCGCCTCGAGCAGGAGCGACTGGAGCAGGAGCGCATCGCCCAGGAGGAGGAGGCCAAGCGCCTCGCGGAGATCGAGCGCGAGGAGGAGGCCCGCCGCAAGAAGAAGGAGGCGCGCGCCGCTAAGATCGAGCGCCAGAAGAAGGAGGGCACGTACATGACCAAGGCCCAGAAGCAGAAGGCGGCCAAGGCCCAGGCGGCCCTTGAGGCCATGCAGCAGGCCGGAATCGTGCCCACCACTGCC------GGCGAGAAGAAGAAGGTCGTGTACACGGACAACAAGAAGAAGAACAAGAAGAATAAGAAGAAGAGGCCGAGGAGGACGAGGCGCCCGACGCATGGGACGACGAGGACTGGGAGACCAGCGACGTCGTGGCCTCGCTGGAGGACAAGCTAGCAGAAGAAGGAGCAGGAGCGTCTGCGTCAGCAGGGTATTGCGCTGAAGAAGCGCCAGGAAGAGCAGGCTGAGCAGATGCGTCTGGACGATGAGGAGGAACGCCAGCGCTCCAAGGCTAAGCAGGAGGCGGATCGCAAGAAGAAGGAGGCAGCTGAACGCCGTCGTGAGCGTGAGGCCGCCGCGCGCGCCAACGTGTCTCTGGACAACCTCCGCTCGCCTATTTGCTGTATCATGGGCCACGTCGATACCGGTAAGACCAAGCTGCTTGACAACATCCGAAAGACCAAGGTGCAGGACGCGGAGGCTGGTGGTATTACCCAGCAGATTGGTGCGACCTTCTTCCCCGTGGAAGCCATCAAGCAGAAAACGGCGCGCCTCGCCGAGACGATGAAGCTGGAGTACCGCCTGCCCGGTTTGCTTGTGATTGATACGCCCGGTCACGAATCTTTCACGAACTTGCGTACGCGTGGTTCGTCGCTGTGTGATATCGCCATTCTTGTGGTTGATATCATGCACGGTCTGGAACCGCAGACGTTGGAGTCGCTGCGTCTGCTGCGCCAGAAGAAGGCTCCATTTGTTGTGGCGCTGAACAAGATCGATCGCTGCTACGGCTGGAAGACGATGCCGGATATGCCCGTTCAGGAGGCGCTCAAGCACCAGAACGAACACGTCATTCGCGAGTTTGAGGACCGCATGAAGTCAATTATCGTTGAGTTTGCCGAGCAGAAGCTGAACGCTGAGGTGTACTGGCGCAACAAGGACCTGGCGCGCACAGTCTCGCTCATTCCCACTAGTGCCATCTCGGGCGAGGGTGTGCCAGATCTGCTTATGATGCTGACGCGTTTGACGCAGGAGCGCATGGCGAAGTCGCTGGCTTTCGTGGATATTCTGCAGTGTACCGTGCTTGAAGTGAAGGTTATCGAGGGTCTCGGTACTACAATTGATACGATTCTGGTGAATGGTACTCTGGAGGAAGGCGCGACTATTGTCGTGTGTACATTGGACGGACCTGTTGTGACGACCATCCGTTCGTTGCTGACCCCGCACCCCATGAAGGAAATCCGTGTGAAGGGCGAGTACATCCACCACCAGAAGATGAAGGCTGCAATGGGTGTTAAGATTTGCGCGCAGGGTCTTGAAAAGGCGGTTGCAGGTACGCAGATTTACGTGGTTGGCCCCGATGACGATGTCGAGGAGCTTAAGGACTCGGTCATGTCCGACCTGACGGGCATCCTGGACTCGGTGAAGGCCACCCGCCGCGGTGTGATGGTGCAGGCTTCGACGCTTGGTGCCCTGGAGGCCCTTCTGGAGTTCTTGCGTTCGTGTGACCCGCCCATTCCGGTCTCGTGCGTGAACATCGGACCCGTGCACAAGAAGGATGTTATGCGTGCCTCGGTACAGCTGGAACACCAGCCCGAGTTCGGCACCATTCTGGCATTCGACGTGAAGGTTCACAGTGATGCTACGGAGCTGGCCACCGAGCTCGGCGTGCGCATTTTCACGGCTGACATCATCTACCACCTGTTCGATCAATTCACGGCCTACATGGACAACTTCCGTCAGGCACGCCGTGAGGAGTTCGCAGAGGTCGCCGTGTTCCCGTGCGTATTGAAGATCCTGCCAAACTGCGTATTCAACAAAAAGGATCCGATCATTCTCGGCGTGGATGTCGAAGAGGGCATCCTGAAGGTGGGTACGCCGCTCGTTGTGCCGTCCGCAGGTG---GCTTCCTGGTGGGCAAGGTCGGCTCTATTGAGCGCGAGCACAAGGAGGTGGACCGCGCCAAAAAGGGTGCGTCAGTGGCTGTGCGGATAGACAACGAGAGCAGCGTCATGTACGGCCGCCACTTCGACCACAACAACAAGCTCGTGTCGCGCCTGACGCGTGAGAGCATCGACGCGCTGAAGGAGAATTTCAGGGAAGACCTGCAGAAGGAGGACTGGCAGCTCGTCATCAAGCTCAAGAAGGTGTTCGATATCATTATGGAGGGCTGGCTCACCAAGCGCCGCGACCACATGAACCTCATCTGGCTGGATCGCTACTTCCTGCTGGACGGCAACCAGCTCCGCTACTACCGCAAGCGCGGAGACCCGGCGCCTCGAGGCACGTACATCCTCACGGACGCGTGCGTGGTGAGCCCCGTGTTCAACTCGGAGGAGAAGCACAAGCACCACGGGCCCGTGTGGATGTTCCGCATCACGTGAGAAGTCGTACAAGAAGGACCGGTTCCTGGACCTGGGGGCCAAGAGCGAGGAGAACGCCAGAGAGTGGAAGCGCGCGCTGGAGAACGCCGTGCGCTCCATCCGCGAGCTCGTGGCCCACCAGAAGAAGCTCAAGACGGACGCCGAGAT---CTTCCCCACGCCCACGCAC---------GTGCCGCGGCTGCTCAAGACGGACGAGGACACGGTGTCGCTCTTCAATCTGGACGCGGACAACGTCAAGGCGGAGCAGGCCTGGTGGCTGGCCAGGGTGGAGGGCGGCTTGAGGATCATGCAGGAGTGTCCACTTGGTCCGAACGTGGCGGCCAACCTGTACGCGCAGCACGCGGCCACCACGAACGGACTCATCTTCAGCGCCTTTGTGGGCGCGGCCTTGAGCTTCAGCAGCATGTACTCGTTCGCGAACAACATGCTGCTGAGCTTCATGACGGCGCTGGTGGCGATCGCCGTGTGTTGTGCAGCAGTGACGCCTACGCAGACGCCCGAGATCCCGAGCTTCCGGGTCTCGCAGGTGGTGCACGGATCCCCGACGGAGGTGTTTAGGCTCATCATGAACTCCAAGAGATTCCAGAGATGGGACTCGGCCACGGCCACCATGCGCGTGATTCAGCAGCTGGACGACCACGCCGATATCGTCTACGTGACCCAACGTCCAACACACTTGTGGCCGCTGTGGCAGAAGGCTCGGGACCTCGTGCTCATGCGCTACTGGCGCCGGGAAGAAGACGGGTCCTATTTTGTCATGTACCAGAGCATCGAGCACCCGGAGTGCCGTGTGCGTCACAACTACGTCCGCGCGAACCTGCTGGGTGGCGGCTTCGTTATCGCCCCTCAGCGTGTTCCTAGT---GGGTCCATTCGCACGCTGGTCACGTACGTGCTCCGATATGATCCGGGAGGCTG---GTCGCGCATCTACCACCAGCTCGGCATGGACGTGGATCTCGTTATGCCCATGCTGCGCAGCGTTGTGGGCATCCGCGACGAGATGAGCGCCACGGACTTCATCACACCCAACGTCTCGGTGGCACCTGCCGGAGAGGGCGGAGGCTGGCCAAGAAGCGTGGA---CTCCATGGGGGTTGTCATGGGCGTGCAGAAGCTTCAA---ACCAGTTTGCCGGAGAAGATGTGGGCCGAGCCGGATGCCTCGCTCTTCAGCGTGCGCGGTCACAACTACCTCAGCGACAAGAAGAAGATCCCGTCCGCGCCGGCAATGTTCCATACCGTGGGCGTGGATCTGCTGTCGTTCGAGAGTGCTGCCGAGAGATACAACATTTCGTCTCGACCGGACAGCATCGGACGCACCAG---CGCCAAGTTCACGTTCGTTGTGAACATGATTATCCCAGGACCGGAGAACACGTGCATGGTGTTCTACTTCCATCCCGTGCGTGACAACGTGTTCGAAGACGGCTCGCCCTTCTCCGAGCTGCTGAACGACTTCTTCGATGGCGACGACCAGTTCCGGAACTCGCGCTTCAAGCTCATTCCTACTGTCGTCGAGGGAAGCTTCATCATCAAGCAGTCGGTAGGCTCCAAGCCTACGCTGCTCGGCAACAAGCTCAAGTGTCCGTACCACCGCGGAGAAAACTACTTCGAGGTGGACATCGACATTTCGTCCAACTCAGTTGCCAACACTGTCGTTGGGATGGTGCAAGGCGTGATCAAGTCACTGGTGGTGGACATGGCCTTCCTGCTCGAGGCCCAGACCGACGAAGAGCTGCCTGAAATTATCCTCGGAGCAGTCCGCATGCAGCACATTTCGCTGGACAATCCCCGCCGAATGCCGAGGCATGGGGAACGGGCAGCAAGGACGAGTTCACCTTCTGAGGCCTTCACCGAAGCTCTGCGCGTCAACCTGCAGCGGCTGATCGTCTACGCGCGCTCGGCGGACACGGCGCTGCAGCGAGAAGTGGCCGAGAAGTTGGCCAACGAGGCCGTCAAGATCGACAGGTGCAGATCGTGGAGTTGGACGGCCTGCAGTTGCTGCTGCCGCTCACCAAGTCCAAGGACACGGAGGTGCAGCGTCTGGCAGCGCACGCACTCGCCAACCTCTCAGTCAACTCGGAGAACCAGTCCAAGATGGCGACGGAGGGAGGCATCGACATGCTCATTGACCTCCTGAGCAGCACGAACGAACACGTGCAGCGGCAGGCGGCGAAGGCGCTCGCCAACCTCGGCGTGAACGTGGATAACAAGGAGCGGATCGCCAAGGCGGGTGGCATTAAACCGCTCATTGACTTGGCCAGCTCCCGCCAGATCGGCGTCGCTGTGGAGGCCATCGCAGCGCTGGCAAATCTGGCTGTCAACG---ATGCAAACGAGGTGGAGATTGCACGCAAAGGCGGCTTGAAGCCTATTATTGACGGCGCGCACTCGGAATCCGTGGAGCTGCAGTCCCAAGTAGCGCGTGCACTGCGCAACCTATCGGTTAATCCGGAAAACAAGCAAGCGATCGTGGAGCTGGGCGGAGTGGAAGCTTTGCAGTCACTCGTGCGCTCCACCAACGACCGGATATGCCAGCAGGCTACGCGTGCGCTCGTCCCCTGCGTCGTGCTCACGCGCTCGTCCCCCACAATCTGCTG---GCCGTGGTGCCTGTGGCCCG---------CGTCAAGCAGGAGGC------CGCGCCCTACGACGGCGCCATGGTGGACGTCGAGTACTCGGCCACCGTCAAGACGGTC---------GCCGTGCCTGCCGCCGCCGCTGCTGACAACGCGGCCGGC---GGCTTCTCGTCCATCTCCGACCGCTTCCCGCTCTCCAAGGTGGGCAAAGTGAGCTGCGGCATG---CCCATGCCCATGAGCCG---CTTGCTCAATG---CCACGCCCGAGATGACGC---AGGCAGACCAG-------------ATGACGGTGCTCGTGGGGGACGGCACGGTCGACGACAAC---ACGATCAAGGCCGAG---CCCGAGGGTGAGCGACGTGTACCGGTACCAGGCCACGGA--CGTGGAAGAGAACGAGATGGAGCAACTGCGATGGAACATCCAGGAGCAGGTTTTCCAGCGGTACTTCCAGCAAGGACAGAAGTACGAGGAGTGGGGGAAGCTCGAGGAGCAGGACACCGTGTCTTCTTCATCCCAGGCGGACGACGCGAGAACATTCTGGCCGAGAAGCGGTTCCACGACAATGCCGTCCGGGACGCCATGGGGAAGGGAAACGCCACGTGGGTCAAGGAGCGACGGGCGCGATTCGAGGAGGAGATCCGCCAGTTCTAAGAAGAAACACGACACAAGCAGGAGCTGATGCTTACGCGGCTTGAAAAGGTGTCTCCGTTCTCCAAGTTCCCGATCCTGGGCAACCGCTTCTTGCTGCTTCGTCTGCGAGGAAAGGGAGGCAATGGAGAGGTGTGGGACGTCGTGGACTACGCCAACAACAAGCAACTCTGCGCTCTCAAGCTGTCCACTTCCATTCGACACGCCCAACGCGAGCACCACACGCACACTCTACCACCCCA--ACATCGTCCAGGTTGGCGAGGTGCCTTTCCTCATCCGATATCAGCAACAGCAGTACACAGCTTTCACTGTAGCAAGTGTGCAATCTGACCTGCAGCAGCTCATTGAGA---TGTACGAGTACCTCGATGATGACTCGGCCTACAAGGTGCTCTCGCAGCTTCTGAGCTCGCTCAAGTACTTGCACGATGGGTGTTGCTCACTACGACTTGAAGCCGTCCAACATCCTGATCGACCACGATGGGTGTGTGAAGCTG------ACGGACTTTGACTTGACGCGCAACGCCAAATCAGCTACGTCAAACTCTACGGTGGGCACTCTACGCTACCTTCCTCCCGAGTGCTTCCGGCCAAACCGAGGCGA---------CTGCGAAGCCACGGCTGAGAAGGCGGACATGTGGATGGTTGGGATCGTGTACTGCATGATGGTCACTGGAAAGCACCCAATCTGCCCAGACAAGTCCACGCCAGCGGATGTCAAGTCTACGATGGCTCAGTACACGGGC---GAGCTTCGGTACGCACGACCGATCTCCGACTTGTCGCGATGGATCTTACAGGGATGTCTCCACCCGGACCCACGGTGTCGGCCTACTGCCGCTCAGTTACTGGTGGCAATGCAG

Phytophthora_idaei TGGACTACCACCGCCAGGTGGCCAGGGAAGTGGCCGCCTTCTTCCACTCCTTCTACCGCTTCGGCGCCGCCTCCAACCGCTTCGATCTGTTCCTGCACAACGTTCTCTTTGGCTCCATGTGCATCGGGGCCGTCGTCTTCCTCGTGCTCTGCGTGATCGTCGTGCGCCGCACCATCCTGCACATCCGCAGGCCTTTGTTCGACGCACATCGATGGATTCTACGTCCGTGGAGCTACTGGCCGTCGGACTGCTGACATTCCTGGCTATTAGCGCACTCGGAGGACTGCTGGGAGAAGCTGTCTGCAGACCAGCAACAGCTTGCGCGTCAATGCTGATAATTTAATCACGTCATTCAACGACAGTGAACTACCAGCTGAAGCCTTCAAGATGTCAGTGGAGGCGCTGGGCTAGTGCACGCGTCCAAGGAGCTGTGGAATCAGACGGACGCGCTGCTGCCCAAGAACTACTCAGATATTGCCAAGGAGTGGGAGTTCAACTACTTCGTGCTCAAGTCGTCGACCAACGGCGCCATTCTTGCAGTATCACTGGCCAGTTTCCTATCTATTGCATCGGTCGGATGGGCCATGGTCTTGCCACTGCGCGTCTCGATTCTGGTGATCCTGAGCGTTGTACCCATTTCACACACGCTGATCGGCGCCTACCTGTCGTCGACGATCATGACGGCGGACTTCTGTACCGCTCCCTTGAACAACACGCTCGAACTGATCGGCACGTGTCCAGCCAACGCTTCCTGCCTTTCGCTGACGCTATGGCAACGTAGCGCTTCTCGTCAGGGACCAACTGGCCAGCGTATGAAGAGAGAATTCCTGGACCCCATCGGAGCAACTGTGGGCGTCATGCTCGGCTATTTCGCAGGAACTGTGAGCAATTGGGAGAGTTTCTCTCTCATCCAGACGTGGCGCTGGCCGTTCCTGCTCCAGTTTCTGGCTCTGCTGCCTATTAATGTAGCCATCTTTTTCGTACCAAAGAAACACTTGGTGATTCGCAGTGAGATCAACGAGTTGGAGCCACCCGTGGCCGGGACGCCCATGAGCACAAGATCCACGACGTATCACGACGAAGAAGACGCGTTGCTCGCCAGTGCCAGCATGCAACGTACGGGGCTTTGGACACGAGGTGTACGAACAGGGCGCCTTCTCGGAGGGCGTCTACGAGCTGCTGCATATCCCAGTCTTCTGCTTGATCGTCTTCGGCCTCACGACCGTCTACTTCGTCGTCACTGGCGTGCAGTATTGGAGCACCATCTTTATGATCAAGTCGCTGCATGCGTCCAAGTATCTTGTCAACGCGCTCTTCGTCGTTGTCTCCGGCACGGGACCGATCCTCGGCGTGTTCTTTGGTGGCTGGCTTATCGACAGATACGGAGGATACATCGGCGTGGAACAGCGCGCAAAAGCTCTGGGTATCTGCATGATCCTGGGACTCACAGCGTTCGCGATCTCAGCGGTGACGACGTTCTTCGACGACATTTACATCACTGCTGGGTTCCTGTGGCTCTTGCTCTTCTTCGGTGGCGCCATCCTGCCAGCGCATCGTGCGCTCGCCTCGTCCTTCTCCGTGATGGTCTTCAATCTGTTTGGCTACGCGCTTTCTCATGAGCGACTTGCAGATGGTGGTGGGCGCCAAGTGCTTTGTGCCGGATGAGACGCACGTGTGGCTCGCGGCCGAAGTATTGCGTGACGAGAACTCGGGTGATGGCAAGACGCGCAAGATCTACTGCAACGTGGAGCTGCCCGATGGAAACCGAGGAGCGCTGCGTGGACATGCTGTCCAAGAAGACAAAGGCGCTGCTGATGTGCACCAGCTCGAGTCGCTTCCCTACCAGAATGAGAACGTGGGGGCCGAGGGCATCGAGGACATGATCACACTCAACTATTTGCACGAGGCCGCCATCCTGTTTAACATCAAGAAGCGCTTCCTCTGCGAGCTGCCCTACACGTACACAGGCGACATCTGCATCGCTATTAACCCCTATAAATGGCTCCCGGACTTGTACGCTGAAGAGCAGCACCTCCGCTACCTCAACCAGCCCAAGGAGGAACTGCCCCCGCACGTGTACGCCACATCCGTCGCTGCCTACGACAGCATGCGGCGTTCGGAGCGCAACCAGTCCATTCTGGTCAGCGGTGAATCCGGTGCCGGTAAAACGGAGACTACTAAGATCCTCATGAACCACCTGGCCACCATCGCCGGTGGTCTCAATAACTCCACCATCAAGAGGATTATCGAGGTGAATCCACTGCTCGAGTCTTTCGGTAACGCCAAGACTGTCCGTAACGACAACTCCAGTCGATTCGGCAAGTTCACACAGCTTCAGTTCGACACAAACGGCACACTTGTCGGCGCTAAGTGCCGCACGTATCTTCTGGAGAAGACGCGTGTCATCCAGCACGAAGCGCCTGAACGCAACTACCATATCTTCTACCAGTTGACTCCTCCGACATTGCGTCGGAGTTGCAGCTCGATTCGACGAAGCATTATGTGTACACGGGTGACAACACGGCACAGAAGATCGAAGGGCTCTCTGACGAGAAACACTTCAACAAGACACGCGAGGCACTGGAGCTCGTCGGTCTGTCTCGGGATGACCAGCGGCCACTATTTGAAGTTCTGGCCGGTGTGCTCCACCTCGGTGAGGTGCAGCTGCAGTCAGACCCTGCAGATGACGAGAAGTCTCTCATCACTGAGGGCGACAGTGGTGCAAATTCTGCCACGGAGATGTTGGGAGTCACGTGGGAGGCACTTCAGACGGCTCTGTGTTCGCGTACGATGCGTGCTGTGAACGATGTGTACAGTGTACCGCTCAAGAAGGAGCTGGCCATGGACTGTCGTGATGCCCTCGCCAAGGCCATCTACTCCAATGTGTTTGACTGGCTTGTGGCTACGATTAACCAGTCGCTTGCTGACGATGCCAACATGGCCAACCACGTGGGTGTACTCGATATCTTCGGTTTCGAGCACTTCAAGCACAACTCGTTCGAGCAGTTCTGTATTAACTACGCCAACGAGAAGCTTCAGCAGAAGTTCACACAGGATGTGTTCAAGACGGTGCAAATTGAGTACGAGGAGGAAGGCATCGTGTGGGACCACATTGAGTACGCGGATAACCAGGACGTGCTGTCCGTGATTGAGTCGCGCATGGGTATCATTTCGCTGCTGAACGAAGAGGTGATGCGACCGAAGGGTAGTGAGGAGTCCTTCATGTCCAAGGTGACGTCCCTGCACAAGGAT---GATATGGCGCACGTTATTGAGTTCCCTCGCACGTCGCGTACCGAGTTCCTCATCAAGCACTACGCCGCTCCTGTCATGTACGACTCGGTGGGATTCCTGGAGAAGCACAAGGACTCGCTGCTGCCTGACCTGTCCGAGCTCATGCGTGGTTCGTCCAAGCCGTTCATCGCCAAGCTCTTCGAGCCCAAACCGGAGCCTAAGCCGCCGGAAGC---------GTGGTGGTGCTCTGTCCATCACGACTGTGGGCACCCAGTTCAAGGAGAGTCTGACGGAGCTGATGGCTACCATCAACTCGACCCGTGTGCACTACGTGCGTTGTATCAAGCCCAACCCGATCAA---------GAGTGCAACTGTCATGGACCAAGCTATGGTGGTGTCCCAGTTGCGATGTGCCGGTGTGATTGAGGCCATTCGTATCTCGCGTGCAGCGTATCCGAACCGGTTGCAGCACACGGAGATCCTGGACAAGTTCTGGCTGTTTGTTCCGAGTGGCGGTGAGACGGCTGCAGACAAGTGCCAGCTGCTCATGGACAAGCTTAAGCTGGAGACCCCCACTCAGTACCAGATGGGTAAGACCCGTGTGTACTTCCAGCTAGGCGTCCTTGAAGAGCTTGAGGATCGCCGTAAGAAGTTCCTCGATGCCAAGGCCACATACCTGCAGAACATCATGGTTGGCTTCACGCAGCGTATCAAGTATCTCCGGCAACTGGAAGCAATCATCAAGTTCAGTGATTCGGTGCGTGATTGCCATGCGCAGATACAACACGTTCTTGAAGGGCCTCATCTCGGCGCAAGCTCACTGGCGAGGCATGCAGGGCCGCAAGGTGGCAGCTGAAGTCAAGAGCAACCACAACGCTGTCATTATCCAGCGGTATGTGCGTGGATTTGTCAAGCGTCACCGGTACGTTAAGATGCGCGAGATGGTCATCCGTGTGCAAGCTGTGGTTCGTATGACGATCCAGCGGCCAAAGTATCTGGCTGCTCTCGAGGAGAAGCGGCGTGAGGCGGACATGGCGTATCAGCTGCACAAGCTGAAGGCTGCTCTGCAGGAAGAGCAGGAGCGGAATGCGCAGCT------GCAGCGCCGCAGCAGCAGCGTGGC--GACGGCGCGTCCGCTGCCTCGAGTGTTGTGATGGCTGATGCCGGCGGAATGATCGAGACTCTTACAGACGAGAACAAGAAGCTGCGTGAGAAGAACGAGGATATGAAGGTTACGATGAAGGGCTTGAAGGCGGAGATCGAGAAGTTTAAGAGCGACAAGGAGTTCTCGTCGGCCGGTAACCACGTGAAGGTCCGCCAACTGCAAGACACAGTGCGTGACAAGGACAAAAAGATCTCGCAGCTCGAGGCCGAAAACAAGAAGCTGACGGAGCAGATCGCCAAGCTTCACGCGGAAGGTGTTGT---TCCGGAGAAGAAGCCCACGCCGAAGAAGTCGATCTTCCGCACGCTGGGCTCCAAGAAGGAGAAGAAGCCTC---------------GCGAGACAGTGCTGATGGACTCGCTCACCGGCTCGGACGACCTCAACCGTCGGGAGACGCGTCCGTCGGA---GCGCCACTTCCCGCGCATGC---CGAGCATGAGTGTGGGCTCT---CGATTCTGGAACTCGAACAAG---AAGGGCGAC------------AACGGCGACGGCTTCGACGTCTCGGAGGTTGAAGGTGAGGACAGCCCCACGAACCCACGCGCAACACGATCGTGAAGAGCATGGAAGTGGGTGTGACCGGTGCCATGTCGAGTCTAAAGGGCCGCATG---------TCGGTCGTCAAGGGGATGTACGAAGCAAGCGCACGAACAGGACCGCCCCGTGCGCGAGTCGTTCAACCTGGACGCGTTGCCTGAGGTCTCACTTCCTGTCGGCTGGGAGGCCAAGGTGTCGCGCTCGACGGGTCGTGTGTACTACGTGAACCGCAAGCTCGGCAAGTCGCAGTTCGAACGCCCCACTCTGGCCTCGCTCAA------GGCGCAGAAGTTGGCGCGCCAAAAGTCGGCTAACGGGCTG-AAGGCTGTGGTCGCTGATTTCGGCTGCGGAGATGCCAGACTGGCTGAAAGCGTGCCGAATAAAGTGCACTCGTTCGACTTGGTGTCCATCGATATTGCCGTGTACTGCTTGGCTCTTATGGGAACGAGTGTCCGTGAGTATGTGCGTGAAGTGTACCGTGTGCTGAATCCAGGAGGCGTGCTGAAAGTTGCTGAGGTCAAGAGTCGCTTCGAGAGCGAGTCACTCGGAGGATGGTGGCGGTGTTTGTGACGGCGGCGCAGGGCGTTGTGGTGACGCTGCGCAACACGACGGTGTTCGTGGAGAGACCAAATACGCAGTTATTGTGGGCCAGAAGACAGGCGAGGCCGTGGCCATGCCGTTCCCGGACGTGGACCGCGACCTAAAGACTCTGCTGGGCCACACCACGTCCATGGTCACCCACGTGGCCGTAAATCTCATCGCTGCTACTAACGGCCGACCGTGATGAGAAGCTGCGTGTGTCAAGGTTGCCGAATGCTGCGATCATTGAGAGCTACTGCTTGGGACACGCTGCCAGTCTGACCAAGGTGGCCTGCAGTGCAGTGACCCCCGACCTTGTGGTGTCCACGTCCATGGACAACACTCTAAAGCTGTGGCAGATGACGACGGGCAAACTGGCCATTTGCCCCAAGACGACATTGTGGCCGTGCTTGTGAACTACCAGCACGTGCGATTCTTCGAGATCGTGAGAACCCATTCCTGCAGTTGTTTTCGATCTCAAAGGGCAAGTGCTGCATTCACCCGCTGTGCGCGACTTTCGCAAGCTTCAGAACGACCCGCCGTCGGGCGTGTCGGGTGCCCCCATGGACAACAACATCATGCTCTGGCAGGCCGTCATCTTCGGACCGGACGACACGCCCTGGGAAGGCGGCACTTTCAACCTGACGCTTGAGTTCAGCGAAGACTACCCCAACAAGGCGCCGACCGTCAAGTTCATCACGAAAATGTTCCACCCTAACATCTACAACGACGGCCAGATCTGCCTGGACATTCTGCAGAACCAATGGTCGCCGATCTACGACATCTCGGCCATCCTCACGTCCATCCAGTCGCTGCTGTGCGATCCCAACCCGAACTCGCCAGCCAACTCGGAGGCCGCGCGCCTGTTCCAGGAGAACCGACGGGAGTACAACCGCCGCGTGCGCGAGATCGTCGAGCAAAGCTGGCAGGCGATCGATGGTGTCGGCCGCCAAGATGCGTGGCGCTGAGAACCGCATGAACGACGGCCGCCCCTTCACCGCGTGGCTGGACAATGTCTCGTCGGACGCTGGTCTGTGCGGCGGTATCAACTCGGGTATTGCCAAGACCACCCGCAGGCAGACCACTCGCAGATCTTCGTGATCGGCGACAAGGCCCGCGCCCAGCTGCGTCGCGACCTCGGCGACAACATCCGTGGCAACGTCACGGAGACGTACCAGTCGCCCCCCAACTTCACGGTGGCCAGCGCCATCGCGGAGGCTGTCGAGTCGGAGAGCAGTCGTCCCGTATGGCTGCCATGGAGAACGCCACCAGCAACGCTGAGGACCTCACGCTCACGATTGTGTACAACAAGGCTCGCCAGGCCCGTATTACCACGGAACTGATTGAAATTATCTCGGGTGCCGCCTCGCTGGACAGTGACTGGCAGCGTGGTGAGCGTCCAGAGCGTGGCGCAGTTTGACGAGGCGACGGTGCGCGAGTCGACGCTGTCCGTGAGTCTCTTTTGGGCCGAGTTCCAGGCCTGTCGCCCCAATGGGCAGCTGGACGTGGGGCCGCGCCGTGCTGGACAAACTCGAAGGCGCCAATGTGGCCGAGCTGGCCAAGCGCGTCTCCAATACCGCCTCAAGAAGCTCATCAGTGCGTCACCCGTCATGCTCTTCATGAAGGGCAACCCGACGGAGCCCAAGTGCGGCTTTAGTCGACAGATAGTGGCGCTTCTGAACGAAGAAAAAGTCCAATTTGGCACGTTTGACATCCTCAATGACGACGAAGTGCGTCAAGGACTCAAGCAGTTCTCCAACTGGCCCACGTATCCACAACTCTATGTGAACGGATCGCTTATTGGCGGTCTGGACATCGTCAAGGAGATGAAGTCGGAGGGATCCATCGTCGAGCAACTAGGCGTCGAGGAGGCTGAGGCTGCCTTCCAGGAGAGTCTGCATGCGCTCGTCAACTCAGCTCCAGTGCTGCTCTTCATGAAACTTCGACATCCTGTGTGGAGCTACCAACGCCAGGACCAAGACGTCGCCAAGGTGGTGACTCTCACGCCACCCTCATTCCGTCGTGATATGTGCCGAGGCCATCTCATGGAGCCCGAAACCGCATGGCCATGGCATTCCGTGATCCTTTGCAGGAAAAGGGAGAGTTCTGCATTGTGCAGCTCCTCGTATCACCTCGAAGCGCACTGTTCTGCATTCACGAGGTATTTCTGCGATCGACTGGCAAGTTTAATGTCCTGCACCGAGACCACCGCAGCGAAGTCAAGTCGCTCTGTGTTCACTCTCAGCGTGACTGTTTGTACACGGGCGGTCTTGACGGATTGTTGATCCGATATGACCTGAAGAAGGGGCGTAGTGAAGTTGTGATGGAGCGTCGCAAACGTATCTCCAAGATCAACGCAGTACTGGAGCATCCACACAATCCGAATCTTCTTTTGGTCAGCTCCGTGGAGCAGTAGACACAATTTGTTGCTGCACGACCTGCAACGCTATGATCGTAACGTGTCGGGAAGCTCGATGAGTCAGTACGTCGTGCCGCGGTGGTCGCCTGCTGGTTATCACGTCTCGTGCGGCAGCAAATGCGCTACTGCCACGGAAGCTTCTCGACACCCACGTCAGCGACCATCGGGGCCTCGTTCCTGCAGAAGCGCGTGATCGTCGGTGGGAAACTGACGCTTCAGATATGGGATACAGCCGGCCAGGAGCGCTTCCGCTCCATGGCGCCCATGTATTACCGCAATGCCAAGGCGGCTATCCTGGTCTTTGACCTTCAGAGCGAAGCCAGCTTCGAGAAGATCAAGGAGTGGCTTCACCTGCAGCATCACGTGGGTGACGACATGTGGGCAACAAGAGCGACGTTTCGACTTTGCCAAGGCCCAGCAGTTCGCGGATGAAATCGGTGCGCTGGTGTTCCGCACGAGCGCCAAGACGGGTGAAGGAGTGCAGTCACTGTTCGAGAGTCTGGCGGTGCAGCTACTTAAGAAGCACGATCAGGAGCAGCGAAGAGCTTCTACGTTGTGTACGTGCCAGCGAATGAGGCCAAGGAGCTGGAGGAGTGGACGGTCAAGCTGCCGGAAGACAAGGGGGCGCAGCTCGGCTGCTTGACGGAGCGTCTGCGCAAGCATTTTAAGAGCGGATCCATCCAAGCAGCAGCAGAGACTTTCAAGCAGCAATTGCTGACCCAGTTGCCCAAGGGCGCCACATTGACGGATGAGATGCTGCAGATGATGCATGGACAGCCTTGTGGACTCGATCCCGCTCGTGCTCAACACGCCCGACGTCAAGCACGTTGGAGGTCAAGGGCGACGCATTCATTGGACGAGTGTTCGATAGACGACTTTGTGCGTATGGACTTCCGGTTGAGTGAGGTTTCGGGTGACGCGCCTTGGGTGCAGGCGGCCAAGGTGCAGAGGTCACGCAGTCGCTAGAGGAGATCCTGGCACTGGGCCAGGGCCTAGACGACGAGGACTACAAGGCCGAGCTCGAAGTGTTGCAGAACGACCCCAACTCCAACCTCTATTCGGCTGTCACCTTCGAGGAACTCAACCTACCGGAGGCGCTGCTCAAGGGCGTGTACAACATGAAATTCGCCAAGCCGTCCAAGATTCAGTCCGTGGCGCTGCCACTGATTCTCGCCGATCCTCCAGAGAACCTGATCGGCCAGGCCCAGAGTGGCACCCCACCCGCGAACTGGTGCGACAGATTATGGCCGTGGTGAACGCCATGGGCAAGTTGTGGTCACGTTCCTGGCCATCCCCGGCAACGATCTGGCTCGCGGTGAGACGCTGAAGGCACAGGTGATCGTCGGTACCCCCGGCAAGGTGGAGGGTATGATCAAGAAGAAGCAACTGGACACTCGTGACGTCAAGATCTTCGTGCTGGACGAGGCTGACGTGATGGTGGCCGAGGACGGCATGCGCGAGCGCTCCGTGTCGATCAAGCGCCTCATCAGGAACCGCGCGTGCCAGTACCTGCTCTTCTCGGCCACGTACGCTGACGATGTGCGCGACTTTGCCCAGAAAATGGTGCCCGACCACAACATTATCACTGTCAAGAAGGAGAAGCTCACGCTGGACGGCATCAAGCAGTTCTGGATCGACTGCAAGACCCGCGACAACAAGTTCCAGGTGCTGTCGGACATTTTCGCCATCCTGAGCATCGGCAAGTGCGTCATTTTCGTGCAGCAGCGTGAGACCGCCAAGGAACTGACGCGTAGTATGCGTGAGAAGGGCCACTCGGTCGGCATCCTGCACGGCGCCGACATGGCCAAGGAGGTGCGTGACCAGGTTATCGACGAGTTCCGTGCCGGTACGACCAACGTGCTGATCACCACGAACGTTCTGGCTCGCGGTATCGATGTGGCGGGCGTCTCACTTGTCGTGAACTTCGACATCCCGCTGACTCGCGATCACCGTCCGGACCCGGCGACGTATCTGCACCGTATCGGCCGTACCGGTCGGTTCGGCCGCAAGGGCTGCGCTATTAACTTCGTCTACGACAACAAGAGCAAGCAGGACCTCGCCGAGATTGAAGAGTACTACGCACGTCCGATTACTCAGGGCCCCGCAGACGACATTGAGGAACTTGAGAAGCTACTGGCTTGGGGGGCAGCAAGCGCCGCCTGGCCAAGTTCGGCAAGGAGCAGCGCAAGGGCATGCGAATCTACCCTCGCGAGCCCAAGAAGAAACCTTCGGGCAAGGATAAGACGTACTACTACCTCAAAGACGTCATGTTCCTGGCCCACGAGCCGGTTCTGGAGAAGTTCCGGGCGTTTAAAACCTTTATGAAAAAAGTCAAGAAGGCCATGGGCAAGAAGGACTACGAAGATGCCAAACGCAAGCACGAGAACCGCCCTACCTATGAGCTGGACCACATTGTTAAGGAGCGTTATCCGCGCTTCGTGGACGCTCTAGGAGACCTGGACGACGCTCTGTGTGGCAGAACTACGTTGTAGCCACTAGAGCCTTGACCAAGGTGTTTGTGAGCATTAAGGGCATCTACTACCAGGCTAACGTCCAGGGTCAGACCATCACGTGGCTCGTGCCCCACCAGTTCACCCCAACGGTGGATAAACGTGTGGATGTGCGTGTGATGCTCACGTTCCTCGAGTTCTACGAGGTTCTACTCAAGTTCGTGAACGCGCAGCTCTACCTGCAGATCGGTGTGGCTTATCCGCCCAAGATCAACCTCCAGTTGGACGCTGCTGGTGCGCAGTTGGCTGCTCTGGAGCTAGCAGCAGAAGGAGTCGGAGAAGAGGATCAAGCTACAGCGAGCAGCGCAAGGAACGCAGCGAGAAGAGCTTGTTCAGTGGCTTGACGTTCTTCCTGTCGCGTGAAGTGCCTTCTGCGTGTCTGGAGCTGGTGCTGCGCTCGCACGGCGCTACGTTGGGCTGGGACGGCGAGGGATCTCCGTTCGACGAGAAGAGCTCGCGTATCACGCACCACATCATGGATCGTCCGCATCAAGGGCATCGGTACTTCAACCGCGAGTACGTGCAGCCTCAGTGGGTGTTTGACTCGGTGAACAATGGCATGTTGTTGCCGTTGACGCGTTACAGAAAAGAACTGGAGAAACTCAAGTCTGCCTCGCAGGTTCTACGTGAGGTGGGGGCTGTGGGCGACGAAGAAGAAAACGGGAGGCCTACGCCGCGGATCTCAAGGCTGAGATGGATGGTAACAAGAAGTCGAAGCGTGTTAAGAAGGATGAGGAGGCTGCAGAGCTCAAGGAACTGGCTAAGACTATGATGTCCAAGAAGGCCAAGCGTCTGTACGACCGCATGCAGTACGGTCTGGGCAAGAAGGCTGACAAGATTCAGAAGCTTGAGGACAAGCGTGCCAAGATCGAAGCAAGACCAAGAAGTTGCTGGCGGGTGCGCTGTCGGGTATGATCTCGGCGTTGGCCTTTCAGCCTTTGGGGCGCTGCGCTGGCGCGGCACGTCTCCCACGCTTGTGCGCGTGGCTGGCGGTGCCGGACTCTACTTCGCCACGCTGGATCACTGCCTCAACGTGTTCCCACACTCTGCAGTCAACACGTTCCTGGCTGGAGCCTTCGCGCGTACTTTTGCAGGTGGCGTGATGTCGCCCTTCACTATTGTCAAGGCGCGGATGGATGCCGCCCGGTACTTTCGACAGCAAACGGACGTGCCATTTTCTGGACTGTATGTTCTCATATACACGGCACCCAGCCGATGTGGTGAAGACACGCATGCATGCGACAGACCGTGGCCAAGATCTATCACCACGAAGGTCTGCGTGGCTTCGCGAAGGGTATCATTCCGCGTGTGGTCAAACGAACGCTGTCCACCGCAGTCACGTGGACAATCTACGAGCTACTCGTTCGACCCGTCGGGTCTGGGTCACGCCAAGGAAGCCTTTAACCTGGCCAAGGAGACGGAGCGCACCAAGCAGATGGAGAATCAGGCCAAGATTAAGGAGAACGAGGCGCTCTACAAGCAGTACGAAATCGTGCGCGTGCAGAAGGAGGGCGAGGAGCGCCGTAAGACGCTCGAGGAGGAGACGCGCCAGCACCAGCAGCGTGCGCAATACCAGGACCAGCTCAAGCGCAAGCAATACGCTGACCAGCTGGCTGCCCAGAAGTACATGAAGGAGCAGGAACTTAAGAAACAGGAGGAGATCCTCGCCCGCCAGGAGGCCTCACGCCGAAAGACGCTCGACTATGAGGCTGAGCTGCGTCAGAAGACTGAGCTGGTCAAGGTCGCGGCCGAGACTGAGGGCCGCATTAAGCAGGAGCGTCTTAACCACGACCTGCACCTCGAGGAGGCCCGTGTGCGCGCCAAGGAGTACCGCGAGACCGTCATGGAGGGTATTAAGCTGGCTGGTAACACCGTCGGATCCGGCATTATGACCTTCGTGGACGATAAGGAGAAGCTCACGGCCACTGTGGTCTCTCTGACCGCTCTGGCCGTCGGTATCTACACCGCTAAGGTGTCCACCAGCGTTGCAGGCAAGTACATTGAGGCACGCATGGGAAAGCCGTCGCTGGTGCGTGAGACGTCGCGTCGCTCAGCTGCGCAGGTGCTGGCCAACCCCATTCCGTCCATCAAGCGCGCGCTGCGTCTGCAGAAGGCGACGGATGCCCTCGAGGGCGTCGTGCTTGAGCCCAAGTTGGACGAGCGTCTGCGCAGCGTGGCTGTGTCCACGTTTAACACGAAGAAGAACCGTGCGCCGTTCCGCCACTTGCTGCTGCACGGCCCGCCCGGTACAGGTAAGACGCTGTTCGCTAAGGCTCTTGCTCGTCACTCCGGTCTCGAGTACGCCATCCTTACGGGTGGTGATGTTGCCCCATTGGGTCGCGAGGGTGTGACTGAGATCCACAAGCTGTTCGACTGGGCATCGCACAGCCGTCGCGGTCTGCTGCTGTTCGTCGATGAGGCTGATGCGTTCCTGCAGAAGCGTAGCAACACCGTCATGAGCGAGGACATGCGTAACGCACTGAATGCCTTCCTGTACCGCACGGGTGAGGCCAGCGACAAGTTCATGATCGTGTTCGCCAGTAACCAGCCGGAACAATTCGACTGGGCCATCAACGATCGTATCGATGAGATGGTGGAGTTCCGCCTGCCCGGATTCGACGAGCGTGTGCGCATGCTGAAGCAGTACTTCGACGACTACATCCGTGCGCCGACGAACTCGCGTGCCAAGAAGATTTACGCGAGGGCGTCATTTTCACGCTGGACCCTGTAGCCAACTTCCTCGTGCCAAGACGCGCATCTTCCAGCTCGAGGCGCTGCAAGGCGTGTCCGGCGAAGCTCAGGCTATTTTCGATGCACTCAACAAGAACCTATGCGCATCTACTGGGTCGTCTTCGTCGTGGTGCTGGGCAACGGACTGTATACGTACTTCGCCGGCAAGGACGAGTCGTTCTTAGTGGAAAAGCTCAAGAGAAAAATCCCGTCCGAGTACCGCATCTCTGAGAAGTGGGACAAGTGCCTGGAGAACTTCACGCTCTACTTTGGCGTTGGCCTCGTTGCTGGTGGCCTCTCGTCCCTCGTGCTGGCTCGTTCTGGAGCTGGTCGCGGACTGGTCACTGGTCTAGGCGTTCGGGCACTGGATCAAGCTGGACGACGTGCCAGTTGGCATTCGCGGGCAACACACAGGCAGCTCTGGACAAGACGGACAAGGCTGTGGGCGACTTGCCAGCCTTCCAGCAGCAGACGATCGTGGACTTTGTGACGGCCTCGGCCACGCAGCAGCAGCTGCAGACCGTGCACCCGTGGCTTCTGCGAGGAGGCCAGTGTGAAGGTCTTGGAGACGTACCTGGCCGAGCAGGTGCAGAACAAGACCGTGGACATTGACGCCAGCCTGGCGCTGCTCAAACTGTACCAGGTGTACCCGGCCACGACCAACGCCGAGAGCCAAGGTGCTGGTCAAGGGCGTCATGGCACTGCCTTCCACCTTCTTCACGGGTGTCTACCATGGTCCCGGAGAGCATCCGCGAGGATGCCAACGTGACTGCGGTGCTTCACACGGGCTTCATGCTCCAGTCGTGCCTGTTCGAGGACTTTTGGAAGGAGAAGGTGTCTTTCGCCGAGAAGGTGCCGGGCTTCCTCGAGTCGGTGCGCGCCTACATCCTGACGGCCATCAGCCGCAGCCACAGCGTCATCTCCACGGAGGTCTTTAAGTCCAAGCTTAACGTGTCTGACAAGGAGGTGGCCGAGATCGTTGCCGAGAAGTGGACGCCGACAACTTGATCCAGATCAACCCCAACGAGGACAACCAGATGCAGGCCAAGAAGGTCCAGGAGAACATTGAGTTCGAGGACGTGCTCAAGGTCATCCACACCCTTTCTAGATCCAACGTCTGCGCCAGTGGCGGCGGCGTGGCTGATGCCGAGGAGGTCTGTGAGAATCTAGCCAAGCACTTGAGGCTAGACGGGGGCTCCCAGGTGCTGCCCTGCTCCACGGGTGTCATTGGATGGCGTATCCCGGTGGATGCCATGGTCGAGAACCTCCCCAAGCTCGTCAACGGCCTGCAGTCCGACTCGGTTCTACCTGCTGCCGAGGGCATTATGACCACTGATTTGTACCCGAAGATCCGCTCCATGGACGTGTGTGGCGGCCGCGTCGTCGGCATTGCCAAGGGTGCTGGTATGATTGAACCCAACATGGCGACTATGCTGTCCTACATCCTCACCGATCTGTCGATTCCGCGTGACGTGCTGCGTCAGCTGCTGACGGAGGTGGTTGGAGACACGTACAACTCGCTCAGTGTGGACACGGACCAGAGCACGTCCGACACGGTGACGCTCGTTTCGTCGGACCAGATACCGTTCGACATGAA---GCAGCTGGACGAGTTCAAGAAGGCTCTGCATGAGGTGTGTCTGGGACTGAGCGAGGACATTGTACGCAACGGTGAGGGTGCGCACCACGTGATGCGTGTGCGTGTGAGTGGCGCGTTGTCCAAGGAGATGGCCAAGGGCGTCGGCAAGTCGGTGGTCAACTCGCCTCTGCTCAAGTGTGCGGTCGCAGGCAACGACCCCAACGTGGGCCGCCTCGTCATGGCTGTGGGCAAGTACATGGGTCTGCACCACAAGGACGTGAACATTCAGCGTCGCATGAAGATCCGGATGGGTGGCGTTCTCATCTTCGAGAACGGCGAGATGGTGCTCAACGACGATATTGAGGCCAAGCTGGTGGCCCACATGCGCGACGCGATGCTGATTGAGCCTACTCGTGGTGGCCTCGACGCCAGCTCGCACAACTACCCGCCACACGGGAAGTTTGTCGAGATGGAGATTGATCTGGGTGTTGGCTCCGAGGAGACTCAGGTGTTGGGCATCGACCTTACGCACGAGTACGTGGCCATTAACGCCGACTACCGCTCG--ATGGCGCC---GAAGAAGGGGAAAACTGCCGCCGCGTACCTGGCGTCCCTGGGTGTGACCCCTAC---TGCGGGAGGTGAGAAGAAGGACGAC---AAGAAGAAGAAAAAGAAAGGAAAGAAGAAACCCAAAAGAAGCCCATATCGGCCGCGGCGAAGGCCCTGA-TGGAGCGCCAGGCGGCCATGAAGGCCGAGCAGGAGCGCCTCGAGCAGGAGCGTCTGGAGCAGGAGCGCATCGCCCAGGAGGAAGAGGCCAAGCGACTGGCCGAGATCGAGCACGAGGAGGAGATTCGCCGCAAGAAGAAAGAGGCGCGCGCCACCAAGATCGAGCGTCAGAAGAAGGAGGGAACGTACATGACCAAGGCCCAGAAACAGAAGGCGGCCAAGGCCCAAGCCGCACTAGAGGCTATGCAGCAGGCGGGTCTCGTGCCCGACGCTGCT------GGCGAGAAGAAGAAGGTCGTGTACACGGACAACAAGAAGAAAAACAAGAAGAACAGGTGGAAGAGGAGGAGGAGGCCGAGGCACCCGATGCGTGGGATGACGAGGACTGGGAGACCAGCGATGTTGTGGCCTCACTGGAGGATAAGTTAGCAGAAGAAGGAGCAGGAGCGTCTGCGCCAGCAGGGAATCACTCTGAAGAAACGTCAGGAGGAGCAGGCCGAACAGATGCGTCTGGACGCCGAGGAGGAGCGTCAGCGCTCCAAGGCAAAGCTCGAGGCTGAGCGCAAGAAGAAGGAGGCAGCCGAGCGTCGTCGTGAGCGTGAGGCTGCTGCGCGTGCCAACGTTTCTCTGGACAACCTCCGTTCGCCTATTTGCTGTATCATGGGCCACGTCGATACTGGTAAGACGAAGCTTCTTGACAACATCCGTCGGACCAAGGTGCAGGACGCCGAAGCTGGTGGTATTACCCAGCAGATTGGTGCGACCTTCTTCCCTGTGGAGGCGATTAAGCAGAAGACGGCGCGCCTTGCCGAAACGATGAAGCTAGATTACCGCCTGCCCGGTCTGCTTGTGATCGATACGCCCGGTCACGAGTCGTTCACGAACCTGCGTAATCGTGGTTCGTCGCTCTGTGATATCGCCATTCTGGTGGTGGATATCATGCACGGACTGGAGCCGCAGACTCTGGAATCACTGCGTCTGCTACGTCAGAAGAAGGCCCCGTTTGTGGTGGCGCTGAACAAGATCGATCGTTGCTACGGATGGAAAACCATGCCCGACATGCCCGTCCAGGAGGCACTCAAGCACCAGAACGAGCACGTCATTCGCGAGTTCGAGGATCGCATGAAGGCGATCATTGTGGAGTTTGCTGAGCAGAAGCTGAACGCCGAGGTGTATTGGCGCAACAAGGACTTGGCGCACACGGTATCGCTTATCCCCACTAGTGCTATCTCGGGTGAAGGTGTGTCGGACCTGTTGATGATGCTGACGCGATTGACTCAGGAACGCATGGCCAAGTCGCTGGCGTTCGTTGACATCCTGCAGTGTACTGTGCTCGAAGTGAAGGTTATTGAAGGTCTTGGTACAACGATTGATACAATTCTGGTGAATGGTACGCTAGAGGAAGGTGCAACTATTGTAGTGTGTACATTGGATGGACCAGTTGTGACAACGATCCGTTCGTTGCTGACTCCACACCCGATGAAGGAAATCCGTGTCAAGGGCGAGTATATCCACCACCAGAAGATGAAGGCTGCTATGGGTGTTAAGATTTGCGCTCAGGGCCTTGAGAAGGCAGTTGCTGGTACGCAGATTTACGTGGTTGGCCCCGATGACGACGTTGAGGAGCTGAAGGACTCCGTCATGTCCGATCTGACGGGTATTCTGGACTCCGTGAAGGCTACCCGCCGCGGTGTAATGGTTCAGGCTTCCACCCTTGGTGCCCTGGAAGCGCTTCTGGAGTTCTTGCGCACATGTGACCCGCCCATCCCAGTGTCCTGCGTGAACATCGGCCCCGTCCACAAGAAGGACGTTATGCGTGCTTCGGTGCAGCTGGAGCACCAGCCTGAGTTTGCCACCATCCTGGCGTTCGACGTGAAAGTCCACAGTGACGCTACTGAGCTGGCCACAGAGCTTGGAGTGCGCATTTTCACTGCCGATATCATTTACCACCTGTTCGATCAGTTCACGGCGTACATGGACAACTTCCGTATGGCCCGTCGTGAGGAGTTCGAGGAGGTGGCTGTGTTCCCGTGTGTGTTGAAGATCCTGCCCAACTGCGTCTTCAACAAGAAGGACCCGATCATTCTCGGTGTGGACGTTGAGGAGGGTATCCTTAAGATCGGCACGCCACTCGTGGTGCCGTCCGCCGGCG---GTTTCCTGGTCGGCAAAGTGGGCTCCATCGAACGCGAGCACAAGGAGGTGGACCGCGCCAAGAAGGGAGCGTCTGTGGCTGTGCGGATAGACAACGAGGGCAGCGTCATGTACGGTCGCCACTTCGACTACAAGAACAAGCTGGTGTCGCGTCTGACACGCCAGAGCATCGACGCACTGAAGGAAAATTTCCGGGAAGACTTGCAGAAGGAGGACTGGCAGCTCGTCATCAAGCTCAAGAAGGTGTTCGATATCATTATGGAGGGCTGGCTCACCAAGCGCCGCGACCACATGAATCTCATATGGTTGGATCGCTATTTCCTCCTGGACGGCAACCAGCTCCGCTACTACCGCAAACGCGGCGACCCTGCGCCTCGAGGGACCTACATTCTGACTGATGGCTGCGTAGTGAGCCCCGTGTTCAATTCCGAGGAGAAACACAAGCACCACGGGCCCGTCTGGATGTTCCGCATCACTTGAAAAGTCGTACAAGAAGGACCGATTCCTGGATCTAGGAGCCAAGAGCGAGGAGAACGCCAAGGAGTGGAAACGCGCTCTGGAGAACGCCGTGCGGTCGCTGCAAGAGCTCGTAGCGCACCAGAAGAAGCTCAAGACCGACGCCGAGAT---CTTCCCCACGCCCACGCAC---------GTCCCGAGGATGTTGAAAGCCGATGAAGACACGATGTCTCTCTTTAATCTGGACGCAGACAATGTCAAGGCGGAGCAGGCCTGGTGGTTGGCCAGAGTGGAAGGGGGGCTCAGAATCATGCAGGAATGTCCGCTTGGACCCAACGTTGCGGCCAATCTGTACGCACAGCATGCAGCGACGACAAACGGCCTGATCTTCAGTGCGTGTGTGGGCGCGGCGCTGAGTTTTAGCAGCATGTATTCCTTCGCGAACAACATGTTGCTGAGCTTTATGACGGCACTGGTGGCCATTGCCGTGTGCTGCGCTGCGGTGACGCCTACACAGGCTCCCGAGATCCCGAGCTTCCGTGTGTCGCAGGTGGTGCATGGCTCCCCAACTGAAGTCTTCAGACTCATCATGAACCCCAAGAGGTTCCAGAGATGGGACTCAGCGACGGCGACCATGCGCGTGGTGCAGCAGCTGGACGACCACGCGGATATCGTGTACATTACGCAGCGCCCGACGCACTTGTGGCCGCTGTGGCAGAAAGCTCGCGACCTTGTGCTCATGCGCTACTGGAGACGTGAAGAGGACGGATCGTACTTTGTCATGTACCAGAGTATCGAGCACCCGGAGTGTCGTGTGCGTCACAATTATGTGCGTGCGAACTTGCTGGGCGGCGGCTTCGTTATCGCTCCCCAGCGTGTTCCCAGT---GGATCTATCCGTACCCTGGTAAGCTACGTGCTTCGTTACGATCCTGGTGGCTG---GTCGCGGATTTACCACCAGCTCGGTATGGACGTGGATCTGGCGATGCCCATGCTGCGTAGTGTTGTGGGTATTCGCGACGAAATGAGCGCCACGGACTTCATCACGCCCAATGTGTCGATGGCTCCTGCTTCAGAGGGCGGAGGTTGGACAGGAAGCGTGGA---CTCTATGGGAGTTGTGATGGGCGTACAGAAGCTGCAG---ACCAGTCTCCCGGAGAAGATGTGGGCCGAGCCTGACGCCTCTCTCTTCAGCGTCCGTGGACACAACTATCTGAACGACAAGAAGAAAATTCCTTCGGCGCCGGCGATGTTCCACACTGTGGGCGTGGATCTGTTGTCGTTCGAGAGTGCAGCCGAGAGGTACAACATCTCGTCTCGTCCGGACAGCATCGGCCGCACCAG---CTCCAAGTTCACGTTCGTTGTGAGCATGATCATTCCTAGCCCAGAGAATGTCTGCATGGTCTTCTACTTCCAGCCTGTGCGTGACAACGTGTTCGAGGACGGCTCTGGCTTCTCCGAGCTGCTGAACGACTTCTTCGACGGCGACGACCAGTTCCGGAACTCGCGCTTCAAGCTCATCCCCACTGTCGTTGAAGGCAGCTTCATTATCAAACAGTCGGTCGGTTCCAAGCCAACACTGCTCGGTAACAAGCTGAAATGTCCGTACCACCGAGGAGAAAACTACTTCGAAGTGGACATCGATATCTCGTCCAACTCAGTTGCCAACACGGTGGTGGGGATGGTGCAAGGAGTGACCAAGTCGCTGGTGGTGGACATGGCGTTCCTGCTCGAAGCCCAGACCGACGAAGAGCTGCCTGAAGTCATCCTGGGCGCTGTTCGCATGCAGCACATTTCGCTTGACAACCCGCGCCGCATGCCCAAGCATGGGGAACGGGCAGCAAGGACGAGTTCACGTTTTGAGGCTTTCACCGAGGCTCTGCGTGTGAATCTGCAGCGACTCATCGTCTACGCGCGTTCGGCCGATACGGCGCTGCAGCGAGAAGTCGCCGAGAAACTGGCCAACGAGGCTGTCAAGACCGACAGGTGCAGATTGTAGAGCTGGACGGACTGCAGCTGCTGCTGCCGCTGACCAAGTCGAAGGACACGGAGGTGCAGCGTCTAGCAGCGCACGCACTCGCCAACCTCTCAGTCAACTCTGAGAACCAGTCCAAAATGGCAACGGAGGGAGGCATCAACATGCTGATCGATCTACTGGGCAGCACGAATGAGCACGTACAGCGGCAGGCCGCGAAGGCGCTTGCCAACCTCGGCGTGAATGTGGACAACAAGGAGCGGATCGCCAAGGCGGGCGGCATTAAACCGCTCATTGACTTGGCCAGTTCTCACCAGATCGGCGTCGCCGTCGAGGCCATCGCAGCGCTTGCCAATCTGGCTGTCAACG---ACGCCAATGAGGTCGAGATCGCACGTAAAGGAGGCTTGAAGCCAATTATTGACGGAGCGCATTCCGAGTCCGTGGAGCTTCAGTCCCAAGTGGCTCGCGCTCTACGCAACCTATCGGTCAATCCGGAGAACAAGCAAGCGATCGTGGAGCTGGGCGGGGTGGAGGCTCTACAGTCACTTGT-----------------TGATATATCTCCA----TCTTGCTCCCTCTTCCCCAACGTGGTCCTCACGCGCTCCTCGCCCACCACCTGTTG---GCCGTAGCGCCTGTAGCTCGCGTGGCTGAAGTCAAGGAGGAAGC------GATGCCCTACGACGGCGCAATGGTGGACGTCGAGTACTCGACCACAGTCAAGACGGTC---------ATTGTGCCTGCAGCAAAGCCCA---ACGACACGACCGGA---GGCTTCTCGTCCATCTCCGACCGCTTCCCGCTCTCCAAAGTGGGGAAAGTCAGCTTTGGCATGGCTCCTATTCCCATGAGCCG---CTTGCTCAACG---CCACGCCGGAGATGACAC---AGGCGGACCAG-------------ATGACTGTGCTCGTGGATGATGGCACCGTGGATGATAAC---ACCATAAAGGCTGAG---CCGGAAGGTGAGCGACGTGTACAAGTACCAGACCACTGA--CGTAGAAGAGAACGAGATGGAGCAACTTCGATGGAACATCCAAGAGCAGGTTTTCCAGCGGTACTTCAAGCAAGGACAGAAGTTCGAGGAATGGGGACAACTGGAGGAGAAAGACTCCGAGTGTTCTTCATTCCGGGTGGACGCAGAGAGAATATCCTGGCTGAGAAGCGGTTCCACGACAACGCTGTCCGAGACGCGATGGGAAAGGGAAACGCCACTTGGGTCAAGGAGCGACGGGATCGATTCGAGGTCGAAATCCGCAGATTCTACGACGAAACCAGGCACAAACAGGAGCTGATGCTCACGCGCCTTGAAAAAGTGTCCCCGTTCTCCAAGTTCCCGATTCTTGGCAACCGGTTTCTGCTGCTTCGTCTGCGAGGAAAAGGAGGCAATGGAGAAGTATGGGATGTCGTGGACTACGCCAACAACAAGCAGCTCTGCGCTCTTAAGCTCTCCACCTCAATACGACACGCCCAACGCGAACACCACACGCACACTCAACCACCCCA--ACATTGTTCAGGTGGGCGAGGCCCCCTTCCTTATCCGATACCAGCAGCAGCAGTATACTGCGTTCACTGTGGCAAGCGTTCAGTCCGATCTTCAGCAACTCATTGAGA---TGTTCGAGCACTTTGATGATGACTATGCATACAGAGTGCTTTCCCAGCTTCTAAACGCACTGAAGTACTTGCACGATGGGCGTTGCTCACTACGACCTGAAGCCGTCGAACATTCTGATCGACCAGGGCGGGTGTGTGAAGCTA------ACCGACTTCGACTTGACTCGCGACGCCAAGGCACCTACGTCCACCTCTACCGTTGGAACTCTGCGATATCTCCCTCCTGAGTGCTTCCGTCCGAACCGCAGCGA---------CTGGGAAGCGACGGCTGAGAAGGCGGACATGTGGATGGTGGGCATCGTGTACTGCATGATGGTTACGGGAAAGCACCCAATCTGCCCGGACAAGTCGACGCAAGCGGAAGTGAAAGCGACGATGGCTCAGTACACTGGC---GAACTCCGATATGCGAGGACTATTTCTGATCTGTCTCGGTGGGTCTTACAGGGCTGCCTGCACCCCGACCCGCGGTGTCGGCCAACGGCCGCACAATTGCTGGTGGCGCTCCAG

Phytophthora_infestans TGGACTACCACCGCCAAGTGGCCAGGGAAGTGGCTGCATTCTTCCACTCCTTCTACCGCTTCGGCGCTGCCTCCAATCGCTTCGATCTGTTCCTACACAACGTTCTCTTTGGCTCCCTGTGCATCGGGGCCGTCGTCTTCCTCGTGCTCTGCGTGGTCGTCGTGCGCCGCACCATCCTGCACATCCGCAGGCTTTTGTTCGACGCACCTCGATGGATTCCACGTCCGTAGAGCTACTGGCTGTCGGACTGCTGACCTTCCTGGCGATAAGTGCACTCGGGGGCCTGCTGGGAGAAGCTGTCTACAGACCAGCAATGCCTTGCGTCTTAATGCTGATAATTTAATCACGTCATTCAACGACAGTGAACTGCCAGCTGAAGCTTTCAAGATGTCGGTGGAGGCGCTGGACTCGTGCACGCGTCCAAGGAACTAACGAACCTGACAGACGCGCTCCTGCCCAAGAACTACTCAGACATTGCCAAGGAGTGGGAGTTCAGCTACTTCGTACTCAAGTCGTCGACAAACGGTGCCATTCTTGCAGTGGCGTTGGCAAGTTTCCTATCGATTGCGTCAGTAGGCTGGGCCATGGTCTCGCCACTGCGTGTCTCGATTCTGGTGATCTTGAGTGCTGTACCCATTTCGCACACGCTGATCGGCGCGTACCTGTCATCCACGATCATGACGGCAGATTTCTGTGCCGCTCCAATGAACAACACGCTCCAACTGATCGGCGCATGTCCAGCCAACGCTTCCTGCCTTTCACAGACGCTGTAGTCACGCAAGGCTGCTCACCAGGGAGAAACTGGTCAGAGAATGAAGAGGGAGTTCCTGGATCCCATTGGCGCAGCTGTGGGCGTCATGCTCGGCTACTTCGCTGGAACTGTGAGCAACTGGGAGAGTTTTTCTCTCATTCAGACGTGGCGCTGGCCATTTCTGCTCCAGTTTTTGGCCTTGTTGCCAATTAATGTAGCCATCTTCTTTGTGCCAAAGAAACATTTGATGATTCGCAGTGAGATCAATGAGTTGGAGCCACCAGTAGCTGGGACGCCCCTGAGCACAAGATCCACCACGTATCACGACGAAGAAGACGCGTTGCTTGCCAGTGCCAGTATGCAACGTACGGAGCGCTAGACACGAGGTGTACGAACAAGGCGCATTCTCCGAGGGCGTCTACAAACTGCTGCATATCCCAGTCTTTTGTTTGATCGTGTTTGGCCTCACGACTGTCTATTTCGTCGTCACTGGCGTGCAGTATTGGAGCACCATCTTCATGATCAAGTCGCTGCATGCCTCCAAGTACCTGGTCAACGCGCTCTTCGTTGTTGTCTCCGGTACTGGCCCCATCCTGGGCGTGTTCTTTGGTGGCTGGCTCATCGATCGGTATGGAGGATACATCGGCGTGGAACAGCGTGCAAAAGCGTTAGGCATCTGCATGATCCTGGGACTTACAGCGTTCGCTATCTCGGCAGTGACGACGTTCTTCAACGACATTTACATCACTGCTGGTTTCCTGTGGCTCTTGCTCTTCTTCGGTGGCGCTATCCTGCCAGCGCATCGGGCGCTCGCCTCGTCGTTCTCTGTGATGGTCTTCAACCTGTTCGGCTACGCGCTGTCCCATGAGCGACCTGCAGATGGTGGCGGGCGCCAAGTGCTTCGTGCCGGATGAGACGCACGTGTGGCTTGCGGCTGAAGTACTGCGTGATGAGAACTCGGGCGATGGCAAGACACGTAAGATCTACTGCAAAGTGGAACTTCCCGATGGAGACTGAGGAACGCTGCGTGGACATGCTGTCGAAGAAGACAAAGGCGCTACTGATGTGCATCAACTCGAGTCGCTTCCGTACCAGAACGAGAACGTTGGTCTCGAGGGCATTGAGGACATGATCACGCTCAACTATTTGCACGAGGCCGCCATCCTGTTCAACATCAAGAAGCGCTTCCTGTGCGAGCTGCCCTACACGTACACAGGCGACATTTGCATCGCTATTAACCCCTACAAGTGGCTCCCAGACCTCTACGCTGAAGACCAGCACCTCCGCTACCTTAACCAGCCCAAAGAGGAGCTGCCCCCGCACGTGTACGCCACATCCGTCGCTGCCTACGACAACATGCGGCGCTCGGAGCGCAACCAGTCCATTCTGGTCAGTGGCGAGTCCGGTGCTGGTAAGACCGAGACCACCAAAATCCTCATGAACCACCTGGCCACCATCGCAGGCGGTCTCAACAACTCCACCATCAAGAGGATTATCGAAGTAAACCCGCTGCTCGAGTCCTTTGGTAACGCCAAAACTGTGCGTAATGACAACTCCAGTCGTTTCGGCAAGTTTACACAGCTGCAGTTTGACAAGAATGGCACGCTTGTCGGTGCCAAGTGCCGCACGTATCTTCTGGAAAAGACGCGTGTCATCCAGCACGAAGCGCCTGAACGCAACTACCATATCTTCTACCAATTGACTCGCCCGACATTGCGTCGGAGTTGCAGCTCGAATCGTCGAAGCACTATGTGTACACGGGCGACAACACGGCACGGAAGATCGAAGGACTCTCCGACAAGAAACACTTCAACCAAACACGCGAGGCCTTGGAGCTTGTGGGTCTGTCTCGCGATGACCAGCGGCCACTGTTCGAAGTGCTGGCTGGAGTGCTCCACCTTGGTGAGGTACAGCTGCAGTCAGACCCGGCAGATGATGAGAAGTCTCTCATCGCTGAGGGCGACGGTGGTGCGAATTGTGCTACTCAGATGCTTGGTGTCACGTGGGAGGCACTTCAGAAGGCTCTTTGTTCGCGTACTATGCGTGCTGTGAATGATGTGTACAGTGTACCTTTAAAAAAAGAGCTGGCGATGGACTGTCGTGACGCCCTCGCTAAAGCGATCTACTCCAACGTGTTCGACTGGCTTGTGGCTACGATCAACCAGTCGCTTGCTGATGATGCCAACATGGCCAACCACGTGGGTGTGCTCGATATCTTCGGGTTCGAGCATTTCAAGCACAACTCGTTTGAGCAGTTCTGCATTAACTTCGCTAACGAGAAGCTTCAGCAGAAATTCACACAGGATGTGTTTAAGACGGTGCAGATTGAGTATGAGGAGGAAGGAATCGTGTGGGACCACATTGAGTACGCGGATAACCAGGACGTGCTGACTGTGATTGAATCGCGCATGGGTATCATTTCGCTGCTGAACGAAGAGGTGATGCGACCGAAGGGCAGTGAGGAGTCCTTCATGTCCAAGGTGACGTCTTTGCACAAGGAT---GATATGGCGCACGTTATTGAGTTCCCTCGCACGTCTCGTACCGAGTTCCTCATCAAGCACTATGCCGCTCCTGTCATGTACGACTCGGTGGGATTCCTGGAGAAGCACAAGGACTCACTGCTGCCTGACTTGTCCGAGCTCATGCGTGGCTCATCCAAGCCGTTCATCGCCAAGCTCTTCGACCCGAAGCCGGAGCCTAAGCCGCCGTAAGC---------GTGGCGGTGCTCTGTCCATCACGACCGTGGGTACCCAATTCAAAGAGAGTCTGACGGAGCTGATGGCAACCATCAACTCGACGCGTGTACATTACGTACGCTGCATCAAGCCCAACCCGATCAA---------GAGCGCTACTGCCATGGACCAAAACATGGTGGTGTCCCAGTTGCGATGTGCCGGTGTGATTGAAGCCATTCGTATCTCCCGTGCAGCGTACCCGAACCGGCTGCAGCACACGGAGATCCTGGACAAGTTCTGGCTGTTCGTGCCGAGTGGTGGCGAGACTGCTGCAGACAAGTGCCAGCTGCTCATGGACAAGCTCAAGCTGGAATCCCCAACTCAGTATCAGATGGGTAAGACTCGTGTGTACTTCCAGCTAGGTGTGCTCGAAGAGCTTGAGGACCGCCGTAAGAAGTTCCTGGATGCCAAGGCCACGTACGTACAGAACATCATGGTTGGCTTTACGCAGCGTATCAAGTACCTCAGACAGCTAGAAGCAATCATCAAGTTCGGTTATTCGGTGTGTAATCGCTATGCGCAGATACAACACGTTCATGAAGGGCCTCATTTCAGCGCAGGCTCACTGGCGAGGCATGCAGGGCCGCAAGGTAGCTGCGGAAGTCAAGAGTAACCACAACGCTGTCATCATTCAGCGGTATATGCGTGGATTTGTCAAGCGTCACCGTTATGTCAAGATGCGCGAAATGGTCATTCGTGTGCAGGCTATGGTTCGTATGACGATTCAGCGGCCGAAATATCTAGCTGCTCTCGAGGAGAAGCGTCGTGAGGCGGACATGGCGTACCAGCTGAACAAGCTGAAGGCTGCTCTGCAGGAAGAACAGGAGCGGAATGCTCAGCT------GCAGCGTC---GCAGCAGCGTCGC--GACGGCGCCTCTGCTGCTTCGAGTGTTGTGATGGCTGATGCTGGCGGTATGATCGAGACTCTTACAGACGAGAACAAGAAGCTGCGTGAGAAGAATGAGGACATGAAGGTTACTATGAAGGGCTTGAAGGCCGAGATCGAGAAGTTCAAGAGCGACAAGGAGTTCTCTTCAGCTGGCAACCACGTCAAGGTTCGCCAACTGCAAGATACTGTGCGTGACAAGGACAAGAAGATTTCGCAGCTCGAGGCCGAAAACAAGAAGCTGACGGAGCAGATCGCCAAGCTTCACGCTGAAGGTGTTGT---TCCCGAGAAGAAACCCACGCCGAAGAAGTCGATCTTCCGCACGCTGGGCTCCAAGAAAGAGAAGAAACCTC---------------GTGAGACTGTACTGATGGACTCGCTCACCGGCTCGGACGACCTTAACCGCCCAGAGACGCGTCCGTCCGA---GCGCCACTTCCCGCGCATGC---CCAGCATGAGTGTAGGCTCT---CGCTTCTGGAACACGAACAAA---AAGGGAGAC------------AGTGGGGACGGCTTCGACGTCTCGGAGGTTGAAGGTGAGGACAGCCCCACCAACCCACGCGCAACACGATTGTCAAGAGCATGGAAGTGGGCGTGACTGGTGCCATGTCGAGTTTGAAGGGCCGCATG---------TCGGTCGTCAAGGGGATGTACGAAGCAAGCGCACAAACAGGATCGTCCTGTGCGCGAGTCGTTCAACCTGGACGCGTTGCCGGAAGTCTCTCTTCCCGTCGGCTGGGAGGCGAAGGTGTCGCGCTCGACGGGTCGTGTGTACTACGTGAACCGCAAGCTCGGCAAGTCTCAGTTTGAGCGCCCCACTCTGGCTTCTCTCAA------GGCACAGAAGCTGGCGCGGCAGAAGTCGACAGGCGGGCTGTAAGGCTGTGGTTGCTGATTTCGGCTGTGGAGATGCCAGATTGGCTGAAAGTGTGCCGAATAAAGTGCACTCGTTCGACTTGGTGTCCATTGATATTGCCGTCTACTGCTTGGCACTGATGGGAACGAGTGTTCGCGAATACGTGCGTGAAGTGTACCGTGTGCTGAAGCCTGGTGGCGTGCTGAAAGTTGCTGAAGTGAAGAGCCGCTTCGAGAGCGAATCACTCGGAGGATGGTGGCGGTGTTTCTGGCGGTGGTGCAGGACGTCTTGGTGACGCTACGCAACACGACGGTGTTTGTGGAGAGACCAAATACGTAGCTATCGTAGGCCAGAAGACAGGCGAGGCTGTGGCCGTGCCCTTCCCGGACGTAGACCGTGATCTCAAGACGCTACTGGGCCACACCACGTCCATGGTCACTCACGTGGCTGTCAACTTCGTCGCTACTACTTACAGCCGATCGTGACGAGAAGCTGCGTGTGTCGAGGTTCCCGAACGCCGCAATCATTGAAAGCTACTGCTTGGGCCACGCAGCTAGTCTCACCAAGGTAGCATGCAGTGTTGTGACCCCGGAGCTTGTGGTGTCCACGTCTATGGACAACACTTTGAAGCTCTGGCAGATGACGACAGGCAAACTTACCGTTTGCCCCAAGACGACATCGTGGCCGTGCTCGTGAACTACCAACACGTGCGGTTCTTCGAGATCGTGAGGATCCTTATCTGCAGTTGTTCTCGGTCTCTAAGGGCGAGTGCTGCGTTCAATCGCTGTGCGCGACTTCCGCAAGCTTCAAAACGACCCGCCGTCGGGCGTATCGGGCGCCCCCATGGACAACAACATCATGCTCTGGCAGGCCGTAATCTTCGGACCGGACGACACGCCATGGGAAGGCGGCACCTTCAATCTGACGCTTGAGTTTAGCGAAGACTATCCCAACAAGGCGCCGACTGTCAAATTCATCACGAAAATGTTCCACCCGAACATCTACAACGACGGCCAGATCTGCTTGGACATCCTGCAGAACCAGTGGTCTCCGATCTACGACATCTCGGCCATCCTCACGTCCATCCAGTCGCTGCTCTGCGATCCCAACCCGAACTCGCCGGCCAACTCGGAGGCCGCGCGTCTTTTCCAAGAGAACCGACGGGAGTACAACCGCCGCGTGCGCGAGATCGTCGAGCAGAGCTGGCAGGCGATCGATGGTGTCGGCCGCTAAGATGCGTGGCGCTGAGAACCGCATGAACGCCGGCCGCCCCTTCACCGCGTGGTTGGACAACGTCTCGTCGGACCGTGGTCTGTGTGGCGGTATCAACTCGGGTATTGCTAAGACCACCCGCAGACAGACCACTCGCAGGTCTTTGTGATCGGCGACAAGGCTCGCGCTCAACTGCGTCGCGACCTCGGCGACAACATCCGTGGCAACGTCACGGAGACGTACCAGTCGCCCCCGAACTTCACTGTGGCCAGCGCTATCGCTGAGGCCGTCGAGTCGGAGAGGAGTCGTCCCGTATGGCTGCTATGGAGAACGCCACTACTAACGCTGAAGACCTCACGCTAACGCTTGTGTACAACAAGGCTCGTCAGGCCCGTATTACCACGGAGCTGATTGAAATTATCTCGGGTGCCGCTTCTCTGGACGGTGACTGGCAGTGTGGTAAGCGTTCAGAGTGTGGCGCAGTTCGACGAGGCAACGGCGCGCGAGTCGACGCTATCCGTGAGTTTCTTCTGGGCCGAATTTCAGGCCTGTCGTCCCAATGGGCAGCTGGACGTGGGGTCGCGCCGTGCTGGACAAACTCGAGGGCGCCAATGTGGCCGAACTGGCCAAGCGCGTCTCGAATACCGTCTCAAGAAGCTCATCAGCGCGTCACCCGTCATGCTCTTCATGAAGGGCAATCCGACGGAGCCCAAGTGCGGCTTTAGTCGACAGATGGTGGCGCTGTTGAACGAAGAAAAGATCCAATTCGGCACCTTTGACATCCTCAATGACGACGAAGTGCGTCAAGGACTCAAGCAGTTTTCCAACTGGCCCACTTACCCTCAGCTCTATGTGAATGGCTCGCTCATTGGCGGACTGGACATCGTCAAGGAGATGAAGTCCGAGGGATCCATCGTCGAGCAACTAGGCGTCGAGGAGGCTGAAGCTGCCTTCCAAGAGAGTCTCCGTGCACTCGTCAACTCCGCTCCAGTGCTGCTCTTCATGAAAGTTCGACATCTTGTGTGGAGCTACCAACGTCAGGACCAAGACATCGCCAAGGTGGTAGCTCTCACGCCACCATCATTCCGCCGTGATATGTGCTGAAGCTATCTCGTGGAGCCCGAAACCGCATGGCTATGGCTTTCCGTGACCCTTTGCAAGAGAAAGGAGAGTTCTGCATTGTGCAACTCCTCGAATCACCTCAAAGCGCACCGTTCTGCACTCACGAGGTATATCTGCGATCGACTGGCAAGTTTACTGTGCTGCACCGAGACCACCGCAGTGAAGTTAAATCGCTCTGTGTTCACTCTCAACGTGACTCTCTGTACACTGGCGGACTTGACGGATTGCTGATTCGTTATGACCTGAAAAAGGGATGCAGCGACATCGTCATGGAGCGTCGCAAACGTATCTCCAAGATCAACGCCGTACTGGAACATCCACACAATCCGAACCTTTTAATGGTCAGCTCTGTGGAGCAGTAGACACAATTTGCTGATGCACGACCTGCAGCGCTATGACCGCAACGTGTCAGGGAGCTCCATGAGTCAGTACGTCGTGCCGCGGTGGTCGCCTGCAGGCTATCACGTCTCGTGCGGCAGCAAATGCGCTACTGCCACGGGAGCTTCTCCACGCCCACTTCAGCCACGATTGGGGCCTCATTTTTGCAGAAGCGCGTGATCGTTGGAGGGAAACTGACGCTTCAGATATGGGATACAGCTGGCCAGGAGCGCTTCCGCTCCATGGCGCCTATGTACTATCGCAACGCTAAGGCGGCTATACTAGTTTTTGACCTCCAGAGTGAGGCCAGCTTTGAAAAGATTAAGGAGTGGCTCCATCTGCAGCATCATGTGGGTGATGACATGTGGGCAACAAGAGTGACGTTTCGACTTTGCCAAAGCCCAACAGTTTGCTGATGAGATCGGTGCGCTGGTGTTTCGTACGAGCGCCAAGACGGGCGAAGGAGTGCAGTCGCTGTTTGAGAGTCTGGCGGTGCAGCTGCTTAAGAAGCACGATCAGGAGCAGCGAAGAACTTCTACGTGGTTTATGTGCCAGCGGATGAGGCCAAGGAGCTGGAAGAATGGGCAGTCGACCTGCCGCAAGACAAGGAGGCGCAGCTCGGCTGCTTGACGGAACGTCTGCGCGAGCACTTTAAGAGCGGTGCCATCCAATCAGCAGCAGCAACTTTCAAGCAGCAACTTCTGGATCAGCTGCCCAAGGGTGCTACGTTGACCGATGAAATGCTGCAGATGATGTATGGACAGTCTCGTAGACTCGATCCCGCTCGTGCTCAACACGGCCGACGTCAAGCACGTTGGAGGTCAAGGGTGATGCGTTCATTGGACGGGTGTTCGATAGACGACTTTGTGCGTATGGATTTCCGGTTGAGTGAGGTGTCTGGTGACGCACCATGGGTACAAACGGCCAAAGTGCAGAGGTCACGCAGTCGCTGGAAGACATCTTGGCGCTTGGTCAGGGTCTGGACGACGAGGACTACAAGGCCGAGCTCGAAGTGCTGCAGAACGACCCCAACTCCAACCTCTACTCGGCCGTCACCTTTGAGGAACTCAACCTGCCGGACCCGCTGCTCAAGGGCGTGTACAACATGAAATTCGCCAAGCCTTCCAAGATTCAGTCTGTGGCACTGCCACTTATCCTCGCCGATCCTCCAGAGAACCTGATCGGCCAGGCTCAGAGTGGCAGCCCACACGTGAGTTAGTGCGTCAAATTATGGCTGTAGTGAACGCTATGGGCAAGTTGTGGAGACGTTCCTGGCCATCCCCGGCAACGACCTGGCTCGTGGTGAGACTCTGAAGGCACAGGTGATCGTCGGCACGCCCGGTAAGATCGAGGGAATGATCAAGAAGAAACAACTGGACACTCGTGAAGTCAAGATCTTCGTACTGGACGAGGCTGACGTCATGGTGGCCGAAGACGGCATGCGCGAACGCTCGGTGTCTATCAAGCGATTCATCAAGAACCGTGCCTGTCAATACCTGCTCTTCTCAGCCACATACGCCGACGATGTGCGTGATTTCGCCCAGAAAATGGTGCCCGACCACAACATTATCACCGTCAAGAAGGAGAAACTCACGCTGGACGGCATCAAGCAGTTCTGGATCGACTGCAAGACCCGCGACAACAAGTTCCAAGTCTTGTCGGACATTTTCGCCATCCTGAGTATCGGCAAGTGCGTCATCTTCGTGCAGCAACGTGACACTGCCAAGGAATTGACGCGTCGGATGCGTGAGAAGGGCCACTCGGTCGGCATTTTACACGGTGCCGACATGGCCAAGGAGGTGCGCGACCAGGTCATCGACGAGTTCCGTGCCGGCACGACCAACGTGCTGATCACCACGAACGTTCTGGCGCGTGGTATCGACGTGGCGGGCGTCTCGTTGGTCGTCAACTTTGACATCCCGTTGACTCGTGACCACCGTCCCGACCCGGAGACGTATCTGCACCGTATCGGGCGTACCGGTCGGTTCGGCCGCAAAGGTTGCGCTATTAACTTCGTGTACGACAACAAGAGCAAGCAGGACCTCGCCGAGATCGAAGAGTACTACGCACGTCCGATTACGCAGGCCCCTGCAGACGATATCGAGGAACTCGAGAAGCTACTAGCTTGGGGGGCAGCAAGCGCCGTCTGGCCAAGTTCGGCAAGGAGCAGCGCAAGGGTATGCGAATCTACCCTCGCGAGCCCAAAAAGAAGCCTTCGGGCAAGGACAAGACGTACTACTACCTCAAAGACGTCATGTTCCTGGCCCACGAACCGGTTCTCGAGAAGTTCCGCGAGTTTAAATCCTTCATGAAAAAAGTCAAAAAGGCTATGGGCAAGAAGGACTACGAAGACGCCAAGCGCAAGCACGAGAACCGCCCTACCTACGAGCTGGACCATATCGTCAAGGAGCGTTACCCTCGCTTCGTGGACGCTCTAGGAGACCTGGACGACGCGCTGTGTGGCAGAACTACGTTGTCGCCACTAGAGCGCTGACCAAGGTGTTTGTGAGCATTAAGGGCATCTACTACCAAGCCAATGTGCAAGGACAGACCATCACGTGGCTCGTGCCCCACCAATTCACCCCTACCGTGGACAAACGTGTGGATGTGCGTGTTATGCTCACGTTCCTCGAGTTCTACGAGGTTCTACTCAAGTTTGTGAACGCCCAACTCTACATGCAGATCGGAGTGGCGTATCCTCCCAAGATCAACCTCCAACTGGACGCTGCGGGCGCTCAATTGGCTGCGATCGAGCTAGCAACAGAAGGAGTCCGAGAAGAGGATCAAGTTACAGTGAACAACGCAAGGAACGCAGCGAGAAGAGCTTGTTCAGTGGGCTGACGTTCTTCCTGTCGCGTGAAGTGCCTGCTGCGTGTCTGGAGCTGGTTCTACGCTCTCACGGCGCTACGTTAGGCTGGGATGGCGAGGGCTCTCCGTTCGACGAGAAGAACACGCGTATCACGCACCACCTCATGGATCGTCCGCATCAAGGACATCGCTACTTTAACCGCGAGTATGTGCAGCCTCAGTGGGTCTTTGACTCGGTGAACAACGGCATGTTGTTGCCGCTTACACGTTACAGGAAGGAGCTGGACAAGCTCAAGTCTGCGTCTCAAGTACTACGTGAGGTGGGCGCAGTGGGCGAGGAAGAAGACGACGAGAGGCCTACGCGGCTGATCTCAAGGCTGAGGTGGATGGAGACAAAAAGTCGAAGCGTGTTAAGAAGGAAGAGGAGGCTGCAGAACTCAAGGAACTGGCCAAGACTATGATGTCCAAGAAGGCCAAGCGTCTGTATGACCGCATGCAGTACGGTCTGGGTAAGAAGGCGGACAAGATCCAGAAACTCGAAGACAAACGTGCCAAGATCGAAGCAAGACCAAGAAGTTATTGGCCGGCGCACTGTCAGGTATGATCTCTGCACTGGCCTTCCAACCTCTAGGGCGCTACGCTGGCGAGGCACGTCGTCTACACTCGTTCGAGTGGCAGGCGGCGCTGGTCTCTACTTCGCTACGCTGGATCACTGCCTCAACATGTTTCCGCACACTGCAGTCAACACGTTCCTGGCTGGAGCCTTCGCTCGCACTTTTGCAGGCGGCGTCATGTCTCCTTTCACTATTGTCAAGGCACGAATGGATACCGCCGGGTACTTTCGACAGCAAACGGATGTACCGTTCTCTGGTCTGTATGTTCTCATATACACTCCACCCAGCCGATGTTGTAAAGACGCGCATGCATACGCCAGACAGTGGCCAAGATCTATAACCTTGAAGGATTGCGCGGCTTCGCTAAAGGAATCCTCCCGCGTGTGGTCAAGCGAACGCTATCCACCGCAGTCACATGGACAATCTACGAGCTACTCGTTCGACCCGTCGGGTCTGGGCCATGCCAAGGAGGCTTTTAACCTGGCCAAGGAAACGGAGCGCACCAAGCAGACGGAGAACCAGGCCAAAATTAAGGAGAATGAAGCTCTCTACAAGCAGTACGAGATCGTGCGAGTGCAGAAGGAGGGTGAGGAGCGCCGCAAGACGTTGGAGGAGGAAACCCGCCAGCACCAGCAGCGCGCGCAGTATCAGGATCAGCTTAAGCGTAAGCAGTACGCTGACCAGTTGGCTGCCCAGAAGTACATGAAGGAGCAGGAACTTAAGAAACAGGAGGAGATTCTCGCCCGCCAGGAGGCCTCCCGACGCAAGACGCTGGACTACGAAGCTGAGCTGCGTCAGAAGACTGAGCTGGCTAAGGTATCGGCAGAGACCGAGGGCCGCATCAAGCAGGAGCGTCTCAACCACGACCTGCACCTCGAGGAGGCCCGTGTGCGCGCCAAGGAATACCGTGAGACCGTGATGGAGGGTATTAAGCTTGCCGGTAACACTGTCGGGTCAGGCATCATGACTTTCGTGGATGACAAGGAGAAGCTCACGGCCACTGTGGCCTCTCTGACAGCTCTTGCTGTCGGTATTTATACCGCTAAGGTGTCCACCGGCGTTGCAGGCAAATACATTGAGGCGCGCATGGGAAAGCCGTCGCTGGTACGTGAGACGTCACGTCGTTCTGCTACGCAGGTGCTGGCCAACCCCATTCCGTCCATCAAGCGTGCGCTGCGACTGCAGAAGGCGACGGATGCGCTCGAGGGCGTCGTGCTTGAACCCAAGCTAGACGAGCGTCTGCGCAGCGTGGCTGTGTCTACGTTTAATACGAAGAAGAACCGCGCGCCATTCCGCCACTTGCTGCTTCACGGCCCGCCCGGTACGGGTAAGACTCTGTTCGCCAAGGCCCTTGCTCGTCACTCTGGCCTCGAGTACGCCATCCTCACGGGTGGTGACGTTGCCCCACTGGGTCGCGAGGGTGTGACGGAGATTCACAAGTTGTTTGACTGGGCATCGCACAGCCGTCGCGGTCTGCTACTATTCGTTGATGAGGCTGATGCGTTCTTGCAGAAGCGTAGCAACACCGTCATGAGCGAGGACATGCGTAACGCACTCAATGCCTTCCTGTACCGCACGGGCGAAGCCAGCGACAAATTCATGATCGTGTTCGCCAGTAACCAGCCGGAACAGTTTGACTGGGCCATCAACGATCGTATCGATGAGATGGTGGAGTTCCGTCTGCCTGGTTTCGACGAGCGTGTGCGCATGCTGAAGCAGTACTTCGACGACTATATTCGTGCACCGAAGAACTCGCGTGCCAAAAAGATCTACGCGATGGTGTTATCTTCACGCTGGACCCTGTGGCTAATTTTCTCGTGCCAAGACGCGCATTTTCCAGCTTAAAGCGCTGCAGGGCGTATCCAGCGAAGCGCAAGTCATCTTCGACGCACTCAACAAGAGCCTATGCGTATATACTGGGTCGTTTTTGCTGTAGTACTGGGCAACGGACTGTACACGTACTTTGCCGGCAAGGACGAGTCATACCTGGTGGAGAAGCTAAAGAGAAAAATCCCGTCCGAGTACCGCATCTCCGAGAAGTGGGACAAGTGTCTGGAGAACTTCACGCTCTACTTTGGCGCTGGCCTCGTGGCTGGCGGCCTCACATCCCTCGTGCTGGCTCGCTCTGGTGCTGGCCGCGGACTAGTCACGGGTATTGGCGTGCAGGTGCTGGGTCGAGCTGGACGACTTGCCAGCTGGCGTTCGCGGGCAACGCAAAGGCAGCACTGGACAAGACGGACAAGGCCGTGGGCGACTTGCCGGCCTTCCAGCAGCAGACGATCGTGGACTTCGTGACGGCTTCGGCCACGCAGCAGCAGCTGCAGGCCGTGCACCCGCGGCTTCTGTGAGGAGGCCAGTGTGAAGGTCTTGGAGACGTACCTGGCCGAGCAGCTGCAGAGCAAGACCGTGGACATCGACGCCAACTTGGCGCTGCTCAAGCTGTACCAGGTGTACCCGGCCACCACCAACGCCGAGAGCCAAGGTGCTGGTGAAGGGCGTAATGTCACTGCCCTCCACCTTCTTCACTGGTGTCTACTATGGTCCCGGAGAGCGTTCACGAGGACGCCAATGTGACGGCAGTGCTGCATACCGGTTTTATGCTTCAGTCATGCCTCTTCGAGGAGTTTTGGAAGGAGAGTGTGGCTTTTGCTGAGAAGGTGCCGGGCTTCCTGGAGTCGGTGCGCGCCTACATTCTCACCGCTATCAGCCGCAGCCACAGTGCGATCTCCACGGAAGTCTTTAAGGCCAAACTTAATGTGTCTGACAAGGAAGTGGCCGAGATTGTCGCCGAGAAGTGGACGCCGACAACCTAATCCAGATCAACCCCAACGAGGACAACCAGATGCAGGCCAAGAAGGTCCAGGAGAACATCGAGTTTGAGGACGTGCTTAAGGTTATCCACACTCTTTCTAGATCCAACGTCTGCGCCAGTGGTGGTGGCGTGGCTGATGCCGAAGAAGTCTGCGAGAATCTAGCCAAGCACCTTAGACTGGAGGGAGGCTCTCAGGTGTTGCCCTGCTCCACGGGCGTCATCGGATGGCGCATCCCGGTAGACTCTATGGTCGAGAACCTCCCCAAGCTCGTCACCAACCTGCAGTCCGAGTCCGTTCTACCTGCTGCCGAGGGGATTATGACCACTGATTTATACCCGAAGATCCGCTCCATGGACGTGTGTGGCGGTCGCATCGTCGGCATTGCCAAGGGTGCCGGTATGATTGAACCCAACATGGCGACGATGCTGTCCTACATCCTCACCGATCTGTCGATCCCGCGTGACGTCTTGCGTCAACTACTGACGGAGGTGGTTGGAGACACGTACAACTCACTGAGTGTGGACACGGACCAGAGCACGTCCGACACGGTGACGCTCGTTTCGTCGGACCAGATCCCGTTCGACATGAA---GCAGCTGGACGAGTTCAAGACGGCGCTGCACGAGGTGTGTCTGGGGCTGAGCGAGGACATTGTACGCAACGGTGAAGGTGCGCACCACGTGATGCGTGTGCGTGTGAGCGGCGCGCTGTCCAACGAGATGGCGAAGGGTGTCGGCAAGTCGGTGGTCAACTCGCCTCTGCTCAAGTGTGCGGTGGCTGGCAACGACCCCAATGTGGGCCGCCTCGTCATGGCTGTGGGCAAGTACATGGGAACGTACCACAAGGACGTGAACATTCAGCGTCGCATGAAGATCCGGATGGGCGGCGTTGTCATCTTCGAGAACGGCGAGATGGTGCTCAACGACGACATTGAGGCCAAGCTGGTGGCCCACATGCGCGACGCAATGCTGATTGAACCCACGCGTGGTGGACTGGACGCCAGCTCGCACAACTACCCGCCGCACGGGAAGTTTGTCGAGATTGAGATCGACCTGGGTGTTGGCTCCAAGGAGACGCAGGTGCTGGGCATCGACCTGACGCACGAGTACGTGGCCATTAATGCCGACTACCGCTCGTAATGGCTCC---GAAAAAAGGGAAAACTGCCGCCGCGTACCTGGCGTCTCTTGGTGTGGCTCCTGT---CGCCGGAGGTGAGAAGAAGGACGAC---AAGAAGAAGAAGAAAAAGGGCAAGAAGAAGCCGAAAAGAAACCCATGTCGGCTGCAGCCAAGGCCCTGA-TGGAGCGCCAGACAGCCATTAAGGCCGAGCAGGAGCGTGTCGAGCAGGAGCGTCTGGAGCAGGAGCGCATCGCCGAGGAGGAAGAGGCCAAGCGCCTGGCAGAGATTGAGCGGGAGGAGGAGATCCGACGCAAGAAGAAAGAGGCGCGCGCCGCCAAGATCGAGCGTCAGAAAAAAGAGGGTACGTACATGACCAAGGCTCAAAAGCAGAAGGCGGCCAAAGCCCAGGCTGCTCTCGAGGCCATGCAACAGGCGGGTCTCGTTCCCGATGCTGCT------GGCGAGAAGAAGAAGGTTGTGTACTCGGACCACAAGAAGAAAACCAAGAAGAACAAGAGGACGAGGAGGAGGAGGCGGAGGCCCCCGATGCCTGGGATGACGAGGACTGGGAGACCAGCGATGTTGTGGCCTCGCTGGAGGACAAACTAGCAAAAGAAGGAGCAGGAGCGTCTGCGTCAACAGGGAATCGCTCTGAAGAAGCGCCAGGAGGAGCAGGCCGAGCAGATGCGTCTGGACGCCGAGGAGGAACGTCAGCGCTCCAAGGCAAAGCTCGAGACTGATCGAAAGAAGAAGGAGGCAGCTGAGCGTCGTCATAAGCGCGAGGCCGCTGCTCGTGCCAACGTGTCCTTGGATAACCTACGATCGCCTATTTGCTGTATCATGGGGCACGTCGATACTGGTAAGACAAAGCTTCTTGACAACATTCGCAAGACCAAGGTGCAGGACGCCGAAGCTGGTGGTATTACTCAACAAATTGGTGCGACTTTCTTCCCTGTGGAGGCTATTAAGCAGAAAACGGCACGCCTTGCCGAGACAATGAAGCTGGAGTACCGCTTGCCTGGTCTCCTCGTGATTGATACGCCCGGTCACGAGTCGTTCACGAATCTGCGTACACGTGGTTCATCGCTCTGTGATATCGCCATTCTGGTGGTGGATATCATGCACGGTCTGGAGCCGCAGACTCTGGAGTCACTGCGTCTGCTGCGTCAGAAGAAGGCCCCGTTTGTGGTGGCCCTTAACAAGATCGATCGTTGTTACGGCTGGAAAACCATGCCTGACATGCCCGTGCAGGAGGCACTGAAGCACCAGAACGAGCACGTTATTCGCGAGTTCGAGGATCGCATGAAGGCGATCATTGTGGAGTTTGCTGAGCAAAAGCTGAATGCTGAGGTGTACTGGCGCAACAAGGACTTGGCCCACACGGTATCTCTCATCCCGACTAGTGCTATCTCAGGTGAAGGTGTGGCGGACCTGTTGATGATGCTGACGCGGTTGACACAGGAGCGCATGGCCAAGTCGCTGGCGTTCGTCGACATCCTGCAGTGTACCGTGCTGGAAGTGAAGGTCATTGAAGGTCTTGGTACAACGATTGATACGATTCTGGTGAACGGTACACTAGAGGAAGGTGCAACCATTGTTGTTTGTACGCTGGACGGACCAGTTGTGACAACGATCCGTTCTTTGCTGACTCCGCACCCTATGAAGGAGATCCGTGTCAAGGGCGAGTACCTTCACCACCAGAAGATGAAGGCCGCTATGGGTGTGAAGATTTGCGCTCAGGGCCTTGAGAAGGCAGTCGCCGGTACGCAGATTTACGTGGTCGGCCCCGATGATGACGTTGAGGAGCTGAAGGATTCCGTCATGTCCGATCTGACGGGTATTCTGGACTCCGTGAAGGCTACCCGCCGCGGTGTCATGGTTCAGGCGTCCACCCTCGGTGCCTTGGAGGCGCTTCTGGAGTTCTTGCGCACGTGTGACCCGCCAATTCCAGTGTCCTGCGTGAACATCGGCCCAGTCCACAAGAAGGACGTCATGCGTGCTTCGGTGCAGCTGGAGCACCAGCCTGAGTTTGCCACCATCCTGGCGTTCGATGTGAAAGTCCACAGTGACGCTACTGAGCTGGCCACAGAGCTTGGGGTGCGCATTTTCACTGCCGATATCATTTACCACCTGTTTGATCAGTTCACGGCGTACATGGACAACTTCCGCATGGCTCGTCGTGAGGAGTTCGCCGAGGTGGCTGTATTCCCGTGTGTGCTGAAGATTCTGCCCAATTGCGTTTTCAACAAGAAAGACCCGATTATTCTTGGTGTGGACGTCGAGGAAGGTATCCTCAAGATCGGTACCCCCCTCGTGGTGCCGTCCGCCGCCG---GTTTCCTGGTCGGCAAGGTGGGCTCTATTGAACGTGAGCATAAGGAGGTGGACCGCGCCAAGAAGGGAGCGTCTGTGGCCGTTCGGATAGACAACGAGGGGAGCGTCATGTACGGCCGTCACTTCGACCATAAGGACAAACTGGTGTCGCGTCTGACCCGTCAGAGCATCGACGCACTCAAGGAGAATTTCCGGGAAGACTTGCAGAAGGAGGATTGGCAGCTCGTCATCAAGCTCAAAAAGGTGTTCGATATCATTATGGAGGGATGGCTCACCAAGCGCCGCGACCACATGAATCTTATCTGGCTGGATCGCTACTTCCTCCTGGACGGCAACCAGCTCCGCTACTACCGCAAACGCGGCGACCCTGCACCTCGTGGGACCTACATTCTGAGTGATGGCTGTGTGGTGAGCCCCGTGTTCAACTCAGAGGAGAAACACAAACACCACGGACCCGTGTGGATGTTCCGCATCACTTGAAAAGTCGTACAAGAAGGACCGATTTCTGGATCTAGGAGCCAAGAGTGAGGAGAATGCCAAGGAATGGAAACGCGCGCTGGAGAACGCCGTGCGGTCGCTGCAAGAGCTGGTGGCGCATCAGAAGAAGCTCAAGAACGACGCCGAGAT---CTTCCCCACGCCCACCCAC---------GTGCCGAGGATGTTGAAGGCCGATGAAGACACGATGTCTCTCTTTAACTTGGACGCAGACAACGACAAGGCGGAGCAAGCCTGGTGGTTGGCCAGGGTGGAAGGAGGACTCCGGATCATGCAGGAATGTCCACTGGGTCCCAACGTTGCGGCCAATCTGTTCGCTCAACAAGCAGCCACAACCAACGGACTGATCTTCAGTGCGTGTGTGGGCGCAGCGCTGAGCTTTAGCAGCATGTATTCGTTCGCGAGCAACATGTTGCTGAGCTTTATGACGGCACTAGTGGCTATTGCTGTATGCTGCGCTGCAGTGACCCCTACCCAGGCGCCCGAGATCCCGAGCTTCCGTGTGTCCCAAGTAGTACATGGCTCTCCAACTGAAGTCTTCAGACTCCTCATGAACCCCAAGAGATTCCAGAGATGGGACTCGGGGACGGCGACGATGCGTGTGGTGCAGCAGCTGGATGACCACGCTGACATCGTGTACATTACGCAGCGACCGACGCACTTGTGGCCTCTGTGGCAGAAAGCTCGTGACCTTGTGCTCATGCGCTACTGGAGACGCGAAGAGGACGGATCGTACTTTGTCATGTACCAGAGCATCGAGCATCCCGAGTGTCGGGTGCGTCACAACTATGTGCGAGCGAACTTACTGGGCGGCGGCTTCGTTATCGCTCCCCAGCGTGTTCCTAGT---GGATCGATCCGAACTCTGGTTACTTACGTGCTTCGTTACGATCCAGGCGGTTG---GTCCCGTATTTACCACCAGCTTGGCATGGATGTGGATCTCGTAATGCCAATGCTGCGTAGTGTCGTGGGGATTCGTGATGAAATGAGCGCTACAGACTTCATCACGCCCAATGTCTCGATGGCGCCTGCAGCAGAGGGCGGAGACTGGACCGCAAGTGTGGA---CTCGATGGGCGTCGTCATGGGCGTACAGAAACTGCAG---ACCAGTTTCCCAGAGAAGATGTGGGGCGAGCCGGACGCTTCGCTGTTTAGTGTTCGAGGACACAACTACCTGACCGACAAGAAGAAGATCCCTTCGGCCCCCGCCATGTTCCATACTGTGGGTGTCGATCTCTTGTCCTTTGAGAGTCCTGCTGAGAGGTACAACATCTCGTCTCGATCGGACAGTGTCGGCCGCACCAG---CTCCAAATTCACGTTTGTAGTCAACATGATCATCCCAGGTCCAGAGAATGTCTGCATGGTCTTCTACTTCCACCCTGTGCGCGACAATGTGTTCGAGGACGGCTCTGGCTTCTCCGAGCTACTGAACGACTTCTTCGACGGCGACGACCAGTTCCGGAACTCTCGCTTCAAGCTCATCCCTACCGTCGTTGAAGGCAGCTTCATCATCAAACAATCGGTCGGGTCCAAGCCGACGCTGCTCGGTAACAAGCTCAAGTGTCCATATCACCGTGGAGAAAACTACTTCGAAGTGGACATCGACATCTCCTCCAACTCGGTTGCCAACACAGTGGTGGGAATGGTGCAGGGAGTGACCAAGTCGCTGGTGGTGGACATGGCCTTCCTGCTCGAAGCCCAGACCGACGAAGAGCTGCCTGAAGTCATCCTGGGAGCTGTTCGCATGCAGCACATTTCGCTTGACAACCCGCGCCGCATGCCCAAGCATGGGGAACGGGCAGCAAGGATGAGTTCACGTTCCGAGGCCTTCACCGAGGCTCTGCGTGTGAATCTGCAGCGACTCATCGTCTACGCGCGTTCGGCTGACACTGCGCTGCAGCGAGAAGTCGCCGAGAAGCTGGCCAATGAGGCCGTCAAGACCGACAAGTGCAGATTGTGGAGCTGGACGGACTGCAGCTGCTGCTACCACTAACCAAGTCGAAGGACACGGAGGTGCAGCGCTTAGCAGCCCATGCACTCGCCAATCTCTCAGTCAACTCCGAGAACCAGTCCAAAATGGCAACGGAGGGAGGCATCGACATGCTCATTGATCTTTTGAGTAGCACGAACGAGCATGTGCAGCGGCAGGCTGCAAAGGCACTTGCCAACCTCGGCGTGAATGTGGACAACAAGGAGCGAATCGCTAAGGCCGGCGGTATTAAACCGCTCATCGACCTGGCCAGCTCTCGTCAGATCGGTGTTGCCGTCGAGGCCATCGCAGCGCTGGCCAATCTGGCTGTCAATG---ATGCCAACGAGGTTGAGATCGCACGTAAAGGCGGCTTGAAGCCAATTATTGACGGAGCGCACTCCGAGTCCGTGGAGCTTCAGTCCCAAGTGGCTCGCGCACTACGCAACTTATCGGTCAATCCTGAGAACAAACAAGCAATTGTGGAGCTGGGAGGAGTGGAAGCTCTTCAGTCGCTCGTGCGTTCCACCAACGACCGGATATGCCAGCAAGCGACACGCGCCCTCGTCCCCAATGTGGTACTCACGCGATCGTCACCCTCCACCTATTG---GCCGTAGCGCCCGTCGCTCGTGTAGCTGAGGTCAAGGAGGAAGC------TATGCTCTACGACGGCGCAATGGTGGACGTTGGGTACTCGACTACAGTCAAGACAGTC---------ATTGTGCCTGCAGTTAAGCCCA---ACGGCACAACTGGAGGCGGCTTCTCGTCCATCTCCGAGCGCTTCCCGCTCTCCAAGGTGGGAAAAGTCAACTGTGGCTTGGCTTCTATTCCCATGAGTCG---CTTACTCAACG---CCACACCGGAGATGACAC---AGATGGAGCAA-------------ATGACTGTGCTAATGGAGGACGGTACGGTGGATGAAAAC---ACCATTAAAGCTGAG---CCGGAAAGTGAGCGACGTGTACCGGTACCAGGCTACTGA--CGTGGAAGAAGACGAGATGGAGCAACTTCGATGGAAAATTCATCAGCAGGTTTTCCGGCGATACCTCAAGCAAGGACAGGAGTTCGAGGAATGGGGAAAACTGGAAGTGAAAGATTCCGGGTGTTCTTCGTTCCGGGTGGACGAAGGGAGAACATCATCGCTGAGAAGAGGTTCCACGACAACGCTGTCCGAGACGCTATGGGCAAGGGGAATGCCACGTGGGTCAAGGTACGACGTGATCAGTTCGAGGCCGAAGTCCGCCAATTTTACGAGGAAACCAAACACAAACAGGAGTTGATGCTTTCGCGTCTTGAGAAAGTGTCTCCGTTCTCCCAATTTCCGATTCTTGGCAACCGATTTCTTTTGCTTCGATTGCGAGGGAAAGGAGGCAACGGAGAAGTTTGGGATGTCGTCGACTACGCCAACAACAAACAGCTCTGTGCTCTCAAGCTCTCCACTTCAATACGGCACGCTCAACGCGAACACCACACACACACTCAACCACCCTA--ACATTGTTCAGGTCGGCGAAGCACCCTTCCTTATCCGATACCAGCAACAACAGTACACTGCTTTCACTGTTGCTAGCGTCCAGTCAGATCTTCAGCAACTCATTGAGA---TGTACGAGCATTTTGATGAGGACTCTGCATACAAAGTACTCTCCCAGCTTCTGAGCGCGCTCAAGTACTTGCACGATGGACATTGCTCACTACGATCTGAAGCCGTCGAATATTCTGATCGACCAGGGAGGGTGTGTAAAGCTG------ACCGATTTTGACTTGACTCGCGACGCCAAGGCAGCCACCTCCAACTCGACCGTTGGCACCTTGCGATATCTTCCTCCTGAGTGTTTCCGCCTCAACCGCAGTGA---------CTGTGAAGCTACGGCTGAGAAGGCTGATATGTGGATGGTTGGCATCGTGTACTGCATGATGGTAACTGGAAAGCACCCGATCTGCCCAGACAAGTCGACACACGCAGAAGTGAGAGCGATGATGGCTCAATACACGGGC---GACCTTCGATATGCGCGACCAATATCTGATCTGTCTCGGTGGATCTTACAAGGCTGCTTGCATCCGGACCCGAGGTGTCGACCAACGGCCACACAGTTGCTGGTGGCGCTCCAG

Phytophthora_kernoviae TAGACTACCACCAACAAGTGGCGCGAGAGTTGGCCTCCTTCTTCCACGGCTTCTACCGCTTCGGAGCTGCCTCCAACCGCTTCGATCTGTTCCTCCACAATGTGCTCTTTGGCTCGCTCTGTATCGGAGCCGTGGTGTTCTTCATGCTTTGCGTCATCGTCGTGCGGCGCTGCATCCTGCACATCCGCAGGCGTTTGTACGACGCATGGAGATGGATTCCACGTCTGTGGAGCTGATGGCAGTGATGCTGTTGACGTTCCTAGCTGTTAGTGCTTTAGGGGGACTCTTAGGAGAAGCTGTTTGAAGACAAGTGACGCTCTACGTTTCACGGCAGACAATTTGGTGACGTCGTTCAATGAATCGGAGCTGCCTGCTGAAGCGTATCAGCTCTCAGTGGAGTCTCTGGACTCCTGCATGCTTCAAAAGAGCTGTGGAACCAGACAGACACTCTACTGCCCAAGAACTACTCGGACATTGCCAAGGAATGGGAGTTCAGCTACTTTGCGCTCAAATCTTCGACCAATGGAGCAATCCTGGCAGTGGCACTTGCTAGCTTCTTGTCCATTGCATCAGTTGGATGGGCTTTGGTGGCGCCACTGCGTGTCTCCATCTTGGTGATCTTGAGTGTTGTTCCTATCTCGCACACGCTCATTGGAGTGTATTTGGCGTCGACGATCATGACGGCGGACTTTTGTGCAGCACCGATGAACAGCACGCTGGAGCTGCTGGGTTCATGTCCTGCTAACGCTTCCTGCCTTTCCACGACTATACGACCAGACTACGCTTCCCACCACGGAGACGTTGGGCTTCGGATGAAG---GAGTTCCTGAACCCTATTGGCGCAACTGTGGGGGTCATGCTAGGCTATTTTGCAGGCACAGTCTCCAATTGGGAGAGCTTTTCGCTCATTCAGACATGGCGCTGGCCGTTTCTCGTGCAATTCTTGGCGTTGCTTCCCATTAACGTTGCCATTTTCTTCGTGCCCAAGAAACATCTCGTCATCCGCAGTGAGATCAGCGAGCTTGAACCGCCTGTGGCTGGAACGCCCATGAGCTCGAGGTCGACTACGTACCATGACGAGGAGGATGCGCTACTGGCCAGCGCCAGCATTCGACCTACGGCACACTAGACACGAGGTGTATGAGCAGGGCGCATTCTCTGATGGGGTATACGAACTATTGCACATTCCGGTGTTTTGCCTGATTGTGTTCGGCCTTACAACTGTGTACTTCGTGGTCACGGGTGTGCAGTACTGGAGCACCATCTTCATGATTAAGTCGCTTCATGCGTCGAAGTATTTGGTAAACGGTTTATTCGTCGTGGTGTCCGGGACCGGGCCCATTTTTGGTGTGTTCTTTGGTGGCTGGATTATCGATCGATACGGTGGATATATCGGCGTGGAGCAGCGGGCCAAGGCGCTGGGCATTTGCATGATTCTGGGTCTGATCGCCTTCTCTGTCTCAGCGGTGACAACGTTCTTCGATGACGTTTACATCACTTCAGGCTTCCTATGGCTGCTGCTCTTCTTCGGGGGAGCGATCCTGCCGGCACATCGCGCGCTTGCATCGTCTTTCTCCGTCATGATCTTCAATCTGTTTGGTTATGCGCTTTCGCATGAGCGACCTGCAGATGGTGGTGGGGGCCAAGTGCTTCGTGCCCGACGAAACACACGTGTGGCTCGCAGCCGAAGTGCTGCGTGACGAGAACTCTGGCGACGGCAAGACCCGCAAGATCTACTGCACCGTGGAGCTCCCCGATGGAGACGGAGGAGCGATGCATTGACATGCTATCCAAGAAGACCAAGGCGCTGTTGATGTGCACCAGCTTGAGTCTCTGCCCTACCAGAACGAAAACTTGGGGTCTGAGGGCATTGAAGACATGATCATGCTCAACTACCTGCATGAGGCTGCAATTCTCTACAATGTCAAGAAGCGTTTCCAGCAGAGTCTGCCATACACGTACACAGGTGACATCTGTATCGCCGTGAACCCGTACCAGTGGCTCCCAGACCTCTATGCTGAGGAACAACACCTCCGGTACCTCAACCAGCCCAAGGAGGAGCTCCCCCCGCACGTGTACGCCACGTCAGTGGCGGCTTACGACAATATGAGACGCTCTGAATGCAACCAGTCCATTCTGGTGAGTGGTGAGTCTGGTGCTGGTAAGACAGAGACCACCAAGATCCTCATGAATCACTTGGCTACCATTGCTGGTGGTCTGAACAACTCTACCATCAAGAAAATTATTGAGGTAAACCCCCTGCTGGAGTCCTTCGGTAATGCCAAGACTGTGCGTAACGACAACTCCAGTCGTTTCGGTAAGTTTACTCAGCTCCAGTTCGACAAAAACGGCACGCTTGTTGGCGCCAAGTGCCGCACATATCTTCTTGAGAAGACACGTGTCATCCAACACGAATCCCCTGAACGCAACTACCACATCTTCTATCAATGGAGTCCCCTGATATTGCTCCAGAGCTACTACTAGATCCGTCCAAGCACTATGTGTACACGGGTGACAATACGGTGCAGAAGATCGAGGGAATGTCTGATGCCACCCACTTCAACAAAACCCGTGAGGCACTGACATTGGTTGGCCTCTCGCACGATGACCAGAAGCCGCTGTTTGAGGTTCTTGCAGGTGTGTTGCACCTCGGTGAAGTCCTCCTGATGTCAGACCCGTCGGATGATGAGAAGTCGCTCATTACCGAGGGTGACAATGGTGCACAAGCTGCAACCCAAATGCTTGGTGTAACTTGGGAGGCGCTGCAGACTGCGCTGTGTTCTCGTACCATGCGTGCTGTCAACGATGTGTACAGTGTGCCACTGAAAAAGGAGTTGGCCATGGACTGTCGTGACGCACTCGCCAAGGCTATCTATTCAAACGTATTCGACTGGCTTGTGACTGCGATTAACCAGTCGCTTGCTGATGATGCCAACATGGCGAATCACGTGGGTGTGTTGGATATCTTCGGTTTCGAGCACTTCAAACACAACTCGTTCGAGCAATTCTGTATCAACTACGCCAATGAGAAACTTCAGCAGAAGTTCACACAGGATGTGTTCAAGACGGTGCAGATCGAATATGAAGAAGAAGGCATCGTATGGGACCACATCGAGTACGCAGATAACCAGGATGTGTTGAGCGTAATTGAGTCACGCATGGGTATCATCTCACTGCTTAATGAAGAGGTGATGCGGCCAAAAGGCAATGAAGAATCGTTCATGTCGAAGGTGACTTCACTACACAAGGAT---GACATGGCTCACGTCATTGAATTTCCCCGTACGTCTCGCACGGAATTCCTTATTAAACACTACGCTGCACCTGTCATGTACGATTCGGTTGGTTTCCTGGAGAAGCACAAGGACTCATTGCTGCCCGACCTGTCGGAGCTCATGCGTAGCTCGTCTACACCATTCATCGCTAAGCTCTTTGAACCGAAGCCGGAGCCTAAGTCGCCGAAAGC---------GCGGTGGCGCTTTGTCCATCAGAACTGTTGGAACACAGTTTAAGGAGAGCTTAACAGAGCTGATGGCCACCATCAACGCAACCAAGGTGCACTACGTGCGTTGCATCAAACCCAACCCGATCAA---------GAGCTCGACAGCCATGGACCAGGAAATGGTGGTGTCCCAACTACGTTGCGCTGGTGTAATTGAGGCCATTCGTATTTCACGTGCGGCTTATCCAAACCGCCTACAGCACACAGATATTTTGGACAAGTTCTGGCTGTTTGTGCCAAGTGGAGGTGAGACGGCGGCGGACAAGTGTCAGCTTCTCATGGACAAGCTGAAGCTTGAGACGCCTACCCAATATCAAATGGGTAAGACTCGTGTGTACTTTCAGTTGGGAGTACTCGAGGAGCTAGAGGACCGCCGTAAGAAGTTCCTCGACGCCAAGGCCTCGTACCTGCAGAACATCATGGTGGGCTTCACTCAGCGTATCAGATACTTGCGGCAGCTGGAGGCGATCATCAAGTTCCGTGATTCGGTGCGTGATTGCCATGCGGAGATACAACACATTCCACAAAGGTCTCGTTTTGGCGCAAGCCCAGTGGCGAGGTATTCAGGGGCGTAAGTTGGCTGTCGACGTGAAGAGCAACCACAACGCTGTCATCATCCAACGTTACGTGCGTGGTTTTGTTAAGCGTCACCGCTACGTCAAGATGCGAGCGATGGTTATTCGTGTACAGGCTGTGGTTCGCATGACGATCCAGCGTCCGAAGTACTTGGCTGCTCTTGAGGAGAAGCGTCGTGATGCTGACATGGCTTACCAGTTGCATAAGTTGAAGGCAGCTTTGCAGGAGGAGCAGGAGCGCAATGCGCAGCT------GCAGCGCC---GCAGCAGCGTTGC--GACTTCGCAACCGCTGCCTCGAGCGTGGTTATGGCCGATGCTGGCGGCATGATCGAGACTCTCACGGAAGAGAACAAGAAGATCCGTGACAAGAATGAGGAGATGAAGATTACGATGAAGGGATTGAAGAAAGAAATCGAGAAGTTCAAGAGCGACAAGGAATTCTCGTCGGCTGGTAACCATGTCAAGGTCCGCCAGCTTCAGGACACGGTACGCGATAAGGATAAGAAGATTAGCCAGCTCGAGGCCGAGAACAAGAAGTTGACAGAGCAACTGAACAAACTTCACGTGGATGG---TAT---TCCTGAGAAGAAAACTACGCCGAAGAAATCAATCTTCCGCACGCTGGGATCTAAGAAGGAGAAGAAGCCAC---------------GCGAGACTGTGCTCATGGATTCGCTCACCGGCTCGGACGACCTGAACCGCCGGGAGACACGCCCGTCGGA---GCGCCACTTCCCGCGCATGC---CGAGCATGAGCGTGGGCTCC---CGCTTCTGGAGCTCGACTAAGACTAAGGGCGAC------------AATGGTGATGGCTTCGACGTGTCGGAGGTAGACGGTGAGGACAGCCCCACGAACCAACGCGCAACACAATCGTGAAAAGCATGGAGGTGGGTGTGTCAGGTGCCATGTCCACCCTGAAGGGCCGGATG---------TCCGTCGTGAAGGGTATGTATGAAACAAGCGTACCAACAGGACCGCCCTGTTCGTGAATCATTCAACCTGGATGCACTTCCGGAGGTGTCGCTGCCAGTCGGTTGGGAGGCCAAGGTGTCTCGCTCCACCGGTCGCGTGTACTACGTGAACCGCAAGCTCGGTAAGTCTCAGTTCGAGCGCCCAACGCTGGCCTCGCTGAA------GGCGCAGAAGTTGGCACGTCAGAAGTCGGCCAACGCGCAGTAAGGCGGTTGTGGCAGATTTCGGTTGTGGAGATGCACGACTCGCTGAGAGTGTATCAAACAAGGTGCACTCATTCGACCTAGTGTCGCCAGATATTGGTGTGTACTGTTTGGCGCTGATGGGGAAAAATGTTCGTGAATATGTTCGTGAAGTGTATCGTGTACTCAAGCCTGGAGGAGTATTGAAGGTAGCTGAAGTCAAAAGCCGGTTTGAGAGTGAGTCGCTTGGAGGATGGTGGCGGTGTTCGTGGCGGCTGCGCATGACGTCGTGGTGACCGTCCGCAATACGACGGTGTTTGTGAAGAGACCAAATACGCAGTGGTTGTGGGCCAGAAGACGGGCGAGGCGGTAGCTGTGCCTTTTCCTGACGTGGACAGAGACCTCAAGACGTTACTGGGACATACGACGTCCATGGTCACTCACATTGCAGTGAACCTCGTCGCTGCTGTTGACGGCAGACAGAGATGAGAAGATCAGGGTGTCGAGATTCCCTACTGCTGCTATTATCGAGAGCTACTGCTTAGGTCATGCGGCGAGTCTGACCAAGGTGGCGTGCTGTGCTGTCACCCCGAAGCTGGCGGTTTCCACGTCAATGGACAACACATTGAAGCTGTGGGAGATGCAGTCGGGCAAATTGGCTGTCTGTCCCAAGACAACGTTGTGGCTGTGCTTGCAAACTACGAACATGCACGCTTCTTTGAGGTTGTAAGAGCCCGTTCTTGCAGCTATATTCTTTTACGAAGGGCGACTGCCGCGTTCAGCCGCTGTGCGCGACTTCCGCAAGCTTCAAAACGACCCGCCCTCGGGAGTCTCGGGCGCGCCAATGGACAATAACATCATGCTCTGGCAGGCCGTCATCTTTGGACCCGACGACACGCCATGGGAAGGTGGCACCTTCAACCTGACACTGGAGTTCAGCGAAGACTACCCGAATAAGGCGCCCACGGTCAAGTTCATCACGAAAATGTTCCATCCCAACATCTACAATGACGGACAAATTTGTCTGGACATCCTGCAGAACCAGTGGTCTCCAATCTACGATATTTCGGCTATCCTGACGTCCATCCAGTCGCTATTGTGCGATCCGAACCCGAATTCGCCAGCCAACTCGGAGGCAGCGCGACTCTTTCAGGAAAACAGACGGGAGTACAACCGCCGTGTTCGCGAGATCGTCGAGCAGAGCTGGCAGGCGATCGATGGTGTCGGCCGCCAAGATGCGTGGCGCTGAGAACCGCATGAACGCTGGTCGCCCCTTCACGGCGTGGATGGACAAGGTGTCTTCGGACCGTGGTCTTTGCGGTGGTATCAACTCTGGTATTGCCAAGACCACCCGCAAGCAGCCAACACTCAGATCTTTGTGATCGGAGACAAGGCTCGTGCTCAGCTCCGTCGTGACTTGGGGAACAATATCCGTGGCAACGTGACGGAGACGTTCTCGACGCCCCCGAACTTCACTGTGGCCAGTGCCATTGCTGAAGGTGTGGACAATGAGAGGAGTCCTCGCGTATGGCTGCTATGGAGAACGCCACCACGAACGCCGAGGACCTCACGCTCACGCTCGTGTACAACAAGGCCCGTCAGGCCCGTATTACCACGGAGCTGATTGAAATTATTTCGGGTGCCGCCTCCCTGGACTGTCACTGGTAGCGTCGTGAGCGTGCAGAGCGTGGCGCAGTTCGACGAGGCGACTGCGCGCGAGTCGACTCTGTCTGTAACGTTCTTCTGGGCAGAATTCCAGCTCTGCCGCCCCAACGGCCAACTGGACGTGGGGTCGTGCCGTGCTGGACAAATTAGAGGGCGCCAATGTGGCAGAACTGGCCAAGCGCGTCTGCAGTACCGTCTTAAGAAACTCATCAGTGCGTCACCTGTGATGCTCTTTATGAAGGGCAATCCAACAGAACCAAAGTGTGGCTTCAGTCGTCAGATGGTGGCGCTCCTTAACGAGGAGAAGGTTCAGTTTGGGACGTTTGATATTCTCAATGACGATGAAGTGCGTCAAGGACTGAAGCAGTTTTCCAACTGGCCAACTTTCCCTCAGCTCTACGTGAACGGTTCGCTGATCGGAGGACTGGATATTATCAAGGAGATGAAGTCGGAAGGATCAATTGTGGAACAGCTTGGTGTTGAAGAGTCAGAGGCGGCTTTCCAGGAAAGTTTGAAGGCGCTTGTTAACTCTGCACCAGTTCTTCTCTTTATGAAGCTTTGATATTCTCTGTGGAGCTACCAGCGCCAGGACCAGGATATTGCGAAGGTGGTGAGTCTTCTTCCGGCGTCTTTCCGCCAGGACATGTGCGGAGGGCATTTCGTGGAGCCCGAAATCGGTTGGCGATGGCATTCCGCGACCCGCTCCATGGGAAAGGAGAGTTCTGTATTGTGCAGCTACCCGAATCACTTCTACGAGTACTACACTGCATTCTCGAGGTATCTCCACGATCGATTGGCAAGTGGAGCGTGCTCCATCGCGACCACCGGAGCGAAGTCAAGTCCCTCTGTGTGCACTCTAAGCGCGACAGTCTTTATACGGGTGGTCTGGATGGACTGCTCATTCGATATGACATGAACAAGGGACGTACGGAAATTGTGATGGAGCGTCGCAAACCCATTTCCAAGATGAATGCAGTGCTGGAGCATCCGCATAACCCCAACCTACTTTTGGTTAGCTGCGTGGAGCAGTAAGCATAACCTGCTCCTCCACGATCTTCAGCGGTACCGTGGTGATGTGTCAGGCAGCTCAATGAGTCAGTATGTCGTGCCGCGATGGTCGCCAGCGGGCTATCACATCTCGTGCGGCAGCAAATGCGCTACTGCCACGGAAGCTTCTCCACGCCCACGTCGGCGACCATCGGGGCCTCGTTCCTGCAGAAACGCGTGATCGTGGGGGGGAAACTGACGCTGCAGATTTGGGACACGGCGGGCCAGGAGCGATTCAGGTCCATGGCGCCTATGTATTACCGCAATGCCAAGGCAGCGATCTTGGTGTTTGATCTACAGAGCGAGGCCAGCTTTGAGAAGATTAAAGAGTGGCTGCACCTGCAGCACCACGTCGGGGACGACATGTGGGCAACAAGAGCGACGTTTTGACTTTGCTAAGGTGCAGCAGTTCGCAGATGAGATTGGGGCGCTGGTATTCCGCACAAGTGCCAAGACTGGAGAGGGAGTACAGTCGCTGTTTGAAAGTTTGGCAGTGCAGCTGCTCAAGAAACATGACCAGGAGGAACGGACAGCTTCTACGTAGTGTATATCCCTTCGGACGAGAGCAAGTCTCTGGAGGAATGGACGGTGGATCTACCTGCAGACAAGGAGGCACAGCTAGGATGCCTCACAGAGCGTCTGCGCGCGCATTTCAAGGCCGGTGCCATCCAAGCGTGAGCAGAGACTTTCAAGAAGCAGCTGCTGAGCCAATTGCCCAAGGACGCCACGATAACCGACGAAATGCTGCAGATGATGCATGGATAGCCTTGTGGACTCGGTTCCTCTGGTGCTCAACACGCCAGAGGCTAAACACGTTTGAGGTCAAGGGTGATGCATTCATTGGTCGGGTCTTTGATAGATGATTTCGAGCGCAAGGACTTCCGGTTGAGTGAAGTGACAGGTGACGCGCCTTGGGTGCAAATGGGGAAGAAGCAGAGGTGACGCAGTCGCTGGAGGAGATCCTGGCGCTGGGCCAAGGACTGGATGACCAGGACTACAAGGCCGAGCTGGAGGTGCTGCAGAACGACCCCAACTCCAATCTGTACTCGGCCGTCACTTTTGAGGAGCTTAATCTACCGGAGGCGCTGCTCAAGGGTGTATACAACATGAAGTTTGCCAAGCCGTCCAAGATTCAATCCGTTGCTCTGCCATTGATTCTCGCCAATCCGCCCGAGAACCTAATTGGTCAGGCTCAGAGTGGCACCCCACCCGTGAGCTGGTGCGCCAGATTATGGCTGTGGTGAACGCCATGGGCAAATTGTCGAAACTTTTCTGGCCATCCCAGGCAACGATTTGGCTCGTGGAGAGACGCTGACGGCTCAGGCGATTGTCGGTACCCCCGGTAAAGTGGAGGGTATGATCAAGAAGAAGCAGCTGGACACGCGCAATCTCAAGATCTTTGTGCTGGATGAGGCAGATGTCATGGTCGCCGAGGACGGCATGCGCGAGCGCTCAGTGGCTATTAAGCGGTTGATCAAGAACCGCTCGTGCCAGTTCCTTCTGTTCTCGGCAACGTACGCTGATGACGTGCGCGATTTCGCCCAGAAGATGGTGCCTGACCACAACATTATTACGGTCAAGAAAGAAAAGCTCACCTTAGATGGCATCAAGCAGTTCTGGATCGACTGCAAGACTCGTGATAACAAATTCCAGGTGCTATCGGACATTTTCGCAATCCTGTCCATCGGCAAGTGCGTCATCTTCGTACAGCAGCGTGAGACGGCAAAGGAGTTGACTCGCAGCATGCGCGAGAAGGGCCACTCAGTGGGCATTCTGCATGGTGCAGACATGGCCAAGGAAGTACGAGACCAAGTTATCGATGAGTTCCGCGCGGGCACGACCAACGTGCTGATCACGACAAACGTGCTCGCTCGTGGTATCGATGTGGCAGGCGTGTCACTCGTGGTGAACTTTGACATCCCGCTCACACGAGACCACCGGCCTGACCCGGAGACGTATCTGCACCGTATTGGCCGTACGGGTCGTTTCGGCCGCAAGGGCTGTGCCATTAACTTCGTTTACGACAACAAGAGCAAGCAGGACTTAGCTGAGATTGAAGAATACTACGCGCGCCCCATCACGCAGGCTCCGGCAGACGACATTGAGGAACTCGAGAAGCTGCTTGCCTGGGGGGCAGCAAGCGCCGTTTGGCCAAGCTCGGCAAGGAACAGCGCAAAGGAATGCGAATTTACCCTCGAGAACCCAAGAAAAAGCCGTCAGGCAAGGACAAGACGTACTACTACATCAAGGACGTCATGTTCCTGGCCCACGAACCGGTTCTGGAGAAGTTCCGAGCCTTTAAAACCTTCATGAAGAAGGTCAAGAAGGCCATGGGCAAGAAGGACTACGAAGATGCCAAGCGTAAACATGAGAACCGCCCCACTTATGAGCTGGACCACATTGTCAAGGAGAGATACCCACGCTTTGTGGACGCTCTGGGAGATCTGGACGATGCGCTGTGTGGCAGAACTATGTGGTGGCCACACGTGCATTGACCAAGGTCTTTGTGAGCATCAAGGGTATTTACTACCAGGCCAATGTACAGGGGCAGACCATCACGTGGTTGGTGCCCCACCAGTTCACGCCCACCATGGACAAACGCGTGGATGTTCGTGTCATGCTGACGTTCGTGGAGTTTTACGAGGTGCTGCTCAAGTTCGTCAACGCGCAGTTGTACATGCAGATTGGAGTGGCATATCCACCCAAGATTAACCTGTCGCTAGATGCTGCTGGTGCCCAGCTCGCGGCCATTGAACTAGCAGCAGAAGGACTCGGAGAAACGCATCAAACTACAGTGAGCAGCGCAAGGAGCGCAGCGAGAAGAGTCTCTTCAGCGGACTTTGTTTCTTCTTGTCACGTGAAGTGCCTGCGGCCTGTCTGGAGCTGGTGCTTCGTTCACACGGCGCCACGCTTGGCTGGGATGGTGCCGGATCGCCTTTCGATGAGAAGAGCGATCGCATTACGCACCACATCATGGATCGTCCCCACCAGGGCCACCGTTACTTCAACCGCGAGTACGTGCAGCCGCAATGGGTGTTTGACTCCGTGAATAACGGCGTTCTGCTACCACTGGCTCGCTACCGCAAGGAGCTGGAGAAGCTCAAGTCTGCTTCACAGGTTCTGCGCGAGGTCGGCGCCGTTGGCGGCGAGGACGAGGACGGGAGTCCTACGCCGCGGATCTCAAGGCCGAGCTGGATGGCAATAAGAAGTCAAAGCGCGCCAAGAAGGAGGAGGAGGCAGCCGAGCTTAAGGAGCTAGCCAAAACGATGATGTCGAAGAAGTCGAAGCGTCTGTACGACCGCATGCAGTATGGCCTGGGCAAGAAGGCCGATAAGATCCAAAAGCTTGAGGACAAGCGCATCAAGCTGGAGGCAAAACGAGGAAGCTGTTAGCAGGCGCGTTGTCCGGCGTGATCTCGGCCCTTGCCTTCCAGCCTCTGGGGCGATCCGCTGGCGTGGTACATCCCCCACGCTCGTGCGTGTGGCTGGCGGTGCAGGTCTCTACTTCTCCACGCTGGACCACTGCATGAGCATGTTCCCTCAGACTGCGGTCAATACATTCCTTGCTGGAGCTTTTGCACGGACATTCGCAGGTGGCGTCGTGTCACCCTTCACGATCGTGAAGGCGCGGATGGATCCCTCCTGGCACTTTTGACACCAAATGGACGTGCCGTTCTCCGGGCTATATGTGCTCATTTATACGGCATCCAGCTGACGTAGTGAAGACGCGCATGCATACGACAAACAGTGGCCAAGATCTACCGCCATGAAGGTCTGCGAGGCTTCACGAAGGGGATATTACCCCGTGTGGTGAAGCGCACACTATCCACCGCCGTCACGTGGACCATCTACGAGCTACTCGTTCGACCCGTCAGGTCTGGGCCACGCTAAGGAAGCCTTCAACTTGGCCAAGGAGACGGAGCGCACCAAGCAGACGGAGAACCAAGCCAAGATCAAGGAGAACGAGGCGCTCTACAAACAGTATGAGATCGTGCGTGTGCAGAAGGAGGGTGAGGAGCGTCGCAAGACGTTGGAGGAGGAGACGCGTCAACATCAGCAGCGTGCGCAGTACCAGGACCAGCTCAAGCGAAAGCAATATGCCGACCAGCTGGCCGCACAAAAGTACATGAAGGAGCAGGAGCTCAAGAAGCAAGAGGAGATTCTTGCTCGTCAGGAGGCTTCGCGTCGCAAGACGCTCGACTATGAGGCTGAACTGCGTCAGAAGACGGAGCTGTCCAAGGTCGCGGCTGAGACGGAAGGTCGCATCAAGCAGGAGCGTCTCAATCACGACCTGCATCTCGAGGAAGCACGTGTGCGTGCTAAGGAGTACCGTGAGACGGTCATGGAAGGCATTAAACTGGCAGGAAACACGGTCGGATCGGGCATTATGGCTTTCGTGGACGACAAGGAGAAGCTCACGGCTACAGTTGTTTCGTTGACGGCGTTGGCTGTCGGTATTTACACGGCCAAGGTATCCACGGGCGTTGCTGGCAAGTACATTGAGGCGCGCATGGGCAAGCCATCGCTGGTGCGCGAGACGTCACGCCGTTCGGCGACGCAGGTTTTGGCAAATCCAATCCCGTCGATCAAGCGCGCACTGCGTCTACAGAAGGCGACTGATGCTCTGGAGGGTGTGGTGCTGGAGCCTAAGCTTGACTCGCGTCTTCGCAGCGTGGCCGTATCCACGTTCAACACGAAGACGAACCGTGCACCATTCCGCCACTTGCTGTTGCACGGCCCGCCCGGTACGGGTAAGACGCTGTTTGCCAAGGCTCTGGCGCGTCACTCTGGTCTGGAATACGCCATTCTTACGGGTGGTGATGTGGCTCCGCTTGGTCGCGAGGGTGTGACGGAGATCCACAAACTGTTCGACTGGGCGTCACACAGCCGTCGCGGTCTGCTGCTGTTCGTCGATGAGGCCGATGCATTCCTTCAGAAGCGTAGTAACACGGTGATGAGTGAGGACATGCGTAACGCGCTGAACGCCTTCCTGTACCGCACGGGTGAGGCTAGCGACAAGTTTATGATCGTGTTCGCGAGTAATCAGCCGGAACAGTTCGACTGGGCCATCAACGATCGTATCGATGAGATGGTGGAGTTCCGGCTGCCGGGCTTCGACGAGCGTGTGCGCATGCTAAAGCAGTACTTCCGCGACTACATTAGTGAACCGAAGAACTCGCGGGCGAAGAAGATTTACGCGAGGGCGTCATCTTCACGGTGGATCCCGTCGCCAACTTCCTCGTGCCAAGACACGGATCTTCCAGATGGAGACACTGCAGGGAGTTTCTGCTGAAGCTCAGACCATCTTTGATGCACTCAATAAGAGGCCATGCGTGTCTACTGGATCGTCTTTGGCGTCGTGATACTCAACGGGGTCTACACGTATTTTGCTGGCAAAGATGAGTCGTTTTTGGTGGAGAAGTTGCGGAGAAATATCCCGTCAGAGTACCGCATCTCGGAGAAGTGGGACAAGTGTCTGGAGAACTTCACACTCTACTTCGGTGCTGGTCTGGTAGCTGGCGGCCTCACGTCCCTCGTCTTGGCTCGCTCTAGTGCTGGTCGTGGACTGATTACGGGCGTGGGCGTGCAGGCGCTGGATCGAGCTGGACGACGTGCCAGATGGCGTTCGCAGGCGACGCACAGGCGGCTCTGGACAAGACGGACAAGGCCATTGGTGACTTGCCGGCCTTCAAGCAGCAAACGATCGTGGACTTCGTGACGGCCTCGGCCACGCAGCAGCAGCTGCAGGTGGTGCACCCGTGGCTTCTGTGAAGAGGCCAACGTGAAGATCTTGGAGACGTACGTGGCCGAGCAGGTGAAGAACTCGAGCGTGGACATCGACGCCAATCTGGCGCTGCTGAAGCTCTACCAGATCTACCCGGTGACGGCCAACGCCGAGAGCGACGGTGCTGGTCAAGGGCGTCATGTCGCTGCCCTCCACGTTCTTCACGGGCGTCTACGATGGTGCCGGAGAACACCCGCGAGGACACCAATGTGGCGGCTGTGTTGAACGCAGGCTTCCTGTTGCAGTCGTGCCTGTTTGAGGACTTCTGGAAGGCGGATATGACGTTCGCTGAGAAGGTGCCTGGCTTCGTGGAGTCGGTGCGCGCGTACATTCTGGGTGCCATCAGCCGTAGCCACAACGCTATTGCCACCGACGTCTTCAAGGCTAAGCTGAACATGTCGGACAAGGAAGTGGCGGACATTGTGGCTGAGAAGTGGACGAGAGTAACTTGATCCAGATCAGCCCCAATGAGGGCAACCAGATGCAGGCCAAGAAGATTCAGGAGAACATTGAGTTCGAGGACGTGCTTAAGGTCATCCACACCCTTTCTAGATCCAATGTCTGTGCAAGTGGAGGAGGTGTCGCTGATGCTGAGGAAGTCTGTGAACAACTGGCCAAGCATTTGAGACTGGAGGCTGGTTCCCAAGTCTTACCGTGCTCCACTGGAGTCATTGGCTGGAGGATTCCAGTTGACGACATGGTGGAGAACCTCCCAAAGCTGGTGTCCAATCTGCAGTCAGACTCAGTGCTGCCTGCTGCCGAGGGAATTATGACCACGGATTTGTACCCGAAGGTCCGCTCCATGGACGTGTGTGGTGGCCGCATCGTCGGTATTGCCAAGGGTGCTGGAATGATTGAGCCCAACATGGCCACCATGCTGTCGTACATCCTCACGGATCTCTCAGTACCGCGTGATGTGCTGCGCCAACTGCTGACGGAGGTGGTCGCGGACACGTACAATGCTCTCAGTGTGGACACGGACCAGAGCACGTCGGATACGGTGACGCTGGTGTCGTCGGACCAGATCCCTTTCGACATGGC---GCTGCTGGACGAGTTCAAGGCGGCTCTCAAGGAGGTGTGCCTGGGACTGAGTGAAGACATTGTGCGCAACGGTGAGGGTGCGCACCACGTCATGCGCGTGAGCGTCCGTGGCGCCGTAACCAAAGAGATGGCTAAGGGTGTCGGCAAATCCGTCGTGAACTCTCCGCTCCTCAAATGTGCTGTGGCCGGTAACGACCCGAACGTGGGTCGGGTAGTGATGGCTGTGGGCAAGTACATGGGTACCCACCACAAGGACGTGAACATTCAACGTCGTATGTCCATCCGTATGGGTGGTGTTTCAATCTTTGAGAATGGCGAGATGATTCTCAACGATGACAATGAAGTTAAGCTTGTGAAGCACATGAAAGACGCGATGCTGATTGAACCCACCCGTGGTGGCCTCGATGCCAGCTCGCACAATTACCCTCCGCACGGAAAGTTTGTCGAGATGGAGATTGACCTGGGTGTTGGATCCGAGGAGACACAGGTACTGGGCATTGACCTTACGCACGAGTATGTGGCGATCAACGCGGATTACCGCTCGTAATGGCGCC---CAAGAAGGGGAAGACCGCCGCCGCCTATTTGGCGTCCCTCGGTGTGGCTCCCAC---TGCTGGAGGGGACAAGAAGGATGAC---AAGAAGAAGAAGAAGAAAGGAAAGAAGAAACCCAAAAGAAACCGACGTCGGCAGCGGCCAAGGCGCTCG-TAGAGCGCCAGGCTGTGCTAAAGGCGGAGCAGGATCGTCTGGAGAAGGAGCGTCTGGAACAGGAGCGTATTGCTGAGGAGGAAGAGGCCAAGCGTATGGCGGAGATTGAGCAGCAAGAGGAGATCCGCCGAAAGAAGAAAGAAGCTCGTGCCGCGAAGATTGAGCGTCAGAAGAAGGAGGGGACGTACATGACAAAGGCACAGAAGCAGAAGGCAGCCAAGGCGCAGGCGGCGTTGGAAGCCATGCAGCAGGCTGGTCTTGTGCCTGAGGCAGCCGGTGAGGAGGAGAAGAAGAAGGTTGTATATTCGGATAACAAGAAGAAGGCTAAAAAGAACTGGAGGAAAAGCACGAAGAAGCAGAGGCCCCTGACGCGTGGGATGACGAGGACTGGGAGACGAGCGATGTGGTGGCCTCTCTGGAGGACAAGATAACAGAAGAAGGAACAAGAGCAGTTACGTCAGCAGGGTATTGCGCTGAAGAAGCGTCAAGACGAACAGGCTGAGCAGATGCGTGTGGAAGCTGAGGAGGAACGTCAGCGTTCGAAGGCTAAGATGGAAGCCGACCGCAAAAAAATGGAAGCTGCAGAACGCCGTCGCGAACGTGAGGCGGCTGCACGTGCCAACGTATCTGTGGATAATCTGCGTTCCCCTATTTGTTGTATTATGGGCCACGTCGACACAGGCAAAACTAAACTTCTCGATAACATTCGTAAAACCAAGGTGCAGGATGGGGAGGCTGGTGGTATTACCCAGCAGATTGGTGCGACGTTCTTCCCTGTGGATGCTATCAAGCAGAAAACGGCTCGACTCGCCGAGACCATGAAGCTTGAGTATCGCCTTCCTGGTCTATTGGTGATCGATACACCCGGTCACGAATCTTTTACGAATCTTCGAACACGTGGTTCGTCATTGTGTGATATCGCCATTTTGGTGGTTGATATCATGCACGGCCTGGAGCCCCAGACGCTGGAATCCCTACGACTGCTGCGCCAGAAGAAGGCCCCTTTCGTGGTGGCACTTAATAAGATCGATCGTTGCTATGGCTGGAAGGCGATGCCCGACATGCCCGTCCAGGAAGCTCTTAAGTATCAGAATGAGCACGTCATTCGCGAGTTTGAGGACCGTGCTAAGGCAATTATGGTGGAATTTGCCGAGCAGAAATTGAACGCTGAAATTTATTGGCGGAACAAGGATCTGGCTCGCACGGTTTCTCTCATCCCTACCAGTGCCATTTCTGGTGAAGGTGTGCCGGATCTGCTGATGATGCTCACGCGCTTGACACAGGATCGTATGGCGGAGTCGCTGGCATTTGTGGACATTTTGCAATGTACTGTCCTGGAAGTCAAGGTCATTGAGGG------------------------------------------------------------------------ACTGGATGGTCCTGTCGTGACAACAATCCGTTCATTGCTGACTCCGCATCCGATGAAGGAGATCCGTGTGAAGGGTGAATACATTCACCACCAGAAGATGAAGGCTGCGATGGGTGTGAAGATTTGTGCGCAGGGCCTTGAGAAGGCTGTGGCTGGCACGCAGATTCACGTGGTTGGCCCCGACGACGATGTGGAGGAACTGAAGGACTCTGTCATGTCTGACTTGACGGGCATTCTGGACTCTGTGAAGGCCACTCGTCGCGGTGTGATGGTTCAGGCGTCTACCCTAGGTGCACTTGAGGCCCTTTTGGAGTTTTTACGCACATGTGACCCTCCCATTCCAGTGTCGTGCGTGAACATTGGCCCCGTTCACAAGAAGGATGTAATGCGTGCGTCTGTCCAGTTGGAACACCAGCCTGAGTTTGCCACCATTCTGGCTTTCGATGTGAAAGTACACAGCGATGCCACAGAGTTGGCGACGGAGCTGGGAGTGCGCATTTTCACTGCGGACATCATTTACCACTTGTTCGATCAGTTCACAGCGTACATGGATAACTTCCGTATGGCTCGTCGCGAGGAGTTCGAGGAGGTCGCCGTGTTCCCATGCGTTCTGAAGATCCTACCCAACTGTGTCTTCAATAAGAAGGACCCGATTATTCTTGGCGTGGACATCGAGGAGGGAATCCTGAAGATTGGCACCCCTCTCGTGGTTCCATCCGCCGGGA---GCTTCTTGGTGGGCAAGGTGGGCTCCATCGAGCGCGACCACAAGGAGGTTGACCGCGCTAAGAAGGGCGCATCTGTGGCAGTGCGGATAGACAACGAGGGTAGCGTCATGTACGGACGTCATTTTGATCACAAGAACAAGCTGGTGTCACGTTTGACTCGCCAGAGTATCGATGCGCTGAAGGAAAATTTCCGGGAAGACTTGCAGAAGGAGGACTGGCAGCTCGTCATCAAGCTCAAGAAGGTGTTCGATATCATTATGGAGGGGTGGCTCACCAAGCGTCGAGACCACATGAACCTTATCTGGCTCGACAGATACTTCCTTCTGGACGGGAACCAACTCCGTTACTACCGGAAACGAGGCGATCCTGCGCCCAGAGGCACGTATATCCTCACGGATGGCTGTCTAGTGAGTCCAGTGTTTAATTCAGAGGAGAAGCACAAACATCACGGCCCCGTCTGGATGTTCCGCATCACTTGAAAAATCCTACAAAAAGGACCGATTCCTGGACTTGGGGGCCAAGAGCGAAGAAAACGCCAAGGAGTGGAAGAGAGCGCTGGAGAATGCAGTGAGATCTCTGAGAGAGTTGGTGCTGCACCAGCGAAAACTCAAAACGGACGCAGAGAT---CTTCCCCACCCCCACGCAC---------GTCCCGAGACTGCTGAAGGCCGACGAGGACACCGTCTCGCTCTTCAATTTGGATGCAGATAACGTCAAGACAGAACAAGGCTGGTGGCTGGCCAGAGTGGAAGGAGGGCTGCGTGTGATGCAGGAATGTCCTCTGGGACCAAGTGTGGCGGCCAACTTGTACGCACAACATGCAGCCACGACCAATGGACTCATCTTCAGTGCGTGTGTCGGGGCTGCACTGAGCTTCAGCAGCATGTATTCCTTCGCAAATAACATGCTGCTAAGTTTCATCACAGCGTTGGCGGCGATTGCAGTGTGTTGTGCTGCAGTGACGCCCACGCAAGCGCCTGAGATCCCGAGCTTCCGCGTCTCGCAAGTGGTTCATGGATCACCAACTGAGGTTTTCCGTCTGATCATGAACTCAAAGAGGTTTCAGAGATGGGAATCAGCAACTGCTACCATGCGTGTTGTGCAGCAGTTGGATGACCACGCGGACATCGTGTACGTCACGCAGCGACCAACACATCTGTGGCCGCTCTGGCAAAAGGCTCGGGATCTAGTGCTCATGCGCTATTGGCGGCGCGAGGAAGATGGCTCTTACTTTGTCATGTACCAGAGTATCGACCACCCCGAGTGCCGCGTGCGCCATAACTATGTGCGCGCGAATCTACTGGGCGGTGGCTTTGTTATCGCTCCGCAACGCGTCCCAAGC---GGATCCATTCGCACATTGGTAACGTACGTGCTGCGATATGACCCTGCAGGCTG---GTCGCGCATCTACCACCAACTGGGTATGGATGTGGATTTGGCCATGCCCATGCTACGTAGCGTTGTTGGTATCCGCGATGAGATGAGCGCCACAGACTTTATTACACCCAATGTCACGATACCGTCAGCC---GATGGCGGAGGCTGGCCAAAAAGTGTGGA---CTCTATGGGTGTTGTTATGGGCGTACAGAAGCTGCAG---ACCTGCCTTCCGGAGAAAATGTGGGGAGAGCCCGATGCTTCTCTCTTTAGTGTGCGTGGACACAACTACCTGAGTGACCGCAAAAAGATCCCGTCATTGCCTGCCATGTTCCATACAGTGGGCGTGGACCTGCTGTCGTTTGACAGTCCAGGCGAGAGATACAACCTCTCTGCGCGCCCAGACAGCATTGGCCGAACCAG---CACGAAGTTCACGTTTGTGGTGAACATGATCATTCCAGGTTCAGAAAACATTAGTATGGTGTTCTACTTCCAGCCTGTTCGAGACAACATATTTGAGGATGGTTCGCCCTTCTCCGAGTTGCTGAACGACTTTATTGATGGCGACGATCAGTTTCGGAACTCGCGCTTCAAGCTCATTCCTACTGTCGTAGAAGGCAGCTTCATTATTAAGCAGTCGGTCGGCTCGAAGCCCACGTTGCTCGGCAACAAGCTGAAGTGCCCGTACCACCGCGGAGACAACTACTTCGAAGTGGACATCGACATTTCGTCCAACTCAGTGGCTAACACCGTGACAGGCATGGTGCAAGGTGTCACAAAGTCGCTCGTGGTGGATATGGCCTTCTTGTTGGAAGCTCAAAGCGACGAAGAGCTGCCTGAGATCATCCTTGGTGCTGTTCGGATGCAACACATATCGCTTGACAACCCGCGCCGTATGCCGAAGCATGGGGAATGGGCAGCAAGGACGAGTTCACCTTCTGAGGCCTTCACTGAAGCGCTACGTGTGAATCTGCAGCGTCTCATCGTCTATGCGCGTTCGGCAGATACAGCGCTGCAGCGTGAGGTCGCTGAGAAATTGGCCAACGAAGCTGTGAAGACCGACAAGTGCAGATTGTGGAGCTGGACGGTCTTCAGTTGCTGCTGCCACTCACAAAGTCCAAGGATACGGAAGTGCAGCGTCTAGCGGCGCACGCTCTTGCCAACCTCTCAGTCAACTCGGAGAATCAATCCAAGATGGCAACGGAGGGGGGTATCGACATGCTGATCGACCTTCTTGGAAGCTCTAATGAACATGTCCAGCGACAAGCAGCGAAGGCGCTTGCCAATCTTGGCGTGAATGTGGATAACAAGGAGCGCATTGCGAAGGCGGGTGGTATCAAGCCACTCATCGACTTGGCCAGCTCCCTTCAAATCGGCGTCGCCGTGGAGGCCATCGCTGCCCTCGCAAACCTCGCCGTCAACG---ATGCAAATGAGGTTGAGATTGCACGCAAGGGCGGGCTGAAGCCTATCATTGACGGCGCACACTCGGAGTCCATAGAGCTGCAGTCCCAAGTGGCTCGCGCTCTGCGTAATCTTTCGGTCAACCCGGAGAACAAACAAGCAATCGTGGAGTTGGGCGGCGTCGAGGCGCTGCAATCACTGGTGCGCTCAACAAATGACCGGATATGCCAGCAAGCTACACGTGCACTCGTC-----------------------------------------------------------------------------------------------------------------ATGGTCGACGTCGAGTACTCGGCTACAGTCAAGACGGTG---------GTGGTATCTACAATTACAGCAG---GCGATGCTGTAAAC---GGCTTCTCATCCATCTCAGACCGTTTCCCTCTGCCCAAAGTGGGGAAAGTGAACTGTGGCATGGCTCCGATCCCCATGAGCCG---CTTGCTCAATT---CTACGCCAGAGATGACCC---AGGCAGATCAG-------------ATGACGGTGCTGGTGGAAGACACTACGGTGGACGATAAC---ACAGTCAAGGCTGAA---CCAGAGACTGAGCGACGTGTACAGATACCGAGCTACTGA--CGTGGAAGAAAACGAGATGGAGCAACTCCGATGGAACATTCAAGAGCAGGTTTTCCAGCGATACTTTGAGCAGGGCCAAAAGTTCGAGGAGTGGGGCAAGGTGGAAGAGCGAGACTCCATGTCTTCTTCATTCCTGGTGGACGAAGAGAAAACATCATGGCCGAAAAGCGGTTTCATGACAACGCTGTCCGAGATGCAATGGGAAAAGGAAATGCCAATTGGGTCAAAGACCGACGGTCACGTTTTGAGGAAGAAATCCGCATGTTCTACCAAGAAACTAGCCACAAGCAAGAGTTGATGCTTACGCGGCTTGAAAAGATTTCCCCGTTTTCTAAGTTTCCGATCCTTGGTAACCGCTTCCTACTTCTCCGTCTTCGAGGCAAGGGAGGCAACGGAGAAGTGTGGGATGTCATCGACTATGCCAACAACAAGCAAGCCTGTGCTCTCAAGCTGTCGACATCAATCCGACACGCGCAGCGTGAGCACCAGACTCACACTCAACCACCCCA--ACATCGTTCAGGTCGGTGACGTGCCCTTCCTCATCCGATACCAACAGCAGCAGTACACGGCATTTACAGTAGCAAGCGTCCAGTCAGACTTGCAGCATCTCATCGAGG---TGTTTGAGTACTTCGACGATGACTCAGCTTACAAGGTGATCTCGCAGCTTCTGAGTGCGCTGAAATACCTGCACGATGAGCGTTGCCCACTACGACTTGAAGCCATCCAACATCTTGATCGACCACGATGGGTGCGTGAAGCTG------ACCGACTTCGATTTGGCGCGTGATGCTAAGTCTGCTACATCAAACTCTACGGTCGGCACACTGCGATACCTTCCTCCTGAATGCTTCCGCCCAAACCGGGGTGA---------CTGCGAAGCCACAGCTGAGAAAGCAGACATGTGGATGGTGGGCATTGTGTACTGCATGATGGTCACCGGAAAGCACCCGATCTGCCCGGATAAATCCACTCTCGCGGATGTTAAGACGATGATGGCTCAGTACACCGGC---GAGCTTCGATACGCACGGCCGATTTCGGACCTATCTCGATGGATACTGCAGGGGTGTCTCCATCCTGACCCACGGTGTCGACCAACAGCCGCACAATTACTTGTGGCTCTGCAA

Phytophthora_megakarya TGGACTACCACCGTCGCGTGGCGCAGGAGGTGATTGCCTTCTTCCACTCGTTCTACCGCTTCGGCTCGGCCGCCAACCGCTTCGACCTGTTCCTGCACAACGTCCTATTCGGCTCGCTCTGCATCGGAGCGGTCGTGTTCCTCGTGCTCTGCGTCATCGTCGTGCGCCGCACCATTCTGCACATCCGTAGGCTTATGTTCGACGCACGTCGATGGATTCCACGTCGGTGGAGCTGCTCGCAGTGGCTTTGCTGACGTTCCTGGCTATTAGCGCTCTTGGGGGACTGCTGGGAGAAGCTGTCTGCACACAAGTGACGCGCTACGTGTCAACACAGACAATTTGATCACGTCATTCAACGACAGTGAACTGCCAACTCAAGCCTTCAAGATGTCGGTGGAGTCACTAGCCTTGCACACGCGTCCAAGGACTTTTGGAACAAGACGGACGCTCTGCTACCCAAGAACTACTCGGCCACTGCCAACGACTGGCAGTTCAGCTACTTCGTGCTCAAGTCTTCCACGAACGGAGCAATTCTGGCAGTAGCACTGGCCAGTTTCATGTCCATAGCGTCGGTGGGGTGGGCGATGGTTTCTCCACTGCGCGTCTCGATTCTGGTGATCTTGAGTGTTGTGCCCATTTCACACACACTGATTGGTGCCTACTTGTCGTCGACGATCATGACAGCAGACTTTTGCGTTGCCCCGATGAACAACACGCTCGCTCTGATCGACACGTGTCCTGCCGATGTGTCCTTCCTTTTGCTGACGCTGCGGCTACGCGCCGCATCTCACCAAGGAGAAATCGGTCGTCGTATGAAGAAAGAGTTCCTGGACCCCATCGGAACAACTGTGGGCGTCATGCTCGGCTATTTCGCAGGCACAGTGAGCAACTGGGAGAGTTTTTCACTCATCCAAACGTGGCGCTGGCCGTTTTTGCTACAATTTTTGGCCTTGTTGCCCATTAACGTAGCCATCTTCTTTGTGCCGAAGAAACACCTCGTGATTCGCAGTGAGATTGATGAGTTGGAACCGTCAGTTGCCGGCACCCCAATGAGTACGAGATCGACAACATATCACGATGAGGAGGATGCGTTACTGGCAAGTGCCAGTATGCTTCGTACGGTGCATTGGATACGAGGTGTACGAACAGGGAGCGTTTTCAGAGGGGGTATACGAATTGCTGCACATTCCCGTCTTCTGCTTGATCGTCTTCGGCCTCACCACCGTGTACTTCGTCGTCACGGGTGTGCAGTACTGGAGCACCATCTTCATGATCAAGTCGCTGCATGCGTCCAAGTATCTCGTCAACGCGCTCTTCGTTGTTGTCTCTGGTACTGGACCTATCCTCGGTGTTTTCTTCGGCGGTTGGCTCATCGATCGATATGGAGGATATATCGGCGTTGAGCAACGTGCTAAGGCTCTGGGCATTTGCATGATTTTGGGACTGACAGCCTTTTCGATTTCAGCGGTGACGACGTTCTTCAATGACGTCTACATCACTTCCGGTTTCCTATGGTTATTGCTCTTCTTCGGAGGAGCTATTCTACCAGCGCACCGCGCGCTCGCTTCGTCGTTCTCTGTGATGATTTTCAACTTGTTCGGATACTCGCTCTCGC---------------ATGGTGGTAGGCGCCAAGTGCTTCGTGCCGGACGAGGCGCACGTGTGGCTGGCGGCCGAAGTGCTGCGTGACGAGAACTCGGGCGACGGCAAGACCCGCAAGATCTTCTGCACCGTGGAGCTGCCGGACGGAGACGGAGGAGCGATGCGTGGACATGCTCGACAAGAAGACCAAGGCGCTGCTGCCGTGCATCAGCTCGAGTCGCTCCCGTACCAGAACGAGAACGTGGGGCCCGAGGGCATCGAAGACATGATCACGCTCAACTACCTGCACGAAGCGGCCATTCTGTACAACGTGAAGAAGCGCTTCCTCTGCGAACTGCCCTACACGTACACGGGCGACATTTGCATTGCTGTGAACCCGTACAAGTGGCTCCCCGACCTCTACGCGGAGGAGCAGCATCTCCGCTACCTCAACCAGCCCAAGGAGGAGCTCCCTCCCCACGTCTACGCCACGTCAGTCGCTGCGTACGATAATATGCGGCGATCGGAACGTAACCAGTCCATTCTGGTGAGTGGCGAGTCTGGAGCTGGTAAAACGGAGACCACCAAGATCCTTATGAACCATTTGGCTACGATTGCTGGTGGTCTCAACAACTCTACTATTAAGAGGATTATCGAGGTGAATCCACTCCTCGAGTCCTTTGGTAATGCCAAGACTGTGCGTAACGACAACTCTAGTCGATTCGGCAAGTTCACACAGCTGCAGTTCGACCAGAACGGCACGTTGGTCGGTGCCAAATGCCGTACCTATTTGCTGGAGAAGACACGTGTCATTCAACACGAAGCTCCAGAGCGCAACTACCACATCTTCTACCAGTGGATTCACCCGACATTGCACCAGAGCTACTCCTTGACTCGTCTAAGCACTATGTGTACACGGGTGACAACACAGCACAGAAGATTGAAGGACTCTCCGATGAGAAGCACTTCAACAAGACGCGCGAGGCGCTGGAGCTCGTGGGCCTGTCCCGTGACGACCAGCGACCGCTGTTTGAGGTGCTGGCTGGTGTACTTCACCTCGGTGAGGTTCAGCTCATGTCCGATCCGGCAGATGATGAAAAGTCTCTTATCACGGAAGCTGACGTCGGCGCAACTTCTGCAACTGCAATGCTTGGTGTCACATGGGAGGCGCTGCAGACTGCTCTGTGTTCGCGTACTATGCGCGCTGTGAACGATGTGTACAGCGTGCCGTTGAAGAAGGAATTGGCTATGGACTGTCGTGATGCACTCGCCAAGGCTATCTACTCCAACGTGTTCGACTGGCTCGTGTCTACGATCAACGCGTCGCTTGCCGATGACGCTCACATGACTAACCACGTTGGTGTGCTCGATATCTTTGGTTTCGAGCACTTCAAGCACAACTCGTTTGAACAGTTTTGCATTAACTTCGCCAACGAGAAGCTTCAGCAGAAATTTACTCAGGATGTGTTCAAGACTGTGCAGATTGAGTACGAAGAAGAAGGCATCGTGTGGGATCACATTGAGTATGCGGATAACCAGGACGTGCTGACTGTGATTGAATCGCGCATGGGTATCATTTCGTTGCTGAATGAAGAGGTGATGCGACCAAAGGGTAACGAGGAGTCTTTCATGTCCAAGGTGACATCGTTGCACAAGGAC---GATATGGCTCACGTTATTGAATTCCCTCGCACGTCGCGCACCGAGTTCCTCATCAAGCACTACGCAGCTCCTGTCATGTACGACTCGGTGGGCTTCCTCGAGAAACACAAGGACTCACTTCTGCCTGATCTTTCTGAACTAATGCGTGGATCATCGAAACCATTCATTGCCAAGCTCTTCGAGGAGAAACCGGATCCGGCTTGGCCGAAAGC---------GTGGTGGTGCTATGTCCATCAAGACCGTTGGTACCCAATTCAAGGAGAGTTTGACGGAGCTGATGGCTACCATCAACGCGACCCGGGTGCACTACGTGCGTTGTATTAAGCCCAACCCGATCAA---------AAGCTCGACCGTTATGGACCAGAACATGGTGGTGTCCCAGTTGCGATGTGCCGGTGTGATTGAGGCCATTCGTATTTCGCGCGCAGCGTATCCGAACCGATTGCAGCACACGGAGATCCTGGACAAGTTCTGGTTGTTTGTGCCAACTGGCGGTTCGACCGCTGAAGAGAAGTGCCAGTTGCTCATGGATAAACTGAAGCTCGAATCTCCCAGACAGTACCAAATGGGTAAGACTCGCGTGTACTTCCAACTTGGCGTGCTTGAAGAGCTTGAGGACCGCCGTAAGAAGTTCCTGGATGCCAAGGCTTCGTACCTGCAAAATATTATGGTTGGTTTCACGCAGCGTATCAAGTACCTCCGACAACTGCAAGCGATTGTCAAGTTCGGTTATTCGATGCGTAATTGCTATGCGGAGATACAACACGTTCTTGAAGGGTCTCACTAAGGCGCAAGCGCGCTGGAGAGGTATTCAGGGTCGCAAGGTGGCAGCCGATGTCAAGAGCAACCACTACGCTGTCATCATTCAGCGGTATGTGCGCGGTTTTGTCAAGCGTCACCGTTACGTTTTGATGCGCGCCATGGTCATCCGTGTGCAGGCCGTTGTTCGTATGACGATTCAGAGACCGAAGTATCTGGCAGCTCTTGAAGAGAAGCGTCGTGAGGCGGACATGGCTTATCAGTTGCAAAAATTGAAGGCCGCTCTGCAGGAAGAACAGGAGCGGAATGTGGAGCT------GCAGCGTC---GTAGCAGTGTCGC--AACGGCGCATCCGCTGTCTCGAGTGCAGTGATGGCCGACGCTGGCGGCATGATTGAGACTCTTACGGATGAGAACAAGAAGCTCCGTGAAAAGAATGGAGAGATGAAGGTCACGATGAAGGGTTTGAAGGTTGAGATCGAGAAGTTCAAGAGCGACAAGGAGTTCTCGTCGGCTGGCAACCACGTTAAGGTTCGCCAACTGCAGGACACTGTGAACGACAAGGACAAAAAGATCCACCAGCTGGAGGCCGAAAACAAGAAGCTCACGGAACAGATCGCCAAGCTTCACGCGGAGGGTGTTGT---TCCTGAGAAGAAACCCACGGTGAAGAAGTCGATCTTCCGCACTCTAGGTTCCAAGAAGGAGAAGAAGCCGC---------------GCGAGACCGTGTTGATGGACTCTCTTACTGGCTCGGATGACCTTAACCGCCGAGAGAACCGTAATTCGGA---GCGCACCTTCCCGCGCATGC---CAAGCATGAGCGTGGGTTCT---CGCTTCTGGAACACGAACAAG---AAGGGTGAC------------AACGGCGACGGCTACGATGTCTCTGAGGTTGATGGCGAGGACAGCCCTACCAACCCACGCGCAACACGATTGTGAAGAGCATGGAGGTAGGCGTGACGGGTGCCATGTCGACTCTGAAGGGCCGCATG---------TCGGTCGTGAAGGGTATGTATGAAGCAAGCGCACGAACAGGATCGGCCTGTGCGCGAGTCGTTCAACCTGGACGCATTGCCAGAGGTATCTCTGCCTGTCGGCTGGGAAGCGAAGGTGTCTCGCTCTACGGGCCGTGTGTACTACGTGAACCGTAAGCTGGGCAAGTCCCAGTTCGAGCGCCCCACGCTGGCTTCACTCAA------GGCACAGAAGCTGGCGCGCCAGAAGTCGGGTAGCGCACTG-AAGGCTGTGGTCGCGGATTTCGGCTGTGGGGATGATCGATTGGCCGAGAGTGTGTCGAACAAAGTGCACTCGTTCGACTTGGTGTCCCTGGACATTGGGGTCTACTGCTTGGCACTCATGGGAACGAGTGTACGCGAGTATGTGCGTGAAGTGTACCGTGTGCTGAAGCCTGGAGGTGTGCTGAAGGTTGCTGAGGTGAAGAGCTGCTTTGAGAAAGAGTCGTTAGGCGGATGGTGGCGGTGTTCGTAGCGGCGGTCCAAGGCGTCGTAGTGACTGTGCGTAACACTACAGTGTTCGTAGAGAGACCAAATATGCAGTTATTGTGGGCCAGAAGACGGGCGAGGCTGTGGCCGTGCCCTTCCCGGATGTGGACCGTGACATCAAGACGCTACTGGGCCACACGACATCTATGGTCACCCACGTGGCTGTGAACCTCGTCCTTACTGCTGACGGCCGACCGAGACGAGAAGCTCCGTGTATCGAGATTCCCGAACGCTGCCATCATTGAGAGCTACTGCTTGGGACATGCTGCTAGTTTGACCAAGGTGGCATGCAGTGCTGCCACTCCGGACCTCGTGGTGTCCACGTCAATGGACAACACGCTCAAGTTGTGGCAGATGACGACGGGCAAACTAGCGATCTGTCCGAAGACGATATTGTGGCGGTACTCGTGAACTACCAACATGTGAGGTTCTTTGAGATCATGAGGATCCGTTCCTGCAGTTGTTTTTGATCACAAAGGGCGTCTGTTGCATTCAACCGCTGTGCGCGACTTCCGCAAGCTGCAGAACGACCCTCCGTCGGGCGTGTCGGGTGCGCCTATGGACAACAACATCATGCTCTGGCAAGCCGTCATCTTCGGACCGGACGACACTCCGTGGGAAGGCGGCACCTTCAACCTCACGCTCGAGTTCAGCGAAGACTACCCGAACAAGGCGCCCACCGTCAAGTTCATCACCAAAATGTTCCACCCCAACATCTACAATGACGGGCAGATCTGTCTGGACATCCTGCAGAACCAGTGGTCGCCGATCTACGACATCTCGGCCATCCTCACCTCCATCCAGTCGCTGCTGTGCGACCCCAACCCGAACTCGCCAGCCAACTCGGAGGCCGCGCGCCTCTTCCAGGAGAACCGACGGGAATACAACCGCCGCGTGCGTGAGATCGTCGAGCAGAGCTGGCAGGCCATCGATGGTGTCGGCCGCCAAGATGCGTGGCGCTGAGAACCGCATGAACGCCGGCCGCCCCTTCGTGGCGTGGCTGGACAACGTCTCGTCGGACCGTGGTCTGTGCGGTGGCATCAACTCGGGTATTGCCAAGACCACCCGCAAGCAGACCACTCGCAGATCTTCGTGATCGGTGACAAGGCCCGTGCCCAACTGCGTCGCGACCTGGGCGACAACATCCGTGGCAACGTGACCGAGACGTACCAGTCGCCGCCCAACTTCACGGTGGCCAGTGCCATTGCGGAGGCTGTCGAGTCGGAGAGGAGTCGTCCCGTATGGCGGCCATGGAGAACGCCACCACCAACGCCGAGGACCTCACGCTCACGCTTGTCTACAACAAGGCTCGCCAGGCCCGTATTACCACGGAGCTCATTGAAATTATCTCGGGTGCTGCCTCCCTGGACAGTGACTGGCAGCGTAGTGAGCGTGCAGAGCGTGGCGCAGTTCGACGAGGCGACTGCGCGCGAGTCGACGCTGTCCGTGAGTTTCTTCTGGGCGGAGTTCCAGGCCTGTCGTCCCAACGGGCAGCTGGACGTGGGGCCGTGCCGTGCTGGACAAGCTGGAAGGCGCCAACGTGGCGGAGCTGGCCAAGCGCGTCTGCAGTACCGACTCAAGAAACTCATCAGTGCGTCGCCCGTCATGCTCTTCATGAAGGGCAACCCCACGGAGCCCAAGTGCGGCTTCAGTCGGCAGATGGTAGCGCTGCTCAATGAAGACAAGATCCAGTTCGGCACGTTCGACATCCTCCAGGACGATGAGGTGCGCCAAGGCTTAAAACAGTTCTCTGACTGGCCTACTTACCCCCAACTCTACGTGAACGGGTCGCTGGTCGGCGGCTTGGACATTCTCAAAGAGATGAAAGCGGAGGGCTCGATTGTCGAGCAGTTGGGCGTCGAGGAAGCTGAAGCCGCCTTCCAGGAGAGTCTCCGTGCACTGGTGAACTCAGCTCCCGTGCTGCTCTTTATGAAAGTTCGACATCTTGTGTGGAGCTACCAACGGCAAGATCAAGACATTGCGAAAGTAGTGACTTTGACACCATCGTCTTTCCGCAGGGATATGTGCTGAGGCCATCTCATGGAGCCCGAAATCGTATGGCTATGGCATTCCGTGATCCTCTGCACGAAAAAGGAGAGTTTTGCATTGTTCAACTTCTCGTATTACCTCGAAATGCACTACTTTGCATTCACGAGGCATTTCAACGATCGATTGGCAAGTTTAGTGTTCTGCACCGAGATCACCGCAGTGAGGTCAAGTCGCTGTGCGTTCACTCTAAACGTGAATGCCTGTATACGGGTGGTCTGGACGGTTTGTTGATTCGATATGACTTGCAAAAGGGGCATAGTGAAATTGTGATGGAACGCAGGAAGCGCATCTCTAAGATCAACGCTGTATTGGAGCATCCGCACAACCCAAATTTGCTTTTGGTTAGTTCCGTGGAGCAGCAAACACAATTTGTTGTTGCACGATCTGCAGCGTTATGATCCGAAAGTGAGAGGTAACTCGATGAGTCAGTATGTCGTGCCGCGGTGGTCGCCTGCGGGTTATCACGTCTCCTGCGGCAGCAAATGCGCTACTGCCACGGCAGCTTCTCCACGCCCACGTCGGCCACCATCGGGGCCTCGTTCCTACAGAAGCGCGTGATCGTTGGAGGGAAACTGACGCTGCAGATCTGGGACACGGCCGGCCAGGAGCGCTTCCGCTCCATGGCGCCCATGTACTACCGCAACGCGAAGGCAGCCATCCTGGTCTTCGACCTCCAGAACGAAGCCAGCTTTGAGAAGATTAAGGAGTGGCTTCACCTGCAGAACCACGTGGGTGACGATATGTGGGCAACAAGAGCGACGTTTCGACTTCTCCAAGGCCCAACAGTTCGCGGATGAGATCGGGGCACTGGTGTTCCGCACGAGCGCCAAGTCAGGAGAAGGAGTACAGTCGTTGTTCGAGAGTCTGGCGGTGCAACTGCTCAAGAAACACGATCAGGAACAGCGGAGAGCTTCTACGTCGTGTACGTCCCTGCGGATGAGAGCAAGGAGCTGGAAGAGTGGACGGTGAAGCTGCCGCAGGACAAGGAGGCGCAGCTGGGCTGTCTCACGGAGCGTCTGCGCGAGCACTTCAAGGGCGGAGGCATCCAAGCAGCAACAGAGACGTTCAAGCAGCAACTGCTGGCTCAGTTGCCCAAGGGCGCCACGCTCACGGACGAGATGTTGCAGATGATGCATGGACAGTCTCGTGGATTCCATCCCGCTCGTACTCAACACGCCCGAGGTCATGCACGTTGGAGGTGAAGGGCGATGCATTTATTGGCCGAGTCTTTGATAGACGACTTTGTGCGCATGGATTTCCGCTTGACTGAGGTGTCGGGTGAAGCGCCGTGGGTGCAGTCGGCCAAGTTACAGAGGTCACGCAGTCGCTCGAGGATATCCTGGCGCTAGGCCAAGGACTGGACGACGAGGACTACAAGGCCGAGCTCGAAGTGCTACAGAACGACCCCAACTCGAACTTGTACTCGGCCGTCACGTTCGAGGAGCTAAACTTACCGGATGCCCTGCTGAAGGGGGTGTACAACATGAAGTTCGCCAAACCGTCCAAGATTCAGTCCGTGGCGTTACCACTGATCCTTGCTGATCCTCCAGAGAATTTGATCGGTCAGGCGCAGAGCGGTATCCTACGCGTGAATTGGTGCGACAAATTATGGCTGTGGTGAATGCCATGGGCAAATTGTGGAGACGTTCTTGGCCATTCCTGGCAACGATCTGGCTCGTGGTGAGACTCTCAAGGCCCAGGTGATTGTCGGCACCCCTGGTAAAATTGAAGGTATGATCAAGAAGAAGCAGCTGGACACCCGTAGTGTCAAGATTTTCGTGCTGGATGAGGCTGATGTGATGGTTGCGGAAGACGGCATGCGTGAACGCTCGGTGTCCATCAAGCGCTTTATCAAGAACCGCGCTTGCCAGTACCTCCTGTTCTCGGCCACCTATGCAGATGATGTGCGTGATTTTGCTCAGAAGATGGTGCCTGACCACAACATTATCACGGTCAAGAAGGAAAAGCTCACTCTAGACGGCATCAAGCAGTTCTGGATCGACTGCAAAACGCGCGAGAACAAGTTCCAGGTGCTGTCGGATATTTTCGCCATCTTGACTATTGGCAAGTGCGTTATCTTCGTGCAGCAGCGTGAGACCGCCAAGGAGTTAACGCGTGCTATGCGTGACAAGGGACACTCGGTTGGCATCCTGCATGGTGCCGACATGGCCAAGGAAGTGCGAGATCAGGTTATCGATGAGTTCCGTGCTGGCACGACGAACGTGCTGATCACCACAAATGTGCTTGCTCGCGGTATCGATGTGGCGGGTGTGTCTCTTGTGGTAAACTTCGACATTCCGCTTACCCGCGACCACCGCCCGGACCCGGAGACGTATCTGCACCGTATCGGTCGTACAGGTCGCTTTGGCCGCAAGGGTTGCGCTATTAACTTCGTCTACGACAACAAGAGCAAGCAAGACCTCGCGGAGATTGAAGAGTACTACGCGCGGCCCATTACACAAGCTCCCGCAGACGACATCGAAGAACTCGAGAAGCTGCTGGCGTGGGGGGCAGCAAGCGCCGCCTGGCCAAGTTCGGCAAGGAGCAGCGCAAGGGCATGCGCATTTACCCGCGTGAACCCAAGAAGAAACCGTCGGGTAAAGACAAGACGTACTACTACATCAAGGACGTCATGTTCCTGGCTCATGAGCCGGTGCTGGAGAAGTTCCGAGCTTTCAAGTCTTTCATGAAAAAGGTCAAGAAAGCCATGGGCAAAAAGGACTACGAAGACGCCAAGCGCAAGCACGAGAACCGTCCGACGTACGAGCTCGACCACATTGTCAAGGAACGTTATCCTCGCTTTGTGGACGCTCTCGGAGACTTGGACGACGCTCTGTGTGGCAGAACTACATTGTTGCTACCCGTGCATTGACCAAGGTCTTTGTGAGCATCAAGGGCATTTACTACCAGGCCAATGTGCAAGGTCAGACCATCACGTGGCTAGTGCCCCATCAATTCACTCCGACAGTGGACAAGCGTGTGGATGTGCGTGTGATGCTCACGTTCTTGGAGTTCTACGAAATACTGCTCAAGTTCGTCAACGCCCAACTCTACATGCAGATCGGAGTGTCGTACCCACCGAAGATTAATCTGCAGTTGGATGCTGCTGGTGCTCAACTAGCGGCAATCGAGCTAGCAGCAGAAGGAATCCGAGAAGCGCATTAAGATACAGTGAACAGCGCAAGGAGCGCAGCGAGAAGAGCTTGTTCAGCGGTCTCACGTTTTTCCTGTCGCGTGAGGTTCCTTCGGCTTGTCTGGAGCTGGTCCTGCGCTCTCATGGAGCCACTTTGGGCTGGGATGGTGCTGGTTCTCCTTTCGATGAGAAGAACACGCGCATTACGCACCACATCATGGATCGCCCGCACCAGGGCCACCGTTATTTCAACCGTGAATACGTGCAGCCGCAATGGGTGTTCGACTCCGTCAACAACGGTATCTTGTTGCCGCTGAGCCGGTACCGCAAAGAGATCGAAAAGCTCAAGTCAGCCTCTCAGGTGCTGCGCGAGGTGGGTGCCGTCGGCGACGAAGGCGAGGACGAGAGGCTTACGCTGCCGATCTCAAGGCTGAGATGGATGGCAACAAGAAGTCGAAGCGTGTCAAGAAGGAGGAGGAGGCTGCGGAACTCAAGGAGCTGGCCAAGACCATGATGTCGAAGAAGGCCAAGCGTCTGTACGACCGCATGCAGTACGGTTTGGGCAAGAAGGCCGACAAGATTCAGAAGCTCGAGGACAAGCGTGTCAAGCTGGAGGCAAGACCAAGAAGCTGCTGGCTGGCGCGTTATCCGGTATGATCTCCGCACTTGCCTTCCAGCCTCTAGGGCGCTGCGCTGGCGTGGCACTTCGCCCACTCTCGTGCGTGTGGCGGGTGGTGCCGGACTCTACTTCGCCACGTTGGATCACTGCCTCGACATGTTCCCACACTCGGCAGTCAACACGTTCTTGGCCGGAGCCTTCGCGCGCACATTTGCGGGTGGCGTCATGTCGCCGTTCACTATTGTGAAGGCCAGGATGGATCCCGCCCGGTACCTTCGACAGCAAAAGGATGTACCGTTCTCCGGACTTTATGTGCTCATTTACACGACATCCGGCAGATGTGGTGAAGACTCGCATGCATGAGGCAAACGGTGGCCAAGATCTACCATCATGAAGGTCTGCGCGGTTTCGCCAAAGGAATCATTCCTCGTGTGGTGAAGCGGACGCTGTCGACTGCTGTCACTTGGACGATCTACGAGCTACTCGTTCGACCCGTCAGGTCTCGGTCACGCCAAGGAAGCGTTCAATCTCGCCAAGGAGACAGAGCGCACCAAGCAGACGGAGAACCAGGCCAAGATCAAGGAGAACGAGGCGCTCTACAAGCAGTACGAGATCGTGCGCGTGCAGAAGGAGGGCGAGGAGCGTCGCAAGACGCTCGAGGAGGAGACGCGTCAGCACCAGCAGCGCGCGCAGTACCAGGACCAGCTCAAGCGCAAGCAGTACGCGGACCAGCTGGCGGCTCAGAAGTACATGAAGGAGCAGGAGCTCAAGAAGCAGGAGGAGATTCTGGCTCGTCAGGAAGCTTCGCGTCGTAAAACACTCGACTATGAAGCGGAATTGCGTCAGAAGACTGAGCTGGCTAAGGTGTCGGCGGAGACTGAGGGACGTATCAAGCAGGAACGACTCAACCACGACCTCCACCTTGAGGAAGCTCGTGTGCGTGCGAAGGAGTACCGTGAGACGGTGATGGAAGGCATCAAGCTGGCTGGTAACACTGTCGGCTCGGGTATTATGACTTTTGTGGACGACAAGGAGAAACTCACGGCCACAGTGGCCTCGTTGACCGCTCTGGCTGTCGGTATCTACACTGCTAAGGTCTCTACTGGTGTTGCGGGCAAGTACATTGAAGCGCGCATGGGCAAACCGTCACTTGTGCGTGAGACCTCGCGTCGCTCAGCTACACAAGTGCTGGCGAACCCCATCCCCTCTATTAAGCGTGCCCTGCGTCTGCAGAAGGCGACCGATGCACTCGAGGGTGTCGTGCTTGAGCCCAAACTCGACGAGCGTCTCCGTAGCGTGGCTGTGTCCACGTTCAACACGAAGAAGAACCGCGCGCCGTTCCGCCACTTGCTGCTGCACGGTCCGCCTGGTACCGGTAAGACGCTGTTCGCCAAGGCTTTGGCGCGTCACTCTGGACTGGAGTACGCTATTCTCACGGGTGGTGACGTCGCCCCGCTTGGCCGTGAGGGTGTGACCGAGATCCACAAGCTCTTCGACTGGGCTTCGCACAGTCGTCGTGGTCTGCTGCTGTTTGTCGACGAGGCTGATGCTTTCCTGCAGAAGCGTAGCAACACCGTCATGAGCGAGGACATGCGTAACGCGCTGAACGCCTTCCTGTACCGCACGGGTGAGGCCAGCGACAAGTTCATGATCGTGTTCGCCAGTAACCAGCCGGAGCAGTTCGACTGGGCCATTAACGATCGTATTGATGAGATGGTGGAGTTTCGCCTGCCGGGTTTCGATGAGCGTGTGCGCATGCTGAAGCAGTACTTCGACGATTACATTCGTGCGCCGAAGAACTCGCGTGCCAAGAAGATTTACGCGAGGGTATCATCTTCACGCTGGACCCTGTGGCCAAGTTCCTCGTGCCAAGACACGTATCTTCCAGCTCGATGCACTGCAGAACGTGTCCGGTGAAGCTCAAGCCATCTTCGACGCTCTCAACAAGAGCCCATGCGCATCTACTGGGTTATCTTCAGCGTGGTGCTGCTCAACGGATTGTACACGTACGTTGCTGGCAAGGACGAATCGTTTCTGGTGGAGAAACTCAAAAGAAAAATCCCGTCCGAGTACCGCATCTCCGAGAAGTGGGACAAGTGTCTGGAGAACTTCACGCTCTACTTCGGCGCTGGGCTCGTGGCTGGCGGCCTCACGTCCCTCGTGCTGGCGCGTTCTGGTGCTGGGCGTGGGCTGGTCACGGGCATTGGCGATCTGGCGCGGGATCGAGCTGGACGACGTGTCAGCTGGCGTTCGCAGGCGACGCACAGGCAGCTCTGGACAAGACGGACAAGGCTGTGGGCGACTT---GGCGTTCCAGCAGCAGACGATCGTGGACTTCGTGACGGCCTCAGCCACGCAGCAGCAGCTGCTGGCGGTACACCCGCGGCTTCTGCGAGCAGGCCAACGTGGCCGTGTTGGAGACGTATCTGGCCGAGCAGGTGAAGAACTCGACGGTGGACATTGACGCCAACCTGGCGCTGCTCAAGCTGTACCAAGTGTACCCGGCTACGGTCAACGCGGAGAGCCAAGGTGTTGGTGAAGGGCGTCATGTCGCTTCCCTCCACGTTCTTCACTGGCGTCTACCATGGTGCCGGAGAGCATTCGTGAGGACGCTAATGTGACGGCGGTGCTGCACACCGGCTTCATGCTGCAGTCGTGTCTGTTCGAGGACTTCTGGAAGGAGAACGTGACCTTCGCGGAGAAGGTGCCAGGCTTCCTGCAGTCGGTGCGCGCCTACATTCTGACGGCAATCAGCCGCAGCCACAGTGCTATCTCTACGGGCGTGCTCAAGGCCAAACTTAATGTGTCGGACAAGGAAGTGGCTGACATTGTCGCCGAGAAGTGGACGCCGGTGACTTGATCCACATCAACCCCAACGAGGACAACCAGATGCAGGCCAAGAAGGTCCAGGAGAACATTGAGTTCGAGGACGTGCTCAAGGTCATCCACACCCTTTCTAGATCCAACGTCTGCGCCAGCGGTGGCGGCGTGGCCGATGCCGAGGAGGTTTGCGACAATTTGGCCAAGCATCTGCGTCTGGAGGGAGGCTCCCAAGTGCTGCCGTGCTCCACGGGTGTCATCGGATGGCGCATCCCCGTGGATGCCATGGTGGAAAACCTCCCGAAACTCGTCACCAATTTGCAGTCCGACTCGGTGCTGCCTGCTGCCGAGGGAATCATGACCACCGATCTGTACCCGAAGGTCCGCTCCATGGATGTGTGCGGTGGGCGTATCGTCGGTATCGCCAAGGGTGCTGGCATGATTGAGCCCAATATGGCTACGATGCTGTCGTACATTCTCACTGACTTGTCGATTCCCCGTGACGTGCTACGTGAGTTGCTCACGGAGGTGGTTGGAGACACATACAACTCGCTCAGTGTCGATACGGACCAAAGTACGTCCGACACGGTGACGCTCGTGTCATCGGACCAGATTCCGTTCGATATGGC---GAAGCTGGACGAGTTCAAGAAAGCTCTGCACGAGGTCTGCCTCGGACTGAGCGAAGACATTGTGCGCAATGGTGAAGGTGCACACCACGTTATGCGAGTGCGTGTGAGTGGTGCAGTGGATAAGGAAATGGCCAAGGGCGTCGGGAAGTCGGTGGTCAATTCGCCTCTACTCAAGTGTGCAGTAGCTGGCAACGACCCGAATGTGGGACGTCTGGTCATGGCTGTCGGCAAGTACATGGGTCTCCACCACAAGGATGTGAACATTCAGCGTCGCATGAAGATCCGTATGGGTGGCGTGGTCATTTTCGAGAACGGCGAAATGGTGCTCAACGACGGTATTGAAGCTAAGCTTGTGGCACACATGCGCGACGCCATGCTGATTGAGCCCACCCGAGGTGGTCTGGACGCCAGTTCGCACAACTTCCCGCCACACGGAAAGTTTGTTGAGATCGAAATTGACTTGGGCGTTGGATCTGAGGAAACGCAAGTTTTGGGCATTGACCTTACGCATGAGTACGTGGCAATTAACGCCGACTACCGCTCG--ATGGCTCC---GAAAAAAGGGAAGACCGCCGCTGCGTACCTGGCGTCTTTGGGTGTTGCCCCTGC---CGCTGGAGGCGATAAGAAGGATGAC---AAGAAGAAGAAGAAAAAGGGCAAGAAGAAACCCGCAAGAAGCCTGTGTCAGCTGCAGCAAAGGCGCTGA-TGGAGCGTCAAGCGGCCATGAAGGCCGAGCAGGAGCGTCTCGAGCAGGAGCGACTGGAGCAGGATCGCATTGCCCAGGAGGAGGAAGCCAAGCGTCTGGCGGAGATTGAGCGCGAGGAAGAGCTCCGCCGCAAGAAGAAAGATGCGCGCGCAGCTAAAATCGAGCGTCAGAAGAAGGAGGGAACGTACATGACCAAGGCTCAGAAGCAGAAGGCAGCCAAGGCTCAGGCTGCTCTCGAAGCCATGACCCAGGCGGGTCTCGTGCCTGAAGTTGGT------GGTGAGAAGAAGAAGATCGTGTACTCGGACAACAAGAAGAAGAACAGGAAAAACCGGAGGAAGAG---GAGGAGGCCGAAGCCCCTGACGCGTGGGATGACGAAGACTGGGAAACGAGTGATGTCGTGGCCTCGCTGGAGGATAAGTTAGCAGAAGAAGGAGCAAGAGCGTCTGCGACAGCAGGGTATTGCGCTGAAGAAGCGTCAGGAGGAACAAGCCGAACAGATGCGACTGGAGGCTGAAGAGGAACGCCTGCGCTCTAAAGCTAAGCTTGAAGCCGAGCGCAAAAAGAAAGAAGCAGCGGAACGTCGTCGAGTACGCGAGGCTGCTGCTCGTGCTAACGTTTCCCTGGACAACCTGCGTTCGCCGATTTGCTGCATCATGGGTCACGTCGATACTGGTAAGACTAAGCTTCTTGACAATATTCGTAAAACCAAGGTGCAGGACGCCGAAGCCGGTGGTATTACCCAGCAGATTGGCGCCACCTTCTTCCCTGTGGACGCTATCAAGCAGAAGACCGCACGTCTTGCTGAAACGATGAAGCTGGAGTACCGGTTGCCCGGTTTGCTTGTGATTGATACACCTGGTCACGAGTCATTCACAAACTTGCGTAATCGTGGTTCATCGCTGTGTGATATTGCCATTTTGGTGGTAGATATCATGCACGGTCTGGAGCCGCAGACTTTAGAGTCGCTCCGCCTGCTACGCCAAAAGAAGGCCCCGTTTGTTGTGGCGCTTAACAAGATCGATCGTTGTTACGGCTGGAAGACCATGCCCGACATGCCCGTCCAGGAGGCGCTCAAGCACCAGAACGAGCATGTCATCCGCGAGTTTGAGGACCGCATGAAGGCGATCATTGTGGAGTTTGCTGAGCAGAAGCTGAACGCTGAAGTATACTGGCGCAACAAGGACTTGGCGCACACTGTCTCGTTGATTCCTACCAGCGCCGTCTCGGGTGAAGGTGTGTGTGATCTACTGATGATGCTGACGCGATTGACGCAGGAGCGTATGGCTAAGTCGCTGGCGTTTGTTGACATCTTGCAGTGTACAGTCCTGGAAGTGAAGGTCATTGAGGGTCTTGGTACAACAATTGATACAATTCTAGTGAATGGTTCACTGGAAGAAGGCGCAACTATTGTAGTGTGTACACTGGATGGACCTGTTGTTACAACTATCCGATCGTTGCTGACCCCGCACCCCATGAAAGAAATCCGTGTCAAGGGAGAGTATCTTCATCACCAAAAGATGAAAGCCGCTATGGGTGTGAAGATTTGCGCACAGGGTCTTGAGAAGGCTGTTGCTGGTACTCAGATTTACGTTGTGGGTCCCGATGACGATGTGGAGGAGCTGAAGGACTCTGTCATGTCCGACTTGACGGGTATCCTGGACTCGGTGAAGGCTACCCGCCGTGGTGTGATGGTGCAGGCTTCGACTCTGGGTGCACTAGAGGCGCTTCTGGAGTTCTTGCGCACGTGTGACCCGCCCATACCGGTGTCTTGTGTGAATATTGGACCTGTGCACAAGAAGGACGTCATGCGTGCCTCTGTGCAGCTAGAGCACCAGCCTGAGTTTGCCACCATCCTGGCGTTCGACGTGAAGGTTCACAGCGATGCGACTGAGCTGGCCACAGAACTTGGTGTGCGCATTTTCACGGCCGACATCATTTATCACCTGTTTGACCAGTTCACGGCATACATGGACAACTTTCGTATGGCTCGCCGCGAAGAGTTCGCTGAGGTGGCTGTGTTCCCGTGTGTATTGAAGATCCTGCCGAACTGCGTATTCAATAAGAAGGACCCGATCATTCTCGGTGTCGACGTCGAAGAGGGTATCGTCAAGATCGGCACTCCACTCGTGGTACCGTCCGCTGGTG---GTTTCCTGGTTGGCAAAGTGGGCTCCATCGAGCGCGAACACAAGGAGGTGGATCGTGCTAAGAAGGGTGCGTCTGTGGCCGTACGGATAGACAACGAGGGAAGCGTCATGTATGGTCGCCACTTCGACCACAAGAACAAGCTGGTGTCGCGCCTGACGCGCCAGAGCATTGACGCGCTGAAGGAAAATTTCCGGGAAGACTTGCAGAAGGAGGACTGGCAGCTCGTCATCAAGCTCAAGAAGGTGTTCGATATCATTATGGAAGGCTGGCTCACGAAGCGCCGTGACCACATGAACCTCATATGGTTGGATCGATATTTCTTGTTGGACGGCAACCAGCTCCGCTATTACCGTAAACGCGGCGATCCAGCCCCTCGAGGGACCTACATTCTCACTGACGGCTGCGTGGTGAGTCCCGTGTTTAATTCCGAGGAGAAACACAAGCACCACGGTCCTGTGTGGATGTTTCGCATCACTTGAAAAGTCCTACAAGAAGGACCGGTTCCTAGATCTTGGCGCCAAGAGTGAAGAGAACGCCAAGGAGTGGAAACGCGCACTGGAGAACGCCATCCGCTCGCTTCGGGAGCTCGTGGCGCACCAGAAGAAGCTCAAGAATGACGCCGAGAT---CTTCCCCACTCCCAACAAC---------GTCCCGAGGATGTTGAAGACGGACGAAGACACAGTGTCGCTCTTTAATCTTGATGCTGACAACGTCAAAGCGGAGCAGGCCTGGTGGCTGGCGAGAGTGGAAGGAGGACTCCGGATCATGCAGGAATGCCCACTGGGACCAAATGTTGCTGCCAATTTGTACGCACAACATGCTGCGACCACGAACGGACTGATCTTCAGTGCGTTTGTGGGTGCTGCACTGAGCTTTAGCAGCATGTATTCGTTTGCGAACAACATGTTGCTGAGCTTCGTCACGGCGCTCATGGCGATTGCGGTGTGTTGTGCGGCGGTGACGCCTACGCAGGCGCCGGAGATCCCGAGCTTCCGTGTTTCGCAGGTGGTGCATGGATCCCCGACTGAGGTCTTTAGACTCATCATGAACCCCAAGAGATTCCAGAGATGGGATTCAGGAACGGCAACCATGCGAGTGGTGCAGCAGCTGGATGACCACGCTGATATTGTGTATGTGACACAGCGACCAACGCACTTGTGGCCGCTGTGGCAAAAAGCTCGTGACCTGGTGCTCATGCGCTACTGGCGACGTGAAGAGGATGGATCGTACTTTGTCATGTACCAGAGTATCGAACACCCTGAGTGTCGTGTCCGCCACAACTACGTGCGAGCCAATCTACTGGGTGGAGGCTTCGTCATTGCGCCACAGCGTGTTCCTAGT---GGTTCGATTCGCACACTGGTTACTTACGTGCTTCGATACGATTCGGGAGGCTG---GTCTCGCATTTATCACCAGCTCGGCATGGATGTGGACTTGGTTATGCCCATGCTGCGCAGTGTTGTTGGTATTCGCGACGAAATGAGTGCTACGGACTTCATCACGCCTAACGTCTCGGTTG---CCGCTGGAGAGGGCGGAAGTTGTTCAGGAAGTGTGGA---TTCAATGGGCGTTGTCATGGGAGTACAGAAACTCCAG---ACTAGTCTGCCTGAGAAGATGTGGGCCGAACCTGATGCTTCTCTTTTCAGTGTTCGCGGTCACAACTACCTCACCGACAAGAAAAAGATTTCTTCAGCGCCGTCAATGTTCCATACTGCCGGTGTGGATCTCCTATCGTTCGAGAGTGCAGCGGAGAGATACAACATCTCGTCTCGTGCGGATAGCATCGGCAGCACCAG---CTCCAAATTCACATTTGTGGTGAACATGATTATTCCGGGTCCTGAGAACACATGCATGGTATTCTACTTCCAGCCCCGACGCGACAATGTTTTCGAGGACGGTTCACCTTTCTCCGAGCTGCTGAACGATTTTTTCGATGGCGACGATCAGTTTCGGAACTCGCGCTTCAAGCTCATTCCAACTGTCGTCGAAGGAAGCTTCATTATCAAGCAGTCGGTCGGCTCGAAGCCGACGTTGCTCGGCAACAAGCTCAAATGTCCATACTACCGCGGAGAAAACTACTTTGAAGTGGACATTGATATTTCGTCCAACTCGGTTGCCAACACCGTCGTGGGTATGGTGCAAGGTGTGACCAAATCACTAGTGGTAGACATGGCATTCCTTCTCGAGGCCCAAACTGATGAAGAGCTGCCTGAAATCATCCTCGGCGCTGTTCGCATGCAGCATATTTCGCTTGACAATCCCCGCCGCATGCCAAAGCATGGGGAACGGGCAGCAAGGACGAGTTCACTTTCTGAGGCCTTCACCGAAGCACTGCGCGTGAACCTGCAGCGACTGATCGTCTACGCGCGCTCGGCGGACACGGCGCTGCAGCGAGAAGTCGCCGAGAAGTTGGCCAATGAGGCTGTGAAGATCGACAGGTGCAAATTGTGGAGCTGGACGGACTGCAACTGCTGCTGCCGCTCACCAAGTCCAAGGACACGGAGGTGCAACGTCTTGCAGCACACGCACTGGCCAATCTCTCAGTCAACTCCGAGAACCAATCCAAGATGGCGACGGAGGGAGGCATCGACATGCTCATTGACCTCTTGAGCAGCACCAATGAGCACGTGCAACGCCAGGCTGCTAAGGCATTGGCGAATCTCGGTGTTAACGTGGATAACAAGGAACGGATTGCTAAGGCTGGTGGCATTAAACCGCTTATCGACTTGGCCAGCTCCCACCAGATTGGTGTCGCTGTTGAAGCAATTGCAGCCCTTGCAAACTTGGCTGTAAACG---ATGCGAATGAGGTGGAGATTGCACGCAAAGGTGGATTAAAACCCATTATTGATGGAGCACATTCCGAGTCTGTGGAACTTCAATCCCAAGTGGCTCGCGCACTACGCAACCTGTCTGTGAATCCGGAGAACAAACAAGCAATTGTGGAGTTAGGCGGGGTGGAGGCTCTGCAATCACTCGTGCGTTCTACCAACGACCGGATATGCCAGCAGGCTACACGCGCTCTCGTCCCCCAC---GTGCTCACGCGCTCGTCCCCTACAATCTGCT------TGTGGCGCCTGTGGCCCGCGTGGCCGAAGTCAAGGAGGAAGC------CATGCCATATAACGGAGCCCTGGTGAACGTCGAGTACTCTACGACTGTGAAGACGGTG---------GTGGTGCCTGTATCCACGCCGA---ACGACGTGGTGGGA---GGCTTCTCGTCCATCTCCGACCGCTTTCCGCTCTCCAAGGTGGGGAAGGTCAGCT---GCATGGCCCCGTTGCCGATGAGTCG---CTTGCTCAATG---CCACGCCCGAGATGACGC---AGATGGAGCAG-------------ATGACGGTGCTGGTGGAAGACGCCACGGTCGATGAGAAC---ACGATCAAGGCGGAA---CC------TGAGCGACGTGTACCGGTACCTAGCTACTGA--CGTGGAAGAGAACGAGATGGAGCAGCTGCGTTGGAACATCCAAGAGCAAGTGTTCCAAAGATACTTCCAGCAAGGACAGAAGTACGAAGAGTGGGGCAAATTGGAGGAGCAAGACAACGTGTATTCTTCATTCCCGGAGGACGACGAGAAAATATTATGGCTGAAAAAAGGTTCCACGACAACGCCGTCCGAGATGCCATGGGGAAGGGAAATGCTACATGGGTCAAGGACCGAAGGGCTCGATTCGAGGAGGAAATCCGCCAATTCTATGAAGAAACACGGCACAGACAAGAACTCATGCTCACTCGACTTGAAAAGGTATCTCCGTTCTCCAAGTTCCCGATTCTTGGCAACCGGTTTTTGTTACTTCGTCTACGAGGAAAGGGAGGCAACGGAGAAGTATGGGATGTTGTCGACTACGCCAACAACAAGCAACTTTGCGCTCTGAAGCTTTCCACCTCGATTCGACATGCCCAGCGCGAACACCACACGCACCCTCAATCACCCCA--ATATTGTTCAAGTTGGTGAAGTACCATTCCTCATTCGATACCAACAGCAGCAATACACGGCATTCACGGTTGCAAGCGTTCAATCGGATCTACAGCAACTCATTGAGA---TGTACGAATATTTGGATGATGATTCTGCGTACAAAGTGTTTTCGCAGCTTTTGAGTGCACTAAAGTACTTGCACGATGGGTGTTGCTCACTACGACTTGAAGCCGTCGAACATTCTGATCGACCACGACGGGTGTGTAAAGCTA------ACGGATTTTGATCTCACGCGCGACGCTAAATCAGCTACATCCAACTCGACAGTCGGCACTTTGCGATATCTTCCTCCCGAATGCTTCCGCCCGAACCGAGGAGA---------TTGCGAAGCCACGGCTGAGAAGGCGGACATGTGGATGGTGGGCATCGTGTACTACATGATGGTTACGGGTAAACACCCGATCTGCCCCGATAAGTCGACGCACATGGAAGTTAAATCGGCGATGATTCAGTACACAGGC---GAGCTTCGATACGCACGGCCCATCTCCGATTTGTCCAAATGGATCTTGCAGGGGTGCCTTCACCCGGACCCGCGGTGTCGAATAACAGCCGCACAGTTGCTGGTAGCGCTCCAG

Phytophthora_melonis TGGACTACCACCGCCAGGTGGCGCGCGAGCTGGCCGCCTTCTTCCACTCGTTCTACCGCTTCGGGGCCGCCTCCAGCCGCTTCGACCTGTTCCTGCACAACGTGATCTTCGGCTCGCTCTGCATCGGCGCCGTCGTCTTCCTCGTGCTCTGCGTCATCGTCGTGCGGCGCTGCATCCTGCACATCCGCAGGCCTATGTGCGGCGAACGTCGATGGACTCCACGTCCGTGGAGCTGCTGGCCGTGGGACTGCTGACCTTCCTGGCTATCAGTGCACT-GGGGGACTGCTGGGAGAGGCTGTCTGGAGACGAGCAACGCCCTGCGCGTCAATGCTGACAACCTCATCACGTCGTTCAACGACAGTGAACTGCCGGCCGACGCCTTCAAGATGTCGGTGGAGGCGCTGGCCTCGTGCACGCGTCCAAAGAGCTCTGGAACCAGACGGACGTGCTGCTGCCCAAGAACTTCTCGGGGCTCGCCAAGGACTGGGAGTTCAGCTACTTCGTGCTCAAGTCGTCGACCAACGGCGCCATCCTAGCGGTGACGTTGGCCAGCTTCCTGTCCATCGCGTCTGTCGGGTGGGCCATGGTCTCGCCTCTGAGGGTCTCGATCATGGTCATTCTGAGCGTCGTGCCCATTTCGCACACGCTCATCGGCGCGTATCTGTCGTCGACGATCATGACGGCGGACTTCTGCGCCGCTCCGATGAACAACACACTTGAGCTCGTGGGCTTATGTTCAGCGAACCCGTCCTGCCCTTTGCTGAGGCACTGGATACGCAATGCGTCGCACCA-GGGGACATCGGCCAGCGTATGAAGAAGGAGTTCCTGGACCCCATCGGAGCAGCTGTGGGCGTCATGCTCGGCTACTTTGCTGGCACCGTCAGCAACTGGGAGAGCTTCTCGCTCATCCAGACGTGGCGCTGGCCCTTCCTGCTGCAGTTCCTGGCGCTGCTGCCCATCAACGCCGCCATCTTCTTCGTGCCTAAGAAGCACCTCCTCATCCGCAGTGAGGTCAGCGAGCTGGAGCCGCCGGTGGCCGGCACGCCTATGAGCACCAGGTCCACGACGTACCATGACGAGGAGGACGCGCTCCTCGCCAGCGCGAGCATGCCACGTACGGCGCTTTGGATACGAGGTCTACGAGCAGGGCGCGTTCTCGGAGGGCGTGTACGAGCTGCTCCACATCCCCGTCTTCTGTTTGATCGTCTTTGGGCTCACGACCGTGTACTTCGTCGTCACGGGCGTGCAGTACTGGAGCACCATCTTCATGATCAAGTCGCTGCACGCGTCCAAGTACCTGGTCAACGGACTGTTCGTCGTGGTCTCCGGAACGGGCCCCATTCTAGGCGTGTTCTTCGGCGGCTGGATCATCGACAGATATGGAGGATACATCGGCGTGGAGCAGCGTGCCAAGGCGTTGGGGATCTGTATGATCCTGGGGCTGACAGCGTTTTCGATCTCGGCGGTGACGACGTTCTTCGACGACGTTTACATCACTGCGGGGTTTCTGTGGCTGCTGCTCTTCTTCGGCGGCGCCATCCTGCCGGCGCACCGCGCGCTCGCCTCGTCGTTCTCCGTGATGGTGTTCAACCTCTTCGGGTACGCGCTGTCGCATGAGCGACCTGCAGATGGTGGTGGGCGCCAAGTGCTTCGTGCCGGACGAGACGCACGTGTGGCTCGCGGCCGAGGTGCTGCGCGACGAGAACTCGGGCGACGGCAAGACGCGCAACATCTACTGCAAGGTGGAGCTGCCCGACGGAGACCGAGGAGCGATGCGTAGACATGCTGTCCAAGAAGACCAAGGCGTTGCTGCCGTGCACCAGCTTGAGTCGCTGCCCTACCAGAACGAGAACGTCGGGCCCGAGGGCATTGAGGACATGATCACGCTCAACTACCTGCACGAGGCCGCCATTTTGTACAACGTCAAGAAGCGCTTCCTCTGCGAGCTGCCCTACACGTACACGGGCGACATCTGTATCGCTGTGAACCCGTACAAGTGGCTCCCGGACCTCTACGCGGAGGAGCAGCACCTGCGCTACCTCAACCAGCCCAAGGAGGAGCTGCCCCCGCACGTGTACGCCACCTCTGTGGCGGCCTACGACAACATGCGCCGCTCGGAGCGCAACCAGTCCATCCTGGTGAGCGGAGAGTCCGGCGCCGGTAAGACGGAGACCACCAAGATCCTCATGAACCACCTGGCCACCATCGCCGGAGGTCTCAACAACTCGACCATCAAGAGGATTATCGAGGTCAACCCGCTGCTCGAGTCGTTCGGTAACGCCAAGACGGTGCGCAACGACAACTCGAGTCGATTCGGCAAGTTCACGCAGCTGCAGTTCGACAGGAACGGCACGCTCGTCGGCGCCAAGTGCCGCACGTACCTACTGGAGAAGACGCGTGTCATCCAGCACGAAGCACCCGAGCGCAACTACCACATCTTTTACCAGTGGACT---CGGACATTGCCCCCGAGCTGCAGCTGGAGGCGGGCAAGCACTACGTGTACACGGGCGACAACACAGCGCAGAAGATCGAGGGGCTCTCGGACGCCAAGCACTTCAACAAGACGCGCGAGGCGCTGGGGCTCGTCGGTCTGTCACCTGACGACCAGCGACCGCTGTTTGAGGTGCTGGCTGGTGTGCTGCACCTTGGTGAGGTGCAGTTGCAGTCCGACCCGTCTGACGATGAGAAGTCTCTGATCGTTGAAGGCGACAGTGGTGCTAACTCAGCTACTCAGATGCTTGGCGTTACCTGGGAGGCCATTCAGACGGCTCTGTGTTCGCGTACCATGCGCGCTGTGAACGATGTCTACAGTGTGCCGCTAAAGAAGGAGCTGGCCATGGACTGCCGTGACGCTCTTGCCAAGGCTATCTACTCCAACGTGTTCGACTGGCTCGTAACCACGATCAACCAGTCGCTCGCGGACGACGCGAACATGGCCAATCACGTGGGTGTGCTCGATATCTTCGGTTTTGAGCACTTCAAACACAACTCGTTCGAGCAGTTCTGCATCAACTACGCCAACGAGAAGCTTCAGCAGAAGTTCACCCAGGACGTGTTCAAGACCGTGCAGATCGAGTACGAGGAGGAAGGCATTGTTTGGGACCACATCGAGTACGCGGATAACCAGGATGTGCTGACTGTGATTGAGTCGCGCATGGGTATCATTTCGCTGCTAAACGAAGAAGTGATGCGTCCCAAGGGTAACGAGGAGTCCTTCATGTCCAAGGTTACTTCCCTGCACAAGGAT---GACATGGCGCACGTGATTGAGTTCCCTCGCACGTCGCGCACCGAGTTCCTCATCAAGCACTACGCCGCTCCCGTCATGTACGACTCGGTGGGCTTCCTGGAGAAGCACAAGGACTCGCTGCTTCCCGATCTGTCCGAGCTCATGCGTGGCTCGTCCAAGCCCTTCATTGCCAAGCTCTTCGAGCCGAAGCCGGCGCCTAAGACGCCGGAAGC---------GTGGAGGTGCTCTGTCCATCACGACTGTCGGTACCCAGTTCAAGGAAAGTCTGACGGAGCTGATGGCTACGATCAACTCGACGCGTGTGCACTATGTGCGCTGCATTAAGCCGAACCCGATCAA---------GAGTGCGACCGTCATGGACCAGAACATGGTGGTGTCCCAGTTGCGTTGCGCTGGTGTCATTGAAGCCATTCGTATCTCGCGCGCTGCGTACCCGAACCGATTGCAGCACACTGAGATCCTGGACAAGTTCTGGCTGTTCGTGCCGAGTGGCGGTGAGACTGCGGCGGAAAAGTGCCAGCTGCTCATGGACAAGTTGAAGCTTGAGACGCCCGTGCAGTACCAGATGGGTAAGACTCGAGTATACTTCCAGCTTGGTGTCCTTGAAGAGCTTGAGGACCGTCGCAAGAAGTTCCTGGACGCCAAGGCCACGTACCTGCAGAACATCATGCTTGGTTTCACGCAGCGCATCAAGTACCTCCGACAACTGGAAGCGATCATTAAGTTCAGTGATCCGGTGCGTGATTGCCATGCGGAGATACAACACGTTCATGAACGGCCTTGTGAAGACTCAAGCTCACTGGCGAGGCATTCAGGGCCGTAAGGTGGCGGCCGACGTCAAGAGCAACCACAACGCTGTCATCATCCAGCGGTATGTGCGTGGTTTCGTCAAGCGTCACCGCTACGTTAAGATGCGCGCGATGGTCATCCGTGTGCAGGCCGTGGTTCGTATGACGATTCAGCGGCCCAAGTACCTGGCTGCCCTCGAGGAAAAGCGCCGCGAGGCTGACATGGCTTACCAGCTGCAGAAGCTCAAGGCAGCTTTGCAAGAGGAGCAGGAGCGGAATGCGCAGCT------GCAGCGCC---GTAGTAGTGTGGT--GACGGCGCATCCGCTGCCTCGAGCGAGGTTATGGCTGACGCTGGCGGCATGATTGAAACCCTGACGGGTGAGAACAAGAAGTTGCGTGAGAAGAACGAGGAGATGAAGGTTACGATGAAGGGCTTGAAGGCAGAGATCGAGAAGTTCAAGAGCGACAAGGAGTTCTCGTCTGCTGGCAACCACGTCAAGGTCCGTCAGCTGCAGGACACCGTGCGCGACAAGGACAAGAAAATTTCGCAGCTTGAGGCCGAAAACAAGAAGCTGACGGAGCAGATCGCCAAGCTTCACGCTGAGGGCGTCGT---GCCCGAGAAGAAGTCGATTCCGAAGAAGTCGATCTTCCGCACTCTTGGCTCCAAAAAGGAGAAGAAGCCGC---------------GCGAGACCGTGCTGATGGACTCGCTCACGGGCTCGGACGACCTGAACCGCCGGGAGGCGCGGATGTCGGA---GCGCCACTTCCCGCGCATGC---CGAGCATGAGCGTGGGCTCG---CGCTTCTGGAACTCGAACAAG---AAGGGCGAC------------AACGGCGATGGCTTCGACGTGTCGGAGGTTGACGGTGAGGACAGTCCCACGAACCCACGCGCAACACAATCGTGAAGAGCATGGAGGTGGGTGTCACGGGCGCCATGTCGACTCTGAAGGGCCGCATG---------TCGGTCGTGAAGGGCATGTACGAAGCAAGCGCACGAACAGGACCGCCCCGTGCGCGAGTCGTTCAACCTGGATGCGCTGCCGGAGGTCTCTCTCCCTGTTGGCTGGGAGGCCAAGGTGTCGCGCTCTACGGGCCGCGTGTACTACGTGAACCGCAAGCTCGGCAAGTCCCAGTTCGAGCGCCCCACGCTGGCCTCGCTCAA------GGCGCAGAAGCTGGCGCGCCAGAAGTCGGCTAACGCGCTGTAAGGCGGTGGTGGCGGATTTCGGCTGCGGAGACGCCAGACTGGCGGAGAGCGTGCCGAACAAGGTGCACTCCTTCGATCTCGTGTCGCTCGATATCGGCGTGTATTGCCTGGCGCTTATGGGGACGAGTGTTCGCGAGTATGTGCGCGAGGTGTACCGCGTGCTGAAGCCAGGCGGTGTGTTGAAGGTCGCGGAGGTCAAGAGTCGCTTTGAGAGCGAATCTCTGGGCGGATGGTGGCAGTGTTTGTGGCGGCGGCGCGCGGCGTCGTGGTGACGGTGCGCAACACGACGGTGTTCGTGGAGAGACCAAGTACGCTGTCGTTGTGGGCCAGAAGACGGGCGAGGCCGTGGCCGTGCCGTTCCCGGACGTGGACCGCGACATCAAGACACTGCTGGGCCATACCACGTCCATGGTCACCCACGTGGCAGTGAACCTCGTCGCTGCTGCTGACGGCCGATCGCGACGAGAAGCTGCGCGTCTCGAGGTTCCCGAACGCTTCCATCATCGAGAGCTACTGCCTGGGACATGCTGCCAGTCTGACCAGGGTGGCGTGCAGCGCTGTCACGCCCGAGCTGGTGGTGTCCACGTCAATGGACAACACGCTCAAGCTGTGGGAGATGAAGACCGGCAAGCTAGCGATCTGCCCCACGACGACGTTGTGGCGGTGCTCGTGAACTACCAGCACGCGCGTTTCTTCGAGATCATGAGAGCCCTTTCCTGCAGTTGTTCTCGATCTCCAAGGGCCGCCGCTGCGTTCAACTGCTGTGCGCGACTTCCGCAAGCTGCAGAACGACCCACCCTCGGGCGTGTCGGGCGCCCCCATGGACAACAACATAATGCTCTGGCAGGCCGTCATCTTCGGACCGGACGACACTCCCTGGGAAGGCGGCACCTTCAACCTGACGCTCGAGTTCAGCGAGGACTACCCGAACAAGGCGCCGACCGTCAAGTTCATCACTAAAATGTTCCACCCCAACATCTACAACGACGGCCAGATCTGCCTGGACATTCTGCAGAACCAGTGGTCGCCGATCTACGACATCTCGGCCATCCTCACGTCCATCCAGTCGCTGCTGTGCGATCCCAACCCGAACTCGCCGGCCAACTCGGAGGCTGCACGCCTCTTCCAGGAGAACCGGCGGGAGTACAACCGTCGCGTGCGCGAGATCGTTGAGCAGAGCTGGCAGGCGATCGATGGTGTCGGCCGCCAAGATGCGCGGCGCTGAGAACCGCATGAACGCTGGCCGCCCCTTCACGGCGTGGCTGGACAACGTCTCGTCGGACCGCGGTCTGTGCGGCGGCATCAACTCGGGTATCGCCAAGACCACGCGCAAGCAGACCACTCGCAGATCTTCGTGATCGGCGACAAGGCCCGCGCCCAGCTGCGCCGCGACCTCGGCGACAACATCCGCGGCAACGTGACGGAGACGTACGCGTCGCCCCCCAACTTCACGGTGGCCAGCGCCATCGCGGAGGCCGTCGACTCGGAGAGGAGTCGTCGCGTATGGCCGCCATGGAGAACGCCACCACGAACGCCGAGGACCTCACGCTCACGCTCGTCTACAACAAGGCTCGCCAGGCCCGCATTACCACGGAGCTGATTGAAATTATCTCGGGTGCCGCCTCGCTCGACGGTGACTGGCAGCGTGGTGAACGTGCAGAGCGCGGCGCAGTTCGACGAGGCCACGGCGCGCGAGTCGACGCTGTCCGTGACCTTCTTCTGGGCCGAGTTCCAGGCCTGCCGCCCCAACGGGCAGCTGGACGTGGGGCCGCGCCGTGCTGGACAAGCTCGAGGGCGCCAACGTTGCCGAGCTGGCCAAGCGCGTCTGCAGTACCGCCTCAAGAAGCTCATCAGCGCGTCGCCCGTCATGCTCTTCATGAAGGGCAACCCGACGGAGCCCAAGTGCGGCTTCAGCCGGCAGATGGTCGCGCTGCTGAACGAGGACAAGATCCAGTTCGGCACCTTCGACATCCTCAACGACGACGAGGTGCGCCAGGGGCTCAAGCAGTTCTCCAACTGGCCCACGTACCCGCAGCTCTACGTGAACGGCTCGCTGGTCGGCGGTCTGGACATCCTCAAGGAGATGAAGTCGGAGGGCTCGATCGTCGAGCAGCTCGGCGTCGAGGAGGCCGAGGCCGCGTTCCAGGAGAGTCTGCGCGCGCTCGTCAACTCGGCGCCCATGCTGCTCTTCATGAAGGTTCGACATCCTGTGTGGAGCTACCAGCGCCAAGACCAAGACGTCGCGAAGGTGGTGACTCTGATGCCAGCGTCTTTCCGCCGGGAGATGTGCCGAGGCCATTTCGTGGAGTCCGAAATCGCATGGCCATGGCATTCCGCGACCCTGTTGACGGGAAGGGCGAGTTTTGCATTGTGCAGCTCCCCGCCTTACCTCGAAGCGCACTACCCTGCATCAACGAGGTGTTTCTGCGATCGACTGGCAAGTGGAGTGTGCTGCACCGCGACCACCGAAGTGAAGTCCGATCGCTCTGTGTGCACTCGAAGCGCGACTGCTTGTACACGGGTGGTCTGGATGGCCTGATGATTCGATATGATCTGAACAAGGGCCGCACCCAGACCGTGATCGAGCGCCGCAAGCACATTTCCAAGATCAACGCGGTGCTGGAGCACCCGCACAACCCGAACCTGCTTTTGGTCAGTTGTGTGGAGCAGCAACCACAGCTTGCTGCTGCACGATCTCCAGCGCTACGAGCCCAACGTGTCGGGTGGTGCCATGAGTCAGTACGTTGTGCCGAGGTGGTCGCCTGCAGGATATCACGTGTCGTGCGGCAGCAAATGCGCTACTGCCACGGCAGCTTCTCCACGCCCACGTCGGCCACCATCGGGGCCTCGTTCCTGCAGAAGCGCGTGATCGTGGGCGGGAAACTGACGCTGCAGATCTGGGACACCGCCGGCCAGGAGCGCTTCCGCTCCATGGCGCCCATGTACTACCGCAACGCCAAGGCGGCCATCCTGGTCTTCGACCTGCAGAGCGAGGCCAGCTTCGAGAAGATCAAAGAGTGGCTGCACCTGCAGCACCACGTGGGCGACGACATGTGGGCAACAAGAGCGACGTTTCGACTTCTCCAAGGCCCAGCAGTTCGCGGACGAGATCGGCGCGCTGGTGTTCCGCACTAGCGCCAAGACAGGCGAAGGGGTGCAGGCGCTGTTCGAGAGTCTCGCGGTGCAGCTGCTCAAGAAGCACGACCAGGAGCAGCGGACAGCTTCTACGTCGTGTACGTGCCCGCGGATGAGCGCAAGGAGCTGGAGGAGTGGACGGTGGCGCTGCCCAAGGACAAGGAGGCGCAGCTTGGCTGCCTGACGGAGCGTCTACGCGCGCACTTCAAGGCGGGGGCCATCCCAGCAGCAGCAGAGACGTTCAAGCAGCAACTGCTCGCGCAGCTGCCCAAGGGCGCCACCATGACGGACGAGATGCTGCAGATGATGCATGGACAGCCTCGTGGACTCGATCCCGCTCGTGCTCAACTCGCCCGACGCCAAGCACGTTGGAGGTCAAGGGAGACGCGTTCATTGGACGGGTATTTGATAGACGACTTTGTGCGTATGGACTTCCGATTGAGCGAAGTGTCGGGTGACGCTCCGTGGGTGCAGCAGGGCAAGCAGCAGAGGTCACGCAGTCGCTTGAGGAGATCCTGGCGCTGGGCCAAGGCTTGGACGACGAGGACTACAAGGCCGAGCTCGAGGTGCTGCAGAACGACCCCAACTCGAACCTTTACTCGGCCGTCACCTTCGAGGAGCTCAACCTACCGGAGGCCCTGCTCAAGGGCGTGTACAATATGAAGTTCGCCAAGCCGTCCAAGATCCAGTCGGTGGCGCTGCCGCTCATCCTCGCCGACCCGCCTGAGAACCTGATCGGCCAGGCGCAGAGTGGCACCCCACCCGTGAGCTGGTGCGCCAGATTATGGCCGTGGTGAACGCCATGGGCAAGTTGTGGACACGTTCCTGGCTATCCCGGGCAACGACCTGGCTCGTGGGGAGAGTCTCAAGGCGCAGGTGATCGTGGGCACCCCCGGCAAGGTCGAGGGCATGATCAAGAAGAAGCAGCTGGACACCCGCAACGTCAAGATCTTCGTTCTTGACGAGGCCGACGTCATGGTCGCCGAGGACGGCATGCGCGAGCGCTCCGTGGCGATCAAGCGTCTAATTAAGAACCGAGCGTGCCAGTACCTGCTGTTCTCGGCCACGTACGCTGACGACGTGCGCGACTTCGCCCAGAAGATGGTGCCTGACCACAACATTATCACGGTCAAGAAGGAGAAGCTGACGCTGGACGGCATCAAGCAGTTCTGGATCGACTGCAAGACTCGTGAGAACAAGTTCCAGGTGCTGTCGGACATTTTCGCCATCCTGTCTATCGGCAAGTGCGTCATCTTCGTGCAGCAGCGTGACACGGCCAAGGAGCTGACGCGCTCCATGCGTGAGAAGGGTCACTCGGTCGGAATCCTGCACGGTGCCGACATGGCCAAGGAGGTGCGCGACCAGGTCATCGACGAGTTCCGCGCCGGCACGACCAACGTGCTGATCACCACCAACGTGCTCGCTCGCGGTATCGATGTGGCGGGTGTGTCTCTTGTGGTGAACTTTGACATCCCTCTCACGCGCGACCACCGTCCCGACCCGGAGACGTACCTGCACCGTATCGGTCGCACGGGTCGTTTCGGCCGCAAGGGCTGCGCCATCAACTTCGTCTACGACAACAAGAGCAAGCAGGACCTGGCCGAGATTGAGGAGTACTACGCGCGTCCGATCACGCAGGCCCCCGCTGACGACATCGAGGAGCTCGAGAAGCTGTTGGCTTGGGGGGCAGCAAGCGCCGCCTGGCCAAGTTCGGCAAGGAGCAGCGCAAGGGCATGCGCATCTACCCGCGCGAGCCCAAGAAGAAGCCCTCGGGCAAGGACAAGACGTACTACTACATCAAGGACGTCATGTTCCTGGCGCACGAACCGGTGCTCGAGAAGTTCCGCGAGTTCAAGACCTTCATGAAGAAAGTCAAGAAGGCCATGGGCAAGAAGGACTACGAGGACGCCAAGCGCAAGCACGAGAACCGCCCCACGTACGAGCTCGACCACATCGTCAAGGAGCGCTACCCGCGCTTCGTGGACGCGCTGGGCGACCTGGACGACGCGCTGTGTGGCAGAACTACATTGTGGCCACGCGCGCGCTCACCAAGGTGTTCGTGAGCATCAAGGGCATCTACTACCAGGCCAACGTGCAGGGACAGACCATCACGTGGCTCGTGCCCCACCAGTTCACCCCCACCGTGGACAAGCGCGTGGACGTGCGCGTCATGCTCACGTTCCTCGAGTTCTACGAGGTGCTGCTCAAGTTCGTCAACGCCCAGCTCTACATGCAGATCGGCGTTGCCTACCCGCCCAAGATCAACCTGCAGCTGGATGCCGCTGGTGCGCAGCTTGCGGCGATTGAGCTAGCAGCAGAAGGAGTCCGAGAAGCGTATCAAGCTACAGCGAGCAGCGCAAGGAGCGCAGCGAGAAGAGTCTGTTCAGCGGCCTGACCTTCTTCCTGTCGCGCGAGGTGCCGTCGGCCTGCCTGGAGCTGGTGCTGCGCTCGCACGGCGCTACGCTTGGCTGGGACGGCGCCGGCTCGCCTTTCGACGAGAAGAGCCCGCGTATCACGCACCACATCATGGATCGCCCGCACCAGGGTCACCGCTACTTCAACCGCGAGTACGTGCAGCCGCAGTGGGTGTTCGACTCGGTGAACAACGGCATTCTGCTGCCGCTGACTCGCTACCGCAAGGAGCTGGAGAAGGTCAAGTCGGCCTCGCAGGTGCTGCGCGAGGTCGGCGCCGTGGGCGACGAAGACGAGGACGGGAGGCGTACGCCGCTGACCTTAAGGCCGAGATGGACGGCAACAAGAAGTCGAAGCGTGCCAAGAAGGAGGAGGAGGCCGCGGAGCTCAAGGAGCTGGCCAAGACGATGATGTCGAAGAAGGCCAAGCGTCTGTATGACCGTATGCAGTACGGCCTGGGCAAGAAGGCCGACAAGATTCAGAAGCTCGAAGACAAGCGCGCCCAGCTGGAGGCAAGACCAAGAAGCTGCTGGCTGGTGCACTGTCTGGCATGATCTCGGCGTTGGCCTTCCAGCCTCTGGGGAGATGCGCTGGCGCGGCACGTCGCCCACGCTCGTGCGCGTGGCGGGCGGCGCCGGCCTCTACTTCGCCACGCTGGACCACTGCCTCAACCTGTTCCCGCAGTCGGCGGTCAACACTTTCATGGCTGGGGCCTTCGCGCGCACGTTTGCAGGCGGTGTCATGTCGCCGTTCACGATCGTGAAGGCGCGGATGGATGCC-CCGGGCACCTTCGACAGCAAACGGACGTGCCGTTCTCCGGCCTCTACGTGCTCATTTACACGGCACCCGGCTGACGTGGTAAAGACGCGCATGCATGCGGCAAACCGTGGCCAAGATCTACCGCCACGAGGGCCTGCGCGGATTCGCGAAGGGCATCCTTCCGCGTGTAGTCAAGCGTACGCTGTCTACTGCAGTCACATGGACGATATACGAGCTACTCGTTCGACCCGTCGGGTCTGGGCCATGCCAAGGAGGCCTTCAACCTGGCCAAGGAGACCGAGCGCACCAAGCAGATGGAGAACCAGGCCAAGATCAAGGAGAACGAGGCGCTCTACAAGCAGTACGAGATCGTGCGCGTGCAGAAGGAGGGCGAGGAGCGCCGCAAGACGCTCGAGGAGGAGACGCGCCAGCACCAGCAGCGCGCGCAGTACCAGGACCAGCTCAAGCGCAAGCAGTACGCCGACCAGCTGGCTGCCCAGAAGTACATGAAGGAGCAGGAGCTCAAGAAGCAGGAGGAGATCCTCGCTCGCCAGGAGGCTTCGCGCCGCAAGACGCTCGACTATGAGGCCGAGCTGCGTCAGAAGACGGAGCTGGCCAAGGTGGCCGCTGAGACCGAGGGCCGCATCAAGCAGGAGCGTCTCAACCACGACCTGCACCTCGAGGAGGCCCGTGTGCGTGCTAAGGAGTACCGCGAGACGGTCATGGAAGGTATCAAGCTGGCCGGTAACACCGTCGGCTCAGGTATTATGGCCTTCGTCGACGACAAGGAGAAGCTTACGGCTACCGTGGCCTCGCTGACGGCCCTGGCTGTCGGTATTTACACCGCCAAGGTCTCCACCAACGTTGCAGGCAAGTACATTGAGGCTCGTATGGGTAAGCCGTCGCTGGTGCGTGAGACGTCGCGTCGCTCGGCTACTCAGGTGCTGGCCAACCCGATTCCGTCGATCAAGCGTGCGCTGCGTCTGCAGAAGGCGACGGATGCCCTGGAGGGCGTTGTGCTTGAGCCCAAGCTGGACGAGCGTCTGCGCAGCGTGGCCGTGTCCACGTTTAACACGAAGAAGAACCGCGCGCCATTCCGCCACTTGCTGCTGCACGGTCCGCCTGGTACGGGTAAGACCCTGTTCGCCAAGGCTCTGGCGCGTCACTCCGGCCTCGAGTACGCCATCCTCACGGGTGGTGATGTCGCCCCGCTGGGCCGCGAGGGTGTGACGGAGATCCACAAGCTGTTTGACTGGGCGTCGCACAGCCGTCGCGGTCTGCTGCTGTTCGTCGACGAGGCCGACGCGTTCCTGCAGAAGCGTAGCAACACCGTCATGTCCGAGGACATGCGTAACGCGCTGAACGCCTTCCTGTACCGCACGGGTGAGGCCAGCGACAAGTTCATGATCGTGTTCGCCAGTAACCAGCCGGAGCAGTTCGACTGGGCCATCAACGATCGTATCGATGAGATGGTGGAGTTCCGTCTGCCGGGCTTCGACGAGCGTGTGCGCATGCTCAAGCAGTACTTCGACGACTACATTCGCGCGCCGAAGAACTCCCGTGCCAAGAAGATTTACGCGAGGGCATTATCTTCACTCTGGACACTGTGGCCAACTTCCTCGTGCCAAGACACGCATCTTCCAGCTCGAGGCACTACAGGGCGTGTCCATTGAAGCCCAAGCCATCTTCGATGCGCTCAACAAAAGGCCATGCGCCTCTACTGGGTCGTCTTCGGTCTGGTGCTGGCCAACGGCCTGTTCACGTACGTGGCCGGCAAGGACGAGTCGTTCCTGGTGGAGAAGCTCAAGAGAAAGATCCCGTCCGAGTACCGCATCTCGGAGAAGTGGGACAAGTGCCTGGAGAACTTCACGCTTTACTTCGGCGCCGGCCTCGTGGCTGGCGGCCTCACGTCCCTCGTGCTGGCGCGTTCTGGTGCGGGCCGTGGACTGATCACGGGTCTGGGCGCTCCGGCGCCGGCTCGAGCTGGACGACGTGCCAGCTGGCGTTCGCGGGCAACGCACAGGCCGCGCTGGACAAGACGGACAAAGCTGTGGGCGACTTGCCCGCCTTCCAGCAGCAGACCATCGTGGACTTCGTGTCGGCCTCGGCCACGCAGCAGCAGCTGCAGGCCCTGCACCCGCGGCTTCTGCGAGCAGGCCAACGTGAAGGTCCTGGAGACGTACCTGGCCGAGCAGGTCCAGAACAAGACCGTGGACATCGACGCCAATCTGGCGCTGCTCAAGCTGTACCAGGTGTACCCGGCCACGGCCAATGCCGAGAGCCACGGTGCTGGTCAAGGGCGTCATGGCCCTGCCCTCCACCTTCTTCACGGGCGTCCACCATGATCCCGGAGAACATCCGCGAGGACGCCAACGTGACGGCCGTGCTGCACGCGGGCTTCCTGCTGCAGTCGTGCCTGTTCGAGGACTTCTGGAAGGAGAGCGTGGCCTTCGCCGAGAAGGTGCCTGGCTACCTGGAGTCTGTGCGTGCGTACATTCTGACGGCCATCAGCCGCAGCCACAGCGCCATCTCGCCGGACGTCTTCAAGGCCAAGCTCAACGTGTCCGACAAGGAGGTGCCCGACATCGTCGCCGAGAAGTGGACTCGGCCAACCTGATCCAGGTCAACCCCAACGAGGACAACCAGATGCAGGCCAAGAAGGTTCAGGAGAACATCGAGTTCGAGGACGTGCTCAAGGTCATTCACACCCTTTCTAGATCCAACGTCTGCGCCAGCGGCGGCGGCGTGGCCGACGCTGAGGAGGTCTGCGAGAACCTGGCCAAGCACCTGCGGCTCGAGGGCGGCGCGCAGGTGCTGCCCTGCTCCACGGGCGTCATCGGCTGGCGCATCCCCGTGGAGTCCATGGTGGAGAACCTGCCCAAGCTCGTGAACAACCTGCAGTCCGACTCGGTGCTGCCCGCTGCCGAGGGCATCATGACCACGGACCTGTACCCCAAGATCCGCTCCATGGACGTGTGCGGCGGCCGCATCGTGGGCATCGCCAAGGGCGCCGGCATGATCGAGCCCAACATGGCTACCATGCTGTCGTACATTCTCACAGACCTGTCGATCCCGCGCGACGTGCTGCGGCAGCTGCTGACGGAGGTGGTGGGCGACACGTACAACGCACTGAGCATCGACACGGACCAGAGCACGTCCGACACGGTGGCGCTCGTGTCGTCGGACCAGATCCCGTTCGACATGGC---TAAGCTCGACGAGTTCAAGAAGGCGCTGCACGAGGTCTGTCTCGGTCTGAGCGAGGACATCGTGCGCAACGGCGAGGGCGCGCACCACGTCATGCGCGTGCGCGTGCGCGGCGCCGTGACCAAGGAGATGGCCAAGGGCGTGGGCAAGTCGGTGGTCAACTCGCCGCTGCTCAAGTGCGCCGTGGCCGGCAACGACCCGAACGTGGGCCGCCTCGTCATGGCTGTGGGCAAGTACATGGGTCTGCACCACAAGGACGTTGACATCCAGCGCCGCATGACGATCCGCATGGGCGGTGTCGTCATCTTCGAGAACGGCGAGATGGTGCTCAACGACGAGATCGAGGCCAAGCTGGTGGCCCACATGCGCGACGCGATGCTGATCGAGCCCACGCGCGGTGGCCTCGACGCCAGCTCGCACAATTACCCACCGCACGGAAAGTTCGTTGAGATGGAGATTGACCTGGGTGTTGGCTCGGAGGAGACTCAGGTGCTGGGCATCGACCTTACGCACGAGTACGTGGCCATCAACGCCGACTACCGCTCGTAATGGCGCC---GAAAAAGGGGAAGACCGCCGCCGCGTACCTGGCGTCTCTGGGC---------------------------------------------------------------------AAACCCAAAAGAAGCCCATGTCGGCGGCGGCCAAGGCGCTGA-TGGAGCGCCAGGCGGCGCTCAAGGCGGAGCAGGAGCGCCTCGAGCAGGAGCGTTTGGAGCAGGAGCGCATCGCCCAGGAGGAGGAGGCCAAGCGCCTTGCTGAGCTCGAGCGTGAGGAGGAGGCCCGCCGCAAGAAGAAGGAGGCCCGTGCCGCCAAGATCGAGCGCCAGAAGAAGGAGGGCACGTACATGACCAAGGCCCAGAAGCAGAAGGCCGCCAAGGCCCAGGCGGCCCTCGAGGCCATGCAGCAGGCCGGAATCGTACCCACCGCTGCC------TCGGAGAAGAAGAAGGTCGTGTACTCGGACAACAAGAAGAAGAACAAGAAGAAC-----------------------------------------------GAGGTCAAGAAGACCAGCGACGTCGTGGCCTCGCTGGAAGACAAGTTAGCAGAAGAAGGAGCAGGAGCGTCTGCGCCAGCAGGGTATTGCGCTCAAGAAGCGTCAGGAGGAGCAGGCCGAGCAGATGCGCCTGGAGGCTGAGGAGGAACGTCAGCGCTCGAAGGCCAAGATGGAGGCGGAGCGCAAGAAGAAGGAGGCGGCTGAGCGTCGTCGCGAGCGTGAGGCTGCTGCGCGCGCCAACGTGTCCCTGGACAACCTCCGTTCGCCTATTTGCTGTATCATGGGTCACGTCGATACCGGTAAGACCAAACTGCTTGACAACATCCGTAAGACCAAGGTGCAGGACGCGGAGGCTGGTGGTATCACCCAGCAGATTGGTGCGACCTTCTTCCCAGTGGAAGCGATCAAGCAGAAGACCGCTCGCCTCGCCGAGACGATGAAGCTCGAGTACCGCCTGCCCGGTTTGCTTGTGATTGATACCCCCGGTCACGAGTCTTTCACGAACTTGCGTACGCGTGGTTCGTCGCTATGTGATATCGCTATTCTGGTGGTTGATATCATGCACGGTCTGGAGCCGCAGACGTTGGAGTCGCTGCGCCTGCTGCGCCAGAAGAAGGCCCCGTTCGTGGTTGCTCTTAACAAGATCGATCGTTGCTACGGCTGGAAGACGATGCCCGACATGCCCGTCCAGGAGGCGCTCAAGCACCAGAACGAGCACGTCATTCGCGAGTTCGAGGACCGCATGAAGTCCATTATTGTTGAGTTTGCCGAGCAGAAGCTGAACGCTGAGGTGTACTGGCGCAACAAGGACCTGGCGCACACGGTCTCGCTCATTCCCACCAGTGCTATCTCGGGTGAGGGTGTGCCGGATCTGCTTATGATGCTGACGCGTTTGACGCAGGAGCGCATGGCCAAGTCGCTTGCTTTCGTGGATATTCTGCAGTGTACCGTGCTTGAAGTGAAGGTTATCGAGGGTCTCGGTACCACTATTGATACGATTCTGGTGAATGGTACACTGGAGGAAGGTGCGACTATTGTCGTGTGTACACTGGACGGACCTGTGGTCACGACGATCCGATCCTTGCTGACCCCGCACCCCATGAAGGAGATCCGCGTGAAGGGCGAGTACCTCCACCACCAGAAGATGAAGGCCGCTATGGGTGTCAAGATTTGCGCTCAGGGTCTCGAAAAGGCTGTGGCTGGTACTCAGATTTACGTGGTTGGTCCCGACGACGATGTCGAGGAGCTCAAGGACTCGGTCATGTCCGACCTTACGGGTATCTTGGACTCAGTGAAGGCCACCCGCCGCGGTGTGATGGTGCAGGCTTCGACTCTTGGTGCGCTGGAGGCGCTTCTGGAGTTCCTGCGTACGTGTGACCCGCCCATTCCGGTTTCGTGTGTGAACATCGGACCCGTGCACAAAAAAGATGTTATGCGTGCTTCGGTGCAGCTCGAGCACCAGCCCGAGTTCGCTACCATTCTGGCGTTCGACGTGAAGGTTCACAGCGACGCCACGGAGCTGGCCGCGGACCTCGGCGTGCGCATTTTCACGGCTGACATCATTTACCACCAGTTCGATCAGTTCACGGCGTACATGGACAAATTCCGTCAGTCACGCCGCGAGGAGTTCGCAGAGGTTGCCGTGTTCCAGTGCGTGCTGAAGATCCTGCCCAACTGCGTGTTCAACAAGAAGGACCAGATTATCCTCGGAGTGGACGTCGAAGATGGCTTCCTGAAGGTGGTCACGCCGCTCGTGGTGCCTTCAGCTGGCG---GCTTCCTGGTGGGTAAGGTGGGCTCCATTGAGCGCGAGCACAAGGAGGTAGACCGCGCCAAGAAGGGCGCGTCCGTGGCCGTGCGGATAGACAACGAGGGCAGCGTCATGTACGGCCGCCACTTCGACCACAAGAACAAGCTCGTCTCGCGCCTGACCCGCCAGAGCATCGACGCGCTGAAGGAGAATTTCAGGGAAGACCTGCAGAAGGAGGACTGGCAACTTGTCATCAAGCTCAAGAAGGTGTTCGATATCATTATGGAGGGCTGGCTCACCAAGCGCCGCGACCACATGAACCTCATCTGGCTGGATCGCTACTTCCTGCTGGACGGCAACCAGCTCCGCTACTACCGCAAGCGCGGCGACCCAGCGCCTCGAGGCACCTACATCTTGACGGACGCGTGCGTGGTGAGCCCCGTGTTCAACTCGGAGGAGAAGCACAAGCACCACGGCCCCGTGTGGATGTTCCGCATCACGTGAGAAGTCGTACAAGAAGGACCGGTTCCTGGACCTGGGTGCCAAGAGCGAGGAGAACGCCAAGGAGTGGAAGCGCGCGCTGGAGAACGCCGTGCGCTCCATCCGCGAGCTCGTGGCGCACCAGAAGAAGCTCAAGACGGACGCCGAGAT---CTTCCCCACGCCCACGCAC---------GTGCCGCGGCTGCTCAAGACGGATGAGGACACGGTGTCACTCTTCAATCTGGACGCGGACAACGTCAAGGCGGAGCAGGCCTGGTGGCTGGCCAGAGTGGAGGGAGGCCTGAGGATCATGCAGGAGTGTCCGCTCGGACCCAATGTCGCGGCCAACCTGTACGCGCAGCATGCGGCAACTACCAACGGACTCATCTTCAGCGCCTGTGTGGGCGCGGCCCTGAGCTTCAGCAGCATGTACTCGTTCGCCAACAATATGCTGCTCAGCTTCATGACGGCGCTCGTGGCGATCGCCGTGTGTTGCGCTGCGGTGACGCCTACGCAAGCTCCGGAGATCCCGAGCTTCCGCGTCTCGCAGGTGGTGCACGGATCCCCGACCGAGGTGTTTCGACTCATCATGAACTCCAAGCGGTTCCAGCGCTGGGACTCGGCCACGGCCACCATGCGCGTGATCCAGCAGCTGGACGACCACGCCGACATCGTCTACGTGACTCAACGGCCCACGCACCTGTGGCCGCTGTGGCAGAAGGCGCGCGACCTGGTGCTCATGCGCTACTGGCGCCGCGAGGAAGACGGATCGTACTTTGTCATGTACCAGAGCATCGAGCACCCGGAGTGTCGTGTGCGTCACAACTACGTGCGCGCGAACCTGCTGGGCGGCGGCTTCGTCATCGCCCCTCAGCGCGTCCCCAGT---GGGTCCATCCGCACGCTGATCACGTACGTGCTCCGATACGACCCGGGCGGCTG---GTCGCGCATTTACCACCAGCTCGGCATGGACGTGGATCTCGTCATGCCCATGCTGCGCAGCGTTGTGGGCATCCGCGACGAGATGAGCGCCACGGACTTCGTCACGCCCAACGTGTCGGTGGCCCCTGCTGGAGAGGGCGGAGGCTGGCCAGGAAGCGTGGA---CTCCATGGGCGTTGTCATGGGCGTGCAGAAGCTTCAG---ACCAGTCTGCCGGAGAAGATGTGGGCCGAGCCGGATGCCTCGCTCTTCAGCGTGCGTGGCCACAACTACCTCAACGACAAGAAGAAGATCCCGTCGGCGCCTGCGATGTTCCACACGGTGGGCGTGGATCTGCTGTCGTTTGAGAGCGCGGCCGAGCGATACAACATCTCGTCTCGACCGGACAGCATCGGCCGCACCAG---CTCCAAGTTCACGTTCGTCGTCAACATGATCATCCCGGGCCCGGAGAACGTGTGCATGGTGTTCTACTTCCAGCCCGTGCGCGACAACGTGTTCGAGGACGGCTCGCCCTTCTCCGAGCTTCTCAACGACTTCTTCGACGGCGACGACCAGTTCCGGAACTCGCGCTTCAAGCTCATCCCGACGGTCGTCGAGGGAAGCTTCATCATCAAGCAGTCGGTCGGCTCCAAGCCCACGCTGCTCGGCAACAAGCTCAAGTGCCCGTACCACAGTGTGGAGAACTACTTCGAGGTGGACATCGACATTTCGTCCAACTCGGTGGCCAACACTGTCGTCGGCATGGTGCAGTGCGTGACCAAGTCGCTGGTCGTGGACAGGGCCTTCCTGCTCGAGGCGCAGACCGACGAGGAGCTGCCGGAAATTATCCTGGGCGCCGTTCGCATGCAACACATTTCGCTGGACAACCCCCGCCGGATGCCGAGGCATGGGGAACGGGCAGCAAGGACGAATTCACCTTCTGAGGCCTTCACCGAAGCTCTGCGCGTGAACCTGCAGCGGCTCATCGTCTACGCGCGCTCGGCGGACACGGCGCTGCAGCGAGAGGTAGCCGAGAAGCTGGCGAACGAGGCCGTCAAGACCGACAGGTGCAGATCGTGGAGCTGGACGGCTTGCAGCTGCTGCTGCCGCTCACCAAGTCCAAGGACACGGAGGTCCAGCGTCTGGCAGCGCACGCACTCGCCAACCTCTCGGTCAACTCGGAGAACCAGTCCAAGATGGCGACGGAAGGAGGCATCGATATGCTCATTGACCTCCTGGGCAGCACGAACGAGCACGTGCAGCGGCAGGCGGCCAAGGCTCTCGCCAACCTCGGAGTGAACGTGGACAACAAGGAGCGGATCGCCAAGGCGGGCGGCATTAAACCGCTCATTGACTTGGCCAGCTCCCGCCAGATCGGCGTCGCAGTGGAGGCCATCGCAGCGCTCGCGAATCTGGCTGTCAACG---ATGCCAACGAGGTGGAGATTGCTCGCAAGGGCGGCTTGAAGCCCATCATTGACGGTGCGCACTCGGAATCCGTCGAGCTGCAGTCCCAAGTAGCCCGTGCACTACGCAACCTATCGGTCAACCCGGAAAACAAACAAGCGATCGTGGAGCTGGGCGGAGTGGAGGCCTTGCAGTCACTCGTGCGCTCCACCAACGACCGGATATGCCAGCAGGCCACGCGCGCGCTCGTCCCCAACGTGGTGCTCACGCGCTCGTC-CCCACAACCTTCTG---GCCGTGGCGCCCGTAGCCCGCGTGGCCGACGTCAAGCAGGAGGC---CGTCGCACCCTACGACGGCGCCATGGTGGACGTCGAGTACTCGGCCACAGTCAAGACGGTC---------GCCGTGCCCGCCGCCGCCGCCACCGACGCCGCGGCCGGC---GGCTTCTCGTCCATCTCGGACCGCTTCCCGCTCTCCAAGGTGGGCAAGGTGAGCTGCGGCATGGCCCCCATCCCCATGAGCCG---CCTGCTCAACG---CCACGCCCGAGATGACGC---AGGTGGACCAG-------------ATGACGGTGCTCGTCGAGGACGGCACGGTCGATGACAAC---ACGATCAAGGCCGAG---CCGGAAGGTGAGCGACGTGTACCGGTACCAGGCCACGGA--CGTGGAAGAAAACGAGATGGAGCAACTGCGATGGAACATCCAGGAGCAGGTTTTCCAGCGCTACTTCCAGCAAGGCCAGAAGTACGAAGAGTGGGGCAAGCTCGAGGAGCAGGACAGCGAGTCTTCTTCATCCCCGGCGGACGACGCGAGAACATCCTGGCCGAGAAGCGGTTCCACGACAACGCCGTGCGGGACGCCATGGGCAAGGGAAACGCCACGTGGGTCAAGGAGCGACGAGCTCGCTTCGAGGAGGAGATCCGCCTGTTTTACGAAGAAACTCGACACAAGCAGGAGCTGATGCTTACGCGGCTTGAAAAGGTGTCGCCGTTCTCCAAGTTCCCGATTTTGGGCAACCGCTTTCTGCTGCTCCGTCTGCGAGGCAAGGGAGGCAACGGAGAGGTGTGGGACGTCGTCGACTACGCCAACAACAAGCAGCTCTGCGCTCTCAAGCTCTCCACCTCCATCCGACACGCCCAACGCGAGCACCACACGCACACTCAACCACCCCA--ACATCGTCCAAGTTGGTGAGGTGCCCTTCCTCATCCGGTACCAGCAGCAGCAGTACACCGCTTTTACTGTCGCGAGCGTTCAGTCTGACCTCCAGCAGCTCATTGAGA---TGTACGAGTACCTGGATGACGACTCGGCCTACAAGGTGCTCTCGCAGCTCCTGAGCGCGCTCAAGTACTTGCACGATTGGTGTGGCTCACTATGACTTGAAGCCGTCCAACATCCTGATCGACCACGACGGTTGTGTGAAGCTG------ACCGACTTCGACTTAACGCGCAACGCCAAATCAGCGACGTCCAACTCGACGGTGGGCACTCTTCGCTACCTGCCTCCCGAGTGCTTCCGCCCGAACCGGGGCGA---------TTGCGAAGCCACAGCGGAGAAGGCGGACATGTGGATGGTTGGGATTGTGTACTGCATGATGGTCACGGGAAAGCACCCGATCTGCCCCGACAAGTGCACGCCAGCGGAAGTCAAGGCTACGATGGCCCAATACACTGGC---GAGCTCCGGTACGCACGACCGATCTCCGACCTATCGCGATGGATCTTACAGGGATGCCTCCACCCGGACCCACGGTGTCGGCCGACGGCCGCTCAGTTGCTGGTGGCGATGCAG

Phytophthora_nicotianae TGGACTACCACCGCCAGGTGGCCAGGGAGGTGGCCGCCTTCTTCCACTCCTTCTACCGCTTCGGCGCCGCCTCCAACCGCTTCGATCTGTTCCTGCACAACGTTCTCTTTGGCTCCATGTGCATCGGAGCCGTCGTTTTCCTCGTGCTCTGCGTGATCGTCGTGCGCCGCACCATCCTACACATCCGCAGGCGTTTGTTCGACGTACATCGATGGATTCTACGTCCGTGGAGCTACTGGCCGTCGGATTACTGACATTCCTGGCGATTAGTGCGCTTGGAGGACTACTGGGAGAAGCAGTCTGCAGACCAGCAACGCCTTGCGTGTCAACGCTGACAACTTGATCACATCATTCAACGACAGTGAACTGCCAGCTGAAGCCTTCAAGATGTCCGTGGAGGCGTTGGACTCGTGCACGCGTCGAAGGAGCTACTGAACCAGACAGACGCGTTGCTGCCCAAAAACTACTCCGATATTGCCAAGGAGTGGGAATTCAGCTATTTCATGCTCAAATTGTCGACCAACGGAGCCATTCTCGCTGTAGCGCTGGCCAGTTTCTTGTCCATCGCTTCTGTTGGCTGGGCCATGGTCTCGCCGCTACGTGTGTCGATTCTCGTGATCTTGAGCGTTGTACCCATTTCACACACGCTGATCGGCGCCTACCTGTCGTCCACGATCATGACTGCGGATTTCTGTGCCGCTCCGATGAATAATACACTCGAACTGGTGGGCACGTGTCCAGCCAACGCTTCCTGCCGTTCGCTGACGTTATGGCTACGCCATGCAGCTCACGAGGGAGAAACTGGTCAGCGTATGAAGAGAGAATTCCTCGATCCCATCGGAGCAGCTTTGGGCGTCATGCTCGGCTATTTCGCTGGCACGGTCAGTAACTGGGAGAGTTTTTCACTCATCCAGACGTGGCGCTGGCCATTTCTGCTTCAGTTTCTGGCTCTGTTGCCCATTAACGTAGCCATCTTCTTCGTGCCAAAGAAACATTTAGTGATTCGCAGTGAGATCAACGAGTTGGAGCCTTCGGTGGCTGGAACTCCCATGAGCACAAGATCCACGACGTATCACGACGAAGAAGACGCGTTGCTGGCCAGCGCCAGTATGCAACGTACGGCGCACTGGACACGAGGTGTACGAACAGGGCGCGTTCTCCGAGGGCGTCTACGAACTGCTGCATATCCCCGTCTTCTGCTTGATCGTGTTCGGGCTCACGACTGTGTACTTCGTCGTCACGGGCGTGCAGTACTGGAGCACCATCTTCATGATCAAGTCGTTGCATGCGTCCAAGTATCTGGTCAACGCGCTGTTCGTCGTTGTCTCCGGTACTGGCCCAATCCTGGGCGTCTTCTTTGGTGGCTGGCTCATCGACAGATACGGAGGATACATCGGCGTGGAGCAGCGTGCCAAAGCCCTGGGAATCTGCATGATCCTGGGCCTCATCGCGTTCTCCATCTCAGCAGTGACGACGTTCTTCGACGACATCTACATCACTGCTGGGTTCCTGTGGCTCCTGCTCTTCTTCGGCGGCGCTATCCTGCCAGCGCATCGAGCGTTGGCTTCGTCCTTCTCCGTCATGGTCTTCAACCTGTTCGGCTACGCGCTCTCCCATGAGTGACCTGCAGATGGTGGTGGGCGCCAAGTGCTTCGTGCCGGATGAGACTCATGTTTGGCTCGCGGCCGAGGTACTGCGTGACGAGAATTCGGGCGATGGCAAGACACGTAAGATCTACTGCAAAGTTGAGCTGCCCGACGGAGACCGAGGAGCGCTGCGTAGACATGCTGTCCAAGAAGACAAAGGCGCTGCTGATGTGCACCAGCTCGAGTCGCTTCCTTACCAGAACGAGAACGTTGGGCCCGAGGGCATCGAGGACATGATCACTCTCAACTACTTGCACGAGGCAGCTATCCTGTTCAATATCAAGAAGCGCTTCCTCTGCGAGCTGCCCTACACGTACACAGGCGACATTTGTATCGCTGTCAACCCCTACAAGTGGCTCCCAGACCTGTACGCCGAAGAGCAGCACTTGAGGTACCTCAACCAGCCCAAGGAGGAGTTGCCCCCGCACGTGTATGCCACATCCGTCGCTGCCTACGACAACATGCGACGCTCAGAGCGTAACCAGTCCATTCTGGTCAGTGGTGAGTCCGGTGCCGGTAAAACGGAGACCACCAAGATCCTTATGAACCACTTAGCCACTATCGCCGGTGGTCTCAACAACTCCACTATCAAGAGGATTATCGAAGTAAACCCACTGCTTGAGTCCTTCGGTAACGCCAAGACTGTGCGTAATGACAACTCCAGTCGATTTGGCAAGTTCACACAGCTGCAGTTCGACAAGAACGGCACGCTTGTCGGAGCCAAGTGCCGCACGTATCTTCTGGAGAAGACGCGTGTCATTCAGCACGAAGCGCCTGAGCGTAACTACCACATCTTCTACCAGTTGACTCCTCCGACATTGCGTCGGAGTTGCAGCTCGACTCGTCAAAGCACTATGTGTACACGGGCGACAACACGGCACAGAAGATTGAAGGACTTTCTGACGAGAAACACTTCAACAAAACACGTGAGGCGCTGGAGCTTGTGGGTTTGTCTCGCGACGACCAGCGGCCACTGTTTGAAGTGCTGGCTGGTGTGCTCCACCTCGGTGAGGTGCAGTTGCAGTCGGACCCGACAGATGACGAGAAGTCTCTCATCACCGAGGGCGACAGTGGTGCGAATTCCGCCACGCAGATGCTGGGCGTCACGTGGGAGGCCCTTCAGACCGCTCTGTGTTCGCGTACCATGCGTGCTGTGAACGATGTGTACAGTGTACCGCTCAAGAAGGAGCTGGCCATGGACTGTCGCGACGCTCTTGCTAAGGCTATCTACTCCAACGTGTTCGACTGGCTTGTGACTACTATTAACCAGTCGCTTGCTGACGATGCCAACATGGCCAACCACGTGGGTGTGCTCGATATCTTCGGTTTCGAGCACTTCAAGCACAACTCGTTCGAGCAGTTCTGCATCAACTACGCCAACGAAAAGCTTCAGCAAAAGTTCACTCAGGATGTGTTCAAGACCGTGCAAATTGAGTACGAGGAGGAAGGAATCGTGTGGGACCACATTGAGTACGCAGATAACCAGGACGTGCTGACTGTGATCGAGTCGCGCATGGGTATCATTTCACTGCTGAACGAAGAGGTGATGCGACCGAAGGGTAATGAGGAGTCGTTCATGTCCAAGGTGACGTCCTTGCATAAGGAT---GATATGGCGCACGTTATTGAGTTCCCTCGTACGTCGCGTACTGAGTTCCTCATCAAGCACTACGCCGCTCCTGTCATGTACGACTCGGTGGGATTCCTGGAGAAGCACAAGGACTCACTGTTGCCTGATCTGTCCGAACTTATGCGTGGTTCATCCAAGCCGTTCATCGCCAAGCTCTTCGAGCCGAAGCCTGAGCCTAAGTCGCCGGAAAC---------GTGGTGGTGCTCTGTCCATCACGACTGTGGGTACCCAGTTCAAAGAGAGTCTGACGGAACTGATGGCTACTATCAACTCGACTCGTGTTCACTACGTGCGCTGCATCAAGCCCAACCCGATCAA---------GAGCGCGACTGCCATGGACCAAAACATGGTGGTGTCCCAGTTGCGATGTGCCGGTGTGATTGAAGCCATTCGTATCTCGCGTGCAGCATACCCGAACCGGTTGCAGCACACGGAGATCCTTGACAAATTCTGGCTGTTCGTACCGAGTGGTGGTGACACTGTTGCAGACAAATGCCAACTGCTTATGGACAAGCTGAAGTTGGAGTCCCCCACTCAGTACCAGATGGGTAAGACCCGCGTGTACTTCCAGCTAGGTGTGCTCGAAGAGCTTGAGGATCGCCGTAAGAAGTTTCTGGATGCCAAGGCCACATACCTGCAGAACATCATGGTTGGATTCACCCAGCGTATCAAGTACCTCCGTCAACTGGAAGCGATCATCAAGTTCAGTGATTCGGTGCGTGATTGCTATGCGCCGATACAACACGTTCCTCAAGGGCCTTATCTCGGCGCAAGCTCACTGGCGAGGCATGCAGGGCCGCAAGGTGGCTGCTGAAGTCAAGAGCAACCACAACGCTGTCATTATCCAGCGGTATGTGCGTGGTTTTGTCAAGCGTCACCGTTACGTCCTGATGCGCGAGATGGTTATCCGTGTCCAAGCTGTGGTTCGTATGACGATCCAGCGACCGAAGTATCTTGCTGCTCTTGAGGAGAAGCGTCGCGAGGCCGACATGGCATACCAGCTGCAGAAGCTAAAGGCTGCTCTGCAGGAAGAGCAGGAGCGGAATGCGCAGCT------CCAGCGCC---GCAGCAGTGTGGC--GACGGCACGTCCGCTGCCTCGAGCGTTGTGATGGCTGATGCTGGCGGTATGATCGAGACTCTCACTGACGAGAACAAGAAGCTGCGTGAGAAGAACGAGGACATGAAGGTGACAATGAAGGGCTTGAAGGCCGAGATCGAGAAGTTTAAGAGTGACAAGGAGTTCTCGTCGGCTGGTAACCACGTGAAGGTCCGTCAACTGCAGGACACTGTGCGTGACAAGGACAAGAAGATCTCGCAGCTCGAGGCCGAAAACAAGAAGCTGACGGAGCAGATCGCCAAACTTCACGCTGAGGGTGTTGT---TCCTGAGAAGAAGCCCACACCGAAGAAGTCGATCTTCCGCACGCTGGGCTCCAAGAAGGAGAAGAAGCCAC---------------GCGAGACTGTGCTCATGGACTCGCTCACCGGCTCGGACGACTTGAAC---CGCGAGACGCGTCCGTCGGA---GCGCCACTTCCCTCGCATGC---CGAGCATGAGTGTGGGCTCT---CGCTTCTGGAACTCGAGCAAG---AAGGGCGAC------------AACGGCGACGGCTTCGACGTCTCGGAGGTTGATGGTGAGGACAGCCCCACGAACCCACGCGCAACACGATCGTCAAGAGCATGGAAGTGGGCGTGACTGGTGCCATGTCTAGCCTCAAGGGCCGCATG---------TCGGTTGTCAAGGGGATGTACGAAGCAAGCGCACGAACAGGACCGCCCCGTGCGCGAGTCGTTCAACCTGGACGCGTTGCCGGAAGTCTCTCTTCCTGTCGGCTGGGAGGCCAAGGTGTCGCGCTCCACGGGTCGTGTGTACTACGTGAACCGCAAGCTGGGCAAGTCTCAGTTCGAACGCCCCACGCTGGCCTCTCTCAA------GGCACAGAAGCTGGCGCGCCAGAAGTCGGCTAGCGGACTGTAAGGCTGTGGTCGCTGATTTTGGCTGTGGAGATGCGAGATTGGCGGAAAGCGTGCCGAATAAAGTGCACTCGTTCGACTTGGTGTCCATGGATATTGGCGTTTACTGCTTGGCGCTCATGGGGACGAGTGTTCGTGAATATGTGCGGGAAGTGTACCGTGTGTTGAAGCCTGGAGGCGTGCTGAAGGTCGCTGAAGTCAAGAGCCGCTTCGAGAGTGAATCGCTCGGAGGATGGTGGCGGTGTTTGTGGCGGTGGCGGAGGGCGTTGTGGTGACGCTACGCAACACCACGGTGTTTGTAGAGAGACCAAATACGCAATTGTGGTGGGTCAGAAGACGGGCGAGGCTGTGGCCGTGCCCTTCCCGGACGTGGACCGCGACCTCAAGACGTTGCTGGGCCACACCACATCCATGGTCACTCACGTGGCCGTAAACCTCATCACTGCTGCTCACGGCCGACCGAGACGAGAAGCTGCGTGTATCCAGGTTTCCGAACGCTGCGGTCATTGAGAGCTACTGCTTGGGCCACGCTGCCAGTCTGACAAAAGTGGCTTGCAGTGCTGTGACCCCAGACCTTGTGGTGTCCACATCCATGGACAACACCTTAAAGCTGTGGCAGATGACGACGGGCAAGCTGGCCATTTGCCCCAAGGCGACGTTGTGGCCGTGCTTGTGAACTACCAGCATGTGCGATTCTTCGAGGTCGTGAGAACCCATTCCTGCAGTTGTTTTCGCTTTCTAAGGGTGAGCGCTGCATTTAACCGCTGTGCGCGACTTCCGCAAGCTTCAGAACGACCCGCCGTCGGGCGTGTCAGGCGCCCCCATGGACAACAATATCATGCTCTGGCAGGCCGTCATCTTCGGACCGGATGACACGCCCTGGGAAGGCGGCACCTTCAACCTGACGCTTGAATTCAGCGAAGACTACCCTAACAAGGCGCCGACTGTCAAGTTCATTACGAAAATGTTCCACCCCAACATCTACAACGACGGCCAGATCTGCTTGGACATCCTGCAGAACCAGTGGTCTCCGATATACGACATCTCGGCCATCCTCACGTCCATCCAGTCGCTGCTGTGCGATCCCAACCCGAACTCGCCCGCCAACTCGGAGGCCGCTCGCCTGTTCCAGGAGAACCGACGGGAGTACAATCGCCGCGTGCGCGAGATCGTCGAGCAGAGCTGGCAGGCCATCGATGGTGTCGGCCGCCAAGATGCGTGGCGCTGAGAACCGCATGAACGCTGGCCGCCCCTTCACCGCGTGGCTGGACAACGTCTCGTCGGACCGTGGTCTGTGCGGTGGTATCAACTCGGGTATTGCCAAGACCACCCGCAAGCAGACCGCTCGCAGATCTTCGTGATCGGTGACAAGGCCCGCGCTCAGCTGCGTCGCGACCTTGGCGACAACATCCGCGGCAACGCCACGGAGACGTACCAGTCGCCCCCCAACTTCACGGTGGCCAGTGCCATCGCTGAGGCTGTGGAGTCGGAGAGGAGTCGTCCCGTATGGCTGCTATGGAGAACGCTACCACCAACGCCGAGGAGCTCACGCTCACGCTCGTGTACAACAAGGCTCGTCAGGCCCGTATTACCACCGAGCTGATTGAAATTATTTCGGGTGCCGCCTCCCTGGACAGTGACTGGCAGCGTAGTGAGCGTCCAGAGCGCGGCACAGTTCGACGAGGCGACTGCGCGCGAGTCGACGCTGTCCGTGAGCTTCTTCTGGGCCGAATTCCAGGCCTGTCGCCCCAATGGGCAGCTGGACGTGGGGCCGTGCCGTGCTGGACAAACTCGAGGGCGCCAATGTGGCCGAACTAGCCAAGCGCGTCTCCAATACCGCCTCAAGAAGCTCATCAGCGCGTCACCGGTCATGCTCTTCATGAAGGGCAACCCCACGGAGCCCAAGTGCGGCTTTAGTCGCCAGATGGTGGCTCTCTTGAACGAGGAAAAGATCCAATTCGGCACCTTTGACATCCTGAATGACGACGAAGTGCGCCAAGGACTCAAGCAGTTCTCCAACTGGCCTACTTACCCGCAACTTTACGTGAACGGCTCGCTAATTGGCGGTCTGGACATCGTCAAGGAGATGAAGTCCGAGGGATCCATCGTCGAGCAACTGGGCGTCGAAGAGGCTGAAGCTGCCTTCCAAGAGAGTCTCCGTGCACTCGTCAACTCAGCTCCTGTACTACTCTTCATGAAGGTTCGATATCTTGTGTGGAGCTACCAACGTCAGGACCAAGACATCGCCAAGGTTGTGACTCTCACGCCACCTTCATTCCGTCGTGATATGTGCCGAGGCCATCTCATGGAGTCCGCAACCGAATGGCGATGGCTTTCCGTGATCCTTTGGAGGAAAAGGGAGAGTTCTGCATTGTGCAGCTCCCCGTATGACATCAAAGCGCACTGTTCTGCATTCACGGGGTATTTCTGCGATCGACTGGGAAGTTTACTGTGCTGCACCGAGACCACCGCAGTGAAGTCAAGTCGCTCTGTGTTCACTCTCAACGCGACTGTCTGTATACTGGTGGCCTTGACGGATTGTTGATCCGCTACGACATCAAGAAGGGTCGCACTGAAGTTGTGATGGAGCGTCGCAAACGCATCTCCAAGATCAACGCGGTGCTGGAGCATCCACACAATCCAAATCTCCTGTTGGTCAGCTCCGTGGAGCAGTAGACACAATTTGTTGATGCACGATCTGCAGCGCTACGACCGCAACGTGTCAGGAAGCTCCATGAGTCAGTATGTTGTGCCCCGGTGGTCGCCGGCAGGTTATCACGTCTCGTGTGGCAGCAAATGCGATACTGTCACGGGAGTTTCTCCACGCCCACGTCAGCCACCATCGGAGCCTCGTTCCTGCAGAAGCGCGTGATCGTCGGTGGGAAACTGACGCTGCAGATCTGGGATACAGCCGGCCAGGAGCGCTTCCGCTCCATGGCGCCTATGTACTACCGCAACGCCAAGGCAGCTATCCTGGTCTTTGATCTTCAGAGCGAGGCCAGCTTCGAGAAGATCAAAGAATGGCTCCACCTACAGCATCACGTGGGTGACGACATGTGGGCAACAAGAGCGACGTTTCGACTTTGCCAAAGCTCAGCAGTTTGCAGATGAGATCGGTGCGCTGGTGTTCCGCACGAGCGCCAAAACGGGCGAAGGAGTGCAGTCGCTGTTCGAGAGTATTGCAGTGCAGCTACTTAAGAAACACGATCAGGAGCAGCGAAGAGCTTCTACGTGGTATATATCCCGGCGAACGAGACCAAGGAGCTGGAGGAATGGACGGTCAAGCTTCCGCAAGACAAGGAGGCGCAACTTGGCTGTTTGACGGAGCGTCTGCGCGAGCATTTTAAGAGCGGCGCCATCGAAGCAGCAGCAGAGACTTTCAAGCAGCAATTACTGGCCCAGCTTCCCAAGGGCGCCACTTTGACGGATGAAATGCTGCAGATGATGTATGGACAGCCTTGTAGATTCCATTCCGCTCGTGCTCAACACGCCCGACGTCAAGCACATTGGAGGTCAAGGGCGATGCATTCATTGGTCGGGTGTTCGACAGACGACTTTGTGCGAATGGATTTCCGGTTGAGTGAGGTGTCAGGTGACGCGCCTTGGGTACAGACAGCCAAGTTGCAGAGGTCACGCAGTCGTTGGAGGAGATCTTGGCTCTGGGCCAGGGTCTGGATGACGAGGACTACAAGGCCGAGCTCGAGGTGTTGCAGAATGACCCCAACTCCAACCTCTACTCGGCCGTCACCTTCGAGGAGCTCAACCTGCCGGAACCGCTGCTCAAGGGCGTATACAACATGAAATTCGCCAAGCCGTCCAAGATTCAGTCCGTGGCGCTGCCGCTTATCCTCGCCGATCCCCCGGAGAACTTGATCGGCCAGGCTCAGAGCGGCATCCCACCCGTGAGTTAGTGCGACAGATTATGGCCGTGGTGAACGCTATGGGTAAGTTGTGGACACGTTCCTGGCCATCCCCGGCAACGATCTGGCTCGGGGTGAGACTTTGAAGGCACAGGTGATCGTCGGTACCCCCGGCAAGATCGAGGGTATGATCAAGAAGAAACAACTGGACACTCGTAACGTCAAGATCTTCGTACTGGACGAAGCTGACGTCATGGTGGCCGAGGACGGCATGCGCGAACGCTCGGTGTCTATCAAGCGATTCATCAAGAACCGCGCGTGCCAGTACCTGCTCTTCTCGGCCACATACGCTGACGATGTGCGTGATTTCGCCCAGAAAATGGTGCCCGACCACAACATTATCACGGTCAAGAAGGAGAAGCTCACGCTCGACGGCATCAAGCAGTTCTGGATCGACTGCAAGTCCCGCGAGAACAAATTCCAGGTGCTATCGGACATTTTCGCCATCCTGAGCATCGGCAAGTGCGTCATCTTCGTGCAACAGCGCGAGACTGCCAAGGAACTGACGCGTCGTATGCGTGAGAAGGGCCATTCGGTCGGTATCTTACACGGCGCCGACATGGCCAAGGAGGTGCGTGACCAGGTCATCGACGAGTTCCGTGCCGGCACGACCAACGTGCTGATCACCACGAACGTTCTGGCTCGTGGTATCGACGTGGCGGGCGTCTCACTTGTCGTCAACTTCGACATCCCGCTGACTCGCGACCACCGTCCCGACCCGGAGACGTATCTGCACCGTATCGGCCGTACCGGTCGCTTTGGCCGCAAGGGCTGTGCTATTAATTTCGTGTACGACAACAAGAGCAAGCAGGACCTCGCAGAGATTGAAGAGTACTACGCACGTCCGATCACTCAGGCCCCTGCAGACGACATTGAGGAACTCGAGAAGCTACTGGCTTGGGGGGCAGCAAGCGCCGCTTGGCCAAGCTCGGCAAGGAGCAGCGCAAGGGCATGCGAATCTACCCTCGCGAGCCCAAAAAGAAGCCCTCGGGCAAGGATAAGACGTACTACTACCTCAAAGACGTCATGTTCCTGGCGCACGAACCGGTTCTTGAAAAATTCCGGGCGTTTAAAACGTTTATGAAAAAAGTGAAAAAAGCCATGGGCAAGAAGGACTACGAGGACGCCAAGCGCAAGCACGAGAACCGCCCTACCTACGAACTGGACCACATCGTTAAGGAGCGTTATCCTCGCTTTGTGGACGCTCTAGGCGATCTGGACGACGCGCTGTGTGGCAGAACTACATTGTAGCCACCAGAGCGCTGACCAAGGTGTTTGTGAGCATTAAGGGCATCTACTACCAAGCCAACGTGCAGGGTCAGACCATCACGTGGCTCGTGCCCCACCAGTTCACCCCTACAGTGGACAAGCGTGTGGATGTGCGTGTCATGCTCACATTCCTCGAGTTCTACGAGGTTCTACTCAAGTTCGTGAACGCCCAGCTCTACATGCAGATCGGCGTGGCGTATCCTCCCAAGATCAACCTCCAGTTGGACGCTGCGGGTGCTCAGTTGGCTGCTCTTGAGCTAACAGCAGAAGGAGTCCGAGAAGAGGATCAAGCTACAGCGAGAAACGCAAGGAACGCAGCGAGAAGAGCTTGTTCAGTGGCTTGACGTTCTTCCTGTCGCGTGAGGTGCCTGCTGGTTGTCTGGAGCTGGTTCTACGCTCTCACGGCGCCACGTTGGGCTGGGATGGAGAAGGCTCTCCGTTCGACGAGAAGAGCACTCGTATCACGCACCATATCATGGACCGGCCGCACCAGGGTCATCGCTACTTTAACCGCGAGTACGTGCAGCCGCAGTGGGTGTTTGACTCGGTGAACAACGGCATATTGCTGCCTTTGACGCGTTACAGGAAGGAGCTGGACAAGCTCAAGTCGGCGTCGCAGGTGTTGCGTGAGGTTGGAGCTGTGGGCGATGAAGAAGAAGACGGGAGGCCTACGCTGCTGATCTTAAGGCTGAGATGGATGGTCACAAGAAGTCGAAGCGTGTTAAGAAGGAAGAGGAGGCTGCCGAGCTCAAGGAGCTGGCCAAGACGATGATGTCGAAGAAGGCCAAGCGTCTGTATGACCGCATGCAGTACGGTCTGGGCAAGAAGGCCGACAAGATTCAGAAGCTGGAGGACAAACGTGCCAAGATCGAAGCAAGACCAAGAAATTGTTGGCGGGCGCACTGTCCGGTATGATTTCCGCGTTGGCCTTCCAGCCTCTGGGGCGCTTCGCTGGCGCGGCACCTCGTCCACGCTCATTCGCGTCGCGGGCGGCGCCGGGCTCTACTTCGCCACTCTGGACCACTGCCTCAACCTTTTCCCACAAACTGCAGTCAACACCTTCCTGGCAGGAGCTTTTGCGCGTACCTTTGCAGGTGGTGTCATGTCTCCTTTCACTATTGTCAAGGCGCGAATGGATACCGCCTGGTACTTTCGATAGCAAAGGGACGTGCCGTTTTCAGGACTGTATGTGCTCATATACATGGCATCCTGCCGATGTGGTAAAGACACGCATGCATGCGACAGACTGTGGCCAAGATCTATCACCACGAAGGTCTGCGTGGCTTCGCAAAGGGAATCCTTCCACGTGTGATCAAACGAACGCTGTCCACGGCAGTCACGTGGACAATCTACGAGCTACTCGTTCGACCCATCGGGTCTGGGTCATGCCAAGGAGGCTTTCAACCTAGCCAAGGAGACGGAGCGCACCAAGCAGATGGAGAACCAGGCCAAAATTAAGGAAAACGAGGCGCTTTACAAGCAGTACGAGATCGTGCGGGTGCAGAAGGAGGGTGAGGAGCGCCGCAAGACGCTCGAGGAGGAGACGCGCCAGCACCAGCAGCGCGCACAGTACCAGGACCAGCTTAAGCGCAAGCAATACGCTGACCAGCTGGCCGCTCAGAAGTACATGAAGGAGCAGGAGCTTAAGAAGCAGGAGGAAATTCTGGCCCGCCAGGAGGCTTCGCGCCGTAAGACGCTCGACTACGAGGCGGAGCTGCGTCAGAAGACTGAGCTTGCTAAGGTTGCGGCTGAGACTGAGGGGCGCATCAAGCAGGAACGTCTCAACCACGACCTGCACCTCGAGGAGGCTCGTGTGCGTGCCAAGGAGTACCGCGAGACTGTCATGGAGGGTATTAAGCTGGCCGGTAACACTGTCGGCTCCGGTATCATGACCTTTGTGGACGACAAGGAGAAGCTCACGACCACTGTGGTCTCTCTGACGGCTCTGGCGATCGGTATCTACACCGCAAAGGTGTCCACCAGCGTTGCGGGCAAGTATATTGAGGCTCGTATGGGAAAGCCGTCGCTAGTGCGTGAGACGTCGCGTCGCTCAGTTACGCAAGTGTTGGCCAACCCCATCCCGTCCATCAAGCGCGCTCTGCGTCTACAGAAGGCGACGGATGCCCTGGAGGGCGTCGTGCTGGAGCCCAAGCTGGACGAGCGTCTGCGCAGCGTGGCTGTGTCCACGTTCAACACGAAGAAGAACCGTGCGCCGTTCCGCCACTTGCTACTGCACGGCCCGCCCGGTACGGGTAAGACTCTGTTCGCCAAGGCACTTGCTCGCCACTCCGGCCTCGAGTACGCTATCCTGACAGGTGGTGACGTCGCCCCACTGGGTCGGGAGGGTGTGACGGAGATTCACAAGCTGTTTGACTGGGCGTCCCACAGCCGTCGCGGTCTGCTGCTCTTTGTCGATGAGGCCGATGCGTTCCTGCAGAAGCGTAGCAACACCGTCATGAGCGAGGACATGCGTAACGCACTGAACGCCTTCCTGTACCGCACGGGTGAGGCCAGCGACAAGTTCATGATCGTGTTCGCCAGTAACCAGCCAGAGCAGTTCGACTGGGCCATCAACGATCGTATCGATGAGATGGTGGAGTTCCGCCTGCCTGGATTCGACGAGCGTGTGCGCATGCTAAAGCAGTACTTTGACGACTACATTCGTGCGCCGAAGAACTCGCGTGCCAAGAAGATCTACGCGAGGGCGTCATCTTCACGCTGGACCCTGTGGCCAACTTCCTCGTGCCAAGACGCGTATCTTCCAGCTTGAAGCGTTGCAGGGCGTGTCCGGTGAAGCTCAGGCTATTTTCGACGCACTCAACAAGAACCCATGCGCATCTACTGGGTCGTATTCGTTGTAGTGCTGGGCAACGGTCTATATACGTATTTCGCCGGCAAGGACGAGTCGTACCTGGTTGAGAAGCTTAAGAGAAAAATCCCGTCCGAGTACCGCATCTCCGAGAAGTGGGACAAGTGCCTGGAGAACTTCACGCTCTACTTTGGCGCTGGCCTCGTGGCTGGTGGCCTTACGTCCCTCGTGTTGGCTCGTTCTGGAGCTGGTCGCGGACTGGTGACGGGTCTGGGCGTGCAGGTGCGGGATCGAGCTGGACGACGTGCCAACTGGCGTTTTCGGGTAACACACAGCAAGCTCTGAACAAGACGGACAAGGCTGTGGGCGACTTGCCGGCCTTCCAGCAGCAGACGATCGTGGACTTCGTGACGGCCTCGGCCACGCAGCAGCAGCTGCAGGCCGTTCATGCGTGGCTTCTGCGAGGAGTCCAGTGTGAAGGTCTTGGAGACGTATCTGGCCGAGCAGGTGCAGAACAAGACCGTGGACATTGACGCCAGTCTGGCGCTGCTCAAGCTGTACCAGGTGTACCCGGCCACAATCAGCGCCGAGAGCCAAGATTCTGGTCAAGGGCGTCATGGCCCTGCCCTCCACATTCTTCACCGGCGTCCACGATGGTCCCGGAGAGCATCCGCGAGGACGCCAACGTGACGGCAGTGCTTCACACGGGTTTCATGCTCCAGTCGTGCCTGTTCGAGGATTTCTGGAAGGAGAGCGTGACTTTCGCCGAGAAGGTGCCAGGCTTCCTGGAGTCGGTGCGCGCCTACATTCTGACAGCTATCAGCCGCAGCCACAGCGCCATCTCCACGGAGGTCTTCAAGGCTAAACTCAACGTGTCTGACAAGGAGGTGGCCGACATCGTTGCCGAGAAGTGGACGCCGACAACTTGATTCAGATCAACCCCAACGAGGACAACCAGATGCAAGCCAAGAAGGTCCAGGAGAACATTGAGTTCGAGGACGTGCTCAAGGTCATCCACACTCTTTCTAGATCCAACGTCTGCGCCAGTGGCGGTGGCGTGGCTGATGCTGAGGAGGTGTGTGAGAATCTAGCGAAGCACTTGAGACTGGACGGAGGCTCCCAGGTGTTGCCCTGCTCTACGGGTGTCATCGGTTGGCGAATTCCTGTAGACGCTATGGTCGAGAACCTTCCGAAACTCGTCACCAATCTGCAGTCCAACTCGGTTCTACCTGCTGCTGAGGGCATCATGACCACCGATTTGTACCCGAAGATCCGCTCCATGGACGTGTGCGGTGGCCGCATTGTCGGCATTGCCAAGGGTGCTGGTATGATCGAACCCAATATGGCGACTATGCTGTCCTACATCCTCACCGATCTATCGATTCCGCGTGACGTTTTGCGTCAGCTACTGACGGAGGTGGTTGGAGACACGTACAATTCGCTCAGTGTGGACACGGACCAGAGCACGTCCGACACGGTGACGCTCGTGTCGTCGGACCAGATCCCGTTCGACATGAC---ACAGCTGGAAGAGTTCAAGAAGGCACTGCATGAGGTGTGTCTTGGACTCAGTGAGGACATTGTACGCAACGGTGAAGGTGCGCACCACGTGATGCGCGTGCGTGTAAGCGGTGCGTTGTCTAAGGATATGGCTAAGGGTGTCGGCAAGTCGGTGGTCAACTCGCCACTGCTCAAGTGTGCGGTGGCAGGCAACGACCCGAACGTGGGTCGTCTAGTTATGGCTGTGGGTAAGTACATGGGATCGTACCACAAGGATGTGAACATTCAACGTCGCATGAAGATCCGAATGGGTGGTGTCGTCATCTTCGAGAACGGCGAGATGGTGCTCAACGATGATATTGAGGCCAAGTTGGTGGCCCACATGCGCGACGCGATGCTGATTGAGCCCACTCGCGGTGGACTTGATGCCAGCTCACACAACTATCCGCCGCACGGAAAGTTTGTCGAGATTGAGATTGACCTGGGTGTTGGCTCCGAGGAGACTCAGGTGTTGGGCATTGACCTTACGCACGAGTATGTGGCCATTAACGCCGACTACCGCTCGTAATGGCGCC---GAAAAAGGGGAAAACTGCCGCCGCGTATCTGGCGTCTCTGGGTGTGGCCCCTGC---TGCTGGAGGTGAGAAGAAGGACGAT---AAGAAGAAGAAGAAAAAGGGCAAGAAGAAACCCAAAAGAAACCCATGTCGGCTGCGGCCAAGGCTCTGA-TGGAGC------------------------------------------------------------------------------AGGTCAAGCGTCTGGCCGAGCTCGAGCGCGAGGAGGAGATCCGTCGCAAGAAAAAAGAGGCGCGCGCCGCTAAGATCGAGCGTCAGAAGAAGGAGGGAACGTACATGACCAAGGCACAGAAGCAGAAGGCTGCCAAGGCTCAGGCCGCGCTTGAGGCGATGCAGCAGGCGGGCCTCGTTCCTGAGGCTGCC------GGCGAGAAGAAGAAGGTTGTGTACTCGGACAACAAGAAGAAAAATAAGAACAACAGGAAGAAGAGGAGGAGGAGGCGGAGGCTCCCGATGCGTGGGATGACGAGGACTGGGAGACCAGTGACGTTGTGGCCTCGCTGGAGGATAAGCTAACAGAAGAAGGAGCAGGAGCGTCTGCGTCAACAGGGCATCGCTCTGATGAAACGCCAGGAGGAACAGGCTGAGCAGATGCGTCAGGAGGCCGAAGAGGAGCGTCAGCGCTCCAAGGCCAAGCTCGAGGCTGAACGCAAGAAGAAGGAGGCGGCCGAGCGTCGTCGTGAGCGTGAGGCCGCTGCGCGTGCCAACGTGTCCCTGGACAACCTGCGTTCGCCTATTTGCTGTATTATGGGCCACGTCGATACTGGTAAGACGAAGCTTCTTGATAACATCCGCCGGACCAAGGTGCAGGACGCCGAAGCTGGTGGTATCACGCAGCAGATTGGTGCGACCTTCTTCCCTGTGGAGGCTATTAAGCAGAAGACGGCACGCCTTGCCGAGACCATGAAGCTCGAGTACCGCCTGCCCGGTCTGCTTGTGATCGATACGCCCGGTCACGAGTCGTTCACGAACCTGCGTACTCGTGGCTCTTCGCTCTGTGATATCGCCATTCTGGTGGTGGATATCATGCACGGTCTTGAGCCGCAGACTCTGGAATCTCTGCGTCTACTGCGTCAGAAGAAGGCCCCGTTTGTGGTGGCGCTGAACAAGATCGATCGTTGCTACGGCTGGAAAACTATGCCCGACATGCCCGTCCAGGAGGCGCTCAAGCATCAGAACGAGCACGTCATTCGCGAGTTTGAAGATCGCATGAAGTCGATCATTGTGGAGTTTGCAGAGCAGAAGCTGAACGCCGAGGTGTATTGGCGCAACAAGGACTTGGCGCACACGGTCTCGCTCATCCCGACTAGTGCTATCTCGGGTGAAGGTGTGCCGGATCTGCTGATGATGCTGACGCGATTGACACAGGAGCGCATGGCCAAGTCGCTGGCATTCGTCGACATCCTACAGTGTACCGTGCTCGAAGTGAAGGTCATTGAGGGTCTTGGTACAACGATTGATACGATTCTGGTTAATGGTACACTAGAGGAAGGCGCAACAATTGTAGTGTGTACATTGGACGGACCTGTTGTGACAACGATCCGTTCCTTGCTGACCCCTCACCCCATGAAGGAAATTCGAGTCAAGGGCGAGTACCTTCACCACCAGAAGATGAAGGCTGCTATGGGTGTGAAGATTTGCGCCCAGGGCCTCGAGAAGGCAGTTGCTGGTACTCAGATTCATGTGGTTGGCCCCGATGATGACGTTGATGAGCTGAAGGACTCCGTCATGTCCGATCTGACTGGAATTCTGGACTCCGTGAAGGCTACCCGCCGCGGTGTCATGGTTCAGGCTTCCACCCTTGGTGCCCTGGAGGCACTACTGGAGTTCCTGCGCACGTGTGACCCGCCCATTCCTGTGTCCTGTGTGAACATCGGTCCTGTCCACAAGAAAGACGTCATGCGTGCTTCGGTGCAGTTGGAGCACCAGCCTGAGTTTGCCACCATCTTGGCGTTCGATGTGAAAGTCCATAGTGACGCTACTGAGCTGGCCACTGAGCTTGGTGTGCGTATTTTCACGGCCGACATCATTTACCACCTGTTCGATCAGTTCACGGCGTACATGGACAACTTCCGCATGGCCCGTCGCGAGGAGTTTGCCGAGGTGGCTGTGTTCCCGTGTGTGTTGAAGATTTTGCCCAACTGCGTCTTCAACAAGAAGGACCCGATTATTCTTGGTGTGGACGTCGAGGAAGGTATCCTCAAGGTCGGCACTCCGCTGGTGGTGCCGTCCGCTGGTG---GCTTCCTAGTCGGTAAGGTTGGCTCCATTGAACGCGAGCACAAGGAGGTGGATCGCGCCAAGAAGGGCGCGTCCGTGGCCGTGCGGATAGACAACGAGAGCAGCGTCATGTACGGCCGTCACTTCGACCACAAGAACAAGCTAGTGTCGCGTCTGACACGCCAGAGCATTGATGCCCTGAAGGAGAATTTCAGGGAAGACTTGCAGAAGGAGGACTGGCAGCTCGTCATCAAGCTCAAGAAGGTGTTCGATATCATTATGGAGGGCTGGCTCACCAAGCGCCGCGACCACATGAATCTCATCTGGCTGGATCGCTACTTCCTCCTGGACGGCAACCAGCTCCGCTACTACCGCAAACGCGGCGACCCTGCACCTCGAGGGACCTACATTCTGACTGACGGTTGCGTAGTGAGTCCCGTGTTCAATTCCGAGGAGAAACACAAACATCACGGACCCGTCTGGATGTTCCGCATCACTTGAAAAGTCGTACAAGAAGGACCGATTCCTGGATCTAGGAGCCAAGAGCGAGGAGAACGCCAAGGAATGGAAACGCGCGCTGGAGAACGCCGTGCGGTCGCTGCAGGAGCTCGTAGCGCACCAAAAGAAGCTCAAGAACGACGCCGAGAT---CTTCCCCACGCCCACCCAC---------GTGCCGAGGATGTTGAAGGCCGATGAAGACACGATGTCGCTCTTTAATCTGGACGCAGACAACGTCAAGGCAGANCAGGCCTGGTGGCTAGCCAGAGTGGAAGGAGGGCTCAGAATCATGCAGGAATGTCCTCTCGGACCCAACGTTGCGGCCAATCTGTACGCTCAACACGCAGCGACAACCAACGGACTGATCTTCAGTGCATGTGTGGGCGCAGCGCTGAGTTTCAGCAGCATGTATTCGTTCGCGAACAACATGTTGCTGAGCTTTATGACTGCGCTAGTGGCTATTGCTGTGTGCTGCGCTGCAGTGACGCCCACACAGGCTCCTGAGATCCCGAGCTTCCGTGTGTCCCAGGTGGTGCATGGCTCCCCGACTGAAGTCTTCAGACTCATCATGAACTCCAAGAGATTCCAGAGATGGGACTCGGCCACGGCGACTATGCGCGTGATCCAGCAGCTGGACGACCACGCGGACATCGTGTACATTACGCAGCGACCGACGCACTTGTGGCCATTGTGGCAGAAAGCTCGAGACCTTGTGCTCATGCGCTACTGGAGACGCGAAGAGGATGGGTCGTACTTTGTCATGTACCAGAGTATCGAGCATCCCGAGTGCCGTGTACGTCACAATTATGTGCGTGCGAACTTGCTGGGCGGCGGCTTCGTCATCGCTCCTCAGCGTGTTCCCAGT---GGATCTATCCGTACTCTGGTTACCTACGTGCTACGTTACGATCCTGGAGGCTG---GTCCCGTATCTACCATCAGCTCGGAATGGATGTGGATCTCGTGATGCCGATGCTGCGTAGTGTCGTGGGGATTCGGGACGAAATGAGCGCTACGGACTTCATCACGCCCAATGTATCAATGGCTCCTGC---AGAGAACGGAGACTGGACCGGAAGTGTGGA---CTCGATGGGTGTCGTTATGGGCGTACAGAAGCTGCAG---ACCAGTCTTCCCGAGAAGATGTGGGGCGAGCCGGACGCTTCGCTCTTCAGTGTTCGTGGGCATAACTACTTGACCGACAAGAAGAAGATCCCGTCAGCGCCTGCGATGTTCCACACTGTGGGTGTGGATCTCTTGTCGTTCGAGAGTCCAGCTGAGAGGTACAACATCTCGTCTCGACCGGACAGTATCGGTCGCACCAG---CTCCAAGTTCACGTTCGTAGTGAACATGATCATCCCTGGCCCAGAGAACGTCTGCATGGTCTTCTACTTCCAGCCAGTGCGTGACAATGTGTTCGAGGACGGCTCGGGCTTCTCCGAGCTGCTGAACGACTTCTTCGACGGCGACGACCAGTTCCGGAACTCGCGCTTCAAGTTGATTCCTACGGTCGTAGAGGGAAGCTTCATCATTAAACAGTCGGTCGGTTCCAAGCCCACTCTACTCGGCAACAAGCTGAAGTGTCCGTATCATCGTGGAGAAAACTACTTTGAAGTGGACATCGACATCTCGTCCAACTCGGTTGCCAACACGGTGGTGGGGATGGTGCAAGGCGTGACCAAGTCGCTGGTGGTGGACTTGGCCTTCCTGCTCGAAGCCCAGACCGACGAAGAGCTGCCTGAAGTCATCTTGGGCGCTGTCCGCATGCAGCACATTTCGCTGGACAATCCACGCCGCATGCCCAAGCATGGGGAACGGGCAGCAAGGACGAGTTCACGTTCCGAGGCCTTTACCGAGGCTCTGCGTGTGAATCTGCAGCGACTCATCGTCTACGCGCGTTCGGCCGATACGGCGCTGCAGCGAGAAGTCGCCGAGAAGCTGGCAAATGAAGCAGTTAAGACCGACAGGTGCAGATTGTGGAGCTGGACGGACTGCAGCTGCTGCTGCCACTGACCAAGTCGAAGGACACGGAAGTGCAGCGTCTAGCAGCCCACGCACTTGCCAATCTCTCAGTCAACTCTGAGAACCAGTCCAAAATGGCCACGGAAGGAGGTATCGACATGCTGATTGATCTATTGAGCAGTACGAACGAGCACGTGCAGCGGCAGGCCGCGAAGGCGCTTGCCAACCTCGGTGTG---------------------------------------------------------------------------------------------------------------------------AACG---ACGCCAATGAGGTCGAGATCGCACGTAAAGGAGGTTTAAAGCCAATTATCGACGGTGCGCACTCTGAATCCGTCGAGCTTCAGTCCCAAGTGGCTCGTGCACTACGCAACTTATCGGTCAATCCGGAGAACAAACAAGCCATTGTGGAGTTGGGAGGCGTCGAGGCTCTGCAATCACTCGTACGTTCCACTAATGATCGGATATGCCAGCAAGCTACACGCGCCCTCGTCCCCAACGTGGTACTCACGCGCTCGTCGCCCACCATCTGTTG---GCTGTAGCGCCAGTAGCTCGCGTGGCTGAGATCAAGGAGGAAGC------TATGCCCTACGATGGCGCAATGGTAAACGTCGAGTACTCTACTACAGTCAAGACGGTC---------ATAGTGCCTGCAGTAAAGCCCA---ACGACGCGACCGGG---GGCTTCTCGTCCATCTCCGACCGTTTCCCTCTCTCCAGAGTGGGGAAAGTCAGCTGTGGTATGGCTCCAATTCCCATGAGCCG---CTTGCTCAATG---CCACACCGGAGTTGACAC---AGGAGGACCAG-------------ATGACTGTACTCGTGGAGGACGGGACGGTGGATGAGAAC---ACCATTAAAGCCGAG---CCGGAAGGTGAGCGATGTGTACAAGTACCAGGCCACTGA--TGTGGAAGAGAACGAGATGGAGCAACTTCGATGGAATATTCAAGAGCAGGTTTTCCAGCGGTACTTCGAGCAAGGACAGAAGTTTGAGGAATGGGGACACCTGGAGGAGAAAGACTCCGAGTGTTCTTCGTTGCTGGTGAACGAAGAGAGAACATCATGGCCGAGAAGCGGTTCCACGACAATGCTGTCCGAGATGCCATGGGCAAAGGAAACGCCACGTGGGTCAAGGAGCGACGAGATCGGTTCGAGGCTGAAATCCGCAGATTCTACGACGAAACCAGACACAAACAGGAGTTGATGCTCACGCGGCTTGAAAAAGTGTCGCCATTCTCAAAATTCCCGATTCTTGGCAACCGGTTTCTTCTGCTTCGTCTGCGAGGAAAAGGAGGCAACGGAGAAGTATGGGATGTCGTGGACTACGCCAACAGCAAACAGCTCTGCGCTCTTAAGCTCTCCACTTCAATACGACACGCCCAACGCGAACACCACACGCACACTCAACCACCCCA--ATATTGTTCAGGTCGGCGAGGCACCCTTCCTTATCCGATATCAGCAACAGCAATACACTGCGTTCACTGTTGCCAGCGTTGAGTCAGATCTTCAGCAACTCATTGAGA---TGTACGAGCACTTCGATGATGACTCTGCATACAAAGTGCTTTCTCAGCTTCTGAACGCCCTGAAATACTTGCACGATGGGCGTTGCTCATTACGACCTGAAGCCGTCGAACATTCTCATCGCCCAGGGCGGATGTGTGCAGCTT------ACCGACTTCGACTTGACTCGCGACGCCAAGGCAGCAACGTCCAACTCTACCGTTGGCACTTTGCGATATCTTCCTCCTGAGTGCTTCCGTCCGAACCGTAGCGA---------CTGCGAAGCGACGGCTGAGAAGGCTGATATGTGGATGGTGGGCATCGTGTACTGCATGATGGTCACTGGAAAGCACCCAATCTGCCCGGACAAATCGATGCACGCTGAAGTAAAAGCGATGATGGCTCAGTACACGGGC---GAACTTCGATATGCACGACCCATTTCTGATCTGTCTCGGTGGATCTTACAAGGCTGCCTGCATCCAGACCCGCGATGTCGACCAACGGCCGCACAATTGCTAGTGGCGCTCCAG

Phytophthora_palmivora TGGACTACCATCGCCACGTGGCTCGCGAGTTGGCCGCGTTCTTCCACTCCTTCTACCGCTTCGGCGCTGCCTCCAACCGCTTCGATCTATTCCTCCACAACGTCCTCTTCGGGTCGCTCTGCATCGGAGCCGTTGTGTTCCTTGTGCTCTGCGTTGTGGTCGTGCGTCGCACGATCCTACACATTCGTAGGCCTTTGTCCGTCGCACGTCGATAGATTCCACGTCAGTGGAGCTGCTGGCAGTGGCTCTATTGACGTTCCTGGCTATTAGTGCGCTTGGGGGACTCCTGGGAGAAGCTGTCTGCAGACAAGTGACGCTTTACGTCTCAATGCTGACAATTTGATCACGTCGTTTAACGACAGTGAATTGCCAGCGCAAGCTTTCAAGATGTCGGTGGAATCGCTAGACTCGTGCACGCATCCAAGGAACTGTGGAACCAGACGGACGCACTATTGCCCAAGAACTACTCGGACATTGCCAGTGAATGGCAATTCAGCTACTTCGTACTTAAGTCTTCGACAAACGGCGCGATTTTGGCAGTGGCTCTAGCTAGCTTCTTGTCCATTGCGTCGGTTGGGTGGGCGATGGTCTCCCCGCTGCGCGTCTCGATTCTGGTGATCTTGAGTGTAGTGCCGATTTCGCACACGCTCATTGGTGCCTACTTGTCATCGACGATCATGACGGCGGACTTTTGTGCTGCTCCATTGAACAACACGCTCGGACTAGTCGGCACGTGTCCTGCAAATGCGTCCTGCCTTTTGCTGATGCTATGGCTACGCAACGCTTCTCACCAGGGAGAGATCGGTCGTCGCATGAAGAAAGAGTTCCTGGACCCTATCGGAACAGCTGTGGGCGTCATGCTCGGCTATTTCGCAGGCACAGTGAGCAACTGGGAGAGTTTCTCGCTCATTCAGACGTGGCGATGGCCATTTTTGCTGCAATTTCTGGCACTTTTGCCAATTAATGTAGCCATTTTCTTTGTGCCAAAGAAACACCTGGTCATTCGCAGTGAGATGAGCGAGTTGGAGCCACCAGTCGCTGGCACGCCCATGAGTACGCGCTCGACGACTTATCACGATGAGGAGGACGCGCTGCTCGCCAGTGCCAGCATGCGACCTACGGTGCATTGGACACGAGGTGTATGAGCAGGGGGCATTTTCAGAGGGAGTGTATGAGTTGTTGCACATCCCTGTTTTCTGTTTGATCGTGTTTGGCCTGACGACCGTGTACTTTGTTGTCACGGGCGTGCAGTACTGGAGCACTATCTTCATGATCAAGTCGCTGCACGCGTCCAAGTATCTAGTCAACGCACTCTTCGTTGTTGTCTCTGGTACAGGGCCTATCCTGGGTGTTTTTTATGGTGGCTGGCTCATTGACCGATACGGAGGTTATATCGGCGTGGAGCAGCGCGCGAAGGCCCTGGGTATTTGCATGATTCTGGGTCTGACAGCCTTCTCAATCTCAGCGGTGACAACGTTCTTCGATGACGTTTATATCACTTCCGGTTTCCTGTGGTTGTTGCTCTTCTTTGGAGGTGCAATTTTGCCGGCGCACCGCGCACTTGCTTCCTCGTTCTCCGTGATGGTCTTCAACCTGTTCGGCTACTCGCTCTCGC---------------ATGGTGGTAGGCGCCAAGTGCTTCGTGCCAGACGAGACGCACGTGTGGCTCGCGGCTGAAGTACTGCGTGACGAGAACTCGGGCGACGGTAAGACCCGCAAGATCTACTGCAAAGTGGAGCTGCCCGATGGAGACGGAGGAGCGATGCGTGGACATGCTGTCCAAGAAGACCAAGGCGCTAATGACGTGCACCAGCTCGAGTCGCTTCCCTACCAGAACGAGAACGTGGGGCCTGAAGGTATTGAAGACATGATCACGCTCAACTACCTCCATGAAGCTGCCATTCTGTACAATGTGAAGAAGCGTTTCCTCTGTGAATTGCCCTACACGTACACGGGCGACATTTGCATTGCTGTGAACCCGTACAAGTGGCTCCCTGACCTCTACGCTGAAGAGCAACACCTCCGGTACCTTAACCAACCCAAGGAGGAGCTTCCCCCGCACGTCTACGCCACATCAGTTGCAGCTTATGACAATATGCGGCGTTCGGAACGCAACCAGTCCATTCTGGTGAGTGGAGAGTCTGGTGCTGGTAAAACAGAGACTACCAAGATCCTCATGAACCATCTGGCCACAATTGCTGGTGGTCTCAACAACTCGACTATTAAGAGGATTATCGAGGTGAACCCACTCCTTGAGTCCTTTGGTAATGCCAAGACTGTACGTAACGACAACTCCAGTCGATTCGGCAAGTTCACACAGCTGCAGTTCGACAAGAACGGTACGCTTGTAGGCGCCAAGTGTCGCACGTACTTGCTGGAGAAGACACGTGTCATCCAGCACGAAGCGCCGGAGCGCAACTACCATATCTTCTACCAGTGGACTCCCCCGACATTGCGCCGGAGCTGCAACTTGACTCGTCAAAGCACTACGTGTACACGGGTGACAACACAGCACAGAAGATCGAAGGACTTTCTGATGAGAAGCACTTCAACAAGACACGCGAGGCGCTGGAGCTGGTGGGTCTTTCGCGCGATGACCAGCGGCCGCTGTTTGAGGTGCTCGCTGGCGTGCTTCACCTCGGTGAAGTACAGCTGCAGTCGGATCCCGCAGATGACGAGAAGTCTCTCATCGCTGAAGGCGACATCGGCGCGACGTCTGCGACGCAGATGCTTGGAGTCACGTGGGAGGCACTCCAGACTGCTCTGTGTTCACGTACCATGCGCGCTGTAAACGACGTGTACAGCGTACCTCTGAAGAAGGAATTGGCCATGGACTGCCGTGATGCACTCGCCAAGGCCATCTACTCGAACGTGTTTGATTGGCTCGTGGCTACGATTAACCAGTCGCTTGCTGACGATGCCAATATGGCAAATCATGTTGGTGTGCTTGATATCTTTGGTTTCGAGCATTTCAAGCACAACTCGTTCGAGCAGTTCTGCATTAATTATGCCAACGAGAAGCTCCAGCAGAAGTTCACCCAGGATGTATTCAAAACTGTGCAGATCGAGTACGAGGAGGAAGGCATCGTGTGGGACCACATTGAGTATGCGGATAATCAGGACGTACTGACCGTGATTGAATCGCGCATGGGTATCATTTCGTTGCTGAACGAAGAGGTGATGCGACCAAAGGGTAATGAGGAGTCCTTCATGTCCAAGGTGACATCTCTGCACAAGGAT---GATATGGCACACGTTATTGAGTTCCCTCGTACGTCGCGCACCGAGTTCCTCATCAAGCACTATGCTGCCCCTGTCATGTACGACTCGGTGGGCTTCCTCGAGAAGCACAAGGACTCGCTTCTTCCTGATTTGTCCGAGCTCATGCGTGGTTCGTCCAAGCCGTTCATTGCCAAACTTTTCGAACCGAAGCCGGAGCCAAAGTGGCAGAAAGC---------GTGGTGGTGCTTTGTCCATCACGACTGTTGGTACTCAATTTAAGGAGAGCCTGACGGAGCTGATGGCTACCATCAACTCTACCCGGGTGCACTATGTGCGTTGCATCAAACCAAACCCGATTAAGAGCTCGACGAGCTCGACGGTCATGGATCAAAACATGGTGGTGTCCCAGTTGCGGTGTGCAGGTGTGATTGAGGCTATTCGTATCTCACGTGCAGCGTACCCGAACCGGCTGCAGCACACGGAGATCCTGGACAAGTTCTGGTTGTTTGTACCGAGTGGCGGTTCGACCGCAGAAGAGAAATGCCAGCTGCTTATGGACAAATTGAAGCTCGAGTCTCCTAGACAGTACCAAATGGGTAAGACTCGCGTGTACTTCCAGCTTGGTGTTCTTGAAGAGCTTGAAGACCGCCGTAAAAAGTTCCTGGATGCCAAGGCCACGTACCTACAGAATATCATGGTTGGCTTCACGCAGCGCATCAAATACCTCCGACAACTGGAAGCAATCATCAAGTTCTGTCATTCGATGCGTCATTGCTATGCGGAGATACAACACGTTCCTGAAGGGACTTACTAAGGCCCAAGCTCGCTGGCGAGGCATTCAGGGTCGCAAGGTGGCAGCCGATGTCAAGAGCAACCACTACGCAGTCATCATCCAGCGGTATGTGCGTGGCTTTGTCAAACGTCATCGCTACGTTTTGATGCGCGCAATGGTCATCCGTGTGCAGGCTGTGGTTCGTATGACGATTCAGAGACCGAAGTATCTGGCTGCTCTCGAGGAGAAGCGTCGTGAGGCAGATATGGCTTATCAGCTGCAGAAACTGAAGGCTGCCTTACAGGAAGAGCAGGAACGGAATGCACAGCT------ACAGCGCC---GCAGCAGTGTGAC--GACGGCACGTCCGCCGCCTCGAGCGTGGTGATGGCCGACGCTGGTGGTATGATTGAGACTCTTACGGATGAGAACAAGAAGCTCCGTGAGAAGAATGAAGATATGAAGGTTACGATGAAGGGCTTGAAGGTCGAGATCGAGAAGTTCAAGAGTGACAAGGAGTTCTCGACGGCTGGTAACCATGTTAAGGTCCGTCAGTTGCAGGACACTGTGCGCGACAAGGACAAAAAGATCCAGCAGCTTGAGGCTGAAAACAAAAAGCTCACCGAGCAGATTGCCAAGCTTCACGCGGAGGGTGTTGT---TCCCGAGAAGAAGCCCACGCCAAAGAAGTCGATCTTCCGCACGTTGGGTTCGAAGAAGGAAAAGAAGCCGC---------------GCGAGACCGTGCTGATGGACTCCCTTACTGGATCGGACGATCTTAACCGGCGCGAGACACGTCCGTCAGA---GCGTCACTTTCCGCGTATGC---CGAGCATGAGCGTGGGCTCT---CGCTTCTGGAACTCGACCAAG---AAGGGAGAC------------AACGGTGACGGCTTCGATGTCTCCGAGGTCGACGGCGAGGACAGCCCCACTAACCCACACGCAACACAATCGTGAAGAGCATGGAGGTGGGCGTGACGGGTGCCATGTCTAGCCTGAAGGGCCGTATG---------TCGGTCGTGAAGGGCATGTACGAAGTAAGCGCACAAACAGGATCGCCCTGTGCGCGAGTCGTTTAACCTGGATGCGTTGCCAGAGGTTTCCCTGCCTGTCGGCTGGGAAGCCAAGGTGTCCCGTTCCACGGGTCGCGTGTACTACGTGAACCGCAAGCTGGGCAAGTCCCAATTCGAGCGCCCCACGCTGGCTTCACTCAA------GGCACAGAAGTTGGCGCGCCAGAAGTCGGGTAACGCGCTGTAAGGCTATTGTAGCGGATTTCGGCTGTGGAGATGCTAGACTGGCTGAAAGTGTGTCGAACAAGGTGCACTCGTTCGACTTGGTGTCGCTGGATATTGGAGTGTACTGCTTGGCACTTATGGGAACGAGTGTTCGCGAGTATGTGCGTGAAGTCTACCGTGTACTGAAGCCTGGAGGGGTGTTGAAGGTTGCTGAGGTCAAGAGCCGCTTTGAGAACGAGTCGCTGGGCGGATGGTCGCGGTGTTTATAGCGGCAGCGGAGGGCGTCGTCGTGACGATCCGCAATACTACAGTGTTCGTCGAGAGACCAAGTATGCAGTTATTGTGGGCCAGAAGACAGGTGAGGCGGTGGCTGTGCCATTCCCAGACGTGGACCGTGACCTCAAGACTCTATTGGGTCACACTACGTCCATGGTGACCCATGTAGCTGTGAACCTCTTCGCTGTTGTTAACAGCTGACCGTGACGAGAAGCTCCGTGTCTCGAAGTTCCCCAATGCTGCTATTATTGAGAGCTACTGCTTGGGTCATGCTGCTAGTCTGACGAAAGTCGCGTGCAGTGCTGTGACGCCGGATCTTGTGGTGTCGACGTCTATGGATAACACGCTGAAGCTGTGGCAGATGTCAACAGGTAAGTTGGCGATCTGTCCTAAGACGACATTGTCGCGGTCCTTGTGAACTACCAGCACGTGCGCATCTTCGAGATCGTGAGAGTCCATTCCTGGAGCTGTATTCTATCTCCAAGAGCGTCTGCTGCATTCAACTGCTGTGCGCGACTTCCGCAAGCTTCAGAACGACCCTCCATCGGGCGTATCGGGTGCTCCCATGGACAACAACATCATGCTATGGCAAGCCGTCATCTTCGGACCGGACGACACGCCCTGGGAAGGCGGCACATTTAACCTCACGCTCGAGTTCAGCGAAGACTACCCGAATAAGGCGCCGACGGTCAAATTCATCACGAAAATGTTTCACCCCAACATCTACAATGACGGTCAGATCTGTCTGGACATCCTACAGAACCAGTGGTCGCCGATCTACGACATTTCGGCCATCCTCACGTCCATCCAGTCGCTGCTGTGCGACCCGAACCCGAACTCGCCAGCCAACTCGGAGGCTGCGCGCCTCTTCCAGGAGAACCGACGGGAGTACAACCGCCGCGTGCGTGAGATCGTCGAACAGAGCTGGCAGGCGATCGATGGTGTCGGCCGCCAAGATGCGTGGCGCTGAGAACCGCATGAACGCCGGTCGCCCCTTCACCGCGTGGCTGGACAACGTCTCGTCGGACCGTGGTCTCTGCGGTGGTATCAACTCGGGTATTGCCAAGACCACTCGCAAGCAGACCACTCGCAGATCTTCGTGATTGGTGACAAGGCTCGTGCTCAGCTGCGTCGTGACCTTGGTGACAACATCCGTGGTAACGTGACCGAGACGTACCAGTCGCCCCCCAACTTCACGGTGGCCAGTGCCATCGCAGAGGGTGTCGAGTCGGAGAGGAGTCGTCCCGTATGGCTGCTATGGAGAACGCCACCACTAACGCTGAGGACCTCACGCTCACGCTCGTGTACAACAAGGCTCGCCAGGCCCGTATTACCACGGAGCTGATTGAAATTATTTCGGGTGCCGCCTCCCTGGACAGTGACTGGCAGCGTGGTGAGCGTGCAGAGCGTCGCGCAGTTCGACGAGGCAACTGCGCGCGAGTCGACGCTGTCCGTGAGTTTCTTCTGGGCTGAATTCCAGGCCTGTCGTCCCAACGGCCAGCTGGACGTCGGGTCGCGCTGTCCTGGACAAACTCGAAGGCGCCAATGTGGCGGAACTGGCCAAGCGCGTCTGCAATATCGTCTCAAGAAACTCATCAATGCGTCGCCCGTGATGCTCTTTATGAAGGGCAATCCGACGGAGCCCAAGTGCGGCTTTAGTCGTCAGATGGTGGCGCTGCTGAATGAGGACAAGATTCAATTTGGCACCTTTGACATCCTCAATGATGACGAGGTGCGTCAAGGCTTGAAGCAATTCTCCAACTGGCCTACGTACCCACAGCTCTATGTCAACGGATCGCTCATCGGGGGCTTGGACATTCTCAAAGAGATGAAATCCGATGGATCGATCGTCGAGCAGTTGGGCGTGGAGGAAGCTGAAGCGGCGTTCCAGGAGAGTCTAAGTGCACTGGTGAACTCTGCCCCTGTACTGCTCTTTATGAAAGTTCGATATCTTGTGTGGAGCTACCAACGTCAGGACCAAGATATCGCGAAGGTGGTTACTCTTATGCCAGCATCATTCCGCCGGGATATGCGCTGAGGCTATCTCGTGGAGCCCGAAACCGCATGGCAATGGCATTCCGTGATCCTTTGCAGGAAAAGGGAGAGTTCTGCATTGTGCAGCTCCTCGTATCACGTCTAAGCGTACAACCTTGCATTCACGAGGTATTTCAGCGATCGACTGGCAAGTTTAATGTTCTGCACCGGGACCACCGCAGTGAGGTGAAGTCGCTATGCGTTCACTCTAAGCGTGACTGTTTGTACACAGGTGGCCTAGACGGATTGTTAATTCGATACGACTTGCAAAAGGGGCGTAGTGAAATTGTGATGGAGCGCCGCAAGCACATTTCCAAGATCAACGCAGTACTAGAGCACCCGCACAATCCAAATTTGCTTTTGGTAAGTTCGGTAGAGCAGCAGGCACAATTTATTGCTTTACGATCTGCAGCGTTATGACCCGAATGTGAGCGGCAACTCGATGAGTCAGTACGTCGTGCCGCGGTGGTCCCCTGCAGGCTATCACGTCTCCTGTGGCAGCAAATGCGCTATTGCCACGGCAGCTTCTCCACGCCCACATCCGCCACCATCGGGGCATCGTTCCTGCAGAAGCGCGTGATCGTAGGAGGGAAACTGACACTGCAGATATGGGATACAGCCGGTCAGGAGCGCTTCCGCTCCATGGCACCCATGTACTACCGCAATGCGAAGGCAGCTATCCTGGTCTTTGACCTGCAAAATGAGGCCAGCTTTGAGAAAATCAAGGAATGGCTTCACTTACAGCATCACGTGGGTGACGACATGTGGGCAACAAGAGCGATGTTTCGACTTCTCCAAAGCTCAGCAGTTTGCGGACGAGATCGGCGCTCTAGTTTTTCGCACAAGCGCTAAGTCGGGAGAAGGAGTACAGTCGTTGTTTGAGAGTCTGGCAGTGCAGCTACTTAAGAAGCATGATCAGGAGCAGCGAAGAGCTTCTACGTCGTGTATATACCGGCAGATGAGAGCAAGGAGCTGGAGGAGTGGACAGTGAAGCTGCCACAAGACAAGGAGGCACAGCTGGGATGTCTCACGGAGCGTCTGCGCGAGCACTTTAAGGGCGGAGCCATCAAGGCAGCAGCAGAGACGTTCAAGCAGCAATTGCTGGCTCAGTTGCCCAAGGGCGCGACGCTCACCGATGAAATGCTGCAAATGATGCATGGACAGCCTCGTGGATTCGATTCCACTTGTTTTAAACACGCCCGATGTCAAACACGTTGGAAGTTAAGGGTGACGCTTTTATTGGACGGGTTTTTGATAGACGACTTTGTGCGCATGGATTTCCGGTTGAGTGAAGTGTCGGGTGACGCGCCGTGGGTGCAGACAGCCAAGCTGCAGAGGTCACTCAGTCACTTGAGGAGATTCTGGCGCTAGGACAAGGTCTGGACGATGAGGACTACAAGGCTGAGCTCGAAGTGCTGCAGAATGACCCCAACTCGAATTTATACTCGGCCGTGACATTCGAGGAGCTCAATTTACCCGAGCCACTGCTGAAAGGTGTGTACAACATGAAATTCGCCAAACCATCCAAGATTCAGTCGGTCGCATTGCCCTTGATCCTCGCTGATCCACCGGAGAACCTCATTGGCCAGGCACAGAGTGGCATCCTACCCGTGAACTGGTGCGACAGATTATGGCTGTGGTAAACGCCATGGGCAAATTGTGGAGACGTTCTTGGCCATCCCTGGCAATGATCTCGCACGTGGTGAGACTCTCCAGGCTCAGGTCATTGTCGGCACTCCAGGCAAGATTGAAGGTATGATCAAGAAGAAGCAACTGGACACTCGTAGTGTCAAGATCTTCGTGCTGGATGAGGCAGATGTGATGGTTGCTGAGGACGGCATGCGTGAGCGCTCGGTGTCTATCAAGCGGCTGATCAAGAACCGCGCGTGCCAGTACTTGCTCTTCTCGGCCACGTATGCGGACGATGTGCGTGACTTTGCTCAGAAAATGGTGCCTGACCACAACATTATCACGGTCAAGAAGGAAAAGCTCACTCTGGACGGTATTAAGCAATTCTGGATCGACTGCAAGACTCGTGACAACAAGTTCCAAGTGCTGTCGGACATTTTTGCCATTCTGTCTATTGGCAAGTGCGTGATCTTCGTGCAACAGCGTGAGACCGCCAAGGAACTAACACGCAGTATGCGTGATAAGGGCCACTCGGTGGGCATCCTACACGGTGCTGACATGGCCAAGGAAGTGCGAGACCAGGTTATTGACGAGTTCCGTGCTGGTACGACCAACGTGCTGATCACCACAAACGTGCTTGCTCGCGGTATCGATGTGGCGGGTCTGTCGCTTGTTGTAAACTTTGACATCCCGCTTACCCGCGACCACCGTCCAGACCCAGAGACATACCTGCACCGTATCGGTCGTACGGGTCGCTTCGGCCGCAAGGGTTGTGCTATTAACTTTGTCTACGACAACAAGAGCAAGCAGGATCTCGCTGAGATTGAAGAATACTACGCGCGACCGATTACTCAGGCTCCAGCAGACGACATTGAGGAGCTCGAGAAGCTGCTGGCATGGGGGGCAGCAAGCGTCGTCTGGCTAAGCTCGGTAAAGAGCAGCGCAAGGGCATGCGCATTTACCCTCGTGAACCCAAGAAAAAACCTTCAGGAAAAGATAAGACGTACTATTACATTAAAGACGTCATGTTCTTGGCTCATGAGCCCGTGCTGGAGAAGTTCCGTGCCTTTAAAACCTTCATGAAGAAGGTTAAGAAAGCCATGGGCAAGAAAGACTATGAAGATGCCAAGCGGAAACATGAGAATCGACCGACGTACGAGCTCGACCATATTGTCAAGGAGCGATATCCTCGCTTTGTGGATGCACTAGGAGACTTGGACGACGCTCTGTGTGGCAGAACTACATTGTGGCGACACGTGCATTGACCAAGGTCTTTGTGAGTATCAAGGGCATTTACTACCAGGCCAATGTGCAAGGACAAACCATTACGTGGTTGGTACCTCATCAGTTTACTCCTACGGTGGACAAACGAGTGGATGTGCGTGTGATGCTCACGTTCTTGGAGTTCTACGAGGTGCTGCTCAAGTTTGTCAATGCTCAGCTCTACATGCACATTGGTGTGGCGTATCCGCCGAAGATTAATCTGCAATTGGATGCTGCTGGTGCTCAGCTTGCAGCGATTGAGCTAACAGCAGAAGGAGTCGGAGAAGCGTATTAAGCTACAGCGAACAGCGCAAGGAGCGCAGCGAGAAGAGCTTGTTCAGTGGTCTCACCTTTTTCCTGTCGCGTGAGGTTCCTTCGGCTTGTCTGGAGCTGGTGCTTCGCTCACACGGCGCCACTTTGGGTTGGGATGGTGCTGGTTCTCCTTTCGACGAGAAAAACACGCGCATCACGCACCACATCATGGATCGCCCGCACCAAGGACACCGTTACTTCAACCGCGAGTACGTGCAGCCGCAGTGGGTGTTTGACTCTGTGAACAACGGCATCCTGCTACCATTGACACGCTACCGCAAGGAGCTGGAGAAGCTCAAATCGGCCTCCCAAGTGCTGCGCGAGGTGGGCGCCGTTGGCGACGAAGGTGAAGATGGGAGGCTTACGCCGCCGACCTCAAGGCTGAGATGGATGGCGACAAAAAGTCGAAGCGTGCCAAGAAGGAGGAGGAGGCTGCGGAGCTCAAGGAGCTGGCCAAGACTATGATGTCGAAGAAGACCAAGCGTCTCTACGACCGTATGCAGTATGGTCTGGGCAAGAAGGCCGACAAGATTCAGAAGTTGGAGGACAAGCGTGTCAAGCTGGAGGCAAGACGCAGAAGTTGGTGGCTGGTGCTTTGTCTGGGATGATTTCAGCATTGGCTTTCCAGCCTCTCGGGCGTTGCGCTGGCGTGGCACGTCGCCTACACTCGTGCGCGTGGCAGGCGGTGCCGGACTTTACTTTGCCACATTAGACCACTGCCTCAATCTGTTCCCGCACTCAGCAGTCAACACGTTTCTGGCTGGAGCCTTCGCGCGCACGTTCGCGGGTGGCATTATGTCACCTTTTACTATTGTGAAAGCCAGGATGGATTCCTCCTGGTACCTTTGACAGCAAAAGGACGTGCCGTTCTCTGGACTCTATGTGCTCATTTACACGGCATCCGGCAGATGTGGTGAAGACGCGGATGCATGAAACAAACGGTGGCCAAGATCTACCACCATGAAGGCCTGCGCGGCTTCGCCAAGGGCATCATTCCCCGTGTCGTCAAGCGAACGCTGTCCACTGCTGTGACCTGGACGATCTACGAGCTACTCGTTCGATCCGTCGGGTCTGGGTCATGCCAAGGAGGCCTTCAACTTGGCTAAGGAGACGGAACGCACCAAGCAGATGGAGAACCAGGCCAAGATCAAGGAGAATGAGGCACTCTACAAGCAGTATGAGATTGTGCGCGTGCAGAAGGAGGGTGAGGAGCGCCGCAAGACGCTGGAAGAGGAGACGCGTCAGCACCAGCAGCGTGCGCAGTACCAGGACCAACTCAAGCGCAAGCAATATGCCGATCAACTGGCCGCCCAGAAGTACATGAAGGAACAGGAACTCAAGAAGCAGGAGGAAATCCTCGCACGTCAGGAAGCTTCACGCCGCAAGACTCTCGACTACGAGGCAGAATTGCGTCAGAAGACGGAGCTGGCCAAGGTGGCGGCTGAGACGGAGGGCCGCATCAAACAGGAGCGTCTCAATCACGATCTGCACCTTGAGGAAGCGCGTGTTCGTGCTAAGGAGTACCGCGAGACTGTCATGGAGGGTATTAAATTGGCAGGTAACACAGTTGGCTCTGGTATCATGACTTTTGTCGACGACAAGGAGAAACTTACGGCCACTGTGGCCTCATTGACGGCTCTGGCTGTCGGTATCTACACCGCCAAGGTCTCTACCAGCGTTGCGGGCAAGTATATTGAAGCTCGTATGGGCAAGCCATCGCTGGTGCGTGAGACCTCGCGTCGCTCAGCTACGCAGGTGCTGGCTAACCCCATTCCTTCCATCAAGCGCGCGTTGCGTCTGCAGAAGGCAACGGATGCTCTCGAGGGCGTCGTGCTGGAGCCCAAGCTCGACGAGCGTCTACGCAGCGTGGCCGTGTCTACGTTTAACACGAAGAAAAACCGTGCGCCGTTCCGCCACTTGCTGCTGCACGGCCCGCCTGGTACGGGTAAGACGCTGTTCGCCAAGGCCCTCGCCCGTCACTCTGGTCTGGAGTACGCCATTCTCACGGGTGGTGATGTGGCTCCGCTGGGCCGCGAGGGTGTGACAGAGATTCACAAGCTCTTCGATTGGGCTTCACACAGCCGTCGTGGTCTGTTGCTTTTTGTCGATGAGGCTGATGCCTTCCTTCAGAAGCGTAGCAACACTGTCATGAGCGAAGACATGCGTAACGCGCTTAACGCTTTCTTGTACCGCACGGGTGAAGCCAGCGACAAATTCATGATCGTTTTCGCCAGTAACCAGCCGGAGCAGTTCGACTGGGCCATCAACGATCGTATTGATGAAATGGTGGAGTTCCGTCTGCCTGGCTTTGATGAGCGTGTGCGCATGCTGAAGCAATACTTCGATGACTACATCCGTGCGCCGAAGAACTCGCGTGCCAAGAAGATTTACGCGAGGGCATCATCTTCACACTGGACCCTGTCGCCAACTTCCTCGTCCCAAGACGCGTATCTTCCAGCTCGAGGCGCTGCAGGGCGTGACCGCTGAAGCTCAGGCCATCTTTGATGCACTCAACAAGAGCCCATGCGCATCTATTGGCTTGTCTTCGGTGTGGTACTGATCAACGGATTGTTCACTTATGTTATGGGCAAGGACGAGTCGTTCTTGGTGGAGAAACTCAAGAGAAAAATCCCGTCCGAGTACCGCATCTCTGAGAAGTGGGACAAGTGCCTGGAGAACTTCACGCTCTACTTTGGCGCTGGCCTCGTGGCTGGCGGCCTCACGTCCCTCGTGTTGGCGCGTTCTGGTGCTGGTCGCGGACTGGTGACGGGTATTGGCGAGCTGGTGCTGGATCGAGCTGGACGACTTGTCAGATGGCATTCGCAGGTGACGCACAGGCAGCTCTGGACAAGACGGACAAGGCTGTGGGCGACTT---GGCGTTCCAGCAGCAGACGACCGTGGACTTTGTGACGGCCTCGGCCACGCAGCAGCAGTTGTTGGCAGTGCACCCGCGGGTTCTGCGAGCAGGCCAATGTGGCCATCCTGGAGACGTACTTGGCCGAGCAGGTGAAGAACTCGACTGTGGACATTGATGCCAGCCTGGCGCTGCTCAAGCTGTACCAGGTGTACCCGGCCACGGTCAATGCTGAGAGCCAAGGTGCTGGTCAAGGGTATCATGTCGCTGCCCTCCACCTTCTTCACTGGCGTCGACGATGGTGCCCGAGAGCATCCGTGAGGACGCTAATGTGACGGCGGTGCTGCACACGGGCTTCTTGCTACAGTCATGTCTGTTCGAAGACTTCTGGAAGGAGGATGTGACTTTCGCCGAAAAGGTGCCAGGCTTCCTGCAGTCGGTGCGTGCCTACATTCTGACGGCTATTAGCCGCAGCCACAGTGTCATCTCTACGGACGTCTTCAAGGCCAAGCTCAATGTGTCTGACAAGGAGGTGGCTGATATCGTCGCTGAGAAGTGGACGCCGGTAGCCTGATCCAGATCAACCCCAACGAGGACAACCAGATGCAGGCCAAGAAGGTTCAGGAGAACATTGAGTTCGAGGACGTGCTCAAGGTCATCCACACCCTTTCTAGATCCAACGTCTGCGCCAGCGGTGGCGGTGTCGCTGATGCTGAAGAGGTCTGTGACAACTTGGCCAAGCATTTGAGACTCGAGGGGGGCTCCCAAGTGCTGCCCTGTTCCACGGGTGTCATTGGATGGCGAATTCCGGTCGATGCCATGGTCGAGAACCTCCCCAAACTCGTCACCAATTTGCAGTCCGACTCGGTGCTGCCAGCTGCCGAGGGCATCATGACCACCGACCTTTACCCGAAGGTCCGCTCTATGGACGTGTGCGGCGGACGCATTGTCGGCATTGCCAAGGGTGCCGGCATGATTGAGCCTAATATGGCTACGATGTTGTCGTACATTCTCACTGATCTGTCGATTCCCCGTGACGTGCTGCGTCAGTTGCTGACGGACGTGGTTGGAGACACGTACAACTCACTTAGTGTGGACACAGACCAGAGTACGTCCGACACCGTGACGCTCGTGTCGTCGGACCAGATCCCGTTCGATATGAA---GCAGTTGGACGAGTTCAAAAAGGCTCTCCACGAGGTGTGTCTCGGACTGAGTGAAGACATTGTTCGCAACGGTGAGGGTGCACACCACGTTATGCGGGTGCGTGTGAGTGGTGCAATGTCCAAGGAGATGGCTAAGGGTGTCGGCAAGTCGGTGGTCAACTCGCCACTGCTCAAGTGCGCAGTGGCGGGCAACGACCCGAACGTGGGTCGACTGGTCATGGCTGTGGGCAAGTACATGGGCCTGCACCACAAGGACGTGAACATTCAGCGTCGCATGAAGATCCGTATGGGTGGCGTGGTCATCTTCGAGAACGGCGAGATGGTGCTCAACGACGATATTGAAGCCAAGCTGGTAGCGCACATGCGCGACGCGATGCTGATTGAGCCCACTCGCGGTGGTCTTGACGCCAGTTCGCACAACTTCCCGCCCCACGGAAAGTTTGTCGAGATCGAGATTGATCTTGGTGTTGGATCGGAGGAGACGCAAGTTTTGGGCATTGACCTTACGCATGAGTACGTGGCCATTAACGCTGACTCCCGCTCGTAATGGCGCC---GAAAAAAGGGAAGACTGCCGCTGCGTACCTGGCATCTCTGGGTGTGGCCCCCAC---CGCTGGAGGTGAGAAGAAGGATGAC---AAAAAGAAGAAAAAGAAGGGAAAGAAGAAACCTGCAAGAAGCCCATGTCGGCAGCGGCGAAGGCTCTGA-TGGAGCGTCAAGCGGCTATGAAGGCTGAACAGGAGCGTCTCGAGCAGGAACGACTGGAGCAGGAACGCATTGCCCAGGAGGAAGAGGCCAAGCGTCTGGCGGAGATTGAACGCGAGGAGGAGATTCGCCGCAAGAAGAAGGAGGCACGCGCGGCTAAGATCGAACGTCAGAAGAAGGAAGGTACATATATGACTAAGGCACAGAAGCAGAAGGCAGCCAAGGCACAGGCTGCCCTTGAAGCCATGCATCAGGCAGGCCTCGTGCCTGAAGTTTCG------GGTGAAAAGAAGAAGATCGTGTACTCGGACAACAAGAAGAAGAATAAGAAAAACAGGAGGAAGAGGAAGAGGAGGCCGAAGCCCCAGACGCGTGGGATGACGAGGACTGGGAAACTAGTGATGTCGTGGCCTCACTCGAGGACAAATTAACAGAAAAAAGAGCAGGAGCGTCTGCGGCAACAGGGAATTGCACTGAAGAAGCGCCAGGAAGAGCAGGCCGAACAAATGCGATTAGAGGCTGAAGAGGAGCGTCAGCGCTCTAAAGCTAAGCTCGAGGCTGAGCGCAAGAAGAAAGAGGCAGCGGAACGTCGTCGCGAGCGCGAGGCTGCCGCTCGTGCCAATGTATCCCTTGACAACCTTCGGTCGCCGATTTGTTGTATCATGGGTCACGTCGATACTGGTAAGACTAAGCTTCTTGATAACATCCGTAAAACCAAGGTGCAGGACGCCGAAGCTGGTGGTATTACCCAGCAGATTGGTGCGACCTTCTTTCCTGTGGAGGCTATCAAGCAGAAGACGGCACGCCTTGCCGAGACGATGAAGTTGGAGTACCGCCTGCCCGGTTTGCTAGTAATTGACACACCTGGACACGAGTCGTTCACGAACTTGCGTACTCGTGGTTCGTCACTGTGTGATATCGCCATCTTGGTGGTTGATATCATGCACGGTCTGGAGCCACAGACTTTGGAGTCGCTCCGCCTACTGCGTCAAAAGAAGGCCCCGTTTGTGGTGGCGCTTAACAAGATCGATCGTTGCTACGGCTGGAAGACCATGCCTGACATGCCCGTGCAGGAGGCACTCAAGCACCAGAACGAGCACGTCATTCGTGAGTTTGAAGACCGCATGAAGGCGATTATTGTTGAGTTTGCCGAGCAGAAGCTC-------------------------------------------------------------------------------------------------------------------------------------------------------------------------------------------------------------------------------------------------------------------------------------------------------------------------------------------------------------------------------------------------------------------------------------------------------------------------------------------------------------------------------------------------------------------------------------------------------------------------------------------------------------------------------------------------------------------------------------------------------------------------------------------------------------------------------------------------------------------------------------------------------------------------------------------------------------------------------------------------------------------------------------------------------------------------------------------------------------------------------------------------------------------------------------------------------------------------------------------------------------------------------------------------------------------------------------------------------------------------------------------------------------------------------------------------------------------------------------------------------------------------------------------------------------------------------------------------------------------------------------------------------------------------------------------------------------------------------------------------------------------------------------------------------------------------------------------------------------------------------------------------------------------------------------------------------------ATGTATTCGTTCGCGAACAACATGTTGCTGAGCTTCATCACGGCACTGGTGGCTATTGCTGTGTGTTGTGCCGCGGTGACGCCTACGCAGGCGCCAGAGATCCCGAGCTTCCGTGTTTCACAGGTGGTGCATGGCTCCCCGACAGAGGTCTTTAGGCTGATCATGAACCCCAAGAGATTTCAGCGATGGGATTCAGCTACGGCAACTATGCGCGTGGTGCAGCAGCTGGATGACCACGCTGATATAGTTTATGTGACTCAACGACCGACGCACTTATGGCCGTTGTGGCAAAAGGCTCGCGATCTCGTGCTCATGCGTTACTGGCGACGAGAAGAGGATGGATCGTACTTTGTCATGTACCAGAGCATCGAGCACCCGGAGTGTCGTGTCCGCCACAACTACGTGCGCGCCAATCTACTGGGTGGTGGCTTCGTTATCGCTCCTCAGCGTGTTCCCAGT---GGATCTATTCGCACTCTGGTTACTTACGTGCTCCGATATGACCCTGGTGGTTG---GTCGCGCATTTACCATCAGCTTGGCATGGATGTAGACCTGGTCATGCCAATGCTGCGTAGTGTTGTTGGTATTCGCGATGAAATGAGCGCTACGGACTTCATCACGCCCAACGTCTCGATGGCGCCTGCTGGAGAGGGCGGAGGTTGGACAGGAAGTGTGGA---CTCCATGGGTGTTGTCATGGGAGTACAGAAACTTCAG---ACAAGTTTGCCGGAGAAGATGTGGGCCGAGCCCGATGCTTCTCTTTTCAGTGTGCGTGGGCACAACTATCTGAACGACAAGAAAAAAATCCCCTCAGGGCCGGCAATGTTCCACACAGTGGGTGTGGATCTCCTATCATTTGAAAGTGCAGCTGAGAGATACAACATCTCGTCTCGTGCGGACAGCATCGGCTGCACTAG---TTCCAAGTTCACGTTTGTTGTGAACATGATTATTCCAGGACCTGAAAACACCTGCATGGTGTTCTACTTCCAGCCCCGGCGTGACAATGTTTTCGAGGATGGTTCAGCCTTCTCTGAACTGTTGAACGATTTTTTTGATGGCGACGATCAGTTCCGGAACTCGCGCTTCAAGCTCATTCCAACTGTCGTCGAGGGAAGCTTCATTATCAAACAGTCAGTCGGCTCCAAGCCGACGTTGCTAGGCAACAAGCTGAAGTGTCCATACTACCGCGGAGAAAACTACTTTGAAGTGGACATCGACATTTCATCCAACTCGGTTGCCAACACCGTTGTGGGCATGGTGCAAGGTGTGACCAAGTCGCTAGTGGTAGATATGGCGTTCCTATTGGAAGCCCAAACCGACGAAGAGCTGCCTGAGATCATCCTTGGCGCTGTTCGCATGCAACACATTTCGCTTGACAACCCGCGCCGCATGCCGAAGCATGGGGAACGGGCAGCAAGGACGAGTTCACCTTCTGAGGCCTTCACCGAAGCTCTGCGCGTGAATCTGCAGCGATTGATCGTCTATGCGCGCTCAGCTGACACAGCGCTGCAGCGAGAAGTCGCCGAGAAGTTGGCCAATGAGGCTGTGAAGATCGACAGGTGCAGATCGTGGAGCTGGATGGACTGCAACTGCTGCTGCCACTTACCAAGTCCAAGGACACAGAGGTGCAGCGTCTAGCAGCGCATGCACTGGCAAATCTCTCGGTCAACTCTGAGAATCAATCCAAGATGGCGACGGAGGGAGGCATCGACATGCTCATTGACCTGTTGGGCAGCACGAATGAGCACGTGCAGCGGCAGGCTGCGAAGGCGCTTGCCAATCTTGGTGTGAACGTGGATAACAAGGAGCGGATCGCTAAGGCAGGCGGTATCAAGCCACTCATCGACTTAGCGAGCTCCCGCCAGATTGGCGTTGCCGTCGAAGCTATTGCGGCACTGGCAAATTTGGCTGTCAACG---ATGCCAATGAGGTGGAGATCGCACGTAAAGGCGGATTAAAGCCAATTATTGATGGAGCGCACTCGGAGTCCGTGGAACTTCAATCCCAAGTGGCCCGTGCACTACGCAACCTGTCAGTGAATCCGGAGAACAAGCAAGCGATCGTAGAGTTGGGTGGGGTGGAGGCTCTGCAATCACTTGTGCGTTCAACGAACGACCGAATATGCCAGCAGGCCACTCGAGCTCTCGTCCCCAACGTGGTGCTCACACGCTCGTCTCCTACAATCTACT------TGTAGTCCCTGTGGCCCGGGTAGCCAAAGTCAAGGAGGAAGC------CGTGCCTTATGACGAAGCTGTGGCGAACGTCGAATACTCGACCACAGTCAAGACGGTC---------GTAGTGACTACTACAATGCCAA---AGAACGCGGCTGGA---GGCTTCTCGTCCATCTCGGAACGTTTTCCTCTCTCTAAGGTGGGAAAGGTCAGTT---GCATGGCTCCCATGCCCATGAGCCG---CTTACTCAATG---CGACGCCCGAGATGACGC---AGATGGACCAG-------------ATGACCGTGCTCGTGGAGGATGGCACAGTGGATAATAAT---ACTGTCAAGGCTGAA---CCCGAGAATGAATGATGTATACCGGTATCAGGCTACTGA--CGTGGAAGAAAACGAGATGGAGCAACTTCGATGGAACATCCAAGAGCAAGTCTTTCAGAGATACTTCCAGCAAGGACAGAAGGACGAAGAGTGGGGCAAACTAGAGGAGCAAAACTCCGTGTATTCTTCGTTCCAGGTGGGCGACGGGAAAATATCATGGCCGAAAAGCGGTTCCACGACAACGCTGTGCGAGATGCCATGGGTAAGGGAAATGCAACTTGGGTCAAGGACCGAAGGGCGCGGTTTGAGGAGGAAATTCGTCAGTTTTATGAAGAGACAAGGCACAGACAGGAACTGATGCTCACTCGTCTCGAAAAGGTGTCCCCGTTCTCAAAGTTCCCAATTCTTGGCAACCGGTTTCTATTGCTTCGTCTGCGAGGAAAAGGAGGCAATGGAGAAGTGTGGGATGTCGTCGACTACGCCAACAGCAAGCAGCTTTGCGCTCTTAAACTTTCCACCTCAATTCGGCACGCCCAGCGCGAACACCACACGCAC-------------------------------------------------------------------------------------------------------------------TAAGAATTA---TGTACGAGTATTTGGATGATGATTCTGCGTACAAGGTGCTTTCACAGCTATTGAGCGCACTGAAGTACTTACACGATGGGCGTCGCTCACTACGACCTTAAACCATCGAACATTTTGATCGACCACGACGGGTGTGTGAAGTTG------ACTGACTTTGACTTGACGCGCGACGCTAAATCAGCTACGTCCAGCTCGACAGTCGGCACTCTGCGGTATCTTCCTCCCGAGTGCTTCCGCCCGAATCGAGGCGA---------TTGTGAAGCCACGGCTGAGAAGGCAGACATGTGGATGGTGGGTATTGTGTACTGCATGATGGTTACGGGTAAGCACCCGATCTGCCCAGACAAATCGACCCACGCAGAGATGAAGACGATGATGGCTCAGTACACGGGT---GAGCTTCGGTATGCACGACCTATTTCCGACCTTTCTCGGTGGATCTTACAGGGGTGCCTCCACCCGGACCCCCGATGTCGACCAACGGCCGCACAGTTGTTGGTAGCGCTTCAA

Phytophthora_parasitica TGGACTACCACCGCCAGGTGGCCAGGGAGGTGGCCGCCTTCTTCCACTCCTTCTACCGCTTCGGCGCCGCCTCCAACCGCTTCGATCTGTTCCTGCACAACGTTCTCTTTGGCTCCATGTGCATCGGAGCCGTCGTTTTCCTCGTGCTCTGCGTGATCGTCGTGCGCCGCACCATCCTACACATCCGCAGGCGTTTGTTCGACGTACATCGATGGATTCTACGTCCGTGGAGCTACTGGCCGTCGGATTACTGACATTCCTGGCGATTAGTGCGCTTGGAGGACTACTGGGAGAAGCAGTCTGCAGACCAGCAACGCCTTGCGTGTCAACGCTGACAACTTGATCACATCATTCAACGACAGTGAACTGCCAGCTGAAGCCTTCAAGATGTCCGTGGAGGCGTTGGACTCGTGCACGCGTCGAAGGAGCTACTGAACCAGACAGACGCGTTGCTGCCCAAAAACTACTCCGATATTGCCAAGGAGTGGGAATTCAGCTATTTCATGCTCAAATCGTCGACCAACGGAGCCATTCTCGCTGTAGCGCTGGCCAGTTTCTTGTCCATCGCTTCTGTTGGCTGGGCCATGGTCTCGCCGCTACGTGTGTCGATTCTCGTGATCTTGAGCGTTGTACCCATTTCACACACGCTGATCGGCGCCTACCTGTCGTCCACGATCATGACTGCGGATTTCTGTGCCGCTCCGATGAATAATACACTCGAACTGGTGGGCACGTGTCCAGCCAACGCTTCCTGCCGTTCGCTGACGTTATGGCTACGCCATGCAGCTCACGAGGGAGAAACTGGTCAGCGTATGAAGAGAGAATTCCTCGATCCCATCGGAGCAGCTTTGGGCGTCATGCTCGGCTATTTCGCTGGCACGGTCAGTAACTGGGAGAGTTTTTCACTCATCCAGACGTGGCGCTGGCCATTTCTGCTTCAGTTTCTGGCTCTGTTGCCCATTAACGTAGCCATCTTCTTCGTGCCAAAGAAACATTTAGTGATTCGCAGTGAGATCAACGAGTTGGAGCCTTCGGTGGCTGGAACTCCCATGAGCACAAGATCCACGACGTATCACGACGAAGAAGACGCGTTGCTGGCCAGCGCCAGTATGCAACGTACGGCGCACTGGACACGAGGTGTACGAACAGGGCGCGTTCTCCGAGGGCGTCTACGAACTGCTGCATATCCCCGTCTTCTGCTTGATCGTGTTCGGGCTCACGACTGTGTACTTCGTCGTCACGGGCGTGCAGTACTGGAGCACCATCTTCATGATCAAGTCGTTGCATGCGTCCAAGTATCTGGTCAACGCGCTGTTCGTCGTTGTCTCCGGTACTGGCCCAATCCTGGGCGTCTTCTTTGGTGGCTGGCTCATCGACAGATACGGAGGATACATCGGCGTGGAGCAGCGTGCCAAAGCCCTGGGAATCTGCATGATCCTGGGCCTCATCGCGTTCTCCATCTCAGCAGTGACGACGTTCTTCGACGACATCTACATCACTGCTGGGTTCCTGTGGCTCCTGCTCTTCTTCGGCGGCGCTATCCTGCCAGCGCATCGAGCGTTGGCTTCGTCCTTCTCCGTCATGGTCTTCAACCTGTTCGGCTACGCGCTCTCCCATGAGTGACCTGCAGATGGTGGTGGGCGCCAAGTGCTTCGTGCCGGATGAGACTCATGTTTGGCTCGCGGCCGAGGTACTGCGTGACGAGAATTCGGGCGATGGCAAGACACGTAAGATCTACTGCAAAGTTGAGCTGCCCGACGGAGACCGAGGAGCGCTGCGTAGACATGCTGTCCAAGAAGACAAAGGCGCTGCTGATGTGCACCAGCTCGAGTCGCTTCCTTACCAGAACGAGAACGTTGGGCCCGAGGGCATCGAGGACATGATCACTCTCAACTACTTGCACGAGGCAGCTATCCTGTTCAATATCAAGAAGCGCTTCCTCTGCGAGCTGCCCTACACGTACACAGGCGACATTTGTATCGCTGTCAACCCCTACAAGTGGCTCCCAGACCTGTACGCCGAAGAGCAGCACTTGAGGTACCTCAACCAGCCCAAGGAGGAGTTGCCCCCGCACGTGTATGCCACATCCGTCGCTGCCTACGACAACATGCGACGCTCAGAGCGTAACCAGTCCATTCTGGTCAGTGGTGAGTCCGGTGCCGGTAAAACGGAGACCACCAAGATCCTTATGAACCACTTAGCCACTATCGCCGGTGGTCTCAACAACTCCACTATCAAGAGGATTATCGAAGTAAACCCACTGCTTGAGTCCTTCGGTAACGCCAAGACTGTGCGTAATGACAACTCCAGTCGATTTGGCAAGTTCACACAGCTGCAGTTCGACAAGAACGGCACGCTTGTCGGAGCCAAGTGCCGCACGTATCTTCTGGAGAAGACGCGTGTCATTCAGCACGAAGCGCCTGAGCGTAACTACCACATCTTCTACCAGTTGACTCCTCCGACATTGCGTCGGAGTTGCAGCTCGACTCGTCAAAGCACTATGTGTACACGGGCGACAACACGGCACAGAAGATTGAAGGACTTTCTGACGAGAAACACTTCAACAAAACACGTGAGGCGCTGGAGCTTGTGGGTTTGTCTCGCGACGACCAGCGGCCACTGTTTGAAGTGCTGGCTGGTGTGCTCCACCTCGGTGAGGTGCAGTTGCAGTCGGACCCGACAGATGACGAGAAGTCTCTCATCACCGAGGGCGACAGTGGTGCGAATTCCGCCACGCAGATGCTGGGCGTCACGTGGGAGGCCCTTCAGACCGCTCTGTGTTCGCGTACCATGCGTGCTGTGAACGATGTGTACAGTGTACCGCTCAAGAAGGAGCTGGCCATGGACTGTCGCGACGCTCTTGCTAAGGCTATCTACTCCAACGTGTTCGACTGGCTTGTGACTACTATTAACCAGTCGCTTGCTGACGATGCCAACATGGCCAACCACGTGGGTGTGCTCGATATCTTCGGTTTCGAGCACTTCAAGCACAACTCGTTCGAGCAGTTCTGCATCAACTACGCCAACGAAAAGCTTCAGCAAAAGTTCACTCAGGATGTGTTCAAGACCGTGCAAATTGAGTACGAGGAGGAAGGAATCGTGTGGGACCACATTGAGTACGCAGATAACCAGGACGTGCTGACTGTGATCGAGTCGCGCATGGGTATCATTTCACTGCTGAACGAAGAGGTGATGCGACCGAAGGGTAATGAGGAGTCGTTCATGTCCAAGGTGACGTCCTTGCATAAGGAT---GATATGGCGCACGTTATTGAGTTCCCTCGTACGTCGCGTACTGAGTTCCTCATCAAGCACTACGCCGCTCCTGTCATGTACGACTCGGTGGGATTCCTGGAGAAGCACAAGGACTCACTGTTGCCTGATCTGTCCGAACTTATGCGTGGTTCATCCAAGCCGTTCATCGCCAAGCTCTTCGAGCCGAAGCCTGAGCCTAAGTCGCCGGAAAC---------GTGGTGGTGCTCTGTCCATCACGACTGTGGGTACCCAGTTCAAAGAGAGTCTGACGGAACTGATGGCTACTATCAACTCGACTCGTGTTCACTACGTGCGCTGCATCAAGCCCAACCCGATCAA---------GAGCGCGACTGCCATGGACCAAAACATGGTGGTGTCCCAGTTGCGATGTGCCGGTGTGATTGAAGCCATTCGTATCTCGCGTGCAGCATACCCGAACCGGTTGCAGCACACGGAGATCCTTGACAAATTCTGGCTGTTCGTACCGAGTGGTGGTGACACTGTTGCAGACAAATGCCAACTGCTTATGGACAAGCTGAAGTTGGAGTCCCCCACTCAGTACCAGATGGGTAAGACCCGCGTGTACTTCCAGCTAGGTGTGCTCGAAGAGCTTGAGGATCGCCGTAAGAAGTTTCTGGATGCCAAGGCCACATACCTGCAGAACATCATGGTTGGATTCACCCAGCGTATCAAGTACCTCCGTCAACTGGAAGCGATCATCAAGTTCAGTGATTCGGTGCGTGATTGCTATGCGCCGATACAACACGTTTCTCAAGGGCCTTATCTCGGCGCAAGCTCACTGGCGAGGCATGCAGGGCCGCAAGGTGGCTGCTGAAGTCAAGAGCAACCACAACGCTGTCATTATCCAGCGGTATGTGCGTGGTTTTGTCAAGCGTCACCGTTACGTCCTGATGCGCGAGATGGTTATCCGTGTCCAAGCTGTGGTTCGTATGACGATCCAGCGACCGAAGTATCTTGCTGCTCTTGAGGAGAAGCGTCGCGAGGCCGACATGGCATACCAGCTGCAGAAGCTAAAGGCTGCTCTGCAGGAAGAGCAGGAGCGGAATGCGCAGCT------CCAGCGCC---GCAGCAGTGTGGC--GACGGCACGTCCGCTGCCTCGAGCGTTGTGATGGCTGATGCTGGCGGTATGATCGAGACTCTCACTGACGAGAACAAGAAGCTGCGTGAGAAGAACGAGGACATGAAGGTGACAATGAAGGGCTTGAAGGCCGAGATCGAGAAGTTTAAGAGTGACAAGGAGTTCTCGTCGGCTGGTAACCACGTGAAGGTCCGTCAACTGCAGGACACTGTGCGTGACAAGGACAAGAAGATCTCGCAGCTCGAGGCCGAAAACAAGAAGCTGACGGAGCAGATCGCCAAACTTCACGCTGAGGGTGTTGT---TCCTGAGAAGAAGCCCACACCGAAGAAGTCGATCTTCCGCACGCTGGGCTCCAAGAAGGAGAAGAAGCCAC---------------GCGAGACTGTGCTCATGGACTCGCTCACCGGCTCGGACGACTTGAAC---CGCGAGACGCGTCCGTCGGA---GCGCCACTTCCCTCGCATGC---CGAGCATGAGTGTGGGCTCT---CGCTTCTGGAACTCGAGCAAG---AAGGGCGAC------------AACGGCGACGGCTTCGACGTCTCGGAGGTTGATGGTGAGGACAGCCCCACGAACCCACGCGCAACACGATCGTCAAGAGCATGGAAGTGGGCGTGACTGGTGCCATGTCTAGCCTCAAGGGCCGCATG---------TCGGTTGTCAAGGGGATGTACGAAGCAAGCGCACGAACAGGACCGCCCCGTGCGCGAGTCGTTCAACCTGGACGCGTTGCCGGAAGTCTCTCTTCCTGTCGGCTGGGAGGCCAAGGTGTCGCGCTCCACGGGTCGTGTGTACTACGTGAACCGCAAGCTGGGCAAGTCTCAGTTCGAACGCCCCACGCTGGCCTCTCTCAA------GGCACAGAAGCTGGCGCGCCAGAAGTCGGCTAGCGGACTG-AAGGCTGTGGTCGCTGATTTTGGCTGTGGAGATGCGAGATTGGCGGAAAGCGTGCCGAATAAAGTGCACTCGTTCGACTTGGTGTCCATGGATATTGGCGTTTACTGCTTGGCGCTCATGGGGACGAGTGTTCGTGAATATGTGCGGGAAGTGTACCGTGTGTTGAAGCCTGGAGGCGTGCTGAAGGTCGCTGAAGTCAAGAGCCGCTTCGAGAGTGAATCGCTCGGAGGATGGTGGCGGTGTTTGTGGCGGTGGCGGAGGGCGTTGTGGTGACGCTACGCAACACCACGGTGTTTGTGGAGAGACCAAATACGCAATTGTGGTGGGTCAGAAGACGGGCGAGGCTGTGGCCGTGCCCTTCCCGGACGTGGACCGCGACCTCAAGACGTTGCTGGGCCACACCACATCCATGGTCACTCACGTGGCCGTAAACCTCATCACTGCTGCTCACGGCCGACCGAGACGAGAAGCTGCGTGTATCCAGGTTTCCGAACGCTGCGGTCATTGAGAGCTACTGCTTGGGCCACGCTGCCAGTCTGACAAAAGTGGCTTGCAGTGCTGTGACCCCAGACCTTGTGGTGTCCACATCCATGGACAACACCTTAAAGCTGTGGCAGATGACGACGGGCAAGCTGGCCATTTGCCCCAAGGCGACGTTGTGGCCGTGCTTGTGAACTACCAGCATGTGCGATTCTTCGAGGTCGTGAGAACCCATTCCTGCAGTTGTTTTCGCTTTCTAAGGGTGAGCGCTGCATTTAACCGCTGTGCGCGACTTCCGCAAGCTTCAGAACGACCCGCCGTCGGGCGTGTCAGGCGCCCCCATGGACAACAATATCATGCTCTGGCAGGCCGTCATCTTCGGACCGGATGACACGCCCTGGGAAGGCGGCACCTTCAACCTGACGCTTGAATTCAGCGAAGACTACCCTAACAAGGCGCCGACTGTCAAGTTCATTACGAAAATGTTCCACCCCAACATCTACAACGACGGCCAGATCTGCTTGGACATCCTGCAGAACCAGTGGTCTCCGATATACGACATCTCGGCCATCCTCACGTCCATCCAGTCGCTGCTGTGCGATCCCAACCCGAACTCGCCCGCCAACTCGGAGGCCGCTCGCCTGTTCCAGGAGAACCGACGGGAGTACAATCGCCGCGTGCGCGAGATCGTCGAGCAGAGCTGGCAGGCCATCGATGGTGTCGGCCGCCAAGATGCGTGGCGCTGAGAACCGCATGAACGCTGGCCGCCCCTTCACCGCGTGGCTGGACAACGTCTCGTCGGACCGTGGTCTGTGCGGTGGTATCAACTCGGGTATTGCCAAGACCACCCGCAAGCAGACCGCTCGCAGATCTTCGTGATCGGTGACAAGGCCCGCGCTCAGCTGCGTCGCGACCTTGGCGACAACATCCGCGGCAACGTCACGGAGACGTACCAGTCGCCCCCCAACTTCACGGTGGCCAGTGCCATCGCTGAGGCTGTGGAGTCGGAGAGGAGTCGTCCCGTATGGCTGCTATGGAGAACGCTACCACCAACGCCGAGGAGCTCACGCTCACGCTCGTGTACAACAAGGCTCGTCAGGCCCGTATTACCACCGAGCTGATTGAAATTATTTCGGGTGCCGCCTCCCTGGACAGTGACTGGCAGCGTAGTGAGCGTCCAGAGCGCGGCACAGTTCGACGAGGCGACTGCGCGCGAGTCGACGCTGTCCGTGAGCTTCTTCTGGGCCGAATTCCAGGCCTGTCGCCCCAATGGGCAGCTGGACGTGGGGCCGTGCCGTGCTGGACAAACTCGAGGGCGCCAATGTGGCCGAACTAGCCAAGCGCGTCTCCAATACCGCCTCAAGAAGCTCATCAGCGCGTCACCGGTCATGCTCTTCATGAAGGGCAACCCCACGGAGCCCAAGTGCGGCTTTAGTCGCCAGATGGTGGCTCTCTTGAACGAGGAAAAGATCCAATTCGGCACCTTTGACATCCTGAATGACGACGAAGTGCGCCAAGGACTCAAGCAGTTCTCCAACTGGCCTACTTACCCGCAACTTTACGTGAACGGCTCGCTAATTGGCGGTCTGGACATCGTCAAGGAGATGAAGTCCGAGGGATCCATCGTCGAGCAACTGGGCGTCGAAGAGGCTGAAGCTGCCTTCCAAGAGAGTCTCCGTGCACTCGTCAACTCAGCTCCTGTACTACTCTTCATGAAGGTTCGATATCTTGTGTGGAGCTACCAACGTCAGGACCAAGACATCGCCAAGGTTGTGACTCTCACGCCACCTTCATTCCGTCGTGATATGTGCCGAGGCCATCTCATGGAGTCCGCAACCGAATGGCGATGGCTTTCCGTGATCCTTTGGAGGAAAAGGGAGAGTTCTGCATTGTGCAGCTCCCCGTATGACATCAAAGCGCACTGTTCTGCATTCACGGGGTATTTCTGCGATCGACTGGGAAGTTTACTGTGCTGCACCGAGACCACCGCAGTGAAGTCAAGTCGCTCTGTGTTCACTCTCAACGCGACTGTCTGTATACTGGTGGCCTTGACGGATTGTTGATCCGCTACGACATCAAGAAGGGTCGCACTGAAGTTGTGATGGAGCGTCGCAAACGCATCTCCAAGATCAACGCGGTGCTGGAGCATCCACACAATCCAAATCTCCTGTTGGTCAGCTCCGTGGAGCAGTAGACACAATTTGTTGATGCACGATCTGCAGCGCTACGACCGCAACGTGTCAGGAAGCTCCATGAGTCAGTATGTTGTGCCCCGGTGGTCGCCGGCAGGTTATCACGTCTCGTGTGGCAGCAAATGCGATACTGTCACGGGAGTTTCTCCACGCCCACGTCAGCCACCATCGGAGCCTCGTTCCTGCAGAAGCGCGTGATCGTCGGTGGGAAACTGACGCTGCAGATCTGGGATACAGCCGGCCAGGAGCGCTTCCGCTCCATGGCGCCTATGTACTACCGCAACGCCAAGGCAGCTATCCTGGTCTTTGATCTTCAGAGCGAGGCCAGCTTCGAGAAGATCAAAGAATGGCTCCACCTACAGCATCACGTGGGTGACGACATGTGGGCAACAAGAGCGACGTTTCGACTTTGCCAAAGCTCAGCAGTTTGCAGATGAGATCGGTGCGCTGGTGTTCCGCACGAGCGCCAAAACGGGCGAAGGAGTGCAGTCGCTGTTCGAGAGTATTGCAGTGCAGCTACTTAAGAAACACGATCAGGAGCAGCGAAGAGCTTCTACGTGGTATATATCCCGGCGGACGAGACCAAGGAGCTGGAGGAATGGACGGTCAAGCTTCCACAAGACAAGGAGGCGCAGCTCGGCTGTTTGACGGAGCGTCTGCGCGAGCATTTTAAGAGCGGCGCCATCGAAGCAGCAGCAGAGACTTTCAAGCAGCAATTACTGGCCCAGCTTCCCAAGGGTGCCACTTTGACGGATGAAATGCTGCAGATGATGTATGGACAGCCTTGTAGATTCCATTCCGCTCGTGCTCAACACGCCCGACGTCAAGCACATTGGAGGTCAAGGGCGATGCATTCATTGGTCGGGTGTTCGACAGACGACTTTGTGCGAATGGATTTCCGGTTGAGTGAGGTGTCAGGTGACGCGCCTTGGGTACAGACAGCCAAGTTGCAGAGGTCACGCAGTCGTTGGAGGAGATCTTGGCTCTGGGCCAGGGTCTGGATGACGAGGACTACAAGGCCGAGCTCGAGGTGTTGCAGAATGACCCCAACTCCAACCTCTACTCGGCCGTCACCTTCGAGGAGCTCAACCTGCCGGAACCGCTGCTCAAGGGCGTATACAACATGAAATTCGCCAAGCCGTCCAAGATTCAGTCCGTAGCGCTGCCGCTTATCCTCGCCGATCCCCCGGAGAACTTGATCGGCCAGGCTCAGAGCGGCATCCCACCCGTGAGTTAGTGCGACAGATTATGGCCGTGGTGAACGCTATGGGTAAGTTGTGGACACGTTCCTGGCCATCCCCGGCAACGATCTGGCTCGGGGTGAGACTTTGAAGGCACAGGTGATCGTCGGTACCCCCGGCAAGATCGAGGGTATGATCAAGAAGAAACAACTGGACACTCGTAACGTCAAGATCTTCGTACTGGACGAAGCTGACGTCATGGTGGCCGAGGACGGCATGCGCGAACGCTCGGTGTCTATCAAGCGATTCATCAAGAACCGCGCGTGCCAGTACCTGCTCTTCTCGGCCACATACGCTGACGATGTGCGTGATTTCGCCCAGAAAATGGTGCCCGACCACAACATTATCACGGTCAAGAAGGAGAAGCTCACGCTCGACGGCATCAAGCAGTTCTGGATCGACTGCAAGTCCCGCGAGAACAAATTCCAGGTGCTATCGGACATTTTCGCCATCCTGAGCATCGGCAAGTGCGTCATCTTCGTACAACAGCGCGAGACTGCCAAGGAACTGACGCGTCGTATGCGTGAGAAGGGCCATTCGGTCGGTATTTTACACGGCGCCGACATGGCCAAGGAGGTGCGTGACCAGGTCATCGACGAGTTCCGTGCCGGCACGACCAACGTGCTGATCACCACGAACGTTCTGGCTCGTGGTATCGACGTGGCGGGCGTCTCACTTGTCGTCAACTTCGACATCCCGCTGACTCGCGACCACCGTCCCGACCCGGAGACGTATCTGCACCGTATCGGCCGTACCGGTCGCTTTGGCCGCAAGGGCTGTGCTATTAATTTCGTGTACGACAACAAGAGCAAGCAGGACCTCGCAGAGATTGAAGAGTACTACGCACGTCCGATCACTCAGGCCCCTGCAGACGACATTGAGGAACTCGAGAAGCTACTGGCTTGGGGGGCAGCAAGCGCCGCTTGGCCAAGCTCGGCAAGGAGCAGCGCAAGGGCATGCGAATCTACCCTCGCGAGCCCAAAAAGAAGCCCTCGGGCAAGGATAAGACGTACTACTACCTCAAAGACGTCATGTTCCTGGCGCACGAACCGGTTCTTGAAAAATTCCGGGCGTTTAAAACGTTTATGAAAAAAGTGAAAAAAGCCATGGGCAAGAAGGACTACGAGGACGCCAAGCGCAAGCACGAGAACCGCCCTACCTACGAACTGGACCACATCGTTAAGGAGCGTTATCCTCGCTTTGTGGACGCTCTAGGCGATCTGGACGACGCGCTGTGTGGCAGAACTACATTGTAGCCACCAGAGCGCTGACCAAGGTGTTTGTGAGCATTAAGGGCATCTACTACCAAGCCAACGTGCAGGGTCAGACCATCACGTGGCTCGTGCCCCACCAGTTCACCCCTACAGTGGACAAGCGTGTGGATGTGCGTGTCATGCTCACATTCCTCGAGTTCTACGAGGTTCTACTCAAGTTCGTGAACGCCCAGCTCTACATGCAGATCGGCGTGGCGTATCCTCCCAAGATCAACCTCCAGTTGGACGCTGCGGGTGCTCAGTTGGCTTCTCTTGAGCTAACAGCAGAAGGAGTCCGAGAAGAGGATCAAGCTACAGCGAGAAACGCAAGGAACGCAGCGAGAAGAGCTTGTTCAGTGGCTTGACGTTCTTCCTGTCGCGTGAGGTGCCTGCTGGTTGTCTGGAGCTGGTTCTACGCTCTCACGGCGCCACGTTGGGCTGGGATGGAGAAGGCTCTCCGTTCGACGAGAAGAGCACTCGTATCACGCACCATATCATGGACCGGCCGCACCAGGGTCATCGCTACTTTAACCGCGAATACGTGCAGCCGCAGTGGGTGTTTGACTCGGTGAACAACGGCATATTGCTGCCTTTGACGCGTTACAGGAAGGAGCTGGACAAGCTCAAGTCGGCGTCGCAGGTGTTGCGTGAGGTTGGAGCTGTGGGCGATGAAGAAGAAGACGGGAGGCCTACGCTGCTGATCTTAAGGCTGAGATGGATGGTCACAAGAAGTCGAAGCGTGTTAAGAAGGAAGAGGAGGCTGCCGAGCTCAAGGAGCTGGCCAAGACGATGATGTCGAAGAAGGCCAAGCGTCTGTATGACCGCATGCAGTACGGTCTGGGCAAGAAGGCCGACAAGATTCAGAAGCTGGAGGACAAACGTGCCAAGATCGAAGCAAGACCAAGAAATTGTTGGCGGGCGCACTGTCCGGTATGATTTCCGCGTTGGCCTTCCAGCCTCTGGGGCGCTTCGCTGGCGTGGAACCTCGTCCACGCTCATTCGCGTCGCGGGCGGCGCCGGGCTCTACTTCGCCACTCTGGACCACTGCCTCAACCTTTTCCCACAAACTGCAGTCAACACCTTCCTGGCAGGAGCTTTTGCGCGTACCTTTGCAGGTGGTGTCATGTCTCCTTTCACTATTGTCAAGGCGCGAATGGATACCGCCTGGTACTTTCGATAGCAAAGGGACGTGCCGTTTTCAGGACTGTATGTGCTCATATACACGGCATCCTGCCGATGTGGTAAAGACACGCATGCATGCGACAGACTGTGGCCAAGATCTATCACCACGAAGGTCTGCGTGGCTTCGCAAAGGGAATCCTTCCACGTGTGATCAAACGAACGCTGTCCACGGCAGTCACGTGGACAATCTACGAGCTACTCGTTCGACCCATCGGGTCTGGGTCATGCCAAGGAGGCTTTCAACCTAGCCAAGGAGACGGAGCGCACCAAGCAGATGGAGAACCAGGCCAAAATTAAGGAAAACGAGGCGCTTTACAAGCAGTACGAGATCGTGCGGGTGCAGAAGGAGGGTGAGGAGCGCCGCAAGACGCTCGAGGAGGAGACGCGCCAGCACCAGCAGCGCGCACAGTACCAGGACCAGCTTAAGCGCAAGCAATACGCTGACCAGCTGGCCGCTCAGAAGTACATGAAGGAGCAGGAGCTTAAGAAGCAGGAGGAAATTCTGGCCCGCCAGGAGGCTTCGCGCCGTAAGACGCTCGACTACGAGGCGGAGCTGCGTCAGAAGACTGAGCTTGCTAAGGTTGCGGCTGAGACTGAGGGGCGCATCAAGCAGGAACGTCTCAACCACGACCTGCACCTCGAGGAGGCTCGTGTGCGTGCCAAGGAGTACCGCGAGACTGTCATGGAGGGTATTAAGCTGGCCGGTAACACTGTCGGCTCCGGTATCATGACCTTTGTGGACGACAAGGAGAAGCTCACGACCACTGTGGTCTCTCTGACGGCTCTGGCGATCGGTATCTACACCGCAAAGGTGTCCACCAGCGTTGCGGGCAAGTATATTGAGGCTCGTATGGGAAAGCCGTCGCTAGTGCGTGAGACGTCGCGTCGCTCAGTTACGCAAGTGTTGGCCAACCCCATCCCGTCCATCAAGCGCGCTCTGCGTCTACAGAAGGCGACGGATGCCCTGGAGGGCGTCGTGCTGGAGCCCAAGCTGGACGAGCGTCTGCGCAGCGTGGCTGTGTCCACGTTCAACACGAAGAAGAACCGTGCGCCGTTCCGCCACTTGCTACTGCACGGCCCGCCCGGTACGGGTAAGACTCTGTTCGCCAAGGCACTTGCTCGCCACTCCGGCCTCGAGTACGCTATCCTGACAGGTGGTGACGTCGCCCCACTGGGTCGTGAGGGTGTGACGGAGATTCACAAGCTGTTTGACTGGGCGTCCCACAGCCGTCGCGGTCTGCTGCTCTTTGTCGATGAGGCCGATGCGTTCCTGCAGAAGCGTAGCAACACCGTCATGAGCGAGGACATGCGTAACGCACTGAACGCCTTCCTGTACCGCACGGGTGAGGCCAGCGACAAGTTCATGATCGTGTTCGCCAGTAACCAGCCAGAGCAGTTCGACTGGGCCATCAACGATCGTATCGATGAGATGGTGGAGTTCCGCCTGCCTGGATTCGACGAGCGTGTGCGCATGCTAAAGCAGTACTTTGACGACTACATTCGTGCGCCGAAGAACTCGCGTGCCAAGAAGATCTACGCGAGGGCGTCATCTTCACGCTGGACCCTGTGGCCAACTTCCTCGTGCCAAGACGCGTATCTTCCAGCTTGAAGCGTTGCAGGGCGTGTCCGGTGAAGCTCAGGCTATTTTCGACGCACTCAACAAGAACCCATGCGCATCTACTGGGTCGTATTCGTTGTAGTGCTGGGCAACGGTCTATATACGTATTTCGCCGGCAAGGACGAGTCGTACCTGGTTGAGAAGCTTAAGAGAAAAATCCCGTCCGAGTACCGCATCTCCGAGAAGTGGGACAAGTGCCTGGAGAACTTCACGCTCTACTTTGGCGCTGGCCTCGTGGCTGGTGGCCTTACGTCCCTCGTGCTGGCTCGTTCTGGAGCTGGTCGCGGACTGGTGACGGGTCTGGGCGTGCAGGTGCGGGATCGAGCTGGACGACGTGCCAACTGGCGTTTTCGGGTAACACACAGCAAGCTCTGAACAAGACGGACAAGGCTGTGGGCGACTTGCCGGCCTTCCAGCAGCAGACGATCGTGGACTTCGTGACGGCCTCGGCCACGCAGCAGCAGCTGCAGGCCGTTCATGCGTGGCTTCTGCGAGGAGTCCAGTGTGAAGGTCTTGGAGACGTATCTGGCCGAGCAGGTGCAGAACAAGACCGTGGACATTGACGCCAGTCTGGCGCTGCTCAAGCTGTACCAGGTGTACCCGGCCACAATCAGCGCCGAGAGCCAAGATTCTGGTCAAGGGCGTCATGGCCCTGCCCTCCACATTCTTCACCGGCGTCCACGATGGTCCCGGAGAGCATCCGCGAGGACGCCAACGTGACGGCAGTGCTTCACACGGGTTTCATGCTCCAGTCGTGCCTGTTCGAGGATTTCTGGAAGGAGAGCGTGACTTTCGCCGAGAAGGTGCCAGGCTTCCTGGAGTCGGTGCGCGCCTACATTCTGACAGCTATCAGCCGCAGCCACAGCGCCATCTCCACGGAGGTCTTCAAGGCTAAACTCAACGTGTCTGACAAGGAGGTGGCCGACATCGTTGCCGAGAAGTGGACGCCGACAACTTGATTCAGATCAACCCCAACGAGGACAACCAGATGCAAGCCAAGAAGGTCCAGGAGAACATTGAGTTCGAGGACGTGCTCAAGGTCATCCACACTCTTTCTAGATCCAACGTCTGCGCCAGTGGCGGTGGCGTGGCTGATGCTGAGGAGGTGTGTGAGAATCTAGCGAAGCACTTGAGACTGGACGGAGGCTCCCAGGTGTTGCCCTGCTCTACGGGTGTCATCGGTTGGCGAATTCCTGTAGACGCTATGGTCGAGAACCTTCCGAAACTCGTCACCAATCTGCAGTCCAACTCGGTTCTACCTGCTGCTGAGGGCATCATGACCACCGATTTGTACCCGAAGATCCGCTCCATGGACGTGTGCGGTGGCCGCATTGTCGGCATTGCCAAGGGTGCTGGTATGATCGAACCCAATATGGCGACTATGCTGTCCTACATCCTCACCGATCTATCGATTCCGCGTGACGTTTTGCGTCAGCTACTGACGGAGGTGGTTGGAGACACGTACAATTCGCTCAGTGTGGACACGGACCAGAGCACGTCCGACACGGTGACGCTCGTGTCGTCGGACCAGATCCCGTTCGACATGAC---ACAGCTGGAAGAGTTCAAGAAGGCACTGCATGAGGTGTGTCTTGGACTCAGTGAGGACATTGTACGCAACGGTGAAGGTGCGCACCACGTGATGCGCGTGCGTGTAAGCGGTGCGTTGTCTAAGGATATGGCTAAGGGTGTCGGCAAGTCGGTGGTCAACTCGCCACTGCTCAAGTGTGCGGTGGCAGGCAACGACCCGAACGTGGGTCGTCTAGTTATGGCTGTGGGTAAGTACATGGGATCGTACCACAAGGATGTGAACATTCAACGTCGCATGAAGATCCGAATGGGTGGTGTCGTCATCTTCGAGAACGGCGAGATGGTGCTCAACGATGATATTGAGGCCAAGTTGGTGGCCCACATGCGCGACGCGATGCTGATTGAGCCCACTCGCGGTGGACTTGATGCCAGCTCACACAACTATCCGCCGCACGGAAAGTTTGTCGAGATTGAGATTGACCTGGGTGTTGGCTCCGAGGAGACTCAGGTGTTGGGCATTGACCTTACGCACGAGTATGTGGCCATTAACGCCGACTACCGCTCG--ATGGCGCC---GAAAAAGGGGAAAACTGCCGCCGCGTATCTGGCGTCTCTGGGTGTGGCCCCTGC---TGCTGGAGGTGAGAAGAAGGACGAT---AAGAAGAAGAAGAAAAAGGGCAAGAAGAAACCCAAAAGAAACCCATGTCGGCTGCGGCCAAGGCTCTGA-TGGAGCGTCAGGCGGCCATGAAGGCCGAGCAGGAGCGCATCGAGCAGGAGCGTCTGGAGCAGGAGCGCATCGCCCAGGAGGAAGAGGCCAAGCGTCTGGCCGAGCTCGAGCGCGAGGAGGAGATCCGTCGCAAGAAAAAAGAGGCGCGCGCCGCTAAGATCGAGCGTCAGAAGAAGGAGGGAACGTACATGACCAAGGCACAGAAGCAGAAGGCTGCCAAGGCTCAGGCCGCGCTTGAGGCGATGCAGCAGGCGGGCCTCGTTCCTGAGGCTGCC------GGCGAGAAGAAGAAGGTTGTGTACTCGGACAACAAGAAGAAAAATAAGAACAACAGGAAGAAGAGGAGGAGGAGGCGGAGGCTCCCGATGCGTGGGATGACGAGGACTGGGAGACCAGTGACGTTGTGGCCTCGCTGGAGGATAAGCTAACAGAAGAAGGAGCAGGAGCGTCTGCGTCAACAGGGCATCGCTCTGATGAAACGCCAGGAGGAACAGGCTGAGCAGATGCGTCAGGAGGCCGAAGAGGAGCGTCAGCGCTCCAAGGCCAAGCTCGAGGCTGAACGCAAGAAGAAGGAGGCGGCCGAGCGTCGTCGTGAGCGTGAGGCCGCTGCGCGTGCCAACGTGTCCCTGGACAACCTGCGTTCGCCTATTTGCTGTATTATGGGCCACGTCGATACTGGTAAGACGAAGCTTCTTGATAACATCCGCCGGACCAAGGTGCAGGACGCCGAAGCTGGTGGTATCACGCAGCAGATTGGTGCGACCTTCTTCCCTGTGGAGGCTATTAAGCAGAAGACGGCACGCCTTGCCGAGACCATGAAGCTCGAGTACCGCCTGCCCGGTCTGCTTGTGATCGATACGCCCGGTCACGAGTCGTTCACGAACCTGCGTACTCGTGGCTCTTCGCTCTGTGATATCGCCATTCTGGTGGTGGATATCATGCACGGTCTTGAGCCGCAGACTCTGGAATCTCTGCGTCTACTGCGTCAGAAGAAGGCCCCGTTTGTGGTGGCGCTGAACAAGATCGATCGTTGCTACGGCTGGAAAACTATGCCCGACATGCCCGTCCAGGAGGCGCTCAAGCATCAGAACGAGCACGTCATTCGCGAGTTTGAAGATCGCATGAAGTCGATCATTGTGGAGTTTGCAGAGCAGAAGCTGAACGCCGAGGTGTATTGGCGCAACAAGGACTTGGCGCACACGGTCTCGCTCATCCCGACTAGTGCTATCTCGGGTGAAGGTGTGCCGGATCTGCTGATGATGCTGACGCGATTGACACAGGAGCGCATGGCCAAGTCGCTGGCATTCGTCGACATCCTACAGTGTACCGTGCTCGAAGTGAAGGTCATTGAGGGTCTTGGTACAACGATTGATACGATTCTGGTTAATGGTACACTAGAGGAAGGCGCAACAATTGTAGTGTGTACATTGGACGGACCTGTTGTGACAACGATCCGTTCCTTGCTGACCCCTCACCCCATGAAGGAAATTCGAGTCAAGGGCGAGTACCTTCACCACCAGAAGATGAAGGCTGCTATGGGTGTGAAGATTTGCGCCCAGGGCCTCGAGAAGGCAGTTGCTGGTACTCAGATTCATGTGGTTGGCCCCGATGATGACGTTGATGAGCTGAAGGACTCCGTCATGTCCGATCTGACTGGAATTCTGGACTCCGTGAAGGCTACCCGCCGCGGTGTCATGGTTCAGGCTTCCACCCTTGGTGCCCTGGAGGCACTACTGGAGTTCCTGCGCACGTGTGACCCGCCCATTCCTGTGTCCTGTGTGAACATCGGTCCTGTCCACAAGAAAGACGTCATGCGTGCTTCGGTGCAGTTGGAGCACCAGCCTGAGTTTGCCACCATCTTGGCGTTCGATGTGAAAGTCCATAGTGACGCTACTGAGCTGGCCACTGAGCTTGGTGTGCGTATTTTCACGGCCGACATCATTTACCACCTGTTCGATCAGTTCACGGCGTACATGGACAACTTCCGCATGGCCCGTCGCGAGGAGTTTGCCGAGGTGGCTGTGTTCCCGTGTGTGTTGAAGATTTTGCCCAACTGCGTCTTCAACAAGAAGGACCCGATTATTCTTGGTGTGGACGTCGAGGAAGGTATCCTCAAGGTCGGCACTCCGCTGGTGGTGCCGTCCGCTGGTG---GCTTCCTAGTCGGTAAGGTTGGCTCCATTGAACGCGAGCACAAGGAGGTGGATCGCGCCAAGAAGGGCGCGTCCGTGGCCGTGCGGATAGACAACGAGAGCAGCGTCATGTACGGCCGTCACTTCGACCACAAGAACAAGCTAGTGTCGCGTCTGACACGCCAGAGCATTGATGCCCTGAAGGAGAATTTCAGGGAAGACTTGCAGAAGGAGGACTGGCAGCTCGTCATCAAGCTCAAGAAGGTGTTCGATATCATTATGGAGGGCTGGCTCACCAAGCGCCGCGACCACATGAATCTCATCTGGCTGGATCGCTACTTCCTCCTGGACGGCAACCAGCTCCGCTACTACCGCAAACGCGGCGACCCTGCACCTCGAGGGACCTACATTCTGACTGACGGTTGCGTAGTGAGTCCCGTGTTCAATTCCGAGGAGAAACACAAACATCACGGACCCGTCTGGATGTTCCGCATCACTTGAAAAGTCGTACAAGAAGGACCGATTCCTGGATCTAGGAGCCAAGAGCGAGGAGAACGCCAAGGAATGGAAACGCGCGCTGGAGAACGCCGTGCGGTCGCTGCAGGAGCTCGTAGCGCACCAAAAGAAGCTCAAGAACGACGCCGAGAT---CTTCCCCACGCCCACCCAC---------GTGCCGAGGATGTTGAAGGCCGATGAAGACACGATGTCGCTCTTTAATCTGGACGCAGACAACGTCAAGGCAGAGCAGGCCTGGTGGCTAGCCAGAGTGGAAGGAGGGCTCAGAATCATGCAGGAATGTCCTCTCGGACCCAACGTTGCGGCCAATCTGTACGCTCAACACGCAGCGACAACCAACGGACTGATCTTCAGTGCATGTGTGGGCGCAGCGCTGAGTTTCAGCAGCATGTATTCGTTCGCGAACAACATGTTGCTGAGCTTTATGACTGCGCTAGTGGCTATTGCTGTGTGCTGCGCTGCAGTGACGCCCACACAGGCTCCTGAGATCCCGAGCTTCCGTGTGTCCCAGGTGGTGCATGGCTCCCCGACTGAAGTCTTCAGACTCATCATGAACTCCAAGAGATTCCAGAGATGGGACTCGGCCACGGCGACTATGCGCGTGATCCAGCAGCTGGACGACCACGCGGACATCGTGTACATTACGCAGCGACCGACGCACTTGTGGCCATTGTGGCAGAAAGCTCGAGACCTTGTGCTCATGCGCTACTGGAGACGCGAAGAGGATGGGTCGTACTTTGTCATGTACCAGAGTATCGAGCATCCCGAGTGCCGTGTACGTCACAATTATGTGCGTGCGAACTTGCTGGGCGGCGGCTTCGTCATCGCTCCTCAGCGTGTTCCCAGT---GGATCTATCCGTACTCTGGTTACCTACGTGCTACGTTACGATCCTGGAGGCTG---GTCCCGTATCTACCATCAGCTCGGAATGGATGTGGATCTCGTGATGCCGATGCTGCGTAGTGTCGTGGGGATTCGGGACGAAATGAGCGCTACGGACTTCATCACGCCCAATGTATCAATGGCTCCTGC---AGAGAACGGAGACTGGACCGGAAGTGTGGA---CTCGATGGGTGTCGTTATGGGCGTACAGAAGCTGCAG---ACCAGTCTTCCCGAGAAGATGTGGGGCGAGCCGGACGCTTCGCTCTTCAGTGTTCGTGGGCATAACTACTTGACCGACAAGAAGAAGATCCCGTCAGCGCCTGCGATGTTCCACACTGTGGGTGTGGATCTCTTGTCGTTCGAGAGTCCAGCTGAGAGGTACAACATCTCGTCTCGACCGGACAGTATCGGTCGCACCAG---CTCCAAGTTCACGTTCGTAGTGAACATGATCATCCCTGGCCCAGAGAACGTCTGCATGGTCTTCTACTTCCAGCCAGTGCGTGACAATGTGTTCGAGGACGGCTCGGGCTTCTCCGAGCTGCTGAACGACTTCTTCGACGGCGACGACCAGTTCCGGAACTCGCGCTTCAAGTTGATTCCTACGGTCGTAGAGGGAAGCTTCATCATCAAACAGTCGGTCGGTTCCAAGCCCACTCTACTCGGCAACAAGCTGAAGTGTCCGTATCATCGTGGAGAAAACTACTTTGAAGTGGACATCGACATCTCGTCCAACTCGGTTGCCAACACGGTGGTGGGGATGGTGCAAGGCGTGACCAAGTCGCTGGTGGTGGACTTGGCCTTCCTGCTCGAAGCCCAGACCGACGAAGAGCTGCCTGAAGTCATCTTGGGCGCTGTCCGCATGCAGCACATTTCGCTGGACAATCCACGCCGCATGCCCAAGCATGGGGAACGGGCAGCAAGGACGAGTTCACGTTCCGAGGCCTTTACCGAGGCTCTGCGTGTGAATCTGCAGCGACTCATCGTCTACGCGCGTTCGGCCGATACGGCGCTGCAGCGAGAAGTCGCCGAGAAGCTGGCAAATGAAGCAGTTAAGACCGACAGGTGCAGATTGTGGAGCTGGACGGACTGCAGCTGCTGCTGCCACTGACCAAGTCGAAGGACACGGAAGTGCAGCGTCTAGCAGCCCACGCACTTGCCAATCTCTCAGTCAACTCTGAGAACCAGTCCAAAATGGCCACGGAAGGAGGTATCGACATGCTGATTGATCTATTGAGCAGTACGAACGAGCACGTGCAGCGGCAGGCCGCGAAGGCGCTTGCCAACCTCGGTGTGAACGTGGACAACAAGGAGAGGATCGCTAAGGCTGGCGGCATTAAACCGCTCATTGACTTGGCCAGCTCCCGCCAGATCGGTGTAGCCGTCGAGGCCATCGCAGCACTGGCCAATCTGGCTGTCAACG---ACGCCAATGAGGTCGAGATCGCACGTAAAGGAGGTTTAAAGCCAATTATCGACGGTGCGCACTCTGAATCCGTCGAGCTTCAGTCCCAAGTGGCTCGTGCACTACGCAACTTATCGGTCAATCCGGAGAACAAACAAGCCATTGTGGAGTTGGGAGGCGTCGAGGCTCTGCAATCACTCGTACGTTCCACTAATGATCGGATATGCCAGCAAGCTACACGCGCCCTCGTCCCCAACGTGGTACTCACGCGCTCGTCGCCCACCATCTGTTG---GCTGTAGCGCCAGTAGCTCGCGTGGCTGAGATCAAGGAGGAAGC------TATGCCCTACGATGGCGCAATGGTGAACGTCGAGTACTCTACTACAGTCAAGACGGTC---------ATAGTGCCTGCAGTAAAGCCCA---ACGACGCGACCGGG---GGCTTCTCGTCCATCTCCGACCGTTTCCCTCTCTCCAGAGTGGGGAAAGTCAGCTGTGGTATGGCTCCAATTCCCATGAGCCG---CTTGCTCAATG---CCACACCGGAGTTGACAC---AGGAGGACCAG-------------ATGACTGTACTCGTGGAGGACGGGACGGTGGATGAGAAC---ACCATTAAAGCCGAG---CCGGAAGGTGAGCGATGTGTACAAGTACCAGGCCACTGA--TGTGGAAGAGAACGAGATGGAGCAACTTCGATGGAATATTCAAGAGCAGGTTTTCCAGCGGTACTTCGAGCAAGGACAGAAGTTTGAGGAATGGGGACACCTGGAGGAGAAAGACTCCGAGTGTTCTTCGTTGCTGGTGAACGAAGAGAGAACATCATGGCCGAGAAGCGGTTCCACGACAATGCTGTCCGAGATGCCATGGGCAAAGGAAACGCCACGTGGGTCAAGGAGCGACGAGATCGGTTCGAGGCTGAAATCCGCAGATTCTACGACGAAACCAGACACAAACAGGAGTTGATGCTCACGCGGCTTGAAAAAGTGTCGCCATTCTCAAAATTCCCGATTCTTGGCAACCGGTTTCTTCTGCTTCGTCTGCGAGGAAAAGGAGGCAACGGAGAAGTATGGGATGTCGTGGACTACGCCAACAGCAAACAGCTCTGCGCTCTTAAGCTCTCCACTTCAATACGACACGCCCAACGCGAACACCACACGCACACTCAACCACCCCA--ATATTGTTCAGGTCGGCGAGGCACCCTTCCTTATCCGATATCAGCAACAGCAATACACTGCGTTCACTGTTGCCAGCGTTGAGTCAGATCTTCAGCAACTCATTGAGA---TGTACGAGCACTTCGATGATGACTCTGCATACAAAGTGCTTTCTCAGCTTCTGAACGCCCTGAAATACTTGCACGATGGGCGTTGCTCATTACGACCTGAAGCCGTCGAACATTCTCATCGCCCAGGGCGGATGTGTGCAGCTT------ACCGACTTCGACTTGACTCGCGACGCCAAGGCAGCAACGTCCAACTCTACCGTTGGCACTTTGCGATATCTTCCTCCTGAGTGCTTCCGTCCGAACCGTAGCGA---------CTGCGAAGCGACGGCTGAGAAGGCTGATATGTGGATGGTGGGCATCGTGTACTGCATGATGGTCACTGGAAAGCACCCAATCTGCCCGGACAAATCGATGCACGCTGAAGTAAAAGCGATGATGGCTCAGTACACGGGC---GAACTTCGATATGCACGACCCATTTCTGATCTGTCTCGGTGGATCTTACAAGGCTGCCTGCATCCAGACCCGCGATGTCGACCAACGGCCGCACAATTGCTAGTGGCGCTCCAG

Phytophthora_plurivora TGGACTACCACCGCCAGGTGGCCCGGGAGTTGGCCGCCTTCTTCCACTCGTTCTACCGCTTCGGCGCCGCCTCCAACCGCTTCGACCTGTTCCTGCACAACGTCCTCTTTGGGTCGTTATGTATCGGAGCCGTCGTCTTCCTCGTGCTCTGCGTCATTGTGGTGCGCCGCACAATCCTGCACATCCGCAGGCTTTTGTTCGCCGCACGTCGATGGAATCCACGTCCGTGGAGCTACTGGCAGTCGGACTATTGACTTTCCTCGCTATCAGTGCACTAGGAGGATTACTGGGAGAGGCTGTCTCCAGACCAGCAACGCTCTGCGCGTCAATGCAGACGGCTTGATCACGTCGTTCAACGACAGTGAGCTGCCTGCTGAAGCCTTCAAGATGTCGGTGGAGGCTCTGGGCTCGTGCACGCGTCCAAGGAACTGGCCAACCGGACGGACGCGCTGCTGCCCAGTAACTACTCGGAGATCGCCAAGGACTGGCAGTTCAGCTACTTCGTGCTCAAGTCCTCCACGAACGGGGCGATCTTGGCTGTAGCTCTCGCCAGCTTCTTGTCCATCGCGTCGGTTGGCTGGGCCATGGTTTCACCACTTAGAGTCTCGATTCTGGTGATTCTGAGCGTTGTACCCATCTCGCACACGCTGATCGGCGCGTACTTGTCGTCGACGATCTTGACGGCGGACTTTTGCGCCGCGCCACTGAACAACACGCTCGGTCTGGTTGGTACGTGTCCTGCCAATGCGTCCTGCCTTTTGCCGATGCTATGGCAACGCCATGCAGCGCAACAGGGAGAAATCGGCCAGCGGATGAAGAAAGAGTTCCTGGATCCAATCGGAGCAGCTCTGGGCGTCATGCTCGGCTATTTCGCTGGGACCGTCAGCAATTGGGAGAGTTTTTCACTGATCCAGACGTGGCGGTGGCCGTTTTTGCTTCAGTTTGTGGCACTTTTGCCGATTAATGTGGCGATTTTCTTCGTGCCAAAGAAACACTTGGTGATTCGCAGTGAGACGGGCGAGATGGAGCCGCCCGTGGCTGGAACGCCCATGAGCACGAGATCCACGACGTACCACGACGAGGAGGACGCGCTGCTGGCCAGTGCCAGCATGCGACGTACGGCGCTTTAGACACGAGGTGTACGAACAGGGTGCGTTCTCGGAGGGCGTCTACGAGCTGCTGCACATTCCCGTCTTCTGCTTGATCGTCTTCGGTCTTACGACCGTGTACTTCGTCGTCACGGGCGTACAGTACTGGAGTACCATCTTCATGATCAAGTCCCTACACGCGTCCAAGTACCTGGTGAACGGGCTCTTCGTCGTCGTGTCCGGTACTGGCCCCATCCTGGGCGTCTTCTATGGCGGCTGGTTAATCGACCGCTACGGAGGTTACATCGGCGTGGAGCAGCGCGCGAAGGCTCTGGGCATTTGCATGATCCTGGGCCTCACGGCGTTCTGTATCTCGGCGGTCACGACGTTCTTCGACGACATTTACATGACCGCCGGCTTCCTGTGGCTGCTGCTCTTCTTCGGAGGCGCGATCCTGCCAGCGCACCGCGCGCTGGCGTCCTCGTTCTCCGTCATGGTCTTCAACCTCTTCGGGTATGCGCTGTCGCATGAGCGACCTGCAGATGGTGGTGGGGGCCAAGTGCTTCGTGCCGGACGAGACCCACGTGTGGCTTGCGGCCGAAGTCCTGCGCGACGAAAACTCGGGCGACGGCAAGACCCGCAAGATTTACTGCCAGGTGGAGCTGCCCGACGGAGACTGAAGAGCGCTGCGTGGATATGCTGTCCAAGAAGACTAAGGCGCTGCTGACGTGCACCAGCTGGAGTCGTTGCCCTATCAGAACGAGAACGTGGGCCCTGAAGGTATTGAGGACATGATCACGCTCAACTACCTGCACGAAGCTGCCATTCTCTTCAATATCAAGAAGCGTTTCATGTGTGAGCTACCATACACGTACACGGGCGACATTTGCATCGCCCTCAACCCTTACAAGTGGCTCCCGGACCTCTACGCTGAGGAGCAGCACCTCCGCTACCTCAACCAGCCCAAGGAGGAGCTTCCCCCGCACGTCTACGCCACGTCAGTGGCTGCTTACGACAATATGCGGCGCTCGGAACGCAACCAGTCCATCCTGGTGAGTGGAGAGTCCGGTGCTGGTAAGACGGAGACCACGAAAATCCTCATGAACCACTTGGCTACCATCGCCGGCGGTCTGAACAACTCTACTATTAAGAGGATTATCGAGGTGAACCCACTGCTCGAGTCCTTTGGTAATGCCAAGACCGTCCGTAACGACAACTCCAGTCGATTCGGAAAGTTCACACAGCTGCAATTCGATGCGAACGGCACGCTTGTCGGCGCCAAGTGCCGCACATACCTGCTCGAGAAGACCCGTGTGATCCAGCATGAAGCACCTGAACGCAACTACCACATCTTCTACCAGTGGACTCCTCTGAGATTGCACCGGAGCTGCAGCTTGACCCCAGTAAGCACTACGTGTACACGGGCGACAACACGGCGCAGAAGATCGAAGGCCTCTCGGACGAGAAGCACTTCAACAAGACACGCGAGGCGCTTGAGCTAGTGGGTCTGTCCCGCGACGACCAGAAGCCGCTCTTTGAGGTGCTAGCTGGTGTACTTCACCTTGGTGAGGTGCAGCTGCAGTCTGATCCGGCTGATGACGAGAAATCTCTCATCACTGAAGGTGACAGTGGTGCGAACTCCGCGACTCAGATGCTCGGAGTCACCTGGGAGGCGCTTCAGAACGCTCTGTGTTCGCGTACAATGCGTGCTGTAAACGACGTGTACAGCGTGCCACTGAAGAAGGAGCTGGCTATGGACTGCCGTGATGCACTCTCCAAGGCCATCTACTCGAACGTCTTCGACTGGCTTGTGGAAACAATTAACCAGTCGCTTGCTGACGATGCCCATATGGTCAATCATGTCGGTGTCCTTGATATCTTCGGCTTCGAGCACTTCAAGCACAACTCGTTCGAGCAGTTCTGTATTAACTACGCCAACGAGAAACTGCAGCAAAAGTTCACGCAGGATGTGTTCAAGACCGTGCAGATCGAGTACGAAGAGGAAGGCATCGTGTGGGACCACATTGAGTACGCTGATAACCAGGATGTGCTGTCCGTGATTGAGTCGCGCATGGGTATCATTTCGCTATTGAACGAAGAGGTGATGCGACCGAAGGGTAACGAGGAGTCCTTCATGTCCAAGGTGACGTCCCTGCACAAGGAT---GACATGGCACACGTCATTGAGTTCCCTCGTACGTCCCGCACAGAATTCCTCATCAAGCACTATGCTGCTCCTGTGATGTACGATTCGGTGGGCTTCCTGGAGAAGCACAAGGACTCGTTGCTGCCTGATCTGTCCGAGCTCATGCGTGGTTCGTCCAAGCCGTTCATCGCCAAGCTCTTCGAACCCAAGCCGGAGCCCAAGCCGCCGAAAGC---------GTGGTGGTGCTCTGTCCATCACGACTGTCGGTACCCAGTTCAAGGAGAGTCTGACAGAGCTGATGGCTACTATTAACTCGACCCGTGTGCACTATGTGCGTTGCATCAAGCCCAACCCGATCAA---------GAGCTCGACGGCCATGGATCAAAACATGGTGGTCTCCCAATTGCGATGCGCTGGTGTGATCGAAGCCATTCGTATCTCGCGTGCAGCATACCCGAACCGATTGCAACACACTGAAATCCTGGACAAGTTCTGGCTGTTTGTTCCGAGTGGCGGCGAGACTGCAGCAGACAAGTGCCAACTGCTTATGGATAAGCTGAAGCTGGAGTCACCGACGCAGTACCAGATGGGTAAGACTCGCGTGTACTTCCAGCTTGGTGTGCTTGAAGAGCTTGAGGACCGTCGTAAGAAGTTCCTGGATGCCAAGGCCACCTACCTGCAAAACATCATGGTTGGCTTCACGCAGCGTATCAAGTACCTCCGACAACTGGAAGCGATCATCAAGTTCAGTTATTCGGTGTGTGATTGCTATGCGCAGATACAACATGTTCTTGAAGGGCCTCATTTCGGCACAGGCACACTGGCGAGGCATGCAGGGCCGCAAGGTGGCTGCCGATGTCAAGAGCAACCACTACGCTGTCATCATCCAGCGGTATGTTCGTGGACTTGTCAAGCGTCACCGTTACGTTAAGATGCGCGCAATGGTCATCCGTGTGCAGGCTGTGGTTCGTATGACGATCCAGCGACCAAAGTACCTGGCTGCCCTTGAGGAGAAGCGTCGTGAGGCGGACATGGCTTACCAGCTGCAGAAGCTTAAAGCTGCTCTGCAGGACGAGCAGGAGCGGAATGCTCAACT------GCAGCGCC---GCAGCAGCGTGGC--GACGGCTCGTCCGCTGCCTCGAGCGTGGTCATGGCTGACGCTGGTGGTATGATTGAGACTCTCACGGACGAGAACAAGAAGCTGCGTGAGAAAAACGAGGACATGAAGGTGACGATGAAGGGTTTGAAGGCGGAGATCGAGAAGTTTAAGAGTGACAAGGAGTTCTCGTCTGCTGGTAACCACGTTAAAGTCCGGCAGCTGCAGGATACTGTGCGTGACAAGGACAAGAAGATCTCTCATCTTGAGGCCGAAAACAAGAAGCTCACGGAGCAGATCGCGAAGCTTCATGAGGACGGTGTTGT---TCCCGAGAAGAAGCCCACGCCGAAGAAGTCGATCTTCCGCACGCTGGGTTCCAAGAAGGAGAAGAAGCCTC---------------GAGAGACTGTGCTGATGGACTCACTCACTGGCTCGGACGACCTTAACCGTCGGGAAACGCGTCCTTCGGA---GCGCCACTTCCCGCGCATGC---CTAGCATGAGCGTGGGCTCT---CGCTTCTGGAACTCTAACAAA---AAGGGTGAC------------AACGGCGACGGCTTTGACGTCTCGGAGGTTGACGGCGAGGACAGCCCCACGAACCCACGCGCAACACGATCGTGAAGAGCATGGAAGTGGGCGTGACCGGTGCCATGTCGAGTCTGAAGGGCCGCATG---------TCGGTCGTGAAGGGCATGTACGAAGCAAGCGCACGAACATGACCGCCCCGTGCGCGAGTCGTTCAACCTAGATGCGTTGCCGGAGGTGTCTCTGCCTGTCGGTTGGGAAGCGAAGGTGTCGCGCTCGACGGGTCGCGTGTACTACGTCAACCGCAAGCTCGGCAAGTCCCAGTTCGAGCGCCCCACTTTGGCGTCGCTGAA------GGCGCAGAAGCTGGCGCGCCAGAAGTCGGCTGGCGCGCTGTAAGGCTGTGGTCGCCGATTTCGGCTGTGGAGATGCTAGACTGGCCGAGAGTGTGCCGAACAAGGTGCACTCGTTCGACTTGGTGTCCCTTGACATTGCCGTGTACTGCTTGGCTCTCATGGGGACAAGTGTTCGCGAATATGTGCGAGAAGTTTATCGCGTGCTGAAGCCTGGAGGTGTGCTGAAGGTAGCTGAAGTGAAGAGCCGCTTTGAGAGCGAATCCATCGGTGGATGGTGGCGGTGTTCGTAGCGGCGGCCCAGGGCGTCGTAGTGACTGTACGCAATACGACGGTGTTCGTTGAGAGATCAAGTACGCGGTTATTGTGGGCCAGAAGACTGGAGAAGCTGTGGCGGTTCCTTTCCCAGACGTTGACCGGGACCTGAAGACTCTACTGGGTCACACTACATCCATGGTAACTCACGTGGCTATGAACCTCGTCGCTGCTGCTTACAGCCGACCGTGATGAGAAGCTGCGGGTGTCGAGGTTCCCCAATGCTGCAATTATTGAGAGTTACTGCTTGGGCCACGCTGCGAGTCTCACGAAGGTGGCGTGCAGCGCTGTAACGCCGGACCTGGTGGTGTCCACGTCCATGGACAACACACTAAAGCTGTGGCAGATGTCCACAGGCAAACTGGCGATCTGCCCCAAGACGACGTTGTGGCGGTGCTCGTGGACTACCAGCACGTGCGATTCTTCGAGATCGTGAGGCTCCGTTTCTGGAGTTGTTTTCGATCTCCAAGGTCTACCGCTGCGTTCACCTGCTGTGCGCGACTTCCGCAAGCTGCAGAACGACCCGCCGTCTGGCGTGTCGGGCGCTCCCATGGACAACAACATCATGCTCTGGCAGGCTGTCATCTTCGGGCCGGACGACACGCCCTGGGAAGGAGGTACCTTCAACCTGACGCTCGAGTTCAGCGAGGACTACCCAAACAAGGCGCCGACCGTCAAGTTCATCACGAAAATGTTCCACCCCAACATCTACAACGACGGTCAGATCTGCCTGGACATCCTGCAGAACCAGTGGTCTCCGATCTACGACATCTCAGCCATCCTCACGTCCATCCAGTCGCTGCTGTGCGATCCCAACCCGAACTCGCCGGCCAACTCGGAGGCCGCGCGTCTCTTCCAGGAAAACCGACGGGAGTACAACCGCCGCGTGCGTGAGATCGTCGAGCAGAGCTGGCAAGCCATCGATGGTGTCGGCCGCCAAGATGCGTGGCGCTGAGAACCGCATGAACGCCGGCCGCCCCTTCACCGCGTGGCTGGACAACGTCTCGTCGGACCGTGGTCTGTGCGGTGGTATTAACTCGGGTATTGCCAAGAACACCCGCAAGCAGACCACTCGCAGATCTTCGTGATCGGCGACAAGGCTCGCGCCCAGCTGCGTCGCGACCTGGGCAACAACATCCGTGGTAACGTGACGGAGACGTACCAGTCGCCCCCGAACTTCACGGTGGCCAGTGCCATCGCCGAGGCTGTCGAGTCGGAGAGGAGTCGTCGCGTATGGCTGCCATGGAGAACGCCACCACCAACGCTGAGGACCTCACGCTTACGCTCGTGTACAACAAGGCTCGCCAGGCTCGTATTACCACGGAGCTGATTGAAATTATCTCGGGTGCCGCCTCCCTGGACAGTCACTGGCAGCGTCGTGAGCGTGCAGAGCGTGGCGCAGTTCGACGAGGCCACCGCGCGCGAGTCCACTCTGTCCGTCTCCTTCTTCTGGGCCGAATTCCAGGCCTGTCGCCCCAACGGCCAATTGGACGTCGGGTCGCGCCGTGCTGGACAAGCTGGAAGGCGCCAACGTGGCGGAACTGGCCAAGCGCGTCTACAGTACCGTCTTAAGAAGCTCATCAGTGCTTCTCCTGTCATGCTCTTCATGAAGGGCAACCCGACGGAGCCCAAGTGCGGCTTTAGTCGGCAGATAGTGGCTCTGCTTAACGAAGAGAAGATCCAGTTCGGTACTTTCGACATTCTTAACGATGACGAAGTGCGTCAAGGGCTCAAGCAGTTCTCCAACTGGCCCACGTACCCGCAGCTGTACGTGAACGGCACGCTCGTTGGCGGTCTGGACATTCTGAAGGAGATGAAGTCGGACGGATCTATCGTCGAGCAGCTCGGCGTTGAAGAAGCGGAAACTGCTTTCCAGGAGAGTCTGCGTGCACTGGTTAACTCTGCTCCTGTGCTGCTGTTCATGAAGCTTCGACATTTTGTATGGAGCTACGAGCGTCAGAAGCAAGAAGTCGCGAAGGTGGTGAGTTTGACCCCTACGTCGTTCCGAAAGGAAATGTGCTGAATCCATTTCGTGGAGCCCGAAACCGAATGGCTATGGCATTCCGTGATCCTGTGGAGGATATGGGGGAGTTCTGCATTGTGCAGCTTCCCGTATTACCTCGAACCGCACTGTTTTGCATCCTCGGGGTATTGCTGCGATTGACTGGCAATTTTAATGTGCTCCACCGAGACCACAAAAGCGAAGTCAAGGCGCTTTGTGTGCACTCAACGCGTGACTGTTTGTACACGGGTGGTCTGGACGGACTGGTGATTCGTTATGACCTAAACAAGGGTAACAGGAATGTCCTGATCGAGCGTCGCAAGC--ATTTCCAAGATCAACTCGATTCTGGAGCACCCGCACAACCCGAATTTGCTCTTGGTCAGCTCGATCGAGCATAACGCAGAATCTGTTGCTGCACGACCTTCAGCCGTATGACCCCAACGTGTCAGGCACCGGAATGAGTCAGTACCTGGTTCCACGGTGGTCGCCTGCTGGTTACCACGTCTCCTGTGGCAGTAAATGCGCTACTGCCACGGCAGCTTCTCCACGCCCACGTCGGCCACCATCGGAGCTTCGTTCCTACAGAAGCGCGTGATCGTCGGGGGGAAGCTAACGCTGCAGATCTGGGACACGGCAGGTCAGGAGCGCTTCCGCTCGATGGCTCCAATGTACTACCGCAACGCTAAGGCGGCCATCCTGGTGTTCGATCTACAAAGCGAGGCCAGCTTCGAGAAAATTAAAGAATGGCTACATCTGCAGCATCACGTGGGCGACGACATGTAGGTAATAAGAGCGACGTTTTGACTTCTCCAAGGCTCAGCAGTTCGCAGATGAGATCGGCGCGCTCGTATTCCGGACGAGCGCTAAGTCGGGCGAGGGAGTGCAATCACTGTTCGAGAGTCTGGCCGTTCAACTGCTTAAAAAGCATGACCAAGAACAGCGGAGAGCTTCTACGTGGTCTACGTGCCTGCAGAGGAGAGCAAGGAGCTGGAGGAGTGGACGGTGAAGCTGCCCAAGGACAAGGAAGCGCAACTGGGATGTCTTACGGAGCGTCTGCGCGATCACTTTAAGCGGGGATCCATCGGAGAAGCAGCAGAGACCTTCAAGCAGCAATTGCTGGCTCAGCTGCCTAAAGGCGCCACTATGACCGATGAAATGCTGCAAATGATGCATGGACAGTCTGGTGGATTCCATTCCTCTCGTACTTAACACGCCCGACGTCGAGCACGTTGGAGGTGAAGGGTGATGCTTTTATTGGTCGGGTGTTCGATGGACGACTTCGTTCGCATGGATTTCCGGTTGGGGGAGGTGTCGGGTGGTGCGCCCTGGGTGCAGACAGCCAAATTACAGAGGTCACGCAGTCGCTGGAGGAGATCCTGGCGCTGGGCCAGGGACTGGACGACCAGGACTACAAGGCCGAACTGGAGGTGCTGCAGAACGACCCCAACTCGAACCTCTACTCTGCCGTCACCTTTGAGGAGCTCAATCTGCCGGAACCGCTGCTCAAGGGCGTGTACAACATGAAGTTCGCCAAACCATCTAAGATTCAGTCGGTGGCGCTGCCCCTCATCCTCGCCGATCCTCCAGAGAACTTGATCGGTCAGGCTCAGAGCGGCACCCCACTCGTGAGCTGGTGCGACAGATTATGGCCGTGGTGAACGCCATGGGCAAGTTGTGGAGACGTTCTTGGCCATCCCCGGCAACGATCTGGCACGTGGTGAGACGCTGAAGGCTCAGGTGATCGTCGGAACCCCCGGCAAGGTCGAGGGCATGATCAAGAAGAAGCAGTTGGACACCCGCAACGTCAAGATCTTCGTGCTGGACGAGGCCGATGTGATGGTTGCAGAGGACGGGATGCGCGAGCGGTCCGTGGCTATCAAGCGCATGATTAAGAACCGCGCGTGCCAGTACCTATTGTTCTCGGCCACATACGCTGACGACGTGCGCGACTTCGCGCAGAAGATGGTGCCGGACCACAACATTATCACGGTCAAGAAGGAGAAGCTCACGCTGGACGGCATCAAGCAGTTCTGGATCGACTGCAAGACCCGTGACAACAAGTTCCAGGTGCTGTCGGACATTTTCGCCATTTTGACCATCGGCAAGTGCGTTATCTTCGTGCAGCAGCGCGAGACGGCCAAGGAGCTCACGCGTCGTATGCGTGAGAAGGGCCACTCGGTTGGCATCCTGCACGGTGCCGACATGGCCAAGGAAGTGCGTGACCAGGTCATCGACGAGTTCCGTGCCGGCACGACCAACGTTCTGATCACCACCAACGTGCTGGCCCGCGGTATTGACGTGGCGGGTGTTTCCCTTGTCGTGAACTTTGATATCCCGCTGACGCGTGACCACCGCCCGGATCCGGAGACGTACCTGCACCGTATCGGACGTACGGGTCGTTTCGGCCGCAAGGGCTGTGCTATTAACTTCGTGCACGACAACAAGAGCAAGCAGGACCTCGCGGAGATCGAAGAGTACTACGCACGCCCTATTACGCAGGCTCCTGCTGACGACATTGAGGAACTCGAGAAAATGTTGGCCTGGGGGGCAGCAAGCGCCGCCTGGCCAAGCTCGGCAAGGAGCAGCGCAAGGGCATGCGCATTTACCCTCGGGAGCCTAAGAAGAAGCCGTCGGGCAAGGACAAGACGTACTACTACGTCAAAGATGTGATGTTCTTGGCGCACGAACCGGTTTTGGAAAAGTTCCGAGCTTTTAAAACCTTCATGAAGAAGGTTAAAAAGGCCATGGGCAAGAAGGACTATGAAGATGCGAAACGCAAGCACGAGAACCGTCCGACGTACGAGCTGGACCACATTGTCAAGGAACGGTACCCTCGCTTTGTGGACGCTTTGGGAGATCTTGATGACGCACTGTGTGGCAGAACTACATTGTCGCCACTCGTTCATTGACAAAAGTGTTCGTGAGTATCAAGGGGATCTACTACCAGGCCAATGTCCAGGGTCAGACCATCACGTGGCTGGTACCTCACCAGTTCACGCCTACAGTGGACAAACGTGTGGATGTGCGTGTGATGCTGACGTTCCTGGAGTTCTACGAGGTGTTGCTCAAGTTCGTCAACGCCCAGCTCTACATGCAGATCGGTGTCTCTTACCCTCCTAAGATCAACCTCCAGTTGGATGCTGCTGGCGCTCAACTGGCAGCGTTGGAGCTAGCAGCAGAAGGAGTCCGAGAAGCGTATCAAGCTACAGCGAGCAGCGCAAGGAGCGCAGCGAGAAGAGTCTGTTCAGTGGATTGACGTTCTACCTGTCACGTGAAGTGCCATCGGCTCCTCTCGAGCTGGTGCTGCGTTCGCACGGCGCTACACTTGGCTGGGACGGGGAGGGTTCTACTTTCGATGAGAAGAGCTCTCGCATCACGCACCACATCATGGACCGCCCGCACCAGGGCCACCGCTACTTTAACCGGGAGTACGTGCAGCCGCAGTGGGTGTTTGACTCAGTGAACAACGGGATTCTACTGCCTTTGGCGCGCTACCGTAAGGAGCTGGAGAAACTCAAGTCGGCTTCGCAGGTGCTGCGCGAGGTTGGAGCCGTTGGGGACGAGGAAGAGGACGGGAGGCCTACGCTGCTGATCTCAAGGCTGAAATGGACGGTAACAAGAAGTCGAAGAAGGCCAAGAAGGAGGAGGAGGCTGCGGAGCTTAAGGAGCTGGCCAAGACCATGATGTCGAAGAAGTCCAAACGTCTGTACGACCGTATGCAGTACGGTCTGGGCAAGAAGGCCGACAAGATCCAGAAGCTCGAGGACAAGCGTGTCAAGATTGAGGCAAGACCAAGAAGCTGTTGGCGGGAGCGCTGTCGGGCATGATCTCGGCGCTGGCGTTTCAGCCTCTGGGGTGCTTCGCTGGCGTGGCACGTCCCCCACGCTCGTGCGTGTAGCCGGCGGTGCAGGCCTGTACTTCGCTACACTGGACCATTGCCTCACCATGTTCCCGCACACTGCAGTCAACACGTTCTTAGCTGGAGCCTTCGCACGCACATTCGCGGGCGGCGTCATGTCGCCCTTTACTATTGTCAAAGCGAGGATGGATACCGCCTGGTACCTTCGACAGTAAAGGGACGTGCCGTTCTCCGGCCTGTATGTGCTCATTTACACGGCATCCTGCCGACGTGGTTAAAACGCGCATGCATGCGCCAGACGGTGGCCAAGATTTACCGCCACGAGGGCCTGCGCGGATTCGCAAAGGGAATTCTTCCGCGCGTGGTAAAGCGAACACTGTCCACCGCTGTCACCTGGACAATCTACGAGCTACTCGTTCGACCCGTCCGGTCTAGGCCACGCCAAGGAGGCTTTTAACTTGGCTAAGGAGACGGAGCGCACCAAACAAATGGAGAATCAGGCCAAGGTGAAAGAAAACGAGGCCCTGTACAAGCAGTACGAGATCGTGCGCGTGCAGAAGGAGGGAGAGGAGCGCCGCAAGACCCTCGAAGAAGAGACGCGCCAGCACCAGCAGCGTGCACAGTACCAAGACCAGCTCAAGCGCAAGCAATATGCCGACCAGCTGGCCGCTCAAAAGTACATGAAGGAGCAGGAACTCAAGAAGCAGGAGGAAATCCTCGCTCGCCAGGAGGCCTCGCGCCGCAAAACACTCGACTACGAGGCTGAGCTGCGTCAGAAAACTGAGTTGGCCAAGGTCGTGGCCGAAACCGAGGGCCGTATCAAACAGGAGCGTCTTAACCACGATCTCCACCTCGAGGAGGCCCGTGTTCGCGCCAAGGAGTACCGCGAGACCGTCATGGAGGGTATTAAGTTGGCTGGTAACACGGTCGGCTCCGGTATTATGACCTTCGTTGATGACAAGGAGAAACTCACGGCTACTGTGGCCTCTCTGACGGCCCTGGCTGTTGGTATCTACACCGCTAAAGTGTCTACCAACGTGGCGGGCAAGTACATCGAGGCTCGCATGGGCAAGCCGTCGCTTGTTCGCGAAACCTCGCGTCGCTCGGCTACACAGGTGCTTGCGAACCCCATCCCGTCCATCAAGCGCGCGCTTCGTCTTCAGAAGGCTACGGATGCGCTCGAAGGCGTCGTGCTGGAGCCTAAGCTGGACGAGCGTCTGCGCAGCGTGGCTGTGTCCACTTTCAACACAAAGAAGAACCGAGCGCCGTTCCGCCACTTGCTGCTGCACGGCCCTCCTGGTACGGGTAAGACGTTGTTTGCCAAGGCTCTGGCGCGTCACTCCGGTCTGGAGTACGCTATCCTTACGGGTGGTGATGTTGCACCTCTGGGCCGTGAGGGTGTAACGGAGATCCACAAGCTGTTCGACTGGGCGTCTCACAGCCGTCGTGGTCTGCTACTGTTCGTTGATGAGGCTGATGCGTTCCTGCAGAAGCGTAGCAACAGCGTTATGAGCGAAGATATGCGCAATGCACTGAATGCCTTCCTGTACCGCACCGGTGAGGCCAGCGACAAGTTCATGATCGTGTTTGCCAGTAACCAGCCGGAGCAGTTTGACTGGGCCATCAACGATCGTATCGATGAAATGGTGGAGTTCGGCCTTCCAGGCTTTGATGAGCGTGTGCGCATGCTGAAGCAGTACTTCGATGACTACATCCGTGCGCCAAAGAACTCGCGAGCGAAGAAAATTTACGCGAGGGCGTTATCTTCACGGTGGACCCCGTGGCCAACTTCCTTGTCCGAAGACGCGGATCTTCCAGCTTGCAGCCTTGCAGGGCGTATCAGGCGAAGCTCAGAGCATTTTCGATGCCCTTAACAAGAGCCCATGCGCATCTACTGGGTTGTCTTCGTTGTCGTGCTGGGCAATGGATTGTACACGTACTTTGCCGGGAAGGACGAGTCGTTTCTGGTGGAGAAGCTCAAGAGAAAAATCCCGTCTGAGTACCGCATCTCCGAGAAGTGGGACAAGTGCCTGGAGAACTTCACGCTCTACTTTGGTGCCGGCCTCGTGGCTGGCGGCCTCACGTCTCTCGTGCTGGCGCGCTCGGGCGCTGGTCGTGGACTTGTGGCAGGACTGGGCGTGCGGGTGCTGGATCGAGCTGGACGACGTGCCAGATGGCGTTTGCCGGTGACGCACAAGCAGCTCTGGACAAAACGGACAAGGCTGTGGGCGACTTGCCGGCCTTCCAACAGCAGACGATCGTGGACTTCGTGACGGCCTCGGCCACGCAGCAGAAGCTGCAGGCTGTGCACACGCGGCTTCTGCGAGAAGGACAGTGTGTCCACCTTGGAGACGTACCTGGCCGAACAGGTGCAGAACAAGTCCGTGGACATCGACGCTAACCTGGCGCTGCTCAAGCTCTACCAGGTGTACCCTGCCACGATCAATGCCGAGAGCTAAGGTCCTGGTTAAGGGCGTCATGGCGCTTCCCTCCACCTTCTTCACTGGCGTCCACGATGGTACCTGAGAGCATCCGTGAGGACGCTAACGTGACGGCGGCGCTGCACACGGGTTTCATGCTGCAGTCGTGCCTGTTTGAGGACTTCTGGAAGGAGGTCGTGAGCTTCGCTGAGAAGGTGCCAGGCTTCCTCGAGTCGGTGCGCGCTTACATTCTGACGGCTATCAGCCGCAGCCACAGCGCTATCTCTACGGAGGTTTTCAAGGCCAAGCTTAACGTGTCTGACAAGGAGGTGGCCGACATTGTCGCCGAAAAGTGGACGCGGACAACTTGATTCAGATCAACTCCAACGAGGACAACCAGATGCAGGCCAAGAAGGTCCAGGAGAACATCGAGTTCGAGGACGTGCTCAAGGTCATCCACACCCTTTCTAGATCCAACGTCTGTGCCAGCGGTGGCGGTGTGGCTGATGCAGAGGAGGTCTGTGAGAACCTGGCCAAGCACCTGAGGCTCGAGGGAGGCACACAAGTGTTGCCATGCTCCACTGGCGTCATCGGTTGGCGTATCCCTGTCGACGCTATGGTCGAGAACCTGCCCAAGCTCGTGAACAACTTGCAGTCCGACTCGGTGCTTCCTGCTGCTGAGGGAATTATGACCACTGACTTGTACCCGAAGATCCGCTCCATGGACGTGTGCGGCGGTCGTATCGTTGGTATTGCCAAGGGTGCGGGTATGATCGAGCCCAACATGGCTACGATGTTGTCCTACATCCTCACCGACCTATCGATTCCGCGTGACGTGCTGCGTCAACTGCTGACGGAGGTGGTGGCAGACACGTACAATGCGCTCAGTGTGGACACCGACCAGAGCACGTCCGACACGGTGACGCTCGTTTCGTCCGACCAGATCCCGTTCGACCTGAA---GCAGCTGGACGAGTTCAAGAAGGCGCTACATGAGGTGTGTCTTGGGCTGAGCGAGGACATTGTGCGCAACGGCGAAGGCGCCCACCACGTCATGCGCGTGCGTGTGAGTGGCGCCTTGTCCAAAGAAATGGCCAAGGGAGTGGGCAAGTCGGTGGTCAATTCGCCGCTTCTCAAGTGCGCGGTGGCTGGCAACGACCCGAACGTGGGCCGTCTGGTCATGGCCGTGGGCAAGTACATGGGTCTGCACCACCAGGATGTGAACATCCAGCGTCGCATGAAGATCTGGATGGGTGGCGTTCTCATCTTCGAGAACGGTGAGATGGTGCTCAACGACGAGATTGAGGCCAAGCTTGTGGCGCACATGCGCGACGCTATGTTGATTGAACCCACGCGCGGAGGCCTCGACGCCAGCTCCCACAACTACCCGCCGCACGGAAAGTTTGTCGAGATGGAGATTGACTTGGGTGTCGGATCTGAGGAGACGCAGGTGCTGGGCATCGACTTGACGCACGAGTACGTGGCCATTAATGCCGACTACCGCTCGTAATGCCGCC---GAAGAAAGGGAAGACCGCCGCCGCATATCTGGCGTCTCTGGGCGTCGCTCCCGC---CGCTGGAGGTGAGAAGAAGGACGAC---AAGAAGAAGAAGAAGAAAGGAAAGAAGAAACCGAAAAGAAGCCCACGTCCGCCGCAGCCAAAGCTCTGA-TGGAGCGCCAGGCGGCCATGAAGGCTGAGCAGGACCGCCTGGAACAGGAGCGTCTGGAGCAGGAGCGTCTCGCTGAGGAGGAGGAGGCCAAGCGCCTAGCCGAGATCGAGCGCGAGGAGGAGATCCGCCGCAAGAAGAAGGAAGCGCGTGCCGCCAAGATCGAGCGCCAGAAGAAGGAAGGAACGTACATGACCAAGGCTCAGAAGCAGAAGGCCGCCAAGGCTCAGGCTGCTCTGGAGGCTATGCAGCAGGCGGGTCTCGTCCCTGATGCTGCT------GGCGAGAAGAAGAAGGTCGTGTACTCGAACAATAAGAAGAACAAGAAGAACAACAAGAGGAAGAG---GAGGAGGACGAGGCCCCCGACGCGTGGGACGATGAGGACTGGGAGACCAGTGACGTCGTTGCCTCACTGGAGGACAAGCTAGCAGAAGAAGGAGCAGGAGCGTCTGCGCCAGCAGGGTATCGCGCTGAAGAAGCGACAGGAGGAACAGGCCGAGCAAACGCGACAGGAGGCGGAGGAGGAGCGTCAGCGTTCGAAGGCGAAGCTTGAGGCCGAGCGCAAGAAGAAAGAGGCAGCTGAGCGTCGTCGCGAGCGTGAGGCTGCTGCACGTGCCAACGTGTCCCTAGACAATCTGCGTTCGCCTATTTGCTGTATCATGGGCCACGTCGATACTGGTAAAACGAAGCTTCTCGACAACATTCGTAAGACCAAGGTGCAGGATGCCGAAGCCGGTGGTATTACGCAGCAGATCGGTGCTACATTCTTCCCTGTGGATGCTATTAAGCAAAAGACTGCGCGCCTCGCTGAAACGATGAAGCTGGAGTACCGCTTGCCTGGTCTGCTTGTGATCGATACGCCTGGTCACGAGTCTTTCACAAACCTGCGTAATCGTGGTTCTTCGCTGTGTGATATCGCCATTTTGGTGGTTGACATCATGCACGGTCTGGAGCCCCAGACGCTCGAGTCACTGCGCCTGCTGCGCCAGAAGAAGGCCCCGTTTGTGGTGGCTCTTAACAAAATCGATCGTTGCTACGGCTGGAAGACTATGCCCGACATGCCTGTTCAGGAGGCCCTCAAGCACCAGAACGAGCACGTCATCCGCGAGTTCGAGGACCGCATGAAGGCGATCATCGTGGAATTTGCTGAGCAGAAGCTGAACGCTGAGGTGTACTGGCGCAATAAGGACTTGGCGCGCACGGTCTCGCTCATTCCTACCAGTGCTATCTCGGGTGAAGGTGTGCCGGATCTGCTGATGATGCTGACCCGATTGACACAGGAGCGTATGGCGAAGTCGCTTGCGTTTGTGGACATCCTACAGTGTACTGTGCTCGAAGTGAAGGTCATTGAGGGTCTCGGTACGACGATTGATACGATCCTGGTAAACGGTACTCTGGAAGAAGGTGCAACTATTGTAGTGTGTACATTGGACGGACCAGTCGTGACAACGATTCGTTCGCTGCTGACCCCACACCCCATGAAGGAAATCCGTGTCAAGGGAGAGTACATTCATCACCAGAAGATGAAGGCTGCAATGGGTGTGAAGATTTGCGCGCAGGGCCTTGAGAAGGCCGTTGCTGGTACTCAGATTTACGTGGTTGGCCCTGATGACGATGTTGAAGAGCTGAAGGACTCCGTTATGTCCGACCTGACGGGCATCTTGGACTCCGTGAAGGCCACCCGCCGTGGTGTCATGGTTCAAGCTTCCACCCTGGGTGCCCTCGAGGCCCTTCTGGAGTTCTTGCGTACGTGTGACCCGCCTATTCCGGTATCGTGTGTGAACATCGGCCCCGTACACAAGAAGGATGTTATGCGCGCCTCAGTACAGCTGGAGCACCAGCCTGAGTTCGCCACTATCTTGGCTTTCGATGTGAAGGTTCATAGCGACGCTACTGAGCTGTCCTCAGAGCTTGGCGTGCGTATTTTCACGGCCGATATCATTTACCATCTTTTCGACCAGTTTACAGCGTATATGGACAACTTCCGTATGGCTCGCCGTGAGGAGTTCGCCGAGGTGGCTGTATTCCCGTGCGTGTTGAAGATCCTGCCTAACTGTGTTTTCAACAAAAAGGACCCAATCATTCTCGGTGTAGACGTCGAGGAGGGTATTCTGAAGATCGGCACCCCGCTCGTGGTCCCATCCGCCGGAG---GCTTCCTGGTCGGCAAAGTGGGCTCCATCGAGCGCGAACACAAGGAGGTGGATCGCGCCAAGAAGGGCGCGTCTGTGGCTGTGCGGATAGACAACGAGAGCAGCGTCATGTACGGTCGCCATTTCGACCACAAGAACAAGCTGGTGTCGCGCCTCACCCGCCAGAGCATTGACGCGCTGAAGGAGAATTTCCGGGAAGACCTGCAGAAGGAGGACTGGCAGCTTGTCATCAAGCTCAAGAAGGTTTTCGATATCATTATGGAGGGCTGGCTCACCAAGCGCCGTGACCACATGAACCTCATATGGCTGGAGCGCTACTTCCTCTTGGACGGCAACCAGCTCCGCTACTACCGCAAACGCGGCGATCCCGCGCCCCGAGGGACCTACATTCTGACTGACGCCTGTGTGGTGAGCCCCGTGTTTAATTCCGAGGAGAAACACAAGCACCACGGCCCAGTTTGGATGTTCCGCATTACCTGAAAAGTCCTACAAGAAGGACCGATTCCTGGATCTTGGGGCCAAGAGTGAGGAGAACGCAAAGGAGTGGAAACGGGCGCTGGAGAACGCCGTCCGCTCGCTGAGGGAGCTGGTGGCGCACCAGAAGAAGCTCAAAACGGACTCCGAGAT---CTTTCCCACGCCCACGCAC---------GTCCCGAGGATGCTGAAGACGGACGAAGACACCGTGTCTCTCTTTAATCTGGACGCTGACAACGTCAAGGCGGAGCAAGCCTGGTGGCTGGCCAGAGTGGAAGGAGGCCTCCGCATCATGCAGGAATGTCCACTTGGCCCCAATGTCGCCGCCAATCTGTTCGCGCAACGTGCAGCCACGACCAACGGACTGATCTTTAGTGCATGTGTAGGTGCAGCTCTGAGCTTCAGCAGCATGTACTCGTTCGCGAACAACATGCTGCTGAGCTTCATGACGGCGTTGGTGGCCATTGCCATCTGCTGCGTCGCCGTGACGCCTACGCAGGCGCCCGAGATCCCGAGCTTCCGAGTCTCGCAGGTGGTGCATGGGTCTCCGACTGAAGTTTTCCGACTCATTATGAACCCCAAGAGATTCCAGCGTTGGGACTCTGGAACGGCCACGATGCGGGTGGTACAGCAACTGGATGATCACGCGGACATTGTGTACGTGACGCAGCGACCAACGCACTTGTGGCCGCTGTGGCAGAAAGCTCGTGACCTTGTGCTCATGCGATACTGGAGACGTGAAGAGGACGGATCGTACTTTGTCATGTACCAGAGCATCGAGCATCCGGAGTGTCGTGTGCGTCACAACTACGTCCGTGCGAATCTATTGGGTGGTGGCTTTGTTATCGCTCCCCAACGCGTTCCAAGT---GGATCCATCCGTACGCTGGTCACGTACGTGCTTCGCTACGACTCGGGCGGTTG---GTCGCGCATTTACCACCAGCTCGGCATGGACGTGGACCTGGTGATGCCAATGCTGCGCAGTGTTGTGGGTATTCGGGACGAAATGAGCGCTACAGACTTCATCACGCCTAATGTTTCGGTGGCTCCTGCGGGCGAAGGCGGCAGCTGGGCAAGAAGCGTGGA---CTCCATGGGCGTCGTCATGGGCGTGCAGAAGCTTCAA---ACGAGTCTTCCGGAGAAGATGTGGGCCGAGCCGGATGCTTCTCTCTTCAGTGTGCGAGGCCACAACTACCTGAACGACAAGAAGAAGATCCCGTCGGCCCCAGCGATGTTCCACACCATGGGAGTGGATCTCCTGTCGTTCGAAAGCCCAGCGGAGAGATACAACATCTCGGCCCGTTCGGACAGCATTGGCCGCACCAG---CTCCAAGTTCACGTTCGTAGTCAACATGATTATCCCTGGCCCGGAGAACATTTGCATGGTTTTCTACTTCCAGCCGGTGCGCGACAACGTCTTCGAGGACGGATCGCCCTTCTCGGAGCTGCTGAACGACTTTTTCGACGGCGACGACCAGTTCCGTAACTCGCGATTTAAGCTCATCCCCACTGTCGTCGAGGGCAGCTTCATCATCAAGCAGTCAGTCGGTTCCAAGCCGACGCTCCTCGGCAACAAGCTTAAGTGTCCGTACCACCGCGGAGAGAACTACTTCGAGGTGGACATCGACATTTCGTCCAACTCGGTGGCCAACACGGTCGTGGGAATGGTGCAGGGTGTGACCAAGTCGCTGGTGGTTGACATGGCGTTCCTGCTGGAGGCCCAATCCGACGAAGAGCTGCCCGAGATCATCCTAGGCGCTGTTCGCATGCAGCACATCTCGCTCGACCACCCACGCCGCATGCCCAAGCATGGGGAACGGGGAGCAAGGACGAGTTCACCTTCTGACGCCTTCACGGAGGCTCTGCGCGTGAATCTGCAGCGTCTGATCGTCTACGCGCGCTCGGCCGACACAGCGCTGCAGCGGGAAGTCGCTGAGAAGTTGGCCAACGAGGCTGTTAAGATCGACAGGTGCAGATCGTAGAGCTGGACGGACTGCAGCTGCTGCTTCCTCTCACCAAGTCCAAGGATACGGAGGTGCAGCGCTTGGCTGCACACGCACTCGCCAACCTCTCGGTCAACTCTGAGAACCAGTCGAAAATGGCGACGGAGGGGGGAATCGACATGCTCATTGATCTCTTGAGTAGCACAAATGAGCACGTACAGCGGCAGGCTGCGAAGGCGCTCGCCAACCTCGGCGTGAACGTCGACAACAAGGAGAGGATCGCCAAGGCGGGTGGCATTAAACCGCTCATCGACTTGGCCAGCTCCCATCAGATCGGCGTCGCTGTCGAGGCCATTGCGGCGCTTGCAAATCTGGCAGTCAACG---ATGCCAATGAGGTGGAAATCGCTCGTAAAGGTGGCTTGAAGCCTATTATTGACGGCGCGCACTCCGAGTCCGTGGAGCTTCAGTCCCAAGTGGCTCGAGCACTGCGCAACTTGTCTGTCAACCCGGAGAACAAACAAGCGATCGTGGAGCTGGGGGGAGTGGAGGCTCTGCAATCCCTTGTGCGTTCCACCAACGACCGGATATGCCAACAGGCTACCCGCGCGCTCGTCCCCAACGTGGTGCTCACGCGCTCCTCACCCACAATTTGCTG------GTGGCGCCTGTAGCTCGCGTGGCTGAGGTCAAGGAAGAGGC------TGTGACTTACAACGGAGCTATGGTGAGCGTCGAGTACTCGGCTGCTGTCAAAGCAGTCGTGGTTCCCGTGGTTCCTACGGCGAAGCCCA---ACAACAAGGCTGGA---GGTTTCTCGTCCATCTCCGACCGTTTCCCGCTTTCTAAAGTGGGGAAAGTCAGCTGCGGAATGGCACCTATTCCCATGAGCCG---TTTGCTCAATG---CTACACCGGAGATGACTC---AGGTAGACCAG-------------ATGACTGTGCTTTTAGAAGATGGCACGGTGGATGACAAC---ACCATCAAGGCCGAG---CCGGAAGGTGAACGACGAGTACAGGTACCAGGCCACTGT--AGAAGAAGAAAACGAAATGGAGCAACTTCGATGGAACATCCAAGAACAGGTCTTCCAAAGATACTTCCAACAAGGACAGAAGGACGAGGAATGGGGGCGACTGGAAGAGCAAGACACCGAGTATTCTTCATTCCCGGTGGACGGCGAGAAAACATAATGGCTGAGAAGCGATTCCACGACAATGCCGTCCGCGACGCTATGGGAAAGGGTAATGCTACATGGGTCAAGCAGCGACGGGAACGGTTTGAGGTGGAAATTCGCCAGTTCTACGATGAAACTCAGCATAAGCAGGAGCTGATGCTTACCCGTCTTGAAAAGGTGTCTCCGTTCTCCAAATTTCCGATTCTTGGCAATAGGTTTCTGTTGCTTCGTCTGCGAGGAAAGGGAGGCAACGGAGAGGTGTGGGACGTCGTTGACTACGCCAACAACAAGCAGCTCTGCGCTCTCAAGCTGTCCACTTCAATTCGACACGCTCAGCGCGAGCATCACACGCACACTTAGCCACCCCA--ACATTGTTCACGTCGGCGATGCGCCCTTCCTGATTGGATACCAGCAGCAGCAATATACTGCATTCACCGTTGCTAGCGTGCACTCCGATCTACAGCAAATCATCGAGA---TGTACGAGTATTTGGATGACGACTCTGCGTACAAGGTTCTTTCACAGCTATTGAGTGCGCTGAAGTACTTGCACGATGGGCGTTGCTCACTACGACCTGAAGCCGTCGAACATTCTGATCGACCACGACGGGTGTGTAAAGCTG------ACCGACTTCGATTTGACTCGCGACGCCAAGGCAGCTACTTCCAACTCGACCGTTGGAACTCTTCGGTACCTTCCCCCTGAGTGCTTCCGACCGAACCGTAGTGA---------CTGCGAAGCCACTGCTGAAAAGGCAGACATGTGGATGGTGGGCATCGTGTACTGCATGATGGTTACAGGAAAGCACCCGGTCTGCCCGGACAAGTCAACACACGCAGATGTGAAGACAACGATGTCTCAGTACACG--------------------------------------------------------GGGGTGTCTACACCCAGATCCGCGGTGTCGGCCAACGGCTGCACAGTTGCTTGTGGCACTCCAT

Phytophthora_pseudosyringae TGGATTACCACCGCCAGATGGCCCGGGAGCTGGCCGCCTTCTTCCACTCCTTCTACCGCTTCGGCGCTGCCTCCAGCCGCTTCGACTTGTTCCTGCACAACGTTCTGTTCGGCTCAATGTGCATCGGCGCCGTCGTCTTCCTCGTGCTCTGCGTCATCGTCGTGCGCCGCACCATCCTGCACATCCGCAGGCCTTTGTCCGACGCACGTCGATGGATTCCACCGCGGTGGAGCTGCTGGCAGTCGGGCTGCTCACCTTCCTGGCTATTAGCGCCCTTGGGGGGCTCCTGGGAGAAGCTGCCTGCAGACAAGCGGCGCCCTGCGCGTCAATGCTGACAATTTGATCACATCGTTCAACGACAGCGAGCTGCCAGCTGCAGCCTTCAAGATGTCGGTGGAGGCGCTGGACTCGTCCACGCCTCCAAGGACCTGAGGAACCAGACAGACGCGCTGCTGCCCAAGAACTACTCGGACATCGCCAAGGACTGGGAGTTCAGCTACTTCGTGCTCAAGTCCTCGACGAACGGCGCGATCCTGGCGGTGGCGCTCGCCAGCTTCTTGTCCATTGCGTCGGTTGGATGGGCCATGGTCTCGCCGCTGCGCGTCTCGATCCTGGTGATCTTGAGTGTTGTGCCCATTTCGCACACGCTGATCGGCGCGTACTTGTCGTCGACGATTATGACGGCAGACTTTTGCGCTGCCCCGATGAACAACACGCTCGGACTGATCGGCACATGTCCAGCTAATGCGTCCTGCCTTTCGCTGATGCGATGGCTACGCCACGCGGCTCGCCAGGGAGAGATCGGCCAGCGCATGAAGAAAGAATTTCTGGACCCCATCGGAGCAACTGTGGGCGTCATGCTCGGCTACTTTGCCGGCACCGTCAGCAACTGGGAGAGTTTCTCACTCATCCAGACGTGGCGCTGGCCGTTTTTGCTCCAGTTCGTGGCGCTGCTGCCGATTAATGTAGCCATCTTCTTCGTGCCCAAGAAACACCTCGTGATTCGCAGCGAGACCAGCGAGTCGGAGCCTCCGGTCGCCGGCACGCCGATGAGCACGAGGTCGACCACGTACCACGACGAGGAGGACGCGCTGCTCGCCAGTGCCAGCATGCGACTTACGGCGCTCTGGACACGAGGTGTACGAGCAGGGCGCGTTTTCGGATGGCGTCTACGAGCTGCTGCACATCCCCGTGTTCTGTTTGATCGTGTTCGGCCTCACGACCGTGTACTTCGTCGTCACGGGCGTGCAGTACTGGAGCACCATCTTCATGATCAAGTCGCTGCATGCGTCCAAGTATCTCGTCAACGCGCTCTTCGTCGTTGTCTCCGGTACGGGGCCCATCCTGGGCGTCTTCTTTGGCGGTTGGCTCATCGACCGATACGGTGGGTACATCGGTGTGGAGCAGCGCGCGAAGGCCCTGGGCATCTGCATGACCCTGGGGCTCATAGCCTTTTCGATCTCAGCGGTGACGACGTTCTTCGATGACATCTACATCACTGCTGGTTTCCTGTGGCTCTTGCTCTTCTTCGGAGGCGCCATCCTGCCAGCGCACCGTGCGCTCGCCTCGTCATTCTCCGTAATGATCTTCAACCTGTTCGGCTACGCGCTCTCGCATGAGCGACCTGCAGATGGTGGTGGGCGCAAAGTGCTTCGTGCCGGACGAGACGCACGTGTGGCTAGCGGCCGAGGTACTGCGTGACGAGAATGCGGGCGACGGCAAGACGCGGAAGATCTACTGCAAGGTGGAGCTTCCCGACGGAGACTGAGGAGCGGTGTGTGGACATGCTGTCCAAGAAGACCAAGGCGCTGCTGACGTGCACCAGCTCGAGTCGCTACCCTACCAGAACGAGAACATGGGGCCTGAGGGCATCGAGGACATGATCATGCTCAACTACCTGCATGAGGCCGCCATCCTCTACAACGTCAAGAAGCGCTTCCTGTGTGAGCTGCCCTACACGTACACGGGTGACATTTGTATCGCCGTCAATCCCTACAAGTGGCTCCCGGACCTCTACGCCGAGGAGCAGCACCTGCGCTACCTCAACCAGCCCAAAGAGGAGCTCCCCCCGCACGTGTACGCTACGTCGGTCGCCGCCTACGACAACATGCGTCGCTCGGAGGTGAACCAGTCCATTCTGGTCAGTGGTGAGTCCGGTGCCGGTAAGACAGAGACCACCAAGATCCTCATGAACCACCTGGCCACCATTGCCGGTGGTCTCAACAACTCCACTATCAAGAGGATTATCGAGGTCAACCCGCTGCTTGAGTCCTTCGGTAACGCCAAGACCGTCCGTAACGACAATTCCAGTCGATTCGGCAAGTTCACACAGCTGCAGTTCGACACGAACGGCACGCTCGTCGGCGCCAAGTGTCGCACGTACCTACTAGAGAAGACGCGTGTCATCCAGCACGAAGCACCCGAGCGCAACTACCACATCTTCTACCAGTGGACTCCTCCGACATTGCACCGGAACTACTGCTCGACTCGGCAAAGCACTATGTGTACACGGGCGACAACACGGCACAGAAGATTGAAGGCCTCTCTGACGAGAAGCACTTCAACAAGACGCGCGATGCGCTAGAACTCGTGGGTCTGTCGCGCGATGACCAGCGACCACTCTTTGAAGTGCTAGCTGGTGTGCTTCACCTCGGTGAGGTGCAGCTGCAGTCAGACCCTGCAGATGACGAGAAGTCTCTCATCACCGAGGGCGACATCGGTGCGAATTCTGCGACGCAGATGCTTGGTGTCACGTGGGAGGCGCTTCAGACTGCATTGTGTTCGCGTACCATGCGCGCTGTGAACGATGTGTACAGCGTGCCGCTGAAGAAGGATCTGGCTATGGACTGTCGTGATGCACTCGCCAAGGCCATCTACTCCAACGTGTTTGACTGGCTTGTGACAACGATTAACCAGTCGCTGGCCGACGATGCCAACATGGCCAACCACGTTGGCGTGCTCGACATCTTCGGTTTCGAACACTTCAAGCACAACTCGTTCGAGCAGTTCTGCATTAACTACGCCAACGAGAAGCTGCAGCAGAAGTTTACGCAGGATGTGTTTAAGACCGTGCAAATCGAGTACGAGGAAGAAGGCATCGTGTGGGACCACATTGAGTACGCGGATAATCAGGACGTACTGACTGTGATTGAGTCGCGCATGGGTATAATTTCGCTGCTGAACGAAGAGGTGATGCGGCCGAAGGGTAGCGAGGAGTCCTTCATGTCCAAGGTGACGTCCCTGCACAAGGAC---GATATGGCGCACGTTATTGAGTTCCCTCGCACGTCGCGCACCGAGTTCCTCATCAAGCACTATGCCGCTCCTGTCATGTATGACTCGGTGGGCTTCTTGGAGAAGCACAAGGACTCGCTACTGCCAGACCTGTCCGAGCTCATGCGTGGTTCATCGAAGCCGTTCATCGCTAAGCTCTTCGAGCCGAAGCCGGAGCCTAAGCCGTCGAAAGC---------GTGGTGGTGCTCTGTCCATCTCGACTGTCGGTACCCAGTTCAAGGAGAGTCTGACAGAGCTGATGGCAACCATCACCTCGACCCGTGTGCACTATGTGCGTTGCATCAAGCCCAACCCGATCAA---------GAGCGCGACGGTTATGGACCAGTCCATGGTGGTATCTCAGTTGCGGTGTGCTGGTGTGATTGAGGCCATTCGTATCTCGCGTGCAGCGTACCCGAACCGGTTGCAGCACACGGAAATCTTGGACAAGTTCTGGCTGTTTGTGCCGAGTGGCGGCGAGACCGCAGCTGACAAGTGCCAGCTGCTCATGGATAAGCTGAAGCTGGAGTCCCCGACGCAGTACCAGATGGGAAAAACCCGCGTGTACTTCCAGCTAGGTGTGCTTGAAGAGCTCGAGGACCGCCGTAAAAAGTTTCTGGACGCCAAGGCTACGTACCTGCAGAACATCATGGTTGGGTTCACGCAGCGTATCAAGTACCTCCGACAACTGGAAGCGATCATCAAGTTCAGTGATTCGGTGCGTGATTGCCATGCGCAGATACAACACGTTCCTTAAGGGCCTCATTACGGCTCAAGCTCACTGGCGAGGCGTTCAGGGCCGCAGGGTAGCTGCCGACGTCAAGAGCAACCACAACGCTGTCATCATCCAGCGGTATGTACGTGGCTTTGTCAAGCGTCACCGTTACGTTAAGATGCGCGCGATGGTCATCCGTGTGCAGGCCGTGGTTCGTATGACGATCCAGCGGCCAAAATATCTGGCTGCTCTCGAGGAGAAGCGTCGTGAGGCGGACATGGCATACCAGCTGCAGAAGCTGAAGTCTGCCTTGCAGGAAGAGCAGGAGCGGAATGCCCAGTT------GCAGCGCC---GCAGCAGCGTGGC--GACGGCTCATCCGCTGCCTCGAGCGTGGTAATGGCTGACGCTGGCGGGATGATCGAGACACTCACGGACGAGAACAAGAAGCTGCGCGAAAAGAACGAGGATATGAAGGTGGCAATGAAGGGCTTGAAGGCGGAGATTGAGAAGTTCAAGAGCGACAAGGAGTTCTCGTCGGCTGGCAATCACGTCAAGGTTCGCCAGCTGCAGGACACTGTCCGTGACAAGGACAAGAAGATCTCGCAGCTCGAGGCCGATAACAAGAAGCTCACGGACCAGATAGCTAAGCTTCACGAGGACGGAGTTGT---TCCCGAGAAAAAGTCCACGCCGAAAAAATCGATCTTCCGCACGCTGGGTTCCAAGAAGGAGAAGAAGCCTC---------------GCGAGACCGTGCTGATGGACTCGCTCACCGGCTCGGACGATCTCAACCGCCGGGAGACGCGTCCGTCGGA---GCGCCACTTCCCGCGCATGC---CGAGCATGAGCGTGGGCTCA---CGCTTCTGGAACTCTAACAAG---AAGGGTGAA------------AACGGCGACGGCTTCGACGTCTCGGAGATTGATGACGAGGACAGCCCCACGAACCCACGCGTAACACGATCGTGAAGAGCATGGAAGTGGGCGTCACCGGTGCCATGTCGACCCTCAAGGGCCGCATG---------TCGGTCGTGAAGGGTATTTACGAAGCAAGCGCACGAACAGGACCGCCCTGTACGCGAATCGTTCAACCTGGATGCCTTGCCGGAGGTATCTCTGCCTGTCGGCTGGGAGGCCAAGGTGTCGCGCTCCACGGGTCGCGTGTACTACGTGAACCGCAAGCTCGGCAAGTCTCAATTCGAGCGCCCCACATTGGCCTCGCTCAA------GGCACAGAAGTTGGCGCGCCAGAAGTCGGCAAACGCGCTG-AAGGCTGTTGTCGCGGATTTCGGCTGCGGAGACGCCAGGCTGGCCGAGAGCGTGCCCAACAAGGTGCACTCGTTCGACTTGGTGTCTCTCGACATCGCCGTGTACTGCTTGGCACTGATGGGGACGAGCGTTCGCGAGTACGTGCGCGAAGTTCACCGTGTGCTCAAGCCTGGCGGCGTGCTGAAGGTGGCTGAGGTGAAGAGCCGCTTCGAGAGCGAGTCGCTGGGCGGATGGTGGCGGTGTTCGTGGCGGCGGCGCGGGGCGTCGTGGTGACGGTGCGCAACTCGACGGTGTTCGTGGGGAGACCAAGTACGCGGTCATCGTGGGCCAGAAGACGGGCGAGGCCGTGGCTGTGCCCTTCCCGGACGTGGACCGCGACCTCAAGACGCTGCTGGGCCACACGACGTCCATGGTCACCCATGTGGCAGTGAACCTCGTCGCTGCTGCTCACCGCGGACCGCGACGAGAAGCTGCGCGTGTCGAGGTTCCCGAACGCCGCCATCGTCGAGAGCTACTGCTTGGGCCACGCCGCTAGTCTGACCAAGGCGGCGTGCAGCGCTGTCACGCCGGACCTTGCGGTGTCTACGTCCATGGACAACACGCTGAAGCTGTGGCAGATGACGACGGGCAAGCTGGCGGTTTGCCCCAAGACGACGTTGTGGCTGTGCTCGTGAACTACCAGCACGTGCGATTCTTCGAGATCGCGAGAGCCCGTTCCTGCAGTTGCTTTCGATTTCAAAGGGCGGCCACCGTTTTCAACCGCCGTGCGCGACTTCCGCAAGCTTCAGAACGACCCGCCCTCGGGTGTGTCGGGCGCCCCCATGGACAACAACATCATGCTATGGCAGGCCGTCATCTTCGGACCGGACGACACGCCCTGGGAGGGAGGCACCTTCAACCTGACGCTCGAGTTCAGCGAAGACTACCCGAACAAAGCGCCGACCGTCAAGTTCATCACCAAAATGTTCCACCCCAACATCTATAATGACGGCCAGATCTGTCTGGACATCCTGCAGAACCAGTGGTCGCCGATCTATGACATCTCGGCCATTCTCACATCCATCCAGTCGCTGCTGTGCGACCCCAACCCGAACTCACCGGCCAACTCGGAGGCCGCGCGCCTCTTCCAGGAGAACCGACGGGAGTACAACCGCCGCGTGCGCGAGATCGTCGAGCAGAGCTGGCAGGCGATCGATGGTGTCGGCCGCCAAGATGCGCGGGGCTGAGAACCGCATGAACGCCGGCCGCCCCTTCACCGCGTGGCTGGACAACGTCTCGTCGGACCGCGGTCTGTGCGGAGGCATCAACTCGGGTATCGCCAAGACCACCCGCAGGCAGACCACTCGCAGATCTTCGTGATCGGCGACAAGGCCCGCGCCCAGCTGCGTCGCGACCTCGGCGACAATATCCGCGGCAACGTGACGGAGACGTTCCAGTCGCCCCCCAACTTCACGGTGGCCAGCGCCATCGCGGAGGCCGTCGAGTCGGAGAGGAGTCGTCGCGTATGGCCGCCATGGAGAACGCCACCACGAACGCCCAGGACCTCACGCTCACGCTCGTGTACAACAAGGCTCGCCAGGCCCGTATTACCACGGAACTGATTGAAATTATTTCGGGTGCCGCCTCCCTGGACGGTGACTGGCAGCGTGGTGAGCGTGCAGAGCGCGGCGCAGTTCGACGAGGCGACGGCGCGCGAGTCGACGCTCTCCGTGAGCTTCTTCTGGGCCGAGTTCCAGGCCTGCCGCCCCAACGGGCAGCTGGACGTGGGGCCGCGCCGTGCTGGACAAGCTCGAGGGCGCCAACGTGGCCGAGCTGGCCAAGCGCGTCTGCAGTACCGCCTCAAGAAGCTCACGAGCGCGGCGCCCGTCATGCTTTTCATGAAGGGCAACCCGACGGAGCCCAAGTGCGGCTTCAGCCGGCAGCTGGTGGCGCTGCTGAACGACGACAAGATCCAGTTCGGTACCTTCGACATCCTCAACGACGACGAGGTGCGCCAGGGGCTCAAGCAGTTCTCCGACTGGCCCACGTACCCGCAGCTGTACGTGAACGGCGCGCTCGTGGGCGGGCTGGACATCGTCAAGGAGATGAAGGCGGACGGGTCCATCGTCGAGCAGCTCGGCGTGGAGGAGGCCGAGGCTGCCTTCCAGGAGAGTCTGCGCGCGCTGGTGGGCTCGGCGCCCGTGCTGCTCTTCATGAAGGTTCGACATCTTGTATGGAGCTACCAACGCCAAGACCAAGACATCGCGAAGGTTGTGTCGCTTATCCCGTCGTCCTTCCGACGGGAAATGCGCCGAGGCTATCTCATGGAGCCCGAAACCGCATGGCCATGGCGTTCCGGGACCCTCTGCAGGATAAAGGAGAATTCTGTATTGTGCAGCTCCTCGCATTACCTCGAAGCGCACCACTCTGCATCCACGAGGAATTTCAGCAATCGACTGGCAAGTTTAATGTGCTGCACCGCGACCACCGCAGTGAGGTCAAGTCGCTATGTGTTCACTCGAAGCGCGACTGTTTGTACACGGGCGGTCTTGACGGCCTGCTGATCCGATATGACCTGAACAAGAGCCGCAGTGAAGTCGTGATTGAGCGTCGCAAGCACATCTCCAAGATCAACGCGGTACTGGAGCATCCGCACAACCCGAACCTGCTCTTGGTCAGCTCCTTGGAGCAGCAGACACAACCTGCTGCTGCACGATCTTCAGCGCTACGACCACAATGTGTCGGGCGGGTCGATGAGTCAGTACGTCGTGCCGCGGTGGTCGCCTGCAGGCTATCACGTCTCGTGCGGCAGCAAATGCGCTACTGCCACGGCAGCTTCTCCACGCCCACGTCGGCCACCATCGGGGCCTCGTTCCTGCAGAAGCGCGTGATTGTCGGGGGGAAGCTGACGCTGCAGATCTGGGACACGGCCGGCCAGGAGCGCTTCCGCTCCATGGCGCCCATGTACTACCGCAACGCTAAGGCGGCCATCCTGGTCTTCGACCTGCAGAGCGAGGCCAGCTTCGAGAAGATCAAAGAGTGGCTGCACCTGCAGCATCACGTGGGCGACGATATGTGGGCAACAAGAGTGATGTTTTGACTTTGCCAAGGCCCAACAGTTTGCGGACGAGATCGGTGCACTGGTGTTCCGTACGAGCGCCAAGACGGGCGAAGGAGTGCAATCGCTGTTCGAGAGTCTGGCGGTGCAGCTGCTCAAGAAACATGACCAGGAGCAGCGGAGAGCTTCTTCGTGGTGTACGTGCCGGCGGATGAGCGCAAGGAGCTGGAGGAGTGGGCGGTCAAGCTGCCGCAGGACAAGGAGGCGCAGCTCGGCTGCCTGACGGAGCGTCTGCGCGAGCACTTCAAGGGCGGCGCCATCCAAGGAGCAGCAGAGACGTTCAAACAGCAACTGCTGGCTCAGCTGCCCAAGGGCGCCACGCTCACGGACGAGACGCTGCAGATGATGCATGGACAGCCTCGTGGACTCGATCCCGCTCGTACTCAACACGCCCGACGTCAAGCACGTTGGAGGTCAAGGGCGACGCCTTCATCGGACGGGTCTTCGATAGACGACTTTGTGCGGATGGACTTCCGGTTGGGGGAAGTGTCGGGTGAAGCACCCTGGGTGCAGACGGCCAAGGTGCAGAGGTCACGCAGTCGCTGGAGGAGATCCTGGCGCTGGGCCAGGGCCTGGACGACCAGGACTACAAGGCCGAGCTCGAGGTGCTGCAGAACGACCCCAACTCCAACCTCTACTCGGCCGTCACCTTCGAGGAGCTCGACCTGCCGGAGTCGCTGCTCAAGGGCGTGTACAACATGAAGTTCGCCAAGCCGTCCAAGATTCAGTCGGTGGCGCTGCCCCTGATTCTTGCCAACCCTCCCGAGAACCTGATCGGCCAGGCGCAGAGCGGCACCCCACCCGCGAGCTGGTGCGACAGATTATGGCCGTGGTGAACGCCATGGGCAAGTTGTGGAGACGTTCCTGGCCATCCCCGGCAACGACCTGGGGCGTGGCGAGAGCCTCAAGGCTCAGGTCATTGTCGGCACCCCCGGCAAGGTCGAGGGCATGATCAAGAAGAAGCAGCTGGACACCCGCAACGTCAAGATCTTCGTGCTGGACGAGGCCGACGTGATGGTTGCTGAGGACGGCATGCGCGAGCGCTCGGTGGCGATCAAGCGTCTCATCAAGAACCACGCGTGCCAGTATCTGCTCTTCTCGGCCACGTACGCTGACGACGTGCGCGACTTCGCCCAGAAGATGGTGCCCGACCACAACATTATCACGGTCAAGAAGGAGAAGCTCACGCTGGACGGCATCAAGCAGTTCTGGATCGACTGCAAGACCCGCGACAACAAGTTCCAGGTGCTATCGGACATTTTTGCCATCCTGAGCATCGGCAAGTGTGTTATCTTCGTGCAGCAGCGCGACACCGCCAAGGAGCTGACGCGCCGTATGCGTGAGAAGGGCCACTCCGTCGGCATCTTGCACGGCGCCGACATGGCCAAGGAAGTGCGTGACCAGGTTATCGACGAGTTCCGTGCCGGCACGACCAACGTGCTGATCACCACAAACGTGCTTGCTCGCGGTATCGATGTGGCGGGTGTGTCACTTGTCGTGAACTTCGACATCCCGCTTACCCGCGACCACCGCCCGGACCCGGAGACGTACCTGCACCGTATCGGTCGTACGGGTCGTTTTGGCCGCAAGGGCTGCGCAATTAACTTCGTCTACGACAACAAGAGCAAGCAGGACCTCGCCGAGATTGAAGAGTACTACGCGCGTCCTATTACCCAGGCTCCCGCAGACGACATCGAGGAGCTCGAGAAACTGCTGGCATGGGGGGCAGCAAGCGCCGCCTGGCCAAGCTGGGCAAGGAGCAGCGCAAGGGCATGCGTATCTACCCGCGCGAGCCCAAGAAGAAGCCGTCGGGCAAGGACAAGACGTACTACTACCTCAAGGACGTGATGTTCCTGGCGCACGAGCCGGTGCTGGACAAGTTCCGCGCCTTCAAGACGTTCATGAAGAAGGTCAAGAAGGCCATGGGCAAAAAAGACTACGAGGACGCCAAGCGCAAGCACGAGAACCGCCCCACGTACGAGCTCGACCACATCGTCAAGGAGCGCTACCCGCGCTTCGTGGACGCGCTGGGGGACTTGGACGACGCGCTCTGCGGCAGAACTTCGTGGTCGCCACCCGCGCGCTCACCAAGGTGTTCGTGAGCATCAAGGGCATCTACTACCAGGCCAGCGTGCAGGGCCAGACGATCACGTGGCTGGTGCCCCACCAGTTCACTCCGACGGTGGACAAGCGCGTGGACGTGCGCGTCATGCTCACGTTCCTCGAGTTCTACGAGGTGCTGCTCAAGTTCGTCAACGCCCAGCTCTACATGCAGATCGGCGTGTCGTACCCGCCCAAGATCAACCTGCAGCTGGACGCCGCGGGGGCGCAGCTCGTCGCTCTGGAGCTAGCAGCAGAAGGAGTCGGAGAAGCGGATTAAGCTACAGCGAGCAGCGCAAGGAACGCAGCGAGAAGAGCCTCTTCAGTGGGCTGACCTTCTTCCTGTCGCGTGAAGTTCCTTCGGCTTGCCTGGAGCTGGTGCTGCGCTCTCACGGTGCCACGCTGGGCTGGGACGGAGCTGGGTCCCCTTTCGACGAGAAGAGCGCACGCATCACGCACCACGTCATGGATCGCCCGCACCAGGGCCACCGTTACTTCAACCGCGAGTACGTGCAGCCGCAGTGGGTGTTCGACTCGGTGAACAACGGCATCCTGCTGCCGCTGACGCGCTACCGCAAGGAGCTGGAGAAGCTCAAGTCGGCCTCGCAGGTGCTGCGCGAAGTGGGCGCTGTCGGCGACGAGGAAGAGGACGGGAGGCATACGCCGCCGACCTCAAGGCCGAGGTGGACGGCAACACGAAGTCGAAGCGTGTCAAGAAGGAGGAGGAGGCCGCGGAGCTCAAGGAGCTGGCCAAGACCATGATGTCGAAGAAGGCCAAGCGTCTGTACGACCGCATGCAGTACGGTCTGGGCAAGAAGGCCGACAAGATTCAGAAGCTCGAGGACAAGCGCGTCAAGATCGAAGCAAGACCAAGAAGCTGCTGGCGGGCGCGCTGTCGGGCATGACCTCGGCGCTGGCCTTCCAGCCGCTGGGGGGCTGCGCTGGCGGGGCACGTCGCCCACGCTCGTGCGCGTGGCGGGCGGCGCCGGACTCTACTTCTCCACGCTGGACCACTGCCTCAACATGTTCCCGCACTCGGCAGTCAATACGTTCCTGGCCGGAGCCTTCGCGCGCACTTTCGCGGGCGGCGTCATGTCGCCCTTCACGATTGTCAAGGCGCGGATGGATGCCGCCCGGCACCTTCGACAGCAGAGGGACGTGCCGTTCTCCGGCCTCTACGTGCTCATTTACACGGCATCCCGCCGATGTGGTCAAGACGCGCATGCATGCGACAGACCGTGGACAAGATGTACCGCCTCGAGGGCCTGCGGGGCTTCGCGAAGGGCATCATTCCGCGAGTGGTCAAGCGGACGCTGTCCACTGCAGTCACGTGGACGATCTACGAGCTACTCGTTCGACCCGTCGGGCCTAGGCCACGCCAAGGAGGCCTTCAATCTTGCCAAGGAGACGGAGCGCACCAAGCAGACGGAGAACCAGGCCAAGATCAAGGAGAACGAGGCGCTCTACAAGCAGTACGAGATCGTGCGCGTGCAGAAGGAGGGCGAGGAGCGCCGCAAGACGCTGGAGGAGGAGACGCGCCAGCACCAGCAGCGCGCGCAGTATCAGGACCAGCTTAAGCGGAAGCAGTACGCTGACCAGCTGGCCGCGCAAAAGTACATGAAGGAGCAGGAGCTCAAGAAGCAGGAGGAGATTCTCGCCCGTCAGGAAGCCTCGCGCCGCAAGACGCTCGACTACGAGGCCGAGCTGCGTATGAAGACCGAGATGGCCAAGGTTTCGGCTGAGACCGAGGGCCGCATCAAGCAGGAGCGTCTCAACCACGACCTGCACCTCGAGGAGGCCCGTGTGCGCGCCAAGGAGTACCGCGAGACCGTCATGGATGGTATTAAGCTGGCCGGTAACACCGTCGGATCCGGCATCATGGCCTTTGTCGACGACAAGGAGAAGCTCACGGCTACCGTGGTCTCTCTGTCTGCTCTGGCCGTTGGTATCTACACTGCCAAGGTCTCCACCAGTGTTGCAGGAAAGTACATCGAAGCGCGCATGGGCAAGCCTTCCCTGGTGCGCGAGACCTCCCGTCGCTCGGCTGCGCAGGTGGTGGCCAACCCCGTCCCGTCCATCAAGCGCGCGCTGCGTCTGCAGAAGGCGACAGATGCCCTAGAGGGCGTCGTGCTTGAACCCAAGCTGGATGAGCGTCTGCGCAGTGTGGCCGTGTCCACGTTCAACACGAAGAAGAACCGCGCGCCGTTTCGCCACTTGCTGCTGCACGGCCCGCCCGGTACGGGTAAGACGTTGTTCGCCAAGGCTCTGGCCCGTCACTCCGGTCTGGAGTATGCCATCCTCACGGGTGGTGACGTTGCCCCGCTGGGCCGCGAGGGTGTGACGGAGATCCACAAGTTGTTCGACTGGGCGTCGCACAGCCGTCGCGGTCTACTGTTGTTCGTCGACGAGGCTGATGCGTTCCTGCAGAAGCGTAGCAACACCGTCATGAGCGAGGACATGCGCAACGCATTGAATGCTTTCCTGTACCGCACGGGTGAGGCCAGCGATAAGTTCATGATCGTGTTCGCCAGTAACCAGCCGGAGCAGTTCGACTGGGCCATCAATGATCGTATCGATGAGATGGTGGAGTTCCGCCTGCCGGGCTACGATGAGCGTGTGCGCATGCTGAAGCAGTACTTCGACGACTACATCCGTGCACCGAAGAATTCGCGCGCCAAGAAGATTTACGCGAAGGCGTCATCTTCACGCTGGACCTCGTCGCCCACTTCCTCCTCCCAAGACGCGCATCTTCCAGCTTGAAGCTCTGCAGGGCGTGTCCGTCGAAGCTCAGTCCGTCTTTGATGCACTCAACAAGAGCCCATGCGCCTCTACTGGCTCGTCTTCGCCGCGGTGCTGGGCAACGGGCTGTACACGTACTGGGCCGGCAAGGACGAGTCGTTCCTGGTGGAGAAGCTCAAGAGAAAGATCCCGTCCGAGTACCGCATCTCGGAGAAGTGGGACAAGTGCCTGGAGAACTTCACGCTATACTTCGGCGCCGGCCTCGTGGCTGGCGGCCTCACGTCCCTCGTGCTGGCGCGTTCTGGCGCTGGCCGCGGCCTGGTGACGGGTCTGGGTGCGCCGGTGCCGGCTCCAGCTGGACCACGTGCCAGATGGCGTTCGCAGGCGACGCACAGGCAGCTCTGAACAAGACGGACAAGGCGGTGGGCGACTTGCCGGCCTTCCAGCAGCAGACGATCGTGGACTTCGTGACGGCCTCGCCCACGCAGCAGCAGCTGCAGGCCGTGCACCCGCGGCTTCTGCGAGGAGGCCAACGTGAAGGTCCTGGAGACGTACCTGGCTGAGCAGGTGCAGAGCAAGACCGTGGACATCGACGCCAACGTGGCGCTGCTCAAGCTGTACCAGGTGTATCCGGCCACGGTGAATGCCGAGAGCCACTATTTTGGTCAAGGGCATCATGACGCTGCCCTCCACCTTCTTCACCGGCGTCCACGATGGTCCCGGAGAGCATCCGCGAGGACGCCAATGTGACGGCGGCGCTGCACACGGGCTTCATGCTGCAGTCGTGCCTGTTCGAGGACTTCTGGAAGGAGGACGCGCCCTTCGCCGAGAAGGTGCCGGGCTTCCTCGAGTCAGTGCGCGCCTACATCGTGACGGCCATCAGCCGCAGCCACAGCGCCATCTCCATGGACGTCGTCAAGGCCAAGCTCAACGTGTCTGACAAGGAGGTGGCCGACATTGTCGCCGAGAAGTGGACGCGGACACCCTGATCCAGATCACCCCGAACGAGAACAACCAGATGCAGGCCAAGAAGGTCCAGGAGAACATCGAGTTCGAGGACGTGCTCAAGGTCATCCACACCCTTTCTAGATCCAACGTCTGCGCGAGCGGCGGCGGCGTGGCCGACGCCGAGGAGGTCTGCGACAACCTGGCCAAGCACCTGCGACTGGAGGCGGGCTCCCAGGTGCTGCCCTGCTCCACGGGCGTCATCGGCTGGCGCATCCCCGTGGACGCCATGGTCGAGAACCTCCCCAAGCTCGTCAACAACCTGCAGTCCGACTCGGTGCTGCCGGCGGCCGAGGGCATCATGACAACGGACCTGTACCCGAAGATCCGCTCCATGGACGTGTGCGGCGGCCGCATCGTAGGGATTGCCAAGGGTGCGGGCATGATTGAGCCCAACATGGCCACGATGCTGTCGTACATTCTGACTGACCTGTCGATCCCGCGTGACGTGCTGCGTCAGTTGCTGACGGAGGTGGTGGGAGACACGTACAACTCGCTCAGCATTGACACGGACCAGAGCACGTCCGACACGGTGACGCTCGTTTCGTCGGACCAGATCCCGTTCGACATGAA---GCAGCTGGACGAGTTCAAGAAGGCGCTGCACGACGTGTGCCTCGGACTGAGCGAGGACATTGTGCGCAACGGCGAGGGTGCGCACCACGTCATGCGCGTGCGTGTGAGCGGTGCCTTGTCCAAGGAGATGGCCAAGGGCGTCGGGAAGTCGGTGGTGAACTCGCCACTCCTCAAGTGCGCGGTGGCAGGCAACGACCCGAACGTGGGCCGATTGGTCATGGCTGTGGGCAAGTACATGGGCCTGCACCACAAGGACGTGAACATCCAGCGCCGCATGAAGATGCGTATGGGCGGCGTCGTTATCTTCGAGAACGGCGAGATGGTGCTTAACGACGATATCGAGGCCAAGCTTGTGGCGCACATGCGCGACGCGATGCTGATTGAGCCCACCCGCGGTGGCCTGGATGCGAGCTCGCACAACTACCCGCCGCACGGAAAGTTTGTCGAGATGGAGATCGACCTGGGTGTCGGATCCGAGGAGACTCAAGTTCTGGGCATCGACCTTACGCACGAGTACGTAGCCATTAACGCCGACTACCGCTCT--ATGGCGCC---GAAGAAGGGGAAGACCGCCGCCGCGTACCTGGCGTCTCTGGGCGTGTCCCCCGC---CGCTGGAGGCGAGAAGAAGGACGAC---AAGAAGAAGAAAAAGAAGGGCAAGAAGAAGCCCAGAAGAAGCCCATGTCGGCGGCGGCCAAGGCCCTGA-TGGAGCGCCAGGCGGCCATGAGGGCGGAGCAGAAGCGCGTCGAGCAGGAGCGTCTGGAGCAGGAGCGCATCGCCCAGGAGGAGGAAGCCAAGCGCCTGGCCGACGTCGAGCGCGAGGAGGAGGCCCGCCGCAAGAAAAAGGAGGCGCGCGCCGCCAAGATCGAGCGCCAGAAGAAGGAGGGCACGTACATGACCAAGGCCCAGAAGCAGAAGGCGGCCAAGGCCCAGGCCGCCCTCGAGGCCATGCAGCAGGCGGGTCTCGTGCCCGAGGCTCCC------GCCGAGAAGAAGAAGGTCGTGTACTCGAACAACAAGAAGAAGAACAAAACGAA-AGGAGGAAGAGGAGGAGGAGGCCGAGGCCCCCGACGCGTGGGACGACGAGGACTGGGAGACCAGCGACGTTGTGGCCTCGCTCGAGGACAAGCTAGCAGAAGAAGGAGCAGGAGCGTCTGCGTCAGCAGGGCATTGCGCTGAAGAAGCGCCAGGAAGAGCAAGCCGAGCAGATGCGCCTAGAGGCCGAGGAGGAGCGTCAGCTCTCGAAGGCCAAGATGGAGGCCGATCGTAAAAAGAAAGAGGCAGCTGAGCGTCGTCGCGAGCGTGAGGCTGCCGCGCGTGCCAACGTGTCCCTGGACAACCTGCGTTCACCCATTTGCTGTATCATGGGCCACGTCGATACGGGTAAGACGAAGCTCCTTGACAACATCCGCCGAACCAAGGTGCAGGACGGCGAAGCTGGTGGTATTACCCAGCAAATTGGCGCCACCTTCTTCCCTGTGGAGGCTATTAAGCAGAAGACAGCACGCCTCGCCGAGACCATGAAGCTGGAGTACCGCCTGCCCGGTCTTCTTGTGATCGATACGCCCGGTCACGAGTCGTTCACGAACCTGCGTTCTCGTGGTTCGTCGCTGTGTGATATCGCCATTCTGGTCGTTGATATCATGCACGGTTTGGAGCCGCAGACTCTGGAGTCGCTGCGCCTGCTGCGTCAGAAAAAGGCCCCGTTTGTGGTGGCGCTTAACAAGATCGATCGGTGCTACGGCTGGAAAGCGATGCCCGACATGCCCGTCCAGGAGGCTCTGAAGCACCAGAACGAACACGTCATTCGCGAGTTCGAGGACCGCATGAAGGCGATCATTGTGGAGTTTGCTGAGCAGAAGCTGAACGCCGAAGTGTACTGGCGCAACACGGACTTGGCGCGCACGGTCTCGCTCATCCCTACCAGTGCTATCTCGGGCGAGGGTGTCCAGGACCTGCTGATGATGCTGACTCGATTGACGCAGGAGCGCATGGCCAAATCGCTGGCGTTTGTTGACATCCTGCAATGTACCGTGCTGGAAGTGAAGGTCATTGAGGGTCTTGGTACGACGATTGATACGATTCTGGTGAACGGTACTCTGGAAGAAGGTTCGACTATCGTAGTGTGTACATTGGACGGCCCAGTTGTGACAACGATCCGTTCGCTGCTGACTCCGCACCCCATGAAGGAAATCCGTGTCAAGGGCGAGTACATCCACCACCACAAAATGAAGGCCGCTATGGGCGTGAAGATTTGCGCGCAGGGCCTTGAGAAGGCCGTCGCCGGTACGCAGATTTACGTGGTGGGCCCCGATGACGACGTTGAGGAGCTGAAGGACACCGTCATGTCCGACTTGACGGGCATTCTGGACTCTGTGAAGGCTACTCGTCGCGGAGTTATGGTTCAGGCTTCCACGCTGGGTGCCCTGGAGGCGCTTCTGGAGTTCTTGCGCACGTGTGACCCGCCCATTCCGGTGTCGTGCGTCAACATCGGCCCCGTGCACAAGAAGGACGTCATGCGTGCGTCCGTGCAGCTGGAGCACCAGCCCGAGTTCGCCACCATTTTGGCGTTCGATGTGAAGGTCCACAGCGACGCTACTGAGCTGTCTGCGGAGCTCGGCGTACGCATTTTCACGGCCGACATCATTTACCACCTGTTCGACCAATTCACGGCGTACATGGACAACTTCCGAATGGCCCGCCGTGAGGAGTTCGCCGAGGTGGCCGTGTTCCCGTGCGTGCTGAAGATCCTGCCCGAATGCGTCTTCAACAAGAAGGACCCGATCATTCTCGGCGTCGACATCGAGGAAGGTATCCTCAAGATTGGCACGCCACTCGTCGTGCCGTCTGCCGGCG---GCTTCCTGGTCGGCAAGGTGGGCTCCATCGAGCGCGAGCACAAGGAGGTGGACCGCGCCAAGAAGGGCGCGTCCGTGGCCGTGCGGATAGACAACGAGGGCAGCGTCATGTACGGCCGCCACTTCGACCACAAGAACAAACTGGTGTCGCGCCTGACCCGCCAGAGCATCGACGCGCTGAAGGAGAATTTCCGGGAAGACCTGCAGAAGGAGGACTGGCAGCTCGTCATCAAGCTCAAGAAGGTGTTCGATATCATTATGGAGGGCTGGCTCACGAAGCGCCGCGACCACATGAACCTCATCTGGCTGGATCGCTACTTCCTGCTGGACGGCAACCAGCTCCGCTACTACCGCAAGCGCGGCGACCCGGCGCCTCGAGGGACCTACATCCTGACCGACGGCTGCGTGGTGAGCCCCGTGTTCAACTCGGAGGAGAAACACAAGCACCACGGCCCCGTCTGGATGTTCCGCATCACGTGAGAAGTCGTACCGGAAGGACCGATTCCTGGACCTCGGGGCCAAGAGCGAGGAGAGCGCCAAGGACTGGAAGCGCGCGCTGGAGAACGCCGTCCGCTCGCTGAGGGAGCTGGTGGCCCACCAGCGGAAGCTCAAGACGGACGCCGAGAT---CTTCCCCACGCCCACGCGC---------GTCCCGAGGATGCTGAAGGCCGACGAGGACACCGTGTCGCTGTTCAACCTGGACGCGGACAACGTCAAGGCGGAGCAGGCCTGGTGGCTGGCGAGGGTGGAAGGAGGGCTCCGGATCATGCAGGAGTGCCCGCTGGGGCCGAGCGTTGCCGCCAACCTGCACGCGCAGCACGCGGCCACGACGAACGGACTGATCTTCAGCGCGTTTGTGGGCGCCGCGCTGAGTTTTAGCAGCATGTACTCGTTCGCGAGCAACATGCTGCTGAGCTTGATGACGGCGCTCGTGGCGATCGCCGTGTGCTGCATTGCGGTCACGCCGACGCAAGCGCCCGAGATCCCGAGCTTCCGGGTCTCTCAGGTGGTGCATGGCTCGCCGACCGAGGTCTTCCGGCTCATCATGAACTCCAAGCGGTTCCAGCGATGGGACTCTGGGACGGCGACCATGCGTGTGGTGCAGCAGCTGGATGACCACGCCGACATTGTCTACGTGACGCAGCGCCCGACGCACTTGTGGCCGCTGTGGCAGAAGGCTCGAGACCTTGTGCTCATGCGCTACTGGCGACGCGAAGAGGACGGATCGTACTTTGTCATGTACCAGAGCATCGAGCACCCGGAGTGCCGTGTGCGCCACAACTACGTGCGCGCAAACCTGCTGGGCGGCGGCTTCGTCATCGCCCCCCAGCGCGTCCCGAGT---GGATCTATCCGTACACTGGTCTCGTACGTACTTCGGTTTGATCCTGGAGGCTG---GTCGCGCATCTACCACCAGCTTGGCATGGATGTGGATCTGGTGATGCCCATGCTGCGCAGCGTCGTGGGTATCCGCGATGAAATGAGCGCTACCGACTTCATCACGCCCAATGTCTCGATGGCTCCTGTCGGGGAGGCCGGAGGCTGGCCAGGAAGCGTGGA---CTCCATGGGCGTCGTCATGGGCGTGCAGAAGCTCCAG---ACAAGTCTACCGGAGAAGATGTGGGCCGAGCCTGACGCCTCTCTCTTCAGCGTGCGTGGACACAATTACTTGAACGACAAGAAGAAGATCCATTCGGCGCCGGCGATGTTCCACACCGTGGGCGTGGATCTCTTGTCGTTCGAGAGCGCAGCCGAGAGATACAACATCTCGTCTCGTCCGGACAGTGTCGGTCGCACCAG---CTCCAAGTTCACGTTTGTTGTGAACATGATCATCCCGGGCCCGGAAAACGTCTGCATGGTATTTTACTTCCACCCCGTGCGTGACAACGTGTTTGAGGACGGCTCGCCTTTCTCCGAGCTGCTGAACGACTTCTTCGATGGCGACGACCAGTTCCGGAATTCGCGCTTCAAGCTCATCCCCACTGTTGTCGAGGGCAGCTTCATTATCAAGCAGTCGGTCGGCTCCAAGCCGACGTTGCTGGGCAACAAGCTCAAGTGTCCGTACCACCGTGGGGAAAACTACTTTGAAGTAGACATCGACATTTCGTCCAACTCAGTGGCCAACACCGTCGTGGGCATGGTACAAGGCGTGACCAAGTCGCTAGTGGTGGACATGGCGTTCCTCCTCGAGGCTCAGTCCGACGAAGAGCTGCCTGAAATCATCCTGGGCGCTGTTCGCATGCAGCACATTTCGCTTGACCACCCGCGTCGCATGCCGAAGCATGGGGAACGGGCAGCAAGGACGAGTTCACCTTCTGAGGCCTTCACCGAGGCTCTGCGCGTGAATCTGCAGCGCCTGATCGTCTACGCGCGCTCGGCGGACACGGCGCTGCAACGAGAAGTGGCCGAGAAGCTGGCCAACGAGGCCGTCAAGACCGACAGGTGCAGATCGTGGAGCTGGACGGGCTGCAGCTGCTGCTGCCGCTCACCAAGTCCAAGGACACCGAGGTGCAGCGTCTGGCGGCGCACGCACTCGCCAACCTCTCGGTCAACTCTGAGAACCAGTCCAAGATGGCCACGGAAGGGGGCATCGACATGCTCATTGGCCTCTTGGGCAGCACGAACGAGCACGTGCAGCGGCAGGCCGCGAAGGCGCTCGCCAACCTCGGCGTGAACGTGGATAACAAGGAGCGGATCGCTAAGGCGGGAGGCATTAAACCTCTCATCGACTTGGCCAGCTCCCGCCAGATCGGAGTCGCCGTAGAGGCCATCGCGGCGCTTGCAAACCTGGCTGTCAACG---ATGCCAATGAGGTGGAGATCGCACGCAAAGGCGGCTTGAAGCCCATTATTGACGGATCGCACTCCGAGTCCGTGGAGCTTCAGTCCCAAGTGGCTCGCGCACTACGCAACCTCTCAGTGAATCCGGAGAACAAGCAAGCGATTGTGGAGCTGGGCGGGGTGGAGGCTTTGCAGTCACTTGTGCGTTCCACCAACGACCGGATATGCCAACAGGCTACACGTGCGCTCGTCCCCAACGTGGTGCTCACGCGCTCCTCGCCCACAACCTGCTG---GCGGTGGCGCCCGTGGCCCGCGTGGCCGAGGTCAAGGAGGAGGC------CGTGCCTTATGACGGCGCCATGGTGGACGTCGAGTACTCGACCACAGTCAAGACGGTC---------GCGGTGCCTGCAGCAACGCCCA---ACGACGCGGCCGGG---GGCTTCTCGTCCATCTCCGACCGCTTCCCGCTCTCCAAGGTGGGCAAAGTCAGCTGCGGTGTGGCCCCCATCCCCATGAGCCG---CTTGCTCAACG---CCACGCCTGAGATGACAC---AGATGGACCAG-------------ATGACTGTGCTCGTAGAGGACGCCACGGTCGATGACAAC---ACCATCAAGGCCGAG---CCGGAGGGTGAGCGACGTGTACCGGTACCAGGCTACCGA--CGTGGAAGAGAACGAGATGGAGCAACTACGATGGAACATCCAAGAGCAGGTCTTTCAGCGATACTTCCAGCAAGGACAGAAGTACGAGGAGTGGGGACGACTGGAGGAGCAAGACACCGAGTATTCTTCATTCCGGGTGGACGGCGGGAAAACATCATGGCAGAGAAGCGATTCCACGACAACGCTGTCCGAGACGCTATGGGAAAGGGAAATGCCACATGGGTCAAGGAGCGACGGGCACGGTTCGAGGAGGAAATCCGTCAATTCTATGACGAAACCAGGCACAAGCAGGAGCTTATGCTTACCCGCCTTGAAAAGGTGTCTCCGTTCTCCAAGTTCCCGATTCTTGGCAAC---------------------CGGGGAAAGGGAGGTAACGGGGAAGTATGGGATGTCGTTGACTACGCCAGCAACAAGCAGCTCTGCGCTCTCAAGCTCTCCACTTCGATCCGACACGCCCAACGCGAGCACCACACGCACACTCAGCCACCCCA--ACATCGTCCAGGTTGGCGAGGTGCCATTCCTGATCCGATATCAGCAACAGCAGTACACTGCATTCACCGTTGCAAGCGTTCAGTCGGATCTTCAGCAACTAATTGAGA---TGTACGAGTACTTAGATGACGATTCTGCGTACAAGGTGCTTTCTCAACTTCTGAGCGCGCTGAAGTACTTGCACGATGGGCGTTGCTCACTACGACCTGAAGCCGTCGAACATTCTGATAGACCACGATGGGTGTGTGAAGCTG------ACCGACTTCGACTTGACTCGCGATGCCAAGGCTGCTACGTCCAACTCTACGGTTGGCACTCTGCGATACCTTCCTCCTGAATGCTTCCGCCCGAACCGTAGTGA---------CTGTGAAGCCACGGCTGAGAAGGCGGACATGTGGATGGTAGGCATCGTGTACTGTATGATGGTTACGGGAAAACACCCGATCTGCCCGGACAAGTCGACGCAAGCAGAAGTGAAAACGATGATGGCTCAGTACACGGGT---GAACTTCGATATGCGCGGCCCATTTCCGACCTGTCTCGGTGGATCTTACAAGGGTGCCTCCACCCGGACCCGCGGTGTCGACCAACGGCCGCACAATTGCTGGTGGCGCTCCAG

Phytophthora_ramorum TGGACTACCACCGCCAGGTGGCGCGCGAGCTCTGCGCCTTCTTCCACTCGTTCTACCGCTTCGGGGCGGCCTCCAACCGCTTTGACCTGTTCCTGCACAACGTTTTAATGGGCTCCTTGTGTATCGGAGCCGTCGTGTTCCTCGTGCTGTGTATCATCGTCGTGCGGCGCTGCATTCTGCACATCCGCAGGCGTTTGTACGTCGAACGGCCATGGATTCCACGTCCGTGGAGCTGCTGGCAGTGGGTCTACTGACGTTCCTGGCGCTGAGTGCCCTGGGGGGACTGCTGGGAGAAGCCATTTGCAGGTCAGCGACGCTCTACGCGTCAATGCAGACAACTTGATCACGTCCTTCAACAACAGCGAGCTGCCTGCTGAAGCCTTTAAGTTGTCGGTCGAGTCGCTGGACTCGTGCACGCGGTCAAGGACCTGTGGAACCAGACGGACGCGCTGCTGCCCAAGAACTACTCGGATCTGGCCAAGGAGTGGCAGTTCAGCTACTTCGTGCTCAAGTCGTCGACCAACGGCGCGATCCTGGCGGTGGCGCTCGCCAGCTTCTTGTCCATCGCGTCGGTCGGGTGGGCCATGGTCTCTCCCCTGCGCGTCTCCATCCTGGTCATCCTGAGCGTCGTGCCCATTTCTCACACGCTTATCGGCGCCTACCTGTCCTCGACGATCATGACGGCGGACTTTTGTGCTGCGCCGATGAACAACACGCTGGAACTCGTGGGTTCGTGTCCCGTGAACGCGTCCTACCTTTCGCTGCTGCCACTGCCAGACTACGCTGCCCACCACGGAGACGTGGGTCTGCGCATGAAGGAGGAATTTCTGGACCCCATCGGAGCAACTGTGGGCGTCATGCTCGGCTACTTTGCAGGCACCGTGAGCAACTGGGAGAGCTTCTCGCTCATTCAGACGTGGCGCTGGCCCTTCCTGCTGCAGTTCGTAGCGCTGCTGCCAATTAATGTAGCCATCTTCTTCGTGCCCAAGAAACACCTCGTGATCCGCAGCGAGACCAGCGAGCTCGAGCCGCCAGTCGCCGGCACGCCCATGAGCTCGAGGTCCACGACGTATCACGACGAAGAGGACGCACTGCTGGCCAGTGCGAGCATGCCACCTACGGCGCCTTGGACACGAGGTGTACGAGCAGGGCGCTTTCTCGGACGGCGTGTACGAACTGCTGCACATCCCCGTGTTCTGTTTGATTGTGTTCGGCCTCACGACGGTGTACTTCGTCGTCACGGGCGTGCAATACTGGAGCACGATTTTCATGATCAAGTCGCTGCATGCGTCCAAGTATCTTGTCAACGCGCTCTTCGTTGTCGTTTCCGGCACAGGACCGATCCTGGGCGTGTTCTTCGGTGGCTGGCTCATCGACAGATGCGGAGGGTATATCGGCGTGGAGCAGCGGGCCAAGGCTCTGGGTATCTGCATGATCCTGGGCCTCACAGCCTTTGCGATTTCCTCAGTGACGACGTTCTTCGACGACGTCTATATCACTGCTGGTTTCCTCTGGCTGTTGCTCTTTTTCGGAGGCGCCATCCTGCCGGCGCACCGGGCGCTTGCCTCGTCGTTCTCCGTGATGATCTTCAACCTGTTCGGCTACTCACTCTCGCATGAGCGACCTGCAGATGGTGGTGGGAGCCAAGTGCTTCGTGCCAGACGAGACGCACGTATGGCTTGCAGCCGAGGTGCTCCGCGACGAGAACTCGGGCGACGGCAAGATCCGCAAGATCACGTGCAAGGTGGAGCTGCCCGACGGAAACCGAGGAGCGTTGCGTGGACATGCTGTCCAAGAAGACCAAGGCGCTGCTGCCGTGCAACAGCTCGAGTCGCTGCCCTACCAGAACGAGAACGTCGGGCCCGAGGGTATTGAGGACATGATCACGCTCAACTACCTGCACGAGGCCGCCATCCTGTACAATGTCAAGAAGCGTTTCCTCTGCGAGCTGCCGTACACCTACACGGGCGACATTTGCATCGCCGTGAACCCGTACAAGTGGCTGCCAGACCTCTACGCCGAGGAGCAGCACCTGAGATACCTCAACCAGCCCAAGGAGGAGCTTCCGCCGCACGTGTACGCCACGTCGGTGGCGGCCTACGACAACATGCGCCGCTCCGAGATCAACCAGTCCATTCTGGTGAGTGGTGAGTCCGGTGCTGGTAAAACGGAGACCACCAAGATCCTCATGAACCACCTGGCTACCATTGCTGGTGGTATGAACAACTCGACCATCAAGAGGATTATCGAGGTGAACCCGCTGCTGGAGTCCTTCGGTAACGCGAAGACTGTGCGTAACGACAACTCCAGTCGATTCGGCAAGTTCACACAGCTGCAGTTCGACCAGAACGGCACGCTTGTTGGTGCCAAGTGTCGCACGTACCTGCTGGAGAAGACTCGTGTCATTCAGCACGAAGCGCCCGAGCGCAACTACCACATCTTCTACCAATGGATAGCTCCGACATTGCGCCGGAACTGCTCCTCGACTCGGCAAAGCACTACGTGTACACTGGCGACAACACAGCACAGAAGATCGAAGGACTCTCGGATGAGAAGCACTTCAAGAAGACGCGTGAGGCGCTGGAGCTTGTGGGTCTGTCTTACGATGATCAGCGGCCGCTATTCGAGGTGCTTGCCGGTGTGCTCCACCTTGGTGAGGCGCAACTGCAGTCCGACCCATCGGATGACGAGAAGTCTCTCATTATCGAAGGCGACAGTGGTGCCAATGCTGCCACTCAGATGCTTGGTGTCACCTGGGAAGCGCTTCAGACTGCGCTGTGTTCGCGTACCATGCGTGCCGTGAATGATGTGTACAGCGTGCCGCTGCGCAAGGAACTGGCTATGGACTGCCGTGATGCACTTTCCAAGGCTATCTACTCCAACGTGTTCGACTGGCTGGTGACTACCATCAACCAGTCGCTTGCCGATGATGCAAACATGGCAAACCACGTGGGTGTGCTCGATATCTTCGGTTTCGAGCACTTCAAGCACAACTCGTTCGAGCAGTTCTGCATCAACTACGCCAATGAGAAGCTTCAACAGAAGTTCACACAGGATGTGTTCAAGACCGTGCAAATTGAGTACGAGGAAGAAGGTATCGTGTGGGACCACATTGAATATGCGGATAACCAGGATGTGCTGAGCGTGATTGAGTCGCGCATGGGTATCATCTCGCTGCTTAACGAAGAGGTGATGCGACCTAAGGGTAACGAGGAGTCTTTCATGTCGAAGGTGACGTCGCTGCACAAGGAT---GACATGGCGCACGTTATTGAGTTCCCTCGTACATCGCGCACCGAGTTCCTTATCAAGCACTATGCTGCTCCTGTCATGTACGACTCGGTCGGTTTCCTGGAGAAGCACAAGGACTCGCTGCTGCCTGACTTGTCTGAGCTCATGCGTGGTTCGTCGAAGCCGTTCATCGCAAAGCTCTTCGAGCCGAAGCCGGAGCCGAAGCCGCCGCAAGC---------GTGGTGGTGCGCTGTCGATTACGACGGTCGGTACCCAATTCAAAGAAAGCTTGACGGAGCTAATGGCTACCATCAACTCGACCCGTGTGCACTATGTGCGTTGCATTAAGCCCAACCCGATCAA---------GAGCGCGACAGTCATGGACCAGTCCATGGTGGTATCCCAGCTGCGATGCGCTGGTGTGATCGAGGCTATTCGTATTTCGCGCGCAGCGTACCCAAATCGGTTGCAGCACACGGAGATTTTGGGCAAATTCTGGTTGTTTGTGCCGAGCGGAGGCGAGACGCCGGCAGACAAGTGCCAGCTGCTCATGGACAAGCTGAAGTTGGAGTCGCCCACGCAGTACCAGATGGGTAAGACCCGTGTGTACTTCCAGCTGGGTGTGCTTGAAGAGCTCGAGGACCGCCGTAAGAAATTCCTCGATGCCAAGGCCACATATCTGCAGAACGTCATGGTTGGCTTCACTCAGCGTATCAAGTACCTACGGCAACTGGAAGCGATTATCAAGTTCGGTGATCCGGTGCGTTATCGCCATGCGGAGATACAACACGTTCTTGAACGGCCTTACTTTGGCGCAAGCTCACTGGCGAGGCATTCAGGGCCGCAAGTTTGCTGCCGATGTGAAGAGCAACCACAACGCTGTTATCATCCAGCGGTATGTGCGTGGCTTTGTCAAGCGCCACCGCTACGTTAAGATGCGCGCGATGGTTATTCGCGTGCAGGCTGTGGTTCGTATGACGATCCAGCGGCCGAAGTATCGGGCTGCTCTTGAAGAGAAGCGTCGTGAGGCTGACATGGCTTACCAGCTGAATAAGCTGAAGGCAGCCTTGCAAGATGAGCAGGAGCGTAATGCGCAACT------ACAACGCC---GCAGCAGCGTGGC--------GCGTCCGCTGCATCCAGTGTGGTCATGGCTGACGCTGGTGGCATGATCGAGACTCTCACGGACGAGAACAAGAAGCTGCGTGAGAAGAACGAGGACATGAAGGTTACGATGAAGGGCTTGAAGGCGGAAATTGAGAAGTTCAAGAGCGACAAGGAGTTCTCTTCGGCTGGCAACCACGTCAAGGTTCGTCAGCTGCAGGACACTGTGCGAGATAAGGACAAGAAGATCAAGCAGCTTGAGGCCGATAACAAGAAGCTGACAGAGCAGCTTGCTAAGCTGCACGTGGAAGG---CGT---GCCCGAGAAGAAGTCCACGCAGAAGAAGTCGATCTTCCGTACGCTGGGCTCCAAGAAGGAGAAGAAACCGC---------------GCGAGACCGTGCTGATGGACTCGCTCACCGGCTCGGATGACCTCAACCGTGGGG------GCGCGTCGGA---GCGCCACTTCCCGCGCATGC---CGAGCATGAGCGTGGCCTCT---CGCCTTTGGAACTCTAACAAA---AAGGGCGAC------------AACGGCGACGGCTTCGACGTCTCGGAGGTCGACGGTGAGGACAGCCCCACGAACCCACGCGAAACACGATCGTGAAGAGCATGGAGGTGGGCGTGACGGGTGCCATGTCAACCCTGAAGGGCCGCATG---------TCCGTCGTGAAGGGCATGTACGAAACAAGCGCACGAACAGGATCGCCCTGTGCGCGAGTCGTTTAACTTGGATGCTCTGCCGGAGGTATCGCTCCCTGTCGGCTGGGAAGCCAAGGTGTCGCGCTCTACGGGCCGCGTGTACTACGTGAACCGCAAGCTCGGCAAGTCCCAGTTCGAGCGCCCCACGCTGGCCTCGCTCAA------GGCGCAGAAGCTGGCACGCCAGAAGTCGGCCAACGCGCTGTAAGGCGGTTGTGGCGGATTTCGGCTGTGGAGATGCCAGGTTGGCCGAGAGTGTGCCGAACACAGTGCACTCGTTTGATTTGGTGTCGCTTGATATCGGCGTGTACTGCTTGGCGCTGATGGGGACGAGCATTCGCGAGTACGTGCGTGAGGTGTATCGCGTGCTGAAGCCTGGAGGTGTGTTGAAAGTTGCTGAGGTGAAAAGCCGCTTTGAAAGTGAGTCGCTTGGCGGATGGTGGGAGTGTTTGTGGCGGCGGCGCGGGGCGTCGTGGCGACGGCGCGCAACTCGACGGTGTTCGTGGAGAGACCAAGTACGCGGTGGTGGTGGGCCAGAAGACGGGCGAGGCCGTGGCTGTGCCCTTCCCTGACGTGGACCGCGACCTCAAGACGCTGCTGGGCCACACGACGTCCATGGTCACCCACGTGGCTGTGAACCTCGTCGTTGCTGCTCACGGCGGATCGCGACGAGAAACTGCGGGTGTCACGGTTCCCGAACGCCTCGATCATTGAGAGCTACTGTCTGGGCCACGCTGCCAGTCTCACCAAGGTGGCGTGCAGTGCGGTGACGCCGCAGCTTGTGGTGTCGACGTCCATGGACAACACGCTCAAGCTGTGGGAAATGACGACTGGCAAGCTGGCGATTTGCCCCAAGACGACGTTGTGGCGGTGCTCGTGAACTACCAGCACGCGCGGTTCTTCGAGATTGTGAGAGCCCGTTCCTGCAGATGTATTCGTTCTCGAAGGGCGACTGCCGCGTTTAACTGCTGTGCGCGACTTCCGCAAGCTTCAAAATGACCCGCCGTCGGGCGTGTCGGGCGCCCCCATGGACAACAACATCATGCTCTGGCAGGCCGTCATCTTCGGACCGGACGATACGCCCTGGGAGGGAGGCACCTTCAACCTCACGCTCGAGTTCAGCGAAGATTACCCCAACAAGGCGCCGACCGTCAAGTTCATCACGAAAATGTTCCACCCCAACATCTACAACGACGGCCAGATCTGTCTGGACATCCTGCAGAACCAGTGGTCGCCGATCTACGACATCTCGGCCATCCTCACGTCCATCCAGTCGTTGCTGTGCGACCCCAATCCGAACTCGCCCGCCAACTCGGAGGCCGCGCGCCTCTTCCAGGAGAACCGACGGGAGTACAACCGTCGCGTGCGCGAAATCGTCGAGCAGAGCTGGCAGGCGATCGATGGTGTCGGCCGCCAAGATGCGCGGCGCTGAGAACCGCATGAACGCCGGTCGCCCCTTCACCGCGTGGCTGGACAACGTCTCGTCGGACCGCGGCCTGTGCGGCGGCATCAACTCGGGCATTGCCAAGACCACGCGCAAGCAGACCACTCGCAGGTCTTCGTGATCGGCGACAAGGCCCGCGCCCAGCTGCGTCGCGACCTCGGCGACAACATCCGCGGCAACGTCACGGAGACGTTCGCGTCGCCCCCCAACTTCACGGTGGCCAGCGCCATCGCCGAGGCTGTCGAGTCGGAGAGGAGTCGTCGCGCATGGCGGCCATGGAGAACGCCACCACCAACGCCGAGGACCTCACGCTCACGCTCGTGTACAACAAGGCTCGCCAGGCCCGCATTACCACGGAGCTGATTGAAATTATTTCGGGTGCTGCCTCCCTGGACGGTGACTGGCAGCGTGGTGACCGTGCAGAGCGCGGCACAGTTCGATGAGGCAACGGCGCGCGAGTCGACGCTGTCCGTGAGCTTCTTCTGGGCCGAGTTCCAGGCCTGCCGCCCCAACGGCCAGCTGGACGTGGGGACGCGCCGTGCTGGACAAGCTCGAGGGCGCCAACGTGGCCGAACTGGCCAAGCGTGTCTGCAGTACCGTCTGCAGAAGCTCATTAGCGCCTCGCCCGTCATGCTCTTCATGAAGGGCAACCCGTCGGAGCCCAAGTGCGGCTTCAGTCGGCAGATGCTGGCGCTGCTGAACGACGACAAGATCCAGTTCGGCACCTTTGACATTCTCAACGACGACGAGGTGCGCCAGGGACTCAAGCAGTTCTCCAACTGGCCCACGTTCCCGCAGCTCTACGTGAACGGCGCGCTGGTCGGCGGCCTGGACATTATCAAGGAGATGAAGGCGGACGGCTCGATCGTCGAGCAGTTGGGCGTGGAGGAGGCCGAGGCTGCGTTCCAGGACAGTCTGCGCGCTCTCGTCAACTCGGCGCCCGTGCTGCTCTTCATGAAGGTTCGACATCTTGTGTGGAGCTACCAACGTCGAGACCAGGAAATTGCCAAGGTGGTGACTCTGAACCCTTCATCTTTCCGCAAGGACATGTGCCGAGGCCATTTCGTGGAATCCGAAACCGTATGGCTATGGCTTTCCGCGATCCCCTGCACGAGAAGGGAGAGTTCTGTATTGTGCAGCTGCCCGGATTACCTCGAAAAGTACTACTCTGCATTCGCGAGGAATTTCTGCAATCGACTGGCAAGTGGAATGTGCTGCACCGCGATCACCGCAGTGAAGTCAGGTCGCTCTGTGTCCACTCCAAGCGTGATTGTTTGTACACGGGTGGCCTCGACGGACTGTTGATCCGATATGACCTGAACAAGGGGCGTACTCAAACTGTGATCGAGCGTCGCAAGCGTATTTCCAAGATCAATGCGGTGCTGGAGCACCCACACAACCCGAACCTGCTTCTGGTCAGTTGCATAGAGCAATAAGTATAACTTGTTGCTGCACGATCTTCAGCGCAACGATGGCAACGTGTCTAGTGGTGCCACGAGCCAGTACATCGTGCCGAGGTGGTCGCCTGCAGGCTACCACGTTTCATGTGGCAGCAAATGCGCTACTGCCACGGCAGCTTCTCCACGCCCACGTCGGCGACCATCGGAGCCTCGTTCCTGCAGAAGCGCGTGATCGTCGGAGGGAAACTGACGCTGCAGATCTGGGACACGGCCGGGCAGGAACGCTTCCGCTCCATGGCGCCCATGTACTATCGCAATGCGAAGGCGGCCATCCTGGTCTTCGACCTGCAGAGCGAGGCCAGCTTCGATAAGATCAAGGAGTGGCTGCACCTCCAGCACCACGTGGGCGACGATATGTGGGCAATAAAAGCGACGTTTCGACTTCGCCAAGGCGCAGCAGTTCGCGGATGAGATCGGCGCGCTGGTGTTTCGTACGAGTGCCAAGTCGGGTGAAGGAGTGCAGGCGCTATTCGAGAGTCTGGCAGTGCAGCTGCTCAAGAAGCACGACCAAGAGCAGCGGAGAGCTTCTACGTGGTGTACATCCCGTCGAATGAGCGCAAGGAGCTGGAGGAGTGGGCCGTGGACCTGCCTGCCGACAAGGAGGCTCAGCTGGGCTGTCTGACGGAGCGTCTGCGCGCGCACTTCAAGGCCGGGGCCAAGCAAACTGCAGCAGAGACCTTCAAGCAGCAACTGCTGGACCAACTACCCAAGGGAGCCACCATGACGGACGAGATGCTGCAGATGATGCATGGACAGTCTCGTGGACTCGATCCCACTCGTGCTCAACACACCCGGCGTCAAGCACGTTGGAGGTCAAGGGCGACGCTTTTATCGGTCGGGTATTCGACAGATGACTTCGTGCGTATGGATTTTCGGTTGGGCGAGGTATCGGGCGACGCGCAGTGGGTGCAGACGGCCAAGCTGCAGAGGTCACGCAGTCACTGGAGGAGATCCTGGCGCTGGGCCAGGGCCTGGACGACCAGGACTACAAGGCCGAGCTCGAGGTGCTGCAGAACGACCCCAACTCGAACCTGTACTCGGCCGTCACCTTTGAGGAGCTCGATCTGCCAGATGCGCTGCTTAAGGGCGTGTACAACATGAAGTTCGCCAAGCCGTCCAAGATTCAGTCGGTGGCGCTGCCGCTGATCCTCGCGGACCCGCCTGAGAACTTGATCGGACAGGCTCAGAGTGGTACCCCACCCGTGAGCTGGTGCGCCAGATTATGGCCGTGGTGAACGCCATGGGCAAGTTGTTGAGACGTTCCTGGCCATCCCCGGCAACGACCTGGCGCGTGGCGAGAGCCTCAAGGCGCAGGTGATTGTGGGCACCCCCGGCAAGGTGGAGGGCATGATCAAGAAGAAGCAGTTGGACACCCGGAACGTCAAAATCTTCGTGCTGGACGAGGCCGACGTCATGGTGGCGGAGGACGGCATGCGCGAGCGCTCGGTGTCGATCAAGCGCCTGATTAAGAACCGTGCGTGCCAGTACCTGCTGTTCTCAGCCACGTACGCCGACGATGTGCGCGACTTTGCCCAGAAGATGGTCCCGGACCACAACATTATCACGGTCAAGAAGGAGAAGCTTACTCTGGACGGTATCAAGCAGTTCTGGATTGACTGCAAGACTCGCGACAACAAGTTCCAGGTGCTGTCGGACATTTTCGCCATCCTGACCATCGGCAAGTGCGTGATCTTCGTGCAGCAGCGTGACACTGCCAAGGAGCTGACGCGCAGTATGCGCGAGAAGGGTCACTCGGTCGGTATTCTTCACGGCGCTGACATGGCGAAGGAGGTGCGCGACCAGGTTATCGACGAGTTCCGTGCCGGCACGACCAACGTGCTCATCACCACAAATGTGCTTGCTCGTGGTATCGATGTTGCTGGTGTGTCGCTTGTCGTGAACTTCGACATCCCGCTCACGCGCGACCGCCGCCCGGACCCGGAGACGTACCTGCACCGTATCGGTCGTACGGGTCGATTCGGCCGCAAGGGCTGCGCTATTAACTTCGTCTACGACAACAAGAGCAAGCAGGACCTGGCCGAGATTGAGGAGTACTATGCGCGTCCTATCACGCAGGCCCCGGCAGACGACATTGAAGAGCTCGAGAAGCTGCTGGCCTGGGGGGCAGCAAGCGCCGCCTGGCCAAGTTCGGCAAGGAGCAGCGCAAGGGCATGCGTATCTACCCGCGCGAGCCCAAGAAGAAGCCCTCGGGCAAGGACAAGACGTACTACTACCTCAAGGACGTCATGTTCCTGGCGCACGAGCCTGTGCTCGAGAAGTTCCGCGCCTTCAAGACCTTCATGAAGAAGGTCAAGAAGGCCATGGGCAAGAAGGACTACGAGGACGCCAAGCGTAAACACGAGAACCGCCCCACCTACGAGCTCGACCACATTGTCAAGGAGCGCTACCCGCGCTTCGTGGACGCGTTGGGCGACCTGGACGACGCCTTGTGTGGCAGAACTATGTGGTGGCCACGCGCGCGCTCACCAAGGTCTTTGTGAGCATCAAGGGCATCTACTACCAGGCCAATGTGCAGGGCCAGACCATCACGTGGCTCGTGCCGCACCAGTTCACGCCCACGGTGGACAAGCGCGTGGACGTGCGCGTTATGCTCACGTTCCTCGAGTTCTACGACGTGCTGCTCAAGTTCGTCAACGCGCAGCTTTACATGCAGATCGGTGTGGCGTACCCGCCCAAGATCAACTTGCAGCTGGACGCTGCCGGAGCCCAGCTCGCGGCGCTGGAGCTAGCAGCAGAAGGAGTCGGAGAAGCGCATCAAGCTACAGCGAGCAGCGCAAGGAGCGCAGCGAGAAGAGCCTGTTCAGTGGTCTGACCTTCTTCCTGTCCCGTGAGGTGCCTTCGGCCTGTCTGGAGCTGGTGCTGCGCTCGCACGGCGCCACTCTGGGCTGGGACGGTGCCGGCTCGCCCTTCGACGAGAAGAGCTTGCGTATCACGCACCACATCATGGATCGCCCGCACCAGGGCCACCGTTACTTCAACCGCGAGTACGTGCAGCCGCAGTGGGTGTTCGACTCCGTGAACAATGGCGTGCTGCTGCCGCTGACGCGGTACCGCAAGGAGCTGGAGAAGCTCAAGTCGGCTTCGCAGGTGCTGCGCGAGGTTGGCGCTGTGGGCGACGAAGACGAGGACGGGAGGCCTATGCCGCCGACCTTAAGGCTGAGTTGGATAGCAACAAGAAGTCAAAGAAGGCCAAGAAGGAGGAGGAGGCCGCAGAGCTCAAGGAGCTGGCCAAGACCATGATGTCGAAGAAGTCCAAGCGTCTGTATGACCGTATGCAGTACGGCCTGGGCAAGAAGGCCGACAAGATTCAGAAGCTTGAGGACAAGCGTGTCAAGCTGGAAGCAAGACAAAGAAGCTGCTGGCGGGCGCGCTTTCGGGAATGATCTCGGCGCTGGCCTTCCAGCCGCTGGGGGTCTGCGCTGGCGCGGCACGTCCCCGACGCTCGTGCGCGTGGCTGGCGGTGCCGGTCTCTACTTCGCCACGCTTGACCACTGCCTGCACCTGTTTCCGCACTCGGCAGTCAACACGTTCCTGGCGGGAGCTTTCGCGCGCACGTTTGCAGGGGGCGTTATGTCGCCCTTCACAATTGTCAAGGCCAGGATGGATGCCGCCCGGCACCTTCGACAGCAAAGGGACGTACCGTTCTCCGGTCTCTATGTGCTGATCTACACGACACCCGGCGGATGTAGTCAAGACGCGGATGCATGCGGCAGACAGTGGCCAAGATCCACTGCCACGAGGGTCTGCGCGGCTTCGCCAAGGGGATCGTTCCGCGTGTGGTCAAGCGTACACTATCCACTGCAGTGACATGGACGATCTACGAGCTACTCGTTCGACCCGTCGGGCCTGGGCCACGCCAAGGAGGCCTTCAACCTGGCCAAAGAGACGGAGCGCACCAAGCAGACGGAGAACCAGGCCAAAATCAAGGAGAATGAGGCGCTCTACAAGCAGTACGAGATCGTGCGCGTGCAGAAGGAGGGCGAGGAGCGCCGTAAGACTCTCGAGGAGGAGACCCGCCAGCACCAGCAGCGCGCGCAGTATCAGGACCAGCTTAAACGCAAGCAGTACGCCGACCAGCTGGCCGCCCAGAAGTACATGAAAGAGCAGGAGCTCAAGAAGCAGGAGGAGATCCTTGCCAAGCAGGAGGCCTCGCGCCGCAAGACGCTCGACTACGAGGCCGAGCTGCGTCAGAGGACGGAGATGGCCAAGGTGTCGGCCGAGACCGAGGGTCGCATCAAGCAGGAGCGTCTCAACCACGACCTGCACCTCGAGGAGGCCCGTGTGCGCGCCAAGGAGTACCGCGAGACGGTCATGGAAGGTATTAAGCTAGCGGGTAACACAGTGGGCTCCGGTATCATGGCCTTCGTCGACGACAAGGAGAAGCTCACGGCCACCGTCGCCTCTCTGACTGCGCTCGCCGTGGGTATTTACACTGCCAAGGTCTCCACCAGCGTTGCAGGCAAGTACATTGAGGCACGGATGGGCAAGCCGTCGCTGGTGCGCGAGACGTCGCGTCGCTCGGTCACACAGGTGGTGGCTAACCCCATTCCGTCCATTAAGCGTGCGCTGCGTCTGCAGAAGGCGACGGACGCCCTGGAGGGTGTCGTGCTCGAGCCCAAGCTGGACGAGCGCCTGCGCAGCGTAGCTGTCTCCACGTTCAACACGAAGAAGAACCGCGCGCCGTTCCGCCACTTGCTGCTGCACGGCCCGCCCGGTACGGGTAAGACGCTGTTCGCCAAGGCTCTGGCGCGTCACTCCGGTCTGGAGTACGCTATCCTGACGGGTGGTGATGTTGCCCCGCTGGGTCGTGAGGGTGTGACGGAGATCCACAAGCTGTTCGACTGGGCGTCGCACAGTCGTCGCGGTCTGCTGCTGTTCGTTGATGAAGCCGATGCATTCCTCCAGAAGCGTAGCAACACTGTTATGAGCGAGGACATGCGTAACGCGCTGAACGCCTTCCTGTACCGCACCGGTGAGGCCAGCGACAAGTTCATGATTGTGTTCGCGAGTAACCAGCCGGAGCAGTTCGACTGGGCTATTAACGATCGTATCGATGAGATGGTGGAGTTCCGTCTACCGGGCTTCGACGAGCGTGTGCGCATGCTGAAGCAGTACTTCGACGACTACATCCGTGCGCCGAAGAACTCGCGTGCCAAGAAAATCTACGCGAGGGCGAAATCTTCACGGTGGACACCGTCGCCAACTTCCTCGTGCCAAGACACGCATCTTCCAGCTTGAGGCTCTGCAGGGAGTGACCGGAGAAGCGCAGACCATCTTCGATGCGCTCAACAAGACCCCATGCGTATCTACTGGGTCGTCTTCGGTCTCGTGCTGGCTAACGGACTGTACACGTACGTGGTGGGCAAGGACGAGTCATTCCTGGTGGAGAAGCTCAAGAGAAAGATCCCGTCCGAGTACCGCATCTCCGAGAAGTGGGACAAGTGTCTCGAGAACTTCACGCTGTATTTCGGTGCCGGCCTTGTGGCTGGCGGCCTCACGTCCCTCGTCCTGGCGCGTTCTGGTGCGGGCCGTGGACTAGTGACGGGTTTGGGCGTGTGGGCGCCGGCTCGAGCTGGACGACGTGCCAGCTGGCGTTCGCGGGCGACGCACAGGCAGCTCTGGACAAGACGGACAAGGCGGTGGGCGACTTGCCGGCCTTCAAGCAGCAGACGATCGTGGACTTCGTGACGGCCTCAGCCACGCAGCAGCAGCTGCAGGCGGTGCACCCGAGGCTTCTGTGAGCAAGCCAGCGTGAAGGTTCTGGAGACGTACCTGGCCGAGCAGGTGCAGAACTCAACCGTGGACATTGATGCCGACCTGGCGCTGCTCAAGCTGTACCTGGTATACCCGGCCACGGTCAACGCTGAGAGCCAAGGTTCTCGTCAAGGGTGTAATGGCACTGCCGTCCACGTTCTTCACCGGCGTCCACGTTGGTGCCCGAGAGCATCCGCGAGGACGCCAGTGTGACGGCCGTGCTGCACACGGGCTTCCTGCTGCAGTCGTGCCTATTCGAGGACTTCTGGAAGGAGGAAGTGACGTTCGCCAAGAAGGTGCCTGGCTTCCTGGAGTCGGTGCGCGCGTACATCCTGACGGCCATCAGCCGCAGCCACAGCGCCATCTCCACGGAGGTCTTTAAGGCCAAGCTTAATGTGTCTGACAAGGAGGTGGCCGACATTGTCGCCGAGAAGTGGACGCGGATAACCTGGTCCAAATCAGCCCCAACGAGGACAACCAGATGCAGGCCAAGAAGGTCCAGGAGAACATTGAGTTCGAAGACGTCCTCAAGGTCATCCACACCCTTTCTAGATCCAACGTGTGCGCCAGTGGCGGCGGCGTTGCAGACGCCGAAGAGGTCTGCGAGAGCCTGGCCAAGCACCTGCGGCTGGAGGGCGGCGCTCAGGTGCTGCCCTGCTCCACGGGCGTCATCGGATGGCGCATTCCCGTGGAAGCCATGGTGGAGAACCTCCCCAAGCTCGTCAACAGCCTGCAGTCCGACTCGGTGCTGCCTGCTGCCGAGGGAATCATGACGACAGACTTGTATCCGAAGATCCGCTCCATGGACGTGTGCGGCGGACGCATCGTCGGCATTGCCAAGGGTGCTGGCATGATTGAGCCCAACATGGCTACGATGCTGTCCTACATCCTCACCGATCTGTCGATCCCGCGTGACGTGCTGCGTCAGCTGCTGACGGAGGTGGTTGGCGACACGTACAACTCTCTCAGCATTGACACGGACCAGAGCACGTCGGACACGGTGACTCTCGTGTCCTCGGACCAAATCCCCTTCGACATGAG---CCAGCTAGATGAGTTCAAGAAGGCGCTGCACGAGGTGTGTCTGGGACTCAGCGAGGATATTGTGCGAAACGGCGAGGGTGCACACCACGTGATGCGGGTGCGCGTGCGTGGCGCTCTGTCCAAGGAGATGGCGAAGGGTGTCGGCAAGTCGGTCGTAAATTCGCCTCTCCTGAAGTGTGCCGTCGCTGGCAACGACCCGAACGTGGGCCGTTTGGTCATGGCGGTGGGGAAGTACATGGGCTTGCACCACAAGGACGTGGATATCACACGTCGCATGAAGATTCACATGGGTGGCGTTATTATCTTCGAGAACGGTGAGATGGTGCTCAACGACGAGATTGAGGCCAAGCTTGTGGAGCACATGCGCGATGCGATGCTGATCGAGCCCACTCGTGGCGGCCTCGATGCCAGCTCGCACAACTTCCCGCCACACGGAAAGTTTGTCGAGATGGAGATTGACCTTGGCGTTGGTTCAGAGGAGACTCAAGTGCTGGGCATTGACCTTACGCACGAGTACGTGGCCATCAATGCCGACTACCGCTCGTAATGGCGCC---GAAGAAGGGGAAGACCGCTGCCGCGTACCTGGCGTCGCTGGGCGTGGCCCCCACCAGCGCCGGCGGTGAGAAGAAAGACGAC---AAGAAGAAGAAAAAAAAGGGTAAGAAGAAACCTAAAAGAAGCCCATGTCGGCCGCGGCCAAGGCGCTGA-TGGAGCGGCAGGCAGCGCTCAAGGCCGAGCAGGAGCGTCTGGACCAGGAGCGTCTGGAGCAGGAACGCATCGCTCAGGAGGAGGAGGTCAAGCGCCTCGCGGAGATCGAGCGCGAGGAGGAGGCTCGTCGCAAGAAGAAGGAGGCCCGTGCCGCCAAGATCGAGCGCCAGAAGAAGGAAGGCACTTACATGACCAAGGCCCAGAAGCAGAAGGCCGCCAAGGCGCAGGCGGCTCTGGAAGCCATGCAGCAGGCGGGACTCGTGCCGGACGCCGCT------GGCGAGAAGAAGAAGGTCGTGTATTCGGACAACAAGAAGAAGAGCAAGAAGAACAGGAGGAAGAGGAGGAGGAGGCTGAAGCCCCCGACGCCTGGGATGACGAGGACTGGGAGACCAGCGACGTTGTGGCCTCGCTGGAGGACAAGCTAGCAGAAGAAGGAGCAGGAGCGGCTGCGTCAGCAGGGTATTGCGCTGATGAAGCGTCAGGAGGAGCAAGCTGAGCAAATGCGTCTTGAAGCTGAGGAGGAGCGTCAGCTCTCCAAGGCCAAGATGGAGGCTGATCGCAAGAAGAAGGAGGCGGCCGAGCGTCGTCGTAAGCGTGAGGAGGCTGCGCGTGCCAACGTGTCGCTGGACAACCTCCGTTCCCCTATTTGTTGCATCATGGGCCACGTCGATACTGGTAAGACCAAGTTGCTTGACAACATCCGACGGACCAAGGTGCAGGACGCCGAAGCCGGTGGTATTACGCAGCAGATTGGTGCGACGTTTTTCCCCGTGGAGGCTATCAAGCAGAAGACGGCGCGCCTCGCCGAGACCATGAAGCTGGAGTACCGCCTGCCCGGTCTGCTTGTGATTGATACGCCTGGTCACGAGTCGTTCACGAATTTGCGTACTCGTGGTTCGTCGCTGTGTGATATTGCTATCCTGGTGGTTGACATCATGCACGGTCTGGAGCCACAGACTCTGGAGTCGTTGCGCCTGCTGCGCCAGAAGAAGGCCCCGTTCGTGGTGGCACTGAACAAGATCGATCGTTGCTACGGATGGAAGGCAATGCCCGACATGCCAATTCAGGAGGCGCTGAAGCACCAGAATGAGCACGTCATTCGCGAATTTGAGGACCGCATGAAGTCGATCATCGTTGAGTTTGCGGAGCAGAAGCTGAACGCCGAGGTGTACTGGCGTAACAAGGACCTCGCGCGCACGGTGTCGCTCATTCCTACTAGCGCTATCTCGGGTGAGGGTGTTCCGGATATGCTGATGATGTTGACGCGTCTGACGCAGGAGCGCATGGCGAAGTCGTTGGCATTTGTGGATATTCTGCAGTGCACTGTGCTGGAAGTGAAGGTAATTGAGGGTCTTGGCACGACGATTGATACGATTTTGGTGAATGGTTCGCTGGAGGAGGGCGCGACGATTGTCGTGTGTACTTTGGACGGACCCGTTGTGACGACCATCCGTTCGTTGCTGACCCCGCACCCTATGAAGGAGATCCGTGTGAAGGGCGAGTACATCCACCACCAGAAGATGAAGGCCGCCATGGGTGTGAAGATTTGCGCGCAGGGCCTGGAGAAGGCGGTGGCTGGCACGCAGATTTACGTGGTTGGTCCCGATGACGATGTCGAGGAGCTGAAGGACTCTGTCATGTCCGATTTGACAGGCATTTTGGATTCCGTGAAGGCTACCCGCCGCGGTGTGATGGTGCAGGCGTCCACCCTGGGTGCTCTGGAGGCGCTTTTGGAGTTCTTGCGCACGTGTGACCCGCCCATTCCGGTGTCGTGCGTGAACATCGGACCTGTGCACAAGAAGGACGTGATGCGTGCTTCCGTGCAGCTGGAGCACCAGCCCGAGTTCGGCACCATTCTGGCATTCGACGTGAAGGTCCACAGCGACGCTACCGAGCTGTCTGCGGAACTTGGCGTGCGCATTTTCACGGCCGATATCATTTACCACCTGTTCGATCAATTCACGGCGTACATGGACAACTTCCGAATGGCGCGCCGTGAGGAGTTCGCGGAGGTCGCTGTGTTTCCGTGCGTACTGAAGATCCTGCCCAATTGCGTCTTCAACAAGAAGGATCCGATCATTCTCGGTGTCGACATCGAGGAAGGCATTCTGAAGATCGGCACGCCCCTCGTTGTGCCCTCCGCCGGCG---GCTTCCTGGTCGGCAAGGTGGGCTCCATCGAGCGCGAGCACAAGGAGGTGGATCGCGCCAAGAAGGGCGCGTCCGTGGCTGTTCGGATAGACAACGAGGGCAGCGTCATGTACGGCCGCCACTTCGACCACAAGGACAAGCTGGTCTCGCGTCTGACGCGCCAGAGCATCGACGCGCTCAAGGAGAATTTCCGGGAAGATCTGCAGAAGGAGGACTGGCAGCTCGTCATCAAGCTCAAGAAGGTGTTCGATATCATTATGGAGGGCTGGCTCACCAAGCGCCGCGACCACATGAACCTCATCTGGCTGGACCGGTACTTCTTGTTGGACGGGAACCAGCTCCGCTACTACCGCAAACGCGGCGACCCGGCTCCCAGAGGCACGTACATCCTCACGGACGGCTGCGTCGTGAGCCCCGTCTTCAACTCGGAGGAGAAGCACAAGCACCACGGCCCCGTCTGGATGTTTCGCATCACGTGAAAAGTCCTACAGGAAGGACCGGTTCCTGGACCTGGGCGCCAAGAGCGAGGAGAGCGCCAAGGAATGGAAACGCGCGCTGGAGAACGCCGTCCGGTCGCTCAGGGAGCTCGTGGCCCACCAGCGCAAGCTCAAGACGGACGCCGAGAT---TTTCCCCACGCCCACGCAC---------GTCCCGAGGATGTTGAAGGCGGACGAAGACACTGTGTCCCTGTTTAATTTGGACGCGGACAACGTCAAGGCCGAGCAGGCGTGGTGGCTCGCCAGGGTGGAAGGCGGACTCCGGATCATGCAGGAGTGTCCGCTGGGGCCCAATGTGGCCGCCAACTTGTACGCACAGCATGCGGCCACGACGAACGGACTCATTTTCAGTGCGCTCGTGGGTGCCGCGCTGAGCTTTAGCAGCATGTACTCGTTCGCGAACAACATGCTGCTGAGCTTCATGACGGCGCTCGCGGCCATTGCGGTGTGCTGCGCGGCGGTGACTCCCACGCAGGCGCCCGAGATCCCGAGCTTCCGAGTTTCGCAAGTGGTGCATGGATCCCCGACGGAGGTTTTCAGGCTCATCATGAACCCCAAGAGATTCCAGCGGTGGGACTCGGCCACGGCCACGATGCGCGTGGTGCAGCAGCTGGATGACCACGCCGACATCGTCTACGTGACGCAGCGCCCGACGCACCTGTGGCCGCTGTGGCAAAAAGCTCGTGATCTCGTGCTCATGCGCTACTGGCGGCGTGAAGAAGACGGGTCGTACTTCGTCATGTACCAGAGTATCGAGCACCCAGAGTGTCGTGTGCGCCACAACTACGTTCGCGCCAATCTTCTGGGCGGTGGCTATGTCATCGCCCCCCAGCGCGTCCCAAAT---GGATCCATTCGCACGCTGGTCACGTACGTGCTCCGATACGACCCTGCAGGCTG---GTCGCGCATTTACAACCAGCTCGGCATGGATGTCGACTTGATCATGCCCATGCTACGCAGCGTGGTGGGCATTCGCGACGAAATGAGTGCTACGGACTTCATCACACCTAACGTCTCGATGGCGCCTGCTGGGGAGGGCGGATGCTGGTCAGGAAGCGTGGA---CTCCATGGGTGTGGTCATGGGTGTGCAGAAGCTCCAG---ACAAGTCTTCCGGAGAAGATGTGGGCTGAGCCTGATGCTTCACTCTTTAGCGTGCGCGGACACAACTACCTGACCGACCGCAAGAAGATCTCCTCGGGGCCAGCGATGTTCCATACTGTGGGTGTGGACTTGTTGTCGTTCGAAAGCGCGGCTGAGAGGTACAACATTTCGTCTCGCCCGGACAGCATCGGCCGCACCAG---CAGCAAGTTTACGTTCGTGGTGAACATGATCATTCCGGGCCCAGAGAACACGTGCATGGTGTTCTACTTCCACCCGGTGCGGGACAACGTGTTCGAGGACGGATCCGCCTTCTCGGAGCTGCTGAACGACTTCTTCGACGGCGACGACCAGTTCCGGAACTCGCGCTTCAAGCTCATTCCCACCGTCGTCGAGGGCAGCTTCATTATCAAGCAATCGGTCGGCTCCAAGCCGACGTTGCTCGGCAACAAGCTCAAGTGTCCGTACCACCGTGGAGATAACTACTTTGAAGTGGACATCGACATCTCGTCCAACTCGGTTGCGAACACTGTCGTGGGCATGGTGCAAGGCGTGACCAAGTCTCTCGTGGTGGACATGGCTTTCCTGCTTGAGGCCCAATCAGATGAAGAGCTGCCTGAAATCATCCTCGGTGCTGTGCGCATGCAGCACGTTTCACTGGACAGCCCACGCCGCATGCCGAAACATGGGGAACGGGCAGCAAGGACGAGTTCACGTTCTGAGGCCTTCACCGAGGCGCTGCGCGTGAATCTGCAGCGACTGATCGTGTACGCGCGCTCGGCGGACACGGCGCTGCAGCGAGAAGTCGCCGAGAAGTTGGCCAACGAGGCCGTGAAAATCGACAGGTGCAAATTGTGGAGCTGGACGGGCTGGAGCTGCTGCTGCCGCTCACCAAGTCCAAGGATACGGAGGTGCAGCGTCTGGCGGCGCACGCACTCGCCAACCTCTCGGTCAACTCTGAGAACCAGTCCAAGATGGCGACGGAAGGAGGCATCGACATGCTCATTGACCTCCTGGGCAGCACGAACGAACACGTGCAGCGGCAGGCTGCCAAGGCACTCGCCAATCTCGGCGTGAACGTGGACAATAAGGAGCGGATTGCCAAGGCAGGCGGCATCAAGCCGCTCATTGACTTGGCCAACTCCCGCCAGATTGGTGTCGCTGTAGAAGCCATCGCAGCGCTTGCAAATTTGGCCGTCAATG---ATGCCAATGAGGTGGAGATTGCACGTAAAGGCGGCTTGAAGCCTATCATTGACGGAGCGCACTCAGAGTCTGTGGAGTTGCAATCTCAAGTGGCCCGCGCACTACGCAATCTGTCGGTCAATCCGGAAAACAAGCAAGCGATTGTAGAGCTGGGCGGCGTGGAGGCTCTTCAGTCACTCGTGCGCTCCACCAACGACCGGATATGCCAACAGGCCACTCGCGCGCTTGTGCCCAACGTGGTACTCACGCGCTCGTCGCCTACAACCTGCTG---GCTGTGGCGCCCGTGGCCCGCGTGGCCGAGGTGAAGCAGGAGGC------CGCGCCCTACGACGGCACCATGGTGGACGTCGAATACTCGGCTACGGTCAAGACGGTC---------GTCGTGCCTGCAGCCGCCGCCA---CCGACGCGGCTGGA---GGCTTCTCGTCCATCTCGGACCGCTTCCCGCTCTCCAAGGTGGGTCGAGTCAGCTGCAGCATG---CCCATCCCCATGAGCCG---CTTGCTCAACG---CCACGCCCGAGATGACGC---AGATGGACCAG-------------ATGACGGTGCTCGTGGAAGAC---ACGGTGGACGACAAT---ACCATCAAGGCCGAG---CCCGAAGGTGAACGACTTGTACCGGTACCAGGCTACTGA--CGTGGAAGAAAACGAGATGGAGCAACTTCGATGGAACATCCAGGAGCAGGTTTTCCAGCGCTACTTCGAGCAGGGACAGAAGTACGAAGAGTGGGGGAAGCTGGAGGAGCAAGACACCGCGTATTCTTCATTCCAGGTGGAAGACGGGAAAACATTCTGGCTGAGAAGCGGTTCCACGACAACGCTGTCCGAGACGCAATGGGAAAGGGCAATGCCACTTGGGTCAAGGAACGACGCACTCAGTTCGAAGCGGAGATCCGCCAATTCTAAGAAGAAACAAGGCACAAGCAGGAACTGATGCTGACGCGCCTTGAAAAGGTGTCTCCGTTCTCCAAGTTCCCGATCCTGGGCAACCGCTTTTTGCTGCTTCGCCTACGAGGAAAGGGGGGCAACGGAGAAGTATGGGACGTCGTTGACTACGCCAACGGCAAGCAGCTTTGCGCTCTCAAACTTTCCACTTCAATTCGACACGCCCAACGCGAACACCACACGCACACTCAACCACCCAA--ACATTGTCCAGGTTGGGGATGTTCCCTTCTTGATTCGATACCAACAGCAGCAGTACACGGCTTTCACTGTGGCAAGCGTTCAATCTGACCTTCAGCAGCTCATCGAGA---TGTTGGAGTACTTGGACGATGACTCTGCGTACAAGGTGCTCTCTCAGCTTCTAAGCGCGCTGAAGTACTTGCACGATGGGCGTTGCTCACTATGACCTGAAGCCGTCGAACATTCTGATCGACCACGACGGGTGTGTGAAGTTG------ACCGACTTCGACTTGACGCGCGACGCGAAGGCGGCCACATCTAACTCGACGGTTGGAACTCTGCGGTATCTTCCCCCCGAGTGCTTCCGCCCGAACCGGGGCGA---------TTGCGAGGCCACGGCGGAGAAGGCAGACATGTGGATGGTGGGCATCGTGTACTGCATGATGGTGACCGGAAAACACCCGATTTGCCCCGACAAGTCAACGCAGGCGGAAGTGAAGACGATGATGGCCCAGTACACTGGC---GAGCTACGATACGAACGGCCGATTTCCGACCTATCTCGGTGGATCTTGCAGGGATGCCTTCACCCAGACCCCCGGTGCCGACCAACGGCCGCGCAATTGCTGGTGGCGCTCCAG

Phytophthora_rubi TGGACTACCACCGCCAGGTGGCGCGCGAGCTGGCCGCCTTCTTCCACTCCTTCTACCGCTTCGGCGCCTCCTCCAGCCGCTTCGACCTGTTCCTGCACAACGTGCTCTTCGGCTCGCTCTGCATCGGCGCCGTCGTCTTCCTCGTGCTCTGCGTCATCGTCGTGCGGCGCTGCATCTTGCACATCCGCAGGCCTATGTGCGACGCACGTCGATGGACTCCACGTCGGTGGAGCTGCTGGCCGTGGGACTTCTGACCTTCCTGGCCATTAGTGCGCTGGGCGGACTGCTGGGAGAAGCTGTCTGGAGACCAGCAACGCTCTGCGCGTCAATGCAGACAACCTCATCACGTCGTTCAACGACAGTGAGCTACCAGCAGACGCCTTCAGGATGTCGGTGGAGGCGCTGGCCTCGTGCACGCGTCCAAGGAGCTCTGGAACCAGACAGACCTGCTGCTGCCCAAGAACTTCTCGGGGCTCGCCAAGGACTGGGAGTTCAGCTACTTCGTGCTCAAGTCGTCGACCAACGGCGCCATTCTAGCGGTGACTCTGGCCAGCTTCCTGTCCATTGCGTCGGTCGGGTGGGCCATGGTCTCGCCTCTGAGGATCTCGATCATGGTCATCTTGAGCGTCGTGCCCATCTCGCACACTCTGATCGGCGCCTACCTGTCGTCGACGATCCTGACGGCAGACTTCTGTGCTTCCCCGATGAACAACACGCTCGGGCTCGTGGGCTCGTGTCCTGCGAACGCGTCTTGCCTTTTGCTGATGCGGTGGATAAGCAACGCGTCACACCAAGGAGAGATCGGTCAGCGCATGAAGAAGGAGTTCCTGGACCCCATCGGAGCAACTGTGGGCGTCATGCTCGGCTACTTCGCCGGCATGGTCAGCAACTGGGAGAGCTTCTCGCTCGTGCAGACGTGGCGCTGGCCCTTCCTGCTGCAGTTCCTGGCGCTGCTGCCCATCAACGTCGCCATCTTCTTCGTGCCCACCAAGCACCTCGTCATCCGCAGCGAGGCCAGCGAGCTGGAGCCGCCGGTGGCCGGCACGCCCATGAGCACCCGGTCCACGACGTACCACGACGAGGAGGACGCGCTCCTCGCCAGCGCGAGCATGCCACGTACGGCGCCTTGGACACGAGGTCTACGAGCAGGGCGCGTTTTCGGAGGGGGTGTATGAGCTCATGCACATCCCCGTGTTCTGTTTGATTGTGTTTGGGCTCACGACCGTGTACTTCGTAGTCACGGGAGTGCAGTATTGGAGCACCATCTTCATGATCAAGTCGCTGCATGCGTCCAAGTACCTGGTCAACGGACTTTTTGTCGTCGTGTCCGGAACTGGACCCATTCTCGGCGTGTTTTTCGGTGGATGGATCATCGACAGATATGGAGGATACATCGGTGTGGAGCAGCGCGCCAAGGCTTTAGGGATCTGCATGATCCTGGGGCTGACAGCCTTCTCGATTTCGGCAGTGACGACGTTCTTCGATGACGTTTACATCACTGCCGGCTTCCTATGGCTGCTGCTCTTCTTCGGCGGCGCCATCCTGCCGGCGCACCGCGCGCTCGCGTCGTCGTTCTCAGTGATGGTGTTCAACCTGTTCGGGTATGCGCTCTCGCATGAGCGACCTGCAGATGGTGGTGGGCGCCAAGTGCTTCGTGCCGGACGAGACGCACGTGTGGCTCGCGGCCGAGGTGCTGCGCGACGAGAACTCGGGCGACGGCAAGACGCGCAACATCTACTGCACGGTGCAGCTGCCCGACAGAGACGGAGGAGCGATGCGTGGACATGCTGTCCAAGAAGACCAAGGCGCTGCTGCCGTGCACCAGCTCGAGTCGCTGCCCTACCAGAACGAGAACGTGGGGCCCGAGGGCATCGAGGACATGATCACGCTCAACTACCTGCACGAGGCCGCCATCCTGTTCAACGTCAAGAAGCGCTTCCTCCGCGAGCTTCCCTACACGTACACGGGCGACATCTGCATCGCCGTGAACCCGTACAAGTGGCTCCCGGACCTCTACGCGGAGGAGCAGCACCTGCGCTACCTCAACCAGCCCAAGGAGGAGCTGCCCCCGCACGTGTACGCCACCTCCGTGGCCGCCTACGACAACATGCGCCGCTCGGAGATCAACCAGTCCATCCTCGTGAGCGGAGAGTCCGGCGCCGGTAAGACGGAGACCACCAAGATCCTTATGAACCACCTGGCCACCATCGCCGGCGGTCTCAACAACTCGACCATCAAGAGGATTATCGAGGTCAACCCGCTGCTCGAGTCCTTTGGTAACGCCAAGACGGTGCGCAACGACAACTCGAGTCGCTTCGGCAAGTTCACGCAGCTGCAGTTCGACCAGAACGGCACGCTCGTCGGCGCCAAGTGCCGCACGTACCTGCTCGAGAAGACGCGTGTGATCCAGCACGAAGCGCCCGAGCGCAACTACCACATCTTCTACCAGTGGACT---CAGACATCGCGCCCGAGCTGCAGCTCGAGTCGGGCAAGCACTACGTGTACACGGGCGACAACACGGCGCAGAAGATCGAGGGACTCTCGGACGCCAAGCACTTCAACAAGACGCGCGAGGCGCTGGAGCTCGTGGGTCTGTCGCCCGATGACCAGCGACCGCTGTTTGAGGTGCTGGCTGGTGTGCTGCACCTTGGTGAGGTGCAACTGCAGTCCGACCCGTCTGATGACGAGAAGTCTCTCATCACTGAGGGCGACATTGGCGCCAGCTCTGCTACCCAGATGCTTGGTGTTACCTGGGAGGCCCTTCAGACGGCTCTGTGTTCGCGCACCATGCGCGCTGTGAACGATGTCTACAGCGTGCCGCTCAAGAAGGAGCTGGCTATGGACTGTCGTGACGCGCTTTCCAAGGCTATTTACTCGAACGTGTTCGACTGGCTCGTGACCACGATTAACCAGTCGCTTGCTGACGATGCGAACATGGCCAACCACGTGGGTGTGCTTGATATCTTCGGTTTCGAGCACTTCAAGCACAACTCGTTCGAGCAGTTCTGTATTAACTACGCCAATGAGAAACTTCAGCAGAAGTTCACGCAGGACGTGTTCAAGACCGTGCAGATTGAGTACGAGGAGGAAGGCATTGTTTGGGACCACATCGAGTACGCGGATAACCAGGATGTGCTGACCGTGATTGAGTCGCGCATGGGTATCATCTCGCTGCTGAATGAAGAAGTGATGCGTCCCAAGGGTAACGAGGAGTCCTTCATGTCCAAGGTTACTTCCCTGCACAAGGAC---GATATGGCGCACGTGATTGAGTTCCCTCGCACATCGCGCACCGAGTTCCTCATCAAGCACTACGCCGCTCCGGTCATGTACGATTCGGTGGGCTTCCTGGAAAAGCACAAGGACTCGCTGCTACCTGATCTGTCCGAGCTCATGCGCGGTTCGTCCAAGCCGTTCATTGCCAAGCTCTTCGATCCGAAGCCGGCGCCTAAGACGTCGGAAGC---------GTGGTGGTGCTTTGTCCATCACGACTGTCGGTACCCAGTTCAAGGAGAGTCTGACAGAGCTGATGGCCACGATCAACTCGACACGCGTGCACTATGTGCGTTGCATTAAGCCCAACCCGATCAA---------GAGTGCGACGGTCATGGACCAGAACATGGTGGTGTCCCAGTTGCGTTGCGCAGGTGTCATTGAGGCCATTCGTATCTCGCGCGCGGCATACCCGAACCGATTGCAGCACACTGAGATCCTGGACAAGTTCTGGCTGTTCGTGCCGAGTGGCGGTGATACTGCAGCGGAAAAGTGCCAGCTGCTTATGGACAAGTTGAAGCTCGAGACGCCGGTGCAGTACCAGATGGGTAAGACTCGTGTATACTTCCAGCTTGGTGTCCTTGAAGAGCTTGAAGACCGCCGCAAGAAGTTCTTGGATGCCAAGGCGACCTACCTGCAGAACATCATGCTTGGTTTCACGCAGCGTATCAAGTACCTCCGACGACTGGAAGCGATCATCAAGTTCGGTGATCCGGTGCGTGATTGCCATGCGGAGATACAACACGTTCATGAACGGTCTTGTTAAAGCTCAAGCTCACTGGCGAGGCATTCAGGGCCGTAAGGTGGCGGCCGATGTCAAGAGCAACCACAACGCTGTCATTATCCAGCGGTATGTGCGTGGTTTTGTCAAGCGTCACCGCTACGTTAAGATGCGCTCCATGGTCATCCGCGTGCAGGCCGTGGTTCGCATGACGATTCAGCGGCCCAAGTACCTGGCCGCTCTCGAGGAGAAGCGCCGCGAGGCTGATATGGCTTACCAGCTGCAGAAGCTCAAGGCAGCTCTGCAAGAGGAACAGGATCGGAATGCACAGCT------GCAGCGCC---GTAGTAGTGTGGT--GATGACGCATCCGTTGCGTCAAGCGAGGTTATGGCTGACGCTGGCGGCATGATCGAGACTCTTACGGGTGAGAACAAGAAGCTGCGTGAGAAGAACGAGGAGATGAAGGTTACGATGAAGGGCTTGAAAGCGGAGATCGAGAAGTTCAAAAGTGACAAGGAGTTCTCGTCGGCTGGCAACCACGTTAAGGTCCGCCAGCTGCAGGACACCGTGCGCGACAAGGACAAGAAGATCTCGCATCTTGAGGCCGAAAACAAGAAGCTTACGGAGCAGATCGCCAAGCTTCACGCGGAGGGTGTTGT---ACCTGAGAAAAAGTCGATCCCGAAGAAGTCGATCTTCCGCACTCTGGGCTCGAAGAAGGAAAAGAAGCCGC---------------GCGAGACCGTGCTGATGGACTCGCTCACGGGCTCAGACGACCTCAACCGCCGGGAGGCTCGTATCTCGGA---GCGCCACTTCCCGCGCATGC---CGAGTATGAGCGTGGGCTCG---CGCTTCTGGAACTCGAACAAG---AAGGGCGAC------------AACGGCGATGGCTTCGACGTCTCGGAGGTTGACGGTGAGGACAGCCCCACGAACCCACGCGCAACACGATCGTGAAGAGCATGGAGGTGGGTGTCACGGGCGCTATGTCGACCCTGAAGGGCCGCATG---------TCGGTCGTGAAGGGCATTTACGAAGCAAGCGCACGAACAGGATCGACCCGTGCGCGAGTCATTCAACCTGGACGCGCTGCCGGAGGTCTCTCTTCCCGTCGGTTGGGAGGCCAAGGTGTCGCGCTCTACGGGCCGCGTGTACTACGTGAACCGCAAGCTGGGCAAGTCCCAGTTTGAGCGCCCCACGCTGGCCTCGCTCAA------GGCGCAGAAACTGGCGCGCCAGAAGTCGGCTAACGCGCTGTAAGGCGGTGGTGGCGGATTTCGGCTGCGGAGACGCCAGGCTGGCCGAGAGCGTGCCGAACAAGGTGCACTCGTTCGATTTGGTGTCCCTCGACATCGGCGTGTATTGCCTGGCACTTATGGGGACGAGTGTGCGCGAGTATGTGCGCGAGGTTTACCGCGTGCTGAAGCCCGGCGGCGTGTTGAAGGTGGCGGAGGTCAAGAGTCGATTTGAGAGCGAGTCGCTGGGCGGATGGTGGCGGTGTTTGTGGCGGCAGCGCGGGGCGTCGTGGTGACGGTGCGCAATACGACGGTGTTCGTGGAGAGACCAAGTACGCTGTCGTTGTGGGCCAGAAGACGGGCGAGGCCGTGGCAGTGCCGTTCCCGGACGTGGACCGCGACCTCAAGACGCTGCTGGGCCACACTACGTCGATGGTCACCCATGTGGCCGTGAACCTCGTCGCTGCTTCTGACGGCCGATCGTGATGAAAAACTGCGCGTCTCGAGGTTCCCGAACGCTGCTATCATTGAGAGCTACTGCCTGGGGCATGCTGCTAGTCTGACCAAGGTGGCGTGCAGTGCTGTCACGCCGGAGCTTGTGGTGTCCACGTCCATGGACAACACACTCAAGTTGTGGGAGATGAAGACGGGGAAACTGGCGATTTGCCCCACGACGACATTGTGGCGGTGCTCGTGAACTACCAGCACGTGCGGTTCTTCGAGATCGTGAGAACCCGTTCCTGCAGTTGTTTTCGATTTCAAAGGGCCGCTGCCGTGTTCAACTGCGGTGCGCGACTTCCGCAAGCTGCAGAACGACCCGCCGTCGGGCGTGTCGGGCGCCCCCATGGACAACAACATCATGCTCTGGCAGGCCGTCATCTTCGGACCGGATGACACGCCCTGGGAAGGCGGCACCTTCAACCTGACGCTCGAGTTCAGCGAGGACTACCCGAACAAGGCGCCGACTGTCAAGTTCATCACGAAAATGTTCCACCCCAACATCTACAACGACGGCCAGATCTGCCTGGACATCCTGCAGAACCAGTGGTCGCCGATCTACGACATCTCGGCCATCCTCACGTCCATCCAGTCGCTGCTGTGCGATCCGAACCCGAACTCGCCGGCCAACTCGGAGGCCGCGCGCCTCTTCCAGGAGAACCGGCGGGAGTACAACCGACGCGTGCGTGAGATCGTCGAGCAGAGCTGGCAGGCGATCGATGGTGTCGGCCGCCAAGATGCGCGGCGCCGAGAACCGCATGAACGCCGGCCGCCCCTTCACGGCGTGGATGGACAACGTCTCGTCCGACCGCGGCCTCTGCGGCGGCATCAACTCGGGCATCGCCAAGACTGCGCGCAAGCAGACCACTCGCAGATCTTCGTGATCGGCGACAAGGCCCGCGCCCAGCTGCGTCGTGACCTCGGAGACAACATCCGCGGCAACGTGACGGAGACGTACCAGTCGCCCCCCAACTTCACGGTGGCCAGCGCCATCGCGGAGGCCGTCGACTCGGAGAGGAGTCGTCGCGTATGGCCGCCATGGAGAACGCCACCACGAACGCCGAGGACCTCACGCTCACGCTCGTGTACAACAAGGCTCGCCAGGCCCGCATTACCACGGAGCTCATTGAAATTATCTCGGGTGCCGCCTCGCTCGACGGTGACTGGCAGCGTGGTGACCATCCAGAGCGCGGCGCAGTTCGACGTGGCCACGGCGCGCGAGTCGACGCTGTCCGTGACCTTCTTCTGGGCCGAGTTCCAGGCCTGCCGCCCCAACGGGCAGCTGGACGTGGGGCCGCGCCGTGCTGGACAAGCTCGAGGGCGCCAACGTGGCCGAGCTGGCCAAGCGCGTCTGCAGTACCGCCTCAAGAAGCTCATCAGCGCGTCCCCCGTCATGCTCTTCATGAAGGGCAACCCGACGGAGCCCAAGTGCGGCTTCAGTCTGCAGCTCGTGGCGCTGCTGAACGACGACAAGATCCAGTTCGGCACCTTCGACATCCTGAACGACGACGAGGTGCGGCAGGGGCTCAAGCAGTTCTCCGACTGGCCCACGTACCCGCAGCTCTACGTGAACGGCGCGCTGGTCGGAGGGCTGGACATCGTCAAGGAGATGAAGGCGGACGGCTCGATCGTCGAGCAGCTCGGCGTCGAGGAGGCCGAGGCCGCGTTCCAGGAGAGTCTGCGCGCGCTCGTGCTGTCGGCGCCCGTGCTGCTCTTCATGAAGGTTCGACATCTTGTGTGGAGTTACCAACGCCAGGACCAAGATATCGCCAAGGTGGTGACGCTGATGCCATCGTCTTTCCGCCGGGAAATGTGCCGAGGCCATTTCGTGGAGTCCGAAACCGCATGGCCATGGCATTCCGCGATCCCGTCGACGGAAAGGGTGAATTTTGCATTGTGCAGCTTCTCGACTAACCTCAAAGCGCACTACCCTGCATCAACGAGGTATTTCTGCGATCGACTGGCAAGTGGAACGTGCTGCACCGCGACCACCGCAGTGAAGTTCGATCGCTCTGTGTGCACTCGAAGCGCGACTGCTTGTACACGGGTGGTCTGGATGGCCTATTGATCCGATATGATCTCAACAAAGGGCGCACCCAGAACGTGATCGAACGCCGCAAGCACATCTCCAAGATCAACGCGGTGCTGGAGCACCCGCACAACCCGAACCTGCTTCTGGTCAGTTGCGTGGAGCAGTACTCACAACTTGTTGCTGCATGATCTCCAACGCTACGACCCGAAAGTGTCGGGTGGTGCCATGAGTCAGTACGTTGTGCCGAGGTGGTCGCCTGCAGGCTATCACGTGTCGTGCGGCAGCAAATGCGCTACTGCCACGGCAGCTTCTCCACGCCCACGTCGGCCACTATCGGGGCCTCGTTCCTGCAGAAGCGCGTGATCGTGGGCGGGAAACTGACGCTGCAGATCTGGGACACGGCCGGCCAGGAGCGCTTCCGCTCCATGGCGCCCATGTACTACCGCAACGCCAAGGCGGCCATCCTGGTCTTCGACCTGCAGAGCGAGGCCAGCTTCGAGAAGATCAAGGAGTGGCTGCACCTGCAGCACCACGTGGGTGATGATATGTAGGCAACAAGAGCGACGTTTCAACTTCTCCAAGGCCCAGCAGTTCGCGGACGAGATCGGCGCGCTCGTGTTCCGCACGAGCGCCAAGACGGGCGAAGGGGTGCAGGCACTGTTCGAGAGTCTCGCAGTGCAGCTGCTCAAGAAGCATGACAAGGAGCAGCAAAGAGCTTCTTCGTGGTGTACGTGCCCGCCGATGAGCGCAAGGAGCTGGAGGAGTGGGCGGTGGCGCTGCCCAAGGACAAGGAGGCGCAGCTCGGCTGTCTGACGGAGCGACTGCGCGCGCACTTCAAGGCGGGGGCCATCCAAGCAGCAGCAGAGACGTTCAAGCAGCAACTGCTGGCTCAGCTTCCCAAGGGCGCCACTATGACGGACGAGATGTTGCAGATGATGCATGGACAGCCTCGTGGACTCGATCCCACTCGTGCTCAACACGCCCGACGCCAAGCACGTTGGAGGTCAAGGGCGATGCATTCATCGGCCGGGTGTTTGATAGACGACTTTGTGCGTATGGACTTCCGGTTGAGTGAGGTGTCGGGTGGCGCTGCGTGGGTGCAACAGGGCAAGCAGCAGAGGTCACGCAGTCGCTGGAGGAGATCCTGGCGCTGGGCCAGGGCCTGGACGACCAGGACTACAAGGCCGAGCTCGAGGTGCTGCAGAACGACCCCAACTCGAACCTCTACTCGGCCGTCACCTTCGAGGAGCTCAACCTACCGGAGCTCCTGCTCAAGGGCGTGTACAACATGAAGTTCGCCAAGCCGTCCAAGATCCAGTCGGTGGCGCTGCCGCTCATCCTCGCCGACCCACCAGAGAACCTGATCGGCCAGGCGCAGAGCGGCACCCCACACGCGAGCTGGTGAGGCAGATTATGGCTGTGGTGAACGCCATGGGCAAGTTGTGGACACGTTCCTGGCCATCCCCGGTAACGACCTGGCACGTGGCGAGACTCTGAAGGCTCAGGTGGTCGTAGGCACCCCCGGCAAGGTCGAGGGCATGATCAAGAAGAAGCAGCTGGACACCCGCAACGTCAAGATCTTCGTGCTGGATGAGGCCGATGTCATGGTTGCCGAGGACGGCATGCGCGAGCGCTCCGTGGCGATTAAGCGTCTGATCAAGAACCGTGCGTGTCAGCACCTGCTGTTCTCGGCCACGTACGCTGACGACGTGCGCGACTTCGCCCAGAAGATGGTGCCCGACCACAACATTATCACGGTCAAGAAGGAGAAGCTGACGCTGGACGGCATCAAGCAGTTCTGGATCGACTGCAAGTCTCGCGAGAACAAGTTCCAGGTGCTGTCGGACATTTTCGCCATCCTGACCATCGGCAAGTGCGTCATTTTCGTGCAGCAGCGTGACACCGCCAAGGAGTTGACGCGTGCGATGCGTGAGAAGGGCCACTCAGTTGGCATCCTGCACGGTGCCGACATGGCGAAGGAGGTGCGCGACCAGGTCATCGATGAATTCCGCGCTGGCACGACCAATGTGCTGATTACCACAAACGTGCTGGCGCGTGGCATTGATGTGGCGGGTGTGTCTCTTGTGGTGAACTTTGACATCCCTCTGACGCGAGACCACCGTCCCGACCCGGAGACGTACCTGCACCGTATCGGTCGTACGGGTCGTTTCGGCCGCAAGGGCTGCGCTATTAACTTCGTCTACGACAACAAGAGTAAGCAGGACCTGGCGGAGATTGAGGAGTACTACGCGCGTCCTATTACCCAGGCTCCTGCTGACGACATTGAGGAACTCGAGAAGCTGCTGGCTTGGGGGGCAGCAAGCGCCGCCTGGCCAAGTTCGGCAAGGAGCAGCGCAAGGGCATGCGCATCTACCCGCGCGAGCCCAAGAAGAAGCCCTCGGGCAAGGACAAGACGTACTACTACCTCAAGGACGTCATGTTCCTGGCGCACGAGCCCGTGCTCGAGAAGTTCCGCGAGTTCAAGACGTTCATGAAGAAAGTCAAGAAGGCCATGGGCAAGAAGGACTACGAGGACGCCAAGCGCAAGCACGAGCACCGACCCACGTACGAGCTCGACCACATCGTCAAGGAGCGCTACCCGCGCTTCGTGGACGCGCTGGGCGACCTGGACGACGCGCTGTGTGGCAGAACTACGTCGTGGCCACGCGCGCGCTCACCAAGGTCTTCGTGAGCATCAAGGGCATCTACTACCAGGCCAACGTGCAGGGCCAGACCATCACGTGGCTCGTGCCCCACCAGTTCACCCCCACCGTGGACAAGCGCGTGGACGTGCGCGTCATGCTCACGTTCCTCGAGTTCTACGACGTGCTGCTTAAGTTCGTCAACGCCCAGCTCTACATGCAGATCGGCGTGGCCTACCCGCCCAAGATCAACCTGCAGCTGGACGCGGCCGGTGTGCAGCTCGCGGCGCTGGAGCTAGCAGCAGAAGGACTCCGAGAAGCGCATCAAGCTACAGCGAGCAGCGCAAGGAGCGCAGCGAGAAGAGTCTGTTCAGCGGTCTCACCTTCTTCCTGTCGCGCGAGGTGCCTTCGGCCTGCCTCGAGCTGGTGCTGCGCTCGCACGGCGCAACTCTCGGCTGGGACGGTGCTGGGTCGCCTTTTGACGAGAAGAGCTCGCGCATTACGCACCACATCATGGATCGTCCGCACCAGGGCCACCGCTACTTTAACCGCGAGTACGTGCAGCCGCAGTGGGTGTTCGACTCGGTCAACAACGGCGTTCTGCTGCCTCTGACTCGGTACCGCAAGGAGCTCGAGAAGCTCAAATCGGCCTCGCAGGTGCTTCGCGAGGTCGGCGCCGTGGGCGACGAAGACGAGGATGAGAGGCTTACGCCGCTGACCTTAAGGCTGAGATGGACGGTAACAAGAAGTCGAAGCGGGTCAAGAAGGAGGAGGAGGCTGCGGAACTCAAGGAGCTGGCCAAGACCATGATGTCGAAGAAGTCCAAGCGCCTGTACGACCGTATGCAATACGGCCTGGGCAAGAAGGCCGACAAGATTCAGAAGCTCGAGGACAAGCGCGCTAAGCTGGAGGCAAGACCAAGAAGCTGTTAGCTGGCGCGCTGTCTGGCATGATCTCGGCGCTGGCCTTCCAGCCGCTGGGGCGGTTCGCTGGCGCGGCACGTCGCCCACGCTCGTGCGCGTGGCGGGTGGCGCCGGCCTCTACTTCGCCACGCTGGACCACTGCCTCAACCTGTTCCCGCAGTCGGCGGCCAACACTTTCCTGGCCGGAGCCTTCGCGCGCACGTTCGCAGGCGGCGTAATGTCGCCATTCACGATCGTGAAGGCGAGGATGGATCCCCCCGGGCACCTTCGACAGCAAACGGACGTGCCGTTCTCCGGCCTCTACGTGCTCATCTACACGGCACCCGGCCGACGTGGTAAAAACGCGCATGCATGCGGCAAACAGTGGCCAAGATCTACCGCCACGAAGGCCTGCGCGGATTCGCCAAGGGGGTCGTTCCGCGTGTAGTCAAGCGCACGCTGTCTACTGCAGTCACGTGGACGATATACGAGCTACTCGTTCGACCCGTCGGGTCTGGGCCACGCCAAGGAGGCCTTCAACCTGGCCAAGGAGACGGAGCGCACCAAGCAGACGGAGAACCAGGCCAAGATCAAGGAGAACGAGGCGCTCTACAAGCAGTACGAGATCGTGCGCGTGCAGAAGGAGGGCGAGGAGCGCCGCAAGACGCTCGAGGAGGAGACGCGCCAGCACCAGCAGCGCGCGCAGTACCAGGACCAGCTCAAGCGCAAGCAGTACGCCGACCAGCTGGCTGCCCAGAAGTACATGAAGGAGCAGGAGCTCAAGAAGCAGGAGGAGATCCTCGCTCGCCAGGAGGCCTCGCGCCGCAAGACCCTCGACTACGAGGCTGAGCTGCGCCAGAAGACGGAGCTGGCCAAGGTGTCCGCGGAGACCGAGGGCCGCATCAAGCAGGAGCGTCTCAACCACGACCTGCACCTCGAGGAGGCCCGCGTGCGTGCCAAAGAGTACCGTGAGACGGTCATGGAGGGCATCAAGCTGGCCGGTAACACTGTCGGCTCGGGAATTATGACCTTCGTCGACGACAAGGAGAAGCTTACGGCCACCGTGGCCTCGCTCACGGCCCTTGCTGTTGGTATTTACACCGCCAAGGTCTCCACTAGCGTTGCGGGCAAGTACATTGAGGCGCGCATGGGCAAGCCGTCGTTGGTGCGCGAAACGTCGCGTCGCTCGGCTACCCAGGTGGTGGCCAACCCCATCCCGTCCATCAAGCGTGCGCTACGTCTGCAGAAGGCGACGGACGCCCTCGAGGGCGTTGTGCTCGAGCCCAAGCTGGACGAGCGTCTGCGCAGCGTGGCCGTGTCCACGTTCAACACGAAGAAGAACCGCGCGCCGTTCCGCCACTTGCTGCTGCACGGCCCCCCTGGTACGGGTAAGACCCTGTTCGCCAAGGCTCTGGCGCGTCACTCCGGCCTGGAGTACGCCATCTTGACGGGTGGTGATGTTGCCCCACTGGGCCGCGAGGGTGTGACGGAGATCCACAAGCTGTTTGACTGGGCGTCGCACAGCCGTCGCGGTCTGCTGCTGTTCGTCGATGAAGCCGATGCGTTCCTGCAGAAGCGTAGTAACACCGTCATGTCCGAAGACATGCGTAACGCGCTGAATGCTTTCCTGTACCGCACAGGTGAGGCCAGCGACAAGTTCATGATCGTGTTCGCCAGTAACCAGCCGGAGCAGTTCGACTGGGCCATTAACGACCGTATCGATGAGATGGTGGAGTTCCGCTTGCCGGGCTTCGACGAGCGTGTGCGCATGCTGAAGCAGTACTTCGACGACTACCTTCGTGCGCCGAAGAACTCGCGCGCCAAGAAGATCTACGCGAGGGCGTCATCTTCACGCTGGACCCTGTGGCCAACTTCCTCGTGCCAGGACGCGCATCTTCCAGCTTGAGGCTCTGGAGGGTGTAACGGGTGAAGCTCAGGCCATCTTCGATGCACTCAATAAGAGGCCATGCGCGTGTACTGGCTCGTCTTCGCGCTGGTGGTGGCCAACGGCCTGTTCACATACGCAACCGGCAAGGACGAGTCGTTCCTGGTGGAGAAGCTCAAGAGAAAGATCCCGTCCGAGTACCGCATCTCCGAGAAGTGGGACAAGTGCCTGGAGAACTTTACGCTGTACTTCGGAGCTGGCCTCGTGGCTGGCGGCCTCACGTCCCTCGTGCTGGCGCGTTCTGGCGCGGGTCGCGGGCTGATCACGGGTCTGGGTGCGCCGGTGCCGGCTCGAGCTGGACGACGTGCCAGATGGCATTCGCAGGCGACGCACAGGCCGCGCTGGACAAGACGGACAAGGCTGTGGGCGACTTGCCCGCCTTCCAGCAGCAGACGATCGTGGACTTCGTGTCGGCCTCGGCCACGCAGCAGCAGCTGCAGGCTGTCCACCCGCGGCTTCTGCGAGCAAGCCAGCGTGAAGGTCCTGGAGACGTACCTGGCCGAGCAGGTCAAGAACTCGACCGTGGACATCGACGCCAACCTGGCGCTGCTCAAGCTGTACCAGGTGTACCCGGCCACGGCCAACGCCGAGAGCCACGGTGCTGGTCAAGGGCGTCATGGCGCTGCCCTCCACGTTCTTCACGGGCGTCCACCATGGTCCCGGAGAACATCCGCGAATATGTCAATGTGACGGCGGTGCTGCACGCGGGCTTCCTGCTGCAGTCGTGCCTGTTTGAGGACTTCTGGAAGGAGAGCGTGGCTTTCGCCGAGAAGGTGCCGGGCTACCTGGAGTCGGTGCGCGCGTACATCCTGACGGCCATCAGCCGCAGCCACAGCGCCATCTCCACGGACGTCCTTAAGGCCAAGCTCAACGTCTCCGACAAGGAGGTGGCTGACATCGTCGCCGAGAAGTGGACGCGGCCAACCTGATCCAGATCAACCCCAACGAGGACAACCAGATGCAGGCCAAGAAGGTCCAGGAGAACATCGAGTTCGAGGACGTGCTCAAGGTCATCCACACCCTTTCTAGATCCAACGTCTGCGCCAGCGGCGGCGGCGTGGCCGACGCAGAGGAGGTCTGCGAGAGCCTGGCCAAGCACCTGCGGCTGGAGGGCGGCGCGCAGGTGCTGCCCTGCTCCACGGGCGTCATCGGCTGGCGCATCCCCGTGGACGCCATGGTCGAGAACCTGCCCAAGCTCGTGAGCAACCTGCAGTCCGACTCGGTGCTGCCTGCTGCTGAAGGCATCATGACCACGGACCTGTACCCCAAGATCCGCTCCATGGACGTGTGCGGCGGCCGCATCGTGGGCATCGCCAAGGGCGCCGGCATGATCGAGCCCAACATGGCTACAATGCTGTCGTACATCCTGACAGACCTGTCGATTCCGCGTGACGTGTTGCGTCAGCTGCTGACGGAGGTGGTGGGCGACACGTACAACGCGCTCAGTGTGGACACGGACCAGAGCACGTCCGACACGGTGACGCTCGTGTCGTCCGACCAGATCCCGTTCGACATGGA---CAAGCTCGACGAGTTCAAGAAGGCGCTGCACGAGGTCTGTCTCGGACTGAGCGAGGACATTGTGCGCAACGGAGAGGGCGCGCACCACGTCATGCGCGTGCGCGTGCGTGGCGCCGTGACCAAGGAGATGGCCAAGGGGGTGGGCAAGTCGGTGGTCAACTCGCCGCTGCTCAAGTGCGCCGTGGCCGGCAACGACCCGAACGTGGGTCGCCTTGTCATGGCCGTCGGCAAGTACATGGGTCTGCACCACAAGGACGTGGACATCCAGCGCCGCATGAAGATCCGCATGGGTGGCGTCGTCATCTTCGAGAACGGGGAGATGGTGCTCAACGACGAGATCGAGGCCAAGCTGGTGGCGCACATGCGCGACGCGATGCTGATCGAGCCCACGCGCGGTGGCCTCGACGCCAGCTCGCACAACTTCCCGCCCCACGGGAAGTTCGTCGAGATGGAGATTGACTTGGGCGTTGGTTCCGAGGAGACTCAGGTGCTGGGCATCGACCTCACGCACGAGTATGTGGCCATTAACGCGGACTACCGCTCGTAATGGCGCC---GAAAAAGGGGAAGACCGCCGCCGCGTACCTGGCGTCTCTGGGCGTGGCCCCCGC---CGCTGGAGGCGAGAAGAAGGACGAC---AAGAAGAAGAAGAAAAAGGGCAAGAAGAAACCCAAAAGAAGCCCATGTCGGCGGCGGCCAAGGCGCTGA-TGGAGCGCCAGGCGGCCCTCAAGGCGGAGCAGGAGCGCCTCGAGCAGGAGCGACTGGAGCAGGAGCGCATCGCCCAGGAGGAGGAGTCCAAGCGCCTCGCGGAGATCGAGCGCGAGGAGGAGGCCCGCCGCAAGAAGAAGGAGGCGCGCGCCGCTAAGATCGAGCGCCAGAAGAAGGAGGGCACGTACATGACCAAGGCCCAGAAGCAGAAGGCGGCCAAGGCCCAGGCGGCCCTTGAGGCCATGCAGCAGGCCGGAATCGTGCCCACCGCTGCC------GGCGAGAAGAAGAAGGTCGTGTACACGGACAACAAGAAGAAGACCAAGAAGAAAAAGAAGAAGAGGCCGAGGAGGACGAGGCGCCCGACGCATGGGACGACGAGGACTGGGAGACCAGCGACGTCGTGGCCTCGCTGGAGGACAAGCTAGCAGAAGAAGGAGCAGGAGCGTCTGCGTCAGCAGGGTATTGCGCTGAAGAAGCGCCAGGAAGAGCAGGCTGAGCAGATGCGTCTGGACGATGAGGAGGAACGCCAGCGCTCCAAGGCTAAGCAGGAGGCGGATCGCAAGAAGAAGGAGGCAGCTGAACGCCGTCGTGAGCGTGAGGCCGCCGCGCGCGCCAACGTGTCTCTGGACAACCTCCGCTCGCCTATTTGCTGTATCATGGGCCACGTCGATACCGGTAAGACCAAGCTGCTTGACAACATCCGAAAGACCAAGGTGCAGGACGCGGAGGCTGGTGGTATTACCCAGCAGATTGGTGCGACCTTCTTCCCCGTGGAAGCCATCAAGCAGAAAACGGCGTGCCTCGCCGAGACGATGAAGCTGGAGTACCGCCTGCCCGGTTTGCTTGTGATTGATACGCCCGGTCACGAATCTTTCACGAACTTGCGTACGCGTGGTTCGTCGCTGTGTGATATCGCCATTCTTGTGGTTGATATCATGCACGGTCTGGAACCGCAGACGTTGGAGTCGCTGCGTCTGCTGCGCCAGAAGAAGGCTCCATTTGTTGTGGCGCTGAACAAGATCGATCGCTGCTACGGCTGGAAGACGATGCCGGATATGCCCGTTCAGGAGGCGCTCAAGCACCAGAACGAACACGTCATTCGCGAGTTTGAGGACCGCATGAAGTCAATTATCGTTGAGTTTGCCGAGCAGAAGCTGAACGCTGAGGTGTACTGGCGCAACAAGGACCTGGCGCGCACAGTCTCGCTCATTCCCACTAGTGCCATCTCGGGCGAGGGTGTGCCAGATCTGCTTATGATGCTGACGCGTTTGACGCAGGAGCGCATGGCGAAGTCGCTGGCTTTCGTGGATATTCTGCAGTGTACCGTGCTTGAAGTGAAGGTTATCGAGGGTCTCGGTACTACAATTGATACGATTCTGGTGAATGGTACTCTGGAGGAAGGCGCGACTATTGTCGTGTGTACATTGGACGGACCTGTTGTGACGACCATCCGTTCGTTGCTGACCCCGCACCCCATGAAGGAAATCCGTGTGAAGGGCGAGTACATCCACCACCAGAAGATGAAGGCTGCAATGGGTGTTAAGATTTGCGCGCAGGGTCTTGAAAAGGCGGTTGCAGGTACGCAGATTTACGTGGTTGGCCCCGATGACGATGTCGAGGAGCTTAAGGACTCGGTCATGTCCGACCTGACGGGCATCCTGGACTCGGTGAAGGCCACCCGCCGCGGTGTGATGGTGCAGGCTTCGACGCTTGGTGCCCTGGAGGCACTTCTGGAGTTCTTGCGTTCGTGTGACCCGCCCATTCCGGTCTCGTGCGTGAACATCGGACCCGTGCACAAGAAGGATGTTATGCGTGCCTCGGTACAGCTGGAACACCAGCCCGAGTTCGGCACCATTCTGGCATTCGACGTGAAGGTTCACAGTGATGCTACGGAGCTGGCCACCGAGCTCGGCGTGCGCATTTTCACGGCTGACATCATCTACCACCTGTTCGATCAATTCACGGCCTACATGGACAACTTCCGTCAGGCACGCCGCGAGGAGTTCGCAGAGGTCGCCGTGTTCCCGTGCGTATTGAAGATCCTGCCAAACTGCGTATTCAACAAAAAGGATCCGATCATTCTCGGCGTGGATGTCGAAGAGGGCATCCTGAAGGTGGGTACGCCGCTCGTTGTGCCGTCCGCAGGTG---GCTTCCTGGTGGGCAAGGTCGGCTCCATTGAGCGCGAGCACAAGGAGGTGGACCGCGCCAAAAAGGGTGCGTCAGTGGCTGTGCGGATAGACAACGAGAGCAGCGTCATGTACGGCCGCCACTTCGACCACAACAACAAGCTCGTGTCGCGCCTGACGCGTGAGAGCATCGACGCGCTGAAGGAGAATTTCAGGGAAGACCTGCAGAAGGAGGACTGGCAGCTCGTCATCAAGCTCAAGAAGGTGTTCGATATCATTATGGAGGGCTGGCTCACCAAGCGCCGCGACCACATGAACCTCATCTGGCTGGATCGCTACTTCCTGCTGGACGGCAACCAGCTCCGCTACTACCGCAAGCGCGGAGACCCGGCGCCTCGAGGCACGTACATCCTCACGGACGCGTGCGTGGTGAGCCCCGTGTTCAACTCGGAGGAGAAGCACAAGCACCACGGGCCCGTGTGGATGTTCCGCATCACGTGAGAAGTCGTACAAGAAGGACCGGTTCCTGGACCTGGGGGCCAAGAGCGAGGAGAACGCCAGAGAGTGGAAGCGCGCGCTGGAGAACGCCGTGCGCTCCATCCGCGAGCTCGTGGCCCACCAGAAGAAGCTCAAGACGGACGCCGAGAT---CTTCCCCACGCCCACGCAC---------GTGCCGCGGCTGCTCAAGACGGACGAGGACACGGTGTCGCTCTTCAATCTGGACGCGGATAACGTCAAGGCGGAGCAGGCCTGGTGGCTGGCCAGGGTGGAGGGCGGCTTGAGGATCATGCAGGAGTGTCCACTTGGTCCGAACGTGGCGGCCAACCTGTACGCGCAGCACGCGGCCACCACGAACGGACTCATCTTCAGCGCCTTTGTGGGCGCGGCCTTGAGCTTCAGCAGCATGTACTCGTTCGCGAACAACATGCTGCTGAGCTTCATGACGGCGCTGGTGGCGATCGCCGTGTGTTGTGCAGCAGTGACGCCTACGCAGACGCCCGAGATCCCGAGCTTCCGGGTCTCGCAGGTGGTGCACGGATCCCCGACGGAGGTGTTTAGGCTCATCATGAACTCCAAGAGATTCCAGAGATGGGACTCGGCCACGGCCACCATGCGCGTGATTCAGCAGCTGGACGACCACGCCGATATCGTCTACGTGACCCAACGTCCAACACACTTGTGGCCGCTGTGGCAGAAGGCTCGGGACCTCGTGCTCATGCGCTACTGGCGCCGGGAAGAAGACGGGTCCTACTTTGTCATGTACCAGAGCATCGAGCACCCGGAGTGCCGCGTGCGTCACAACTACGTCCGCGCGAACCTGCTGGGTGGCGGCTTCGTTATCGCCCCTCAGCGTGTTCCTAGT---GGGTCCATTCGCACGCTGGTCACGTACGTGCTCCGATATGATCCGGGAGGCTG---GTCGCGCATCTACCACCAGCTCGGCATGGACGTGGATCTCGTTATGCCCATGCTGCGCAGCGTTGTGGGCATCCGCGACGAGATGAGCGCCACGGACTTCATCACACCCAACGTCTCGGTGGCACCTGCCGGAGAGGGCGGAGGCTGGCCAAGAAGCGTGGA---CTCCATGGGGGTTGTCATGGGCGTGCAGAAGCTTCAA---ACCAGTTTGCCGGAGAAGATGTGGGCCGAGCCGGATGCCTCGCTCTTCAGCGTGCGCGGTCACAACTACCTCAGCGACAAGAAGAAGATCCCGTCCGCGCCGGCAATGTTCCATACCGTGGGCGTGGATCTGCTGTCGTTCGAGAGTGCTGCCGAGAGATACAACATTTCGTCTCGACCGGACAGCATCGGACGCACCAG---CGCCAAGTTCACGTTCGTTGTGAACATGATTATCCCAGGACCGGAGAACACGTGCATGGTGTTCTACTTCCATCCCGTGCGTGACAACGTGTTCGAAGACGGCTCGCCCTTCTCCGAGCTGCTGAACGACTTCTTCGATGGCGACGACCAGTTCCGGAACTCGCGCTTCAAGCTCATTCCTACTGTCGTCGAGGGAAGCTTCATCATCAAGCAGTCGGTAGGCTCCAAGCCTACGCTGCTCGGCAACAAGCTCAAGTGTCCGTACCACCGCGGAGAAAACTACTTCGAGGTGGACATCGACATTTCGTCCAACTCAGTTGCCAACACTGTCGTTGGGATGGTGCAAGGCGTGACCAAGTCACTGGTGGTGGACATGGCCTTCCTGCTCGAGGCCCAGACCGACGAAGAGCTGCCTGAAATTATCCTCGGAGCAGTCCGCATGCAGCACATTTCGCTGGACAATCCCCGCCGAATGCCGAGGCATGGGGAACGGGCAGCAAGGATGAGTTCACCTTCTGAGGCCTTCACCGAAGCTCTGCGCGTGAACCTGCAGCGGCTGATCGTCTACGCGCGCTCGGCGGACACGGCGCTGCAGCGAGAAGTGGCCGAGAAGCTGGCCAACGAGGCCGTCAAGATCGACAGGTGCAGATCGTGGAGCTGGACGGCCTGCAGCTGCTGCTGCCGCTCACCAAGTCCAAGGACACGGAGGTGCAGCGTCTGGCAGCGCACGCACTCGCCAACCTCTCGGTCAACTCGGAGAACCAGTCCAAGATGGCGACGGAGGGAGGCATCGACATGCTCATTGACCTCCTGAGCAGCACGAACGAACACGTGCAGCGGCAGGCGGCGAAGGCGCTCGCCAACCTCGGCGTGAACGTGGACAACAAGGAGCGGATCGCCAAGGCGGGTGGCATTAAACCGCTCATTGACTTGGCCAGCTCCCGCCAAATCGGCGTCGCTGTGGAGGCCATCGCAGCGCTGGCAAATCTGGCTGTCAACG---ATGCAAACGAGGTGGAGATTGCACGCAAAGGCGGATTGAAGCCTATTATTGACGGCGCGCACTCGGAATCCGTGGAGCTCCAGTCCCAAGTAGCGCGTGCACTACGCAACCTATCGGTTAACCCGGAAAACAAGCAAGCGATCGTGGAGCTGGGCGGAGTGGAGGCTTTGCAGTCACTCGTGCGCTCCACCAACGACCGGATATGCCAGCAGGCTACGCGTGCGCTCGTCCCCTGCGTCGTGCTCACGCGCTCGTCCCCCACAATCTGCTG---GCCGTGGCGCCTGTGGCCCGCGTGGCCGACGTCAAGCAGGAGGC------CGCGCCCTACGACGGCGCCATGGTGGACGTCGAGTACTCGGCCACCGTCAAGACGGTC---------GCCGTGCCTGCCGCCGCCGCTGCTGACAACGCGGCCGGC---GGCTTCTCGTCCATCTCCGACCGCTTCCCGCTCTCCAAGGTGGGCAAAGTGAGCTGCGGCATG---CCCATGCCCATGAGCCG---CCTGCTCAATG---CCACGCCCGAGATGACGC---AGGCAGACCAG-------------ATGACGGTGCTCGTGGAGGACGGCACGGTCGACGACAAC---ACGATCAAGGCCGAG---CCCGAGGGTGAGCGACGTGTACCGGTACCAGGCCACGGA--CGTGGAAGAGAACGAGATGGAGCAACTGCGATGGAACATCCAGGAGCAGGTTTTCCAGCGGTACTTCCAGCAAGCACAGAAGTACGAGGAGTGGGGGAAGCTCAAGGAGCAGGACACCGTGTCTTCTTCATCCCAGGCGGACGACGCGAGAACATTCTGGCCGAGAAGCGGTTCCACGACAATGCCGTCCGGGACGCCATGGGGAAGGGAAACGCCACGTGGGTCAAGGAGCGACGGGCGCGATTCGAGGAGGAGATCCGCCAGTTCTAAGAAGAAACACGACACAAGCAGGAGCTGATGCTTACGCGGCTTGAAAAGGTGTCTCCGTTCTCCAAGTTCCCGATCCTGGGCAACCGCTTCTTGCTGCTTCGTCTGCGAGGAAAGGGAGGCAATGGAGAGGTGTGGGACGTCGTGGACTACGCCAACAACAAGCAACTCTGCGCTCTCAAGCTGTCCACTTCCATTCGACACGCCCAACGCGAGCACCACACGCACACTCTACCACCCCA--ACATCGTCCAGGTTGGCGAGGTGCCTTTCCTCATCCGATATCAGCAACAGCAGTACACAGCTTTCACTGTAGCAAGTGTGCAATCTGACCTGCAGCAGCTCATTGAGA---TGTACGAGTACCTCGATGATGACTCGGCCTACAAGGTGCTCTCGCAGCTTCTGAGCTCGCTCAAGTACTTGCACGATGGGTGTTGCTCACTACGACTTGAAGCCGTCCAACATCCTGATCGACCACGATGGGTGTGTGAAGCTG------ACGGACTTTGACTTGACGCGCAACGTCAAATCAGCTACGTCAAACTCTACGGTGGGCACTCTACGCTACCTTCCTCCCGAGTGCTTCCGGCCAAACCGAGGCGA---------CTGCGAAGCCACGGCTGAGAAGGCGGACATGTGGATGGTTGGGATCGTGTACTGCATGATGGTCACTGGAAAGCACCCGATCTGCCCGGACAAGTCCACGCCAGCGGATGTCAAGTCTACGATGGCTCAGTACACGGGC---GAGCTTCGGTACGCACGACCGATCTCCGACTTGTCGCGATGGATCTTACAGGGATGTCTCCACCCGGACCCACGGTGTCGGCCTACTGCCGCTCAGTTACTGGTGGCAATGCAG

Phytophthora_sojae TGGACTACCACCGCCAGGTGGCGCGCGAGCTGGCCGCCTTCTTCCACTCCTTCTACCGCTTCGGCGCCGCCTCCAGCCGCTTCGACCTGTTCCTGCACAACGTGATCTTCGGCTCGCTCTGCATCGGCGCCGTCGTCTTCCTCGTGCTCTGCGCCATCGTCGTGCGGCGCTGCATCCTGCACATCCGCAGGCCTATGTGCGACGTACATCGATGGACTCCACGTCCGTGGAGCTGCTGGCCGTGGGACTGCTGACCTTCCTGGCTATCAGTGCGCTGGGGGGACTGCTGGGAGAGGCCGTCTGGAGACGAGCAGCGCCCTGCGCGTCAATGCTGACGACCTCATCACGTCCTTCAACGACAGCGAGCTACCGGCCGACGCCTTTAGGATGTCGGTGGAGGCGCTGGCCTGGTGCACGCGTCCAAGGAGCTCTGGAACCAGACGGACCTGCTGCTGCCCAAGAACTTTTCGGGGCTCGCCAAGGACTGGGAGTTCAGCTACTTCGTGCTCAAGTCGTCGACCAACGGCGCCATTCTAGCGGTGACGCTGGCCAGCTTCCTGTCCATCGCGTCCGTCGGCTGGGCCATGGTCTCGCCTCTGAGGGTCTCGATCATGGTCATTCTGAGCGTCGTGCCCATTTCGCATACGCTGATCGGCGCGTACCTGTCGTCGACGATCATGACGGCGGACTTCTGTGCCGCCCCGATGAACAACACGCTCAAGCTCGTGGGCTCGTGTCCAGCAAACGTGTCCTGCCCTTTGCTGAGGCGCTGGATACGCAATGCGTCGCACCAGGGAGACATCGGCCAGCGCATGAAGAAGGAGTTCCTGGACCCCATCGGAGCAGCTGTGGGCGTCATGCTCGGCTACTTCGCCGGCACGGTCAGCAACTGGGAGAGCTTCTCGCTCATCCAGACGTGGCGCTGGCCCTTCCTGCTGCAGTTCCTGGCGCTGCTGCCCATCAACGTCGCCATCTTCTTCGTGCCCAAGAAGCACCTCCTTATCCGCAGTGAGGCCACCGAGCTGGAGCCGCCGGTGGCCGGCACGCCCATGAGCACCAGGTCCACGACGTACCACGACGAAGAAGACGCGCTCCTCGCCAGCGCGAGCATGCCACGTACGGCGCTTTGGACACGAAGTCTACGAGCAGGGCGCGTTCTCGGAGGGAGTGTACGAGCTGCTGCACATCCCTGTCTTCTGTTTGATCGTGTTTGGGCTCACGACCGTGTACTTCGTCGTCACGGGCGTGCAGTACTGGAGCACCATCTTCATGATAAAGTCGCTGTATGCGTCCAAGTACCTGGTCAACGGTCTGTTCGTCGTCGTATCTGGAACAGGACCGATTCTAGGCGTGTTCTTCGGTGGCTGGATCATCGACAGATATGGAGGATACATCGGCGTGGAGCAGCGTGCCAAGGCTTTGGGAATCTGCATGATCCTGGGACTGACAGCGTTTTCGATCTCGGCGGTGACGACGTTCTTCGACGACGTTTACATCACTGCGGGGTTCCTGTGGCTGCTGCTCTTCTTCGGCGGCGCCATCCTGCCGGCGCACCGCGCGCTCGCCTCGTCGTTCTCCGTGATGGTGTTCAACCTCTTCGGGTATGCGCTGTCGCATGAGCGACCTGCAGATGGTGGTGGGCGCCAAGTGCTTCGTGCCGGACGAGACGCACGTGTGGCTCGCGGCCGAGGTGCTGCGCGACGAGAACTCGGGCGACGGCAAGACGCGCAACATCTACTGCAAGGTGGAGCTGCCCGACGGAGACCGAGGAGCGATGCGTGGACATGCTGTCCAAGAAGACCAAGGCGCTGCTGCCGTGCACCAGCTCGAGTCGCTGCCCTACCAGAACGAGAACGTCGGGCCCGAGGGCATCGAGGACATGATCACGCTCAACTACCTGCACGAGGCCGCCATCCTGTACAACGTCAAGAAGCGCTTCCTCTGCGAGCTGCCCTACACGTACACGGGCGACATCTGTATCGCCGTGAACCCGTACAAGTGGCTCCCGGACCTCTACGCGGAGGAGCAGCACCTGCGCTACCTCAACCAGCCCAAGGAGGAGCTGCCCCCGCACGTGTACGCCACCTCCGTGGCGGCCTACGACAACATGCGTCGCTCGGAGCGCAACCAGTCCATCCTGGTCAGCGGAGAGTCCGGCGCCGGTAAGACGGAGACCACCAAGATCCTCATGAACCACCTGGCCACCATCGCCGGCGGTCTCAACAACTCGACCATCAAGAGGATTATCGAGGTCAACCCGCTGCTCGAGTCCTTCGGTAACGCCAAGACGGTGCGCAACGACAACTCGAGTCGATTCGGCAAGTTCACACAGCTGCAGTTCGACAAGAACGGCACGCTCGTCGGCGCCAAGTGCCGCACGTACCTGCTCGAGAAGACGCGTGTCATCCAGCACGAAGCGCCCGAGCGCAACTACCACATCTTCTACCAGTGGACT---CGGACATTGCCCCCGAGCTGCAGTTGGAGGCGGGCAAGCACTACGTGTACACGGGCGACAACACGGCGCAGAAGATCGAGGGACTCTCGGACGCCAAACACTTCAACAAGACGCGCGAGGCGCTGGGGCTCGTCGGCCTGTCGCCCGATGACCAGCGGCCGCTGTTTGAGGTGCTGGCTGGTGTGCTGCACCTTGGTGAGGTGCAGTTACAGTCCGACCCGTCTGACGATGAGAAGTCTCTGATCGTTGAAGGCGACACTGGCGCTAACTCTGCTACTCAGATGCTTGGCGTTACCTGGGAGGCCCTTCAGACGGCTCTGTGTTCGCGTACCATGCGCGCTGTGAACGATGTCTACAGCGTGCCTCTTAAGAAGGAGCTGGCCATGGACTGCCGTGACGCTCTTGCCAAGGCTATCTACTCCAATGTGTTCGACTGGCTCGTGGCCACGATTAACCAGTCGCTCGCGGACGACGCGAACATGGCCAACCACGTGGGTGTGCTTGATATCTTCGGTTTCGAGCACTTCAAGCACAACTCGTTCGAGCAGTTCTGCATTAACTACGCCAACGAGAAGCTTCAGCAGAAGTTCACCCAGGACGTGTTCAAGACCGTGCAGATCGAGTACGAGGAGGAAGGCATTGTTTGGGACCACATCGAGTACGCGGATAACCAGGATGTGCTGACCGTGATCGAGTCGCGCATGGGTATCATTTCGCTGCTGAACGAAGAAGTGATGCGTCCGAAGGGAAACGAGGAGTCCTTCATGTCCAAGGTTACTTCCCTGCACAAGGAT---GACATGGCGCACGTCATTGAGTTCCCTCGCACGTCGCGCACCGAGTTCCTCATCAAGCACTACGCCGCTCCGGTCATGTACGATTCGGTGGGTTTCCTGGAGAAGCACAAGGACTCGCTGCTTCCCGATCTCTCCGAGCTCATGCGTGGTTCGTCGAAGCCGTTTATTGCCAAGCTCTTCGAACCGAAGCCCGCGCCTAAGACGCCGGAAGC---------GTGGAGGTGCTCTGTCCATCACGACTGTCGGCACCCAGTTCAAGGAGAGTCTGACGGAACTGATGGCTACGATCAACTCGACGCGTGTGCACTATGTGCGTTGCATTAAGCCGAACCCGATCAA---------GAGTGCGACGGTCATGGACCAGAACATGGTGGTGTCCCAGTTGCGTTGCGCTGGTGTCATTGAAGCCATTCGTATCTCGCGCGCTGCGTACCCGAACCGATTGCAGCACACTGAAATCCTGGACAAGTTCTGGTTGTTCGTGCCAAGTGGCGGTGAGACTGCGGCGGAAAAGTGCCAGCTGCTCATGGACAAGCTGAAGCTTGAGACGCCCGTGCAGTACCAGATGGGTAAGACTCGTGTATACTTCCAGCTTGGTGTCCTTGAAGAGCTTGAGGACCGTCGCAAGAAGTTCTTGGATGCCAAGGCCACGTACCTGCAGAACATCATGCTTGGTTTCACACAGCGTATCAAGTACCTCCGACAACTGGAAGCGATCATCAAGTTCAGTGATCCGGTGCGTGATCGCCATGCGGAGATACAACACGTTCATGAACGGCCTTGTGAAGGCTCAAGCTCACTGGCGAGGCATTCAGGGCCGCAAGGTGGCGGCCGATGTCAAGAGCAACCACAACGCTGTCATCATCCAGCGGTATGTGCGTGGTTTCGTCAAGCGTCACCGCTACGTTAAGATGCGCGCGATGGTTATCCGTGTGCAGGCCGTGGTTCGTATGACGATTCAGCGGCCCAAGTACCTGGCCGCCCTCGAGGAGAAACGCCGTGAGGCTGACATGGCTTATCAGCTGCAGAAGCTCAAGGCAGCTTTGCAAGAGGAACAGGAGCGGAATGCGCAGCT------GCAGCGTC---GTAGTAGTGTAGT--GACGGCCCATCCGCTGCCTCGAGCGAGGTTATGGCTGACGCTGGTGGCATGATCGAGACCCTGACGGGTGAGAACAAGAAGCTGCGCGAGAAGAACGAGGAGATGAAGGTTACGATGAAGGGCTTGAAGGCGGAGATCGAGAAGTTCAAGAGCGACAAGGAGTTCTCGTCTGCTGGCAACCACGTCAAGGTCCGCCAGCTGCAGGACACCGTGCGTGACAAGGAAAAGAAGATATCGCAGCTCGAGGCCGAAAACAAGAAGCTGACGGAGCAGATCGCCAAGCTTCACGCTGAGGGTGTCGT---GCCCGACAAGAAGTCGATCCCGAAGAAGTCGATCTTCCGCACTCTTGGCTCCAAAAAGGAGAAGAAGCCGC---------------GCGAGACCGTGCTGATGGACTCGCTCACGGGCTCGGACGACCTCAACCGCCGGGAGGCGCGGATGTCTGA---GCGCCACTTCCCGCGCATGC---CGAGCATGAGCGTGGGCTCG---CGCTTCTGGAACTCGAATAAG---AAGGGCGAC------------AACGGCGATGGCTTCGACGTGTCGGAGGTTGACGGTGAGGATAGCCCCACGAACCCACGCGCAACACGATCGTGAAGAGCATGGAGGTGGGTGTCACGGGCGCCATGTCGACTCTGAAGGGACGCATG---------TCGGTCGTGAAGGGCATGTACGAAGCAAGCGCACGAACAGGACCGCCCCGTGCGCGAGTCGTTCAACCTGGACGCGCTGCCGGAGGTCTCTCTCCCCGTCGGCTGGGAGGCCAAGGTGTCGCGCTCTACAGGCCGCGTGTACTACGTGAACCGTAAGCTCGGCAAGTCCCAGTTCGAGCGCCCCACGCTGGCCTCGCTCAA------GGCGCAGAAGCTGGCGCGCCAGAAGTCGGCTAACGCGCTACAAGGCGGTGGTGGCGGATTTCGGCTGCGGAGACGCCAGACTGGCGGAGAGCGTGTCGAACAAGGTGCACTCGTTCGATCTGGTGTCCCTCGATATCGGCGTGTATTGCCTGGCGCTTATGGGGACGAGTGTTCGCGAGTATGTGCGCGAGGTGTACCGCGTGCTGAAGCCCGGCGGTGTGTTGAAGGTCGCGGAGGTCAAGAGTCGCTTTGAGAGCGAGTCGCTGGGCGGATGGTGGCGGTGTTTGTGGCGGCGGCGCACGGCGTCGTGGTGACGGTGCGCAACACGACGGTGTTCGTGGAGAGACCAAGTACGCTGTCGTTGTGGGTCAGAAGACGGGCGAGGCCGTGGCCGTGCCGTTCCCCGACGTGGACCGCGACCTCAAGACGCTGCTGGGCCACACCACGTCCATGGTCACCCATGTGGCGGTGAACCTCGTCGCTGCTGCTGACGGCAGATCGCGACGAGAAGATGCGCGTCTCGAGGTTCCCAAACGCCTCCATCATCGAAAGCTACTGCCTGGGACACGCTGCCAGTCTGACCAAGGTGGCGTGCAGCGCCGTCACGCCCGAGCTTGTGGTGTCCACGTCAATGGACAACACGCTCAAGCTGTGGGAGATGAAGACCGGCAAGCTGGCGATTTGCCCCACGACGACATTGTGGCGGTGCTCGTGAACTACCAACACGCGCGTTTCTTCGAGTTCGTGAGAACCCTTACCTGCAGTTGTTCTCGATCTCAAAGGGCCGCTGCCGCATTCAACTGCTGTGCGCGACTTCCGCAAGCTGCAGAACGACCCGCCCTCGGGCGTGTCGGGCGCCCCCATGGACAACAACATCATGCTCTGGCAGGCCGTCATCTTCGGACCGGACGACACTCCCTGGGAAGGCGGCACCTTCAACCTGACGCTCGAGTTCAGCGAGGACTACCCGAACAAGGCGCCGACCGTCAAGTTCATCACTAAAATGTTCCACCCCAACATCTACAACGACGGCCAGATCTGCCTGGACATTCTGCAGAACCAGTGGTCGCCGATCTACGACATCTCGGCTATCCTCACGTCCATCCAGTCGCTGCTGTGCGATCCCAACCCGAACTCGCCGGCCAACTCGGAGGCCGCACGCCTCTTCCAGGAGAACCGGCGGGAGTACAACCGTCGCGTGCGCGAGATCGTCGAGCAGAGCTGGCAGGCGATCGATGGTGTCGGCCGCCAAGATGCGCGGCGCTGAGAACCGCATGAACGCCGGCCGCCCCTTCACGGCGTGGCTGGACAACGTCTCGTCGGACCGCGGTCTGTGCGGCGGCATCAACTCGGGCATCGCTAAGACCACGCGCAAGCAGACCACTCGCAGATCTTCGTGATCGGCGACAAGGCCCGCGCCCAGCTGCGCCGCGACCTCGGCGACAACATCCGCGGCAACGTGACGGAGACGTACGCGTCGCCCCCCAACTTCACGGTGGCCAGCGCCATCGCGGAGGCCGTCGACTCAGAGAGGAGTCGTCGCGTATGGCCGCCATGGAGAACGCCACCACGAACGCCGAGGACCTCACGCTCACGCTCGTCTACAACAAGGCTCGCCAGGCCCGCATTACCACGGAGCTGATTGAAATTATCTCGGGTGCCGCCTCGCTCGACGGTGACTGGCAGCGTGGTGAACGTGCAGAGCGCGGCGCAGTTCGACGAGGCCACGGCGCGCGAGTCGACGCTGTCCGTGACCTTCTTCTGGGCCGAGTTCCAGGCCTGCCGCCCCAACGGGCAGCTGGACGTGGGGCCGCGCCGTGCTGGACAAGCTCGAGGGCGCCAACGTGGCCGAGCTGGCCAAGCGCGTCTGCAGTACCGCCTCAAGAAGCTCATCAGCGCGTCGCCCGTCATGCTCTTCATGAAGGGCAACCCGACGGAGCCCAAGTGCGGCTTCAGCCGGCAGATGGTGGCGCTGCTGAACGAGGACAAGATCCAGTTCGGCACCTTCGACATCCTCAACGACGACGAGGTGCGCCAGGGGCTCAAGCAGTTCTCCAACTGGCCCACGTACCCGCAGCTCTACGTGAACGGCTCGCTGGTCGGCGGCCTGGACATCCTCAAGGAGATGAAGTCGGAGGGCTCGATCGTCGAGCAGCTCGGCGTCGAGGAGGCCGAGGCCGCGTTCCAGGAGAGTCTGCGCGCGCTCGTCAACTCGGCGCCCGTGCTGCTGTTCATGAAGGTTCGACATCCTATGTGGAGTTACCAGCGCCAAGACCAAGACATCGCAAAGGTGGTGACTCTGATGCCAGCATCTTTCCGCCGGGAGATGTGCGGAGGCCATCTCGTGGAGTCCGAAACCGCATGGCTATGGCGTTCCGGGATCCTGTCGATGGAAAGGGTGAGTTTTGCATTGTGCAGCTCCTCGTCTTACCTCGAAACGCACTACCCTGCATCAACGAGGTGTTTCTGCGATCGACTGGCAAGTGGAACGTGCTGCACCGCGACCACCGCAGTGAAGTCCGATCGCTCTGCGTGCACTCGAAGCGCGACTGTTTGTACACGGGTGGCCTGGATGGCCTAATGATTCGATATGATCTGAACAAGGGCCGCGCCCAAACCGTTATCGAGCGCCGCAAGCACATCTCCAAGATCAACGCGGTGCTGGAGCACCCGCACAACCCGAACCTGCTTTTGGTCAGCTGTGTGGAGCAGCAACCACAACTTGTTGCTGCATGATCTCCAGCGCTACGACCCCAACGTGTCGGGTGGTGCCATGAGTCAGTACGTTGTGCCGAGGTGGTCGCCTGCAGGCTATCACGTGTCGTGCGGCAGCAAATGCGCTACTGCCACGGCAGCTTCTCCACGCCCACGTCGGCCACCATCGGGGCCTCGTTCCTGCAGAAGCGCGTGATCGTGGGCGGGAAACTGACGCTGCAGATCTGGGACACCGCCGGCCAGGAGCGCTTCCGCTCCATGGCGCCCATGTACTACCGCAACGCCAAGGCGGCCATCCTGGTCTTCGACCTGCAGAGCGAGGCCAGCTTCGAGAAGATCAAAGAGTGGCTGCACCTGCAGCACCACGTGGGCGACGATATGTGGGCAACAAGAGCGACGTTTCGACTTCTCCAAGGCCCAGCAGTTCGCGGACGAGATCGGCGCGCTGGTATTCCGCACGAGCGCCAAGTCGGGCGAAGGGGTGCAGGCGCTGTTCGAGAGCCTCGCGGTGCAGCTGCTCAAGAAGCACGACCAGGAGCAGCGGACAGCTTCCACGTCGTGTACGTGCCCGCCGATGAGCGCAAGGAGCTGGAGGAGTGGGCGGTGGCGCTGCCCCAGGACAAGGAGGCGCAGCTCGGCTGCCTGACGGAGCGGCTGCGCGCGCACTTCAAGGCGGGGGCCATCCCAGCAGCAGCAGAGACGTTCAAGCAGCAACTGCTCGCGCAGCTGCCCAAGGGCGCCGCCATGACGGACGAGATGCTGCAGATGATGCATGGACAGCCTCGTGGACTCGATCCCGCTCGTGCTCAACTCGCCCGACGCCAAGCACGTTGGAGGTCAAGGGAGACGCGTTCATTGGACGGGTGTTTGATAGACGACTTTGTGCGCATGGACTTCCGCTTGAGTGAAGTGTCGGGTGACGCTCCGTGGGTGCAGCAGGGCAAGCAGCAGAGGTCACGCAGTCGCTGGAGGAGATCCTGGCGCTGGGCCAGGGCCTGGATGACGAGGACTACAAGGCCGAGCTCGAGGTGCTGCAGAACGACCCCAACTCGAACCTCTACTCGGCCGTCACCTTCGAGGAGCTCAACCTGCCGGAGGACCTGCTCAAGGGCGTGTACAACATGAAGTTCGCCAAGCCGTCCAAGATCCAGTCGGTGGCGCTGCCGCTCATCCTCGCCGACCCGCCTGAGAACCTGATCGGCCAGGCGCAGAGTGGCATCCCACCCGTGAGCTGGTGCGCCAGATTATGGCTGTGGTGAACGCCATGGGCAAGTTGTGGACACGTTCCTGGCCATCCCCGGCAACGACCTGGCCCGTGGCGAGACGCTCAAGGCGCAGGTGATCGTGGGCACCCCCGGCAAGGTCGAGGGTATGATCAAGAAGAAGCAGCTGGACACCCGCAACGTCAAGATCTTCGTGCTGGACGAGGCCGACGTCATGGTCGCCGAGGACGGCATGCGCGAGCGCTCCGTGGCGATCAAGCGTCTGATCAAGAACCGCGCGTGCCAGTACCTGCTCTTCTCGGCCACGTACGCTGACGACGTGCGCGACTTCGCCCAGAAGATGGTGCCCGACCACAACATTATCACGGTGAAGAAGGAGAAGCTGACGCTGGACGGCATCAAGCAGTTCTGGATCGACTGCAAGACTCGCGAGAACAAGTTCCAGGTGCTGTCGGACATTTTCGCCATCCTGTCTATCGGCAAGTGCGTCATTTTCGTGCAGCAGCGTGAGACCGCCAAGGAGCTGACGCGTGCCATGCGTGAGAAGGGCCACTCGGTTGGCATCCTGCACGGTGCCGACATGGCCAAGGAGGTGCGTGACCAGGTCATCGACGAGTTCCGCGCCGGCACGACCAACGTGCTGATCACGACAAACGTGCTGGCTCGCGGTATCGATGTCGCGGGTGTGTCTCTTGTGGTGAACTTTGACATCCCCCTTACGCGCGACCACCGCCCCGACCCGGAGACGTACCTGCACCGTATCGGTCGCACGGGTCGTTTCGGCCGCAAGGGCTGCGCCATCAACTTCGTCTACGACAACAAGAGCAAGCAGGACCTGGCCGAGATCGAGGAGTACTACGCTCGTCCTATCACTCAGGCCCCCGCTGACGACATCGAGGAGCTCGAGAAGCTACTGGCTTGGGGGGCAGCAAGCGCCGCCTGGCCAAGCTCGGCAAGGAGCAGCGCAAGGGCATGCGCATCTACCCGCGCGAGCCCAAGAAGAAGCCCTCGGGCAAGGACAAGACGTACTACTACATCAAGGACGTCATGTTCCTGGCGCACGAGCCCGTGCTCGAGAAGTTCCGCGAGTTCAAGACCTTCATGAAGAAAGTCAAGAAGGCCATGGGCAAGAAGGACTACGAGGACGCCAAGCGCAAGCACGAGAACCGCCCCACGTACGAGCTCGACCACATCGTCAAGGAGCGCTACCCGCGCTTCGTGGACGCGCTGGGCGACCTGGACGACGCGCTGTGTGGCAGAACTACGTCGTGGCCACGCGCGCGCTCACCAAGGTGTTCGTGAGCATCAAGGGCATCTACTACCAGGCCAACGTGCAGGGCCAGACCATCACGTGGCTCGTGCCCCACCAGTTCACCCCCACCGTGGACAAGCGCGTGGACGTGCGCGTCATGCTCACGTTCCTCGAGTTCTACGAGGTGCTGCTCAAGTTCGTCAACGCCCAGCTCTACATGCAGATCGGCGTGGCCTACCCGCCCAAGATCAACCTGCAGCTGGATGCCGCCGGTGCGCAGCTCGCGGCGATTGAGCTAGCAGCAGAAGGAGTCCGAGAAGCGTATCAAGCTACAGCGAGCAGCGCAAGGAGCGCAGCGAGAAGAGTCTGTTCAGCGGTCTGACCTTCTTCCTGTCGCGCGAGGTGCCGTCGGCCTGCCTGGAGCTGGTGCTGCGCTCGCACGGCGCTACGCTCGGCTGGGACGGCGCCGGCTCGCCTTTCGACGAGAAGAGCTCGCGTATCACGCACCACATCATGGATCGCCCGCACCAGGGTCACCGTTACTTCAACCGCGAGTACGTGCAACCGCAGTGGGTGTTCGACTCCGTCAACAACGGCATTCTGCTGCCGCTGACTCGCTACCGCAAGGAGCTGGAGAAGCTCAAGTCGGCCTCGCAGGTGCTGCGCGAGGTCGGCGCCGTCGGCGACGAAGACGAGGACGGGAGGCTTACGCCGCTGACCTTAAGGCCGAGATGGACGGCAACAAGAAGTCGAAGCGTGCCAAGAAGGAGGAGGAGGCCGCGGAGCTGAAGGAGCTGGCCAAGACGATGATGTCGAAGAAGGCCAAGCGTCTGTATGACCGCATGCAGTACGGCCTGGGCAAGAAGGCCGACAAGATTCAGAAGCTCGAGGACAAGCGCGCCAAGCTGGAGGCAAGACCAAGAAGCTGCTGGCTGGCGCGCTGTCCGGCATGATCTCGGCGCTGGCCTTCCAGCCTCTGGGGAGCTGCGCTGGCGCGGCACGTCGCCAACGCTCGTGCGCGTGGCGGGCGGCGCCGGCCTCTACTTCGCCACGCTGGACCACTGCCTCAACCTGTTCCCGCAGTCGGCGGTGAACACGTTCCTGGCTGGCGCCTTCGCGCGCACGTTTGCGGGCGGCGTCATGTCGCCGTTCACGATTGTGAAGGCGCGGATGGATGCCCCCGGGCACGTTCGACAGCAAACGGACGTGCCGTTCTCCGGCCTCTACGTGCTCATTTACACGGCACCCGGCCGACGTGGTAAAGACGCGCATGCATGCGGCAAACAGTGGCCAAGATCTACCGCCACGAGGGTCTGCGCGGATTCGCCAAGGGCATCCTTCCGCGTGTAGTCAAGCGCACGCTGTCTACTGCTGTCACATGGACGATATACGAGCTACTCGTTTGACCCGTCGGGTCTGGGTCATGCCAAGGAGGCCTTCAACCTCGCCAAGGAGACCGAGCGCACCAAGCAGATGGAGAACCAGGCCAAGATCAAGGAGAACGAGGCGCTCTACAAGCAGTACGAGATCGTGCGCGTGCAGAAGGAGGGCGAGGAGCGCCGCAAGACGCTCGAGGAGGAGACGCGCCAGCACCAGCAGCGCGCGCAGTACCAGGACCAGCTCAAGCGTAAGCAGTACGCCGACCAGCTGGCTGCCCAGAAGTACATGAAGGAGCAGGAGCTCAAGAAGCAGGAGGAGATCCTCGCTCGCCAGGAGGCTTCGCGCCGCAAGACGCTCGATTATGAGGCCGAGCTGCGTCAGAAGACGGAGCTAGCCAAGGTGGCCGCTGAGACCGAGGGCCGCATCAAGCAGGAGCGTCTCAACCACGACCTGCACCTTGAGGAGGCCCGCGTGCGTGCTAAGGAGTACCGCGAGACTGTCATGGAGGGCATCAAGCTGGCGGGTAACACTGTCGGCTCGGGTATTATGGCCTTCGTCGACGACAAGGAGAAGCTGACGGCTACCGTGGCCTCGCTTACGGCCCTGGCTGTCGGTATTTACACCGCCAAGGTCTCCACTAACGTTGCAGGCAAGTACATTGAGGCGCGCATGGGCAAGCCGTCGCTGGTGCGTGAGACGTCGCGCCGCTCGGCTACTCAGGTGCTGGCCAACCCGATCCCGTCGATCAAGCGTGCGCTTCGCCTGCAGAAGGCGACGGATGCCCTGGAGGGCGTTGTGCTCGAGCCCAAGCTGGACGAGCGTCTGCGCAGCGTGGCCGTGTCCACGTTCAACACGAAGAAGAACCGCGCGCCATTCCGCCACTTGCTGCTGCACGGCCCGCCTGGTACGGGTAAGACACTGTTCGCCAAGGCTCTGGCGCGTCACTCCGGCCTGGAGTACGCCATCCTCACGGGTGGTGATGTCGCCCCGCTGGGCCGCGAGGGTGTGACGGAGATCCACAAGCTATTTGACTGGGCGTCGCACAGCCGTCGCGGTCTGCTGCTGTTCGTCGATGAGGCCGACGCGTTCCTGCAGAAGCGTAGCAACACCGTCATGTCCGAGGACATGCGTAACGCGCTGAACGCCTTCCTGTACCGCACGGGTGAGGCCAGCGACAAGTTCATGATCGTGTTCGCCAGTAACCAGCCTGAGCAGTTCGACTGGGCCATCAACGATCGTATCGATGAGATGGTGGAGTTCCGTCTGCCGGGCTTCGACGAGCGTGTGCGCATGCTCAAGCAGTACTTCGACGACTACATTCGCGCGCCGAAGAACTCGCGTGCCAAGAAGATTTACGCGAGGGCATCATCTTCACTCTGGACCCCGTGGCCAACTTCCTCGTGCCAAGACGCGCATCTTCCAGCTCGAGGCGCTGCAGGGCGTGTCCGCTGAAGCCCAAGCGATCTTCGATGCGCTTAACAAAAGGCCATGCGTATGTACTGGATCATCTTCGGTCTGGTGCTGGCCAACGGCGTGTTCACGTACGTGGCCGGCAAGGACGAGTCGTTCCTGGTGGAGAAGCTCAAGAGAAAGATCCCGTCCGAGTACCGCATCTCGGAGAAGTGGGACAAGTGCCTGGAGAACTTCACGCTTTACTTCGGCGCCGGCCTCGTGGCTGGAGGCCTCACGTCCCTCGTGCTGGCGCGTTCTGGCACGGGTCGTGGACTGATCACGGGTCTGGGCGCTCCGGCGCCGGCTCGAGCTGGACGACGTGCCAGCTGGCGTTCGCGGGGAACGCACAGGCCGCGCTGGACAAGACGGACAAGGCTGTGGGCGACATGCCCGCCTTCCAGCAGCAGACTATCGTGGACTTCGTGTCGGCCTCGGCCACGCAGCAGCAGCTGCAGGCCGTGCACCCGCGGCTTCTGCGAGCGGGCCAACGTGAAGGTCCTGGAGATGTACCTGGCCGAGCAGGTCAAGAACAAGACCGTGGACATTGACGCCAACCTGGCGCTGCTCAAGCTGTACCAGGTGTACCCGGCCACGGCCAATGCCGAGAGCCACGGTGCTGGTAAAGGGCGTCATGGCCCTTCCCTCCACCTTTTTCACGGGCGTCCACGATGATCCCGGAGAACATCCGCGAGGACGCCAACGTGATGGCCGTGCTGCACGCGGGCTTCCTGCTGCAGTCGTGCCTGTTCGAGGACTTCTGGAAGGAGAGCGTGGCCTTCGCCGAGAAGGTGCCCGGTTACCTGGAGTCTGTGCGCGCGTACATCCTGACGGCCATCAGCCGCAGCCACAGCGCCATCTCCCCGGACGTCTTCAAGGCCAAGCTCAACGTGTCCGACAAGGAGGTGGCCGACGTCGTCGCCGAGAACTGGACTCGGCCAACCTGATCCAGGTCAACCCCAACGAGGACAACCAGATGCAGGCCAAGAAGGTCCAGGAGAACATCGAGTTCGAGGACGTGCTCAAGGTCATCCACACCCTCTCTAGATCCAACGTCTGCGCCAGCGGCGGCGGCGTGGCCGACGCCGAGGAGGTCTGCGAGAACCTGGCCAAGCACCTGCGGCTCGAGGGCGGCGCCCAGGTGATCCCGTGCTCCACGGGCGTCATCGGCTGGCGCATCCCCGTGGACTCCATGGTCGAGAACCTGCCCAAGCTGGTGAACAACCTGCAGTCCGATTCGGTGCTGCCCGCTGCTGAGGGCATCATGACCACCGACCTGTACCCCAAGATCCGCTCCATGGACGTGTGCGGCGGCCGCATCGTGGGCATCGCCAAGGGCGCCGGCATGATCGAGCCCAACATGGCTACCATGCTGTCGTACATTCTCACGGACCTGTCGATCCCGCGCGACGTGCTGCGCCAGCTGCTGACCGAGGTGGTGGGCGACACGTACAACTCGCTCAGCGTCGACACGGACCAGAGCACGTCCGACACGGTGACGCTCGTGTCGTCGGACCAGATCCCGTTCGACATGAC---CAAGCTCGACGAGTTCAAGAAGGCGCTGCACGAGGTGTGTCTCGGTCTGAGCGAGGACATCGTGCGCAACGGCGAGGGCGCGCACCACGTCATGCGCGTGCGCGTGCGCGGCGCCATGACCAAGGAGATGGCCAAGGGCGTGGGCAAGTCGGTGGTCAACTCGCCGCTGCTTAAGTGCGCCGTGGCCGGCAACGACCCGAACGTGGGCCGCCTTGTCATGGCTGTGGGCAAGTACATGGGGCTGCACCACAAGGACGTGGACATCCAGCGCCGCATGACGATCCGCATGGGCGGCGTCGTCATCTTCGAGAACGGCGAAATGGTGCTGAACGACGAGATCGAGGCCAAGCTCGTGGCCCACATGCGCGACGCGATGCTGATCGAGCCCACCCGCGGTGGCCTCGACGCCAGCTCGCACAACTACCCGCCGCACGGAAAGTTTGTTGAGATGGAGATTGACCTGGGTGTTGGCTCGGAGGAGACGCAGGTGCTGGGCATCGACCTTACGCACGAGTACGTGGCCATCAACGCCGACTACCGCTCGTAATGGCGCC---GAAAAAGGGGAAGACCGCCGCCGCGTACCTGGCGTCTCTGGGCGTGGCCCCCGC---CGCTGGAGGCGAGAAGAAGGACGAC---AAAAAGAAGAAGAAAAAGGGCAAGAAGAAACCCAAAAGAAGCCCATGTCGGCGGCGGCCAAGGCGCTGA-TGGAGCGCCAGGCCGCCCTCAAGGCCGAGCAGGAGCGCCTCGAGCAGGAGCGTCTGGAGCAGGAGCGCATCGCCCAGGAGGAGGAGGCCAAGCGCCTCGCGGAGCTCGAGCGCGAGGAGGAGATCCGCCGCAAGAAGAAGGAGGCCCGCGCCGCCAAGATCGAGCGCCAGAAGAAGGAGGGCACGTACATGACCAAGGCCCAGAAGCAGAAGGCGGCCAAGGCCCAGGCGGCCCTCGAGGCCATGCAGCAGGCCGGAATCGTGCCCACCGCTGCT------GGCGAGAAGAAGAAGGTCGTGTACTCGGACAACAAGAAGAAGAACAAGAAGAGCAAGAGGAAGAG---GAGGAGGACGAGGCCCCCGACGCGTGGGATGACGAGGACTGGGAGACCAGCGACGTCGTGGCCTCGCTGGAGGACAAGCTAGCAGAAGAAGGAGCAGGAGCGTCTGCGCCAGCAGGGTATTGCGCTCAAGAAGCGTCAGGAGGAGCAGGCTGAGCAGATGCGCCTGGAGGCGGAGGAGGAGCGTCAGCGTTCCAAGGCCAAGATGGAGGCGGAGCGCAAGAAGAAGGAGGCGGCTGAGCGTCGTCGCGAGCGTGAGGCTGCTGCGCGCGCCAACGTGTCCCTGGACAACCTCCGTTCACCTATTTGCTGTATCATGGGTCACGTCGATACCGGTAAGACCAAGCTGCTTGACAACATCCGTAAGACCAAGGTGCAGGACGCGGAGGCTGGTGGTATTACCCAGCAGATCGGTGCGACCTTCTTCCCCGTGGAGGCCATCAAGCAGAAGACCGCTCGCCTCGCCGAGACGATGAAGCTCGAGTACCGCCTGCCCGGTCTGCTTGTGATTGATACCCCTGGTCACGAGTCTTTCACGAACCTGCGTACGCGTGGTTCGTCGCTGTGTGATATCGCCATTCTGGTGGTTGATATCATGCACGGTCTGGAGCCGCAGACGTTGGAGTCGCTGCGCCTGCTGCGCCAGAAGAAAGCTCCGTTCGTGGTTGCTCTTAACAAGATCGATCGTTGCTACGGATGGAAGACGATGCCCGACATGCCCGTCCAGGAGGCGCTCAAGCACCAGAACGAGCACGTCATTCGCGAGTTTGAGGACCGCATGAAGTCCATTATTGTTGAGTTTGCCGAGCAGAAGCTGAACGCCGAGGTCTACTGGCGCAACAAGGACCTGGCGCACACGGTCTCGCTTATTCCCACCAGTGCTATCTCGGGTGAGGGTGTGCCGGATCTGCTCATGATGCTGACGCGTTTGACGCAGGAGCGCATGGCCAAGTCGCTGGCTTTCGTGGATATTCTGCAGTGTACCGTGCTTGAGGTGAAGGTTATCGAGGGTCTCGGTACCACAATTGATACGATCCTGGTGAATGGTACACTTGAGGAAGGCTCGACTATTGTCGTGTGTACATTAGATGGACCTGTGGTGACCACGATTCGGTCCCTGCTGACCCCGCACCCCATGAAGGAAATCCGCGTGAAGGGCGAGTACCTCCACCACCAGAAGATGAAGGCCGCTATGGGTGTCAAGATTTGCGCTCAGGGTCTCGAAAAGGCGGTTGCTGGTACGCAGATTTACGTGGTTGGCCCCGATGACGACGTCGAGGAGCTGAAGGACTCGGTCATGTCCGACCTTACGGGCATCTTGGACTCAGTGAAAGCCACCCGCCGCGGTGTCATGGTGCAGGCTTCGACTCTTGGTGCGCTGGAGGCGCTTCTGGAGTTCTTGCGTACGTGTGACCCGCCTATTCCCGTCTCGTGCGTGAACATCGGACCCGTGCACAAGAAGGACGTTATGCGTGCCTCGGTGCAGCTCGAGCACCAGCCCGAGTTCGCTACCATTCTGGCGTTCGACGTGAAGGTTCACAGCGACGCCACGGAGCTGGCCGCGGACCTTGGCGTGCGCATTTTCACAGCTGACATCATTTACCACCTGTTCGACCAGTTCACGGCGTACATGGACAACTTCCGTCAGGCACGTCGCGAGGAGTTCGCAGAGGTTGCTGTGTTCCCGTGCGTGCTGAAGATCCTGCCCAACTGCGTGTTCAATAAGAAGGACCCGATTATTCTCGGCGTGGACGTTGAAGAGGGCATCCTGAAGGTGGGTACGCCGCTCGTGGTGCCGTCTGCTGGCG---GCTTCCTGGTGGGTAAGGTGGGCTCGATTGAACGTGAGCACAAGGAGGTGGACCGCGCCAAGAAGGGCGCGTCCGTGGCCGTGCGGATAGACAACGAGGGCAGCGTCATGTACGGCCGCCACTTCGACCACAAGAACAAGCTCGTGTCGCGCCTGACCCGCCAGAGCATCGACGCGTTGAAGGAGAATTTCAGGGAAGACCTGCAGAAGGAGGACTGGCAGCTCGTCATCAAGCTCAAGAAGGTGTTCGATATCATTATGGAGGGCTGGCTCACCAAGCGCCGCGACCACATGAACCTCATCTGGCTGGATCGCTACTTCCTGCTGGACGGCAACCAGCTCCGCTACTACCGCAAGCGCGGCGACCCAGCGCCTCGAGGCACCTACATCCTCACGGACGCGTGCGTGGTGAGCCCCGTGTTCAACTCGGAGGAGAAGCACAAGCACCACGGGCCCGTGTGGATGTTCCGCATCACGTGAGAAGTCGTACAAGAAGGACCGGTTCCTGGACCTGGGCGCCAAGAGCGAGGATAACGCCAAGGAGTGGAAGCGCGCGCTGGAGAACGCCGTGCGCTCCATCCGCGAGCTCGTGGCGCACCAGAAGAAGCTCAAGACGGACGCCGAGAT---CTTCCCCACGCCCACGCAC---------GTGCCGCGGCTGCTCAAGACGGACGAGGACACGGTGTCGCTCTTCAATCTGGACGCGGACAACGTCAAGGCCGAGCAGGCCTGGTGGCTGGCCAGGGTGGAGGGAGGCCTGAGGATCATGCAGGAGTGCCCGCTCGGACCCAATGTCGCGGCCAACCTGTACGCGCAGCATGCGGCGACTACCAATGGGCTCATCTTCAGCGCCTGTGTGGGCGCGGCGTTGAGCTTCAGCAGCATGTACTCGTTCGCCAACAATATGCTGCTAAGCTTCATGACGGCGCTCGTGGCGATCGCAGTGTGTTGCGCTGCGGTGACGCCTACGCAAGCTCCGGAGATCCCGAGCTTCCGTGTCTCGCAGGTGGTGCACGGATCTCCGACCGAGGTGTTTCGACTGATCATGAACTCCAAGAGGTTCCAGCGTTGGGACTCGGCCACGGCCACCATGCGCGTGATCCAGCAGCTGGACGACCACGCGGATATCGTTTACGTGACCCAACGGCCAACCCACCTGTGGCCGCTGTGGCAGAAGGCGCGCGACCTGGTGCTCATGCGCTACTGGCGCCGCGAGGAAGACGGATCCTACTTCGTCATGTACCAGAGCATCGAGCACCCGGAGTGTCGTGTGCGTCACAACTACGTGCGCGCGAATCTGCTGGGCGGCGGCTTCGTCATCGCCCCTCAGCGCGTCCCCAGC---GGGTCCATCCGCACGCTGGTCACGTACGTGCTCCGATACGACCCGGGCGGCTG---GTCGCGCATTTACCACCAGCTCGGCATGGACGTGGACCTCGTCATGCCCATGCTGCGCAGCGTTGTGGGCATCCGCGACGAAATGAGCGCCACGGACTTCGTCACGCCCAACGTCTCGGTGGCTCCTGCTGGAGAGGGCGGAGGCTGGCCAGGAAGCGTGGA---CTCCATGGGAGTTGTCATGGGCGTGCAGAAGCTTCAG---ACCAGTCTGCCGGAGAAGATGTGGGCCGAGCCTGATGCGTCGCTCTTCAGCGTGCGCGGCCACAACTACCTCAACGACAAGAAGAAGATCCCGTCGGCGCCGGCAATGTTCCACACGGTGGGCGTGGATCTGCTGTCGTTCGAGAGCGTGGCCGAGCGATACAACATCTCGTCTCGACCGGACAGCATCGGCCGCACCAG---CTCCAAGTTCACGTTCGTCGTCAACATGATCATCCCGGGCCCGGAGAACGTGTGCATGGTGTTCTACTTTCAGCCCGTGCGCGACAACGTGTTCGAGGACGGCTCGCCCTTCTCCGAGCTGCTCAACGACTTCTTCGACGGCGACGACCAGTTCCGAAACTCGCGCTTCAAGCTCATTCCGACGGTCGTCGAGGGAAGCTTCATCATCAAGCAGTCGGTCGGCTCCAAGCCCACGCTGCTCGGCAACAAGCTCAAGTGTCCGTACCACCGTGGAGAGAACTACTTCGAGGTGGACATCGACATTTCGTCCAACTCGGTGGCCAACACTGTCGTCGGCATGGTGCAAGGCGTGACTAAGTCGCTGGTGGTGGACATGGCCTTCCTGCTCGAGGCCCAGACCGACGAAGAGCTGCCGGAAATTATCCTGGGCGCCGTTCGGATGCAACACATTTCGCTGGACAACCCCCGCCGGATGCCGAAGCATGGGGAACGGGCAGCAAGGACGAGTTCACCTTCTGAGGCCTTCACCGAAGCGCTGCGCGTGAACCTGCAGCGGCTCATCGTCTACGCGCGCTCGGCGGACACGGCGCTGCAGCGGGAGGTGGCCGAGAAGCTGGCCAACGAGGCCGTCAAGACCGACAGGTGCAGATTGTGGAGCTGGACGGCTTGCAGCTGCTGCTGCCGCTCACCAAGTCCAAGGACACGGAGGTGCAGCGTCTGGCAGCGCACGCACTCGCCAACCTCTCGGTCAACTCGGAGAACCAGTCCAAGATGGCGACGGAAGGAGGCATCGACATGCTCATCGACCTCCTGGGCAGCACGAACGAGCACGTGCAGCGGCAGGCGGCCAAGGCTCTCGCCAACCTCGGAGTGAACGTGGACAACAAGGAGCGGATCGCCAAGGCGGGCGGCATCAAACCGCTCATTGACTTGGCCAGCTCCCGCCAGATCGGCGTCGCTGTGGAGGCCATCGCAGCGCTCGCGAATCTGGCGGTCAACG---ATGCCAACGAGGTGGAGATTGCACGCAAAGGCGGCTTGAAGCCGATCATTGACGGCGCGCACTCGGAATCCGTCGAGCTGCAGTCCCAAGTAGCCCGTGCACTACGCAACCTATCGGTCAATCCGGAAAACAAGCAAGCGATCGTGGAGCTGGGCGGAGTGGAGGCTTTGCAGTCACTCGTGCGCTCCACCAACGATCGGATATGCCAGCAGGCCACGCGCGCGCTCGTCCCCAACGTGGTGCTAACGCGCTCGTCCCCCACAACCTCCTG---GCCGTGGCGCCCGTGGCCCGCGTGGCCGAGGTCAAGCAGGAGGCGGCCGTGGCGCCCTACGACGGCGCCATGGTGGACGTCGAGTACTCGACCACAGTCAAGACGGTC---------GCCGTGCCCGCCGCCGCAGCCGCCGACGCCGCGGCCGGC---GGCTTCTCGTCCATCTCGGACCGCTTCCCGCTCTCCAAGGTGGGCAAGGTGAGCTGCGGCATGGCCCCCATCCCCATGAGCCG---CCTGCTCAACG---CCACGCCCGAGATGACGC---AGGTGGACCAG-------------ATGACGGTGCTCGTCGAGGACGGCACGGTCGATGACAAC---ACGATCAAGGCCGAG---CCCGAAGGTGAGCGACGTGTACCGGTACCAGGCCATGGA--CGTGGAAGAAAACGAGATGGAGCAACTGCGATGGAACATCCAGGAGCAGGTTTTCCAGCGCTACTTCCAGCAAGGCCAGAAGTACGAAGAGTGGGGCAAGCTCGAGGAGCAGGACAGCGAGTCTTCTTCATCCCAGGCGGACGACGCGAGAACATCCTGGCCGAGAAACGGTTCCACGACAACGCCGTGCGGGACGCCATGGGCAAGGGAAACGCCACGTGGGTCAAGGAGCGACGAGCTCGCTTCGAGGAGGAGATCCGCCTGTTCTAAGAAGAAACTCGGCACAAGCAGGAGCTGATGCTTACGCGGCTTGAAAAGGTGTCGCCGTTCTCCAAGTTCCCGATTCTGGGCAACCGCTTTCTGCTGCTTCGTCTGCGAGGAAAAGGAGGCAACGGAGAGGTGTGGGACGTCGTTGACTACGCCAACAACAAGCAGCTCTGCGCCCTTAAGCTCTCCACCTCAATCCGACACGCCCAACGCGAGCACCACACGCACACTCAACCATCCCA--ACATCGTCCAGGTTGGCGAGGTGCCCTTCCTCATCCGGTACCAGCAGCAACAGTACACCGCTTTCACTGTCGCAAGCGTTCAGTCTGACCTCCAGCAGCTCATTGAGA---TGTACGAGTACCTGGATGATGACTCGGCCTACAAGGTGCTCTCGCAGCTCCTGAGCGCGCTCAAGTACTTGCACGATTGGCGTGGCTCACTATGACTTGAAGCCGTCCAACATCCTGATCGACCATGACGGGTGTGTGAAGCTG------ACCGACTTTGACTTGACGCGCAACGCCAAATCAGCCACTTCGAACTCGACGGTGGGCACTTTGCGCTACCTCCCGCCCGAGTGTTTCCGCCCGAACCGGGGCGA---------TTGCGAGGCCACTGCGGAGAAGGCGGATATGTGGATGGTTGGGATCGTGTACTGCATGATGGTCACTGGAAAGCACCCGATCTGCCCCGACAAGTCCACGCCAGCGGAAGTCAAGGCTACGATGGCTCAGTACACGGGT---GAGCTTCGGTACGCACGACCGATTTCGGACCTGTCCCGATGGATTTTACAGGGATGCCTCCACCCGGACCCACGGTGTCGGCCGACGGCCGCTCAGTTGCTGGTGGCGTTGCAG

Plasmopara_halstedii TAGACTACCGCCGTCAAGTGGCAAAAGAACTGGCTGCCTTTTTCCACACTTTTTACCGCTTTGGTGCTGCTTCTAATAAATTCAATTTATTCCTACACAATGTTCTCTTGGGCTCCATGTGTATCGGAGCCGTCGTCTTCCTCGTACTAAGCGTAATTGTTGTGCGCCGCACCGTTCTGCATATTCGAAGGCATTTGTTCGACGCACATCGATGGATTCCACGTCTGCAGAGTTAATGGCAGTTGGATTGTTAACATTTTTGGCTATGAGTGCCTTAGGTGGACTCCTAGGTGAAGCTGTCTCCAGACTTGCAACGCTTTGCGTCTAAATGCTGACAACATGATCACGTCATTCAACGACAGTCAATTGCCACCTGATGCCTTTCAGATGTCAGTCGAAGCATTAGACTCGTGCACGCTTCAAAGGACTTGTGGAACCAAACGGATGCGCTTATGCCCAAAAACTACTCGAATATAGCCAAGAATTGGGAATTCAGCTACTTTGTTCTCAAATCGTCGACTAATGGTGCCATTTTGGCTGTAGCACTCGCCAGTTTTCTCTCAATTGCGTCTGTTGGATGGGCCATGGTGTCGCCACTACGTGTTTCTATCTTGATCATCCTGAGTGTTGTACCCATCTCCCATACACTCATTGGAGCCTATTTGTCGTCAACGCTCATGACGGCAGATTTTTGTGCTGCGCCAATGAACAACACTTTGGGACTGATTGGCTTGTGTCCCGCAAATGCATCTTACCTTTTGCCGACGCGATGGCAACGCAATATGACGCACCAGGGAGAGATTGGTCAACGTATAAAGCGCGAGTTTCTAGATCCTATTGGAGCAATTGTGGGTGTAATGTTTGGGTACTTTGCAGGCACAGTGAGCAATTGGGAAACGTTTGCACTCATTGAAACATGGCGATGGCCATTTCTACTTCAATTTGTCGCGTTATTACCTATGAATGTTGCAATTTTCTTTGTACCACAGCGACATTTGATCATTCGAAGTGAGAGTAGTGAAGTGGAGCCGC----AGTTGGTACTCCAATCAGTACTCGATCGACAACGTATCACGACGAAGAAGACGCTTTGCTAGCTAGTGCTAGTATGCAACGTACGGTGCTTTGGATACGAAGTGTACGAACAGGGTGCGTTTTCAGAAGGAGTGTACGAATTGCTACATATTCCCGTCTTCTGTCTGATTGTCTTTGGCCTCACGACCGTCTACTTTGTCGTCACGGGGGTTCAATATTGGAGCACGATCTTTATGATCAAATCTCTCCATGCGTCCAAGTATTTAGTAAATGGACTCTTTGTCATCGTGTCAGGAACTGGACCAATTCTAGGTGTGTTCTACGGTGGCTGGCTCATTGATCGATACGGTGGATACATTGGTGTTGAACAGCGTGCCAAAGCCCTTGGACTTTGTATGATCCTAGGACTCACTGCTTTTGCTATCTCTGCAGTGACGACTTTCTTCAATGACATTTACGTCACTGCAGGGTTTCTTTGGCTTTTGCTCTTCTTTGGTGGTGCAATTCTACCAGCACATCGCGCTCTTGCCTCGTCATTTTCTGTCATGATTTTTAATCTGTTTGGCTATGCCTTGTCTCATGAGTGACTTACAGATGGTGACGGGCGCAAAATGTTTCGTACCGGACGAAACACACGTGTGGCTTGCGGCTGAAGTGCTTCGTGACGAGAATTCGGGTGACGGCAAGACGCGCAAAATCTATTGCCGGGTAGAGTTACCCGACGGCAATCGAGGAGCGCTGTGTAGATATGCTGGCAAAAAAAACCAAGGCACTGCTGATGTGCACCAACTTGAATCTCTTCCCTATCAGAACGAAAACGTCGGACGTGAGGGAATCGAGGACATGATCACACTTAATTACTTGCATGAGGCCGCCATTTTGTACAATGTTAAAAAGCGCTTTCTTTGTGAGCTTCCTTACACGTACACAGGGGATATTTGTATCGCCGTTAATCCGTACAAATGGTTACCGGATCTTTACGCTGAAAAGCAGCATCTGCGGTACCTTAACCAGCCTAAAGAGGAACTTCCGCCACACGTGTATGCCACATCCGTTGCTGCTTATGACAATATGCGGCGCTCGGAAAAAAATCAATCCATCCTTGTTAGTGGTGAGTCCGGTGCTGGTAAAACAGAGACGACTAAGATCCTCATGAACCATTTGGCTACAATCGCGGGTGGTCTCAACAACTCAACTATAAAGAAAATTATAGAAGTCAATCCGCTGCTCGAATCCTTCGGAAATGCCAAGACTGTCCGCAATGACAACTCGAGCCGATTTGGCAAGTTTACACAGTTGCAATTTGATGAAAATGGAACTCTTGTTGGCGCTAAATGTCGCACGTATCTCCTGGAAAAGACGCGAGTAATTCATCACGAAGCACCTGAGCGCAACTACCACATTTTTTATCAGTTGACTCTTCTGACGTTTCATCAGAGCTACAGCTCGAATCGTCCAAAATTTATTCGTATACGGGCGACAATAGAGACCAAAAAATTGAAGGTCTGTCAGACGAGAAGCACTTTAACAAGACAAGAGAAGCTCTTGAGCTCGTGGGCTTGTCTCGTGATGACCAACAACCACTATTTGAAGTGTTGGCTGGTGTGCTTCACCTTGGTGAAGTGCAACTGCAATCGGATCCTACTGATGACGAAAAGTCGCTGATTCCCGAAAATGACGGTGGGGCTAATTCTGCAACGCACATGCTTGGTGTCACATGGGAAGCTCTTCAAACGGCACTTTGCTCCCGTACCATGCGTGCGGCCAATGATGTGTACAGTGTGCCGCTAAAGAAAGAGTTGGCGATGGACTGTCGTGATGCCCTAGCCAAGGCTATTTACTCGAATGTATTTGATTGGCTTGTGAATACAATCAACCAGTCATTAGCTGATGATGGCCATATGGCCAATCACGTGGGTGTGCTCGACATTTTCGGGTTCGAACATTTCAAACACAATTCATTCGAACAATTTTGTATCAATTTTGCCAACGAGAAGTTACAACAAAAATTCACTCAAGATGTCTTCAAGACCGTGCAGATCGAGTACGAGGAGGAAGGAATTGTGTGGGACCACATCGAGTACGCCGATAATCAGGATGTACTGAACGTAATCGAGTCGCGGATGGGTATAATATCACTGCTAAACGAGGAAGTTATGCGACCAAAGGGTAATGAGGAGTCGTTTATGTCCAAAGTAACATCATTGCACAAGGAT---GA---AACGCATGTCATCGAATTCCCTCGCACGTCTCGCACCGAGTTCCTCATCAAGCACTATGCTGCTCCTGTGATGTATGACTCGGTGGGATTTCTTGAGAAGCACAAAGACTCACTTCTGCCGGATTTATCTGAGCTCATGCGTGGATCGTCCAAGCCGTTTATCGCTAAACTTTTCGAGTCCAAGCCAGAGCCAAGTCCGCCGAAAGC---------GGGGTGGAGCTTTGTCAATAACGACTGTTGGTACTCAATTCAAAGAGAGCTTGACAGAACTGATGGCTACAATCAATTCGACACGTGTGCACTACGTGCGTTGTATCAAGCCTAACCCGATTAA---------AAGCGCAACTGTGATGGATCAGAGCATGGTAGTGTCTCAATTGCGATGCGCTGGTGTTATTGAAGCCATTCGCATCTCGCGTGCGGCGTACCCAAACCGGTTGCAGCACACCGAGATTCTGGACAAATTCTGGCTGTTTGTACCGAGTGGTGGCCACACAGTTGAAGAAAAGTGTCAATTGCTTATGGACAAACTAAAGCTTGAATCCCCTCGTCAATATCAAATGGGTAAAACCCGCGTGTACTTCCAACTGGGAGTGCTTGAAGAGCTTGAAGATCGCCGCAAAAAGTTCTTAGATGCTAAGGCCACTTACCTGCAATACACCATGCTTGGCTTTACGCAGCGCATCAAGTATCTTCGAAAACTTGAGGCAATCATAAAGTTCGGTCATCCGATGCGTGATTGCAATGCGCAGATACAACATGGTTCTCAAAAATATCATCGTGGTGCAAGCTCGCTGGCGAGGCGTCCAAGGCCGCAAGGTGGCTGCCGACATAAAGAGTAATCACTACGCTGTTATTATACAGCGATATGTGCGTGGTTTTGTGAAGCGTCATCGCTACGTTAAGATGCGCGCGATGGTCATTCGCGTGCAGGCCCTGGTTCGCATGACAATCCAGCGGCCAAAATATTTGGCTGCTCTCGATGAAAAGCGCCGTGAGGCAGATATGGCGTACCAGTTGCATAAGCTTAAGGCTGCCCTACAAGACGAGCAAGAACGTAATGCTCAACT------GCAGCGTC---GCAACAGCTTGAC--GACAGCGCTTCCGCTGCCTCGAGTGTTGTTATGGCTGATGCCGGTGGTATGATCGAAACTCTCACAGACGAGAACAAGAAACTTCGTGAGAAAAACGAGGATATGAAGGCTGCGATGAAGGGTATGAAAGCGGAAATTGAGAAGTTCAAGAATGACAAGGAGGTCTCCTCGGCTGGCAACCATGTGAAGGTCCGTCAGCTACAGGACACTGTGCGTGACAAGGACAAGAAAATTGCTCAGCTTGAGGCTGAAAACACTAAGCTGACAGAACAGATTGCTAAGCTTCACGCGGAAGGTATTGT---TCCCGACAAAAAATCCATTCCGAAAAAGTCGATCTTCCGTACACTCGGTTCAAAGAAGGAAAAGAAGCCAC---------------GTGAAACAGTTTTAATGGACTCGCTGACTGGATCAGACGACCTTAACCGTCAAGAAACGCGTCCGTCGGA---GCGCCACTTCCCGCGTATGC---CGAGCATGAGTGTGGGAAGC---CGTTTCTGGAACTCAAATAAA---AAAGGGGAC------------AACGGTGATGGCCTAGACGTTTCTGAGGTCGAAGGTGAAGATAGTCCAACTAACCTACGCGCAACACGATCGTAAAAAGCATGGAAGTTGGGGTGACTGGAGCTATGTCGAGTCTGAAAGGCCGTATG---------TCGGTAGTGAAGGGAATGTACGAAACAAGCGAACAAACAGGATCGCCCTGTCCGCGAGTCATTTAATCTGGATGCGTTGCCTGAGGTGTCATTGCCTGTCGGCTGGGAGGCAAAAGTTTCTCGTTCCACAGGCCGTGTGTACTACGTGAATCGCAAGCTTGGCAAATCGCAATTCGAGCGCCCCACACTGGCCTCACTCAA------GGCACAGAAGCTGGCGCGCCAGAAATCGGGTCCCGCGCCATGAAGCGGTAGTTGCTGATTTTGGCTGTGGAGATGCCAGACTGGCTGAAAGTGTACCAAATAAGGTGCACTCATTCGATTTGGTGTCAATGGACATTGCCATCTACTGTTTGGCGCTTATGGGAACAAGTATTCGTGAGTATTTACTTGAAGTGCACCGTGTGCTGCGTCCTAAAGGTGTGCTGAAGGTCGCTGAAGTCAAAAGCCGCTTTGAAACCGAGTCACTAGGAGGATGGTAGCCGTCTTTACGGCCGCAACAAATGGTGTCGTTGTCTCGGTCAGAAACACGACAGTGCTTGTGGAGATACCAAGTACGTGGTTATTATAGGCCAAAAAACGGGTGACGTCGTCGCCGTGCCTTTTCCAGACATGGACCGTGACCTAAAAACGCTGTTGGGCCACACCACATCAATGGTCACTCACATGGCAGTCAATTTCGACATTACTTTTAACGGCAGACCGTGACGAAAAGATTCGTATTTCACGATTTCCGAATACATCAATTATCGAAAGTTACTGCTTGGGACACGTTGCAAGTCTCACTAAAATAGCTTGTAGCACGTTGACCCCTGAACTGGTGGTTTCGTCGTCTATGGACAACACTATAAAGTTATGGAAGATAAAGACAGGAGAGTTGACTATTTGTCCAAAAACGACATTGTAGCTGTGCTCGTGGACTCCCAGCTTGTGTTATTTTTTGAAATCGTTAGCATCCATTTCTGCAGCTTTTTAAATTATCAAAGAGGAACTTCTGCTTTCGACCGCTGTGCGCGATTTCCGCAAGCTTCAGAATGATCCTCCGTCAGGTGTGTCGGGTGCCCCCATGGACAACAACATTATGCTTTGGCAAGCTGTTATTTTTGGACCAGATGACACACCATGGGAAGGAGGTACCTTTAATCTGACGCTTGAATTCAGTGAGGATTATCCAAACAAGGCGCCAACTGTCAAGTTTATCACAAAAATGTTCCATCCCAACATTTATAACGATGGTCAAATCTGTCTGGACATTTTACAGAACCAGTGGTCGCCAATATACGACATTTCGGCCATCTTGACGTCTATTCAGTCGTTGCTGTGCGACCCGAACCCAAATTCACCGGCGAACTCAGAAGCTGCACGCCTATTTCAGGAGAACAGACGGGAATACAATCGTCGCGTGCGTGAGATCGTCGAGCAAAGTTGGCAGGCGATTGATGGTATCGGCCGCGAAGATGCGTGGCGCAGAAAACCGCATGAATGCAGGGCGCCCTTTCACTGCGTGGATAGATAACGTTTCGTCAGACCGTGGTCTTTGCGGTGGTATCAATTCCGGTATTGCCAAGACCACTCGCAAGCAGATCACACACAGATTTTCGTAATTGGTGACAAGGCTCGCGCTCAGTTGCGCCGCGATCTTGCCAGTAACATTCGTGGCAATGTCACAGAAACATACCAATCACCTCCCAACTTCACGGTTGCCAGTGCGATTGCTGAAGCCATGGAATCGGAGAAGAGTCTTCTCGTATGGCTGCTATGGAAAACGCGACTACTAATGCTGAGGACCTCACGCTCACGCTCGTATACAACAAGGCCCGTCAAGCTCGCATTACTACTGAGTTAATTGAAATTATCTCGGGTGCTGCGTCGTTGGACTGTGACTGGAAGTGTCGTGATCGTGCAGAGCATAGCGCAGTTTGACGAGGCGACGGCGCGCGAGTCAACAATATCTGTGACATTTTTCTGGGCTGATTTCCATGCTTGTCGTCCTAATGGACAGCTCGACGTGGGGTCGTTCTGTACTGGATAAGCTTGAAAATGCTAATGTGGCGGATCTGGCGAAACGCGTCTACAGTATCGTTTACAAAAGCTTATTAGTGCATCTCCTGTTATGCTCTTTATGAAAGGCAATCCGAGTGAGCCTAAGTGCGGCTTTAGTCGTCAAATTGTAGCACTATTGAATGACGAAAAGATTCAATTTGGTTCATTTGACATTCTTAATGACGATCAAGTGCGTCAAGGATTAAAGGAATATTCAAACTGGCCGACATATCCACAGCTATATGTAAATGGTAAACTTGTTGGTGGATTGGATATATTGAACGAAATGAAGTCGGAAGGATCGATTGCTGAACAGTTAAATGTAGAGGAAGCGGAGGCTGCGTTTGAAGAAGGGTTACGTGCACTTGTGAACTCAGCGCCTGTTCTGCTTTTTATGAAAGTTTGACATTTTATGTGGAGCTACCAACGTCAGGATCAAGACATTTCAAAGGTGGTGACTCTCACGCCACCTTCGTTTCGCCGAGATATGTGCCGAGTCTATCGCGTGGAGCCCGAAATCGTATGGCCATGGCGTTTCGAGATCCTGTTGAGGAAAAAGGAGAATTTTGCGTTGTGCAGCTCCCCGCATCACGTCGAAGTGCACAACGTTGCACTCACGAGGAATTTCTGCCATCGACTGGCAAGTTTAATGTCTTGCATCGGGATCATCGCAGTGAGGTGAAGTCGCTCTGTGTTCACTCGAAGCGTGAGTGTTTGTTTACCGGCGGTCTTGACGGTTTACTTATCCGATATGACCTGAACAAGGGGAGCAGTAGCATTGTGATGGAACGTCGAAAGCATATTTCTAAAATAAATTCGATACTGGAACATCCACACAATCCAAACATGCTTTTGGTCAGCTCGGTGGAACAGTAGACACGATCTGTTGCTTTATGACCTGCAGCGTTATGACCATACGATGTCGGGCACCTCGATGAGTCAGTACGTCGTGCCAAGGTGGTCGCCTGCCGGTTATCATGTTTCTTGTGGCAGCAAATGCGTTACTGTCATGGCAGCTTTTCGACGCCCACATCTGCTACGATTGGGGCCTCATTCTTGCAGAAGCGCGTAATTGTTGGAGGGAAATTAACTCTTCAAATTTGGGACACAGCTGGCCAGGAGCGCTTTCGGTCCATGGCGCCGATGTATTACCGTAACGCTAAAGCGGCTATTCTGGTGTTTGACCTTCAGAACGAGGCCAGCTTTGAAAAGATTAAAGAGTGGCTTCACCTACAACATCATGTGGGTGATGACATGTAGGCAATAAGAGCGACGTTTTGATTTTTCAAAAGCTCAGCAGTTTGCAGATGAAATTGGTGCGCTGGTCTTTCGTACTAGTGCCAAAACGGGCAAAGGGATACAGTCGCTTTTTGAAAGTTTGGCGGTGCAGCTTTTGAAAAAACATGATCAAGACCAACGAACTCGTTCTTCGTGGTATACATACCGGCAGATGAAGCCAAGGGCCTAGAAGAGTGGACTGTTAAGCTTCCGCAAGACAAAAAGGCACAACTAGGATGTCTCACTGAGCGTCTCCGTGACCATTTCAAGAGTAGTGCTATCCAAGCTGCAACAGAGACGTTCAAGCAGCAATTGCTGACTCAACTGCCCAAAGGTGCCACACTGACGGATGAGATGCTACAGATGATGTATGGATAGTCTTGTGGATGCGATCCCGCTTGTATTAAATACACCAGATGTTAAGCACGTTGGAGGTAAAAGGCGACGCGTTTATCGCACGTGTATTTGACAGATGACTTTGTGCGCATGGACTTTCGGTTAAGTGAAGTGTCGGGTGATGCTCCATGGGTAGAAATGGCTAAGTTGCAGAGGTCACACAGTCACTTGAAGATATATTGGCACTTGGCCAAGGTCTAGACGACGAGGACTACAAAGCTGAACTTGAGGTTTTGCAGAACGACCCCAACTCGAATCTCTATTCTGCTGTTACCTTTGAGGAACTGAATCTTCCGGAACAGCTTCTGAAAGGCGTGTATAACATGAAATTTGCCAAGCCATCCAAGATTCAATCAGTGGCGCTTCCACTTATTCTCGCCAATCCTCCCGAAAACCTTATTGGCCAGGCACAGAGTGGTATCCGACTCGGGAATTAGTGCGACAGATCATGGCTGTGGTAAACGCTATGGGAAAATTGTGGACACTTTTCTAGCCATTCCAGGCAACGATTTAGGTCGTGGTGAGAGTTTAAAGGCACAGATTGTCGTTGGTACTCCTGGCAAAGTTGAGGGCATTATCAAGAAAAAACAGTTAGATACTCGTAATGTCAAGATTTTTGTGCTGGATGAAGCGGATGTGATGGTTGCTGAAGACGGTATGCGCGAGCGTTCAGTAGCAATCAAACGTCTGATTAAATCCCGTGCGTGTCAGTATCTGCTCTTCTCGGCCACTTACGCTGACGATGTGCGCGACTTTGCTCAAAAAATGGTGCCTGATCACAACATTATAACGGTCAAGAAGGAAAAGCTCACGCTGGACGGTATTAAACAGTTCTGGGTCGACTGTAAGTCTCGCGAAAATAAGTTTGAGGTGCTGTCGGATATTTTCGCTATCTTGAGCATTGGAAAGTGTGTCATTTTTGTCCAGCAGCGGGAAACAGCCAAAGAACTAACGCGTCGTATGCGTGACAAGGGCCATTCTGTTGGGATCTTGCATGGTGCCGATATGGCAAAAGAAGTGCGTGACCAGGTGATCGACGAATTTCGTGCCGGCACAACAAATGTGCTGATCACAACAAACGTACTTGCTCGTGGAATCGATGTGGCAGGTGTTTCTCTTGTTGTCAATTTTGACATTCCGCTTACTCGTGATCAACGGCCAGATCCAGAGACGTATTTGCACCGTATTGGCCGAACGGGTCGTTTTGGTCGCAAAGGGTGCGCAATTAATTTTGTACACGACAACAAGAGCAAGCAGGATCTAGCTGAGATTGAAGAGTACTATGCACGTCCCATTACCCAGGCTCCTGCAGACGACATTGAGGAACTCGAAAAGTTGTTAGCGTGGGGGGTAGTAAGCGCCGTTTAGCCAAACTTGGCAAGGAACAGCGCAAAGGCATGCGCATTTATCCTCGTGAACCAAAGAAAAAACCTTCTGGCAAGGACAAGACGTATTACTACTTGAAAGACATCATGTACTTGGCTCATGAGCCTGTTTTAGATAAATTTCGAGCTTTCAAGACATTTATGAAAAAGATCACGCGAGCGATGGGAAAAAAAGACTATGAAGGTGCTAAACGAAAGCATGAAAATCGTCCGACATATGAATTGGACCATATTGTGAAAGAGCGGTATCCTCGCTTTGTTGATGCTTTGAGTGACTTGGATGATGCCTTGTGTGGCAGAATTTTATTGTGGCAACAAGAGCATTAACAAAAGTTTTTGTAAGTATCAAGGGTATTTACTATCAAGCCAATGTTCAGGGTCAAACCATCACATGGCTTGTACCGCATCAATTTACACCCACGGTGGATAAACGTGTAGATGTGCGAGTGATGCTCACGTTTCTTGAGTTTTATGAAATTTTACTCAAGTTTGTAAATGCTCAGTTGTATATGCATATTGGTGTAGCATATCCACCCAAAATTAATTTGCAATTGGATGCTGCTGGTGCTCAATTGGCTGCTTTGGAGCTAACAACAGAAAGAGTCCGAAAAACGGATCAAATCACAGCGATGAGCAAAAAGAACGCAGTGAAAAAAGTCTGTTTAGTGGACTCACTTTCTTTTTATCACGCGAGGTGCCCTCAGCTTGTCTGGAGTTGGTACTTCGCTCTCATGGTGCTCAGTTGGGCTGGGATGGTGCTGGGTCTCCATTTGATGAGAAGGATCCACGTATTACGCACCACATAATGGATCGTCCACATCAAGGCCATCGATACTTTAATCGAGAGTATGTACAACCTCAGTGGGTTTTCGATTCGGTGAACAATGGCATTCTGTTGCCATTGACGCGCTATCGCAAAGAATTGGAGAAACTCAAATCAGCGTCGCAAGTTTTACGTGAAGTAGGAAAAATCGAAAATGAGGTTGAAGACAAGAAACTTATGCCGCTGATCTCAAGGCCGAAATTAATGGCAACAAGAAGTCGAAACGTGTCAAGAAGGAAGAAGAAGCAGCAGAATTAAAAGAACTAGCTAAGACGATGATGTCCAAGAAGGCCAAACGACTGTATGACCGCATGCAGTACGGACTTGGCAAAAAGGCCGATAAGATCCAGAAGCTTGAGGACAAACGTCTCAAAATTGCAATAAGACGAAAAAGTTGCTGGCAGGTGCGATCTCGGGAATGATATCAGCGTTGGCATTTCAACCCTTGGGGCGCTTCGGTGGCGGGGCACGTCACCCACTCTCGTTCGCGTGGCTGGCGGCGCCGGGCTCTACTTTGCTACGCTGGATTACTGCCTTAATATGTTTCCTCCGACTGCAGTCAACACATTTTTGGCCGGAGCCTTTGCGCGTACGTTTGCAGGCGGTTTAATGTCACCATTCACTATTATCAAGGCGCGGATGGATGCCGCCTCGTACCTTCAACGGCAAATTGACGTACCATTTTCTGGACTGTATGTTCTTATTTACACTGCATCCGGCTGACGTGGTCAAGACACGCATGCATGCGACAGACGGTATCCAAGATCTATCGTCACGAGGGCTTACGCGGCTTTGCTAAGGGCATCATACCGCGTGTTGTCAAGCGAACGCTGTCTACTGCAGTCACATGGACAATCTACGAGCTATTCGTTTGACCCGTCGGGCTTGGGCCACGCCAAGGACGCTTTTAGCCTGGCAAAAGAAACTGAACGCACGAAACAGTTAGAGAATCAGGCAAAGATTAAGGAGAATGAGGCACTCTACAAGCAATACGAGATTGTACGCGTGCAGAAGGAGGGTGAGGAACGTCGCAAGACACTAGAGGAAGAAACGCGTCAACACCAGCAGCGTGCACAGTACCAGGACCAGCTTAAGCGCAAACAGTATGCCGATCAACTCGCTGCGCAGAAATACATGAAGGAGCAGGAGTTAAAAAAGCAGGAGGAGATCTTAGGACGTCAGGAAGCCTCGCGTCGCAAAACACTTGACTATGAGGCTGAGCTGCGGCAAAAAACTGAACTAGCCAAAGTTGCAGCCGAAACTGAGGGACGCATCAAACAAGAGCGTCTAAACCACGATTTACACCTTGAGGAGGCCCGTGTGCGTGCTAAGGAGTACCGTGAGACAGTACTGGAAAGTATCAAGCTAGCTGGTAACACTGTTGGCTCGGGTATCATGACCTTTGTCGACGACAAAGAAAAGCTCACGGCAACTGTCGCAACTTTGACGGCTTTGGCTCTTGGTATCTACACCGCTAAGGTGTCCACTAATGTTGCGGGCAGATACATCGAAGCACGTATGGGCAAACCTTCTCTGGTTCGTGAGACGTCGCGTCGCTCAGCCGTACAGGTAGTGGCCAACCCCATTCCGTCCATTAAGCGTGCTTTGCGTCTTCAGAAAGCAACAGATGCCCTCGAGGGAGTCGTACTTGAGCCCAAATTAAATGAACGCCTGCGCAGCGTGGCTCTGTCCACGTTTCACACAAAAGCTAATCGGGCACCCTTTCGCCACTTGCTGCTGCACGGCCCCCCCGGTACAGGAAAAACACTTTTTGCAAAGGCATTGGCCCGTCACTCTGGCCTCGAGTACGCCATTCTCACGGGTGGTGACGTTGCTCCGCTTGGTCGCGCAGGTGTGACGGAAATTCATAAGCTTTTTGATTGGGCGTCGCAAAGTCGTCGGGGTTTGCTTCTTTTCGTTGACGAGGCTGATGCGTTCCTCCAAAAACGCAGCAACACCGTCATGAGCGAAGATATGCGTAATGCACTTAATGCGTTTCTTTATCGCACGGGCGAGGCTAGTGACAAGTTTATGATCGTGTTCGCAAGTAATCAGCCAGAGCAATTTGACTGGGCCATCAACGATCGTATAGATGAAATTGTAGAATTTCGACTGCCTGGTTATGATGAGCGTGTGCGCATGCTCAAACAGTATTTCGACGAATACATACGTGAACCAAAAAATTCTCGTGCTAAAAAGATTTACGTGAAGGCACTGTATTTACGCTGGATCCTGTCGCCAATTTTCTTGTGCCAGGACGCTTATCTTCCATTGCAATGCGTTGCAGGGTGTTACCGGGGAAGCTCAGGCCATTTTTGATGCTCTTAATAAGAGCCCATGCGCATCTATTGGATCGTTTTTGCTGTGGTACTGGGCAATGGTTTGTACACCTACTTTGCTGGCAAGGACGAGTCGTTCTTGATAAATAAACTGAAGAGAAAAATCCCGTCTGAGTACCGCATCTCTGAGAAGTGGGACAAATGCTTGGAAAATTTCACTCTATACTTTGGCGCTGGTCTTGTGGCTGGAGGGCTCACGTCGGTTGTTCTAGCGCGCTCTGGTGCTGGTCGTGGACTAATCACGGGGTTAGGCGATCTGGTGCTGGATCAAGCTGGACAACGTGTCAGATGGCAGAAATTTTCAAGCCCGACGTCGGGCTAGGCCAGATAAACGATCTGAGCTACATCATGCCAGCCTTCCAGCAGCAAGCAATCGTGGACTTCATGACGCCCACGACTACGCAGCAGCAGCTTCAAAGTGTGCACTCGAGGCTTCTGCGATCTGACCAATGTCGAGTTGCTTGAGGCGTACTTAGCAGAGCAGCTGCAAAACAAAACCGTGGACATCGATGCTAATCTGGCGTTGCTCAAGCTGTATCTGGTTTATCCCGCTACGGCTAACGCTACGGGCCAAAATTCTAGCAAAGGGAATAATGGCGATACCCTCAACCTTCTTTTCCGGCGTCCACGATGATCCCGGAAAACATTCGCGAGGATGCAGTTGTGACGAAAATGCTGCGAGCGGGTTTTCTGCTTCAATCGTGCTTATTCGAGGATTATTGGAAAGAAGAAGTTGCTCTTACCAAGGAGTTGCCAGGTTTTCTGGATTCGGTACGCGCCTTTATTCTGACAGCCGTTAGCCATAGCCACAGTGCCATCTCTATTGAAGTCTTGGCAGCAAAGATTAACGTTTCTAACAAGCGGGTACCGGAAATAGTTGCCGAGAAGTGGACGCTGACAACGTGATCCAAATAAGCCCGAATGAGGAAAATCAGATGCAGGCAAAAAAAGTTCAGGAGAATATCGAGTTCGAGGACGTGCTTAATGTCATTCATGTCCTCTGTAAATCTAATGTCTGCGCAAGCGGTGGTGGGGTAGCTGATGCAGAAGAAGTTTGTGAACATCTTGCCAAACACCTTAGACTTGAAGGAGGATCCCAAGTGCTGCCATGTTCCACAGGCGTCATTGGCTGGCGAATTCCTGTGGATGCCATGGTCGAGAATCTCCCAAAGCTTGTTAATAATTTACAATCTGATTCGGTATTACCAGCTGCTGAGGGTATCATGACAACCGACTTGTATCCAAAAATCCGTTCCGTGGACGTATGTGGTGGTCGTATTGTTGGGATCGCTAAAGGTGCTGGGATGATTGAACCGAACATGGCTACCATGTTATCGTATATTCTCACTGATTTATCGATTCCGCGTGATGTGCTGCGTCAGCTACTGACTGATGTTGTGGGTGATACGTACAATTCCCTTAGTATAGACACAGATCAAAGCACGTCTGACACGGTGACGCTCGTGTCGTCCGACCAAATTCCATTTGACATCAA---GCAGTTGAATGAGTTTAAAGAAGCGTTGCACGCCGTGTGTCTTGGGTTAAGTGAAGACATTGTACGCAACGGTGAAGGTGCGCACCACGTTATGCGAGTGCGCGTGAGTGGCGCACTATCGAAGGATATGGCTAAAGGAGTTGGCAAATCGGTTGTAAACTCACCTCTGCTCAAATGTGCAGTGGCTGGCAATGATCCAAACGTGGGTCGTCTCGTCATGGCTGTAGGTAAGTACATGGGTCTACACCACAAGGACGTGAACATTCAGCGACATATGAAGATTCGAATGGGTGGCGTTTTGATTTTTGAAAACGGTGAAATGGTACTCAACAACGACATTGAAGCGAAACTTGTGGCGCACATGCGCGATGCAATGCTGATTGAGCCTACGCGCGGTGGTTTGGACGCAAGCTCGCACAATTATCCGCCACATGGAAAGTTTGTCGAGATTAATATTGACTTGGGAGTTGGTTCTGAAGAGACACAAGTACTAGGAATTGACTTGACGCACGAGTACGTGGCCATAAACGCCGACTACCGTTCGTAATGGCTGC---AGCGCAATCGAGTCCCGCTGCCGCTTACCTGGCATCTTTAGGCGTGGCTCCTTC---TGCTGGCGGCGAAAAGAAGGATGAT---AAGAAAAAGAAGAAAAAGGGCAAGAAGAAGCTGAAAGGAAGCCCATATCAGCTGCTGCAAAGGCGCTGG-TGGAGCGCCAGGCAGCTTTGAAGGCTGAGCAAGAGCGTCTTGAGCAGGAGCGTTTAGAGCAGGAGCGTATAGCCCAAGAGGAAGAATCTAAGCGACTGGCCGAAATTGAGCGCGAGGAAGAAATACGCCGCAAGAAGAAGGAGGCGCGCGCCGCCAAGATTGATCGTCAAAAGAAGGAGGGAACGTACATGACTAAGGCTCAAAAGCAGAAAGCCGCCAAAGCACAAGCTGCACTCGAAGCGATGCAGCAAGCAGGTCTTGTACCGGGGGTTGCG------GATGAGAAGAAAAAGGTTGTGTATGCATCCCACAAGAAGAAGACCAAGAACACAAAGTGACATAAGAAAAAGAGGTGGAAGCTCCAGATGCATGGGACGACGAAGACTGGGAAAAAAATGATGTTGTCGCCTCGCTGGAAGACAAGCTAGCACAAGAAGGAGCAGGAGCGTTTACGTTTGCAGGGAATTGCATTGAAAAAACGTCAGGAGGAGCAGGCCGAGCAGATGCGTCAGGATGCCGAGGAAGAGCGTTTGTGCTCTAAAGCCAAGCTTGAAGCCGAGCGCAAGAAAAAAGAGGCTGCAGAGCGTCGACACAAGCGCGAAGCCGAAGCGCGTGCCAATGTTTCTTTGGACAATTTGCGCTCACCTATTTGCTGTATCATGGGTCACGTTGACACTGGTAAAACGAAGCTTTTAGACAATATTCGTCGCACGAAGGTGCAGGATGCAGAAGCCGGTGGAATCACGCAGCAGATTGGTGCGACATTCTTTCCTGTGGAGGCCATCAAGCAGAAGACTGCCAGGCTTGCTGAGGTGATGCATCTTGAGTATCGTCTGCCAGGTCTGCTCGTGATTGATACTCCTGGTCACGAGTCATTCACGAATTTACGTTCTCGTGGTTCCTCGCTGTGTGACATTGCGATTCTAGTTGTAGATATCATGCATGGGCTGGAGCCTCAGACTTTAGAGTCACTACGCCTATTGCGTCAAAAGAAAGCTCCTTTTGTGGTGGCGCTCAACAAAATCGATCGTTGTTATGGCTGGAAAACAATGCTTGACACGCCTGTTCAAGAGGCATTAAAGCATCAAAATGAGCATGTCATTCGCGAATTCGAGGATCGAATGAAGTCGATTATTGTGGAATTTGCGGAGCAAAAGCTAAATGCAGAGGTGTACTGGCGCAATAAGGACTTGGCACACACGGTTTCACTCATCCCTACTAGTGCTATCTCTGGTGAAGGGGTATCGGACCTGTTGATGATGCTCACGCGCCTTACGCAGGAGCGTATGGCCAGATCGCTGGCATTTGTCGATATTCTTCAGTGTACTGTGCTGGAAGTGAAGGTTATTGAAGGCCTAGGTACAACAATTGATACAATTTTAGTAAATGGAACACTAGAGGAAGGTGCAACAATTGTTGTCTGCACATTGGATGGTCCGGTGGTGACAACAATCCGATCGTTGCTCACCCCGCACCCGATGAAAGAGATTCGTGTTAAGGGCGAGTACATCCACCACCAAAAGATGAAAGCAGCGATGGGTGTAAAAATTTGCGCGCAGGGCCTCGAGAAGGCCGTTGCTGGTACACAAATATATACGGTTGGCCCTGATGACGATGTGGAAGAATTGAAAGACTCGGTCATGTCTGACCTAACAGGTATACTGGACTCCGTGAAGGCTACCCGCCGCGGTGTCATGGTTCAGGCTTCCACATTGGGCGCCTTAGAGGCACTATTGGAATTCCTCCGTACGTGCGATCCGCCCATTCCAGTGTCTTGCGTCAACATTGGTCCTGTCCACAAAAAAGACGTGATGCGTGCTTCAGTGCAGCTTGAGCACCAGCCCGAATTTGCTACCATTCTCGCATTTGATGTGAAGGTCCACAGCGATGCCACAGAGCTTGCTACGGAACTTGGAGTGCGAATTTTCACAGCCGATATCATTTATCACCTGTTTGATCAATTCACGGCATACATGGATAATTTTCGCATGGCACGTCGAGAGGAATTTGCGGAGGTAGCTGTGTTTCCATGCGTGCTAAAAATTCTGCCTAATTGTATATTCAACAAGAAGGATCCAATTATTCTTGGTGTCGATGTTGAGGAAGGTATCGTCAAGATTGGTACACCTCTCGTTGTCCCATCTGCCGGCG---GTTTATATATTGGCAAAGTAGGGTCCATTGAGCGTGAGCATAAGGAAGTAGACCGCGCCAAAAAGGGCGCATCTGTAGCTGTGCGGATAGACAACGAGAGTAGCGTCATGTACGGCCGTCACTTTGACTCCAAAAATCAATTAGTATCGCGCCTTACTCGCCAGAGCATCGATGCATTAAAAGAAAATTTCCGGGAAGATTTGCAGAAGGAGGATTGGCAGCTCGTCATTAAGCTCAAAAAGGTATTCGATATCATTATGGAGGGCTGGTTAACAAAGCGTCGAGACCACATGAACCTCATTTGGTTGGATCGATACTTCCTCCTTGATGGTAATCAGCTTCGTTACTATCGAAAACGCGGTGATCCTGCTCCACGAGGAACGTATGTTCTAACTGATGGTTGTGTTGTAAGTCCTGTCTTTAATTCTGAAGAGAAACATAAACACCATGGACCTGTGTGGATGTTCCGCATAACATGAAAAATCCTACAAAAAGGACCGTTTTCTTGATCTTGGAGCTAAGAGTGAAGAGAATGCCAAGGAGTGGAAGCGCGCTTTAGAAAACGCTGTGCGCTCTCTGCGTGAACTCGTGGCACACCAAAAGAAGCTCAAGACAGATGCCGAGAT---TTTTCCAACGCCGACGCAT---------GTTCCAAGAATGCTAAAAGCTGATGAAGACACTGTGTCACTGTTTAATTTGGATGCAGATAATGTCAAGGCAGAGCAGGCATGGTGGTTGGCTCGAGTAGAAGGTGGACTCCGAATTATGCAGGAATGTCCGCTTGGACCAAATGTGGCCGCAAATCTGT-----------------------------------------------------GCAGCACTGAGTTTCAGCACTATGTATTCCTTCTCGAACAATATGCTCATGAGCTTCGTAACAGCACTTGCAGCTATTGCC-------------------------------------------------------------------------------------AAGTTTTTCGTCTCATTATGAACTCCAAGCGATTCCAGCGATGGGACGCAGCAACGGCTACTATGCGCGTCGTACAGCAATTAGATGATCACGCGGATATTGTCTACGTGACGCAACGACCGACGCAGTTGGGGCCATTCTGGCAAAAAGCGCGTGACCTTGTACTTATGCGCTACTGGCGACGTGAAGAGGATGGATCATATTTTGTCATGTACCAGAGTATTGACCATCCAGAATGTCGGGTGAGGCATAATTACGTGCGTGCAAACTTCCTAGGCGGCGGCTTTGTTATTGCCCCCCAGCGTGTTCCAAGT---GGATCGATTCGCACGTTAGTAACGTACGTGATTCGCTACGATCCGGGAGGTTG---GTCCCGCATTTACCATCAACTTGGTATGGACGTGGATTTGGTCATGCCGATGCTTCGTAGTGTTGTGGGAATCCGAGATGAAATGAGTGCTACGGACTTTATTACGCCTAATGTCTCGATGGCACCTGCTAATGACGACGAAAACTGGTCAGGGAGCGTGGA---CTCTGCGGGTGCTGTACTGGGCGTTCAAAAGTTGGAA---ACGAGTCTTCCCGAAAAAATGTGGGCGGAACCTGATGCTTCGCTTTTCAGTGTCCGTGGACATAATTACTTATCTGACAAGAAAAAGATTTCGTCGGCACCTGCAATGTTTCATACTGTGGGTGTGGATCTTCTCTCGTTTGAAAGTCCCGCAGAAAGGTATAATGTCTCTTCTCGTACTGATAGTGTCGGCCGCACCAG---CTCCAAATTTACGTTTGTTGTCAACATGATCATTCCAAGTCCAGAAAATGTCTGCATGGTGTTCTACTTCCAGCCTGTGCGTGACAACGTTTTTGAAGATGGATCGAGTTTTTCGGAGCTGCTGAACGACTTTATCGATGGTGATGACCAGTTCCGGAACTCACGCTTTAAGCTTATACCTACAGTCGCCGAAGGAAGCTTCATCATCAAGCAGTCGGTTGGATCAAAGCCCACATTGCTCGGCAACAAGCTGAAGTGTCTCTATTATCGAGAAGAAAACTATTTTGAAGTGGACATTGATATTTCGTCCAACTCAGTTGCCAATACGGTGGTAGGCATGGTACAAGGTGTGACCAAGTCGCTCGTAGTGGATCTGGCCTTCCTACTTGAAGCCCAGTCCGACGAAGAGTTACCGGAAGTTATCCTCGGCGCAGTTCGCATGCAACACGTTTCGCTCGACAAACCACGCCGAATGCCAGCACATGGGAAACGGGCAGCAAGGACGAATTTACATTCTGAGGCCTTCACTGAGGCTTTGCGTGTGAATCTGCAACGACTTATCGTCTACGCACGTTCGGCCGACACAACATTGCAGCGAGAAGTTGCCGAGAAGTTGGCCAATGAAGCTGTTAAAACAGACAGGTGCAAATTGTGGAGCTTGATGGACTGCAACTGTTGCTTCCGCTCACAAAGTCAACGGACACGGAGGTACAGCGTCTAGCGGCACATGCACTTGCTAATTTATCAGTTAACTCTGAAAATCAGTCCAAGATGGCCACGGAAGGAGGAATCGACATGCTTATTGACCTATTGAGCAGCTCAAACGAGCACGTACAGCGACAGGCCGCCAAAGCTCTTGCCAATCTTGGGGTCAACGTGGATAATAAGGAGCGCATCGCCAAAGCGGGTGGCATCAGACCACTTATTGACTTAGCCAGCTCCCGTCAGATAGGCGTCGCCGTTGAGGCCATTGCAGCGCTCGCAAATCTAGCAGTCAACG---ATGCTAATGAAGTCGAGATTGCACGAAAAGGCGGTTTAAAGCCTATTGTTGATGGAGCGCACTCCGATTCTGTCGAGCTTCAGTCTCAAGTCGCACGCGCATTGCGCAATTTGTCAGTCAACCCGGAAAATAAGCAAGCAATCGTAGAGCTGGGCGGAGTAGAGGCTCTACAATCACTTGTGCATTCTACAAATGACCGAATTTGCCAGCAGGCTACACGCGCGCTCGTTCCCAATGTCGTGCTCACGCGCTCGTCACCAACAATCTCTTA------GTAGTGCCTGTGGCACGTGTGGCCGAGGTCAAGGAAGAGAC------TGTATCCTTTGATGGTACCATGGTAGATGTTGAATTCTCCGCTACAGTTGAGACCGTC---------ATGGTGCCTACTACTACCCCAA---ACAGTGTTGCCAAT---GGCTTTTTGTCTATTTCCGATCGGTTTCCTCTTTCAAAGGTGGGAAAAGTTTGCATCGGTACGGCTCCAATTCCCATAAGTCG---TCTACTAAATG---ACACACCGGAAATGACCC---AGGTTGAGCAA-------------ATTACTGTTCTCGTGGAAGATGGTACGATAGATGATAAC---ACGATCAAAGCTGAG---CCGGAAGGTGAGCGACGTGTACCACTATCAAGCCACAGA--TGTACAAGAAAATGAGATGGAGCAACTTCGATGGAACATTCAAGAACAAGTTTTTCAACGATATTTCCATCAAGCACAGAAATACGAAGAGTGGGGAAAGCTGGAGGAAAAAGACGCCGAGTGTTTTTTATTCCAGGTGGAAGACGGGAAAACATCAAAGCCGAAAAGCGATTTCACGATAATGCTGTTCGCGATGCGACGGGAAAGGGAAATGCAACATGGGTAAAAGAACGACGAGATCGGTTTGAGGAAGAAATTCGACTGTTTTTTGACGAAACGAGGCACAAACAAGAGCTGATGCTTACGCGACTTGAAAAGGTGTCGCCGTTCTCGAAGTTTCCGATTCTCGGTAACCGATTTTTACTACTTCGTTTACGGGGAAAAGGAGGCAACGGAGAAGTATGGGATGTCGTCGATTACGCTAATAATAAGCAGCTCTGTGCTCTTAAACTTTCTACTTCAATTCAACACGCCCAACGCGAACATCTAACGCACACTATGCCATCCGA--ATATTGTTCAGGTTGGTGACACGCCTTTTCTTATACGGTATCAACAGCAGCAATACACCGCATTCACAGTCGCAAGCGTTCAGTCGGATCTCCAACAATTGATAGAGG---TATATGAGCATCTAGATGATGACTCCGCCTACAAGGTGCTTTTCCAGCTTTTAAACGCATTGAAATATCTGCATGATTGGTGTTGCTCACTACGACCTCAAGCCGTCAAACATATTGATTGATCACGACGG------------------------------------------CGCCAAAGCACCTACTCATAACTCTACCGTTGGAACTCTACGATACCTTCCTCCTGAGTGCTTTCGTTCCAATCGCAGTGA---------CTGCGAAGCTACTGCTGAAAAAGCAGATGTGTGGATGGTGGGTGTAGTATATTGCATGATGGTAACGGGAAAGCATCCAATTTGCCCTGAGAAGTCGACACAAGATGAAGTAAAGACTCTAATGTCTCAGTACACAGGC---GATTTCCGGTATGCACGGCCCATTTCCGATCTATCGCGATGGATTATGCAGGGTTGTCTCCATCCTGATCCGCGATGCCGACCAACAGCAACCCAGTTGCTTGTCGCGCTTCAG

Plasmopara_muralis TGGACTACCACCGCCAGGTGGCAAAAGAGCTGGCTGCTTTCTTCCACACATTTTATCGCTTTGGTGCCGCGTCCAACCGCTTTGATCTTTTCCTACACAACATCCTCTTGGACTCAATGTGCATTGGAGGCGTCGTCTTTCTCGTGCTCTGCGTGATTGTGGTGCGCCGCACCGTCCTGCACATCCGAAGGCCTTTGTTCGACGCGCCTCGATGGATTCCACGTCCGTGGAGTTATTGGCAGTTGGACTTTTGACTTTCTTGGCTATAAGTGCGCTAGGTGGTCTCTTAGGAGAAGCTATCTACAGACTAGCAATGCTTTGCGCATGAATGCTGACAACTTGATTACATCATTTAACGACAGTATCCTGCCGGCTGATGCTTTTAAGATGTCAGTAGAAGCGCTGGACTCGTGCATGCGTCAAAGGATCTGTGGAACCAAACAGACGTGCTTTTGCCTCAAAACTACTCAAATATTGCCAAGGACTGGGAATTTAGCTATTTCCTACTAAAATCATCGACCAATGGCGCTATATTGATGGTCGCACTTGCCAGTTTTCTTTTAATAGCGTCGGTTGGATGGGCCATGGTCTCGCCACTTCGTGTCTCTATCCTGATCATTTTGAGTGTTGTACCCATTTCTCATTCGTTAATTGGGGCCTATTTGTCGTCAACGATCATGACGGCAGATTTTTGTGCTGCGCCAATGAATAACACTCTTGGATTGATTGGCACGTGCCCTGTGAACGTTTCTTGCCGTTTGCCGACGCAATGGCGATGCAATGCGTCACACCAGGGAGAGATTGGTCAGCGTATAATGCGCGAGTTCCTGGATCCGATCGGAGCAGCTGTGGGGGTGATGTTTGGGTACTTTGCGGGTACAATCAGCAATTGGGAACATTTTGCCTTGATCGAAACGTGGCGGTGGCCCTTTTTGCTCCAATTTGTGGCCCTATTGCCTTTAGTTGGGGCCATTTTTTTCGTTCCGCAAAAACATTTAGTGATTCGTATTGTGAATAAGAAATCCGAGCCTCCAGTGGTTGGTACCCCCGTCAGTACACGGTCCACGACGTACCACGATGAAGAAGATGCTTTGCTGGCGAGTGCCAGTATGCAACCTATGGTGCTTTGGATACGAGGGGTACGAACAGAAAGCGTTCGCGGAAGGATTGTACGAATTGCTGCACATTCCCGTCTTTTGCTTGATTGTTTTCGGCCTCACCACCGTCTACTTTGTCGTCACGGGAGTGCAATATTGGAGTACAATCTTTATGATCAAATCGCTCCATGCGTCCAAGTATTTAGTAAACGGCCTCTTTGTCATCATCTCGGGGACGGGACCAATCTTGGGCGTTTTCTACGGTGGCTGGTTGATCGATCGCTACGGGGGCTACATTGGCGTGGAACAACGGGCCAAAGCTCTTGGGATTTGTATGATCCTTGGCCTCACCGCATTTGCCATCTCGGCCGTGACGACGTTCTTCAATGACATTTATATCACTGCCGGCTTTCTCTGGCTCTTGCTCTTTTTCGGGGGGGCCATCCTTCCAGCACACCGTGCCCTCGCGTCGTCGTTTTCCGTCATGATTTTCAACTTGTTTGGCTACGCCTTGTCGCATGAGTGACTTACAGATGGTGACGGGGGCCAAGTGTTTCGTACCAGATGAGACTCACGTGTGGCTTGCGGCCGAGGTCCTTCGCGACGAGAACGCGGGCGACGGCCTGGCGCGCAACATTTACTGCAAGGTCGAACTGCCCGATGGCGATTGAAGAGCGCTGTGTCAACTTGTTGGCCAAGAAGACAAAGGCTTTGTTGCAGTCCATCAACTGGAATCCCTTCCGTACCAGAACGAGAACGTGGGACCCGAGGGCATTGAGGACATGATTACACTGAATTACCTGCACGAGGCCGCCATCCTCTACAATGTCAAGAAGCGTTTTCTCTGCGAGCTTCCGTACACTTACACGGGGGACATTTGCATCGCCGTGAATCCGTATAAATGGCTCCCCGATCTCTACGAAGAGAGGCAACACCTTCGGTACCTCAACCAGCCAAAAGAGGAACTCCCGCCCCACGTGTACGCCACGTCCGTTGCTGCCTATGACAATATGCGGCGCTCGGAGCAAAACCAATCCATTCTCGTGAGTGGAGAGTCTGGTGCGGGGAAAACGGAGACGACCAAGATCTTGATGAATCATTTGGCCACGATTGCCGGGGGTCTCAACAATTCAACCATTAAAAAAATCATTGAAGTCAATCCTCTCCTCGAGTCGTTTGGGAATGCCAAGACGGTTCGAAACGACAATTCCAGTCGCTTTGGCAAGTTTACACAGCTGCAATTTGATGGGCGAGGCACCCTGGTTGGGGCCAAGTGTCGCACGTATCTCTTGGAAAAGACGCGCGTCATTCACCACGAAGCCCCCGAGCGCAATTACCACATTTTCTATCAATTGACTCGTCAGACGTGTCTGCAGAGCTGCAACTCGATGCGTCCAAGGTGTACGCGTATACGGGGGACAATAAGGGTCAAAAGATCGAAGGGTTGTCGGATGCAAAACACTTTAACAAGACACGCGAGGCCCTGGAGCTCGTGGGGCTGTCGCGTGGTGACCAGCAACCCCTTTTTGAAGTCTTGGCGGGTGTGCTGCACCTTGGAGAAGTGCAATTGCAGGCGGACGCGACAGATGACGAAAAGTCGTTGATTCCCGAGAATGACGAGGGAGCGACGTCTGCGATTCAGATGCTCGGTGTCACGTGGGAGGCCCTCCAGACGGCCTTGTGCTCCCGTACCATGCGTGCGGTTAACGACGTGTATAGTGTCCCGCTTAAGAAGGAACTGGCCATGGACTGCCGTGATGCCCTGGCGAAGGCTATCTACTCGAACGTGTTTGACTGGCTTGTAAATACGATTAACCAGTCACTTGCCGATGACGGCCATATGGTGAATCACGTGGGGGTGCTTGATATTTTTGGCTTTGAGCACTTTAAACACAACTCGTTTGAGCAATTCTGTATCAATTTTGCCAATGAGAAACTACAGCAGAAGTTTACACAAGATGTGTTTAAGACCGTGCAGATTGAGTACGAGGAAGAAGGTATTGTGTGGGACCACATTGAGTACGCTGACAATCAAGACGTGCTGAATGTAATTGAGTCCCGCATGGGTATCATTTCACTCTTGAACGAGGAGGTTATGCGACCAAAGGGAAATGAAGAGTCGTTTATGTCGAAAGTAACATCGTTGCACAAGGAT---GA---AGCGCACGTCATTGAGTTTCCGCGCACGTCTCGCACCGAATTCCTCATCAAGCACTATGCCGCTCCTGTAATGTACGACTCGGTCGGGTTCTTGGAAAAGCACAGAGACTCACTGCTTCCTGATTTATCTGAGCTAATGCGTGGCTCTTCCAAACCGTTTATTGCCAAACTTTTCGAATCAAAGCTCGAGCCAAGTCCGCCGAAAAC---------GGGGTGGTGCTTTGTCCATCACGACCGTTGGTACGCAGTTTAAAGAAAGTCTGGCGGAATTAATGGCTACCATTAATTCGACACGTGTGCACTACGTACGTTGTATTAAGCCCAACCCGATCAA---------GAGTGCAACTGTGATGGATCAAAACATGGTGGTTTCTCAATTGCGGTGTGCTGGTGTGATTGAAGCCATTCGCATCTCGCGTGCGGCATACCCAAATCGGTTGCAACACACGGAGATCTTGAACAAGTTCTGGCTCTTTGTGCCAAGTGGTGGCCACACTGTAGAGGAAAAGTGCCAATTGCTTATGGACAAGCTGAAGCTAGAATCCCCCAGACAGTACCAGATGGGTAAGACCCGCGTATATTTTCAATTAGGCGTGCTTGAAGAGCTCGAGGATCGACGCAAAAAATTTTTGGATGCTAAGGCCACGTACTTGCAATACACGATGCTTGGCTTTACGCAGCGTATTAAGTATCTTCGAAAACTTGAGGCTATTATCAAGTTCAGTGATTCGGTGCGTGATTGCCATGCGCAGATACAACACGTTTCTCAAGGGCCTCATCCTGGCACAAGCTCGCTGGCGAGGCATTCAGGGCCGCAAGGTGGCTGCTGACGTGAAGAGTAACCACTATGCTGTCATTATTCAGCGATATGTGCGTGGCTTTGTAAAGCGTCACCATTATGTCAAGATGCGCGCAATGGTTGTTCGTGTGCAAGCCTTGGTTCGTATGATGATTCAGCGGCCAAAGTATCTGGCTGCTCTTGATGAAAAGCGTCGTGAAGCAGACATGGCTTATCAGCTTCATAAGCTCAAGGCTGCTCTGCATGACGAGCAAGAGCGAAATGCTCAGCT------GCAGCGTC---GTAACAGTTTGAC--GACAGCGCGTCTGCGGCCTCGAGCGTTGTGATGGCTGACGCTGGCGGCATGATTGAGACTCTCACGGACGAGAATAAGAAGCTTCGTGAGAAGAATGAAGACATGAAGGTTGCGATGAAAGGTTTGAAGGCAGAAATCAAGAAATTTGAGAATGACAAGGAAGTTTCATCGGCTGGCAATCATGTGAAAATGCGCCAGCTACAGGATACCGTGCGTGACAAGGACAAGAAGATCTCACAGCTTGAGGCTGATAACAAAAAGCTAACGGAGCAAATTGCTAAGCTTCAGGCGGAAGGTGTTGT---CCCCGATAAAAAGTCCATTCCGAAAAAGTCGATCTTTCGCACACTGGGCTCGAAGAAGGAGAACAAGCCGC---------------GTGAAACCGTGCTGATGGACTCGCTGACTGGATCGGATGACTTGAACCGTGGAGAAACTCGTCCGTCAGA---ACGCAACTTTCCACGCATGC---TAGGCATGAATGTGGGAAGT---CGCTTTTGGAACTCGAATAAA---AAGGGGGAC------------AATGGGGATGGTCTTGACGTGTCTGAGGTTGAAGGTGAAGACAGCCCCACCAATATACACGCAATACGATTGTAAAGAGCGTGGAAGTGGGAGTGACGGGAGCTATGTCAAGCATTAAGGGCCGCATG---------TCGGTGGTAAAGGGGATGTACGAAGCAAGCGTACGAACAGGATCGTCCTGTTCGCGAATCGTTTAATCTAGATGCGTTGCCCGAAGTGTCTTTGCCTGTTGGTTGGGAGGCAAAAGTGTCTCGCTCCACTGGCCGCGTGTACTACGTCAATCGCAAGCTTGGGAAGTCGCAGTTCGAGCGCCCGACTCTGGCCTCTCTTAA------GGCACTGAAGCTGGCACGTCAGAAGTCGGGCAATGCGCCGTAAAGCTGTAGTCGCTGATTTTGGCTGTGGGGATGCGAGACTGGCCGAAAGCGTATTGAACAAGGTGCACTCGTTTGACCTGGTGTCTATTGATATTGCCGTATATTGCTTAGCGCTCATGGGAACGAATGTTCGCGAATATGTGCGTGAAGGGTACCGTGTGTTGCGACCTGGAGGTGTGCTGAAGGTTGCCGAAGTTAAAAGCCGTTTTGAAAGCGAGTCATTGGGAGGATGGTAGCCGTCTTTGTGACCGCGACAAACGGCGTCTTGATCTCGGTCAGGAACACGACCGTACTCGTGGAGAGACCAAGTATGTTGTCATTATCGGTCAAAAGACGGGCGAGGCCATAGCTGTGCCTTTTCCAGACGTGGACCGCGATTTAAAGACGCTGCTGGGCCATACTACATCAATGGTTACCCACGTGGCAGTTAATTTCGTCACTTTTGTTGACTGCAGACCGCGACGAAAAGCTTCGTGTATCGAGGTTTCCAAATACTTCGATTATCGAGAGCTACTGTTTGGGCCACGCTGCAAGTCTCACTAAGGTGGCGTGTAGTTTGTTGACCCCGGAGCTTGTAGTATCAACGTCTATGGATAACACGATAAAGCTCTGGCAGATGAAAACAGGAAAGTTGGCCATTTGCCCCAAAACAACATTGTGGCCGTGCTTGTGAATCATCAATTCGTGCGATTCTTTCACATCGCTCCCATCCGTTTCTCAAACTATTTGCAGTATCAAAGGGAGGCCTCCGCTTTTGACCGCTGTGCGCGATTTTCGCAAGCTCCAGAATGACCCGCCATCGGGCGTATCGGGTGCCCCTATGGACAACAACATTATGCTTTGGCAAGCCGTCATTTTCGGACCGGACGACACGCCGTGGGAAGGAGGGACGTTTAATTTGACTCTTGAGTTCAGTGAAGACTACCCCAACAAGGCGCCGACCGTCAAGTTTATCACGAAAATGTTCCATCCTAACATTTACAACGATGGTCAGATTTGTTTGGATATTTTACAAAACCAGTGGTCACCGATTTACGACATTTCGGCCATTCTCACGTCCATTCAGTCGTTGCTGTGCGATCCCAATCCAAATTCGCCCGCCAATTCGGAAGCCGCGCGTCTCTTTCAGGAGAATAGACGAGAGTATAATCGCCGCGTACGCGAGATTGTCGAGCAAAGTTGGCAGGCCATCGATGGTCTCGGCCGCCAAGATGCGTGGCGCTGAGAATCGCATGAACGCTGGACGTCCTTTTACCGCATGGCTTAACAATGTCTCGTCGGACCGTGGTCTCTGTGGTGGTATCAACTCGGGTATTGCCAAGAACACCCGCAAGCAGACCACGCACAGATCTTTGTAATTGGTGACAAGGCTCGTGCTCAGTTGCGTCGCGACCTGAGCGACAACATTCGTGGCAACGTGACGGAGACCTATCAGTCACCTCCAAACTTTACAGTCGCCAGTGCCATTGCCGAGGCTATGGAGTCGGAGAGGAGTCGTCGCGTATGGCTGCAATGGAAAACGCGACAACCAATGCTCAAGAACTCACGCTCACGCTCGTCTACAACAAGGCTCGTCAAGCACGCATTACGACCGAGCTGATTGAAATTATTTCGGGTGCCGCGTCGCTGGACAGTGACTGGCAGCGTCATCAACGTGCAGAGCGTAGCGCAGTTTGAAGAAGCGAGGGCGCGCGAGTCGACGCTCTCCGTGACCTTTTTCTGGGCGGATTTTCAAGCCTGTCGTCCGAACGGCCAGCTGGACGTGGGGTCGTTCTGTACTCGAAAAGCTCGAAGGCGCGAATGTGGCGGAATTAGCCAAGCACGTCTTGTGTACCGCCTGAAAAAGTTAATTACTGCCTCTCCCGTCATGCTCTTTATGAAAGGCAACCCGACCGAGCCGAAATGTGGCTTTAGTCGGCAAATTGTAGCGCTGCTCGATGCCGAAAAGATTCAATTTGGAACGTTTGACATTCTCCACGACGACGAAGTGCGACAAGGTCTGAAAGAATATTCCAATTGGCCGACGTATCCGCAATTGTATGTGAATGGTTCCCTTGTGGGTGGCTTGGATATTCTCAAAGAAATGAAGTCGGAAGGCTCGATTGCTGAGCAGCTGAACGTACAAGACGCTGAAGCTGCGTTTGAAGAGAGCTTGCGTGCCCTCGTGCATTCCGCACCTGTGCTTCTCTTTATGAAACTTTGACATTTTATGTGGAGCTACCAACGTCAGGACCAAGACATCTCGAAAGTCGTGACTCTGATGCCACCTTCGTTTCGTCGCGATATGTGCTGAAGCCATTGCGTGGAGTCCGAAATCGTATGGCCATGGCGTTTCGCGATCCTCTGGATGAGAAAGGAGAGTTTTGCATTGTGCAGCTCCCCGTATCACGTCGAAGTGCACAACTTTGCACTCACGAGGTATCTCTACAATCGACTGGCAAATTTAATGTGTTGCACCGGGACCACCGCAGTGAGGTCAGGTCGCTCTGTGTCCACTCGAAGCGTGACTGTTTGTATACGGGCGGACTTGATGGATTACTAATTCGGTATGACCTAAACAAGGGGAGTAGCGACATTGTGATCGAGCGTCGAAAACATATCTCAAAGATTAATTCGGTACTGGAACATCCACACAATCCAAACTTGCTATTGGTTAGCTCAATCGAACAATAGGCACAATTTATTGCTTCATGACCTGCAGCGCTATGACCGCGAGGTGTCGGGCAGCTCAATGAGTCAATACGTTGTACCACGGTGGTCTCCTGCCGGCTATCATGTTTCTTGTGGCAGCAAATGCGCTACTGCCACGGCAGCTTTTCCACACCCACGTCAGCCACCATCGGGGCCTCATTCTTGCAGAAGCGTGTGATCGTTGGAGGAAAACTGACGCTTCAAATTTGGGACACGGCTGGCCAAGAGCGCTTTCGCTCCATGGCGCCAATGTACTATCGCAACGCTAAAGCGGCTATCCTGGTCTTTGACCTCCAGAGCGAGGCCAGCTTTGAGAAGATTAAAGAGTGGCTGCATTTGCAGCATCATTTAAATGAGGACATGTGGGCAACAAGAGCGACATTTTGACTTTTCCAAGGCCCAACAGTTTGCGAATGAAATTGGTGCGCTAGTCTTCCGCACCAGCGCCAAAACAGGCGAAGGAGTGCAATCGCTCTTTGAGAGTTTGGCGGTGCAGCTTTTGAAAAAGCACGATAAAGAGCAGCGGAGAGCTTTTACGTGGTGTATATACCGGCACATGAAGCCAAAGAGCTAGAGGAGTGGACAATCAAGCTTCCGCAAGACAAAGAGTTGCAGCTTGGATGTTTGACTGAGCGTCTGCGGGATCATTTCAAGAGCAGTGTCATCCAAGCAGCAACAGAGACCTTCAAGCAGCAATTGCTAACCCAACTACCTCAAGGTGCCACACTGACAAACGAAATGCTCCAAATGATGCATGGACAGCCTTGTGGACTCGATTCCACTGGTTTTGAACACACCCGACATTAAGCACGTTAGAAGTAAAGGGTGACGCGTTTATTGCACGGGTATTTGACGGATGACTTTGTGCGGATGGACTTTCGCTTAAACGAGGTGTCGGGTGATGCACCGTGGGTGCAGGCCGCCAAAATGCAGAAGTCACGCAATCGCTAGAAGAAATTCTCGCGCTGGGCCATGGACTTGACGACGAGGACTACAAAGCCGAGCTCGAAGTTTTGCAGAACGACCCCAACTCGAATCTTTACTCGGCTGTCACGTTCGAAGAGCTTGCTCTCCCAGAGCAGCTTCTTAAGGGCGTGTACAATATGAAATTCGCCAAGCCGTCAAAGATTCAGTCAGTAGCGCTCCCACTCATTCTCGCCAATCCTCCCGAGAATCTTATTGGTCAGGCACAGAGTGGGATCCCACACGGGAGCTGGTGCGACAAATTATGGCTGTTGTCAATGCCATGGGGAAATTGTTGACACGTTCTTGGCCATTCCTGGCAACGATCTGGCGCGAGGTGAGAGCTTAAAGGCCCAGGTTGTCGTCGGAACTCCGGGCAAAGTCGAAAGTATGATTAAGAAGAAACAACTTGACACGCGCGATGTCAAGATCTTTGTGCTAGATGAGGCGGACGTGATGGTCGCGGAGGACGGGATGCGTGAACGCTCAGTGGCGATCAAGCGTCTCCTCAAGAACCGTGCATGCCAGTACCTGCTCTTCTCGGCCACGTACGCTGACGACGTGCGCGACTTTGCCCAAAAGATGGTGTCCGATCACAATATCATTACGGTGAAGAAGGATAAGCTTACGTTGGATGGGATCAAACAATTCTGGATTGACTGCAAGTCCCGTGAAAACAAGTTTGAGGTGTTGTCGGACATTTTTGCCATCTTGTGCATCGGCAAGTGTGTCATTTTTGTCCAGCAGCGTGAGACTGCAAAAGAGTTGACGCATAGGATGCGTGACAAGGGGCATTCTGTCGGCATCTTGCACGGTGCTGACATGGCGAAAGAAGTGCGGGACCAGGTGATTGACGAGTTTCGTGCCGGCACGACGAACGTCCTCATCACTACGAACGTACTTGCTCGTGGCATTGATGTAGCAGGCGTCTCTCTTGTCGTAAACTTTGACATTCCGCTTACCCGTGACCAGCGTCCAGACCCAGAGACGTATTTGCACCGGATCGGCCGAACAGGTCGGTTTGGCCGTAAAGGGTGCGCAATCAATTTCGTCTATGACAACAAGAGCAAGCAGGATCTCGCTGAGATTGAAGAGTACTATGCGCGTCCTATTATCCAGGCCCCTGCAGATGACATTGAGGAACTCGAGAAGTTATTGGCGTGGGGGGAAGCACACGGCGTGCTGCGAAATTTGGCAAGGAACAGCGCAAAGGTTTGCGCATTTACCCGCGCGAGCCGAAAAAGAAGCCGGCGGGCAAGGACAAGACGTACTATTACCTCAAAGACATGCACTTCCTCGCGCACGAGCCCGTGTTGGCCCGCTTTCGCGCGTTCAAGACGTTTATGAAAAAGGTCAAGAAAGCCATGGGCAAGAAAGACTACGCGACGGCGAACCACTTGCATGCGAATCGACCGACGTATGAGTTGGCACACTTGGTCAAAGAACGGTACCCGAAATTTGGCGACGCGTTGAGTGACCTCGACGATGCCTTGTGTGGCAGAATTTTATTGTCGCGACACATGCCTTGACCAAAGTGTTTGTGAGTATCAAGGGCATTTATTACCAAGCCCGAGTCGAGAACGAGACCGTCACGTGGCTCGTGCCCCATCACTTTACACCCACGGTGGACAAGCGCGTCGACGTGCGGGTCATGCTCACGTTTCTCGAGTTTTACACGGTCTTGTTGCCGTTTGTCCTCGCGCAATTGTACCACCATATTGGGCTAGTGTACCCCCCCAAGATCAACTTGCAACTCGATGAAGCAGGTGTGCAATTAGCTGCCCTCGAGCTTGCAACAGAAAACATCGGAAAAGCGGATCAAAGTACCATGCCGAACGCACTGCAGTGGGTACCCCGCGGCTGTTTACGGGCCTCACGTTCTTCCTCTCACGGGAAGTGCCTCTGGCATGTCTTGAGCTCGTCTTAAAGTCCCACGGCGCGACGTTGGGATGGGACGGACCCGGCTCGCCCTTGACCGAGCAAGCGCCGTGCATTACGCACCACGTGATGGACCGCCCGCACCAAGGCCATCGGTACTTTAGTCGCGAGTACGTCCAGCCGCAATGGGTCTTGGATTCCGTCAACAATAAGCTCTTGTTGCCATTAACGCGGTACCGGCACGAACTCACTCGCCTCACGTCAGCGGCGCAAGTGTTGCATGAAGTCAAGGCTTTGGGGGCGGAGTCGGAAACGCAGCGACGTATGCCGCAGAGTTCAAGGCGGAAGTGGAGGGCCATGAGGCGTCGCGACCTGTCAAGAAGGAGGACGAAGCGACGGAATTGAAGGAACTGGCCAAGACCATGATGTCCAAGAAAGCCAAACGACTGTACGATCGCATGCAGTATGGCCTCGGCAAGAAGGCGCACGCGATTCAGACGCTGCATGCGAAACGGAGCAAGCTCCAAAAAAGACGAACAAGCTACTGGCTGGTGCAATTTCGGGCATGGTATCAGCACTGGCATTCCAACCTCTGGGGCGCTCCGATGGCGTGGCACCTCGCCCACTCTCGTTCGCGTGGCTGGCGGTGCTGGGCTGTATTTCGCCACGCTGGATTACTGCCTGAACATGTTTCCGCCGTCTCCAGTCAACACTTTTTTGGCAGGCGCTTTTGCGCGTACTTTTGCCGGCGGTATCATGTCACCTTTTACAATAATTAAAGCGCGTATGGATGCCGTCTGGTACTTTTGACAGTAAAGTGACGTACCATTTTCTGGACTGTATGTGCTCATATACACGGCACCCGGCCGATGTGATTAAGACACGCATGCATGCGACAGACGATATCTAAGATTTATCGTCATGAGGGTTTGCGAGGCTTTGCGAAGGGCATCGTGCCGCGTGTTGTCAAGCGGACGCTGTCCACTGCAGTCACTTGGACGATCTACGAGCTACTCATTTGATCCGTCAGGTCTGGGCCACGCCAAGGAAGCTTTTAGCCTGGCAAAGGAGACGGAACGCACAAAGCAAATGGAGAATCAGGCTAAGATTAAGGAGAACGAGGCTCTTTACAAGCAATACGAGATCGTGCGCGTGCAGAAGGAGGGTGAGGAACGTCGCAAGACGCTTGAGGAAGAAACACGTCAGCATCAGCAACGGGCGCAGTACCAGGATCAACTTAAGCGCAAACAGTACGCTGACCAGCTGGCTGCCCAGAAATATATGAAAGAGCAAGAATTGAAAAAGCAGGAGGAGATCCTAGCCCGTCAGGAGGCCTCGCGTCGTAAAACGCTTGACTATGAGGCTGAGCTGCGTCAGAAGACCGAGCTGGCCAAGGTTGCGGCCGAGACTGAGGGTCGTATCAAACAGGAACGTCTCAACCACGATTTGCACCTTGAGGAGACGCGTGTGCATGCAAAGGAGTACCGTGAGACCGTCCTGGAAGGGATTAAGCTGGCTGGGAACACAGTTGGATCGGGTATAATGACTTTTGTCGATGACAAAGAGAAGCTGACGACCACTGTAGCATCGTTGACGGCCTTGGCTGTTGGTATCTACACTGCTAAGGTGTCTACTAGTGTTGCGGGCAAATATGTTGAAGCTCGGATGGGTAAGCCGTCTCTTGTGCGTGAGACATCGCGTCGCTCTGTTGCGCAGGTATTGGCTAATCCCATTCAGTCTATTAAGCGCGCTTTGCGGCTTCAAAATGCAACAGATGCTCTCGAGGGTGTCGTGCTCGAGCCCAAATTGGACGAACGTCTACGCAATGTGGCTGTGTCTACCTTTAACACGAAGAAAAACCGTGCACCTTTTCGCCACTTGCTGCTGCATGGCCCACCTGGTACAGGCAAGACGTTGTTTGCAAAGGCATTGGCCCGTCACTCAGGTCTCGAGTACGCCATCCTCACGGGTGGTGACGTAGCTCCGCTGGGTCGCGAAGGCGTGACGGAGATTCATAAACTTTTTGACTGGGCGTCACACAGCCGTCGCGGTTTGCTTCTCTTTGTTGATGAGGCTGATGCGTTTCTTCAAAAACGTAGCAACACCGTTATGAGCGAGGATATGCGAAACGCACTAAATGCGTTTTTGTACCGTACGGGCGAGGCTAGCGACAAGTTCATGATCGTATTCGCAAGTAATCAGCCAGAGCAATTTGACTGGGCCGTTAACGATCGTATTGATGAAATGGTCGAGTTTCGTCTGCCTGGTTTTGATGAGCGTGTGCGCATGCTAAATCAATACTTCAACGAATACATTCGTGCACCGAAAAATTCGCGTGCTAAAGAGATTTACGTGAGGGAACTATTTTTACGCTGGACCCCGTGGCCAACTTTCTCGTGCCCGCACACGGGTAATTAATGTGGACGCACTGCAGGGTGTCACTGGGGAAGCCCAGGCCATTTTTGATGCCCTGAACAAGATCCCATGCGTATCTATTGGGTCGTTTTTGCTCTGGTGCTAGGGAATGGCTTGTACACGTACTTTGCCGGGAAAGACGAGTCGTTTCTGGTAGAAAAGCTTAAGAAAAAGATCCCGTCTGAGTACCGCATTTCCGAGAAGTGGGATAAGTGCTTGGAGAATTTCGCTCTCTACTTTGGGGCTGGCCTTGTGGCTGGAGGTGTGACATCAATTGTATTATCGCGATCTGGAGCCGGTCGTGGACTAATAACGGGCTTTGGTGTTCAGGTGCCGGATCAAGCTGGACGACGTGCCAGATGGCGTTTGCTGGTGACGCACAAGAAGCTCTGGACAAGGCGGACAAGGCCGTGGAAGACTTGCCAGCCTTCCAGCAGCAGACGATCGTGGACTTTGTGACGGGCTCGACCACGCAGCAACTACTGCAAAATGTGCACTCGAGGCTTCTGTGAGATCGCTAACGTTAAGATGCTCGAAAGGTACTTGATTGAGCAGCTGCAAAACAAGACACTGGACATCGATGCCAACCTGGCGTTGCTCAAGCTGTATCAGATATATCCGGCAACGGCAAGCGCCGAGAGCCAAGGTGCTGGTAAAGGGTATAATGGCAATGCCTTCTACTTTCTTTACCGGTGACCACGATGATTCCGGAGAACATTCGTGAGGATGCTAATGTGGCGACAATGCTGCGCACTGGTTTTATGCTCCAGTCGTGTTTATTTGAGGAATTTTGGAAAGAAGAAGTGTCTCTCGCCGAAGAGGTGCCAGGCTTTTTGGAGTCGGTACGCGCCTACATTCTGACGGCCGTTGGCCACAGCCACAGCGTCATCTTCATGGATGTCTTGAAGGCAAAGCTCAACGTGTCCGAAAAGGAAGTAACGAAGATTGCCGCCGAGAAGTGGACGTCGACAACTTGATCCATCTCAGCCTTAATGTGGACAATCAGATGCAAGCCAAAAAGGTTCAAGAGAACGTCGAGTTCGAAGACGTGCTTCATGTCATTCACACCCTTTCCAGATCTAACGTTTGCGCGAGCGGTGGTGGAGTGGCTGACGCAGAGGAAGTTTGTGAGAATCTTGCCAAGCACTTGAGACTTGAGGGAGGATCCCAAGTGCTGCCTTGTTCCACGGGCGTCATTGGCTGGCGTATTCCCGTGGATGCCATTGTCCAGAACCTTTCCAAACTTGTTAAAAATTTGCAATCAGATTCGGTACTACCAGCTGCTGAGGGGATCATGACCACCGACCTGTATCCAAAGATCCGCTCAATGGACGTATGTGGTGGCCGTATCGTTGGAATTGCCAAAGGTGCTGGAATGATTGAACCCAATATGGCCACCATGTTATCGTACATTCTCACTGATTTGTCAATACCCCGTGACGTGTTGCGTCAGCTACTCACGGAGGTGGTTGACGACACGTACAACTCGCTTAGTGTTGATACAGATCAGAGCACGTCCGACACAGTGACGCTCGTTTCGTCCGATCAAATTCCGTTTGACATGAA---GCAGTTAAGCAAGTTTAAACAGGCGTTGCACGACGTGTGTCTTGGATTAAGTGAGGATGTTGTACGCAACGGTGAAGGAGCGCATCATGTGATACGAGTGCGTGTAAGTGGCGCGTTGTCTAAGAAAATGGCTAAAGGAGTCGGCAAATCGGTAGTCAACTCACCTCTGCTCAAGTGTGCGGTGGCCGGCAATGATCCAAATGTGGGTCGTCTCGTTATGGCTGTTGGCAAGTACATGGGGTTACACCACAAGGACGTGAACATTCAGCGACGTATGAAGATTCGAATGGGTAACGTTTTAATCTTTGAAAGCGGGGAAATGGTACTCAACGACGACATTGAAGCCAAACTTGTGGCACACATGCGCGACGCGATGCTGATTGAGCCCACGCGTGGTGGACTCGATGCGAGCTCGCACAACTATCCGCCACATGGAAAGTTTGTCGAGATCGAGATTGACTTGGGGGTTGGCTCTGAAGATGCACAGGTGCTCGGAATCGACCTCACGCACGAGTACGTGGCCATTAACGCCGATTACCGTTCGTAATGGCGCC---AAAGAAGGATAGAACCGCCGCCGCGTACCTGGCATCGTTAGGCGTGGCTTCTTC------------CGACAAGAAGGATGAC---AAGAAAAAGAAGAAGAAGGGCAAGAAGAAGCCTACAGGAAACCCATGTCAGCTGCAGCAAAGGCCTTGG-TGGAGCGCCAAATGGCGATGAAGGCCGAGCAGGAGCGTCTTGAACAGGAGCGTCTTGAACAGGAGCGCATTGCTCAAGAGGAAGAGGCTAAACGCTTGGCCGAAATCGAGCGCGAGGAAGAACTTCGTCTCAAAAAGAAGGAGGCGCGTGCTGCCAAGATTGAACGTCAGAAGAAGGAGGGAACGTACATGACCAAGGCTCAGAAGCAGAAAGCGGCGAAAGCTCAAGCTGCACTCGAGGCCATGCAGCAGGCAGGTCTAGTACCCGAGACCCTT------GATGAAAAGAAGAAAGCTGTGTATTCAAACCACAAGAAGAAGAACAAAACCACGAGGTTGATGGAGACGATGAAACCGAAGCTCCCGATGCGTGGGACGATGAAGACTGGGAGAACAGTGACGTTGTGGCCTCGCTAGAGGACAAGCTAGCAGCAGAAGGAGCAGGAGCGTTTGCGTCTGCAGGGCATTGCATTGAAAAAGCGTCAAGAAGAGCAGGCTGAGCAACTGCGTCTGGACGCTGAGGAAGAACGTCAGTGCTCTAAAGCTAAGCTCGAGGCTGAGCGCAAGAAGAAAGAAGCAGCCGAGCGTCGACGCAAGCGCGAGGTCGCGGCGCGTGCCAGCGCTTCGTTGGACAATTTGCGTTCGCCTATTTGCTGTATCATGGGTCACGTTGATACTGGAAAAACCAAGCTTTTAGACAACATTCGTCGGACCAAGGTGCAGGACGCGGAAGCCGGTGGCATTACCCAGCAGATTGGGGCGACCTTCTTCCCTGTGGAAGCTATCAAGCAAAAGACGGCCCGACTTGCTGAGGTGATGGAGCTTGAGTACCGTCTGCCTGGTCTTCTTGTGATTGATACTCCTGGTCACGAATCGTTCACGAACTTGCGTTCCCGTGGCTCATCGCTCTGTGATATTGCCATTTTGGTGGTTGATATCATGCACGGTCTAGAGCCACAGACTCTGGAGTCACTGCGCTTACTGCGCCAAAAGAAGGCCCCGTTTGTGGTGGCGCTTAATAAAATTGATCGTTGTTATGGCTGGAAAACTATGCCTGATATGCCCGTTCAAGAGGCACTCAAGCATCAAAATGAGCACGTTATTCGCGAGTTCGAGGATCGAATGAAGTCGGTCATTGTAGAATTTGCTGAACAAAAGCTGAATGCAGAGGTATATTGGCGCAACAAAGACTTGGCACACACGGTCTCGCTCATTCCTACCAGTGCCATCTCGGGCGAAGGTGTGTCAGACCTCTTGATGATGCTCACGCGTCTGACGCAGGAACGTATGGCCAAATCGCTTGCATTTGTCGACATTCTTCAGTGTACTGTGCTAGAGGTGAAGGTCATTGAGGGTCTTGGTACAACTATTGATACAATTTTAGTGAATGGAACCCTAGAAGAAGGGGCAACAATTGTTGTTTGCACTTTAGATGGCCCAGTGGTGACAACAATCCGGTCGTTGCTTACCCCGCACCCTATGAAAGAGATTCGTGTTAAGGGCGAGTACATCCACCACCAAAAGATGAAGGCTGCGATGGGAGTGAAAATCTGTGCACAGGGCCTTGAGAAGGCCGTCGCTGGCACACAGATTTACATGGTTGGCCCCGATGATGATGTGGACGAACTAAAAGACCTGGTCATGTCCGACCTCACAGGTATTCTAGACTCTGTAAAGGCTACTCGCCGCGGCGTCATGGTTCAGGCTTCCACATTAGGTGCCTTGGAGGCGCTATTGGAATTCCTTCGCACCTGTGATCCGCCCATTCCAGTGTCTTGCGTCAACATTGGTCCCGTTCACAAGAAGGACGTGATGCGTGCTTCCGTGCAGCTCGAACACCAGCCCGAATTTGCTACCATTCTGGCATTTGATGTAAAGGTTCACCATGATGCCACCGAGCTTGCGACGGAACTTGGAGTGCGTATTTTTACAGCCGACATCATTTACCACCTGTTTGACCAATTTACTGCATACATGGACAACTTTCGCATGGCCCGTCGCGAGGAATTTGCGGACGTGGCTGTATTTCCTTGTGTGCTTAAAATCCTGCCCAATTGTATATTCAACAAGAAGGATCCCATCATTCTCGGTGTCGATGTTGAAGAGGGTATTGTCAAGATTGGCACCCCTCTCGTAGTGCCATCTGCCGGCT---CTTTGTACATCGGCAAGGTAGGGTCCATCGAACGCGAGCACAAGGAGGTGGACCGCGCTAAAAAAGGCGCGTCTGTAGCTGTGCGGATAGACAATGAGAGCAGCGTCATGTACGGTCGCCACTTTGACCACAAAAATCAGCTTGTATCGCGTCTCACTCGCGAGAGCATCGACGCTTTGAAAGAAAATTTTCGGGAAGATCTGCAGAAGGAGGACTGGCAACTCGTCATTAAGCTTAAAAAGGTGTTTGAAATCATTATGGAGGGCTGGCTCACGAAGCGTCGAGACCACATGAATCTCATCTGGTTGGATCGCTACTTCCTCCTCGATGGCAATCAGCTTCGCTACTACCGCAAACGTGGCGACCCTGCGCCGCGAGGGACCTACATTTTGACCGACGGTTGTGTTGTTAGTCCTGTCTTTAACTCTGAGGAGAAACATAAGCACCACGGGCCTGTGTGGATGTTCCGCATCACGTGAAAAATCATACAAGAAAGATCGATTTTTGGATCTAGGAGCCAAGAGTGAGGAAAATGCCAAGGAGTGGAAGCGCGCACTAGAGAACGCCGTGCGCTCGTTGCGGGAGCTCGTGGCGCACCAGAAAAAGCTTAAGACCGACGCCGAAAT---TTTTCCCACGCCTACGCAC---------GTTCCGAGGATGTTGAAGGCTGATGAAGACACCGTGACGCTGTTTAATCTTGACGCAGACAATGTAAAGGCAGAACAGGCGTGGTGGCTGGCTCGAGTCGAAGGCGGACTTCGCATCATGCAGGAATGTCCGCTCGGACCGAATGTGGCCGCAAATCTGTATGCACAACACGCGGCTACAATGAACGGATTGATAGTCAATGCCTGTCTAGGTGCAGCACTGAGTTTTAGCACCATGTATTCCTTCGCGAGCAATATGCTGGTGAGCTTCATGACAGCACTCGTGGCCATCGCCGTGTGTTGCGCAGCCGTGACGCCGAAACAAGCGCCAGAAATCCCAAGTTTACGTGTCTCGCAGGTCGTATACGGGTCTCCAACGGAAGCTTTCCGTTTAATCATGAACACTAAGCGATTCCAGCGCTGGGATGCAGCAACAGCGACCATGCGTGTGGTACAGCAGCTGGACGATCACGCGGACATCGTGTATCTGACGCAACGACCGACGCAATTGTGGCCATTTTGGCAAAAAGCGCGGGACCTTGTGCTCATGCGCTACTGGCGTCGTGAAGAAGACGGATCGTACTTTGTCATGTACCAGAGTATCGACCATCCTGAGTGTCGGGTTCGGCATAATTACGTGCGTGCAAACTTTCTGGGTGGTGGCTTCGTTATTGCTCCTCAGCGTGTTCCAAGT---GGATCGATCCGTACACTGGTAACGTATGTGCTTCGGTACGATCCGGGAGGTTG---GTCCCGTATTTACCACCAACTTGGCATGGACGTGGATCTAGTCATGCCGATGCTGCGCAGTGTTGTGGGCATTCGTGATGAAATGAGCGCCACGGACTTTATCACGCCCAACGTCTCGATGGCACCTGCTGGAGACGGCGGAACCTGGTCAAGAAGCGTGGA---CTCAACAGGTGTCGTAATGGGCATCCAGAAGCTAGAA---ACGAGCCTTCCAGAAAGGATGTGGGCTGAGCCTGACGCTTCTCTTTTCAGCGTCCGGGGACACAATTATTTGACGGACAAGAAAAAGATCCCGTCGGCACCTGCAATGTTTCACACTGTGGGTGTGGATCTGCTGTCGTTCGAAAGTCCTGCAGAGAGGTACAACATTTTGTCTCGTCCTGACAGTATCAGTCGCAACAG---CTCCAAATTTACATTTGTCGTTAACATGATCATTCCAAGTCCTGAGAATGTTTGCATGGTCTTCTACTTTCACCCTGTGCGTGACAATGTCTTTGAAGACGGCTCGGGCTTTTCAGAGCTGTTAAACGACTTTATTGATGGCGATGACCAATTTCGGAACTCACGTTTTAAGCTCATTCCAACTGTTGTCGAAGGCAGCTTCATTATTAAGCAGTCGGTCGGTTCCAAGCCCACACTGCTCGGAAATAAGCTCAAGTGTCCGTACCATCGTGGAGAAAATTATTTTGAAGTGGACATTGATATTTCGTCCAACTCAGTAGCTAACACGGTGGTAGGCATGGTGCAAGGTGTGACCAAGTCGCTTGTGGTTGACCTGGCGTTCCTGCTCGAGGCACAGTCCGACGAAGAGCTGCCGGAAGTCATCCTTGGTGCAGTTCGCATGCAACACATTTCGCTTGACAAACCACGTCGCATGCCAGCGCATGGGGAACGGGCAGCAAGGACGAGTACACCTTTTGAGGCCTTCACCGAGGCTTTGCGCGTGAATCTGCAGCGACTTATTGTTTACGCGCGCTCAGCTGACACGGCGTTGCAGCGAGAAGTCGCCGAGAAACTGGCTAATGAAGCTGTTAAGACCGACAGGTGCAGATTGTGGAGCTGGACGGACTGCAGTTGTTACTCCCGCTGACAAAGTCGAGGGACACGGAAGTACAGCGTCTAGCAGCGCATGCACTTGCTAATTTATCTGTCAACTCTGAAAATCAGTCCAAGATGGCAACAGAAGGAGGCATTGACATGCTAATTGATCTTTTGGGCAGTACAAACGAACACGTGCAGCGCCAAGCCGCCAAAGCGCTTGCCAATCTTGGTGTTAATGTGGACAACAAGGAGCGGATTGCAAAAGCGGGTGGTATTAAACCGCTTATTGATTTGGCCAGCTCCCGTCAGATTGGCGTTGCCGTCGAAGCCATTGCCGCGCTTGCAAATTTGGCAGTCAATG---ATGCCAATGAAGTCGAGATTGCCCGAAAAGGTGGATTGAAGCCCATTATTGATGGAGTGCACTCTGAATCGATCGAACTTCAGTCTCAAGTCGCACGTGCATTACGCAATCTATCGGTCAATCCCGAAAACAAGCAAGCGATCATAGAGCTGGGCGGAGTCGAGGCTCTTCAGTCACTCGTGCGTGTTACCAGCGACCGAATTTGCCAGCAAGCTACACGTGCGCTCGTCCCCAATGTTACGCTCACGCGCTCCTCACCCACAACTTGCTA------GTAGCGCCCGTGGCCCACGTGGCCGAAGTTAAAGAGGAGGC------TGTTGCCTATGATGGTACCATGGTCGACGTCGAGTTCTCCACCACAGTTGAGACCGTC---------ATGGTGCCTCTTGCAACCCCGA---CCAACGCGGCCAAT---GGCTTTTTGTCTATTTCGGATCGGTTTCCTTTATCCAAGGTGGGAAAGGTCAGCTTTAATATGGCTCCAGTTCCTATGAGCCG---CTTGCTGAATG---GTACACCTGAAATGACAC---AAGTCGAGCAG-------------ATGACTGTGCTGATAGAAGATGGCACAGTAGATGACAAC---ACGATCAAGGCTGAG---CCAGAAGGTGAGTGATGTCTACCAGTATCAAGCTTCTGA--TGTAGAAGAAAATGAAATGGAGCAACTTCGATGGAATATACAAGAACAAGTGTTTCAGCGATATTTTCATCAAGCTCAGAAGTATGAAGAGTGGGGAAGATTAGAGGAGCAAAACACCGAGTTTTTTTTGTGCCGGGTGGAAGACGGGAAAACATTAAAGCTGAGAAGCGGTTTCATGATAATGCTGTTCGCGATGCCATGGGAAAGGGAAATGCCACTTGGGTGAAGGAACGACGGGATCGGTTCGAAGAGGAAATTCGACTATTTTATGACGAGACAAGGCACAAACAAGAGTTGATGCTTACGCGACTTGAAAAGGTGTCACCATTTTCAAAGTTTCCGATTCTGGGCAATCGTTTTTTACTGCTTCGTTTACGAGGAAAAGGAGGCAATGGAGAGGTGTGGGATGTTGTTGATTACGCTAATAAGAAGCAGATTTGTGCTTTAAAGCTTTCCACTTCAATTCGACACGCCCAACGCGAACACCATACGCACACTTAACCATCCAA--ATATTGTCCAAGTTGGCGAGATGCCTTTTCTCATACGATATCAACAACAGCAGTACACTGCGTTTACTGTCGCAAGCGTTCAGTCAGATTTGCAACAACTTATTGAAG---TATACGAGCATCTAGATGATGACTCGGCTTACAAGGTGCTGTCTCAGCTTTTAAATGCATTGAGATATTTGCATGATTGGCGTTGCTCACTACGACCTCAAGCCGTCGAACATTTTGATTGACCACGACGGGTGTGTGAAGCTG------ACTGATTTTGATTTGACTCGCGACGCCAAGGCAGCTATTTTCAACTCCACCGTTGGCACTCTGCGATATCTCCCTCCGGAGTGCTTTCGCCCAAATCGAGGCGA---------CTGTGAAGCCACTGCTGAAAAGGCAGATATGTGGATGGTGGGCATTGTATATTGCATGATGGTGACTGGAAAACACCCTGTCTGCCCGGACAAGTCGACACACGTGGAAGTGAAGACAGTGATGGCTCAATACACAGGT---GAGTTTCGGTATGCACGGCCTGTTTCCGATTTGTCTCGGTGGATCATGCAGGGGTGCCTTCACCCGGATCCGCGGTGTCGACCGACAGCTGCCCAGTTGCTAGTCGCACTCCAA

Plasmopara_viticola TGGACTACCACCGCCAAGTGGCAAAAGAGCTGGCCGCTTTTTTCCACACATTCTATCGCTTTGGTGCCGCGTCCAACCGCTTTGATCTTTTTTTACACAACATCCTCTTGGACTCCATGTGCATCGGAGGCGTCGTCTTTCTCGTGCTCTGCGTGATTGTGGTGCGCCGCACCGTCCTGCACATCCGAAGGCCTTTGTCCGACGCGCTTCGATGGATTCTACGTCCGTGGAGTTGTTGGCAGTTGGCCTTTTGACTTTTTTGGCTATAAGTGCGTTAGGTGGTCTCTTAGGAGAAGCTATCTACAGACTAGCAATGCTTTGCGTCATAATGCTGACAACTTGATTACATCATTCAACGACAGTGTTCTGCCAGCTGATGCCTTTAAGATGTCAGTAGAAGCGCTGGACTCGTGCATGCGTCAAAGGATCTGTGGAACCAAACTGACGTGCTACTTCCACAAAACTACTCAAATATTGCCAAGGACTGGGAATTTAGCTATTTTTTACTAAAATCATCGACCAATGGCGCTATATTGATGGTCGCACTTGCCAGTTTCCTGTCGATTGCGTCGGTTGGATGGGCCATGGTCTCGCCACTTCGTGTCGCTATTCTGATTATTTTGAGTGTTGTACCCATTTCCCATTCGCTAATTGGGGCCTATTTGTCGTCAACAATCATGACGGCAGATTTTTGTGCCGCGCCAATGAATAACACTCTTGGATTGATTGGCACGTGCCCTGCAAACGCTTCTTGCCGTTTGCCGACGCAATGGTGACGCAATGCGTCACACCAGGGAGAAATTGGTCAGCGAATAAAGCGCGAGTTCCTAGATCCGATCGGAGCAGCTGTGGGGGTCATGTTTGGGTACTTTGCGGGTACAATCAGCAATTGGGAACATTTTGCCTTGATCGAAACGTGGCGGTGGCCCTTTTTGCTCCAATTTGTGGCCCTCTTGCCGCTAAATGGGGCCATTTTTTTCGTGCCTCAAAAACATTTAGTCATTCGTATGGTGAATAAGAAATCCGAGCCTCCGGTTGTTGGGACCCCCGTTAGTACGCGCTCGACGACGTACCACGATGAAGAAGATGCGTTGCTGGCCAGTGCCAGTATGCGACCTATGGTGCTTTGGATACGAAGGGTTCGAACAGAAAGCGTTCGCGGAAGGACTGTACGACCTGCTGCACATTCCCGTCTTTTGCTTGATTGTGTTCGGCCTCACGACCGTCTACTTTGTCGTGACGGGAGTCCAATATTGGAGTACAATCTTTATGATCAAATCGCTCCATGCGTCCAAGTACTTAGTCAACGGCCTCTTTGTCATCATCTCGGGGACGGGACCAATCTTGGGCGTGTTCTACGGCGGCTGGTTGATCGATCGCTACGGCGGCTACATTGGCGTGGAACAGCGGGCCAAAGCTCTTGGGATTTGCATGATCCTGGGCCTCACGGCATTTGCCGTCTCGGCCGTGACGACGTTCTTCAACGACATTTACGTCACTGCCGGCTTTCTCTGGCTCATGCTCTTTTTCGGAGGCGCCATCCTGCCAGCACACCGTGCCCTCGCGTCGTCGTTCTCCGTCATGATTTTCAACTTGTTTGGCTACGCCTTGTCGCATGAGTGACTTACAGATGGTGACGGGTGCCAAGTGTTTCGTGCCAGATGAGACTCACGTGTGGCTTGCGGCCGAGGTCCTTCGCGACGAGAACGCGGGCGACGGCTTGGCGCGCAACATTTACTGCAAGGTCGAACTGCCCGATGGCGATTGAAGAGCGCTGTGTCAACTTGTTGGCAAAAAAGACAAAGGCGTTGTTGCAGTCCATCAACTGGAATCCCTGCCGTACCAGAACGAGAACGTGGGACCCGAGGGCATTGAGGACATGATTACACTGAATTACCTGCACGAGGCCGCCATCCTCTACAATGTCAAGAAGCGTTTCCTATGCGAACTTCCTTACACCTATACGGGGGACATTTGCATCGCCGTGAATCCGTACAAATGGCTCCCGGATCTCTACGAAGAGAAGCAGCACCTTCGGTACCTCAACCAGCCAAAAGAGGAACTCCCGCCCCACGTGTATGCCACGTCCGTTGCTGCCTATGACAACATGCGGCGCTCGGAGCAAAACCAATCCATTCTCGTGAGTGGAGAGTCGGGTGCGGGGAAAACGGAGACGACCAAGATCTTGATGAACCATTTGGCCACGATTGCCGGCGGGCTCAACAATTCCACGATTAAAAAGATTATTGAAGTCAATCCGCTCCTCGAGTCGTTTGGGAATGCCAAGACGGTCCGAAACGACAATTCCAGTCGCTTTGGCAAATTTACCCAGTTGCAATTTGATGGGCGTGGGACCCTGGTCGGGGCCAAGTGTCGCACGTATCTCTTGGAAAAGACGCGCGTTATTCATCACGAGGCCCCCGAGCGCAATTACCACATTTTCTATCAATTGACTCGACGGACGTGTCTGCCGAGCTGCAACTCGATGCGTCCAAGGTGTACGCGTATACGGGGGACAATCGGGATCAAAAGATTGAAGGGTTGTCGGATGAGAAACACTTTAACAAGACACGCGAGGCCCTCGAGCTCGTGGGGCTGTCGCGGAATGACCAGCAACCACTGTTTGAAGTCTTGGCGGGTGTCCTTCACCTTGGAGAAGTGCAAGTGCAGTCGGACGCGACCGATGACGAAAAGTCGTTGATTCCCGAGAATGACGAGGGAGCGACGTCTGCGATTCAGATGCTTGGTGTCACGTGGGAAGCCCTCCAGACGGCCTTGTGCTCCCGTACCATGCGTGCGGTCAACGATGTGTATAGTGTCCCGCTCAAGAAGGAACTGGCCATGGACTGCCGGGATGCCCTTGCGAAGGCTATCTACTCGAACGTGTTTGACTGGCTTGTGAACACGATCAATCATTCACTTGCCGATGATGGTCATATGGTGAATCACGTGGGCGTGCTTGATATTTTTGGCTTTGAGCACTTTAAACACAACTCGTTTGAGCAATTCTGTATCAATTTTGCCAATGAAAAACTACAGCAGAAATTTACGCAAGATGTGTTCAAGACTGTGCAGATTGAGTACGAGGAAGAAGGGATTGTGTGGGACCACATTGAGTACGCTGACAACCAAGACGTGCTGAATGTGATTGAGTCCCGCATGGGTATCATTTCACTCTTGAACGAGGAGGTGATGCGACCAAAGGGGAATGAAGAGTCGTTCATGTCGAAAGTAACTTCGTTGCACAAGGAT---GA---AACGCACGTCATTGAGTTTCCACGTACGTCGCGTACCGAATTCCTCATCAAGCACTATGCTGCTCCTGTGATGTACGACTCGGTCGGATTCTTAGAAAAGCACAAAGACTCTCTGCTTCCTGACTTGTCTGAGCTAATGCGTGGCTCCTCCAAACCGTTTATTGCCAAGCTTTTCGAATCAAAGCCCGAGCCAAGTCCGCCGAAAAC---------GTGGTGGTGCGCTGTCCATCACGACTGTTGGTACGCAATTCAAAGAAAGTTTGACGGAATTGATGGCTACCATTAATTCAACCCGTGTGCACTACGTACGTTGTATCAAGCCCAACCCGATTAA---------GAGTGCAACTGTGATGGATCAAAACATGGTGGTGTCTCAATTGCGATGTGCTGGTGTGATTGAAGCCATTCGCATCTCGCGTGCGGCGTACCCTAATCGGTTGCAACACACGGAGATTTTGAATAAGTTTTGGCTTTTTGTGCCAAGTGGTGGCCACACTGTAGAGGAAAAGTGTCAATTGCTTATGGACAAGTTGAAGCTAGAATCCCCTAGACAGTACCAGATGGGTAAGACCCGCGTGTATTTTCAATTGGGCGTGCTTGAAGAGCTCGAGGATCGACGCAAGAAGTTTCTGGATGCGAAAGCGACGTATCTGCAATACACGATGCTTGGCTTTACGCAGCGAATCAAGTATCTTCGAAAACTTGAGGCAATCATCAAGTTCAGTGATTCGGTGCGTAATTGCTATGCGCAGATACAACACGTTTCTCAAGGGCCTCATCCTGGCACAAGCTCGCTGGCGAGGCATTCAGGGCCGCAAGGTGGCTGCTGACGTGAAAAGTAACCACTATGCTGTCATTATTCAGCGTTATGTGCGTGGCTTTGTAAAGCGTCACCATTATGTCAAGATGCGCGCAATGGTTGTCCGTGTGCAAGCCTTGGTTCGTATGATGATTCAGCGGCCAAAGTATCTGGCTGCTCTTGATGAAAAGCGTCGTGAAGCAGACATGGCTTATCAGCTTCAAAAGCTCAAGGCTGCTTTGCATGACGAGCAAGAGCGAAATGCTCAGCT------GCAGCGTC---GTAACAGTTTGAC--GACAGCGCGTCTGCGGCCTCGAGCGTCGTGATGGCGGACGCTGGCGGTATGATTGAGACTCTCACGGACGAGAATAAGAAGCTGCGTGAGAAGAATGAAGACATGAAGGTTGCGATGAAAGGTTTGAAGGCAGAGATCAAGAAATTCATGAATGACAAGGAGGTTTCATCGGCTGGCAATCATGTGAAATTGCGCCAGCTACAGGATACTGTGCGTGACAAGGACAAGAAGATTTCACAGCTTGAGGCGGATAATAAGAAGCTATCGGAGCAAATTACGAAGCTACAGACGGAAGGTGTTGT---CCCCGATAAAAAGTCCATTCCGAAAAAGTCGATCTTTCGCACACTGGGCTCGAAGAAGGAAAACAAGCCGC---------------GTGAAACCGTGCTGATGGACTCGCTAACGGGATCGGATGACTTGAACCGTGGGGAAACTCGGCCGTCAGA---ACGCCACTTTCCACGCATGC---TGAGCATGAATGTGGGAAGT---CGCTTTTGGAACTCGAACAAA---AAGGGGGAC------------AATGGTGATGGTCTTGACGTGTCTGAGGTTGAAGGTGAAGACAGCCCCACGAATATACACGCAATACGATTGTAAAGAGCATGGAAGTGGGGGTGACGGGAGCCATGTCAAGCCTCAAGGGCCGCATG---------TCGGTGGTAAAGGGGATGTACGAAGCAAGCGTACAAACAGGATCGTCCTGTTCGCGAATCATTTAACCTCGATGCGTTGCCCGAAGTGTCTTTGCCCGTTGGTTGGGAGGCCAAAGTGTCTCGCTCCACTGGCCGCGTGTACTACGTCAATCGCAAGCTGGGTAAATCGCAGTTTGAGCGCCCGACTCTGGCCTCTCTTAA------GGCACTGAAGCTGGCGCGTCAGAAGTCAGGCAATGCGCCGTAAAGCTGTCGTTGCTGATTTTGGCTGTGGGGATGCGAGACTGGCCGAAAGCGTATCGAACAAGGTGCACTCGTTTGACCTGGTGTCTATTGATATTGCCGTATATTGCTTAGCGCTCATGGGAACGAATGTTCGCGAATATGTGCGTGAAGGGTACCGTGTGTTGCGACCTGGAGGTGTGCTGAAGGTTGCCGAAGTGAAAAGCCGATTTGAAAGCGAGGCATTGGGAGGATGGTAGCCGTCTTTGTGACCGCGACAAACGGCGTCTTGATCTCGGTCAGGAACACTTCCGTGCTCGTGGAGACATCAAGTATGTTGTCATTATAGGCCAAAAGACGGGCGAGGCCATAGCTGTGCCTTTTCCAGACGTGGACCGCGATTTAAAGACGCTGCTGGGCCATACTACATCAATGATCACCCACGTGGCAGTTAATTTCGTCACTTTTGTTGACTGCAGACCGCGACGAAAAGCTTCGTGTATCGAGATTTCCAAATACTTCGATTATCGAGAGCTACTGCTTGGGCCACGCTGCAAGTCTCACTAAGGTAGCGTGTAGTTTGGTGACCCCGGAGCTCGTAGTATCAACGTCTATGGACAACACGATAAAGCTGTGGCAGATGAAGACAGGAAAGTTGGCCATTTGCCCCAAGACAACATTGTGGCCGTGCTTGTGAATCATCAATTTGTGCGATTCTTTTACATCGCTCCCATCCATTTCTCAAGCTTTTTGCAATATCAAAGGGGGGCCTCCGCTTTCGACCGCTGTGCGCGATTTTCGCAAGTTGCAGAATGACCCGCCATCGGGCGTGTCGGGCGCCCCTATGGACAACAACATTATGCTTTGGCAAGCCGTCATTTTCGGACCGGACGACACGCCGTGGGAAGGAGGGACCTTTAATTTGACGCTTGAGTTCAGTGAAGACTACCCCAACAAGGCGCCGACCGTCAAGTTTATTACGAAAATGTTCCATCCTAACATTTACAACGACGGTCAAATTTGTTTGGATATTTTACAAAACCAGTGGTCACCAATTTACGACATTTCGGCCATTCTCACGTCCATTCAGTCGTTGCTGTGCGATCCCAATCCAAACTCGCCCGCTAATTCCGAAGCCGCGCGTCTCTTTCAGGAGAATAGACGAGAGTATAATCGCCGCGTACGCGAAATCGTCGAGCAAAGTTGGCAGGCCATCGATGGTCTCGGCCGCCAAGATGCGTGGCGCTGAGAATCGCATGAACGCGGGACGTCCTTTTACCGCATGGCTTAACAATGTCTCGTCGGACCGTGGTCTCTGTGGTGGAATCAACTCGGGTATTGCCAAAAACACCCGCAAGCAGACCACGCCCAGATCTTTGTAATTGGTGACAAGGCTCGTGCTCAGTTGCGTCGCGACCTCAGCGACAACATTCGTGGCAACGTTACGGAAACCTTTCAGTCACCTCCGAACTTTACAGTCGCCAGTGCCATTGCCGAAGCTATGGATTCGGAGAGGAGTCGTCGCGCATGGCTGCGATGGAAAACGCGACAACCAATGCTCAAGAACTCACGCTCACGCTCGTGTACAACAAGGCTCGTCAAGCACGCATTACGACCGAGCTGATTGAAATCATCTCGGGTGCCGCGTCGCTGGACAGTGACTGGCAGCGTCATCACCGTGCAGAGCGTAGCGCAGTATGAAGAAGCGAGGGCGCGCGAGGCGACGCTCTCCGTGACCTTTTTCTGGGCGGATTTCCCAGCCTGTCGTCCGAACGGACAGCTGGACGTCGGGTCGTTCCGTTCTCGAAAAGCTTGAAGGCGCGAATGTGGCGGAATTGGCCAAGCACGTCTCGTGTACCGCCTTAAGAAGCTGATTACTGCCTCTCCCGTCATGCTCTTTATGAAAGGCAACCCGACCGAGCCGAAATGTGGCTTTAGTCGCCAGATCGTCGCGCTGCTCGACGCCGAAAAGATTCAATTCGGGACCTTTGACATTCTCCACGACGACGAAGTGCGTCAAGGTTTGAAAGCCTATTCCCACTGGCCTACGTATCCACAATTGTATGTAAATGGTTCCCTTGTGGGGGGCGTGGATATTCTCAACGAAATGAAGGCCGACGGCTCGCTTGCTGAGCAGCTCAATGTCCAAGACGCTGACGCTGCGTTTGAAGCGAGCTTGCGTGCCCTCGTGCATTCAGCACCTGTGCTTCTCTTTATGAAACTTTGACATTCTATGTGGAGCTACCAACGTCAGGACCAAGACATCTCGAAAGTCGTGACTTTGACGCCACCTTCGTTTCGTCGCGATATGTGCTGAAGCCATTGCGTGGAGTCCGAAATCGTATGGCCATGGCGTTTCGCGATCCTCTGGATGAGAAAGGAGAGTTTTGCATTGTGCAGCTCCTCGTATCACGTCGAAGTGCACAACTTTGCACTCACGAGGTATCTCTTCCATCGACTGGCAAATTTAATGTGTTGCACCGGGATCACCGCAGTGAGGTCAGGTCGCTCTGTGTCCACTCGAAGCGTGACTGTTTGTATACGGGTGGACTTGATGGATTACTGATTCGTTATGACCTGAACAAGGGGAGTAGTGACATCGTGATCGAGCGTCGAAAACATATCTCAAAGATTAATTCGGTACTGGAACATCCACACAATCCAAACTTGCTACTGGTTAGCTCAATCGAACAATAGGCACAATTTATTGCTACATGACCTGCAGCGCTATGATCGCGAGGTGTCGGGTAGCTCAATGAGTCAATACGTTGTACCACGGTGGTCTCCTGCCGGCTATCATGTTTCTTGTGGCAGCAAATGCGCTACTGCCACGGCAGCTTTTCCACACCCACGTCAGCCACCATCGGGGCCTCATTCTTGCAGAAGCGTGTGATCGTTGGAGGAAAGCTGACGCTTCAAATTTGGGACACGGCTGGTCAAGAGCGCTTTCGCTCCATGGCGCCAATGTACTATCGCAACGCTAAAGCGGCCATCCTGGTCTTTGACCTCCAGAGCGAGGCCAGCTTTGAGAAGATCAAAGAGTGGCTGCATTTACAGCATCATTTAAATGAAGACATGTGGGCAACAAGAGCGACATTTTGACTTTTCCAAGGCCCAACAATTTGCGAATGAAATTGGCGCGCTAGTCTTCCGCACAAGCGCTAAAACAGGTGAAGGAGTGCAATCGCTTTTCGAAACTTTGGCGGTGCAGCTTTTGAAAAAGCACGACAACGAGCAGCGGAGAGCTTCTACGTGGTGTACATACCGACCGATGAAGCCAAGGAGCTAGAGGAGTGGACGATTGAGCTTCCGCACGACAAAGAGTTGCAGCTTGGATGTTTGACTGAGCGTCTGCGGGATCATTTTAAGAGCAGTGTTATCCAAACAGCAACAGAGACCTTCAAGCGGCAATTGCTGACCCAACTGCCTCAAGGTGCCACACTGACGAACGAAATGCTCCAGATGATGCATGGACAGCCTTGTGGACTCGATTCCACTAGTTTTGAACACACCCGACGTTAAGCACGTTAGAGGTAAAGGGTGATGCTTTTATTGCACGGGTATTTGACGGATGACTTTGTGCGCATGGACTTTCGCTTAAGCGAGGTGTCGGGTGATGCACTGTGGGTACAGGCCGCCAAAATGCAGAGGTCACGCAATCACTAGAGGAGATTCTCGCGCTGGGCCATGGACTTGACGACGAGGACTACAAAGCCGAGCTCGAGGTTTTGCAGAACGACCCCAACTCGAACCTTTACTCGGCTGTCACGTTCGAAGAGCTTGCTCTCCCAGAGCAGCTTCTTAAGGGCGTGTACAATATGAAGTTCGCCAAGCCGTCAAAGATTCAGTCAGTAGCGCTCCCACTCATTCTCGCCAATCCTCCAGAGAATCTTATTGGTCAGGCACAGAGTGGGATCCCACGCGTGAGCTAGTGCGACAAATTATGGCTGTTGTCAAGGCCATGGGGAAATTGTTGACACGTTCTTGGCCATTCCTGGCAACGATCTGGCGCGTGGTGAGAGCTTAAAGGCACAGGTTGTCGTCGGAACGCCGGGCAAAGTCGAAAGCATGATTAAGAAGAAACAGCTTGACACGCGTGATGTCAAGATCTTTGTGCTTGATGAAGCGGACGTGATGGTCGCGGAGGACGGGATGCGTGAACGCTCCGTGGCGATCAAGCGTCTCCTCAAGAACCGTGCATGCCAGTATTTGCTCTTCTCGGCCACGTACGCTGACGACGTGCGCGACTTTGCCCAAAAGATGGTGTCCGATCATAACATTATTACAGTGAAGAAGGATAAGCTCACGCTGGATGGGATTAAACAGTTTTGGATTGACTGCAAGTCCCGTGAAAACAAGTTTGAGGTGCTATCGGACATTTTTGCCATCTTGTGCATTGGCAAGTGTGTCATTTTTGTCCAGCAGCGAGAGACGGCGAAAGAATTGACGCATCGGATGCGGGACAAGGGACATTCTGTTGGCATCTTGCACGGTGCTGACATGGCGAAGGAAGTGCGGGACCAGGTTATTGACGAGTTTCGTGCCGGCACGACGAACGTCCTCATCACGACGAACGTACTTGCTCGTGGCATTGATGTAGCAGGCGTCTCTCTGGTCGTAAACTTTGACATTCCGCTTACCCGTGACCAGCGTCCAGATCCAGAGACGTATTTGCACCGGATCGGTCGAACTGGTCGGTTTGGCCGCAAAGGGTGCGCAATCAATTTCGTCCATGACAACAAGAGCAAGCAGGATCTTGCTGAGATTGAAGAGTACTATGCGCGTCCTATCATCCAGGCCCCTGCAGATGACATTGAGGAACTCGAAAAGTTATTGGCGTGGGGGGGAGCACGCGTCGTGCTGCGAAATTCGGCAAAGAACAGCGCAGGGGCTTGCGCATTTACCCGCGCGAGCCGAAAAAGAAGCCGGCGGGCAAGGACAAGACGTACTACTACGTCAAAGACGTGCACTTCCTCGCGCACGAGCCCGTCTTGGCCCGCTTTCGCGCGTTCAAGACGTTTATGAAAAAGATCAAGAAAGCCATGGGCAAGAAAGACTATGCTACGGCCCACCACTTGCACGCGCATCGACCGACGTACGACGTGGCCCATTTGGTCAAGGAACGGTACCCGAAATTTGGCGACGCCCTGAGTGACCTCGACGATGCCTTGTGCGGCAGAACTATATTGTCGCGACCCACGCCTTGACCAAGGTGTTTGTGAGCATCAAGGGCATTTATTACCAAGCTTGCGTCGAGACCGAGACCGTCACGTGGCTCGTGCCCCATCACTTTACGCCCACGATGGACAAGCGCGTCGACGTGCGCGTCATGCTCACGTTTCTCGAGTTTTACACCGTCTTGTTGCCGTTTGTCCATGCGCAATTGTATCAACGGAGGGGGATGGTGTACCCGCCCAAGATCAACTTGCACCTTGACGAAGCGGGGGTGCAATTAGCGGCACTGGAGCTCGCAACAGAAAGCCTCGGAAAAGCGGATCAAGGTACCAGACCGAGTGCACTGCCGCGCGTCCCACGTGCCTGTTTACGGGCCTCACGTTCTTCCTCTCCCGGGAAGTGCCGTTGGCGTGTCTCGAGCTCGTCTTAAAGGCCCACGGCGCGACGTTGGGATGGGACGGACCCGGCTCGCCCTTGGCCGAGCACGCGCCGTGCATTACGCACCACGTGGTGGACCGGCCGCACCAGGGCCACCGGTACTTTGGGCGAGAGTACGTCCAACCGCAATGGGTCTTGGATTCCGTCAACAATGGGCTCTTGTTGCCACTCACGCGGTATCGGCAAGAACTCAACCGACTCACGTCCGCGGCGCAAGTGTTGCGGGAAGTCAACGCACTGGGGCCCGAGGCGGAACCGAAGCCACGTATGCCGCAGAATTCAAGGCGGAAGTGGATGGCAATGAGGCGTGGCGACCTGTCAAGAAGGAGGACGAAGCGACGGAGTTGAAGGAACTGGCCAAGACCATGATGTCCAAGAAAGCCAAACGGCTGTACGACCGCATGCAGTATGGCCTCGGCAAGAAGGCGCACGCGATCGAGACGCTGCATGCGAAACGGATCAAGCTGCAAACAAGACGAACAAGCTACTTGCTGGCGCGATTTCGGGCATGATATCAGCACTGGCATTCCAACCTCTCGGGCGCTCCGATGGCGTGGCACCTCGCCCACTCTCGTTCGCGTGGCTGGCGGCGCGGGGTTGTATTTTGCCACGCTGGATTACTGCCTGAACATGCTTTCGCCGTCTCCAGTCAACACTTTTTTGGCAGGAGCATTTGCGCGCACTTTTGCCGGCGGTGTTATGTCACCTTTTACAATAATTAAAGCGCGTATGGATGCCGCCTGGTGCTTTTGACAGCAAAGTGACGTACCATTTTCTGGACTTTATGTGCTTTTATACACGGCACCCAGCCGATGTGATTAAGACGCGCATGCATGCGGCAGACGATATCTAAGATTTATCGTCATGAGGGTTTGCGAGGCTTTGCGAAGGGCATCGTGCCGCGTGTTGTCAAGCGGACGCTGTCCACTGCTGTCACTTGGACGATCTACGAGCTACTCATTTGACCCGTCTGGTCTGGGCCACGCCAAGGAAGCTTTCAGCTTGGCAAAGGAGACAGAACGCACAAAGCAATTGGAGAGTCAGGCTAAGATCAAAGAGAACGAGGCTCTTTACAAGCAATACGAGATCGTGCGCGTGCAGAAGGAGGGTGAGGAACGTCGCAAGACGCTTGAGGAAGAAACACGTCAGCATCAGCAGCGGGCGCAGTACCAGGATCAACTAAAGCGCAAACAGTACGCTGACCAGCTGGCTGCCCAGAAATATATGAAAGAGCAAGAATTGAAAAAGCAGGAGGAGATCCTAGCACGTCAGGAGGCCTCGCGTCGTAAAACGCTTGACTATGAGGCTGAGCTGCGTCAGAAGACCGAGCTGGCCAAGGTTGCGGCCGAGACTGAGGGTCGTATCAAGCAGGAACGTCTAAACCACGATTTGCACCTTGAGGAGACGCGTGTGCATGCAAAGGAGTACCGCGAGACCGTCCTGGAAGGGATTAAGCTGGCCGGGAACACAGTTGGATCGGGTATAATGACTTTTGTCGATGACAAAGAGAAGCTTACGACTACTGTAGCATCATTGACGGCCTTGGCTGTCGGTATCTACACCGCTAAGGTGTCTACTAGTGTTGCGGGCAAATATGTTGAAGCTCGGATGGGTAAGCCGTCTCTTGTGCGTGAGACATCGCGTCGCTCTGTCGCGCAGGTATTGGCTAATCCCATTCAGTCTATCAAGCGCGCTTTGCGATTTCAAAATGCAACAGATGCTCTTGAGGGCGTCGTGCTCGAGCCCAAATTGGACGAACGTCTACGCAATGTGGCTGTGTCCACCTTTAACACGAAGAAAAATCGTGCACCTTTTCGCCACTTGCTGCTGCATGGCCCACCTGGTACAGGCAAGACGTTGTTTGCAAAGGCACTGGCCCGTCACTCAGGTTTGGAGTACGCCATCTTCACGGGTGGTGACGTTGCTCCGCTGGGTCGCGAAGGCGTGACGGAGATTCATAAACTCTTTGACTGGGCGTCACACAGCCGTCGCGGTTTGCTTCTCTTTGTTGATGAGGCTGATGCGTTTCTCCAAAAACGTAGCAACACCGTTATGAGCGAGGATATGCGAAACGCACTGAATGCGTTTTTGTACCGTACGGGCGAGGCTAGCGACAAATTCATGATCGTGTTCGCAAGCAATCAGCCAGAGCAATTTGACTGGGCCGTTAACGATCGTATTGATGAAATGGTTGAATTTCGACTGCCTGGTTTTGATGAGCGTGTGCGCATGCTAAACCGATACTTTAACGAATACATTCGTGCACCGAAAAATTCGCGTGCGAAAGAGATTTACGTGAGGGAATTATTTTTACGCTGGACCCCGTGGCCAACTTTCTCGTGCCCGCACGCGTGTTTTTAATGTGGACGCACTGCAGGGTGTCACTGGAGAAGCTCAGGCCATTTTTGATGCACTGAACAAGAGCCCATGCGTATCTATTGGGTCGTCTTTGCTCTGGTGCTAGGGAATGGCTTGTACACGTACTTTGCCGGGAAAGACGAGTCGTTTCTGGTAGAAAAGCTCAAGAAAAAGATCCCGTCTGAGTACCGCATTTCCGAGAAGTGGGATAAGTGCCTTGAGAACTTCGCTCTCTACTTTGGTGCCGGCCTTGTGGCTGGAGGTGTCACGTCAATTGTATTATCGCGATCTGGAGCCGGTCGTGGACTAATAACGGGCTTTGGTGTTCAGGTGCTGGATCAAGCTGGATGACGTGCCAGATGGCATTTGCTGGTGACGCACAAGCAGCTCTGGACAAGGCGGATAAGGCCGTGGAAGACTTGCCAGCCTTCCAGCAGCAGACGATCGTGGACTTTGTGACGGGCTCGACCACGCAGCAACTGTTGCAAAGTGTGCACTCGAGGCTTCTGCGAGATGGATAACGTAAAAATGCTCGAAAGGTACTTGATTGAGCAGCTGCAAAACAAGACAGTGGATATCGATGCCAACTTGGCGTTGCTCAAGCTGTATCAGATCTATCCGGCAACAGCAAGCGCCGAGAGTCAAGGTGCTGGTCAAGGGTATAATGGCAATGCCTTCTACCTTCTTCAACGGTGTCCTCGATGATTTCGGAGAACATTCGTGAGGATGCTAATGTGGCGACAATGCTGCGCACGGGTTTCATGCTCCAGTCGTGTTTATTTGAGGAATTTTGGAAGGAAGAAGTGTCTCTCGCCGAAGAGGTGCCAGGCTTTCTGGAGTCGGTACGCGCCTACATCGTGACGGCCATTGGCCACAGCCACAGCGTTATATCCATGGATGTCTTTATGGCAAAGCTTAATGTGTCCGAAAAGGAGGTGGCGAAGATTGCCGCCGAGAAGTGGACGCCGACAACTTGATTCAAATCAGCCCGAATGTGGACAATCAGATGCAAGCTAAGAAGGTCCAAGAGAACATCGAGTTCGAAGACGTGCTTCAGGTCATTCAAACCCTTTCCAGATCGAACGTTTGCGCGAGCGGTGGTGGAGTGGCTGACGCAGAGGAAGTTTGTGAGAATCTTGCCAAGCACTTAAGACTTGAGGGAGGATCCCAAGTGCTGCCTTGTTCCACGGGCGTCATTGGCTGGCGGATTCCCGTGGATGCCATTGTCCAGAACCTTTCAAAACTTGTGAAAAATTTGCAATCAGATTCGGTACTACCAGCTGCCGAGGGGATCATGACGACCGACCTGTATCCAAAGATCCGCTCAATGGACGTATGTGGTGGTCGTATCGTTGGAATTGCCAAAGGTGCTGGAATGATTGAACCCAATATGGCCACCATGTTATCGTACATTCTCACGGATTTGTCGATACCCCGTGACGTGTTGCGTCAGCTACTCACGGAGGTGGTTGGTGACACGTTCAACTCGCTTAGTGTTGATACAGATCAGAGCACGTCCGACACAGTGACGCTCGTTTCGTCCGATCAAATTCCGTTCGACATCAA---GCAGTTAAGCAAGTTTAAACAGGCGTTGCACGACGTGTGTCTTGGATTAAGTGAGGATGTTGTACGCAACGGTGAAGGAGCGCATCATGTGATACGAGTGCGTGTGAGTGGCGCGTTGTCTAAGAAAATGGCTAAAGGAGTCGGCAAATCGGTAGTCAACTCACCTCTGCTCAAGTGTGCGGTGGCCGGCAACGACCCAAATGTGGGTCGTCTCGTTATGGCTGTTGGCAAGTACATGGGGTCACACCACAAGGACGTGAACATTCAGCGACGTATGAAGATTCGAATGGGTGGCGTTTTAATCTTTGAGAGCGGGGAAATGGTACTCAACGACGACATTGAAGCCAAACTTGTGGCACACATGCGCGACGCGATGCTGATTGAGCCCACGCGTGGTGGACTAGATGCGAGCTCGCACAACTACCCGCCACATGGAAAGTTTGTCGAGATCGAGATTGACTTGGGGGTTGGCTCTGAAGATGCACAGGTGCTGGGAATCGACCTTACGCACGAGTACGTGGCCATTAACGCCGATTACCGTTCATAATGGCGCC---CAAGAAGGATAGAACCGCCGCTGCGTACCTGGCATCTTTGGGCGTGGCTTCTTC------------CGACAAGAAGGATGAC---AAGAAAAAGAAGAAGAAGGGCAAGAAGAAGCCAAAAGGAAGCCCATGTCAGCTGCAGCCAAGGCTTTGG-TGGAGCGCCAGATGGCTATGAAGGCCGAGCAGGAGCGTCTTGAACAGGAGCGTCTTGAACAAGAGCGCATTGCTAGAGAGGAAGAGGCTAAACGCTTGGCCGAAATCGAGCGCGAGGAAGAAATTCGTCTCAAGAAGAAGGAGGCGCGTGCTGCCAAGATTGAACGTCAGAAGAAGGAGGGAACGTACATGACCAAGGCTCAGAAGCAGAAAGCGGCGAGAGCTCAGGCTGCACTCGAGGCCATGCAGCAGGCAGGTCTAGTACCCGAGACCCTT------GATGAAAAGAAGAAAGCTGTGTATTCAAACCACAAGAAGAAGACCAAAAGCACGAGGTCGATGAAGACGACGAAGCAGAAACTCCCGATGCGTGGGACGATGAAGACTGGGAAAACAGTGACGTTGTGGCCTCGCTGGAGGACAAGCTAGCAGAAGAAGGAGCAGGAGCGGTTGCGTTTGCAGGGCATTGCATTGAAAAAGCGTCAGGAAGAGCAGGCTGAGCAAATGCGTCTGGACGCTGAAGAAGAACGTCAGTGCTCGAAAGCTAAGCTCGAGGCCGAGCGCAAGAAGAAAGAAGCAGCCGAGCGTCGACGCAAGCGCGAGGCCGCAGCACGTGCCAGCGCTTCGTTGGACAACTTGCGTTCGCCTATTTGCTGTATCATGGGTCACGTTGATACTGGCAAAACCAAGCTTTTAGACAACATTCGCCGGACCAAGGTGCAGGACGCGGAAGCCGGTGGCATTACCCAGCAGATTGGGGCGACCTTCTTCCCTGTGGAAGCTATCAAGCAAAAGACTGCCCGACTTGCTGAAGTGATGGAGCTGGAGTACCGTCTGCCTGGTCTTCTTGTGATTGATACTCCTGGTCACGAATCATTCACGAACTTGCGTTCCCGTGGCTCGTCGCTCTGTGATATTGCCATTTTGGTGGTCGATATCATGCACGGCCTAGAGCCACAGACTCTGGAGTCTTTACGCTTATTGCGTCAAAAGAAGGCCCCGTTTGTGGTGGCGCTTAATAAAATTGATCGTTGTTATGGCTGGAAAACTATGCTTGATATGCCCGTGCAAGAGGCACTCAAGCATCAAAATGAGCACGTTATTCGCGAGTTTGAGGATCGGATGAAGTCGGTCATTGTAGAATTTGCCGAACAAAAGCTAAATGCAGAGGTTTACTGGCGCAACAAGGACTTGGCACACACGGTCTCGCTCATTCCTACTAGTGCTATCTCGGGTGAAGGTGTGTCGGACCTCTTGATGATGCTCACGCGTCTGACGCAGGAACGTATGGCCAAATCGCTAGCATTTGTCGACATTCTCCAGTGTACCGTGCTGGAAGTAAAGGTCATTGAGGGTCTTGGTACAACCATTGATACAATCTTAGTGAATGGAACTCTAGAAGAAGGTGCAACAATTGTTGTTTGCACTTTAGATGGCCCCGTGGTGACCACAATTCGGTCGTTGCTCACCCCGCACCCCATGAAAGAAATTCGTGTAAAGGGTGAGTACATCCACCATCAAAAGATGAAGGCTGCAATGGGAGTGAAAATCTGTGCTCAGGGCCTCGAAAAGGCCGTCGCTGGCACACAGATTTACATGGTTGGCCCCGATGATGATGTAGACGAATTAAAAGACCTGGTCATGTCCGACCTCACAGGTATTCTAGACTCTGTAAAGGCCACCCGCCGCGGCGTCATGGTTCAGGCTTCCACATTAGGTGCCTTGGAGGCGCTCTTGGAATTCCTTCGTACCTGTGATCCGCCCATTCCCGTGTCTTGCGTCAACATTGGTCCCGTCCACAAGAAGGATGTGATGCGTGCTTCCGTGCAGCTCGAACACCAACCCGAGTTTGCTACCATTCTCGCATTTGATGTAAAGGTTCACCATGATGCCACCGAGCTCGCGACAGAACTTGGAGTGCGTATTTTTACAGCCGACATCATTTACCACCTGTTTGACCAGTTTACTGCATACATGGACAACTTTCGCATGGCTCGGCGCGAGGAATTTGCGGACGTGGCTGTCTTTCCTTGTGTGCTTAAAATCCTGCCCAATTGTATATTCAACAAAAAGGATCCTATCATCCTCGGTGTCGATGTCGAAGAGGGTATTGTCAAGATTGGCACCCCTCTCGTGGTGCCATCTGCCGGCT---CTCTGTATATTGGCAAGGTAGGGTCCATCGAGCGCGAGCACAAGGAGGTGGACCGCGCCAAAAAAGGCGCGTCTGTAGCTGTGCGGATAGACAATGAAAGCAGCGTCATGTATGGTCGCCACTTTGACCACAAAAATCAGCTCGTCTCGCGCCTCACTCGCCAGAGCATCGACGCTTTGAAAGAAAATTTTCGGGAAGATCTGCAGAAGGAGGACTGGCAACTCGTCATTAAGCTTAAAAAGGTGTTCGAAATCATTATGGAGGGCTGGCTCACGAAGCGTCGAGACCACATGAATCTCATCTGGTTGGATCGCTACTTCCTCCTCGATGGCAATCAGCTTCGCTACTACCGTAAACGGGGCGACCCTGCGCCACGAGGGACCTACATTTTGACCGACGGATGTGTTGTCAGTCCTGTCTTCAATTCGGAGGAGAAACACAAGCACCACGGGCCGGTGTGGATGTTCCGCATCACGTGAAAAATCATACAAGAAAGATCGATTCTTGGATCTCGGAGCCAAGAGTGAGGAGAATGCCAAGGAGTGGAAGCGCGCACTAGAGAACGCCGTGCGCTCGTTGCGGGAGCTCGTGGCGCACCAGAAAAAGCTGAAGACCGACGCCGAAAT---CTTTCCCACGCCGACGCAC---------GTGCCGAGGATGTTAAAGGCTGATGAAGACACTGTGACGCTGTTTAATCTCGACGCAGACAATGTGAAGGCAGAACAGGCGTGGTGGCTGGCTCGAGTCGAAGGCGGACTTCGCATCATGCAAGAATGTCCGCTCGGACCGAATGTGGCCGCAAATCTGTACGCACAACACGCGGCTACAATGAACGGATTGATCTTCAATGCCTGTCTCGGTGCAGCACTGAGTTTCAGCACCATGTATTCCTTCGCGAACAACATGCTGGTGAGCTTCATGACAGCCCTCGTGGCCATTGCCGTGTGTTGCGCAGCCGTGACGCCGAAACAAGCGCCAGAAATCCCAAGTTTACGTGTCTCGCAGGTCGTATACGGGTCCCCAACGGAAGCTTTTCGCTTAATCATGAACACTAAGCGATTCCAGCGATGGGATGCAGCAACAGCGACCATGCGTGTAATACAGCAGCTGGACGACCACGCGGACATTGTGTACGTGACGCAACGACCGACGCAATTGTGGCCCTTTTGGCAAAAAGCGCGGGACCTTGTGCTCATGCGCTACTGGCGTCGTGAAGAGGACGGATCGTACTTTGTCATGTACCAGAGTATCGACCATCCCGAGTGTCGGGTGCGGCACAATTACGTGCGTGCAAACTTTCTAGGTGGTGGCTTCGTAATTGCTCCCCAGCGTGTGCCAAGT---GGATCGATCCGTACACTGGTCACGTATGTGCTTCGGTACGATCCGGGAGGTTG---GTCCCGTATTTACCACCAACTTGGCATGGACGTGGATCTAGTCATGCCTATGCTGCGCAGTGTCGTGGGCATTCGGGATGAAATGAGCGCCACGGACTTTATCACGCCCAACGTCTCGATGGCACCTGCTGGAGACGGCGGAACCTGGTCAAGAAGCGTGGA---CTCAACAGGTGTTGTACTGGGCATTCAGAAGCTAGCA---ACGAGCCTTCCAGAAAAAATGTGGGCTGAGCCTGACGCTTCTCTCTTTAGTGTCCGGGGACACAATTATCTGACGGACAAGAAGAAGATCTCGTCGGCACCTGCAATGTTTCACACTGTGGGTGTGGATCTGCTGTCGTTCGAAAGTCCTGCAGAGAGGTACAACATTCTGTCTCGTCCTGATAGTATCAGTCGCACCAG---CTCCAAGTTTACATTTGTCGTTAACATGATCATCCCGAGTTCAGAGAACGTTTGCATGGTCTTTTACTTTCAGCCTGTGCGTGACAATGTCTTTGAAGACGGCTCGGGCTTTTCAGAGCTGTTAAACGACTTTATTGACGGCGATGACCAATTTCGGAACTCACGATTCAAGCTCATTCCAACAGTTGTCGAAGGCAGCTTCATTATCAAGCAGTCGGTCGGTTCCAAGCCCACACTGCTCGGAAATAAACTCAAGTGTCTGTACCATCGTGGAGAAAATTATTTTGAAGTGGACATTGACATTTCGTCCAACTCGGTAGCTAACACGGTTGTTGGCATGGTGCAAGGTGTGACCAAGTCGCTTGTGGTTGACCTGGCGTTCCTGCTCGAGGCACAGTCCGACGAAGAGCTGCCGGAAGTCATCCTTGGTGCAGTGCGCATGCAACACGTTTCGCTTGACAAACCACGTCGCATGCCAGCGCATGGGGAACAGGCAGCAAGGACGAGTACACCTTTTGAGGCGTTCACGGAGGCTTTGCGCGTGAATCTGCAGCGACTTATTGTCTACGCGCGCTCAGCTGACACGGCATTGCAGCGAGAAGTCGCCGAGAAACTGGCTAATGAAGCTGTAAAGACCGACAGGTGCAGATTGTGGAGCTGGACGGACTGCAGCTGTTACTTCCGCTGACAAAGTCGAGGGACACGGAAGTACAGCGTCTAGCAGCGCATGCACTTGCCAATCTATCTGTCAACTCTGAAAACCAGTCCAAGATGGCAACAGAAGGAGGGATCGACATGCTAATTGATCTCTTGGGCAGTACAAACGAACACGTGCAACGCCAAGCCGCCAAAGCACTTGCCAATCTTGGTGTAAACGTGGACAACAAGGAGCGGATTGCAAAAGCCGGTGGCATTAAACCGCTTATTGATTTGGCCAGCTCCCGTCAGATTGGCGTTGCCGTCGAAGCCATCGCAGCACTTGCAAATTTGGCAGTCAATG---ATGCCAATGAAGTCGAGATTGCCCGAAAAGGTGGTCTGAAGCCCATTATTGATGGAGCGCACTCTGAATCAATCGAACTGCAGTCTCAAGTCGCACGTGCATTACGCAATCTATCGGTCAATCCCGAAAACAAGCAGGCGATCGTAGAGCTGGGCGGAGTCGAGGCTCTTCAATCCCTCGTGCGTGTTACCAGCGACCGAATTTGCCAACAAGCTACACGAGCGCTCGTCTCCAATGTTACGCTCACGCGCTCCTCACCCACAACTTGCTA------GTAGCGCCCGTGGCCCGCGTGGCCGAAGTTAAAGAGGAGGC------TGTTGCCTATGATGGTTCCATGGTAGACGTCGAGTTCTCCACCACAGTCGAGACCGTC---------ATGGTGCCTCTTACAACCCCGA---CGAACGCGGCCAAT---GGCTTTTTGTCTATTTCGGATCGGTTTCCTTTATCCAAGGTGGGAAAGGTCAGCTTTAATACGGCTCCACTTCCTATGAGCCG---CTTGCTGAATG---ATACATCTGAAATGACAC---AAGTCGAGCAA-------------ATGACTGTGCTGATAGAAGATGGCACGGTAGATGACAAC---ACCATTAAGGCGGAG---CCAGAAGGTGAGTGATGTATACCATTATCAAGCCACTGA--TGTAGAAGAAAATGAGATGGAGCAACTTCGATGGAATATACAAGAACAAGTATTTCAGCGATATTTTCATCAAGCTCAGAAGTATGACGAGTGGGGAAAATTGGAGGAGCAAGACACCGAGTTTTTTTTGTACCGGGTGGAAGACGTGAAAATATTAAAGCCGAAAAGCGGTTTCATGATAATGCTGTTCGCGATGCCATGGGAAAAGGAAATGCCACTTGGGTGAAGGAACGACGAGATCGGTTTGAAGAGGAAATTCGACTGTTTTATGACGAGACGAGGCACAAACAAGAGCTGATGCTTACGCGACTAGAAAAGGTGTCACCATTTTCAAAGTTCCCGATTCTGGGCAATCGTTTTTTACTCCTTCGTTTACGAGGAAAAGGAGGCAATGGAGAGGTGTGGGATGTTGTTGATTACGCTAATAAGAAGCATATTTGTGCTTTAAAGCTTTCCACTTCAATTCGACACGCTCAACGCGAACACCATACGCACACTCAACCATCCAA--ATATTGTTCAAGTTGGCGAGATGCCTTTTCTCATACGATATCAACAACAGCAGTACACTGCGTTTACTGTCGCCAGCGTCCAGTCAGATCTGCAACAACTTATTGAAG---TATACGAGCATCTAGATGATGACTCGGCTTACAAGGTGCTGTCTCAGCTTTTAAATGCATTGAGATATTTGCATGATTGGCGTTGCTCACTACGACCTCAAGCCGTCTAACATTTTGATCGACCACGACGGGTGTGTGAAGCTG------ACTGATTTTGATTTAACTCGCGATGCCAAGGCAGCTATTTTCAACTCTACCGTTGGCACTCTGCGATATCTTCCTCCGGAGTGCTTTCGCCCAAATCGAGGCGA---------CTGTGAAGCCACTGCCGAAAAGGCAGATATGTGGATGGTGGGCATCGTATATTGCATGATGGTTACGGGAAAACATCCTATTTGCCCGGACAAGTCGACCCACGTGGAAGTGAAGACGTTGATGGCTCAATACACAGGC---GAGTTTCGGTACGCACGGCCCGTTTCCGACTTATCTCGGTGGATCATGCAGGGTTGCCTTCACCCGGATCCGCGGTGTCGACCGACAGCTGCCCAGTTGCTAGTCGCACTCCGA

Pythium_aphanidermatum CCGATTACCACCGCCAGGTGGCGCGCGAGCTCGTGAGCTTCTTCCACTCGTTCTACCGCTTCGGCGGCACCTCCAACCACTTCGATCTCCTGCTGCAGAACGTGCTCTTCGCGTCCCTCACAGTCGGAGCGCTCGTGTTCATTGTGCTCTGCATTGTGGTCGTGCGTCGTACGATCCTCCATGTTCGCAGGCGTACGCTAACCGCGTATCACTTGAAGCTCAGACGATGGAGATTTCGGCCGTTATCCTCTTGACCGTGCTCGCGATCAGCTCGCTCAGTGGACTGCTGGGGGAAGCGCTTCCAACACGAGCGCCAGTGTCAGGTTCCTTGCCGAGAACATGATTGTGTCCTTTAATGAGAGTGACCTCCCGGAACAAGCCTTCAAGCTCTCGATCGAGTCTCTGGCATGACCCACGCGGCTAAAGATCTCACCAATGCTACCCATGCACTGCTTCCATCCAATTATGGTGAGATGGCCAAGAACTGGGAGTTTAGCTACTTCATGCTCAAGTCGACGACGAACTTTGCCATTCTGTCAGTCTCACTCGCCAGCTTCTTGTCAATCTCCGCCATCGGCTGGTCAATGGTCACGCCGCTACGCATCGCCATCTTCGTTATTCTCAGCATTGTGCCTATCTCGCACACGTTGATTGGAGTGTACCTCTCGTCGACTATGATCACGGCCGACTTTTGCGCCGCACCAGCCAACAGCACTGCAACGCTGCTTCACGTATGCCCGCAGAACGTATCCAGCCATTCACACCGTATGCTGCAAGACTATGCAACCCATCACGGCGACGTCGGTGCTCGCATGAAGCAGAACTTCTTGGATCCAATCGGTTCAGATTTGGGCGTCATGCTCGGGTACTTTGTTGGCACGGTCTCGAATTGGGAGAGTTTCCAGCTCTTCCAGACATGGCGCTGGCCGTTCCTCGTGCAGTTTGTCGCCATGTGCCCCATCATGATCGCCGTCTTCTTCGTGCCGCGCAAGCACCTTGTGATCCGAAGCGAACTCAACGCGCTTGTGTCCCTTGGTGCCAACCCGCCATTAAGTTGGAGCGG---CCCCAACCATGAGGAAGAGGACGTGCTGCTGGCGAGTGCGTCCATGCCGGATATGGTGCATTTGACCCGAAGTGTACGAGCAAGGCTCCTTTGCTGAAGGTCTGATGGTGCTGCTTAAGATCCCGATCTACTGCTTCATTGTGGGTGGACTCACAGCCGTGTACTTCGTCGTCACGGGTGTGCAGTACTGGAGTACAATCTTCATGATCAAGTCGCTGCACGCATCCAAGTACCTCGTAAACGGTCTGTTCGTGCTTGTCGCTGGCACAGGACCGATTCTCGGTGTCTTTTACGGCGGCTGGTTGATTGATCGCTACGGCGGCTACATTGGTGTGGAACAGCGCGCCAAGTCACTGGGGATTTGCATGATACTCGGCGTTGTCGCTTTCGTCATCTCAGCGGCGACGACTTTCTTCGACGATATCTACACGACTGCGGCGTTCTTGTGGCTGCTCCTCTTCTTCGGTGGCTCCATCCTCCCCGCGCATCGTGCGCTCGCATCGTCGTTCTCAGTTATGATTTTCAACCTCGTCGGATACGCATTGTCACATGAGCGACCTGGAGATGGTGCCGGGCGTCAAGTGCTATGTGCCGGACGAGACGCACGTCTGGCTTGAAGCGGAGGTTCTCAAGGAAGAGCGCACCGCCGACGGCAAGGGCCGTCTCATCCACACCAAGGTCACATTGCCGGATGGAGCGCGAGGACCGCGTCGTGGACCTCAGCGACAAGAAGACCAAGGCGCTGCTGAGGTGCACCAGCTCGAGTCCCTTCCCTACCAGAATGAGGGCTTGGGACCAGAGGGTATTGAAGATATGATCACCCTCAACTATTTGCACGAAGCTGCGATCTTGTTTAACGTCAAGACGCGTTTCATGCAGAAGCTTCCATACACGTACACGGGTGATATCTGTATCGCTGTGAACCCTTACCAATGGCTGCCAGAGCTTTATGCCGAGGAGGAGCACCTTAAGTACCTGAACCAACCCAAGGAAGAACTCCCTCCTCATGTGTATGCGACCTCGGTCGCATCGTACGACAACATGCGTCGCTCGGAGCACAACCAGTCGATCCTCGTCAGTGGCGAGTCCGGTGCTGGTAAGACGGAGACGACGAAAATTCTGATGAACCACCTTGCCACAATCGCCGGTGGTCTCAACAACGTGACCATCAAAAAGATTATCGAAGCCAGTCCTCTGCTTGAAAGTTTCGGTAACGCCAAGACCGTCCGTAACGATAACAGTTCGCGTTTCGGTAAATTCACGCTGCTACAGTTCGATACTGAGGGGACGCTCGTCGGTGCTAAATGCCGCACTTATCTTCTCGAGAAGACGCGTGTGATCTCGCATGAAGCGCCTGAACGCAACTACCACATTTTCTACCAGTGGAGTCTGGCGACCTCGCCAAAGAAATGCTGTTGAACCCTGACACAGTGTACAAGTACGCTGGTGACAGCAAGGCTCAGAAAATCGAGGGAATGTCAGACGCCAAGCATTTCGAGAAGACTCGTAAGGCCCTGAGCCTCATTGGTCTGGACGAGGCCGCTCAGCGACCACTGTATGAAGTCCTCGCTGGTATTTTGCACCTTGGTGAAATGGAGCTCATGTCGAACCCAAGCAACGATGAAGAATCGCTCATTGTAGAAGGTGACACCGGTGCGCTAGGTGCTACTCAGATGCTTGGTGTCACGTGGGAAGCGATTGAAAAGGCTCTGTGTTCCCGTACCATGCGTGCTGTGAACGACGTGTACAGTGTGCCACTCAAGAAGGAGCAGGCCATGGATTGTCGTGATGCTCTCGCGAAAGCGATCTACTCGAACGTGTTCGATTGGCTTGTCGCTGCCATCAACCTTTCGCTCGCTGATGACAAGAACATGCACAACCACATTGGTGTCCTTGATATTTTCGGTTTCGAGCACTTCAAGCACAACTCTTTCGAGCAGTTCTGTATCAACTACGCAAACGAGAAGCTGCAACAGAAGTTCACGCAGGACGTCTTCAAGACGGTTCAGATCGAGTACGAGGAGGAAGGTATCAAGTGGAACCACATCGAATACGCTGATAACCAGGATGTTCTCACCGTGATCGAGTCGAAGATGGGTATCATCTCGCTGCTTAACGAAGAACTCATGCGACCGAAGGGTAACGAAGAATCTTTCATGTCGAAGGTGGCATCTCTGCACAAGGAT---GATATGGCTCATGTCATTGAGTTCCCTCGTACTTCGCGCACGCAGTTCCTGATCAAGCACTATGCTGCTCCTGTCATGTACGAGTCTGTCGGCTTCTTGGAAAAGCACAAGGACTCCCTCCTCCCTGACCTTTCGGAGCTGATGCGTGGTTCTTCGAAGCCTTTCCTTGCTGCTCTTTTTGAGCCGAAGCCAGAACCAAAGTGCTCGCAAGC------GTGGCGGTGGTGCCCTCACTCTGTCCACAGTTGGTACCCAATTCAAGGAAAGTCTTGGCGAACTCATGACAACGATTAACGCGACCAAGGTGCACTATGTGCGATGCATCAAGCCGAACCCGATCAA---------GAGCCCGACGGCGATGGATCAGAACATGGTTGTGTCCCAACTGCGTTGCGCTGGTGTCATTGAAGCTATCCGTATTGCGCGCGCAGCCTACCCGAACCGACTCCAGCACTCCGAGATCTTGGACAAGTTCTGGCACTTCGTTCCAAGTGCTGGCTCAACGCCGGCGGAGAAGTGCCAGCTTCTGATGGAGAAGCTGAAGCTCGCCACTCCTGAGCAGTACCAGATGGGTAAGACTCGTGTTTACTTCCAGTTGGGTGTTCTTGAAGAGTTGGAAGACCGCCGCAAGAAGTTCCTCGATGCGAAGGCTACTCGTCTGCAAGCGATCATGAAGTGTTTCCATGCCCGTATCCAGTATCTCCGCAAGCTGGAGGCGATTGTGAAGCTCTGTTGTCCGCTGTGTGACGACTATGCGTCGGTACAACACTATTCGAAAGGGTGTTATCAAGGCGCAGGCTCAGTGGCGTGGTGTCCAGGGTCGCCGTCTTGCGGTCGAGGTGAAGCGTAACCACAATGCCGTCATCATTCAACGATACACGCGCGGATACGTCCTCCGCTCGAAATACGTCAAGGTCCGCCACATGGTCATCCGTGTGCAGGCTATGGTCCGTATGCACATTCAGCGTCCGAAGTACCTCGCTGCTCTTCAGGAGCAGAAGCTGCAGGCAGACATGGCATACCAGCTTCAGAAACTGAAAGATCGTCTTCGCGAGGAGCAGGAGCGCAACGCCCGCCT------TCAGCAGGAACGCGAGAGCGCACC--AGCTCCTTTGCTGCCGAATCCAACGTTATCATGGCTGATGCTGGTGGTATGATCGAGAAGCTGCAGGAAGAGAATGCCAAGTTGCGTAAGAAGAACGTTGAGCTGAAGGAGTCGTTGGCTACTGTCAAGCTCGACAACGAGAAGTTCAAGGGTGACAAGGAAGTTTCATCTGCTGGTTACATCGTGAAGGTCCGTCAGCTTGAGGAAAGTGGCCGTGAGAAGGACAAGCGTATCAAGCAGCTTGAAGAGGAGAACAAGAAGCTGCAGGATCAGGTAAAGGATCTCATTGCCAACGG---TGTGCCAGCTGAGAAGAAGCCGAAGGAGAAGCGCGCATTCTTCCGCACTCTCGGGGCGAAGAAGGAGAACAAGAAGA---------------CCGAAGAGGTCTACATGGACTCTGTTACCGGTGAGGACGATGTGACCCGTGGGGACTCGCGTCCATCGGA---GCGTTCGATGCCAAAGCTGC---CAACGATGAGCTTGGCCAAGGCTAAGTTCTGGGGTACTGGCAGC---AAGCAGACTGCCAAGCATTCGGACGGTGAGGACAGCCACGATGAGGAGAACGGCAGCCAGAACGGCACGATGA--TGACTCGCCATACCCTTGTTGGCACTATCGGTGTTGGTGTCACAGGCACGATGTCGAGCATCAAGGGACGTATG---------AAGGGTATCTATGACTCGACTACAGCAAGAGTGGAGAGATGATCGACCAATTCGTGAATCGTTCAACCTTGATGCATTGCCAGAAGTGTCGCTCCCGCCAGGTTGGGAAGCAAAGGTGTCGCGTTCAACAGGCCGTGTGTACTACGTCAACCGCAAGCTGGGCAAGTCCCAGTTTGAGCGTCCAACAATTGCTTCGCTCAA------GGCCCAGAAGATGGCGCGCCAGAAGTCGACGGCGAACTAG-AAGGCAGTGGTCGCTGATTTCGGCTGCGGTGATGCGCGGCTTGCAGAGAGCGTCAAGAACAAGGTTCACTCATTTGATCTCGTGTCCCTTGATATTGCTGTCTTCTGTCTTGCGCTCATGGGAACAACTGTCCATGAGTACATCCTTGAAGCGAATCGCGTTCTCAAACCGAAGGGTGTGTTGAAGGTGGCTGAAGTTAAGAGTCGTTTCGAGACTGATGAGCTTGAAGGATGGCAGGCGTGGTGCTTGCGGCGACGCACGATGCGGTGGCGATGATCCGCCACTCGACGCTCTTCCTAGAGAGACCAAGCACGTTGTGATCGTCGGTGAGAAGACAGGCGAGGCTGTCGCGATGCCGTTCCCTGACTTGCAGCGCGACCTGAAGACGCTCTTGGGCCACACCACGTCCATGATCACATACATGGCGGCCACCGCAAAAGCTGCTCCTTTCTGCTGACCGTGACGAGAAGATCCGTGTTTCGCACTTCCCACGCACTGCGCTCGTGCAGAGCTACTGCCTGGGCCACAAGGCGGCGCTCACACGTGTTGCAGTCAGCACCGTGAAGCCACAGCTCGCTGTGTCAACGTCGCTAGACGGCACCATCAAGCTCTGGAACGTCGTCGATGGCACGCTGGCTGTCTGCCCCATTAAGACGTCGTTGTCATCGTCATTAACAACCGTGATCTGCGGTTCTTCGCCATTGCCAGTCCCCATTCCTGCGTTTCTTTGATGTGACCAAGAGCTACCGACGAGCTCGCAAGCTGTGCGCGACTTCCGTAAGCTGCAGAACGACCCCCCGTCGGGTGTGTCGGGAGCGCCCATGGACAACAACATCATGCTGTGGCAAGCCGTGATCTTTGGACCCGATGACACGCCGTGGGAGGGCGGCACCTTCAATCTGACGCTCGAGTTCAGCGAGGACTACCCGAACAAGGCCCCAACTGTCAAGTTCATCACCAAGATGTTCCACCCAAACATCTACAACGACGGCCAGATCTGTTTGGATATTCTGCAGAACCAGTGGTCGCCAATCTATGACATCTCGGCCATTCTGACGTCTATCCAGTCGCTCCTGTGCGATCCGAACCCGAACTCGCCCGCGAATTCCGAAGCTGCTCGCCTCTTCCAGGAGAACCGACGCGAATACAACCGACGAGTGCGTGAGATTGTCGAGCAGAGCTGGGTCTCGATCTATGGTGTCCGCCGCCAAGATGCGTGGCGCTGAGAACCGCCTCATGGCGGGCCGTCCGTTCGTGGCCTGGCTCGACAACATCTCGTCGGACCGTGGTCTCTGCGGTGGTATCAACTCGGGTGTGGCCAAGACCGTGCGCAAGCAGCCACCACGCAGCTCTTCGTGATCGGTGACAAGGGCCGTGCGCAGCTCCGTCGTGACCTCGGTGAGATCATCCGTGGTAACGCAACGGAGGCGTACGTGAACGCCCCAACGTTCACGGTGGCTTCGGCCATCGCGGAGGCCATCGAGAACGAGAGGAGAGCTCGCGTATGACGGCCATGGAGAACGCCACGAGCAACGCCCAGGACCTCACGCTCACGCTCAAGTACAACAAGGCCCGTCAGGCCCGCATTACGACCGAGCTCATCGAAATCATCTCGGGTGCCGCCTCGCTCGACCACGCCCGGCACGGTCGTGGAGATCCAGGACGCAGCACAGTACGACGAGCTCACGGCGCGCGAGACCACGCTGTCCATCGCGTTCTTCTGGGCCGAGTTCCAGCTCTGCCGCCCCAACGGACAAATCGACGTCGGGCAAGAGCGTGCTCGAGAAGCTCGAAGGCGCCAATGTGGCTGAGCTGGCCAAGCGCGTCTTGAATACCGCCTCAAAAAGCTCACCAACGCGTCGCCAGTGATGTTGTTCATGAAGGGCAGCCCGAACGACCCCAAGTGCGGCTTCAGTCGCCAGATCGTCGCGCTGCTGAACGAGGAGAACATCCAGTATGGCACGTTTGACATTCTCAACGACAACGATGTGCGCCAGGGCCTGAAGCAGTTCTCCAACTGGCCGACGTTCCCGCAGTTGTACGTAAATGGCACGCTCATCGGTGGCCTTGACATCGTGAAGGAGATGAAGTCGGAAGGCTCGCTCGTAGAGCAACTGGGCGTCGAAGAAGCCGAGGCGGCGTTCAACGAGAGCCTGCGCGCCCTCATCAACTCGGCGCCTGTGCTGCTGTTCATGAAGGTTTGATATTCTGTGTGGGATTACTCACGACAGAACCAAGCAATCAGCAAGGTCGTGAGCTTTGACCCACTGTCATTCCGGCGAGAGATGCGCCGAGTCGATTGCGTGGAGCCCGATGGCGCCTCGCGATCGCCTTCCGTGACCCCATGTCGTCCATGGGCGAGTTTTGTGTCCTCCGTCTCCTCGAGTCAGGTCCAAGGCAACTACATTGCACCAGAAGGGCATCAGTGTGATCGACTGGGAAGTGGAGGATCCTTCACCGTGAACACCGTAGCGACGTCCGCACGATCTGCATGCACTCACAGGGGAATGCTATGTACACTGGTGGCTTGGATGGTTGTGTCGTACGCTACGATATGAACTCGCACCACGCACACACAATCATGGAGCGCCGTCGCCAGATTTCCAAGATTAATGCCGTGATCGAGAACCCACACAATCCTAACCTGCTGCTGGTCAGCAGCGTCGAACCGTGAACACAATCTGCTGCTGCACGATCTGCAGCGCTATGAGCCGTCGCTGACCTCCAAGTCGATGAGTCAGTACATTGTGCCCCGCTGGTCACCTGGTGGCTTCCACGTCTCGTGTGGCAGCACATGCGCTACTGCCACGGCAGCTTCACGACCCCAACGTCGGCCACCATCGGCGCGTCGTTCTTACAGAAGCGCATCGTTGTGGG--GCAAGATGACGCTGCAGATTTGGGACACTGCTGGCCAGGAGCGCTTCCGCTCGATGGCGCCGATGTACTACCGCAATGCCAAGGCAGCGATCCTGGTGTTCGACCTGACCAACCAGACGACGTTCGAGAAGGTGAAGGAATGGCTACATCTGAAACAACACGTCGGCGATGATATGTAGGCAACAAGTGCGATTGTTCAACTTCACAGCGGCGCAGCAGTTCGCACAGGAAATTGGTGCGATGGTATTCCGAACGAGTGCGAAGACGGGTGATGGCGTTGCAGACCTCTTTGAGTCTGTCGGCGAAGAGCTCCTGAAGAAACACGAAGAAGAGACCCGGAGAGCTTCTACGTGGTGTATGTGCCCACAGACGACTCCAAGGAGCTGGAGGAGTGGACTGTAGCGCTGCCCGATGACAAGGAGGCGCAGATCGGCTGCCTGACCGACCGCTTGCGCACGCATTTCAAACACGCGTCGAAGTGAGAAGGAGCAGAGATCTTCAAGAAGCAACTCCTGAGCCAGTTACCAGAGGGAGCGCAGCTCGACAGCGGTATGCTGCAGATGATGCATGGATTCGCTCGTCGACTCGGTACCTCTCCTGCTCAACACGAAGGCCGTCGGCCACGTTCGAAGTAAAGGGCGACGCCTTCATTGCACGTGTGTTCGATAGACGATTTCATCCGCATGGACTTCCGTCTGTCTGAAGTGCGCGGTGACGCGGAGTGGATCCAGCTCGCCAAGAGCCAGAGGTCGCACAGACGCTGGAGGACCTGCTGGCGCTTGGCCAGGGTCTCGACGATGAGGACTACAAGGCGGAGCTTGAGGTCCTGCAGAACGACGAGAGCTCGAACCTGTTCTCGGCCGTGACGTTCGAGGAGCTCGGACTGCCCGAGCCATTGCTGAAGGGCGTGTATGCGATGAAGTTCTCCAAGCCGTCCAAGATCCAGTCGGTGGCTCTGCCAATGATCCTCGCCAACCCACCAGAGAACCTGATAGGGCAGGCGCAGAGTGGCACCCCACGCGTGAGCTCGTGCGTCAGATTGCTGCTGTGGTCAAGGCCATGGGCCGCTAACGGAGATTTTCCTCGCGATTCCTGGCAACGATGTCGAGCGTGGTGACACGCTGACCGCACAGATCGTCGTAGGTACGCCTGGCAAGGTCGAAAGTCTGCTCAAGAAGAAGCTTCTGGACACGCGCGATATCAAGATCTTCGTGCTCGATGAGGCCGATGTCATGGTCGCTGAAGACGGCATGCGCGATCGCTCGGTTGCCATTAAGAAGGCCATTCGCAACCGCGCATGCCAGTTCCTTTTGTTCTCGGCTACGTACGCTGACGATGTGCGTGAGTTCGCCATGAAGATGGTGCCGAACCACAACATCATCACAGTCAAGAAGGAGCGTCTGACGCTCGACGGTATCAAGCAATTCTGGATCGACTGCGGTACCAAGGATAACAAGTTCAAGGTGCTCTCGGATATTTTCGCGATCCTGACGATTGGCAAGTGTGTCATCTTCGTGCAGTCGCGTGAGACGTGTAAGGAACTGACCAAGCGCATGCGTGCCGATGGTCACTCGGTTGGTATTCTGCACGGTGCCGACATGGCCAAGGAGGTTCGTGATCAGGTCATCGATGAGTTCCGTGCTGGTACAACCAACGTGCTTATCACGACCAACGTTCTCGCGCGTGGTATCGATGTCGCCGGCGTGTCCCTCGTCGTCAACTTCGACGTGCCGCTCACTCGCGACCAGCAGCCGGACCCTGAAACGTACCTGCACCGTATCGGTCGTACGGGTCGTTTCGGTCGCAAGGGTTGTGCCATCAACTTTGTGCACGACCGCAAGAGCAAGGAGGACCTGGCGCAAATCGAGGCGTACTACCAGCGTCCTATTGAGCAGGCCCCAGCTGACGATATTGAAGCACTGGAGAAGATGCTGCAGTGGAGGGCAGCAAGCGCCGCCGCGCCAAGTTCGGCAAGGAGCAGAAGAAGGGCATGAGCATCTACCCGCGTGACCCGAAGAAGAAGGTCAAGGGCAAGGACAAGACCTACTACTACCTTAAGGATGTCATGTACCTGGCGCACGAGCCGGTGCTCGAGAAGTTCCGCGAGTTCAAGACGTTTATGAAGAAGGTCAAGAAGGCCATGGGCAAGAAGCAGTACGATGACGCCAAGCGCATTCACGAGAACCGTCCTCAGTACTCGCTTGACCACATCGTCAAGGAGCGTTACCCACGCTTCATGGACGCCCTGGGCGATCTCGATGACGCCCTGTGTGGCAGAACTACGTCGTCGCGAGCGGCTCGTTGACCAAGGTGTTTGTGAGCATCAAGGGGATCTACTACCAGGCCAAGGTGCACGGCCAGAACATCACATGGCTTGTGCCGCACCAGTTCACGCCAACTGCTGATGAACGCGTCGACTTCCGTGTAATGCTCACGTTCCTCGAGTTCTACCAGGTGCTTCTCAAGTTCGTCAACTTCCAGCTCTACACGGAGCTCGGCATCGCGTACCCTCCGAAGATTATCCTTCAGCTTGATGAGGCTGGTGCCCAGCTCGCGGTGCTTGACCTAGCAGCAGAAGGAGTCTGAGAAGCGTATCAAGCCACAGCGAACTGCGTAAGGAGGCCAGCGAGAAGAGCCTGTTTAGCGGCCTGTACTTCTTCTTGTCGCGTGAGGTGCCTGCTGCTCCTTTGGAGCTTGTGCTGCGCTCGCATGGTGCTCAGATCGGCTGGGAGGGAGAAGGCTCTCCGTACGCTGAGAGCAGCGACCGCATCACGCACCACATCATGGATCGTCCTCACCAGGGCCACCGCTACTTCAACCGCGAGTACGTGCAGCCACAGTGGGTTTTTGACAGCGTCAACAACGGTATTCTCTTGCCAATCACTCGCTACCGCAAGCAGATCGACAAGCTCAAGTCCGCCGCCCAGGTTCTCCGCGAGGTTGGAGGCGAAGAGGGCTACGACGACGAAGGGAGGCCTTCGCCGCCGGCATCAAGGCAGAGCGTGATGGCAACAAGAAGTCGAAGAAGGAGAAGAAGGAGGAGGAAGACGCCGAGCTGCACGAGATGGCCGTGAACATGATGTCCAAGAAGGCGCGTCGTCTCTACGACCGTATGCAGTACGGTCTCGGAAAGAAGTCGGACAAGATCCAGGCACTTGAGGCCAAGAAGGCCAAGCTTGACCCAAGACCAAGAAGCTGGCGGCAGGTGCTCTCTCGGGTATGATTTCTGCACTGGCCTTCCAGCCACTTGGGCGATGCGTGGGCGCGGCACGTCTGCAACTCTGATTCGAGTCGCTGGTGGCGCCGGACTCTACTTCACGACGCTGGACCAGTGTCTTTCGGCGGTGCCCAACACAGCGATCAACACGTTTCTCTCCGGTTCATTCGCACGAACGTTTGCGGGTTGTGTGATGTCGCCGTTCACGATCATTAAGGCGCGCATGGATGCCGCCAGGCACGTACTCTGGTAAACGGATGTGCCATTCTCGGGGCTCTACGTGTTGATCTACAAGGCATCCCGCAGACGTGATCAAGACGCGCATGCATGCAGCAAACAATCCTCAAAATTTGGAGTGAGGAGGGCTGGTGCGGCTTCGCCAAGGGCATTGTGCCTCGCGTGCTCAAGCGCACGCTCTCGACCGCCGTGACCTGGACCATCTACGAGCTACTCGTTCGACCCGACGGGCCTCGGCCACGCGAAGGAGGCGTTCCAGATCGCGAAGGAGACGGAGCGCACGAAGCAGCTCGAGCAGCAGGCTAAGATCAAGGAGAATGAGGCCCTGTACAAGCAGTACGAGATCGTGCGTGTGCAGAAGGAAGGCGAGGAGCGCCGCAAGACCCTCGAGGAAGAGACTCGCCAGAACCAGCAGCGCGCACAGTACCAGGATCAGCTCAAGCGTAAGCAGTATGCTGACCAGCTGGCTGCGCAGAAGTACATGAAGGAGCAGGAGCTCAAGAAGCAGGAAGAGATGATCGCCCGTCAGGAGGCTTCGCGCCGCAAGACCCTCGAGTACGAGGCTGAGCTCCGTCAGAAGACGGAACTCGCCAAGGTTGCCGCTGAGGTCGAGGGTCGCATCAAGCAGGAGCGTCTCAACCACGATCTGCACCTTGAAGAGGCCCGTGTGCGTGCAAAGGAATACCGTGAGACCGTCATGGAGGGTATCAAGCTCGCTGGTGCCACGGTCGGTGCCGGTGTCATGTCGTTCGTCGACGACAAGGAGAAGCTGACCGCGACCGTTGCCTCGCTTACGGCGCTCGCTCTCGGTATCTACACGGCCAAGGTGTCGACTGGCGTTGCTGGCAAGTACATTGAGGCGCGCCTTGGCAAGCCATCGCTTGTCCGTGAGACCTCGCGTCGCTCGGTTGCCCAGGTGGTCAGCAACCCATTGCCATCGATCAAGCGTGTGCTTCGTCTGCAGAAGCCACAGGACGCTCTTGCTGGCGTCGTGCTTGAGCCAAAGCTCGATGAGCGTCTGCGCAGCGTTGCCGTGTCGACGTTCAACACGAAGAAGAACCGCGCGCCATTCCGTCACTTGCTGCTGCACGGTCCACCAGGTACGGGTAAGACCTTGTTCGCGAAGGCCCTTGCCCGTCACTCTGGCCTCGAGTATGCGATTCTTACGGGTGGTGATGTTGCTCCACTTGGTCGTGAGGGTGTGACTGAGATTCACAAGCTCTTCGACTGGGCGTCGCACAGCAACCGTGGTCTGCTTCTGTTCGTTGATGAAGCCGATGCGTTCCTTCAGAAGCGTAGCAACGCTGTGATGAGTGAAGATATGCGTAACGCACTCAATGCGTTCCTCTACCGTACTGGTGAGGCCAGTGACAAGTTCATGATTGTGTTTGCCTCGAACCAGCCAGAGCAGTTCGATTGGGCTATCAACGATCGTATCGATGAGATGGTTGAGTTCAAGCTGCCTGGTCATGCTGAGCGTGTGCGCATGCTCAAGCAGTACGTGAACAACTACCTGGTCAACCCATCCAACAAGAAGTCGAAGAAGATCTACGCGAGGGCTCGATCTTCACGCTGGACCCCGTGGCCAACTTCCTGGTGCCAAGACGCGTATCCTACAGATGGAGGCAATCAAGGATGTGTCGGCTGAGGCACAGGCCATTTTCGATGCTCTCAACAAGACGCGATGAAGCTGTACTGGGGTATCTTTGCGATCGTGCTGCTCAATGGTGCATTTACGTACTTCGCTGGAAAAGATGAGTCGTATCTTGTCGACAAAGTCCAGAGAACCATCCCATCGGAGTACCGCATCTCGGAGAAGTGGGACAAGTGCATCGAGAACTTTGCGCTTCACTTCTCGGCTGGCCTCGTCGCCGGTGGTCTCACGTCCATCGTTCTTGCTCGTACTGGTGGCGCTCGTGGTGTGCTGACTGGCTTCGGCGCGCCGGTGCTGGTTCGAGCTGGACGACTTGCCAACTGGCATTCAAGGGCGACAGGCAGGCGAAGCTCGACAAGGCCGACAAGGTCGTGGAGGAGATGGCAGCGTTCAAGAAGCAGACGCTCGTGGATTACACGGCGGCTTCGGCCGTGCAGAAGCAGCTCCTGCAGGCGTACCGCCGGCTTCTTCGATGAGGCTGTGGTCAAGACGCTCGAAGCGTACCTCGACGAGCAGCTCAAGAACGGCTCGGTGGACATTGACGCCGACCTGGCGCTGCTCAAGCTCTACCTCGTGTACCCTGCTGTGGCCAAGGTTGACAGCGAGCGTGCTCGTCAAGGCCGTGAGCGTGCTCCCGTCGTCGTTCTTCAGTGGCGTCGGCTGTCGTGCCAGAGAGCTTCCGTGAGGACGCGAATGTGAAGCAGATCTTGAGTGCTGGCTACCTACTGCAGGCGTGCCGCTTTGAGGACTTCTGGACGGTGGACGTTGCTTTTGCCAACAAGGTTGCCGGCTTCTCGGACGCCGTGCGCGCTTACATCCTCGAGACGCTGAGCAAGAGCCACAGCACCATCTCGCCGGCCTTCCTCAAGGCCGCACTCAACGTGTCAGACGCTCAGGTGAAGGAGATCGTCGCGGAGAAGTGGACGAGGGCGACCTTATCAAGGTGACTCCGAACGAGGACAACCAGGTGCGACCGAAGAAGGTCCAGGAGAACATCGAGTTCGAGGACGTCCTGAAGGTCATTCACACACTGTCGAGATCGAACGTGTGTGCGAGTGGCGGAGGCGTCACAGACGCGCAAGAAGTGTGTGATGCTCTGTCGAGGCATCTTCGTCTGGAAGACAGTGCCGTGGTGCTCCCTAGCTCCACGGGTGTGATCGGCTGGCGTATCCCAGTCGACTCGATGATCCAGGCTATGCCACAACTCGTTGAGAACCTCCAGTCGGAGAGTGTGCTGCCTGCTGCTGAAGGTATCATGACCACTGATCTCTACCCGAAGATCCGATCGGTGGACGTCTGTGGAGGTCGCATTGTCGGTATTGCCAAGGGTGCGGGCATGATCGAGCCGAACATGGCCACGATGCTTTCGTACATCCTCACGGACCTCTCGGTGCCCCGCGAGACTCTGCGTCAGCTCCTGAAGGAAGTTGTTGGTGACACTTTTAACGCTATGAGCATCGACACGGACCAGAGCACATCTGATACGGTTGCGATTGTGTCGTCCGACCAGATTCCATTCGACTCAGACTCGCAGCTGCAGGAATTCAAGAAGGCACTTCACGAAGTGTGCCAGGGTTTGTGTGAAGACATTGTGCGCAATGGTGAGGGTGCACAACACGTGATGCGCGTCGTTGTGCGCGGTGCTCAGTCCAAGGAGATGGCTAAGGGTGTTGGCAAGTCGGTCGTCAACTCGCCACTGCTCAAGTGCGCGGTCGCCGGTAACGACCCGAACGTTGGTCGACTTGTCATGGCTGTTGGCAAGTACATGGGTCAGCACCACAAGGAGCTCGACATCACGCGCCGCATGAAGATTACCATGGGTGGCGTCTCGATCTTTGAGAACGGCGAGTTTGTGCTCAACAGTGAGATTGAGCACCTTCTTGTCGGCCACATGCAGCGCGCCCAGCTTACCGAGTCAACTCGTGGTGGCCTGGATGTCACCTCGCGAGACTACCCACCGCATGGCAACTTCGTCGAAATCGACATCGACCTTGGTATCGGCAATGAGAAGTCGCAGGTCCTCGGTATTGATCTTACCCACGAGTACGTCGCCATCAACGCTGATTACCGTTCCTAATGCCGCC---GAAGAAGGGGAAGACTGCGGCCGCGTACCTGGCGTCGCTCGGCCTGGATCCTTC---GGCTGGTGGAGCCGAGAAGAAGGAT---AACAAGAAGAAGAAGAAGGGTAAGAAGAAGGATGAAAGAAGCCACTCTCGGCCGCCGCCAAGCAGCTGC-TGGAGCGTCAGGCTGCGCTCAAGGCCGAGCAGGAGCGCCTCGAGAAGGAGCGCCTGGAGCAGGAAGCCCGTGAGCGTGAGGAGGAGGAGCGCCGCCTTGCGGAGATCGCCCGTGAAGAGGAGATCAAGCGCAAGAAGCGTGAGGCCAAGCAGGCCAAGATCGAGCGCCAGAAGAAGGAGGGCACGTACATGACCAAGGCGCAGAAGGCCAAGGCAGCGCGTGCACAGGCTGCTCTGGAGGCCATGCAGCAGGCGGGTCT---------------------------------GAAAAAGCAGCAGCCAAACAAGGAGGAAGAGCCAAAGAAGGAGAGGATGACGCACCTGCGGAGGAGGAGGCTCCTGACGCCTGGGACGATGAGGACTGGGAGTCGAGCGACCTCGTCGCCTCGCTTGAAGACAAGCTAGCAGAAGAAGGAACAGGAGCGCCTGCGCCAGCAAGGTATTGCGCTTGCCAAGCGTCAGCAGGAACTTGAGGAACAGGCTCGCCTGGATGCCGAGGAAGAGCGTCAGCGCTCGAAGGCGCAGCAGGAAGCTGACCGTAAGAAGGCCGAGGCTGCTGAGCGTCGTCGTGTCCGAGAGGAGGAGGCGCTTGCCAACGTGTCGACTGACAACCTTCGCTCGCCTATCTGTTGTATCATGGGTCACGTCGATACTGGTAAGACCAAGCTTCTCGACAACATCCGTCGTACCAAGGTCCAGGATGCCGAAGCTGGTGGTATTACCCAGCAAATTGGTGCGACTTTCTTCCCAGTGGAAGCCATCAAGCAGAAGACTGCTCGTCTCGCAGACAGTATGAAGCTTCAGTACAAGCTGCCAGGTCTCCTTGTCATTGATACGCCTGGTCACGAGT-ATTCACCAACCTTCGTTCTCGTG-----------------------------------------------------------------------------------------------------------------------------------------------------ACCATGCCTGACATGCCGATCCAGGAGGCACTCAAGCATCAGAACGAGCACGTTATCCGTGAATTCGAGGACCGTACCAAGCAGATCATTGTCGAGTTTGCCGAGCAGAAGCTCAACGCTGAGATCTACTGGCGCAACAAGGACCTTGGTCGCACGGTCTCGCTCATTCCAACGAGTGCTATCTCGGGTGAGGGTGTGCCTGATCTTTTGGGCATGCTCACCAAGCTCACGCAAGAGCGTATGGCGAAGTCGTTGGCGTTCGTGGACATCCTCCAGTGTACCGTTCTCGAAGTCAAGGTGATTGAAGGTCTTGGTACCACCATTGATACTGTGCTTGTCAATGGTCGTCTGAATGAAGGCGACAGGATTGTT------------------------------------------------------------------GAAATCCGTGTCAAGGGCGAGTACGTGCACCACCAGACCATCAAGGCCGCTATGGGTGTCAAGATCTGTGCTCACGGTCTTGAGAAGGCTGTTGCTGGTACGCAGATCTACGTGGTTGGCCCAGACGATGATGAAGAAGAGCTCAAGGATGCCGTCATGTCCGATCTTACGGGCATTTTGGACTCTGTCAAGGCCACGAAGCGTGGTGTCATGGTGCAGGCGTCGACGCTGGGTGCTCTTGAGGCCCTTCTCGAGTTCCTGCGCACGTGTGACCCACCGATCCCAGTCTCGTGTGTCAACATCGGTCCTGTGCACAAGAAGGACGTCATGCGTGCGTCCGTGCAGCTCGAGCACCAGAAGGAATTCGCCACCATTTTGGCATTCGATGTCAAGGTCCACAACGATGCCACTGAGTTGGCGCAAGAGCTGGGTGTGCGCATTTTTACGGCTGACATTATCTACCACCTCTTCGACCAGTTCACAGCCTACATGGAGAACTTCCGGCAAGCGCGTCGTGAGGAGATGGCCGATGTCGCTGTCTTCCCGTGCGTGTGTAAGATTCTGCCCAACTGCATCTTCAACAAGAAGGACCCCATCATTCTCGGTGTGGACGTCGAGGAAGGTATCCTCAAGGTTGGTACGCCGTTGGTAGTCCCAAGTGCTGGTA---TGCTTCTCGTGGGTAAGGTTGGCTCGATTGAGCGTGACCACAAGGAAGTCGATCGCGCCAAGAAGGGTGCATCCGTGGCCGTGCGTATCGATAACGAAGGCTCGGTCATGTACGGCCGTCACTTTGACCACAAGAACCGTCTCGTGTCGCGCCTCACGCGTGCCAGTATCGATGCCCTGAAAGAGAACTTCCGAGATGATCTCCAGAAGGAAGACTGGCAGCTGGTGATCAAGCTCAAGAAGGTCTTCGACATCATT-------------------------ATGGCCGTTTTTTCCTTGACAG--TGGAGCGCTACTTCGTCCTCGATGGCGCGCAGCTCCGCTACTACCGGAAGCGTGGCGATCCAGCGCCTCGTGGCACGTACGTGCTCACGGATGGTTGCCTCGTGAGCAACGTGTTCAATTCGGAAGAGAAGCACCGTCACCATGGGCCCGTGTGGATGTTCCGCATCACGTGAGAAATCGTACAAGAAAGATCGGTTCCTGGACCTTGGCGCCAAGACGGAGGAGAACGCCAAGGAGTGGAAGAAGGCCATTGAGAATGCCGTGCGCTCGATCCGCGAGCTTGTGTTGCACCAGCGCAAGCTGCGCACCGACGCTGAGCT---CTTCCCGATCTCGGGCCCGCAGCGTGAGCCACCGCGTCTCCTCAAGGCGGATGAAGACACGGTCTCACTGTTCAACCTGGACGCGGATAACGTACGCACGGAGCAGGGATGGTGGCTAGCGCGTGTGGAGGAGGGGCTACGTGTGATGCAGGAGTGTCCACTGGGCCACCAGGTCGCAGCCTCGCTCTACGCGCAGCACGCTCAGACCACGAATGGGCTCATTATGTCGTCAACCATCGCTGCCATGATCTCGTTCAGCATCATGTACTCATTCGTGAGTAACATGCTGCTGTGCTTCCTCACTGCGGCCGTCGCTGTTGCTGTGTGCGGCATCGCGATCACTCCAACGCAGAACATCGAGATCCCGTCGCTACGTGTCCAGCAAGCTGTGCATGGGTCACCAACTGAAGTCTTCCGTCTGCTCATGAACCCGAAGCGCTACCAGCGCTGGGATCCATCAGTCGCTACTATGCGCGTGATCCAGCAGATGGATGACCAGGCCGATATCGTGCACATCACGCAACGCCCCGTGTTCATTTGGCCAATGTGGCAAAAGCCGCGTGACCTCGTGCTCATGCGCTACTGGCGTCGGGAGGAAGACGGCTCATACTTCGTCATGTACCAAAGTGTTGAGCACCCTGAGTGCCGTGTGCGCCACAACTACGTGCGAGCCACAGTGCTCGGCGGCGGCTTCGTCATCGCCCCTCAAAAAATGCCAAGC---GGTACGATGCGTTCGCTCGTGACCTATGCTCTGCGTTACGACCCAGGCGGGTGGACGTCGCGCATCTACCACCAGCTCGGTATGGATGTCGATCTGGTCATGCCAATGCTGCGTAGTGTCGTGGGTATCCGCGATGAGATGAGCGGAAATGACTTTATCACGCCAATGGTGACGGTGAACGGCGGTGCCGTGAGCCCCAGCAACTGGAGACGTGTAGATCCTTCCACAAACACGGTCGTGGGAGTGCAGCGCTTGAAG---ACAACACTACCACAGAAGATGTGGGCCGAACCCGATGCCTCCCTGTTCC------------------------------------AAATCCCGTCGTCCCAGGCCAAGTTCCACCTTGTTGGTGTCGATTTTTTCTCGTTCGAAAGCACCAGTGATCGCTACCACATCTCGGCCCGCGCTGACAGTGTTGGAAAGACTTC---GGGCAAGTTCACATTCGTCGTCAACATGATTATTCCAGGTCCGGAGAACACGACGCTGGCCTTCTACTTCCACCCTGCCAAGGACAATATCTTTGACGATGGATCTCCGTTCTCGGAACTGCTTAACGACTTCATCGATGGCGACGACGCATTCCGCAACTCCCGTTTTAAGCTCATTCCGACCGTCGTGGAAGGCAGCTTCATCATTAAGCAGTCAGTCGGTTCCAAGCCAACGCTCCTCGGTAACAAGCTCAAGTGCCCGTACTACAGGGGCGACACGTACTTCGAAGTGGACGTGGATATCTCGTCAAACTCGGTCGCGAACACGGTTGTTGGCATGGTGCAGGGAGTGACCAAGTCCCTGGTCGTGGACATGGCGTTCTTGCTTGAGGCCCAAGGCGAGGAAGAGCTGCCAGAGGTCCTGCTCGGAGCTGTGCGTCTGCAGCACGTGTCCCTCGACAAACCTACCCGTGTACCAAAGGATGGGCAACG--CAGCAAGGACGAGTTCACGTTCGGACACGTTCACGCAGGCGCTGCGCGTCAACCTGCAGCGCCTCATCGTGTATGCCAAGTCGGCCGACACGGCGCTGCAGCGAGAGGTGGCCGAGAAACTGGCGAATGAGGCCGTCAAGACCGACAGGTGCAGATCGTGGAGCTCGATGGACTGCAGCTGCTACTCCCGTTGACGCAGTCCAAGGACACGGAAGTGCAGCGTCTCGCGGCGCATGCGCTCGCGAACCTTTCGGTTAACTCTGATAACCAGACCAAGATGGCGACGGAGGGAGGCGTTGACATGCTCATCGACCTGCTGGGGAGCACGAACGAGCATGTGCAACGCCAGGCAGCCAAGGCGCTGGCGAATCTTGGTGTGAACGTGGACAACAAGGAACGCATCGCCAAGGCTGGAGGCATCAAGCCTCTGATCTCACTCGCCAGCTCCAAACACATTGGCGTCGCAGTCGAAGCTATTGCAGCACTTGCCAATCTCGCTGTGAATG---ACGCGAACGAGGTCGAGATAGCTCGCGAGGGTGGACTGAAGCCCATCATCGACAGTGCTCGCTCCGACTCGGTGGAGCTTCAGTCACAGGTGGCTCGTGCGCTGCGTAATCTCTCAGTGAACCCGGAGAACAAGCAACTCATCATTGATCTTCGTGGCGTCGACGCGCTGCAGACACTGATAGAGTTTGC-----ATCCAAAATCACAGTGATCCA----------------------------------------------------------------------------------------------------------------------------------------------------------------------------------------------------------------------------------------------------------------------------------------------------------------------------------------------------------------------------------------------------------------------------------------------------------------------------------------------------------------------------------------------------------------------------------------------------------------------------------------------------------------------------------------------------------------------------------------------------------------------------------------ATGTTGACGCATCTCGAGAAAACATCGCCCTTTAGCAAGATCCCCATCCTTGCGAACCGCTTTCTTCTGTCTCGGCTTCTTGGAAAAGGAGGGAACGGGGAAGTCTGGGATGTCATCGACTACAAAGATCAGTCCACCCGATGGGCGCTGAAGCTGTCTGTTTCCCACAAGCACGCGCAGCGAGAATTTTACACCCACATTGAAGCATCAGA--ATATTGCCCGCGTTGGGGATGCTGCGTTCACCATTGAATACCGTCAACAGCTCTACACAGCGTTTACAGTCGAGTGCGTCGAATCGGATCTTCAGCAAGTTATCG----------------------------------------------------------------------------------TGAAGATTGCACACTATGACCTGAAGCCAACGAACATCCTCGTTGGACACAACCAGGGTATCAAGCTT------ACCGACTTCGACCTTGCACGAAACGTCCACGAACCGATCTCGTCGTCTGTTGTTGGAACTTTGAGGTATCTTCCGCCAGAATGCTTCAAGCACAACTTCGGCGA---------TTGTCAGGGTACGGCTGAGAAGGCAGACATCTGGATGGTTGGTGTTGTGTACTGCATAATGCTATGGGGAAAGCACCCGATCATCCCAGACAGGGCGTCACATGACGATGCCCGATCAATGTTGGCTCGATACAACGGC---CAAATCCAGTATCAGCGGCCCGTATCCGACCTGTCAAGATGGATCATCCAAGGATGCATGCATCCTGATCCACGTTGCCGACCGACTGCCTACCAGCTGCTGCAAGCACTTCAG

Pythium_irregulare ACGACTACCACTCGGAGGTGGCGCGCGAGCTGGTGGCGTTTTTCCACTCGTTCTACCGCTTCGGCGCGGCGTCCAACGTCTTCGACCTGTTCCTGCACAACGTGCTCTTCGGTTCGCTCGCGATTGGTGCGATCGTGTTTATCGCGCTCGCAATCATCGTCGTGCGCCGGTGCTTCCTGCACATCCGCAGGCGTATGTGCGCAAGATCTCGATCGAGTCGAACGCGACCGAGTTCGCGGCCGTGCTGCTCATGACGTTCATCGCCGTGAGCGCGCTCAGCGGCCTGCTGGGAGAGGCGGCGTCGCAGCCAGCGACGTCATCCGCACACGCGCCGAGAACCTTGTGGTGTCGTTCGATGAGAGCGACTTGCCACCAGAGGCGTACAAGCTGTCAGTGGAGGCCATGGCCTGTTCCACTCGGCCAAGGATCTCGTGAACACGACAGACATACTGCTGCCTAAGCACTACGGCGAGATGGCGGAGGAGTGGCAGTTCAGTTACTTCATGCTCAAGTCGGCGACGAACGCGGCGATCCTGTCGGTCGCGCTCTCGAGCTTCCTGCTAATCTCGGCGATCGGCTGGTCGATGGTCTCGCCACTGCGCCTCTCGATCTTGGTCATCTTGAGCGTCATCCCCGTGTCGCACACACTGATCGGCGTGTACCTGTCGTCGACGATCATGACGTCAGACTTCTGCGCCGCGCCAATGAACTCCACGGCGACGCTGCTGCACCCGTGCCCCGTGAACACCTCAGCCCGCTGGTCGCGCCGGCGGTCGATCTATGCGCAGAAGCGCGGCGACATCGGCAAGCGCATGAAGAGCGAGTTCCTGGACCCGATCGGCGCAGATCTGGGCGTCATGCTCGGGTACTTCATCGGCACGGTGTCAAACTGGGAGAGCTTCCAGCTGATCGAGACGTGGCGCTGGCCGTTCGTCGTGCAGTTCATCGCCATGTGTCCGATTATGGTGGCGATTTTCTTTGTCCCCAAGAAGCACCTGATCATCCGCAGCGAAGCACGACAACAGCAACAACTGGTGCGTGGCGCGCCCGTGAGTCGCAGCGGCTTCCTGCACAAAGAAGAAGAGGACGTACTACTCGCGAGTGCCGGCATGCTACGTACGGCGCGTTCGACACGAAGTGTACGAGCAGGGATCGTTTTCCGACGGGCTCTACGAGCTCGCGCACATCCCGATCTTCTGTCTGATCGTCGGCGGGCTCACAGCCGTGTACTTTGTCGTCACGGGCGTGCAGTACTGGAGCACGATCTTCATGATCAAGTCGCTGCACGCGTCCAAGTACCTCGTGAATGGGCTCTTCGTGGTCGTCGCTGGCACGGGTCCGATCTTTGGCGTGTTCTATGGCGGCTGGCTCATCGACCGCTACGGCGGGTACATTGGCGTGGAGCAGCGCGCCAAGTCGCTGGGCATCTGTATGGTGTTGGGCATCACGGCGTTCGGCATCTCGGCGGTCACGACGTTTTTCAACGACATCTATATCACGGCGGCGTTCCTGTGGCTGCTGCTCTTCTTCGGCGGCTCGATTCTGCCGGCGCACCGCGCGCTGGCGTCGTCGCTCTCGGTCATGGTCTTCAACCTCTTTGGCTACTCGCTGTCGCATGAGCGGTCTGGAGATGGTCGTCGGCGCCAAGTGCTACGTGCCGGACGAGACGCACGTGTGGCTCGCGGCTGAGGTGGTGCGTGAGGAGAACTCCGGGGACGGCAAAGTGCGCAAGATCCACGCGCGCGTGGAGCTGCCACCCAGAGTTTGAAGAACGCATCGTCGACATGAACGACAAGCAGACCAAGGCGTTGCTGCGGTGCACCAGCTCGACTCGCTCCCGTACCAGAACGAAAACGTCGGCGAGTACGGTATTGAAGACATGATCACACTCAACTACTTGCACGAAGCCGCGATCCTGTTCAACGTCAAGACGCGTTTCCTGCAGAAGCTCCCATACACGTACACGGGCGACATTTGTATCGCCGTGAACCCGTACCAGTGGCTCCCAGATCTCTATGCGGACGAGCAGCACCTGCGCTATTTGAACCAACCAAAGGAAGAGCTCCCGCCGCACGTGTACGCGACGTCGGTGGCTTCCTACGACAACATGCGTCGCACGGAGCGCAACCAGTCGATCTTGGTGTCGGGAGAGTCTGGTGCCGGTAAGACCGAGACCACGAAGATCTTAATGAACCACTTGGCGACTATTGCCGGCGGTTTGAACAACTCGACGATCAAGAAGATTATCGAAGTTAACCCTTTGCTTGAGTCTTTTGGTAACGCAAAGACCGTGCGTAATGACAACAGTTCGCGTTTCGGCAAGTTCACGCAGCTGCAGTTTGACCGCGAAGGCACGCTGGTCGGCGCCAAGTGCCGCACATACCTGTTGGAAAAGACGCGTGTAATCAACCACGAAGAACCAGAGCGAAACTACCACATCTTTTATCAGTGGA---AGGTGACTCCGCTCAGGAACTGCTCTTGGACTCGTCGAAGACATATGCGTACACGGGCAACAACAACGCGGTGAAGATCGAAGGCATGTCGGATGCCAAGCACTTTGAGAAGACGCGCGTTGCGCTGGAGCTCATTGGGCTCACGCCAAACGAGCAAAAGCCACTTTTCGAGGTCTTGGCTGGCGTGCTCCAGCTCGGTGAAGTCTCGCTGCTGTCCAACCCAGCGAATGACGAAGAATCGCTGATCGCGGAGGGTGATGCGGGCGCGCTCGCGTGTGTTCAGCTTTTGGGCGTGACGTGGGAAGCGATTGAGAAGGCGCTCTGCTCGCGCACGATGCGTGCCGTGAACGATGTCTACAGCGTGCCATTGCGCAAGGAGCAGGCCATGGATTGCCGTGACGCGCTTGCGAAAGCTATTTATTCGAACGTGTTCGACTGGCTCGTGTCGTCGATCAACCACTCGCTCTCTGACGACAGCAACATGTTCAACCACGTCGGTGTGCTCGATATTTTCGGTTTCGAGCACTTCAAGCACAACTCGTTTGAACAATTCTGCATCAACTATGCCAACGAAAAACTGCAGCAGAAGTTCACGCAAGATGTGTTCAAGACCGTGCAGATCGAGTATGAGGAAGAAGGCATCACGTGGAACCACATCGAGTACGCCGATAACCAGGACGTGCTGACTGTGATTGAGTCGAAGATGGGTATCATCTCGCTCTTGAACGAAGAGCTGATGCGTCCAAAGGGTAACGAGGAGTCGTTCATGTCGAAGGTGGCGTCGCTGCACAAGGAA---GACATGGCGCACGTCATTGAGTTCCCAAGAACTTCGCGCACGGAGTTTTTGATCAAGCACTACGCCGCGCCTGTCATGTACGACTCGGTCGGTTTCTTGGAGAAGCACAAGGATGCCTTGCTCCCAGACTTGTCGGAATTGATGCGTAGCTCTTCGAAGCCATTCATCTCGGCGCTGTTTGAGCCAAAGCCGGCGCCAAAGTCGCCGCAAGCG---TGGTGGCGGTGGCGCGTTGACTCTGTCCACTGTCGGAACACAGTTCAAGGAGAGTTTGGCGGAGTTGATGACCACGATTCAAGCCACGAAGGTGCACTACGTGCGATGCATCAAGCCGAACTCGATCAA---------GAGTTCTACGACAATGGACCAAGCGATGGTTGTCTCTCAGCTGCGATGCGCTGGTGTGATTGAAGCGATTCGTATTTCCCGCGCTGCGTACCCGAACCGTCTGCAGCACACGGAGATCCTGGACAAGTTCTGGCTGTTTGTTCCTCGTCAGGGAGAAACCGCGAGCGAGAAATGCCAGTTGCTGATGGAGAAGCTCAAGTTGACGACTCCAGAGCAGTACCAAATGGGTAAGACTCGTGTGTATTTCCAGCTCGGCGTACTTGAAGAGCTCGAGGACCGTCGTAAGAAGTTCTTGGACGCCAAGGCGACGTACCTCCAGGGTGTGATGAGAACGTTCACCCAGAGAATTAAATACGTGCGACAGCTGGCTGCGATCATCAAGTTCTGTTATTCGCTGCGTCATCGCCATGAGAAGATACAACACGTTCATGAATGGTCTTGTGAAGGCACAAGCACGATGGAGAGGTATTGAAGGACGCAAGCACTACACGGAAGTGCGAAGCAACCACTACGCCGTGATCATCCAGCGATACGTGCGCGGATTCGTTTTGCGTCACCAGTACACGAAGGTGCGCGCAAGCATCATTCGTGTGCAAGCTATGGCGCGAATGAAGATCCAGCGTCCAAAGTACCTCGCTGCTCTCCAGGAAGAGAAGCTGCAAGCCGATATGGTGTACCAGCTGAGCCAGCTCAAGGCTCGTCTTCAAGAAGAGCAGGAGCGCAATGCCCGATT------GCAAGAAGAGCGCAACAACGTCGC--GACCACGCTGCCGCT---------------------------------------------CTCCAGGAAGAGAACTCGAAGATCCGTGCCAAGAACGAGGACTTGAAGAACACGGTGAAGCAGCTGAAGGCTGAAGTTGAGAAATACAAGTCTGAAAAGGAGGTGAGCCACGCTAGCCTGTACGTGAAGATTCGGCAGTTGGAAGACACTGTTCGCGAGAAGGAGAAGCGTGTTACTCAGCTGGAGGGTGACAACCTGAAGCTGACGGACCAAGTGCAGGACCTGATCGCGAACGGCCACGTGCAATTCGAGAAGAAGACCAAGGAGAAGCGCTCGATTTTCCGCACGCTCGGCTCCAAGAAGGAGAAGAAG---------------------GAAGCCGTGTTGCTGGACTCGCTGACCGGGGACGACGACGTGAACGGATCGGACGTGCGCGCGTCGAATGTGCGTCACCTCCCAAAGCTGCTCCCTTCCATGAACATGTCGAAG---AAGTTTTGGGGCAAGTCCAAG---GGCGAGAAT------------GGCGCCGACGGCAACGACGATCGCGATTCGAGCGGCAACGACTCTCAGAACGGCGACCTCGCAACACGATTGTCAAGAGCATGGAGGTCGGTGTGACTGGTGCGATGACGAACCTGAAGGGCAAGATG---------GCGGCTGTGAAGACCATGTACGAGTGAAGCGTTCGGGCAGGACCGACCAATCCGCGAATCGTTCAACTTGGATGCGCTGCCAGAAGTGTCGCTGCCTGTTGGATGGGAAGCCAAGGTGTCTCGCTCCACTGGTCGCGTGTACTACGTGAACCGCAAGCTCGGCAAGTCTCAGTTTGAGCGTCCAACGCTCGCGTCGCTCAA------GGCGCAGAAGTTGGCACGTCAGAAATCTGTGAGCGGTCCGAAAGGCTGTGGTCGCAGACTTTGGATGTGGCGACGCGCGCCTGGCCGAGAGCGTGCCGAACAAAGTGCACTCGTTCGACCTCGTTTCACTGGACATCGCGGTGTACTGCCTCGCGCTCATGGGCACGTCCGTGCGCGAGTTTATCATGGAGGCCCACCGCGTGCTCAAGCCAAACGGCGTGCTTCAAGTGGCCGAGGTTAAGAGCCGCTTCGAGACCGAGTCGCTCGGCGGATGGTGGCGTCTTTCCTCTCCGTGTCCGGCGACGCCGTGGTGCTCGTGCGCGACAAGACGCTCTTCGTGGCGAGCGCAAGTATGTGGTGATCGCCGGCGTGAAGACCGGCGAAGCCGTCGCGATGCCGTTCCCGGACATCAACCGCGACGTCAAGATGCTGCTCGGCCACACGACATCGATGATTGTGCACTTGACCGTGAACCTCGACGCTGTTGCTCACGGGCGACCGCGACGAGAAGATCCGTGTCTCGGGCTTCCCGCGCACGTCGCTCATCCAGAGCTACTGCCTGGGCCACAGCGAGAGTCTCACTAAGGTGGCGACCAGCTCGCTCACGCCAGACCTCGCGGTCTCGACATCGCTGGACAACACAATTAAGCTCTGGAGCATGTCAACGGGCACGCTTGCGGTCTCGGCGCAGTCGACCTTGTGGCGGTCGTGCTCAACAACCAGTCGGTGCGTCTCTTCGCGATTGTTGTGCGCCGTTCGTGGGTTTCTACAATGTCAACAAGGGGCGACGAAGTGCTGGCAGTTTGTGCGCGACTTCCGCAAGTTGCAGAACGACCCGCCGTCAGGCGTCTCCGGCGCGCCGATGGACAACAACATCATGCTCTGGCAAGCCGTCATCTTTGGACCGGATGACACGCCGTGGGAGGGTGGTACTTTCAACCTGACGCTCGAGTTCAGCGAAGACTACCCGAACAAAGCACCGACCGTGCGATTTATCACAAAGATGTTCCATCCAAACATCTACAATGACGGCCAGATCTGTCTCGATATCCTGCAGAACCAATGGTCACCGATCTACGATATCTCGGCGATCCTGACGTCGATTCAGTCGCTGCTGTGCGACCCAAACCCGAACTCGCCGGCGAACTCGGAGGCCGCGCGCCTTTTCCAGGAAAACCGCCGCGAGTACAATCGTCGCGTGCGCGAAATCGTCGAGCAGAGTTGGGTTGCGATCTATGGTGTCGGCCGCCAAGATGCGTGGTGCTGAGAACCGCATGAACGCTGGTCGTCCATTCACTGCGTGGTTGGACAACGTGTCCTCGGACCGTGGTCTCTGCGGTGGTATCAACTCGGGTATTGCCAAGGCCGTGCGCAAGCAGTGTCGGCGGAGCTGTTCGTGATCGGTGACAAGGGTCGCGCCCAGCTTCGTCGCGACCTCGGTAACCAGATCCGCGGTAACGTGACTGAGACCTTCGCGTCGCCACCAAACTTCACGGTTGCCAGTGCCATCGCTGAGGGTGTGGAGAACGAGAAGAGAGTTCGCGTATGTCGGCCATGGAGAACGCCACCACGAACGCCCAGGAACTCACGCTCACCCTTGTGTACAACAAGGCGCGTCAATCCCGCATTACGACGGAGTTGATTGAAATTATTTCCGGTGCCGCGTCTCTCGACCCTCACTGGCTCCGTCATCGACGTGCAGGACGCCGCGCAGTTCGACGAGCTCACGGCGCGCGAGTCGACGCTGAGCATCTCGTTCTTCTGGGCGGACTTCCAGCTCTGCCGCCCGAACGGCCAAATCGACGTGGGGCAAGAGCGTGCTCGAGAAGCTCGAAGGCGCGAACGTCGCCGAGCTCGCCAAGCGCGTCTCGAGTACCGTCTCAAGAAGCTCATCAACGCCTCGCCAGTGATGCTCTTCATGAAGGGCAATCCGAGCGACCCAAAGTGCGGCTTCAGTCGCCAGGTCGTCGCGCTCTTGAACGAAGAGAACATCCAGTATGGCACGTTCGACATCCTCAACGACGAAGAAGTACGTCAGGGCTTGAAGCAGTTCTCCAACTGGCCGACATTCCCGCAGCTCTACGTGAACGGCGCGCTGATCGGCGGGCTCGATATCCTCAAGGAAATGAAGTCGGAGGGCTCGATTGTCGAAGAGCTCGGCGTCGAAGAAGGTGAGGCCGCGTTCAACGAGAGTCTCCGCGCGCTCGTCAACTCGGCGCCTGTGCTGCTCTTTATGAAGGTTCGACATCTTGTGTGGAGCTACGAGCGTCCAAAGCACGAGATCTCCAAGATGGCGACTTTCGCGCCGACGTCGTTCCGCAAAGACATGTGCGGAGAGCATCGTGTGGAGTCCGCAATCGCTTGGCGATGGCATTTCGCGATCCGATCGACGGCATGGGCGAGTTCTGCGTCGTGATGCTGCTCGAGTCACGTCCAAATCCACGACGCTGCACTCGCGCGGCATCTCGGCGATCGAGTGGAAAGTGGAACGTGCTGCACCGGGAGCACCGCTCGGAGGTCAAGTCGATCTGTGTGCACTCGCAGCGCCAAGCGATTTACACAGGCGGCTTTGATGGCCAGGTGATCCGCTACGACATGCACAAGTTCGCACCGCACACCGTGATGGAGCGTCGCAAGCGCATCTCGAAGATCAACGCCGTGCTGGAGCACCCGCACAACCCGAACATTTTGCTCGTGAGCAGCGTCGAGCAGGGAGCACAGCATCCTGCTGCACGACCTGCAGCGGTACAACAGCAGTCTGACCTCCAAGTCGATGAGCCAGTACATCGTGCCACGTTGGTCGCCCGCGGGTATGCATGTGTCCTGCGGGAGCAAATGCGCTACTGTCATGGGAGCTTCTCGACACCGACGTCCGCGACCATCGGCGCGTCGTTCTTGCAGAAGCGCATCATCGTGGGCGGGAAGATGACGCTGCAGATCTGGGACACGGCCGGACAGGAGCGCTTCCGTTCCATGGCGCCGATGTACTACCGAAACGCCAAAGCCGCGATCCTCGTGTTTGACTTGCTGAATGAGGCGACGTTCGAGAAGGTCAAGGAGTGGCTGCATCTCCGACATCACGTGGGCGACGATATGTGGGCAACAAGTGCGACGTTTCAACTTTGCGAAGGCGCAGCAGTTCGCGCAGGAGATCGGCGCGCTGGTGTTCCGCACGAGCGCGAAGACGGGCGATGGCGTGCAGAACCTGTTCGAGAGCGTCGCAGTGCAGCTGCTGAAGCGCCATGAGGAGGAGGAGAAACGAGCTTCTTCGTCGTGTGCGTGCCCGCGGACGGCGAGAAGCAGCTAGAGGAATGGGCGGTGGCGCTTCCGGACGACAAGGAGGCGCAGATCGGCTGCCTCACGGACCGCCTGCGCGCGCACTTCAAACAGAGCGGCATCGACAGCGGAGCAGAGATTTTCAAGAAGCAGTTGCTGAGCCAACTGCCCAAGGGCCAAACCATGACCGACGACATGCTGCAGATGATGCATGGACAGCCTCGTGGACTCGTTCCCGCTCATGCTCAACACGCGCGCCGTGAACCACGTTCGAAGTGCGCGGCGACGCGTTCATCGCGCGCGTGTTTGACAGACGACTTTGTGCGCATGGACTTCCGCGTGTCCGAGGTGTCGATGGACGCCGACTGGATCAAGATCGGTAAGGAGCAGAAGTGACGCAGTCGCTCGAAGAGATCCTCGCGCTTGGCCAGGGACTCGACGACGAAGACTACAAGGCAGAACTCGACGTGCTGCAGAACGACCCGAACTCGAACCTCTACTCGGCTGTGACGTTCGAGGAGCTCAACCTCCCAGCGATGCTGCTGAAGGGCGTGTATTCGATGAAGTTCTCGAAGCCATCGAAGATCCAGTCCGTCGCGTTGCCATTGATCCTCGCCGACCCGCCTGAGAATCTGATCGGCCAGGCGCAGAGCGGATTCCGACCCGCGAGCTTGTGCGCCAGATTATGGCGGTCGTGAACGCGATGGGCAAGTTGTGCAGACGTTCCTCGCGATCCCGGGTGTCGACGTCGCCAAGGGCGAACAGGTGCAGGCACAAGTGGTGGTCGGCACGCCCGGCCGCGTCGAAGGGCTCATCAAGAAGAAGCAGCTTGACACGCGCCACATCAAGATCTTTGTGCTCGACGAAGCCGACGTGATGGTGTCGGAGGACGGCATGCGCGACCGCTCGGTCGCGATCAAGAAGATGATCAAGAACCGCAGCTGCCAGTACCTGCTCTTCTCGGCGACGTACGCCGACGAAGTGCGCGACTTTGCGCAGCGCATGGTGCCGGACCACAACATCATCACCGTCAAGAAGGAGAAGCTGACACTGGACGGGATCAAGCAGTTCTGGATTGACTGCAAGACCAAGGACAACAAGTTCAAGGTGCTCTCGGACATCTTTGCTGTGCTCACGATCGGCAAGTGTGTGATCTTTGTGCAATCCCGCGACACGTGCAAAGAGCTCACGAAGCGCATGCGTGCGGGCGGCCACTCGGTCGGCATTTTGCACGGCGCTGACATGGCGCGCGAAGTGCGCGACCAGGTCATCGACGAGTTCCGCGCCGGCACGACGAACGTGCTGATCACAACGAACGTGCTCGCGCGTGGCATTGACGTCGCGGGCGTGTCGCTCGTTGTCAACTTTGATATCCCGCTCAACCGAGACCAACAGCCGGACCCAGAGACGTACTTGCACCGCATCGGTCGTACCGGTCGCTTCGGTCGCAAGGGCTGCGCGATCAACTTTGTGCACGACGACCGAAGCAAGCAGCATCTGGCGGAGATCGAGGCCTACTACGCGCGCGAGATCGTGCAGGCGCCTGCCGACGATATTGAAGAACTGGAGAAACTGCTCGCCTGGGGGGCAGCAAGCGCCGCCTGGCCAAGTTCGGCAAGGAGCAGCGCAAGGGCATGAGTATCTACCCGCGCGAGCCGAAGAAGAAGCCAAAGGGCAAGGACAAGACGTACTACTACCTCAAGGATGTCATGTACCTGGCGCACGAGCCCGTGCTCGAGAAGTTCCGCGAGTTCAAGACGTTCATGAAGAAAGTCACCAAGGCCATGGGCAAGCGCGACTTTGAGGACGCCAAACGCAAGCACGACAACCGCCCGATGTACACGCTTGACCACATCGTGAAGGAGCGATACCCGCGCTTCGTGGATGCGCTCGGCGACATGGATGACGCGCTCTGTGGAACAACTACGTGGCCACCTCGCGCTCGCTCACCAAGGTGTTTGTGAGTATCAAGGGCATCTACTACCAGGCGAACGTGCAGGGCCAGACCGTGACGTGGCTCGTGCCGCACCAGTTCACGCCGACCATGGATAAGCGCGTGGATGTGCGTGTGATGCTCACATTCCTTGAGTTCTACGAAGTGTTGCTCAAGTTCGTCAACTACCAGCTGTACACGGACCTGGGCGTTGCGTACCCACCAAAGATCGACCTGCAGTTGGACCAGGCGGGTGCCCAGCTCGCCGCCTTGCAGTTAGCAGCAGAAGGAGTCGGAGAAGCGCATCAAGCTACAACGACCAGCGCAAGGAGGCGAGCGAAAAGAGTCTATTCAGCGGACTGTTCTTCTACCTGTCGCGTGAGGTGCCGTCGGCGTGCCTGGAGCTCGTGCTCCGCTCGCACGGCGCGCAGATCGGCTGGGACGGCGCTGGCTCGCCGTACACGGAAAAGGACGAGCGCATCACACATCACATCATGGACCGTCCCCACCAGGGCCACCGCTACTTCAACCGCGAGTATGTGCAGCCGCAATGGGTGTTTGACAGTATCAACAACGGTCTGTTGCTGCCGCTCGCGCGCTACCGCAAGCAGCTCAACAAGCTCAAGTCGGCCTCACAGGTGCTGCGCGAGGTGGGCGACCTCGACAACGGCGACGACGTCTGGAAGCGTACGCTGCTGGTATCAAGGCCGAGCTGGACGGTAACAAAAAGTCGAAGAAGGTGAAGCGCGAGGAGGAGGAGGAGGAGCTCCACGAGCTGGCCAAGAGCATGATGTCGAAAAAGTCGAAGCGCTTATACGACCGCATGCAGTTCGGTCTGGGCAAGAAGGCGGACAAGATCCAGGTGCTGGAGGCCAAGCGCGCCAAGTTAGACGCAAGACCAAGAAGCTCGTGGCTGGCGCGCTCTCCGGCATGATCTCGGCGCTGGCTTTCCAGCCGCTCGGGCGATGCGCGGGCGTGGCACGTCATCCACGCTCATCCGCGTCGCAGGCGGCGCCGGACTCTACTTCACGACGCTCGACCACTGCATGACCATGTTCCCCACGTCGACGATCAACACGTTCCTCTCGGGCGCATTTGCGCGGACGTTCGCGGGCTGTGTCATGTCTCCGTTCACGATCGTGAAGGCACGCATGGAAGGCACCGGGCACGTACAGCAGCAAAGGGACGTGCCATTCTCGGGGCTCTACGTTCTCATCTACACAGCACCCGGCGGACGTGGTGAAGACGCGCATGCATGCGCCAGACGGTGCGCAAGATCGTTAGCGAGGAGGGCTGGCGGGGCTTCAAGAAGGGCATCGCGCCGCGGGTGCTCAAGCGCACGCTCTCGACCGCCGTCACCTGGACCATCTACGAGCTACTCGTTCGACCCCTCCGGTCTCGGCCACGCGAAGGAGGCGTTCCAGATCGCCAAGGAGACCGAGAAGACCAAGCAGCTCGAACACACCTCCAAGGTGAAGGAGAACGAAGCGCTCTACAAGCAGTACGAGATCGTGCGCGTGCAGAAGGAAGGCGAAGAGCGCCGCAAGACCCTCGAAGAGGAGACGCGCCAGCACCAGCAGCGCGCGCAGTACCAAGATCAGCTCAAGCGCAAACAGTATGCCGATCAGCTGGCGTACCAGAAGCAGATGAAGGAGCAGGAGCTCAAGAAGCAGGAAGAGATGCTCGCACGTCAGGAGAGCTCGCGACGAAAGACCCTCGAGTACGAAGCCGAGCTTCGTCAGAAGACCGAGCTCTCCAAGGTCGCGGCCGAGGTCGAAGGTAAGATCAAGCAGGAGCGATTGAACCACGATCTACACTTGGAAGAAGCGCGTGTGCGCGCCAAGGAATACCGTGAGACCGTCATGGAAAGTATCAAGCTCGCGGGTTCTACGATCGGTTCCGGTGTCATGGCGTTTGTGGACGACAAGGAGAAGCTCACTGCCACCGTCGCGTCGCTCACCGCCCTTGCGCTTGGTGTGTACACTGCGAAAACCGGTACGGGCGTCGCAGGCAAGTACATCGAAGCGCGTCTCGGCAAGCCGTCGCTTGTGCGTGAGACCTCGCGCCGCACTGTTGCGCAGGTTGTCGCGAACCCAGTGCCATCGATCAAGCGCGTACTGCGTCTCCAGAAGCCGCATGATGCGCTGGAAGGCGTTGTCTTGGAACCAAAGCTTGACGAGCGTCTGCGCAGCGTGGCGGTATCCACGTTCAACACGAAGAAGAACCGCGCGCCATTCCGTCACTTGCTGCTCCACGGTCCGCCAGGTACCGGTAAGACGCTGTTCGCGAAGGCGCTCGCGCGTCACTCTGGTCTCGAATACGCTATCTTGACCGGTGGTGACGTCGCGCCGCTCGGTCGCGAGGGTGTGACGGAGATCCACAAGCTGTTCGACTGGGCGTCGCATTCTCGTCGGGGTCTCTTGCTCTTTGTCGACGAAGCCGACGCGTTCCTGCAGAAGCGTAGCAACACCGTGATGAGCGAAGACATGCGCAATGCGCTCAACGCGTTCCTTTACCGCACGGGTGAGGCGAGCGACAAGTTCATGATCGTGTTTGCGAGTAACCAACCGGAGCAGTTCGATTGGGCGATCAACGATCGTATTGATGAAATGGTCGAGTTCTGTCTGCCGGGCCACGCCGAGCGCGTGAAGATGCTCAAGCAGTACTTTGACAGCTACATCCGCAAGCCGAAGAACTCGTCGGCGAAGAAGATCTACGCGAGGGCGTGATCTTCACAGTCGACCCCGTCGCGAGCTTCCTCATCCCAAGACGCGCATGTTCCAGATCGAAGCGCTCAAGGACGTGTCGGCGGAAGCACAGACCATCTTTGACGCGCTCAACAAGAGCCCATGCGCCTCTACTGGATCATCTTTGGCGTCGTGCTCGCGAACGGCGTGTACACGTACTTCAGCGGCAAAGACGAGAAGTACCTGGTCGACAAGATCCAGAGAAACGTGCCATCGGAGTTCCGCATCTCGGAGAAGTGGGACAAGTGCATCGAGAACTTCACGCTGCACTTCGCCGCGGGCCTCGTCGCCGGCGGCCTCACCTCCGTCGTCCTCGCGCGCTCTGGTGCGGCTCGCGGTGCGCTGACGGGCTTCGGTGCGCCGGCGCCGGCTCCAGCTGGACGACGTGCCAGCTCGCGTTCAAGGGTGACAAGCAGGCGGTGCTGGACAAGTCGGACAAGATCGTGGACGAGATGGCTACCTACAAGACCCCCTCGCTCGTGAGCTACACGGCCGCCTCTCCTGCGCAGAAGCAGCTGCTCGCGTTCTACCAACAGCTTCTACGATGACGCCGCCATCAAGATGCTCGAGGCGTACCTCGCCGAGCAGATCGCGAACTCGACCGTGGACATTGACGCCAACCTGGCGCTCTTGAAGCTCTACCTCGTGTTTCCAGCGCACTTGGACGCCGACAGCCAAGGTGCTCGTCAAGGGCATCACGGTCACGCCATCGCCGTTCTTCACTGGCGTCGAGCATCGTGCCAGAAAGCGTTCGCGAGGATGCGAACGTGAAGCTCGCGCTGGAAGCCGGCTTCCTCCTGCAGTCGTGCTTGTTCGCGGAGTTCTGGAAGCTTGACCTGACGTTCGCCAGCGCCGTCACGGGCTTCTCGGATGCCGTGCGTGCGTTCATCCTCGACGCGCTCCGCAAGAGCCACAGCGTGATCTCCACGGCGGTGCTCAAGTCCGCACTCCACATCTCGGACAAAGAAGTGGCGGACGTGATCGCGGAGAAGTGGGCGAGGGCGACCTGGTCAAGGTCACGCCGAACGAAGACAACCAGGTGCGACCAAAGAAGATCCAGGAGAAGATCGAGTTCGAGGACGTGCTGAAGGTCATCGACACGCTCTCTAGATCGAACGTGTGTGCGAGCGGTGGCGGCGTCACGGACGCGCAGGAAGTGTGCGACGCGCTCGCCAAGCACCTGCGTCTCGAGAGCGGCAACATGGTGCTCCCGAGCTCCACGGGCGTCATCGGCTGGCGCATCCCTGTGGAGGCCATGATCGAGCAGATCCCGCAGCTCATCGAGAACCTGCAGTCAGAGTCCGTGCTGCCAGTTGCGGAAGGCATCATGACCACCGATTTGTACCCAAAGATCCGCTCGATTGACGTGTGCGGTGGCCGCATCACGGGTATCGCCAAGGGCGCCGGCATGATCGAGCCTGACATGGCGACGATGCTCTCGTACATCCTCACGGACCTGTCGGTGCCGCGCGACGTGCTGCGCCAGCTGCTGTCGGAGGTCGTGGGCGATACGTACAACGCCATGAGCATCGATACGGACCAGAGCACGTCAGACACGGTGTCGATCGTGTCGTCGGACCAGATCCCGTTCGACATGAG---CAAGCTCACCGAGTTCAAGCAGGCGCTGCAGGAGGTCTGCCAGGGGCTCTGCGAGGACATTGTGCGCAACGGCGAGGGCGCGCACCACGTCATGCGCGTGGTCGTGCGCGGCGCCGAGTCGGTCGAAATGGCCAAGGGCGTGGGCAAATC---------------------------------------------------------------------------------GTACATGGGCAAGCACCACAAGGACGTGAATATCACGCGCCGCATGAAGATCAGCGTGGGCGGCATCTCCATCTTCGAGAACGGCGAGTTCGTACTCAACAGCGACATCGAGCAGCTACTCGTGGATCACATGCGCCAGGCCCAGCTCATCGAACCAACGCGAGGCGGTCTGGACGTCACCTCGCACGACTACCCGCCGCACGGGCGCTTTGTTGAGATCGATATCGACCTGGGTGTTGGCACTGAGCGCGCGCGCGTGCTCGGCATCGATCTCACGCACGAGTACGTTGCGATCAACGCCGACTACCGATCCAA-------------------------CAGCCGCCGCGTACCTCGCGTCGCTGGGCGTAGACACGTC---TGCCGGCGGTGACAAGAAGGACAAC---------AAGAAGAAGAAGGGCAAGAAGAAGCCAGAAAGAAGCCCGTGTCCGCCGCCGCCAAGCTCCTGG-CGGAGCGACAAGCGGCTCTCCGCGCCGAGCAGGAGCGCATTGAACGCGAGCGCTTGGAGCAGGAGCGCATCGAGCGCGAGGAAGAGGAGCGCCGTCTCGCCGAGGAAGCGCTCCAGGAGGAGATCCGCCGCAAGAAGAAGGAGGCCAAGGCGGCCAAGGTGGAGCGTCAGAAGAAGGAAGGCACGTACATGACAAAGGCGCAGAAGCTCAAGGCTGCGAAGGCGCAGGCCGCGCTCGAGGCACTGCAGCAGGCTGGTCTTGTACCAGACGCGGCTCCGGAAGAGGAGAAGAAGAAGGTCGTGTACACGAACAACAAGAAGGGCAAGAAACAACCAACGACGTCA----AGTCGAGGCCGAAGCTCCAGACGCGTGGGATGACGAGGACTGGGAGACCAGCGACGTGGTCGCCTCACTCGAGGACAAGCTAGCAGAAGAAGGAGCAGGAGCGTCTCGTGCAGCAGGGCATCGCGCTGAAGAAGCGCCAGGAGGAGCAGGAGGAGCAGGCGCGCATCCACGCCGAGGAAGAGAAGATTCGCTCCAAGGCGCAGCAGGAGGCCGACCGCAAGAAGCGCGAGGCCGCAGACCGCCGTCGCGTGCGCGAGGAAGAGGCGCTCGCGAACGTGTCGCTCGACAACCTGCGCTCGCCGATCTGTTGTATCATGGGCCACGTCGATACCGGTAAGACCAAGCTGCTTGATAACATTCGTAAGACCAAGGTGCAAGACGGCGAAGCTGGCGGTATCACGCAGCAGATCGGTGCGACGTTCTTCCCAGTGGAGGCGATTAAGCAAAAAACCGAGCGTCTGAACAAGACCGTGAAGCTCGACTACAAGCTGCCGGGTCTGCTTGTGATCGATACTCCCGGTCACGAGTCGTTCACGAACCTGCGCTCGCGCGGTTCGTCGCTCTGTGATATCGCGATCCTGGTGGTCGATATCATGCACGGGCTAGAGCCGCAGACGCTTGAGTCGCTGCGCCTGCTGCGCCAGAAGAAGGCGCCATTTGTGGTCGCGCTCAACAAAATCGATCGCTGCTACGGCTGGAAGACCATGCCGGACATGCCGATCCAGGAGGCGCTCAAGCACCAGAACGAGCACGTAATCCGCGAGTTCGAGGACCGTACCGCGAAGATCATGGTCGAGTTCTCGGAGCAGGCGCTGAACGCCGAGATCTACTGGCGCAACAAGGAACTGGGCCGCACGATCTCGCTCATCCCGACGAGCGCCATCTCGGGCGAAGGTGTGCCGGACCTCTTGATGATGATCACGCGCCTCACCCAGGAGCGCATGGCCAAGTCGCTCGCGTTCGTCGACATCTTGCAGTGTACCGTGCTGGAAGTGAAGGTGATTGAAGGCTTGGGCACGACCATCGATACGATCTTGGTGAACGGCACGCTGCACGAAGGCGATACGATCGTCGTGTGTACGCTGGACGGTCCCGTGGTGACGACGATTCGATCCATTTTGACGCCGCATCCGATGAAGGAGATGCGCATCAAGGGCGACTACATCCACCACCAGACCATGAAGGCGGCGATGGGTGTCAAGATCTCGGCGCACGGGCTCGAGAAGGCAGTGGCTGGTACGCAGATCCACGTGGTCGGTCCTGACGACGATGTCGAGGAGCTCAAGGACTCCGTCATGTCCGACCTCACGGGCATCTTAGACAGTGTCAAGGCCACGAAGCGTGGTGTGATGGTGCAGGCATCGACGCTCGGAGCGCTCGAGGCGCTGCTGGAGTTCCTGCGCACATGTGACCCGCCGATCCCCGTGTCATGCGTGAACCTGGGACCGGTGCACAAGAAGGACGTGATGCGTGCGTCGGTGCAGCTCGAACACCAGAAGGAGTTCGCAACGATCTTGGCGTTCGACGTGAAGGTGCACAACGACGCGACCGAGCTGTCGCAAGATCTGGGCGTGCGCATCTTCACGGCCGACATTATTTACCACTTGTTTGACCAGTTCACGGCGTACATGGACAATTTCCGGGAAGCGCGTCGCGAGGAGTTTGAGGATGTCGCGGTGTTCCCGTGCGTGCTCAAGATCCTGCCGAACTGCATTTTCAACAAGAAGGACCCGATCATTCTCGGTGTGGATGTGGAAGAGGGTATCCTGAAGATCGGCACGCCGCTCGTGGTGCCGACGGCCGGCG---GTGTGTTTGTGGGCAAGGTCGGCTCGATCGAGCGCGAGCACAAGGAGGTGGACCGCGCGAAGAAGGGCGCATCGGTAGCCGTGCGCATCGACAACGAAGGCAGCATCACGTACGGCCGCCAGTTCGACCACAAGCACAAGCTCGTGTCGCGATTATCGCGTGCAAGCATCGACGCACTCAAGGAGAACTTCCGCAACGACCTGCAGAAGGAGGACTGGCAGCTCGTCATCCGTCTCAAGAAGGTGTTTGACATCATTATGGAGGGATGGCTCACCAAGCGCCGCGACCACATGAACCTCATCTGGCTCGAGCGCTATTTCGTGCTTGACGGGAACCAGATGCGGTACTACCGCAAGCGCGGGGACCCGGCACCGCGTGGTACATACGTGCTCACGGACGGCTGCCTCGTCAGCAACGTCTTCAACTCGGAGGAGAAGCACCGTCACCATGGTCCGGTGTGGATGTTCCGCATCACGTGAGAAGTCGTACAAAAAAGACCGGTTTCTGGACCTCGGCGCCAAGACCGAGGAGAATGCGCGTGAGTGGAAGAAGGCGATTGAGAACGCCGTGCGCTCGCTGCGGGAGCTTGTGCTGCACCAGCGCAAGATGAAGACTGACGCCGCGAT---CTACCCGACGCAGTACTCG---------GTGCCACGACTGCTCAAGGCGGACGAAGACACAGTCTCGCTGTTCAATCTGGACGCGGACAACGTCAAGGCCGAGCAAGGATGGTGGCTCGCACGCGTGGAAGAAGGACTGCGCATCATGCAGGAATGTCCGCTGGGACCGAACGTCGCGGCGTCGCTCTACGCGCAGCACGCGAACACGACGAACGGACTCATCTTCTCGTCGCTGATCAGCGCGATCCTCACGTTCAGCATCATGTACTCCTTT---------------------------------------------------------------------------------------ATCCCGTCGTTCCGCGTCTCGCAGGTCGTGAACGGCTCGCCCACGGAAGTGTTTCGGCTGCTCATGAATCCGAAGCGGTACCAGCGCTGGGATCCGTCAATCGCGACGATGAAAGTGATCCAGGAAATGGACGACCACGCCGACATTGTGTACATCACGCAGCGTCCGACGTATCTCTGGCCGCTCTGGCAGAAGCCGCGCGACCTCGTGCTCATGCGCTACTGGCGCCGCGAAGAAGACGGCTCGTACTTTGTCATGTACCAGAGCATCGAGCACCCAGAGTGCCGCGTGCGTCACAACTACGTGCGCGCGACACTGCTGGGCGGCGGGTTTGTGATCGCACCGCAGAAAGTGCCGAGC---GGCGCGATCCGCTCGCACGTCACGTACGTGCTCCGCTACGACCCGGGCGGTTG---GTCGAGAATCTACCACCAGCTCGGTATGGACGTGGACTTGGTGATGCCCATGCTGCGAAGTGTCGTGGGCATCCGCGACGAACTGAGCAGCGTCGACTTCATCACGCCGAACGTCACCGTCGCGGCAACTGGTGCAGAAGGAGGGCGCTGGCGGAGCGTCGA---CGCGTCAGGTGTCGTGGTCGGCGTCACGAAGCTCAAG---ACGAGTCTTCCGGAGAAGATGTGGTCGGAGCCCGATGCGTCGGTGTTCCAGGTGCGCGGCCCAAACTACCTGATCGACAAGAAGAAGATGCCGTCGGCCGAGTCCAAGTTCCACTTGGTGGGCGTCGACTTGCTCTCGTTCGAGAGCGCGAGCGACCGGTACCACGTGTCGTCTCGCAATGGTAGCATCGGCCGTACGTC---GACCAAGTTTACGTTTGTGATCAACATGATCATCCCCGGCGCGGAGAATGTGTGCATGGTGTTCTACTTCCAGCCGGCGAAGGACAACATCTTTGAAGACGGCTCGCCGTTCTCGGAGCTACTGAACGACTTCATCGACGGCGACGACCAGTTTCGCAACTCGCGCTTCAAGCTCATCCCGACGGTCGTGGAAGGAAGCTTCATCATCAAGCAGTCGGTGGGCTCCAAGCCCACACTGCTGGGCAACAAGCTCAAGTGTCCGTACCACAAGGGCGAGAACTACTTTGAAGTGGACATTGACATCTCGTCCAACTCGGTCGCAAACACGGTCGTGGGCATGGTGCAGGGCGTGACCAAGTCGCTCGTGGTCGACATGGCGTTCCTGCTCGAATCGCAATCGGAGGAAGAGCTCCCGGAGAACATTCTCGGGGCCGTACGCCTGCAGAACATATCACTCGACAAGCCCCTGCGCGTGCCCAAGTATGGGCAACGGGGAGCAAGGACGAGTACACGTTCTGACGCGTTCACCGAGGCACTCCGCGTGAACCTGCAGCGACTGATCGTGTACGCCAAGTCGGCGGACACGAGCCTCCAGCGCGAGGTCGCCGAAAAGCTTGCGAATGAGGCAGTCAAGACAGACAGGTACAGATCGTGGAACTCGAGGGACTCAAGCTCCTGCTGCCGCTGACGCAGTCCAAGGACACGGAGGTGCAGCGATTGGCGGCACACGCGCTCGCCAACCTTTCGGTCAACTCGGACAACCAGTCGCGCATGGCGACCGAGGGCGGCATCGACATGCTCATCGATCTGCTTGAGAGCACGAACGAGCACGTGCAGCGTCAGGCAGCGAAGGCGCTTGCGAACTTGGGCGTGAACGTTGATAACAAGGAGCGCATCGCGAAGGCTGGCGGCATCAAACCGCTCATCGCACTCGCGAGCTCCAAGCAGATCGGCGTGGCGGTCGAAGCCATTGCGGCACTGGCAAACCTGGCTGTCAACG------------------------------------------------------------------------------------------------------------------------------CTGAGAACAAGCAGCTGATCATCGATCTTGGGGGCGTGGACGCGCTGCAGGGGCTTGTGCGGTCGCCCAATGACCGGATATGCCAGCAAGCGACCCGCGCACTCGTGGCCGATGAAGAGTTATGCCGATCGACATCAGT---CAGCCGGTCACGTTCATGGCCGTGACGGCGATGGACCAAGACAGCGAGTACAG---CTCGTCGGGGATGGAAGTTAGCCCCGAGTTTGTGCGAAGCGAAGCGACAGCGTTGACTTGC------CACCGAGATGCTGTGACGCCCACGC-GGCTGGTACGGAAAGT--CGCGTTCTCGTCTATCGAAGAAGACTACCCGCTGTCTTTCACAAGCAACACCAGTGTGGCGCCGAGGATCACGCC------------GGTAATGAACC---CGATTCAAGAAGACTCGCACGAGGAGGAGGAC-------------ATGACTGTGTTGATTCGGTCCAACTCGGTCGAGAGAGAC---GCTGATGGAG---AT---CGCAAGAGCTCCCAAGCTCTACCAATACCGACCGACAAA--GACTGAAGAAGATGAGATGGAGATCCTTCGTTGGGACATTCAAGGGAACACGTTCAAGAAGTATTTCGCGCTGCAACAGCGAGAGCCGCAATGGGGGACTCTCGACGAGCGAGACAACGACTTTTCTTCCACCCTGGCAAACGACGAGAAGCCATCATGATTGAGAAGAAGTTCCATGATTCTACCTACCGAGAGGGAATTGCGGAGGGAGACGCGTCCTGGTACAAGACCAGAAGAGAGCAGTTCGCCGAAGTCCACCGCGAGTTCATGGAAGAAACGCGATACAAGCAAGAGCTTATGCTTACTCGACTTGAGAAGAATTCAACGTTTTCGAAATTGCCCATTTTGGCGAACAGATTCCTCCTTGTCCGCCTTCTCGGGAAAGGAGGCAACGGAGAAGTGTGGAATGTCATCGATTTCGCTGACAACCAGCGCTCGTGTGCTTTGAAGCTGTCGACCTCAGTGAAGCACGCTCACCAAGAGCACTTCACCCACATTGAAACATCCAC--ACATTGTGGGTGTTGGCGATACCGCGTATTTGATCGAGTATCGCCAGCAATTCTACACCGCTTTCACCGTTGACAGCGTGGAAACCGACTTGCAGCAGCTCATTGAGA---TCTACGGGTACCTGAACGAAGGTTCTGCTGTCAAAGTATTGTTTCAGCTTTTGCAATCGCTGTCGTATCTACACAATGGAGATTGCGCACTACGACTTGAAGCCGTCAAACATCTTGATCTCGAAGGATGAAAGCGTCAAGCTC------ACAGATTTTGATCTGGCTCGAGATGTGAACGAGCCAATCGCGTCTTCTTCGATCGGCACGTTGCGCTACCTGCCACCAGAGTGCTACCGCCCGAATTTTGGAGA---------CTGTCAAGGCACTGCCGAGAAAGCAGACATTTGGATGGTTGGCATCGTGTACTGCCTGATGATCTGGGGGAAACACCCGATCTGCAATGAAAAGGGCTCTCAGCAAGAGGCGAAAGAGAGCCTGGCGCGGTACAATGGA---CAGCTCACGTACTTCCGACCAATGTCCGATCTTTCCCGATGGATCGTTCAAGGGTGCTTGCACCCTGACCCAAGATGCCGTCCGACCGCTTCTCAGCTGCTCCAGGCGCTGCAG

Pythium_iwagamai CGGACTACCACTCAGAGGTGGCGCGCGAGCTCGCGGCCTTCTTCCACTCGTTCTACCGCTTTGGCGCGGCCTCGAACGTCTTCGACCTGTTCCTGCACAACGTGCTCTTCGGCTCGCTCGCGATCGGCGCGATCGTGTTCGTCGCGCTTGCGATCATCGTCGTGCGGCGCTGCTTCCTGCACATCCGCAGGCGTATGTGCGCAAGATCTCGATCGAGTCGAACGCGACCGAGTTCGCGGCCGTGCTGCTCATGACGTTCATCGCTGTGAGCGCGCTCAGCGGTCTCCTGGGCGAGGCGGCCTCGTGTCGAGTGAAGTGATCCGCTCGCGCGCCGAGAACCTCGTGGTGTCGTTCAAGGAGAGCGACCTGCCTGAAGAGGCCTTCAAGCTTTCCGTGGAAGCCATGGCCTGCTGCACTCGAGCAAGGATCTCGTGAACACGACCAACACGCTGCTGCCCAAGCATTACGGCGCCCTGGCGGACGAGTGGCAATTCAGCTACTTCATGCTCAAGTCGACGACGAACGCGGCGATCCTCTCGGTCGCGCTCTCGAGCTTCCTGCTCATCTCGTCCATCGGCTGGTCCATGGTCTCGCCACTGCGACTCGCGATCCTCGTCATCCTGAGCGTCGTTCCCGTGTCGCACACGCTCATCGGCGTCTATCTCTCGTCGACGATCATGACGTCGGACTTTTGCGCCGCGCCCATGAACGCGACCGTGACGCTCTTGCACCCGTGCCCAGCGAATACCTTAGTCCATTGATCGCGCCAGCTGTGGGTCTACGCGAAGACACATGGCGACGTGGGCCAGCGCATGAAAGCCGAGTTCCTCGACCCCATTGGTGCAGATCTGGGCGTCATGCTCGGCTACTTCATCGGCACCGTGTCCAACTGGGAGAGCTTCCAGCTCTTCGAGACATGGCGCTGGCCCTTCGTCGTCCAGTTCGTCGCCATGTGCCCTATCATGGTCGCCATTTTCTTCGTGCCAAAGAAGCACCTGATCATCCGCAGTGAAGCCACTCAACCTGCGCCACTGATCCGAGGCGCGCCCGTGAGCCGCAGCGGGTTCATCCACAAGGAGGAGGAGGACGTGCTGCTCGCGAGCGCAGCCATGCCACGTACGGCGCATTCGACACGAGGTGTACGAGCAGGGCTCGTTCTCGGACGGGCTCTACGAGCTCGCGCACATCCCGATCTTCTGCCTCATCGTCGGCGGCCTCACGGCCGTGTACTTTGTGGTCACGGGTGTGCAGTACTGGTCCACGATCTTCATGATCAAGTCGCTCCACGCGTCCAAGTACCTCGTGAACGGGCTCTTCGTGTTAGTCGCCGGCACGGGGCCCATCTTCGGCGTCTTCTACGGTGGCTGGCTCATCGACCACTACGGCGGCTACATCGGCGTCGAGCAGCGCGCCAAGTCGCTGGGCATCTGCATGGTGCTGGGCATCACGGCGTTCCTCATCTCGGCCGTCACGACGTTCTTCAACGACATCTACATCACGGCCGCGTTCCTCTGGTTGCTGCTCTTCTTCGGCGGCTCGATCCTGCCGGCGCACCGTGCGCTGGCGTCCTCGCTCTCGGTCATGGTCTTCAACCTCTTCGGCTACTCGCTCTCGCATGAGCGGACTCGAGATGGTCGTGGGCGCCAAGTGCTACGTGCCGGACGAGACGCACGTGTGGCTCGCGGCCGAGGTCGTGCGCGAGGAGAACTCGGGCGACGGCAAGCTTCGCAAGATCCACGCGCGCGTGGAGCTCCCGCCCAGAGTTCGAGGACCGCGTCGTGGACATGAACGACAAGCAGACCAAGGCGTTGCTGCCGTGCACCAACTCGACTCGCTCCCGTACCAGAACGAGAGCACGGGCGCGTACGGCATCGAGGACATGATTTCGCTCAACTACCTCCACGAGGCCGCGATCCTCTTCAACGTCAAGACGCGCTTCCTCCAGAACCTCCCGTACACGTACACGGGCGACATCTGCATCGCCGTCAACCCGTACCAGTGGCTCCCGGATCTCTACGCCGACGAGCAGCACCTACGGTACCTGAACCAGCCCAAGGAAGAGCTCCCGCCGCACGTGTACGCGACGTCCGTGGCCTCGTACGACAACATGCGCCGCACGGAGCGCAACCAATCGATCCTCGTGAGCGGTGAGTCGGGTGCCGGCAAGACGGAGACCACGAAGATCCTCATGAACCACTTGGCCACGATCGCCGGCGGCCTCAACAACTCGACGATCAAGAAGATCATTGAGGTCAACCCGCTGCTCGAGTCCTTTGGTAACGCCAAGACCGTGCGCAACGACAACAGTTCGCGCTTCGGCAAGTTCACGCAGCTTCAGTTCGACCGCGAAGGCACGCTCGTGGGCGCCAAGTGCCGCACGTACCTGCTCGAGAAGACGCGAGTGATCAACCATGAGTACCCAGAGCGCAACTACCACGTCTTCTACCAGTGGA---AGGCGAGATGTCGGCTGAGCTGATGCTGGACTCGTCCAAGACGTACACGTACACGGGCAGCAACAGCGCGCAGAAGATCGAGGGCATCTCGGACGCCAACCACTTTAACAAGACGCGCGTGGCGCTCGAGCTCATCGGCCTCACGCCGGACGCACAGAGACCGCTCTTTGAGGTCCTCGCGGGCGTGCTTCATCTCGGCGAAGTCTCGCTGCTCTCGAACCCTGCGAATGATGAGGAGTCGCGGATCACCGAGGCCGATGCCGGCGCGCTCGCGTGCGTGCAGCTGCTGGGCGTGACGTGGGAAGCGATCGAGAAGGCGCTGTGTTCGCGCACGATGCGCGCCGTGAACGACGTGTACAGCGTGCCGCTGCGCAAGGAGCAGGCCATGGACTGTCGTGACGCGCTCGCCAAGGCGATTTACTCGAACGTGTTTGACTGGCTCGTGGACTCGATCAACCGCTCGCTCGCGGACGACAACAACATGTTCAACCACGTCGGCGTGCTCGATATCTTTGGCTTCGAGCACTTCAAACACAACTCGTTTGAGCAATTCTGCATTAACTACGCGAATGAGAAGCTCCAGCAAAAGTTCACGCAGGACGTGTTCAAGACCGTGCAGATCGAATACGAAGAGGAAGGCATCACGTGGAACCACATTGAGTACGCGGACAACCAGGACGTGTTGACGGTGATCGAGTCCAAGATGGGCATCATTTCGCTCCTGAACGAAGAGCTCATGCGCCCCAAGGGCAACGAAGAGTCGTTTATGTCCAAGGTCGCATCGCTGCACAAGGAG---GACATGGCGCACGTGATTGAGTTCCCGCGCACGTCGCGCACCGAGTTCCTGATCAAGCACTACGCCGCGCCGGTCATGTACGACTCGGTGGGTTTCCTAGAAAAGCACAAAGACGCGCTTCTGCCGGACCTGTCTGAGCTCATGCGTAGCTCATCCAAACCGTTCATCGCGGCGCTCTTTGAGCCAAAGCCTGCGCCGAAGGCGACGAAAGCG---AGGTGGCGGCGGCGCACTGACGCTGTCGACGGTGGGCACGCAGTTCAAGGAGTCTCTCGCGGAGCTCATGACAACGATCAATGCCACGAAGGTCCACTACGTGCGCTGCATCAAGCCCAACTCGATCAA---------GAGCTCGACGACGATGGACCAGACCATGGTGGTCGCGCAACTGCGTTGTGCAGGTGTGATTGAGGCGATTCGCATCTCCCGCGCGGCGTACCCAAACCGCTTGCAGCACACGGATATCCTGGACAAGTTCTGGCTGTTTGTGCCTCGCCAGGGTGAGACGGCGCGCGAGAAGTGCGAGCTGCTCATGGAGAAGCTCAAGCTCTCGACCCCGGAGCAGTACCAGATGGGCAAGACCCGCGTGTACTTCCAGCTGGGAGTGCTTGAAGAGCTCGAGGACCGTCGTAAGAAGTTCCTGGACGCCAAGGCGACGTACGTCCAGGGCGTCATGAAGACGTTCACGCAGCGCATCAAGTACCTCCGACAGCTCGCTGCGATCATCAAGTTCTGTCATTCGCTGTGTGATTGCCATGCGCCGCTACAACGTGTTCATGAATGGACTCGTCAAGGCCCAGGCGCGCTGGAGAGGCATTGAGGCGCGCAAATACTACGTGGAGGTGCGCAGCAACCATCACGCTGTGATCATCCAGCGATACGTGCGCGGCTTCGTTCTGCGTCACCAGTACGTGAAGGTGCGCGCGAGCATCATCCGCGTGCAAGCCATGGCGCGCATGAAGATCCAGCGTCCCAAGTACCTAGCGGCGCTCCAGGAGGAGAAGCTGCAGGCGGACATGGTGTACCAGCTCAGTCAGCTCAAGGCTCGTCTCCAGGAAGAGCAGGACCGCAATGCGCGCCT------GCAGGAGCAGCGCAACTCGGTG-----GCGGCGGTGCCGAAGTGTCGAGCGTCGTGATCGCCGACGCTGGCGGCATGATTGAGAAGCTCCAGGAAGAGAACTCGAAGATCCGCTCGAAGAACGAGGACCTGAAGGCGACCGTGAAGGCGCTTAAGGCCGAAGTCGGAACCTCCAAGTCCGAGAAGGAGATGAGCCACGCCGGCCTGTACGTGAAGATCCGTCAGCTCGAGGACACTGTGCGCGAGAAGGAGAAGCGTGTTACTCAGCTTGAGACCGACAACCAGAAGCTCACGGACCAGGTGCAGGACCTGATCGCGAACGGCCACGTGCAGTCTGAGAAGAAGACGAAGGAGAAGCGCTCGATCTTCCGCACGCTCGGCTCAAAGAAGGAGAAGAAGCC------------------CGAGGCCGTGCTCCTCGACTCGATGACGGGCGAGGAGGACGTGAACGGCTCGGACGCGCGCGCGTCG---------CACTTCCCAAAGCTGCTGCCGACCATGAACATGTCGAAGGCCAAGTTCTGGGGCAAGGCTAAG---GGCGAGAAC------------------GACGGCGCCGACGACCGCGACTCGAGCGGCAACGACTCGCAGAACGGCCGCCGCGCAACACGATCGTCAAGAGCATGGAGGTGGGCGTGACGGGCGCGATGACGAACCTCAAGGGCAAGATG---------GCGGCCGTGAAGACCATGTACGAGGCAAGCGCTCGGGCAGGATCGCCCGATCCGCGAGTCGTTCAACCTGGATGCGCTCCCCGAGGTGTCGCTCCCCGTGGGCTGGGAGGCCAAGGTCTCGCGCTCCACGGGCCGCGTCTACTACGTGAACCGCAAGCTCGGCAAGTCGCAGTTCGAGCGCCCGACGCTCGCGTCGCTTAA------GGCGGCCAAACTCGCGCGCCAGAAGTCCGCCAGCATCACCGAAGGCTGTGGTTGCGGACTTTGGCTGCGGCGACGCGCGCCTGGCCGAGAGCGTGCCCAACAAGGTGCACTCGTTCGACCTCGTGTCGCTGGATATCGCGGTCTACTGCCTCGCGCTCATGGGCACGTCCGTGCGCGAGTTCTTGCTCGAGGCGCACCGCGTGCTCAAGCCCAACGGCGTGCTGCAGGTGGCTGAGGTCAAGAGCCGCTTTGAGACCGAGTCGCTCGGCGGATGGTGGCGTCGTTCCTCGCGGTCTCTGGCGACGTCGTCGTGCTTGTGCGCGACAAGACGCTCTTCGTGGCGAGCGCAAGTACGTGGTGATCGCGGGCGTGAAGACGGGCGAGGCCGTCGCGATGCCCTTCCCGGACATCAACCGCGACGTCAAGACGCTGCTGGGCCACACGACGTCGATGATCGTGCATCTGACGGTCAACCTCGTCGCTGCTGCTCACGGGCGACCGCGACGAGAAGATCCGCGTCTCGGGCTTCCCACGCACGTCGCTCATCCAGAGCTACTGCCTGGGACACAGCGAGAGCCTCACCAAGGTCGCGACGAGCTCTCTCACGCCCGACCTCGCGGTTTCGACGTCGCTGGACAACACAATCAAGCTCTGGAGCATCACCTCCGGCACGCTTGCGGTCTCGGCGCTCTCGATGTCGTTGCGGTCGTGCTCAACAACCAGTTGGTGCGTCTCTTTGCGATCGTGGCGCACCATTCGTGAGCTTCTTCAACGTCCACCAGGGATGACAGCGTGCTGGCAAAATCTGCGCGACTTCCGCAAGCTGCAGAATGACCCGCCCTCGGGCGTCTCCGGCGCGCCCATGGACAACAACATCATGCTCTGGCAGGCCGTCATTTTCGGTCCGGATGACACGCCGTGGGAGGGCGGGACTTTCAACCTGACGCTCGAGTTCAGCGAAGATTACCCGAACAAGGCACCGACCGTGCGATTTATCACAAAGATGTTCCACCCCAACATCTACAACGACGGCCAGATCTGTCTGGATATTCTGCAGAACCAGTGGTCGCCGATCTACGACATCTCGGCCATCCTGACGTCGATCCAGTCGCTGCTGTGCGACCCGAACCCGAACTCGCCCGCGAACTCGGAGGCCGCGCGCCTCTTCCAGGAGAACCGTCGCGAATACAACCGCCGCGTGCGGGAGATCGTCGAGCAGAGCTGGGTCGCGATCTATGGTGTCGGCCGCCAAGATGCGCGGCGCTGAGAACCGCATGCGCGCCGGTCGCCCGTTCACCGCCTGGTTGGACAATGTGTCCTCGGACCGTGGCCTCTGCGGTGGTATCAACTCGGGTATCGCCAAGGCCGCGCGCAAGCAGCCTCGGCCTCGCTCTTCGTGATCGGCGACAAGGGCCGCGCCCAGCTGCGTCGTGACTTTGGCGCGCAGATCCGCGGCAACGTGACCGAGACGTTCGCGACCCCACCAAACTTCACGGTGGCCTGCGCGGTCGCCGAGGCCGTCGAGAACGAGAGGAGAGCTCGCGCATGGCCGCCATGGAGAACGCCACGACGAACGCCCAGGACCTCACGCTCACGCTCGTGTACAACAAGGCCCGCCAGTCGCGCATTACGACCGAGTTGATCGAAATTATTTCCGGTGCCGCGTCGCTCGACCGTCACGGGCTCCGTGATCGACGTGCAGGACGCCGCGCAGTTCGACGAGCTCACGGCGCGCGAGTCGACGCTCAGCATATCGTTCTTCTGGGCGGACTTCCAGCTCTGCCGCCCGAACGGCCAGATCGACGTAGGGCAAGAGCGTGCTCGAGAAGCTTGAGGGCGCCAACGTCGCCGAGCTCGCCAAGCGCGTCTCGAGTACCGTCTCAAGAAGCTCATCAACGCGTCGCCAGTGATGCTTTTCATGAAGGGCAACCCGAGCGACCCAAAGTGTGGCTTCAGTCGCCAGGTCGTCGCACTCCTGAACGAGGAGAACATCCAGTACGGCACGTTTGACATCCTCAACGACGAAGAGGTGCGTCAGGGCCTGAAGCAGTTCTCCAACTGGCCGACGTTCCCGCAGCTCTACGTGAACGGAGCGCTCATCGGCGGCCTCGACATCCTCAAGGAGATGAAGTCTGAGGGCTCGATTGTCGAAGAGCTCGGCGTCGAAGATGGCGAAGCGGCGTTCAACGAGAGTCTTCGCGCGCTCGTGGGCTCGGCGCCCGTGCTGCTGTTCATGAAGGTTCGACATCCTGTGTGGAACTACGAGCGCCCGAAGCACGAGATCACCAAGCTGGCGACGTTCGCGCCGACGTCGTTCCGGAGAGAGATGCGCCGAGAGCCTGGTGTGGAGTCCGCAACCGCCTAGCCATGGCATTCCGTGACCCGATTAACGGTCAAGGCGAGTTTTGCGTTGTGTTGCTGCGCGCGTGACGTCCATGTCCACGCCGCTCCACTCGCGCGGCATCTCTGCGATCGAGTGGAAAGTGGAACGTGTTGCATCGCGAGCATCGGGCAGAAGTCAAATCTATCTGCGTGCACTCGCAGCGCCAGGTGATCTACACGGGCGGCTTTGACGGCCAGGTGATCCGTCACGACCTGCACAAGTCCATGTCGACCGTTGTGATGGAGCGCCGCAAGCGCATCTCGAAGATCAACGCCGTGCTCGAGCACCCGCACAACCCGCACATCCTCCTCGTGAGCAGCGTCGAACAGAGAGCACAGTATCCTGCTGCACGACCTTCAGCGGTACAACAGCAACCTGACCTCCAAGTCCATGAGCCAGTACATCGTTCCGCGGTGGTCGCCCGCGGGCTTGCACGTCTCGTGCGGCAGCAAATGCGCTACTGCCACGGGAGCTTCTCGACGCCGACGTCCGCGACCATCGGCGCATCGTTCCTGCAGAAGCGCATCATCGTAGGCGGGAAGATGACGCTCCAGATCTGGGACACGGCTGGCCAAGAGCGCTTCCGCTCCATGGCACCGATGTACTACCGGAACGCCAAGGCTGCGATCCTCGTGTTCGACCTGCTCAACGAGGCGACGTTCGAGAAGATCAAGGAGTGGCTGCATCTTCGACATCATGTGGGCGACGACATGTGGGCAACAAGTGCGACTGTTCAACTTTGCGAAAGCGCAGCAGTTTGCACAGGAGATCGGCGCGCTGGTGTTTCGCACAAGCGCGAAAAACGGCGACGGTGTGCAGAACCTGTTTGAGAGCGTCGCAGTGCAGCTCCTGAAGCGTCACGAGGAAGAGGAGAAGTCAGCTTCGTCGTCGTGCACGTGCCCGCGGACGACAACAAACAGCTGGAAGAGTGGTCTGTGGTGCTCCCGAAGGACAAGGAGGCGCAGCTCGGGTGCCTCACAGACCGCCTGCGCGCGCACTTCAAGCAAAGCGGCATCGACGGCCGAGCAGAAATCTTCAAGAAGCAGTTGATGAGCCAGCTGCCCAAGGGCCAGACCATGACCGACGACATGCTGCAGATGATGCATGGACAGCCTTGTGGACTCGTTCCCGCTCATGCTCAACTCGCGCGCTGTAAGCCACATTCGAAGTGCGCGGCGACGCGTTCATCGCGCGTGTGTTTGACAGACGACTTTGACCGTATGGACTTCCGCCTGAGCGAGGTCACGATGGACGCCGACTGGATCAAGATCGGCAAGGAGCAAATGTGACGCAGTCGCTCGAGGAGATCCTCGCGCTCGGCCAGGGGCTCGACGACGAGGACTACAAGGCCGAGCTCGACGTGCTGCAGAACGACCCGAGCTCCAACTTGTACTCGGCCGTGACCTTCGAGGAGCTCAACCTGCCGGAGCTACTGCTCAAGGGCGTGTACTCGATGAAGTTCTCCAAGCCATCCAAGATCCAGTCCGTCGCGCTCCCACTCATCCTCGCAGACCCGCCTGAGAACCTGATCGGCCAGGCGCAGAGCGGCTGCCGACGCGCGAGCTCGTGCGCCAAATCATGGCCGTCGTCAAGGCCATGGGCAAGTAGTCGACACGTTCCTCGCGATCCCGGGCGAGGATGTGGCCCGCGGCGAGCAGGTGCAGGCCCAGGTCGTTGTCGGTACGCCCGGACGCGTCGAGGGGCTCATCAAGAAGAAGCAACTGGACACGCGCCACATCAAGATCTTCGTGCTGGACGAGGCCGACGTGATGGTCTCAGAGGACGGCATGCGCGACCGGTCCGTGGCGATCAAGAAGATGATCAAGAACCGCAGCTGCCAGTACCTGCTCTTCTCGGCCACGTACGCCGACGAGGTGCGCGACTTTGCGCAGCGCATGGTGCCGGACCACAACATCATCACGGTCAAGAAGGAGAAGCTCACGCTCGACGGGATCAAGCAATTCTGGATCGACTGCGGCTCCAAGGATAACAAGTTCAAGGTGCTCTCGGATATCTTCGCCGTGCTCACTATCGGCAAGTGCGTCATCTTTGTGCAGTCGCGCGAGACCGCCAAGGAGCTCACAAACCGCATGCGCGCGAACGGCCACTCTGTCGGCATCCTCCACGGCGCCGACATGGCACGCGAGGTGCGCGACCAGGTCATCGACGAGTTCCGCGCGGGTACAACCAACGTGCTCATCACGACCAACGTGCTGGCGCGCGGCATCGACGTCGCCGGCGTCTCGCTCGTCGTCAACTTTGACATCCCGCTTAACCGCGACCACCAGCCGGACCCTGAAACGTACCTGCACCGCATTGGGCGCACGGGCCGCTTCGGCCGCAAGGGCTGCGCGATCAACTTTGTGCACGACGACCGCAGCAAGGGCCACCTGGCGGAGATCGAGGCATACTACGCACGCGGGATCGTCCAGGCGCCCGCGAACGACATTGAGGAGCTGGAAAAGATGCTCGCGTGGGGGGCAGCAAGCGCCGCCTGGCCAAGTTCGGCACGGAGCAGCGCAAGGGCATGCGCATCTACCCGCGCGAGCCCAAGAAGAAGCCCAAGGGCAAGGACAAGGCGTACTACTACCTCAAGGATGTCATGTACCTCGCGCACGAGCCGATCCTCGAAAAGTTCCGCGAGTTCAAGACGTTCATGAAGAAGATCAAGAAGGCCATGGGCAAGCGTGACTTTGAGGACGCCAAGCGCAAGCACGACAACCGCCCGACGTACGTGCTCGACCATATCGTGAAGGAGCGCTACCCGCGCTTCGTGGACGCGCTTGGCGACATGGACGACGCGCTCTGTGGAACAACTACGTGGCCACGTCGGGCTCGCTCACCAAGATCTTTGTGAGCATCAAGGGCATCTACTACCAGGCGAACGTGCAGGGCCAGACGATCACGTGGCTCGTGCCGCACCAGTTCACGCCGACGATGGACAAGCGCGTCGATGTGCGTGTGATGCTCACGTTCCTCGAGTTCTACGAGGTGCTGCTCAAGTTCGTCAACTACCAGCTCTACACGGAGTTGGGCATTGCGTACCCGCCGAAGATCGACCTGCAGCTCGACCAGGCCGGTGCCCAGCTCGCTGCCATGCAGCTAGCAGCAGAAGGAGTCGGAGAAGCGCATTAAGCTACAACGACCAGCGCAAGGAGGCGAGTGAGAAGAGCCTGTTCAGCGGTCTGTACTTCTACCTATCGCGTGAAGTGCCGTCGGCGTGCCTGGAGCTCGTGCTTCGCTCGCACGGCGCCACGATCGGCTGGGAAGGCGCCGGCTCCCCGTACACGGAGAAAAACGAGCGCATCACACATCACATCATGGACCGTCCGCACCAGGGCCACCGCTACTTCAACCGCGAGTACGTGCAGCCGCAGTGGGTCTTTGACAGTGTGAACAACGGCATGTTGCTCCCACTGGCGCGTTACCGCAAGCAGCTCAACAAGCTCAAGTCGGCCACTCAGGTGCTGCGCGAGGTCGGCGACATGGATGTCGACGACGAGGTGTGGAGGCCTACGCGGCTGGGCTCAAGGCCGAGCTCGACGGCAACAAGAAGTCGAAGAAGGTGAAGCGTGAGGAGGAGGCCGAGGAGCTCCACGAGCTGGCGAAGAGCATGATGTCGAAGAAGTCCAAGCGCTTATACGACCGCATGCAGTACGGCCTCGGCAAGAAGTCGGACAAAATCCAGGCGCTTGAGGACAAGAAGGCCAAGCTCGAGGCAAGACCAAGAAGCTCGTGGCCGGCGCGCTCTCGGGCATGATCTCAGCGCTGGCCTTCCAGCCACTGGGGCGATGCGCGGGCGCGGCACGTCGCCCACGCTAATCCGCGTTGCGGGCGGCGCCGGCCTTTACTTCACGACGCTCGACCACTGCATGACCATGTTTCCCAGCTCGGCGATCAACACGTTCCTCTCCGGCGCCTTCGCGCGCACCTTCGCTGGTTGCGTCATGTCGCCGTTCACGATCGTCAAGGCGCGCATGGAAGGCGCCGGGCACGTACAGCAGCAAAGGGACGTGCCCTTCTCGGGGCTCTACGTGCTCATCTACACGGCACCCAGCCGACGTGGTCAAGACGCGCATGCATGCGCCAGACGGTGCGCAAAATCCTGGGCGACGAGGGCTGGCGGGGCTTCAAGAGGGGCATCGTGCCCCGTGTCCTGAAGCGCACGCTCTCCACCGCTGTCACGTGGACCATCTACGAGCTACTCTTTCGACCCGTCGGGCCTCGGCCACGCCAAGGACGCGTTCCAGCTCGCCCGCGAGGGCGAGAAGACCAAGCAGCTCGAGCACACGGCCAAGGTGAAGGAGAACGAGGCGCTCTACAAGCAGTACGAGATCGTGCGTGTGCAGAAGGAGGGCGAGGAGCGCCGCAAGACCATCGAGGAGGAGACCCGCCAGCACCAGACCCGCGCGCAGTACCAGGACCAGCTCAAGCGCAAGCAGTACGCCGACCAGCTCGCGTACCAGAAGCAGATGAAGGAGCAGGAGCTCAAGAAGCAGGAGGAGATGCTCGCGCGCCAGGAAAGCTCGCGACGAAAGACCCTCGAGTACGAGGCCGAGCTGCGTCAGAAGACCGAGCTCTCCAAGGTCGCTGCGGAGGTCGAGGGCAAGATCAAGCAGGAGCGTCTCAACCACGACTTGCACCTTGAAGAAGCGCGTGTGCGCGCCAAGGAGTACCGTGAGACCGTCATGGAAGGCATCAAGCTCGCCGGTTCCACTATCGGCTCTGGTGTCATGGCGTTTGTGGACGACAAGGAGAAGCTCACGGCCACCGTTGCTTCGCTCACTGCTCTCGCGCTTGGTGTGTACACTGCGAAGACCGGTACGGGCGTCGCTGGCAAATACATTGAAGCGCGTCTCGGCAAGCCGTCGCTCGTGCGTGAGACTTCGCGCCGCACGGTCGCACAGGTTGTCGCGAACCCCGTGCCGTCGATCAAGCGCGTTCTGCGTCTTCAAAAGCCAACGGACGCCTTGGAGGGCGTCGTCCTTGAGCCAAAGCTCGATGAGCGCCTGCGCAGCGTTGCGGTATCCACGTTTAACACGAAGAAGAACCGCGCGCCGTTCCGCCACTTGCTGCTCCATGGCCCGCCAGGTACTGGTAAGACGCTGTTCGCAAAGGCTCTCGCGCGCCACTCGGGCCTCGAATACGCGATTCTCACCGGTGGTGACGTTGCGCCACTCGGCCGTGAGGGCGTGACGGAGATCCACAAGCTCTTCGACTGGGCGTCGCACAGCAGCCGGGGGCTCTTGCTCTTTGTCGACGAGGCCGACGCGTTCCTGCAGAAGCGTAGCAACACGGTGATGAGCGAGGACATGCGCAACGCGCTCAACGCGTTCCTGTACCGCACAGGCGAGGCGAGCGACAAGTTCATGATCGTGTTTGCGAGTAACCAGCCGGAGCAATTTGACTGGGCGATCAACGATCGCATTGACGAGATGGTCGAGTTCATGCTCCCAGGCCACGCCGAGCGCGTCAAGATGCTTAAGCAGTACTTCGACAACTACATCCGCGCGCCGAAGAACTCGTCGGCCAAGAAGATCTACACGAGGGCGTGGTCTTCACGCTCGACCCCGTCGCGAGCTTCCTCATCCCAAGACGCATATGTTCCAGATCGAGGCGCTCAAGGGCGTGACCACCGAGGCGCAGACCATCTTCGACGCGCTCAACAAGAGCCCATGCGGCTCTACTGGCTCCTCTTTGGCGTCGTGCTCGCGAACGGCGCATACACGTACTTCACCGGCAAAGACGAGGCGTACCTGGTCGAGAAGATCCAGAGAAGCGTGCCGTCCGAGTTCCGCATCTCGGAGAAGTGGGACAAGTGCATCGAGAACTTCACGCTGCACTTCACCGCCGGCCTCGTCGCCGGCGGCCTCACCTCCGTCGTCCTCGCGCGTACTGGAGCGGCGCGTGGCGTGCTCACAGGCTTTGGCGCGCCGGCGCTGGCTCGAGCTGGACGACGTGCCAGCTCGCGTTCAAGGGCGACAGGCAGGCCGTGCTCGACAAGTCGGACAAGATCGTGGACGAGATGGCTGCCTACAAGACCCCGACGCTCGTGAGCTACACGGCCGCGTCCGCTGCGCAGCAGCAGCTGCTCACGTTCTCCCAACAACTTCTACGACGCCGCGGCCGTCAAGGTGCTCGAGACGTACCTCGCCGAGCAGCTCGCGAACGCGACCATCGACATTGACGCCAATCTGGCGCTGCTCAAGCTCTACCTCGTGTTCCCGGAGCACCTGGACGCCGACAGCCAAGGTGCTCGTCAAGGGCATCCAGGCCACGCCGTCGCCGTTCTTCACCGGCGTCCACGATCGTGCCCGAGAGCGTGCGCGAAGACGCCAACGTCAAGCTCGCGCTGGAAGCCGGCTTCCTCCTGCAGTCGTGCCTCTTCGAGGACTTCTGGAAGCTCGACTTGTCGTACGCCAGCGCCGTCGCGGGCTTCACGGACGCCGTGCGCGCGTTCATTCTCGACGCGCTCCGCAAGAGCCACAGCGTGCTCTCGACCGCCGTGCTCAAGTCGGCGCTGCACATCTCGGACAAGGAGGTCGCGGACGTCATTGCCGAGAAATGGACGAAGGCGACCTCGTCAAGGTCACGCCGAACGAGGACAACCAGGTGCGACCGACCAAGATCCAGGAGAAGATCGAGTTCGAGGACGTGCTCAAGGTCATCGACACGCTCTCTAGATCCAACGTGTGCGCGAGCGGCGGTGGCGTCACGGACGCGCAGGAGGTGTGCGACGCGCTCGCCAAGCACCTGCGCCTGCCCAGCGGCAACCTCGTGCTGCCGAGCTCCACGGGCGTCATCGGCTGGCGCATCCCCGTCGAGGCCATGATCGAGCAGATCCCGCATCTGGTCGAGAACTTGCAGTCTGAGTCCGTGCTGCCGGCCGCCGAGGGCATCATGACCACCGATCTGTACCCGAAGATCCGCTCGATGGACGTTTGTGGCGGCCGCATCACTGGTATCGCCAAGGGCGCTGGCATGATCGAACCCGACATGGCGACGATGCTCTCGTACATTCTCACTGACCTGTCGGTACCGCGCGACGTGCTGCGCCAGCTGCTCACGGAGGTCGTGGGCGACACGTTCAACGCCATGAGTATCGATACGGACCAGAGCACGTCAGACACGGTCTCGATCGTCTCGTCCGACCAGATCCCATTCGACAAGGC---GCAACTCGGTGAGTTCAAGAAGGCGCTGCAGGAGGTCTGCCAAGGACTCTGCGAGGACATTGTGCGCAACGGCGAGGGCGCGCACCACGTCATGCGCGTGGTTGTGCGCGGCGCCGAGTCGGTCACGATGGCCAAAGGCGTGGGCAAGTCGGTCGTGAACTCGCCGCTGCTCAAGTGCGCGGTCGCCGGCAACGACCCGAACGTCGGTCGCCTCGTCATGGCCGTGGGCAAGTACATGGGCAAGCACCACAAGGACGTCGACATCACGCGCCGCATGAAGATCAGCGTCGGCGGCATCTCGATCTTCGAGAACGGCGAGTTCGTGCTCAACGGCGACATTGAGCAGCTGCTCGTGGACCACATGCGCCAGGCCCAGCTCACAGATACGACACGAGGCGGCCTGGACGTCACGTCGCACGATTACCCGCCGCACGGCAAGTTTGTTGAGATCGACATTGACTTGGGCATCGGCAACCAGCGTGCGCGCGTGCTCGGCATCGACCTCACACACGAGTACGTGGCCATCAACGCCGACTACCGCTCGTA------------------------------------------------------------------------------------------------------------------------------------------------------------------------------------------------------------------------------------------------------------------------------------------------------------------------------------------------------------------------------------------------------------------------------------------------------------------------------------------------------------------------------------------------------------------------------------------------------------------------------------------------------------------------------------------------------------------------------------------------------------------------------------------------------------------------------------------------------------------------------------------------------------------------------------------------------------------------------------------------------------------------------------------------------------------------------------------------------------------------------------------------------------------------------------------------------------------------------------------------------------------------------------------------------------------------------------------------------------------------------------------------------------------------------------------------------------------------------------ATGATCACGCGCCTGACGCAGGAGCGCATGGCCAAGAGTCTCGCGTTCGTCGACATTCTCCAGTGCACGGTGCTCGAGGTGAAGGTCATCGAGGGGCTCGGTACGACCATTGATACGATCTTGGTGAACGGCACGCTCCATGAAGGCGACACCATTGTTGTGTGTACACTGGATGGGCCCGTCGTGACGACGATCCGATCGATCCTGACGCCGCACCCGATGAAGGAGATCCGCATCAAGGGCGAGTACATCCACCACCAGACCATGAAGGCTGCCATGGGTGTTAAGATCTGCGCGCACGGGCTCGAGAAGGCTGTCGCTGGCACGCAGATCCACGTCGTCGGTCCGGATGACGACGTCGAGGAACTCAAGGACTCTGTCATGTCCGACCTCACGGGCATCTTGGACTCTGTCAAGGCCACGAAGCGTGGCGTGATGGTGCAGGCGTCGACCCTTGGCGCGCTCGAGGCGCTGCTTGAGTTCCTGCGCACGTGCGACCCGCCGATCCCCGTGTCGTGCGTGAACCTGGGCCCCGTGCACAAGAAGGACGTGATGCGCGCGTCCGTGCAACTTGAGCACCAGAAGGAGTTCGCGACGATCCTGGCGTTCGACGTCAAGGTCCACAACGACGCGACCGAGCTGTCGCAGGAGCTCGGCGTGCGCATTTTCACGGCCGACATTATCTACCACCTCTTCGACCAGTTCACGGCCTACATGGACAACTTCCGGGAAGCGCGGCGCGAGGAGTTCGAGGACGTGGCGGTCTTCCCGTGCGTGCTCAAGATCCTGCCCAACTGCATCTTCAATAAGAAGGACCCGATCATCCTGGGCGTGGATGTCGAGGAGGGCATCTTGAAGATCGGCACGCCGCTCGTGGTGCCCATGGCCGGCG---GCGTCTATATCGGCAAGGTCGGCTCGATCGAGCGCGAGCACAAGGAGGTGGAGCGCGCCAAGAAGGGCGCGTCCGTGGCCGTGCGCATCGACAACGAAGGCAGCATCACGTACGGCCGCCAGTTCGACCACAAGCACAAGCTCGTATCGCGGTTAACGCGCGCGAGCATCGACGCGCTCAAGGAGAACTTCCGCACGGACCTGCAGAAGGAGGACTGGCAGCTCGTGATCCGTCTCAAGAAGGTCTTCGACATCATTATGGAGGGCTGGCTCACGAAGCGGCGCGACCACATGAACCTCATCTGGCTGGAGCGCTACTTTGTGCTGGATGGGAACCAGATGCGGTACTACCGCAAGCGCGGAGACCCGGCGCCGCGCGGCACGTACGTGCTCACGGACGGCTGCCTCGTGAGCAGCGTCTTCAACTCGGAGGAGAAGCACAAGCACCACGGCCCCGTGTGGATGTTCCGCATCACGTGAGAAGTCGTACAAGAAGGACCGGTTCCTGGACCTGGGCGCCAAGACGGAGGAGAGCGCGCGCGACTGGAAGCGCGCGATCGAGAACGCCGTGCGCTCGCTGCGCGAGCTTGTGCTGCACCAGCGCAAGATCAAAACGGACGCCGCGACGAGCTACCCGACGCAGTACTCG---------GTGCCGCGCCTGCTCAAGGCCGACGAGGACACGGTCTCGCTCTTCAACCTGGACGCCGACAACGTCAAGGCCGAGCAGGGTTGGTGGCTCGCGCGCGTCGAGGAAGGGCTACGCATTATGCAGGAGTGCCCGCTTGGCCCGAACGTCGCGGCCTCGCTCTACGCGCAGCACGCCAACACGACGAACGGACTCATCTTCTCGTCGCTCGTGAGCGCCGTGCTCACGTTCAGCATCATGTACTCGTTCGTGCACAACATGCTGTTTTGCTTCACGACCGCGGCGGTTGCGGTGGCGGTCTGCTGCGTGGCCATTACGCCGTCGCTCGCGATCGAGATCCCGTCGTTTCGCGTCTCGCAGGTCGTCAATGGCTCGCCCACGGAGGTCTTCCGCCTGCTCATGAACCTTAAGCGGTTCCAGCGCTGGGACCCGTCAATCGCGACCATGCGCGTGGTGCAGCAGATGGACGACCACGCCGACATTGTGTACATTACGCAGCGGCCGACGTACCTCTGGCCGCTCTGGCAGAAGCCGCGCGACCTCGTGCTCATGCGCTACTGGCGCCGCGAGGAGGACGGCTCGTACTTCGTCATGTACCAGAGCATCGAGCACCCGGAGTGTCGCGTGCGCCACAACTACGTGCGCGCGACGCTGCTGGGCGGCGGCTTTGTGATCGCGCCGCAGAAAGTGCCGAGC---GGCGCCATCCGCTCACACGTGACCTACGTGCTGCGCTACGACCCAGGCGGCTG---GTCGCGCATCTACCACCAGCTGGGCATGGATGTGGACCTCGTCATGCCCATGCTGCGTAGCGTCGTGGGCATTCGCGATGAGCTCAGTAGCGCGGACTTCATCACGCCCAACGTGACTGTGGCCTCGGGCGACAGCGAGCGCAGAGGACCAGGAAGCGTGGA---CTCCTCAGGCGTCGTCGTGGGCGTCTCGCGCCTCAAG---ACGAGCTTGCCGGAGAAGATGTGGTCTGAGCCGGACGCGGCGGCCTTCATGTTGCGCGGGCCCAACTATCTCGTGGACAAGAAGAAGATCCCGTCGGCGCAGTCCAAGTTTCACCTCGTGGGCGTCGACTTGCTGTCCTTCGAGAGCGCGAGTGACCGGTACCACATCTCGGCGCGTAGCGACAGCATCGGGCGCACTGCCGGCGGCAAGTTCACGTTCGTGATCAACATGATCATCCCCGGCGCCGAGAACGTGTGCATGGTGTTCTACTTCCAGCCGGCGCGCGACAACGTGTTCGAGGACGGCTCGGCCTTCTCGGAACTGCTCAACGATTTTATCGACGGCGACGACGCGTTCCGCAACTCGCGCTTCAAGCTCATCCCGACGGTCGTCGAGGGCAGCTTCATCATCAAGCAGTCGGTCGGGTCCAAGCCGACGCTGCTGGGCAACAAGCTCAAGTGCCCGTACCACAAGGGCGAGAACTACTTTGAGGTGGACATTGACATCTCGTCCAATTCGGTGGCCAACACGGTCGTGGGCATGGTGCAGGGCGTGACCAAGTCGCTCGTGGTCGACATGGCCTTCCTGCTCGAGGCGCAGTCCGAGGAGGAGCTCCCGGAGGTCATCCTTGGCGCCGTGCGGCTGCAGAACATCTCGCTCGACAAGCCCAGGCGCGTGCCCAAGGGACGGCAGCAAGCAGCAAGGACGAGTTCACCTTCTGACGCGTTCACCGAGGCACTTCGCGTCAACCTACAGCGCCTGATCGTGTACGCCAAGTCGGCGGACACGAGTCTGCAGCGCGAGGTCGCCGAGAAGCTCGCGAACGAAGCCGTCAAGACCGCCAGGTGCAGATCGTGGAGCTCGAGGGGCTCAAGCTCTTGTTGCCGCTGACGCAGTCCAAGGACACGGAGTCGC---------------------------------------------------------GCATGGCGACTGAGGGCGGCATCGACATGCTCATTGACCTACTCGAGAGCACGAACGAGCACGTGCAGCGCCAGGCAGCCAAGGCGCTCGCCAACCTGGGCGTCAACGTCGACAACAAGGAGCGCATCGCAAAGGCCGGCGGCATCAAACCGCTCATCGCGCTCGCGAGCTCCAAGCAGATCGGCGTGGCGGTGGAAGCGATCGCGGCTCTCGCGAACTTGGCTGTCAACG---ATGCCAACGAGGTCGAGATCGCGCGCGAAGGCGGCCTCAAGCCCATAATCGAGGGCGCGCGCTCAGAATCCGTCGAGCTCCAGTCCCAAGTGGCGCGAGCGCTTCGCAACCTCTCTG---------------------------------------------------------GGCTCGTGCGGTCCCCGAACGACCGGATATGCCAGCAAGCGACGCGCGCACTCGTTGCAGACGACGAGGTCTTCCGGTCGACGACGAT---CAGCCG---GCCTTCTCGGCCAT------------TCGAGACAACTTGTACCA---CGACTCGTCGATGGACGTCACGCACGAGTTTGAGTCTGGCGCGGTGACGGCGTCGACTTGC------CACGGTG---------CCAACACGC-AGATGGTGCCGAAAGT--CGAGTTCTTGTCCATCGCTGAGGAGTACCCGCTTCACAGTACGAGCCACGCCAGCTTGGCGTCGCGAGTCACGCCAGTGCGCGA---CCAGCTCAGCC---CCATTCAAGAAGGCGAAA---AGACAAAAGTC-------------ATGACTACGCTAAATCAGTTGAACTCGATGGAGGACGAC---TTCGACGAGG---AC---CGCAAGAGTCCCCAAACTTTACCAGTATAGGGCGACAAA--GACCGAGGAAGATGACATGGAGCAACTCCGATGGGAGATCCAAGAGAGTGTGTTTCGCAGATACTTCGCATTGCAGCAGCGAGAGCCACAATGGGGGACCCTAGAGGAACGAGACTCCGAGTTTTCTTCCATCCTGGCAAACGGAGAGATGCCATCATGACCGAGATGAAGTTCCATGATGCTACTTATCGAGA---------------------------------------------------------------------------CGAGGAAACGAAGCACAAACAGGAGATATCGCTGACGCGGCTTGAGAAAAATTCGACGTTCTCGAAGTTCCCGATTCTCGGAAACAGGTTCTTGCTTGTCCGCCTTCTCGGCAAAGGGGGCAACGGTGAAGTCTGGAGTGTCCTCGATTACGCCGACAACAAGCGTCCGTGTGCTTTGAAATTGTCCACGTCTGTGAAGCACGCACAACAAGAGCATCTCACCCACACTGAAACACACGA--ATATCGTGGATGTTGGCGACACCGCGTATCTGATCGAGTACCGCCAGCAGTACTACACCGCGTTCACAGTTGACAGCGTCGAAACCGACCTGCAGCAGCTAATCGAGG---AATTTGAGCAGTTCGACGAGTCGTCGGCTCTCCGAGTCCTGTTTCAGCTGCTGAAGTCGCTGTCGTATCTGCACGATGGAGATTGCGCACTACGACCTGAAGCCATCCAACATCTTGATCACCAAGGATGAGCTCGTTAAGCTG------ACAGATTTCGATCTCGCTCGAGACGTGCGCACACCAATCTCGTCTTCTTCGGTTGGCACACTGCGCTACCTGCCGCCCGAGTGTTACCGCCCCAATTTCGGCGA---------CTGCCAAGGAACTGCGGAGAAAGCCGACATCTGGATGGTTGGCATCGTGTTCTGCATCTTGCTGTGGGGGAAGCATCCTATCTGCAGCGAGAAAAGCACGATTGAAGAGGCCAAGGCGAGCATTGCACGATTCAACGGG---CAGCTCACGTACTTAAAACCAGTGTCCGATCTTTCGCGATGGATCATCCAAGGATGCCTGCACCCGGATCCGAGATGTCGACCAGCGGCTTCCCAGCTTCTTCAGGCGCTGCAG

Pythium_oligandrum CCGACTACCACAGGGAAGTCGCCCGCGAGCTCGTGGAGTTTTTCCACACGTTCTACCGCTTTGGCAGCGCCACCAACACCTTCAACTTGTTCCTGCACAACCTGCTCTTTGGCTCGCTCACCATCGGCGCCCTCGTCTTTATTATTCTATGCATCATCGTCGTGCGTCGCACGATCCTGCATATCCGTAGGCCTATGCGCGACGATGGGCGATGGAGTCCAACTCGTTGGAGATCTCGGCCGTGATCTTGCTCACTGTGTTGGCAATCAGCTCGTTGAGCGGATTGCTGGGCGAGGCTGCGCCAACGCCAGCGCCGACGTGCGCTTCCGAGCTGACAGTATGATTGTGTCATTCAATGAAAGCGACCTGCCACAGCAGGCGTCCAAGCTCTCTTTGGAATCGCTAGCATGCTACATGCCGCGCGCGACCTAGCCAACGTTACGGACGCACTGCTCCCAAAGAACTATGCCGATGTGGCCAAGGAGTGGCAGTTTAGCTACTTCATGCTCAAGTCGACCACCAACTTCGCGATCCTTTCCGTGGCCTTGGCGAGCTTCCTGTCCATCTCGTCCATTGGATGGTCGATGCTGACGCCATTGCGGGTCTCCATCTTGATCATCTTGAGTGTGGTGCCAATCTCGCACACATTGATTGGTGTCTACCTCTCGTCGACCATCATGACGGCCGACTTCTGTGCTGCTCCGATGAACAGTACTGTCACTCTGCTTGACAGGTGCCAGCCTAATGTGACAATCCGTTCAAGTCTTACGTGAGCAGACTACGCCAAATCCCATGGAGATATCGGAAGCCGCATGAAGAATGATTTCCTCGACCCTATTGGCGCAGATCTGGGTGTCATGCTCGGTTATTTCGTTGGTACCGTCGCGAATTGGGAAAGTTTCCGGTGGTTCCAGACGTGGCGGTGGCCATTTCTCGTGCAATTCATCGCAATGTGCCCGATTATGATCGCGATTTTCTTTGTGCCAAAGAAGCATCTCATCATCCGCAGTGAGACCAACGAATTGGCTCCGCAGCTTGGTGCGAATCCTACGTTTAGCTGGAGTGGACCGAATCACGAAGAAGAGGACGTCCTGTTGGCGAGTGCATCCATGCAGGGTACGGTGCTTTCGATGTGACGAATACGAAGAAGGATCGTTCACAGAGGGTATCCGAACACTACTGCAAATCCCGATCTTCTGCTTGATCGTCGGTGGTCTCACGGCTGTGTACTTTGTCGTGACGGGTGTCCAGTACTGGAGTACTATCTTCATGATCAAGTCACTCCATGCCTCCAAGTACATTGTCAACGCTCTTTTTGTGTTTGTCGCAGGCACCGGACCAATCTTTGGTGTCTTTTTCGGTGGATGGCTGATTGACCGCTGCGGAGGCTACATTGGTATCGAGCAACGTGCAAAGGCCCTCGGTATCTGTATGGTATTGGGTATCATCGCTTTCATCGTCTCGGCTGCCACAACTTTCTTCGATGACATCTACATCACAGCTGCTTTCTTGTGGCTCCTGTTGTTCTTTGGTGGATCGATTTTGCCAGCGCACCGTGCGGTCGCTTCTTCGTTCTCTGTCATGATCTTCAACCTTATCGGCTACGCATTGTCTCATGAGTGACCTAGAGATGGTGCCCGGGGTCAAGTGCTACGTGCCGGACGAGACCCACGTCTGGCTCGAGGCCGAGGTGCTCAAGGAAGAGCGCACGGCCGATGGCAAGGGACGTCTCGTCCACACCAAGGTGACACTGCCCGATGGAGCAAGAAGAGCGCGTGGTGGATCTCTACTCCAAGAAGGTCAAGTCGCTGCTGCGGTGCATCAGCTCGAGTCGCTGCCGTACCAGAACGAGAACCTCGGCGTCACCGGTATGGAAGATATGATCTCGCTCAACTACCTGCACGAAGCTGCCATTCTTTACAACGTCAAGCAGCGTTTCCAGCAGAAGCTCCCATACACGTACACGGGTGACATTTGTATCGCCGTGAACCCGTACCAGTGGCTGCCAGAGCTCTACGCCGAAGACCAGCACCTCAAGTACCTCAACTTTCCCAAGGAAGAGCTCCCACCGCACGTCTACGCGACGTCGGTGGCCTCGTACGACAACATGCGTCGCACGGAGCGCAACCAGTCGATCCTGGTGAGTGGTGAGTCTGGTGCCGGTAAGACCGAGACGACCAAGATTCTCATGAACCACTTGGCTACCATCGCTGGTGGTCTCAACAACGTCACCATTAAGAAGATTATTGAAGTCAGTCCGTTGCTGGAATCCTTCGGTAACGCCAAGACCGTGCGTAACGATAACAGTTCGCGTTTCGGTAAGTTCACATTGCTCCAGTTCGACAACCAGGGTACCTTGGTCGGTGCCAAGTGTCGTACCTACTTGCTCGAGAAGACCCGTGTGATCTCGCACGAAGCGCCAGAGCGTAACTACCACATCTTCTACCAGTGGAGTCGGGAGACCTCGCCAAGGAAATGTTCCTCGAGCCTGGTGTCATCTACAAGTACACTGGTGACAACAAGGAGCAGAAGATTGAAGGTATGTCGGACGCCAAGCACTTTGAGAAGACTCGCCACTCGCTCAGCCTGATCGGTCTCGCTGATGAGGACCAGCGTCCTTTGTACGAGGTGCTTGCTGGTATCCTCCACCTTGGTGAAGTCAACTTCCTCTCGAACCCAAGCAACGATGAAGAGTCTCTCATTGCTGAAGGTGATGTTGGTGCCAAGGCCGTGACGCAGTTGTTCGGTGTGACGTGGGAAGCCCTTGAGAAGGCCCTGTGTTCGCGTATGATGCGTGCTGTCAACGATGTCTACCAGGTGCCTTTGAAGAAGGAACAGGCGATGGATTGTCGTGATGCGTTGTCCAAGGCCATCTACTCGAATGTGTTCGACTGGCTTGTGAAGAGCATCAACACGTCGTTGGCGGATGACAAGAACATGCACAACCACGTTGGTGTGTTGGATATCTTCGGTTTTGAACACTTCAAGCACAACTCGTTCGAGCAGTTCTGTATCAACTACGCCAACGAGAAGCTTCAGCAGAAGTTCACGCAGGATGTGTTCAAGACCGTGCAGATTGAGTACGAAGAGGAAGGTATCACGTGGAGCCACATTGAGTACGCTGATAACCAGGATGTGCTGACGGTGATTGAGTCCAAGATGGGTATCATCTCGCTCCTCAACGAAGAGTTGATGCGTCCAAAGGGTAACGAAGAGTCGTTCATGTCCAAGGTTGCCTCGCTCCACAAGGAT---GATATGTCGCATGTCATTGAGTTCCCTCGTACTTCGCGAACCCACTTTACGATCAAGCACTACGCGGCGCCAGTCACCTACGAATCGGTTGGTTTCCTCGAGAAGCACAAGGACTCGTTACTGCCTGACTTGTCCGAGCTGATGCGTGGATCGTCGAAGCCATTCATCTCGGCTTTGTTTGAGCCTAAGCCTGAGCCAAAGTGCGCGCAAGC------GTGGTGGTGGTGCCCTCACGTTGTCTACTGTGGGTACCCAGTTCAAGGAAAGTCTCGCTGAGCTCATGACCACGATTCAGGCGACGAAGGTGCACTATGTGCGATGCATCAAGCCCAACCCAATCAA---------GAGTTCAACCGCGATGGATCAGAACATGGTCGTTTCGCAGTTGCGATGTGCTGGTGTGATTGAAGCCATTCGTATCGCTCGTGCCGCCTACCCGAACCGACTCCAGCACTCTGAGATTTTGGACAAGTTCTGGCATTTTGTACCAAGCTCGGGCGACAATCCGCGTGACAAGTGTCAAATCCTCATGGAGAAGCTCCGTCTCACCACCCCTGAACAGTACCAGATGGGTAAGACGCGTGTCTACTTCCAGCTTGGCGTTCTCGAGGAGTTGGAGGACCGTCGTAAGAAGTTCTTGGATGCCAAGGCGGTGCGACTCCAGGCGATTATGAAGTGCTTCATGGCCCGGATTCAGTATTTGCGCAAGATCAACGCTATTGTCAAGTTCGGTCATCCGTTGCGTCATGGCTATGCGTCGATACAACACATTCAAGAAGGGTCTCATCTTGGCTCAGGCTCAGTGGCGCGGTGTTCAGGGCCGCAAGCTGGCTGTCGAAGTCAAGCGCAACCACAATGCTGTCATCATCCAGCGATACACCCGTGGCTACCTTCAGCGTATTAAGTACGTCAAGATCCACTGGATGGTTGTGCGCGTGCAGGCTATTGTGCGTATGAAGATCCAGCGTCCAAAGTACTTGGCCGAGCTCCGTGAGCAGAAGCTGCAAGCCGATATGGCCTACCAGCTTCAGCAGCTCAAGGACCGTCTTCGCGAAGAACAGGATCGCAATGCTCGCCT------TCAGGAGGAGCGAGAAACCGCTCCTCGACGGGATCGTCGCTGCCTCGAATGTCGTCATGGCCGACGCTGGTGGTATGATTGAGAAGCTGCAGGAAGAGAACACCAAGTTGCGCTCGAAGTACGATGAGATCAAGGCCACGTTGTCGCAGGTCAAGGCCGAGAACGAGAAGTTCAAGAGCCAGAGCGAGATGTCGTCGGCGGGTTTCCACGTCAAGGTTCGCCAGTTGGAGGACTCAGTTCGCGACAAGGAGAAGCGTATCCAGCAGTTGGAGGCGGAGAACGCCAAGCTTACGGACCGTGTGAAGGATCTCTCTGTCAACGGCCCTCCACCAGCCGAGAAGAAGCCAAAGGAGAAGCGTGCTTTCTTCCGTACCCTCGGTGCCAAGAAGGAGACCAAGA------------------AGGACGCCGTGCTCCTCGACTCGCTTACGGGAGAAGAGGATTTG-------------CATGCCGACCACA---ACACCACCCGTACGATGCGT---CTCCCATCGAT-----------TCGTTTCTGGGCTGGTAGCAAG---ACGACCCGT---------TCGGACG------------ACGGTCAGGATGAGGATGGCTCGAACGGCACGATGA--ACACGCGTCAGAGCATTGTCAACACTGTTGGTGTCGGCGTCAGCGGCGCCATGTCCAACCTCAAGGGCCGCATC---------GTTGCTGTCAAGGGCGTCTACGAAGTGTCAGCTCGAAGGTGACCGTCCAATCCGTGAGTCGTTCAACCTGGACGCGTTGCCAGAGGTGTCGTTGCCACCAGGATGGGAAGCCAAGGTTTCGCGTTCGACCGGCCGTGTGTACTATGTGAACCGCAAGCTCGGCAAGTCGCAGTTCGAGCGCCCAACGATCGCGTCGCTCAA------GGCGCAGAAGATGGCGCGTCAGAAGGCGGCTCCAGCTGTCAAAGGCTATCGTCGCCGACTTTGGGTGTGGTGACGCTCGACTGGCCGAGAGCGTCAAGAACAAAGTGCACTCGTTTGATCTCGTCTCTCTCGACATTGCCGTGTATTGTCTTGCGCTGATGGGTACCAGCATCCGTGAATATGTGCTCGAGGTCTACCGAGTGCTTAAGCCTAAGGGCATCCTCAAGGTCGCAGAAGTCAAGAGTCGCTTTGAGACAGAGGGCCTTGGTGGATGACGAATGTGGTGCTCACGGCCGCGCGCGACGCGTTGGCGCTGATCCGACACACGACGCTGCTGCTGAAGAGACCAAGCATGTTGTGATCGTCGGCGAGAAGACTGGCGAGGCTGTCGCGATGCCGTTCCCACACCTGAACCGCGATCTGAGGACACTGTTGGGCCACACAACGTCGATGATCACACACATGGCGGTGAACCCGCTCGCTGCTTCTTACGGCCGACCGAGATGAAAAGATTCGTATCTCGCATTTCCCACGCACGTCGCTTGTCCAGAGCTACTGCCTGGGCCACAAGGCCTCCGTTACCCGCATCGCCACCAGTGCCGTCACGCCTCAACTTGTTGTCTCGACCTCGCTGGATAACTCCATCAAGCTCTGGAATGTCGAGGATGGTACCCTTGCTGTCTGTCCAGTGCGAACCTGGCGCTTGTTGTGCTTAACAACTCGGAACTGCGCTTCTTCGCGATCGAGGATCCCCGTTTGTGCGGCTCTTTGACGTCTCAAGTGGCCCATGATGAGCTTACAAGCAGTGCGGGACTTCCGCAAGCTCCAGAACGACCCGCCGTCGGGCGTCTCGGGTGCACCCATGGACAACAACATTATGCTCTGGCAGGCCGTCATCTTTGGTCCGGATGATACGCCATGGGAGGGTGGTACGTTCAACCTGACCCTCGAGTTCAGCGAAGACTACCCGAACAAGGCGCCGACGGTCAAGTTCATCACCAAGATGTTCCATCCAAACATCTACAATGATGGCCAGATTTGTCTCGATATCTTGCAGAACCAGTGGTCGCCGATCTACGACATCTCGGCCATTCTCACGTCCATCCAGTCGCTCCTGTGCGATCCCAACCCCAACTCGCCTGCAAATTCCGAAGCCGCGCGTCTCTTTCAGGAGAACCGCCGCGAATACAACCGCCGCGTGCGTGAAATTGTCGAACAGAGCTGGGTGTCGATCTATGGTGTCCGCCGCCAAGATGCGTGGTGCCGAGTTGCGTATGGTCGCCGGCCGCCCTTTTGCGGGCTGGTTGGACAACATCTCGTCGGACCGTGGTCTCTGTGGTGGTATCAACTCGGGTATTGCCAAGACTACCCGCAAGCAGACAAGCTCCAGCTTTTCATTCTCGGTGACAAGGGTCGTGCCCAGCTTCGTCGTGATTTCGCCCCACAGATCCGTGGTAGCGCCACCGAAGCCTACGTGAACCCACCAAACTTCGCCGTCGCCAGTGCCATCGCCGAGGCCATCGACAACGAGAAGAGAGTTCGCGTATGTCGGCCATGGAGAACGCCACGAGCAACGCGCAGGACTTGAGCTTGACGTTGCAGTACAACAAGGCTCGTCAGGCCCGTATTACGACCGAGTTGATTGAAATTATTTCGGGTGCCTCGTCGTTGGATGACGCCCGGCACGGTCGTGGCCATCCAGGACGCCGCCCAGTTCGACGAGTTGACGGCGCGCGAGTCGACGCTGTCCATCTCGTTCTTCTGGGCCGAGTTCCAGCTCTGTCGTCCGAATGGACAGATGGACGTGGGGCAAGAGCGTGCTCGAAAAGCTCGAGGGCGCGAACGTCGCCGAACTCGCCAAGCGTGTCTCGAGTACCGCCTCAAGAAGCTCATCAATGCGTCGCCAGTGATGCTCTTCATGAAGGGCAGTCCAAACGAGCCAAAGTGCGGCTTCAGTCGCCAGGTCGTGGCGTTGCTCAACGAAGAGAACATCCAGTACGGCACCTTTGACATCCTCAACGACAACGACGTGCGTCAGGGTCTCAAGGAGTTCTCCAACTGGCCAACGTTCCCACAGCTCTACGTCAACGGCTCGCTCATCGGCGGTCTCGATATCCTCAAGGAGATGAAGACGGAAGGCTCGCTTGTGGAGCAGCTCGGCGTGGAAGAGGCGGAGAAGGCCTTCCAGGAGAGTCTGCGCGCGTTGATCAACTCGGCGCCAGTGCTTCTCTTCATGAAGGTTCGACATCCTCTGTGGAAGTACACGCGAAACAACCACGAGGTGTCCAAGGTTGTAAGCTTTGCTCCGACATCTTTCCGAAAGGAAGTGTCCTGAAAGCATTGCTTGGAGTCCGTGACCGACTCGCGATTGCATTCCGGGACCCGGTCGATTCATTGAGTGAATTTTGCGTTGCGAGACTACTCGTCTCAAAGCCAAATCAACAACTCTTCACACCCGAGGTATCTCGGCCATTGAATGGAAAATGGCGCGTACTGCACCGAGAGCATCGTAGTGATGTGCGGTCTATCTGTATGCATTCCCAGCGAAATTCGATTTACACGGGTGGATTGGATGGCCTTGTTATACGGTACGATATGAATTCGTTCCGAACACACACCGTCATGGAACGCCGGCGCCACATATCCAAGATCAACGCTGTATTGGAGAACCCACACAATCCAAATCTCCTCCTGATCAGCAGTGTTGAGCAGGGAACACAATCTTCTTCTGCACGATCTGCAGCGCTATGATGAGAAGCTGACTTCGAAATCGATGAGCCAGTATATTGTGCCGAGATGGTCGCCTGGCGGCTTCCACGTCTCATGTGGCAGCACATGCGCTACTGCCACGGCAGCTTCTCCACGCCCACGTCCGCCACCATCGGCGCCTCGTTCCTGCAGAAGCGCATCGTCGTCGG--GCAAGATGACGCTGCAGATCTGGGACACGGCCGGGCAGGAGCGCTTCCGCTCCATGGCGCCCATGTACTATCGGAATGCGAAAGCGGCCATCTTGGTCTATGATCTGGGCAGTGAAGCGTCGTTCGACAAGATCAAGGAATGGCTACACTTGCGGCATCACGTGGGCGATGATATGTGGGGAACAAATGCGACTGTTCAACTTCGCAGTGGCACAACAATTCGCACAGGAAATCGGCGCGATGGTCTTCCGGACGAGCGCCAAGACGGGAGACGGCGTCGCGAATCTGTTCGAGAGCGTCGGCGAGCAGCTGCTCAAGAAGCACGACGAAGAAGAGAAGCGAGCTTTTACGTGGTCTACGTGCCGACGGACGAGGCCAAGGAGCTCGAGGAATGGACGATCGCCTTGCCCGACGACAAGGAAGAGCAGATCGGCTGTCTCACCGACCGGCTGCGGCAGCATTTCAAGGAATCGACGAAGCACCAAGGAACAGAGATCTTTAAGAAGCAATTGCTGAGCCAACTGCCGCCTGGCACGCAGATGAACGACGCGATGCTTCAGATGATGCATGGACTCGCTCGTGGATTCGATTCCGCTCGTGCTCAACACCAAGGCAGTGCAGCACGTTCGAAGTCAAGGGCGACGCCTTCATCGCTCGTGTGTTTGACAGACGACTTTGTGCGTCTTGACTTCCGTCTGGGTGAAGTGACCATGGACGCCGAGTGGATCCAGCTCGCCAAGACCCAGAGGTGACGCAGTCGCTCGAAGAGCTCCTCGCGCTGGGCCAGGGCCTCGACGATGAAGACTACAAGGCCGAGCTCGAGGTCTTGCAGAACGACGTCAACTCGAACCTTTTCTCGGCCGTGACGTTCGAGGAGCTCGGACTCCCAGAATCGCTGCTCAAGGGCGTCTACGCCATGAAGTTTTCCAAGCCGTCCAAGATCCAGTCGGTGGCGTTGCCCATGATCCTCGCCAACCCGCCAGAGAACCTCATTGGTCAGGCGCAGAGTGGTTTCCGACGCGTGAGCTGGTCCGTCAGATCGCAGCGGTTGTCAACGCCATGGGCAAGTTACTGAGTTGTACTTGGCGATTCCAGGTAACGATCTTGCCAAGGGAGAGCAGGTCACGGCGCAGATCGTCGTGGGTACGCCTGGTAAGGTCGAGGGTTTGATCAAGAAGAAGCTTTTGGACACCCGTGATATCAAGATCTTCGTGCTTGACGAAGCTGATGTCATGGTTGCTGAAGATGGTATGCGTGACCGCTCGGTGGCGATCAAGAAGATGATCAAGAACCGTGCGTGCCAGTTCCTCTTGTTCTCGGCCACATACGCGGATGACGTCCGTGAATTTGCCATGAAGATGGTGCCAAACCACAATATTATCACCGTCAAGAAGGAACGTTTGACCCTTGATGGTATCAAGCAGTTCTGGATCGACTGTGGCACCAAGGACAACAAGTTCAAGGTGTTGTCGGACATTTTCGCGATTTTGACCATTGGCAAGTGCGTCATCTTCGTGCAGTCGCGAGACACGTGTAAGGAGCTGACCAAGCGCATGCGTGAGAACGGCCACTCGGTCGGTATCCTCCACGGTGCCGATATGGCCAAGGAAGTCCGTGATCAGGTCATTGACGAATTCCGTGCCGGTACGACGAACGTCTTGATCACGACCAACGTGTTGGCTCGTGGTATCGATGTTGCCGGTGTCTCGCTCGTTGTCAACTTCGACATTCCTGTTACCCGTGACCACCAGCCTGACCCTGAGACGTACTTGCACCGTATTGGTCGTACCGGTCGTTTCGGTCGCAAGGGTTGTGCCATCAACTTCGTGCATGACCAGAAGAGCAAAGAGAACTTGGCCATGATCGAAGCGCACTACCAGCGCCAGATCGTCCAGGCGCCAGCTGACGACATTGAAGAGCTCGAGAAGATGCTCCAGTGGAGGGCAGCACGCGTCGGCGCGCCAAGCTCGGCAAGGAGCAGCGCAAGGGCATGAGCATTTACCCGCGCGACCCAAAGAAGAAGGTCAAGGGCAAGGACAAGACCTACTACTACATCAAGGATGTCATGTACATGGCGCACGAGCCCGTCTTGGAAAAGTTCCGCGAGTTCAAGACGTTCATGAAGAAGGTCAAGAAGGCCATGGGCAAGAAGCACTACGAGGACGCCAAGCGGCTGCACGAGAACCGTCCCGAGTACTCGCTCGACCACATTGTCAAGGAGCGATACCCGCGCTTCACGGACGCGCTCAGTGACTTGGATGACGCCTTGTGTGGCAGAACTACGTGGTCGCGAGCCGCTCGCTGACCAAGGTCTTTGTGAGTATCAAGGGCATTTACTACCAGGCCAACGTGCACGGCCAGAACTTGACGTGGCTCGTGCCGCACCAGTTCACGCCAACGGCCGATGAGCGTGTCGACTTCCGTGTCATGCTCACGTTCCTCGAGTTCTACCAGATCCTGCTCAAGTTTGTCAACTTCCAGCTCTACACGGAGATTGGCATCGCATACCCACCAAAGATCAACTTGCAGTTGGACGAGGCCGGTGTGCAGTTGACTGCGTTGGAGGTAGCAGCAGAAGGAGTCGGAGAAGCGTATCAAGGTACAACGAGCAGCGCAAGGAAGCGAGTGAGAAGGGTCTCTTCAGTGGCTTGTACTTCTTCCTTTCGCGTGAAGTGCCAGCTGCGCCTTTGGAGCTCGTGTTGCGCTCGCATGGCGCTCAGATCGGCTGGGAAGGCGAGGCTTCCCCATACAACGAGAGCAGCGACCGTATCACACATCACGTCATGGATCGACCACAGCAGGGTCACCGCTACTTCAACCGCGAGTACATTCAGCCACAGTGGGTCTTTGACAGCGTGAACAACGGTGTGATCCTCCCAGTGGCCCGATACCGCAAGCAGCTCAACAAGCTCAAGTCGGCGGCCCAAGTCATCCGCGAGGTCGGCGCCGAGGACGTGGACGCCGAGTCCTAGAATCCTACGCCGCTGGCCTCAAGGCCGAGCGTGACGGTAACAAGAAGTCCAAGAAGGCGAAGAAGCAGGAGGAAGACGAGGAGTTGCACGAGATGGCCGTCAACATGATGTCCAAGAAGGCTCGTCGTCTGTATGACCGTATGCAGTACGGTCTCGGCAAGAAGTCGGACAAGATCCAGGCGCTGGAGGCCAAGAAGGCGCAGCTGGACTCAAGTCCAAGAAGCTGGCGGCTGGCGCCCTCTCAGGCATGGTCTCGGCGCTGGCCTTCCAGCCGCTGGGGCGGTGCGCTGGAGAGGCACGTCATCAACGCTCATCCGTGTCTCTGGTGGTGCTGGTCTCTACTTCATGACACTCGATCATTGTCTGGGTATTGCCCCCAACACGGCGCTCAACACGTTCCTTTCGGGTGCGTTCGCGCGAACGTTTGCTGGATGTGTCATGTCCCCCTTCACGATCGTTAAGGCGCGGATGGAAAGCACCGGGTACTTACAAGAGCAAATGGATGTTCCATTTTCTGGACTTTATGTGCTCATCTACAAGGCATCCAGCCGATGTAGTCAAGACACGAATGCATGCGAGATACTGTCCGGAAAATCTGGATTGAAGAAGGCTCAGCCGGTTTCGCTAAGGGCATTGCCCCACGTGTACTCAAGCGCACGCTCTCCACAGCAGTCACGTGGACGATCTACGAACTACTCCTTCGACCCCTCGGGCTTGGGGCATGCCAAGGACGCGTTCATGATCGCCAAGGAGACGGAACGCACCAAGCAAATGGAGCAGCAGGCCAAGATCAAGGAGAACGAGGCGCTCTACAAGCAGTACGAGATCGTGCGCGTGCAGAAGGAAGGCGAGGAGCGCCGCAAGACGCTCGAGGAGGAGACGCGACAGAACCAGCAGCGTGCGCAGTACCAGGACCAGCTCAAGCGCAAGCAGTATGCCGACCAGTTGGCCGCGCAAAAGTACATGAAGGAACAGGAACTCAAGAAGCAGGAGGAGATGATTGCGCGTCAGGAGGCCTCGCGTCGTAAGACGCTCGAGTACGAGGCCGAGCTCCGTCAGAAGACCGAGATGGCCAAGGTCGCGGCGGAGGTCGAAGGCCGCATCAAGCAGGAGCGTCTCAACCACGATCTCCACTTGGAGGAAGCGCGTGTGCGTGCCAAGGAGTACCGTGAGACGGTCATGGACGGTATCAAGTTGGCGGGTTCGACCGTTGGTGCTGGTGTCATGACCTTTGTCGGTGACAAGGAAAAGCTCACGGCTACGGTTGCCTCGCTCACGGCGTTGGCGCTTGGTATCTACACGGCCAAGGTCTCGACGGGTGTTGCCGGCAAGTACATCGAGTCGCGTCTCGGCAAGCCATCGTTGGTGCGTGAGACCTCGCGTCGCTCGGTTGCCCAGGTCGTTGCCAACCCAATCCCATCGGTCAAGCGTTTGCTTCGTCTTCAGAAGCCACAGGACGCCTTGGAGGGTGTGGTCTTGGCGCCAAAGCTCGACGAGCGCTTGCGTAGCGTGGCCGTCTCGACCTTCAACACGAAGAAGAACCGTGCGCCATTCCGTCACTTGTTGCTCCACGGTCCTCCTGGTACCGGTAAGACGCTCTTCGCCAAGGCCCTGGCGCGTCACTCGGGCTTGGAGTACGCCATCTTGACCGGTGGTGACGTTGCTCCTCTCGGCCGTGAAGGTGTGACGGAGATCCACAAGCTCTTTGACTGGGCCTCGCACAGCCGTCGCGGTCTCTTGCTCTTCGTCGATGAAGCCGATGCTTTCTTGCAGAAGCGTAGCAACACCGTCATGAGTGAAGACATGCGTAACGCGCTCAACGCCTTCTTGTACCGTACCGGTGAGGCCAGTGACAAGTTCATGATCGTGTTCGCCAGTAACCAGCCAGAACAGTTTGACTGGGCCATCAACGACCGTATTGATGAGATGGTCGAGTTCATCTTGCCAGGCCAGGCCGAGCGTGTGCGTATGCTCAAGCAGTACTTCAACAGCTACCTCGTGAACCCGTCGAACAAGGCTTCCAAGAAGATCTACGGGAGGGCGTCATCTTCACGCTCGACCCCATCGCCAACTTCCTCATCCCAAGACGCACATCTTCCAGATGGACGCGCTCAAGAACGTGTCGGCGGAGGCGCAGGCGATCTTTGACGCCCTCAACAAGAGGCCATGCGTTTCTATTGGGTGATTTTCACTGTCGTCCTGGCGAACGGCGCATATACATACTTTACAGGAAAAGATGAATCGTACCTCGTGGAAAAGGTGCAGAGCGAGATCCCGTCCGAGTACCGCATCTCGGAGAAGTGGGACAAGTGCATTGAGAACTTTGCGCTCCACTTCTCGGCCGGGCTCGTCGCCGGTGGCCTCACGTCCATCGTCTTGGCCCGTACCGGTGGTGCGCGTGGTGTCTTGACGGGTTTCGGTGTGCCGGTGCCGGCTCGAGCTGGACAACGTGCCAATTGGCGTTCAAGGGCGACATCCAAGCCAAGTTGGACAAGGCCGAAAAGGTCGTGGAAGATATGGTCGCGTTCAAGCCCCAGACGATCGTGGACTACACGACACCGTCGGCCACGCAGAAGACGCTGCTCGCCGCCTTTCAACGCCGCTTACGACCAGAGCGTCGTCCAGACGCTCGAGCAGTACGTGGACGAGCAGATCCAGACGGCCACCGTCGACGTCGACGCCAACCTCGCGCTGCTCAAGCTCTACTTGGTCTACCCAGAGGCTGCGAATGTCGACACAGAAGGTGCTGCTCAAGGCCATCACGGCCTTGCCATCGTCGTTCTTCCTCGGTGGCGGCGATCGTCCCAGAAAGCTTCCGAGAGGATGCGACCGTGAAGCAGATCTTGGACGCTGGTTTCCTCTTGCACGCGTGCCGCTTTGAGGAGTTCTGGCAGCTCGACCTCGCATTCGCGGGCGCCGTCGCCGACTTTGCCGACGCCGTGCGTGCGTACGTCTTGACTGTCGTGGAGAAGACGCACAGCAGCATCTCCTCGGCCGTGCTCCAGGCCAAGCTCAACGTGTCGGAC------GTCAAGGACATTGTGGCCAAGAAGTGGACGAGGGCGACGTCGTGCAGGTGCCCGCCAACGACGACAACCAGATGCGACCAAAGAAGATCCGCGAGAACATTGAGTTTGACGATGTGCTCAAGGCCATCCACACCTTGTCTAGATCGAACGTCTGTGCGAGCGGTGGAGGCGTCACGGACGCCCAGGAGGTCTGTGACGCGCTGGCCAAGCACCTGCGTCTCGAGTCGGGCAACGCTGTGCTGCCGAGCTCCACTGGTGTCATTGGCTGGCGCATTCCCGTCGACTCAATGATCCAGGCATTGCCCAAGCTCGTGGAGAACCTGCAGTCCGAGTCGGTCTTGCCTGTCGCCGAGGGTATCATGACCACAGATTTGTACCCAAAGATCCGATCCGCCGACGTCTGTGGTGGTCGTATCGTCGGCATTGCTAAGGGCGCGGGCATGATCGAGCCAGACATGGCCACGATGCTCTCGTACATCCTCACAGACCTCTCGGTGCCGCGCGAGACGTTGCGTCAGCTGCTTCGTGAGGTTGTCGCCGACACGTTCAACGCCATGAGCATCGACACGGATCAGAGCACGTCGGACACCGTCGCGATCGTCTCGTCCGACCAGATCCCATTCAACGAAGCAGAGCACTTGCCAGCGTTCAAGCAGGCGCTCCACGAAGTCTGCCAGGGCCTCTGTGAGGACATTGTCCGCAACGGCGAAGGCGCTCAGCACGTGATGCGCGTGGTCGTGCGAGGCGCTGCCTCCACGGAAATGGCCAAGGGCGTCGGCAAGTCGGTCGTGAACTCGCCACTGCTCAAGTGCGCCGTCGCTGGTAACGATCCAAATGTTGGTCGTCTCGTTATGGCCGTCGGCAAGTACATGGGCAAGCACCACAAGGACATCAACATCACGCGTCGCATGAGTATCACGATGGGCGGCGTGCGCATCTTTGAGAATGGCGAGTTCGTGCTCAACCACGAGATCGAACACTTGCTCGTGGACCACATGTACCGTGCCCAGCTCACTGAATCGACCCGAGGCGGTCTGGACGTCACGTCGCGAGACTACCCACCACACAGCAACTTTGTCGAGATTGACATTGATCTCGGCATCGGCAACGAGCAAGCGCGCGTCCTCGGCATCGACTTGACGCACGAGTACGTCGCCATCAACGCCGACTACCGATCCTAATGCCGCC---GAAGAAGGGAAAGACCGCCGCGGCGTATCTCGCCTCGCTTGGTCTGGATCCATC---GGCCGGTGGCGCCGAGAAGAAGGAT---AACAAGAAGAAGAAGAAGGGCAAGAAGAAGGGCGAAAGAAGCCACTGTCTGCCGCTGCCAAGCTCGTGC-TCGAGCGTCAGGCTGCGCTCAAGGCCGAGCAGGATCGCTTGGAACAGGAGCGGTTAGAGCGCGAGAAGCAGGAGCGTGAGGAGGAGGAGCTCCGTCTTGCTGAGCTCGAACGTCAGGAGGAGGCCAAGCGTAAGAAGAAAGAGGCCAAGGCCGCCAAGGTCGAACGCCAGAAGAAGGAAGGCACGTACATGACCAAGGCGCAAAAGCAGAAGGCTGCTCGTGCTCAGGCTGCGCTTGAAGCGATGCAGCAGGCGGGTCTCGTCGCCCCACCAACG------GAGGAAAAGAAGAAGGTGGTGTACTCGAACAACAACCGTAAGAAGAACCAGAAGAGGAAGACAAGGAGGAAGAAGAGGAAGCTCCTGACTCATGGGATGCCGAAGACTGGGAGAGCAGCGATATCGTGGCCTCGTTGGAGGAGAAGCTAGCAGAAGAAGGAACAGGAGCGTTTGCGCCAGCAGGGTATTGCTTTCCAGAAGCGTCAGCAGGAGCTCGAAGAGTTGGCCAAGCAGGAGGCTGAAGAGGAGCGTATTCGTTCGAAGGCCCAGCAGGAAGCTGACCGCAAGAAGCAGGAAGCTGCCGAGCGCCGTCGCTTGCGTGAAGAAGCCGCCTTGGCTAATGTCTCTACTGACAACCTCCGTTCGCCAATTTGTTGTATCATGGGCCACGTCGATACCGGTAAGACCAAGTTGCTCGATAACATTCGTAAGACCAAGGTCCAGGACGGCGAAGCTGGTGGTATTACCCAGCAGATTGGTGCCACGTTCTTCCCTGTTGAGGCTATCAAGCAGAAGACTGCTCGCCTCGCTAAGACGATGGACTTGGACTACAAGCTGCCTGGTTTGCTGGTTATCGATACTCCTGGTCACGAGTCGTTCACCAACTTGCGTACGCGTGGTTCATCCCTCTGTGATATTGCCATTTTGGTGGTCGATATCATGCACGGTCTCGAGCCTCAGACTCTCGAGTCGCTTCGTTTGTTGCGTCAGAAAAAGGCTCCATTCGTCGTTGCTCTTAACAAGATTGATCGTTGTTACGGCTGGAAGACGATGCCAGACATGCCAATCCAGGAGGCGCTCAAGCACCAGAACGAACACGTCATTCGTGAATTCGAGGACCGTGCCAAGCAGATCATGGTGCAGTTCGCCGAACAGAAGCTCAACGCCGAGATCTACTGGCGCAACAAGGACCTCGGTCGCACGGTCTCGCTCATCCCAACGAGTGCTATCTCGGGTGAAGGTGTGCCAGATCTCCTCATGATGCTCACTCGTCTAACGCAGGAGCGTATGGCCAAGTCGTTGGCTTTCGTTGACATCTTGCAGTGTACTGTGCTTGAAGTCAAGGTGATTGAGGGTCTTGGTACCACCATCGATACCGTGTTGGTGAATGGTACATTGAATGAAGGTGACAAGATCGTGGTGTGTACATTAGACGGTCCGGTCGTCACAACCATTCGATCTCTGCTCACACCACATCCAATGCGTGAGATTCGTGTCAAGGGGGAGTACGTGCATCACCAAACGATCAAGGCCGCCATGGGTGTCAAGGTTTGTGCTCACGGCCTCGAAAAGGCTGTCGCTGGTACCCAGATCTACGTCATTGGTCCAGATGATGACGAAGAAGAACTCAAGGATGCCGTCATGTCCGATCTTACGGGCATTCTCGACTCGGTAAAGGCCACGAAACGTGGTGTCATGGTCCAGGCGTCGACCCTTGGTGCCCTCGAGGCGTTGTTGGAGTTCCTTCGCACCTGTGACCCTCCAATTCCTGTGGCCTGTGTCAACATTGGTCCAGTGCACAAGAAGGATGTGATGCGTGCTTCTGTGCAACTCGAGCACCAGAAGGAGTTTGCGACGATCTTGGCGTTCGATGTCAAGGTGCACAACGATGCCCAGGAGCTTTCGCAGGAACTCGGCGTGCGTATCTTCACGGCCGATATCATTTACCACTTGTTCGATCATTTCACAGCGTACATGGAGAACTTCCGACAAGCGCGTCGTGATGAAGTTGCCGACATTGCCGTCTTCCCGTGTGTGCTCAAGATCCTGCCTAACTGCATTTTCAACAAGAAGGACCCGATCATTCTCGGTGTGGACGTGGAAGAAGGTATTCTCAAGATTGGTACTCCTTTGGTGGTACCATCAGCAGGCG---GTATCCTGGTTGGTAAGGTCGGTTCGATCGAACGTGAGCACAAGGAAGTGGATCGTGCCAAGAAGGGTGCCTCGGTCGCTGTGCGTATCGATAACGAAAGCAGCGTCATGTACGGTCGTCACTTCGACCACAAGAACAAGCTCGTGTCGCGCCTCACTCGTCAGAGCATTGACGCGCTGAAGGAGAACTTCCGAGAAGACTTGCAGAAGGAGGACTGGCAGTTGGTCATCAAGCTCAAGAAGGTGTTTGATATCATTATGGAAGGATGGCTCACCAAGCGCCGTGACCACATGAACATCATCTGGTTGGAGCGCTACTTCGTGCTCGATGGCAACCAGCTGCGTTATTACCGCAAGCGCGGCGACCCGGCGCCACGCGGGACGTACGTGCTCACGGACGGCTGCGTCGTGAGCAGCGTGTTCAATTCCGAGGAGAAGCACCGCCACCACGGGCCCGTGTGGATGTTCCGCATCACGTGAAAAGTCGTACAAGAAAGACCGGTTCCTGGACCTCGGCGCCAAGACGGAGGAGAGCGCCAAGGACTGGAAGCGTGCGATCGAGAACGCCGTGCGCTCGATTCGCGAGCTCGTGCTGCACCAGCGTAAACTCAAGGACGACGCCGGTCT---TTTACCGACGCAGAGAGAG---------CCGCCACGTCTGCTCAAGGCCGACGAAGACACGGTGAGTCTCTTCAATCTCGACGCCGACAACGTCAAGGCCGAGCAGAGTTGGTGGCTCGCCCGCGTCGAAGAAGGTCTGCGCGTGATGCAGGAGTGTCCGCTCGGTCACCAGGTCGCGGCGTCGTTGTATGCCCAGCATGCGCAGACCACGAACGGGTTGCTTTTCTCTTCGGTCATCAGCGCCGTGCTCTCGTTCAGCATCATGTATTCCTTCGTCAACAACATGCTCGCGTGCTTTTTGACCGCGGGTATCGCGGTCGGTGTGTGCTGCATCGCCATTACGCCGACCAACAGCATCGAAATCCCTTCGCTTCGTGTCTCGCAGGTCGTGCAGGGCTCGCCGACCGAAGTCTTCCGCTTGCTCATGAACCCAAAGCGCTACCAGCGCTGGGACCCGTCGATTGCGACCATGAAGGTCATTCAGGAGATGGACGACCACGCCGACATTGTGTACATCACGCAGCGTCCTGTGTACTTGTGGCCATTGTGGCAGAAGCCGCGTGACCTCGTGCTCATGCGATACTGGCGTCGCGAGGAAGACGGCTCGTACTTTGTCATGTACCAGAGCGTGGAGCACCCTGAGTGCCGTGTGCGTCACAACTACGTGCGTGCCACGATCCTCGGTGGTGGCTTTGTCATTGCGCCTCAGAAGATCCCAAGT---GGTTCGATGCGTTCGCTTGTGACCTACGTGCTCCGTTACGACCCAGGAGGATG---GTCGCGTATCTACCACCAGCTCGGTATGGACGTCGACCTCGCGATGCCTATGCTTCGCAGTGTGATTGGTATCCGTGATGAGATGAGTGGCTCGCACTTTATCACGCCTATGGTGACGGTGGCGGGTGCGACTAGCA-ACGCCGGTACTTCGACACGGTGGA---CGACTCCGGTTTGGTCGTCGGCGTGCAGCGCTACAAG---ACGAGTCTCCCAGAGAAGATGTGGGCGGAACCTGATGGATCGCTCTTCCAGGTCCGCGGTCCAAACTACCTCACGGATAAGAAGAAGGCGGCATCGGCCGCGTCCAAGTTCCACCTCGTGGGTGTCGACTTCCTTTCGTTCGAGAACCCAAATGACCGCTACCACGTCTCGTCCCGTGCCGACAGCATCGCCAAGACGTC---GGGCAAGTTCACTTTTGTCGTCAACATGATCATCCCAGGCCCAGAGAACACGACGCTCGTGTTCTACTACCACCCAGTCAAGGACAACATCTTCGACGACGGCTCGCCTTTCTCGGAACTGTTCAACGACTTCATTGACGGTGATGACCAGTTCCGTAACTCGCGCTTCAAGCTCCTGCCAACCGTTGTCGAAGGCAGCTTCATCATCAAGCAGGCTGTGGGTTCCAAGCCAACGCTGCTCGGTAACAAGCTCCAGTGTCCATATTATAAGGGCGACACGTACTTTGAAGTGGCCATCGACATTTCGTCGAACTCGGTCGCCAACACGGTCGTGGGCATGGTCCAGGGTGTGACCAAGTCGCTCGTGGTCGACATGGCCTTCTTGTTGGAAGCCCAGAGTGAGGAAGAGTTGCCTGAGGTGATTCTCGGCACTGTGCGTCTGCAGCACGTGTCGCTCGACAAGCCTCGTCGCGTGCCAAAGAATGGGCAACGGGAAGCAAGGACGAGTTCACGTTCGGATACCTTCACCGAGGCGCTGCGCGTCAACCTCCAGCGATTGATTGTGTACGCCAAGTCGGCGGATCCAACGCTGCAGCGCGAAGTGGCTGAGAAGCTCGCTAACGAAGCCGTCAAGATCGTCAGGTGCAAATCGTGGAGCTCGATGGACTTCAACTGCTGCTGCCGCTGACGCAGTCCAAAGACACGGAGGTGCAGCGTTTGGCGGCGCATGCGCTCGCGAACCTGTCCGTCAACTCCGACAACCAGACCAAGATGGCGACGGAGGGCGGCGTGGACATGCTCATTGATCTCTTGGAGAGCAACAACGAGCACGTGCAGCGTCAAGCAGCCAAGGCGCTCGCCAACCTCGGCGTAAATGTGGACAATAAGGAGCGCATTGCCAAGGCCGGCGGCATCAAACCGCTCATTCGTCTGGCAAGCTCCAAGCATATCGGCGTCGCAGTCGAAGCCATCGCAGCACTCGCCAACCTCGCTGTCAATG---ATGCCAACGAGGTCGAGATCGCACGCGAGGGCGGACTCAAGCCCATCATCGACAGTGCTCGCTCGGAGTCGGTGGAGCTGCAGTCACAGGTGGCCCGTGCACTGCGCAATCTGTCCGTTAACCCGGAGAACAAGCAGCTCATCATGGATCTTGGAGGTGTTGAGGCCTTACAGAGTCTCGTGCGCTCGCCCAACGACCGAATATGCCAACAGGCGACGCGTGCGCTCGTGTCGGACCAGACCGTGTTCCGGTCGACCCCGGTAATTCGGTT--CGCGACGTCGCCGGTCATGTC------------------------------GGATCGCATGAGTGACACGATGGAGTTTGTCGCGGGCT-----ACAGTGTCAGTGGAC----------GGGATAGTACGTCGCCGTCGC-AGTTGGTGCGGAAAGT--CGAGTTTTCGTCCATTGCCGAAGACTTCCAGTCGTCGACCATGACCAATCTCATGCAGTTCCAACGAGCTACCCC------------ACCCTTGGGTA---CGATTTCCGAGATCACCC---AGCCTGATGAG-------------CAGACGATCTTGAATCGATCTGCTACGATCAAGAAGGAG---AGCGGAGAGGAGGAT---CGACACAGAACCCCAGACGTACCAGTACAAGGCAACCAA--GTCGGAACTCGACGAGGTGGAGCAACTCAAGTGGAACATTCAACAACAAGTGTTTCAGCGATACCATGATCTGAAGGCCAAGGAAGTCCAGTGGGGTTCACTAGAAATTCGGGACAGCGAGTCTTCTTCCACCCAGGAAAGCGACGAGAGGCAATCATGAGGGAGAAGAAGTTTCATGACGCCACATACAAGGATGGTCTGGCCGATGGCGATCCTTCATGGTACAAGACACGACAAGCTCAGTTTGCAGAGCTGATGCGAGAATTTGTAGATGAAACAAAGCACAAGCAAGAATTGATGCTCGCACATCTGGAGAAGTCATCGCCATTTTGCAAGTTTCCAATCCTGGGAAATCGTTTCATATTGATGCGACTTCTTGGAAAGGGAGGAAACGGTGAAGTGTGGGAGGTGATCGACTACGCGAACAAGAAACAGCGATGTGCGTTGAAGCTGTCTGTGTCACACCGACATGCGTGGCGAGAGCACTACACCCACACTATGCCATCAAC--ATATCGTGGGCGTCGGGGACAGTGCATACACGATCGAATACCGACAGCAGCTGTACACGGCTTTCACTGTAGAGTGCGTGGATTCGGATTTGCAACAACTTATTGAGA---TGTTCGAGCATTTCGATGAGACATCGGCTTTGAAGGTCCTATACCAGCTTCTTACCGCGTTGACTTACCTTCACGATGAAGATCGCTCACTACGACCTGAAGCCAACCAATATTTTGGTTGGCCGAGATGAGTGTGTCAAGCTG------ACCGACTTCGATTTGGCGCGAAACGTCAACGAGCCGATCGTGTCGTCAGTTGTGGGCACGCTCCGATATCTCCCACCTGAGTGCTTTCGACCCAACTTTGGTGA---------CTGCCAGGGTACTGCTGAGAAGGCAGATATCTGGATGGTAGGCGTCGTGTATTGCGTGATGCTGTGGGGCAAGCATCCAATTGTTGCGGACAAGGCGTCTCATGATGAAGCTCGTGCCATGCTGGGTCGCTACAACGGA---ACATTGCACTATCCACGCGAAGTGTCTGACTTGTCTCGATGGATCATTCAGGGATGCTTGCATCCTGATCCTCGTTGTCGACCGAGTGCTTTCCAATTACTCCAGGCATTACAA

Pythium_spinosum ACGACTACCACTCGGAGGTGGCGCGCGAGCTGGTGGCGTTCTTCCACTCGTTCTACCGTTTCGGCGCGGCGTCCAACGTCTTCGACCTGTTCCTGCACAATGTGCTCTTCGGCTCGCTCGCGATCGGCGCGATCGTGTTTATCGCGCTCGCGATCATCGTCGTGCGCCGGTGCTTTCTGCACATCCGCAGGCGTATGTGCGCAAGATCTCGATGGAGTCAAACGCGACCGAGTTCGCGGCCGTGTTGCTCATGACGTTCATCGCCGTGAGTGCGCTCAGCGGCTTGCTGGGTGAGGCGGCGTCGCGACCAGCGACGTCATCCGCACGCGCGCCGAGAACCTCGTGGTGTCGTTCGACGAGAGCGACTTGCCACCAGAGGCGTACAATCTGTCAGTGGAGGCCATGGCCTGTTCCACTCGGCCAAGGACCTCGTGAACACGACGGACATGCTGCTGCCCAAGCACTACGGCGAGATGGTGGAGGAGTGGCAGTTCAGCTACTTCATGCTCAAGTCGGCGACGAACGCGGCGATCCTGTCGGTCGCGCTCTCGAGCTTCCTGCTGATCTCGTCGATCGGCTGGTCGATGGTCTCGCCGCTGCGCCTCTCGATCCTGGTCATCCTGAGCGTCATCCCCGTGTCGCACACACTGATCGGCGTGTACCTGTCGTCGACGATCATGACGTCGGACTTCTGTGCCGCGCCGATGAACGCCACGGCGACGCTGCTGCACCCGTGCCCCGCGAACACCTCAGCCCGCTGATCGCGCCGGCAGTCGATCTATGCGCAGAAGCGCGGCGATGTCGGCAAGCGCATGAAGAGCGAGTTCCTGGACCCGATCGGCGCAGATCTGGGCGTCATGCTCGGATACTTCATCGGCACGGTGTCGAACTGGGAGAGCTTCCAGCTGATCGAGACGTGGCGCTGGCCGTTCGTTGTGCAGTTCATCGCCATGTGTCCGATCATGGTGGCGATTTTCTTTGTCCCCAAGAAGCACCTGATCATCCGCAGCGAAGCACGACAACAGCAACAACTGGTGCGCGGCGCGCCCGTGAGTCGCAGCGGCTTCTTGCACAAAGAAGAAGAGGACGTGCTGCTCGCGAGCGCCGGCATGCCACGTACGGGGCGTTCGACACGAAGTGTACGAGCAGGGGTCATTCTCCGACGGGCTCTACGAGCTCGCGCACATCCCGATCTTCTGTCTGATCGTCGGCGGACTCACGGCCGTGTACTTTGTCGTCACGGGCGTGCAGTACTGGAGCACAATCTTCATGATCAAGTCGCTGCACGCGTCCAAGTACCTCGTGAACGGGCTCTTCGTGGTCGTCGCTGGCACGGGTCCGATCTTTGGCGTGTTCTACGGCGGTTGGCTCATCGACCGCTACGGCGGGTACATTGGCGTGGAGCAGCGTGCCAAGTCGCTGGGCATCTGCATGGTGCTGGGCATCACAGCGTTCGGCATCTCGGCGGTCACGACGTTCTTCAACGACATCTACATCACGGCGGCGTTCCTGTGGCTGCTGCTCTTCTTCGGCGGCTCGATCCTGCCGGCGCACCGCGCGCTGGCGTCGTCGCTCTCGGTCATGGTCTTCAACCTCTTTGGCTACTCGCTGTCGCATGAGCGGTCTGGAGATGGTCGTCGGCGCCAAGTGCTACGTGCCGGACGAGACGCACGTGTGGCTCGCGGCCGAAGTGGTACGCGAGGAGAACTCCGGGGACGGCAAAGTGCGCAAGATTCACGCGCGCGTGGAGCTGCCGCCTAGAGTTTGAAGAACGCATCGTCGACATGAACGACAAGCAGACCAAGGCGTTGCTGCGGTGCACCAGCTTGACTCGCTCCCGTACCAGAACGAAAACGTCGGTGAGTACGGCATTGAAGACATGATCACACTTAACTACTTGCACGAAGCCGCGATCCTGTTCAACGTCAAGACGCGTTTCCTGCAGAAGCTCCCGTACACGTACACGGGTGACATTTGTATCGCCGTGAACCCGTACCAGTGGCTCCCAGATCTCTACGCAGATGAGCAGCACCTGCGCTACTTGAACCAACCAAAGGAAGAGCTCCCGCCGCACGTGTACGCGACGTCGGTGGCTTCCTACGACAATATGCGTCGCACGGAGCGCAACCAGTCGATCTTGGTGTCGGGAGAGTCTGGTGCCGGTAAGACCGAGACCACGAAGATCTTGATGAACCACTTGGCGACCATTGCCGGCGGTTTGAACAACTCGACGATCAAGAAGATTATTGAAGTGAACCCTCTGCTCGAGTCTTTTGGTAACGCAAAGACCGTGCGTAACGACAACAGTTCGCGTTTCGGCAAGTTCACGCAGCTGCAGTTTGACCGCGAAGGCACGCTGGTCGGCGCCAAGTGCCGCACATACCTGCTGGAAAAGACGCGTGTGATCAACCACGAAGAACCAGAGCGAAACTACCACATCTTTTACCAGTGGA---AGGTGACTCCGCTCAGGAACTGCTCTTGGACTCGTCGAAGACGTACGCGTACACGGGCAACAACAACGCGGTGAAAATCGAAGGCATGTCGGACGCCAAGCACTTTGAGAAGACGCGCGTTGCGCTGGAGCTCATTGGGCTCACGCCAAACGAGCAAAAGCCACTTTTCGAGGTCTTGGCTGGTGTGCTCCAGCTCGGTGAAGTCTCGCTGCTCTCCAACCCAGCGAATGACGAAGAATCGCTGATCGCGGAGGGTGATGCGGGCGCGCTCGCGTGTGTGCAGCTTTTGGGCGTGACGTGGGAAGCGATTGAGAAGGCGCTCTGCTCGCGCACGATGCGTGCCGTGAACGATGTCTACAGCGTGCCATTGCGCAAGGAGCAGGCCATGGACTGCCGTGACGCGCTTGCGAAGGCTATTTATTCGAACGTGTTCGACTGGCTCGTGTCGTCGATCAACCACTCGCTCTCTGACGACAGCAACATGTTCAACCACGTCGGTGTGCTCGATATTTTCGGTTTCGAGCACTTCAAGCACAACTCGTTTGAACAATTCTGCATCAACTACGCCAACGAAAAGCTGCAGCAGAAGTTCACGCAAGATGTGTTCAAGACCGTGCAGATCGAGTATGAGGAAGAAGGCATCACGTGGAACCACATCGAGTACGCCGATAACCAGGACGTGCTGACTGTGATTGAGTCTAAGATGGGTATCATCTCGCTCTTGAACGAAGAACTGATGCGTCCAAAGGGTAACGAGGAGTCGTTCATGTCGAAGGTGGCGTCGCTGCACAAGGAA---GACATGGCGCACGTCATTGAGTTCCCAAGAACTTCGCGTACGGAGTTTTTGATCAAGCACTACGCCGCGCCTGTTATGTACGACTCGGTCGGTTTCTTGGAGAAGCACAAGGATGCCTTGCTCCCAGACTTGTCGGAATTGATGCGTAGCTCTTCAAAGCCATTCATCTCGGCGCTGTTTGAGCCAAAGCCGGCGCCAAAGTCGCCGCAAGCG---TGGTGGCGGTGGCGCGTTGACTCTGTCTACTGTCGGAACGCAGTTCAAGGAGAGTTTGGCGGAGTTGATGACCACAATTCAAGCCACGAAGGTGCACTACGTGCGATGCATCAAGCCGAACTCGATCAA---------GAGCTCTACGGCAATGGACCAAGCGATGGTTGTCTCTCAGCTGCGATGCGCTGGTGTGATTGAAGCGATTCGTATTTCCCGCGCTGCGTACCCGAACCGTCTGCAGCACACGGAGATTCTGGACAAGTTCTGGCTGTTTGTTCCTCGTCAGGGAGAAACCGCGAGCGAAAAGTGCCAGTTGCTGATGGAGAAGCTCAAGTTGACGACTCCAGAGCAGTACCAAATGGGTAAGACTCGCGTGTACTTCCAACTCGGCGTGCTTGAAGAGCTCGAGGACCGTCGTAAGAAGTTCTTGGACGCCAAGGCGACGTACCTCCAGGGTGTGATGAAAACGTTCACTCAGAGAATCAAATATGTGCGACAGCTGGCTGCGATCATCAAGTTCTGTTATTCGCTGCGTCATCGCCATGAGAAGATACAACACGTTCATGAATGGTCTCGTGAAGGCGCAAGCACGATGGAGAGGTATTGAAGGACGCAAGCACTACACGGAAGTGCGAAGCAACCACTACGCCGTGATCATCCAGCGATACGTGCGCGGATTCGTTTTGCGTCACCAGTACACGAAGGTGCGCGCAAGCATCATTCGCGTGCAAGCTATGGCGCGAATGAAGATCCAACGTCCGAAGTACCTCGCTGCTCTCCAGGAAGAGAAGCTGCAAGCCGATATGGTGTACCAGCTGAGCCAACTCAAGGCTCGTCTTCAAGAAGAGCAGGAGCGCAATGCCCGATT------GCAAGAAGAGCGTAACAGCGTCGC--GACCACGCTGCCGCT---------------------------------------------------GAAGAGAACTCGAAGATCCGTACCAAGAACGAGGACTTGAAGAACACGGTGAAGCAGCTGAAGGCTGAAGTTGAGAAGTACAAGTCTGAAAAGGAGATGAGCCACGCTAGCCTGTACGTGAAGATTCGTCAGCTGGAAGACACTGTTCGCGAGAAGGAGAAGCGTGTTACTCAGCTGGAGGGTGACAACCTGAAGCTGACAGACCAAGTGCAGGATCTGATCGCGAACGGCCACGTGCAATTTGAGAAGAAGACCAAGGAGAAGCGCTCAATTTTCCGCACGCTCGGCTCCAAGAAGGAGAAGAAG---------------------GAAGTCGTGTTGCTGGACTCGCTAACTGGAGACGACGACGTGAACGGATCGGACGTGCGCGCGTCGAATGTGCGTCACCTTCCAAAGCTACTCCCTTCCATGAACATGTCGAAG---AAGTTCTGGGGCAAGTCCAAG---GGCGAGAAT------------GGCGCCGACGGCAACGACGATCGCGATTCGAGCGGCAACGACTCTCAGAACGGCGACCTCGCAACACGATCGTCAAGAGCATGGAAGTCGGTGTGACTGGTGCGATGACAAACCTGAAGGGCAAGATG---------GCGGCTGTGAAGACCATGTACGAGTGAAGCGTTCGGGCAGGACCGACCAATCCGCGAATCGTTCAACTTGGATGCACTGCCAGAAGTGTCACTGCCTGTTGGATGGGAAGCCAAGGTGTCTCGCTCCACTGGTCGCGTGTATTACGTGAACCGCAAGCTCGGCAAGTCTCAGTTTGAGCGTCCAACGCTCGCGTCGCTCAA------GGCGCAGAAGCTGGCGCGTCAGAAATCTGTGAGCGGTCCGAAAGGCTGTGGTCGCAGACTTTGGGTGTGGCGACGCGCGCCTGGCCGAGAGCGTGCCGAACAAAGTGCACTCGTTCGACCTCGTGTCGCTGGACATCGCGGTGTACTGTCTCGCGCTCATGGGCACGTCCGTGCGCGAGTTCATCATGGAGGCGCACCGCGTGCTCAAGCCGAACGGCGTGCTCCAGGTGGCCGAGGTCAAGAGCCGCTTCGAGACCGAGTCACTTGGCGGATGGTGGCGTCCTTCCTCTCCGTGTCCGGCGACGTCGTGGTGCTCGTGCGCGACAAGGCGCTCTTCGTGGCGAGCGCAAGTATGTGGTGCTCGCCGGCGTGAAGACCGGCGAAGCCGTCGCGATGCCGTTCCCAGACATCAACCGCGACGTCAAGACGCTGCTCGGCCATACGACGTCGATGATCGTGCACTTGACCGTGAACCTCGACGCTGTTGCTCACGGGCGACCGCGACGAGAAGATCCGCGTCTCGGGCTTCCCGCGCACGTCGCTCATCCAGAGCTACTGCCTGGGCCACAGCGAGAGTCTCACAAAGGTGGCGACCAGCTCGCTCACGCCGGACCTCGCGGTCTCGACGTCGCTGGATAACACGATCAAGCTCTGGAGCATGTCGGCGGGCACGCTTGCGGTCTCGGCGCAATCGATCTCGTGGCGGTCGTGCTCAACAACCAGTCGGTGCGTCTCTTCGCGATCGTTGTGCGCCATTCGTGGGCTTCTACAATGTCAACAAGGGACGACGAAGTGCTGGCAGTTTGTGCGCGACTTCCGCAAGTTGCAAAACGACCCGCCGTCGGGCGTCTCTGGCGCGCCGATGGACAACAACATCATGCTCTGGCAAGCCGTCATCTTCGGACCGGATGACACGCCGTGGGAGGGCGGTACTTTCAACCTGACGCTCGAGTTCAGCGAAGACTACCCGAACAAAGCACCGACCGTGCGATTTATCACAAAGATGTTCCACCCAAACATCTACAATGACGGCCAGATCTGTCTCGATATCCTGCAGAACCAGTGGTCGCCGATCTACGATATCTCGGCGATCCTGACGTCGATCCAGTCGCTGCTGTGCGACCCGAACCCGAACTCGCCGGCGAACTCGGAGGCCGCGCGCCTTTTCCAGGAAAACCGCCGCGAGTACAATCGCCGCGTGCGCGAAATCGTCGAGCAGAGCTGGGTTGCGATCTATGGTGTCGGCCGCCAAGATGCGTGGTGCTGAGAACCGCATGAACGCTGGTCGCCCATTCACTGCGTGGTTGGACAACGTGTCCTCGGACCGTGGTCTCTGCGGTGGTATCAACTCGGGTATCGCCAAGGCCGTGCGCAAGCAGTGTCGGCGGAGCTGTTCGTGATCGGTGACAAGGGTCGCGCCCAGCTTCGTCGCGACCTCGGCAACCAGATTCGCGGTAACGTGACTGAGACCTTCGCGTCGCCACCAAACTTCACGGTTGCCAGTGCCATCGCTGAGGGTGTGGAGAACGAGAGGAGAGTTCGCGTATGTCGGCCATGGAGAACGCCACCACGAACGCCCAGGAACTCACGCTCACCCTTGTGTACAACAAGGCGCGTCAATCCCGCATTACGACGGAGTTGATTGAAATTATTTCCGGTGCCGCGTCGCTCGACCCTCACTGGCTCCGTCATCGACGTGCAGGACGCCGCGCAGTTCGACGAGCTCACGGCGCGCGAGTCGACGCTGAGCATCTCGTTCTTCTGGGCGGACTTCCAGCTCTGCCGCCCGAACGGCCAGATCGACGTGGGGCAAGAGCGTGCTCGAGAAGCTCGAAGGCGCGAACGTCGCCGAGCTCGCCAAGCGCGTCTCGAGTACCGCCTCAAGAAGCTCATCAACGCCTCGCCAGTGATGCTCTTCATGAAAGGCAACCCGAGCGACCCAAAGTGCGGCTTCAGTCGCCAGGTCGTCGCGCTCTTGAACGAAGAGAACATCCAGTATGGCACGTTCGACATCCTCAACGACGAAGAAGTGCGTCAGGGCTTGAAGCAGTTCTCCAACTGGCCGACGTTCCCACAGCTCTACGTGAACGGCGCGCTGATCGGCGGACTCGACATCCTCAAGGAAATGAAGTCGGAGGGCTCGATTGTCGAAGAGCTTGGCGTTGAAGAAGGTGAGGCCGCGTTCAACGAGAGTCTCCGTGCGCTCGTCAACTCGGCGCCTGTGCTGCTCTTCATGAAGGTTCGACATCTTGTGTGGAGCTACGAGCGTCCGAAGCACGAGATCTCCAAGATGGCGACTTTCGCGCCGACGTCGTTCCGCAAAGACATGTGCGGAGAGCATCGTGTGGAGTCCGCAATCGCTTGGCGATGGCGTTCCGCGATCCGATCGACGGCATGGGCGAGTTTTGCGTCGTGATGCTGCTCGAGTCACGTCCAAGTCCACGACGCTGCACTCACGCGGCATCTCGGCGATCGAGTGGAAAGTGGAACGTCCTGCACCGGGAGCACCGCTCGGAGGTCAAGTCGATCTGCGTGCATTCGCAGCGCCAAGCGATCTACACAGGTGGCTTTGACGGCCAGGTGATCCGCTACGACATGCACAGGTTCGCGCCGCACATCGTGATGGAGCGTCGCAAGCGTATCTCGAAAATCAACGCCGTGCTGGAGCACCCGCACAACCCGAACATTCTGCTCGTGAGCAGCGTCGAGCAGGGAGCACAGCATCCTGCTGCACGACCTGCAGCGGTACAACAGCAGTCTGACCTCCAAGTCGATGAGCCAGTACATCGTGCCTCGCTGGTCGCCCGCGGGCATGCATGTATCCTGCGGGAGCAAATGCGCTACTGTCACGGGAGCTTCTCAACACCGACGTCCGCGACCATCGGCGCGTCGTTCTTGCAGAAGCGCATCATCGTGGGCGGGAAGATGACGCTGCAGATCTGGGACACGGCCGGACAGGAGCGCTTCCGCTCCATGGCGCCGATGTACTACCGCAACGCCAAAGCCGCGATCCTCGTGTTTGACTTGCTGAATGAGGCGACGTTCGAGAAGGTCAAGGAGTGGCTGCATCTCCGACATCATGTGGGCGACGATATGTGGGCAACAAGTGCGACGTTTCAACTTTGCGAAGGCGCAGCAGTTCGCGCAGGAGATTGGCGCGCTGGTGTTCCGTACGAGCGCGAAGACGGGCGACGGCGTGCAGAACCTGTTCGAGAGCGTTGCAGTGCAGCTACTGAAGCGTCACGAGGAGGAGGAGAAGCGAGCTTCTTCGTCGTATGCGTGCCTGCGGACGGCGAGAAGCAGCTGGAGGAGTGGGCGGTGGCGCTTCCGGACGACAAGGAGGCGCAGATCGGCTGCCTCACGGACCGCCTGCGCGCGCACTTCAAGCAGAGCGGCATCGACGGCGGAGCAGAGATCTTCAAGAAGCAGTTACTGAGCCAACTGCCCAAGGGCCAGACCATGACCGACGACATGCTGCAGATGATGCATGGACAGCCTCGTGGACTCATTCCCGCTCATGCTCAACACGCGCGCCGTGGGCCACGTTCGAAGTGCGTGGCGACGCGTTCATCGCGCGCGTGTTTGACAGACGACTTTGTGCGCATGGACTTCCGCGTGTCCGAGATGTCGATGGACGCCGACTGGATCAAGATCGGCAAGGAGCAGAAGTGACGCAGTCGCTCGAAGAGATCCTCGCGCTCGGCCAGGGACTCGACGACGAAGACTACAAGGCAGAGCTCGACGTACTGCAGAACGACCCGAACTCGAACCTCTACTCGGCTGTGACGTTCGAGGAGCTCAACCTCCCAGAGATGCTGCTGAAGGGCGTGTACTCGATGAAGTTTTCGAAGCCATCGAAGATCCAGTCCGTCGCGTTGCCATTGATCCTCGCCGACCCGCCTGAGAATTTGATCGGCCAGGCGCAGAGCGGATTCCGACCCGCGAGCTCGTACGCCAGATCATGGCGGTCGTGAACGCGATGGGCAAGTTGTGCAGACGTTCCTCGCGATCCCGGGCGTCGACGTCGCCAAGGGCGAGCAGGTGCAGGCGCAAGTGGTGGTCGGCACGCCCGGCCGCGTCGAAGGGCTCATCAAGAAGAAGCAGCTCGACACGCGCCACATCAAGATCTTTGTGCTCGACGAAGCCGACGTGATGGTGTCGGAGGACGGCATGCGCGACCGCTCGGTCGCGATCAAGAAGATGATCAAGAACCGCAGCTGCCAGTACCTGCTCTTCTCGGCGACGTACGCCGACGAAGTGCGCGACTTTGCGCAGCGCATGGTGCCGGACCACAACATCATCACCGTCAAGAAGGAGAAGCTCACGCTGGACGGGATTAAGCAGTTCTGGATCGACTGCAAGACCAAGGACAACAAGTTCAAGGTGCTCTCGGACATCTTTGCCGTGCTCACGATCGGCAAGTGCGTGATCTTTGTGCAGTCCCGCGACACGTGCAAAGAGCTCACGAAGCGCATGCGCGCGGGCGGCCACTCGGTCGGCATTTTGCACGGTGCCGACATGGCGCGCGAAGTGCGCGACCAGGTCATCGACGAGTTCCGCGCCGGCACGACGAACGTGCTGATCACAACAAACGTACTCGCGCGTGGCATTGATGTCGCGGGCGTGTCGCTCGTTGTCAACTTTGATATCCCGCTCAACCGAGACCACCAGCCGGACCCGGAGACGTATTTGCACCGCATCGGTCGTACCGGTCGCTTCGGCCGCAAGGGCTGCGCGATCAACTTTGTGCACGACGACCGAAGCAAGCAGCACCTGGCGGAGATCGAGGCCTACTACGCGCGCGAGATCGTGCAGGCGCCTGCCGACGACATTGAAGAGCTGGAGAAGCTGCTCGCCTGGGGGGCAGTAAGCGCCGCCTGGCCAAGTTCGGCAAGGAGCAGCGCAAGGGCATGAGCATCTACCCGCGCGAGCCGAAGAAGAAGCCAAAGGGCAAGGACAAGACGTACTACTACCTCAAGGATGTCATGTACCTGGCACACGAACCCGTGCTCGAGAAGTTCCGCGAGTTCAAGACGTTCATGAAGAAAGTCACCAAGGCCATGGGCAAGCGCGACTTTGAGGACGCCAAACGTAAGCACGACAACCGTCCGATGTACACGCTTGATCACATCGTGAAGGAGCGATACCCGCGCTTCGTGGATGCGCTCGGCGACATGGACGACGCGCTCTGTGGAACAACTACGTGGCCACCTCGCGCTCGCTCACCAAGGTGTTTGTGAGTATCAAGGGCATCTACTACCAGGCGAACGTGCAGGGCCAGACCGTGACGTGGCTCGTGCCGCACCAATTCACGCCGACCATGGACAAGCGCGTGGATGTGCGTGTGATGCTCACGTTCCTCGAGTTCTACGAAGTGCTGCTCAAGTTCGTCAACTACCAGCTGTACACGGACCTGGGCGTTGCGTACCCACCAAAGATCGACCTGCAGTTGGACCAGGCGGGTGCCCAGCTCGCCGCCCTGCAGTTAGCAGCAGAAGGAGTCGGAGAAGCGCATCAAGCTACAACGACCAGCGCAAGGAGGTGAGCGAGAAGAGTCTGTTCAGCGGACTGTTCTTCTACCTGTCGCGTGAGGTGCCGTCGGCATGCCTGGAGCTCGTGCTTCGCTCGCACGGCGCGCAGATCGGCTGGGATGGGGCCGGCTCGCCGTACACGGAGAAGGACGAGCGCATCACACATCACATTATGGACCGTCCCCACCAGGGCCACCGCTACTTCAACCGCGAGTACGTGCAGCCGCAGTGGGTGTTCGACAGCATCAACAACGGTCTGTTGCTGCCGCTCGCGCGCTACCGCAAACAGCTCAACAAGCTCAAGTCGGCCTCGCAGGTGCTGCGCGAGGTGGGCGACCTCAACAACGACGACGACGTCTGGAAGCGTACGCCGCTGGTCTCAAGGCCGAGCTGGACGGCAACAAGAAATCGAAGAAGGTAAAGCGCGAGGAGGAGGAGGAGGAGCTCCACGAGCTGGCCAAGAGCATGATGTCGAAGAAGTCGAAGCGCTTATACGACCGCATGCAGTTCGGTCTGGGCAAGAAGGCAGACAAGATTCAGGCACTGGAGGCTAAGCGCGCCAAGTTAGACGCAAGACCAAGAAGCTCGTGGCCGGCGCGCTTTCCGGCATGATCTCAGCGCTGGCGTTCCAGCCGCTCGGGCGATGCGCGGGCGCGGGACGTCATCCACGCTCATCCGCGTCGCGGGCGGCGCCGGACTCTACTTCACGACGCTCGACCACTGTATGACCATGTTTCCCACGTCAACGATCAACACGTTCCTCTCGGGCGCGTTTGCGCGGACGTTCGCGGGCTGCGTCATGTCTCCGTTCACGATCGTGAAGGCACGCATGGAAGGCGCCAGGCACGTACAGCAGCAAAGGGACGTGCCGTTCTCGGGGCTCTACGTGCTCATCTATACGGCACCCGGCGGACGTGGTGAAGACGCGCATGCATGCGCCAGACGGTGCGCAAGATCGTTAGCGAGGAGGGCTGGCGGGGCTTCAAGAAGGGCATCGCGCCGCGGGTGCTCAAGCGCACGCTCTCGACCGCCGTCACCTGGACCATCTACGAGCTACTCGTTCGACCCCTCAGGTCTCGGCCACGCGAAGGAAGCGTTCCAGATCGCCAAGGAGACCGAGAAGACCAAGCAGCTCGAACACACCTCCAAGGTGAAGGAGAACGAAGCGCTCTACAAACAGTACGAGATCGTGCGCGTGCAGAAGGAAGGCGAAGAGCGCCGCAAGACCCTCGAAGAAGAGACGCGCCAGCACCAGCAGCGCGCACAGTACCAAGATCAGCTCAAGCGCAAGCAGTATGCCGACCAGCTGGCGTATCAGAAGCAGATGAAGGAGCAGGAGCTCAAGAAGCAGGAAGAGATGCTCGCGCGTCAGGAGAGCTCGCGACGAAAGACCCTCGAATACGAAGCCGAGCTTCGTCAGAAGACCGAGCTCTCCAAGGTCGCGGCCGAGGTCGAAGGTAAGATCAAGCAGGAGCGCTTGAACCACGACCTGCACTTGGAAGAAGCGCGTGTGCGCGCCAAGGAGTACCGTGAGACCGTCATGGAAGGTATCAAGCTCGCGGGTTCCACGATCGGTTCCGGTGTCATGGCGTTTGTGGACGACAAGGAGAAGCTCACTGCCACCGTCGCGTCGCTCACCGCCCTTGCTCTTGGTGTGTACACTGCGAAAACCGGTACGGGCGTTGCGGGCAAGTACATCGAAGCGCGTCTCGGCAAGCCGTCGCTTGTGCGTGAGACCTCGCGCCGCACTGTCGCGCAGGTTGTCGCGAACCCAGTGCCGTCTATCAAGCGCGTGCTGCGTCTTCAGAAGCCGCATGACGCGCTGGAAGGCGTTGTCTTGGAGCCAAAGCTCGATGAGCGTCTGCGCAGTGTGGCGGTGTCCACGTTCAACACGAAGAAGAACCGCGCGCCGTTCCGTCACTTGCTGCTCCACGGTCCGCCAGGTACTGGTAAGACGCTGTTCGCAAAGGCGCTTGCGCGTCACTCTGGTCTCGAATACGCTATTTTGACCGGTGGTGACGTCGCGCCGCTCGGTCGCGAGGGTGTGACGGAGATCCACAAGTTGTTCGACTGGGCGTCGCACTCTCGTCGTGGTCTCTTGCTCTTCGTCGACGAAGCCGACGCGTTCCTGCAGAAGCGTAGCAACACCGTGATGAGTGAAGACATGCGCAATGCGCTCAACGCGTTCCTGTACCGCACGGGTGAGGCGAGCGATAAGTTCATGATCGTGTTTGCGAGTAACCAGCCGGAGCAGTTCGATTGGGCGATCAACGATCGTATCGATGAGATGGTCGAGTTCTGTCTGCCGGGCCACGCCGAACGCGTGAAGATGCTCAAGCAGTACTTTGACAGCTACATCCGCAAGCCGAAGAACTCGTCGGCGAAGAAGATCTACGCGAGGGCGTGATCTTCACAGTCGACCCCGTCGCGAGCTTCCTCATCCCAAGACGCGCATGTTCCAGATCGAAGCGCTCAAGGACGTGTCGGCGGAGGCGCAGACCATCTTTGATGCGCTCAACAAGAGCCCATGCGCCTCTACTGGATCATCTTTGGCGTCGTGCTCGCGAACGGCGTGTACACGTACTTCAGCGGCAAAGATGAGAAGTACCTGGTCGACAAAATCCAGAGAAACGTGCCGTCGGAGTTCCGCATCTCGGAGAAGTGGGACAAGTGCATCGAGAACTTTACGCTGCACTTCGCCGCGGGCCTCGTCGCTGGCGGCCTCACCTCCGTCGTCCTCGCGCGCTCTGGTGCGGCTCGCGGTGTACTGACGGGCTTCGGTGCGCCGGCGCCGGCTCCAGCTGGACGACGTGCCAGCTCGCGTTCAAGGGCGACAGGCAGGCGGTGCTGGACAAGTCGGACAAGATCGTGGACGAGATGGCTGCCTACAAGACCCCCTCGCTCGTGAGCTACACGACCGCCTCTCCTGCGCAGAAGCAGCTGCTCGCGTTCTACCAACAGCTTCTACGACGACGCCGCCGTCAAGGTGCTCGAGGCGTACCTCGCCGAGCAGATCGCGAACTCGACCGTGGACATTGACGCCAACCTGGCACTCCTGAAGCTCTATCTCGTGTTCCCAGCGCACTTGGACGCCGACAGCCAAGGTGCTCGTCAAAGGCATCACGGTCACGCCATCGCTGTTCTTCACCGGCGTCGAGCATCGTACCAGAAAGCGTTCGCGAGGATGCGAACGTGGAGCTCGCGCTGGAAGCCGGCTTCCTCCTGCAGTCGTGCTTGTTCGCGGAGTTCTGGAAGCTCGATCTGACGTTCGCCAACGCCGTCACGGGCTTCTCGGACGCCGTGCGCGCGTTCATCCTCGACGCGCTCCGCAAGAGCCACAGCGTGATCTCCACGGCGGTGCTCAAGTCGGCGCTCCACATCTCGGACAAGGAAGTGGCGGACGTGATCGCGGAGAAGTGGGCGAGGGCGACCTGGTCAAGGTCACGCCGAACGAAGACAACCAGGTGCGACCAAAGAAGATCCAAGAGAAGATCGAGTTCGAGGACGTGCTGAAGGTCATCGACACGCTCTCTAGATCGAACGTGTGCGCGAGCGGCGGCGGCGTCACGGACGCGCAGGAAGTGTGCGACGCGCTCGCCAAGCACCTGCGTCTCGAGAGCGGCAACATGGTGCTCCCGAGCTCCACGGGCGTCATCGGCTGGCGCATCCCTGTGGAGGCCATGATCGAGCAGATCCCGCAGCTCATCGAGAACCTGCAGTCCGAGTCCGTGCTGCCAGTCGCGGAAGGCATCATGACCACCGATTTGTACCCAAAGATCCGCTCGATTGACGTGTGCGGCGGCCGCATCACGGGTATCGCCAAGGGCGCCGGCATGATCGAGCCTGACATGGCGACGATGCTCTCGTACATCCTCACGGACCTGTCGGTGCCGCGCGACGTGCTGCGCCAGCTGCTGTCGGAGGTCGTGGGCGATACGTACAACGCCATGAGCATCGACACGGACCAGAGTACGTCGGACACGGTGTCGATCGTGTCGTCGGACCAGATCCCGTTCGACATGAG---CAAGCTCGCCGAGTTCAAGCAGGCGCTGCAAGAGGTCTGCCAGGGGCTCTGCGAGGACATTGTGCGCAACGGCGAGGGCGCGCACCACGTCATGCGCGTGGCCGTGCGCGGCGCCGAGTCGGTCGAGATGGCCAAGGGCGTGGGCAAGTC---------------------------------------------------------------------------------GTACATGGGCAAGCACCACAAGGACGTGAACATCACGCGCCGCATGAAGATCAGCGTGGGCGGCATCTCCATCTTCGAGAACGGCGAGTTCGTGCTCAACAGTGACATCGAGCAGCTGCTCGTGGACCACATGCGCCAGGCCCAGCTCATCGAACCAACGCGAGGCGGTCTGGACATCACCTCGCACGACTACCCGCCACACGGGCGCTTCGTTGAGATCGATATCGACCTGGGCATTGGCACTGAGCGCGCGCGTGTGCTCGGCATCGATCTCACGCACGAGTACGTCGCGATCAACGCCGACTATCGATCCAAATGCCGCC---GAAGAAAGGGAAGACGGCCGCCGCGTACCTCGCGTCGCTGGGCGTAGACACGTC---TGCCGGCGGCGACAAGAAGGACAAC---------AAGAAGAAGAAGGGCAAGAAGAAACCGGAAAGAAGCCCGTGTCCGCCGCCGCCAAGCTCCTGG-CGGAGCGACAAGCAGCTCTCCGCGCCGAGCAAGAGCGCATTGAACGCGAGCGCTTGGAGCAGGAGCGCATCGAGCGCGAGGAAGAGGAGCGCCGCCTCGCCGAGGAAGCGCTCCAGGAGGAGATCCGCCGCAAGAAGAAGGAGGCCAAGGCGGCCAAGGTGGAGCGTCAGAAGAAGGAAGGCACGTACATGACAAAGGCGCAGAAGCTCAAGGCCGCGAAGGCGCAGGCCGCGCTCGAGGCGCTGCAGCAGGCTGGTCTCGTGCCAGACGCAGCTCCGGAAGAGGAGAAGAAGAAGGTCGTGTACACGAACAACAAGAAGGGCAAGAAGCAGCCAACGACGTCG----AGCTGAGGCCGAAGCTCCAGACGCGTGGGATGACGAGGACTGGGAGACCAGCGACGTGGTCGCCTCGCTTGAGGACAAGCTAGCAGAAGAAGGAGCAGGAGCGTCTCGTGCAGCAGGGCATCGCGCTGAAGAAGCGCCAGGAGGAGCAGGAGGAACAGGCGCGCATCCACGCCGAGGAAGAGAAGATTCGCTCCAAGGCGCAGCAGGAGGCTGACCGCAAGAAGCGCGAGGCCGCGGACCGCCGTCGCGTGCGCGAGGAAGAGGCGCTCGCGAACGTGTCGCTCGACAACCTGCGCTCGCCGATCTGTTGTATCATGGGCCACGTCGATACCGGTAAGACCAAGCTGCTCGACAACATTCGTAAGACCAAGGTGCAGGACGGCGAAGCCGGCGGTATCACGCAGCAGATCGGCGCGACATTCTTCCCAGTGGAGGCGATCAAGCAGAAGACCGAGCGTCTGAACAAGACCGTGAAGCTCGACTACAAGCTGCCGGGTCTGCTTGTGATCGATACCCCCGGTCACGAGTCGTTCACGAACCTGCGCTCGCGCGGTTCGTCGCTCTGTGATATCGCGATCCTGGTGGTCGACATCATGCACGGGCTGGAGCCGCAGACGCTCGAGTCGCTGCGCCTGCTGCGCCAAAAGAAGGCGCCGTTCGTGGTCGCGCTCAACAAGATCGATCGCTGCTACGGCTGGAAGACCATGCCGGACATGCCGATCCAGGAGGCGCTCAAGCACCAGAACGAGCACGTGATCCGCGAGTTCGAGGACCGCACCGCGAAGATCATGGTCGAGTTCTCGGAGCAGGCGCTGAACGCCGAGATCTACTGGCGCAACAAGGACCTGGGCCGCACGATCTCGCTCATCCCGACGAGCGCCATCTCGGGTGAAGGTGTGCCGGACCTCTTGATGATGATCACACGCCTCACCCAGGAGCGCATGGCCAAGTCGCTCGCGTTCGTCGATATCTTGCAGTGTACCGTGCTGGAAGTGAAGGTGATTGAAGGCTTGGGCACGACCATCGATACGATCTTGGTGAACGGCACGCTGCACGAAGGCGACACGATCGTCGTGTGTACGCTGGACGGTCCCGTGGTGACGACGATCCGATCCATTTTGACGCCGCACCCGATGAAGGAGATGCGCATCAAGGGCGACTACATCCACCACCAGACCATGAAGGCAGCGATGGGTGTCAAGATCTCGGCGCACGGGCTCGAGAAGGCGGTGGCTGGTACGCAGATCCACGTGGTCGGCCCCGACGACGATGTCGAGGAGCTCAAGGACTCCGTCATGTCCGACCTCACGGGCATCTTGGACAGTGTCAAGGCCACGAAGCGCGGTGTGATGGTGCAGGCGTCGACGCTCGGAGCGCTCGAGGCACTGCTGGAGTTCCTGCGCACGTGTGACCCGCCGATCCCCGTGTCGTGCGTGAACCTGGGACCGGTGCACAAGAAGGATGTGATGCGCGCGTCGGTGCAGCTCGAACACCAGAAGGAGTTCGCGACGATTCTGGCGTTCGATGTGAAGGTGCACAACGACGCGACCGAGCTGTCGCAAGATCTGGGCGTGCGCATCTTCACGGCCGACATTATTTACCACTTGTTTGACCAGTTCACGGCGTACATGGACAACTTCCGGGAAGCGCGTCGCGAGGAGTTTGAGGATGTCGCAGTGTTCCCGTGCGTGCTCAAGATCCTACCGAACTGCATTTTCAACAAGAAGGATCCGATCATTCTCGGTGTGGACGTGGAAGAGGGTATCCTGAAGATCGGCACGCCGCTCGTGGTGCCAACGGCCGGCG---GCGTGTTTGTGGGCAAGGTCGGCTCGATCGAGCGCGAGCACAAGGAGGTGGACCGCGCGAAGAAGGGCGCGTCGGTGGCCGTGCGCATCGACAACGAAGGCAGCATCACGTACGGCCGCCAGTTCGACCACAAGCACAAGCTCGTGTCGCGATTATCGCGCGCGAGCATCGACGCGCTCAAGGAGAACTTCCGCAACGACCTGCAGAAGGAGGACTGGCAGCTCGTCATCCGTCTCAAGAAGGTATTTGACATCATCATGGAGGGCTGGCTCACCAAGCGCCGCGACCACATGAACCTCATCTGGCTCGAGCGCTATTTCGTGCTCGATGGGAACCAGATGCGGTATTACCGTAAGCGCGGAGACCCGGCGCCGCGCGGCACGTATGTGCTCACGGATGGCTGCCTCGTCAGCAACGTCTTCAACTCGGAGGAGAAGCACCGTCATCACGGTCCGGTGTGGATGTTCCGCATCACGTGAGAAGTCATACAAGAAAGATCGGTTTCTGGACCTCGGCGCCAAGACCGAAGAGAACGCGCGCGAGTGGAAGAAGGCGATCGAGAACGCCGTGCGCTCGCTGCGGGAGCTCGTGCTGCACCAGCGCAAGATGAAGACTGACGCCGCGAT---CTACCCGACGCAGTACTCG---------GTGCCACGACTGCTCAAGGCGGACGAAGACACGGTCTCGCTATTCAATCTGGACGCGGACAACGTCAAGGCCGAGCAAGGATGGTGGCTCGCGCGCGTGGAAGAAGGACTGCGCATCATGCAGGAGTGTCCGCTGGGACCGAACGTCGCGGCGTCGCTCTACGCGCAGCACGCGAATACGACGAACGGACTCATCTTCTCGTCGCTGATCAGCGCGATCCTCACGTTCAGCATCATGTACTCCTTC---------------------------------------------------------------------------------------ATCCCGTCGTTCCGTGTCTCGCAGGTCGTGAACGGCTCGCCCACGGAAGTGTTTCGGCTGCTCATGAACCCGAAGCGGTACCAGCGCTGGGACCCGTCGATCGCGACGATGAAAGTGATCCAGGAAATGGACGACCACGCCGACATTGTGTACATCACGCAACGTCCGACATACCTCTGGCCGCTCTGGCAGAAGCCACGCGACCTCGTGCTCATGCGCTACTGGCGCCGCGAAGAAGACGGCTCGTACTTTGTCATGTACCAGAGCATCGAGCACCCAGAGTGCCGCGTGCGCCACAACTACGTGCGCGCGACGCTGCTGGGCGGCGGGTTTGTGATCGCGCCGCAGAAAGTGCCGAGC---GGCGCGATCCGCTCGCACGTCACGTACGTGCTCCGCTACGACCCGGGTGGTTG---GTCGAGAATCTACCACCAGCTCGGTATGGACGTGGACTTGGTGATGCCCATGCTGCGAAGTGTCGTGGGCATCCGCGACGAACTGAGCAGCGTCGACTTCATCACACCGAACGTCACCGTCGCGGCGACTGGTGCAGAAGGAGGGCGCTGGCGGAGCGTCGA---CGCGTCAGGCGTCGTGGTCGGCGTCACGAAGCTCAAG---ACGAGTCTTCCGGAGAAGATGTGGTCGGAGCCCGACGCGTCGGTGTTCCAGGTGCGCGGCTCAAACTACCTGATCGACAAGAAGAAGATGCCGTCGGCCGAGTCCAAGTTCCACTTGGTGGGTGTCGACTTGCTCTCGTTCGAGAGCGCGAGCGACCGGTACCATGTGTCGTCTCGCAATGGCAGCATCGGCCGCACGTC---GACCAAGTTTACGTTTGTGATCAACATGATCATCCCCGGCGCGGAGAACGTGTGCATGGTGTTCTACTTCCAGCCGGCGAAGGACAACATCTTTGAAGATGGCTCGCCGTTCTCGGAGCTGCTGAACGACTTCATCGACGGCGACGACCAGTTCCGCAACTCGCGCTTCAAGCTCATCCCGACGGTCGTGGAAGGAAGCTTCATCATCAAGCAGTCGGTGGGCTCCAAGCCCACGCTGCTGGGCAACAAGCTCAAGTGTCCGTACCACAAGGGCGAGAACTACTTTGAAGTGGACATTGACATCTCGTCCAACTCGGTCGCAAACACGGTCGTGGGCATGGTGCAGGGCGTGACCAAGTCGCTCGTGGTTGACATGGCGTTCCTGCTCGAGTCACAGTCGGAGGAAGAACTCCCGGAGAACATTCTCGGCGCCGTGCGCCTGCAGAACATCTCGCTCGACAAGCCCCTGCGCGTGCCCAAGGATGGGCAACGGGGAGCAAGGACGAGTACACGTTCTGACGCGTTCACCGAGGCGCTCCGCGTGAACCTGCAACGACTGATCGTGTACGCCAAGTCGGCGGACACGAGCCTCCAGCGCGAGGTCGCCGAGAAGCTCGCGAACGAGGCAGTCAAGACAGACAGGTGCAGATTGTAGAGCTCGAGGGACTCAAGCTCCTGCTGCCGCTGACGCAGTCCAAGGACACGGAGGTGCAGCGGCTGGCGGCACACGCGCTCGCCAACCTCTCGGTCAACTCGGACAACCAGTCGCGCATGGCGACTGAGGGCGGCATCAACATGCTCATCGACCTGCTTGAGAGCACGAACGAGCACGTGCAGCGCCAGGCAGCGAAGGCGCTTGCGAACTTGGGCGTGAACGTTGACAACAAGGAGCGCATCGCGAAAGCTGGCGGCATCAAACCGCTCATCGCGCTCGCGAGCTCCAAGCAGATCGGCGTGGCGGTCGAAGCCATCGCGGCGCTGGCAAACCTGGCTGTCAACG------------------------------------------------------------------------------------------------------------------------------CCGAGAACAAGCAGCTGATCATCGATCTTGGGGGCGTGGACGCGCTGCAGGGGCTTGTGCGGTCGCCCAATGACCGAATATGCCAGCAAGCGACCCGCGCACTCGTGGCCGATGAAGAGTTATGCCGATCGACATCAGT---CAGCCGGTCACGTTCATGGCCGTGACAGCGATGGACCAAGACAGCGAGTACAG---CTCGTCGGGGATGGAAGTTAGCCCCGAGTTTGTGCGAAGCGAAGCGACAGCGTTGACTTGC------CACCGAGACGCTGTGACGCCCACGC-GGCTGGTACGGAAAGT--TGCGTTCTCGTCTATCGAAGAAGACTACCCGCTGTCTTTCACAAGCAACACCAGTGTGGCGTCGAGAATCACGCC------------GGTAATGAACC---CGATCCAAGAAGATTCGCACGAGGAGGAGGAC-------------ATGACTGTGTTGATTCGGTCCAACTCGGTCGAGAGAGAC---CCTGATGAGG---AT---CGCAAGAGCGCCCAAGCTCTACCAATACCGACCGACAAA--GACTGAAGAAGATGAGATGGAGATCCTTCGTTGGGACATTCAAGGGAACACGTTCAAGAAGTATTTCGCGCTGCAACAGCGAGAGCCGCAATGGGGGACTCTCGACGAGCGAGACAACGACTTTTCTTCCACCCTGGCAAGCGACGAGAAGCCATCATGATTGAGAAGAAGTTCCATGATTCTACCTACCGAGAGGGAATTGCGGAGGGGGACGCGTCCTGGTACAAGACCAGAAGAGAGCAGTTCGCCGAAGTCCACCGCGAGTTCATGGAAGAAACGCGATACAAGCAAGAGCTTATGCTTACCCGACTTGAGAAGAATTCAACGTTTTCGAAATTGCCCATTTTGGCGAACAGATTTCTCCTTGTCCGCCTTCTCGGGAAAGGAGGCAATGGAGAAGTGTGGAATGTCATCGATTTTGCTGACAACCAGCGCTCGTGTGCTTTGAAGCTGTCGACCTCAGTGAAGCACGCTCAGCAAGAGCACTTCACCCACATTGAAACATCCAC--ACATTGTGGGTGTTGGCGATACCGCGTATTTGATCGAGTATCGCCAGCAATTCTACACCGCTTTCACCGTTGACAGCGTGGAAACCGACTTGCAGCAGCTCATTGAGA---TCTACGGGTATCTGAACGAAGGTTCTGCTGTCAAAGTACTGTTTCAGCTTTTGCAATCGCTGTCGTATCTACACAATGGAGATTGCGCACTACGACCTGAAGCCGTCAAACATCTTGATCTCGAAGGATGAAAGCGTCAAGCTC------ACAGATTTTGATCTGGCTCGAGATGTGAACGAGCCAATCGCGTCTTCTTCGATCGGCACGTTGCGCTACCTGCCACCAGAGTGCTACCGCCCGAATTTTGGAGA---------TTGTCAAGGCACTGCCGAGAAAGCGGACATTTGGATGGTTGGCATTGTGTACTGCCTGATGATCTGGGGGAAACACCCGATCTGTAATGAAAAGGGCTCTCAGCAAGAGGCGAAAGAGAGCCTGGCGCGGTACAATGGA---CAGCTCACGTACTTCCGACCAATGTCCGATCTTTCCCGGTGGATCGTTCAAGGGTGCTTGCACCCGGACCCAAGATGCCGCCCGACCGCTTCCCAGCTGCTCCAGGCGCTGCAG

Pythium_ultimum TCGACTACCACTCGGAGGTTGCGCGCGAGCTCGTCGAATTCTTCCACACGTTCTATCGCTTCGGCGCGGCCTCGAACGTATTCGACCTGTTCTTGCACAACGTGCTCTTTGGCTCACTCGTGATCGGTGCCGTCGTGTTCGTCGCGCTCGCGATCATCGTCGTGCGTCGATGCTTCCTGCATATCCGCAGGCGTTTGTGCGGAAGGTGTCGATGGAGTCCAACTCGGTCGAGTTCTCGGCTGTGATCCTCATGACGATCATCGCGATCAGCTCGCTCAGCGGACTCTTGGGTGAAGCCGCGGTGTTGCCAGTGAAGTGATTCGCAACCGTGCCGATAATATGGTGGTTTCGTTTAGTGAAAGCGAGTTGCCGCCAGAGGCGTTCAAGTTGTCGGTTGAGTCACTGGCCTGATGCACTCATCCAAGGAACTTGTGAACAAGACAGACACGATGCTACCAAAGTACTACCGCGATATGGCCGATGAGTGGCAGTTCAGCTACTTTATGCTCAAGTCGTCGACCAACGCGGCGATTCTATCTGTAGCCCTTGCGAGTTTTCTTTTGATCTCGTCCGTCGGGTGGTCGATGGTCACGCCGTTGCGCATCTCCATTCTTGTGATCTTGAGTGTGGTGCCAGTCTCGCACACGTTGATCGGAGTGTATCTTTCGTCAACCATTATGACGTCGGATTTCTGCGCTGCTCCAATGAACGCTACCGTGTCGCTCTTGCATCCATGCCCGAAGAATGTCGTAGTCCTTTGATCGCGCCAGCTTCCAACTTACGCGGACAAGCATGGTGACGTTGGAAAGCGCATGAAAACGGAATTCCTCGACCCAATTGGAGCAGATTTGGGCGTCATGCTCGGGTACTTTATCGGCACGGTCTCGAATTGGGAGAGCTTCCAGCTCATCGAGACGTGGCGCTGGCCGTTTGTCGTGCAGTTTATCGCGATGTGTCCGATCATGATTGCAATTTTCTTTGTGCCCAAGAAGCACCTGATCATCCGCAGCGAAGCACCGGAATCTGCGCCACTGATGCGTGGCGCTCTCGTGAGTCGAAGCGGATTGCTGCGACACGAGGAAGAAGACGTGCTGCTGGCGAGTGCGAGCATGCAAACTACGGCGCGTTTGACACGAAGTGTACGAGCAGGGCTCGTTCTCGGAAGGCCTCTACGAGCTCGCGCACATCCCGATCTTCTGTCTGCTCGTCGCGGGCCTCACGTCGGTGTACTTTGTGGTCACGGGCGTGCAGTACTGGAGCACGATCTTCATGATCAAGTCGCTACATGCGTCCAAGTACCTTGTGAATGGCCTGTTCGTGATCGTCGCGGGCACGGGGCCGATCTTCGGCGTATTCTATGGCGGATGGCTCATCGACCGCTACGGCGGCTACATCGGCGTGCAGCAGCGCGCCAAGTCGCTCGGCATCTGCATGATCCTCGGCATTATCGCGTTCGGGATCTCTGCCGTGACGACCTTCTTCAACGACATCTACATCACGGCGGGTTTCCTCTGGCTGCTGCTGTTCTTCGGCGGATCGATCCTCCCGGCGCACCGCGCACTCGCGTCGTCGTTGTCGGTGATGGTGTTCAACTTGCTCGGCTACTCGCTGTCGCATGAGCGGCCTGGAAATGGTCCACGGGGCCAAGTGCTACGTGCCGGATGAGACCCACGTGTGGCTCGCGGCCGAGGTGATCCGCGAGGAGAACTCGGGCGACGGCAAGGTGCGCAAGATCTACGCCAAGGTGGAGCTCCCCCCCAGAGTATGAAGAGCGCATGATCGACATGAACGACAAGCAAATCAAGGCGCTCATAACGTCCACCAGCTCGAGTCGCTGCCGTACCAGAATGAAAACACGGGCGAGTACGGCATTGAAGATATGATCACGCTCAACTACCTCCACGAAGCTGCGATCTTGTTCAACGTCAAGACCCGTTTCCAGAAGAGTCTCCCGTACACGTACACGGGGGATATCTGTATCGCGGTGAATCCGTACCAGTGGCTTCCGGATCTCTATGCCGACGAGCAACACTTGCGCTACTTGAACCAACCAAAAGAAGAACTCCCACCCCACGTGTATGCGACCTCGGTCGCGTCCTACGACAACATGCGTCGCACGGAACGCAACCAGTCGATCTTGGTCAGTGGTGAATCTGGTGCCGGTAAAACGGAGACGACCAAGATTCTGATGAACCACTTGGCAACGATCGCCGGTGGTTTAAACAACTCGACCATTAAGAAGATTATTGAAGTGAATCCTCTGTTGGAATCTTTTGGTAACGCCAAGACGGTGCGTAACGACAACAGTTCTCGATTCGGCAAGTTCACACAATTGCAGTTTGATCGCAATGGAACACTGGTGGGTGCCAAGTGCCGCACGTACCTTTTGGAAAAGACGCGTGTGATCAACCATGAAGCCCCCGAGAGAAACTATCACATTTTCTACCAGTGGA---AGGAGACAGTTCTGCAGAGCTTCTGTTGGACTCGTCCAAGACGTACGCGTACACTGGTAACAACAGCGCTCAAAAGATCGAGGGCGTGTCGGATGCGAAGCACTTTGAGAAGACGCGCGTTGCGTTGGAGCTGATTGGTCTTACCCCAGACGCTCAGAAGCCGCTCTTTGAAGTCTTGGCGGGTGTGTTGCAACTCGGTGAGATCAGCTTGCTGTCGAACCCAGCGAATGACGAAGAGTCTTTGATTGCCGAGGGAGATGCCGGTGCAACCGCGGCCACGCAATTGCTCGGCGTTTCCTGGGAAGCGATTCAGAAAGCGCTTTGTTCGCGTACGATGCGCGCAGTGAATGATGTGTACAGTGTGCCATTGCGCAAGGAGCAAGCGATGGACTGCCGTGACGCGCTCGCGAAAGCTATTTATTCCAACGTCTTTGATTGGCTCGTGACGTCGATCAACCAGTCTCTTTCCGATGACGATAACATGTTCAACCACGTTGGTGTGTTGGATATCTTCGGTTTTGAGCACTTCAAGCACAACTCGTTCGAACAGTTTTGCATCAACTACGCCAATGAAAAGCTGCAGCAGAAGTTCACGCAAGATGTATTCAAGACGGTGCAAATCGAATACGAAGAAGAAGGCATTACGTGGAACCACATCGAATACGCGGACAACCAAGACGTGTTGACCGTGATTGAGTCGAAGATGGGTATCATTTCGCTCTTGAACGAAGAATTGATGCGTCCGAAGGGTAACGAAGAATCGTTCATGTCGAAGGTGGCGTCCTTGCACAAGGAA---GACATGGCGCACGTTATTGAATTCCCAAGAACGTCCCGCACTGAATTCTTGATTAAACACTACGCCGCGCCAGTCATGTACGATTCGGTTGGTTTCTTGGAAAAGCACAAGGACGCTCTGCTCCCAGACTTGTCGGAGTTGATGCGCGGATCCTCGAAGCCATTCATTTCGGCTTTGTTTGAACCGAAGCCTGTGCCACAATCGACGAAAGCG---TGGCGGCGGTGGTGCCCTGACGCTGTCCACTGTTGGTACTCAGTTCAAGGAAAGCTTGAGCGAGCTGATGACCACGATCCAGGCCACGAAGGTGCACTACGTTCGTTGCATCAAGCCAAACTCGATTAA---------GAGCCCAACTGCTATGGATCAACAGATGGTTGTGTCTCAATTGCGTTGTGCTGGTGTGATTGAAGCCATTCGTATCGCGCGTGCTGCGTATCCGAACCGTCTCCAGCACAAGGAGATCTTGGACAAGTTCTGGTTGTTTGTTCCTCGCCAAGGAGAAACTGCGCGTGAAAAGTGCGAGTTGCTGATGGAGAAGCTCAAGTTGTCGACTCCTGAGCAATACCAAATGGGTAAGTCTCGCGTGTACTTCCAACTTGGGGTCCTTGAAGAGCTCGAGGATCGCCGTAAGAAGTTCTTGGATGCCAAGGCGACGTACCTCCAGGGTGTCATGAAGACTTTTACACAGAGAATTAAATACGTTCGACAACTGGCTGCGATCATCAAGTTCTGTTATTCGTTGCGTTATTGCGATGCGAAGATACAACACTTTCATGACTGGTCTCGTCCAGGCCCAAGCACGCTGGAGAGGCATTGAGGGACGCAAGCACTATGCCGAAGTAAGAAGCAACCACTACGCTGTGATTATCCAGCGCTACGTGCGTGGGTTTGTTTTGCGTCACAAGTACACGAAATTGCGCGCGATGATCATTCGCGTGCAAGCCATGGTCCGTATGAAGATCCAACGACCAAAGTACCTTGCTGCGCTCCAAGAAGAGAAGTTGCAGGCCGATATGGTCTACCAACTGCAACAGTTGAAGGCCCGCCTTCAAGAAGAGCAAGAGCGCAATGCGAGATT------ACAAGAAGAGCGACTGAGTCTT-----ACCGCTTCGTTGCTGCGTCGAATGTTGTCATCGCCGATGCTGGTGGCATGATTGAGAAGCTCCAAGAAGAAAACTCGAAGTTCCGCTCGAAGAACGATGAGCTGAAGGCTGCCGTGAAGGCTCTGAAGGCCGAAGTTGAGAAGTACAAGTCGGAGAAGGAAATGAGCCACGCCGGTAACCACGTGAAGGTTCGTCAGTTGGAGGACACTGTGCGCGAAAAGGACAAGCGCATCGCTCAATTGGAGGCTGAAAACGCAAAGTTGACGGAACAGGTGCACGAGCTTCAGGCGAACGGCCACGTGACGTTCGAGAAGAAGGCAAAGGAGAAGCGCTCGATTTTCCGTACGCTCGGCTCGAAGAAGGAAAAGAAGTC---------------------TGAAGTGCTTCTCGACTCGCTGACTGGCGATGACGATCCTACTGGCTCGGATGTGCGTCCTTCGGA---GCGTCACTTCCCTAAGCTTC---CAAGCATGAGCATCCCGAAGGCGAAGTTCTGGAGCAAGTCCAAG---GCCGAAAAC---------------GGTGAGGGCCACGAGGACCGCGACTCCAACGGCGAGGATTCGCCAAACGGCCGCCACGTAACACTATCGTGAAGAGCATGGAAAGCGGTGTGACCGGTGCGATGACGAACCTCAAGGGCAAGATG---------GCAGCTGTGAAGACCATGTACAAGGCAAGCGATCGGGCAGGACCGACCAATCCGTGAATCGTTCAACTTGGATGCGCTTCCAGAGGTGTCGTTGCCTGTTGGATGGGAAGCCAAGGTGTCTCGATCGACGGGCCGCGTGTACTACGTGAACCGCAAGTTAGGTAAATCGCAGTTTGAGCGTCCGACGCTCGCGTCGCTGAA------GGCGCAGAAGCTCGCGCGTCAGAAGTCGTCCAGTACCACTGAAGGCCGTGGTTGCAGACTTTGGATGCGGCGATGCGCGCTTGGCCGAGAGCGTGCCAAACAAAGTGCACTCGTTCGACCTGGTGTCGCTCGACATTGCCGTGTACTGCCTCGCGCTCATGGGTACATCGATCCGCGAGTACATTCTCGAAGTGTACCGCGTGCTCAAGCCGAACGGCGTGCTCAAGGTCGCGGAAGTGAAAAGCCGCTTTGAGAACGAATCGCTGGGCGGATGGTGGCTATGTTCCTCGCGGTCGTGAACGACGTTATCGTGCTTCTGCGCAACCGCTCGCTCTTCGTCGCGAGCGCAAGCATGTCGTGATTGTCGGTGTGAAGACCGGCGAAGCGCTCGCGATGCCGTTCCCGGACATCGCGCGCGACATCAAGACGCTTCTCGGACACACGACGTCGATGCTCACGCAGCTGGCGATCAACCTCGTCGCTGCTTCTCACTGCGGACCGTGACGAGAAGATCCGCGTGTCAGCGTTCCCGCGCACGTCGCTCATCCAGAGCTACTGCTTGGGTCACGCTGCGAGTCTCACGGAAGTCGCTGCGAGCTTGTTGACGCCAGAACTCGCTGTCTCGACGTCGTTGGACAACACGATCAAGCTCTGGGGGATCGCATCAGGCGAACTCGCAGTCTCGGCGCAGTCGACCTCGTCGCCGTTGTGTTGAACAACCAGTGCGTGCGTCTGTTTGCGATCGTGATGCGCCATTCGTCAGCTTCTACAATGTCACGAAGAGACGAGGCAGCATTGGCAGTTTGTGCGCGACTTCCGCAAGCTGCAGAATGACCCGCCGTCGGGCGTCTCGGGCGCACCCATGGACAACAATATCATGCTCTGGCAGGCCGTCATCTTCGGCCCGGATGACACGCCGTGGGAAGGCGGTACTTTCAATCTGACGCTGGAATTCAGCGAAGATTACCCGAACAAAGCACCAACCGTGCGATTTATCACAAAGATGTTCCACCCAAACATCTACAACGACGGCCAGATCTGTCTCGATATTTTGCAGAACCAGTGGTCGCCGATCTACGATATCTCGGCGATCTTGACGTCGATCCAGTCGCTGCTCTGTGATCCGAACCCGAACTCACCTGCGAACTCGGAAGCCGCGCGCTTGTTCCAAGAGAATCGCCGCGAATACAATCGTCGCGTGCGCGAGATCGTTGAGCAAAGCTGGGTCGCGATCTATGGTGTCGGCCGCCAAGATGCGTGGCGCTGAGAACCGCATGAACGCTGGTCGTCCGTTCACCAACTGGTTGGACAACGTGTCGTCGGACCGTGGTCTCTGCGGTGGTATCAACTCGGGTATTGCCAAGGCCGTGCGCAAGCACCATCGGCGGAGCTTGTGATTATCGGTGACAAGGGCCGTGCGCAATTGCGTCGTGAATTTGCTCCTCAGATCCGTGCCAACGTGACGGAAACCTTCGCGACCCCACCAAACTTCACGGTCGCCAGTGCCATTGCCGAAGCGATGGAGAACGAGAGGAGAGTTCGCGTATGTCGGCCATGGAAAATGCCACGACGAACGCGCAGGATCTCACGTTGACGCTCGTGTACAACAAGGCGCGTCAATCCCGTATTACCACGGAATTGATTGAAATTATTTCCGGTGCCGCGTCGCTCGACCCTCACGGGCACCGTCGTGGACATCCAGGACGCCGCGCAGTTCGACGAGCTCACGGCGCGCGAGAGCACGCTTAGCATCTCGTTCTTCTGGGCCGACTTTCAGCTCTGCCGCCCAAACGGCCAGATCGACGTGGGGCAAGTCGGTGCTTGAGAAGCTCGAGGGCGCCAACGTCGCGGAGCTCGCCAAGCGCGTCTCGAATACCGCTTGAAGAAGCTCATCAACGCGTCGCCTGTGATGCTGTTCATGAAGGGCAACCCAACGGATCCAAAGTGCGGCTTCAGTCGCCAAATGGTGGCGCTCCTGAACGAGGAGAGCATCCAGTACGGCACGTTTGATATCCTCAACGACGAAGAGGTGCGTCAAGGACTGAAGGTGTTTTCGAACTGGCCAACGTTCCCGCAACTATACGTGAACGGCGCCCTCATCGGCGGCCTCGATATCCTCAAGGAAATGAAGTCGGAAGGCTCGATTGTCGAAGAGCTCGGCGTGGAAGAAGCCGAAGCCGCGTTCAACGAGAGTCTGCGTGCGCTCGTCAACTCGGCGCCCGTGCTGCTCTTCATGAAGGTTTGATATCCTGTATGGAACTATGAGCGACCGAACCATGAGATCTCGAAGCTGGTGAGCTTTGCACCGAATTCATTCCGACAAGACATGCGCCGAGAGCATTGTCTGGAGTCCGGAATCGCTTGGCGATCGCATTTCGTGACCCGATGGATGGAAGAGGCGAGTTTTGTATCGTCCGCCTGCCCGAGTGTCGTCGACGACTTCAACGCAGCATTCCCGTGGCATCTCTGCGATCGAGTGGAAAGTGGAAGATTCTGCACCGCGAGCATCGTTCCGAAGTCAAGACGATCTGCGTGCATTCGCAACGACAGGCAGTCTTCACGGGCAGTTTTGATGGACAAGTGATTCGGTACGATATGGGGAAGTTTTTGCCGCATACTGTGATGGAGCGTCGCAAGCCGATTTCCAAGATCAATGCGGTGCTCGAACATCCGCATAACCCAAACATCCTGCTGGTCAGCAGCGTGGAGCAGGGAGCATAGCATTTTGCTGCACGACCTCAAACGCTACGTCAGCAGCCTGACCGCCAAGTCGATGAGCCAGTACATAGTGCCGCGCTGGTCTCCAGCGGGGATGCACGTGTCCTGCGGAAGCAAATGCGCTACTGCCACGGCAGCTTCTCGACGCCGACGTCGGCCACGATCGGTGCATCGTTTTTGCAGAAGCGCATCATTGTGGGCGGGAAGATGACGCTGCAGATCTGGGACACGGCCGGACAGGAGCGCTTCCGCTCCATGGCGCCCATGTACTACCGCAACGCCAAGGCTGCGATCCTCGTCTTCGACCTGCTCAACGAAGCGACCTTCGAGAAGATCAAAGAGTGGCTGCACCTGCGCCACCACGTCGCCGACGACATGTGGGAAACAAGTGCGACGTTTCAATTTCGCCAAGGCGCAGCAGTTTGCACAGGAGATCGGCGCGCTGGTGTTCCGCACGAGCGCCAAGACGGGCGACGGCGTGCAGAACCTCTTTGAGAGCGTAGCCGTGCAGCTGCTGAAGCGCCACGAAGAGGAGGAACGCGAGCGTTCTACGTCGTGTACGTACCCGCGGACGCCGAGAAAGAGCTGGAGGAGTGGCAGGTGGCGCTGCCCGAGGACAAGGAGGCCCAGTTGGGCTGCCTCACGGACCGCCTGCGCGCGCATTTCAAGCAAAATGGCATCGGAGGCCAAGCAGAGATCTTCAAGCAACAACTGCTGAGTCAGTTGCCCAAGGGCACGCAGATGACGGACGAGATGCTGCAAATGATGCATGGACAGTCTCGTGGACTCGTTCCCGCTCATGACCAACACGCGCGCTGTCAGTCACATTCGAAGTGCGCGGTGACGCGTTCATTGCACGTGTCTTTGACAGACGACTTTGAGCGCATGGATTTTCGCTTGAGCGAGGTCACGATGGACGCCGACTGGATCAAGATCGGCAAAGAGCAGAGGTGACTCAGTCGCTCGAGGAGATCCTGGCGCTCGGCCAGGGGCTCGACGACGAGGACTACAAGGCCGAGCTCGAGGTGCTGCAGAATGACCCAAACTCGAACCTGTACTCGGCCGTGACGTTCGAGGAACTGAACCTGCCGGAATTGCTGCTCAAGGGCGTGTACTCGATGAAGTTCTCCAAGCCGTCGAAGATCCAATCGGTTGCGCTGCCGTTGATCCTCGCCAACCCGCCAGAGAACTTGATCGGTCAGGCGCAAAGTGGTATCCCACGCGCGAGCTCGTGCGTCAGATCATGGCGGTCGTGAACGCCATGGGGCGCTTGTTGAGACGTTCCTCGCGATCCCGGGCGTCGATGTCGCCAAGGGCGAGCAGGTGCGCGCGCAGGTCGTGGTCGGTACTCCAGGCCGCGTCGAGGGTCTGATCAAGAAGAAGCAGCTCGACACGCGCCAGGTCAAGATCTTTGTTCTGGACGAAGCTGACGTGATGGTGTCGGAGGACGGAATGCGCGATCGCTCGGTCGCGATCAAGAAGATGATCAAGAATCGCGACTGCCAGTACCTGCTCTTTTCTGCAACGTACGCCGATGAGGTGCGCGATTTCGCGCAGCGCATGGTGCCGGACCACAATATCATCACGGTGAAGAAGGAGAAGCTCACGCTCGATGGCATCAAGCAGTTCTGGATCGACTGTGGCTCCAAGGGTAACAAGTTCAAGGTGCTGTCGGACATTTTCGCCGTGCTCACGATCGGCAAGTGCGTCATCTTCGTGCAATCGCGCGACACGTGTAAGGAACTGACGCGACGCATGCGCGAAGACGGCCACTCGGTCGGCATCCTCCACGGCGCCGATATGGCGCGCGAAGTGCGTGATCAGGTCATTGACGAGTTCCGCGCCGGAACGACAAACGTGCTGATCACGACCAACGTGTTGGCGCGTGGTATCGATGTCGCTGGCGTCTCGCTCGTCGTCAACTTTGATATTCCGCTCACCAAGGACCAGCAACCGGACCCGGAGACCTACTTGCACCGTATCGGTCGCACCGGTCGATTCGGCCGCAAGGGCTGCGCAATCAACTTTGTGCACGACGACCGAAGCAAGAAGAACCTGGCGGAGATCGAGGCGTACTACTCGCGCGAGATCGTCCAGGCGCCCGCGAACGACATTGAAGAACTGGAAAAGCTGCTCGCATGGGGGGCAGCAAGCGCCGCCTGGCCAAGTTCGGCAAGGAGCAGCGCAAGGGCATGAGCATCTACCCGCGCGAGCCCAAAAAGAAGGTCAAGGGCAAGGACAAGACGTACTACTACCTTAAGGATGTGATGTACCTGGCCCACGAGCCGATCCTCGAAAAATTCCGCGAGTTCAAGACGTTCATGAAGAAGGTCAAGAAGGCCATGGGTAAGCGCGACTTTGAGGACGCCAAGCGCAAGCACGACAACCGACCAACGTACTCGCTCGACCACATCGTCAAGGAGCGTTACCCGCGCTTCGTGGACGCGCTCGGCGACTTGGACGACGCGCTCTGTGGAACAACTACGTCGTGACGTCGCGGTCGCTGACCAAGGTGTTTGTGAGCATCAAGGGCGTGTACTACCAGGCGAACGTGCAGGGCCAGACCGTGACGTGGCTTGTGCCGCACCAGTTCACGCCGACGATGGACAAGCGCGTCGATGTGCGCGTGATGCTCACGTTCCTCGAGTTCTACGAAGTGCTGCTTAAGTTTGTCAACTACCAGTTGTACACGGAACTGGGCATCGCGTACCCACCAAAGATCGATTTGCAATTGGACCAGGCCGGTGCGCAACTCGCGGCTCTGCAGTTAGCAGCAGAAGGAGTCCGAGAAGCGCATCAAGTTACAGCGAGCAGCGCAAGGAAGAGAGCGAAAAGAGTCTGTTTAGCGGCTTGTATTTTTACCTGTCGCGCGAAGTGCCGTCCGCTTGTTTGGAGCTGGTGCTGCGCTCGCACGGCGCGCAGATCGGCTGGGAAGGCGCGGGTTCGCCGTACACGGAGAAGAGCGAACGCATCACACATCATATTATGGATCGTCCACACCAAGGCCACCGATACTTCAACCGCGAGTATGTGCAGCCCCAGTGGGTGTTTGACAGTATCAACAACGGCATGTTGCTGCCGCTGGCGCGCTACCGCAAGCAGCTCAACAAGCTCAAGTCGGCGACCCAGGTGCTCCGTGAAGTGGGCGACTTGGACAACGGGGACAACGTGTAGAGGCGTATGCCGCCGGTATCAAGGCCGAATTGGACGGCAACAAGAAGTCGAAGAAGGCGAAGCAAGAGGAAGAAGAGGAAGAGCTTCACGAACTGGCCAAGAGTATGATGTCGAAGAAGTCCAAGCGCTTATACGATCGCATGCAGTTCGGCCTGGGCAAGAAGGCCGACAAGATTCAGAAGCTCGAGGACAAGCGCGCCATGTTAGAAGCAAGACCAAGAAGCTCGTGACCGGTGCGCTCTCGGGCATGATCTCCGCGCTGGCGTTCCAGCCGCTGGGGGGATGCGCGGGCGAGGCACGTCATCAACGCTGATTCGTGTGGCTGGTGGTGCCGGGTTGTACTTCACGACACTCGACCACTGCATGACTATGTTCCCGAACTCGGCCATCAACACGTTCCTCTCGGGTGCATTTGCACGGACGTTTGCGGGCTGCGTGATGTCCCCAGTTACAATCGTGAAAGCGCGCATGGAAGGCACCTGGTACATACCGGAGCAAAGGGACGTTCCGTTCTCAGGACTCTATGTGCTGATTTACACTGCACCCAGCAGATGTAGTGAAGACGCGCATGCATGCGACAGACTGTGGCGAAGATACTCAGCGAGGAAGGTTGGCGAGGGTTCAAGAAGGGGATCGCGCCGCGGGTGCTGAAGCGAACGCTCTCGACTGCTGTCACGTGGACCATCTATGAGCTATTCGTTCGACCCGTCGGGCCTCGGCCACGCCAAGGACGCGTTCCAGATCGCCAAGGAGACCGAGCGCACCAAGCAGCTCGAGCACCAGGCCAAGGTGAAGGAGAACGAAGCGCTCTACAAGCAGTACGAGATTGTACGTGTGCAGAAAGAGGGCGAGGAGCGCCGCAAGACGCTGGAGGAAGAGACGCGCCAGCACCAGCAGCGCGCGCAGTACCAAGATCAGCTCAAGCGCAAGCAGTATGCTGACCAGTTGGCGTACCAGAAGCAAATGAAGGAACAGGAGCTCAAGAAGCAGGAAGAGATGCTCGCGCGCCAGGAGAGTTCGCGCCGCAAGACGCTCGAGTACGAAGCCGAATTGCGTCAAAAGACCGAACTCTCCAAGGTCGCGGCCGAAGTGGAAGGCAAGATCAAGCAAGAGCGTTTGAACCACGACTTGCACTTGGAAGAAGCGCGTGTGCGCGCCAAGGAATACCGTGAGACCGTGATGGAGGGTATCAAGCTCGCTGGTTCCACGATCGGTTCCGGCGTCATGACGTTTGTCGATGACAAGGAGAAGCTCACGGCCACCGTTGCGTCGCTCACGGCGCTTGCCCTCGGTGTGTACACTGCCAAGACGTCGACGGGTGTTGCGGGCAAGTACATCGAAGCGCGTCTCGGCAAGCCGTCGCTTGTGCGTGAGACTTCGCGCCGCACGCTTCCGCAGATCGTCGCAAACCCGATCCCATCGATCAAGCGCGTGCTTCGTCTGCAAAAGGCGACCGACGCCCTCGAAGGTGTCGTCTTGGAGCCAAAGCTCGACCAGCGCTTGCGCAGCGTCGCGGTGTCGACGTTCAACACGAAGAAGAACCGCGCGCCATTCCGCCACTTGCTACTCCACGGTCCTCCAGGTACGGGTAAGACGCTGTTCGCCAAGGCGCTCGCGCGCCACTCGGGTCTCGAATACGCGATCTTGACCGGTGGTGATGTGGCTCCGCTCGGCCGCGAAGGTGTGACGGAGATCCACAAGCTGTTCGATTGGGCGTCGCACAGCCGTCGCGGTCTGCTGCTGTTCGTGGATGAAGCCGATGCGTTCTTGCAGAAGCGTAGCAACACCGTCATGAGTGAAGACATGCGCAATGCGCTGAATGCGTTCCTGTACCGCACGGGTGAGGCGAGCGACAAGTTCATGATCGTGTTCGCGAGTAACCAACCGGAGCAGTTCGATTGGGCCATTAACGATCGTATCGATGAGATGGTCGAGTTTGCGCTGCCGGGCCACGCTGAGCGCGTGCGCATGCTTAAGCAGTACTTCGACAACTACATCCGCACGCCGAAGAACTCGTCGGCCAAGAAGATCTACACGAGGGCGTCATCTTCACGATCGACCCCATTGCGAGCTTCCTCATCCTAAGACGCGTATGTTCCAGATCGACGCGCTCAAGGACGTCTCGTCCGAAGCACAAGCGATCTTTGACGCTCTCAACAAAAGCCCATGCGTATCTACTGGATCATCTTCGGCGTCGTGCTTGCGAACGGCGCGTACACGTACTTTGCCGGCAAAGATGAGTCGTATCTGGTGGAGAAACTGCAGAAAAACGTGCCGTCGGAGTTCCGCATCTCGGAGAAGTGGGACAAGTGCATTGAGAACTTCACGCTACACTTCGCGGCGGGTCTCGTGGCCGGCGGTCTCACCTCCGTTGTCCTGGCGCGCTCGGGTGCTGGTCGTGGTGTGTTGACGGGCTTCGGTGTGCGGGCGCCGGCTCGAGCTGGACGACGTGCCAGCTCGCGTTTAAGGGCGACAGGCAGGCGGCCCTGGACAAGTCGGACAAGCTGGTCGACGAGATGGCTGCGTTCCCGACCCAGACGCTCGTGAGCTACACGGCGGCGTCGCCTGTGCAGCAAAAGCTGCTCGCGTTCTATCAACAGCTTCTACGACGCCGCGGCCGTCGCCGACTTCGAGGTGTACCTCGCCGAGCAGATCAGCGCGTCGACGATCGACATCGACGCCAACTTGGCGCTTCTCAAGCTTTACTCGCTATTCCCTGAACACCTGGACGTCGACAGCCAAGGTGCTCGTCAAGGGCATCCAGGCGCTGCCATCCACGTTCTTCACGGGCGTCGACCGTCGTGCCGGAAAGCATCCGTGAGGACGCGAATGTGAAGGCTGTGCTTGAAGCTGGTTTCCTCTTGCAGTCGTGCTTGTTTGAAGAGTTCTGGCAGGTGGACTTGACGTACGCCAAGAGCGTCGCGGGCTTCACGGACGCCGCGCGCGCGTTCATCCTCGACGCGGTGCGCAAGAGCCACAGCGCCATCTCGACGAGCGTGCTGAAGGCGAAGCTCAACATCTCGGACAAGGAAGTGCAGGACGTCGTGGCGGAGAAGTGGAGGAGGGCGACGTGATCAAGATCACGCTGAACGAAGACAACCAAGTGCGACCAAAGAAGATCCAAGAGAACATTGAGTTCGAGGACGTGCTCAAGGTCATCCACACCCTCTCTAGATCGAACGTGTGCGCGAGCGGCGGCGGCGTCACGGACGCGCAGGAAGTGTGCGACGCGCTCGCCAAGCACCTGCGTCTCGAGGGCGGCAACCAGGTGCTCCCGAGCTCCACGGGTGTCATCGGCTGGCGCATCCCCGTCGATGTCATGATCGAGAACATCCCGCAATTGGTGGAGAACCTGCAGTCGGAATCCGTGCTGCCTGTCGCGGAAGGAATCATGACCACCGACTTGTACCCGAAGGTCCGCTCGATGGACGTCTGTGGCGGCCGCATCACCGGTATCGCCAAGGGCGCAGGTATGATCGAGCCTGACATGGCGACGATGCTCTCGTACATCCTCACTGATCTGTCGGTGCCTCGCGATGTGCTCCGTCAGTGCCTCTTGGAGGTCGTCGGCGACACGTACAACTCGATGAGCATCGACACGGACCAGAGCACGTCGGACACAGTGTCGATCGTCTCGTCGGACCAGATCCCCTTCGACATGGC---CAAGCTGCCCGAGTTTAAGGCCGCGCTTCAGGAGATCTGCCAAGGCCTGTGCGAGGACATCGTTCGCAACGGCGAAGGTGCGCACCATGTCATGCGTGTGGTCGTGCGCGGCGCGCAGTCGGTCGAGATGGCCAAGGGCGTGGGCAAGTCAGTCGTGAACTCACCGCTGCTCAAGTGCGCCGTCGCAGGCAACGACCCGAACGTCGGCCGCCTGGTCATGGCCGTCGGCAAGTACATGGGCAAACACCACAAGGACGCAGACATTACGCGCCGCATGAAGATCCGCGTCGGCGGCATCTCAATCTTCGAGAACGGCGAATTCGTGCTCAATAGCGACATTGAGCAGCTGCTCGTGGATCACATGCGCCAGGCGCAGCTCATCGAACCAACTCGTGGTGGCCTCGACGTGACGTCCCACGACTACCCACCGCACGGCAAATTCGTTGAGATTGATATCGACTTGGGCATCGGCAATGAACGCGCTCGCGTGCTCGGCATCGATCTCACGCACGAGTACGTCGCGATCAACGCCGACTACCGTTCGTAATGCCGCC---GAAGAAGGGAGGGACGGCCGCCGCGTACCTGGCGTCGCTCGGCGTTGACGCTTC---GGGCGGTGCCGACAAGAAGGACAAT------AAAAAGAAGAAGAAGGGCAAGAAGAAGCCCGAAAGAAGCCGATCTCAGCGGCCGCCAAGGCGCTCG-CCGAGCGCCAAGCCGCGCTGAAGGCCGAACAGGAGCGCATCGAGCAGGAGCGACTGGAGCAGGAGCGCATTGAACGCGAAGAAGAGGAAGCTCGTCGTAAACAGGAAGAAATTGAGGAGGAGATCCGCCGCAAGAAGAAGGAAGCCAAGGCCGCGAAGATCGAGCGTCAGAAGAAGGAAGGCACGTACATGACGAAGGCCCAGAAGCAGAAGGCTCAGCGTGCGCAGGCGGCGCTCGAGGCGTTGCAACAAGCGGGTCTTGTGCCGGACGCTGTCTCG---GAAGAGAAGAAGAAGGTGGTCTACTCGAACAACAGGAAGAAGAAGCAGCAGCCAACGACGTCGAGCCCGAGGAAGCCGAAGCGCCTGATGCGTGGGACGACGAGGACTGGGAAACGAGCGATGTGGTCGCGTCCTTGGAAGACAAATTAGCAAAAGAAGGAGCAAGAGCGTCTTGTGCAGCAGGGTATCGCGCTGAAGAAGCGCCAAGAGGAGATGGAAGAGCAGGCGCGTATTAATGCGGAAGAAGAGAAGATCCGCTCGAAGGCGCAAATGGAAGCCGAGCGCAAGAAGAAGGAAGCCGCCGACCGCCGTCGCGTGCGTGAAGAAGAGGCGCTGGCCAATGTCTCGATGGACAACTTGCGTTCCCCGATTTGTTGTATCATGGGTCACGTCGATACCGGTAAGACCAAGTTGTTGGATAACATCCGTAAGACCAAGGTGCAGGACGGTGAAGCTGGTGGTATTACGCAGCAGATTGGTGCGACGTTTTTCCCTGTCGAGGCCATTAAGCAGAAGACCGCTCGCCTGGCCAAGACCATGAAATTGGACTACAAGCTACCGGGTCTGCTCGTCATCGATACCCCAGGTCACGAGTCGTTCACGAACTTGCGTTCGCGTGGTTCGTCGTTGTGTGATATCGCCATTTTGGTCGTTGATATCATGCACGGACTGGAACCGCAGACGTTGGAGTCGCTTCGTCTTTTGCGTCAGAAGAAGGCGCCATTCGTCGTCGCGTTGAACAAGATCGATCGTTGTTACGGATGGAAGACTATGCCTGACATGCCGGTCCAGGAAGCGCTCAAGCACCAGAACGAGCACGTGCTCCGCGAATTTGAGGACCGTACTGCCAAGATTATGGTCGAGTTTGCGGAACAGAAGATCAATGCCGAGATTTACTGGCGCAACAAGGACCTCGGTCACACGGTGTCGCTGATCCCAACGAGTGCTATCTCGGGTGAAGGTGTCCCGGATCTGTTGATGATGATCACGCGTCTGACCCAGGAACGCATGGCCAAGTCGCTCGCGTTCGTCGACATTTTGCAATGTACGGTGTTGGAAGTCAAGGTGATTGAAGGTCTCGGTACGACCATCGATACGATTTTGGTCAACGGTACGTTGAACGAAGGCGATACAATCGTCGTGTGTACACTGGATGGTCCCGTGGTGACGACGATTCGATCCATATTGACACCGCACCCGATGAAGGAAATCCGTATCAAGGGCGAGTACATCCACCATCAGACCATGAAGGCCGCGATGGGTGTCAAGATCGCCGCCCACGGTCTTGAAAAGGCTGTCGCCGGCACGCAGATCCACGTCGTTGGCCCCGATGACGATGTCGAAGAGCTGAAGGATGCCGTCATGTCCGATTTGACGGGCATCTTGGACTCAGTCAAGGCCACGAAGCGTGGTGTCATGGTGCAGGCGTCGACACTTGGTGCCTTGGAAGCGCTGTTGGAGTTCTTACGTACGTGTGACCCACCGATCCCCGTGGCGTGCGTGAACCTCGGCCCCGTGCACAAGAAGGATGTGATGCGCGCGTCCGTGCAGTTGGAGCACCAGAAGGAGTTTGCGACCATTTTGGCGTTCGATGTCAAGGTGCACAACGACGCGACTGAGCTTTCGCAGGAACTCGGCGTGCGCATCTTCACGGCAGATATTATTTATCACTTGTTTGATCACTTCACGGCATACATGGACAACTTCCGAGAAGCACGTCGTGAAGAGTTTGCCGACGTCGCGGTGTTCCCGTGCGTGCTCAAGATTCTGCCAAACTGCGTCTTCAACAAGAAGGATCCGATCATTTTGGGTGTGGACGTTGAAGAAGGCATTTTGAAGGTGGGCACGCCGCTCGTTGTGCCATCGGCCGGTG---GCGTGTTGGTCGGTAAAGTGGGCTCGATCGAGCGTGAACACAAGGAAGTGGACCGTGCCAAGAAGGGTGCGTCCGTCGCCGTGCGAATCGACAACGAAGGCAGCATCACGTACGGCCGCCAGTTCGACCACAAGAACAAGCTCGTGTCCAAACTCACGCGTGCAAGTATCGACGCGCTGAAGGAGAACTTCCGCAACGACCTGCAGAAGGAGGACTGGCAGTTGGTGATCCGCCTGAAGAAGGTCTTTGACATCATCATGGAGGGCTGGCTCACGAAGCGCCGCGACCACATGAACCTCATCTGGCTGGAGCGTTACTTTGTGCTGGATGGGAACCAGATGCGGTACTACCGGAAGCGTGGCGATCCGGCGCCGCGCGGCACGTACGTACTCACGGACGGCTGCCTCGTGAGCAACGTGTTCAACTCGGAGGAGAAGCACAAGCACCACGGGCCCGTGTGGATGTTCCGCATCACGTGAGAAGTCCTACAAGAAGGACCGGTTCCTCGACCTCGGCGCCAAGACCGAGGAGAACGCGCGCGAGTGGAAGAAGGCGATTGAGAACGCCGTGCGCTCGCTGCGCGAGCTCGTGCTGCACCAGCGCAAGCTCAAGACCGACGCGGCCAT---CTACCCGACGTTCAGCTCG------GCTGTGCCGCGTCTGCTCAAGGCCGATGAGGACACGGTCTCGCTGTTCAACCTTGATGCCGACAACGTCAAAGCCGAGCAGGGCTGGTGGCTCGCGCGCGTTGAGGAAGGATTGCGCATCATGCAGGAGTGTCCATTAGGTCCGAACGTCGCGGCGTCGCTGTATGCGCAGCATGCGAACACAGCGAATGGCCTCATCTTCTCGTCGCTCATCAGCGCCGTGCTCACGTTCAGCATCATGTACTCGTTCGTGACCAACATGCTGTTCTGCTTCACGACGGCGGCGGCCGCGGTCGCCGTGTGCTGCGTCGCGATCACGCCGACGCATGCGATCGAGATCCCGAGCTTCCGCGTCTCACAGATCGTCAACGGCTCGCCGACCGAGGTCTTCCGCTTGCTCATGAACTCGAAGCGGTACCAGCGCTGGGACCCGTCAATTGCGTCCATGCGCGTGATCCAGGAGATGGACGACCACGCCGACATCGTTTACATCACGCAGCGCCCGACATACTTGTGGCCACTGTGGCAGAAGCCGCGCGACCTCGTGCTCATGCGCTACTGGCGCCGCGAAGAAGACGGCTCGTACTTTGTCATGTACCAGAGCATCGAACACCCGGAGTGCCGTGTGCGCCATAACTACGTGCGCGCAACAGTGTTGGGCGGTGGCTTCGTGATTGCACCGCAAAAGGTACCGAGT---GGCGCCATCCGCTCGCATGTCACGTACGTGCTGCGCTACGACCCGGGCGGCTG---GTCGCGCATCTATCACCAGCTCGGCATGGACGTCGACCTCGTGATGCCGATGCTGCGCAGCGTCGTGGGCATCCGTGACGAAATGAGTGGCGTAGACTTCATCACGCCGAATGTCACGGTGGACTCAGCTGCGAGCGAGTGCTGGAGGAAAGGAAGCGTCGA---TGCCTCAGGGCTTGTCACGGGCGTCACGCGCTTCAAG---ACCGGACTGCCCGACAAGATGTGGGCCGAGCCTGACGCTTCGCTCTTCCAGGTTCGAGGCACCAACTACCTGACCGACAAGAAGAAGGTGCCGTCGGCGACGTCCAAGTTCCACCTTGTGGGCGTCGACCTGCTCTCGTTCGAGAGCGCGAGCGACCGGTACAACATCTCGGCGCGCAGCGACAGCGTCGGCCGCACGTC---GAGCAAGTTCAGCTTCGTGATCAACATGATCATCCCGGGACCCGAGAACATCTGCATGGTGTTCTACTTCCACCCGGCCAAGGACAACATCTTCGAGGACGGCTCGCCGTTCTCGGAGCTGCTCAACGACTTTATCGACGGCGACGACGCGTTCCGGAATTCGCGGTTCAAGCTCATCCCGACGGTCGTCGAAGGCAGCTTCATCATCAAGCAGTCGGTTGGCTCCAAGCCCACGCTGCTCGGCAACAAGCTCAAGTGTCCGTACCACAAGGGCGACAACTACTTTGAGGTGGACATCGACATTTCGTCCAACTCGGTCGCCAACACCGTCGTGGGCATGGTACAGGGAGTGACCAAGTCACTGGTCGTAGACATGGCCTTCCTGCTCGAGTCGCAGTCCGAGGAAGAGCTTCCAGAGAACCTTCTCGGCGCCGTGCGGCTCCAGAACGTGTCGCTCGACAAGCCCACGCGCGTGCCGCGTGATGGGTAACGGGGAGCAAGGACGAGTTCACGTTCTGACGCGTTCACGGAGGCGCTGCGCGTGAACCTGCAGCGGCTGATCGTGTACGCCAAGTCGGCCGACACGAGTCTCCAGCGCGAGGTTGCCGAGAAACTCGCCAACGAGGCCGTCAAGACCGGCAGGTGCAGATCGTGGAGCTCGAGGGGCTCAAGCTGCTGCTGCCGCTGACGCAGTCCAAGGACACGGAAGTGCAGCGGCTGGCAGCACACGCGCTCGCAAACCTCTCGGTCAACTCGGACAACCAGTCGAGAATGGCGACCGAGGGTGGCATCGACATGCTGATCGACCTGCTGCAGAGCCCGAACGAGCACGTGCAGCGTCAAGCGGCCAAGGCGCTCGCCAACCTGGGCGTCAATGTCGACAACAAGGAGCGCATTGCCAAGGCCGGCGGCATCAAACCGCTGATCGACCTCGCGAGCTCCAAGCAAATCGGCGTCGCGGTCGAAGCCATCGCGGCGCTCGCCAACTTGGCCGTCAATG---ATGCAAACGAAGTCGAGATCGCGCGAGAAGGCGGGCTGAAGCCGATCGTCGATGGCGCGCGCTCGGAATCCGTCGAGCTCCAGTCCCAAGTCGCGCGTGCTTTGCGCAACCTCTCGGTCAATCCCGAGAACAAGCAGCTGATCATCGATCTTGGCGGTGTCGATGCGCTCCAAACGCTCGTGCGGTCCCCAAACGACCGGATATGCCAGCAAGCCACGCGAGCGCTTGTGGTCGAGGAAGAGGTGTTCCGGTCGACACCATCCAGTTTGTGGTCGCACCCTCGTCAGTGTCGTCGATGGAGCAAGATGACCAGTTTAA------CGCGTCAATGGATTACGACACCGAGTATGGAGCCAACTCGGTGACCGAAGCGACGTTC---------CGGGATTCAACTTCGACATCCT-ATTTCGTGCGGAAGGT--CGAGTTCTCTTCGATCGCAGACGACGTACCGTTATCGACGACGAGCAAGAGCAGTGTGGGGCAGAAAATCACGCC------------ATTGCTAAACC---CTATCCAAGAAGACACGC---AGCTGGATGAC-------------CAGACAGTGCTAAACCGCTCCAAGACAATTGAACGAGAG---TCTGACG------AT---CGCCAAAGCACCAACAGCATACCAGTACCTTGCTACGAA--AACTGAAGAAGACGAAATGGAGCACATTCGTTGGGAGGTTCAGCACACGGTGTTTCAACGATACTACGACCTGACGCAGAGAGAACCACAATGGGGATCGATTGACGTCCGAGACGG--------------------------------------------------------------------------------------------------------------------------------------------------------------------------------GGAGTTGCCGACGGAGATGTATCCTGGCTCTCCGTTTTCCAAGTTCCCAACGCTCGGAAAGCGCTTTATTTTGCTTCAGCTTCTTGGAAAAGGCGGTAACGGAGAAGTTTGGAACGTGATTGACTACGCTGACAATCAGCACAACTGTGCCTTGAAGCTATCCACTTCACTCAAGCACGCGCAGCGAGAACATCTCACACACATTGAAGCATGAAA--ATATTGTTGCCGTTGGTGATACCGCATACTCCATTGAATACCGTCAACAGTTCTACACAGCTTTCACCGTTGAAAGCGTGAAAACGGATCTTCAGCAGCTTATCGAGA---TGTATGAGTATTTTGACGAGAGCTTAGCTCTTAAAGTGCTATTTCAGCTCTTGAATGCGCTCACTTATCTGCACGGTGAAAATCGCGCATTACGATTTAAAGCCATCGAACATTTTGATCACCGAGGACGAGTGCGTCAAGCTG------ACCGATTTCGATTTGGCTCGGCACATTAACGAGCCAATTGCATCTTCTTCGGTCGGGACGTTGCGCTACTTGCCTCCAGAATGCTACCGACCAAATTTCGGAGA---------TTGCGAAGGCACTGCTGAAAAAGCTGACATTTGGATGGTTGGCATTGTGTATTGCTTGATGCTTTGGGGCAAGCACCCGATCTGTCATGACAAGACCAGTCACGAAGAGGCAAAAGCGACTCTCGCGAGATACAATGGC---CAGATCACATATGTCCGGCAAGTGTCGGATCTCTCTCGATGGATCATCCATGGCTGCTTACACCCCGACCCAAGATGCCGACCCACTGCATTTCAGCTCCATCAATCACTGCAG

Pythium_vexans TGGACTACCACAAGGAGGTGGCGCGCGAGCTCGTAGACTTTTTCCACTCGTTCTACCGCTTCGGCGCGGCCACGAGCGCCTTCAACCTGTTCCTGCACAACCTGCTCTTCGCGTCGCTCGCCATCGGCGCCGTCGTCTTTGTCGTGCTCTGCGTCATCGTCGTGCGCCGCTGCATCCTGAACATCCGCAGGCGTATGCGCGCCGTGCGTCGAGCGAGTCGAACGCGATCGAGATCACGGCCGTGATCCTGCTGTCCTTCCTGGCGATCAGCTCGCTGAGCGGTCTGCTTGGCGAGGCGGCCTGAAGGCAAGCGAGGCCGTCCGGCTCACGGCCGACAACCTCGTCATGTCGTTCAACGAGAGCGATTTGCCGGAGCAGGCGTTCAAGTTGTCTGTCGAGAGTCTTGGCTGCTGCACTCGGCCAAGGACTGGCACAACGAGACGGACGCGCTGCTCCCGAAGAACTACTCGGAGATGGCGCAGGACTGGGAGTTCGGCTACTTTATGCTCAAGTCGACGACGAACGGCGCGATCCTGGCGGTGGCGCTCGCGAGCTTCCTGTCCATCTCGTCGATCGGGTGGGCCATGGTGGCGCCGCTGCGGTTCTCGATCATCATCATCTTGAGCGTCGTGCCCATCTCGCACACGCTCATTGGCGCGTACCTCGCGTCGACGATGATGACGGCCGACTTTTGCGCGGCGCCGCTCAACAGCACGCTCACGCTGCTGGACGCGTGTCCGACGAACTCGACCTGCCGTTCGCCGAATCGACTGTGAGCGTACGCACTGGCCCATGGCGACATCGGCGCTCGTATGAAGCGCGACTTCCTCGACCCGGTAGGGCTCGCTGTGGGCGTCATGCTCGGGTACTTTATCGGCACCGTCTCGAACTGGGAGGGCGGCGCGTTGGTCGCGACGTGGCGCTGGCCGTTCATCGTGCAGTTTGTCGCCTTGTGCCCCATCAACGTAGCCGTCTTTTTCGTGCCGAAGAAGCATCTCTTGATTCGAAACGAAGCCAATGACCT---GCCGTCCATGAC---GACACCGATGAGCAGTCGCTC---GACCCGCCATGACGAAGAAGATGTGCTACTCGCCAGCGCCGCAATGCGACCTACGGAGCGTTGGATACGAAGATTTCGAGCAGGGAGCGTTCTTGGATGGAGTCTACGAGCTGTGCCACATCCCAGTGTTCTGCCTGATTGTTCTCGGGCTCACAACCATCTACTTCGTGGTGACGGGAGTGCAGTACTGGAGCACGATCTTCATGATCAAGTCGTTGCACGCGTCCAAGTACCTCGTGAACATTCTGTTCGTATTCGTTGCAGGCACTGGGCCGATTCTTGGTGTCTTTTTCGGCGGGTGGCTCGTCGACCGGTGTGGTGGATACATTGGCGTCGAGCAGCGTGCCAAGGCTCTGGGGATCTGCATGATATTGGGTCTCACGGCGTTTGGCATTGGTGCGGGAGCCACGTACTTTGAAGATGTGTACATCACGGCTGGGTTCCTCTGGATGCTGCTCTTCTTCGGAGGCTCTATACTTCCAGCGCACAGAGCACTGGCATCGTCATTCTCGGTGATGGTGTTCAACCTGTTTGGCTACTCGCTGTCTCATGAGCGAGCTCGCGATGGGCGTGGGCCAGAAGGTCTACGTGCCGGACGAGACGCACGTGTGGCTCGAGGCCGAGGTGGTGCGCGAGCAAGGGACGGCCGTGGTGG------------------TCAAGGTGCGGGTTCCCGATGGAAGATGAGGAGCGGACCGTGGATCTCGCCGACAAGAAGGTCAAGGCGATGCTGCGTCGCACCAGCTGGAGGCGCTGCCGTACCAGAACGAGAACGTCGGGGAGGAAGGCACGGAGGACATGATCTCGCTCAACTACCTGCACGAGCCGGCGATCCTGTTCAACATGAAGCAGCGCTTCCTCAAGCAGCTGCCGTACACGTACACGGGAGATATCTGCATCGCCGTGAACCCGTACCAGTGGTTGCCCGAGCTCTACGCCGAGGAGCAGCACGTGCGGTACCTGACGCAGCCGAAGGAGGAACTTCCGCCGCACGTGTACGCCACGTCGGTGGCTTCGTACGACAACATGCGTCGCTCGAACCGCAATCAGTCGATCCTCGTGAGTGGCGAGTCTGGCGCCGGCAAGACGGAGACAACCAAGATCTTGATGAACCACCTGGCCACCATTGCTGGCGGTATGAACAACTCGACTATCAAACGCATCATTGACGTGAACCCGCTGCTCGAATCGTTCGGCAACGCCAAGACGGTGCGCAACGACAACTCGTCGCGTTTCGGCAAGTTCACGCAGCTCCAGTTCGACGTGGACGGGACGCTTGTTGGTGCCAAGTGCCGCACGTACCTGCTGGAGAAGACGCGTGTGATCAGCCACGAAGCTCCGGAGCGCAACTACCACATTTTCTACCAGTCGAGACGCCCGCGCTCGCGGACGATCTGAAGCTGGTCCCCGGCAAGCACTACTCGTTCACGGGCGACAGCTCGGTGCAGAAGATCGAGGGCATGTCGGACGCGACCCACTTTGAGCGCACTCGCGCCGCGCTTACGCTCGTGGGACTGTCAGAGGAAGATCAGAAGCCGCTTTTCGAGGTGCTTGCTGGTGTGCTGCACCTCGGTGAAGTCGCGATCGAAGCCAACCCTGCGGATGACGAGGAGTCGCGGATCGTGGAGGGCGACGTTGGTGCGGCTTCCGCTCAAGCCATGCTGAACGTGACTAGCGAGGCGCTGCAGAAGGCGCTGTGTTGGCGTACGGTGCGCGCTGTGAACGACGTGTACAGCGTGCCGTTGCGACGCGAGCAGGCCGTCGACTGTCGTGATGCGCTCGCTAAGGCCATTTACGGCAACGTGTTCGACTGGCTCGTGAGCACCATCAACGCTTCGCTGGCGGACGACGCGCGCATGGCGTACCACATTGGCGTGCTCGACATTTTCGGCTTTGAGCACTTCAAGCACAACTCTTTCGAGCAGTTTTGCATCAACTACGCGAACGAGAAGCTCCAGCAGAAGTTCACGCAGGACGTGTTCAAGACTGTGCAGATCGAGTACGAGGAGGAAGGTATCGCGTGGAACCACATCGAGTACTCTGACAACCAAGATGTGCTGACGGTGATCGAGTCGCGCATGGGTATCATCTCGCTGCTGAACGAGGAGGTGATGCGTCCGAAGGGTAACGAGGCGTCGTTCATGTCGAAGGTGGCGTCGCTGCACAAGGAG---GACATGGCCCACGTGATCGAGTTCCCGCGCACGTCGCGCACTGAGTTCACGATCAAGCACTACGCCGCGCCGGTGCTGTACGACTCGGTCGGGTTCCTTGAGAAGCACAAGGATGCGCTGCTGCCGGACCTGTCGGAGCTCATGCGCGGTTCGGCGACTCCGTTTGTGGCGGCGCTGTTCGCAGCGAAGGAGACGCCGAAGTCGGCGCAAGTCGCGCGTCGGCGGCGGTGCTCTTACGGTCGCGACTGTGGGCACTCAGTTCAAGGAGAGTCTGAGCGAGCTGATGACGACTATCCAGTCGACCAAGGTGCACTACGTGCGCTGCATCAAGCCGAACCCGATCAA---------GAGCTCGACGGTGATGGATCAAGCCATGGTGATCTCGCAGCTGCGATGTGCCGGTGTGATCGAGGCTATCCGCATTTCGCGTGCTGCGTACCCGAACCGACTCGCGCACATCGAACTGCTCGACAAGTTCTGGCTGTTTGTGCCTCACGGTGGCGAGACGGCTGCGGACAAGTGCCAGCTCTTGCTGGACAAGCTGAAGCTGTCGGCGCCTGAGCAATACCAGATGGGCAAGACGCGTGTGTACTTCCAGCTGGGCGTGCTCGAAGAGCTCGAGGACCGCCGCAAGAAGTTCCTGGACGCCAAGGCGACGTACCTGCAGAATGTCATGGTTGGGTTCACGCAGCGCATCAAGTACCTGCGGAAGCTGGAGGCCATCATCAAGTTCTGTTGCTCGGTGTGTCATCGCCATGCGCCGATACAACACGTTTGTGACGGGTCTCATCCAGGCGCAGGCGCGGTGGCGCGGTATTGAGGGTCGGCGCGTGGCCCACGAGGTGAAGCGCAACCACAAGGCGGTGGTCATCCAGAAGATGGTTCGCGGCCACCTGGAGAAGGTGAGGTACGTCAAGATCCGCGACATGATCATCCGCGTGCAGGCTATTGTGCGCATGAAGATCCAGCGTCCGAAGTACCTGGCGGCGCTGGAGGAGGACCGCATGCAGCGCGACATGGCGTTCCAGCTGGAGATCCTGAAGAAGCGTCTCGCGGAGGAGAAGGACCGTAACGCGCAGCTTGTGAACGAGCGGC---GCAACAGCCTGCT--GTCGATGCGGTTGGCGGCGCGTCGACGGCGATGGCGGACGCCGGCGGTATGATTGAGACGCTGCAGGACGAGAACTCTAAGCTGCGCGAGAAGAACCAGGAGATGGAGACTACGCTGAAGGCTCTGAAGGCTGAGGTGGAGAAGTTCAAGACGGACAAGGAGTACTCTTCGGCGAGCAACCACGTCAAGATCCGTCAGCTGCAGGACACTGTCAAGGATAAGGACAAGCGCATTGCGCACCTGGAGGCCGAGACGGCGAAGCTGACGACGCAGCTCGAGAACGCTGGTGTGACTCCCGGCCA---CGAGGACAAGAAGCCGCGCGAGAAGAAGTCGATCTTCCGCACGCTCGGGTCGAAGAAGGAGAAGAAGCCGT-CGACCGCGCG--TGGCGACACGGTGCTGATGGACTCGCTTACGGGTTCGGATGACCTCAACCGTGGGGACGCGGTGACGCGTGG---GTCGCACTTCCCGCGCATGC---CGAGCATGTCGGCGGCCAAG---TTCTGGGGCAACAAGACTCCC---AAGGGCGAG------------GCTGGCGATGGCAGCGACGTGACGGAGGCCATCGACGAGGACTCGCCGACGGCTCGACGCGGCAGACGCTGGTGAAGAGCGTGGAGGTGGGTGTGACTGGCGCGATGTCGAGCATCAAGGGCCGCATG---------TCGGTC----------------------------------------------------TCGTTCAACCTGGACGCGCTTCCGGAGGTGTCGCTCCCGGTCGGGTGGGAGGCCAAGGTGTCGCGCTCGACGGGTCGGGTGTACTACGTGAACCGCAAGCTTGGCAAGTCTCAGTTCGAGCGTCCGACGCTTGCGTCGCTGAA------GGCGCAGAAGCTGGCCCGGCAAAAGACGGGCGACAAGAGCAAAGGCGGTGGTGGCGGACTTTGGCTGCGGTGACGCTCGGCTCGCCGAGTCAGTGCCCAACAAGGTGCACTCGTTCGACCTCGTCTCGCTTGACGTGGCCGTCTATTGCCTCGCGCTCATGGGGACGAGCATCCGCGAGTACCTGCTCGAGGCGCGGCGCGTGCTCAAACCAAAGGGCGTGCTCAAGGTGGCCGAGGTCAAGAGCCGGTTCGAGAGCGAGTCGCTCGGCGGATGGCGGCGGTCTTCCTGTGCGTGGCCAAGGAGGCGCTCGCCTCGGTGCGCAACACGACGCTCTTCGTTACGAGCGCAAGCACGTGGTTCTCGCGAGCGAGAAGACCGGTGAGGCTGTCGCCATGCCGTTCCCGGACATCGCGCGCGATCTGAAGACGCTGCTGGGTCATACGACGTCGATGATCACGCACATGGCGGTCAACCTCGAGCCTCTTGATCACTGGCGACCGCGACGAGAAGATCCGTGTCTCGCGGTTCCCGCAGACCGCGATCGTCGAGAGCTACTGCCTCGGACACAAGGCCGCCATCACGCGCGTGGGCTGCAGCGTCGTCACTCCGGATCTGCTGGTGACGACCTCGTTGGACAACACGCTCAAGCTCTGGAGCATCTCGACGGGAAAGCTCGCAGTCTCGCCGGTGGCCATGTCGCGATCGCGCTCATCGACAACCGCTACCTTCGCGTGTTCGAGATTGTGACGCGCCGTTCGTCCAAGTGTTTGAAGTGCAAATTGGACAACGCGACGTACAGCCGCTGTGCGCGACTTCCGCAAGCTGCAGAATGATCCTCCATCTGGAGTCTCTGGCGCGCCTATGGATAACAACATTATGCTGTGGCAGGCCGTGATCTTCGGGCCGGACGACACACCGTGGGAGGGCGGCACGTTCAACCTGACGCTCGAGTTTAGCGAGGACTACCCGAACAAGGCGCCGACCGTTAAGTTCATCACCAAGATGTTCCACCCGAACATCTACAATGACGGACAGATCTGCCTGGACATTCTGCAAAACCAGTGGTCCCCGATCTACGACATCTCGGCGATCCTCACGTCCATCCAGTCTCTGCTGTGCGATCCGAACCCGAACTCGCCGGCCAACTCGGAGGCCGCGCGTCTGTTCCAGGAGAACCGGCGCGAGTACAACCGCCGCGTGCGCGAGATCGTCGAGCAGAGTTGGCAGGCCATCGATGGTGTCGGCCGCGAAGATGCGCGGCGCCGAGAACCGCCTGAACAGCGGCCGCCCGTTCGCGTCGTGGCTGGACAACGTGTCGTCGGACCGCGGTCTCTGCGGCGGTATCAACTCGGGTATCGCCAAGGCCGTGCGCAAGCAA---ACGCTCAGCTCTTCGTGATTGGTGACAAGGGTCGCGCGCAGCTGCGTCGCGACTACGCCGACAAGATCCGCGGCAACGTGACGGAGACGTTCGCGACGCCGCCGAACTTCGCTGTGGCGTCGGCCGTGGCCGAGGGCGTGGACAAGGAGAGGAGTCGTCGCGCATGGCTGCCATGGAGAACGCGACGAACAACGCGCAGGAGCTCACGCTGACGCTCCAGTACAACAAGGCCCGCCAGGCGCGCATCACGACCGAGCTGATTGAAATTATTTCGGGCGCCGCTTCCCTCGAATGTGCCCGGGAGCGTCGTGTCCGTGCAGGACGCGGCGCAGTTCGACGAGCTGACGGCGCGTGACGCGACGCTGTCGATCGCCTTCTTCTGGGCGGACTTCCAGCTGTGCCGTCCGAACGGCCAGATCGACGTCGGGGCACACTGTGCTGGAGAAGCTGGAGGGAGCCAATGTCGCGGAGCTCGCCAAGCGCGTCTTGAGTACCGCCTCAAGAAGCTCGTCAATGCGTCGCCCGTGATGCTCTTCATGAAGGGCAACCCGACGGACCCCAAGTGCGGCTTCAGCCGCCAGATTGTCGCGCTGCTGAACGAGGAGAACATCCAGTACGGCACCTTCGACATTCTGAACGACAACGAGGTGCGCCAAGGACTCAAGCAGTTCTCCAACTGGCCGACGTACCCGCAGCTGTACGTGAACGGCGCGCTGATCGGTGGACTCGACATCCTCAAGGAGATGAAGTCGGAAGGCTCCATCGTTGAACAGCTCGGCGTTGAGGAAGCCGAGGCTGCGTTCACGGAGAGCTTGCGCGCGCTGGTCAACTCGGCACCGGTGCTGCTCTTCATGAAGGTTCGACATTTTGTGTGGAACTACAACAACTCGAGCAAGGAGATCGCCAAGGTCGTGAGCCTCGTGCCGACGTCGTTCCGCCACGACATGCGCCGAGAGCATCGCGTGGAGTCCGGAACCGCCTCGCGATGGCGTTCCGCGACCCGCTGGACGGCAAGGGCGAGTTCTGCGTCGTGCGCCTGCCCGCCTGACGTCCAAGGTGACGACGCTGCACCCGAAGGGGATCTCGGCGATCGACTGGAAACTGGCAGGTGCTCCACCGCGAGCACAAGAGCGACATCAAGGCGCTGTGCATCCACTCGCAGCGGAACCTGATCTACACGGGCGGACTCGACGGCTTGGTGGTGCAGTACGACGTGAACCAGCTGCAGACGCAGGTGGTGATGGAGCGCCGCAAGCCCATCTCGAGGATCAACGCCGTGCTGGAGCACCCGCACAACCCGAACATCCTACTCGTGAGCAGTCTGGAGCAGCGAGCACAACATTCTGCTCCACGACCTGCAGCGGTACCGCAGCCAGCTGACGTCCAAGTCGACGAGCCAGTACATCGTGCCGCGCTGGTCGCCCGCCGGCTACCACGTCTCGTGCGGCAGCAAATGCGCTACTGCCACGGCAGCTTCTCGACGCCGACGTCGGCCACCATCGGCGCGTCCTTCCTGCAGAAGCGCATCATCGTGGGCGGCAAGATGACGCTGCAGATCTGGGACACGGCCGGCCAGGAGCGGTTCCGGTCCATGGCTCCCATGTACTACCGCAACGCCAAGGCCGCCATCCTCGTCTTCGACCTCACGAGCGCCGCCACCTTCGAGAAGATCAAGGAGTGGCTGCACCTGCAGAGCCACGTGGGCGAGGACATGTGGGCAACAAGAGCGACTGTTCGACTTTGCGCAGGCGCAGCAGTTCGCCGACCAGATCGGCGCGCTCGTGTTCCGCACGTCCGCCAAGTCGGGCGACGGCGTGCAGACGCTCTTCGAGGCGCTCGCGGCCGAGCTGCTCAAGAAGCACGAGCAGGAGGAGCGGAGAGCTTCTTCGTGGTGTTCGTGCCGGCGGACGCGGCGGCGGCCGTGGAGGAGTGGGCGGTGGACCTGCCGGAGGACACGGAGGCGCAGATCGGATGCCTCACCGAGCGACTGCGCGCGCACTTCAAGGCGAGCGGCGTCCATTGCTGAGCAGAGACGTTCAAGCAGCAGATCCTGGCGCAGATGCCCGAGGGTGCGACGATGGACGCCAACATGCTGCAGATGATGCATGGACAGCTTGGTGGACTCGGTGCCGCTCGTGCTCAACACGCGCGCCGTGCAGCATGTTCGAGGTGCGCGGCGACGCCTTCATCGGCCGCGTCTTCGACAGACGCCTTTGTGCGCATGGACTTCCGGATGGACGAGCTCTCGACCGATGCGCCGTGGGTCCAGCTCGCCAAGCAGCAGAGGTGACGCAGTCTCTGGAGGAGATCCTGGCGCTCGGCCAGGGTCTGGACGACCAGGACTACAAGGCCGAGCTGGACATCCTGCAGAACGACCCGAACTCGAACCTGTACTCGGCCGTCACGTTCGAGGAGCTCAATCTACCGGAGTCGCTGCTCAAGGGCGTGTACGCCATGAAGTTCTCCAAGCCGTCGAAGATCCAGTCGGTGGCGCTGCCGCTCATCCTGGCCGATCCGCCAGAGAACCTGATCGGCCAGGCGCAGAGCGGTAACCGACGCGCGAGCTCGTGCGACAGATCGCGGCGGTCGTGAAGGCGATGGGCAAGTTGTCGACGTGTTCCTGGCGATCCCGGGCAATGACGTGGCTCGCGGCGAGACGCTGCAGTCGCAGGTGGTGATCGGCACGCCTGGCAAGGTGGAGGGCATGATCAAGAAGCGACTGATCGACACGCGGCGCATCAAGATCTTTGTGCTGGACGAGGCGGACGTGATGGTGGCCGAGGACGGCCAGCGCGAGCGGTCCGTGGCCATCAAGAAGATGATCAAGAACCGGTCGTGCCAGTTCCTGCTCTTCTCGGCCACGTACGCGGACGACGTGCGCGAGTTCGCGATGAAGATGGTTCCCGACCACAACATCATCACGGTCAAGAAGGAGAAGCTGACGCTGGACGGCATCAAGCAGTTCTGGATCGACTGTGGCACGCAGGACCACAAGTACCAGGTGCTGTCGGACATTTTCGCGATCCTCACGATCGGCAAGTGCGTCATCTTCGTGGCGTCGCGCGAGACTGCCAAGTCGCTGACGAAGCGCATGCGCGAGAACGGCCATGCCGTGGGTATCTTGCACGGCGCCGACATGGCCAAGGAGGTGCGCGACCAGGTGATCGACGAGTTCCGCGCGGGCACGACAAACGTGCTGATCACGACGAACGTGCTGGCTCGCGGCATCGACGTTGCTGGCGTGTCGCTCGTCGTCAACTTCGACATCCCGCTGACGCGCGACCGGCGTCCGGACCCGGAGACGTACCTGCACCGCATCGGACGCACGGGCCGCTTCGGTCGCAAGGGCTGCGCCATCAACTTTGTCTACGACAACCAGAGCAAGAAGGACCTGGCCGAGATCGAGGACTACTACCAGCGCTCCATCGAGCAGGCTCCTGCCGACGACATCGAGGAGCTCGAGAAGCTGCTCAAGTGGGGGGCAGCAAGCGGCGGCTGGCCAAGTTCGGCAAGGAGCAGCGCAAGGGCATGAGCGTGTACCCGCGCGAGCCGAAGAAGAAGCCTTCGGGCAAGGACAAGACGTACTACTACCTCAAGGACATCATGTTCCTGGCGCACGAGCCGGTGCTGAACAAGTTCCGCGAGTTCAAGACGTTCATGAAGAAGGTGAAGCGCGCGATGGGCCGCAAGGACTACGCCGACGCGCGCCGCAAGCACGAGAACCGGCCCGAGTACGAGCTCGACCACATCGTCAAGGAGCGGTACCCGCGCTTCGTGGACGCGCTCGGCGACCTGGACGACGCGTTGTGCGGCAGAACTACATCGTGTCCTCGCGCGCGCTCACCAAGGTGTTCGTGAGCATCAAGGGTATCTACTACCAGGCCAGTGTGCAGGGCCAGACGATCACGTGGCTCGTGCCGCACCAGTTCACGCCCACGGTTGACAAGCGCGTGGACCTGCGCGTCATGCTCACCTTCCTCGAGTTCTACCAGGTGCTGCTCAAGTTCGTGCACTTCCAGCTCTACACGGACCTCGGCGTCGCCTACCCGCCGAAGATCAATCTTGCCCTTGACCAGGCTGGCGTGCAGCTCGCGGCGCTCGATCTAGCAGCAGAAGGAGTCGGAGAAGCGCATCAAGCTACAACGAGCAGCGCAAGGAGGCGAGCGAGAAGGGTTTGTTCGACGGGCTTGTGTTCTTCCTCTCGCGCGAGGTGCCGGCGGCGCCGCTCGAGCTTGTGCTGCGCTCGCACGGCGCGCAGATCGGCTGGGACGGCGAGGGGTCTCCCTTCTCGGAGAAGAGCGAGCGCGTGACCCACCATCTGATGGACCGGCCGCACCAGGGCCACCGGTACTTCAACCGCGAGTATGTCCAGCCGCAGTGGGTGTTCGACAGCGTCAACAACGGTCTTTTGCTCCCGCTCGCGCGCTACCGCACGCAGCTCGAGAAGCTCAAGTCTGCGTCGCAGGTGCTGCGCGAAGTGGGCGACCTTGAAGAAGGTGACGATGTGTAGAGGTGTACGCGGCTGGGCTCAAGGCTGAGCGCGAGGGCAAAAAGAAGTCTGCCAAGGCCAAGAAGGAGGAGGAGGAGGCCGAGCTGCACGAGCTGGCCAAGAACATGATGTCCAAGAAGGCGAAGCGCCTGTACGACCGCATGCAGTACGGACTCGGCAAGAAGGCCGACAAGATCCAGGCGCTCGAGGCCAAGCGCGCCAAGATTGAGGCAAGACCAAGAAGCTGGTGGCCGGCGGGCTGTCGGGCATGCTCTCGGCGCTCGCCTTCCAGCCGCTCGGAGGGTGCGCGGGCGCGGCACGTCGGCGACGCTGATCCGCGTGTCGGGCGGCGCGGCGCTGTACTTCGGGTCGCTGGACCACCTGCTCGCGCTGTTCCCGCACACGGCGGTCAACACGTTCCTGGCCGGGGCGTTCGCACGGACGTTCGCGGGCAGCGTCATGTCGCCGCTGACGGTCGTCAAGGCGCGCATGGAAGGCGCCTGGCGCGGCCCACGCGCGACGGACGTGCCCTTCTCGGGGCTGTACGTGCTCATCTACACGGCACCCGGCCGACGTGGTCAAGACACGCATGCATGCGCCAGACCGTGGCGCGCATCGTGCAGGACGAGGGCTGGCGGGGCTTCGCCAAGGGCATCATGCCGCGCGTCGTCAAGCGAACGCTGTCCACCGCCGTCACGTGGACCATCTACGAGCTACTCGTTCGACCCGTCCGGACTGGGCTATGCCAAGGACGCGTTCAGCATCGCCAAGGAGACGGAGCGCACGAAGCAGATGGAGCACCAGGCCAAGGTGAAGGAGCAGGAGGCTTTGTACAAGCAGTACGAGATCGTGCGCGTGCAGAAGGAGGGCGAGGAGCGCCGCAAGACGCTTGACGAGGAGACCAAGCAGCACCAGACGCGCGCGCAGTACCAGGACCAGCTCAAGCGCAAGCAGTACGCCGACCAGCTGGCGGCGCAAAAGTACATGAAGGAGCAGGAGCTGAAGAAGCAGGAGGAGATGATCGCGCGGCAGGAGGCCTCGCGCCGCAAGACGCTCGAGTACGAGGCCGAGCTGCGCCAGAAGACGGAGCTCGCCAAGGTGGCGGCCGAGGTGGAGGGGAAGATCAAGCAGGAGCGCCTCAACCACGACCTGCACCTCGAGGAGGCGCGCGTGCGTGCCAAGGAGTACCGCGAGACGGTGCTCGAGGGCATCAAGCTTGCCGGCTCGACTGTCGGCGCCGGTGTCATGGCGTTCGTGGACGACAAGGAGAAGCTGACGGCGACCGTCGCGTCGCTCACGGCGCTTGCGCTCGGTATCTACACCGCCAAGGTGTCGACCGGCGTGGCGGGCAAGTACGTCGAGGCGCGTCTCGGCAAGCCGTCGCTCGTGCGCGAGACGTCGCGCCGCTCGGTCGCGCAGGTGGCGACCAACCCGATCCCGTCGCTCAAGCGCGTGCTGCGTCTGCAGAAGCCGCACGACGCGCTCGAGGGCGTTGTGCTCGAGCCGAAGCTGGACGAGCGCCTGCGCAGCGTGGCCGTGTCGACATTCAACACGAAGAAGAACCGCGCGCCGTTCCGCCACCTGCTGCTGCACGGCCCGCCCGGTACGGGTAAGACGCTGTTCGCGAAGGCGCTGGCGCGGCACTCGGGCCTCGAGTACGCCATCCTGACGGGCGGTGACGTCGCGCCGCTCGGCCGTGAGGGTGTGACGGAGATCCACAAGCTGTTTGACTGGGCGTCGCACAGCCGCCGCGGCCTGCTGCTGTTCGTTGATGAGGCCGATGCGTTCCTGCAGAAGCGGTCGAACACGGTCATGAGTGAGGACATGCGCAACGCGCTGAACGCGTTCCTGTACCGGACGGGCGAGGCGAGCGACAAGTTCATGATTGTGTTCGCGAGTAACCAGCCGGAGCAATTCGACTGGGCCATCAACGACCGTATCGACGAGATGGTCGAGTTCAAGCTGCCGGGCCACGCCGAGCGCGTGCGCATGCTCAAGCAGTACTTTGACGACCTCATCCGCGCGCCGAAGAACTCGCGCGCCAAGAAGATCTACGCCAGGGCGTTGTGTTTACGCTCGATCCGGTCGCCAACTTCCTGATTCCAAGACGCGCATCTTCCAGTTGGACGCGCTAGAGGACGTGTCGTCGGACGCTCAGGCCATCTTTGACGCTCTCAACAAAAGCCGATGCGCATCTACTGGGTGATCTTCGGGGTCGTGCTGGCCAACGGCGCGTACACGTACTTCACTGGCAAGGACGAGAGCTTCATCGTGGAGAAGCTGCGGACAAAGATCCCGTCCGAGTACCGCATCTCGGAGAAATGGGACAAGTGCCTTGAGAACTTCACGCTGCACTTCACGGCCGGTCTGGTCGCGGGCGGGCTCACGTCCCTCGTGCTGGCGCGATCGGGGAAGACGCGCGGCGCGATCGCCGGGTTCGGTGAGCTGGCGCGGGGTCTAGCTGGACGACGTGCCAGCTTGCCTTCGCCGGGGACAGTCAGGAGAAGCTCGACAAGGCGGACAAGGTGATTGACGAGATGCCCGCGTACAAGCAGCCGACGCTGGTGGACTACACCGCCGCCTCGGACGTGCAGAAGAAGCTGCTGGCTGCTTACCAACAATGCCTACGACGAGGCTGTCGTGCCGCTGCTCGAGGCGTACGTCGCCGAGCAGGTCAAGAACTCGACTGTCGACGTCGACGCCGACCTGGCGCTGCTGAAGCTGTACCTCGTGTACCCTGGCAAGACGAACGTGGAGAACCCAGGTGCTGGTGAAGGGCGTCACGTCACTTCCCTCGACGTTCTTCACGGGTGACCACCATGATCCCCACGAGCGTTCGTGAGGACGCCAACGTTCAGGCCGTCATCGAGGCCGGCTTCCTCCTCCAGTCCTGCCTCTTTGAGGAGTTCTGGACGAAGGACCTTGCGTTCGCCAAGGCGGTGCCGGGCTTCGAAGATGCCGCGCGCGCCTTCATCCTCGAGACGATCAGCCGCAGCCACAACGCGATCGCTGCGTCGGTTGTCAAGGCCAAGCTCAACGTCTCGGACGCCGACCTCAAGACTGCCATTGCCGAGGGCTGGACGAGGGCGATGTCGTCAAGATCACTGCGAACGAGCATAACCAGATGCGACCGAAGAAGGTGCAGGAGGACCTTCAGTTCGAGGACGTCATGAAGGTCATCCACACTCTGTCCAAATCGAACGTCTGCGCCAGCGGCGGTGGTGTCGCCGACGCGCAGGAGGTCTGCGACGCCGTCGCCAAGCACCTGCGGCTCGAGAGCGGTAACCAGGTGCTGCCGTGCTCCACGGGCGTCATCGGCTGGCGCATCCCCGTCGACGCCATGGTCGAGAACGTGCCGCATCTGCTCGAGAACCTGCAGTCCGAGTCGGTGCTTCCCGCCGCCGAGGGCATCATGACGACTGACTTGTACCCAAAGATCCGGTCCAAGGACGTGGCGGGTGGTCGCATTGTCGGCATCGCAAAGGGAGCGGGCATGATCGAGCCGGACATGGCGACGATGCTCTCGTACATCCTCACTGATCTCTCGGTCCCGCGCGAGTTGCTGCGCCAGGCGCTGTTCGAGGTGGTGCAGGACACGTACAACGCGCTGAGCGTCGACACGGACCAGAGCACGTCCGACACGGTGACGATCGTGTCGTCGGACCAGATCCCGTTCGACGTTGCGAACCAGCTGGACGAGTTCAAGCAGGCGCTGAAGGAAGTGTGCCAAGGCCTGAGCGAGGACATTGTGCGCAACGGAGAGGGAGCCCACCATGTGATGCGTGTTGTGGTGCGCGGCGCCACGTCGGTGCAGATGGCCAAGGGCGTGGGCAAGTCGATCGTCAACTCGCCGCTGCTCAAGTGCGCGGTGGCGGGCAACGACCCGAACGTTGGGCGGCTCGTCATGGCTGTCGGCAAGTACATGGGAAAGCACCACAAGGACGTGGACATCACGCGCCGCATGAAGATGAAGGTCGGCGGCGTCGTCATCTTTGAGAACGGCGAGATGGTGCTGAACGACGAGATTGAGGAGCTGTTGGTCAAGCACATGCGCGCGGCGCAGCTCACCGAGCCTACTCGCGGCGGTCTCGACGTCACTTCGCGGGACTACCCGCCGCACGGCAAGTTCGTCGAGATGGAGATCGACCTCGGAATCGGCAGCGAGCAGTCGCAGGTGCTTGGTATCGACCTTACGCACGAGTATGTGGCCATCAACGCCGACTACCGCTCGTA-------------------------CGGCGGCGGCNNNNNNNNNNGCTC--GGCGTCGACACGGC---GGCTGGGGGCGAGAAGAAGGACAAC------AAGAAGAAGAAGAAGGGCAAGAAGAAGCCCGAAAGAAGCCCGTGTCTGCAGCCGCCCGAGCGCTGG-TGGAGCGCCAGGCCGCGTTGAAGGCGGAGCAGGAGCGCCTCGAGCAGGAGCGGCTGGAGCAGGAGAAGCGCGAGAAGGAGGAGGAGGAGCGCCGTCTGGCCGAGCTCGCGGAGCAGGAGGAGATCCGCCGCAAGAAGAAGGAGGCCAAGGCCGCCAAGGTCGAGCGCCAGAAGAAGGAGGGCACGTACATGACCAAGGCGCAGAAGCAGAAGGCTGCGCGTGCCCAGGCCGCTCTGGAGGCAATGCAGCAGGCTGGACTGGTGCCAGACGCCTTGAAC---GAGGAGAAGAAGAAGGTGGTGTACAGCAACAACAAGAAGAAGAACCAGAAGCCGAGGAGGTCGAGGAAGAGGAGGAGGAAGCACCGGACGCGTGGGACGATGAAGACTGGGAGTCGAGCGACCTTGTCGCCTCCCTGGAGGACAAGCTAGCAGAAGAAGGAGCAGGAGCGCCTTCGTCAGCAGGGCATCGCGCTCAAGAAGCGCCAGGAGGAAATGGAGGAGCAGCTCCGACGTGAGGCCGAGGAGGAGCGTCTGCGGTCCAAGGCGCAGCAGGAGGCCAACCGCAAGAAGAAGGAGGCTGCCGAGCGCCGTCGTCTGCGCGAGGAGGAGGCCCTCAAGAACGTTTCCACCGACAACCTGCGCTCGCCAATCTGCTGTATCATGGGCCACGTCGATACGGGCAAGACCAAGCTGCTGGACAACATTCGCAAGACCAAGGTCCAGGACGCCGAAGCTGGCGGTATCACGCAGCAGATCGGTGCGACGTTCTTCCCTGTGGAGGCGATCAAGCAGAAGACGGCTGCGCTGGCGAAGACCATGAATCTGGAGTACCGTTTGCCGGGTCTGTTGGTCATCGACACGCCCGGTCACGAGTCGTTCACAAACTTGCGATCGCGTGGTTCGTCGTTGTGTGACATCGCCATTTTGGTGGTTGACATCATGCACGGTCTGGAGCCGCAGACGCTGGAGTCGCTGCGCCTGTTGCGTCAGAAGAAGGCTCCGTTCGTGGTCGCGCTCAACAAGATCGATCGCTGCTACGGGTGGAAAACGATGCCCGACATGCCGGTCCAGGAGGCGCTCAAGTACCAGAACGACCACGTGCTGCGCGAGTTTGAGGACCGCGCCAAGCAGGTGCAGGTGGAGTTTTCCGAGCAGGGTCTGAACGCCGAAATTTACTGGCGCAACAAGAACCTGGGCAACACGGTGTCGCTGATTCCGACCAGTGCGATCTCAGGCGAGGGTGTTCCGGATCTGCTCATGATGCTGACCCGTCTCACCCAGGAGCGCATGGCCAAGTCGCTCGCGTACGTGGAGATTCTGCAGTGTACCGTGCTGGAGGTCAAGGTGATCGAGGGTCTGGGCACGACGATCGACACGGTGCTGGTGAATGGCCGGCTCAACGAAGGCGATCAGATCGTCGTGTGTACACTGGAAGGTCCAGTGGTAACAACGATCCGGTCGCTGCTGACCCCACACCCCATGAAGGAGATCCGCGTGAAGGGCGAGTACCTCCACCATCCGACGATCAAGGCCGCTATGGGTGTCAAGATTTGCGCGAACGGACTCGAGAAGGCCGTCGCTGGCACTCAGATCTGCGTCGTCGGCCCGGACGACGACGTGGACGAGCTGAAGGACTCGGTCATGTCGGACCTGACGGGCATTTTGGACTCGGTCAAGGCGACCAAGCGTGGCGTGATGGTGCAGGCGTCTACGCTCGGCGCGCTGGAGGCGCTGCTCGAGTTCTTGCGCACGTGCGAGCCCCCTATCCCGGTGTCGTGCGTGAACATCGGCCCAGTGCACAAGAAGGACGTGATGCGTGCGTCGGTCCAGCTGGAGCACCAGCGCGAGTTCGCGACGATTCTGGCCTTCGACGTCAAGGTGCACAACGACGCCCACGAGCTGGCGCAGGAGCTGGGCGTGCGCATCTTCACGGCCGACATTATCTACCACCTGTTCGACCAGTTCACGGCGTACATGGACAACTTCCGGCAGGCGCGCCGCGAGGAGTTTGCGGACGTGGCCGTGTTCCCGTGCGTGCTCAAGATCCTGCCCAACTGCGTGTTCAACAAGAAGGACCCGATCATTTTGGGCGTGGACGTCGAGGAGGGCATCCTGAAGATCGGCACGCCGCTCGTGGTGCCGTCCGCGGGCG---GCCTGCTCGTGGGCAAGGTGGGCTCGATCGAGCGCGACCACAAGGAGGTGGACCGCGCCAAGAAGGGTGCGTCGGTCGCCGTGCGCATCGACAACGAGGGCAGCGTCATGTACGGCCGCCACTTTGACCACAAGAACAAGCTCGTGTCGCGGCTCACGCGCCAGAGCATCGACGCGCTCAAGGACAACTTCCGCGAGGACCTGCAGAAGGAGGACTGGCAGCTCGTCATCAAGCTCAAGAAGGTGTTCGACATCATCATGGAGGGCTGGCTCACCAAGCGGCGGGACCACATGAACCTCATCTGGCTGGAGCGCTACTTTGTGCTGGACGGCAACCAGCTCCGGTACTACCGCAAGCGCGGCGACCCGGCCCCGCGGGGGACGTACGTGCTCACGGACGGCTGCCTGGTCGGACCGGTGTTCAACTCGGAGGACAAGCACAAGCACCACGGACCCGTGTGGATGTTCCGCATCACGTGAGAAGTCGTACAAGAAGGACCGGTTCCTGGACCTCGGCGCCAAGACGGAGGAGAACGCGAAGGAGTGGCGGAAGGCGCTGGAGTCGGCGGTGCGGTCCATCCGAGAGCTCGTGCTGCACCAGCGCAAGATGAAGACGGACGCCGACCTGTTCGGCCCTGCCACGAGCTCGTCCACGACCGTGCCGCGACTGCTCAAGGCGGACGAGGACACGGTGTCGCTCTTCAACCTGGACGCCGACAACGTCAAGGCCGAGCAGAGCTGGTGGCTCGCGCGCGTCGAGGAGGGGCTGCGCGTCATGCAGGAGTGTCCGCTGGGTCCGAGCGTCGCCGCCTCGGTGTTCGCCCAGCACGCCAACACGAGCAACGGGCTGCTGCTGTCGGCGACCGTGTCGGCTCTGCTAAGCTTCAGCATCATGTTCTCGTTCGTGCAGAACGTGCTGGTGGGGGTGGCGACCGCGTTGGCGGCGGTCGGTGTCTGCTGCCTGGCGATTACTCCTGCGACGGCGCTCGAGATCCCGTCGTTCCGCGTCTCGCAGGTCGTGCACGGGTCGCCGACCGAGGTTTTCCGGCTGCTCATGAACCCGAAGCGGTTCCAGCGATGGGACCCATCGATCGCGACGCTGCGCGTGATCCAGGAGATGGACGACCACGCCGACATTGTGTACATCACGCAGCGGCCGACGTACCTGTGGCCCATGTGGCACAAGCCGCGCGACCTCGTGCTCATGCGCTACTGGCGGCGCGAGGAGGACGGCTCGTACTTTGTCATGTACCAGAGCGTGGACCACCCGGAGTGCCGCGTGCGCCACAACTACGTGCGCGCGAATGTGCTCGGCGGCGGCTTCGTCATCGCGCCGCAGCGCGTGCCGAGC---GGTGCCATCCGCTCGTTCGTGACGTACGTGCTGCGGTACGACCCCGGCGGCTG---GTCGCGCATCTACCACCAGCTCGGGATGGACGTGGACCTGGCGATGCCGATGCTGCGCAGCGTCGTGGGGATCCGCGACGAGATGAGCGGCAGCGACTTCATAACTCCGATGGTGACGGTCGCGCCGGAGGTGGCAG-AAGTGAGCAGTATGGCTTCGTGGA---CGCCTCGGGTCTCGTGCAAGGCGTCGCGCGGTTCAAG---ACGAGTCTGCCGGACGCCATGTGGGCCGAGCCGGACGCGTCGCTCTTCCAGGTGCGCGGTCCGAACTACCTGATCGACAAGAAGAAGGTGCCGTCGCCGCCCTCGCGCTTCCACCTCGTCGGCGTCGACCTGCTCTCGTTCGAGTCTGCGAGCGACCGGTACAATATCTCGGCGCAGTCGGGGAGCGTCGCGAGATCCAC---GCCCAAGTTCATGTTCGTCATCAACATGATCATTCCTGGTCCGGAGAACGTGAGCATGGTCTTCTACTTCCAGCCGACGCGCGACAATTGGTTCGAGGACGGTTCGGCCTTCTCGGAGCTGCTCAACGACTTCCTCGACGGCGACGACCAGTTCCGCAACTCGCGCTTCAAGCTCATCCCGACCGTCGTCGAGGGCAGCTTCATCATCAAGCAGTCGGTCGGGTCCAAGCCCACGCTGCTCGGCAACAAGCTCAAGTGTCCGTACCACCGCGGCGACAACTACTTCGAGGTGGACATCGACATTTCCTCCAACTCGGTCGCCAACACGGTGGTCGGTATGGTGCAGGGCGTGACCAAGTCACTGGTCGTCGACATGGCGTTCCTGCTCGAGGCGCAGTCGGAGGAGGAACTGCCCGAGACGATGCTCGGCGCCGTGCGGCTGCAGCACATATCGCTGGACAACCCGCGACGGGTGACGCGCG--------------------------------------------------------------------------------------------------------------------------------------------------------------------------------------------------------------------------------------------------------------ATGGTGGTCTGGACAG--CCGACAACCAGTCCAAGATGGCGACCGAGGGAGGTATCGACATGCTCATCGACCTGCTCGGTAGCTCGAATGAGCACGTGCAGCGCCAGGCGGCCAAGGCGCTCGCCAACCTTGGCGTCAATGTGGACAACAAGGAGCGGATCGCCAAGGCCGGTGGCATCAAGCCTCTCATCGCGCTCGCGAGCTCCAAGCAGATCGGCGTGGCGGTCGAGGCGATTGCCGCGCTTGCCAATCTCGCCGTCAACG---ACGCCAACGAAGTCGAGATCGCGCGAGAGGGCGGGCTCAAACCCATCATCGACGGCGCCTTCTCGGAATCTGTCGAGCTCCAGTCGCAGGTTGCTCGCGCTCTTCGCAACCTGTCGGTCAACCCCGAGAATAAGCAGGCGATCGTCGAGTTGAATGGCGTCGAGGCCCTGCAGAGCCTTGTGCGCTCTCCGAACGACCGGATATGCCAGCAAGCAACGCGAGCGCTCGTGGCGATCCCCGACGTGTGTCGCTCCTCGCCGAC---CCGCTG---CAGATCTCGTCCGCGGCGGGGGTGAACGAGGCCGAGGCGAT---------GTTGGGCGAGGCCCAGACG------------GAGCGCACGGTCAAGGGTGCGCCGGTC---------------------------------------------------GAGTTCTCGTCGCTCTCGGTCCGCTACCCGCTCTCCACGG------GTGACAGGTGCTTGTCGGCGCCGATCCCGATGAATCGGCCTTTGACGGCCA---GCACGCTCGACATGAGTC---AGCAGGACCAG-------------ATGACGGTGGTCATGGAAACCGCCAGCAGCAATCTGAGC---CGCAAGACGATCAGC---CAGGA---CGGACACTGTCTATCTGAACAAGATCACCAA--GAGCGAGCAGGACGAGATGGATCTGCTGAAATGGAGCATTCAAAAGCAGCAGTTCGAGCGGTATGCGGCCCTCGAGCAGAAGCACCAGTTGTGGGGATATGTGGACGAGCAGAACAGCGTGTGTTCTTTCACCCTGGAACGAGACGCGCAGCGATCCTATCTGAGAAGCGATACCACGACGAGCAATATCGAGCTGCGGTCGGAAAGGGGGAGATGGCTTGGTATCAAGAGCGCCAGCAAGAGTTCGAGAACCTCGTTCGCGACTTTACCGAAGAAACCAAGCACAAGCAAGATCTGATGCTGGCTCGTTTGGAAAAGACCTCGCCATTCTCGACGCTTCCGATTTTGGGGAACAGGTTTCTGCTCGTACGTCTCCGCGGCAAAGGCGGCAATGGCGAAGTATGGGACGTCATCGACTACGCGAACCAAAAGCAGTGCTGTGCTTTGAAGCTGTCGACGTCAGTCAAGCACGCTCAGCGCGAGTACTATACTCATACTGCACCATCCCA--ACATCGTTCAAGTTGGAGAGAGTGCGTTGTTGATTGAGCACCGGCAGCAAATGTACACGGTATTTACTCTCGAGAGAGCGGAAGCAGACCTGCAGCATCTTCTCGAGG---TGAACGAGACCTTCTCTGAGGCCACTGCTGTGAATGTCCTCTACCAGCTGCTGGACGCTCTGACGTATCTGCACCATGAGAATCGCGCACTACGACATCAAACCGTCCAACATCTTGGTGACAGCGAACGACCAGATCAAGTTG------ACTGACTTTGACTTGACACGACATGTGAACGAGCCCATTGCGTCGTCCTCGATTGGGACGTTCCGATATCTTCCTCCGGAGTGCTTTCGCCCCAAGTTCGGCAA---------TTGCGATGGCACGGCCGAGAAAGCCGACATCTGGATGGTGGGGATCGTCTTCTACCTCATGCTGGTCGGCAAGCACCCGATCTGCAGCGACAAGATGCCTCATGAAGACGCCAAGGTGACACTGATGCAGTTCAATGGC---GACATCAAGTACCCTGCCGGGATCTCGGATGTGGCGCGCTGGATCTGCCAGAGCTGTCTGCAACCAGACCCGCGATGTCGACCTGCCGCGTCCCAGCTCCTCGTTGCGGTTCGC

Saprolegnia_parasitica TGAGCAACCGCAGCAGCGCCGCCGCCGAGGCCATCTCGTTCCTGCATGACCTCCACCGCTGGGGCGCGGGCTCGATGGACTTTGCGCCGTTCCTCCGCCATGTGGCCATGGTCTGCTTCAGCATTGGCCTCGTCGCGTTCGCCCTCTTGCTCCTCATCGTGGCCCGGCGTATCTTCTTGGCCGCGCCAGCCGCTTCCGCCGACGTGCGCA-----ATGAGGCTTCGTTTGACGTCATGGCCGTGGTCTTGCTGGGTATCACGGCGCTGAGCGCGCTCAGTGGCCTGCTCGCCGAGGCGTCGAGTATTCGGCCAGCGGCGTCCGAGCGACCGTCGACGACATGATTGTGGCGTTCAAT---AGCTCGTTGCCGCACGACGCCAACGACCTCGCGATCGAGGCCTTAGCATGGACCACGCCGGGCAGGCGCTCTTGGAGC--ACGGCCGCGCC-CTCCCTAGCAACCTCTCGTCCATGGCGTTCAACTGGGAAGCCGAGTACTTTTGGATCAAGTCGTCGACGAACGGGATTATTTTGACCATGGCGCTCTCGTGCTTCCTCGCCATTGCGGCCATTGGGTGGTCCATGTCGGGGTCGCTGCGCTTTGCTATCTTTCTCGTGCTCGTGATCATTCCGTCGTCGCATGCGCTCTTCGGCGTCTACTTGTCCAACTCGATCGAGGCCGCCGACTACTGCATTGCGCCATCGCACAACACGCTCCGCCTCTTCCGCAAGTGTCCGTCGAACGCGACTTG-TACGCGCCGTCGTGGGCGCGAGACTACGCCAAGACGC-TGCCGACGATGGACCGCGAGAACAGAGCATGTACCTAGACCCGATTCAAGCAGTTATCGGCGTCATGTTTGGCTACCTCTTTGGGTCCGTCTCCAACTGGGAAGACTCGTGCGGCATGGCGTGCTGGCGCTGGCCGTTCGTGCTGCAGTTCCTTCTCGTGCTGCCGCTCATGGTTGGCGTCTTCTTGATCCCGAGCGAGCACATCTCGATC--------GCC----------GTGCTGC------------------------------GCGCATCGATCCGCGCCTCGTACATCACACCGCGCTTCCGCATGGACTCG-------CGGCACCTCTCACTGGTGCCGGACGACCTCAGCGTCCTTC---AGAGCGTCGTCGTGCTGCTCTCCAAGCCCATCTTTACGCTCATCGTGCTCGGTCTCTCGGCCATCTACTTTGTCGTGACCGGCGTCCAGTTCTGGTGCACGATCTACCTCCAAAAGAACTTCCACACAAGCGTTGTCATTGTCAACGGCCTCTATGTGCTCGTCGCTGGCACGGGGCCGATCCTCGGCGTCTTCTTCGGCGGCGCCCTCATCGACCGCGCAGGTGGCTACATTGGCGCCGTCGCGCGGGCCAAAGCGCTGAGCTACTGCGTCGGTCTCGGGCTCATCGCGTTCCTCCTCGGTATCCTCACCACGTTCCTTCCGGACGTCTATACGACGGCCATGTGCCTCTGGCTGCTCCTCTTCTTCGGCGGGTCCATCTTGCCGGCGCTCCGGTCGCTCGCGTCGTCGGTCTCGGTCATGGTCTTCAATATCTTGGGGTATGCGCTGGCGCATGTCGTCGCTCGAGATGGTTCCCGGGGCGCTCTGCTACGTGCCCGATGAGGTCGACGTATGGCTGCCGGCCGAGGTCGTCGGGCAGAAGG---CGGGC---------ACGAAGCAGATCACATGCAAGGTCTGGACCGTCGATGGACGTCGAGGAGCGCGTCGTCGACCTCGAAGACAAGAAGACGCGCGCGCTCATGCCATGGAGACCGTCGACACGCTGCCGTTCCAGAACGAAAACGTCGGCGACGACGGAATTGAGGACATGATCACATTGAACTACCTCCATGAGGCCGCCATCTTGTACAACGTCAAGAAGCGCTTCTTGCGCGAGCTCCCATACACATACACGGGCACGATCTGCATTGCGGTCAACCCGTACAAGTGGCTGCCCGAGCTGTATGCGGAAGAGCAGCACGTGCGGTACCTCAACATCCCACGCGAAGAGCTGCCGCCGCACGTGTACGCGACCTCGGTCGCGTCGTACGAAAACATGAAGACGCACAGCCGCAACCAGTCGATCCTCGTCAGTGGCGAGTCGGGCGCTGGTAAGACGGAGACGACCAAGATTCTCATGAACCACCTCGCGACGATCGCCGGTGGCATGAACGATGGCACGATCAAGAAGATCATCCAAGTCTCACCGCTGCTCGAGTACTTTGGTAACGCCAAGACGGTCCGCAACGACAACAGTTCGCGCTTCGGCAAGTTCACCCAGCTCCAGTTTGACCGCGCGGGCACGCTCGTCGGTGCCAAGTGTAAGACGTACCTCCTCGAGAAGACGCGTGTCATTGGCCACGAGTTTCCCGAGCGCAACTACCACATTTTCTACCAGTCGACTCGGGGGACATGGCCAAGACGCTCTTCCTTGATCAGGACGCTACATACCGGTACATTGGCGAGAAGAGCACGGCGCTCATCGAAGGCAAGTCGGACGCACACCACTTTATGGTCACGGCCGAGCGCCTCAGCCTCATTGGCTACTCGGCGGCGGCGCAGCTCGACCTCTACAAGACGCTTGCTGGTATCTTGCACCTCGGCAACCTGCAGATCATCTCGGACCCGTCCGATGACGAAAAGTCCATGATCGCGCCCGGCGATGTCTCGGCGACGTACATCACGCAGCTCATGGGCCTCACGATGGAGTCGCTCGAGAAGGCGCTCTGCTCGCGTACGATGCGGGCGCGCAACGACGTGTACAGCGTGCCGCTCAAGAAGAGCGATGCGGAAGCGTGTTTGGACGCGCTCGCCAAGGCCATCTACTCGCGCGTCTTTGACTGGCTCGTCGAGCTCATCAACTTGTCGCTCTCGAACGATGCCAAGATGACCAACCACATTGGCGTGCTCGATATCTTTGGGTTCGAGCACTTCAAGTTCAACTCGTTTGAGCAGTTTTGCATCAACTACGCCAACGAGAAGCTGCAGCAAAAGTTCACGTCGGACGTGTTCAAGACGGTCCAGATCGAGTACGAGGAAGAGGGCATCCAGTGGGAACACATTGAGTTCTCGGACAACCAAGACGTGCTCAACGTCATTGAAGACCGCATTGGTATCATCTCGCTCCTCAACGAAGAGCTCAAGAAGACCAAGGGCTCGGAGGCCGGCTTCATGTCGACGATCTCGTCGATCAACAAGGATTTGTACAAGCCCAGCGTCATTGAGTTTCCACGTACGTCGCGCACCGAGTTTATCATCAAGCACTACGCGGCGCCCGTCAAGTACGAGTCGCTCGGGTTTCTCGAGAAGCACAAGGATAACTTGCTCCCGGATCTCTCGGACCTCATGCGCGGCTCGGACACGACGTACATCCAAAAGCTCTTTGAGGAAAAGGCCG---TCGAGTCGCCG------------TCGCGGCGGCACGCTCAACATCTCGACCGTCGGGTCGCAGTTCAAGGACAGCTTGACGGAGCTCATGACGACGATCCAGTCGACCAACGTGCAGTACGTGCGTTGCATCAAGCCCAATTCGATCAA---------GAGCGCGACGACGATGGAGAACGCGATGGTTGTCTCGCAGCTGCGGTGCGCCGGTGTCATTGAAGCCATTCGCATCTCGCGCGCCGCGTACCCGAACCGACAGACGCACGAGGAGTTCTTGGCCAAGTTCCGCCTCTTCGTCCCCGCGGGGCCGGGCAACCCGCGCGAAAAGTGCGAGCAGCTCATGACCAAGCTCAAGCTCACGTCGCCGGCGCAGTACCAGATGGGCTGGACCAAGATCTACTTCCAGCTCGGCGTGCTCGAAGAGCTCGAGGACCGCCGCAAGAAGTTCCTCGACAAGAAGGCGCGCTTCCTCCAGAAGATCATGAAGGGCTTCACGCAGCGCATCAAGTACCTGCGCCAGCTGCGCGCGATCGTCAAGCTCGGTCATCCGCTGCGTGCTTGCGATGCACCGCTACAACACGTTCCTCAAGGGCCTCGTCAAGGCGCAGGCGCGCATCCGCGGCATTCTTGGCCGCAAGGTGGCGCTCGAAGTCAAGCGCAACCACTGCGCCGTCATCATCCAGCGCCATCTTCTCGGGTATGCCAAGCGCATCACGTACAAGAAGAAGCGCCGCCTCATCATCCGCGTCCAAGCGTATGCGCGTATGCACCTCGAGCGCCCGAAATACCTCGCCGCGCTCAAGGAAAAGAAGATGGAAGCCGACATGCAGTACCAGCTCAAGATGCTGCAGCAGCGTCTCCTCGAAGAGCAGGCGCGCAACGCGCAGCTC-----GCGCAGGC---GCAGTCGCA-GCC--GACGACCCGACGGAC------AAGGCGATTATTTCCGACTCGGCCGATATGATTGGGTCGCTCCAGGACGCCAACGCGATCTTCAAGAAGGAGAACGACGAGATGAAGTCGAGCCTCGCGTCGCTCAAGTCGGAGCTCGAGAAGCTCAAGCTCGACAAGGAGCTCGCGATGGCCAACACGATCGTCAAGCTCAAGCAAGAGCAGGAAGCGCTCAAGGTCGCGCACAAGAAGATCGAAGCGCTCGAGGCCGAGAACGCCAAGCTCAAGGACACGATTGCCAAGGGCGGGTTCCCAGCCCACGA---CGCCAGCGCCAAGCCCGCGCGCCGCAAGC---TCTTCCGCACGCTCGGCGCCAAGAAGATCACCGAGGCCGACGTGCGCGCGGCCGAAGGCGGCGTCATGCTCGACTCGCTCACGGACGAGAAGGATGTCTCGACGGGAGCGCCCCGCCTTCTGGG---GCAACGCGACGCCGGCACAC---GCTACACCGGCGCACGCCGA---CGACGCGACGTCCGACACGGA--GAAGCGCAACT---------CGGACTACAACGACTCGACGGTCAAGACGGACGTCGAGAGCGACGGCGGCTCGCGCCGGTGCTGGGCATGATCTCGTCCGCGTCGACCTCGAGCGTGAGTGGCGCCATGAGCCGCATCAAGGGCGGCATGGGTATCCCGTCGGGTTTCAAGAGCTTCTACGGGGCGAGGAAAAGAAGGCAACTTGCCGCCGCAGGAGTCGTTCAACCTGGACTCGCTTCCAGAGGCGCCGCTGCCGATGGGCTGGGAAGCCAAGGTCTCGCGCAACAACGGCAAGGTGTACTACGTCAACAAGTCGCTCAAGCTCACGCAGTGGGACCGTCCGACGATCGAGACGCTCAAGCTCATGAAGCAGAAGAAGGCGCAGGAGACGACGGCGGCGGCGGCGAAAGGCGGTGGTCGCCGACTTTGGCTGCGGCGACGCGCGCCTCGCCGCGTCTGTGCCCAACACGGTGCACTCGTTCGACCTCGTCTCGCTGGACGTTGCTGTCTACTGCTTGGCGCTCATGGGCACGACGCTGGTCGAGTACCTCTTGGAAGGTCGCCGTGTGCTCAAGCCCAACGGCGTCATGAAGATCGCCGAGGTCAAGAGCCGCATGGAGACGCCGTCGCT---CGGATGCTCTTCTCGCTCGTCGCTTGCGCGTATGGCCAAGCCATCTTCATCTACAACGGTGCGTCGTCCTTCACGGCGGCAAGGAAGCCATCCTCGTGGGCGGCAACGCCGGCGAGGTCAACGCGTACCCGCTGCCGGACGTGACGGCGGGCGAGAAGAGCCTCCTTCAGCACACAACGTCGATCATCACGGACGTCGCGATGAGCCGGCAAGTACCTTCTCACGGCGGACCGCGACGAGAAGGTCCGCGTCTCCCACTTTCCGGCGACGACGCTCGTGCAAAACTACTGCTTGGGCCACCGCCAATGCGTGCGTCAGATTGCGACGTGCACCGTGACGCCGTCGCTCTTTGTCTCGGTCGGCCTCGACGACGCGTTGCACCTCTGGGACGTCGAGACGGGCGCGCTCTCGGTGTGCCCGCTCACGACATCGCGCTCGTCCGCAACACCGACATGGCTGTGCGCTTCTTTAGCATCAACCAGCGCCGTTTTTGCAGCAGTTTGATATTGCCGGCGGGTGTCGATGCGTTCGCTCGCCGTGCGCGACTTCCGGAAGCTCCAGAACGACCCGCCGTCGGGCGTCTCGGGCGCGCCGCTGGACAACAACATCATGGTGTGGCAGGCGGTCATCTTTGGCCCGGACGACACGCCATGGGAGGGCGGGACCTTCAACCTCCTCCTCGAGTTCAGCGAAGACTACCCGAACAAGGCGCCGTCGGTCAAGTTTGTGACCAAGATGTTCCACCCCAACATTTACAACGACGGCCAGATCTGCCTCGACATTTTGCAAAACCAGTGGAGCCCCATTTACGACATTTCCGCGATCCTGACCTCGATCCAGTCGCTGCTCTGCGACCCGAACCCGAACTCGCCCGCCAACTCGGAGGCCGCGCGCCTCTTCCAAGAGAACCGCCGCGAGTACAACCGCCGCGTGCGCGAGATTGTAGAGCAGAGCTGGGCCGCCATGTATGGTGTCGGCCGCCAAGATGCGTGGTGCCGAGCGTCGCATGAACGACGGCCGCCCGTTCGCCACGTGGTTGGAAAACATCACGTCCGACCGTGGTCTCTGCGGCGGTTGCAACTCGTACATTGCCAAGGCCATGCGCCTCCAGAGAACTCGAGCATCTTCACGATTGGTGACAAGGGCCGCGGCCAGCTTCGCCGCACGCACGGCAAGTACATCATTGGTAACGCCACGGAGACGTGGATCAACCCGACCAACTTTGCCAAGGCGTCGGCGCTCGCCGAGGTCGTGGCGGACGAGAGGAGTCGTCGCGCATGACGGCCATGGAGAACGCGTCGAGCAACGCGTCCGACTTGAGCCTCCGCCTCGTGTACAACAAGGCCCGCCAGTCGCGCATTACGACCGAGCTCATCGAAATTATTTCCGGCGCGGCTTCGTTGGACAGTGGTCGAGGTCACGTCGGAAGAGCAATGGAAGACCCTGTCGGGCGTCGCCAAGACGTACGTCATGTTCTTCTACGCCAAGAACGTGAGCATCCCGTCCACAACGGGCGACTGGCTCAGCACGGCCAGCGTCCGGCAACAAGGTGCTCGAGCGCATCGAGGGCATGAAGGTCGCGGAGCTCGCGCAGGCCATCTCCGCAGTCGCTTGGAAAAGCTCATCTCGGCGTCGCCGGTCATGGTGTTCATGAAGGGCAACCCGAGCGAGCCCAAGTGCGGCTTCTCGCGCAAGCTCATCGAGCTCCTCAAGAGCGAGAACATTGAGTGCGGCACGTTTGACATCCTCCTCGACGAGACCGTGCGCCAAGGCCTGAAGCTCTTCTCCGACTGGCCGACGTTTCCGCAGGTGTACGTTAACGGGTCGCTCGTCGGCGGCCTCGATATTCTCCTCGAAATGAAGGAGGAGGGCAACCTCGCCGACCAGCTCGGCGAAGAGGAAGCGGAAAAGGCGCTGTTTGCGAGCTTGACGGAGCTCTTGCAGTCGGCGCCCGTGCTACTCTTCATGAAGCTTTGACATCCTCTGTGGCAGTACCAGCCGCATTTTCGGGACCTCAAGCACGTTCTCCAAGTGCCACCGTCCATCTTTCGCCACACTGTGCGTCGAGGACTTTCAATGGTACCCGCAACAAGATTGCTCTCGGGTTTCAAAAGCCGAATGCGGACGCAGGTGAGATCTGCGTCGTCGAGTGGACCGCGTCTGGAACCGGTCGTCGTCGCTTGCGTCCAAGGGTGTCTCGTGCATCGATTGGCAACATGAAGACGCTCCACGCGGACCATCGCAGCGAGATCCGGTCGCTCTGCGTGCACCCGCACTCCCCGTCAGTCTTCTCTGGCGCGCTCGACGGGCTTGTCATCGGCTACGATCTGCACCACCAGTCGGCGACGACCGTGCTTGAGAAGCGGCAGAACATTGCCAAGATCAACGCCGTCCTCGCGCACCCCAACCACCCGCATTTGCTCATGGTGTCATGCGTGCACTCGGCAGCAGACGATGCTGTTGCACGACCTCCACGAGCATGCCGTCCATGTGGTCGCCCGGGCCATGAGCCAGTACACAACGCCGCGCTGGTCCACGGCCGGGATGCACGTGTCGTGTGGCAGCACATCCGGTACTGCCACAACAACTTTGCGACGCCGACGGCCGCGACCATCGGCGCGTCCTTCCTCCAGAAGCGCATCCTCGTCGACAAGAAAATGACGCTGCAGATTTGGGACACGGCCGGCCAAGAGCGCTTCAAGTCGATGGCCTCCATGTACTACCGCAACGCCAAGGCCGCGATCCTCGTCTTTGACGTGACCAAGCAAGACACGTTCGATAAGGTCAAGGAGTGGCTGAAGCTGCGAAAACACGTCGACAATGACATGTCGGCAACAAGTGCGACCTTTTAACTTTGGCCTCGCCGAGGCGTACGCGAAGGAGATTGGTGCGACGGCCCACCGCACGAGCGCGCAGTCGGGCCACGGCGTGACCGAGATGTTTGAGACGGTCTCGCGCGGCCTCTTGGCGCAGTACTTGGACAATGAGCAGAGCAATTCACGGTCGTCTACATCCCTGCGACGGACGACAAGGAGCTCGCCGAGTGGCAGATCGACCTCCCAAAGGACATTGACGGCCAGATCTCGTGCCTCACGGAGCGCCTCCGCGCGCACTTCAAGGAGCAGTCCTTCCAGCGCCGAGCAGAAGCCTTTCGCCAGCAGATCCTCGCGCAGATGCCCAAGGGCTCGACGATGAACGACGAGGTCATGTCGATGATGCATGGACAGCTTGGTGGACTCGGTTCCGCTCATCACCAACTCGGCTGCGGCCAAGCACATTCGAGGTGCGCGGTGACGCGTTCATTGGCCGCGTCTTTGACAGACGCCTTTGTTCGCATGGACTTCAAGTTGAGCGAGATCAACGCCGATGCCGAGTGGATCAAGATCGCGCAGAGCCAGACATTGCCAACACGCTCGAGCACATCATGGCCATGGGCCAGGGCCTCGACGACCAAGAATACACGGACGAGCTGCATGTGCTTCAAAACGACCCCAACACCAACTTGTACTCGGCCGTGACGTTCGAAGAGCTCAACTTGCCCGAGAACTTGCTCAAGGGCGTGTACGCGATGAAGTTTACCAAGCCCTCGAAGATTCAGTCGGTCGCGCTGCCCATGATCCTCTCCAATCCGCCCGAGAACCTCATTGGCCAAGCGCAGAGCGGCAGCCGACGCGCGAGCTCGCCCGTCAGATCATGGCCGTCGCGACCATCATGGGCAAGTTATTGAAACGTTCCTCGCCGTCCCGGGCAACGACCTCGAGAAGGGCCAGTCGGTCACGGCGCAGATCGTCGTTGGCACGCCGGGTCGCATCGAGAGCCTCATCAAGCGACGCCAGCTCGACCTCAAGGACCTCAAGATCTTTGTCCTCGACGAAGCCGACGTCATGATTGACGAGTCGGGCATGCGGGACCGGTCGCTCGCGATCAAAAAGCAAATCCGCAACCCCAACTGCCAGATGCTGCTGTTTTCGGCCACGTACGCGGACGAAGTACGCGAGTTTGCCGAGAAGCTTGTGCCCAACCACAACATTATCACGGTCAAGAAGGAGAAGCTGTCGCTCGATGGCATCAAGCAGTTTTGGATCGACTGCAAGTCGCAGGCCCACAAGTACGACGTGCTCTCGGACCTCTTCGGGCTCCTCAACGTCGGCAAGAGCGTCATTTTCGTGCAGTCGCGCGAGACGGCCAAGAAGCTCACGGCGAGCATGCGCGAGAAGGGCCACTCGTGCGGCATCCTGCACGGCGCCGACATGGCCTTGGAGGTGCGCGACAAGGTCCTCGACGAGTTCCGCCTCGGCACGACCAAAGTGCTCATCACGACCAACGTGCTGGCGCGTGGCATTGACGTGCTCGGCGTCTCGCTCGTTGTCAACTTTGACATTCCCGTCAACCGCGACAACCAGCCGGACCCGGAGACGTATCTGCACCGCATTGGTCGCACGGGCCGCTTTGGCCGGAAGGGCGCGGCCATCAACTTCGTCCATGACAACAAGTCGAAGCGCGACCTGGCCGAGATTGAAAACTACTACGAGAAAGAGATCGTCATGGCGCCGGCCGACGACCTCGAAGCGCTCGAGAAGCTGCTTTCGTGGATGGCTCCAAGCGCCGCCTGGCGAAGCTCGGGAAGGAGCAGCGCAAGGGCATGAGTATCTACCCGCGCGACCCGAAGAAGAAGGCGAGCGGCAAGGCCACGACGTACTACTACATCAAGGACATTATGCACCTCGCCCACGAACCGATCCTCAACAAGTTCCGCGAGTTCAAGACCTTCATGAAGAAGCTCAAGAAGTCGCTTGGCCGCAAGGACATCTCCGAGGCGCGCCGCAAGAACGACCAGAAGCCTACGTACGAGCTCGACCACATTGTCAAGGAGCGCTACCCGCGCTTTGTTGACGCGCTCGGCGACCTCGATGATGCGCTCTGCGGCAGAACTACGTCTCGGTCTCTCGCAGCTTGACCAAGGTGTTTGTGTCGATCAAGGGCATCTACTACCAGGCGTCGGTCCTCGGCGAAACCATCACGTGGCTCGTGCCCCACACGTTCACGCCGCAGATGGACAAGCACGTTGACCTCCGCGTCATGCTTACGTTCCTCGAGTTTTACGAAGTCCTCCTCAAGTTTGTCAACTTCAAGCTCTACACGGACATTGGCGTCGCATACCCGCCCCAGATTGACCTGCAGCTCGACGGCGCTGGCGTCCAGCTTGCCGCCATGAAGCTCGCAGCAGAAGGCCTCGGAAGGTCGCATTGCGTGTCGCAGACACGAACGCCGAGGAGTCGCACCACAAGCTCTTCAACGGTCTCCGGTTCTTTCTCTCGCGCGAAGTGCCGGGTGCCCCGTTGGAGTTTGTGATTCGCGCGCAGGGCGGCGTCGTTGGCTGGGAAGGCCAGGGCTCGCCGTTCAACGAGAAGAGCGATCTCATCACGCACCACATTATGGATCGCCCGCACCAAGGTCACCGCTACTTTACCCGCGAGTACGTCCAGCCGCAGTGGATTTTCGACTCTGTCAACAACGGCGTGCTCTTGCCGCTGGACAAGTACCGCAAGCAGCTCGACAAGCTCAAGTCGGCGCTCGACGTCCACGCTGAACTTGCTGAGGAAGAAGACGACGACGAGGTGTAGCCGTGTACGCGCAGGGCCTCAAGGACGAAGCCGACGGCAACAAGCCCACCAAGAAGATGAAGAAGCAGGCCGAAGACGAGGAGCTCCATGAAATGGCTGTCTCGATGATGAACAAGAAGGCCAAGAGATTATACAGCCGCATGCAGCACGGCATTGGCAAGAAGTCGGACGCTGTCAAGGCCCTCGAGGACAAGAAGAAGGCGTTGGACACAAGTGGAAGCACTTTAGCCGCGGCGCGATGGCCGGCGGCCTCGCCGCCATCCTCTTCCAGCCCTTCGGGCAGACCGGGGGCGCGGCACGACGCCGACGCTGCTGCGTGTCTGCGGTGGCGCCGGGCTCTACTTCTCCACCTTGCATCAGCTAGATGCTCAAAACCTTAAC---GTACTCGGCTCCTTTTGCGCAGGCGCCTTTGCGCGGTCGTTCGCAGGCTTTGTTATGTCGCCGCTCACGGTCGTCAAGGCGCGCATGGACGCCGTCGCACGACAAGGGCGCGATCCGGACGTGCCGTTCTCTGGCTTGTACGTCACGCTCTACTCGGCACCCGGCCGACGTGGTCAAGACGCGCATGCATCCGCGCGACCTTGCGCCGCATCTACGTAGAAGAAGGCCCGAGAGGCTTCCTTCGCGGCGTCGTCCCGCGTGTCTTCAAGCGCACGTTCTCCACCGCGCTCACGTGGAGCATGTACGAGTTACTCGTTCGACCCGTCGGGCCTCGAGTACGCCAAGGAAGCCTTCAACTTGGCCAAGGAGACCGAGACGACCAAGCAGATGGAGTACCAGGCCAAGGTCAAGGAGAACGAAGCACTTACGAAGGCGTACGAAGTGCAGCGCATTCAGAAGGAAGGCGAGGAGTCGCGCAAGAACATGGAAGCGCAGACCGAACACAACCAGCGCCGCGCGCAGTACCAGGACCAGCTCAAGCGCAAGCAGTGGGCTGACCAGGCGGCGGCGCAGAAGCTCTTCAAGGAGCAGGAGATGAAGAAGCAGGAAGAGATGCTCGCGCGCCAGGAAGCCTCGCGCCGCAAGACGCTCGAGTACGAAGCCGAGCTCCGCACCAAGACGGAGATGGCCAAGGTGGCGGCTGAAGTGGAAGGTCGCATCAAGCAGGAACGCTTGAACCACGATCTGCATCTCGAGGAAGCGCGCGTCCGCGCCAAGGAGTACCGCGAGACGGTCATGGAAGGCATCAAGCTCGCCTCCGACACGGTCGGGTCGGGTGTCATGGCCTTCTTGGGCGACTCGGAGAAGCTCTCTGCCACCGTCATGTCGCTCTCGCTCCTTGCGCTTGGTGTGTACACGGCCAAGGTCTCGACGAGCGTCACGGGCCGCTACATTGAAGCCCGCCTCGGCAAGCCGAGCCTTGTCCGTGAGACGTCGCGCCGATCGGCACTCCAGGTTGCGACCAACCCGATCCCGTCGGTGAAGCGCCTCCTTCGCATGGGCGCGCCCTCGGACGCCCTTGAAGGCGTCGTCCTCGAGCCCAAGCTCGACGAACGCCTTCGCTCGGTCGCGGTCTCGACGTTCAACACGAAGAAGAACCGTGCGCCGTTCCGTCATTTGCTGCTGCACGGCCCGCCCGGTACGGGTAAGACGCTCTTTGCCAAGGCGCTCGCGCGCCACTCGGGCCTCGAGTACGCGATCCTCACGGGCGGTGACGTGGCGCCGCTCGGCCGCGAAGGCGTCACGGAGATCCACAAGCTCTTTGACTGGGCGTCGCACTCGAACCGCGGCCTCCTCCTCTTCGTCGATGAAGCCGACGCGTTCCTCCAGAAGCGCTCGAACGCCGTCATGAGTGAAGACATGCGCAACGCGCTCAACGCGTTCTTGTACCGGACGGGTGAAGCCAGCGACAAGTTTATGATTGTGTTTGCGTCCAACCAGCCCGAACAGTTTGACTGGGCGATCAACGATCGTATCGACGAGATGGTCGAGTTCCAGCTCCCGGGCCTCGACGAGCGCGTCCGCATGCTCCAGCAGTACGTTGACAAGTACCTCCTCAAGTC---CAACTCCAAGGCCAAGCAAATCGTGGGGAAGGCAGCCTCTTCACGCTGGACCCGACCTCGGGCTTCCTCGTGCCGCGACGCAGCTCGTGCACATGGACGCGGTCGTGAATGTGTCGCCGACCGGCCAAGCCATCTTTGACGCGCTGAGCAAGACACGATGAAGGTCTACTGGTCCATCTTTGGCCTCTTCATCTTGAACGGCGCCGTGACGCAGATGACGGGCCGCGACGAGTTTCACTACTACGACTTGGTCAAACGGAAGATCCCGTCCGAGTACCGCATCTCCGAGAAGTGGGACAAGTGCATCGAGAGCTTCATGGTCAACTTCTCGGCCGGCCTCGTCGCGGGTGGCTTGACGTCGCTTGTGCTTGCGCGTACGGGCGCGGGCCGCTCGGCCTTGACGGGCTTTGGCGTGCTGGCGCTGGCGCGACCTGGACGACGTGCAGCTTGGCGTTTGAAGAAGAGGCGAGCGACAACTAA----------------------------TGAGCC------------------------GCTTCGTGAGCGCGACGTCCAAG--GCCGCGACCGCGGTCCGCGCGCCGCCGATGGCATGGACGAGAAGCTCGTCGGCCTCCTCGAGGCCTACCTGGACGAGCAAATCGCGAGCAACGTCGTCGACGAGGAAGCCAACCTCATGCTCCTCAAGCTCTACACCATCTACCCGGTCAAGCCCGAGCAGCACCGCCCAGGTGCTCGCCAAGGGCCTCATGGCGTTGCCGTCCAAGTACTTCCTTGGTGACGTACATGGTCACGGAGGCGCTGCGCAAGGACAAGAACATTACCGACTTGCTCAAGGCCGGCGACGTCTTGCAGTCGTGCTTGTTCACGGATTTCTGGACGCTCGACTTGCCGTTCGCCAAGAAGATTCCTGGCTTCGAGCAGGCCGTGCGCGCGTTCATTCTGTCGACGATTGCCAAGAGCCACGAAGTGGTCGCCAAGGCGTTTGTGCAGGCCCAGCTGAACTTGTCAGAAAAGGACGTGGCGTCGCTCGTCGCGCTCGGCTGGACAAGGACGCGTCGTACGTTGTGACGCCCAACTCGGAGAACCAGATGCGCCCCAAGAAGTTCAAGGAAGACATTGAGTTTGCCGACATTCTGGACACGATCCAGGTGCTCTCCCGCTCGAACGTCTGCGCCAGCGGTGGCGGCGTCGCCGACGCGCAAGAGATCTGCGAGGCCATCGCCGACCACCTTGACCTCCCGAGCGGCAAGATGGTGCTACCAAGCTCGACAGGCGTCATTGGCTGGCGCCTCCCCGTCGATGCCATGATCCAGCACATTCCCGAAGTCGTCGACTCGCTCCAAGACACGAGCATCTACCCAGCTGCCAGTGGCATCATGACGACAGACTTGTACCCCAAGATTCGATCGGCGGATGTCTGCGGCGGCCGCATCGTCGGCATCGCCAAGGGCGCCGGCATGGTCGAGCCCAACATGGCCACGATGCTTTCGTACATCCTCACAGATCTGGCCGTGCCCCGGGACACGCTGCGCGCGCTCCTCTCCGAGGTTGTCGAGAAAACGTACAATGCCATGAGCGTCGACACGGACGAGAGCACGTCCGATACGCTGGCGATCGTCTCGTCCGGTCAGATCCCGTTTGACGAAGCGACGCAGCTCGAGGCCTTTCGCGCAGCGCTGTACGACGTCTGCGGCGGCCTCGCCGAAGACATTGTGCGCAACGGCGAAGGCGCCCACCACGTCATGCGCGTGCGCGTCAAGGGCGCAGTCGACGAGGTCATGGCCAAGGGCGTCGGCAAGTCGATCGTCAACTCGCCGCTGCTCAAGTGCGCCGTGGCCGGGAACGACCCGAACGTCGGCCGCCTCGTCATGGCCGTCGGCAAGTACATGGGCAAGCACCACAAGGGTGTCGCGGTCGCCGATGCGATGAGCATCTCGATGGGCGGGCTTCGCATCTTTGAGCACGGCGAGTTTGCGCTCAACAGCGACGTCGAAGCGCTCCTCGTCAGCCACATGAAGCGTGCGCAGCTCACCGAGCCGACGCGCGGCGGCCTCGAGGTCGCGTCGCACGACTACCCGCCGCACGACCACTGTGTCGAGATCACCGTCGACCTCGGGCTCGGGCAGGCCGAGAGCACCGTGCTCGGCATCGACTTGACGCACGAGTACGTCGCCATCAACGCCGACTACCGGTCGTAATGCCACC---GAAGAAGGGGAAGACGGTGGCGGCCCACTTGGCCAGCTTGGGTATCTCGGCCAG---CGCGGCTGCGGACAAGAAGAAGAAG---AAGAAGGGCAAGAAGAAGGGCGACGATGCCAAGGAAAGAAGCCCCTCTCGGCTGCTGCCAAGGCGCTCT-TGGAACGCCAAGCGGCCCTCAAGGCCGAGCAGGAGCGCCTCCAGGCGATCCAGGAAGAAGAGGAGCGCCTTGAGCGGGAAGCCGAGGCCGCGCGCCTCGAAGTGATCCGCAAGAAGGAAGAGGCGGCCGAGCGCAAGCGTCTCGCCAAGAAGGCCAAGGTCGAAGCTGCCAAGGCCGAGGGTACCTACATGACGAAGGCGCAGAAGCTCAAGGCGCAGCGCGCGCAGGCGGCTTTGGAAGCCATGAAGGCGCAGGGTCTTGTCTCGATTGCTGCC------GACGAAGAGAAGAAGCCTGTTGTGTACACCAACACGAAGAAGAACAAGAAGCAGAGGACGTGCACAAGGCCGAGGCCGACGCCCCGGACGCGTGGGACGATGATGAGTGGGACGACTCGGCGCTCATTGCGACGATCCAGCAAAAGGTAGAAAGAAAAGAGA---------------------------------AGGAGCCGCCAGGCCGAGCAGGAAGCCGAGGCGGCTCTCGCGGCCGAGGCGGAGACCGTCCGCTCCAAGGCCAAGCAAGAGGAAGACCGCAAGAAGCGCGAGGCTGCCGAACGCCGCCGCGTTCGCGAGGAGGAAGCCCGTGCGGCCATCTCGGAGGATAACCTTCGGTCGCCGATTTGCTGTATCATGGGCCACGTCGATACGGGCAAGACCAAGCTCCTCGACAACATCCGCAAGACCAAGGTGCAGGACGCCGAGGCTGGCGGTATCACGCAGCAGATTGGTGCGACCTTCTTCCCTGTCGAGGCCATCAAGCAAAAGACGGCTGCGCTTCAGAAGGTGACCAACCTCCAGTACAAGCTGCCGGGTCTCCTCGTCATTGACACGCCGGGCCACGAGAGTTTTACCAACCTTCGCTCGCGCGGCTCGTCGCTCTGCGACATTGCCATCCTCGTCGTCGATATCATGCACGGCCTCGAACCCCAGACGCTCGAATCGCTGCGCCTCTTGCGTGCCAAGCGCGCGCCGTTTGTTGTCGCCCTTAACAAGATTGATCGCTGCTACTCGTGGAAGACGTGCCCGGACATGCCGATCCGCGACGCGCTCGCGCTCCAGAACGAACACGTGGTGCGCGAGTTTGAAGACCGCGCCAAGAAGATCATGGTCGAGTTTGCCGAGCAGGGCCTCAACGCTGAGGTCTACTGGCGCAACAAGGATCTCGGGCGCACCGTGTCGCTCATCCCGACGTCGGCGATTTCCGGCGAAGGCGTCCCGGATCTGCTCATGATGCTCACCAAGCTCACGCAGGAGCGCATGGCCAAGTCGCTTGCGTTTGTCGACATTCTCCAGTGTACCGTGCTCGAAGTCAAGGTCATCGAAGGCCTCGGCACGACCATTGATACGATTCTCGTCAACGGCTCGCTGAGCGAAGGCCAGACGATTGTCGTCTGTACGCTGGAAGGCCCGGTGGTCTCGACGATTCGATCGCTACTGACGCCGCACCCGATGAAGGAGATCCGCGTCAAGGGCGAGTACATCCACCACCAGACCATCAAGGCCGCGATGGGTGTCAAGATTTGCGCCCAAGGCCTCGAGAAGGCGGTGGCCGGCACGCAGATCCACGTCGTGGGCCCTGACGACGACGTCGAGGAGCTCAAGGACTCGGTTATGTCGGACCTCTCCAACATTATGGACTCTGTCAACGCGACCAAGCGCGGTGTCATGGTGCAGGCGTCGACGCTCGGTGCGCTCGAAGCACTCCTCGAGTTTCTTCGGTCGTGCGACCCGCCGATCCCGGTGGCGTGCGTCAACATTGGTCCGGTCCACAAGAAGGACGTAGTCCGCGCCAGTGTCCAGCTCGAGCACAACAAAGAGTACGCGACGATCCTCGCATTCGACATCAAGATTCAAAACGACGCCAACGAACTCGCCAACGAGCTCGGCGTGCGCATCTTCACGGCCGACATCATCTACCATCTCTTTGACCAGTTTACGGCGTACATGGACAACATCAAGGCGCTCCGCCGCGAGCAGTTTGCCGACGTCGCGGTCTTCCCCGTCGTCCTCCGCATCATCCCGACGTGCGTCTTCAACAAGAAGGACCCGATCATCCTCGGCGTCGACATTGAAGACGGTATCCTCAAGGTGGGCACGCCGCTTGTGGCGATCGTGGGCGGCGCGCTCGTCTCTGTCGGCCGCGTCGGCTCGATCGAGAAGGACAACAAGCCGACCGAGACGGCCAAGAAGGGCGCGAGCGTCGCGGTGCGTATTGAAAGCGACTCGAACGTGACGTACGGCCGCCACTTTGACCACACGGCCAAGCTCTACTCGCATCTGAGCCGCGCGTCCATCGATGCGCTCAAGGAGAACTTCAAGGAGGACCTCGCCAAGGACGACTGGCGGCTCGTCATTCGTCTCAAGGGCGTCTTTGGCATTATCATGGAGGGATGGCTCACCAAGCGGCGCGACCACATGAACATCATCTGGACGGAGCGCTACTTCATCCTCGACGGCAACAACCTGCGCTACTACAAGAAGCGGGGCGACCCTGCGCCGAGAGGCGCGTATGTGCTGACGGACGGCTGCGTCGTGGGCAAGGTCTTCAACTCGGAGGAGAAGCACAAGCACCATGAGCCCGTGTGGATGTTCCGCATCACGTGAGAAGAGCTACAAGAAGGACCGGTTCGTCGACCTCGGCGCCAAGACGGAGGCGCTGGCGAACGAGTGGAAGAAGACGCTCGAGCAGACTATCTCGGGCGTGAAGGAGATGTTCAAGCACGTGCGCACAATGAAGTACGAGGCCGACCA---CGGTGCCACGAACCCTGAA---------GTCGTCTCGAAGCTCCTTGCCGAGGACGAGAACATCTCGCTCTTCAACCTTGACGAAGACAACGCCAAGGCCGAGCAAGGCTGGTGGCTCGCGCGGGTCGAGGACGGGCTGCGCATCATGCAGGAGTGCCCGTTGGGCCATCAAGTCGCGGCGTCCATGTACGCGCACCACGCAACGCTCACCAACAACCTCCTCTTTGCGTCCGCCATCAGCTCCATCTTGGCGTTCAGCATCATGTACTCGTTCATCCACAACATGCTTATCTGCATCCTCACGGCCGTCGGTGCCGTCGTCGTGTGTGGCATCACGCTCATGCCCAAGTCGTACATTGAGATCCCGAGCTTGCGCGTCTCCCAGGTCGTGCATGGTTCGCCCGCAGACGTCTTCCGCCTCATCATGAACCCGTCGCGGTACCAGCGATGGGACGCGTCGGTGGCGTCGATGAAGGTGATCCAGACCATGGACGACCACGCCGACATCATTTACATCACGCTCCGCCCCGTGTACATTTGGCCCATGTGGCAGAAGCCGCGCGACCTCGTGCTCATGCGCTACTGGCGCCGCGAGGAAGACGGCTCGTACTTTGTCATGTACCAGAGCACGACGCACCCCGAGTGCCGCGTGCGCCACAACTATGTGCGCGCGAACCTCCTCGGCGGTGGCTTTGTGATTGCGCCGCAAAAGACGGCGATCGCGGGCGGTGTCCGGTCGCTCGTCACGTACGTCTTGCGCTACGACCCGAAGGGCAG---TGCGGCGCTGTACCACCAGCTCGGGATGGACGTCGACATGGTCGTCCCGATGCTTCGCGCGATCGTTGGCATTCGCGACGAAATGAACACGAACGACTTTATTACGCCCATTGTGACCGTCTCGGAGCCCGCGTCGA-CGGGTGCCGCCGGGCGACTGTCGA---TGCGGCCGGCCTTGTCCACGGCGTGAAGAAGTGGAAG---ACGTCGCTGCCCGAAAAGATGTGGGGCGAGCCGGACGCGTCCCTCTTTGCCGTGCGTGGGCCCAACTACCTTGTCGATAAGAAGAAGACGCCGTCGGCGCCATCGCGCTTCCACCTCGTGGGCGTCGACCTCCTCTCGTACGAAGTCGCGTCGGAGCGGTACCACGTCGCGTCGCGCCCCGGGTCGCTCGTGCAGAGCGC---GACGAGCTTTACGTTTGTCGTCAACATGATCATCCCGAGCCCGAACAACCTGAGCATGGTGTTTTACTTCCAGCCCGACTCGGCCAACATTTTCACCGAAAACACGCCGTTCTCGGAACTCCTCAATGACTTTCTGGATGGCGACGACGCGTTCCGGAACTCGCGCTTCAAGCTCATCCCGACGGTCGTCGAAGGCAGCTTTATCATCAAGCAGAGCGTCGGGTCCAAGCCGACGCTGCTCGGGAACAAGCTCAAGTGCCCGTACCACCGCGGCGACAACTACTTTGAGGTCGACTTGGACATTTCGTCGAGCTCGGTCGCCAACACGGTCGTGGGCATGGTCACGGGCGTCACCAAGATCCTCGTCGTCGACATGGCGTTCCTCCTCGAGGCGCAGACGGAAGAAGAGCTCCCCGAAGCCATCCTCGGCACGGTGCGATTGCAGCACGTGTCGCTCGACAACCCGATCAAGGTCCCCAAACATGGGGGCCTGGCAGCAAGGAAGAGTTCACGTACCGAGGGCTTCACAGAGGCCTTGAAGGTCAACCTCCAGCGCCTCATTGCGTACGCCAAGAGCGCCGATGCCAGCTTGCAGAGGGAAGTCGCCGAGAAGCTCGCGAATGAGGCTGTGAAGACCGCCAAGTGCAAATCGTCGAGCTAGACGGTCTCCAGCTGCTTTTGCCCTTGACGCAGTCGAAGGACACGGAGGTGCAGCGCCTCGCTGCCCACGCACTCGCCAACCTCTCGGTCAACTCCGACAATCAAACCAAGATGGCGAATGAAGGCGGCATTGACATGCTCATCGATCTCTTGGCGTCGGACAACGAACATGTGCAGCGCCAGGCGGCGAAAGCTCTGGCGAACCTTGGCGTCAATGTCGACAACAAGGAAAAGATCGCCAAGGC-GGCGGGATCAAGCCACTCATCAAGCTGGCGTGCTCGAAGCAAGTCGGTGTCGCCGTCGAGGCCATTGCCGCGCTCGCCAACTTGGCTGTCAACG---ACGCGAACGAGGTGGAGATTGCACGCGACGGTGGCTTGGAACCAATTATTCGCGGAGCCAAGTCGGATTCGATGGAGCTGCAGTCGCAAGTCGCGCGTGCACTGCGCAACCTTTCCGTCAATGCCGAGAACAAGGAGCTCATTGTGCGCCTTGGTGGCGTCGACGCGCTGCAGTTTCTGGTGCGTTCGCCCAACGACCGTATTTGCCAACAAGCCACGCGCGCGCTCGTG----------------CGCCGTCGGCGTCTAC---------------------------------------------------------------------------------------------------------ACGGCAAAGAGGTCCACTG----------------------------GTCCGACGCAGGCAAT--G----------------------------------------------------------------------------------------------------GCATGCTCGTGGGCACGC---CGGTCGAGCAGCGCATTCGCTGGGAAGACGGCG-----------------------------------------------------------------------ACGAGCATCCAGCAGCAGATGCGTGGCCTCAGCTGCAAGGGCGCGGTGTCTTTGTCGCCGCCAGCGGCCAGCGT-------------CGAATGGCGCCGGACGTTTGAAGCGGCGACGTTGACGATGAAGCATGCGCTGCCGAGCTCGGGAGCGACATTGAGCGGCGGCAGCG---------CGACGTGCAGCTCGAGGATTTGTACGTCAAACGCACGCTGAGCACGGGCGACTCCGCGTACATCCAGTCGCTCCAGGACAAGTACGCGGCGAATGTGAGCGCGCTGGAGGCCAAAGCCCACTTTCGCCGCCACGAAAAACGCGTCCACCTGCACGCAACCACGGCCTTTGCCAGCCTTCCGTTCCTGCACGATCGCTACCAGCTCAAGGCGCTCATCGGGCAAGGCGGCAACTCGGAAGTGTGGGAGGCGT---ACGACGTGCATACCAGTACGTTTCGCGCCATCAAGATTTGCACCAAGCTGCACCAAGCAGAGATCGAGTTCCGACACCACTTCCTACCGTCCGAGCACACGGTCCGCGTCCATGGGCCGCTGCTCATGACATCCCATCGCGGCGCGCCGTACTCGCTCATGGTCATGGACCGCATGGAGTGCGACTTGCACCAGTTCATGGAGAACCTCGGCGGGCCCTGCGAGATGGGTCTCGCACGCCACTTGCTCTTTCAACTCCTCCTCGGCCTCGACTACGTCCACC-AGGGCATGGCGCACTGCGATCTCAAGCCGGCCAACGTGCTCTTGGCCACGGTCGCCGGCGCGCAGCTGCGTTTAACGGACTTTCACCTCTCGCAGCGCAAGCACGCGATCATTCTCGTGGGCCAGAACGCGTCGCCGGCGTTCGCACCGCCCGAGTGGTACCTCTTGTCGCCGCATGAAGCGGCGAGCCTCGGCGCGACGTATGAAAAGTTTGACGTGTGGGCGATCGGACTCGTCTTCTACATGCTGCTGTGCCACGCCCACCCGCTCGGGACGCAGGCCAGCAAGCCCGAGATGACGGAGCGCATGAAGGCGTACCGAGCGACCCCGACGCTGCACTTTCCCGCGCACGTGCCCGCCGACGCACAGCATCTGATACGGCAGTGCATGCACCTCGATTGGCACAAACGACCGACAGTGCAGGAGCTGCTCGCCGCGGTCTGC

\## ALIGNMENT FILE ##

alignment = 24_singlecopy_merged_trimmed.phy;

\## BRANCHLENGTHS: linked | unlinked ##

branchlengths = unlinked;

\## MODELS OF EVOLUTION: all | allx | mrbayes | beast | gamma | gammai | <list> ##

models = allx;

\# MODEL SELECCTION: AIC | AICc | BIC #

model_selection = aicc;

\## DATA BLOCKS: see manual for how to define ##

[data_blocks]

OG0006201_pos1 = 1-849\3;

OG0006201_pos2 = 2-849\3;

OG0006201_pos3 = 3-849\3;

OG0006202_pos1 = 850-1615\3;

OG0006202_pos2 = 851-1615\3;

OG0006202_pos3 = 852-1615\3;

OG0006203_pos1 = 1616-5314\3;

OG0006203_pos2 = 1617-5314\3;

OG0006203_pos3 = 1618-5314\3;

OG0006204_pos1 = 5315-5544\3;

OG0006204_pos2 = 5316-5544\3;

OG0006204_pos3 = 5317-5544\3;

OG0006205_pos1 = 5545-6079\3;

OG0006205_pos2 = 5546-6079\3;

OG0006205_pos3 = 5547-6079\3;

OG0006206_pos1 = 6080-6508\3;

OG0006206_pos2 = 6081-6508\3;

OG0006206_pos3 = 6082-6508\3;

OG0006207_pos1 = 6509-6947\3;

OG0006207_pos2 = 6510-6947\3;

OG0006207_pos3 = 6511-6947\3;

OG0006208_pos1 = 6948-7525\3;

OG0006208_pos2 = 6949-7525\3;

OG0006208_pos3 = 6950-7525\3;

OG0006209_pos1 = 7526-8108\3;

OG0006209_pos2 = 7527-8108\3;

OG0006209_pos3 = 7528-8108\3;

OG0006210_pos1 = 8109-8533\3;

OG0006210_pos2 = 8110-8533\3;

OG0006210_pos3 = 8111-8533\3;

OG0006211_pos1 = 8534-8938\3;

OG0006211_pos2 = 8535-8938\3;

OG0006211_pos3 = 8536-8938\3;

OG0006212_pos1 = 8939-10134\3;

OG0006212_pos2 = 8940-10134\3;

OG0006212_pos3 = 8941-10134\3;

OG0006213_pos1 = 10135-11374\3;

OG0006213_pos2 = 10136-11374\3;

OG0006213_pos3 = 10137-11374\3;

OG0006214_pos1 = 11375-11858\3;

OG0006214_pos2 = 11376-11858\3;

OG0006214_pos3 = 11377-11858\3;

OG0006215_pos1 = 11859-13200\3;

OG0006215_pos2 = 11860-13200\3;

OG0006215_pos3 = 11861-13200\3;

OG0006216_pos1 = 13201-13327\3;

OG0006216_pos2 = 13202-13327\3;

OG0006216_pos3 = 13203-13327\3;

OG0006217_pos1 = 13328-13434\3;

OG0006217_pos2 = 13329-13434\3;

OG0006217_pos3 = 13330-13434\3;

OG0006218_pos1 = 13435-13682\3;

OG0006218_pos2 = 13436-13682\3;

OG0006218_pos3 = 13437-13682\3;

OG0006219_pos1 = 13683-14340\3;

OG0006219_pos2 = 13684-14340\3;

OG0006219_pos3 = 13685-14340\3;

OG0006220_pos1 = 14341-15377\3;

OG0006220_pos2 = 14342-15377\3;

OG0006220_pos3 = 14343-15377\3;

OG0006221_pos1 = 15378-17957\3;

OG0006221_pos2 = 15379-17957\3;

OG0006221_pos3 = 15380-17957\3;

OG0006222_pos1 = 17958-19999\3;

OG0006222_pos2 = 17959-19999\3;

OG0006222_pos3 = 17960-19999\3;

OG0006223_pos1 = 20000-20750\3;

OG0006223_pos2 = 20001-20750\3;

OG0006223_pos3 = 20002-20750\3;

OG0006224_pos1 = 20751-22340\3;

OG0006224_pos2 = 20752-22340\3;

OG0006224_pos3 = 20753-22340\3;

\## SCHEMES, search: all | user | greedy | rcluster | rclusterf | kmeans ##

[schemes]

search = greedy;
