# Supplementary material for: Complete telomere-to-telomere genomes uncover virulence evolution conferred by chromosome fusion in oomycete plant pathogens
Source: Nat Commun. 2024 May 30;15:4624. doi: 10.1038/s41467-024-49061-y (PMC11139960; doi:10.1038/s41467-024-49061-y)
Supplement: Supplementary file 11 — Source Data [file 41467_2024_49061_MOESM11_ESM.zip › Zhang_et_al_SourceData/Fig. 2.zip!/Fig. 2/Fig.2c_configure_files.docx]

Files for the ancestral chromosome reconstruction analyses.

**Contents**

#1. Configuration settings for reconstructing ancestral chromosomes with different alternative tree topologies

Pages 2-5: The configure file is: my_agora-iterative-constrained.ini

#2. Running parameters for AGORA

‘./Agora-dev/src/agora.py ./my_agora-iterative-constrained.ini -workingDir=output’

########################################

\# General configuration file for AGORA #

########################################

\# Section 1: folder structure

##############################

> Files

\# Note: these two paths are relative to the directory of this .ini file

speciesTree = ./38-single-copy/SpeciesTree.nwk

#geneTrees = ../example/data/GeneTreeForest.nhx.bz2

orthologyGroups = ./38-single-copy/orthologyGroups/orthologyGroups.%s.list # Alternative to geneTrees

genes = ./genes/genes.%(name)s.list

\# Files for results

###################

\# Note: these paths are relative to where you run agora.py from, or

\#	its -workingDir command-line option

\# the %(filt)s and the %(name)s should not be modified as they are substituted

\# with values / identifiers.

ancGenesData = ancGenes/%(filt)s/ancGenes.%(name)s.list.bz2

ancGenesLog = ancGenes/%(filt)s.log

geneTreesWithAncNames = GeneTreeForest.withAncGenes.nhx.bz2

pairwiseOutput = pairwise/pairs-%(filt)s/%(name)s.list.bz2

pairwiseLog = pairwise/pairs-%(filt)s/log

ancBlocks = ancBlocks/%(method)s/blocks.%(name)s.list.bz2

ancGraphs = ancBlocks/%(method)s/graph.%(name)s.txt.bz2

ancLog = ancBlocks/%(method)s/log

ancGenomesOutput = ancGenomes/%(method)s/ancGenome.%(name)s.list.bz2

ancGenomesLog = ancGenomes/%(method)s/log

\# In all the following sections

\#	' ! Ancestor_list ' at the end of the line restricts the command to some ancestors

\# Section 2: ancestral gene sets

#################################

> AncGenes

\# Syntax:

\#	name = all

\# Function:

\#	Extract the complete set of ancestral genes from the gene trees and use the requested

\#	name in the rest of this configuration file.

all = all

\# Syntax:

\#	name = min max

\#	name1,name2,...namen = min1 max1, min2 max2, ... min_n max_n

\# Function:

\#	Given the complete set defined above, and located in 'ancGenes/all/',

\#	this tells AGORA to launch the size filter program, which keeps the families where at least min% of the species are present, and the size does not exceed max%

\#	The result of the filter is a set of "constrained" ancestral genes, further refered in this configuration file by 'name'

\# Note:

\#	It is more efficient to bundle all filters in the same line

1,2,3,4 = 1.0 1.0, 0.9 1.1, 0.77 1.33, 0.2 2.0

\## Instead of

#1 = 1 1

#2 = 0.9 1.1

#3 = 0.77 1.33

\# Section 3: pairwise comparisons

##################################

> Pairwise

\# Syntax:

\#	name = method arg1 arg2 ... argn

\# Function:

\#	Tells AGORA to launch the pairwise program named 'method' with arguments (arg1 arg2 ... argn), using the ancestral gene set 'name'

\# Supported methods:

\#	conservedPairs: List of conserved pairs

all = conservedPairs

1 = conservedPairs

2 = conservedPairs

3 = conservedPairs

4 = conservedPairs

\# Section 4: integration

#########################

> Integration

\# Syntax:

\#	[custom_name] method arg1 arg2 ... argn (input_pairwise) > {output_integration_alias} < {input_integration_alias}

\# Function:

\#	Tells AGORA to launch the integration program named 'method' with arguments (arg1 arg2 ... argn), using the result of the pairwise comparison 'input_pairwise'

\#	The list of integrations must begin with a method that produces ancestral blocks from scratch (such as 'denovo').

\#	Subsequents methods should act like filters, reading ancestral blocks (by default, the results of the previous method) and producing new ones.

\#	A method can use a specific method as input with the '< {input_integration_alias}' directive, with the alias defined by '> {output_integration_alias}'

\# Supported methods:

\#	denovo

\#	fillin

\#	fusion

\#	insertion

\#	scaffolds

\#	copy

\# Directory names:

\#	Directory names are generated by concatening the integration names and the input data names

\#	-> denovo-all.fillin-all.fusion-all

\#	They can be customized with the [custom_name] directive

\#	'[custom] fillin' tells to use 'custom' instead of the integration name ('fillin')

\#	-> denovo-all.custom-all.fusion-all

\#	'[custom/] fillin' tells to use 'custom' instead of the integration name ('fillin'), without adding the input data name

\#	-> denovo-all.custom.fusion-all

\#	'[/custom] fillin' tells to start a new directory name with 'custom'

\#	-> custom-all.fusion-all

\#	'[/custom/] fillin' tells to start a new directory name with 'custom', without adding the input data name

\#	-> custom.fusion-all

\# Ancestral species list:

\#	Anc: all the ancestors below Anc (included)

\#	=Anc: Anc (only)

\#	\Anc: all the ancestors above Anc (included)

\#	/Anc: all the outgroup ancestors of Anc (included)

\#	These can be concatenated with ',', and negated with '_'

\#	If none given, AGORA will use the same list as the previous step, starting with the entire species-tree

denovo (1)

fillin (2)

fusion +onlySingletons (2)

insertion (2)

fillin (3)

fusion +onlySingletons (3)

insertion (3)

[4/] fillin (4)

[4/] fusion +onlySingletons (4)

[4/] insertion (4)

fillin (all)

fusion +onlySingletons (all)

insertion (all)

scaffolds

[/iterative-constrained-workflow] publish
